# Supplementary material for: Care-seeking behaviours of mothers and associated factors for possible serious bacterial infection in young infants during COVID-19 pandemic in Ethiopia: mixed-methods formative research
Source: BMJ Open. 2023 Jul 5;13(7):e073118. doi: 10.1136/bmjopen-2023-073118 (PMC10335490; doi:10.1136/bmjopen-2023-073118)
Supplement: Author's manuscript [file bmjopen-2023-073118.draft_revisions.pdf]

BMJ Open

BMJ Open is committed to open peer review. As part of this commitment we make the peer review history of every article we publish publicly available.

When an article is published we post the peer reviewers' comments and the authors' responses online. We also post the versions of the paper that were used during peer review. These are the versions that the peer review comments apply to.

The versions of the paper that follow are the versions that were submitted during the peer review process. They are not the versions of record or the final published versions. They should not be cited or distributed as the published version of this manuscript.

BMJ Open is an open access journal and the full, final, typeset and author-corrected version of record of the manuscript is available on our site with no access controls, subscription charges or pay-per-view fees (http://bmjopen.bmj.com).

If you have any questions on BMJ Open's open peer review process please email info.bmjopen@bmj.com

BMJ Open

Influences of COVID-19 on care-seeking behaviors of mothers for possible serious bacterial infection in young infants in Ethiopia: mixed-methods formative research

Journal:	*BMJ Open*
Manuscript ID	bmjopen-2023-073118
Article Type:	Original research
Date Submitted by the Author:	24-Feb-2023
Complete List of Authors:	Tiruneh, Gizachew; JSI Research and Training Institute Inc, M&E and Research; JSI Research and Training Institute Inc, RMNCH<br>Hirschhorn, Lisa; Northwestern University<br>Fesseha, Nebreed; JSI Research & Training Institute<br>Emaway, Dessalew; JSI Research and Training Institute Inc<br>Eifler, Kristin; John Snow Inc<br>Betemariam, Wuleta; JSI Research & Training Institute
Keywords:	COVID-19, NEONATOLOGY, Paediatric infectious disease & immunisation < PAEDIATRICS

SCHOLARONE™
Manuscripts

I, the Submitting Author has the right to grant and does grant on behalf of all authors of the Work (as defined in the below author licence), an exclusive licence and/or a non-exclusive licence for contributions from authors who are: i) UK Crown employees; ii) where BMJ has agreed a CC-BY licence shall apply, and/or iii) in accordance with the terms applicable for US Federal Government officers or employees acting as part of their official duties; on a worldwide, perpetual, irrevocable, royalty-free basis to BMJ Publishing Group Ltd ("BMJ") its licensees and where the relevant Journal is co-owned by BMJ to the co-owners of the Journal, to publish the Work in this journal and any other BMJ products and to exploit all rights, as set out in our licence.

The Submitting Author accepts and understands that any supply made under these terms is made by BMJ to the Submitting Author unless you are acting as an employee on behalf of your employer or a postgraduate student of an affiliated institution which is paying any applicable article publishing charge ("APC") for Open Access articles. Where the Submitting Author wishes to make the Work available on an Open Access basis (and intends to pay the relevant APC), the terms of reuse of such Open Access shall be governed by a Creative Commons licence – details of these licences and which Creative Commons licence will apply to this Work are set out in our licence referred to above.

Other than as permitted in any relevant BMJ Author's Self Archiving Policies, I confirm this Work has not been accepted for publication elsewhere, is not being considered for publication elsewhere and does not duplicate material already published. I confirm all authors consent to publication of this Work and authorise the granting of this licence.

For peer review only

Influences of COVID-19 on care-seeking behaviors of mothers for possible serious bacterial infection in young infants in Ethiopia: mixed-methods formative research

Gizachew Tadele Tiruneh[1]*, Lisa Ruth Hirschhorn[3], Nebreed Fesseha[1], Dessalew Emaway, Kristin Eifler[2], and Wuleta Betemariam[1]

[1]The Last Ten Kilometers (L10K) Project, JSI Research & Training Institute, Inc., Addis Ababa, Ethiopia

[2]John Snow Inc. 44 Farnsworth St, Boston, MA

[3]Feinberg School of Medicine, Northwestern University, 625 N Michigan Avenue, Room 14-013, Chicago, IL

*Correspondence: gizachew_tadele@et.jsi.com

Email addresses of authors: gizt121@gmail.com (GT), nebreed_fesseha@et.jsi.com (NF), dessalew_emaway@et.jsi.com (DE), kristin_eifler@jsi.com (KE); wuleta_betemariam@jsi.com (WB); and lisa.hirschhorn@northwestern.edu (LH)

For peer review only

Abstract

Background: Implementation research was employed to examine rates and contextual factors associated with mothers' care-seeking for their sick neonates and identify system challenges for community-based possible serious bacterial infection (PSBI) services access and implementation during the COVID-19 pandemic.

Methods: We conducted a cross-sectional household survey and a programmatic qualitative study in Dembecha and Lume woredas of Amhara and Oromia regions, respectively, from 18 April-24 May 2021. Data were captured from 4,262 mothers aged 15-49 years who gave live birth 2-14 months before data collection, and interviews with 18 program managers and 16 service providers. A multilevel regression model was employed to identify predictors of maternal care-seeking for PSBI and thematic qualitative analysis to inform strategy development to strengthen PSBI implementation and uptake.

Results: Overall 12% [95% CI: 11.0-12.9] and 8% [95% CI: 7.9-9.6] of mothers reported any newborn illness and severe neonatal infection (PSBI), respectively. More than half of mothers sought formal medical care, 56% [95% CI: 50.7-60.8] for PSBI. Women who received postnatal care within 6 weeks [AOR: 2.08; 95% CI: 1.12-3.87] and complete antenatal care (i.e., weight measured, blood pressure taken, urine and blood tested [AOR:2.04; 95%CI:1.12-3.75] had higher odds of care-seeking for PSBI. Conversely, fear of COVID-19 [AOR:0.27; 95%CI:0.15-0.47] and residing more than 2 hours walking distance from the health center [AOR:0.39; 95%CI:0.16-0.93] were negatively associated with care-seeking for severe newborn infection. Multiple pre-existing health system bottlenecks were identified from interviews as barriers to PSBI service delivery and exacerbated by the COVID-19 pandemic.

Conclusion: We found gaps in and factors associated with care-seeking behavior of mothers for their sick young infants including fear of COVID-19 and preexisting health system-level barriers that exacerbated the fragile community-based newborn health services delivery in Ethiopia.

Keywords: COVID-19, Ethiopia, implementation science research, newborn, possible serious bacterial infection, young infants

For peer review only

41

For peer review only

Background

Between 1990 and 2019 the global neonatal mortality rate (NMR) declined from 37 deaths per 1,000 live births to 17 [1]. However, sub-Saharan Africa has the highest neonatal mortality rate at 27 per 1,000 live births [1]. While Ethiopia has seen a substantial decline in childhood mortality over the last two decades, the change in NMR lagged behind the decrease in post-neonatal and child mortality. The NMR in 2019 was 33 per 1,000 live births, contributing to approximately 56% of the under-five child mortalities [2].

A prospective cohort study conducted between 2012 and 2016 showed that possible serious bacterial infection (PSBI) is a leading cause of mortality in young infants in Sub-Saharan African countries, contributing to 37% of the 2.1 million neonatal deaths [3]. In sub-Saharan Africa, the incidence of sepsis was estimated to be 7.6% with a case fatality rate of 9.8% [4]. The World Health Organization (WHO), recommends that young infants aged 0–59 days with clinical signs of infection should receive hospital-based treatment [5]. However, existing evidence shows that in resource-limited settings many young infants with signs of infection do not receive the recommended inpatient treatment due to limited access to affordable care. Most families in resource-limited settings do not follow through on referrals [6] for multiple reasons, including distance and/or difficult terrain to travel, direct and indirect cost of services, lack of family or neighborhood support, lack of counseling skills, and low caretaker awareness of care-seeking benefits. Evidence from low- and middle-income countries (LMICs) has demonstrated the effectiveness of a simplified regimen of treatment, including injectable and oral antibiotics [5]. Based on these studies, the WHO recommends the management of sepsis with a simplified regimen outside of the hospital setting when referral is not feasible [5].

In 2012, Ethiopia introduced community-based newborn care (CBNC) in its flagship Health Extension Program (HEP) to improve access to and use of appropriate treatment including for neonatal sepsis when referral is not possible [7]. Despite national efforts, uptake of sick newborn care is still very low due to multiple barriers at multiple levels. These include overall decline in the performance of the HEP observed in recent years [8], service inaccessibility [9], HEWs' lack of competence and motivation [10-12], erratic supply of essential drugs

For peer review only

[10, 12], suboptimal supportive and referral linkages [10-12], communities' misperceptions about newborn illnesses [13, 14], socio-cultural beliefs, and mothers' lack of decision-making power [10, 13-15].

Between March 12, 2020, and November 30, 2020, Ethiopia had 110,074 confirmed cases of COVID-19 and 1,706 deaths [16]. COVID-19 positive cases were reported throughout Ethiopia. As of February 21, 2022, a cumulative of 468,379 cases and 7,440 deaths were reported [16] with the highest number of cases reported from Addis Ababa, followed by Oromia, SNNP, Amhara, Sidama, Dire Dawa, Tigray, Harari, Benishangul Gumuz, Afar, Somali, and Gambela [17]. There is emerging evidence and concerns that the COVID-19 pandemic placed significant stresses on the already weak health care system in LMICs [18, 19] and Ethiopia [20, 21], exacerbating already fragile newborn and child health services. However, there remains a gap in understanding of the extent and factors associated with the COVID-19 pandemic's effect on implementation and uptake of PSBI in Ethiopia.

Between November 2020 and April 2022, the Bill & Melinda Gates Foundation (BMGF) funded JSI Research & Training Institute Inc./The Last Ten Kilometers (L10K) Project conducted IR in Ethiopia to understand the demand- and supply-side challenges of management of PSBI when referral is not feasible during the COVID-19 pandemic. The project conducted formative work to understand challenges, inform implementation strategies, and provided technical support and monitoring to inform adaptation and progress. We describe results from the formative work on mothers' care-seeking behavior and practices for their sick neonates during the pandemic, along with their determinants, and use them to inform the development of PSBI implementation strategies during and after the pandemic.

Methods

Setting and health system

Ethiopia's health system has primary level care (encompassing a primary hospital, health centers, and health posts), secondary level care, and tertiary level care [22]. Primary health care provides preventive and

For peer review only

promotive community and outreach services through the expansion of the HEP and the engagement of community volunteers [22].

In the past decade, Ethiopia has developed community-based strategies and programs to ensure newborn and child survival. These include the 2010 introduction of community management of pneumonia through integrated community case management of childhood illnesses and newborn care (iCCM), and community-based newborn sepsis management through CBNC, incorporated in 2012 within the iCCM platform to improve the use of iCCM services for newborns and young infants [7]. In 2017, national efforts were made to fully integrate the continued implementation of iCCM and CBNC to strengthen the delivery of quality maternal, newborn, and child health (MNCH) services at the primary care level [23]. The iCCM program is being integrated with the continuum of maternal and child health care framework from pregnancy to childhood, and from household to health facilities employing the "Four Cs Model": (1) prenatal and postnatal **Contact** with the mother and newborn; (2) **Case-identification** of newborn with signs of PSBI; (3) **Care**, or treatment that is appropriate and initiated as early as possible; and (4) **Completion** of a full seven-day course of appropriate antibiotics [23].

The study was conducted in Dembecha and Lume woreda of Amhara and Oromia regions, respectively. The two woredas are comprised of 11 health centers, 66 health posts, and an estimated 250,000 people.

Study design and data

The overall study is multiple methods using the Implementation Research Logic Model (IRLM), a combination of RE-AIM and CFIR [24] to understand the complex challenges to PSBI implementation before and during the pandemic, how implementation strategies need strengthening or adaptation and to influence quality delivery of PSBI and improve implementation outcomes, and how implementation strategies were designed, reported and synthesized to determine outcomes and learnings. The IRLM is an implementation research framework that combines a number of existing frameworks to link contextual factors to strategies through mechanisms

For peer review only

to targeted selected outcomes. We report on the results of the formative multiple methods cross-sectional results.

Data were collected from 18 April-24 May 2021. The quantitative component of the study was a cross-sectional population-based study interviewing parturient women who gave live birth 2-14 months before data collection and/or other caregivers of infants aged 2-14 months old as of April-May 2021. A structured questionnaire translated into local languages (Amharic and Oromifa) was used to capture the data (Additional file 1). The quantitative component also included health post (HP) assessments and self-administered interviews with HEWs available on the day of the visit. The HP assessment was conducted using an adapted tool from the WHO Service Availability and Readiness Assessment (SARA) [22].

The survey was used to collect information about household and socio-demographic characteristics of mothers, awareness of PSBI, and experiences related to the use of maternal and newborn health services across the continuum of care. Prior to data collection, interviewers, supervisors, and coordinators were thoroughly trained during a one-day training on the questionnaire. A pre-test of research instruments was also conducted. The data were collected and archived using a web-based mHealth platform (i.e., SurveyCTO) using smartphones. Survey supervisors and coordinators monitored and supervised the fieldwork, and ensured data quality by randomly revisiting selected households to validate responses.

The qualitative component employed a programmatic qualitative study using the Consolidated Framework for Implementation Research (CFIR) as included in the IRLM [25]. The framework was used to understand factors that influenced implementation outcomes, including inner (characteristics of the health system), intervention, actor (provider and caregivers), and outer settings. We used a semi-structured questionnaire, to conduct in-depth interviews (IDI) (Additional file 3) with health facility workers, HEWs, and program managers at each level of the health system, as well as with caretakers and community volunteers in the intervention kebeles. Interviews were conducted in Amharic and Oromiffa by experienced qualitative

For peer review only

research consultants at a convenient place in the community, such as a community hall or the respondent's house.

Sample size and sampling technique

In the household survey, 4,262 mothers with infants 2-14 months were identified and interviewed. During the interview, we identified 508 sick young infants and 371 severe neonatal infection cases.

Our study contains the following assumptions: of 3% estimated annual live birth rate, 5% prevalence of newborn sepsis, and 56% of newborn sepsis cases sought medical care [26], an estimated 375 neonatal sepsis cases and 210 cases sought medical care could be obtained in the study woredas (out of an estimated population of 250,000). To calculate the sample size we assumed a 95% confidence level ($Z\alpha/2=1.96$), power of 80%, and design effect of 1.5, given that 56% of mothers sought medical care for ill babies in the neonatal period [26]. Accordingly, all 66 kebeles were included in the study and relying on a census, we identified and interviewed mothers with infants 2-14 months. The household survey sample size was deemed adequate as it was estimated to double population formula for a comparative before and after cross-sectional study design with sufficient power to detect a 20% change in sick newborn care-seeking behavior due to the intervention.

We recruited all eligible households with children under-15 months of age from updated lists obtained from the Community Health Information System (CHIS) of all 66 HPs, which included information on households with under-15 month infants and their unique household identifiers. These records were available at HPs and stored in numeric order. When a complete list of households was not available at HPs, an ad hoc list of households was established by data collectors with the help of the HEWs, Women Development Armies (WDAs), and community volunteers before data collection.

For the qualitative component of the study, we conducted 34 IDIs including program managers (6), development partners (4), service providers (16), and WDA volunteers (8).

For peer review only

Measurement

The dependent variable of interest was the proportion of women who sought medical care from appropriate providers for their sick young infants during the first 2 months of life. Sick young infants were defined as those whose mothers' reported symptoms of severe infection, including difficulty breathing, chest in-drawing, no longer feeding well, being unusually hot or cold, being less active than usual, and/or having convulsions during the first two months of life. The independent socio-demographic and obstetric characteristic variables included: maternal education, household wealth index, number of children, distance of residence from a health facility, utilization of ANC services, whether complete ANC was received, place of delivery, administrative residence (woreda), and level of fear of COVID-19 and its impact on seeking care.

ANC services were considered complete if the mother received blood pressure measured, weight taken, blood and urine tested during the pregnancy of their gest infant. A wealth index score was constructed for each household with the principal component analysis of the household's possessions (electricity, watch, radio, television, mobile phone, telephone, refrigerator, table, chair, bed, electric stove, and kerosene lamp), and household characteristics (type of latrine, water source, floor and wall materials). Subsequently, households were ranked according to wealth score and then divided into five quintiles using the Principal Component Analysis [27]. Mothers' fear of COVID-19 was measured by taking the maximum value of yes/no responses to each of the following questions: fear of infection during ANC or a delivery visit, fear of fetus/baby being infected during ANC or delivery visit, or fear of spreading infection. This variable was measured for those who delivered after the first case of COVID-19 was reported in Ethiopia in March 2020.

Data analysis

Quantitative data were analyzed using Stata version 15.1. Socio-demographic data were summarized by frequency tables and summary statistics. Random-intercept logistic models were fitted to estimate associations between the individual and community variables, and the likelihood of seeking care for PSBI. This enabled the probability of PSBI care-seeking to vary randomly across communities based on the

For peer review only

182 assumption that the effects of individual characteristics were the same in each community, (i.e., the
183 coefficients of all explanatory variables were fixed across communities using *xtlogit* and *xtmelogit* Stata
184 commands). The null model, Model 1, was fitted without the explanatory variables. In Model 2, intercept
185 model was fitted with level 1 and level 2 variables. In Model 3, a cross-level interaction between woreda of
186 residence and distance to the health centers was fitted to reveal any evidence of effect modification of the
187 association between distance to the health center and PSBI care-seeking by woreda of residence. Explanatory
188 variables were identified based on the CFIR and RE-AIM (Reach, Effectiveness, Adoption, Implementation,
189 and Maintenance) frameworks included in the models. We present the adjusted odds ratios and confidence
190 intervals at the 95% level.

191 Models' goodness-of-fit tests were completed using the global Wald test, and the regression diagnostic
192 Akaike Information Criterion (AIC) was used to determine the suitability of the model.

193 Qualitative interviews were audio-taped and transcribed verbatim, and the transcript texts were exported to
194 ATLAS.ti software for thematic analysis. We conducted familiarization, coding, categorizing, identifying
195 themes, and interpretation of data. Both deductive and inductive coding approaches were applied starting
196 with a codebook reflecting the components of the IRLM (determinants, strategies, outcomes). Data were
197 iteratively read and subsequently classified into one of the codes.

198 Results

199 A total of 4,262 mothers of children aged 2-14 months were interviewed from the household survey. More
200 than two-thirds of the respondents were 20-34 years old. The majority did not have any education (56%) and
201 most lived two hours or more walking distance from a health center (Table 1).

202 *Table 1: Background characteristics of the household survey participants, 2021*

Background characteristics	Frequency	Percent
**Age of the mother**		
15-19	211	5.0

20-34	2,943	69.0
35-49	1,108	26.0
**Maternal education**		
Cannot read/no school	2,365	55.5
Primary	853	20.0
Secondary and higher	1,044	24.5
**Wealth quintile**		
Most poor	805	18.9
More poor	827	19.4
Poor	849	19.9
Less poor	879	20.6
Least poor	903	21.2
**Number of children**		
1	1,043	24.5
2-3	1,565	36.7
4+	1,653	38.8
**Distance to health center (walking distance)**		
<2 hrs.	534	12.5
>=2 hrs.	3,728	87.5
**Woreda**		
Lume	1,768	41.5
Dembecha	2,494	58.5
**Total # of respondents**	**4,262**	100.0

Care-seeking for neonatal illness

Twelve (11.6%) percent [95% CI: 9.8-13.7] of mothers reported a history of illness in their youngest child during their neonatal period with 8.4% [95% CI: 7.0-10.0-] reporting symptoms of severe infection. A little more than half of mothers sought formal medical care, 57.6% [95% CI: 47.2-67.4] for any illness and 56.4% [95% CI: 45.5-66.7] for severe infection, primarily from health centers (Fig. 1).

Barriers to delivery of PSBI services: COVID-19 and other pre-existing conditions

PSBI service registers from the 66 HPs showed a decline SYI HP visits from 1,675 cases in 2019 to 538 in 2021. Interview participants also noticed a general decline in PSBI performance and an overall decline in the HEP. *"The PSBI service is declining because of the declining of the overall Health Extension Program"* [IDI-PM-MoH01]. Respondents highlighted several factors that caused low uptake and delivery of PSBI services, including 1) the COVID-19 pandemic, 2) low confidence and competence of HEWs, 3) complexity of the intervention, 4) closure and poor infrastructure of HPs, 5) unavailability of essential drugs, 6) weak support and linkage system, and 7) suboptimal functionality of the WDA.

For peer review only

Influence of COVID-19 on maternal, newborn, and child health care-seeking and delivery

The qualitative analysis found that the COVID-19 pandemic had a detrimental impact on care-seeking and delivery of PSBI services. We found that program managers and service providers perceived that the pandemic generally affected all child health services, including PSBI activities related to generating awareness, or social and behavioral change communication (SBCC) efforts such as interrupted social gatherings, community meetings, and community sensitization programs, particularly in the first 2-3 months of the pandemic in Ethiopia.

> *"The pandemic has affected all activities related to awareness creation or SBCC efforts. It affected advocacy works, community sensitization, pregnant women conferences, and other activities in that it made things very stagnant or not moving forward" [IDI-PM-MoH02].*

One-third of mothers from the household survey reported fear of getting an infection in seeking maternal and newborn care (Fig. 2)

Low confidence and competence of HEWs

Survey was completed in the 79 of 117 (68%) HEWs that were available at the time of data collection. We found that only 41% knew all signs of PSBI. This finding was confirmed by program managers and service providers also perceived that low competence and confidence levels of HEWs to administer curative services were barriers to quality PSBI services.

> *"The Health Extension Workers' confidence is low... Since they had not practiced the curative services after they received the PSBI training, they are afraid of providing services, they are rather referring cases and not promoting the PSBI service" [IDI-PM-P02].*

In-depth interview participants also reported that the community perceived that HEWs were not capable of treating newborns and that the services given at HCs or HPs were of low quality. *"Even when communities*

For peer review only

are aware of the services given by HEWs, they perceive that HEWs aren't able to treat newborns" [IDI-PM-MoH03]. As a result, the community prefers home remedies and traditional healers to health facilities.

Complexity of the PSBI interventions for HEWs

Some respondents believed that PSBI interventions were too complex for HEWs. Participants said that HEWs had difficulty with identifying danger signs, assessing and classifying cases, and administering gentamicin injections for newborns using the proper techniques. *"Health Extension Workers are challenged in how to administer antibiotics to newborns intramuscularly…they are challenged in how to assess, classify, identify treatment, and do follow-up" [IDI-PM-MoH01].* HEWs also mentioned that PSBI service implementation was too complex for them. *"The treatment guide is too complex, especially classifying the severity of the illness is too hard" [IDI-SP-HEW03].*

Health post closure and poor infrastructure

Facility closures and service interruptions affected PSBI service delivery in the rural communities studied. HEWs were responsible for many preventive and promotive services, resulting in a heavy load of community outreach responsibilities, as such, the HPs were closed.

> *"HEP activities are broad and mainly involve outreach, through home-to-home visits. As a result, the HP stays closed and mothers or other people are missing us from here. So, they conclude that HP is closed always" [IDI-SP-HEW05].*

Unpredictable HP closures affected PSBI service demand. *"…. a mother won't come again to the health post if she experienced the closure of the health post previously" [IDI-PM-P04].* In addition to outreach services, other services were interrupted when HEWs were not available at their HPs. Closures were also due to HEWs residing far away from their HPs, which affected their ability to return to a HP after their outreach activities concluded.

For peer review only

"Health posts are usually closed. HEWs may not be found at their workplace most of the time; they either live in towns or work by coming and going back home in the town. This increases the chance of the health post to be closed for many hours" [IDI-PM-P03].

A shortage of HEWs and their heavy workload affected PSBI service delivery and access to services. Only two HEWs were assigned in each kebele and tasked to provide preventive and promotive services, home visits, and curative services –tasks that are very difficult for only two people to address in a single kebele.

"Shortage of human resources is the biggest challenge. In principle, health posts shall be open 24/7. If one HEW is assigned to a health post, how can she manage to open and provide curative services while she is tasked to visit 8–9 households in a day in rural kebele?" [IDI-PM-MoH02].

Informants believed that even if HPs were accessible, their general condition affected the healthcare-seeking behaviors of the community. *"The building of health posts is sub-standard infrastructure and cannot be considered as medical service centers … this is I think one factor" [IDI-PM-RHB01].*

Erratic supply of drugs and commodities

Similar to the facility surveys, Informants affirmed that stock out of drugs was a challenge to providing PSBI treatment services. The Ethiopian health care system follows a demand-based input, or "pull" delivery system. However, there are often challenges to estimating and quantifying needs. As a result, understocked HPs could not adequately meet PSBI service delivery requirements due to inaccurate forecasting and planning, insufficient medical supplies, inconsistent supply of drugs, drug expiration, failure to request and refill drug stocks and supplies, and stock-outs.

Moreover, some essential drugs were not available in the local market, which meant that even when there was no stock in the facility, it was also not possible to procure drugs from local private sector sources. This also led to insufficient or inconsistent medical supplies and stock-outs.

For peer review only

"Some essential drugs are not available in the local market. For example, amoxicillin dispersible syrups and gentamicin injection 20mg/2ml preparation is not available" [IDI-PM-P02].

The HP assessment also demonstrated that on the day of the visit, a significant number of HPs were inadequately stocked with essential commodities. Most lacked gentamicin injection, chlorhexidine gel, and ambu-bag and mask (Fig. 3).

Weak support, integration, and facility linkages

Declining mentorship or supervision, lack of training and motivation of HEWs, and suboptimal ownership at each level were other factors attributed to weak PSBI program implementation. Some HEWs had not had any PSBI training, suggesting the need for routine training for new HEWs, as well as refresher training for established workers.

> *"There are many HEWs who did not take training and the existing HEWs like me have forgotten the training, so training is needed to enhance the newborn treatment in the health post. In addition to this regular input, supply is needed" [IDI-SP-HEW03].*

Program ownership and sustained support from the government were also found to affect PSBI services. Program support was strong and there was close follow-up when development partners were actively supporting the implementation of PSBI services. When PSBI became integrated with the national service package, PSBI service delivery was slowed due to overall system performance issues, including a lack of resources and competition with other programs.

> *"It (PSBI) is not owned by regions, zones, or woredas. A little bit is better at the ministry level. It is not equally getting focus like EPI and other child health programs. Once the development partners' support phased out, mentorship and supervision activities are quit" [IDI-PM-P01].*

The fragmented referral system was one of the challenges that led to the low uptake of PSBI services. In the referral system, weak linkages between health facilities resulted in insufficient communication, feedback, or

For peer review only

follow-up on referred cases. The mechanisms for referrals were also not consistent or well-followed and were largely paper-based, which lead to mismanagement of referred cases. We identified high fragmentation of services across the continuum of care. *"…the linkages between health post and health center became weaker and to the extent, HEWs are not provided referral slip as before" [IDI-PM-MoH02].*

The lack of free emergency ambulatory services for newborns greatly inhibited care-seeking for referred cases. "*…the newborn referral system is not clearly defined. Some woredas use an ambulance for newborn referral but most are not doing this" [IDI-PM-P02].*

Means of transportation, time, long distances, and high accommodation costs were other problems associated with the referral system.

> *"The main challenge related to the referral system is accessibility. Mostly in rural areas, there is a problem with transportation for referral, including public transportation. The other is opportunity costs associated with the referral" [IDI-PM-P01].*

For peer review only

Sub-optimal community engagement and low functionality of WDAs

Even when PSBI services were available at HPs, inadequate community-level service emerged as an important factor for the low demand and uptake of PSBI services.

> *"Community members may not be aware of the availability of newborn care at health post level because HEWs haven't fully disseminated information though they were directed to do so during PSBI trainings" [IDI-PM-MoH03].*

WDA volunteers carried out community education and mobilization activities and served as the HEWs' voice in communities. As a part of the community, they were engaged in early pregnancy identification, birth notification, health promotion, identifying and referring sick newborns, and other social support activities. However, various factors contributed to a decline in WDA functionality. These included poor support and supervision, women's low literacy and negotiation skills, the perception of low commitment, and that many

had overlapping tasks to manage. *"The role of WDA is declining over time due to the decline of the program support. ...." [IDI-PM-MoH01].*

Ideally, all households would be included in the WDA networks across all kebeles. This, however, made the structure very broad and hard to manage. *"It is difficult to manage WDA as it is huge in number..." [IDI-PM-P01].*

As volunteers, WDA members' efforts were not expected to be consistent and their contributions relied on their willingness to work. The government system was also not able to provide close follow-up and strong program support needed for the WDA members to perform at their expected level. The HEP has declined in recent years [8]and HEWs who were already stretched, were tasked with supervising WDA members. Many WDA volunteers were unable to read and understand written materials, which made it challenging to introduce learning materials requiring literacy, such as guidelines and job aids. WDA members also reported that their spouses disapproved of their work on a volunteer basis for no pay, particularly when it replaced potential income-generating work or household responsibilities. *"They may also face challenges from their spouse. Their spouses may ask them why you are serving for free and leaving our work" [IDI-PM-MoH01].*

Predictors of care-seeking for PSBI

In the null model, the intra-class correlation coefficient (ICC) showed that 23% of the residual variation in the propensity to the uptake of PSBI is attributable to unobserved community characteristics. The likelihood ratio test showed that there was strong evidence that the between-community variance was non-zero (chi2= 41.25; p <.01). Model 2, the intercept model, showed that women who received PNC within 6 weeks, and received complete ANC [i.e., weight measured, BP taken, urine and blood tested] had higher odds of care-seeking for PSBI. Women who resided in Lume woreda had higher odds of seeking care for PSBI than their counterparts in Dembecha [AOR: 2.79; 95% CI: 1.17-6.66]. Furthermore, fear of COVID-19 infection was negatively associated with uptake of care-seeking for PSBI [AOR: 0.30; 95% CI: 0.16-0.55]. However, maternal

For peer review only

education, household wealth, and knowledge of COVID-19 were not significantly associated with mothers' care-seeking for PSBI.

Model 3, the cross-level interaction model, revealed evidence of effect modification of the association between distance to the health center (> 2 hrs. walking distance) and PSBI uptake by woreda of residence. The within-community effect of the administrative woreda in Lume disappeared, while the within-community effect of distance to the health center on the odds of receiving PSBI were significantly lower for women who resided within 2 hours walking distance to the health center as compared to women who resided further away from the health center [AOR: 0.39; 95 % CI: 0.16-0.93] (Table 2).

Table 2: Predictors of care-seeking for young infants with severe neonatal infection, multilevel logistic intercept models, 2021

Predictors	Seek care for PSBI	Model 1: Null model	Model 2: Intercept model		Model 3: Cross-level interaction	
			AOR (95% CI)	P-value	AOR (95% CI)	P-value
**Maternal education**						
Cannot read/no school	109 (50.2)		1.00		1.00	
Primary	41 (60.3)		1.47 (0.74-2.94)	0.270	1.45 (0.73-2.91)	0.294
Secondary and higher	57 (66.3)		1.48 (0.75-2.92)	0.263	1.50 (0.76-2.98)	0.247
**Wealth quintile**						
Most poor	32 (41.0)		1.00		1.00	
More poor	45 (54.9)		1.40 (0.64-3.08)	0.401	1.38 (0.63-3.04)	0.424
Poor	48 (57.8)		1.62 (0.72-3.63)	0.245	1.56 (0.69-3.53)	0.285
Less poor	35 (61.4)		1.85 (0.73-4.72)	0.198	1.77 (0.69 -4.56)	0.235
Least poor	47 (66.2)		1.34 (0.57-3.59)	0.567	1.36 (0.50-3.69)	0.549
**Received ANC**						
Yes	174 (61.1)		1.76 (0.85-3.66)	0.131	1.77 (0.85-3.71)	0.129
No	33 (38.4)		1.00		1.00	
**Complete ANC**						
Yes	123 (67.2)		2.08 (1.14-3.79)	0.018	2.04 (1.12-3.75)	0.021
No	84 (44.7)		1.00		1.00	
**Place of delivery**						
Home	52 (41.6)		1.00		1.00	
Facility	155 (63.0)		1.18 (0.63-2.19)	0.603	1.21 (0.65-2.27)	0.542
**Any PNC within 6 weeks**						
Yes	80 (72.7)		2.15 (1.16-3.98)	0.015	2.08 (1.12-3.87)	0.021
No	127 (48.7)		1.00		1.00	
**Fear of COVID-19**						
Yes	69 (44.2)		0.30 (0.16-0.55)	<0.01	0.27 (0.15-0.47)	<0.01
No	138 (64.2)		1.00		1.00	

For peer review only

**Distance to the health center (walking distance)**						
<=2 hrs.	47 (73.4)		1.00		1.00	
>2 hrs.	160 (52.1)		0.51 (0.23-1.12)	0.095	0.39 (0.16-0.93)	0.033
**Woreda of residence**						
Dembecha	156 (52.4)		1.00		1.00	
Lume	51 (69.9)		2.79 (1.17-6.66)	0.020	0.62 (0.09-4.23)	0.624
Distance to health facility* Woreda						
>2 hrs.*Lume					5.69 (0.74-43.50)	0.094
Community-level intercepts (SE)		1.68 (0.35)	0.69 (0.39)		0.89 (0.53)	
Random effects						
Community-level variance (SE)		0.99 (0.40)	0.58 (0.30)		0.60 (0.31)	
Log-likelihood ratio test		41.25	12.81		12.67	
Model fit statistics						
ICC (SE)		0.23 (0.07)	0.15 (0.07)		0.15 (0.07)	
AIC		472.07	442.58		441.92	

AOR: adjusted odds ratio; SE: standard error

Discussion

This study revealed a high prevalence of severe neonatal infection and low care-seeking behavior of mothers for their sick young infants in rural communities in Lume and Dembecha woredas of Ethiopia. Fear of COVID-19 infection negatively affected care-seeking for sick young infants. Out of 4,262 participants, one-third of the mothers reported fear of getting COVID-19 infection in seeking care. Conversely, receiving ANC and PNC consultations and residing more than 2 hrs. walking distance to the health center predicted care-seeking for PSBI. In addition, multiple pre-existing health system factors were identified as barriers to PSBI service delivery.

Our results on prevalence are similar to other community-based studies in Ethiopia identified a prevalence of PSBI ranging from 5-14% [11, 26, 28-30]. However, despite the availability of evidence-based, cost-effective interventions to manage neonatal infections [31], only a little more than half of the Ethiopian mothers sought medical care for ill babies in the neonatal period [26, 30].

This study shows that fear of COVID-19 infection negatively affected care-seeking for their sick young infants in rural settings of Ethiopia. Previous modeling studies [32, 33] and observational studies [18, 34-36] showed

For peer review only

that fear of contracting COVID-19 infection was the main reason for access to newborn care services and delayed health-seeking for child health services in LMICs including Ethiopia [20, 21]. Analysis of continuity of maternal and child health service delivery amid COVID-19 in eight sub-Saharan countries revealed significant service disruptions in outpatient care and child vaccinations [36]. Similarly, a cross-sectional study in Ghana and South Africa showed a significant decline in clinic attendance and hospital admissions of children during the pandemic [37, 38] indicating fear of COVID-19 might intensify delays and reduce access to newborn care. These results are similar to a rapid assessment conducted in 2020 by JSI Research & Training Institute, Inc. through The Last Ten Kilometers Project (L10K) showed that the emergence of COVID-19 affected the uptake and delivery of essential maternal and newborn health; exceptionally low coverage was observed in under-5 pneumonia treatment and under-5 outpatient attendance [39]. The qualitative component of this current study showed that the pandemic affected child health services, including PSBI in the first two-to-three months of the pandemic. It showed that the pandemic continued to affect activities related to generating awareness or SBCC efforts, such as interrupting social gatherings, community meetings, and community sensitization programs [40].

Maternal health care uptake and receipt of quality ANC services were significantly associated with care-seeking for PSBI. Evidence from other studies has shown that uptake of the continuum of care services was associated with uptake of newborn and child health services [41-43] and their survival [44]. Antepartum and postpartum care offered the opportunity for a woman to establish an informal forum to discuss the benefits of skilled care throughout their pregnancies and Child-rearing periods [22, 45]. Evidence from previous studies indicates that attending the recommended number of ANC and postpartum visits provided women with a chance to receive quality care, and more counseling during pregnancy and postpartum care regarding newborn care [46, 47]. It also improved women’s trust in providers and increased their positive experience with them, which was found to increase a woman’s odds of receiving a continuum of maternal and child health services [45].

For peer review only

Our cross-interaction model showed that woreda of residence modified the association between distance to health center and uptake of PSBI care. There was variability in care-seeking for PSBI by residence, and shorter distance to the health center was positively associated with mothers' care seeking for their sick young infants. This suggests the existence of differential geographic access to PSBI, despite the program being pro-poor, as documented in other studies regarding access to postpartum care in Ethiopia [48, 49]. In addition, despite the expansion of primary care services in Ethiopia, distance remained a major barrier for many people. Previous studies reported that distance to reach health facilities, topography, and lack of transportation deterred utilization for treating SYIs [9, 10, 50-52]. Evidence from developing countries also suggested uptake of services when they were more proximate [53]. These examples underline the need for expansion of health facilities and the design of appropriate strategies to reach disadvantaged communities and their newborns in Ethiopia.

Maternal education and household wealth were not found to determine care-seeking for PSBI. This may have related to the HEP service delivery strategy where all services, including PSBI, were provided free of charge. Previous studies in Ethiopia, however, reported maternal education [9, 52] and household wealth were associated with the use of iCCM/CBNC services [9]. This needs further exploration. In addition, further study is needed to establish the intermediate and long-term impacts of COVID-19 on community-based child health programs.

This study demonstrated the unprecedented challenges posed by the COVID-19 pandemic on PSBI care-seeking during the pandemic in Ethiopia. The following pre-existing conditions proved to be the most formidable barriers and were also identified as challenges in previous studies: poor integration of PSBI in the woreda work stream when partners' support was phased out, weak support systems, erratic supply and poor logistics management systems, low competence and motivation of HEWs, the complexity of the PSBI intervention, and low community demand [10-12, 14, 51, 54-56]. We found that these challenges could be mitigated with several strategies, including integration of PSBI in the district health system work stream,

For peer review only - http://bmjopen.bmj.com/site/about/guidelines.xhtml

424 integration of COVID-19 services with the primary health care services, continuous on-site coaching and training of HEWs, strong support system from health centers and woreda health offices, the use of participatory design and implementation methods, the strengthening of the supply chain system, and the presence of community-level SBCC activities. These approaches were effective to engage community volunteers, HEWs, and the primary health care system and in better integrating PSBI for sustained implementation and scale up.

430 Our study had limitations. Like any survey, responses were potentially influenced by recall and social desirability bias. Thus, to improve reporting, memory aids were used.

For peer review only

432 Conclusions

433 Care seeking for sick young infants is influenced by physical access, antepartum and postpartum care service delivery, health system bottlenecks, and fear of COVID-19. Accordingly, there is a need to strengthen the primary health system to be more resilient to provide quality PSBI services during and after the COVID-19 pandemic. Evidence-informed social and behavioral change communication interventions are pivotal to providing current and comprehensive information on transmission and prevention of COVID-19 in multiple and accessible formats to boost the confidence of clients and mitigate fear of COVID-19 infection. Active engagement of communities to create continuous community awareness activities, actively identify pregnancies, notify births to HEWs, identify sick newborns and link them to the HEP is critical to maintaining service delivery and reducing the negative impacts of COVID-19 on newborns health outcomes.

442 Abbreviations

ANC	antenatal care
CBNC	community-based newborn care
HEP	Health Extension Program

HEW	Health Extension Worker
iCCM	integrated community case management of childhood illnesses and newborn care
IR	implementation research
LMICs	low-and-middle-income countries
L10K	The Last Ten Kilometers Project
MNCH	maternal, newborn, and child health
NMR	neonatal mortality rate
PHCU	Primary Health Care Unit
PNC	postnatal care
PSBI	possible serious bacterial infection
SYI	sick young infant
WDA	Women Development Army
WHO	World Health Organization

For peer review only

Declarations

Ethics approval and consent to participate

Ethical clearance was obtained from the Ethiopian Public Health Association (EPHA) Research Ethics Review Board (Reference #: EPHA/OG/166/21 dated April 16, 2021) which has the mandate to review research protocols and undertakings involving humans in the region or the institution, or other institutions that do not have their own IRBs and beyond [57]. All methods were performed in accordance with the Declaration of Helsinki and National Research Ethics Review Guideline. Written permission to undertake the study was sought from the zonal health departments and institutions. The objectives, methodology, purpose of the study, and the benefits and risks of the study were explained to all study participants. Before data collection, participants were also informed of their right to voluntarily participate in the study. Informed consent was sought and documented before conducting any interviews and discussions. Because the majority of the respondents were not expected to be able to read or write; written consent was not sought. If the respondent agreed to be interviewed after listening to the consent statement, the interviewer marked the consent form as consent given below the consent statement and signed below that. The interviewer continued with the interview only after receiving and documenting consent. If the respondent was less than 18 years old, then consent was sought from her and her husband or guardian. The survey protocol submitted to the ethical review committee included the study questionnaire with the statement that described the consent-obtaining procedure. The primary investigator oriented data collectors on ethical and methodological issues and supervisors followed them throughout the data collection period to monitor any ethical breach.

To ensure the privacy of study participants, respondents were interviewed in a conducive environment; and confidentiality of the data were guaranteed by preserving the anonymity of the study participants. Individual personal identifiers were not collected, to ensure the anonymity of data and the researcher would keep the information obtained from the research participant in private or will withhold information from others, to

For peer review only

respect the confidentiality and privacy of study participants. Identifying information (names and addresses) were not included in the data collection instrument.

Consent for publication

Not applicable

Competing interests

The authors declare that they have no competing interests. The authors [GT, NF, DE, WB, and KE] work for JSI Research & Training Institute, Inc., a commercial company. We declare that this commercial affiliation does not alter our adherence to BMC policies on sharing data and materials.

Funding

The article write-up and publication fee were supported by the Bill & Melinda Gates Foundation, Investment Number INV-024241. JSI Research & Training Institute, Inc. has supported us in the form of salaries for authors [GT, DE, NZ, WB, and KE]. However, none of the funders have a role in study design, data collection, analysis, decision to publish, or preparation of the manuscript.

Availability of data and materials

The dataset used and analyzed during this study is included as supplementary information to this article (Additional files 2 and 4).

Authors' contributions

GT, NF, DE, KE, and WB: conceived the study design and methods. GT, NF, DE, KE, WB, and LH: formulated the research question, analysis method, interpretation, and critical review. GT: drafted the manuscript. WB: received the grant. All authors read and approved the final manuscript.

Acknowledgments

We would like to thank the Bill & Melinda Gates Foundation (BMGF) for funding this implementation research. The implementation of this survey would not have been possible without the support of the

For peer review only

Ministry of Health and Amhara and Oromia Regional Health Bureaus. We acknowledge the interviewers and the supervisors for their hard work, dedication, and for accomplishing the fieldwork on schedule. Finally, we take this opportunity to extend our gratitude to all study participants who took their time to respond to the survey questionnaires and provide us with invaluable information. Alexandra Kamberos is acknowledged for editing the manuscript.

References

1. Ezbakhe F, Pérez-Foguet A. Child mortality levels and trends. Demographic Research. 2020;43:1263-96.
2. Ethiopian Public Health Institute (EPHI) [Ethiopia], ICF. Ethiopia Mini Demographic and Health Survey 2019: Final Report. Rockville, Maryland, USA: EPHI and ICF; 2021.
3. Ahmed I, Ali SM, Amenga-Etego S, Ariff S, Bahl R, Baqui AH, et al. Population-based rates, timing, and causes of maternal deaths, stillbirths, and neonatal deaths in south Asia and sub-Saharan Africa: a multi-country prospective cohort study. The Lancet Global Health. 2018;6(12):e1297-e308.
4. Seale AC, Blencowe H, Manu AA, Nair H, Bahl R, Qazi SA, et al. Estimates of possible severe bacterial infection in neonates in sub-Saharan Africa, south Asia, and Latin America for 2012: a systematic review and meta-analysis. The Lancet infectious diseases. 2014;14(8):731-41.
5. WHO. Guideline: managing possible serious bacterial infection in young infants when referral is not feasible. World Health Organization; 2015.
6. Wammanda RD, Adamu SA, Joshua HD, Nisar YB, Qazi SA, Aboubaker S, et al. Implementation of the WHO guideline on treatment of young infants with signs of possible serious bacterial infection when hospital referral is not feasible in rural Zaria, Nigeria: Challenges and solutions. PloS one. 2020;15(3):e0228718.
7. MOH. National Strategy for Newborn and Child Survival in Ethiopia: 2016-2020. Addis Ababa, Ethiopia: Ministry of Health, Ethiopia; 2015.
8. MOH. Realizing Universal Health Coverage through Primary Health Care: A Roadmap for Optimizing the Ethiopian Health Extension Program 2020 - 2035. Addis Ababa, Ethiopia: Ministry of Health, Ethiopia; 2020.
9. Gebremedhin T, Atnafu A, Dellie E. Community-based newborn care utilisation and associated factors in Geze Gofa rural district, South Ethiopia: a community-based cross-sectional study. BMJ Open. 2020;10(8):e037792. doi: 10.1136/bmjopen-2020-037792.
10. Tareke KG, Lemu YK, Feyissa GT. Exploration of facilitators of and barriers to the community-based service utilization for newborn possible serious bacterial infection management in Debre Libanos District, Ethiopia: descriptive qualitative study. BMC pediatrics. 2020;20(1):1-14.
11. Leul A, Hailu T, Abraham L, Bayray A, Terefe W, Godefay H, et al. Innovative approach for potential scale-up to jump-start simplified management of sick young infants with possible serious bacterial infection when a referral is not feasible: Findings from implementation research. PloS one. 2021;16(2):e0244192.
12. Gebretsadik A, Melaku N, Haji Y. Community Acceptance and Utilization of Maternal and Community-Based Neonatal Care Services Provided by Health Extension Workers in Rural Sidama Zone: Barriers and Enablers: A Qualitative Study. Pediatric health, medicine and therapeutics. 2020;11:203-17. doi: 10.2147/phmt.S254409.
13. Tareke KG, Lemu YK, Yidenekal SA, Feyissa GT. Community's perception, experiences and health seeking behavior towards newborn illnesses in Debre Libanos District, North Shoa, Oromia, Ethiopia: Qualitative study. PLoS One. 2020;15(1):e0227542. doi: 10.1371/journal.pone.0227542.

For peer review only

14. Asfaha MD, Comeau DL, Spangler SA, Spratt BL, Alamineh L, Gobezayehu AG, et al. Neonatal care and community-level treatment seeking for possible severe bacterial infection (PSBI) in Amhara, Ethiopia. BMC health services research. 2020;20:1-12.
15. Onarheim KH, Moland KM, Molla M, Miljeteig I. 'I wanted to go, but they said wait': Mothers' bargaining power and strategies in care-seeking for ill newborns in Ethiopia. PLOS ONE. 2020;15(6):e0233594. doi: 10.1371/journal.pone.0233594.
16. Worldometer: Coronavirus (COVID-19) statistics-Ethiopia. https://www.worldometers.info/coronavirus/country/ethiopia (22). Accessed 02/22/2022.
17. EPHI: COVID-19 Data Anaytics and Modeling Platform. https://vizhub.ephi.gov.et/covid-19/covid_19_comments_mini (2022). Accessed 02/22/2022.
18. Kc A, Gurung R, Kinney MV, Sunny AK, Moinuddin M, Basnet O, et al. Effect of the COVID-19 pandemic response on intrapartum care, stillbirth, and neonatal mortality outcomes in Nepal: a prospective observational study. The Lancet Global Health. 2020;8(10):e1273-e81. doi: https://doi.org/10.1016/S2214-109X(20)30345-4.
19. Rao SPN, Minckas N, Medvedev MM, Gathara D, Y NP, Seifu Estifanos A, et al. Small and sick newborn care during the COVID-19 pandemic: global survey and thematic analysis of healthcare providers' voices and experiences. BMJ Glob Health. 2021;6(3). doi: 10.1136/bmjgh-2020-004347.
20. Estifanos AS, Kazmi K, Morris SK. Could COVID-19 Reverse the Modest Gains Made in Newborn Health in Ethiopia? Maternal and Child Health Journal. 2021;25(6):849-54.
21. Gebreegziabher SB, Marrye SS, Kumssa TH, Merga KH, Feleke AK, Dare DJ, et al. Assessment of maternal and child health care services performance in the context of COVID-19 pandemic in Addis Ababa, Ethiopia: evidence from routine service data. Reproductive Health. 2022;19(1):42. doi: 10.1186/s12978-022-01353-6.
22. MOH. Ethiopia Health Sector Transformation Plan (2015/16-2019/2020). Addis Ababa, Ethiopia: Ministry of health, Ethiopia; 2015.
23. MOH. National Implementation Guideline For Integrated Community Case Management of Childhood Illnesses and Newborn Care. Addis Ababa, Ethiopia: Ministry of Health, Ethiopia; 2017.
24. Smith JD, Li DH, Rafferty MR. The Implementation Research Logic Model: a method for planning, executing, reporting, and synthesizing implementation projects. Implementation science : IS. 2020;15(1):84. doi: 10.1186/s13012-020-01041-8.
25. Damschroder LJ, Aron DC, Keith RE, Kirsh SR, Alexander JA, Lowery JC. Fostering implementation of health services research findings into practice: a consolidated framework for advancing implementation science. Implementation Science. 2009;4(1):50. doi: 10.1186/1748-5908-4-50.
26. Berhanu D, Okwaraji YB, Defar A, Bekele A, Lemango ET, Medhanyie AA, et al. Does a complex intervention targeting communities, health facilities and district health managers increase the utilisation of community-based child health services? A before and after study in intervention and comparison areas of Ethiopia. BMJ open. 2020;10(9):e040868.
27. Filmer D, Pritchett LH. Estimating wealth effects without expenditure data—or tears: an application to educational enrollments in states of India. Demography. 2001;38(1):115-32.
28. Berhane M, Girma T, Tesfaye W, Jibat N, Abera M, Abrahim S, et al. Implementation research on management of sick young infants with possible serious bacterial infection when referral is not possible in Jimma Zone, Ethiopia: Challenges and solutions. PLOS ONE. 2021;16(8):e0255210. doi: 10.1371/journal.pone.0255210.
29. Berhanu D, Avan B. Community based newborn care: quality of CBNC programme assessment-midline evaluation report. London: IDEAS, London School of Hygiene & Tropical Medicine; 2017.
30. Berhanu D, Avan B. Community Based Newborn Care Baseline Survey Report Ethiopia. London: IDEAS, London School of Hygiene & Tropical Medicine; 2014.
31. Lassi ZS, Bhutta ZA. Community-based intervention packages for reducing maternal and neonatal morbidity and mortality and improving neonatal outcomes. The Cochrane database of systematic reviews. 2015(3):Cd007754. doi: 10.1002/14651858.CD007754.pub3.

For peer review only

32. Roberton T, Carter ED, Chou VB, Stegmuller AR, Jackson BD, Tam Y, et al. Early estimates of the indirect effects of the COVID-19 pandemic on maternal and child mortality in low-income and middle-income countries: a modelling study. The Lancet Global Health. 2020.
33. Riley T, Sully E, Ahmed Z, Biddlecom A. Estimates of the potential impact of the COVID-19 pandemic on sexual and reproductive health in low-and middle-income countries. Int Perspect Sex Reprod Health. 2020;46:46.
34. Goyal M, Singh P, Singh K, Shekhar S, Agrawal N, Misra S. The effect of the COVID-19 pandemic on maternal health due to delay in seeking health care: Experience from a tertiary center. International journal of gynaecology and obstetrics: the official organ of the International Federation of Gynaecology and Obstetrics. 2021;152(2):231-5. doi: 10.1002/ijgo.13457.
35. Medeiros JPB, Neves ET, Pitombeira MGV, Figueiredo SV, Campos DB, Gomes ILV. Continuity of care for children with special healthcare needs during the COVID-19 pandemic. Revista brasileira de enfermagem. 2021;75(2):e20210150. doi: 10.1590/0034-7167-2021-0150.
36. Shapira G, Ahmed T, Drouard SHP, Amor Fernandez P, Kandpal E, Nzelu C, et al. Disruptions in maternal and child health service utilization during COVID-19: analysis from eight sub-Saharan African countries. Health policy and planning. 2021;36(7):1140-51. doi: 10.1093/heapol/czab064.
37. Abdul-Mumin A, Cotache-Condor C, Bimpong KA, Grimm A, Kpiniong MJ, Yakubu RC, et al. Decrease in Admissions and Change in the Diagnostic Landscape in a Newborn Care Unit in Northern Ghana During the COVID-19 Pandemic. Frontiers in pediatrics. 2021;9:642508. doi: 10.3389/fped.2021.642508.
38. Jensen C, McKerrow NH. Child health services during a COVID-19 outbreak in KwaZulu-Natal Province, South Africa. South African Medical Journal. 2021;111(2):114-9. doi: doi:10.7196/SAMJ.2021.v111i2.15243.
39. L10K. Responsiveness to COVID-19 pandemic and its Immediate Impacts on Essential RMNCH Services in Addis Ababa: Findings of a rapid assessment. JSI Research and Training Institute Inc./The Last Ten Kilimeters Project2020.
40. L10K, MOH. Maintaining high-quality treatment of neonatal sepsis amid COVID-19 in Ethiopia: Formative assessment findings from an implementation research. Addis Ababa, Ethiopia: The Last Ten Kilometers Project/ JSI Research and Training Institute Inc. and Ministry of Health, Ethiopia; 2021.
41. Tiruneh GT, Demissie M, Worku A, Berhane Y. Community's experience and perceptions of maternal health services across the continuum of care in Ethiopia: A qualitative study. Plos one. 2021;16(8):e0255404.
42. Usman M, Anand E, Siddiqui L, Unisa S. Continuum of maternal health care services and its impact on child immunization in India: an application of the propensity score matching approach. Journal of Biosocial Science. 2021;53(5):643-62.
43. James KS, Mishra US, undefined R, Pallikadavath S. Sequential impact of components of maternal and child health care services on the continuum of care in India. Journal of Biosocial Science. 2021:1-23. doi: 10.1017/S002193202100016X.
44. Usman M, Banerjee A, Srivastava S. Association between maternal health continuum of care and child Survival: Evidence from a population based survey. Children and Youth Services Review. 2021;128:106134. doi: https://doi.org/10.1016/j.childyouth.2021.106134.
45. WHO. WHO recommendations on antenatal care for a positive pregnancy experience. World Health Organization; 2016.
46. Mageda K, Mmbaga EJ. Prevalence and predictors of institutional delivery among pregnant mothers in Biharamulo district, Tanzania: a cross-sectional study. Pan African Medical Journal. 2015;21(1).
47. Doctor HV, Nkhana-Salimu S, Abdulsalam-Anibilowo M. Health facility delivery in sub-Saharan Africa: successes, challenges, and implications for the 2030 development agenda. BMC Public Health. 2018;18(1):765. doi: 10.1186/s12889-018-5695-z.
48. Angore BN, Tufa EG, Bisetegen FS. Determinants of postnatal care utilization in urban community among women in Debre Birhan Town, Northern Shewa, Ethiopia. J Health Popul Nutr. 2018;37(1):10. doi: 10.1186/s41043-018-0140-6.

For peer review only

49. Tiruneh GT, Worku A, Berhane Y, Betemariam W, Demissie M. Determinants of postnatal care utilization in Ethiopia: a multilevel analysis. BMC pregnancy and childbirth. 2020;20(1):1-12.
50. Tefera W, Tesfaye H, Bekele A, Kayessa E, Waltensperger KZ, Tadesse Y, et al. Illness recognition, home care, and care-seeking for sick infants less than two months of age in Shebedino District, Sidama Zone, Ethiopia. Ethiopian Medical Journal. 2014;52:157-61.
51. Gebremedhin T, Daka DW, Alemayehu YK, Yitbarek K, Debie A. Process evaluation of the community-based newborn care program implementation in Geze Gofa district, south Ethiopia: a case study evaluation design. BMC Pregnancy and Childbirth. 2019;19(1):492. doi: 10.1186/s12884-019-2616-9.
52. Samuel S, Arba A. Utilization of Integrated Community Case Management Service and Associated Factors Among Mothers/Caregivers Who Have Sick Eligible Children in Southern Ethiopia. Risk Management and Healthcare Policy. 2021;14:431.
53. Sakeah E, Aborigo R, Sakeah JK, Dalaba M, Kanyomse E, Azongo D, et al. The role of community-based health services in influencing postnatal care visits in the Builsa and the West Mamprusi districts in rural Ghana. BMC Pregnancy Childbirth. 2018;18(1):295. doi: 10.1186/s12884-018-1926-7.
54. Taylor ME. The Four Cs in Ethiopia: Framing a Community Approach to Management of Possible Serious Bacterial Infection in Neonates. Ethiopian Medical Journal. 2019(3).
55. Okwaraji YB, Hill Z, Defar A, Berhanu D, Wolassa D, Persson LÅ, et al. Implementation of the 'Optimising the Health Extension Program'Intervention in Ethiopia: A Process Evaluation Using Mixed Methods. International journal of environmental research and public health. 2020;17(16):5803.
56. Kahsay AB, Luel A, Hailu T, Mulugeta A. Referral practices on community based management of sick young infants with Possible Serious Bacterial Infection in Tigrai, Ethiopia: the caregivers' perspectives. East African Journal of Health Sciences. 2019;1(2):128-37.
57. MoST. National Ethics Review guideline. 5 ed: Ministry of Science and Technology; 2014.

For peer review only

Supporting Information

Additional file 1: Survey questionnaire.xlsx. A household survey questionnaire we used to collect information from study participants. The first sheet contains variable definitions (data dictionary) in English and local languages, and the second sheet contains variable answer choices.

Additional file 2: Survey dataset.xls. This is household survey data with variables and the values we used for this analysis.

Additional file 3: In-depth interview guides.docx. These are interview guides for program managers, service providers, and community volunteers

Additional file 4:Transcripts.doc. These are the qualitative interview transcripts we used for this analysis.

For peer review only

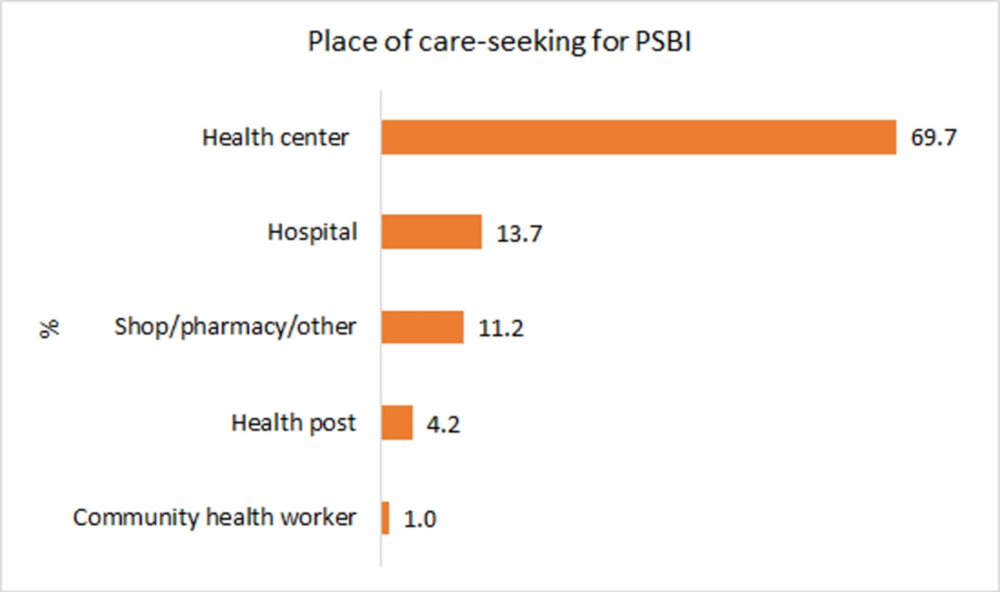

Figure 1: Place of care-seeking for the PSBI cases, 2021

119x70mm (300 x 300 DPI)

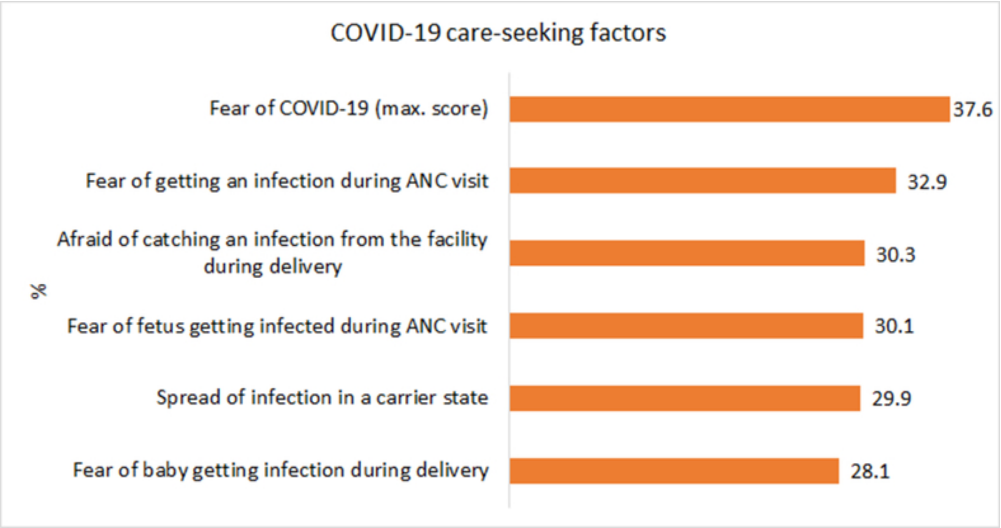

Figure 2: Fear of COVID-19 to seek maternal and newborn health services, 2021

66x35mm (300 x 300 DPI)

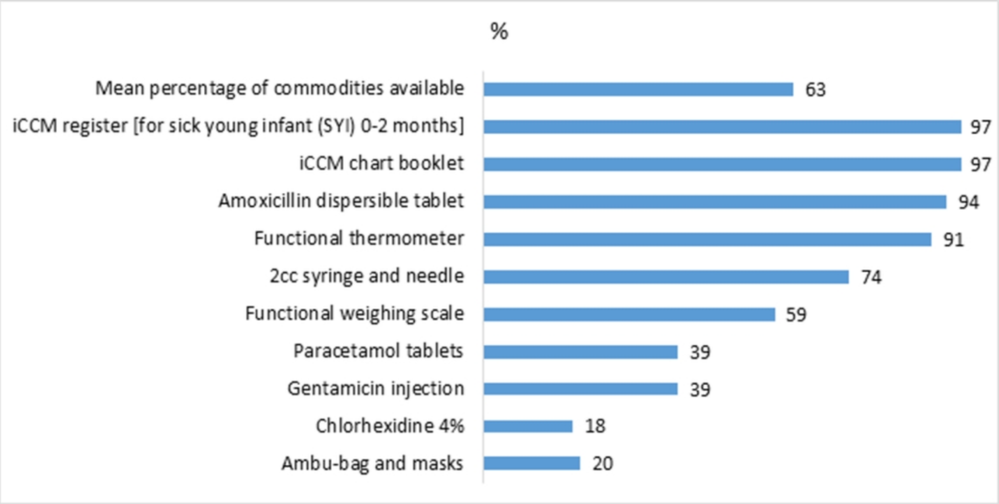

Figure 3: Availability of essential commodities at surveyed HPs, 2021

169x85mm (236 x 236 DPI)

note	intronote
note	section1
integer	q101_respnumb
date	q106
geopoint	gpslocation
note	consent_note
select_one yesno	consent
begin group	consented
select_one yesno	alive
text	q107
select_one gender	q108
integer	q109
select_one yesno	q111
integer	q112
integer	q114
note	section2
integer	q200
select_one yesno	q201
select_one yesno	q202
select_one grade	q203
select_one religion	q204
select_one married	q205
select_one yesno	q206
select_one yesno	q207
select_one grade	q208
integer	q210
integer	q211
integer	q213
select_one yesno	q214
integer	q215
select_one watersource	q217
select_one yesno	q220
select_one treat	q221
select_one toilet	q222
select_one roof	q223
select_one wall	q224
integer	q225
select_one yesno	q226
decimal	q227
select_one cookingfuel	q228
begin group	asset
select_one yesno	q230a
select_one yesno	q230b
select_one yesno	q230c

select_one yesno	q230d
select_one yesno	q230e
select_one yesno	q230f
select_one yesno	q230g
select_one yesno	q230h
select_one yesno	q230i
select_one yesno	q230j
select_one yesno	q230k
select_one yesno	q230l
select_one yesno	q230m
select_one yesno	q230n
end group	asset
begin group	q232
integer	q232a
integer	q232b
integer	q232c
integer	q232d
integer	q232e
integer	q232f
integer	q232g
integer	q232h
integer	q232i
end group	q232
note	Section3
decimal	q300hph
decimal	q300hch
select_one yesnodk	q301
select_one satisfy	q302
select_one yesnodk	q303
select_one yesno	q304
select_one yesno	q305
select_one HADtype	q306
select_one mapavl	q307
select_one yesnodk	q308
select_one yesno	q309
select_one yesno	q310
begin group	problemchild
select_one problem	q311a
select_one problem	q311b
select_one problem	q311c
select_one problem	q311d
select_one problem	q311e
select_one problem	q311f
select_one problem	q311g

select_one problem	q311h
select_one problem	q311i
select_one problem	q311j
select_one problem	q311k
end group	problemchild
begin group	problemchildCOVID
select_one problem	q311l
select_one problem	q311m
select_one problem	q311n
select_one problem	q311o
select_one problem	q311p
select_one problem	q311q
select_one problem	q311r
select_one problem	q311s
select_one problem	q311t
select_one problem	q311u
end group	problemchildCOVID
select_one yesno	q313
select_one havefhc	q314
select_one yesno	q318
select_one yesnodk	q324
begin group	q325
select_one yesno	q325a
select_one yesno	q325b
select_one yesno	q325c
select_one yesno	q325d
select_one yesno	q325e
end group	q325
select_one yesno	q326
note	Section4
select_one yesnodk	q403
select_multiple isource	q404
select_one yesnodk	q405
select_one yesnodk	q406
integer	q407
select_one yesno	q410
integer	q411
select_one yesnodk	q412
integer	q413
select_multiple ancsource	q414
integer	q416
integer	q417
select_one yesno	q418

integer	q419
integer	q420
select_one transport	q421
select_one satisflevel	q422
begin group	q434
select_one yesnodk	q434a
select_one yesnodk	q434b
select_one yesnodk	q434c
select_one yesnodk	q434d
select_one yesnodk	q434e
select_one yesnodk	q434f
select_one yesnodk	q434g
select_one yesnodk	q434h
select_one yesnodk	q434i
select_one yesnodk	q434j
select_one yesnodk	q434k
select_one yesnodk	q434l
select_one yesnodk	q434m
select_one yesnodk	q434n
end group	q434
begin group	q435
select_one yesnodk	q435a
select_one yesnodk	q435b
select_one yesnodk	q435c
select_one yesnodk	q435d
select_one yesnodk	q435e
select_one yesnodk	q435f
select_one yesnodk	q435g
select_one yesnodk	q435h
select_one yesnodk	q435i
select_one yesnodk	q435j
select_one yesnodk	q435k
select_one yesnodk	q435l
select_one yesnodk	q435m
select_one yesnodk	q435n
select_one yesnodk	q435o
end group	q435
select_one yesnodk	q436
select_one peat	q441
select_one yesnodk	q442
integer	q443
select_one yesnodk	q444
note	bpreparedness

For peer review only - http://bmjopen.bmj.com/site/about/guidelines.xhtml

select_one yesno	q445
select_multiple bprepare	q446
select_multiple pregcomp	q447
select_one yesno	q448
select_multiple pregcomp	q449
note	birthing
select_one plandelp	q500
select_multiple whyathome	q500a
select_multiple whyatHF	q500b
select_one yesnodkmwh	q501
select_multiple transport	q502
select_multiple transport	q503
select_one dassistp	q504
select_one yesno	q505
select_one yesno	q506
integer	q507
select_one yesno	q507a
integer	q508
begin group	q509
select_one yesno	q509imm
end group	q509
select_one fmilk	q510
select_one yesnodk	q511
decimal	q512
select_one bsize	q514
select_one ga	q514a
select_one cut	q515
select_one cordtie	q516
select_one yesnodk	q517
select_one yesnodk	q518
integer	q519
select_one yesnodk	q520
select_one yesnodk	q521
integer	q522
select_one yesnodk	q523
integer	q524
select_one yesnodk	q525
select_one babyplaced	q526
select_one yesnodk	q527
select_multiple babybreath	q528
select_one wbreath	q529
integer	q530
select_one yesnodk	q531

For peer review only

select_multiple gdrink	q532
select_one yesnodknr	q533
select_multiple bwarm	q534
select_one skin	q535
select_one sleep	q536
select_multiple dangercb	q537
select_one yesno	q538
select_multiple gofcomp	q539
select_one yesnodk	q540
select_one yesno	q541
select_multiple gofcomp	q542
select_one satisflevel	q543
note	postnatal
select_one yesnodk	q600
select_one yesnodk	q601
integer	q602
select_one yesnodk	q603
begin group	mompnc
select_one yesno	q604
integer	q605
select_one pnccheckplace	q606
select_one whodidpnc	q607
integer	q607x
select_one yesno	q608
integer	q609
select_one pnccheckplace	q610
select_one whodidpnc	q611
select_one yesno	q612
integer	q613
select_one pnccheckplace	q614
select_one whodidpncmom	q615
select_multiple pnccompomom	q616
select_one satisflevel	q617
end group	mompnc
begin group	babyPNC
select_one yesno	q618
integer	q619
select_one pnccheckplace	q620
select_one whodidpnc	q621
integer	q621x
select_one yesno	q622
integer	q623
select_one pnccheckplace	q624

select_one whodidpnc	q625
select_one yesno	q626
integer	q627
select_one pnccheckplace	q628
select_one whodidpnc	q629
select_multiple pnccompobaby	q630
select_one satisflevel	q631
select_one yesno	q632
integer	q633
select_one yesno	q634
integer	q935
select_one yesno	q636
integer	q637
end group	babyPNC
select_one satisflevel	q638
note	newbornhseeking
select_one KAPlevel	q639
select_one KAPlevel	q640
select_multiple dangerpnc	q641
select_one yesno	q642
select_multiple dangerpnc	q643
note	section5
select_one yesnodk	q705
select_multiple itemforbaby	q706
select_multiple reasonfornonBF	q707
select_one yesno	q708
select_multiple stopbf	q709
integer	q710
integer	q711
select_one yesnodk	q712
select_one agefeed	q713
integer	q714
select_multiple feed	q715
select_one yesno	q716
select_one yesno	q717
select_one yesnodk	q718
select_one yesnodk	q719
select_one vita	q720
note	section6
begin group	syi
select_one yesno	q800
select_multiple symptoms	q801
integer	q802
select_multiple problems	q803

select_one yesno	q804
integer	q806
select_multiple reason	q805
select_one where	q807
integer	q808
select_one yesno	q809
select_multiple drugs	q810
integer	q811
select_one satisflevel	q812
begin group	sat
select_one satisflevel	q812a
select_one satisflevel	q812b
select_one satisflevel	q812c
select_one satisflevel	q812d
select_one satisflevel	q812e
select_one satisflevel	q812f
select_one satisflevel	q812g
select_one satisflevel	q812h
select_one satisflevel	q812i
select_one satisflevel	q812j
end group	sat
end group	syi
begin group	death
select_multiple problem	q813
select_one yesno	q814
select_one provider	q815
select_one diagnosis	q816
end group	death
note	section7note
select_one risk	q900
select_one risk2	q901
select_multiple covidsxs	q902
select_multiple transm	q903
select_multiple ways	q904
select_multiple practice	q905
select_one yesnodk	q906
select_one incub	q907
select_one yesnodk	q908
select_one risk	q909
select_multiple correct	q910
select_one pinf	q911
note	covid
select_one yesno	q912

For peer review only - http://bmjopen.bmj.com/site/about/guidelines.xhtml

select_one yesno	q913
select_one yesno	q914
select_multiple covidimpact	q915
select_one yesno	q916
select_one yesno	q917
end group	consented

For peer review only

Maintaining high-quality treatment of neonatal sepsis amid COVID-19 in Ethiopia: F
**Section 1: Identification & consent**
Respondent number
q106. Date of data collection
location
Introduction and Consent My name is ${q105}. I am collecting data for my research
Do you agree to participate?
Is the infant born in the last 12-15 months a live?
q107. What is the name of your infant (0 to 15 months)?
q108. Sex of ${q107}
q109. What is the age of ${q107}?
q111. Do have a live birth before this infant ${q107}?
q112. What is the birth order of ${q107} (first, second, etc)?
q114. What is the age of the child elder than ${q107}?
**Section 2: Background and Household Characteristics**
q200. How old were you on your last birth day?
q201. Are you able to read or write a simple sentence?
q202. Did you ever attend normal school?
q203. What is the highest grade you completed?
q204. What is your religion?
q205. Marital status
q206. Are your husband able to read or write a simple sentence?
q207. Did your husband ever attend formal school?
q208. What is the highest grade your husband completed?
q210. How many times were you pregnant?
q211. How many times have you given birth?
q213. How many children have you given birth to in total?
q214. Have you ever had a pregnancy that didn't end up in a live birth?
q215. How many pregnancies have you had that did not end in a live birth?
q217. What is the main source of drinking water for members of your household?
q220. Do you treat your water in any way to make it safer to drink?
q221. What do you usually do to the water to make is safer to drink at home?
q222. What kind of toilet facility does most members of your household use?
q223. Main material of the dwelling roof. Record observation.
q224. Main material of the dwelling walls. Record Observation
q225. How many rooms in this household are used for sleeping? (Excluding kitchen
q226. Does any member of the household own any agricultural land?
q227. How many hectar of agricultural land do members of this household own cur
q228. What is the main source of cooking fuel?
Does the household have
Electricity
Watches or clocks
A radio

A television
A mobile telephone
A non-mobile telephone
A refrigerator
A table
A chair
Mattresses and/or beds
An electric mitad
A kerosene lamp/pressure lamp
Any cattle, sheep, or goats
Any jewelry (gold/silver)
q232. Number of livestock, herds, other farm animals, or poultry this household hav
Chickens
Goats
Sheep
Donkeys
Horses
Mules
Camels
Milk cows
Bulls
**Section 3: Awareness and access to health services**
q301hph. How many hours/minutes does it take to walk to thenerest health post?
q301hch. How many hours/minutes does it take to walk to the nearest health cente
q301. Have you visited the health post in the last 12 months?
q302. In general, how would you rate the service you received from the health post
q303. Did the HEW visit your household during the past 6 months to talk about you
q304. Have you heard of or do you know about health development army in your co
q305. Are you one of the Women Development Army (WDA) leaders?
q306. If you are one of the WDA leaders, are you a 1:5 or 1:30 leader?
q307. Do you have a map that displays the households under your catchment?
q308. Are there other WDAs in the neighborhood who have mapped the household
q309. Did any WDA visit you in the home to talk about your and/or your child healtl
q310. Did you meet any WDA somewhere outside your home to talk about your anc
q311. When your child is sick and want to get medical advice or treatment, is each c
Not knowing where to go
Not Getting permission to go
Not getting money needed for treatment
Not having a health facility nearby
Transportation problem
Not wanting to go alone
Concern that there may not be a female health provider

Concern that there may not be any provider
Due to household chores
COVID-19
Other
When your child is sick and want to get medical advice or treatment, how big, smal
Not knowing where to go
Not Getting permission to go
Not getting money needed for treatment
Not having a health facility nearby
Transportation problem
Not wanting to go alone
Concern that there may not be a female health provider
Concern that there may not be any provider
Due to household chores
Other
q313. Have you read the FHC or somebody and discussed on it?
q314. Do you/your household have a Family Health Card (FHC)?
q318. Is the family currently recognized as model family?
q324. Did anyone sleep under mosquito net(s) last night?
q325. If yes, who slept under mosquito net(s) last night? Probe: anyone else? Mark
Self
Newborn baby
Other children (under 5)
Spouse
Other
q326. Did ${q107} sleep under a bed net last night?
**Section 4: Maternal health**
q403. Did any community health workers visit you during your pregnancy of ${q107
q404. Who visited you during your pregnancy? ASK: Who else? Record all responses
q405. Did the WDA visit you at your home during your pregnancy to talk about heal
q406. While you were pregnant with ${q107} did any community health worker (WI
q407. How many times the discussion with you and with your family together occur
q410. During your last pregnancy have you attended a pregnant women's conferen
q411. If yes, how many times did you attend pregnant women's forum in your last p
q412. When you were pregnant with ${q107} did you go to a health facility for ante
q413. How many months pregnant were you when you first received antenatal care
q414. Where did you receive antenatal care for this pregnancy?
q416. How many times did you receive antenatal care in the health facility during th
q417. How many months pregnant were you when you received your last antenatal
q418. Did you receive pregnancy care from a health post?

q419. How many times did you attend the health post for pregnancy care in that pr
q420. How many months pregnant were you when you first received antenatal care
q421. The last time you visited the health post for pregnancy care, how did you trav
q422. Can you tell us the level of satisfaction or dissatisfaction for the pregnancy ca
q434 During this pregnancy were any of the following done at least once during you
Weight Taken
Blood pressure Measured
Urine Sample Given
Blood Sample Given
Received Breastfeeding Information
Received post partum Family Planning Information
Received Information about HIV/AIDS
Tested for HIV/ STI
Maternal Nutrition Information
Given Iron/Folate Supplementation
told about danger signs during pregnancy
counseled on birth preparedness and complication readiness
counseled on neonatal care
told about neonatal complications
q435. During this pregnancy were any of the following done at least once during yo
Weight Taken
Blood pressure Measured
Urine Sample Given
Blood Sample Given
Given drugs for Malaria
Received Breastfeeding Information
Received Family Planning Information
Received Information about HIV/AIDS
Tested for HIV/ STI
Maternal Nutrition Information
Given Iron/Folate Supplementation
told about danger signs during pregnancy
counseled on birth preparedness and complication readiness
counseled on neonatal care
told about neonatal complications
q436. During this pregnancy, were you given an injection in the arm to prevent the
q441. When you were pregnant with ${q107} did you eat, less than usual, the same
q442. When you were pregnant with ${q107} did you regularly take iron tablets?
q443. For how many months did you take iron tablets regularly when you were preg
q444. When you were pregnant with ${q107} did you take any drugs for intestinal p
Birth Preparedness

q445. During pregnancy of ${q107} did you make any preparations for your delivery
q446. What preparations did you make for the delivery of ${q107}?
q447. Sometimes mothers during pregnacy have severe illnesses and should be take
q448. Did you have any of the symptoms of the danger signs of pregnancy when pre
q449. Which of the symptoms of the danger signs of pregnancy did you have when
**Delivery and newborn care**
q500. Where did you give birth to ${q107}?
If delivered at home, why?
If delivered in health facility why?
q501. Did you or anybody in your family or neighborhood notify the health worker/
q502. What type of transportation means you used to reach to the place you delive
q503. What type of transportation means you used to go back to home?
q504. Who was the primary person that assisted you with the delivery of [q107]?
q505. Was ${q107} delivered by caesarean, that is, did they cut your belly open to t
q506. Did you go to the Health center or Hospital before having labor/delivery pain
q507. For how many days you stayed there before your delivery?
q507a. Did you stay at the MWH after delivery?
q508. How long after delivery you stayed there? (Hours)
q509. Immediate breast feeding
q509imm. Did you initiate breastfeeding immediately after birth?
q510. What did you do with the first milk?
q511. Was ${q107} weighed at birth?
q512. How much did ${q107} weigh?
q514. When ${q107} was born, was he/she very large, larger than average, average
q514. When ${q107} was born, was he/she at term, early, or very early?
q515. When ${q107} was delivered, what instrument was used to cut the cord?
q516. When ${q107} was delivered, what was used to tie the cord?
q517. When ${q107} was delivered, did anybody apply anything on the stump after
q518. Was chlorhexidine applied to the cord?
q519. If yes, for how many days was chlorhexidine applied?
q520. Did ${q107} receive TTC eye ointment soon after delivery?
q521. Was your baby ${q107} dried/wiped before the placenta was delivered or imr
q522. How long after ${q107} was born was s/he dried/wiped?
q523. Was your baby ${q107} wrapped in cloth before the placenta was delivered o
q524. How long after ${q107} was born was s/he wrapped in a cloth?
q525. Was your baby ${q107} put on the breast before the placenta was delivered c
q526. Where was the baby ${q107} placed before the placenta was delivered or imr
q527. Did the baby ${q107} cry or breath easily immediately after birth?
q528. What was done to help the baby ${q107} cry or breath easily immediately aft
q529. Who took these measures to help the baby ${q107} cry or breathed?
q530. How long after birth was your baby ${q107} bathed for the first time?
q531. In the first three days after delivery, was the baby ${q107} given anything to c

q532. What was the baby ${q107} given to drink?
q533. Did you do something to keep ${q107} warm following delivery?
q534. What did you do to keep the baby ${q107} warm following delivery?
q535. In the first fifteen days of life how frequently per day did you hold ${q107} ski
q536. In the first fifteen days did you sleep with ${q107} against you at night, or did
q537. What do you think are the complications in a woman during childbirth needir
q538. Do you know where to go if you experienced delivery complication, e.g. sever
q539. Where can you go if you experienced delivery complication, e.g. sever head a
q540. Did you have any of the symptoms of the danger signs during the birth of ${q
q541. When you had the symptoms, did you go some where to seek care?
q542. Where did you go when you had any of the symptoms?
q543. Can you tell us whether or not you were satisfied with the delivery care?
**Postnatal care for mother**
q600. Did you or anyone from your family or neighborhood inform the HEW about y
q601. Did you or anyone from your family or neighborhood inform the HEW about y
q602. For how long did you stay in the facility after the delivery?
q603. Did anyone check the baby's health while you were still in the facility
PNC for the mother
q604. Did any health care provider check on your own health within 6 weeks of givi
q605. How many days after giving birth did you have your first health check?
q606. Where did the first check take place?
q607. By whom?
q607x. How many times did you receive postntal care?
q608. Was a second visit conducted?
q609. How many days after giving birth did you have your second health check?
q610. Where did the checking take place?
q611. By whom?
q612. Was a third visit conducted?
q613. How many days after giving birth did you have your third health check?
q614. Where did the checking take place?
q615. By whom?
q616. During visits after giving birth, what was done to check your health?
q617. Can you tell us your satisfaction or dissatisfaction for the postnatal care you r
PNC for the baby
q618. In the first 6 weeks after ${q107} was born, did any health care provider excl
q619. If yes, how many days after birth was ${q107} 's first health check?
q620. If yes, where did the health checks on ${q107} take place?
q621. If yes, who checked on the health of ${q107} ?
q621x. How many times did your baby receive postntal care?
q622. Was a second check conducted?
q623. If yes, how many days after birth was ${q107} 's second health check?
q624. If yes, where did the health checks on ${q107} take place?

q625. If yes, who checked on the health of ${q107}?
q626. Was a third check conducted?
q627. If yes, how many days after birth was ${q107} 's third health check?
q628. If yes, where did the health checks on ${q107} take place?
q629. If yes, who checked on the health of ${q107}?
q630. At any of the health checks, what was done to check the health of ${q107}?
q631. Can you tell us your satisfaction or dissatisfaction for the postnatal care given
q632. The last time you gave birth, did you keep your newborn at home for several
q633. What is the number of days that you kept your newborn in the house?
q634. The last time you gave birth, were there visitors come to your house to see th
q635. The last time you gave birth, how many days passed before you had visitors c
q636. The last time you gave birth, someone other than you had physical contact w
q637. The last time you gave birth, how many days passed before someone other th
Can you tell us whether or not you were satisfied with the care for the your and you
**Now I am going to ask you on the newborn health care seeking issues**
q639. If a newborn infant is healthy, there is no need to take the newborn to the he
q640. A newborn infant should not be taken to the health center or health post for
q641. Sometimes mothers after delivery have severe illnesses and should be taken
q642. Did you face illness after delivery that needs immediate medical attention?
q643. Which of the folowing symptoms did you face?
**Infant feeding & food preparation practices**
q705. Since this time yesterday, did he/she receive any thing other than breast milk
q706. Since this time yesterday, did he/she receive any of the following?
q707. Why did you provide other drinks besides your breast milk?
q708. Are you currently breastfeeding ${q107}?
q709. Why did you stop breastfeeding ${q107}?
q710. Up to what age do you intend to breastfeed ${q107}?
q711. How many times did you breastfeed ${q107} yesterday, between sunrise yest
q712. Did ${q107} drink anything from a bottle with a nipple yesterday or last night
q713. At what age did you first introduce liquids or feeds other than breast milk to t
q714. How many times did you feed ${q107} solid and/or semi-solid food between s
q715. I would like to ask you about the types of foods ${q107} has been fed over the
q716. Have you ever taken ${q107} for a vaccination?
q717. Do you have any record/card where ${q107} vaccinations are written down?
q718. Does the infant ${q107} took appropriate vaccinations for his age?
q719. In the first two months after delivery, did you receive a dose of vitamin A?
q720. Did ${q107} receive a dose of vitamin A in the last 6 months?
**Section 6: Care seeking for infant and children**
**Now I want to talk to you about any sickness your child experienced in the first 2 [illegible]**
q800. Has ${q107} ever been sick during the first 2 months of life?
q801. Has ${q107} ever had any of the following symptoms?
q802. If any sickness/symptom reported, how old was ${q107} when sick for the firs
q803. When ${q107} was sick that first time what was the problem?

q804. Did you seek care for ${q107} outside the home at that time?
q806. If yes, how many times did you seek care for that illness?
805. If no care sought at that time, why didn't you seek care for ${q107} outside the
q807. Where outside the home did you seek care from the first time?
q808. After how many days did you seek care the first time?
q809. If yes to any of the symptoms, at any time during the illness, did ${q107} take
q810. What drugs did ${q107} take?
q811. For how many days did ${q107} take the drugs
q812. Can you tell us whether or not you were satisfied with the care for the sick ne
**How do you rate your satisfaction during your last visit to the HP for SYI care?**
Counseling services received
Visiting time to receive CBNC service is appropriate/good
Consultation time about CBNC service is appropriate/good
CBNC service providers (HEWs) are competent
Received convenient appointment date/time
Working hour of health post are convenient
Available drugs are enough at the health post
HEWs' approach is good
The waiting area of health post are clean
The waiting area of health post are appropriate
**Children no longer alive (and died before reaching 28 days)**
q813. Which symptoms did you observe in ${q107}'s immediately before death
q814. If ${q107} died due to an illness, was care sought for that illness?
q815. If yes, who provided that care?
q816. If care was sought before ${q107} died, what was the cause of death diagnos
**Knowledge and risk perception of COVID-19**
q900. Are you, or have you been, infected with the novel coronavirus?
q901. Do you know people in your immediate social environment who are or have b
q902. I am going to list some possible symptoms. These may or may not be actual sy
q903. I am going to list some possible transmission methods. These may or may not
q904. I am going to list some actions. Please tell me if you think this is a way to prev
q905. Since the beginning of the Coronavirus emergency, what, if anything, have yo
q906. Do you currently believe that there is a drug to treat coronavirus or vaccine?
q907. What is the maximum incubation period (i.e., the time from viral infection to
q908. Are you concerned about the spread of coronavirus?
q909. How do you perceive your level of risk for acquiring COVID-19? Would you sa
q910. Which answer is correct?
q911. What do you consider to be your own probability of getting infected with the
**Impact of COVID-19 on MNCH care practices**
q912. Fear of getting an infection during ANC visit

q913. Fear of fetus getting infected during ANC visit
q914. Spread of infection in a carrier state
q915. Fear of COVID-19 infection and delay in facility visits for ANC, SBA, and or PN[illegible]
q916. Afraid of catching infection from facility during delivery
q917. Fear of baby getting infection during delivery

For peer review only

		no-calendar		
		background		
Note to the InterviewerIf th				
Remember to answer in M(			. <15 and . >=2	
RECORD AGE IN COMPLETE				
Between 15 and 49 (years)			. < 50 and . > 14	
Including those that did no			.>0 and .<15	
			.<${q211} or .=${q211}	
Main construction material				
Main construction material				
Enter 0: if there is one roor			.<10	
If none; record “0”. If unkn				
Please scroll down the full l		field-list		

Please write in minutes.				
Please write in minutes.				
Explain what is an WDA				
Please observe the map.				

		field-list		
Ask this question if the WD				
enter '98' if don’t remembe			(.<6 and .>0) or .=98	
If greater than 9 times; rec			.<10 and .>0	
enter '98' if don’t remembe				
			(.<30 and .>0) or .=98	
Enter '98' if don't remembe			.=98 or (.<10 and .>0)	

READ OUT THE LIST				
enter '98' if don’t remembe			.<9 or .=98	

Only if necessary tell to the				
Do not read responses, ASK				
Do not read responses; ASK				
Select all that apply				
Select all that apply				
Do not read responses, ASK				
Do not read responses, ASK				
			.<60	
If stayed more than a week			.<169	
		field-list		
Initiated within one hour?				
from card; in Kg; between			(.<7 and .>0) or .=99.99	
			.<10 or .=99	
Record in hours; enter '0' if			.<73 or .=99	
Record in hours; enter '0' if			.<73 or .=99	
Do not read responses; ASK				
Record in hours; if more th			.<73 or .=98	

Do not read responses; ASK				
Do not read responses; ASK				
in-to-skin against breast during the daytime and nighttime?				
lay him/her alone on the bed, or elsewhere?				
Do not read responses; ASK				
Do not read responses; ASK				
If the delivery is home deliv				
For Facility delivery				
In Hours. Enter 72 if >72			.<73	
Probe for checks done at th				
Enter number of days , Ente			.<43 or .=99	
Enter number of days , Ente			.<43 or .=99 and .>${q605} (	
Enter number of days , Ente			.<43 or .=99 and .>${q609} (	
Read List out loud and Mar				
Probe for checks done at th				
Enter number of days and E			.<43 or .=99	
Enter number of days , Ente			.>${q619} or .=${q619} and	

Enter number of days , Ente			.>${q623} or .=${q623} and	
Read List out loud and Mar				
Ask all respondents			.<45	
Enter '0' if lessthan a day ar			.>=0 and .<=42 or .=99	
Enter 99 if do not know			.>=0 and .<=42 or .=99	
Mention example like waiti				
Do not read responses; ASK				
Read each item aloud and r				
(in months) if don't know e			.<60 or .=98	
If response is not numeric,			.<16 or .=98	
If response is not numeric,			.<16 or .=98	
Check the vaccination card, age of the infant, and also ask the mother regarding his vaccination s				
Record number of days of a				
Probe for all the following s				

Write number of times				
Write number of days from				
Select all that apply				
Write number of days				
Select all that apply				
Put 9 if not known				
peen infected with the nove				
List each measure for the p				

For peer review only

		yes		
		yes		
		yes		
${consent}=1				
${alive}=1		yes		
${alive}=1		yes		
${alive}=1		yes		
		yes		
${q112}!=1		yes		
		yes		
		yes		
${q201}=1		yes		
${q202}=1		yes		
		yes		
		yes		
${q205}=1		yes		
${q205}=1 and ${q206}=1		yes		
${q206}=1		yes		
		yes		
		yes		
		yes		
		yes		
${q214}=1		yes		
		yes		
		yes		
${q220}=1		yes		
		yes		
		yes		
		yes		
		yes		
		yes		
${q226}=1		yes		
		yes		
		yes		
		yes		
		yes		

		yes		
		yes		
		yes		
		yes		
		yes		
		yes		
		yes		
		yes		
		yes		
		yes		
		yes		
${q230m}=1				
		yes		
		yes		
		yes		
		yes		
		yes		
		yes		
		yes		
		yes		
		yes		
		yes		
		yes		
		yes		
${q301}=1		yes		
		yes		
		yes		
${q304}=1		yes		
${q305}=1		yes		
${q306}=0		yes		
${q306}=1 and ${q307}=0		yes		
${q304}=1 and ${q305}=0		yes		
${q304}=1 and ${q305}=0		yes		
		yes		
		yes		
		yes		
		yes		
		yes		
		yes		
		yes		

		yes		
		yes		
		yes		
		yes		
		yes		
		yes		
		yes		
		yes		
		yes		
		yes		
		yes		
		yes		
		yes		
		yes		
		yes		
		yes		
		yes		
${q324}=1				
		yes		
		yes		
		yes		
		yes		
		yes		
${q324}=1		yes		
		yes		
${q403}=1		yes		
${q403}=1 and ${q305}!=1		yes		
${q403}=1		yes		
${q406}=1		yes		
		yes		
${q410}=1		yes		
		yes		
${q412} =1		yes		
${q412} =1		yes		
${q412} =1		yes		
${q412} =1		yes		
		yes		

${q418} =1		yes		
${q418} =1		yes		
${q418} =1		yes		
${q418} =1		yes		
regex(${q414},'^.*a.*$') or rege				
		yes		
		yes		
		yes		
		yes		
		yes		
		yes		
		yes		
		yes		
		yes		
		yes		
		yes		
		yes		
		yes		
		yes		
${q418} =1				
		yes		
		yes		
		yes		
		yes		
		yes		
		yes		
		yes		
		yes		
		yes		
		yes		
		yes		
		yes		
		yes		
		yes		
		yes		
		yes		
		yes		
		yes		
${q442}=1		yes		
		yes		

		yes		
${q445}=1		yes		
		yes		
		yes		
${q448}=1		yes		
		yes		
${q500}=1 or ${q500}=2		yes		
${q500}=3 or ${q500}=4 or ${q5		yes		
		yes		
${q500}=3 or ${q500}=4 or ${q5		yes		
${q500}=3 or ${q500}=4 or ${q5		yes		
		yes		
${q500}=3 or ${q500}=4 or ${q5		yes		
${q500}=3 or ${q500}=4		yes		
${q506}=1		yes		
${q506}=1		yes		
${q507a}=1		yes		
		yes		
		yes		
		yes		
${q511}=1		yes		
		yes		
		yes		
${q500}=1 or ${q500}=2		yes		
${q500}=1 or ${q500}=2		yes		
		yes		
${q517}=1		yes		
${q517}=1 and ${q518}=1		yes		
		yes		
		yes		
${q521}=1		yes		
		yes		
${q523}=1		yes		
		yes		
		yes		
		yes		
${q527}=2		yes		
${q527}=2		yes		
		yes		
		yes		

${q531}=1		yes		
		yes		
${q533}=1		yes		
		yes		
		yes		
		yes		
		yes		
${q538}=1		yes		
		yes		
${q540}=1		yes		
${q540}=1 and ${q541}=1		yes		
		yes		
${q500}=1 or ${q500}=2		yes		
${q500}=3 or ${q500}=4 or ${q5		yes		
${q500}=3 or ${q500}=4 or ${q5		yes		
${q500}=3 or ${q500}=4 or ${q5		yes		
		yes		
${q604}=1		yes		
${q604}=1		yes		
${q604}=1		yes		
		yes		
${q604}=1		yes		
${q604}=1 and ${q608}=1		yes		
${q604}=1 and ${q608}=1		yes		
${q604}=1 and ${q608}=1		yes		
${q604}=1 and ${q608}=1		yes		
${q604}=1 and ${q608}=1 and $		yes		
${q604}=1 and ${q608}=1 and $		yes		
${q604}=1 and ${q608}=1 and $		yes		
${q604}=1		yes		
${q604}=1		yes		
${alive}=1				
		yes		
${q618}=1		yes		
${q618}=1		yes		
${q618}=1		yes		
		yes		
${q618}=1		yes		
${q618}=1 and ${q622}=1		yes		
${q618}=1 and ${q622}=1		yes		

${q618}=1 and ${q622}=1		yes		
${q618}=1 and ${q622}=1		yes		
${q618}=1 and ${q626}=1		yes		
${q618}=1 and ${q626}=1		yes		
${q618}=1 and ${q626}=1		yes		
${q618}=1		yes		
${q618}=1		yes		
		yes		
		yes		
		yes		
${q634}=1		yes		
		yes		
${q636}=1		yes		
		yes		
		yes		
		yes		
		yes		
		yes		
${q642}=1		yes		
${q109}<6		yes		
${q705}=1		yes		
${q705}=1		yes		
		yes		
${q708}=2		yes		
${q708}=1		yes		
${q708}=1		yes		
		yes		
		yes		
${q713}!=4		yes		
${q713}!=4		yes		
		yes		
${q716}=1		yes		
status and record (yes=if vaccinated for his		yes		
		yes		
		yes		
		yes		
${q800}=1		yes		
regex(${q801},'^.*a.*$') or rege		yes		
regex(${q801},'^.*a.*$') or rege		yes		

		yes		
${q804}=1		yes		
${q804}=1		yes		
${q804}=1		yes		
${q804}=1		yes		
${q804}=1		yes		
${q809}=1		yes		
${q810}=1		yes		
		yes		
		yes		
		yes		
		yes		
		yes		
		yes		
		yes		
		yes		
		yes		
		yes		
		yes		
${alive}=0				
		yes		
		yes		
		yes		
		yes		
		yes		
		yes		
		yes		
		yes		
		yes		
		yes		
		yes		
		yes		
		yes		
		yes		
		yes		
		yes		
		yes		

yes
yes
yes
yes
yes

For peer review only

For peer review only

For peer review only

የክልል ጤና ቢሮ እና JSI/L10K የቤት ለቤት	
መለያ ጥያቄዎች እና ስምምነት	
የተጠያቂዋ ቁጥር	
መረጃ የተሰበሰበበት ቀን	
መተዋወቅና ስምምነትጤና ጥልኝ .....ስሜ ${	ለመረጃ ሰብሳቢዎች፡- የተጠያቂዋ እድሜ ከ15 ዓ
በዚህ ጥናት ለመሳተፍ ተስማምተዋል?	
ባለፉት 3-15 ወራቶች ውስጥ የተወለደው ህፃ	
q107. የመጨረሻ ልጅዎ ስም ማን ይባላል?	
q105_w0_11. የ${q107} ጾታ	
q106_w0_11. የ${q107} ዕድሜ?	በ ወር ይገለጽ ::
በህይወት የተወለደ ሌላ ህጻን አለ ከዚህኛዉ (	
q112. ${q107}(የመጀመሪያ ፣ ሁለተኛ ፣ ወ	${q201} ስንተኛ ልጅዎ ነው (የመጀመሪያ፣ሁለተ
q114. የልጁ ሽማግሌ ዕድሜው ከ ${q107}	የ${q201} ታላቅ ዕድሜ ስንት ነው?
ክፍል 2፡ መሠረታዊ መረጃ እና የቤተሠብ ሁኔ	
q202. ዕድሜዎ ስንት ነው?	ከ15 እስከ 49 ዓመት
q204. ቀላል ዓረፍተ ነገሮችን ማንበብ ወይም	
q205. መደበኛ ትምህርት ተከታትለው ያውቃ	
q206. ያጠናቀቁት ከፍተኛው የትምህርት ደረ	
q207. ሃይማኖትዎ ምንድነው?	
q208. አሁን በጋብቻ ላይ ኖት ወይስ ከወንድ	
q204. ባለቤትዎ ቀላል ዓረፍተ ነገሮችን ማንበ	
q205h. ባለቤትዎ መደበኛ ትምህርት ተከታተ	
q206h.ባለቤትዎ ያጠናቀቁት ከፍተኛው የት	ካላወቁት 99 ይሙሉ
q211. እስከ አሁን ስንት ጊዜ ነፍሰጡር ሆነው	(በሕይወት ያልተወለዱትንም ይጨምራል) ከ14 (
q212. እስከዛሬ ስንት ጊዜ ልጅ ወልደዋል (Pa	
q213. እስከ ዛሬ ድረስ በጠቅላላ ስንት ልጆች	
q214. በሕይወት ልደት ያልጨረሰ እርግዝና (	
q215. በህይወት ልደት ያልጨረሰ ስንት እርግ	
q218. የቤተሰቡ አባላት በዋነኛነት ውሃ የሚያ	አንድ መልስ ይቀበሉ
q220. ለመጠጥ የሚያውሉትን ውሃ ንጽሕና (	
q221. ለመጠጥ እንዲሆን የተፈለገውን ውሃ	ዝርዝሩን አያንብቡ፡፡ አንድ መልስ ይቀበሉ፡፡
q222. አብዛኛው የቤተሰቡ አባላት ምን ዓይነ	አንድ መልስ ይቀበሉ፡፡ካለ መጸዳጃ ቤቱን ተመልከ
q225. የመኖሪያ ቤቱ ጣሪያ በአብዛኛው ከም	Main construction material of the roof
q226. የመኖሪያ ቤቱ ግድግዳ በዋነኛነት የተሠ	Main construction material of the wall
q227. በቤቱ ውስጥ ስንት ክፍሎችን ለመኝታ	አንድ ክፍል ለተለያየ አገልግሎት የሚጠቀሙ ከሆነ
q226. ማንኛውም የቤተሰብ አባል ማንኛውም	
q228. በአሁኑ ወቅት የቤተሰቡ አባላት የእርሻ	(መልሶቹን በሄክታር ይመዝግቡ) ምንም ከሌለ 0 ይ
q228a. በዋነኛነት ምግብ ይምታበስሉት በም	
asset. ቤተሰቡ ከሚከተሉት ውስጥ ምን ምን	ወደታች ወርደው ሙሉ ዝርዝሩን አዎ ወይም የለም
q229a. የኤሌክትሪክ መብራት አለው? (ከሃይ	
q229b. ሠዓት አለው?	
q229c. ሬዲዮ አለው?	

q229d. ቴሌቪዥን አለው?
q229e. ሞባይል ስልክ አለው?
q229f. የቤት ስልክ አለው?
q229g. ማቀዝቀዣ(ፍሪጅ) አለው?
q229h. ጠረጴዛ አለው?
q229i. ወንበር አለው?
q229j. አልጋ አለው?
q229k. የኤሌክትሪክ ምጣድ አለው?
q229l. ፋኖስ/ማሾ/ኩራዝ አለው?
q229m. ከብቶች/በግ ወይም ፍየል
q229n. ጌጦች(ወርቅ/ብር)

q232. ይህ የከብት ፣ የከብት እርባታ ፣ ሌሎች
ዶሮዎች
ፍየሎች
በጎች
አህዮች
ፈረሶች
በቅሎዎች
ግመሎች
ወተት ላሞች
ኮርማዎች/በሬ/ወይፈን

ክፍል 3፡ የጤና አገልግሎት ግንዛቤና አቅርቦ
q301hph.ከቤትዎ ወደሚቀርብዎ ጤና ኬላ Please write in minutes.
q301hph.ከቤትዎ ወደሚቀርብዎ ጤና ጣበ Please write in minutes.
q302. ባለፉት 12 ወራት ወደ ጤና ኬላ ሄደ
q303b. እንዳጠቃላይ ለመጨረሻ ጊዜ በጤና
q306. ባለፈው 6 ወር የጤና ኤክስቴንሽን ሠ
q308. ስለ ጤና ልማት ቡድን (የ1 ለ5 ወይምየጤና ልማት ቡድን ማለት ምን እንደሆነ ያብራሩ፡
q308a. የጤና ልማት ቡድን መሪ ኖት (የ1 ለየጤና ልማት ቡድን ማለት ምን እንደሆነ ያብራሩ፡
q308a1. ከልማት ቡድን መሪዎች ውስጥ አን
q308a2. በእርሶ ክትትል ስር ያሉትን ቤቶች/ ካርታውን ይመልከቱ
q308b. እርስዎ ወይም ሌሎች የጤና ልማት
q310. ባለፉት 6 ወራት ውስጥ የጤና ልማት የጤና ልማት ቡድን ማለት ምን እንደሆነ ያብራሩ፡
q310anyencounter. ባለፉት 6 ወራት ወየጤና ልማት ቡድን ማለት ምን እንደሆነ ያብራሩ፡
q313. ልጅዎ ቢታመምና ምክር ወይም ህክምና ምርጫዎችን ያንብቡላቸው
q313a. ወዴት እንደሚሄዱ አለማወቅ
q313b. ለመሄድ ፈቃድ አለማግኘት
q313c. ለሕክምና የሚሆን ገንዘብ አለማግኘ
q313d. በአቅራቢያዎ የሚሄዱበት ጤና ድር
q313e. የመጓጓዣ ችግር
q313f. ብቻዎትን ለመሄድ አለመፈለግ
q313g. ሴት የጤና ባለሙያ አትኖርም ብሎ

q313h. የጤና አገልግሎት የሚሰጥ ባለሙያ ሰ
q313i. ሌላ የቤት ውስጥ ሥራ መብዛት
ኮቪድ -19
q313j. ሌላ

When your child is sick and want tc
q313a. ወዴት እንደሚሄዱ አለማወቅ
q313b. ለመሄድ ፈቃድ አለማግኘት
q313c. ለሕክምና የሚሆን ገንዘብ አለማግኘ
q313d. በአቅራቢያዎ የሚሄዱበት ጤና ድር
q313e. የመጓጓዣ ችግር
q313f. ብቻዎትን ለመሄድ አለመፈለግ
q313g. ሴት የጤና ባለሙያ አትኖርም ብሎ
q313h. የጤና አገልግሎት የሚሰጥ ባለሙያ ሰ
q313i. ሌላ የቤት ውስጥ ሥራ መብዛት
q313j. ሌላ

q313. FHC የቤተሰብ ጤና መመሪያን አንብ የምሥክር ወረቀቱን ያሳዩዋቸው
q314. እርስዎ / ቤተሰቦችዎ የቤተሰብ ጤና ነ መፀሐፉን እንዲያሳዩ ጠይቋቸው
q318. Is the family currently recogn
q505. ትናንት ማታ በአጎበር ከለላ ውስጥ የተ
q506. አዎን ካሉ በአጎበሩ ከለላ ውስጥ የተኙ ዝርዝሩን አያንብቡ፣ለተዘለዘሩት ሰዎች በሙሉ አያ
q506a. ተጠያቂው
q506b. አራስ ልጅ
q506c. ሌሎች ልጆች (< 5)
q506d. ባለቤትዎ
q506e. ሌላ

wq507. ${q107} ትናንት ማታ በአጎበር ስ
የእናቶች ጤና
wq601. ${q107} ን ነፍሰጡር በነበሩ ጊዜ
wq602. በእርግዝናዎ ወቅት ማን ነበር የጎበኛሌላስ ብለዉ ይጠይቁ/ ዝርዝሩን አያንብቡ/ ሁሉን
wq602a. በእርግዝናዎ ወቅት የጤና ልማት (ይሄ ጥያቄ የሚጠየቀው፣ ልማት ቡድን መሪ ምርጫ
wq603a. ${q107}ን እርጉዝ በነበሩበት ወ
wq603e. በቤትዎ ውስጥ በጋራ ከእርስዎ እናየማያስታውሱት ከሆነ 98 ይሙሉ እና h 5 ከበለ
wq603h. ${q107} ነፍሰ ጡር በነበርሽ ጊ
wq603i. ለጥያቄ 603 h መልሱ አዎ ከሆነ፤ If greater than 9 times; record as9
wq604.${q107}ን እርጉዝ በነበሩ ጊዜ ለቅ
wq605.${q107}ን እርጉዝ በነበሩ ጊዜ ለመ የማያስታውሱት ከሆነ 98 ይሙሉ
wq606${q107}ን እርጉዝ በነበሩ ጊዜ ምርመ
wq608. ${q107}ን ባረገዙበት ወቅት ለስን የማያስታውሱት ከሆነ 98 ይሙሉ፤h 9 በላይ ጉብ
wq608a1.${q107}ን እርጉዝ በነበሩ ጊዜ ለአማያስተውሱት ከሆነ 98 ብላቸሁ ጻፍ፤ ወሩን ጻፍ
q418 እ.ኤ.አ. ከጤና ጣቢያ የእርግዝና እንክ

q419. በዚያ እርግዝና ውስጥ ለእርግዝና እን

q420. ለዚህ እርግዝና የመጀመሪያ የወሊድ እ

q421. ለመጨረሻ ጊዜ ለእርግዝና እንክብካቤ

በጤና ኬላ ስላገኙት የእርግዝና ክትትል መርካ

q434. ${q107}ን ባረገዙበት ወቅት በጤና ‹ ANC Components at health center or h

wq609a. ክብደት ተለክተዋል

wq609c. የደም ግፊት ተለክተዋል

wq609d. የሽንት ምርመራ

wq609e. የደም ምርመራ

wq609g. ስለ ጡት ማጥባት ምክር

wq609h. ስለ ድህረ ወሊድ ጊዜ የቤተሰብ እ

wq609i. ስለ ኤች.አይ.ቪ/አባላዘር በሽታ ም

wq609j. ስለ ኤች.አይ.ቪ/አባላዘር በሽታ ም

wq609k. ስለ እናቶች አመጋገብ ምክር

wq609l. የደም ማነስ እንክብል/ኪኒን ተሰጥ

wq609m. ስለ እርግዝና አደገኛ ምልክቶች ም

wq609n. ለወሊድ ስለሚደረግ ዝግጅት እና

wq609o. ስለአዲስ የተወለደ ሕፃን እንክብካ

wq609p. አዲስ ስለተወለደ ሕፃን በሽታዎች/

wq609hp. ${q107}ን ባረገዙበት ወቅት ከ ANC Components at the Health Post

ክብደት ተለክተዋል

የደም ግፊት ተለክተዋል

የሽንት ምርመራ

የደም ምርመራ

የወባ መድሀኒት ወስደዋል

ስለ ጡት ማጥባት ምክር

ስለ ቤተሰብ እቅድ ምክር

ስለ ኤች.አይ.ቪ. ምክር

ስለ አባላዘር በሽታ ምርመራ

ስለ እናቶች አመጋገብ ምክር

የደም ማነስ እንክብል ተሰጥቷቸዋል

ስለ እርግዝና አደገኛ ምልክቶች ምክር

ለወሊድ ስለሚደረግ ዝግጅት ምክር and cor

ስለአዲስ የተወለደ ሕፃን እንክብካቤ ምክር

አዲስ ስለተወለደ ሕፃን በሽታዎች/ችግሮች

wq610. ${q107} አርግዘው በነበረ ጊዜ በክይህ ክትባት ህጻናት ከተወለዱ በኋላ መንጋጋ ቆልፍ

wq615. ${q107}ን አርግዘው በነበረ ጊዜ አ ምርጫውን ያንብቡ

wq616. ${q107}ን አርግዘው በነበር ጊዜ የ.

wq617. ${q107}ን አርግዘው በነበረ ጊዜ የ.በእርግዝናዋ ጊዜ ስለወሰደችዉ ብቻ ነዉ:: የማያስ

wq619. ${q107}ን አርግዘው በነበረ ጊዜ የ

የወሊድ ቅድመ ዝግጅት

wq701. ${q107} ን ነፍሰጡር በነበሩበት ወ	አስፈላጊ ሆኖ ከተገኘ ብቻ የወሊድ ዝግጅት ምን
wq702. ${q107} ን ነፍሰጡር በነበሩበት ወ	ሌላስ ብለዉ ይጠይቁ/ ዝርዝሩን አያንብቡ/ ሁሉን
አንዳንድ ጊዜ እናቶች በእርግዝናቸዉ ወቅት ባ	ሌላስ ብለዉ ይጠይቁ/ ዝርዝሩን አያንብቡ/ ሁሉን
wq941. ${q107}ን እርጉዝ በነበሩበት ወቅ	
wq942. የትኞቹ አደገኛ ምልክቶች ነበሩ ያጋ	ከነበሩ ቢጠቅሱዋቸውዝርዝሩን አያንብቡሌላስ በ
ወሊድ እና አዲስ ለተወለደ ሕጻን የሚደረግ አ	
wq801. ${q107}ን የተገላገሉት (የወለዱት)	q500. ${q107}የት ነው የወለዱት?
wq801a1. እቤት ከተገላገሉ(ከወለዱ) ለምን?	**የተጠቀሱትን መልሶች ሁሉ ይቀበሉ**
wq801a2. ጤና ተቋም ከተገላገሉ(ከወለዱ) ለም	**የተጠቀሱትን መልሶች ሁሉ ይቀበሉ**
q800a. እርስዎ፣ ከቤተሰብዎ መካከል ወይ	q800a. እርስዎ፣ ከቤተሰብዎ መካከል ወይም ከ
wq801d. የወለዱበት ቦታ ለመድረስ ምን ዓ	ዝርዝሩን አያንብቡ/ ሌላስ በማለት ይጠይቋቸው/
wq801d1. ከወለዱ በኃላ ወደ ቤት ለመመለ	ዝርዝሩን አያንብቡ/ ሌላስ በማለት ይጠይቋቸው/
q802. ${q107} ን ማን አዋለዶት?	በዋናነት ያዋለዳቸውን ሰው ይጠይቁ
wq802cs. ${q107} የተገላገሉት በቀዶ ጥ	q505. ${q107}የቀረበው ቄሳር ነበር ፣ ማለትም
wq801mwh1. ምጥሽ ከመምጣቱ/ ህመም	q506. የጉልበት / የወሊድ ህመም ከመያዝዎ በ
wq801mwh8. ከመውለድዎ በፊት ለምን	
wq801mwh9a. ከወለዱ በኃላ የእናቶች ማ	
wq801mwh10h. ከወለዱ በኁዋላ ለምን	በሰዓት ይሞላ:: ከ 1 ሳምንት በላይ ከቆዩ 168 ሰ
ጡት ማጥባት	ከአንድ ሠዓት ያነሰ ከሆነ 0 ብለው ይመዝግቡ:: ከ
wq804imm. ወዲያውኑ እንደወለዱ ጡት አጥብተዋል? በአንድ ሠዓት ውስጥ	
wq805. ${q107}ን እንደተወለደ ጡት ማ	
wq806. ${q107} ሲወለድ ክብደቱ ተመዝ	
wq807. የ${q107} ክብደት ምን ያህል ነበ	ሕፃኑ ካርድ ካለው ክብደቱን አይተው ይመዝግቡ
wq808. ${q107} ሲወለድ/ስትወለድ ክብ	
q514. ${q107} ሲወለድ በእድሜው ነው	
wq809. ${q107} ሲወለድ እትብቱን ለመ	
wq810. ${q107} ሲወለድ እትብቱን ለማሰ	
wq811. ${q107} እትብቱ ከተቆረጠ በኋላ	
wq812a. የምስራች (chlorhexidine) የአ	
wq812b. ለምን ያህል ቀን ነበር የምስራች የ፤የቀኑን ብዛት ጻፍ፤ እማያውቁት ከሆነ 99 ብላችሁ	
wq812c. ${q107} ወዲያው እንደተወለደ	
wq813. የእንግዴ ልጁ ከመውጣቱ በፊት ${	
wq813a. ${q107}ከተወለደ/ች ከምን ያህ	በሰዓት ይሞላ:: ወዲያዉ ከሆነ '0' ይሞላ ፣ ከ 3
wq814. የእንግዴ ልጁ ከመውጣቱ በፊት ${	
wq814a.${q107}ከተወለደ/ች ከምን ያህል	በሰዓት ይሞላ:: ወዲያዉ ከሆነ '0' ይሞላ ፣ ከ 3
wq815. የእንግዴ ልጁ ከመውጣቱ በፊት ለ	
wq816. የእንግዴው ልጁ ከመውጣቱ በፊት	
wq817. ${q107} እንደተወለደ ወዲያውኑ	
wq818. ${q107} እንዲያለቅስ ወይም በቀ	ሌላስ ብለዉ ይጠይቁ/ ዝርዝሩን አያንብቡ/ ሁሉን
wq819. ${q107} እንደተወለደ አንዲያለቅ	
wq820. ${q107} ለመጀመሪያ ጊዜ ገላውን (በሰአት) ከ 3 ቀን በላይ ከሆነ 72 ያስገቡ፣ እማያ	
wq821. ${q107} ከተወለደ በኋላ ባሉት የ	

wq822. ${q107} ከተወለደ በኋላ ባሉት የ ሌላስ ብለዉ ይጠይቁ/ ዝርዝሩን አያንብቡ/ ሁሉን

wq823a. ${q107}ን ከወለዱ በኋላ ሙቀት

wq823. ${q107}ን ከወለዱ በኋላ ሙቀት ሌላስ ብለዉ ይጠይቁ/ ዝርዝሩን አያንብቡ/ ሁሉን

q535. በህይወትዎ የመጀመሪያዎቹ አስራ አምስት ቀናት ውስጥ በቀን እና በማታ ጊዜ በቀን ${q

q536. በመጀመሪያዎቹ አስራ አምስት ቀናት ውስጥ ${q107} ጋር ተኝተህ ነበር ወይንስ ብቻው

wq826. አንድ እናት በወሊድ ጊዜ ምን ዓይነ ሌላስ ብለዉ ይጠይቁ/ ዝርዝሩን አያንብቡ/ ሁሉን

wq827a. በወሊድ ጊዜ ወሊዱ አስቸጋሪ ቢሀ

wq827. በወሊድ ጊዜ ወሊዱ አስቸጋሪ ቢሆ ሌላስ ብለዉ ይጠይቁ/ ዝርዝሩን አያንብቡ/ ሁሉን

wq828cbexp. ${q107}ን በሚወልዱ ወ

wq829a. ${q107}ን በሚወልዱ ወቅት ከ

wq829. ${q107}ን በአረገዙበት ወቅት ከ

q543. በአቅርቦቱ እንክብካቤ እንደረኩ ወይ

የድህረ ወሊድ እንክብካቤ ለእናት

wq900. ${q107}ን እንደወለዱ ወዳያውኑ ይሄ ጥያቄ ቤት ለወለዱ ነው፡፡

wq901. እርስዎ ወይም ቤተሰብዎ ወይም ጎረ ይሄ ጥያቄ ጤና ድርጅት ለወለዱ ነው፡፡

wq902. ${q107} ጤና ድርጅት ከተገላገሉ በሰአት፡፡ ከ 72 ከበለጠ 72 ተብሎ ይሞላ

wq903. እዚያው ጤና ድርጅት ውስጥ እያላ

የድህረ-ወሊድ ክብካቤ ለእናት በወሊድ ቦታም ይሁን ከዛም በሁዋላ ስለሚደረጉ

wq904. ${q107} ከተገላገሉ በኃላ በ6 ሳም መልሱ የለም ከሆነ፤ ድህረ-ወሊድ እንክብካቤ ለ

q605. ${q107}ን ከተገላገሉ በኃላ ፤ከምን ያየቀኑን ብዛት ይጻፉ፤ እማያውቁት ከሆነ 99 ብለው

wq906. የእርስዎን ጤንነት ለማረጋገጥ የመጀ

wq907. የእርስዎን ጤንነት ለማረጋገጥ የመጀ

q607x. የድህረ ወሊድ እንክብካቤን ስንት ጊ

wq908. የእርስዎን ጤንነት ለማረጋገጥ ሁለተ መልሱ የለም ከሆነ፤ ድህረ-ወሊድ እንክብካቤ ለ

wq909. ${q107} ከተገላገሉ በኃላ ከምን ያ የቀኑን ብዛት ይጻፉ፤ እማያውቁት ከሆነ 99 ብለው

wq910. የእርስዎን ጤንነት ለማረጋገጥ ሁለተ

wq911. የእርስዎን ጤንነት ለማረጋገጥ ሁለተ

wq912. የእርስዎን ጤንነት ለማረጋገጥ ሦስተ መልሱ የለም ከሆነ፤ ድህረ-ወሊድ እንክብካቤ ለ

wq913. ${q107} ከተገላገሉ በኃላ ከምን ያ የቀኑን ብዛት ይጻፉ፤ እማያውቁት ከሆነ 99 ብለው

wq914. የእርስዎን ጤንነት ለማረጋገጥ ሦስተ

wq915. የእርስዎን ጤንነት ለማረጋገጥ ሦስተ

wq916. ${q107} ከተገላገሉ በኃላ በተደረገ ዝርዝሩን አንብቡላቸው፡፡ መልሶችን ይከበቧቸው

wq917. በአገኙት የድህረ-ወሊድ አገልግሎት

የድህረ-ወሊድ ክብካቤ ለጨቅላ ህጻን በወሊድ ቦታም ይሁን ከዛም በሁዋላ ስለሚደረጉ

wq918. ${q107}ን ከተገላገሉ በኃላ በመጀ (ስም) በተወለደበት/ችበት ቦታ እና በተወለደበት/

wq919. ለጥያቄ 918 መልሱ አዎ ከሆነ፤ ${ የቀኑን ብዛት ይጻፉ፤ እማያውቁት ከሆነ 99 ብለው

wq920. ለጥያቄ 918 መልሱ አዎ ከሆነ፤${q

wq921. ለጥያቄ 918 መልሱ አዎ ከሆነ፤ በ ከተሻለ ጤና ባለሙያ አገልግሎት ማግኘቱን/ቷን

q621x. ልጅዎ የድህረ ወሊድ እንክብካቤን ስ

wq922. ${q107}ን ጤንነት ለማረጋገጥ ሁ

wq923. ለጥያቄ 922 መልሱ አዎ ከሆነ፤ ${ የቀኑን ብዛት ይጻፉ፤ እማያውቁት ከሆነ 99 ብለው

wq924. ለጥያቄ 922 መልሱ አዎ ከሆነ፤${q

wq925. ለጥያቄ 922 መልሱ አዎ ከሆነ፤ በ	
wq926. ${q107}ን ጤንነት ለማረጋገጥ ሶስ	
wq927. ለጥያቄ 926 መልሱ አዎ ከሆነ፤ ${	የቀኑን ብዛት ይጻፉ፤ እማያውቁት ከሆነ 99 ብለው
wq928. ለጥያቄ 926 መልሱ አዎ ከሆነ፤የ${q1	
wq929. ለጥያቄ 926 መልሱ አዎ ከሆነ፤ በማን	**ከተሻለ ጤና ባለሙያ አገልግሎት ማግኘቱን/ቷን አጥ**
wq930. በሁሉም የጉብኝት ወቅቶች የ ${q107}	**ዝርዝሩን አንብቡላቸው፡፡ መልሶችን ይከበቧቸው፡፡**
wq931. ለ ${q107} በተሰጠው የድህረ-ወ	
wq932. ${q107}ን በወለዱ ጊዜ፤ ${q107	
wq933. ልጅዎን ከቤት ሳያወጡት/ቧት ለስ	Ask all respondents
wq934. ${q107}ን በወለዱ ጊዜ፤ ${q10	በማንኛውም ጉዳይ የመጡ ጠያቂዎችን ያጠቃልላ
wq934. ${q107}ን በወለዱ ጊዜ፤ ${q10	ከ1 ቀን በታች ከሆነ 0 ያስገቡ ካላወቁ 99 ያስገቡ፡
wq935tou. ${q107}ን በወለዱ ጊዜ፤ ከእር	ሰውነት መንካት ስንል የህጻኑን ስውነት በማንኛው
wq935. ${q107}ን በወለዱ ጊዜ፤ ከእርሰዎ	ሰውነት መንካት ስንል የህጻኑን ስውነት በማንኛው
የእርስዎ እና የሕፃናትዎ PNC እንክብካቤ እን	
አሁን ለጨቅላ ህጻናት ስለሚሰጥ የህክምና እ	
wq937 አዲስ የተወለደው ጨቅላ ህጻን ጤን	
wq938. አዲስ የተወለደው ጨቅላ ህጻን በአ	ለምሳሌ ሣይጠመቅ ከቤት አለማዉጣት
wq940. ከወሊድ በኋላ አንዳንድ ጊዜ እናቶች	ሌላስ ብለዉ ይጠይቁ/ ዝርዝሩን አያንብቡ/ ሁሉን
wq940a. ከወሊድ በኋላ በአስቸኳይ ወደ ጠ	
wq940b. የትኞቹ ከወሊድ በኋላ በአስቸኳይ	
የሕፃናት አመጋገብና የምግብ አዘገጃጀት ልምዶ	
wq1107liq. ከትናንት ጀምሮ ህፃኑ/ኗ ከጡት	
wq1107. ከትናንት ጀምሮ ህፃኑ/ኗ ከሚከተ	እያንዳንዳቸውን መልሶች ያንብቡላቸው/ከአንድ በ
wq1107a. ከጡት ወተት በተጨማሪ ሌላ የ	
wq1108. አሁን ${q107}ን እያጠቡ ነው?	
wq1109. ${q107}ን ጡት ማጥባት ለምን	ዝርዝሩን አያንብቡ
wq1109a. ${q107}ን ስንት ዓመት እስከ	(በወራት) ካላወቁት '98' ያስገቡ፣ ከ 5 አመት በላ
wq1109b. ከትላንትና ጠዋት እስከዛሬ ጠዋ	መልሱን በቁጥር እንዲገልጹ ያበረታቱ:: ካላወቁት
wq1109c. ከትላንትና ጠዋት እስከዛሬ ጠዋ	
wq1110. ለ${q107} ተጨማሪ ምግብ ወይ	
wq1110a. ከትላንትና ጠዋት እስከዛሬ ጠዋ	If response is not numeric, probe for a
wq1110b. ከትላንት ንጋት እስከዛሬ ንጋት ደ	ሌላስ ብለዉ ይጠይቁ/ ዝርዝሩን አያንብቡ/ ሁሉን
q716. $ {q107}ክትባት ወስዶ ያውቃል?	
q717. የ [$ {q107}] ክትባቶች የተፃፉበት	
q718 እ.ኤ.አ. ህፃኑ [$ {q107}] ለዕድሜው ተገቢ	ክትባቶችን ይወስዳል?
wq939. ${q107}ን በወለዱ በመጀመሪያዎ	ብልቃጡን/ እንክብሉን ያሳዩ
wq1001. ${q107} ባለፉት 6 ወራት ቫይታ	የቫይታሚን ኤ ካፕሱል ያሳዩ
የሕጻናት የሕክምና አሰጣጥ ሁኔታ	
አሁን በመጀመሪያዎቹ 2 ወራቶች ልጅዎ ስላጋ	ጠመው ማንኛውም ህመም ከእርስዎ ጋር መነጋገር
q800. በህይወት የመጀመሪያዎቹ 2 ወሮች ወ	
q801. ${q107}ከሚከተሉት ምልክቶች ው	
ቁ 802. ማንኛውም በሽታ / ምልክት ከተዘገ	
q803. ${q107} ለመጀመሪያ ጊዜ ሲታመም	

q804. በዚያን ጊዜ ከቤት ውጭ ለ ${q107}

q806. አዎ ከሆነ ለዚያ ህመም ምን ያህል ጊዜ

805. በዚያን ጊዜ ምንም እንክብካቤ ካልተደረ

q807. ከመጀመሪያው ጊዜ ጀምሮ ከቤት ው

ቁ 808. ለመጀመሪያ ጊዜ ከስንት ቀናት በኋላ

q809. ለማንኛውም ምልክቶች አዎ ከሆነ ፣ በ

q810. ${q107}ምን ዓይነት መድኃኒቶች ወ

q811. ${q107}$ ስንት ቀናት መድኃኒቶቹን

q812. ለታመመው አዲስ ለተወለደው ህፃን እ

ወደ ኤች.አይ.ፒ. (HPI) እንክብካቤ ለመጨረሻ ጊዜ ወደ HP ሲጎበኙ እርካታዎን እንዴት ይሰጡ

የምክር አገልግሎት ተቀብሏል

የ CBNC አገልግሎትን ለመቀበል የመጎብኘት

ስለ CBNC አገልግሎት የምክር ጊዜ ተገቢ / ጥ

የ CBNC አገልግሎት ሰጭዎች (HEWs) ብቃ

ምቹ የቀጠሮ ቀን / ሰዓት ተቀብሏል

የጤና ልጥፍ የሥራ ሰዓት ምቹ ናቸው

በጤና ጣቢያው የሚገኙ መድኃኒቶች በቂ ናቸ

የ "HEWs" አቀራረብ ጥሩ ነው

የጤና ኬላ የሚጠበቅበት ቦታ ንፁህ ነው

የጤና ኬላ ማቆያ ስፍራው ተገቢ ነው

ልጆች ከእንግዲህ በሕይወት አይኖሩም (እና 28 ቀናት ከመድረሳቸው በፊት ሞተዋል)

q813. ከመሞቱ በፊት ወዲያውኑ በ ${q107

q814. ${q107}በህመም ምክንያት ከሞተ ለ

q815. አዎ ከሆነ ያንን እንክብካቤ የሰጠው

q816. ${q107}ከመሞቱ በፊት እንክብካቤ

ስለ COVID-19 ዕውቀት እና አደጋ ግንዛቤ

q900. እርስዎ ነዎት ወይም በልብ ወለድ የኮ

ቁ 901. በአፋጣኝ ማህበራዊ አከባቢዎ ውስጥ

ቁ 902. አንዳንድ ሊሆኑ የሚችሉ ምልክቶችን

q903. አንዳንድ ሊተላለፉ የሚችሉ ዘዴዎችን

ቁ 904. የተወሰኑ እርምጃዎችን ዘርዝሬያለሁ

ቁ .905. የኮሮናቫይረስ ድንገተኛ አደጋ ከጀመ

q906. በአሁኑ ጊዜ ኮሮናቫይረስን ወይም ክት

ቁ 907. የልብ ወለድ የኮሮናቫይረስ ልብ ወለ

ቁ 908. የኮሮናቫይረስ መስፋፋት ያሳሰባችኋ

ቁ 909. COVID-19 ን ለማግኘት የአደጋዎን

q910. የትኛው መልስ ትክክል ነው?

q911. በአዲሱ ልብ ወለድ የኮሮናቫይረስ በሽ

በ MNCH እንክብካቤ ልምዶች ላይ የ COVID-19 ተጽዕኖ

q912. በኤኤንሲ ጉብኝት ወቅት ኢንፌክሽን

q913. በኤኤንሲ ጉብኝት ወቅት ፅንሱ በበሽ
q914. Spread of infection in a carri
q915. የ COVID-19 ኢንፌክሽንን መፍራት
q916. በሚወልዱበት ጊዜ ከተቋሙ ኢንፌክ
q917. በሚወልዱበት ጊዜ ህፃን በበሽታው

For peer review only

Qo'annoon kun Biiroo Eegumsa fayyaa naannolee fi Dhaabbata JSI/L10K waliin kan gaggeeffamuudh

Kutaa 1: Gaaffilee eenyummaa fi heyyamaa

q101_ Lakkoofsa nama gaafatamuu/tuu

q106_Guyyaa ragaan kun itti sassaabame/Guyyaa Daawwannaa

Seensa/walbaruu fi eeyyama: Maqaan koo ${q105} jedhamaYeroo ammaa dhaabanni “ JSI-L10k”, kan Hirmaachuuf heyyemtaniittuu?

Daa'imnikee ji'a 12-15 darbe keessa dhalate lubbuun jiraa?

q107. Daa'imni kee inni xiqqaan (ji'a 0-15) ta'e maqaan eenyu?

q108. Saalli ${q107} maali?

q109. Umuriin ${q107} meeqa?

q111. Daa'ima kana dura daa'ima lubbuun dhalate qabduu ${q107}?

Q112. Daa'imni keessan kun daa'ima keessan meeqaffaa dha?

q114. Umuriin angafa daa'ima keessanii ${q107} meeqa?

Kutaa 2: Waa'ee haala jirenyaa fi maatii

q200. Umriin keessan waggaa meeqa?

q201. Dubbisuu fi barreessuu ni dandeessuu?

q202. Mana barumsaa idilee galtanii barattanii beektuu?

q203. Sadarkaan/kutaan barnoota idilee inni guddaan isin xumurtan meeqa/ isa kami?

q204. Amantaa kam hordoftu?

q205. Haala gaa'ilaa

q206. Dubbisuu fi barreessuu ni dandeessuu?

q207. Abbaan manaa keessan mana barumsaa idilee galanii baratanii beekuu?

q208. Sadarkaan/kutaan barnoota idilee inni guddaan abbaan manaa keessan xumuran meeqa/ isa ka

q210. Yeeroo meeqaaf ulfa/garaatti baattan (ulfa daa’imman lubbuun hin dhalatiin dabalatee)?

q211. Yeroo meeqa deessan?

q213. Hanga ammaatti ,walumaa galatti ijoolle/daa’imman meeqa deessan?(dhiiraa fi dhalaa)

q214. Da'umsa irratti daa'imni du'aan dhalachuun isin mudatee beekaa?

q215. Yoo deebiin gaaffii q214 eyyee ta'e, yeroo meeqa isin mudate?

q217. Maddi bishaan dhugaatii maatii keessani fayyadaman caalaatti maal? (Water for dirinking)

q220. Bishaan dhugaatiidhaaf oolu qulqulleessuuf waant isin gootan jira?

q221. Bishaan dhugaatidhaaf oolu qulqulleesuuf irra caalaa maal gootu?

q222. Iddoo/Bakkii maatiin keessan irra caalaan boobbaadhaaf itti fayyadamu eessa?

q223. Waan baaxiin manaa irraa hojjetame ilaaluudhaan guuti

q224. Girgiddaan manaa caalmaatti waan irraa hojjetamee ilaaluun deebii tokko guutii

q225. Mana jireenyaa keessan keessa kutaa meeqa hirribaaf fayyadamtuu?

q226. Miseensotni maatii keessanii lafa qonnaaf ta'u ni qabuu?

q227. Maatiin keessan Lafa qonnaa Heektaaraan (SAFARTUU NAANNOOTIIN) meeqa qabaa?

q228. Nyaata bilcheeffachuuf caalmaatti madda ho'aa maal fayyadamtu?

q229. Maatiin keessan meeshaalee asii gaditti ibsaman keessaa kamiin qabaa?

q229a. Ibsa elektirikii (Haaydiroo ykn soolaarii)

q229b. Sa’aatii

q229c. Raadiyoonii

For peer review only

q229d. Televizyinii

q229e. Telefoona Moobaayilaa

q229f. Teleefoona manaa(kan mobaayila hin taane)

q229g. Firiijii

q229h. Xarapheezaa (minjaala)

q229i. Teessuma

q229j. Siree

q229k. Eelee Eelektrikaa

q229l. Faanosa/Maashoo/kauraaza

q229m. Horiin hoolaa ykn re'ee

q229n. Meeshaalee miidhaginaa (Warqee/meetii)

q232. Baayina horii manaa, horii qonnaa fi lukkuuwwanii maatiin kun qabanii

Lukkuuwwan
Re'oota
Hoolota
Harroota
Fardoota
Gaangolii
Gaalota
Horii annanii
Sangoota/ korommii

kutaa 3: Hubannoo fi Dhiyeessa tajaajila fayyaa

q301hph. Kellaa fayyaa isa dhiyoo ga'uuf miillaan sa'aatii / daqiiqaa meeqa isinitti fudhata?

q301hch. Buufata fayyaa isa dhiyoo ga'uuf miilaan sa'aatii/ daqiiqaa meeqa isinitti fudhata?

q301. Ji'oota 12n dabran keessa Keellaa fayyaa deemtanii turtanii?

q302. Walumaa galatti yeroo dhumaaf tajaajila keella fayyaa irraa argattanitti hammam gammadoo

q303. Ji'oota jahan (6) dabre keessatti Hojjettuun Ekisteenshinii Fayyaa waa'ee fayyaa mari'achuuf m

q304. Waa'ee Rayyaa Misooma Fayyaa beektuu ykn wanti dhageessanii beektan jiraa?

q305. Isin DRM fayyaatii?(Dursituu1:5 ykn 1:30)

q306. Yoo DRMF keessaa tokko taatan, dursitu 1:5 moo kan 1:30ti?

q307. Miseensa abbaa warraa garee kee kan agarsiisu kaartaa garee ni qabdaa?

q308. Isin ykn DRMF ollaa keessan jiran kaartaa garee isaani hojjettanii qabuu?

q319. Ji'oota Jahan(6) dabran keessatti DRMF/Miseensa RMF waa'ee fayyaa isin mari'achuudhaaf ma

q310. Ji'oota Jahan(6) dabran keessatti DRMF waliin waa'ee fayyaa mana keessaniin alatti mar'attanii

q311. Yoo daa'imni keessan dhukkubsate/tte gorsaa fi wal'aansa barbaaddani argachuuf gufuu ykn ra

q311a. Bakka deemu wallaaluu

q311b. Deemuudhaaf hayyama dhabuu

q311c. Qarshii ittiin yaalamu dhabuu.

q311d. Dhaabbata tajaajila fayyaa itti kennamu dhiyeenyatti dhabamuu

q311e. Rakkoo geejibaa

q311f. kophaa koo deemu hin barbaanne/hin feene

q311g. Ogeessi tajaajila fayyaa kennu dubartii/shamarre hin jirtu jedhee waan yaadeef

q311h. Ogeessi tajaajila laatu kamiyyuu hin jiru jedhee waanan yaadda'uuf

q311i. Hojiin mana keessaa waan natti heddummateef/baayyateef...

q311j COVID-19

q311k. Rakko biroo Yoo jirate haa ibsamu

COVID-19 booda, Yoo daa'imni keessan dhukkubsate/tte gorsaa fi wal'aansa barbaaddani argachuuf g

q311l. Bakka deemu wallaaluu

q311m. Deemuudhaaf hayyama dhabuu

q311n. Qarshii ittiin yaalamu dhabuu.

q311o. Dhaabbata tajaajila fayyaa itti kennamu dhiyeenyatti dhabamuu

q311p. Rakkoo geejibaa

q311q. kophaa koo deemu hin barbaanne/hin feene

q311r. Ogeessi tajaajila fayyaa kennu dubartii/shamarre hin jirtu jedhee waan yaadeef

q311s. Ogeessi tajaajila laatu kamiyyuu hin jiru jedhee waanan yaadda'uuf

q311t. Hojiin mana keessaa waan natti heddummateef/bayyateef...

q311u. Rakko biroo Yoo jirate haa ibsamu

q313. Isin QFM dubbistanii beektuu? YKN. Namni kan biroo isinii dubbisee irratti mar'iattanii beektuu

q314. Isin ykn maatiin keessan qajeelfama fayyaa maatii qabduu?

q318. Yeroo ammaa kanatti maatiin keessan akka adda-dureetti ni beekamaa?

q324. Miseensi maatii kaleessa galgalaa saaphana siree kana jala rafe/bule jiraa?

q325. Eeyyee yoo ta'ee enyuutu jala rafe/ bulee?

q325a. Ana/Nama gaafatamuu

q325b. Daa'ima dhalattuu

q325c. Daa'immaan biroo (kan waggaa < 5)

q325d. Abbaa

q325e. Kan biroo

q326. Halkan dabre ${q107} n agoobaraa/ zaanziira jala bulte/bulee turee/ttee?

Kutaa 4. Fayyaa Haadholii

q403. Yeroo ${q107} ulfa turtan, fayyaa keessan ilaalchisee qaamni manatti dhufee isin ilaale tureera

q404. Eenyutu dhufe isin ilaalee?

q405. Yeroo ulfa turtan DRMF dhuftee isin ilaaltee beektii?

q406. Yeroo ${q107} ulfa turtan, hojjettoon fayyaa hawasaa (DRMF,HEF) kamiyyuu dhufanii mana ke

q407. Mariin kun isinii fi maatii keessan walin yeroo meeqa gaggeeffamee ture?

q410. Yeroo ${q107} ulfa turtan sana marii/Konfireensii durbatoota ulfaa irratti hirmattanii turtan?

q411. Yoo hirmattanii beektu ta'ee, yeroo ${q107}n ulfa taatan turtan yeroo meeqaa hirmattanii ture

q412. Yeroo ${q107} ulfa turtan tajaajila dahuumsaan duraaf dhaabbata fayyaa deemtanii beektu ykn

q413. Yeroo ${q107} ulfa turtan, jalqaba tajaajila da'uumsaan duraaf dhaabbata fayyaa kan deemtan

q414. Yeroo ${q107}n ulfa turtan tajaajila da'umsan dura /ulfa kanaa eessaa argattanii?

q416. Yeroo ${q107} ulfa turtan marsaa meeqaaf dhaabbata fayyaa deemtanii tajaajila/deeggarsa da

q417 Yeroo ${q107} ulfa turtan tajaajila da'umsa dura isa dhumaa kan fudhattan ulfa ji'a meeqaa ta

q418. Tajaajila kunuunsa ulfaa kellaa fayyaatti argattaniittuu?

For peer review only

q419. Yeroo meeqa tajaajila kunuunsa ulfaatiif kellaa fayyaa deemtan?

q420. Ulfi jiha meeqaa taataniiti kan tajajiila fayyaa da'uumsa duraa jalqabaaf itti eegaltani??

q421. Yeroo xumuraatiif gara keellaa fayyaa kunuunsa ulfaatiif gaafa deemtan, geejjiba akkamii fayya

q422. Sadarkaa itti quufinsa tajaajila kellaa fayyaatti argattanii nutti himuu dandeessuu? (itti quufeera

q434. Yeroo ${q107}n ulfa turtan tajaajila armaan gadii keessaa/kan isinii dubbisu keessaa kamtu yo

q434a. Ulfaatinni qaamaa safarameera

q434b. Safarri Dhiibbaa dhiigaa

q434c. Naamunaa qorannoo fincaanii

q434d. Naamunaa qorannoo dhiigaa.

q434e. Gorsa ykn odeefannoo waa'ee harmaa hoosisuu/luugsisuu

q434f. Odeeffannoo/Gorsa karoora maatii dahumsa boodaa

q434g. Gorsa waa'ee Ech ayivii Edisii"/HIV/AIDS ykn dhibeewwan naf-saalaa

q434h. Qorannoo waa'ee HIV/dhukkuba walqunnamtii naf-saalaatiin darbuu

q434i. Gorsa waa'ee sirna nyaata haadhoolee

q434j. Dawaa/kiniini hanqina dhiigaa kennameefiira.

q434k. Gorsa waa'ee mallattoo balaa cimaa yeroo ulfaa

q434l. Gorsa qophii dahumsaaf fi rakkoolee fayyaa wal xaxaa mudachuu danda'an

q434m. Gorsa waa'ee kunuunsa/deeggarsa daa'ima dhalattu godhamuu

q434n. odeeffannooo waa'ee rakkoo fayyaa wal xaxaa daa'imman dhalattu irra gahuu

q435hp. Yeroo ${q107}n ulfa turtan tajaajila armaan gadii kessa/kan isinii dubbisu keessa) kamtu yoo

q435a_hp. Ulfaatinni qaama safarameera

q435b_hp. safarri Dhiibbaa dhiigaa

q435c_hp. Naamunaa qorannoo fincaanii

q435d_hp. Naamunaa qorannoo dhiigaa.

q435e_hp. Dawaa /qoricha yaala dhukkuba busaa

q435f_hp. Gorsa ykn odefannoo waa'ee harmaa hoosisuu/lugisisuu

q435g_hp. Gorsa karoora maatii

q435h_hp. Gorsa waa'ee Ech ayivii Edisii"/HIV/AIDS

q435i_hp. Qorannoo waa'ee HIV/dhukuba walqunamitii saalatiin darbuu

q435j_hp. Gorsa waa'ee sirna nyaata haadhoolee

q435k_hp. Daawaa/kiniini yaala haniqina dhiigaa.

q435l_hp. Gorsa waa'ee mallattoo balaa cimaa yeroo ulfaa

q435m_hp. Gorsa qophii dahumisaaf taasifamuu

q435n_hp. Gorsa waa'ee kunuunsa/deggarsa daa'ima dhalattu godhamuu

q435o_hp. Odeeffanno waa'ee rakkoo hamaa daa'immani dhalattu irra gahuu

q436. Yeroo ${q107} ulfa turte lilimoo/marfee (talaalli) ittisa dhukkuba A'am danqar/"Teetaanasi"/ ce

q441. Yeroo ${q107} ulfa turtan sana haalli nyaata/soorrata keessanii kan yeroo biraa wajjin yoo mac

q442. Yeroo ${q107} ulfa turtan sana kininii ittisa hir'ina dhiigaa walitti fufinsaan fudhachaa turtanii?

q443. Yeroo ${q107} ulfa turtan sana kininii ittisa hri'ina dhiigaa walitti fufinsaan kan fudhattan ji'a m

q444. Yeroo ${q107} ulfaa turtan sana daawaa/qorichaa yaala raammoo garaa keessaa fudhattanii tu

Qophii Da'umsaan duraa

For peer review only

q445. Yeroo ${q107} ulfa turtan sana qophii da'umsa taasiftanii/gootanii turee?

q446. Yeroo ${q107} ulfa turtan dahumsa jala ennaa geessan qophii maalfaa gootanii turtan?

q447. Yeroo tokoo tokko haawwan/haadholiin yeroo ulfa isaanii sababii dhukkuba adda addaatiin ha

q448. Yeroo ${q107}n ulfa turtan mallattoole balaa cimaa yeroo ulfaa isin mudatee turee?

q449. Mallattooleen balaa cimaa isin mudatan maal faa ture?

Kunuunsa Da'umsaa fi Daa'ima dhalattuu

q500. ${q107} kan deessan eessatti ture?

q500a. yoo manatti deessan ta'e maaliif?

q500b. Yoo dhaabbata fayyaatti deessan ta'e maaliif?

q501. Yeroo ${q107}n da'uuf ciniinsuu irra turtan isin, maatii keessan ykn nama ollaa keessaa akka ga

q502. Bakka deessan ga'uuf mala geejibaa maal fayyadamtanii turtan?

q503. Deessanii booda gara manaatti deebitanii galuuf geejjiba maal fayyadamtan?

q504. Yeroo ${q107}n deessan eenyutuu isin deessisee /gargaare?

q505. Yeroo ${q107} deessan sana mala garaa baqaqsanii hodhuun deessan turee? Garaa keessan bac

q506.Ciniinsuun/dhukkubbiin osoo isinitti hin dhagahamiin dura dhaabbata fayyaa deemtanii achuma

q507. Osoo hin dahiin dura bakka turtii dubartoota ulfaa sana guyyoota meeqaaf turtan/teessan?

q507a. Erga deessanii booda bakka turtii dubartoota ulfaa sana turtanittuu?

q508. Erga deessanii booda sa'aatii hangamiif bakka turtii dubartoota ulfaa kessa turtan?

Harma hoosisuu

q509imm. Akkuma deessaniin harma hoosistanii?

q510. ${q107}n dhalatee harma osoo hin hoosisiin dura aannan isa jalqabaa (silga) maal gootan?

q511. ${q107} n yeroo dhalatu/ ttu madaalamee/madaalamtee turee?

q512. ${q107}n hammam madaala/tti ulfaata/tti ture?

q514. Yeroo ${q107}n dhalatee/tte, ulfaatinsa/ishee akkam turee? (Baayyee guddaa, giddu galeessa

q514a. ${q107}n kan dhalate/tte torban meeqaffaa isaa/sheetti ture? (yeroo isaatti, dafee, baay'ee da

q515. ${q107}n yeroo dhalatee/tte, handhuuruuf maal fayyadamtan?

q516. ${q107}n yeroo dhalatee/ttee handhuurri isaa/ishee maaliin hidhamee?

q517. ${q107}n yeroo dhalatee/ttee handhuurrii erga kutamee booda wanti diban ykn irra kaayyaan

q518. Kilooroheksidiinii(chlorhexidine) handhuuratti dibameeraa?

q519. Yoo eeyyee ta'ee, Kilooroheksidiinii(chlorhexidine) kun guyyaa meeqaaf dibamee turee?

q520. Akkuma dhalateen/tteen "TTC" kan ija daa'imaatti dibamuu dibamee turee?

q521. ${q107}n akkuma dhalatee/tteen, osoo obbaatiin/ofkaltiin hin bahiini duratti, dafanii wayyaan

q522. Daa'imni erga dhalatee yeroo hangamiitti booda uccuudhan qoorsani?

q523. ${q107}n akkuma dhalateen /tteen osoo obbaatiin hin bayiin duratti daftanii uccuu/wayyaa go

q524. Erga dhalaate/ttee booda sa'a meeqa keessatti hucuudhaan aguuganii/maranii turanii?

q525. Obbaatiin osoo hin ba'iin dura ${q107} f harma kennitaniifii?

q526. obbaatiin osoo hin ba'iin dura ${q107} eessa keessan /ciibsitan?

q527. ${q107}n akkuma dhalateen/tteen bo'ee/ssee ture/ttee?

q528. Akka ${q107} n rakkoo tokko malee boo'uu ykn afuuraa baafatuu/ttuu maaltu godhaameef tur

q529. ${q107} n akkuma salphaatti akka boo'uu/ssuuf namni gargaare eenyuu ture?

q530. ${q107}n dhalattee/te hangam turte/turee qaama ishee/isa dhiqixan?

q531. ${q107} n dhalatee/tte guyyoota sadii keessatti harma haadha malee akka dhuguuf/dhugduuf

q532. ${q107} n dhalatee/ttee guyyaa sadii keessatti harma haadhaa malee akka dhuguuf/dhugduuf
q533. Yeroo ${q107}n deessan sana akka ho'aa argatuuf/argattuuf ykn akka itti hin qorrineef wanti g
q534. Erga ${q107} n dhalatee/ttee booda akka itti hin qabbanoofneef ykn akka itti ho'uu jettaani ma
q535. ${q107} dhalatee/ttee booda hanga guyyoota 15tti halkanii fi guyyaa akka itti ho'uuf ofitti max
q536. ${q107} dhalatee/ttee booda hanga guyyoota 15tti halkanii fi guyyaa dabalatee caalmaatti eeny
q537. Dubartiin tokko yeroo dahumsaa, rakkoowwan wal xaxaa akkami yoo muudate/quunname wa
q538. Yeroo dahumsa, yoo dahumsi rakkisaa ta'e fakeenyaaf (kan akka bowoo cimaa, dhiiga hedduu k
q539. Yeroo dahumsa, yoo dahumsi rakkisaa ta'e fakeenyaaf (kan akka bowoo cimaa, dhiiga hedduu
q540. Yeroo deessan rakkoowwan dahumsaan wal qabatee isin mudatee turee?
q541. Yeroo ${q107}n deessan sana rakkoolee ulfaa fi dahumsaan wal qabatee isin mudateef tajaajila
q542. Yeroo ${q107}n deessan sana rakkoolee ulfaa fi dahumsaan wal qabatee isin mudateef tajaajila
q543. Sadarkaa itti quufinsa tajaajila da'umsaa argattan sanaa nuuf himuu dandeessuu?

Tajaajila da'umsa boodaa haadhaaf

q600. Isin, miseensi maatii keessani ykn ollaan keessan akka ${q107}n deessaanii battalumaatti HEFt
q601. Isin ykn miseensa maatii keessan keessaa namni waa'ee da'umsa keessanii akkumaa ${q107}n
q602. Erga ${q107} deessanii booda sa'aatii hagamiif dhaabbata fayyaa keessa turtan?
q603. Yeroo dhaabbata fayyaa keessa turtan sana, waa'ee fayyaa ${q107} ogeessi ilaalee tureraa?

Tajaajila da'umsa boodaa haadhaaf

q604. Erga ${q107}n deessanii booda hanga torbee 6tti ogeessi fayyaa kam iyyuu fayyaa keessan isin
q605. Erga ${q107}n deessan guyyaa meeqaa keessatti waa'ee fayyaa keessanii isa jalqabaa ilaalamta
q606. Isa jalqabaa eesaatti ilaalamtan?
q607. Isa jalqabaa eenyutu isin ilaale?
q607x. Tajaajila da'umsa boodaa yeroo meeqa argattan?
q608. Tajaajila wal'aansaa dahumsa boodaa yeroo lammaffaa ilaalamtanii turee?
q609. Erga ${q107}n deessan guyyaa meeqaa keessatti fayyaa keessan isa 2ffaa ilaalamtanii ture?
q610. Fayyummaa keessan mirkaneeffachuuf isa marsaa lammaffaa eessatti ilaalamtan?
q611. Yeroo isa 2ffaa ilaalamtan eenyuutu isin ilaalee turee?
q612. Yeroo sadaffaaf ilaalamtanii turtanii?
q613. Erga ${q107}n deessanii booda marsaa 3ffaa guyyaa meeqa booda ilaalamtan?
q614. Yeroo isa 3ffaa ilaalamtan eessatti ture?
q615. marsaa 3ffaa eenyuutu isin ilaale?
q616. Erga ${q107}n deessanii booda marsaa yeroo adda addaa ilaalamtan keessatti, fayyummaa kee
q617. Tajaajila da'umsaan boodaa argattan ilaachisee tajaajila kanatti quufuu fi quufuu dhiisuu kees

Tajaajila kunuunsa da'umsaan boodaa daa'imaaf

q618. Erga ${q107} deessanii booda hanga torbaan 6tti, deessisituu aadaa osoo hin dabalatiin ogeess
q619. Yoo deebiin eeyyee ta'e, ${q107}n erga dhalatee/ttee guyyaa meeqaa keessaatti yeroo jalqaba
q620. Yoo deebiin eeyyee ta'e, ${q107}n bakka kamitti ilaalamee/ttee turee?
q621. Yoo deebiin eeyyee ta'ee, ${q107}n eenyuutu ilaalee turee?
q621x. ${q107}n tajaajila da'umsa boodaa yeroo meeqa argate?
q622. ${q107}n yeroo lammaffaaf ilaalamee/ttee turee?
q623. Yoo deebiin eeyyee ta'e, ${q107}n erga dhalatee/ttee guyyaa meeqaa keessatti tajaajila isa lam
q624. Yoo deebiin eeyyee ta'e, ${q107}n marsaa 2ffaa bakka kamitti ilaalamee turee?

For peer review only

q625. Yoo deebiin eeyyee ta'ee, ${q107}n eenyuutu ilaalee ture?

q626. ${q107}n yeroo sadaffaaf ilaalamee turee?

q627. Yoo deebiin eeyyee ta'e, ${q107}n guyyaa hangamii booda marsaa3ffaa ilaalamee turee?

q628. Yoo deebiin eeyyee ta'ee, ${q107}n eessattii ilaalame ture?

q629. yoo deebiin eeyyee ta'e ${q107}n enyutu gargaare?

q630. ${q107}n yeroo marasaa adda addaa ilaalametti fayyummaa isaa mirkaneeffachuuf maal maal

q631.Tajaajila kunuunsa dahumsa boodaa ${q107}f keennameef ilalchisee itti qufuu fi dhisuu keessar

q632. ${q107}n yeroo deessan osoo bakkeetti hin baasiin guyyoota dheeraaf ykn torbeedhaaf mana k

q633. Guyyoota meeqaaf mana keessa tursitan

q634vist. ${q107}n yeroo deessan namni daa'ima ilaaluuf ykn gaafachuuf dhufee turee?

q635. Yeroo ${q107}n deessan namni daa'ima ilaaluuf ykn gaafachuuf dhufe kun yeroo jalqabaaf gu

q636. Yeroo ${q107}n deessan isin(haadha) malee namni biraan ${q107}n tuqee turee?

q637. Yeroo ${q107}n deessan isin(haadha) malee namni biraan ${q107}n osoo hin tuqiin guyyaa me

q638. Tajaajila da'umsaan boodaa ${q107}f kenname ilaachisee itti quufuu fi dhiisuu keessan irratti ya

Amma waa'ee gargaarsa yaalii daa'ima dhalattuu/kichuuf godhamuu malu isin gaafadha

q639. Yoo daa'imni dhalattuun fayyaa taatee, gara Buufata fayyaa ykn keellaa fayyaa geessuu hin bar

q640. Daa'imni dhalattuun sababoota amantaa ykn barsiifata adda addaa wal qabateen guyyaa murt

q641. Yeroo tokko tokkoo haadholiin erga dahanii booda baayyee waan dhukkubsataniif gara dhaabl

q642. Dahumsa booda rakkooleen hatattamaan gara dhaabbilee fayyaa akka deemtan kan isin taasis

q642. Eeyyee yoo ta'e mallattoolee ykn rakkoolee maalfaatu akka gara dhaabbata fayyaa deemtan is

Soorrata daa'imaa fi Muuxannoo nyaata daa'imaa qopheessuu

q705. Kaleessa sa'aati kana irraa jalqabee, ${q107} n harmaan alatti akka nyaatu/ttu ykn akka dhugu/

q706. Kaleessa sa'aati kana irraa jalqabee, ${q107} n harmaan alatti akka nyaatu/ttu ykn akka dhugu/

q707. Aannan harmaa irraa kan hafe dhugaatiis ta'e nyaata dabalataa daa'imaaf maaliif ture kan kenn

q708. Amma ${q107} harma hoosisaa jirtuu?

q709. ${q107} maaliif harma hoosisuu dhiiftee/dhaabdan?

q710 . ${q107} hanga waggaa meeqa guututti harma hoosisuu feetuu/barbaduu?

q711. ${q107} n kaleesaa ganamaa hanga har'a ganamaatti yeroo meeqaa harmaa hoosistanittuu?

q712. ${q107}n kaleessa ganamaa hanga har'a ganamaatti dhangala'aa xuuxxoodhaan hoodheeraa?

q713. Nyaata ykn dhuugatii dabalata gaafa ${q107} n umrii meeqa ga'ee irraa jalqabdanii kennitaniif

q714. ${q107}n kaleessaa ganamaa hanga har'a ganamatti yeroo meeqaa nyaata jabaa ykn laafaa kee

q715. Gosa nyaataa isin ${q107} f kaleessa ganama irra jalqabee hanga hara ganamaatti keenitaaniif

q716. Daa'imakee ${q107}n talaalliitti geessitee beektaa?

q717. Daa'imnikee ${q107}n kaardii kan ragaan talaalli isaa itti guutamu qabaa?

q718. Daa'imnikee ${q107}n talaalli umuriisaa waliin sirrii ta'e fudhateeraa?

q719. Erga ${q107} deessanii booda ji'oota lamaan jiran keessatti viitaamiin **"A"** fudhattani turtanii?

q720. Ji'oottan jahan dabran keessatti ${q107} n Viitaamina A argatte/te turee?

Section12. Haala kunuunsa tajaajila fayyaa daa'imaa dhalattuu fi daa'immaniif

Amma waa'ee dhibee fayyaa daa'imnike umurii isaa ji'a lamaan jalqabaa (0-2months) keessatti isa

q800. Daa'imnikee ${q107}n umurii isaa ji'a lamaan duraa (ji'a 0-2) keessatti dhukkubsatee beekaa?

q801. Daa'imnikee ${q107}n takkaa mallattoowwan armaan gadii kana agarsiisee beekaa?

q802. Yoo dhukkubsatee ykn mallattoo kana keessaa agarsiisee beeka ta'e ${q107}n umurii isaa hamr

q803. Dhibeen daa'imakee ${q107} yeroo sanaa maal ture?

For peer review only

q804. Daa'imakee ${q107} yeroo inni dhukkubsate manakeetii alatti yaalchisuuf barbaaddee turtee?
q806. yoo eeyyee ta'e yeroo meeqa yaalchisuuf barbaadde?
q805.Yoo daa'imakee ${q107}, manakeetii alatti yaalchisuuf hin barbaanne / yaalle ta'e sababni isaa n
q807. Manakeetii ala eessatti yaalchisuuf barbaaddee turte?
q808. Daa'imnikee dhukkubsatee guyyaa meeqa booda yaalchisuuf barbaaddee turte?
q809. Yeroo daa'imnikee dhukkubsate yaalchiste qorichi/dawaan ${q107} n fudhate jiraa?
q810. Daa'imnikee ${q107}n qoricha maal fudhate?
q811. ${q107} n qoricha/ dawaa guyyaa meeqaaf fudhate?
q812. Tajaajila daa'imakeef kenname kanatti gammaduu fi dhiisuukee nutti himuu dandeessaa?
sat. Tajaajila yaalii daa'imakeef sadarkaa kellaa fayyaatti kenname akkamitti sadarkeessita?
q812a. Tajaajila gorsaa kenname
q812b. Yeroon tajaajilli CBNC itti kennamu mijataa ta'uu
q812c. Yeroo itti kunuunsa daa'ima kichuuf ogeessa itti mariisisan mijataa ta'uu
q812d. Dandeettii HEF tajaajila CBNC kennituu
q812e. Beellama yaalii mijataa ta'e kennuu
q812f. Yeroon kellaan fayyaa itti tajaajila kennu mijataa ta'uu
q812g. Kellaa fayyaa keessa qorichi ga'aan jiraachuu
q812h. Akkaataa itti hojjettuun ekisteenshinii fayyaa itt nama keessummeessitu
q812i. Qulqullina iddoo turtii kellaa fayyaa jiru (Daiting area)
q812j. Iddoon turtii kellaa fayyaa maamiltootaaf mijataa ta'uu.

Daa'imni lubbuun kan hinjirre (guyyaa 28 utuu hinguutin kan du'e)
q813. ${q107}n utuu hindu'in/ hindadhabin dura mallattoo akkamii agarsiise?
q814. Yoo ${q107}n dhukkubsatee du'e ta'e, yaalii argatee turee?
q815. Yoo yaalii argateera ta'e eenyutu yaale/ eenurraa argate?
q816. Yoo ${q107}n utuu hindu'in/dadhabin yaalameera ta'e dhibeen isaa ogeessaan adda ba'eeraa?

Kutaa 7. Beekumsaa fi ilaalcha waa'ee COVID-19 irrati jiru.
q900. COVID-19n qabamtee beektaa?
q901. Hiriyoota ykn namoota siwaliin hojjetan keessaa kan COVID-19n qabamee at beektu jira?
q902. Amma mallattoolee tokko tokkon tarreessa. Mallattooleen kun mallattoo COVID-19 ta'uus ta'u
q903. Amma karaalee dhibeen ittiin daddarbuu danda'un tarreessa. Karaaleen kun karaa COVID-19 it
q904. Amma maloota ittiin dhibee of irraa ittisuun ittiin danda'amu tokko tokkon tarreessa. Maloota l
q905. Ergaa weerarri koronaa biyyakeenya galee as dhibee kana to'achuuf tattaaffii godhamaa ture ke
q906. Yeroo ammaa kan qorichi ittiin COVID-19 yaalan ykn talaalliin isaa ni jira jettee amantaa?
q907. Namni tokko yoo vaayirasii COVID-19n faalame guyyaa meeqa booda mallattoo dhibee COVID-1
q908. Waa'een COVID-19 kun siyaachisaa?
q909. Carraa COVID-19n qabamuukeetii akkamitti ilaalta? (hinjiru, xiqqaa dha, guddaa dha, baay'ee gu
q910. Deebii kamtu sirriidha?
q911. Carraan COVID-19n qabamuukeetii hammam ta'a jettee yaadda?
Dhiibbaa COVID-19 tajaajila MNCH irratti qabu
q912. Yeroo tajaajila ANCf dhaabbata fayyaa dhaqan nan qabama jedhanii sodaachuu

q913. Yoon ANCf gara dhaabbata fayyaa deeme daa'imni garaakoo keessa COVID-19n qabamuu dand

q914. Yeroo dhaabbata fayyaatti yaaliin kennamu saffisaan babal'ata jedhanii yaaduu

q915. Sababii COVID-19tiin tajaajilli ANC, Da'umsaa fi PNC si'aayina qabu hinkennamu jedhanii sodaac

q916. Dhaabbata fayyaa keessaa COVID-19 nama qaba jedhanii sodaachuu

q917. Yeroo da'umsaa daa'imni dhalatu COVID-19n qabama jedhanii sodaachuu.

For peer review only

1 hanga 50

Hub: Yoo ummuurriin

Remember to answer

Daa’imni keesan kun (ma
Umuriin angafa da’immar ምዕባዩ ንዘብሉ 999 ይምልኡመ

waggaa 15 hanga 49

Waaqeffataa

If don't know enter 9
kan lubbuudhan hin d
yoo hin jiruu ta'ee 0 b

deebi tokko qofa fuud

filannoo hin dubisin;
Deebii tokko fuudhi

Yoo kutaa tokko qofa

(Deebii heektaaraan

hundumaa dubsiifitii

For peer review only

Please write in minu

Please write in minu

Rayyaan misooma fa

DRMF jechuun geegg

Kaartaa garee ilaali

DRMF jechuun geegg

DRMF jechuun geegg

Filannoo dubbiisaaf

For peer review only

waraqaa ragaa itti ag

Qajeelfama fayyaa m

Filannoo hin dubbiisi

Kan bira hoojedhii ga

Gaaffiin kun kan gaaj

yoo hin yaadatan ta'e

Yoo sagalii ol ta'e '9'

Yoo hin yadatan ta'ee

Yoo hin yaadadhu jed

Yoo hin yaadadhu jed

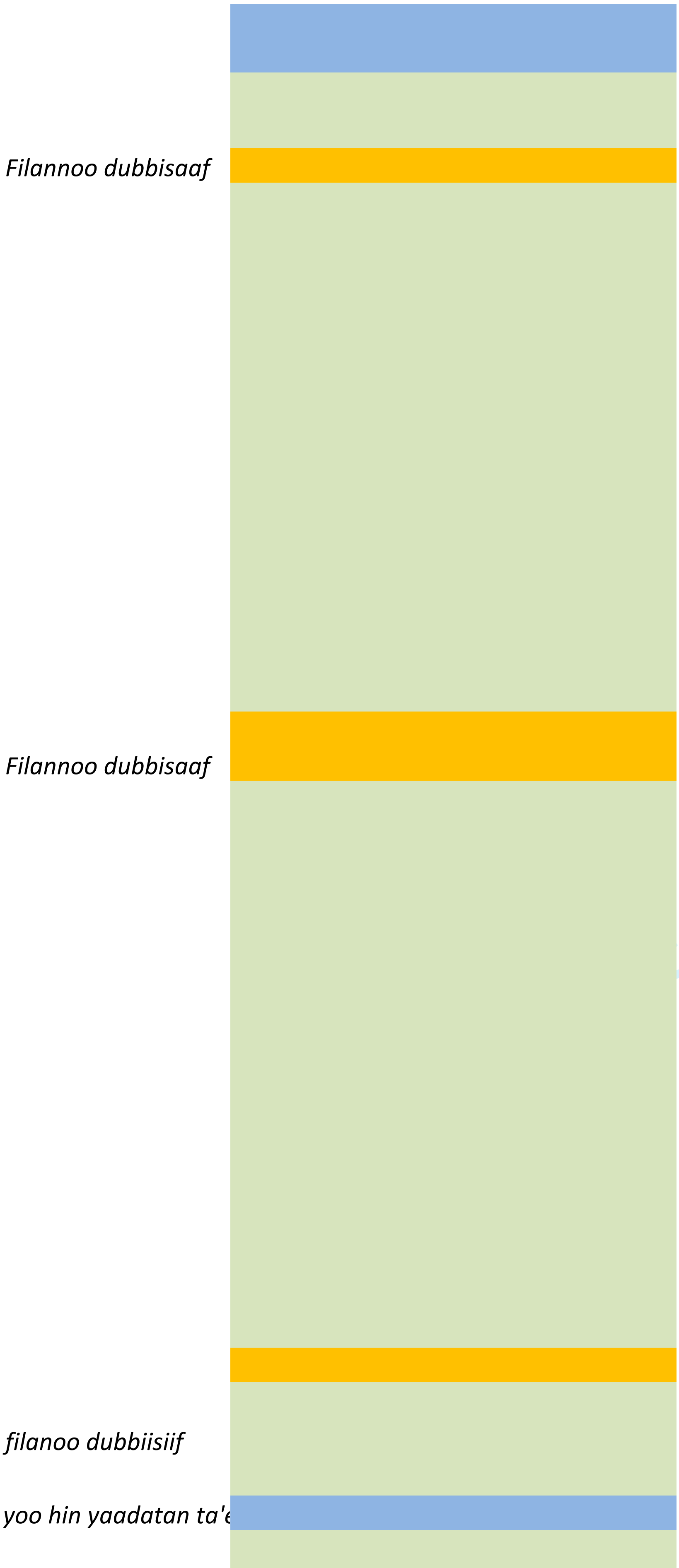
Filannoo dubbisaaf
Filannoo dubbisaaf
filanoo dubbiisiif
yoo hin yaadatan ta'e

only

Yoo barbaachisaa ta'

Kan bira hoo? Filanno

Filannoo hin dubbisin

Filannoo hin dubbisin

Deebii isaan ibsan hi

Deebii isaan ibsan hu

Filannoo hin dubbisiir

Filannoo hin dubbisiir

Kan biroo jechaa nam

Sa'aatii dhaan guuti,y

Sa'aatii tokko keessat

Kaardii daa'imaa ilaa

Baayina guyyaa barre

Sa'aatiin guuti, batta

Sa'aatiin guuti, batta

Kan biroo jechuuun g

sa'aan galmeessi, guy

Filannoo hin dubbisiin

Filannoo hin dubbisiin

Filannoo hin dubbisin

Filannoo hin dubbisin

Gaaffiin kun warra m

Gaaffiin kun warra dh

Sa'aatiidhaan guuti,Y

Gaaffiin kun warra dh

Yeroo deessan maal n

Hin jiru yoo ta'e tajac

Baayina guyyaa barre

Baayina guyyaa barre

Baayina guyyaa barre

Filannoo dubbisi, dee

kunuunsa yerroo dha

Baayyina guyyaa bar

Eenyuummaa ogeesa

Baayina guyyaa barre

only

Eenyuummaa ogeesa
Filannoo dubbisiif dee

Isa dhumaa guyyaa 5
Hunda gaafadhu
Nama sababa kamiifu
Yoo guyyaa tokko gaa
Tuquu jechuun: qaam
Haala barsiifata ulum

Filannoo hin dubbisin

Filanoo dubbiisaaf. D
Sagalee ol kaasii dubl

Filannoo hin dubbisiin
Deebii ji'aan(yoo hin
Deebii lakkoofsaan ar

Deebii akka lakkoofsa

Kan biroo jedchuun g

Bilqaxii ykn kininnii it
Bilqaxii ykn kininii itti

For peer review only

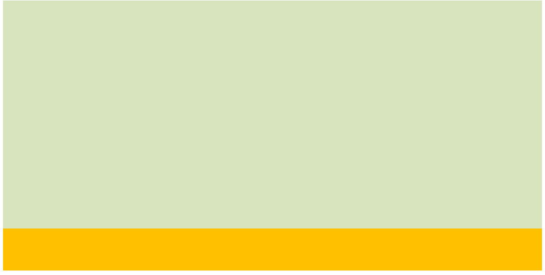

For peer review only

list_name	value	label	image
yesno	1	Yes	
yesno	0	No	
gender	1	Male	
gender	2	Female	
grade	1	1st	
grade	2	2nd	
grade	3	3rd	
grade	4	4th	
grade	5	5th	
grade	6	6th	
grade	7	7th	
grade	8	8th	
grade	9	9th	
grade	10	10th	
grade	11	11th	
grade	12	12th	
grade	13	Technical/vocational certificate	
grade	14	University/college diploma	
grade	15	University/college degree or higher	
religion	1	Orthodox	
religion	2	Catholic	
religion	3	Protestant	
religion	4	Muslim	
religion	5	Traditional	
religion	6	Other	
birthdayverified	1	Child's date of birth verified	
birthdayverified	2	Not possible to verify	
yesnodk	1	Yes	
yesnodk	0	No	
yesnodk	8	Don't know	
yesnodkmwh	1	Yes	
yesnodkmwh	2	No	
yesnodkmwh	8	Don't know	
yesnodkmwh	9	Stays at MWH	
watersource	1	Piped into dwelling	
watersource	2	Piped into compound	
watersource	3	Piped outside compound	
watersource	4	Covered Well	
watersource	5	Protected Spring	
watersource	6	Protected hand pump spring	
watersource	7	Open Well	
watersource	8	Open Spring	
watersource	9	River	
watersource	10	Pond/Lake/Dam	
watersource	11	Rainwater	

For peer review only

watersource	12	Other	
compound	98	Yes, water source in the compound	
compound	2	No	
treat	1	Boil	
treat	2	Add bleach/chlorine	
treat	3	Strain it through a cloth	
treat	4	Use water filter (ceramic, sand, compo	
treat	5	Solar disinfection	
treat	6	Let it stand and settle	
treat	7	Other	
treat	8	Do not Know	
toilet	1	Pit Latrine/traditional pit toilet	
toilet	2	Ventilated improved pit latrine (VIP)	
toilet	3	Flush toilet	
toilet	4	No facility/Bush/Field	
toilet	5	Other	
roof	1	Thatch/leaf	
roof	2	Rustic mat/plastic sheets	
roof	3	Reed/bamboo	
roof	4	Wood planks	
roof	5	Finished roofing Corrugated iron	
roof	6	Wood	
roof	7	Calamine/cement fiber	
roof	8	Cement/concrete	
roof	9	Roofing shingles	
roof	10	Other	
wall	1	No walls	
wall	2	Cane/trunks/bamboo/reed	
wall	3	Rambo/wood	
wall	4	Stone with mud	
wall	5	Uncovered adobe	
wall	6	Plywood	
wall	7	Cartoon	
wall	8	Cement	
wall	9	Stine with lime cement	
wall	10	Bricks	
wall	11	Cement blocks	
wall	12	Covered adobe	
wall	13	Wood planks/shigles	
wall	14	Other	
mention	1	Mentioned	
mention	2	Did not mention	
satisfy	1	Not satisfied	
satisfy	2	Satisfied	
satisfy	3	Very satisfied	
problem	1	Big problem	

For peer review only

problem	2	Small problem	
problem	3	No problem	
havefhc	1	Yes, FHC seen	
havefhc	2	Yes, FHC NOT seen	
havefhc	3	No	
model	1	Yes, graduated (Certificate seen)	
model	2	Yes, graduated (Certificate not seen)	
model	3	No, working towards	
model	4	Not at all	
bnet	1	Permanent net (Permanent)	
bnet	2	Pretreated net	
bnet	3	Untreated net	
bnet	8	Do not know	
reason	a	Family planning	
reason	b	Child immunization	
reason	c	Antenatal care	
reason	d	Postnatal care	
reason	e	Health education	
reason	f	Growth monitoring	
reason	g	Referral of sick child	
reason	h	Diarrhea treatment	
reason	i	Malaria treatment	
reason	j	Pneumonia treatment	
reason	k	Provide or sell bed nets	
reason	l	Delivery care	
reason	m	Neonatal care	
reason	n	Haygine and sanitation	
reason	o	Other	
isource	a	Health Extension Worker	
isource	b	HDAs	
isource	c	TBA	
isource	d	Health worker	
isource	e	Kebele administrators	
isource	f	Don’t know	
isource	g	Other	
youeat	1	More	
youeat	2	Same	
youeat	3	Less	
youeat	8	Don't Know	
stopbf	a	Mother ill/weak	
stopbf	b	Child ill/weak	
stopbf	c	Nipple/breast problem	
stopbf	d	Not enough milk	
stopbf	e	Mother working	
stopbf	f	Child refused	
stopbf	g	Weaning age/age to stop	

For peer review only

stopbf	h	Became pregnant	
stopbf	i	Started using contraception	
stopbf	j	Other	
agefeed	1	Before 4 months	
agefeed	2	Between 4 and 6 months	
agefeed	3	Exactly when it is 6 month of age	
agefeed	5	After 6 months	
agefeed	4	Not started any supplementation	
agefeed	98	Don’t Know	
feed	a	Breastmilk	
feed	b	Water	
feed	c	Formula	
feed	d	Milk other than breastmilk	
feed	e	Fruit Juice	
feed	f	Other liquids (sugar water, coffee, tea,	
feed	g	Any food made from grains (millet, sor	
feed	h	Any food made from pumpkins, carrots	
feed	i	Any other food made from roots or tub	
feed	j	Any other fruits and vegetables? (e.g.,	
feed	k	Meat	
feed	l	Any food made from legumes (e.g. lent	
feed	m	Any food made with oil, fat or butter?	
feed	n	Cooked mashed foods	
feed	o	Egg	
feed	p	Fish	
feed	q	Cheese, Yoghurt	
feed	r	Other, sepcify	
ddrink	1	Much Less	
ddrink	2	Somewhat less	
ddrink	3	About the same	
ddrink	4	More than usual	
ddrink	5	Nothing to drink	
ddrink	8	Don’t Know	
ddrink	9	Did not start fluid	
deat	1	Much Less	
deat	2	Somewhat less	
deat	3	About the same	
deat	4	More than usual	
deat	5	Never gave food	
deat	6	Not started any food	
deat	8	Don't know	
dseek	a	government hospital	
dseek	b	government health center	
dseek	c	government health station/clinic	
dseek	d	government health post	
dseek	e	government community-Based Outlet	

For peer review only

dseek	f	NGO Health facility	
dseek	g	NGO Community-Based Outlet	
dseek	h	Private Hospital	
dseek	i	Private/community doctor/clinic	
dseek	j	Private Pharmacy	
dseek	k	Holy Water	
dseek	l	Shop	
dseek	m	Friend/Relative	
dseek	n	Traditional practitioner	
dseek	o	Other	
dtreat	1	Antibiotic pill or syrup	
dtreat	2	Antimotility pill or syrup	
dtreat	3	Zinc pill or syrup	
dtreat	4	Other pill or syrup (anti-biotic, antimot	
dtreat	5	Unknown pill Or syrup	
dtreat	6	Antibiotic injection	
dtreat	7	Non-antibiotic injection	
dtreat	8	Unknown injection	
dtreat	9	(iv) intravenous injection	
dtreat	10	Home remedy/ herbal medicine	
dtreat	12	Nothing more given	
dtreat	12	Other	
chest	1	Chest only	
chest	2	Nose only	
chest	3	Both	
chest	8	Other/don't know	
drug	a	Chloroquine	
drug	b	Artemether lumefantrine	
drug	c	Quinine	
drug	e	Bacterim	
drug	f	Ampcilin	
drug	g	Amxycilin	
drug	h	Chloriamphinicol	
drug	i	Tetracyclin	
drug	j	Other anti-biotic	
drug	k	Asprin	
drug	l	Ibuproen	
drug	m	Parcytamol	
drug	n	Other	
dnewborn	a	Vomiting	
dnewborn	b	Fever	
dnewborn	c	Poor sucking or feeding	
dnewborn	d	Baby has difficulty/fast breathing	
dnewborn	e	Baby feels cold	
dnewborn	f	Baby too small or born too early	
dnewborn	g	Redness/discharge around cord	

For peer review only

dnewborn	h	Red swollen eyes/discharge	
dnewborn	i	Yellow palms/soles/eyes	
dnewborn	j	Lethargic or unconscious	
dnewborn	k	Convulsions	
dnewborn	o	Baby irritated/cries without any appare	
dnewborn	n	Nothing mentioned	
dnewborn	l	Other	
dnewborn	m	Don't know	
dchild	a	Repeated Watery Stools	
dchild	b	Any Watery Stools	
dchild	c	Repeated Vomiting	
dchild	d	Any Vomiting	
dchild	e	Blood in Stools	
dchild	f	Fast Breathing	
dchild	g	Difficult Breathing	
dchild	h	Noisy Breathing	
dchild	i	Fever	
dchild	j	Convulsions	
dchild	k	Stiff Neck	
dchild	l	Marked Thirst	
dchild	m	Unable to Drink	
dchild	n	Not Eating/Not Drinking Well	
dchild	o	Getting Sicker/Very Sick	
dchild	p	Not Getting Better	
dchild	q	Sick for a long time	
dchild	r	Sunken Eyes	
dchild	s	Cough	
dchild	t	Other	
dchild	u	Don’t Know	
ancsource	a	Hospital	
ancsource	b	Health Center	
ancsource	c	Health post	
ancsource	d	NGO health facility	
ancsource	e	Private health facility	
ancsource	f	Other	
ancsource	g	Do not know	
aprovider	a	Doctor	
aprovider	b	Nurse/midwife	
aprovider	c	Health extension worker	
aprovider	d	Health worker/unknown type	
aprovider	e	Other	
peat	1	Less	
peat	2	Same	
peat	3	More	
peat	8	Don’t Know	
bprepare	a	Financial	

For peer review only

bprepare	b	Transport	
bprepare	c	Food	
bprepare	d	Arrange birth attendants	
bprepare	e	Identified health facility for delivery	
bprepare	f	Prepared clean and appropriate materi	
bprepare	g	Identified blood donors	
bprepare	i	Arrange a person who will care for the	
prepare	h	Other	
plandelp	1	My Home	
plandelp	2	Other Home	
plandelp	3	Government Hospital	
plandelp	4	Government Health Center	
plandelp	5	Health post	
plandelp	6	NGO Health Facility	
plandelp	7	Private Hospital	
plandelp	8	Private Doctor/Clinic	
plandelp	9	Other	
transport	a	Gari	
transport	b	Animals (horse, mule)	
transport	c	Stretcher	
transport	d	Ambulance	
transport	e	Public transport	
transport	f	Government office vehicle	
transport	g	Walk	
transport	i	Carried by relatives	
transport	h	Other	
dassist	a	Doctor	
dassist	b	Nurse/midwife	
dassist	c	Health extension worker	
dassist	d	HAD	
dassist	e	traditional birth attendant	
dassist	f	Relative/friend	
dassist	g	Nobody	
dassist	h	Other(specify)	
fmilk	1	Squeeze out and throw	
fmilk	2	Squeeze out and give to the baby	
fmilk	8	Other/don’t remember	
bsize	1	Large	
bsize	2	Average	
bsize	3	Small	
bsize	4	Born too early	
bsize	8	Don’t know	
cut	1	New Blade	
cut	2	Boiled Blade	
cut	3	Unboiled used blade	
cut	4	Knife	

For peer review only

cut	5	Scissor	
cut	6	Do not know	
cut	7	Other (specify)	
cordtie	1	New string or thread	
cordtie	2	Boiled string or thread	
cordtie	3	Used string or thread	
cordtie	4	Did not tie the cord	
cordtie	8	Do not know	
cordtie	111	Other (specify)	
apply	a	Butter	
apply	b	Oil	
apply	c	Ash	
apply	d	Ointment/powder	
apply	e	Animal dung	
apply	f	Cold water	
apply	h	Chlorhexidine	
apply	g	Other/don’t remember	
babyplaced	1	Alone/on the floor	
babyplaced	2	On the mother’s Belly/chest	
babyplaced	3	Beside the mother	
babyplaced	4	With someone else	
babyplaced	5	On the cot	
babyplaced	7	Don’t know	
babyplaced	6	Other	
babybreath	a	Rubbed/massaged/Stimulating	
babybreath	b	Dried	
babybreath	c	Mouth cleared	
babybreath	d	Mouth to mouth resuscitation	
babybreath	e	Ambu bag used	
babybreath	f	Nothing	
babybreath	i	Suction	
babybreath	h	Don't know	
babybreath	g	Other	
wbreath	1	Health worker	
wbreath	2	Health Extension Worker	
wbreath	3	HDA	
wbreath	4	Traditional birth attendant	
wbreath	7	Family/relative/friend	
wbreath	8	Self	
wbreath	9	Other	
gdrink	a	Milk (other than breast milk)	
gdrink	b	Plain water	
gdrink	c	Sugar or glucose water	
gdrink	d	Fruit juice	
gdrink	e	Infant Formula	
gdrink	f	Tea/infusion/”hamesa”	

For peer review only

gdrink	g	Fresh butter	
gdrink	h	Other	
bwarm	a	Dried the baby	
bwarm	b	Wrapped the baby with clean cloth	
bwarm	c	Put baby beside the mother	
bwarm	d	Keep the baby on bare skin to skin con	
bwarm	e	Bathed in warm water	
bwarm	f	Warmed delivery room	
bwarm	g	Other	
skin	1	Always	
skin	2	Often	
skin	3	A few times	
skin	4	Never	
skin	5	Don't know	
sleep	1	Mother slept with baby	
sleep	2	Laid baby on bed alone	
sleep	3	Baby slept with another person	
sleep	4	Keep the baby on bare skin to skin con	
sleep	5	Other (specify)	
gofcomp	a	Hospital	
gofcomp	b	Health center	
gofcomp	c	Health post	
gofcomp	d	Drug shop/pharmacy	
gofcomp	f	Health development army leaders (HD	
gofcomp	g	TBA/TTBA	
gofcomp	h	Holy water/religious place	
gofcomp	i	Traditional healer/Witchcraft	
gofcomp	j	Other(specify)	
pregcomp	a	Severe headache	
pregcomp	b	Blurred vision	
pregcomp	c	Reduced fetal movement	
pregcomp	d	Unconsciousness	
pregcomp	e	Convulsions	
pregcomp	f	Vaginal bleeding	
pregcomp	i	Severe lower abdominal pain	
pregcomp	j	Fever	
pregcomp	g	Swollen hands and face	
pregcomp	h	No symptom mentioned	
nbcplace	1	Your home	
nbcplace	2	Other home	
nbcplace	3	Health post	
nbcplace	4	Health center	
nbcplace	5	Hospital	
nbcplace	8	Other(specify)	
vita	1	Yes	
vita	2	No	

For peer review only

vita	3	Child age <6months	
vita	8	Do not know	
itemforbaby	a	Vitamin supplements	
itemforbaby	b	Plain water	
itemforbaby	c	Sweetened water or juice	
itemforbaby	d	ORS	
itemforbaby	e	Infant formula	
itemforbaby	f	Milk	
itemforbaby	g	sugar or glucose water	
itemforbaby	h	Tea/infusion/"hamesa"	
itemforbaby	i	Butter	
itemforbaby	j	Other liquids	
itemforbaby	l	Do not know	
dbfeed	1	Much Less	
dbfeed	2	Somewhat less	
dbfeed	3	About the same	
dbfeed	4	More than usual	
dbfeed	5	Did not breastfeed	
dbfeed	8	Don't Know	
KAPlevel	1	Agree a lot	
KAPlevel	2	Agree somewhat	
KAPlevel	3	Disagree somewhat	
KAPlevel	4	Disagree a lot	
HDAtype	1	1:5 leader	
HDAtype	2	1:30 leader	
mapavl	1	Yes displayed on the wall	
mapavl	2	Yes not displayed on the wall	
mapavl	3	Not available	
yesnoNA	1	Yes	
yesnoNA	2	No	
yesnoNA	3	Not Applicable	
dassistp	1	Doctor	
dassistp	2	Nurse/midwife	
dassistp	3	Health extension worker	
dassistp	9	Health worker (unknown type)	
dassistp	4	HAD	
dassistp	5	traditional birth attendant	
dassistp	6	Relative/friend	
dassistp	7	Nobody	
dassistp	8	Other(specify)	
satisflevel	1	Fully satisfied	
satisflevel	2	Somewhat satisfied	
satisflevel	3	Somewhat dissatisfied	
satisflevel	4	Fully dissatisfied	
yesnodknr	1	Yes	
yesnodknr	2	No	

For peer review only

yesnodknr	98	Don’t know	
pnccheckplace	1	In own home	
pnccheckplace	2	Health post	
pnccheckplace	3	Health center	
pnccheckplace	4	Hospital	
pnccheckplace	5	Other	
whodidpnc	1	Doctor	
whodidpnc	2	Nurse/midwife	
whodidpnc	3	HEW	
whodidpnc	4	Health Officer	
whodidpnc	5	HDA	
whodidpnc	8	TBA	
whodidpnc	6	Do not know	
whodidpnc	7	Other	
whodidpncmom	1	Doctor	
whodidpncmom	2	Nurse/midwife	
whodidpncmom	3	HEW	
whodidpncmom	4	Health Officer	
whodidpncmom	8	TBA	
whodidpncmom	6	Do not know	
whodidpncmom	7	Other	
pnccompomom	a	Checked breasts	
pnccompomom	b	Advised breast feeding	
pnccompomom	c	Oriented about danger signs	
pnccompomom	d	Educated on family planning	
pnccompomom	e	Information given on nutrition	
pnccompomom	f	Referred to a health facility	
pnccompomom	g	Measured Blood Pressure	
pnccompomom	h	Checked/treated birth-related wound (	
pnccompomom	j	Family planning counselling	
pnccompomom	k	Assessed temperature	
pnccompomom	l	Assessed breathing	
pnccompomom	i	Other	
pnccompobaby	a	Generally examined /looked at baby's l	
pnccompobaby	b	Weighed baby	
pnccompobaby	c	Checked cord	
pnccompobaby	d	Advised breastfeeding/exclusive breast	
pnccompobaby	e	Observed breastfeeding	
pnccompobaby	f	Advised skin-to-skin contact/warmth	
pnccompobaby	g	Checked baby for danger signs (includi	
pnccompobaby	h	Educated on danger signs	
pnccompobaby	i	Referred to a health facility	
pnccompobaby	j	Provided information on washing hand	
pnccompobaby	k	Advised keeping the cord clean	
pnccompobaby	l	Advised not to bathe the baby within 2	
pnccompobaby	n	Child immunization	

For peer review only

pnccompobaby	m	other	
reasonfornonBF	a	My breast milk is not enough	
reasonfornonBF	b	I do not stay with NAME throughout th	
reasonfornonBF	c	Advised by friends or relatives to do so	
reasonfornonBF	d	Tradition or culture	
reasonfornonBF	e	Other	
cookingfuel	1	Mainly firewood (purchase or collectec	
cookingfuel	2	Animal dung	
cookingfuel	3	Crop residue	
cookingfuel	4	Charcoal	
cookingfuel	5	Kerosene	
cookingfuel	6	Butane gas	
cookingfuel	7	Electricity	
cookingfuel	8	Bio gas	
cookingfuel	10	Does not use any cooking fuel or No fo	
dangerpnc	a	Excessive vaginal bleeding	
dangerpnc	b	Foul-smelling discharge	
dangerpnc	c	High fever	
dangerpnc	d	Severe lower abdominal pain	
dangerpnc	e	Convulsions	
dangerpnc	h	Unconsciousness	
dangerpnc	i	Severe headache	
dangerpnc	j	Blurred vision	
dangerpnc	k	Calf pain	
dangerpnc	l	postpartum psychosis	
dangerpnc	g	Nothing mentioned	
dangerpnc	f	Other	
dangercb	a	Excessive vaginal bleeding	
dangercb	b	Foul-smelling discharge	
dangercb	c	High fever	
dangercb	d	Baby’s hand or feet come first	
dangercb	e	Baby’s in abnormal position	
dangercb	f	Prolonged labor (>12 hours)	
dangercb	g	Retained placenta	
dangercb	h	Rupture uterus	
dangercb	i	Prolapsed cord	
dangercb	j	Cord around neck	
dangercb	l	Convulsions	
dangercb	m	loss of consciousness	
dangercb	n	Preterm	
dangercb	o	Preterm premature rapture of membe	
dangercb	p	Nothing mentioned	
dangercb	k	Other (specify)	
married	1	Married	
married	2	Never married	
married	3	Widowed	

For peer review only

married	4	Divorced	
married	5	Separated	
whyathome	a	Always delivered at home	
whyathome	b	Husband/mother/mother-in-law does	
whyathome	c	Don't like health facilities	
whyathome	d	Expensive to go to health facilities	
whyathome	e	Distance	
whyathome	f	Lack of transportation	
whyathome	g	Tradition/religious reason	
whyathome	h	Other cost (not medical cost)	
whyathome	i	Facility not open	
whyathome	j	Poor quality service	
whyathome	k	No female provider	
whyathome	l	Not necessary	
whyathome	m	Not customary	
whyathome	n	Fear of COVID-19	
whyathome	o	Other	
whyatHF	a	Always delivered at a health facility	
whyatHF	b	Due to existing complications	
whyatHF	c	Referred by HDA/HEW	
whyatHF	d	Advised at pregnant women's forum	
whyatHF	e	Taken to health facility due to prolonge	
whyatHF	f	Convenient	
whyatHF	g	Affordable (free)	
whyatHF	h	Advised by HDAs	
whyatHF	i	Advised during ANC	
whyatHF	j	Other	
problems	a	Fever	
problems	b	Unable to suckle/feed	
problems	c	Difficult/fast breathing	
problems	d	Diarrhea	
problems	e	Convulsions	
problems	f	Persistent vomiting	
problems	g	Yellow palms/soles/eyes	
problems	h	Lethargy	
problems	i	Unconscious	
problems	j	Red/discharging eyes	
problems	k	Skin pustules	
problems	l	Skin around cord was red	
problems	m	Pus from cord	
problems	n	Other	
reason	a	expected him/her to get better	
reason	b	health facility too far	
reason	c	cost of treatment too expensive	
reason	d	don't trust the facility	
reason	e	family member didn't allow	
reason	f	community advisor/TBA advised agains	

For peer review only

reason	g	Fear of COVID-19	
reason	h	facility closed due to COVID-19	
reason	i	Facility not open (at that time)	
reason	j	other	
where	1	health post	
where	2	health centre	
where	3	hospital	
where	4	community health worker	
where	5	shop/pharmacy	
where	6	other	
drugs	a	malaria drugs: SP/Fansidar	
drugs	b	malaria drugs: Chloroquine	
drugs	c	Antibiotic: gentamycin	
drugs	d	Antibiotic: ampicillin	
drugs	e	Tetracycline /other eye ointment	
drugs	f	ORS	
drugs	g	Vitamin A	
drugs	h	Traditional remedy	
drugs	i	Herbs	
drugs	j	other medication	
symptoms	a	Stopped feeding well	
symptoms	b	Difficult or fast breathing	
symptoms	c	Chest in-drawing	
symptoms	d	Unusually hot or cold	
symptoms	e	Baby less active than usual	
symptoms	f	Body became yellow	
symptoms	g	Other	
symptoms	h	No symptoms	
provider	1	WDA	
provider	2	HEW	
provider	3	Nurse/midwife	
provider	4	Doctor	
provider	5	Health Officer	
provider	6	Pharmacist	
provider	7	Other	
diagnosis	1	Pneumonia	
diagnosis	2	Diarrhea	
diagnosis	3	Severe infections	
diagnosis	4	Other	
who	1	HEW	
who	2	WDA	
who	3	Other	
vac	1	Yes	
vac	2	No, but eligible	
vac	3	Not eliigible	
HADtype	1	1 to 30	

For peer review only

HADtype	2	1 to 5	
covidsxs	a	Weakness or fatigue	
covidsxs	b	Fever	
covidsxs	c	Cough	
covidsxs	d	Sore throat	
covidsxs	e	Headache	
covidsxs	f	Runny or stufy nose	
covidsxs	g	Skin rash	
covidsxs	h	Shortness of breath	
covidsxs	j	Muscle or body aches	
covidsxs	l	Diarrhea	
covidsxs	m	Loss of smell and taste	
covidsxs	n	Nausia	
covidsxs	o	Viomitting	
transm	a	Respiratory droplets	
transm	b	Objects and surfaces contaminated wit	
transm	c	Physical contact with an infected perso	
transm	d	Mosquito bites	
transm	e	Cellular mobile networks	
transm	f	Don't know	
risk	1	No risk	
risk	2	Low risk	
risk	3	High risk	
risk	4	Very high	
ways	a	Staying at home as much as you can wl	
ways	b	Socially distancing (at least 2 meters)	
ways	c	Regular washing hands	
ways	d	Sanitizing	
ways	e	Covering cough and sneeze	
ways	f	Wearing a mask	
ways	g	Sun exposure	
ways	h	Drinking alcohol	
ways	i	Lemon or ginger tea	
ways	j	Vitamin supplements	
severity	a	Everyone dies 2-4 weeks	
severity	b	Some survive, most die	
severity	c	Most survive, some die	
severity	d	Almost everyone survives	
severity	e	Do not know/refusal	
risk1	1	No, but not confirmed by a test	
risk1	2	Yes, tested and the result was positive	
risk1	3	Yes, suspected but not confirmed by a test	
risk1	4	No, tested and the result was negative	
risk1	88	Don't know	
risk2	1	No	
risk2	2	Yes, confirmed	

risk2	3	Yes, suspected but not confirmed by a test
risk2	88	Don't know
risk2	99	Refuse to answer
practice	a	Nothing
practice	b	Washing hands regularly with soap and water
practice	c	Disinfecting surfaces
practice	d	Keeping distance from sick people
practice	e	Keeping physical distance from everyone that i
practice	f	Stopped going to social gatherings, churches o
practice	g	Wearing a face mask
practice	h	Stocking up on food, home supplies and medic
practice	i	Changing/cancelling travel plans
practice	j	Clean hands with alcohol and sanitizers
practice	k	Exercised less than I usually do
practice	l	Drank more alcohol than I usually do
practice	m	l ate more unhealthy food than I usually do
practice	n	Avoided going to the doctor with issues that cc
practice	o	Asked family members or friends not to visit m
practice	p	Decided that my child could not meet with a fr
practice	q	Bought drugs that I heard that are good for tre
practice	r	Bought personal protection equipment (masks
practice	s	Refuse to answer
practice	t	Don't know
incub	1	Up to 3 days
incub	2	Up to 7 days
incub	3	Up to 14 days
incub	88	Don’t know
correct	a	There is a drug to treat the novel coronavirus
correct	b	There is a vaccine for the novel coronavirus
correct	c	There is both a drug for the treatment and a va
correct	d	There is currently no drug treatment or vaccine
correct	e	Don’t know
pinf	1	Very high
pinf	2	High
pinf	3	Low risk
pinf	4	Very low
ga	1	post term
ga	2	term(>=37 weeks)
ga	3	early (34-37 weeks)
ga	4	very early (at or belowe 32 weeks)
ga	88	Don't remeember
covidimpact	a	delay ANC visit for 3 months
covidimpact	b	delay ANC visit for at least 6 months
covidimpact	c	delay for facility visit for delivery
covidimpact	d	immediate back home after delivery
covidimpact	e	no PNC visit
covidimpact	f	declined home visit for PNC
covidimpact	g	none

filter	label:Amharic	label:Afan Oromo
	አዎ	Eeeyyee
	የለም	Lakkii
	ወንድ	dhiira
	ሴት	dhalaa
	1ኛ	1ffaa
	2ኛ	2ffaa
	3ኛ	3ffaa
	4ኛ	4ffaa
	5ኛ	5ffaa
	6ኛ	6ffaa
	7ኛ	7ffaa
	8ኛ	8ffaa
	9ኛ	9ffaa
	10ኛ	10ffaa
	11ኛ	11ffaa
	12ኛ	12ffaa
	ቴክኒክና ሙያ ሰርተፊኬት	Waraqaa Ragaa BLTO
	ዩኒቨርሲቲ/ኮሌጅ ዲፕሎማ	Yuniivarsitii/Kolleejii dip
	ዩኒቨርሲቲ/ኮሌጅ ዲግሪ ወይም ከዚያ በላይ	Yuniivarsitii/Kolleejii diig
	ኦርቶዶክስ	Oritodoksii
	ካቶሊክ	Kaatoolikii
	ፕሮቴስታንት	Pirotteestaantii
	ሙስሊም	Musiliima
	ባህላዊ እምነት	Waaqeffataa
	ሌላ	Kan biroo
	ሕፃኑ የተወለደበት ቀን ተረጋግጧል	guyyaan dhalootaa mirk
	ሕፃኑ የተወለደበት ቀን ማረጋገጥ አልተቻለም	mirkaneessu hin dandee
	አዎ	Eeeyyee
	የለም	Lakkii
	አላውቅም	Hin beeku
	አዎ	Eeeyyee
	የለም	Lakkii
	አላውቅም	Hin beeku
	Stays at MWH	Stays at MWH
	ከቧንቧ በቤት ውስጥ	Ujummoon mana keessa
	ከቧንቧ በግቢ ውስጥ	Ujummoon Qe'ee ofii ke
	ከቧንቧ ከግቢ ውጭ	Ujummoon Qe'ee ofii ala
	የተከደነ ጉድጓድ	Bishaan boollaa qadaada
	የተከለለ/የተጠበቀ ምንጭ	Burqaa misoomee keess
	የተጠበቀ የእጅ ጉድጓድ (መነቅነቂያ)-Hand pump	Haand paampii haguuga
	ያልተከደነ ጉድጓድ	Bishaan boollaa hin qada
	ያልተከለለ ምንጭ	Burqaa hin misoomne ke
	ወንዝ	Laga
	ኩሬ/ሀይቅ/ግድብ	Bishaan ciisaa/haroo/bis
	የዝናብ ውሃ	Bishaan bokkaa/roobaa

For peer review only

	ሌላ	Kan biroo yoo jiraate ibs
	አዎ፣ ግቢ ውስጥ አለ	eeeyyeen bishaan dallaa
	የለም	lakkii
	ማፍላት	Danfisuu
	ክሎሪን መጨመር (ውሀ አጋር፣አኳ ታብ፣ ቢሻን ጋሪ...)	Kiloorinii itti dabaluu/na
	በጨርቅ ማጣራት	Uccuu/Carqiin qulqullee
	የውሃ ማጣሪያ መጠቀም (የሸክላ ስረት፣ አሸዋ፣ ለዚሁ ተግባር የተ	Calaltuu ittin qulqulleess
	በፀሐይ ብርሃን ማጣራት	Aduudhan calaluu
	በማቆየት ቆሻሻው እንዲዘቅጥ በማድረግ	Tursuudhaan akka calalu
	ሌላ	Kan biroo
	አላውቅም	Hin beeku
	የጉድጓድ መጸዳጃ ቤት (የተለመደ)	Mana fincaanii boollaa
	አየር የሚያስገባ የተሻሻለ የጉድጓድ መጸዳጃ ቤት	Mana Fincaanii kan ujum
	ዘመናዊ በውሃ የሚሠራ መጸዳጃ ቤት	Kan Hamayyaa, bishaani
	መጸዳጃ ቤት የላቸውም/ጫካ/ሜዳ ላይ	Mana Finacaanii hin qab
	ሌላ	Kan biroo
	ሣር/ቅጠል	citaa/Baala
	ፕላስቲክ	Lastikii
	ቀርከሃ/ሸምበቆ	Leemmana/Shambaqqo
	ያልተላገ እንጨት	Mukkaa hin soqamne/hi
	ቆርቆሮ ተሠርቶ ያለቀ	Qorqoorrodhaan hojjeta
	የተላገ እንጨት	Mukkaa soqqameen/ bo
	ክላሚን/ሲሚንት ፋይበር	Kaalaamayyin/Cement F
	ሲሚንቶ	Siimmiintoo
	ለጣሪያ የተቆራረጠ ሳንቃ	Saanqaa baaxidhaaf mu
	ሌላ	Kan birooo
	ግድግዳ የለውም	Girgiddaa/gidaaraa hin
	አገዳ/ግንድ/ቀርከሃ/ሸምበቆ	Huusa/hagadaa/jirma m
	እንጨትና ጭቃ	Muka fi dhoqqee
	ድንጋይና ጭቃ	Dhagaa fi dhoqqee/sup
	ክፍት ከሆነ ጡብ የተሠራ	Xuubii u'uraa/ qaawwa
	ንብርብር ጣውላ	Xaawulaa wal'irra naqan
	ካርቶን	Kaartoonii
	ሲሚንቶ	Simmiintoo
	ድንጋይና ኖራ	Dhagaa fi noora irraa
	ጡብ	Xuubii irraa
	ድፍን የሲሚንቶ ብሎኬት	Bilookkeettii simmiintoo
	ድፍን ከሆነ ጡብ የተሠራ	Xubii duudaa irraa
	ያልተላገ እንጨት/ሣንቃ	Muka/Saanqaa hin boca
	ሌላ	Kan biroo
	ተጠቅሷል	Ibsameera
	አልተጠቀሰም	Hin ibsamne
	ደስተኛ አልነበሩም	Itti hin gammannen ture
	ደስተኛ ነበሩ	Itti gammadeera/quufer
	በጣም ደስተኛ ነበሩ	Bayyee itti gamadeera/q
	ትልቅ ችግር	Rakkoo guddaa

For peer review only

	ትንሽ ችግር	Rakkoo salphaa
	ችግር አይደለም	Rakkoo hin qabu
	አዎ፣ የቤተሰብ ጤና መምሪያውን ማየት ተችሏል	Eeyyee Qajeelfama fayy
	አዎ፣ የቤተሰብ ጤና መምሪያውን ማየት አልተቻለም	Eeyyee qajeelfama fayy
	የለም	Lakkii
	አዎ፣ ተመርቀዋል (የምስክር ወረቀት ታይቷል)	Eeyyee eebbiffameera w
	አዎ፣ ተመርቀዋል (የምስክር ወረቀት አልታየም)	Eeyyee eebbiffameera g
	የለም ታቅፈው እየሠሩ ናቸው	Lakkii, Hamatanii hojjech
	አልተመረቁም/አልታዩም/አልታቀፉም	Lakki/miti/gonkumaa
	በቋሚነት የተነከረ	Yaroo oomishamu kan cu
	በየጊዜው የሚነከር	Kan dura cuuphame/yer
	ያልተነከረ	Kan hin cuuphamne
	አያውቁም	Hin beeku
	ቤተሰብ እቅድ	a. Karoora maatii
	የሕፃናት ክትባት	b. Talaallii daa'immanii
	የቅድመ ወሊድ ክትትል	c. Hordoffii da'umsa dur
	ድህረ ወሊድ ክትትል	d. Hordoffii da'umsa boo
	የጤና ትምህርት	e. Barnoota fayyaa
	የእድገት ክትትል	f. Hordoffii guddina daa'
	የታመመ ሕፃን ቅብብሎሽ (Referral)	g. Daa'ima dhukkubsate
	የተቅማጥ ህክምና	h. Yaalii garaa kasaa
	የወባ በሽታ ህክምና	i. Yaalii dhukkuba busaa
	የሳምባ ምች ህክምና	j. Yaalii dhukkuba daran
	አጎበር ለመቀበል	k. Saaphana siree hiruu/
	በወሊድ ጊዜ የሚደረግ እንክብካቤ	l. Tajaajila/ gargaarsa Da
	አዲስ ለተወለደ ሕፃን የሚደረግ እንክብካቤ	m. Tajaajila/gargaarsa D
	የአከባቢና የግል ንፅህና አገልግሎት	n. Qulqullina nannoo fi k
	ሌላ	o. Kan biroo
	የጤና ኤክስቴንሽን ሠራተኛ	Hojjettoota ekisteenshin
	የጤና ልማት ቡድን (1:5 ወይም 1:30)	Raayyaa misooma fayya
	የልምድ አዋላጅ	Deessistuu aadaa
	የጤና ባለሙያ	Hojjettoota fayyaa
	የቀበሌ አስተዳደሮች	Bulchitoota gandaa
	አላውቅም	Hin beeku
	ሌላ	Kan biroo
	የበለጠ	Kan caalu
	እንደወትሮው	Akka baramee
	ያነሰ	Kan barameen gadi
	አላስታውስም	Hin Yaadadhuu
	እናት በመታመሟ/አቅም በማጣቷ	Haati waan dhukkubsatt
	ሕፃኑ በመታመሙ/አቅም በማጣቱ	Daa'imni wan dhukkubs
	የጡት ጫፍ ችግር	Rakkoo fixee harmaa
	በቂ ወተት አለመኖር	Aannan gahaan dhabam
	እናት ሰራተኛ በመሆኗ	Haati Waan Hojjeetuu ta
	ህፃኑ ጡት እምቢ በማለቱ	daa'imni wan harma did
	ህፃኑ ጡት ማቆሚያ ዕድሜ ላይ በመድረሱ	Daa'ima yeroo itti harma

For peer review only

	እናት በመጸነሷ	Haati waan ulfoofteef
	እናት የወሊድ መከላከያ መውሰድ በመጀመሯ	Haati waan mala karoor
	ሌላ	kan biroo ibsi
	ከ4 ወር በፊት	ji'a 4 dura
	ከ4 - 6 ወራት መካከል	ji'a4-6 giddutti
	ልክ 6 ወር ሲሞላው	akka ji'a 6 guteen
	ከ 6 ወር በኋላ	Ji'a 6 booda
	ተጨማሪ ምግብ አልጀመረም	Nyaata dabalaataa hin j
	አላስታውስም	Hin Yaadadhuu
	የእናት ጡት ወተት	Aannan harma haadhaa
	ንፁህ ውሀ	Bishaan qulqulluu
	የዱቄት ወተት	Daakuu aannaanii
	ሌላ ወተት / የላም ወተት/	Aannaan bira/annaan sa
	የፍራፍሬ ጭማቂ	Cuunfaa fuduraalee
	ሌላ ፈሳሽ /የስኳር ውሀ፣ ቡና ፣ ለስላሳ መጠጦች፣ ሾርባ/	dhaangala'aa biroo/ kan
	ማንኛውንም ከጥራጥሬ የተሠራ ምግብ /ገብስ፣ ማሽላ፣ በቆሎ፣ሩ	Nyaataa gosota midhaar
	ማንኛውንም ምግብ እንደ ዱባ፣ ካሮት፣ ቀይ ስኳር ድንች፣ አረንጓ	Nyaataa kamiiyyuu kan
	ማንኛውም ከስራስር ወይም ከግንድ የተሰራ ምግብ /ካሳቫ፣ እንሰት	Nyaata kan hudnee fi jir
	ማንኛውም ፍራፍሬና ቅጠላቅጠል /ሙዝ፣ ቲማቲም ፣ አቮካዶ ፣	Nyaata kamiyyuu kan m
	ስጋ	Foon
	ማንኛውም የሚፈለፈል ፍሬ ካላቸው የተሠራ ምግብ (አተር፣ ባቄ	Nyaata gosotaa midhaar
	ማንኛውም ምግብ ከዘይት፣ ከቅቤ ከቅባት የተሰራ	Nyaata gosota kan akka
	ተቀቅሎ የተፈጨ ምግብ	Nyaata affeelamee daak
	እንቁላል	Hanqaaquu/Buuphaa/Ki
	ዓሣ	Qurxummii
	አይብ እርጎ	Baaduu/Itittuu
	ሌላ (ይጠቀስ)	Kan biroo ibsi
	ከወትሮው በጣም ያነሰ	Kan durii irra bayyee xiq
	ከወትሮው ያነሰ	Kan durii irra xiqqaa
	ተመሳሳይ	Akkuma durii
	የበለጠ	Kan caalu
	ምንም አልጠጣም	Gonkuma hin dhugne
	አላስታውስም	Hin Yaadadhuu
	ፈሳሽ አልጀመረም	Dhangala'aa hin jalqabn
	ከወትሮው በጣም ያነሰ	Kan durii irra bayyee xiq
	ከወትሮው ያነሰ	Kan durii irra xiqqaa
	እንደወትሮው	akkuma durii
	ከወትሮ የበለጠ	Kan durii kan caalu
	ምንም አልበላም	Gonkuma hin nyaanne
	ምግብ አልጀመረም	Nyaata hin jalqabne
	አያውቁም	Hin beekani
	የመንግስት ሆስፒታል	Hoospitaala mootumma
	የመንግስት ጤና ጣቢያ	Buufata fayyaa mootum
	የመንግስት ክሊኒክ	Kiliniika mootummaa
	የመንግስት ጤና ኬላ	kellaaa Fayyaa mootum
	የመንግስትየመንደር ጤና አገልግሎት /ውሎገብ/	Tajaajila fayyaa mootum

For peer review only

	መንግሥታዊ ያልሆኑ ጤና ድርጅት/አገልግሎት	Dhaabbata fayyaa miti m
	መንግሥታዊ ያልሆኑ የመንደር ጤና አገልግሎት /ውሎገብ/	Tajaajila fayyaa miti-mo
	የግል ሆስፒታል	Hoospitaala dhunfaa/ha
	የግል/ማህበረሰብ ሀኪም/ክሊኒክ	Kiliniika dhuunfaa/hawa
	የግል ፋርማሲ	Faarmaasii dhunfaa/haw
	ፀበል	Xabalaa
	ሱቅ	Suuqii
	ጓደኛ/ዘመድ	Hiryaa/Fira
	ባህላዊ ሀኪም	Hakimii aadaa
	ሌላ	Kan ibsi
	አንቲባዮቲክ/ፀረ ባክቴሪያ ክኒን /ሽሮፕ	Antibayotikis/kininaa far
	ሠገራ የሚያወፍር ክኒን /ሽሮፕ	Kininii/shirooppii udaan,
	ዚንክ ክኒን /ሽሮፕ	Kininii/Shirooppii zinkii
	ሌላ (ፀረ ባክቴሪያ፣ አንቲባዮቲክ) ያልሆነክኒን /ሽሮፕ	kininii/Shirooppii kan bir
	የማይታወቅ ክኒን ወይም ሽሮፕ	Kininii/Shirooppii hin be
	አንቲባዮቲክ/ፀረ ባክቴሪያ በመርፌ የሚሰጥ	Antibayotikis/farra bacte
	አንቲባዮቲክ ያልሆነ በመርፌ የሚሰጥ	Antibayotikis/farra bacte
	የማይታወቅ መርፌ	Lilmoo hin beekamnee
	በደም ስር የሚሰጥ መድሃኒት	Qorcha hiddaa dhiigaa ja
	ቤት የተዘጋጀ /የባህል መድሃኒት	Qorha aadaa/ kan mana
	ሌላ ምንም አልተሰጠዉም	Kan bira wan tokkoole h
	ሌላ ይገለጽ	Kan biroo ibsi
	ደረት ብቻ	Laphee qofa
	አፍንጫ ብቻ	Funyaan qofa
	ሁለቱም	lamaanuu
	ሌላ/አያውቁም	kan biroo ibsi /hin beeka
	ክሎሮኪን	Kilorokiinii
	ኳርተም	Kowaarteemii
	ኩይኒን	Kuyininii
	ባክትሪየም	Bakteeriyeem
	አምፕስሊን	Ampisiliinii
	አሞክሳሲሊን	Amoksasiliinii
	ክሎራንፌኒኮል	Kilorafeeniikoolii
	ቴትራሳይክሊን	Teetraasaykiliinii
	ሌላ አንቲባዮቲክ	Antiibayootikiis biroo
	አስፕሪን	Aspriinii
	ኢቢዮፕሮፌን	Ibupronii
	ፓራሲታሞል	Parasitamoolii
	ሌላ	Kan biroo ibsi
	ማስታወክ	Balaqqamsiisuu/hooqqis
	ትኩሳት	Gubaa qaama
	ጡት መጥባት አለመቻል	Harma hoodhuu dadhab
	ለመተንፈስ መቸገር/ቶሎ ቶሎ መተንፈስ	Yoo rakkoo afuura bafan
	መቀዝቀዝ	Yoo qamnii isa baayyee
	ክብደቱ በጣም ትንሽ/ያለጊዜው ከተወለደ	Yoo ulfaatinni Daa'ima b
	እትብት መድማት፣ መቅላት፣ መግል መያዝ	Yoo handhuurri diimate/

For peer review only

	የቀሉ /መግል የሚፈሳቸው ዐይኖች	Yoo iji diimate/ yoo mala
	የውስጥ እግር ወይም የመዳፍ/ የዐይን ቢጫ መሆን	yoo barruun/faanaa miil
	ንቁ አለመሆን/ሲነኩትም አለመንቀሳቀስ፣ ራስን መሳት	Yoo xuqaan qofa kan soc
	መንዘፍዘፍ/መንቀጥቀጥ	Kan hurgufu/hollatu yoo
	ሕፃኑ ያለምንም ምክንያት አምርሮ የሚያለቅስ ከሆነ	Daa'imni sababa male yo
	ምንም አልተጠቀሰም	Waan tokkollee hin ibsa
	ሌላ	Kan biroo ibsi
	አላውቅም	Hin beeku
	የተደጋገመ ቀጭን ተቅማጥ	kan irra deddeebi'ee gar
	ማንኛውም ቀጭን ተቅማጥ	Garaa kaasaa qal'a kami
	ተደጋጋሚ ማስመለስ	Irra deddeebi'ee balaqq
	ማንኛውም ማስመለስ	Balaqqamsiisuu kamiyyu
	ደም የቀላቀለ ተቅማጥ	Garaa kaasaa dhiigaan m
	ቶሎ ቶሎ መተንፈስ	Dafeedafee afuura baafa
	የመተንፈስ ችግር	Rakkoo afuura baafanna
	ድምጽ ያለው አተነፋፈስ	Afuura baafannaa sagale
	ትኩሳት (High)	Gubaa qaamaa
	ኮንቬልሽን	Hurgufuu
	ያንገት መግረር/መገተር	Yoo boquu gogse
	ተደጋጋሚ ውሃ መጠማት	Yoo irra deddeebi'ee bis
	መጠጣት አለመቻል	Yoo dhuguu dadhabee
	መብላት/መጠጣት አለመቻል	Yoo nyaachuu/dhuguu d
	ሕመሙ ከባሰ/በጣም ሲታመም	Yoo dhukkubbin itti caal
	ካልተሻለው	Yoo itti fooyya'uu didee
	ለረጅም ጊዜ ከታመመ	Yeroo dheeraaf yoo dhu
	ዓይኖች ከጎደጎዱ	Yoo ijii isa boolla'ee
	ሳል	Qufaa
	ሌላ	Kan biroo
	አያውቁም	Hin beekani
	የመንግስት ሆስፒታል	Hospitaala mootummaa
	የመንግስት ጤና ጣቢያ	Buufata Fayyaa mootum
	የመንግስት ጤና ኬላ	kellaa Fayyaa mootumm
	መንግስታዊ ያልሆነ የጤና ድርጅት	Dhaabbataa Fayyaa miti
	የግል ጤና ድርጅት	Dhaabbata fayyaa dhuu
	ሌላ	Kan biroo (ibsi)
	አያውቁም	Hin beekani
	ሐኪም	Haakimii
	ነርስ/አዋላጅ ነርስ	Nersii/Nersii deessistuu
	የጤና ኤክስቴንሽን ሠራተኛ	HEF
	የጤና ባለሙያ/ሙያው የማይታወቅ	Hojjetaa fayyaa/Ogumm
	ሌላ /ይገለጽ/	Kan biroo (ibsi)
	ከወትሮው ያነሰ	Kan duriirra kan xiqqate
	እንደወትሮው	Akkuma durii
	ከወትሮው የበለጠ	Kan duriirra kan caalu
	አላውቅም	Hin beeku
	የገንዘብ	Qarshii

For peer review only

	የመጓጓዣ	Geejiba
	የምግብ	Nyaata
	የሚያዋልድ ሰው ማዘጋጀት	Nama deessisuu qophee
	የምወልድበት የጤና ድርጅት መለየት	Dhaabbata fayyaa bakka
	ንፁህና አስፈላጊ የማዋለጃ ቁሳቁሶችን ማዘጋጀት	Quliqullinaa fi meeshaal
	ደም ለጋሾችን ማዘጋጀት	Namoota dhiiga naa kee
	ልጆችን የሚንከባከብ ሰዉ ማዘጋጀት	Nama daa'ima kunuunsu
	ሌላ	kan biroo (ibsi)
	ቤት ውስጥ በራሳቸው ቤት	Mana isaan keessatti
	ሌላ ሰው ቤት	Mana namaa keessatti
	የመንግሥት ሆስፒታል	Hospitaala mootummaa
	የመንግሥት ጤና ጣቢያ	Buufata Fayyaa Mootum
	ጤና ኬላ	Keellaa Fayyaa
	መንግሥታዊ ያልሆነ የጤና ተቋም	Dhaabbata Fayyaa Miti-n
	የግል ሆስፒታል	Hospitaala dhuunfaa
	የግል ሃኪም/ክሊኒክ	Kiliinikaa ykn mana haak
	ሌላ	Kan biroo
	ጋሪ	Gaarii
	እንስሳት (ፈረስ፣ በቅሎ)	Beeyilada (Fardaa gange
	ቃሬዛ (ባህላዊ አምቡላነስ)	Wasaasaa
	አምቡላንስ	Ambulaansii
	የህዝብ መጓጓዣ (Public transport)	Geejiba ummataa
	የመንግስት መስሪያ ቤት መኪና	Konkolaataa mootumma
	በእግር	Milaan
	ዘመዶቼ ተሸክመዉኝ	Firaan batameen
	ሌላ	Kan biroo
	ዶክተር	Hakiimii/doktorii
	ነርስ/አዋለጅ ነርስ	Narsii/Narsii deessistuu
	የጤና ኤክስቴንሽን ሰራተኛ	HEF
	የልማት ቡድን መሪ	DRMF
	የልምድ አዋላጅ	Deessistuu aadaa
	ዘመድ/ጓደኛ	Fira/Hiriyaa
	ማንም የለም	Eenyu iyyuu hin jiru
	ሌላ ይጠቀስ	Kan biroo ibsi
	አፍስሰውታል	Dhangalasaniiruu
	ለሕጻኑ አጥብተውታል	Daa'ima hoosisaniiruu
	ሌላ/አያስታውሱም	Kan biroo/Hin yaadadhu
	ትልቅ	Guddaa
	መካከለኛ	Giddu galeessa
	ትንሽ	Xiqqaa
	ያለ ቀኑ የተወለደ	Kan yeroo malee dhalate
	አያውቁም	Hin beekani
	አዲስ ምላጭ	Milaacii haaraa
	የተቀቀለ ምላጭ	Milaacii Affeelamee
	ያልተቀቀለ አሮጌ ምላጭ	Milaacii dulloomaa hin a
	ቢላዋ	Albee ykn Haaduu

For peer review only

	መቀስ	Maqasii
	አያውቁም	Hin beekani
	ሌላ	Kan biroo
	አዲስ ማሰሪያ/ክር	Kirrii haaraa handhuura
	የተቀቀለ ማሰሪያ/ክር	Kirrii Affeelamee
	አሮጌ ማሰሪያ/ክር	Kirrii dulloomaa
	እትብቱ አልታሰረም	Handhuurri hin hidhamn
	አያውቁም	Hin beekani
	ሌላ	Kan biroo (ibsi)
	ቅቤ	Dhadhaa
	ዘይት	Zaayitii
	አመድ	Daaraa
	ቅባት/ዱቄት	Dibata/daakuu
	እበት	Dhoqqee hoorii
	ቀዝቃዛ ውሃ	Bishaan qorraa
	ምስራች ጀል (Chlorhexidine)	Chlorhexidine
	ሌላ	Kan biroo
	ብቻውን በመሬት ላይ	Qofaa isaa / lafa irra
	በእናቱ ደረት/ሆድ ላይ	Laphee/garaa haadhaa
	ከእናቱ አጠገብ	Haadha waliin
	ከሌላ ሰው ጋር	Nama biraa waliin
	ብቻውን ለህጻን በተዘጋጀ አልጋ/ቦታ ላይ	Qofaa siree daa'imaaf qo
	አያውቁም	Hin beekani
	ሌላ	Kan biroo
	ሰውነት ማሸት	Qaama isa sukkumuu
	ማድረቅ	Gogsuu
	አፉን መጥረግ	Afaan isaa haxaahuu/qo
	አፍ ለ አፍ አየር መስጠት	Qilleensa afaaniin afaani
	አምቡባግ ተጠቅመዋል (አፍ ላይ የሚደረግ ፕላስቲክ)	Ambubaagii fayyadamu
	ምንም አልተደረገም	Homayyuu hin goone
	አፍና አፍንጫን በመሳሪያ መጥረግ (መምጠጥ)/ሰክሽን/	Afaanii fi funyaan meesh
	አያውቁም	Hin beekani
	ሌላ	Kan biroo
	የጤና ባለሙያ	Ogeessa fayyaa
	የጤና ኤክስቴንሽን ሠራተኛ	HEF
	የጤና ልማት ቡድን (1:5 ወይም 1:30)	Rayyaa Misooma Fayyaa
	የልምድ አዋላጅ	Deesistuu aadaa
	ቤተሰብ/ዘመድ/ጓደኛ	Fira/Hiryaa/ollaa
	እራስ	Ofiin
	ሌላ ይገለጽ	Kan biro (ibsi)
	የላም ወተት/ከእናት ጡት ሌላ	Aannan sa'aa/harma haa
	ንጹሕ ውሃ	Bishaan Qulqulluu
	የስኳር /የግሉኮስ ውሃ	Bishaan shukkaaraa/gulu
	የፍራፍሬ ጭማቂ	Cuunfaa muduraalee
	የሕጻናት ወተት/ፎርሙላ	Aannan daa'immani/wa
	ሻይ/ሐሜሳ/	Shaayii/hameessaa

For peer review only

	ሌጋ ቅቤ	Dhadhaa ho'aa
	ሌላ	Kan biroo
	ማድረቅ	Gogsuu/qoorsuu
	በንጹሕ ጨርቅ መጠቅለል	Kafanaa/uccuu qulqullu
	ከእናቱ ጋር ማስተኛት	Haadha waliin cibsuu
	ከእናቱ ሰውነት ጋር እንዲነካካ ማድረግ	Qaama haadha waliin ak
	በሞቀ ውሃ ማጠብ	Bishaan ho'aan dhiquu
	የማዋለጃ ክፍሉን ማሞቅ	Kutaa da'umsaa ho'isuu
	ሌላ	Kan biroo
	ሁል ጊዜ	Yeroo hunda
	አብዛኛውን ጊዜ	Yeroo baayyee
	አንድ አንድ ጊዜ	Yeroo tokkoo tokko
	በፍፁም	Gonkumaa
	አያስታውሱም	Hin yaadatan
	ከእናቱ ጋር (ሳይነካካ)	Haadha waliin (osoo wal
	አልጋ ላይ ብቻውን	Sireerra qofa isaa
	ከሌላ ሰው ጋር	Nama biroo waliin
	ገላ ከገላ ጋር እንዲነካካ ተደርጎ (ከእናቱ ጋር)	Qaamnisaa akka qaama
	ሌላ	Kan biroo
	ሆስፒታል	Hospitaala
	ጤና ጣቢያ	Buufata Fayyaa
	ጤና ኬላ	Keellaa Fayyaa
	መድሃኒት ቤት	Mana qorichaa
	የማህበረሰብ ጤና ሠራተኛ	Raayyaa Misooma fayya
	የልምድ አዋላጅ/የሰለጠነ	Deessistuu aadaa/ kan le
	ፀበል	Xabalaa
	የባህል መድሀኒት አዋቂ	Beektota qoricha aadaa
	ሌላ	Kan biroo
	ከፍተኛ የሆነ ራስ ምታት	Mata bowwoo cimaa
	ብዥ ያለ እይታ	Ija dura urrii maruu (lafti
	የሕፃኑ እንቅስቃሴ መቀነስ	Daa'imni sochiin hir'isuu
	ራስን መሳት	Of wallaaluu
	መንዘፍዘፍ/መንቀጥቀጥ	Hurguufuu/Hoollachiisu
	ከማህጸን ደም መፍሰስ	Dhiigni heedduun (garm
	በታችኛው የሆድ ክፍል ላይ የሚሰማ ከባድ ህመም	Garaa ciniinna cimaa
	ትኩሳት	Gubaa Qaama
	የእጆችና የፊት ማበጥ	Dhiita'uu fuulaa fi harka
	ምንም ምልክት አልተጠቀሰም	Mallattooleen homtuu h
	መኖሪያ ቤት ውስጥ	Mana ofii keessatti
	ሌላ ቤት ውስጥ	Mana biroo keessatti
	ጤና ኬላ	Keellaa Fayyaa
	ጤና ጣቢያ	Buufata Fayyaa
	ሆስፒታል	Hoospitaala
	ሌላ (ይጥቀሱ)	Kan biroo (ibsi)
	አዎ	Eeyyee
	የለም	Lakkii

For peer review only

	ሕጻኑ ከ6 ወር በታች ነው	Daa'imni ji'a 6 gadidha
	አያውቁም	Hin beekani
	በእክብል መልክ የተዘጋጀ ቫይታሚን	Vitaamina haala kininiin
	ንፁህ ውሃ	Bishaan qulqulluu
	የስኳር ውሃ የፍራፍሬ ጭማቂ	Bishaan shukkaaraa/cuu
	ኦ.አር.ኤስ	"ORS'/dhangala'aa lubb
	የህፃናት ወተት/ኢንፋንት ፎርሙላ	Aannan da'immanii kan
	የእንስሳት ወተት	Aannan hoorii
	ስኳር/ግልኮስ/ጨው ውሃ የተበጠበጠ	Shukkaara/Gulukoosii/b
	ሻይ/በመዘፍዘፍ የተዘጋጀ ፈሳሽ	Shaayii/Dhangala'aarra
	ቅቤ	Dhadhaa
	ሌሎች ፈሳሾች	Dhangala'aawwan biroo
	አላውቅም	Hin beeku
	ከወትሮው በጣም ያነሰ	Kan durii irra baayyee ga
	ከወትሮው ያነሰ	Kan durii gadi
	እንደወትሮው	Akkuma durii
	ከወትሮ የበለጠ	Kan durii irra ni caala
	ምንም አልጠባም	Waan tokkooyyu hin hoo
	አያውቁም	Hin beekani
	በጣም እስማማለሁ	Baayyeen itti walii gala
	እስማማለሁ	Hanga tokko ittin walii g
	አልስማማም	Hanga tokko itti walii hi
	በጣም አልስማማም	Baayyee itti walii hin gal
	የ 1 ለ 5 መሪ	Dursituu tokko-shanee (
	የ 1 ለ 30 መሪ	Dursituu Garee(1:30)
	አዎ፣ ግድግዳ ላይ ተለጥፏል	Eeyyee, keenyan/gidiraa
	አዎ፣ ግድግዳ ላይ አልተለጠፈም	Eeyyee, girgiddaa/gidira
	የለም	Lakki
	አዎ	Eeyyee,
	የለም	Lakki
	አይመለከታትም	Hin ilaallatuu
	ዶክተር	Doktora/haakima
	ነርስ/አዋለጅ ነርስ	Narsii/Narsii deessistuu
	የጤና ኤክስቴንሽን ሰራተኛ	HEF
	ጤና ባለሞያ (ሙያዉ የማይታወቅ)	Ogeessa fayyaa (Ogumm
	የልማት ቡድን መሪ	DRMF
	የልምድ አዋላጅ	Deessistuu aadaa
	ዘመድ/ጓደኛ	Fira/Hiriyyaa
	ማንም	Eenyuyyuu
	ሌላ	Kan biroo
	በጣም ተደስቻለሁ	Baayyee itti gammadeer
	ተደስቻለሁ	Hanga tokko itti gamma
	አልተደሰትኩም	Hanga tokko itti hin gam
	ምንም አልተደሰትኩም	Gonkumaa itti hin gamm
	አዎ	Eeyyee
	የለም	Lakkii

For peer review only

	አላውቅም	Hin beeku
	በራሴ ቤት	Mana koo keessatti
	ጤና ኬላ	Keellaa Fayyaa
	ጤና ጣቢያ	Buufata Fayyaa
	ሆስፒታል	Hospitaala
	ሌላ	Kan biroo
	ዶክተር	Doktora/haakima
	ነርስ/አዋላጅ ነርስ	Narsii/Narsii deessistuu
	የጤና ኤክስቴንሽን ባለሙያ	HEF
	ጤና መኮንን	Qondaala fayyaa
	የልማት ቡድን መሪ	DRMF
	የልምድ አዋላጅ	Deesistuu aadaa
	አላውቅም	Hin beeku
	ሌላ	Kan biroo
	ዶክተር	Doktora/haakima
	ነርስ/አዋላጅ ነርስ	Narsii/Narsii deessistuu
	የጤና ኤክስቴንሽን ባለሙያ	HEF
	ጤና መኮንን	Qondaala fayyaa
	የልምድ አዋላጅ	Deesistuu aadaa
	አላውቅም	Hin beeku
	ሌላ	Kan biroo
	ጡቶቼን ተመርምረዋል	Harmi koo sakatta'amee
	ስለጡት ማጥባት ምክር	Harma hoosisuu irratti g
	ስለ አደገኛ ምልክቶች ትምህረት	Mallattoolee balaaa cim
	የቤተሰብ እቅድ ትምህረት	Karoora maatiirratti bar
	ስለ ሥራዓተ-ምግብ መረጃ	Odeeffannoo waa'ee sir
	ወደ ጤና ድርጅት ሪፈር	Gara dhaabbata fayyaat
	ደም ግፊቴ ተለክቷል	Dhiibbaan dhiigaa safara
	ከመውለድ ጋር የተያያዙ ቁስሎችን ማከምና መዳናቸውን ማረጋገ	Da'umsaan wal qabatee
	የቤተሰብ እቅድ ምክርና አገልግሎት	Gorsa fi tajaajila mala ka
	የሰውነት ሙቀት መለካት (Assessed temperature)	Ho'ina qaamaa safaruu
	አተነፋፈስ መለካት (Assessed breathing)	Haala afuura baafannaa
	ሌላ	Kan biroo
	አጠቃላይ ምርመራ/የህጻኑን ገላ ማየት	Sakatta'iinsi waliigalaa d
	ህጻኑን መመዘን	Ulfaatina daa'imaa safar
	እትብቱን መመርምር	Handhuura ilaaluu
	ስለ ጡት ማጥባት ምክር/ጡት ብቻ ስለማጥባት	Gorsa waa'ee harma hoo
	ጡት ሳጠባ ተመልክተውኛል	Yeroon harma hoosisu n
	ገላ ለ ገላ ማቀፍ/ሙቀቱን መጠበቅ ምክር	Gorsa barbaachisummaa
	ህጻኑን ለአደገኛ ምልክቶች ምርመራ (መመረዝን ጨምሮ)	Mallattoolee balaaa cim
	ስለ አደገኛ ምልክቶች ትምህርት	Waa'ee mallattoolee bal
	ወደ ጤና ድርጅት ሪፈር	Dhaabbata fayaatti ol-er
	ህጻንዋን ከማቀፍ በፊት እጅን ስለመታጠብ ምክር	Gorsa waa'ee osoo daa'i
	እትብቱን በንጽህና መያዝ ምክር	Gorsa waa'ee qulqullina
	ህጻኑን/ኗን ከ24 ሰአት በፊት እነዳይታጠብ/እንዳትታጠብ ምክር	Gorsa waa'ee daa'imni d
	የህጻናት ክትባት	Talaallii da'immanii

For peer review only

	ሌላ	Kan biroo
	ጡቱ በቂ ወተት የለውም	Harmi koo aannan gahaa
	ቀኑን ሙሉ ከህጻኑ/ኗ ጋር አብሬ ስለማልውል	Guyyaa guutuu daa'ima
	ቤተሰቤ ወይም ጓደኞቼ መከረውኝ	Hiriyyoonni ykn firoonni
	ባህል ወይማ ልማድ	Barmaatilee ykn barsiifa
	ሌላ	Kan biroo
	በዋናነት ማገዶ (ገዝተን/ለቅመን	Irra caalaa qoraan (bitne
	ኩበት	Kaboota
	የሰብል ተረፈ ምርት(አገዳ...)	Galabaa midhaanii(hosse
	ከሰል	Cilee/Kasala
	ነጭ ጋዝ	Gaazii adii
	ቡቴን ጋዝ	Gasii buteeni
	ኤሌክትሪክ	Elektirikii
	ባዮ ጋዝ	Baahoo gaazii
	ማገዶ አይጠቀሙም/ምግብ አያበስሉም	Boba'aa hin fayyadaman
	ከማህጸን ብዙ ደም መፍሰስ	Dhiiga baayyaa gadamee
	መጥፎ ሽታ ያለው ፈሳሽ ከብልት መፍሰስ	Dhangala'aa foolii qabu
	ከፍተኛ ትኩሳት	Gubaa qaamaa cimaa
	በታችኛው የሆድ ክፍል የሚሰማ ከባድ ህመም	Garaa ciniinna cimaa
	መንዘፍዘፍ	Hollachuu/hurgufuu
	ራስን መሳት	Of-wallaaluu
	ከባድ ራስ ምታት	Bowwoo mata Cimaa
	ብዥ ያለ እይታ	Ija dura maruu
	በባት ላይ የሚሰማ ከፍተኛ ህመም (እብጠት ኖረውም አልኖረው	Dhuukuba sarbaa gubba
	ራሷን ወይም ልጇን ልትጎዳ እንደምትችል የሚያመለክት ንግግር/(	Erga deesee boodaa qaa
	ምንም አልተጠቀሰም	waan tokkoolee hin ibsa
	ሌላ	kan biroo
	ከፍተኛ ደም ከማህጸን ሲፈስ	Dhiigin heeduun yoo gad
	ሽታ ያለው ፈሳሽ ከማህጸን ከወጣ	Dhangala'aan foolii qabu
	ከፍተኛ ትኩሳት	Gubaa qama cimaa
	የሚወለደው ሕጻን እጅ ቀድሞ ከታየ	Yoo harki daa'ima dhalat
	ሕጻኑ በማህጸን ውስጥ ትክክለኛ ያልሆነ አቀማመጥ ሲኖረው	Daa'imni gadameessa ke
	ረጅም የምጥ ጊዜ/ከ12 ሠዓት በላይ	Ciniinsuu yoo irra ture/ s
	የእንግዴ ልጅ ሲዘገይ	Yoo obbaatiin turee
	የእንሽርት ውሃ ያለጊዜው ሲፈስ	Yoo bishaan gubbee yere
	እትብቱ ቀድሞ ከታየ	yoo handhuurri dursee c
	እትብቱ ከሕጻኑ አንገት ላይ ሲተበሰብ	yoo handhuurri daa'ima
	እናትየዋ ኮንቨልዥን ሲኖራት	Yoo haati gagabdoo qab
	እናትየዋ እራስዋን ስትስት	Yoo haati of wallaaltu
	ያለ ቀኑ ሲወለድ	Yoo osoo hin ga'iin dhala
	ከ9 ወር በፊት/የመውለጃ ጊዜሽ ሳይደርስ የእንሽርት ውሃ ያለጊዜ	Ji'a 9 dura/ guyyaan da'u
	ምንም አልተጠቀሰም	waan tokkoolee maqaa l
	ሌላ	kan biroo
	አዎ ባለትዳር ነኝ	Married
	ያላገባ	Never married
	የሞተባት	Kan abbaan mana irra du

For peer review only

	የተፋታች	Kan abbaa mana hiiktee
	የተለያየች	Kan abbaa manaa waliin
	ሁሌም እቤት ነው የምወልደው	Yeroo hunda manumatti
	ባለቤቲ/እናቴ/አማቴ ስለማይፈቅዱ	Abbaan warraa /Haati/ A
	የጤና ተቋም መሄድ ደስ ስለማይለኝ	Dhaabbilee fayyaa deem
	የጤና ተቋም ውድ ስለሆነ (expensive service charge)	Gatiin tajaajila dhaabbile
	የጤና ተቋሙ ሩቅ መሆን	Dhaabbani fayyaa waan
	የትራንስፖርት ችግር	Rakkoo geejibaa
	ባህላዊ/ሐይማኖታዊ ምክንያት	Sababiiwwan barmaatile
	ሌሎች ተያያዥ ወጭዎች	Baaswwan waan addaa a
	የጤና ተቋሙ ዝግ ስለነበረ	Dhaabbani fayyaa waan
	የጤና ተቋሙ አገልግሎት ጥራት አናሳ	Qulqulliini dhaabbata fa
	ሴት የጤና ባለሙያ አለመኖር	Ogeesii fayyaa saalaan d
	አስፈላጊ አይደለም	Barbaachiisa miti
	የተለመደ አይደለም	Waan baramee miti
	COVID-19 ን መፍራት	Fear of COVID-19
	ሌላ	Kan biroo
	ሁሌም የጤና ተቋም ነው የምወልደው	Yeroo hunda dhaabbata
	ባሉት ችግሮች ምክንያት	Sababii rakkooleen jirani
	የጤና ኤክስቴንሽን ባለሙያዋ/የልማት ቡድን መሪ ልከውኝ	DRMF/HEF ol na erganiir
	የነፍሰ ጡር ኮንፈረስ ላይ የተሰጠውን ምክር ሰምቼ	Gorsa konfireensii dubar
	ለረጅም ጊዜ ስላማጥኩኝ/ በወሊድ ጊዜ ችግር ስላጋጠመኝ ወደ ጠ	Ciniinsuun yeroo dheera
	ተመራጭ ስለሆነ/ስለማያስቸግር	Mijaawaa
	አቅምን ያገናዘበ/ ነጻ ስለሆነ	Kaffaltiin isaa salphaa wa
	የጤና ልማት ቡድን መሪዎች ስለመከሩኝ	DRMF waan na goorsani
	በቅድመ ወሊድ ጊዜ ስለመከሩኝ	Yeroon Tajaajiila da'ums
	ሌላ	Kan biroo
	ትኩሳት	Dhagna gubaa
	ማጥባት / መመገብ አልተቻለም	Harma hodhuu dadhabu
	አስቸጋሪ / ፈጣን መተንፈስ	Saffisaan afuura baafach
	ተቅማጥ	Garaa kaasaa
	መንቀጥቀጥ	Urgufamuu
	የማያቋርጥ ማስታወክ	Hooqqisiisaa
	ቢጫ መዳፎች / ነጠላ / ዓይኖች	Barruun harkaa/faanni n
	ግድየለሽነት	of dadhabuu/
	ንቃተ ህሊና	of wallaaluu
	ቀይ / የሚለቀቁ ዓይኖች	Dhangala'aa diimaan ija
	የቆዳ pustules	Shiffee qaamaa
	በገመድ ዙሪያ ያለው ቆዳ ቀይ ነበር	Gogaan handhuura cinaa
	Usስ ከገመድ	Malaa handhuura keessa
	ሌላ	Kan biro
	እሱ / እሷ የተሻለ እንደሚሆን ይጠብቃል	Nifooyyofti jennee eegna
	የጤና ተቋም በጣም ሩቅ ነው	Dhaabbatni fayyaa fagoo
	የሕክምና ዋጋ በጣም ውድ ነው	Gatiin wal'aansaa mi'aa
	ተቋሙን አትመኑ	Dhaabbata fayyaa irraa a
	የቤተሰብ አባል አልፈቀደም	Maatiin hayyamamoo ta
	የማህበረሰብ አማካሪ / ቲባ / እንዳይመከሩት መከረዋል	Gorsaan hawaasaa/ dees

	COVID-19 ን መፍራት	Sodaa COVI-19
	ተቋሙ በ COVID-19 ምክንያት ተዘግቷል	Sababa COVID-19n dhaa
	ተቋሙ አልተከፈተም (በዚያን ጊዜ)	Yeroo sana dhaabbatni f
	ሌላ	Kan biroo
	የጤና ኬላ	kellaa Fayyaa mootumm
	ጤና ጣቢያ	Buufata Fayyaa
	ሆስፒታል	Hoospitaala
	የማህበረሰብ ጤና ሰራተኛ	Hojjettuu/taa fayyaa hav
	ሱቅ / ፋርማሲ	Phaarmaasii/ suuqii
	ሌላ	Kan biraa
	የወባ መድኃኒቶች- Quatem	Qoricha busaa: Quartem
	የወባ መድኃኒቶች-ክሎሮኪን	Qoricha busaa: Chloroqu
	አንቲባዮቲክ: - gentamycin	Qoricha farra dhukkubaa
	አንቲባዮቲክ-አሚሲሊን	Qoricha farra dhukkubaa
	ቴትራክሲን / ሌላ የዓይን ቅባት	Dibata ijaa (TTC)
	ኦ.ኤስ.ኤስ.	ORS
	ቫይታሚን ኤ	Vitamin A
	ባህላዊ መድሃኒት	Dawwaa aadaa manatti
	ዕፅዋት	Dawaa biqiloota irraa ar
	other medication	Dawaa Kan biroo
	በደንብ መመገብ አቁሟል	Harma hodhuu /soorach
	አስቸጋሪ ወይም ፈጣን መተንፈስ	Afuura baafachuu dadha
	በደረት ውስጥ ስዕል	Lapheen jalli keessatti ga
	ያልተለመደ ሙቅ ወይም ቀዝቃዛ	Haala hinbaratamneen c
	ከተለመደው ያነሰ እንቅስቃሴ ያለው ህፃን	Sochiin daa'imni agarsiis
	ሰውነት ቢጫ ሆነ	Qaamnio daa'imaa keell
	ሌላ	Kan biroo
	ምልክቶች የሉም	Mallattooleenn hinjiran
	WDA	WDA
	HEW	HEW
	ነርስ / አዋላጅ	Nursii/ Nursii deessistuu
	ዶክተር	Hakiima
	የጤና መኮንን	Qondaala fayyaa
	ፋርማሲስት	Faarmaasistii
	ሌላ	Kan biroo
	የሳንባ ምች	Nimooniyaa
	ተቅማጥ	Garaa Kaasaa
	ከባድ ኢንፌክሽኖች	Dhibee baay'ee cimaa
	ሌላ	Kan biroo
	HEW	HEW
	WDA	WDA
	ሌላ	Kan biroo
	አዎ	Eyyee
	የለም ፤ ግን ብቁ ነው	Lakki, garuu gochuu ni d
	ሊገዛ የሚችል አይደለም	Lakki, gochuus hindanda
	ከ 1 እስከ 30	1 to 30

For peer review only

	ከ 1 እስከ 5	1 to 5
	ድካም	Qufaa walirraa hincinne
	ትኩሳት	Gubaa qaamaa
	ሳል	Qufaa
	በጉንፋን የተዘጋ ጉሮሮ (sore throat)	Qaamni argansuu utaall
	ራስ ምታት	Mata bowuu
	የአፍንጫ ፍሳሽ (runny nose)	Dhangalaa'aa funyaan ke
	የቆዳ ሽፍታ	Shiffee qaamaa
	የትንፋሽ እጥረት	Afuurri ciccituu
	የጡንቻ እና የመገጣጠሚያ ህመም	Dhukkubbii buusaawwa
	ተቅማጥ	Dhukkubbii qomaa
	ማሽተት ወይም ጣዕም ማጣት	Fuunfachuu fi dhandhan
	ማቅለሽለሽ	dhiphina / namatti toluu
	ትውከት	Waan godhan wallaaluu
	የመተንፈሻ አካላት ጠብታዎች (droplets)	Dhangala'aa yeroo qufa'
	ዕቃዎች እና ገጽታዎች	Meeshaalee/ wantoota i
	አካላዊ ንክኪ	Wal tuttuquu
	ትንኝ ንክሻ	Yeroo Bookee busaatiin
	የተንቀሳቃሽ ስልክ አውታረመረቦች	Bilbilaan
	Don't know	Hinbeeku
	አደጋ የለውም	Riskii/ balaa hinqabu
	ዝቅተኛ አደጋ	Riskii/ balaa xiqqaa
	ከፍተኛ አደጋ	Riskii / balaa guddaa
	በጣም ከፍተኛ	Risk/ balaa baa'ee gudda
	ቤት ውስጥ መቆየት	Manuma keessa turuu
	ማህበራዊ መለያየት	Fageeny keenya eeggach
	መደበኛ እጆችን መታጠብ	Deddeebinee harkakeen
	ንጽሕናን መጠበቅ	Saanitaayizerii fayyadam
	በሚያስል እና ማስነጠስ ጊዜ መሸፈን (Covering cough and	Yeroo qufaanuu fi axxinf
	ጭምብል መልበስ	Haguugduu funyaanii (M
	የፀሐይ መጋለጥ	Aduutti of baasuu
	አልኮል መጠጣት	Alkoolii dhuguu
	ሎሚ ወይም ዝንጅብል ሻይ	Shaayii loomii fi Zinjibila
	የቪታሚን ተጨማሪዎች (Vitamin supplements)	Vaayitaaminoota fudhac
	ሁሉም ሰው ከ2-4 ሳምንታት ይሞታል	amni hunduu torbee 2-4
	አንዳንዶቹ ይተርፋሉ ፣ ብዙዎች ይሞታሉ	Namni tokko tokko irraa
	አብዛኛዎቹ ይተርፋሉ ፣ አንዳንዶቹ ይሞታሉ	Namni tokko tokko irraa
	ሁሉም ሰው ማለት ይቻላል በሕይወት ይተርፋል	Namni hundumtuu irraa
	አላውቅም / እምቢ ማለት	Hinbeeku/ hinbarbaadu
	የለም ፣ ግን በላቦራቶሪ አልተረጋገጠም	Lakki, garuu qorannoodha
	አዎ ተፈትኖ ውጤቱ ፖዘቲብ ነበር	Eyyee, qoratamee posetiiv
	አዎ ተጠርጣሪ ግን በሙከራ አልተረጋገጠም	Eyyee, shakkamaadha garu
	የለም ፣ ተፈትኖ ውጤቱ ነጋቲብ ነበር	Lakki, qoratamee Negeti
	አታውቅም	Hinbeeku
	አይ	Lakki
	አዎ ተረጋግጧል	Eyyee, mirkanaa'eera

For peer review only

	አዎ ተጠርጣሪ ግን በላቦራቶሪ አልተረጋገጠም	Eyyee, shakkamaadha ga
	አታውቅም	Hinbeeku
	መልስ ለመስጠት አሻፈረኝ	Deebisuu dide
	ምንም	Homtuu
	አዘውትሮ እጅን በሳሙና መታጠብ	Saamunaaf bishaaniin ar
	ቦታዎችን በፀረ-ተባይ ማጥራት	Kemiikaalaan lafa qulqul
	ከታመሙ ሰዎች ርቀትን መጠበቅ	Fageenya keenya nama
is not a member	የቤተሰብዎ አባል ካልሆኑ ሰዎች ሁሉ (ወይም ከእርስዎ ጋር ከሚ	Namoota miseensa maa
r mosques	ወደ ማህበራዊ ስብሰባዎች ፣ አብያተ ክርስቲያናት ወይም መስጊ	Lafa namootni itti baay'a
	የፊት ጭምብልን መልበስ	Haguugduu afaanii fio fu
:ine	በምግብ ፣ በቤት አቅርቦትና በመድኃኒት መከማቸት	Dhiiyyeessii manaaf bark
	የጉዞ እቅዶችን መለወጥ / መሰረዝ	Karoora deemsaa keeny
	እጆችን በአልኮል እና በንፅህና ማጽጃዎች ያፅዱ	Harkaa keenya alkoolii fi
	ብዙውን ጊዜ እኔ እንደማደርገው የአካል ብቃት እንቅስቃሴ	Sochii qaamaa kan duraa
	እኔ ብዙውን ጊዜ ከሚያደርገኝ የበለጠ አልኮል ጠጣ	Dhugaatii alkoolii kan du
	እኔ ብዙውን ጊዜ ከምመገበው የበለጠ ጤናማ ያልሆነ ምግብ በል	Soorata kanduraa irra ca
ɔuld be postpone	ለሌላ ጊዜ ሊዘገዩ በሚችሉ ጉዳዮች ለምሳሌ ወደ ክትባት ወይም	Tajaajila walittifufiinsa q
ıe	የቤተሰብ አባላት ወይም ጓደኞች እንዳይጎበኙኝ ተጠይቋል	Matiin ykn hiriyoonni ke
iend.	ልጄ ከጓደኛ ጋር መገናኘት እንደማይችል ወሰንኩ ።	Daayimman keeny akka
:ating COVID-19	COVID-19 ን ለማከም ጥሩ የሆኑ የሰማኋቸውን መድኃኒቶች ገ	Dawaa COVID-19 fayyisu
;, gloves).	የግላዊ መከላከያ መሣሪያዎችን (ጭምብሎች ፣ ጓንቶች) ገዝቷል ፡	PPE kanneen akka maasl
	መልስ ለመስጠት አሻፈረኝ	Deebisuu hinbarbaanne
	አታውቅም	Hinbeeku
	እስከ 3 ቀናት	Hamma guyyoota 3'i
	እስከ 7 ቀናት	Hamma guyyaa 7
	እስከ 14 ቀናት	Hamma guyyoota 14
	አታውቅም	Hinbeeku
	ኮሮናቫይረስን ለማከም መድኃኒት አለ	Novel koronavaayirasii w
	ለልብ ወለድ ኮሮናቫይረስ ክትባት አለ	Novel koronavaayirasii d
accine for the no	ለህክምናው መድሃኒትም ሆነ ለኮሮናቫይረስ ክትባት አለ	Novel koronaavaayirasii
e for the novel cc	ኮሮናቫይረስ ምንም ዓይነት የመድኃኒት ሕክምና ወይም ክትባት	Dawwaanis ta'ee talaalli
	አታውቅም	Hinbeeku
	በጣም ከፍተኛ	Baay'ee guddaa
	ከፍተኛ	Guddaa
	ዝቅተኛ አደጋ	Xiqqaa
	በጣም ዝቅተኛ	Baay'ee xiqqaa
	የድህረ ቃል	yeroo isaarra darbee (pc
	term (> = 37 ሳምንታት)	Yeroo isaatti (torbee 37
	መጀመሪያ (34-37 ሳምንታት)	Dafee (torbee 34-37 tti)
	በጣም ቀደም (በ 32 ሳምንቶች ብሎ ወይም)	Baay'ee dafee (torbee 3
	እንደገና እንዳታስታውስ	Hin Yaadadhuu
	ANC ጉብኝትን ለ 3 ወራት ያዘገዩ	ANC dhaaf hamma ji'a 3
	የኤኤንሲ ጉብኝትን ቢያንስ ለ 6 ወራት ያዘገዩ	ANC dhaaf yoo xiqqaate
	በምጥ ጊዜ ጤና ተቋም ጉብኝት መዘግየት (delay facility visi	Da'umsaaf gara dhaabba
	ወዲያውኑ ከወለዱ በኋላ ወደ ቤትዎ	Akkuman da'eenan gara
	የ PNC ጉብኝት አለማድረግ	Tajaajila da'umsa booda
	ለ PNC የቤት ጉብኝት ውድቅ ተደርጓል	Tajaajila da'umsa booda
	የለም	Hinjiru

ilomaa
grii ykn isaa ol

anaa'e
enye

aa kan galee
eessaa
aa
amee
saa/ simmintoon ijjaarame
me
aadammee
eessaa

shaan hidhamee

For peer review only

si
ı keessatti argama

ıquu (Wuha aggarii)
ːssuu/calaluu
san kan akka ashawaa, kan suphee irra hojeetamuu fi kkk fayyadamuu

ı/qararu taasisuu

nmoo qilleensaa qabu
iin kan hojjetu
ɔan/bosona/diree irratti bobba'uu

o/
in bocamnee
ımee kan xumurame
ɔcaameen
ːiber

mmurameen

ɉabu
ıukaa/leemmana/shanbaqqoo

ıee
qabu irra kan hojeetamee
ıeen

ɔ duuda irraa

ımne irra

ː
ːan ture
ɉuufera

For peer review only

yaa maatii arguu danda’eera
raa matii arguu hin dandeenye

waraqaa ragaa argeera
garuu waraqaa ragaa hin argine
haa jiru

uuphameef irra deebi'uun cuuphama kan hin barbaanne
roo yeroon kan cuuphamu

raa
odaa

’immanii
ol-erguu

nyoo Sombaa
’bituu
a’umsaa
aa’ima reefu dhalatee/dhalattee
kan dhunfaa

nii fayya
a (1:5 ykn 1:30)

teef/wan humna dhabdeef
atteef/wan humna dhabdeef

uu
aateef
eef
a gu'uu wan ga'eef

For peer review only

a maatii fayyaadamuu jalqabdeef

alqabnee

a'a/

akka bishaan shukkaaraa, lallaafaa, shorbaa/
n dheedhii adda addaa irraa hojjeetamee/garbuu, bisingaa, boqooloo, Ruuzii, qanmadii, xaafii)
akka buqqee, Kaaroottii, hundee dimaa, dinnichaa/moosee, baaloota magarisaa, Mangoo, Papay
maa irraa hojjetamee/ kan akka kasavaa, worqee nyaataa/
udraa fi fuduraa irraa qopha'ee/ kan akka muzii, timatimii, Abukadoo, Apilii)

n falaffalamaniirraa qophaa'ee (Ataraa, Baqeelaa, Misira, Ochoolonii)
zayitii nyaata, dhaadha irra hojjetame
amee
illee

qaa

ee
qaa

a
maa

maa
ma ganda kessaa/olama-gala/

For peer review only

nootumma
otummaa ganda kessaa/olamaa-gala/
wasaa
saa
vasaa

rra bacteriyaa/Shirooppii
/bobbaatii furdisuu

roo kan farraa (bakiteeriyaa ykn antibayootiki hin tanee)
ekaminee
eriyaa kan lilmoon keenamuu
eriyaa kan hin taane lilmoon kan keenamuu

alaan keenamu
tti qopha'ee
in kenamneef

amu

siisuu

ouu
naa qabaatee/dafee dafee afuura yoo bafata ta'ee
qorree/diilallaa'ee
aayyee xiqqoo ta'ee/yoo daa'imni yeroo male dhalate
/ yoo mala'ee

For peer review only

a'ee
laa jala/ijii keelloo ta'ee
cho'u yoo ta'e
ɔ ta'ee
ɔo hirqiffatee boo'a ta'e
mne

raa kaasaa qal'a
yyuu
amsiisuu
ıu
naka qabu
achuu
ıa
ee qabu

haan dhuge

ladhabee
e/yoo baayyee dhukkubsate

kkubsate

ımaa
ıaa
-mootummaa
nfaa

ıaan isaa hin beekamne

e.

For peer review only

ɛffachuu
ı itti da’uu adda baasuu/filachuu
ee barbaachisoo ta’an qopheessuu
ɛnnan qopheefachuu
ıu qopheeffachuu

ımaa

mootummaa

ɛimii dhuunfaa

ɛeffaa)

ıa

ı

ee

ıffeelamne

For peer review only

an hidhaan

ıee

ophaa'e irra

orsuu
itti kennuu
u

ıaadhaan haxaahuu

ı (1:5 ykn 1:30)

adhaa malee

uukoosii

rshaa (Formulaa)

un maruu/haguuguu

ka wal tuquu gochuu

l hin tuqiin)

haadhaan wal tuqu taasifamee

a
eenjitee

i ittiin maruu)
ı

u
aleen) karaa naf-saalaa dhangala'u

a
ıin ibsamne

For peer review only

qopha'ee

ınfaa muduraalee
ıu baraarsu
warshaa/Formulaa

ulbulaa bishaanii fi ashaboo
kan qophaa'e/"hamesa"

ıdi

onee

;ala
ı galu
u
1:5)

ı irratti maxxanfameera
a irratti hin maxxanfamne

ıaan kan adda hin baane)

ʻa/quufeera
deera/quufeera
ımanne/quufne
ıanne/quufne

For peer review only

For peer review only

ːra
ːorsameera
aa irratti hubannoo argadheera
umsa argadheera
na nyaataa argadheera
ti ol-erguu
ameera
madaan uumamu ilaalameera/ yaalameera (yoo ilaallata ta'e)
aroora maatii

sakata'uu

laa'imaaf godhameera
ːuu

osisuu
a ilaalaniiru
a qaama haadhaa fi daa'imaa wal tuqsiisanii hammachuu
aaf (Faalama dabalaatee) sakatta'insa daa'imaaf gochuu
laaa cimaarratti barsiisuu
ːguu
ima hin tuqiin dura harka dhiqachuu
a fi qabiinsa handhuuraa
dhalatee sa'aa 24 keessatti dhaqna dhiquu dhiisuu

a hin qabu
waliin waan hin oolleef
koo waan nagorsaniif
ta

ee/funaannee)

ee/hoffaa)

/Manatti nyaata hin qopheessan
essa keessaa dhangala'u
naf-saalaa irraa bahuu

tti ciminaan dhagaamu (dhiita'us dhiita'uu baatus)
jja'uu
mne

dameesa kessa dhangala'e
yoo gadameessa keessa ba'ee

tuu dursee ba'ee
essatti tessoon isa sirrii yoo hin taane
sa'atii 12 ol

oo malee dhangala'ee
dhufee
a mormaa isatti maramee
atee

ate/tte
msaa osoo hin ga'iin bishaan gubbee yoo dhangala'e
hinibsine

u'e

For peer review only

ı bakkaa adda addaa jiraatan
in da'aa ture
Amaatiin waan hin heyyamneef
ıuu hin jaaladhu
ee fayyaa guddaa waan ta'eef
fagoo ta'eef

ee/amantaaf
addaa kanaan walqabateef
cufaa tureef
ayyaa gadi aana waan ta'eef
lubartii taate waan hin jirreef

fayyaattiin da'aan ture
iif
ru
rtoota ulfaarratti dhaga'een
ıa waan narra tureef (Rakkoo yeroo da'umsaa namudateef)gara dhaabbata fayyaa nageessan

aan ta'eef
iif
ıa dura fudhaadhu waan na gorsaaniif

ıu
ıuu / afuura baasuu dadhabuu

niillaa/ iji keelloo ta'uu

keessaa ba'uu

a diimachuu
aa ba'u

a
ɔ ta'uu
ta'uu
amantaa dhabuu
ı'uu dhabuu
ssistuun aadaa faallaa isaa waan nagorsiteef

ew only

ıbbatni fayyaa waan cufameef
fayyaa waan hinbanaminiif

ıaa

waasaa

ı
ıine
a : gentamycin
a (Antibiotic): ampicillin

qophaa'u
gamu

ıuu dhaabeera
abuu/ saffisaan afuura baasuu
adi dhooquu
dhagna gubuu / qorrisiisuu
su kan baratamee gadi ta'uu
oo ta'uu

ı

landa'a
a'u

oon cufamuu (sore throut)

essaa yaa'u

nii

nuu dadhabuu
ı dhiisuu
(Confusion)
'an/ haasa'an afaan keessaa ba'u
itti fayyadamnu

nama ciniintu

aa

huu (meetira 2)
ıya dhiqachuu
ıuu
fannu afaanii fi funyaankeeng aguuguu
Maskii) fayyadamuu

qabu dhuguu
chuu
ł keessatti du'a
ı hafu, hedduun ni du'u
ı hafu, tokko tokko ni du'u
ı hafu

an hinmirkanoofne
vii ta'eera
uu qorannoon hinmirkanoofne
iivee ta'eera

For peer review only

aruu qorannoon hinmirkanoofne

mma amma arka dhiqachuu
lleessuu
dhukkubsataa irraa eeggachuu
tii hintaane garuummoo waliin jiraannu irraa fageenya keenya eeggachuu
atan dhaquu dhiisuu (Mana amantaa, gabaa)
ınyaanii fayyadamuu
baachisan (Nyaata, meeshaa fi qorichoota) haalaan qabachuu
a haquu
i saanitaayizeriin qulqulleessuu
anii irraa hir'isuu
ıraa irra dabaluu
ıalaa nyaachuu/ soorachuu
jabuuf bella hakiima waliin qabnu addaan kutuu (Talaallii, ilaalamuu ykn checkup)
enya akka gara manakeenyaa hindhufne gochuu
hiriyoota isaanii waliin wal hinagarree murteessuu
ıuf gargaaru isan dhaga'e sanan bitadhe
kii fi gilaaviin bitadhe

val'aanuuf qorichi jira
dhaaf talaalliin jira
dhaaf dawaanis ta'ee talaalliin jira
in yeroo ammaa kan Novel koronavaayirasiif hinjiru

ɔst term)
fi isaa olitti)

2 fi isa dura)

tti tureen deeme
hamma ji'a 6tti tureen deeme
ata fayyaa dafee hindeemne
manakootti deebi'ee gale
a hinarganne
af daawwannaan manamanaa hi'rateera

For peer review only

For peer review only

For peer review only

yaa

For peer review only

For peer review only

For peer review only

For peer review only

For peer review only

For peer review only

For peer review only

For peer review only

For peer review only

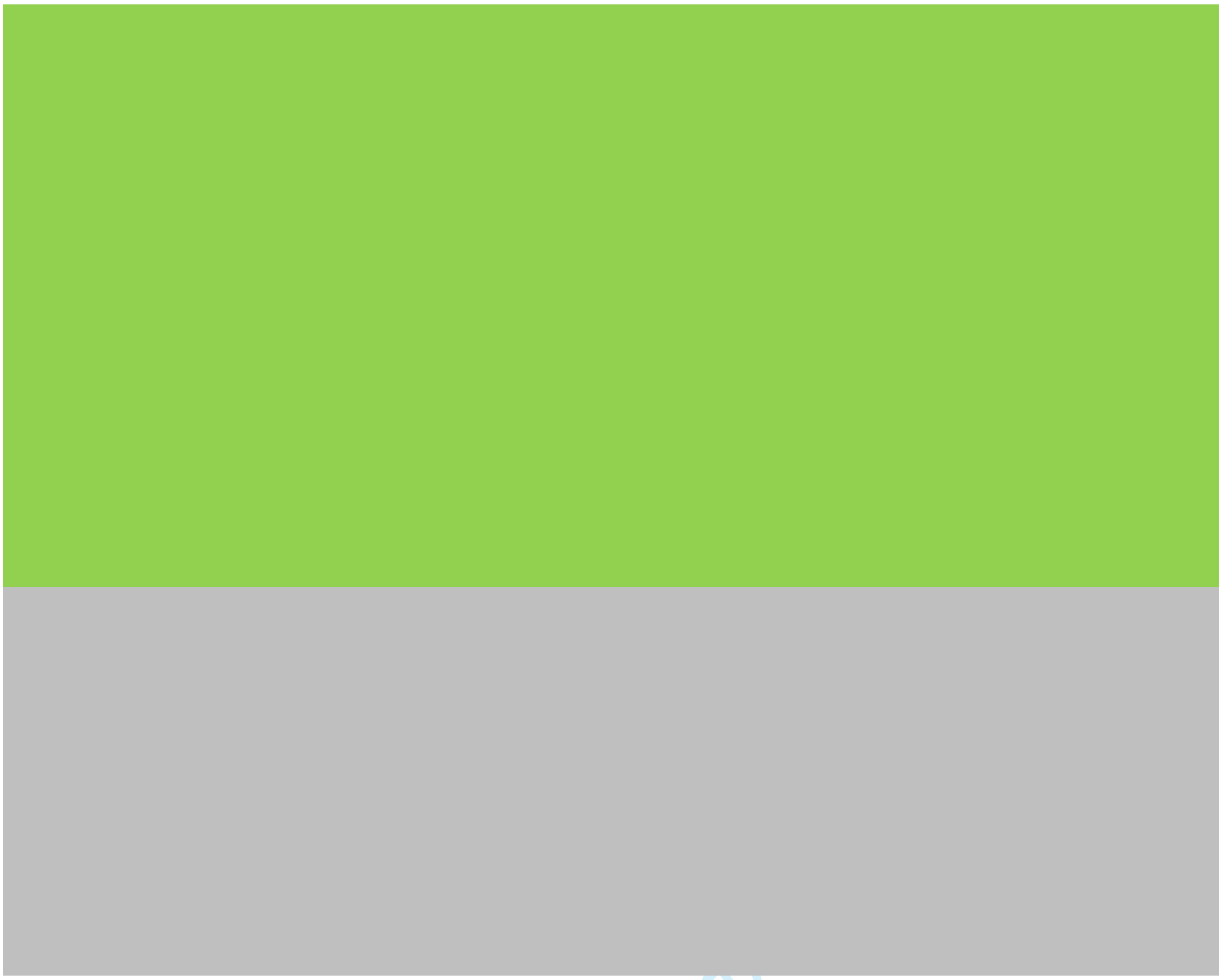

ew only

For peer review only

For peer review only

For peer review only

For peer review only

For peer review only

For peer review only

For peer review only

For peer review only

For peer review only

For peer review only

For peer review only

For peer review only

For peer review only

For peer review only

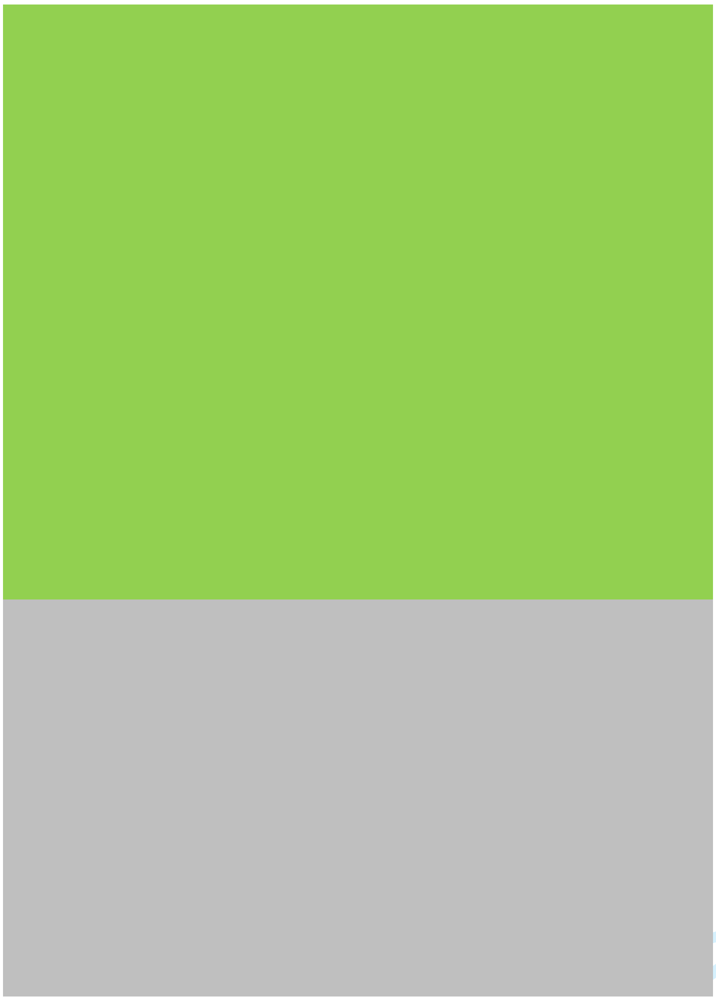

iew only

For peer review only

For peer review only

q108	q109	q111	q112	q114	q200	q201	q202	q203
Male	5	Yes	8	69	38	No		
Male	7	Yes	4	60	30	No		
Female	6	Yes	6	108	36	No		
Male	3	Yes	4	3	35	Yes	Yes	6th
Male	6	Yes	7	81	40	No		
Female	12	No	1		25	No		
Female	14	Yes	6	4	35	No		
Male	4	No	1		20	Yes	Yes	11th
Male	13	Yes	5	84	33	No		
Male	14	No	1		18	Yes	Yes	5th
Male	4	Yes	7	4	40	No		
Male	7	Yes	5	23	31	No		
Male	3	Yes	2	72	30	Yes	Yes	9th
Female	2	No	1		19	Yes	Yes	11th
Male	7	Yes	2	5	30	No		
Female	9	Yes	9	38	38	No		
Male	6	Yes	6	2	35	No		
Female	7	Yes	8	6	42	No		
Female	4	Yes	3	96	23	No		
Male	9	Yes	2	120	28	Yes	Yes	9th
Female	13	Yes	4	1	32	No		
Female	7	Yes	3	84	30	No		
Male	2	Yes	6	43	32	No		
Female	9	Yes	4	5	35	No		
Male	6	Yes	6	52	40	No		
Male	2	Yes	4	4	32	No		
Female	9	Yes	4	3	35	No		
Male	12	Yes	3	36	27	No		
Male	3	Yes	6	31	40	No		
Female	9	Yes	7	2	38	No		
Female	11	Yes	5	3	36	No		
Male	10	Yes	7	35	40	No		
Female	14	Yes	6	27	30	No		
Male	5	Yes	2	3	33	No		
Male	5	Yes	3	36	33	No		
Female	10	Yes	5	4	30	Yes	Yes	8th
Male	3	Yes	3	3	30	No		
Male	3	Yes	3	4	29	Yes	Yes	10th
Female	6	Yes	4	72	28	No		
Male	10	Yes	5	4	30	No		
Female	6	Yes	5	3	34	No		
Male	14	Yes	4	6	35	No		
Female	2	Yes	6	24	35	No		
Female	14	Yes	5	4	38	No		
Female	7	Yes	2	60	20	Yes	Yes	3rd
Male	2	Yes	4	2	34	Yes	Yes	5th
Female	5	No	1		20	No		
Male	6	Yes	3	34	20	Yes	Yes	5th
Female	7	Yes	3	23	28	No		
Female	12	Yes	2	48	27	Yes	Yes	9th
Female	9	Yes	3	6	33	Yes	Yes	2nd

For peer review only

Male	14	Yes	4	3	35	Yes	Yes	5th
Male	9	Yes	5	4	35	No		
Male	3	Yes	2	60	27	Yes	Yes	8th
Female	14	Yes	5	46	30	No		
Female	12	Yes	4	144	35	Yes	Yes	3rd
Male	7	Yes	2	29	30	No		
Female	8	Yes	9	32	45	No		
Female	8	Yes	5	9	38	No		
Male	12	Yes	4	180	36	Yes	Yes	5th
Female	6	Yes	5	5	27	No		
Male	3	Yes	6	48	35	Yes	Yes	4th
Male	13	Yes	4	4	30	No		
Female	12	Yes	9	48	44	No		
Female	2	Yes	7	37	40	No		
Male	6	No	1		30	Yes	Yes	12th
Male	14	Yes	5	41	30	No		
Male	13	No	1		30	No		
Female	7	Yes	5	3	35	No		
Male	14	Yes	2	42	26	No		
Male	2	No	1		22	No		
Male	3	Yes	3	48	30	No		
Male	2	Yes	4	48	35	Yes	Yes	3rd
Female	7	No	1		25	Yes	Yes	10th
Female	7	Yes	2	4	30	Yes	No	
Male	7	Yes	6	37	33	No		
Male	4	Yes	7	84	35	No		
Male	8	Yes	6	5	40	No		
Female	12	Yes	4	72	29	No		
Female	6	Yes	4	7	32	No		
Female	13	Yes	5	4	35	No		
Male	8	Yes	4	2	30	No		
Female	9	Yes	5	3	28	No		
Female	14	No	1		22	Yes	Yes	5th
Female	8	Yes	4	12	42	No		
Male	14	Yes	9	2	45	No		
Male	14	Yes	8	40	40	No		
Male	14	Yes	3	12	42	No		
Male	12	Yes	3	42	27	Yes	Yes	7th
Female	6	Yes	7	28	38	No		
Male	10	Yes	3	3	27	Yes	Yes	4th
Male	12	Yes	3	80	35	No		
Female	10	Yes	2	120	37	Yes	Yes	4th
Male	8	Yes	5	4	35	No		
Male	7	Yes	3	40	27	No		
Female	10	Yes	5	3	35	No		
Female	13	Yes	4	36	35	No		
Female	14	Yes	3	4	27	No		
Female	3	Yes	3	2	27	Yes	Yes	7th
Female	11	Yes	3	2	27	Yes	Yes	10th
Female	12	Yes	4	5	34	No		
Male	2	No	1		28	Yes	Yes	10th
Male	2	Yes	3	4	30	Yes	Yes	8th

For peer review only

Female	10	No	1		21	Yes	Yes	10th
Male	9	Yes	6	43	40	No		
Male	6	No	1		20	Yes	Yes	10th
Male	9	Yes	3	3	28	No		
Male	12	Yes	2	1	22	Yes	Yes	8th
Male	3	Yes	4	2	30	No		
Female	9	Yes	4	7	33	No		
Male	6	Yes	5	4	38	No		
Male	2	No	1		22	Yes	Yes	7th
Female	2	Yes	5	48	35	No		
Female	13	Yes	7	40	37	Yes	Yes	5th
Male	14	No	1		23	No		
Male	14	Yes	5	3	35	Yes	Yes	4th
Female	10	Yes	7	34	38	No		
Female	8	Yes	6	96	40	No		
Male	11	Yes	3	96	35	No		
Male	2	Yes	5	32	30	Yes	Yes	10th
Male	14	Yes	6	3	35	No		
Female	7	Yes	3	56	30	Yes	Yes	7th
Female	4	Yes	4	2	30	Yes	Yes	2nd
Female	4	Yes	10	3	45	Yes	Yes	8th
Male	10	Yes	5	4	37	No		
Female	7	Yes	2	3	25	Yes	Yes	2nd
Male	3	No	1		20	Yes	Yes	8th
Female	9	Yes	8	96	35	Yes	Yes	6th
Female	9	Yes	2	8	30	No		
Female	8	Yes	5	3	37	No		
Female	5	No	1		20	No		
Male	11	Yes	7	3	38	No		
Female	8	No	1		27	Yes	Yes	10th
Male	14	Yes	5	9	35	No		
Female	8	No	1		28	No		
Female	11	Yes	7	40	32	No		
Female	9	No	1		23	Yes	Yes	4th
Male	10	Yes	5	3	35	No		
Female	5	Yes	4	4	30	No		
Male	7	No	1		20	Yes	Yes	7th
Female	14	No	1		22	No		
Female	9	Yes	1		24	No		
Male	11	Yes	4	68	36	No		
Male	9	Yes	4	2	30	No		
Female	2	Yes	3	36	30	Yes	Yes	5th
Female	5	Yes	7	4	43	Yes	Yes	6th
Male	14	Yes	5	72	30	No		
Female	2	Yes	8	48	35	No		
Male	12	No	1		23	Yes	Yes	7th
Female	5	No	1		18	No		
Female	3	Yes	5	3	26	No		
Female	11	Yes	4	48	30	No		
Male	12	Yes	3	3	34	No		
Female	3	Yes	6	30	30	No		
Female	7	Yes	4	3	25	No		

For peer review only

Female	2	Yes	6	20	28	No		
Female	10	Yes	5	3	36	No		
Male	2	Yes	1		25	No		
Male	13	Yes	2	7	25	No		
Female	6	Yes	4	3	28	Yes	Yes	3rd
Female	11	Yes	2	44	27	Yes	Yes	8th
Male	14	Yes	5	35	38	No		
Male	11	Yes	7	3	40	No		
Female	4	Yes	4	18	28	No		
Male	6	Yes	5	55	40	No		
Female	4	Yes	3	3	30	No		
Male	7	Yes	3	5	35	No		
Male	5	Yes	5	4	38	No		
Female	12	Yes	4	38	30	No		
Female	12	Yes	4	36	30	No		
Male	4	Yes	4	40	28	Yes	Yes	6th
Male	14	Yes	6	3	35	No		
Male	14	Yes	1		26	No		
Female	2	Yes	8	35	30	No		
Male	6	Yes	6	42	38	No		
Male	3	Yes	5	24	32	No		
Male	5	Yes	5	5	35	No		
Male	7	Yes	5	8	35	No		
Male	5	Yes	5	3	36	No		
Female	3	Yes	2	0	20	Yes	Yes	8th
Female	8	Yes	5	48	35	No		
Female	2	Yes	4	2	30	No		
Male	2	Yes	3	2	30	Yes	No	
Male	4	Yes	5	3	30	No		
Male	11	Yes	3	4	23	No		
Male	14	Yes	4	4	35	No		
Female	12	Yes	3	2	25	Yes	Yes	4th
Male	14	Yes	2	3	28	No		
Female	9	Yes	6	6	34	No		
Female	13	Yes	2	96	25	No		
Male	6	Yes	4	36	35	No		
Female	3	Yes	5	72	40	No		
Male	6	Yes	3	4	30	Yes	Yes	7th
Female	4	Yes	5	40	28	Yes	Yes	4th
Female	13	Yes	5	2	42	No		
Female	10	No	1		18	Yes	Yes	6th
Male	6	Yes	5	4	35	No		
Male	14	Yes	1		22	Yes	Yes	4th
Female	14	Yes	3	4	40	No		
Male	4	Yes	3	5	26	No		
Female	12	Yes	3	84	30	No		
Female	12	Yes	6	8	37	No		
Male	12	Yes	4	17	40	No		
Male	12	No	1		22	Yes	Yes	7th
Female	3	Yes	4	18	35	No		
Female	13	Yes	8	46	37	No		
Male	8	Yes	5	84	36	No		

For peer review only

Male	5	No	1		20	Yes	Yes	5th
Female	6	Yes	4	4	30	No		
Male	5	Yes	2	5	24	Yes	Yes	5th
Male	3	Yes	2	3	22	Yes	Yes	8th
Male	6	Yes	2	4	22	Yes	Yes	7th
Male	14	No	1		22	Yes	Yes	8th
Male	8	No	1		20	Yes	Yes	7th
Female	12	Yes	4	4	32	No		
Female	13	Yes	7	55	38	No		
Male	10	Yes	4	5	33	No		
Female	3	Yes	5	3	30	No		
Male	6	Yes	3	4	27	No		
Female	12	Yes	6	5	35	No		
Male	11	Yes	7	2	43	No		
Male	8	Yes	5	4	30	No		
Male	6	Yes	2	3	26	No		
Female	3	Yes	5	5	35	No		
Male	11	Yes	5	3	36	No		
Male	13	Yes	3	36	29	No		
Male	3	Yes	4	3	37	No		
Female	11	Yes	3	3	25	No		
Female	6	Yes	3	84	25	No		
Male	3	Yes	10	3	40	No		
Male	12	Yes	4	4	35	No		
Male	12	No	1		22	Yes	No	
Female	7	Yes	4	3	25	Yes	Yes	3rd
Male	9	Yes	4	3	35	No		
Female	6	Yes	4	3	38	No		
Female	6	Yes	2	24	29	No		
Male	3	Yes	5	4	30	Yes	Yes	3rd
Male	10	Yes	5	36	33	Yes	Yes	8th
Male	3	Yes	2	3	28	No		
Female	3	Yes	2	4	23	Yes	Yes	8th
Male	7	No	1		25	Yes	Yes	10th
Male	11	Yes	4	2	30	Yes	Yes	3rd
Female	4	No	1		22	No		
Female	14	No	1		30	No		
Female	4	Yes	5	3	30	No		
Male	7	No	1		23	No		
Female	6	Yes	1		25	No		
Male	14	Yes	6	3	40	No		
Male	7	Yes	2	11	30	No		
Female	7	Yes	3	39	27	No		
Male	7	Yes	5	94	30	Yes	Yes	3rd
Male	11	Yes	7	50	30	No		
Male	13	No	1		19	No		
Male	11	Yes	4	5	32	No		
Male	8	Yes	10	60	42	Yes	Yes	6th
Male	2	Yes	7	2	38	No		
Male	12	Yes	5	4	35	No		
Male	11	Yes	7	36	40	No		
Male	7	Yes	4	28	30	No		

For peer review only

Male	5	Yes	4	3	35	No		
Male	11	No	1		23	No		
Male	14	Yes	6	36	26	No		
Female	10	Yes	5	3	29	No		
Male	14	Yes	7	96	38	No		
Male	5	Yes	6	3	30	No		
Female	13	Yes	3	35	26	Yes	Yes	5th
Female	10	Yes	8	37	35	No		
Female	10	Yes	5	4	35	No		
Male	14	Yes	2	3	27	Yes	Yes	3rd
Male	8	Yes	6	50	35	No		
Female	11	Yes	5	30	33	No		
Female	11	No	1		20	No		
Female	2	Yes	4	3	27	No		
Female	12	Yes	6	5	34	No		
Female	3	No	1		22	Yes	Yes	3rd
Female	3	Yes	3	54	22	No		
Male	10	Yes	2	38	28	No		
Male	9	Yes	4	3	35	No		
Male	6	Yes	2	4	26	Yes	Yes	5th
Female	4	Yes	4	72	30	No		
Male	8	Yes	1		21	No		
Male	7	Yes	2	3	21	Yes	Yes	6th
Female	4	Yes	2	24	22	Yes	Yes	10th
Male	7	Yes	5	6	30	Yes	Yes	6th
Male	6	Yes	7	4	42	No		
Female	3	No	1		20	No		
Female	8	No	1		25	No		
Male	7	Yes	4	24	28	No		
Male	11	No	1		23	Yes	Yes	9th
Male	12	No	1		24	Yes	Yes	10th
Female	14	Yes	3	4	30	Yes	No	
Male	14	Yes	6	3	37	No		
Male	9	No	1		25	No		
Female	14	Yes	1		23	No		
Male	10	Yes	7	48	38	No		
Male	2	Yes	4	36	35	No		
Male	10	No	1		22	No		
Male	9	Yes	7	15	43	No		
Female	2	No	1		20	No		
Female	7	Yes	3	37	30	No		
Female	8	No	1		27	No		
Female	7	No	1		20	Yes	Yes	5th
Male	14	Yes	5	4	35	No		
Female	5	Yes	7	36	30	No		
Male	11	Yes	7	4	40	No		
Female	3	Yes	3	4	27	Yes	Yes	7th
Male	3	Yes	6	3	35	No		
Female	4	Yes	10	3	35	No		
Male	4	No	1		26	No		
Female	7	Yes	4	3	30	No		
Female	6	Yes	3	6	32	No		

Female	14	Yes	3	3	27	No		
Male	2	Yes	3	3	35	No		
Female	12	Yes	5	36	28	No		
Female	9	Yes	7	3	35	No		
Female	14	Yes	4	4	28	No		
Male	12	Yes	7	36	32	No		
Male	12	Yes	6	3	30	Yes	Yes	4th
Female	14	Yes	3	42	28	Yes	Yes	3rd
Male	3	Yes	2	37	30	Yes	Yes	9th
Female	5	Yes	7	2	40	No		
Female	14	No	1		19	Yes	Yes	6th
Female	12	No	1		23	Yes	Yes	4th
Male	3	Yes	3	2	25	Yes	Yes	8th
Male	14	Yes	6	48	32	No		
Female	3	Yes	5	24	27	No		
Female	3	No	1		20	Yes	Yes	5th
Male	7	Yes	3	4	23	Yes	Yes	7th
Female	14	Yes	2	6	30	No		
Male	4	Yes	4	4	30	No		
Male	9	Yes	2	65	22	Yes	Yes	6th
Female	9	Yes	6	3	35	No		
Female	12	Yes	4	4	30	No		
Female	6	No	1		23	Yes	Yes	5th
Male	3	Yes	5	2	30	No		
Female	11	Yes	4	7	35	No		
Female	7	No	1		20	No		
Female	2	Yes	6	28	38	No		
Male	8	Yes	3	2	25	Yes	Yes	5th
Male	2	Yes	4	27	29	Yes	Yes	10th
Male	5	Yes	3	2	26	Yes	Yes	6th
Male	13	Yes	2	78	38	No		
Male	4	Yes	5	36	38	No		
Male	11	Yes	2	36	31	No		
Female	2	Yes	3	30	28	No		
Female	4	Yes	5	60	35	No		
Male	9	Yes	2	5	20	No		
Female	11	Yes	4	60	40	No		
Female	10	Yes	7	59	35	No		
Male	9	Yes	6	41	32	No		
Female	9	Yes	3	96	34	No		
Male	7	Yes	2	60	24	No		
Male	6	Yes	2	58	28	Yes	Yes	5th
Male	7	Yes	3	35	25	Yes	Yes	5th
Female	8	Yes	2	60	27	No		
Male	4	Yes	2	120	30	Yes	Yes	8th
Female	2	Yes	6	36	37	No		
Male	7	Yes	4	240	35	No		
Female	13	Yes	2	96	28	Yes	Yes	6th
Female	6	Yes	5	40	30	No		
Female	4	Yes	3	108	35	No		
Female	9	Yes	6	96	40	No		
Female	4	Yes	3	59	30	No		

For peer review only

Male	8	Yes	3	72	31	No		
Female	13	Yes	4	48	36	No		
Male	8	No	1		17	No		
Female	7	Yes	5	5	35	No		
Female	4	Yes	4	23	35	No		
Male	14	Yes	5	45	38	No		
Female	12	Yes	2	6	25	No		
Female	8	No	1		21	Yes	Yes	4th
Male	3	Yes	5	48	35	No		
Male	14	Yes	4	144	36	No		
Male	5	Yes	3	36	26	Yes	Yes	3rd
Female	3	Yes	2	4	29	No		
Female	9	Yes	5	33	36	No		
Female	6	Yes	5	50	30	No		
Male	2	Yes	4	84	35	No		
Male	10	Yes	3	72	32	No		
Male	2	Yes	9	36	38	No		
Female	8	Yes	4	96	30	No		
Female	10	Yes	3	84	25	Yes	Yes	4th
Female	11	Yes	6	4	35	No		
Female	8	Yes	4	10	38	No		
Male	7	Yes	3	84	29	No		
Male	11	Yes	5	47	31	No		
Male	8	Yes	5	49	35	No		
Female	9	Yes	10	36	40	Yes	Yes	4th
Male	5	No	1		23	Yes	Yes	5th
Female	7	Yes	4	32	38	No		
Male	11	Yes	2	61	30	No		
Male	12	Yes	4	72	30	No		
Male	8	Yes	2	108	26	No		
Male	5	Yes	6	3	27	No		
Female	7	Yes	4	38	40	No		
Female	4	Yes	5	36	35	No		
Male	14	Yes	7	60	36	No		
Male	8		1		22	Yes	Yes	10th
Female	10	Yes	4	5	33	No		
Female	7	Yes	8	1	30	No		
Female	6	Yes	5	2	30	No		
Male	8	Yes	7	4	35	No		
Male	12	Yes	5	3	30	No		
Male	6	Yes	2	84	36	No		
Male	9	Yes	2	72	23	No		
Male	11	Yes	4	4	25	No		
Female	2	Yes	3	38	35	No		
Female	13	Yes	6	4	38	No		
Male	6	No	1		22	Yes	Yes	2nd
Female	9	Yes	4	3	35	No		
Female	12	Yes	3	56	29	Yes	Yes	3rd
Male	5	Yes	2	5	30	No		
Female	14	Yes	3	60	28	Yes	Yes	9th
Male	2	Yes	3	16	28	No		
Female	2	No	1		23	Yes	Yes	11th

For peer review only

Female	5	Yes	2	71	23	No		
Female	14	Yes	2	84	33	No		
Male	12	Yes	5	4	35	No		
Male	3	No	1		29	No		
Male	12	Yes	3	4	30	Yes	Yes	9th
Female	6	Yes	6	4	35	No		
Female	7	Yes	3	3	30	No		
Male	6	Yes	8	3	40	No		
Male	6	Yes	5	3	35	No		
Male	5	Yes	2	4	24	Yes	Yes	Technical/[illegible]
Female	5	Yes	4	4	35	No		
Male	12	Yes	2	3	25	No		
Female	4	Yes	4	4	35	No		
Female	7	Yes	3	7	30	No		
Male	9	Yes	2	4	28	Yes	Yes	9th
Female	4	Yes	7	4	35	No		
Female	9	Yes	3	2	25	No		
Male	14	Yes	3	5	36	No		
Female	5	Yes	5	3	30	No		
Male	2	Yes	4	3	30	No		
Female	9	Yes	3	4	33	No		
Female	14	Yes	1		21	Yes	Yes	10th
Female	8	No	1		30	No		
Female	13	No	1		25	Yes	Yes	7th
Female	2	No	1		20	Yes	Yes	8th
Female	8	Yes	3	7	35	No		
Female	14	Yes	6	3	35	No		
Female	9	Yes	1		27	Yes	Yes	University/
Male	7	No	1		24	No		
Male	14	No	1		25	No		
Female	2	Yes	3	5	35	No		
Female	11	Yes	1		21	Yes	Yes	4th
Male	12	Yes	2	3	23	No		
Male	12	Yes	9	7	35	No		
Male	10	Yes	2	2	30	No		
Female	6	Yes	2	10	30	No		
Male	2	No	7	6	40	No		
Female	5	Yes	4	4	30	No		
Female	7	Yes	2	4	25	No		
Male	8	Yes	3	4	30	No		
Female	12	Yes	4	3	28	No		
Male	11	Yes	5	3	35	No		
Male	7	No	1		24	Yes	Yes	10th
Female	7	Yes	6	5	35	No		
Male	14	Yes	6	3	35	No		
Female	14	No	1		25	No		
Male	3	Yes	4	5	33	No		
Male	3	Yes	5	3	38	No		
Female	6	No	1		25	No		
Female	14	Yes	4	7	35	No		
Male	4	Yes	4	5	35	No		
Female	9	Yes	5	4	35	No		

For peer review only

Male	13	No	1		18	Yes	Yes	8th
Female	3	Yes	11	3	40	No		
Male	12	Yes	4	3	35	No		
Male	4	Yes	2	5	25	No		
Female	14	No	1		22	No		
Female	6	Yes	3	3	33	Yes	Yes	8th
Female	9	No	1		25	Yes	Yes	3rd
Male	7	Yes	3	4	22	Yes	Yes	4th
Male	14	Yes	2	4	30	No		
Female	9	Yes	3	1	30	Yes	Yes	10th
Male	7	No	1		25	No		
Female	12	No	1		23	No		
Female	6	Yes	2	10	27	No		
Female	10	Yes	2	2	24	Yes	Yes	8th
Male	5	Yes	5	12	40	No		
Female	11	No	1		25	Yes	Yes	8th
Female	7	Yes	5	3	30	No		
Male	14	Yes	5	48	40	No		
Male	14	No	1		18	No		
Male	5	Yes	5	27	30	Yes	Yes	4th
Female	7	No	1		18	Yes	Yes	7th
Male	13	Yes	2	999	23	Yes	Yes	7th
Male	13	No	1		18	No		
Female	3	Yes	2	82	22	Yes	Yes	6th
Male	7	Yes	3	48	22	Yes	Yes	5th
Male	7	Yes	3	36	30	No		
Female	14	Yes	2	59	22	Yes	Yes	6th
Female	7	Yes	2	36	21	No		
Male	5	Yes	2	24	30	No		
Male	9	Yes	4	48	25	No		
Male	14	Yes	3	48	28	Yes	Yes	8th
Male	12	Yes	2	36	20	No		
Male	4	Yes	1		26	Yes	Yes	6th
Male	14	Yes	2	72	26	Yes	Yes	9th
Female	4	Yes	5	48	35	No		
Female	7	Yes	2	72	24	Yes	Yes	6th
Female	6	Yes	4	36	27	No		
Male	14	No	1		22	Yes	Yes	8th
Male	12	Yes	2	60	20	Yes	Yes	4th
Male	3	Yes	3	84	28	Yes	Yes	4th
Female	8	Yes	7	132	38	No		
Female	12	Yes	2	96	20	No		
Male	14	Yes	2	2	23	Yes	Yes	5th
Female	2	Yes	5	84	30	No		
Female	10	No	1		18	Yes	Yes	7th
Male	12	Yes	5	96	37	No		
Male	14	Yes	6	60	35	No		
Male	9	Yes	4	108	32	Yes	Yes	4th
Male	4	Yes	4	48	36	No		
Female	14	Yes	4	48	30	Yes	Yes	3rd
Male	7	Yes	2	7	20	Yes	Yes	4th
Female	5	No	1		20	Yes	Yes	7th

For peer review only

Male	9	Yes	3	60	27	Yes	Yes	4th
Male	2	Yes	4	36	27	No		
Male	9	No	1		21	No		
Female	10	No	1		20	Yes	Yes	4th
Male	9	No	1		20	Yes	Yes	8th
Male	7	No	1		21	Yes	Yes	3rd
Male	9	Yes	2	36	26	Yes	Yes	7th
Female	3	No	1		18	Yes	Yes	5th
Female	12	Yes	2	48	20	No		
Female	11	Yes	2	36	22	Yes	Yes	10th
Male	11	No	1		19	No		
Male	12	Yes	3	72	28	Yes	Yes	5th
Male	12	Yes	1		18	No		
Female	10	Yes	1		26	Yes	Yes	10th
Female	11	Yes	2	59	21	Yes	Yes	7th
Female	3	No	1		18	No		
Female	9	Yes	3	29	28	No		
Female	8	Yes	3	72	24	No		
Female	11	Yes	2	92	24	Yes	Yes	5th
Male	8	Yes	5	240	26	No		
Male	10	No	1		20	Yes	Yes	9th
Female	7	Yes	6	36	32	No		
Female	7	No	1		25	Yes	Yes	10th
Male	8	Yes	5	30	38	No		
Female	8	No	1		28	Yes	Yes	9th
Male	14	No	1		26	Yes	Yes	4th
Female	6	Yes	1		24	Yes	Yes	7th
Male	14	No	1		21	Yes	Yes	4th
Male	5	Yes	4	59	35	No		
Male	6	Yes	2	3	25	Yes	Yes	10th
Male	9	Yes	6	4	25	No		
Male	8	Yes	6	59	36	No		
Male	6	Yes	4	3	34	No		
Female	4	Yes	2	52	25	No		
Female	7	Yes	6	3	32	No		
Female	8	No	1		26	Yes	Yes	10th
Male	3	Yes	5	60	30	No		
Female	12	Yes	2	3	22	No		
Female	4	No	7	3	35	No		
Female	2	Yes	4	5	35	Yes	No	
Male	13	Yes	3	60	27	No		
Male	6	Yes	5	3	30	No		
Female	14	Yes	4	42	25	No		
Male	3	Yes	6	5	35	No		
Male	13	No	1		22	Yes	Yes	4th
Female	8	No	5	3	30	No		
Female	10	Yes	3	2	30	No		
Female	10	Yes	3	36	27	No		
Female	12	Yes	2	59	26	Yes	Yes	3rd
Male	8	Yes	6	3	38	No		
Male	2	Yes	2	34	20	Yes	Yes	5th
Female	8	Yes	2	999	26	No		

For peer review only

Female	6	No	1		21	Yes	Yes	7th
Male	11	Yes	4	3	42	Yes	Yes	10th
Female	12	Yes	2	59	25	No		
Male	6	No	1		22	Yes	Yes	10th
Female	6	Yes	5	48	35	No		
Female	14	Yes	5	7	37	Yes	No	
Female	9	Yes	5	3	35	No		
Male	10	Yes	2	12	32	Yes	Yes	8th
Female	5	No	1		24	Yes	Yes	10th
Male	3	Yes	7	7	30	No		
Female	7	Yes	4	30	30	No		
Male	12	Yes	2	3	35	No		
Female	9	Yes	1		24	Yes	Yes	3rd
Female	6	Yes	5	59	35	No		
Female	13	Yes	2	62	25	Yes	Yes	9th
Male	10	No	1		23	Yes	Yes	8th
Female	14	Yes	5	58	34	No		
Male	5	No	1		23	Yes	Yes	4th
Female	3	Yes	6	36	30	No		
Male	12	Yes	6	3	35	No		
Female	4	No	1		24	Yes	Yes	5th
Male	6	Yes	3	3	40	No		
Female	8	Yes	7	33	30	No		
Female	10	Yes	3	3	25	Yes	Yes	6th
Male	12	Yes	5	36	39	No		
Male	14	Yes	3	3	35	Yes	Yes	6th
Female	5	Yes	5	24	30	No		
Female	10	Yes	6	48	35	Yes	Yes	4th
Female	14	Yes	4	72	38	No		
Female	11	Yes	6	36	36	No		
Female	11	No	2	3	23	Yes	Yes	10th
Female	8	No	1		25	Yes	Yes	4th
Male	5	Yes	2	36	25	Yes	Yes	3rd
Female	8	No	1		20	Yes	Yes	5th
Female	11	Yes	6	36	32	No		
Female	11	No	2	4	27	No		
Male	2	Yes	6	4	38	No		
Male	11	No	1		25	Yes	Yes	University/
Male	8	Yes	3	1	32	Yes	Yes	10th
Female	12	No	1		30	No		
Female	7	Yes	2	36	22	No		
Male	3	Yes	3	36	26	No		
Male	5	Yes	2	24	20	Yes	Yes	7th
Female	12	No	5	5	30	Yes	No	
Female	12	No	1		28	No		
Female	14	Yes	1		25	Yes	Yes	9th
Male	2	No	1		20	No		
Male	3	Yes	4	3	30	No		
Female	8	Yes	7	5	38	No		
Female	12	Yes	2	6	25	Yes	Yes	10th
Female	9	Yes	7	3	40	No		
Male	3	Yes	5	3	33	No		

Female	6	No	2	4	27	No		
Male	12	No	1		19	Yes	Yes	11th
Female	12	Yes	2	30	22	No		
Male	6	Yes	3	144	28	No		
Female	7	Yes	6	40	30	No		
Male	9	No	8	5	32	No		
Male	5	Yes	6	48	35	No		
Female	10	Yes	7	35	37	No		
Female	5	Yes	3	26	24	Yes	Yes	3rd
Male	10	Yes	4	5	27	No		
Female	10	Yes	7	36	39	No		
Female	8	Yes	2	3	25	Yes	Yes	8th
Female	11	No	2	4	25	Yes	Yes	10th
Female	14	No	2	5	28	Yes	Yes	10th
Female	2	Yes	5	3	25	No		
Male	7	No	1		27	Yes	Yes	9th
Female	13	No	5	4	36	No		
Female	9	Yes	3	36	35	No		
Female	3	Yes	10	28	36	No		
Male	5	Yes	3	5	30	No		
Female	14	No	1		23	No		
Male	5	Yes	6	3	38	No		
Male	11	Yes	2	43	21	Yes	Yes	3rd
Male	14	Yes	2	6	32	No		
Female	5	Yes	5	36	35	Yes	Yes	3rd
Male	12	Yes	5	40	28	No		
Female	2	Yes	3	48	27	Yes	Yes	9th
Male	7	Yes	5	5	42	No		
Male	4	Yes	3	72	29	No		
Female	9	Yes	2	48	25	Yes	Yes	3rd
Female	7	Yes	3	9	35	No		
Male	6	Yes	3	51	26	No		
Male	8	Yes	5	50	35	No		
Female	9	Yes	6	4	35	No		
Female	10	Yes	2	25	36	No		
Female	6	Yes	7	36	40	No		
Female	5	Yes	5	47	35	No		
Male	7	Yes	3	4	25	No		
Female	5	No	1		25	Yes	Yes	5th
Female	3	Yes	4	54	35	No		
Male	3	Yes	4	32	38	No		
Female	14	Yes	5	48	35	No		
Male	9	No	1		24	Yes	Yes	4th
Male	11	Yes	4	96	35	No		
Male	5	Yes	7	2	30	No		
Female	3	Yes	5	120	35	No		
Female	4	No	1		25	Yes	Yes	7th
Female	8	Yes	4	120	30	Yes	Yes	2nd
Male	3	No	1		25	No		
Male	3	Yes	2	3	23	Yes	Yes	5th
Female	14	Yes	5	120	35	No		
Female	5	No	1		22	No		

For peer review only

Female	13	No	1		20	Yes	Yes	3rd
Male	2	Yes	4	32	25	No		
Female	5	Yes	3	59	25	No		
Male	4	No	1		20	Yes	Yes	6th
Female	3	Yes	5	36	35	No		
Female	3	Yes	2	120	28	No		
Male	8	No	1		20	Yes	Yes	7th
Male	3	Yes	6	39	35	No		
Female	5	Yes	5	4	35	No		
Female	8	Yes	2	144	36	No		
Female	4	Yes	2	15	28	Yes	Yes	5th
Male	6	Yes	2	72	24	No		
Female	3	Yes	6	28	35	No		
Male	5	Yes	4	3	25	Yes	Yes	5th
Female	11	Yes	5	37	35	No		
Male	3	No	1		20	Yes	Yes	5th
Male	3	Yes	2	36	30	No		
Female	12	Yes	3	130	29	No		
Female	8	No	1		18	Yes	Yes	7th
Female	2	Yes	6	36	40	No		
Female	3	Yes	8	3	35	No		
Female	8	No	1		20	No		
Female	9	Yes	4	2	30	No		
Male	11	Yes	5	47	42	Yes	Yes	4th
Female	5	Yes	2	2	26	Yes	Yes	5th
Male	13	Yes	3	4	30	No		
Male	8	Yes	6	38	33	No		
Female	12	No	1		20	No		
Female	12	Yes	6	48	35	No		
Female	10	No	1		19	Yes	Yes	8th
Male	8	Yes	3	70	24	Yes	Yes	5th
Female	9	Yes	4	81	32	No		
Male	2	Yes	2	49	26	No		
Male	9	Yes	5	36	30	Yes	Yes	5th
Female	8	Yes	4	59	35	No		
Male	12	Yes	4	45	30	No		
Male	6	Yes	2	66	23	Yes	Yes	7th
Female	5	Yes	6	36	40	No		
Male	14	Yes	4	50	25	No		
Male	12	Yes	6	45	35	No		
Male	12	No	1		27	No		
Male	8	Yes	4	8	25	No		
Female	14	Yes	4	51	32	Yes	Yes	5th
Female	4	Yes	2	36	28	No		
Male	14	Yes	2	3	28	No		
Male	3	Yes	7	60	35	No		
Male	4	No	5	30	30	No		
Male	12	Yes	3	79	38	Yes	Yes	3rd
Male	2	Yes	5	59	40	No		
Male	8	Yes	2	3	30	Yes	Yes	University/
Male	8	Yes	6	63	40	No		
Female	10	Yes	2	60	40	No		

For peer review only

Male	6	Yes	7	8	30	No		
Female	11	Yes	10	35	44	No		
Male	12	Yes	4	4	35	No		
Male	12	Yes	3	53	35	Yes	Yes	10th
Female	4	Yes	1		21	Yes	Yes	5th
Male	8	Yes	6	4	43	No		
Male	4	Yes	4	36	35	No		
Female	3	Yes	4	35	29	No		
Female	8	Yes	2	72	30	Yes	Yes	10th
Female	4	Yes	2	48	26	No		
Female	8	No	1		25	No		
Male	9	Yes	3	49	29	No		
Male	13	Yes	3	4	23	Yes	Yes	6th
Male	10	Yes	7	51	40	No		
Male	14	Yes	6	59	40	No		
Male	12	Yes	4	40	35	No		
Male	8	Yes	6	59	30	No		
Female	11	Yes	3	36	28	No		
Female	11	Yes	8	6	46	No		
Female	9	Yes	5	3	30	No		
Male	6	Yes	4	38	32	No		
Female	13	Yes	5	48	40	No		
Male	12	Yes	5	50	38	No		
Female	11	Yes	6	48	35	No		
Male	5	Yes	3	6	38	No		
Female	7	Yes	3	5	38	No		
Female	3	Yes	4	3	30	No		
Female	10	Yes	5	36	35	Yes	Yes	4th
Male	5	Yes	3	59	30	No		
Male	14	Yes	36	5	25	Yes	Yes	3rd
Male	6	Yes	5	3	35	No		
Female	6	Yes	5	22	35	No		
Female	3	Yes	49	88	35	No		
Female	11	Yes	2	72	28	Yes	Yes	9th
Male	3	Yes	5	36	30	No		
Female	3	Yes	6	76	40	No		
Female	11	Yes	6	66	40	No		
Male	13	Yes	2	96	27	Yes	Yes	3rd
Male	4	Yes	3	2	30	No		
Female	10	Yes	2	62	29	Yes	Yes	5th
Female	11	Yes	7	60	38	Yes	No	
Male	3	Yes	2	2	20	No		
Male	10	No	1		24	No		
Male	11	No	1		29	Yes	Yes	9th
Female	2	Yes	2	72	25	Yes	Yes	6th
Female	14	No	1		21	Yes	Yes	3rd
Male	6	No	1		21	Yes	Yes	4th
Male	13	No	1		19	No		
Male	14	Yes	3	8	25	No		
Male	12	Yes	3	4	28	Yes	Yes	8th
Male	12	No	1		28	No		
Female	12	Yes	5	6	33	No		

For peer review only

Male	3	Yes	1		18	Yes	Yes	4th
Male	7	No	1		18	Yes	Yes	7th
Female	4	No	1		16	Yes	Yes	5th
Male	2	Yes	3	81	25	Yes	Yes	7th
Male	13	No	1		35	No		
Male	8	No	1		20	No		
Female	3	Yes	2	72	27	Yes	Yes	8th
Male	3	No	1		18	Yes	Yes	10th
Male	5	Yes	3	48	20	No		
Female	14	Yes	1		20	Yes	Yes	3rd
Female	6	Yes	2	61	21	Yes	Yes	5th
Female	8	No	1		20	Yes	Yes	10th
Female	13	Yes	4	48	35	No		
Female	12	No	1		20	Yes	Yes	4th
Female	10	Yes	3	36	25	No		
Male	13	Yes	2	5	23	Yes	Yes	3rd
Female	2	Yes	6	36	37	No		
Male	3	No	1		22	Yes	Yes	3rd
Male	7	No	1		20	Yes	Yes	7th
Male	11	Yes	5	72	36	Yes	Yes	7th
Female	11	Yes	1		25	Yes	Yes	7th
Female	14	Yes	3	36	35	Yes	Yes	8th
Female	3	Yes	6	84	29	No		
Male	4	Yes	5	72	32	Yes	Yes	5th
Female	8	No	1		21	Yes	Yes	7th
Female	3	Yes	2	60	25	No		
Female	11	No	1		19	Yes	Yes	10th
Male	11	Yes	6	98	35	No		
Female	6	Yes	3	3	30	No		
Female	6	Yes	5	2	36	No		
Female	9	No	1		20	Yes	Yes	4th
Female	14	Yes	4	10	35	No		
Male	2	Yes	3	210	21	Yes	Yes	4th
Female	13	No	1		32	Yes	Yes	7th
Female	2	Yes	3	96	27	No		
Male	5	Yes	3	18	26	Yes	Yes	4th
Female	13	Yes	3	7	29	No		
Female	9	No	1		20	Yes	Yes	7th
Female	12	No	1		19	Yes	Yes	8th
Male	12	Yes	4	120	29	Yes	Yes	3rd
Male	8	Yes	1		20	Yes	Yes	4th
Male	6	Yes	2	38	21	Yes	Yes	7th
Female	9	No	1		18	Yes	Yes	5th
Male	2	Yes	7	84	38	Yes	No	
Female	5	No	1		25	Yes	Yes	8th
Male	8	No	1		19	Yes	Yes	7th
Female	4	Yes	4	48	23	Yes	Yes	4th
Female	5	Yes	3	48	25	No		
Female	12	No	1		19	Yes	Yes	8th
Female	8	Yes	4	4	31	No		
Male	12	Yes	2	72	21	No		
Female	8	No	1		20	Yes	Yes	8th

For peer review only

Female	3	Yes	2	18	34	No		
Male	5	No	1		18	Yes	Yes	5th
Male	4	Yes	2	65	23	Yes	Yes	6th
Male	4	Yes	5	156	36	No		
Male	9	Yes	2	60	21	No		
Female	2	Yes	3	60	29	Yes	Yes	8th
Male	3	Yes	3	108	30	No		
Male	3	No	1		21	Yes	Yes	7th
Female	9	No	1		20	Yes	Yes	6th
Male	14	No	1		21	Yes	Yes	4th
Female	3	Yes	3	150	27	Yes	Yes	3rd
Male	4	Yes	4	14	36	No		
Female	13	Yes	2	60	29	No		
Male	6	No	1		22	No		
Female	4	No	1		20	Yes	Yes	10th
Female	14	Yes	3	72	24	Yes	Yes	9th
Male	3	No	1		19	Yes	Yes	7th
Female	9	No	1		20	Yes	Yes	10th
Male	8	Yes	2	6	28	Yes	Yes	10th
Male	12	Yes	2	61	23	Yes	Yes	8th
Male	12	Yes	5	84	40	No		
Female	8	No	1		19	Yes	Yes	5th
Male	11	No	1		18	Yes	Yes	6th
Male	5	No	1		23	No		
Male	12	Yes	3	60	28	Yes	Yes	7th
Male	5	Yes	3	5	35	Yes	Yes	6th
Male	10	No	1		21	Yes	Yes	8th
Male	12	Yes	6	36	34	No		
Male	2	Yes	7	4	37	No		
Male	3	Yes	3	29	22	No		
Male	2	Yes	6	36	35	Yes	Yes	2nd
Male	9	Yes	4	48	28	No		
Male	2	Yes	2	7	27	Yes	Yes	6th
Male	13	Yes	2	84	25	Yes	Yes	7th
Male	8	Yes	3	72	26	No		
Male	10	No	1		20	No		
Male	4	Yes	2	48	20	Yes	Yes	6th
Female	10	No	1		21	Yes	Yes	8th
Female	14	Yes	3	12	29	No		
Female	5	Yes	2	72	25	Yes	No	
Male	8	No	1		36	Yes	Yes	5th
Female	10	Yes	8	6	40	No		
Male	5	No	1		18	No		
Female	6	Yes	4	132	23	Yes	Yes	9th
Female	9	Yes	2	33	22	Yes	Yes	8th
Male	5	No	1		27	No		
Male	3	Yes	10	54	40	No		
Female	14	No	1		30	No		
Male	6	Yes	4	48	35	No		
Female	11	No	1		28	Yes	Yes	10th
Male	3	Yes	6	3	35	No		
Female	3	Yes	2	48	28	No		

For peer review only

Female	5	Yes	5	4	30	No		
Female	2	Yes	3	8	25	Yes	Yes	9th
Male	14	Yes	7	36	35	No		
Male	5	Yes	2	84	30	No		
Male	4	Yes	6	4	35	No		
Female	14	Yes	5	4	28	No		
Female	12	Yes	5	120	37	No		
Male	4	Yes	8	2	40	No		
Female	6	Yes	9	67	34	No		
Male	9	Yes	6	3	40	No		
Male	14	Yes	6	36	35	No		
Male	14	Yes	2	3	30	No		
Female	13	Yes	4	48	33	No		
Female	4	Yes	7	45	40	No		
Female	7	Yes	3	72	27	Yes	Yes	4th
Male	10	Yes	3	96	30	No		
Female	9	No	1		25	Yes	Yes	6th
Male	2	Yes	5	39	34	No		
Male	7	Yes	4	32	23	Yes	Yes	9th
Female	3	Yes	6	60	40	No		
Female	4	Yes	5	59	29	No		
Male	10	No	1		28	No		
Female	14	Yes	6	72	40	No		
Male	7	Yes	2	70	22	Yes	Yes	8th
Female	14	No	1		23	Yes	Yes	8th
Female	12	Yes	2	88	25	No		
Male	13	Yes	5	36	30	No		
Female	5	Yes	2	48	25	No		
Female	3	Yes	6	47	35	No		
Female	14	Yes	5	41	48	No		
Female	11	Yes	2	78	30	No		
Male	14	No	1		25	Yes	Yes	5th
Female	14	No	1		22	Yes	Yes	8th
Male	4	No	4	96	35	No		
Female	9	Yes	6	60	30	No		
Female	12	Yes	5	41	35	No		
Male	10	Yes	3	108	33	No		
Female	14	Yes	1		20	Yes	Yes	6th
Female	8	No	1		19	Yes	Yes	9th
Female	6	Yes	3	3	30	No		
Female	9	Yes	2	33	22	Yes	Yes	8th
Male	11	Yes	9	48	39	No		
Female	10	No	1		20	Yes	Yes	6th
Female	6	Yes	5	46	30	No		
Male	7	Yes	10	36	42	No		
Male	3	Yes	9	42	43	No		
Female	10	Yes	2	48	22	Yes	Yes	8th
Male	3	Yes	5	50	40	Yes	Yes	7th
Male	5	Yes	4	120	45	No		
Female	5	Yes	2	59	22	Yes	Yes	6th
Female	14	Yes	6	5	36	No		
Male	2	Yes	2	48	25	Yes	Yes	6th

For peer review only

Female	10	Yes	3	3	28	Yes	Yes	4th
Male	12	No	1		25	No		
Male	9	Yes	3	280	33	Yes	Yes	6th
Female	7	Yes	5	45	30	Yes	Yes	4th
Male	8	Yes	3	59	25	No		
Female	6	Yes	3	72	25	Yes	Yes	5th
Male	3	No	1		22	Yes	Yes	7th
Female	6	Yes	3	6	30	No		
Female	14	No	1		23	Yes	Yes	6th
Female	6	Yes	6	24	35	No		
Male	11	Yes	4	4	32	No		
Female	3	Yes	3	27	26	No		
Male	10	Yes	1		19	Yes	Yes	5th
Female	6	Yes	3	24	30	No		
Female	3	No	1		20	Yes	Yes	5th
Male	14	No	1		27	No		
Female	2	Yes	4	2	30	No		
Female	14	Yes	5	36	35	No		
Male	2	Yes	2	48	18	Yes	Yes	8th
Male	13	No	1		22	Yes	Yes	10th
Male	14	Yes	6	72	40	No		
Female	9	Yes	4	40	30	No		
Female	14	Yes	3	120	30	No		
Female	13	Yes	5	4	25	No		
Male	3	Yes	3	36	25	No		
Male	5	Yes	6	59	35	No		
Male	3	Yes	5	36	34	No		
Female	10	Yes	6	20	30	No		
Male	2	Yes	4	120	30	No		
Male	6	Yes	6	36	38	No		
Female	5	No	1		19	No		
Male	9	Yes	5	108	30	No		
Male	10	Yes	1		20	Yes	Yes	8th
Female	6	Yes	4	48	35	No		
Female	10	Yes	5	4	30	Yes	Yes	7th
Female	4	Yes	1		38	No		
Female	11	Yes	4	36	35	Yes	Yes	7th
Male	12	Yes	3	46	22	No		
Male	12	Yes	6	14	30	No		
Female	4	Yes	5	3	30	No		
Female	8	Yes	2	96	28	No		
Female	6	Yes	7	96	40	No		
Female	13	Yes	2	36	23	No		
Male	12	Yes	7	36	35	No		
Female	12	No	1		24	Yes	Yes	University/
Male	4	Yes	2	3	22	Yes	Yes	9th
Female	8	Yes	4	6	25	No		
Male	14	Yes	2	60	25	Yes	Yes	6th
Male	11	Yes	7	3	33	No		
Male	3	No	1		21	Yes	Yes	10th
Female	3	Yes	5	59	35	No		
Female	12	Yes	8	38	40	No		

For peer review only

Female	9	No	5	45	28	No		
Male	3	Yes	6	46	42	No		
Male	8	No	1		20	No		
Female	12	Yes	4	12	30	No		
Male	11	Yes	5	3	35	No		
Male	11	Yes	5	59	38	No		
Female	14	Yes	5	48	30	Yes	Yes	2nd
Female	13	No	1		22	Yes	Yes	8th
Female	12	Yes	3	96	30	No		
Female	4	Yes	2	3	18	Yes	Yes	8th
Male	3	Yes	4	120	36	No		
Male	11	Yes	4	59	29	No		
Male	3	Yes	3	72	35	Yes	Yes	4th
Male	14	Yes	2	74	32	No		
Female	11	Yes	3	48	28	Yes	Yes	9th
Female	11	Yes	4	48	26	Yes	Yes	7th
Male	5	Yes	3	13	34	Yes	No	
Female	2	Yes	7	36	42	No		
Male	11	Yes	4	36	30	No		
Male	11	Yes	2	156	27	No		
Male	9	Yes	6	40	36	Yes	Yes	3rd
Female	3	Yes	4	48	27	No		
Female	9	Yes	5	8	27	No		
Male	11	Yes	6	72	35	No		
Female	3	Yes	6	31	35	No		
Female	14	Yes	2	72	26	No		
Male	7	Yes	2	31	25	Yes	Yes	6th
Female	8	Yes	6	85	35	No		
Female	3	Yes	3	144	28	Yes	Yes	3rd
Female	9	Yes	2	70	33	No		
Female	13	No	1		24	Yes	Yes	8th
Male	13	No	1		24	Yes	Yes	9th
Male	12	Yes	2	52	20	No		
Male	11	Yes	6	5	40	No		
Female	8	Yes	4	72	38	No		
Female	13	No	1		20	Yes	Yes	10th
Female	3	No	1		25	No		
Male	3	Yes	3	72	25	No		
Male	9	Yes	2	120	25	No		
Male	11	Yes	7	59	42	No		
Female	4	Yes	8	2	30	No		
Female	7	No	1		25	No		
Male	12	Yes	4	36	25	Yes	Yes	5th
Male	12	Yes	2	156	28	No		
Male	10	Yes	5	40	30	No		
Female	2	Yes	6	4	38	No		
Female	3	Yes	9	32	40	No		
Female	11		4	36	28	No		
Male	9	Yes	2	6	30	No		
Male	11	Yes	2	60	25	No		
Male	8	Yes	6	5	30	No		
Female	12	Yes	7	40	40	No		

For peer review only

Female	6	Yes	6	60	35	No		
Female	11	Yes	4	142	30	No		
Male	10	No	1		21	No		
Female	12	No	1		26	Yes	Yes	University/
Female	6	Yes	2	5	25	No		
Male	2	Yes	8	28	38	No		
Male	14	Yes	3	85	35	No		
Female	11	Yes	7	48	45	No		
Female	11	No	1		20	No		
Male	2	Yes	4	6	27	No		
Male	2	Yes	4	35	27	No		
Female	5	No	1		20	No		
Female	6	Yes	2	5	20	No		
Male	11	Yes	6	36	30	No		
Female	3	Yes	4	2	30	Yes	Yes	4th
Female	5	No	1		26	No		
Female	3	Yes	2	60	28	No		
Male	14	Yes	4	5	35	No		
Male	2	Yes	2	36	30	No		
Male	6	No	1		25	Yes	Yes	8th
Male	13	Yes	2	6	30	Yes	Yes	12th
Male	9	Yes	4	48	28	No		
Female	3	Yes	4	48	30	Yes	Yes	2nd
Male	8	Yes	1		27	No		
Female	11	Yes	2	9	30	No		
Female	3	No	1		22	No		
Female	6	Yes	5	50	36	No		
Male	9	Yes	2	8	25	Yes	Yes	7th
Female	6	Yes	3	6	30	No		
Male	3	Yes	5	83	35	Yes	Yes	3rd
Female	8	Yes	5	40	28	No		
Female	6	Yes	5	36	30	Yes	No	
Female	2	No	1		22	Yes	Yes	8th
Female	6	Yes	5	3	30	No		
Female	13	Yes	3	8	28	No		
Male	3	Yes	2	50	22	Yes	Yes	8th
Female	14	Yes	6	130	40	Yes	Yes	6th
Male	13	Yes	2	59	20	Yes	Yes	3rd
Female	8	Yes	6	3	25	No		
Female	3	No	1		25	No		
Female	14	Yes	4	8	24	No		
Female	5	Yes	8	5	30	No		
Female	9	Yes	3	48	25	Yes	Yes	6th
Female	3	Yes	2	36	20	No		
Female	5	No	1		27	Yes	Yes	8th
Female	4	Yes	6	36	38	No		
Female	8	No	1		20	Yes	Yes	4th
Female	9	Yes	2	87	24	Yes	Yes	9th
Male	3	Yes	2	18	30	No		
Female	3	Yes	6	31	36	No		
Male	10	Yes	6	48	40	No		
Female	10	Yes	5	47	37	No		

For peer review only

Female	10	Yes	5	59	35	No		
Male	6	Yes	3	3	30	No		
Female	3	Yes	2	4	26	Yes	Yes	2nd
Male	12	Yes	6	60	34	Yes	Yes	6th
Male	4	No	1		26	Yes	Yes	University/
Female	8	Yes	6	36	35	No		
Male	4	Yes	2	120	27	No		
Male	3	Yes	2	2	25	Yes	Yes	7th
Male	6	Yes	4	59	35	Yes	Yes	3rd
Male	8	Yes	5	36	27	No		
Female	12	Yes	2	12	30	No		
Male	9	Yes	2	59	24	Yes	Yes	4th
Female	6	Yes	7	3	30	No		
Male	14	Yes	5	36	35	No		
Male	12	Yes	3	36	28	No		
Male	14	Yes	6	78	40	Yes	No	
Female	13	No	1		20	No		
Female	9	No	1		25	No		
Female	3	Yes	3	36	25	Yes	Yes	9th
Female	5	Yes	4	46	35	No		
Male	14	Yes	2	70	22	Yes	Yes	6th
Female	11	Yes	2	59	20	No		
Female	8	Yes	4	39	35	Yes	Yes	6th
Male	9	Yes	4	3	25	No		
Female	3	Yes	8	96	45	No		
Male	14	Yes	7	42	35	No		
Female	10	Yes	6	106	30	No		
Male	8	Yes	2	60	25	Yes	Yes	6th
Male	4	No	1		20	Yes	Yes	8th
Female	4	Yes	2	72	21	Yes	Yes	8th
Male	9	Yes	6	6	35	No		
Male	6	Yes	2	4	22	Yes	Yes	5th
Female	5	Yes	3	5	25	No		
Female	6	Yes	2	6	23	No		
Female	6	Yes	3	5	25	No		
Male	14	Yes	2	54	26	No		
Female	14	Yes	2	7	25	Yes	Yes	9th
Female	12	No	1		20	Yes	Yes	8th
Female	7	Yes	5	48	35	No		
Male	2	Yes	6	3	38	No		
Female	14	Yes	6	36	35	Yes	No	
Female	6	No	1		35	No		
Female	12	No	1		18	Yes	Yes	8th
Female	12	No	1		18	Yes	Yes	7th
Female	12	Yes	3	82	28	Yes	Yes	10th
Male	12	No	1		23	Yes	Yes	8th
Female	6	No	1		18	No		
Female	4	No	1		20	Yes	Yes	3rd
Male	6	Yes	5	48	35	No		
Female	7	Yes	5	3	30	No		
Female	2	Yes	3	5	25	No		
Male	5	Yes	5	3	25	No		

For peer review only

Female	14	Yes	2	3	25	No		
Male	2	Yes	5	5	35	No		
Female	8	Yes	4	4	35	No		
Male	9	Yes	5	2	30	No		
Female	6	Yes	2	3	27	Yes	Yes	10th
Male	10	No	1		20	Yes	Yes	6th
Female	6	Yes	1		25	Yes	Yes	8th
Male	3	Yes	3	2	30	No		
Male	14	Yes	5	50	30	No		
Female	10	Yes	8	3	44	No		
Male	12	Yes	1		18	Yes	Yes	5th
Female	6	Yes	3	54	28	No		
Female	6	Yes	5	3	35	No		
Male	10	Yes	7	50	32	No		
Female	4	Yes	6	46	36	No		
Female	9	Yes	4	3	35	No		
Female	14	Yes	4	3	30	No		
Male	9	Yes	4	3	20	No		
Male	14	Yes	1		24	Yes	Yes	10th
Female	2	Yes	2	3	24	Yes	Yes	6th
Female	2	Yes	5	52	34	No		
Male	10	Yes	5	4	30	No		
		Yes	2	3	38	No		
Male	12	Yes	5	4	35	No		
Female	13	Yes	5	4	35	No		
Male	10	Yes	7	29	32	No		
Male	8	Yes	5	36	31	No		
Female	3	Yes	6	3	38	No		
Male	12	Yes	7	3	35	No		
Female	10	Yes	5	2	30	No		
Male	10	Yes	6	70	41	No		
Female	14	Yes	1		21	Yes	Yes	10th
Female	11	Yes	6	2	40	No		
Female	13	Yes	3	3	27	No		
Female	14	Yes	2	9	24	No		
Male	6	Yes	6	3	30	No		
Male	8	Yes	5	4	38	No		
Male	4	Yes	6	3	38	No		
Male	7	Yes	6	3	30	No		
Female	6	Yes	2	4	23	No		
Male	3	Yes	5	3	30	No		
Male	6	Yes	3	4	28	Yes	Yes	9th
Male	10	No	1		18	Yes	Yes	7th
Male	2	Yes	4	4	35	No		
Male	7	Yes	2	5	20	Yes	Yes	7th
Male	4	Yes	5	2	27	No		
Male	5	Yes	6	84	40	No		
Male	6	Yes	2	37	28	No		
Female	13	Yes	3	3	22	No		
Female	14	No	1		20	Yes	Yes	9th
Female	4	No	1		24	No		
Female	8	Yes	5	5	30	Yes	Yes	4th

For peer review only

Male	7	Yes	6	4	40	No		
Female	5	Yes	2	2	32	Yes	Yes	9th
Female	6	Yes	5	4	30	No		
Female	14	No	1		25	Yes	Yes	9th
Female	13	Yes	8	36	40	No		
Female	12	Yes	3	4	35	No		
Male	11	Yes	6	6	33	No		
Female	11	Yes	10	3	42	No		
Male	4	Yes	6	3	30	No		
Male	7	Yes	8	40	38	No		
Male	8	Yes	3	4	20	No		
Male	9	Yes	2	15	30	No		
Female	14	Yes	7	5	40	No		
Female	7	Yes	3	6	29	No		
Male	8	Yes	4	4	28	No		
Female	14	Yes	7	3	30	No		
Male	5	Yes	5	48	29	Yes	Yes	4th
Male	9	Yes	5	35	36	No		
Female	12	Yes	2	3	28	No		
Male	12	Yes	4	4	35	No		
Male	7	No	1		25	No		
Female	6	Yes	2	4	24	No		
Male	14	Yes	3	5	30	Yes	Yes	7th
Female	14	Yes	5	9	39	No		
Male	6	Yes	5	3	36	No		
Male	6	Yes	3	4	33	No		
Female	9	Yes	3	7	45	Yes	No	
Male	8	Yes	4	58	38	No		
Male	10	No	1		25	Yes	Yes	10th
Female	8	Yes	3	2	26	No		
Female	14	Yes	3	4	35	Yes	Yes	4th
Male	10	Yes	4	5	30	No		
Female	14	Yes	6	4	35	No		
Female	4	Yes	2	60	25	No		
Male	6	Yes	5	2	36	No		
Female	14	Yes	4	4	30	No		
Female	8	Yes	6	2	35	No		
Male	5	Yes	5	36	38	No		
Female	12	Yes	2	5	35	No		
Male	5	Yes	3	3	30	No		
Male	8	Yes	6	3	40	No		
Female	4	No	1		24	Yes	Yes	10th
Female	12	No	1		22	Yes	Yes	8th
Female	7	Yes	6	2	30	No		
Male	4	Yes	3	3	25	No		
Female	14	Yes	3	4	25	Yes	Yes	5th
Male	7	Yes	2	5	25	Yes	Yes	3rd
Male	3	Yes	6	3	35	No		
Female	5	No	1		22	Yes	Yes	3rd
Female	4	No	1		20	No		
Male	11	Yes	7	3	35	No		
Female	7	No	1		19	Yes	Yes	8th

For peer review only

Female	7	Yes	3	2	27	No		
Female	13	Yes	2	5	30	No		
Male	13	Yes	3	3	28	No		
Female	8	No	1		22	No		
Female	11	Yes	6	2	30	No		
Female	10	Yes	3	48	30	No		
Female	7	Yes	5	2	30	No		
Male	10	Yes	8	3	36	No		
Female	9	Yes	4	5	30	No		
Female	3	Yes	2	5	23	Yes	Yes	6th
Male	14	Yes	1		22	No		
Female	12	No	1		19	Yes	Yes	3rd
Female	12	Yes	5	4	33	No		
Male	4	No	1		25	Yes	Yes	4th
Female	3	Yes	1		22	Yes	Yes	10th
Male	14	Yes	1		24	Yes	Yes	9th
Female	14	No	1		20	Yes	Yes	6th
Male	4	Yes	4	24	28	Yes	Yes	3rd
Female	11	Yes	2	33	23	Yes	Yes	10th
Male	4	Yes	2	96	26	Yes	Yes	6th
Male	14	No	1		18	Yes	Yes	10th
Male	14	No	1		26	Yes	Yes	10th
Female	6	Yes	3	9	30	No		
Female	3	Yes	4	80	30	No		
Male	5	Yes	5	120	39	No		
Male	12	Yes	3	6	37	No		
Male	14	Yes	2	60	26	No		
Female	14	Yes	2	8	26	No		
Female	7	Yes	3	6	32	Yes	Yes	8th
Male	13	Yes	4	9	33	Yes	Yes	7th
Female	13	Yes	2	60	36	No		
Female	4	Yes	2	72	24	Yes	Yes	6th
Female	12	Yes	3	124	36	No		
Male	4	No	1		25	Yes	Yes	9th
Female	9	No	1		20	Yes	Yes	4th
Male	9	No	1		22	Yes	Yes	10th
Female	5	Yes	4	59	26	No		
Male	10	No	1		27	Yes	Yes	10th
Male	6	Yes	5	5	38	No		
Male	5	Yes	6	75	35	No		
Male	9	No	1		20	Yes	Yes	3rd
Male	14	Yes	3	96	28	No		
Female	9	Yes	3	144	35	No		
Female	10	Yes	4	120	38	No		
Male	3	Yes	1		21	Yes	Yes	6th
Male	9	Yes	3	120	28	No		
Male	14	No	1		21	Yes	Yes	8th
Male	3	Yes	4	28	36	No		
Female	14	Yes	2	62	27	Yes	Yes	3rd
Male	5	No	1		24	No		
Male	7	Yes	2	7	32	No		
Female	13	Yes	2	4	26	Yes	Yes	4th

For peer review only

Female	11	No	1		19	Yes	Yes	7th
Male	14	No	1		20	Yes	Yes	9th
Male	12	Yes	1		21	Yes	Yes	9th
Female	12	Yes	2	109	26	Yes	Yes	8th
Male	8	No	1		19	Yes	Yes	9th
Male	14	Yes	1		18	No		
Female	10	Yes	3	36	28	Yes	Yes	7th
Male	14	Yes	2	60	26	Yes	Yes	8th
Female	3	Yes	2	96	26	Yes	Yes	7th
Male	14	Yes	2	2	30	No		
Female	8	Yes	5	3	30	No		
Female	14	Yes	2	8	28	Yes	Yes	4th
Female	12	Yes	2	60	20	Yes	Yes	5th
Male	9	No	1		35	Yes	Yes	5th
Male	13	Yes	4	168	35	Yes	Yes	4th
Female	9	Yes	3	4	22	Yes	Yes	8th
Female	7	Yes	2	68	25	Yes	Yes	5th
Female	11	Yes	1		20	Yes	Yes	5th
Male	12	No	1		20	Yes	Yes	5th
Male	2	Yes	4	72	28	Yes	Yes	5th
Female	6	Yes	2	60	20	Yes	Yes	10th
Male	14	Yes	4	11	35	No		
Female	11	No	1		29	Yes	Yes	5th
Female	10	Yes	2	14	35	Yes	Yes	9th
Female	9	Yes	3	108	34	Yes	Yes	3rd
Male	14	Yes	2	84	25	No		
Male	6	Yes	4	128	35	No		
Male	10	No	1		19	Yes	Yes	5th
Female	9	No	1		20	Yes	Yes	3rd
Female	7	Yes	2	5	25	Yes	Yes	10th
Female	12	Yes	7	36	33	No		
Female	14	No	1		20	Yes	Yes	3rd
Male	3	Yes	2	60	28	Yes	Yes	5th
Female	14	No	1		22	Yes	Yes	5th
Male	12	No	1		20	Yes	Yes	4th
Female	9	Yes	3	96	32	No		
Male	4	Yes	4	7	36	No		
Male	4	No	1		25	Yes	Yes	10th
Female	2	Yes	2	8	28	Yes	Yes	5th
Male	14	Yes	3	120	31	No		
Male	3	No	1		23	Yes	Yes	8th
Male	6	No	1		21	Yes	Yes	8th
Male	7	Yes	3	4	33	Yes	Yes	Technical/\
Female	12	Yes	2	76	29	Yes	Yes	7th
Male	3	Yes	3	14	30	Yes	No	
Female	13	No	1		20	Yes	Yes	8th
Male	9	No	1		26	Yes	Yes	10th
Male	6	Yes	5	60	35	No		
Male	7	Yes	2	66	27	Yes	Yes	8th
Female	9	No	1		22	Yes	Yes	7th
Male	12	Yes	4	78	30	Yes	Yes	3rd
Female	5	No	1		18	Yes	Yes	9th

For peer review only

Male	12	Yes	2	96	24	No		
Female	7	Yes	5	60	37	No		
Male	10	Yes	2	60	25	Yes	Yes	10th
Male	7	No	1		25	Yes	Yes	12th
Female	9	No	1		23	Yes	Yes	10th
Male	6	No	1		28	Yes	Yes	7th
Male	5	Yes	5	6	38	No		
Male	5	Yes	3	80	28	Yes	Yes	9th
Female	8	No	1		23	Yes	Yes	8th
Female	5	Yes	1		20	No		
Male	7	Yes	2	48	23	Yes	Yes	9th
Male	14	No	1		28	Yes	Yes	4th
Male	4	No	1		19	Yes	Yes	3rd
Female	8	No	2	2	24	Yes	Yes	Technical/\
Female	6	Yes	2	5	29	No		
Male	13	No	1		27	Yes	Yes	7th
Male	5	Yes	1		25	No		
Female	14	No	1		25	Yes	Yes	4th
Female	10	Yes	2	5	29	Yes	Yes	7th
Female	14	Yes	3	7	38	Yes	Yes	8th
Female	11	Yes	5	140	37	Yes	Yes	2nd
Female	14	Yes	4	135	37	No		
Female	14	Yes	4	96	26	No		
Female	14	Yes	2	5	24	Yes	Yes	10th
Female	3	Yes	2	48	23	No		
Male	5	No	1		19	Yes	Yes	10th
Male	5	Yes	2	71	25	Yes	Yes	9th
Female	5	Yes	2	84	26	Yes	Yes	University/
Female	13	Yes	3	6	27	No		
Male	14	Yes	2	4	24	Yes	Yes	7th
Male	13	Yes	2	72	28	Yes	Yes	5th
Female	7	Yes	2	7	33	Yes	Yes	6th
Female	5	No	1		22	Yes	Yes	8th
Female	12	No	1		34	No		
Male	2	Yes	3	98	31	No		
Male	14	Yes	1		24	Yes	Yes	10th
Female	8	Yes	6	120	35	No		
Female	11	Yes	2	6	29	Yes	Yes	5th
Female	3	Yes	3	3	24	Yes	Yes	6th
Male	3	Yes	3	2	25	Yes	Yes	4th
Female	7	Yes	7	5	37	No		
Male	8	No	1		20	Yes	Yes	5th
Male	6	No	1		21	Yes	Yes	7th
Male	3	Yes	2	7	25	Yes	Yes	7th
Male	14	Yes	2	8	22	Yes	Yes	10th
Female	14	No	1		20	Yes	Yes	9th
Male	10	Yes	2	144	26	No		
Female	2	Yes	2	92	28	Yes	Yes	6th
Female	14	No	1		23	Yes	Yes	12th
Male	10	Yes	3	8	27	Yes	Yes	3rd
Female	13	No	1		28	No		
Male	14	No	1		20	No		

For peer review only

Male	12	Yes	6	96	45	No		
Female	14	No	1		25	Yes	Yes	10th
Male	6	Yes	3	42	35	No		
Female	6	Yes	5	72	35	No		
Female	9	Yes	3	12	40	Yes	Yes	3rd
Female	14	No	1		21	Yes	Yes	8th
Female	3	Yes	3	9	35	Yes	Yes	3rd
Male	14	Yes	2	120	30	Yes	Yes	5th
Male	7	No	1		25	Yes	Yes	12th
Female	13	Yes	6	144	42	No		
Male	14	Yes	4	15	36	Yes	No	
Male	14	Yes	5	10	40	No		
Female	11	No	1		20	Yes	Yes	4th
Male	10	Yes	2	38	23	Yes	Yes	4th
Female	6	Yes	1		19	Yes	Yes	9th
Male	12	Yes	4	120	34	No		
Female	9	Yes	2	9	24	Yes	Yes	5th
Female	5	Yes	2	72	22	No		
Female	7	Yes	2	36	22	No		
Male	12	Yes	2	360	28	Yes	Yes	10th
Female	3	No	1		21	Yes	Yes	9th
Male	11	Yes	1		30	Yes	Yes	4th
Female	2	Yes	3	3	24	Yes	Yes	6th
Female	4	No	1		26	Yes	Yes	10th
Male	5	Yes	1		20	No		
Female	7	Yes	3	60	26	Yes	Yes	3rd
Male	3	Yes	3	96	30	Yes	Yes	5th
Female	7	Yes	2	84	25	Yes	Yes	8th
Female	13	Yes	3	1	36	Yes	No	
Male	13	Yes	1		25	No		
Male	9	Yes	4	4	37	No		
Female	9	Yes	2	11	36	No		
Female	5	Yes	2	98	30	Yes	Yes	7th
Male	4	No	1		20	Yes	Yes	5th
Male	7	No	1		20	Yes	Yes	4th
Female	14	Yes	4	7	39	No		
Male	3	Yes	1		24	Yes	Yes	10th
Male	10	Yes	3	48	32	No		
Female	10	Yes	8	22	43	No		
Female	9	Yes	4	60	30	No		
Female	7	No	3	42	32	No		
Female	12	Yes	6	44	37	No		
Male	4	No	1		20	No		
Female	14	Yes	2	45	28	Yes	Yes	10th
Female	4	Yes	5	48	36	No		
Female	5	No	1		25	Yes	Yes	7th
Male	3	No	1		23	No		
Female	3	Yes	3	72	30	No		
Male	3		2	35	23	No		
Female	13	Yes	6	48	35	No		
Female	14	Yes	5	120	37	No		
Male	3	Yes	3	120	25	Yes	Yes	6th

For peer review only

Male	3	Yes	2	36	23	No		
Female	12	Yes	3	999	35	No		
Female	14	Yes	2	34	24	No		
Female	7	No	1		20	No		
Male	10	Yes	3	38	35	No		
Female	11	Yes	7	48	41	No		
Male	8	No	1		25	Yes	Yes	3rd
Male	12	Yes	4	59	38	No		
Female	10	Yes	4	60	34	Yes	Yes	3rd
Male	13	Yes	3	28	33	No		
Female	3	No	1		20	Yes	Yes	5th
Male	12	Yes	4	59	36	No		
Male	13	Yes	3	48	28	No		
Male	4	Yes	6	24	35	No		
Male	9	Yes	5	59	38	No		
Female	13	Yes	3	999	35	No		
Male	12	Yes	3	59	25	No		
Male	9	Yes	8	59	45	Yes	Yes	2nd
Male	12	Yes	2	41	36	Yes	Yes	5th
Male	11	Yes	3	48	25	No		
Male	7	Yes	3	32	28	Yes	Yes	10th
Female	7	Yes	6	48	32	No		
Male	8	Yes	4	59	26	No		
Male	11	Yes	1		21	No		
Female	3	Yes	4	46	30	No		
Male	9	Yes	2	60	23	Yes	Yes	6th
Male	5	No	1		23	Yes	Yes	5th
Female	6	Yes	1		24	No		
Female	6	No	1		20	Yes	Yes	4th
Male	5	Yes	4	36	28	No		
Female	7	Yes	2	39	25	No		
Female	13	Yes	4	41	35	No		
Male	9	Yes	5	56	30	No		
Female	11	Yes	6	36	40	Yes	Yes	3rd
Male	11	Yes	3	36	28	Yes	Yes	4th
Male	12	No	1		18	Yes	Yes	7th
Female	12	Yes	5	59	28	No		
Female	4	Yes	4	48	28	Yes	Yes	5th
Male	12	No	1		19	Yes	Yes	6th
Female	12	Yes	4	72	30	No		
Female	13	Yes	3	36	25	Yes	Yes	6th
Female	12	Yes	2	59	25	Yes	Yes	4th
Female	11	Yes	4	41	35	No		
Male	12	Yes	2	999	35	No		
Female	6	Yes	2	72	25	No		
Male	7	Yes	9	999	45	Yes	No	
Female	2	Yes	2	48	20	No		
Male	3	No	1		23	No		
Female	12	No	4	4	44	No		
Female	12	Yes	7	48	40	No		
Male	10	No	1		22	No		
Male	3	No	1		19	Yes	Yes	9th

For peer review only

Male	4	Yes	1		23	Yes	Yes	9th
Male	5	Yes	2	999	27	Yes	Yes	10th
Female	12	No	2	7	30	Yes	Yes	5th
Male	2	No	1		20	Yes	Yes	3rd
Female	11	Yes	5	59	35	No		
Male	14	No	2	8	26	Yes	Yes	10th
Female	6	Yes	3	72	30	Yes	Yes	8th
Female	14	Yes	4	36	35	No		
Male	12	Yes	4	3	38	No		
Male	9	No	2	6	27	Yes	Yes	University/
Female	9	Yes	1		25	Yes	Yes	9th
Female	8	Yes	3	72	30	No		
Female	4	Yes	3	36	35	No		
Female	11	No	1		22	Yes	Yes	8th
Male	13	Yes	2	13	29	No		
Male	12	Yes	2	60	28	Yes	Yes	10th
Female	4	Yes	2	48	29	Yes	Yes	10th
Female	10	Yes	4	36	30	Yes	No	
Male	3	No	5	15	35	Yes	Yes	11th
Male	13	Yes	1		30	Yes	Yes	9th
Female	3	Yes	6	32	35	No		
Male	4	Yes	3	999	30	Yes	Yes	6th
Female	10	Yes	3	4	28	Yes	Yes	7th
		Yes	1		23	Yes	Yes	10th
Female	8	Yes	5	48	35	No		
Female	11	No	4	3	32	No		
Female	4	No	1		22	Yes	Yes	6th
Female	14	No	5	6	40	No		
Male	4	No	3	7	28	No		
Female	9	No	3	9	32	No		
Male	7	No	1		24	Yes	Yes	9th
Female	11	No	1		19	No		
Female	12	No	1		22	Yes	Yes	10th
Female	2	Yes	5	4	30	No		
Male	8	No	3	30	28	Yes	Yes	8th
Male	8	No	1		24	Yes	Yes	10th
Male	3	No	1		24	No		
Male	12	Yes	2	50	28	Yes	Yes	10th
Male	5	Yes	2	48	27	Yes	Yes	4th
Female	14	No	1		20	Yes	Yes	8th
Female	13	Yes	4	36	37	No		
Female	6	Yes	4	47	35	No		
Female	3	No	3	8	28	No		
Female	5	Yes	4	2	28	Yes	Yes	10th
Male	8	Yes	1		22	No		
Female	14	Yes	2	72	25	Yes	Yes	4th
Female	12	Yes	1		22	Yes	Yes	6th
Female	10	No	2	3	27	Yes	Yes	Technical/\
Male	9	Yes	2	34	25	Yes	Yes	9th
Female	14	No	2	3	25	No		
Female	11	No	1		24	Yes	Yes	10th
Female	6	Yes	5	59	30	Yes	Yes	2nd

For peer review only

Male	6	Yes	7	29	38	No		
Female	13	No	1		25	Yes	Yes	10th
Male	5	Yes	2	40	27	No		
Female	9	Yes	3	3	25	Yes	No	
Female	6	Yes	2	36	28	Yes	Yes	10th
Female	11	Yes	4	999	30	No		
Male	2	Yes	3	15	30	Yes	Yes	1st
Male	5	Yes	2	48	26	Yes	Yes	7th
Male	3	Yes	3	59	28	No		
Female	11	Yes	9	48	40	Yes	Yes	2nd
Male	7	No	2	5	25	Yes	Yes	4th
Female	8	Yes	1		24	Yes	Yes	6th
Male	3	Yes	3	4	27	No		
Female	4	Yes	3	36	23	Yes	Yes	4th
Female	3	Yes	6	3	35	No		
Male	6	Yes	6	36	37	No		
Male	3	Yes	4	48	36	Yes	Yes	9th
Male	8	No	1		18	Yes	Yes	8th
Female	10	No	1		23	Yes	Yes	University/
Female	14	Yes	2	84	30	No		
Female	7	Yes	4	48	36	No		
Female	8	Yes	3	4	30	Yes	Yes	8th
Male	7	No	1		23	Yes	Yes	4th
Male	3	Yes	2	48	25	Yes	Yes	8th
Female	6	Yes	4	62	34	Yes	Yes	1st
Male	10	No	5	4	40	No		
Male	13	Yes	3	72	35	No		
Male	7	Yes	2	42	27	Yes	Yes	9th
Female	14	Yes	2	59	30	No		
Male	12	Yes	1		28	No		
Female	8	No	1		24	Yes	Yes	4th
Male	14	Yes	2	60	24	Yes	Yes	6th
Male	14	Yes	2	3	24	Yes	Yes	5th
Female	3	Yes	4	120	32	Yes	Yes	9th
Female	7	Yes	3	42	28	Yes	Yes	9th
Male	3	No	1		25	Yes	Yes	4th
Female	13	No	1		24	Yes	Yes	6th
Male	7	Yes	1		21	Yes	Yes	5th
Male	13	No	1		25	Yes	Yes	4th
Male	14	Yes	1		24	Yes	Yes	7th
Male	12	Yes	4	36	35	No		
Female	6	Yes	8	32	38	Yes	No	
Male	14	Yes	2	48	24	Yes	Yes	10th
Female	3	Yes	2	26	33	No		
Female	11	Yes	2	71	26	Yes	No	
Male	6	Yes	5	72	33	No		
Male	3	No	1		27	Yes	Yes	10th
Female	7	Yes	2	39	25	Yes	Yes	9th
Male	11	Yes	1		25	No		
Male	8	Yes	3	108	30	No		
Male	6	Yes	2	0	22	Yes	Yes	5th
Female	8	No	1		25	Yes	Yes	9th

For peer review only

Female	7	Yes	5	59	30	Yes	Yes	4th
Male	5	Yes	2	36	22	Yes	Yes	5th
Male	2	Yes	2	59	23	Yes	Yes	10th
Male	5	Yes	8	40	35	No		
Female	12	Yes	4	36	30	No		
Male	5	Yes	6	40	33	No		
Female	3	No	1		23	Yes	Yes	3rd
Female	13	No	1		24	Yes	Yes	10th
Male	13	Yes	2	108	30	Yes	Yes	4th
Female	11	Yes	2	38	30	No		
Male	4	Yes	4	59	28	Yes	Yes	1st
Male	6	Yes	2	2	22	Yes	Yes	9th
Male	14	Yes	5	4	38	No		
Female	10	No	1		20	No		
Male	10	No	1		33	No		
Female	3	No	1		26	Yes	Yes	9th
Male	9	Yes	2	60	28	No		
Female	4	Yes	2	40	22	Yes	Yes	10th
Male	4	No	2	5	28	Yes	Yes	9th
Male	2	Yes	5	5	35	No		
Female	11	No	1		20	No		
Female	4	Yes	2	72	26	Yes	Yes	10th
Male	13	No	3	12	28	No		
Male	14	Yes	1		25	Yes	Yes	10th
Female	12	Yes	5	48	38	No		
Female	3	No	3	3	30	Yes	Yes	9th
Male	3	No	1		25	Yes	Yes	9th
Female	10	Yes	6	48	38	No		
Female	10	Yes	2	240	38	Yes	Yes	4th
Female	14	No	1		26	Yes	Yes	10th
Male	12	Yes	2	999	33	Yes	Yes	10th
Female	5	Yes	1		17	Yes	Yes	5th
Female	8	Yes	3	13	35	Yes	Yes	10th
Male	3	Yes	2	108	22	Yes	Yes	9th
Female	12	Yes	2	48	28	Yes	Yes	7th
Female	5	Yes	2	60	26	Yes	Yes	8th
Female	5	Yes	5	8	39	Yes	Yes	7th
Female	3	Yes	5	7	35	No		
Female	3	Yes	2	66	35	No		
Male	3	Yes	4	7	38	Yes	No	
Male	3	Yes	1		23	Yes	Yes	10th
Male	5	No	1		23	Yes	Yes	4th
Male	10	No	1		18	Yes	Yes	9th
Female	5	No	1		20	Yes	Yes	8th
Male	3	Yes	3	8	29	No		
Male	3	Yes	2	6	25	Yes	Yes	10th
Female	6	Yes	1		35	Yes	Yes	8th
Female	14	Yes	2	6	35	No		
Female	13	Yes	2	144	33	No		
Male	4	Yes	3	96	28	Yes	Yes	6th
Female	13	Yes	3	18	37	No		
Female	9	Yes	3	6	25	Yes	Yes	5th

For peer review only

Male	6	Yes	3	122	28	Yes	Yes	5th
Male	4	No	3	78	34	No		
Male	14	Yes	3	8	30	No		
Male	5	Yes	2	19	23	Yes	Yes	Technical/\
Male	14	Yes	4	8	24	No		
Female	9	No	1		19	No		
Male	4	No	1		28	No		
Male	14	Yes	2	6	23	Yes	Yes	9th
Female	10	No	1		20	Yes	Yes	8th
Female	14	No	1		19	No		
Female	7	Yes	6	18	41	No		
Male	2	Yes	2	46	26	Yes	Yes	10th
Female	10	No	1		20	No		
Female	2	Yes	3	121	36	No		
Male	3	Yes	6	12	38	No		
Male	7	Yes	3	120	32	Yes	Yes	6th
Female	10	Yes	2	59	28	Yes	Yes	8th
Female	4	Yes	2	9	22	No		
Male	7	No	1		28	Yes	Yes	University/
Female	4	Yes	4	120	38	No		
Male	13	No	1		18	Yes	Yes	6th
Male	14	Yes	2	6	22	Yes	Yes	7th
Female	12	Yes	2	96	24	Yes	Yes	6th
Female	14	Yes	2	59	23	Yes	Yes	5th
Male	3	No	1		22	Yes	Yes	10th
Male	5	Yes	2	48	23	Yes	Yes	8th
Male	9	No	1		24	Yes	Yes	10th
Male	4	No	1		19	Yes	Yes	7th
Male	4	No	1		20	Yes	Yes	10th
Male	12	Yes	7	132	38	Yes	No	
Male	8	No	1		18	Yes	Yes	7th
Male	11	No	2	3	28	Yes	Yes	8th
Female	3	No	2	2	23	Yes	Yes	7th
Male	14	Yes	1		22	Yes	Yes	9th
Male	12	Yes	2	96	27	Yes	Yes	6th
Female	14	No	1		18	Yes	Yes	6th
Male	8	Yes	3	36	25	Yes	Yes	5th
Male	14	Yes	2	72	32	Yes	Yes	8th
Female	12	Yes	2	96	29	Yes	Yes	10th
Male	10	Yes	2	2	23	Yes	Yes	5th
Male	14	Yes	2	48	24	Yes	Yes	7th
Male	3	No	1		23	Yes	Yes	10th
Female	13	Yes	2	59	22	Yes	Yes	Technical/\
Male	6	Yes	5	36	30	No		
Male	9	Yes	2	10	28	Yes	Yes	4th
Female	2	No	1		23	No		
Female	13	No	1		26	Yes	Yes	12th
Male	10	Yes	2	9	30	No		
Female	14	Yes	4	7	35	No		
Female	9	Yes	4	62	32	No		
Male	11	Yes	6	3	32	No		
Male	3	Yes	2	4	24	Yes	Yes	8th

For peer review only

Male	13	Yes	6	4	38	No		
Male	7	Yes	2	59	26	Yes	Yes	7th
Male	7	No	1		24	Yes	Yes	Technical/\
Male	12	No	1		20	Yes	Yes	10th
Male	3	Yes	2	4	21	No		
Male	13	No	2	6	28	Yes	Yes	9th
Male	12	Yes	2	7	22	Yes	Yes	6th
Male	12	Yes	2	5	25	Yes	Yes	7th
Male	14	No	1		20	Yes	Yes	8th
Male	9	No	1		18	Yes	Yes	6th
Male	5	No	1		21	Yes	Yes	10th
Female	10	Yes	3	8	30	No		
Male	5	Yes	3	48	26	No		
Male	14	Yes	2	90	26	Yes	Yes	7th
Female	8	Yes	3	3	28	Yes	Yes	10th
Male	6	Yes	2	34	20	Yes	Yes	4th
Female	14	Yes	1		19	Yes	Yes	4th
Male	3	Yes	1		26	Yes	Yes	University/
Male	3	Yes	3	5	27	No		
Female	3	Yes	4	3	32	Yes	Yes	University/
Female	3	Yes	2	6	30	No		
Male	6	Yes	5	38	35	No		
Male	12	Yes	5	5	40	No		
Female	6	Yes	4	3	23	No		
Male	9	Yes	5	36	28	No		
Male	9	Yes	4	2	35	No		
Male	9	Yes	4	5	30	No		
Female	13	Yes	7	3	38	No		
Male	10	No	1		25	Yes	Yes	8th
Male	8	No	1		27	Yes	Yes	Technical/\
Female	11	Yes	10	3	38	No		
Female	11	Yes	2	3	27	Yes	Yes	8th
Female	3	Yes	3	7	30	Yes	Yes	7th
Male	14	Yes	4	5	30	No		
Female	14	Yes	5	6	35	No		
Female	4	Yes	4	36	30	No		
Female	2	Yes	3	4	25	Yes	Yes	4th
Female	13	No	1		26	Yes	Yes	9th
Female	7	Yes	3	3	25	No		
Female	8	Yes	5	3	35	No		
Female	3	Yes	5	31	38	No		
Female	13	Yes	3	12	36	No		
Female	6	Yes	6	24	35	No		
Male	3	Yes	6	2	30	No		
Male	9	Yes	4	14	40	No		
Female	12	Yes	2	6	23	Yes	Yes	9th
Male	11	Yes	2	6	25	Yes	Yes	8th
Male	8	Yes	6	36	35	No		
Male	14	Yes	2	3	35	No		
Male	8	Yes	2	3	32	Yes	Yes	Technical/\
Male	12	Yes	6	3	35	No		
Female	9	No	1		21	Yes	Yes	10th

Female	9	No	1		30	No		
Female	6	Yes	2	3	22	Yes	Yes	5th
Male	3	Yes	2	3	28	Yes	Yes	8th
Female	13	Yes	7	36	39	Yes	Yes	3rd
Female	14	Yes	3	4	30	Yes	Yes	8th
Female	9	Yes	5	48	30	No		
Female	7	Yes	8	7	39	No		
Female	3	Yes	8	3	36	No		
Male	4	Yes	2	6	23	Yes	Yes	8th
Female	3	Yes	4	48	35	No		
Female	14	Yes	4	8	40	No		
Male	12	Yes	5	3	35	No		
Male	13	Yes	4	5	41	No		
Female	5	No	1		22	Yes	Yes	10th
Male	6	Yes	4	3	30	Yes	Yes	9th
Male	9	Yes	2	4	25	Yes	Yes	6th
Male	4	Yes	3	2	25	Yes	Yes	8th
Male	9	Yes	5	46	38	No		
Female	7	Yes	3	5	30	No		
Female	7	Yes	7	72	40	No		
Female	11	Yes	5	3	30	No		
Male	9	Yes	8	3	38	No		
Male	11	Yes	5	4	35	No		
Female	8	Yes	2	3	20	Yes	Yes	8th
Female	13	Yes	5	44	35	No		
Female	11	Yes	7	3	38	No		
Male	5	Yes	2	156	33	No		
Male	12	Yes	4	6	36	No		
Female	13	Yes	2	60	28	No		
Male	14	Yes	6	4	36	No		
Female	9	Yes	3	42	30	No		
Male	11	Yes	2	84	30	Yes	Yes	5th
Male	12	Yes	4	3	35	No		
Female	3	Yes	2	24	23	Yes	Yes	2nd
Female	3	Yes	6	28	38	No		
Male	5	Yes	8	4	38	No		
Female	14	Yes	7	84	45	No		
Male	6	Yes	4	60	30	No		
Female	12	No	1		30	No		
Female	14	Yes	3	4	30	Yes	No	
Female	7	Yes	5	4	40	No		
Female	4	Yes	5	29	34	No		
Male	9	Yes	5	48	35	No		
Male	14	Yes	2	36	26	No		
Male	9	Yes	5	2	18	No		
Male	10	Yes	6	3	34	No		
Male	4	No	1		29	Yes	Yes	University/
Female	9	Yes	5	4	34	No		
Male	14	No	1		23	Yes	Yes	7th
Male	11	No	1		19	Yes	Yes	10th
Male	3	Yes	2	3	28	Yes	Yes	12th
Female	2	No	4	2	35	Yes	No	

For peer review only

Male	7	Yes	2	5	30	Yes	Yes	10th
Male	5	Yes	3	5	27	Yes	Yes	10th
Female	10	Yes	3	4	30	Yes	Yes	5th
Male	2	Yes	3	7	25	No		
Male	9	Yes	6	5	32	No		
Male	8	No	1		28	Yes	Yes	8th
		No	1		30	Yes	Yes	University/
Male	6	Yes	2	3	22	Yes	Yes	10th
Female	3	Yes	6	60	37	No		
Male	7	No	1		26	Yes	Yes	9th
Female	7	Yes	5	3	35	Yes	Yes	2nd
Male	12	No	1		29	Yes	Yes	7th
Female	14	Yes	6	60	38	No		
Female	11	Yes	4	8	36	No		
Male	4	No	1		22	Yes	Yes	8th
Female	14	Yes	4	132	39	No		
Female	10	Yes	4	4	38	No		
Male	11	Yes	5	9	30	No		
Male	2	No	1		21	Yes	Yes	10th
Male	14	Yes	2	120	28	No		
Male	9	Yes	5	36	29	No		
Female	5	No	1		20	No		
Male	9	Yes	4	46	30	Yes	Yes	6th
Female	11	Yes	2	5	23	Yes	Yes	7th
Female	8	Yes	2	60	24	No		
Male	11	Yes	2	63	22	Yes	Yes	7th
Male	11	Yes	3	70	27	No		
Male	5	Yes	3	45	28	Yes	Yes	3rd
Female	2	Yes	1		19	No		
Male	5	Yes	2	53	25	No		
Female	13	No	1		19	Yes	Yes	8th
Male	12	Yes	2	60	24	Yes	Yes	4th
Male	12	No	1		20	Yes	Yes	4th
Male	5	No	1		20	Yes	Yes	10th
Male	7	Yes	2	84	25	Yes	Yes	5th
Male	6	No	1		19	Yes	Yes	4th
Female	5	Yes	1		27	Yes	Yes	10th
Female	10	Yes	2	96	25	Yes	Yes	7th
Male	6	No	1		20	No		
Female	12	Yes	4	57	30	No		
Male	7	No	1		27	Yes	Yes	4th
Male	10	No	1		23	Yes	Yes	4th
Male	7	Yes	2	13	30	Yes	Yes	3rd
Female	14	No	1		20	Yes	Yes	8th
Female	8	Yes	7	36	36	No		
Male	11	No	1		19	Yes	Yes	8th
Female	5	Yes	5	16	40	No		
Female	3	Yes	2	64	24	Yes	Yes	3rd
Female	4	Yes	2	36	25	Yes	Yes	8th
Male	2	No	1		20	Yes	Yes	1st
Female	7	Yes	4	9	33	No		
Male	9	No	1		20	Yes	Yes	10th

For peer review only

Male	5	No	1		18	Yes	Yes	6th
Female	11	Yes	4	8	36	No		
Male	3	Yes	3	108	30	No		
Male	14	Yes	6	36	30	No		
Male	8	No	0	0	25	Yes	Yes	10th
Female	10	No	1		20	Yes	Yes	3rd
Female	12	Yes	3	72	30	No		
Male	6	Yes	5	3	28	No		
Male	8	Yes	4	2	35	No		
Male	8	Yes	4	96	37	Yes	Yes	5th
Female	11	Yes	2	132	28	Yes	Yes	5th
Male	3	Yes	5	5	35	No		
Female	5	Yes	3	48	32	No		
Female	6	Yes	3	84	38	No		
Male	7	Yes	3	15	30	No		
Male	2	Yes	4	4	32	No		
Male	12	Yes	3	48	32	No		
Female	4	Yes	4	4	28	No		
Female	9	No	3	7	30	No		
Male	9	No	3	120	27	Yes	Yes	5th
Female	14	Yes	14	60	22	Yes	Yes	6th
Male	3	Yes	4	36	30	Yes	Yes	8th
Female	12	Yes	3	6	32	No		
Female	2	Yes	4	12	36	No		
Female	10	No	4	100	35	No		
Male	14	Yes	3	72	32	No		
Female	10	Yes	4	72	35	No		
Male	12	Yes	3	36	28	Yes	Yes	6th
Male	14	Yes	5	156	35	Yes	Yes	4th
Male	14	Yes	3	48	35	No		
Male	3	Yes	2	5	22	No		
Male	10	Yes	2	48	29	No		
Male	9	No	2	36	32	Yes	Yes	9th
Female	11	Yes	7	34	43	No		
Female	3	Yes	2	8	25	Yes	Yes	3rd
Female	3	Yes	2	48	30	No		
Female	13	No	1		20	Yes	Yes	9th
Female	11	Yes	3	84	26	Yes	Yes	6th
Female	7	Yes	3	59	28	No		
Male	4	Yes	3	60	25	Yes	Yes	9th
Male	11	Yes	4	4	35	No		
Female	12	Yes	6	48	38	No		
Female	10	Yes	2	10	28	Yes	Yes	4th
Female	12	Yes	8	84	40	No		
Female	9	Yes	2	42	27	Yes	Yes	University/
Male	12	No	8	5	40	No		
Female	5	Yes	4	5	40	No		
Male	5	Yes	2	3	22	Yes	Yes	3rd
Male	6	Yes	3	36	38	Yes	No	
Female	10	Yes	5	48	38	No		
Male	7	Yes	2	48	25	Yes	Yes	6th
Male	12	Yes	6	3	39	No		

For peer review only

Female	13	Yes	5	7	38	No		
Male	12	No	1		24	No		
Male	7	Yes	1		24	Yes	Yes	12th
Female	5	Yes	2	60	35	No		
Female	9	Yes	9	32	38	No		
Female	12	Yes	3	120	30	Yes	Yes	7th
Male	4	No	1		18	Yes	Yes	5th
Female	11	Yes	5	8	35	No		
Female	6	No	1		29	No		
Female	5	Yes	3	7	28	Yes	Yes	3rd
Female	14	Yes	3	3	32	No		
Female	8	Yes	4	5	36	No		
Male	3	Yes	2	115	26	Yes	Yes	5th
Male	7	Yes	5	72	29	No		
Male	3	Yes	4	36	30	Yes	Yes	8th
Female	3	No	1		25	Yes	Yes	10th
Male	14	Yes	4	3	33	No		
Male	12	Yes	3	36	26	Yes	Yes	4th
Male	6	No	1		22	Yes	Yes	5th
Female	12	Yes	5	48	38	No		
Female	8	Yes	2	59	27	Yes	Yes	5th
Female	14	Yes	2	59	25	Yes	Yes	6th
Female	3	Yes	4	6	28	Yes	Yes	5th
Male	7	Yes	2	5	25	Yes	Yes	8th
Male	8	No	1		24	Yes	Yes	4th
Male	3	Yes	4	3	28	Yes	Yes	University/
Female	11	Yes	7	126	41	No		
Male	14	Yes	5	8	35	No		
Male	13	Yes	3	8	32	No		
Male	3	No	1		23	Yes	Yes	8th
Male	14	Yes	5	59	38	No		
Male	5	No	1		18	Yes	Yes	5th
Female	12	Yes	8	6	45	No		
Female	12	Yes	7	5	30	No		
Male	12	Yes	7	4	39	No		
Female	5	Yes	6	38	25	No		
Female	4	Yes	2	36	22	No		
Female	4	Yes	5	36	32	No		
Male	14	Yes	3	36	25	No		
Male	13	Yes	3	6	30	Yes	Yes	4th
Female	2	No	1		22	No		
Male	12	Yes	5	28	36	No		
Female	14	Yes	6	3	36	No		
Male	6	Yes	2	53	26	Yes	Yes	12th
Male	14	Yes	5	36	35	No		
Male	14	Yes	3	36	32	No		
Male	14	Yes	5	28	32	No		
Male	3	Yes	5	4	27	No		
Female	6	No	1		23	Yes	Yes	5th
Female	14	Yes	4	60	37	No		
Female	3	Yes	6	6	36	No		
Female	6	Yes	3	24	35	No		

For peer review only

Male	10	Yes	5	60	32	No		
Male	13	Yes	5	43	28	No		
Female	10	Yes	8	34	33	No		
Male	14	Yes	5	4	36	No		
Female	8	Yes	0	0	28	No		
Female	9	Yes	6	5	36	No		
Male	14	Yes	2	36	32	No		
Female	7	Yes	3	46	32	No		
Male	14	Yes	6	4	35	Yes	Yes	7th
Male	14	Yes	5	4	35	No		
Male	10	No	1		30	No		
Female	8	No	0	0	18	Yes	Yes	5th
Female	3	Yes	2	51	23	Yes	Yes	8th
Male	14	Yes	5	3	27	No		
Male	10	Yes	9	5	37	No		
Male	6	Yes	5	3	25	Yes	No	
Female	3	Yes	4	3	30	Yes	Yes	3rd
Female	3		2	28	22	No		
Female	6	Yes	2	36	32	No		
Male	3	No	1		18	Yes	Yes	6th
Male	3	Yes	6	48	38	No		
Female	6	No	1		21	Yes	Yes	10th
Female	4	Yes	6	24	36	No		
Male	14	Yes	3	24	36	No		
Female	7	No	1		21	Yes	Yes	6th
Female	6	Yes	3	24	31	No		
Male	6	Yes	2	5	20	No		
Female	12	Yes	4	9	30	No		
Male	5		3	26	32	No		
Male	14	No	1		24	Yes	Yes	5th
Male	5	Yes	8	42	35	No		
Male	5	Yes	5	3	28	No		
Female	11	Yes	6	3	30	No		
Male	9	Yes	5	48	35	Yes	Yes	2nd
Male	4	No	1		20	Yes	Yes	5th
Female	8	Yes	3	28	32	No		
Male	12	Yes	6	7	26	No		
Male	3	Yes	7	27	38	No		
Male	8	Yes	4	39	30	No		
Male	8	No	1		20	Yes	Yes	5th
Male	7	Yes	6	43	40	No		
Male	14	Yes	2	3	28	Yes	Yes	7th
Female	13	No	1		22	No		
Female	10	Yes	2	72	25	No		
Male	6	Yes	2	56	22	No		
Female	14	No	2	3	21	Yes	Yes	10th
Male	8	No	1		20	Yes	Yes	10th
Male	14	Yes	3	64	26	Yes	No	
Male	13	Yes	3	84	28	No		
Male	8	Yes	2	36	28	Yes	Yes	4th
Male	14	No	1		26	Yes	Yes	8th
Male	2	Yes	4	60	33	No		

For peer review only

Male	4	Yes	7	36	40	No		
Male	3	No	1		22	Yes	Yes	8th
Female	6	Yes	5	120	35	Yes	Yes	8th
Male	14	Yes	3	120	30	No		
Female	7	Yes	7	27	30	No		
Female	14	Yes	2	84	23	Yes	Yes	8th
Female	8	Yes	2	7	20	No		
Male	14	Yes	2	84	30	No		
Male	2	No	1		23	Yes	Yes	5th
Male	7	No	1		25	No		
Male	9	Yes	2	7	25	Yes	Yes	6th
Female	12	Yes	3	3	32	No		
Male	7	Yes	3	59	28	Yes	Yes	5th
Female	12	Yes	3	5	30	No		
Female	3	No	1		19	Yes	Yes	6th
Female	11	Yes	2	33	22	Yes	Yes	6th
Male	2	No	1		19	Yes	Yes	5th
Male	6	Yes	2	84	22	Yes	Yes	6th
Male	10	Yes	2	64	25	Yes	Yes	10th
Male	10	Yes	2	72	25	Yes	Yes	3rd
Female	14	No	1		23	Yes	Yes	10th
Female	9	Yes	2	24	32	Yes	Yes	3rd
Male	11	Yes	3	66	28	Yes	Yes	6th
Female	11	No	1		20	Yes	Yes	4th
Male	12	Yes	10	3	38	No		
Male	14	Yes	3	360	30	No		
Female	4	Yes	2	2	28	Yes	Yes	3rd
Female	5	Yes	2	42	22	Yes	Yes	7th
Female	11	No	1		23	Yes	Yes	6th
Male	4	Yes	3	82	36	Yes	Yes	10th
Male	4	Yes	2	72	25	Yes	Yes	2nd
Male	14	Yes	2	66	23	Yes	Yes	9th
Female	7	Yes	4	132	40	No		
Male	14	No	1		22	Yes	Yes	6th
Female	14	Yes	2	7	25	Yes	Yes	10th
Female	14	Yes	5	4	30	No		
Male	7	No	2	84	25	Yes	Yes	7th
Female	8	No	1		19	Yes	Yes	5th
Female	8	Yes	3	3	30	Yes	Yes	4th
Female	7	Yes	2	59	30	Yes	Yes	10th
Male	14	Yes	2	132	32	Yes	Yes	7th
Female	2	Yes	4	64	35	No		
Female	9	No	1		18	Yes	Yes	10th
Male	7	Yes	2	120	25	Yes	Yes	7th
Female	12	Yes	5	60	30	No		
Female	14	Yes	4	60	34	No		
Male	13	Yes	3	5	28	Yes	Yes	9th
Male	10	Yes	2	60	21	Yes	Yes	5th
Female	5	No	1		18	Yes	Yes	10th
Male	10	Yes	2	45	24	Yes	Yes	5th
Female	2	No	1		20	Yes	Yes	10th
Male	6	Yes	1		33	Yes	Yes	8th

For peer review only

For peer review only - http://bmjopen.bmj.com/site/about/guidelines.xhtml

Male	5	Yes	3	8	30	Yes	Yes	8th
Female	7	No	1		22	Yes	Yes	University/
Female	12	No	1		28	Yes	Yes	10th
Female	14	Yes	1		26	Yes	Yes	5th
Female	14	Yes	2	48	28	Yes	Yes	10th
Male	3	Yes	6	36	30	Yes	Yes	3rd
Female	5	No	1		19	Yes	Yes	8th
Male	3	Yes	3	36	27	Yes	Yes	5th
Male	14	Yes	2	84	25	Yes	Yes	10th
Male	14	Yes	2	58	26	Yes	Yes	10th
Female	12	Yes	3	5	35	Yes	No	
Female	14	No	1		19	Yes	Yes	6th
Female	10	No	1		27	No		
Female	3	No	1		19	Yes	Yes	4th
Male	9	Yes	2	36	24	Yes	Yes	10th
Female	9	No	1		24	Yes	Yes	6th
Female	7	Yes	2	31	30	Yes	Yes	9th
Female	8	Yes	5	35	32	No		
Male	6	Yes	2	53	25	No		
Male	10	Yes	2	41	23	Yes	Yes	4th
Male	12	Yes	2	9	22	Yes	Yes	8th
Female	3	Yes	3	42	31	No		
Male	11	No	1		18	Yes	Yes	7th
Male	6	No	1		19	No		
Male	10	No	1		30	No		
Male	8	Yes	2	60	27	Yes	Yes	4th
Female	9	Yes	1		19	Yes	Yes	9th
Female	7	Yes	2	66	25	Yes	Yes	3rd
Female	11	Yes	2	120	30	No		
Male	13	Yes	9	36	39	No		
Male	14	No	1		22	Yes	Yes	6th
Male	12	Yes	3	120	34	Yes	Yes	6th
Female	10	No	1		22	Yes	Yes	10th
Male	11	Yes	7	72	40	Yes	Yes	7th
Male	4	Yes	2	51	28	Yes	No	
Male	4	Yes	2	60	23	Yes	Yes	7th
Male	3	Yes	2	36	28	Yes	Yes	10th
Male	7	No	1		23	Yes	Yes	8th
Male	14	No	1		22	Yes	Yes	5th
Male	4	Yes	6	84	44	No		
Male	8	No	1		19	Yes	Yes	8th
Female	7	No	1		21	Yes	Yes	6th
Male	10	Yes	2	86	30	No		
Male	4	Yes	4	6	36	No		
Female	6	Yes	2	120	30	Yes	Yes	4th
Male	3	No	1		28	No		
Male	10	No	1		24	No		
Male	11	Yes	10	45	44	No		
Male	10	No	1		20	Yes	Yes	10th
Female	12	Yes	8	8	36	No		
Male	2	Yes	3	6	29	Yes	Yes	8th
Female	14	Yes	6	120	32	No		

Female	8	Yes	2	59	22	Yes	Yes	7th
Female	6	Yes	1		32	Yes	Yes	8th
Female	7	No	1		19	Yes	Yes	12th
Female	7	No	1		28	Yes	Yes	2nd
Male	3	Yes	2	98	28	Yes	Yes	3rd
Male	5	No	1		19	Yes	Yes	6th
Male	10	Yes	2	60	24	Yes	Yes	Technical/\
Female	3	Yes	3	28	26	Yes	Yes	7th
Male	4	Yes	4	24	22	No		
Male	8	No	1		26	Yes	Yes	10th
Male	2	No	1		18	Yes	Yes	5th
Female	5	Yes	3	96	35	Yes	Yes	8th
Male	11	No	1		20	No		
Male	14	No	1		20	Yes	Yes	8th
Male	3	Yes	5	84	35	No		
Male	6	No	1		18	Yes	Yes	7th
Male	3	Yes	2	5	27	Yes	Yes	9th
Male	6	Yes	3	56	32	Yes	Yes	10th
Male	12	Yes	4	72	30	No		
Female	10	Yes	4	6	38	Yes	Yes	7th
Male	6	No	2	3	24	Yes	Yes	4th
Male	12	Yes	4	59	32	No		
Female	11	Yes	2	84	30	No		
Male	8	No	1		21	Yes	Yes	10th
Female	14	No	1		21	Yes	Yes	8th
Female	6	No	1		20	Yes	Yes	10th
Male	5	No	1		21	Yes	Yes	10th
Female	4	No	1		20	Yes	Yes	10th
Female	14	No	1		20	Yes	Yes	8th
Male	8	Yes	1		22	Yes	Yes	6th
Male	5	Yes	2	48	25	Yes	Yes	9th
Male	14	Yes	2	64	32	Yes	Yes	Technical/\
Female	9	No	1		21	Yes	Yes	10th
Male	14	Yes	4	36	35	No		
Female	3	Yes	4	15	36	No		
Female	11	Yes	3	97	32	No		
Male	14	Yes	2	9	26	Yes	Yes	2nd
Female	7	Yes	4	72	36	Yes	Yes	7th
Female	5	Yes	2	36	22	Yes	Yes	10th
Female	10	Yes	2	5	25	No		
Female	8	Yes	1		24	Yes	Yes	10th
Male	12	Yes	6	72	32	No		
Female	13	Yes	4	4	32	No		
Male	6	No	1		26	Yes	Yes	10th
Female	13	Yes	8	60	34	No		
Male	2	Yes	3	41	32	No		
Male	12	Yes	3	4	35	No		
Female	10	Yes	2	4	19	Yes	Yes	9th
Male	8	Yes	1		15	Yes	Yes	3rd
Female	12	Yes	3	29	26	No		
Male	10	No	1		18	Yes	Yes	6th
Male	7	Yes	5	3	32	Yes	Yes	8th

For peer review only

Male	14	Yes	2	48	26	Yes	Yes	7th
Female	4	No	1		35	Yes	Yes	10th
Female	9	Yes	3	96	29	Yes	Yes	7th
Female	11	No	1		26	Yes	Yes	10th
Male	12	No	1		25	No		
Female	3	Yes	3	108	28	Yes	Yes	4th
Female	9	Yes	2	69	24	Yes	Yes	9th
Female	9	Yes	2	36	20	No		
Male	14	Yes	2	96	27	No		
Male	9	Yes	4	2	30	No		
Male	11	Yes	1		23	Yes	Yes	8th
Female	8	Yes	2	72	24	Yes	Yes	7th
Male	8	No	1		32	No		
Male	5	No	1		24	Yes	Yes	5th
Female	14	Yes	2	120	26	No		
Female	6	Yes	4	60	32	No		
Female	3	Yes	7	34	35	No		
Female	9	No	2	62	38	No		
Female	8	Yes	3	80	31	Yes	Yes	10th
Male	10	Yes	4	10	38	No		
Female	8	Yes	1		28	Yes	Yes	10th
Female	4	Yes	3	45	25	Yes	Yes	3rd
Male	6	Yes	2	30	24	No		
Male	8	Yes	2	59	24	Yes	Yes	8th
Female	12	Yes	3	60	28	No		
Female	7	No	1		20	Yes	Yes	10th
Female	14	Yes	8	36	36	No		
Female	5	No	1		20	Yes	Yes	4th
Female	10	Yes	2	48	23	Yes	Yes	8th
Male	13	No	1		22	Yes	Yes	7th
Female	14	Yes	5	4	30	No		
Male	6	No	1		21	Yes	Yes	8th
Female	10	No	1		17	Yes	Yes	5th
Female	12	No	3	3	27	Yes	Yes	7th
Female	5	Yes	1		28	Yes	Yes	4th
Female	7	Yes	4	36	28	No		
Female	14	Yes	3	180	43	Yes	Yes	5th
Female	4	Yes	1		27	Yes	Yes	7th
Male	4	Yes	3	36	34	No		
Female	7	Yes	2	24	25	No		
Female	6	No	1		20	Yes	Yes	7th
Male	12	Yes	2	44	22	Yes	Yes	9th
Female	11	No	1		22	Yes	Yes	10th
Male	13	Yes	2	8	28	Yes	Yes	6th
Male	7	Yes	1		23	Yes	Yes	8th
Male	5	No	1		28	No		
Male	12	No	1		20	Yes	Yes	7th
Male	11	No	1		22	Yes	Yes	10th
Male	7	Yes	1		20	Yes	Yes	10th
Female	7	No	1		19	Yes	Yes	10th
Female	14	Yes	1		28	No		
Female	7	Yes	2	7	24	No		

For peer review only

Male	13	Yes	2	72	29	Yes	Yes	8th
Male	3	Yes	3	60	29	Yes	Yes	10th
Female	12	Yes	2	3	31	Yes	Yes	University/
Male	9	Yes	5	60	28	No		
Male	3	Yes	1		30	Yes	Yes	7th
Female	4	No	1		26	No		
Female	14	No	1		21	Yes	Yes	5th
Female	11	No	1		20	Yes	Yes	5th
Male	12	Yes	5	36	37	No		
Male	12	No	1		39	Yes	Yes	10th
Female	12	Yes	3	90	32	Yes	Yes	9th
Male	6	Yes	7	60	40	No		
Male	3	No	1		20	Yes	Yes	9th
Male	13	Yes	2	5	30	Yes	Yes	6th
Male	14	No	1		30	Yes	Yes	10th
Female	8	No	1		18	Yes	Yes	5th
Male	11	Yes	3	36	24	No		
Male	11	Yes	3	7	35	No		
Male	10	Yes	2	30	21	Yes	Yes	5th
Female	4	No	1		23	Yes	Yes	10th
Male	6	Yes	3	108	30	No		
Male	10	Yes	2	86	28	Yes	Yes	7th
Female	10	No	1		31	Yes	Yes	8th
Male	14	Yes	1		20	Yes	Yes	9th
Male	9	Yes	3	7	28	Yes	Yes	4th
Male	11	Yes	5	7	35	No		
Male	6	Yes	2	59	21	Yes	Yes	6th
Male	5	Yes	2	72	28	Yes	Yes	9th
Female	9	Yes	5	3	35	No		
Female	3	Yes	4	3	38	No		
Male	9	No	1		15	Yes	Yes	5th
Female	14	No	1		23	Yes	Yes	9th
Female	14	Yes	2	42	24	Yes	Yes	8th
Female	7	No	1		22	Yes	Yes	7th
Male	5	Yes	2	4	28	Yes	Yes	10th
Female	6	Yes	5	36	28	No		
Female	7	Yes	3	72	30	No		
Female	4	No	1		17	Yes	Yes	8th
Male	13	No	1		19	Yes	Yes	10th
Male	10	Yes	2	8	27	Yes	Yes	University/
Male	14	Yes	6	120	38	No		
Female	14	Yes	4	70	32	No		
Male	7	Yes	2	48	27	Yes	Yes	10th
Male	5	No	1		22	Yes	Yes	University/
Female	4	Yes	6	3	41	No		
Female	4	Yes	8	6	35	No		
Male	13	Yes	3	48	32	Yes	Yes	9th
Female	13	Yes	4	60	35	No		
Female	14	Yes	8	4	40	No		
Male	3	Yes	2	33	28	Yes	Yes	4th
Male	7	Yes	4	2	30	No		
Male	2	No	1		18	Yes	Yes	8th

For peer review only

Female	7	Yes	5	144	35	No		
Male	10	Yes	7	3	37	Yes	Yes	6th
Male	12	Yes	6	84	40	No		
Female	12	Yes	4	132	39	Yes	Yes	4th
Female	3	Yes	2	84	22	Yes	Yes	7th
Female	10	No	1		20	Yes	Yes	8th
Male	12	Yes	3	7	28	Yes	Yes	4th
Female	6	No	1		20	Yes	Yes	8th
Male	10	Yes	2	2	24	Yes	Yes	3rd
Female	11	No	1		19	Yes	Yes	8th
Female	2	Yes	7	3	45	No		
Female	3	Yes	3	4	29	Yes	Yes	10th
Male	6	Yes	3	4	27	Yes	Yes	4th
Male	13	Yes	2	71	22	Yes	Yes	9th
Male	8	Yes	3	8	30	No		
Female	5	Yes	2	6	25	Yes	Yes	8th
Female	11	Yes	5	4	35	No		
Male	14	Yes	2	5	23	Yes	Yes	7th
Male	12	Yes	2	155	34	Yes	Yes	6th
Female	6	Yes	3	43	36	Yes	Yes	2nd
Male	9	Yes	3	3	25	No		
Male	10	Yes	3	47	36	No		
Male	14	Yes	0	7	28	No		
Female	9	Yes	2	5	32	Yes	Yes	4th
Male	13	No	1		20	Yes	Yes	6th
Female	12	Yes	4	48	35	No		
Male	14	Yes	4	5	38	Yes	Yes	3rd
Male	11	Yes	2	84	28	Yes	Yes	10th
Male	2	Yes	2	6	28	Yes	No	
Female	8	Yes	5	5	38	No		
Female	6	Yes	2	72	23	Yes	Yes	9th
Male	13	Yes	3	9	34	Yes	Yes	4th
Female	8	Yes	2	48	20	No		
Female	14	No	1		18	Yes	Yes	4th
Male	8	Yes	3	4	23	No		
Female	14	Yes	3	12	34	Yes	Yes	2nd
Male	7	No	1		20	Yes	Yes	10th
Female	14	Yes	4	9	39	No		
Female	9	Yes	5	3	39	No		
Female	14	Yes	5	12	30	No		
Male	10	Yes	3	11	28	No		
Male	12	Yes	2	121	26	Yes	Yes	6th
Male	5	Yes	4	8	38	No		
Male	6	Yes	2	72	27	Yes	Yes	9th
Male	14	No	1		26	Yes	Yes	7th
Male	14	Yes	6	13	40	No		
Female	13	No	1		20	Yes	Yes	8th
Male	6	No	1		21	Yes	Yes	9th
Male	5	No	1		20	Yes	Yes	7th
Female	11	No	1		23	Yes	Yes	8th
Female	12	Yes	5	4	38	No		
Female	14	Yes	3	125	32	No		

For peer review only

Male	13	Yes	2	6	30	No		
Male	14	Yes	2	9	38	Yes	Yes	7th
Male	14	Yes	2	72	25	No		
Female	6	Yes	60	6	21	Yes	Yes	5th
Female	7	No	1		20	Yes	Yes	7th
Female	14	Yes	2	108	27	Yes	Yes	10th
Male	3	No	1		20	Yes	Yes	10th
Male	14	Yes	1		24	Yes	Yes	10th
Female	9	Yes	10	36	36	No		
Male	2	Yes	2	55	22	Yes	Yes	5th
Male	4	Yes	3	120	32	No		
Female	4	No	1		21	Yes	Yes	8th
Female	11	Yes	3	108	26	No		
Male	9	No	1		20	No		
Male	14	Yes	5	8	29	No		
Male	6	Yes	4	70	26	Yes	Yes	4th
Male	9	No	1		18	No		
Male	5	Yes	1		20	Yes	Yes	8th
Male	11	Yes	3	10	25	Yes	Yes	3rd
Female	6	No	1		26	Yes	Yes	10th
Female	9	Yes	2	58	21	Yes	Yes	5th
Female	9	Yes	1		21	Yes	Yes	8th
Female	9	No	1		22	Yes	Yes	10th
Female	3	Yes	8	5	38	No		
Female	7	Yes	6	36	35	Yes	No	
Male	12	Yes	0	7	24	Yes	Yes	5th
Female	10	Yes	1		22	Yes	Yes	10th
Female	4	No	1		25	Yes	Yes	10th
Female	5	Yes	5	3	40	No		
Female	14	Yes	6	4	38	No		
Male	8	No	1		24	No		
Male	12	Yes	4	7	30	No		
Female	12	Yes	6	39	36	No		
Female	11	Yes	2	60	23	Yes	Yes	8th
Female	8	Yes	5	66	34	No		
Male	4	Yes	4	14	36	No		
Female	13	Yes	3	120	30	Yes	Yes	2nd
Male	11	Yes	2	60	27	Yes	Yes	5th
Female	5	No	1		27	Yes	Yes	9th
Male	14	No	1		20	Yes	Yes	6th
Male	14	No	1		22	No		
Male	14	Yes	2	3	26	No		
Female	9	Yes	7	108	30	No		
Male	12	Yes	4	3	38	No		
Male	14	Yes	3	7	32	Yes	Yes	3rd
Female	5	Yes	2	84	24	Yes	Yes	5th
Female	14	Yes	7	38	39	No		
Female	10	Yes	2	59	24	Yes	Yes	8th
Female	8	Yes	4	72	25	Yes	Yes	7th
Male	2	No	1		22	Yes	Yes	8th
Female	14	Yes	3	7	23	Yes	Yes	6th
Male	10	No	1		21	Yes	Yes	9th

For peer review only

Male	9	No	1		24	No		
Female	7	Yes	1		23	Yes	Yes	10th
Female	5	Yes	5	2	38	No		
Male	6	No	1		19	Yes	Yes	7th
Female	4	No	1		20	Yes	Yes	8th
Female	3	Yes	5	2	35	No		
Male	3	Yes	2	2	20	Yes	Yes	4th
Female	3	No	1		20	No		
Female	6	Yes	2	120	27	Yes	Yes	5th
Male	10	No	1		22	Yes	Yes	10th
Female	13	Yes	1		22	No		
Female	8	No	1		25	Yes	Yes	University/
Female	14	Yes	2	12	36	No		
Female	3	No	1		20	Yes	Yes	6th
Male	3	Yes	5	84	34	No		
Female	12	No	1		28	Yes	Yes	8th
Female	11	Yes	2	10	27	Yes	Yes	7th
Female	12	No	1		19	Yes	Yes	6th
Male	3	No	1		23	Yes	Yes	8th
Male	6	No	1		23	Yes	Yes	7th
Female	14	Yes	0	9	26	Yes	Yes	3rd
Male	4	Yes	4	3	35	No		
Male	8	No	4	96	37	No		
Male	7	Yes	2	60	25	No		
Female	13	No	1		22	Yes	Yes	9th
Female	12	Yes	4	23	35	No		
Female	3	No	1		23	Yes	Yes	4th
Male	3	Yes	1		30	Yes	Yes	8th
Female	9	Yes	5	13	37	No		
Male	3	Yes	7	52	39	No		
Male	9	Yes	0	6	26	Yes	Yes	8th
Male	9	No	1		22	Yes	Yes	8th
Male	14	Yes	4	8	38	No		
Male	14	Yes	2	48	19	Yes	Yes	7th
Male	14	Yes	2	8	28	Yes	Yes	7th
Female	5	Yes	2	72	24	Yes	Yes	7th
Female	12	Yes	3	60	29	Yes	Yes	6th
Female	7	Yes	2	8	24	Yes	Yes	5th
Female	13	No	1		24	Yes	Yes	10th
Female	13	Yes	5	36	35	No		
Male	12	Yes	2	5	30	Yes	Yes	6th
Female	12	Yes	0	14	38	No		
Male	3	Yes	3	28	27	Yes	Yes	6th
Male	3	Yes	2	48	22	Yes	Yes	4th
Male	3	No	1		25	Yes	Yes	University/
Male	13	Yes	3	120	28	Yes	Yes	3rd
Female	2	Yes	8	24	37	No		
Male	7	Yes	1		21	Yes	Yes	4th
Female	10	No	1		20	Yes	Yes	9th
Female	13	Yes	2	40	26	Yes	Yes	University/
Male	6	Yes	3	7	32	Yes	Yes	6th
Male	14	Yes	3	9	24	Yes	Yes	5th

For peer review only

Male	14	No	1		20	Yes	Yes	10th
Male	14	No	1		19	Yes	Yes	4th
Male	10	Yes	3	5	38	No		
Female	12	Yes	2	72	25	Yes	Yes	6th
Male	14	Yes	1		22	No		
Male	14	No	1		25	No		
Male	14	No	1		23	Yes	Yes	10th
Male	7	Yes	5	65	39	No		
Male	7	Yes	4	60	30	No		
Male	10	Yes	4	2	30	No		
Male	3	Yes	2	143	31	Yes	Yes	7th
Female	11	Yes	6	7	30	No		
Female	12	Yes	0	8	27	No		
Female	13	No	4	10	36	No		
Male	12	No	1		23	Yes	Yes	10th
Female	9	Yes	5	4	35	No		
Female	8	Yes	3	84	25	No		
Male	10	Yes	7	2	35	No		
Male	5	Yes	3	5	29	No		
Female	14	Yes	3	4	26	Yes	Yes	8th
Female	6	Yes	2	72	28	Yes	Yes	7th
Female	7	Yes	2	6	23	Yes	Yes	6th
Female	8	Yes	3	60	28	Yes	Yes	2nd
Female	9	No	1		20	Yes	Yes	10th
Female	3	Yes	2	72	30	Yes	Yes	10th
Female	11	Yes	2	84	20	No		
Female	8	Yes	2	89	28	Yes	Yes	7th
Female	13	Yes	2	9	28	No		
Male	14	Yes	2	10	28	Yes	Yes	10th
Female	14	Yes	3	17	35	Yes	Yes	5th
Female	8	Yes	8	60	42	No		
Female	13	Yes	7	3	40	No		
Female	14	Yes	6	45	40	No		
Male	3	Yes	2	5	30	No		
Male	8	Yes	0	4	25	Yes	Yes	7th
Male	6	Yes	2	7	28	Yes	Yes	5th
Male	7	Yes	2	60	22	Yes	Yes	8th
Male	12	Yes	4	15	35	Yes	No	
Male	3	Yes	2	66	25	Yes	Yes	6th
Male	5	Yes	5	10	38	No		
Male	3		2	60	23	No		
Male	14	Yes	3	125	34	No		
Female	6	Yes	2	30	24	Yes	Yes	10th
Male	14	Yes	3	2	25	No		
Male	3	Yes	3	7	27	No		
Female	12	Yes	5	3	40	No		
Male	14	No	1		23	Yes	Yes	6th
Male	14	Yes	4	20	45	No		
Male	5	Yes	1		20	Yes	Yes	10th
Female	4	Yes	0	4	28	No		
Male	5	Yes	4	6	29	No		
Female	8	No	1		20	Yes	Yes	5th

For peer review only

Male	12	Yes	3	82	28	No		
Female	6	Yes	3	10	33	No		
Male	12	Yes	2	8	25	Yes	Yes	3rd
Female	5	Yes	1		22	Yes	Yes	3rd
Female	9	Yes	3	132	31	Yes	Yes	4th
Female	12	Yes	8	4	30	No		
Male	3	Yes	7	4	38	No		
Male	9	Yes	0	12	38	No		
Female	4	No	1		22	Yes	Yes	4th
Female	3	Yes	2	4	26	Yes	Yes	10th
Female	13	Yes	2	72	25	No		
Male	13	Yes	3	11	40	No		
Male	8	Yes	3	59	28	No		
Female	6	No	1		27	Yes	Yes	10th
Male	3	Yes	2	150	32	Yes	Yes	3rd
Male	8	Yes	2	48	23	Yes	Yes	8th
Female	14	Yes	6	90	37	No		
Female	9	Yes	3	102	29	Yes	Yes	6th
Male	11	Yes	1		21	Yes	Yes	9th
Female	14	No	1		23	Yes	Yes	7th
Male	4	Yes	4	64	38	No		
Female	3	Yes	0	8	32	No		
Female	8	No	1		25	Yes	Yes	10th
Female	12	Yes	2	4	25	Yes	Yes	5th
Male	6	Yes	2	3	25	No		
Female	12	Yes	4	2	30	No		
Male	5	Yes	2	4	26	No		
Female	3	Yes	4	30	28	Yes	Yes	3rd
Male	9	Yes	2	36	28	No		
Male	13	Yes	4	3	35	No		
Female	2	Yes	4	10	34	No		
Female	11	Yes	2	72	26	Yes	Yes	9th
Female	10	Yes	2	5	30	Yes	Yes	5th
Female	7	No	1		25	No		
Male	11	Yes	2	120	35	Yes	Yes	6th
Female	6	No	1		19	Yes	Yes	8th
Male	9	No	1		26	No		
Male	14	Yes	2	11	30	Yes	Yes	3rd
Female	8	No	1		22	Yes	Yes	6th
Male	12	No	1		21	Yes	Yes	7th
Male	14	Yes	3	8	32	Yes	Yes	8th
Female	3	Yes	6	2	36	No		
Male	9	Yes	2	76	28	Yes	Yes	5th
Male	9	No	1		23	Yes	Yes	7th
Male	13	Yes	2	96	27	Yes	Yes	6th
Female	7	Yes	2	72	25	Yes	Yes	7th
Male	11	Yes	5	46	35	No		
Female	11	Yes	4	4	35	No		
Female	8	Yes	1		28	Yes	Yes	4th
Male	8	Yes	1		25	No		
Female	6	Yes	6	3	30	No		
Female	7	Yes	2	55	27	Yes	Yes	8th

For peer review only

For peer review only - http://bmjopen.bmj.com/site/about/guidelines.xhtml

Male	2	No	1		18	Yes	Yes	9th
Female	12	Yes	4	66	39	Yes	Yes	8th
Female	9	Yes	2	8	30	No		
Female	2	No	0	0	21	Yes	Yes	8th
Female	8	Yes	0	6	37	No		
Male	14	No	1		20	Yes	Yes	4th
Female	6	Yes	3	15	29	No		
Female	9	Yes	2	8	26	Yes	Yes	7th
Male	9	No	1		22	Yes	Yes	10th
Male	4	Yes	4	4	35	No		
Female	14	Yes	3	120	30	No		
Female	13	Yes	1		21	Yes	Yes	7th
Female	8	Yes	6	5	28	No		
Female	3	Yes	3	74	38	No		
Female	5	Yes	5	96	31	No		
Female	4	No	1		20	Yes	Yes	7th
Female	7	No	1		21	Yes	Yes	8th
Female	14	Yes	5	5	32	No		
Male	9	Yes	4	60	30	No		
Male	14	Yes	2	69	20	Yes	Yes	5th
Female	13	Yes	2	8	28	Yes	Yes	6th
Male	4	Yes	1		20	Yes	Yes	2nd
Male	11	No	1		20	Yes	Yes	6th
Female	7	Yes	4	27	34	No		
Female	14	No	1		21	Yes	Yes	7th
Male	13	Yes	5	7	35	No		
Female	5	Yes	2	120	30	No		
Male	4	Yes	2	84	25	Yes	Yes	7th
Male	2	No	1		19	Yes	Yes	9th
Female	13	Yes	2	72	23	No		
Female	14	Yes	6	7	38	No		
Female	14	Yes	2	6	25	Yes	Yes	9th
Male	10	Yes	0	4	22	Yes	Yes	4th
Male	9	Yes	3	41	36	Yes	Yes	Technical/\
Male	3	No	1		20	No		
Male	5	Yes	2	4	23	Yes	Yes	8th
Male	13	No	1		30	Yes	Yes	8th
Male	7	Yes	3	6	31	Yes	Yes	5th
Male	8	Yes	2	62	30	Yes	Yes	8th
Male	14	Yes	2	12	27	Yes	Yes	2nd
Male	14	No	1		28	Yes	Yes	10th
Female	6	Yes	1		19	Yes	Yes	10th
Male	3	Yes	5	2	37	No		
Male	6	Yes	5	48	38	No		
Male	14	Yes	5	8	35	No		
Female	7	No	1		20	Yes	Yes	7th
Male	14	Yes	2	6	28	Yes	Yes	6th
Female	4	No	1		21	Yes	Yes	9th
Male	9	No	1		30	Yes	Yes	University/
Female	12	Yes	5	8	30	No		
Male	5	No	1		23	Yes	Yes	10th
Female	13	Yes	4	120	37	No		

For peer review only

Male	11	Yes	4	7	30	Yes	No	
Female	3	Yes	3	13	30	Yes	No	
Male	8	No	1		20	Yes	Yes	5th
Male	5	Yes	2	9	22	Yes	Yes	8th
Male	14	Yes	0	6	36	No		
Female	12	Yes	2	6	27	Yes	Yes	9th
Male	3	No	1		19	Yes	Yes	8th
Male	2	Yes	7	3	38	No		
Male	13	Yes	3	8	28	Yes	Yes	3rd
Female	11	No	1		27	Yes	Yes	8th
Male	12	Yes	3	76	33	Yes	Yes	4th
Male	14	Yes	2	120	30	Yes	Yes	6th
Male	9	Yes	3	60	30	Yes	Yes	4th
Female	8	No	1		23	Yes	Yes	6th
Female	9	Yes	2	60	22	Yes	Yes	7th
Male	14	No	1		20	Yes	Yes	4th
Male	2	No	1		20	Yes	Yes	6th
Male	7	Yes	1		19	Yes	Yes	4th
Female	6	No	1		21	Yes	Yes	University/
Female	7	Yes	4	7	37	Yes	Yes	3rd
Male	5	No	1		20	Yes	Yes	6th
Female	5	Yes	2	108	26	Yes	Yes	8th
Male	6	Yes	7	3	30	No		
Female	5	Yes	3	43	25	No		
Male	14	Yes	3	7	27	No		
Female	14	Yes	4	7	36	No		
Female	14	Yes	6	2	38	No		
Female	9	No	1		21	Yes	Yes	9th
Female	2	Yes	1		18	Yes	Yes	4th
Male	14	Yes	2	7	24	Yes	Yes	10th
Female	14	Yes	2	5	28	No		
Female	3	Yes	4	98	37	No		
Male	14	No	1		21	Yes	Yes	9th
Female	7	No	1		20	Yes	Yes	6th
Male	12	Yes	2	7	28	No		
Male	14	Yes	3	36	28	No		
Female	10	Yes	4	72	28	No		
Female	3	Yes	4	127	38	No		
Female	7	Yes	6	32	32	No		
Male	3	No	1		20	Yes	Yes	9th
Female	2	No	1		19	Yes	Yes	7th
Male	11	Yes	3	3	25	No		
Female	10	Yes	5	29	40	No		
Female	4	No	1		20	No		
Male	10	Yes	2	5	24	Yes	Yes	8th
Male	13	No	1		21	Yes	Yes	7th
Male	14	Yes	3	69	26	Yes	Yes	7th
Female	12	No	1		21	Yes	Yes	10th
Female	14	No	1		22	Yes	Yes	4th
Male	8	No	1		20	No		
Female	8	Yes	2	10	25	No		
Male	5	Yes	4	120	30	No		

For peer review only

Female	8	Yes	0	8	31	No		
Male	2	Yes	7	2	30	No		
Female	14	Yes	10	4	45	No		
Male	6	No	1		19	Yes	Yes	6th
Male	8	Yes	3	6	34	Yes	Yes	2nd
Male	8	Yes	4	54	23	Yes	Yes	5th
Male	9	Yes	3	108	35	No		
Female	7	Yes	2	6	25	Yes	Yes	5th
Female	3	Yes	0	6	25	Yes	Yes	5th
Female	14	Yes	4	9	35	Yes	No	
Female	4	Yes	2	3	23	No		
Male	11	Yes	2	14	28	Yes	Yes	3rd
Female	5	Yes	2	7	26	Yes	Yes	7th
Female	12	Yes	2	4	26	Yes	Yes	7th
Male	14	Yes	3	48	26	Yes	Yes	5th
Female	10	Yes	10	4	45	No		
Male	11	No	1		22	No		
Female	10	No	2	2	25	No		
Male	2	Yes	2	19	22	Yes	Yes	8th
Female	11	No	1		19	Yes	Yes	8th
Male	12	No	1		21	Yes	Yes	10th
Female	13	Yes	4	4	30	No		
Female	4	Yes	5	2	33	No		
Female	11	Yes	8	30	37	No		
Male	6	Yes	2	24	22	Yes	Yes	5th
Female	12	No	1		19	Yes	Yes	8th
Female	13	No	2	1	28	Yes	Yes	10th
Female	2	Yes	3	49	33	No		
Female	9	Yes	2	2	22	No		
Female	14	Yes	5	59	35	No		
Female	8	Yes	4	5	36	No		
Female	10	Yes	3	55	40	No		
Female	3	Yes	4	3	25	No		
Female	3	Yes	4	24	32	No		
Male	9	Yes	4	48	29	Yes	Yes	3rd
Female	7	Yes	5	72	38	No		
Male	13	Yes	3	3	25	No		
Male	13	Yes	6	36	37	No		
Male	14	Yes	4	36	28	Yes	Yes	3rd
Female	11	Yes	3	48	32	No		
Female	10	Yes	5	48	32	No		
Female	4	No	1		20	No		
Male	5	Yes	6	84	30	No		
Male	8	Yes	2	24	25	No		
Male	5	Yes	4	72	40	No		
Male	12	Yes	2	4	28	No		
Female	3	Yes	6	3	40	No		
Male	7	No	1		25	Yes	Yes	5th
Female	14	Yes	3	32	35	No		
Male	12	Yes	3	84	33	No		
Female	2	Yes	2	46	23	No		
Male	14	Yes	3	36	32	No		

For peer review only

Female	12	Yes	7	24	32	No		
Female	3	Yes	3	13	30	No		
Female	11	Yes	7	72	35	No		
Female	8	Yes	3	3	32	No		
Male	4	Yes	6	40	40	No		
Male	8	Yes	3	28	32	No		
Male	6	Yes	5	30	27	No		
Male	5	Yes	7	6	40	No		
Male	2	Yes	9	30	38	No		
Female	8	Yes	6	42	30	No		
Female	14	Yes	3	3	24	No		
Female	12	Yes	3	59	27	Yes	Yes	7th
Female	6	Yes	6	3	40	No		
Male	5	Yes	4	3	27	Yes	Yes	5th
Female	12	Yes	5	36	35	No		
Female	3	Yes	5	2	32	No		
Female	3	Yes	2	48	28	Yes	Yes	6th
Female	5	Yes	7	55	40	No		
Female	9	Yes	6	62	38	No		
Female	7	Yes	7	36	37	No		
Female	5	Yes	4	59	25	No		
Female	12	No	1		23	Yes	Yes	8th
Female	9	Yes	2	72	26	Yes	Yes	10th
Male	3	Yes	7	72	35	No		
Female	13	No	1		25	No		
Female	3	Yes	5	36	32	No		
Male	2	Yes	7	42	35	No		
Female	3	Yes	5	3	35	No		
Male	11	Yes	8	49	35	No		
Female	7	Yes	2	36	28	Yes	Yes	5th
Female	3	Yes	3	36	23	Yes	Yes	10th
Female	6	Yes	4	84	30	No		
Male	6	Yes	5	34	25	No		
Male	7	Yes	2	12	25	Yes	Yes	8th
Male	12	Yes	4	4	35	No		
Female	13	Yes	3	46	27	No		
Male	14	Yes	2	33	32	Yes	Yes	10th
Female	14	Yes	2	26	30	No		
Female	3	Yes	5	48	40	No		
Female	8	Yes	10	32	40	No		
Male	6	Yes	3	24	32	Yes	No	
Male	14	Yes	5	3	37	No		
Male	14	Yes	3	48	28	No		
Male	3	Yes	3	46	33	Yes	Yes	8th
Female	10	No	1		19	Yes	Yes	9th
Male	11	Yes	3	38	29	Yes	Yes	8th
Female	3	Yes	3	3	24	Yes	Yes	7th
Female	9	Yes	6	48	35	No		
Male	12	Yes	2	5	24	Yes	Yes	10th
Male	9	Yes	5	96	42	No		
Male	8	Yes	6	36	36	No		
Female	6	Yes	4	34	34	No		

Male	14	Yes	3	32	31	No		
Female	7	Yes	4	39	32	No		
Male	6	Yes	6	3	38	No		
Female	12	Yes	4	24	25	No		
Male	3	Yes	4	4	27	No		
Male	12	Yes	3	51	36	No		
Male	9	Yes	5	9	37	No		
Male	13	Yes	3	24	25	No		
Male	3	Yes	5	5	33	No		
Male	12	Yes	2	4	28	Yes	Yes	5th
Female	9	No	1		22	No		
Female	5	Yes	5	48	30	No		
Female	12	Yes	4	3	35	No		
Male	14	Yes	3	36	32	Yes	Yes	6th
Female	3	No	1		35	No		
Male	12	Yes	2	3	23	Yes	Yes	7th
Male	11	Yes	6	2	37	No		
Female	4	Yes	4	28	31	No		
Female	4	Yes	2	3	27	No		
Male	3	Yes	6	3	35	No		
Female	8	Yes	2	60	32	No		
Male	7	No	1		19	Yes	Yes	9th
Male	10	Yes	5	9	38	No		
Female	9	No	1		20	Yes	Yes	6th
Male	9	No	1		27	Yes	Yes	10th
Male	4	Yes	5	11	40	Yes	Yes	4th
Male	13	Yes	4	6	40	No		
Male	14	Yes	4	2	40	No		
Male	6	Yes	8	60	40	No		
Female	10	Yes	5	3	28	No		
Female	10	Yes	3	24	32	No		
Male	12	Yes	7	30	38	No		
Male	8	Yes	1		19	Yes	Yes	10th
Male	3	Yes	3	40	27	Yes	Yes	5th
Female	10	Yes	3	72	30	Yes	Yes	8th
Male	8	Yes	9	36	42	No		
Male	8	Yes	2	24	23	No		
Female	12	No	1		32	Yes	Yes	6th
Male	12	Yes	3	6	25	No		
Female	8	Yes	7	35	35	No		
Female	5	Yes	4	34	28	No		
Female	2	Yes	4	36	35	No		
Female	9	Yes	2	47	25	Yes	Yes	6th
Female	4	Yes	4	48	24	No		
Female	10	Yes	5	4	30	No		
Male	3	Yes	6	24	35	No		
Female	8	Yes	3	3	28	No		
Male	6	Yes	2	60	35	No		
Male	13	Yes	4	36	34	No		
Male	11	Yes	5	55	35	No		
Female	11	Yes	5	8	35	Yes	No	
Male	11	Yes	3	86	30	No		

For peer review only

Female	4	Yes	3	999	40	No		
Male	14	Yes	3	72	27	No		
Female	3	Yes	6	36	33	No		
Male	12	Yes	4	26	38	Yes	No	
Male	13	Yes	5	36	28	Yes	Yes	7th
Male	14	Yes	2	48	28	Yes	Yes	9th
Female	2	Yes	8	28	38	No		
Male	12	Yes	6	3	42	No		
Male	8	Yes	3	18	28	Yes	Yes	8th
Male	12	Yes	2	52	28	Yes	Yes	8th
Female	14	Yes	3	4	38	No		
Female	14	No	1		20	Yes	Yes	6th
Male	5	Yes	9	3	43	No		
Female	12	No	1		18	No		
Female	10	Yes	5	3	35	No		
Female	5	Yes	7	36	30	No		
Male	5	Yes	5	50	39	No		
Female	9	Yes	4	48	32	No		
Male	3	No	1		19	Yes	Yes	6th
Female	4	No	1		22	Yes	Yes	6th
Female	3	Yes	4	2	23	Yes	Yes	3rd
Male	7	Yes	3	5	28	No		
Female	11	Yes	3	41	28	No		
Male	3	Yes	5	48	42	No		
Male	12	Yes	4	36	32	Yes	Yes	5th
Female	2	Yes	4	30	29	Yes	Yes	2nd
Female	8	Yes	7	42	35	No		
Male	13	Yes	3	36	28	No		
Male	12	Yes	6	24	36	No		
Female	3	Yes	3	3	30	No		
Female	10	Yes	6	4	35	No		
Female	4	Yes	3	3	35	No		
Male	9	No	1		19	Yes	Yes	10th
Female	7	Yes	2	48	22	Yes	Yes	8th
Male	11	Yes	3	7	30	No		
Female	2	Yes	5	36	30	No		
Male	3	Yes	6	3	36	No		
Male	14	No	4	24	32	No		
Male	3	Yes	4	24	32	No		
Female	13	Yes	3	39	32	No		
Male	3	Yes	4	48	27	Yes	No	
Male	12	No	1		20	Yes	Yes	6th
Female	13	No	1		20	Yes	Yes	10th
Female	3	Yes	5	24	36	Yes	Yes	5th
Female	8	Yes	6	11	38	No		
Male	9	Yes	2	120	34	No		
Male	13	No	1		26	Yes	Yes	University/
Male	14	Yes	2	6	28	Yes	Yes	10th
Female	14	No	1		20	Yes	Yes	8th
Female	12	Yes	3	122	35	Yes	Yes	4th
Male	11	No	1		25	Yes	Yes	10th
Female	8	Yes	6	48	31	No		

For peer review only

Male	14	Yes	3	4	27	No		
Female	6	Yes	4	14	35	No		
Male	4	Yes	4	39	39	Yes	Yes	6th
Female	6	Yes	4	3	33	No		
Female	14	Yes	6	22	45	No		
Female	6	Yes	6	36	37	No		
Female	12	Yes	2	84	27	No		
Female	12	No	1		23	Yes	Yes	8th
Female	12	Yes	2	4	20	No		
Female	5	No	1		19	Yes	Yes	10th
Female	12	Yes	1		22	Yes	Yes	10th
Female	2	Yes	2	10	30	Yes	Yes	University/
Female	6	Yes	2	36	21	Yes	Yes	4th
Female	9	Yes	2	9	28	Yes	Yes	4th
Male	7	Yes	4	27	33	No		
Female	9	No	1		21	Yes	Yes	12th
Male	10	Yes	2	48	19	No		
Female	3	No	1		18	No		
Male	11	Yes	4	36	35	No		
Female	8	Yes	2	2	20	No		
Female	12	Yes	6	17	37	No		
Male	3	No	1		25	Yes	Yes	8th
Female	8	Yes	6	3	32	No		
Male	4	Yes	2	8	26	No		
Male	14	No	1		25	Yes	Yes	10th
Female	14	Yes	4	48	35	No		
Female	13	Yes	5	3	39	No		
Female	10	Yes	3	4	21	Yes	Yes	4th
Male	5	Yes	8	3	40	No		
Female	13	Yes	4	5	38	No		
Female	8	Yes	2	3	18	No		
Female	14	No	1		30	Yes	Yes	6th
Male	13	No	1		20	Yes	Yes	7th
Male	12	No	1		20	Yes	Yes	9th
Female	12	Yes	5	3	28	No		
Female	9	Yes	2	36	25	Yes	Yes	8th
Male	9	Yes	7	36	36	No		
Female	14	Yes	1		22	Yes	Yes	Technical/\
Female	14	Yes	2	10	28	Yes	Yes	8th
Female	2	Yes	2	60	21	Yes	Yes	4th
Male	14	No	1		17	Yes	Yes	6th
Female	10	Yes	5	6	39	Yes	Yes	3rd
Female	6	Yes	6	60	30	No		
Male	3	Yes	4	48	29	Yes	Yes	7th
Male	12	Yes	5	4	30	No		
Male	6	No	1		23	Yes	Yes	5th
Male	9	Yes	2	5	27	Yes	Yes	8th
Female	3	Yes	1		18	Yes	Yes	12th
Male	12	No	1		24	No		
Male	7	Yes	2	48	28	No		
Male	13	Yes	1		22	Yes	Yes	10th
Male	13	Yes	5	4	35	No		

For peer review only

Female	14	Yes	2	3	28	Yes	Yes	7th
Female	9	Yes	1		25	No		
Male	13	Yes	3	24	32	Yes	No	
Male	9	Yes	3	48	31	No		
Female	2	Yes	3	25	28	Yes	Yes	University/
Female	2	No	1		23	Yes	Yes	10th
Female	12	Yes	2	36	21	No		
Male	3	Yes	4	4	32	Yes	Yes	10th
Male	4	Yes	5	3	40	No		
Male	3	Yes	4	3	30	No		
Female	2	Yes	6	13	38	No		
Female	5	Yes	2	4	28	Yes	Yes	3rd
Female	10	Yes	6	4	30	No		
Female	11		6	3	35	No		
Male	14	No	3	96	32	No		
Female	5	No	1		32	Yes	Yes	9th
Male	3	Yes	1		19	Yes	Yes	5th
Female	14	Yes	5	4	35	No		
Female	14	Yes	1		20	No		
Male	5	Yes	1		19	No		
Female	12	No	1		20	Yes	Yes	7th
Male	13	Yes	1		20	Yes	Yes	10th
Male	12	Yes	3	5	20	Yes	Yes	7th
Male	9	Yes	4	60	27	No		
Male	7	Yes	7	32	35	No		
Female	14	Yes	6	49	35	No		
Female	3	Yes	2	4	29	Yes	Yes	10th
Female	10	No	1		19	Yes	Yes	8th
Female	11	No	2	44	31	No		
Male	7	Yes	6	36	38	No		
Female	13	Yes	2	60	23	Yes	Yes	6th
Female	14	No	1		24	Yes	Yes	8th
Male	8	Yes	4	22	38	Yes	No	
Female	2	No	1		17	Yes	Yes	4th
Female	3	No	1		32	Yes	Yes	7th
Female	12	Yes	3	5	30	No		
Female	13	Yes	1		21	Yes	Yes	4th
Female	9	Yes	1		20	Yes	Yes	10th
Female	14	Yes	4	3	26	Yes	Yes	4th
Female	14	Yes	3	4	25	Yes	Yes	5th
Male	10	No	1		25	No		
Female	7	No	1		23	Yes	Yes	8th
Male	13	Yes	6	59	34	No		
Female	11		1		20	Yes	Yes	6th
Male	10	Yes	5	3	35	No		
Male	12	No	1		23	Yes	Yes	5th
Male	7	No	1		28	No		
Female	10	No	1		20	Yes	Yes	6th
Male	7	No	1		19	Yes	Yes	3rd
Female	5	No	1		21	Yes	Yes	8th
Female	4	Yes	5	84	39	No		
Male	7	No	1		20	No		

For peer review only

Female	14	Yes	2	60	32	No		
Female	14	No	1		28	Yes	Yes	University/
Male	11	Yes	5	5	39	No		
Male	10	Yes	2	5	18	Yes	Yes	8th
Female	12	Yes	2	24	20	No		
Female	14	Yes	2	41	22	Yes	Yes	7th
Male	5	Yes	2	6	27	No		
Male	4	Yes	3	5	30	No		
Male	2	Yes	3	5	28	No		
Female	4	Yes	3	53	26	Yes	Yes	8th
Female	14	Yes	2	7	29	Yes	Yes	10th
Female	8	Yes	1		22	Yes	Yes	4th
Male	3	Yes	2	60	32	No		
Male	8	Yes	5	36	27	No		
Female	10	No	1		21	Yes	Yes	6th
Female	14	Yes	1		21	No		
Female	10	Yes	1		23	Yes	Yes	6th
Female	14	Yes	2	64	20	No		
Male	5	No	0	0	20	Yes	Yes	7th
Male	14	No	1		25	Yes	Yes	6th
Female	5	Yes	1		20	No		
Male	12	Yes	2	65	28	Yes	Yes	6th
Female	11	Yes	2	5	30	Yes	Yes	10th
Female	12	Yes	3	5	24	No		
Male	9	Yes	6	59	31	No		
Female	14	Yes	2	6	20	Yes	Yes	7th
Female	3	No	1		19	Yes	Yes	6th
Female	14	Yes	2	13	36	Yes	Yes	10th
Male	5	Yes	5	7	42	Yes	Yes	2nd
Male	14	Yes	2	8	35	Yes	Yes	8th
Female	13	Yes	3	9	35	No		
Female	13	Yes	1		20	Yes	Yes	8th
Male	8	Yes	3	6	35	No		
Female	9	Yes	6	62	41	No		
Female	11	Yes	6	6	35	No		
Female	8	No	1		17	No		
Male	7	Yes	5	96	40	No		
Male	9	Yes	2	120	30	Yes	Yes	5th
Female	12	Yes	11	26	42	No		
Male	7	Yes	6	19	37	No		
Male	3	Yes	3	10	30	Yes	Yes	8th
Male	8	Yes	5	72	28	No		
Male	5	Yes	5	42	39	No		
Female	4	Yes	4	36	32	No		
Female	9	Yes	3	12	35	Yes	Yes	6th
Male	3	Yes	7	3	38	No		
Female	13	Yes	2	48	30	No		
Female	3	Yes	2	108	31	No		
Female	14	Yes	6	59	34	No		
Female	14	Yes	2	5	25	Yes	Yes	4th
Male	11	No	1		20	Yes	Yes	9th
Female	9	Yes	2	18	28	Yes	Yes	6th

For peer review only

Male	3	Yes	2	10	28	Yes	Yes	7th
Female	12	Yes	3	135	35	No		
Female	14	Yes	3	60	27	No		
Female	9	Yes	7	60	37	No		
Male	7	Yes	2	2	28	Yes	Yes	6th
Male	9	Yes	3	25	28	No		
Female	6	Yes	2	72	24	No		
Female	3	Yes	4	28	32	No		
Male	11	Yes	6	3	35	No		
Female	10	Yes	1		27	Yes	Yes	7th
Female	10	Yes	2	36	23	Yes	Yes	7th
Male	9	No	1		25	Yes	Yes	8th
Female	8	No	1		19	No		
Female	9	No	1		23	Yes	Yes	5th
Female	11	Yes	4	6	34	No		
Male	4	Yes	2	7	28	Yes	Yes	8th
Female	5	Yes	5	40	30	No		
Female	11	Yes	6	4	35	No		
Female	5		1		17	Yes	Yes	5th
Male	11	Yes	2	34	26	Yes	Yes	6th
Male	11	Yes	4	37	30	No		
Male	3	Yes	2	80	26	Yes	Yes	10th
Male	13	No	7	3	38	No		
Male	13	Yes	4	120	30	No		
Male	13	Yes	4	36	35	No		
Female	10	No	2	7	20	Yes	Yes	8th
Female	4	Yes	2	36	28	Yes	Yes	10th
Male	5	No	6	6	29	No		
Female	8	Yes	3	3	25	No		
Female	14	Yes	2	14	30	No		
Male	13	No	3	8	35	No		
Male	4	Yes	2	60	22	Yes	Yes	7th
Female	5	No	1		26	Yes	Yes	8th
Male	6	No	5	7	35	Yes	No	
Male	10	Yes	3	9	35	No		
Male	9	No	2	5	27	No		
Male	13	Yes	3	72	32	No		
Female	14	No	1		22	Yes	Yes	7th
Male	14	No	1		30	No		
Male	11	Yes	4	59	30	No		
Female	14	Yes	8	4	42	Yes	Yes	3rd
Female	11	Yes	5	36	33	Yes	No	
Male	6	No	4	3	30	No		
Male	8	No	6	5	35	No		
Female	12	Yes	5	4	33	No		
Male	13	No	1		28	Yes	Yes	9th
Male	12	No	5	5	33	No		
Male	14	Yes	3	96	20	Yes	Yes	6th
Female	9	No	2	5	26	Yes	Yes	7th
Male	14	Yes	2	4	25	Yes	Yes	Technical/\
Male	12	No	1		24	Yes	Yes	6th
Female	2	No	1		20	Yes	Yes	5th

For peer review only

Male	6	Yes	6	24	35	No		
Male	14	No	1		24	No		
Male	12	No	4	5	29	Yes	Yes	6th
Male	4	Yes	4	58	25	Yes	Yes	2nd
Female	3	Yes	3	24	28	Yes	Yes	4th
Female	13	No	1		21	No		
Male	6	Yes	3	3	26	Yes	Yes	3rd
Male	4	No	2	7	35	No		
Female	7	Yes	6	48	28	Yes	No	
Female	3	No	1		20	Yes	Yes	10th
Male	5	Yes	1		22	Yes	Yes	12th
Female	9	Yes	3	999	30	Yes	No	
Male	3	No	1		19	Yes	Yes	7th
Female	6	Yes	7	36	38	No		
Male	6	No	1		20	Yes	Yes	10th
Male	8	No	3	6	30	Yes	Yes	6th
Female	3	Yes	2	72	30	No		
Male	5	Yes	7	38	37	No		
Female	7	No	1		27	Yes	Yes	10th
Female	3	Yes	12	4	35	No		
Male	14	No	4	6	30	No		
Male	14	Yes	1		28	Yes	Yes	6th
Female	14	No	1		20	Yes	Yes	6th
Female	11	Yes	5	40	32	Yes	Yes	1st
Female	12	No	1		26	No		
Female	3	Yes	2	24	26	Yes	Yes	10th
Female	12	No	1		18	Yes	Yes	5th
Female	12	No	1		21	Yes	Yes	6th
Male	9	Yes	4	49	28	No		
Male	4	Yes	4	36	34	No		
Female	3	No	1		21	Yes	Yes	Technical/[illegible]
Female	14	No	1		26	Yes	Yes	8th
Male	4	No	1		22	Yes	Yes	8th
Male	10	Yes	3	26	23	Yes	Yes	5th
Female	9	No	5	7	35	No		
Female	3	Yes	5	42	35	No		
Female	11	Yes	4	36	30	No		
Male	11	No	1		19	Yes	Yes	12th
Male	6	No	1		25	Yes	Yes	9th
Female	4	Yes	5	35	33	No		
Female	5	Yes	7	41	35	No		
Male	14	Yes	3	6	32	No		
Female	14	No	1		19	Yes	Yes	8th
Male	11	Yes	4	3	25	Yes	Yes	5th
Female	6	Yes	2	51	27	Yes	Yes	10th
Male	5	No	1		24	Yes	Yes	10th
Female	5	Yes	2	59	28	No		
Female	14	Yes	3	96	28	No		
Male	10	Yes	1		21	Yes	Yes	9th
Male	12	Yes	3	48	30	No		
Male	4	No	1		22	No		
Female	8	No	5	4	35	Yes	Yes	3rd

For peer review only

Male	13	Yes	4	999	30	No		
Female	4	No	4	3	30	Yes	Yes	5th
Male	9	Yes	5	96	35	No		
Male	8	Yes	4	8	37	No		
Female	11	No	1		20	Yes	Yes	9th
Female	11	No	2	2	25	No		
Female	5	Yes	7	999	38	No		
Female	4	Yes	4	60	25	No		
Male	5	Yes	2	59	40	No		
Male	13	No	1		23	Yes	Yes	8th
Female	3	Yes	5	41	38	No		
Male	5	No	3	14	40	No		
Female	10	No	1		22	Yes	Yes	6th
Female	3	Yes	1		18	Yes	Yes	Technical/\
Male	12	Yes	2	48	20	No		
Female	3	No	1		22	Yes	Yes	8th
Male	4	Yes	1		30	No		
Female	7	No	1		19	No		
Female	11	Yes	4	999	28	No		
Female	6	Yes	2	999	25	Yes	Yes	6th
Female	14	Yes	1		37	No		
Female	6	Yes	6	36	30	Yes	Yes	1st
Female	11	Yes	1		28	Yes	Yes	6th
Female	14	No	1		27	Yes	Yes	12th
Male	13	Yes	2	2	25	No		
Male	7	Yes	6	26	30	Yes	No	
Male	4	Yes	4	2	30	No		
Female	3	No	1		20	Yes	Yes	7th
Female	7	No	3	5	20	No		
Female	10	No	7	24	30	Yes	No	
Female	14	Yes	2	14	29	No		
Male	8	Yes	3	999	36	No		
Male	10	Yes	4	36	30	Yes	Yes	3rd
Male	5	Yes	2	36	25	Yes	Yes	9th
Female	13	No	1		27	No		
Male	11	Yes	7	42	35	No		
Female	8	Yes	6	39	38	No		
Male	14	Yes	4	108	34	No		
Male	3	Yes	4	38	32	Yes	Yes	2nd
Male	13	Yes	7	42	38	No		
Female	14	Yes	4	999	40	No		
Male	4	No	6	4	35	No		
Male	8	No	1		20	Yes	Yes	11th
Male	3	Yes	2	64	25	No		
Male	14	No	6	3	27	No		
Female	13	Yes	4	48	35	Yes	Yes	2nd
Female	3	Yes	7	4	35	No		
Female	6	No	7	3	39	No		
Female	5	Yes	3	48	28	Yes	Yes	8th
Male	13	No	1		30	Yes	Yes	4th
Male	11	No	6	4	40	Yes	Yes	4th
Female	14	No	1		24	Yes	Yes	10th

For peer review only

Male	8	Yes	6	4	33	No		
Female	14	Yes	2	144	30	Yes	Yes	2nd
Male	14	Yes	11	36	39	No		
Female	10	No	6	3	30	No		
Female	7	Yes	2	7	18	Yes	Yes	9th
Female	7	Yes	5	46	35	Yes	Yes	6th
Female	12	Yes	1		19	Yes	Yes	11th
Male	14	Yes	10	36	35	No		
Male	11	Yes	3	48	28	Yes	No	
Female	7	No	1		19	Yes	Yes	4th
Male	7	Yes	5	40	28	Yes	Yes	2nd
Female	10	No	1		25	Yes	Yes	10th
Male	11	Yes	3	120	32	Yes	No	
Female	14	No	1		28	No		
Male	14	Yes	4	4	27	Yes	Yes	6th
Male	6	No	1		20	Yes	Yes	12th
Male	8	Yes	7	999	40	No		
Male	12	Yes	8	33	40	No		
Female	3	Yes	4	2	28	No		
Female	3	Yes	1		18	No		
Male	11	Yes	10	48	42	No		
Female	10	Yes	3	60	34	No		
Female	9	Yes	1		25	Yes	Yes	6th
Female	7	Yes	2	48	28	No		
Female	13	No	5	7	30	No		
Male	3	Yes	6	32	36	No		
Female	14	Yes	5	60	35	No		
Male	5	Yes	6	23	34	Yes	Yes	4th
Female	12	Yes	2	6	30	Yes	Yes	8th
Male	13	Yes	6	59	35	No		
Male	6	No	1		26	Yes	Yes	University/
Female	12	No	1		25	Yes	Yes	8th
Female	14	Yes	2	44	29	Yes	No	
Female	11	Yes	5	60	40	No		
Female	9	Yes	3	86	28	No		
Female	11	No	4	3	28	No		
Male	14	Yes	2	62	25	Yes	Yes	6th
Male	14	Yes	3	48	30	No		
Male	3	No	1		20	Yes	Yes	6th
Female	9	No	1		33	Yes	Yes	10th
Female	6	Yes	7	50	45	No		
Male	4	Yes	3	36	28	Yes	Yes	6th
Male	7	Yes	10	7	35	Yes	Yes	2nd
Female	14	No	1		18	Yes	Yes	4th
Female	7	No	1		24	No		
Female	9	No	2	4	21	Yes	Yes	6th
Male	4	Yes	2	43	23	Yes	Yes	6th
Male	6	Yes	7	48	35	No		
Female	6	No	2	8	20	Yes	Yes	6th
Female	3	Yes	2	120	25	No		
Female	14	Yes	5	999	33	No		
Female	3	Yes	2	46	22	Yes	Yes	5th

For peer review only

Female	13	Yes	6	4	39	No		
Male	12	No	1		22	Yes	Yes	5th
Female	4	No	1		19	Yes	Yes	5th
Female	13	No	2	4	23	Yes	Yes	8th
Male	4	No	3	8	25	No		
Female	8	No	1		20	Yes	Yes	4th
Male	13	No	1		23	Yes	Yes	10th
Male	11	No	1		24	Yes	Yes	3rd
Female	10	Yes	2	144	30	No		
Male	13	Yes	3	6	30	Yes	Yes	4th
Male	11	Yes	3	72	28	Yes	Yes	3rd
Female	6	No	1		23	Yes	Yes	9th
Male	9	No	1		20	Yes	Yes	6th
Male	13	Yes	2	36	22	No		
Female	7	Yes	5	42	30	No		
Female	14	No	1		25	Yes	Yes	8th
Male	8	No	3	3	23	No		
Female	4	Yes	3	20	35	No		
Male	6	Yes	3	48	30	No		
Female	4	Yes	2	48	21	Yes	Yes	5th
Male	8	No	3	999	32	No		
Male	5	No	1		24	Yes	Yes	10th
Female	3	No	2	48	25	Yes	Yes	6th
Female	10	Yes	7	999	30	No		
Male	9	No	1		24	Yes	Yes	8th
Female	13	No	2	7	30	No		
Female	2	No	1		20	No		
Female	12	Yes	4	60	27	No		
Male	12	Yes	3	60	22	Yes	Yes	5th
Male	3	Yes	5	36	30	No		
Female	2	No	3	7	30	Yes	Yes	6th
Female	9	No	1		17	Yes	Yes	7th
Female	13	Yes	2	59	23	Yes	Yes	8th
Female	6	No	1		26	Yes	Yes	Technical/\
Female	6	Yes	5	28	30	No		
Male	11	No	5	4	32	No		
Female	4	Yes	2	999	25	No		
Female	9	No	1		23	Yes	Yes	10th
Female	6	No	8	3	30	No		
Male	4	Yes	5	999	35	No		
Male	12	No	3	10	30	No		
Female	10	No	1		20	Yes	Yes	8th
Female	4	No	3	6	35	No		
Female	7	No	1		19	No		
Male	11	Yes	4	36	30	No		
Male	6	No	1		22	Yes	Yes	University/
Female	5	Yes	8	36	36	No		
Female	12	No	1		24	No		
Female	11	Yes	3	999	35	No		
Female	10	No	1		22	Yes	Yes	6th
Male	11	Yes	5	999	35	No		
Male	12	Yes	1		20	Yes	Yes	6th

For peer review only

Male	6	Yes	5	48	36	Yes	Yes	6th
Male	14	Yes	2	39	25	Yes	Yes	Technical/\
Male	3	Yes	2	60	30	No		
Female	6	Yes	1		22	Yes	Yes	10th
Male	10	No	1		21	Yes	Yes	8th
Female	7	No	1		27	Yes	Yes	10th
Female	6	Yes	2	26	25	Yes	Yes	3rd
Male	3	No	2	4	21	Yes	Yes	6th
Male	3	Yes	2	60	28	Yes	Yes	10th
Male	3	Yes	3	6	28	No		
Male	8	Yes	2	59	20	No		
Male	14	Yes	6	60	39	No		
Male	10	No	2	8	30	Yes	Yes	4th
Male	4	Yes	2	96	28	No		
Male	14	No	1		24	Yes	Yes	8th
Female	4	Yes	5	999	35	No		
Female	4	No	1		22	Yes	Yes	5th
Male	8	Yes	2	48	22	No		
Male	12	Yes	2	60	23	Yes	Yes	10th
Female	11	No	1		23	No		
Female	9	Yes	3	38	32	No		
Male	6	Yes	7	59	36	No		
Male	13	No	2	4	26	No		
Male	3	Yes	4	48	28	Yes	Yes	5th
Male	14	No	2	5	30	No		
Female	10	Yes	3	36	26	Yes	Yes	4th
Female	6	No	4	2	27	No		
Male	3	Yes	3	24	25	Yes	Yes	8th
Female	10	Yes	4	54	29	No		
Male	7	Yes	2	36	26	Yes	Yes	7th
Female	9	Yes	5	999	32	No		
Male	13	No	2	4	27	Yes	Yes	10th
Female	12	No	1		20	Yes	Yes	6th
Female	4	Yes	7	36	35	Yes	Yes	1st
Male	14	Yes	8	47	38	No		
Female	4	Yes	5	24	30	No		
Female	13	Yes	6	999	30	No		
Male	9	No	5	4	30	No		
Male	9	Yes	2	12	29	No		
Female	5	Yes	3	60	35	No		
Male	6	No	3	3	26	No		
Male	4	No	2	7	25	Yes	Yes	8th
Male	4	Yes	6	36	32	No		
Male	4	Yes	2	60	40	No		
Female	14	No	1		24	Yes	Yes	8th
Female	14	Yes	5	48	35	No		
Male	6	No	1		21	Yes	No	
Male	10	Yes	2	70	35	No		
Female	4	No	3	3	23	No		
Male	3	Yes	2	48	22	No		
Male	10	No	1		23	No		
Female	12	Yes	4	48	30	Yes	No	

For peer review only

Female	13	No	5	4	38	No		
Female	12	Yes	2	36	25	Yes	Yes	9th
Male	3	Yes	5	36	35	No		
Male	4	Yes	3	36	29	Yes	Yes	7th
Female	10	Yes	1		28	Yes	Yes	9th
Female	14	No	1		20	Yes	Yes	8th
Male	6	Yes	2	34	26	Yes	Yes	10th
Female	14	Yes	5	48	25	Yes	Yes	3rd
Female	3	Yes	5	48	38	No		
Female	3	Yes	2	22	28	Yes	Yes	8th
Female	8	No	4	4	30	No		
Female	9	No	1		18	Yes	Yes	7th
Male	6	Yes	6	48	35	No		
Male	3	Yes	4	999	45	No		
Male	5	Yes	2	36	32	No		
Female	8	No	4	3	27	Yes	Yes	5th
Male	12	Yes	5	34	28	No		
Female	12	Yes	7	60	39	No		
Female	6	Yes	4	3	30	No		
Male	4	Yes	4	4	30	Yes	Yes	6th
Male	12	Yes	2	36	19	Yes	Yes	6th
Male	4	Yes	7	3	30	No		
Male	5	Yes	5	45	35	No		
Male	9	Yes	6	6	35	No		
Female	8	Yes	3	10	27	No		
Male	14	Yes	6	9	32	No		
Female	8	Yes	5	60	40	No		
Female	3	Yes	5	36	32	No		
Female	3	No	1		22	Yes	Yes	6th
Male	9	Yes	3	42	32	No		
Male	12	Yes	5	5	38	No		
Female	12	Yes	2	3	20	No		
Female	14	Yes	2	48	28	Yes	Yes	12th
Female	7	Yes	4	3	28	Yes	Yes	9th
Female	9	Yes	4	72	33	No		
Female	6	Yes	4	52	35	No		
Female	8	Yes	3	36	28	Yes	Yes	6th
Male	14	Yes	6	4	37	No		
Female	6	Yes	5	18	35	Yes	Yes	8th
Male	14	No	1		26	Yes	Yes	9th
Female	9	Yes	5	5	30	No		
Male	14	Yes	5	36	35	No		
Male	12	Yes	5	44	35	No		
Male	9	Yes	4	3	30	No		
Male	13	Yes	4	4	35	No		
Male	11	Yes	2	5	28	No		
Male	13	Yes	4	48	28	No		
Male	13	Yes	8	84	42	No		
Male	13	Yes	4	48	30	No		
Male	14	Yes	5	5	30	No		
Male	6	Yes	3	3	30	No		
Male	3	No	1		22	Yes	Yes	5th

For peer review only

Female	5	Yes	3	14	35	No		
Male	6	Yes	8	25	38	No		
Male	3	Yes	2	10	28	Yes	Yes	7th
Female	11	Yes	7	48	40	No		
Female	6	No	1		28	Yes	Yes	5th
Male	4	Yes	2	42	23	Yes	Yes	10th
Male	14	Yes	3	48	25	No		
Male	14	Yes	2	48	25	No		
Female	4	Yes	4	3	28	No		
Female	10	No	1		18	Yes	Yes	5th
Female	8	Yes	10	7	46	No		
Male	13	No	1		23	Yes	Yes	10th
Male	12		5	4	28	No		
Male	6	Yes	3	3	25	No		
Male	6	Yes	3	2	25	Yes	Yes	5th
Male	9	Yes	4	4	30	No		
Female	4	Yes	2	4	23	No		
Male	3	Yes	3	36	28	No		
Female	4	Yes	2	6	22	No		
Female	9	Yes	5	7	28	No		
Male	8	Yes	4	3	42	No		
Female	3	Yes	5	46	33	No		
Female	13	Yes	6	56	35	No		
Female	11	No	1		20	No		
Female	9	Yes	5	5	30	No		
Male	4	Yes	3	10	42	No		
Male	3	Yes	5	24	35	No		
Male	14	Yes	5	4	30	No		
Male	13	Yes	5	5	25	Yes	Yes	5th
Female	14	Yes	5	37	35	No		
Male	9	No	1		26	Yes	Yes	10th
Female	9	Yes	7	4	39	No		
Female	3	Yes	5	5	38	No		
Female	4	Yes	9	36	38	No		
Male	12	Yes	6	48	38	No		
Female	8	No	1		22	Yes	Yes	3rd
Male	14	Yes	3	30	32	No		
Female	14	Yes	3	7	30	No		
Female	5	Yes	5	3	33	No		
Male	13	Yes	4	3	28	Yes	Yes	4th
Female	8	No	1		28	Yes	Yes	10th
Female	6	Yes	2	6	25	Yes	Yes	9th
Male	14	Yes	2	45	23	Yes	Yes	9th
Male	3	Yes	6	27	35	No		
Male	14	Yes	7	4	37	No		
Male	11	Yes	8	40	20	Yes	Yes	7th
Female	7	No	1		21	No		
Female	3	Yes	5	36	36	No		
Male	10	Yes	4	34	30	Yes	No	
Female	14	Yes	3	9	26	Yes	Yes	7th
Male	5	Yes	5	34	38	No		
Male	14	Yes	6	5	35	No		

For peer review only

Male	9	Yes	9	5	38	Yes	Yes	2nd
Female	14	Yes	5	7	35	No		
Male	3	Yes	2	48	25	Yes	Yes	6th
Female	7	Yes	6	84	42	No		
Female	12	No	1		25	Yes	Yes	University/
Male	6	Yes	4	37	37	No		
Female	6	Yes	2	3	26	Yes	Yes	6th
Female	13	No	1		22	Yes	Yes	5th
Male	4	Yes	9	3	37	No		
Female	8	Yes	3	36	30	No		
Female	12	Yes	2	40	22	Yes	Yes	8th
Female	2	No	2	3	20	No		
Female	8	No	1		28	Yes	Yes	12th
Female	2	Yes	2	6	35	No		
Male	13	Yes	8	3	40	No		
Female	9	No	1		25	Yes	Yes	10th
Male	10	Yes	3	21	36	No		
Male	7	Yes	3	10	35	No		
Male	12	Yes	5	36	35	No		
Male	14	Yes	5	4	35	No		
Female	8	Yes	2	5	24	Yes	Yes	4th
Female	9	Yes	6	4	36	No		
Female	3	Yes	2	26	26	No		
Female	5	Yes	4	39	27	No		
Female	6	Yes	5	4	35	No		
Male	8	Yes	7	3	40	No		
Male	8	Yes	5	4	35	No		
Male	3	Yes	8	3	40	No		
Female	12	No	1		25	Yes	Yes	10th
Male	6	Yes	4	168	36	No		
Female	13	Yes	4	5	35	No		
Female	6	Yes	3	3	32	Yes	Yes	10th
Male	6	No	1		25	Yes	Yes	10th
Female	12	No	1		25	No		
Female	6	Yes	2	46	28	No		
Male	8	Yes	2	4	22	Yes	Yes	8th
Female	5	Yes	7	3	38	No		
Female	10	Yes	6	4	35	No		
Male	4	Yes	8	36	38	No		
Female	4	Yes	6	8	38	No		
Female	11	Yes	5	4	35	No		
Male	3	Yes	3	6	25	No		
Male	3	No	1		25	Yes	Yes	9th
Male	5	Yes	2	5	28	Yes	Yes	9th
Female	10	Yes	8	36	38	No		
Male	5	Yes	5	5	34	No		
Female	3	Yes	7	4	38	No		
Female	4	Yes	2	36	24	Yes	Yes	9th
Male	14	No	0	0	32	Yes	Yes	9th
Male	14	Yes	3	6	30	No		
Female	9	No	1		26	No		
Male	6	No	1		23	Yes	Yes	6th

For peer review only

Male	14	No	2	144	28	No		
Female	9	Yes	8	8	35	No		
Female	7	Yes	9	36	42	Yes	Yes	4th
Female	14	Yes	3	3	27	Yes	Yes	10th
Female	10	Yes	5	72	38	No		
Female	6	No	1		27	Yes	Yes	10th
Male	10	Yes	4	4	30	Yes	Yes	6th
Male	6	Yes	4	3	30	No		
Female	11	Yes	5	48	40	No		
Male	9	Yes	4	6	32	No		
Female	9	Yes	4	48	32	Yes	Yes	8th
Male	4	Yes	2	20	20	Yes	Yes	6th
Male	14	Yes	4	4	32	No		
Male	11	Yes	4	49	30	No		
Male	11	Yes	6	4	38	No		
Female	6	Yes	3	60	28	Yes	Yes	5th
Male	13	No	0	0	22	Yes	Yes	7th
Female	14	Yes	2	2	28	No		
Female	9	Yes	4	5	37	No		
Male	14	No	0	0	19	Yes	Yes	8th
Female	12	Yes	6	60	35	No		
Male	10	Yes	6	48	37	No		
Male	3	Yes	2	2	26	Yes	Yes	5th
Female	5	Yes	6	4	40	No		
Female	9	Yes	3	70	33	No		
Male	13	Yes	3	4	30	Yes	Yes	3rd
Male	6	Yes	2	36	27	Yes	Yes	4th
Male	3	No	1		20	Yes	Yes	5th
Female	14	Yes	2	48	28	Yes	Yes	8th
Female	14	Yes	5	64	40	No		
Male	11	Yes	6	4	35	Yes	Yes	4th
Female	3	Yes	5	30	38	No		
Female	10	Yes	6	72	40	No		
Female	6	Yes	4	48	36	No		
Male	14	Yes	3	7	30	No		
Female	13	Yes	6	60	37	No		
Male	7	Yes	3	48	28	No		
Male	14	No	4	5	30	No		
Female	8	Yes	3	5	30	No		
Female	11	No	1		26	Yes	Yes	5th
Female	11	No	5	4	29	No		
Male	14	Yes	2	15	36	Yes	Yes	7th
Female	6	Yes	4	36	25	Yes	Yes	8th
Female	11	Yes	5	34	35	No		
Male	14	No	1		27	No		
Male	14	No	1		20	Yes	Yes	8th
Male	9	Yes	4	48	40	No		
Male	14	Yes	4	40	35	No		
Male	13	Yes	2	5	25	No		
Male	8	Yes	6	999	41	No		
Female	3	Yes	5	2	28	No		
Male	14	No	1		20	Yes	Yes	5th

For peer review only

Female	13	Yes	1		24	Yes	Yes	10th
Male	11	Yes	6	72	35	No		
Male	2	Yes	5	5	30	No		
Female	11	No	1		23	Yes	Yes	4th
Male	5	No	1		23	Yes	Yes	10th
Female	12	Yes	6	3	35	No		
Female	11	No	1		20	No		
Male	9	Yes	1		22	Yes	Yes	5th
Male	3	No	1		20	Yes	Yes	6th
Male	4	Yes	6	48	40	No		
Male	14	Yes	3	76	35	Yes	Yes	7th
Male	14	No	6	6	35	No		
Male	9	Yes	8	37	40	No		
Female	6	Yes	4	999	38	No		
Female	13	Yes	4	48	25	No		
Male	6	Yes	8	4	42	No		
Female	8	Yes	8	36	35	No		
Male	8	No	6	5	40	No		
Female	3	Yes	2	59	28	No		
Female	7	No	1		19	Yes	Yes	7th
Male	3	Yes	2	28	25	Yes	Yes	10th
Male	12	Yes	5	40	35	No		
Male	9	Yes	2	120	23	No		
Male	9	Yes	4	7	27	No		
Male	14	Yes	7	36	25	No		
Female	5	Yes	4	36	35	No		
Male	4	Yes	3	30	30	Yes	Yes	8th
Male	9	Yes	2	48	23	Yes	Yes	5th
Male	12	Yes	4	48	32	No		
Male	6	No	1		22	Yes	Yes	7th
Male	2	Yes	7	24	37	Yes	No	
Female	12	Yes	4	7	35	No		
Male	9	No	1		24	Yes	Yes	10th
Male	9	Yes	6	36	36	No		
Male	3	Yes	6	33	30	No		
Male	8	Yes	3	6	28	Yes	Yes	7th
Male	8	Yes	7	59	40	Yes	Yes	4th
Male	6	Yes	3	3	23	Yes	Yes	6th
Male	3	Yes	7	4	37	No		
Male	3	Yes	3	6	25	Yes	Yes	8th
Male	11	Yes	4	3	29	No		
Male	11	Yes	6	72	32	No		
Female	10	No	4	5	30	No		
Female	14	Yes	1		26	Yes	Yes	3rd
Male	14	Yes	2	10	25	No		
Male	5	No	1		24	Yes	Yes	6th
Male	7	Yes	4	48	25	No		
Male	14	Yes	4	60	25	No		
Female	8	Yes	4	48	39	No		
Female	12	Yes	6	48	30	No		
Female	13	Yes	4	40	35	No		
Male	4	No	5	6	30	No		

For peer review only

Female	14	Yes	7	40	40	No		
Male	3	Yes	11	2	40	No		
Female	10	Yes	5	192	42	No		
Male	4	Yes	4	36	32	No		
Male	9	No	2	3	27	No		
Female	5	Yes	4	36	27	No		
Female	6	No	1		25	Yes	Yes	4th
Male	5	Yes	4	59	30	No		
Female	13	Yes	7	4	30	Yes	Yes	5th
Female	7	Yes	5	34	30	No		
Female	9	Yes	4	96	34	No		
Female	3	Yes	5	72	30	Yes	No	
Female	12	Yes	5	132	29	No		
Male	5	Yes	2	7	24	Yes	Yes	7th
Male	4	Yes	4	26	30	No		
Female	10	Yes	4	36	29	No		
Female	4	Yes	6	36	35	No		
Male	9	Yes	6	48	38	No		
Male	8	Yes	5	3	30	Yes	Yes	7th
Male	4	Yes	1		20	Yes	Yes	7th
Female	6	Yes	5	52	30	Yes	Yes	7th
Male	14	No	4	5	30	No		
Male	3	Yes	2	3	22	Yes	Yes	9th
Male	6	Yes	4	4	30	Yes	Yes	8th
Male	4	Yes	3	999	25	Yes	Yes	8th
Female	11	Yes	8	5	38	Yes	No	
Female	11	No	2	6	27	Yes	Yes	7th
Male	5	Yes	3	13	34	No		
Female	6	Yes	3	34	30	Yes	Yes	7th
Male	3	Yes	3	3	25	Yes	Yes	8th
Male	14	Yes	1		20	Yes	Yes	10th
Female	7	Yes	3	96	29	No		
Female	8	Yes	2	9	25	Yes	Yes	7th
Female	3	Yes	3	9	30	Yes	Yes	10th
Male	3	Yes	1		26	Yes	Yes	10th
Male	5	Yes	5	52	30	No		
Male	5	No	5	2	30	No		
Male	13	Yes	3	104	38	No		
Female	6	No	1		19	Yes	Yes	8th
Female	14	Yes	6	3	30	No		
Female	3	Yes	1		20	Yes	Yes	10th
Female	12	No	5	3	32	No		
Male	12	No	1		23	Yes	Yes	7th
Female	6	No	1		25	Yes	Yes	10th
Male	7	Yes	4	47	30	No		
Male	11	Yes	8	42	39	No		
Male	3	Yes	1		22	Yes	Yes	5th
Male	12	Yes	4	15	28	No		
Female	8	Yes	7	4	35	No		
Male	14	Yes	1		28	Yes	Yes	8th
Female	7	Yes	6	6	42	No		
Female	11	No	1		20	Yes	Yes	7th

For peer review only

Male	9	No	1		21	Yes	Yes	8th
Male	9	Yes	6	37	35	No		
Female	14	Yes	2	7	23	Yes	No	
Male	14	Yes	2	49	28	Yes	Yes	6th
Female	12	Yes	2	120	33	No		
Male	8	Yes	4	48	32	Yes	Yes	3rd
Male	6	Yes	4	2	30	No		
Male	5	Yes	2	25	22	No		
Female	12	Yes	2	84	28	Yes	Yes	7th
Male	12	Yes	3	10	30	No		
Male	9	Yes	4	46	32	No		
Female	14	Yes	7	48	35	No		
Female	11	Yes	5	48	37	No		
Female	10	Yes	2	72	25	Yes	Yes	8th
Female	12	Yes	3	56	28	No		
Male	8	Yes	2	84	25	Yes	Yes	7th
Male	4	Yes	7	48	30	No		
Female	11	Yes	3	4	30	No		
Female	10	Yes	7	48	35	No		
Female	12	Yes	4	4	37	No		
Female	8	Yes	3	35	32	Yes	Yes	10th
Male	12	Yes	2	101	29	No		
Male	12	Yes	4	4	30	No		
Female	12	Yes	6	3	40	Yes	Yes	4th
Male	2	Yes	5	84	38	No		
Male	9	Yes	5	48	40	No		
Male	12	Yes	2	72	25	No		
Female	10	No	2	6	25	Yes	Yes	8th
Female	14	No	1		29	Yes	Yes	7th
Male	3	Yes	4	84	28	No		
Female	3	No	1		25	No		
Male	9	Yes	1		27	Yes	Yes	6th
Male	13	Yes	2	6	26	Yes	Yes	7th
Male	5	Yes	1		23	Yes	Yes	9th
Male	3	No	1		19	Yes	Yes	8th
Female	14	No	1		29	Yes	Yes	6th
Female	9	Yes	4	58	35	No		
Female	5	Yes	1		20	Yes	Yes	5th
Female	2	Yes	3	52	25	Yes	Yes	6th
Female	5	Yes	1		26	Yes	Yes	10th
Male	9	Yes	4	72	30	No		
Male	14	Yes	2	15	36	Yes	Yes	7th
Male	13	Yes	5	14	36	No		
Male	5	Yes	7	40	41	No		
Female	11	No	4	36	32	No		
Male	12	No	1		25	No		
Male	4	Yes	2	7	25	Yes	Yes	8th
Male	9	No	1		30	No		
Female	3	Yes	2	33	20	Yes	Yes	7th
Male	6	Yes	6	24	37	No		
Male	5	Yes	3	48	32	Yes	Yes	9th
Female	3	Yes	2	8	27	Yes	Yes	8th

For peer review only

Male	10	Yes	5	60	25	No		
Male	13	Yes	9	48	37	No		
Female	10	Yes	4	29	30	No		
Male	14	Yes	7	3	37	No		
Male	6	Yes	2	84	27	Yes	Yes	5th
Male	13	Yes	5	72	36	No		
Male	6	Yes	2	26	25	Yes	Yes	6th
Male	3	Yes	2	27	32	Yes	Yes	University/
Male	5	Yes	3	59	25	Yes	Yes	2nd
Female	4	Yes	6	24	40	No		
Male	14	Yes	3	120	25	No		
Female	2	No	1		22	Yes	Yes	8th
Male	12	No	1		21	Yes	Yes	5th
Male	12	No	7	4	43	No		
Male	14	Yes	2	10	28	No		
Male	13	Yes	7	84	43	No		
Female	9	Yes	2	48	22	Yes	Yes	5th
Female	6	No	3	3	22	Yes	Yes	9th
Female	4	Yes	3	46	30	Yes	Yes	10th
Male	12	Yes	7	48	40	Yes	Yes	3rd
Female	12	Yes	2	999	32	Yes	Yes	5th
Male	3	No	1		25	No		
Female	6	No	3	12	35	No		
Female	3	Yes	3	23	27	No		
Female	14	No	1		30	No		
Male	3	Yes	2	32	25	Yes	Yes	4th
Male	5	No	6	5	40	No		
Female	3	Yes	4	120	30	No		
Male	11	No	5	3	27	No		
Female	11	Yes	1		22	Yes	Yes	10th
Female	7	Yes	6	36	30	No		
Male	3	No	1		22	No		
Male	13	Yes	4	4	35	No		
Female	3	Yes	6	24	35	No		
Female	11	Yes	5	59	32	Yes	Yes	4th
Male	2	Yes	5	28	35	No		
Female	4	Yes	6	50	37	No		
Male	4	No	1		26	No		
Female	12	Yes	4	4	30	No		
Male	14	No	5	3	40	No		
Male	4	No	5	3	35	No		
Male	2	Yes	2	59	30	Yes	Yes	4th
Male	10	No	5	4	30	No		
Female	2	Yes	5	28	30	Yes	Yes	1st
Male	6	No	1		22	Yes	Yes	10th
Male	3	Yes	2	59	21	No		
Male	8	Yes	3	60	27	Yes	Yes	4th
Female	5	No	1		20	Yes	Yes	11th
Male	9	Yes	3	999	34	Yes	Yes	6th
Male	11	Yes	3	5	27	Yes	Yes	5th
Male	3	No	1		21	Yes	Yes	6th
Female	7	Yes	3	60	28	Yes	Yes	9th

For peer review only

Female	11	Yes	2	36	25	Yes	Yes	Technical/\
Female	13	No	5	5	42	No		
Female	5	No	1		33	Yes	Yes	5th
Male	11	Yes	3	3	33	No		
Male	11	No	1		22	Yes	Yes	6th
Female	12	Yes	4	999	37	No		
Female	3	Yes	5	36	36	No		
Female	13	Yes	2	999	28	Yes	Yes	6th
Female	11	Yes	1		25	No		
Female	7	No	1		18	Yes	Yes	6th
Female	14	Yes	6	36	30	No		
Male	2	Yes	6	39	36	No		
Female	6	Yes	1		19	Yes	Yes	10th
Female	4	No	3	4	30	Yes	Yes	10th
Male	10	Yes	5	36	42	No		
Male	11	No	5	3	35	No		
Male	10	No	3	5	27	No		
Male	6	Yes	6	48	41	No		
Male	11	No	1		25	No		
Female	7	No	7	5	33	No		
Female	12	Yes	8	48	37	No		
Male	11	Yes	2	999	27	Yes	Yes	6th
Female	5	Yes	4	48	30	No		
Male	4	Yes	7	30	35	No		
Female	6	No	1		25	No		
Male	11	Yes	5	38	28	No		
Male	13	Yes	1		25	Yes	Yes	5th
Male	13	Yes	4	36	30	No		
Male	13	No	4	5	35	No		
Female	10	No	5	6	30	No		
Female	6	Yes	6	48	30	No		
Male	9	Yes	5	36	30	No		
Male	10	No	1		21	Yes	Yes	5th
Female	9	Yes	3	32	28	Yes	Yes	1st
Female	9	Yes	4	42	30	No		
Female	3	Yes	9	7	42	No		
Male	10	No	2	13	28	Yes	Yes	6th
Male	7	No	1		20	Yes	Yes	8th
Male	7	Yes	8	120	35	No		
Female	3	Yes	4	36	25	Yes	Yes	7th
Male	11	Yes	5	48	30	Yes	Yes	4th
Male	12	Yes	9	53	39	Yes	No	
Male	7	Yes	7	36	40	No		
Male	14	No	7	9	42	Yes	Yes	6th
Female	5	No	5	2	35	No		
Male	14	No	2	6	23	Yes	Yes	8th
Male	9	Yes	2	36	27	Yes	Yes	7th
Male	11	Yes	7	48	30	No		
Female	4	No	1		28	Yes	Yes	3rd
Male	10	Yes	7	36	38	No		
Female	9	No	6	5	35	No		
Male	5	Yes	5	36	35	No		

For peer review only

Male	10	Yes	5	48	32	Yes	Yes	8th
Female	3	No	3	30	25	Yes	Yes	10th
Female	3	Yes	4	36	32	No		
Male	9	Yes	9	999	41	No		
Male	5	Yes	3	48	28	Yes	Yes	10th
Female	13	Yes	5	48	32	No		
Female	3	Yes	2	72	25	Yes	Yes	5th
Male	8	Yes	7	999	41	No		
Female	6	Yes	3	33	25	Yes	Yes	6th
Female	9	Yes	7	120	40	No		
Female	10	Yes	7	48	38	Yes	Yes	3rd
Male	2	No	1		26	Yes	Yes	5th
Female	5	Yes	3	120	30	No		
Male	11	Yes	6	60	33	Yes	Yes	6th
Female	6	Yes	7	35	37	No		
Male	10	No	1		25	Yes	Yes	5th
Female	11	Yes	4	42	32	No		
Male	10	Yes	2	60	26	Yes	Yes	9th
Female	12	Yes	12	36	40	Yes	Yes	6th
Female	9	Yes	2	56	22	No		
Male	8	Yes	4	999	35	No		
Male	3	Yes	6	36	35	Yes	Yes	4th
Female	3	Yes	7	24	45	No		
Male	4	Yes	4	43	30	No		
Male	10	Yes	5	40	38	No		
Female	9	Yes	2	9	30	No		
Female	8	Yes	3	48	30	No		
Female	14	Yes	5	120	30	No		
Female	13	No	1		22	Yes	Yes	10th
Male	5	Yes	5	3	27	No		
Female	3	Yes	7	35	28	No		
Female	4	Yes	2	36	25	Yes	Yes	8th
Male	8	No	5	4	38	Yes	Yes	6th
Female	11	No	3	3	45	No		
Female	2	Yes	7	46	35	No		
Male	3	No	1		21	Yes	Yes	9th
Female	6	No	2	5	30	No		
Male	14	Yes	5	59	37	No		
Female	12	Yes	5	10	33	No		
Male	11	No	7	5	40	No		
Female	4	No	1		25	Yes	Yes	8th
Female	14	Yes	6	7	40	No		
Male	3	Yes	5	37	36	No		
Male	11	Yes	5	47	32	No		
Female	4	No	6	3	32	No		
Female	7	No	4	4	35	No		
Male	10	Yes	3	999	35	No		
Female	10	Yes	6	59	37	No		
Male	6	No	1		20	Yes	Yes	4th
Female	8	Yes	6	39	38	No		
Male	14	Yes	3	53	32	No		
Female	7	Yes	3	49	30	No		

For peer review only

Male	9	Yes	5	36	37	No		
Male	13	Yes	5	51	36	No		
Male	3	Yes	5	5	38	No		
Male	6	Yes	7	38	35	No		
Male	7	Yes	2	59	30	No		
Male	9	No	2	3	23	Yes	Yes	10th
Male	14	Yes	6	36	35	Yes	Yes	1st
Male	8	Yes	6	72	35	No		
Male	5	Yes	3	52	25	No		
Male	8	Yes	5	48	32	No		
Female	13	Yes	7	39	37	No		
Male	8	Yes	2	36	23	Yes	Yes	8th
Male	5	Yes	4	8	35	No		
Female	8	Yes	2	49	28	No		
Male	14	No	1		18	Yes	Yes	8th
Male	6	Yes	4	59	35	No		
Female	12	No	1		22	No		
Male	9	Yes	4	59	37	No		
Female	11	No	6	7	35	No		
Male	9	Yes	4	48	29	No		
Male	14	No	5	7	30	No		
Male	14	No	4	4	32	No		
Female	8	Yes	4	59	33	No		
Female	13	Yes	2	48	22	Yes	Yes	6th
Female	6	Yes	2	72	27	Yes	Yes	6th
Female	13	Yes	5	48	30	No		
Female	6	No	1		26	Yes	Yes	10th
Male	14	Yes	4	72	30	Yes	Yes	3rd
Female	6	No	7	5	40	No		
Male	11	Yes	9	72	35	No		
Female	4	No	4	7	27	No		
Male	12	Yes	3	3	25	No		
Female	8	No	3	4	34	No		
Female	4	Yes	5	48	39	No		
Male	8	Yes	5	59	30	No		
Female	6	Yes	2	36	23	No		
Male	6	No	8	4	38	No		
Male	7	No	1		21	Yes	Yes	10th
Male	12	Yes	8	4	40	No		
Female	7	No	9	6	45	No		
Male	12	Yes	9	48	41	No		
Female	8	Yes	5	999	32	No		
Female	14	Yes	5	48	38	No		
Female	7	Yes	7	36	32	No		
Female	14	Yes	3	11	40	No		
Female	9	No	1		26	Yes	Yes	12th
Female	14	No	1		18	Yes	Yes	7th
Male	8	Yes	7	36	40	No		
Male	6	No	1		18	Yes	Yes	7th
Male	13	Yes	5	45	37	No		
Female	13	Yes	2	36	22	No		
Male	7	Yes	0	13	29	Yes	Yes	6th

For peer review only

Male	12	Yes	4	156	40	No		
Male	14	Yes	5	48	27	No		
Female	11	No	1		24	Yes	Yes	8th
Female	7	Yes	4	42	31	Yes	Yes	7th
Female	12	Yes	6	108	30	No		
Male	4	Yes	3	73	26	Yes	Yes	6th
Male	12	Yes	5	72	27	No		
Male	9	Yes	3	120	35	Yes	Yes	3rd
Female	3	Yes	2	56	23	Yes	Yes	5th
Male	5	No	1		18	Yes	Yes	5th
Male	5	Yes	3	72	26	No		
Female	4	No	1		18	Yes	Yes	4th
Female	5	Yes	5	24	30	No		
Male	14	Yes	1		20	Yes	Yes	10th
Female	10	Yes	4	48	35	No		
Female	9	Yes	4	144	38	No		
Female	9	Yes	6	36	35	No		
Female	14	Yes	2	96	25	Yes	Yes	8th
Female	14	Yes	3	60	28	No		
Male	4	Yes	2	184	24	No		
Female	13	Yes	2	98	27	Yes	Yes	7th
Male	8	Yes	1		19	No		
Female	13	Yes	5	36	30	Yes	No	
Male	8	Yes	2	57	23	Yes	Yes	6th
Male	14	Yes	8	72	35	No		
Male	9	No	1		22	Yes	Yes	University/
Female	4	Yes	4	132	32	No		
Female	4	Yes	3	7	28	No		
Male	14	Yes	3	42	32	No		
Female	14	Yes	2	76	22	Yes	Yes	3rd
Female	6	Yes	0	4	38	No		
Female	4	Yes	2	36	22	Yes	Yes	6th
Male	14	No	7	6	40	Yes	Yes	3rd
Male	13	Yes	4	78	35	No		
Male	9	Yes	4	36	30	No		
Male	14	Yes	5	108	28	No		
Male	14	No	1		23	Yes	Yes	10th
Male	6	Yes	3	36	25	No		
Female	10	Yes	7	48	36	No		
Female	3	Yes	4	84	35	No		
Male	13	Yes	3	59	30	No		
Male	7	No	2	70	25	Yes	Yes	5th
Male	7	Yes	2	36	26	No		
Female	9	Yes	3	32	23	Yes	Yes	7th
Female	11	No	1		20	No		
Male	11	No	1		22	Yes	Yes	9th
Female	11	Yes	2	49	20	Yes	Yes	8th
Female	9	Yes	2	59	21	Yes	Yes	9th
Male	14	No	1		30	No		
Male	13	No	6	4	35	Yes	Yes	4th
Male	14	Yes	2	4	25	Yes	Yes	7th
Male	12	Yes	2	72	20	Yes	Yes	4th

For peer review only

Male	8	No	3	60	35	No		
Female	4	No	1		25	No		
Female	2	Yes	2	34	21	Yes	Yes	4th
Female	13	Yes	4	48	33	No		
Male	13	Yes	2	36	26	Yes	Yes	5th
Female	6	Yes	2	81	26	Yes	Yes	10th
Male	6	No	1		18	No		
Male	9	Yes	4	180	40	No		
Female	8	Yes	5	72	26	No		
Female	11	Yes	2	84	30	No		
Female	7	No	1		27	Yes	Yes	12th
Female	5	Yes	4	52	41	No		
Male	8	Yes	3	84	27	Yes	Yes	5th
Male	12	Yes	5	72	35	No		
Female	9	No	1		20	Yes	Yes	8th
Female	3	No	3	48	30	No		
Male	9	Yes	3	84	27	No		
Male	3	Yes	4	28	28	No		
Male	14	Yes	4	36	33	Yes	Yes	7th
Male	9	Yes	3	72	27	No		
Female	3	Yes	4	72	39	Yes	Yes	5th
Male	14	Yes	2	60	30	No		
Male	5	Yes	2	48	28	No		
Female	3	Yes	2	27	22	No		
Male	8	Yes	3	58	31	Yes	Yes	8th
Female	3	Yes	3	38	25	Yes	Yes	4th
Male	7	Yes	4	96	30	Yes	Yes	5th
Male	9	No	1		25	Yes	Yes	8th
Male	2	Yes	2	6	23	Yes	Yes	6th
Female	4	Yes	3	36	30	No		
Female	10	Yes	2	36	20	No		
Female	3	Yes	2	59	24	Yes	Yes	10th
Male	3	Yes	3	36	28	Yes	Yes	10th
Female	14	No	1		21	No		
Female	7	Yes	4	72	30	No		
Male	14	Yes	1		23	Yes	Yes	9th
Female	5	Yes	3	133	30	No		
Female	8	Yes	8	74	38	No		
Female	4	No	0	0	26	Yes	Yes	7th
Male	14	No	1		17	No		
Male	6	Yes	3	72	22	Yes	Yes	5th
Male	14	No	1		25	No		
Female	3	Yes	2	84	26	No		
Male	8	Yes	4	36	38	Yes	Yes	6th
Female	9	Yes	6	60	35	No		
Female	14	Yes	2	70	25	Yes	Yes	8th
Female	7	No	1		18	Yes	Yes	6th
Female	5	Yes	2	60	25	Yes	Yes	8th
Male	8	Yes	0	7	32	No		
Female	5	Yes	4	59	33	No		
Female	8	No	1		26	No		
Female	11	Yes	3	72	27	No		

Female	11	No	1		20	Yes	Yes	5th
Male	13	Yes	0	7	25	Yes	Yes	5th
Male	12	Yes	2	72	20	Yes	Yes	4th
Female	14	Yes	6	48	33	No		
Female	9	Yes	5	72	28	Yes	Yes	3rd
Male	9	Yes	2	36	24	Yes	Yes	10th
Male	2	No	2	60	29	Yes	Yes	10th
Female	3	No	1		20	Yes	Yes	4th
Female	14	Yes	4	25	35	No		
Male	14	Yes	0	14	32	No		
Female	12	Yes	2	60	30	No		
Male	6	Yes	5	9	28	No		
Male	7	Yes	2	120	28	No		
Female	4	No	1		18	Yes	Yes	3rd
Male	5	Yes	5	72	30	No		
Male	14	No	1		29	No		
Male	5	Yes	2	144	28	No		
Male	2	Yes	1		18	Yes	No	
Female	11	No	1		24	Yes	Yes	8th
Male	7	Yes	1		20	Yes	Yes	7th
Male	9	Yes	4	156	32	No		
Male	7	No	1		28	Yes	Yes	5th
Male	8	Yes	3	26	23	Yes	Yes	6th
Male	14	Yes	1		20	Yes	Yes	5th
Female	8	Yes	0	6	24	Yes	Yes	5th
Female	4	No	1		19	Yes	Yes	11th
Female	9	No	1		18	Yes	Yes	11th
Female	8	Yes	3	48	29	Yes	Yes	4th
Male	14	Yes	4	120	35	No		
Female	5	Yes	6	72	41	No		
Male	9	No	1		20	Yes	Yes	4th
Female	12	No	1		20	Yes	Yes	4th
Female	14	Yes	3	84	35	No		
Female	7	Yes	6	120	35	No		
Male	7	Yes	3	84	34	No		
Male	12	No	1		19	Yes	Yes	5th
Female	8	Yes	4	3	28	Yes	Yes	6th
Male	6	Yes	3	72	26	Yes	Yes	6th
Male	4	Yes	4	52	25	Yes	Yes	5th
Male	3	Yes	4	96	27	No		
Female	5	Yes	5	2	28	No		
Female	11	Yes	5	36	36	No		
Female	3	Yes	3	5	25	Yes	Yes	8th
Female	14	No	1		19	Yes	Yes	10th
Female	13	Yes	10	48	41	No		
Female	6	Yes	2	60	25	Yes	Yes	5th
Male	10	Yes	2	38	30	Yes	Yes	5th
Female	10	Yes	3	96	32	No		
Male	10	Yes	3	59	24	Yes	Yes	6th
Female	9	Yes	3	60	25	Yes	Yes	4th
Male	14	No	1		25	Yes	Yes	5th
Female	7	No	1		26	Yes	Yes	3rd

Female	2	Yes	5	4	30	No		
Male	12	Yes	3	45	31	Yes	Yes	6th
Male	8	Yes	4	60	35	Yes	Yes	7th
Female	13	Yes	1		18	Yes	Yes	6th
Female	4	No	1		23	Yes	Yes	4th
Female	9	No	1		18	Yes	Yes	5th
Female	11	Yes	3	96	36	Yes	Yes	3rd
Female	4	Yes	3	37	40	No		
Male	10	Yes	2	72	23	No		
Male	2	Yes	2	11	31	No		
Male	3	No	1		28	No		
Male	7	No	1		20	Yes	Yes	6th
Male	14	Yes	4	144	38	No		
Male	3	No	1		19	Yes	Yes	7th
Male	14	Yes	4	60	31	No		
Female	8	Yes	8	3	40	No		
Female	11	Yes	2	41	22	Yes	Yes	10th
Female	5	Yes	5	36	35	No		
Male	6	Yes	1		20	No		
Female	12	Yes	4	59	30	No		
Male	5	Yes	0	4	22	No		
Male	14	Yes	2	60	25	Yes	Yes	7th
Male	8	Yes	3	72	32	No		
Male	4	No	1		20	Yes	Yes	3rd
Male	6	Yes	4	60	30	No		
Male	14	Yes	0	3	34	No		
Male	14	Yes	4	24	28	No		
Male	7	Yes	4	60	28	Yes	Yes	7th
Female	8	Yes	3	3	32	No		
Male	7	Yes	2	59	25	Yes	Yes	6th
Female	7	Yes	4	48	30	Yes	Yes	6th
Female	4	Yes	0	7	27	Yes	Yes	7th
Female	11	Yes	4	96	38	No		
Male	12	No	1		30	No		
Male	12	Yes	4	156	38	No		
Male	6	Yes	2	60	28	Yes	Yes	6th
Female	13	Yes	3	36	25	No		
Male	9	Yes	1		19	No		
Female	12	Yes	7	72	38	No		
Male	9	Yes	4	60	29	No		
Female	6	Yes	3	108	28	Yes	Yes	3rd
Female	9	No	1		21	No		
Male	5	Yes	3	60	28	Yes	Yes	4th
Male	3	Yes	2	84	28	No		
Female	2	Yes	5	38	30	No		
Female	6	Yes	2	72	24	Yes	Yes	6th
Male	14	Yes	7	60	35	No		
Male	5	No	1		27	No		
Female	5	Yes	2	84	24	Yes	Yes	8th
Male	12	Yes	2	59	30	Yes	Yes	10th
Male	8	Yes	5	52	34	No		
Male	9	No	1		28	No		

For peer review only

Male	9	No	8	4	42	No		
Male	9	Yes	7	108	30	No		
Female	3	Yes	2	60	28	Yes	Yes	4th
Female	14	Yes	5	72	32	No		
Male	6	Yes	6	36	38	No		
Female	7	Yes	6	35	38	No		
Female	14	Yes	5	27	32	No		
Female	14	Yes	3	48	24	Yes	Yes	8th
Female	14	No	1		21	Yes	Yes	4th
Female	3	Yes	3	132	28	Yes	Yes	3rd
Male	10	Yes	3	84	35	No		
Male	7	Yes	3	84	23	Yes	Yes	5th
Female	11	Yes	6	999	41	Yes	Yes	4th
Female	4	No	1		21	Yes	Yes	6th
Male	6	No	1		20	No		
Male	5	Yes	2	36	20	No		
Male	14	No	1		19	Yes	Yes	8th
Female	14	Yes	0	3	29	No		
Male	12	Yes	2	60	25	Yes	Yes	7th
Male	12	Yes	2	132	34	No		
Male	8	Yes	2	84	25	No		
Female	11	Yes	6	60	35	No		
Male	6	No	1		21	Yes	Yes	10th
Female	5	Yes	6	59	33	No		
Female	3	No	1		19	Yes	Yes	8th
Male	4	Yes	4	26	32	No		
Female	5	Yes	2	48	28	Yes	Yes	7th
Female	13	Yes	4	59	28	No		
Male	6	Yes	5	108	34	Yes	Yes	3rd
Male	3	Yes	2	60	26	Yes	Yes	8th
Male	12	No	7	4	40	No		
Female	10	Yes	6	48	42	No		
Male	11	Yes	5	60	38	No		
Female	13		2	72	23	Yes	Yes	8th
Female	2	Yes	2	48	28	Yes	Yes	8th
Male	7	Yes	3	72	24	No		
Female	8	Yes	2	108	25	Yes	Yes	6th
Female	14	Yes	7	48	40	Yes	Yes	2nd
Male	8	No	1		19	Yes	Yes	9th
Female	12	Yes	6	48	38	No		
Female	9	Yes	3	108	32	No		
Female	14	Yes	2	72	25	No		
Male	14	Yes	8	36	35	No		
Male	11	Yes	0	4	32	Yes	Yes	3rd
Male	9	Yes	2	70	22	No		
Male	3	Yes	0	6	36	No		
Male	14	No	1		20	Yes	Yes	8th
Female	11	Yes	6	48	30	No		
Male	4	Yes	2	72	25	Yes	Yes	6th
Male	13	Yes	2	120	28	Yes	Yes	2nd
Female	7	Yes	1		22	No		
Male	4	Yes	5	70	34	Yes	Yes	3rd

For peer review only

Female	9	No	1		28	Yes	Yes	9th
Male	12	No	4	96	32	Yes	No	
Male	10	No	1		24	No		
Female	12	Yes	2	72	30	Yes	Yes	5th
Female	14	Yes	3	84	30	No		
Male	8	No	1		20	Yes	Yes	3rd
Male	14	Yes	3	84	25	No		
Female	9	Yes	3	36	22	Yes	Yes	9th
Male	9	Yes	5	48	38	No		
Male	8	Yes	1		20	No		
Male	13	Yes	3	72	30	No		
Male	7	No	1		18	Yes	Yes	6th
Male	8	Yes	3	40	29	Yes	Yes	8th
Male	6	Yes	2	70	26	Yes	Yes	8th
Female	4	No	1		23	Yes	Yes	6th
Female	11	Yes	3	7	28	Yes	Yes	4th
Female	8	Yes	2	120	35	No		
Male	4	Yes	3	70	32	No		
Male	2	Yes	2	82	27	Yes	Yes	10th
Male	10	Yes	4	120	32	Yes	No	
Female	6	Yes	3	36	28	No		
Female	12	Yes	5	999	40	No		
Female	7	No	1		19	Yes	Yes	9th
Male	14	No	1		23	Yes	Yes	10th
Male	8	Yes	2	60	27	Yes	Yes	10th
Male	4	Yes	0	5	36	No		
Male	4	Yes	2	999	29	No		
Male	11	Yes	2	96	26	No		
Female	4	No	1		20	Yes	Yes	10th
Female	8	Yes	4	52	30	No		
Female	5	Yes	8	33	40	No		
Male	14	Yes	2	132	35	Yes	Yes	7th
Female	4	Yes	1		20	Yes	Yes	9th
Male	7	Yes	6	120	38	No		
Male	5	Yes	2	22	28	No		
Male	12	Yes	2	7	24	No		
Male	14	Yes	2	48	22	Yes	Yes	5th
Female	10	Yes	2	90	24	Yes	Yes	7th
Female	10	Yes	5	60	29	Yes	Yes	5th
Male	10	No	1		21	No		
Female	7	Yes	5	24	26	No		
Male	14	No	1		26	Yes	Yes	9th
Female	3	Yes	0	0	24	Yes	Yes	8th
Female	5	Yes	4	60	28	Yes	Yes	3rd
Male	10	No	1		20	Yes	Yes	5th
Male	11	Yes	2	6	30	No		
Male	6	No	1		19	Yes	Yes	10th
Female	5	Yes	3	52	28	Yes	Yes	6th
Male	13	No	3	18	38	No		
Male	6	Yes	2	72	23	Yes	Yes	5th
Male	9	Yes	2	4	22	Yes	Yes	6th
Female	10	Yes	0	12	35	No		

For peer review only

Male	14	No	1		22	Yes	Yes	8th
Male	3	Yes	1		18	Yes	Yes	2nd
Female	12	Yes	5	36	30	No		
Female	2	Yes	2	72	35	No		
Male	8	No	1		23	Yes	Yes	10th
Male	3	Yes	2	48	27	No		
Male	8	No	1		20	Yes	Yes	8th
Male	3	No	1		22	Yes	Yes	4th
Female	12	Yes	0	6	35	No		
Male	12	Yes	2	7	24	Yes	Yes	6th
Male	3	No	1		18	Yes	Yes	4th
Female	9	No	4	8	35	No		
Male	10	Yes	2	96	22	Yes	Yes	5th
Female	14	Yes	5	36	28	No		
Female	9	No	1		19	Yes	Yes	5th
Male	10	Yes	2	96	32	No		
Male	13	Yes	2	36	28	Yes	Yes	10th
Female	12	Yes	4	96	30	No		
Male	11	No	1		26	No		
Male	3	Yes	4	60	25	Yes	No	
Female	9	Yes	2	24	20	No		
Female	4	Yes	2	6	24	Yes	Yes	8th
Female	4	No	5	3	38	No		
Male	14	No	6	165	35	Yes	No	
Male	6	Yes	7	39	30	Yes	Yes	6th
Female	9	Yes	3	60	26	No		
Male	2	Yes	1		20	No		
Female	3	No	1		28	Yes	Yes	2nd
Female	12	No	1		30	Yes	Yes	8th
Female	2	Yes	4	76	39	No		
Female	11	Yes	1		19	Yes	Yes	5th
Female	12	Yes	0	5	28	Yes	Yes	6th
Male	12	Yes	2	60	22	Yes	Yes	9th
Female	3	Yes	0	5	26	No		
Male	4	Yes	2	60	22	Yes	Yes	8th
Female	6	Yes	3	120	28	Yes	Yes	3rd
Female	8	Yes	0	12	30	Yes	Yes	4th
Female	7	Yes	3	130	32	No		
Male	2	Yes	3	60	26	Yes	Yes	9th
Female	13	Yes	2	60	25	No		
Female	4	Yes	1		20	Yes	Yes	7th
Female	12	Yes	4	72	30	No		
Male	3	Yes	5	36	28	No		
Male	12	Yes	0	9	35	No		
Female	9	Yes	4	84	36	No		
Male	12	No	1		35	No		
Female	8	Yes	7	37	30	No		
Male	5	Yes	0	9	26	No		
Male	6	Yes	5	60	35	No		
Male	6	Yes	5	60	30	Yes	Yes	8th
Female	3	Yes	3	6	28	Yes	Yes	5th

For peer review only

q204	q205	q206	q207	q208	q210	q211	q213	q214
Orthodox	Married	No			8	7	7	Yes
Orthodox	Married	No			5	4	4	Yes
Orthodox	Married	No			6	6	6	No
Orthodox	Married	No			4	4	4	No
Orthodox	Married	No			7	7	7	No
Orthodox	Married	Yes	Yes	3rd	1	1	1	No
Orthodox	Married	Yes	Yes	3rd	7	6	6	Yes
Orthodox	Married	Yes	Yes	11th	1	1	1	No
Orthodox	Married	Yes	Yes	1st	5	5	5	No
Orthodox	Married	No			1	1	1	No
Orthodox	Married	No			7	7	7	No
Orthodox	Married	Yes	Yes	6th	5	5	5	No
Orthodox	Married	Yes	Yes	8th	2	2	2	No
Orthodox	Married	Yes	Yes	12th	1	1	1	No
Orthodox	Married	No			4	2	2	Yes
Orthodox	Married	No			9	9	9	No
Orthodox	Married	Yes	Yes	4th	7	6	6	Yes
Orthodox	Married	No			8	8	8	No
Orthodox	Married	Yes	Yes	3rd	3	3	3	No
Orthodox	Married	Yes	Yes	8th	2	2	2	No
Orthodox	Married	No			4	4	4	No
Orthodox	Married	Yes	Yes	5th	3	3	3	No
Orthodox	Married	Yes	Yes	8th	6	6	6	No
Orthodox	Married	No			6	4	4	Yes
Orthodox	Married	Yes	No	1st	6	6	6	No
Orthodox	Married	No			4	4	4	No
Orthodox	Married	Yes	Yes	10th	4	4	4	No
Orthodox	Married	Yes	Yes	8th	3	3	3	No
Orthodox	Married	Yes	Yes	3rd	6	6	6	No
Orthodox	Married	No			7	7	7	No
Orthodox	Married	Yes	Yes	3rd	5	5	5	No
Orthodox	Married	Yes	Yes	5th	7	7	7	No
Orthodox	Married	Yes	Yes	3rd	7	7	7	No
Orthodox	Married	Yes	Yes	8th	2	2	2	No
Orthodox	Married	No			3	3	3	No
Orthodox	Married	Yes	Yes	8th	5	5	5	No
Orthodox	Married	Yes	No	1st	3	3	3	No
Orthodox	Married	Yes	Yes	9th	3	3	3	No
Orthodox	Married	No			4	4	4	No
Orthodox	Married	Yes	Yes	4th	5	5	5	No
Orthodox	Married	No			6	5	5	Yes
Orthodox	Married	No			4	4	4	No
Orthodox	Married	Yes	Yes	4th	6	6	6	No
Orthodox	Married	Yes	No	2nd	9	5	5	Yes
Orthodox	Married	No			2	2	2	No
Orthodox	Married	No			4	4	4	No
Orthodox	Married	No			1	1	1	No
Orthodox	Married	No			3	3	3	No
Orthodox	Married	Yes	Yes	8th	3	3	3	No
Orthodox	Married	Yes	Yes	4th	2	2	2	No
Orthodox	Married	Yes	Yes	9th	3	3	3	No

For peer review only

Orthodox	Married	Yes	Yes	8th	5	4	4	Yes
Orthodox	Married	Yes	Yes	3rd	5	5	5	No
Orthodox	Married	Yes	Yes	8th	2	2	2	No
Orthodox	Married	No			5	5	5	No
Orthodox	Married	No			4	4	4	No
Orthodox	Married	No			2	2	2	No
Orthodox	Married	No			10	9	10	Yes
Orthodox	Married	No			5	5	5	No
Orthodox	Divorced				4	4	4	No
Orthodox	Married	No			5	5	6	No
Orthodox	Married	Yes	Yes	5th	6	6	6	No
Orthodox	Married	No			5	4	4	Yes
Orthodox	Married	No			9	9	9	No
Orthodox	Married	Yes	Yes	6th	8	7	7	Yes
Orthodox	Married	Yes	Yes	University/	2	1	1	Yes
Orthodox	Married	Yes	Yes	3rd	5	5	5	No
Orthodox	Married	No			2	2	2	No
Orthodox	Married	Yes	Yes	2nd	5	5	5	No
Orthodox	Married	No			2	2	2	No
Orthodox	Married	Yes	Yes	3rd	1	1	1	No
Orthodox	Married	Yes	Yes	10th	3	3	3	No
Orthodox	Married	No			4	4	4	No
Orthodox	Married	Yes	Yes	9th	1	1	1	No
Orthodox	Married	Yes	Yes	10th	2	3	3	No
Orthodox	Married	No			6	6	6	No
Orthodox	Married	No			8	8	8	No
Orthodox	Married	No			6	6	6	No
Orthodox	Married	No			5	4	4	Yes
Orthodox	Married	No			4	4	4	No
Orthodox	Married	No			6	5	5	Yes
Orthodox	Married	No			4	4	4	No
Orthodox	Married	Yes	Yes	4th	5	5	5	No
Orthodox	Divorced				1	1	1	No
Orthodox	Married	No			4	4	4	No
Orthodox	Married	No			9	9	9	No
Orthodox	Married	Yes	Yes	6th	8	8	8	No
Orthodox	Married	No			4	4	4	No
Orthodox	Married	Yes	Yes	8th	3	3	3	No
Orthodox	Married	Yes	Yes	9th	7	7	7	No
Orthodox	Married	No			3	3	3	No
Orthodox	Married	Yes	Yes	4th	3	3	3	No
Orthodox	Married	Yes	Yes	11th	2	2	2	No
Orthodox	Married	No			5	5	5	No
Orthodox	Married	No			4	3	3	Yes
Orthodox	Married	No			5	0	5	No
Orthodox	Married	No			4	4	4	No
Orthodox	Married	No			3	3	3	No
Orthodox	Married	Yes	Yes	4th	3	3	3	No
Orthodox	Married	No			3	3	3	No
Orthodox	Married	No			4	4	4	No
Orthodox	Married	Yes	Yes	University/	1	1	1	No
Orthodox	Married	Yes	Yes	9th	3	3	3	No

For peer review only

Orthodox	Married	No			1	1	1	No
Orthodox	Married	Yes	Yes	4th	6	6	6	No
Orthodox	Married	Yes	Yes	7th	1	1	1	No
Orthodox	Married	Yes	Yes	7th	6	3	3	Yes
Orthodox	Married	No			2	2	2	No
Orthodox	Married	No			5	4	4	Yes
Orthodox	Married	No			4	4	4	No
Orthodox	Married	No			5	5	5	No
Orthodox	Married	Yes	Yes	6th	1	1	1	No
Orthodox	Married	Yes	Yes	6th	5	5	5	No
Orthodox	Married	Yes	Yes	4th	7	7	7	No
Orthodox	Divorced				1	2	2	No
Orthodox	Married	Yes	Yes	5th	4	5	5	No
Orthodox	Married	Yes	Yes	6th	7	7	7	No
Orthodox	Married	Yes	Yes	4th	8	8	8	No
Orthodox	Married	No			5	3	3	No
Orthodox	Married	Yes	No	1st	6	6	6	No
Orthodox	Married	No			6	6	6	No
Orthodox	Married	No			3	3	3	No
Orthodox	Married	Yes	Yes	2nd	4	4	4	No
Orthodox	Married	Yes	Yes	2nd	10	10	10	No
Orthodox	Married	Yes	Yes	4th	5	5	5	No
Orthodox	Married	No			2	2	2	No
Orthodox	Married	Yes	Yes	4th	1	1	1	No
Orthodox	Married	Yes	Yes	4th	8	8	8	No
Orthodox	Married	No			2	2	2	No
Orthodox	Married	No			5	5	5	No
Orthodox	Married	No			1	1	1	No
Orthodox	Married	No			7	7	7	No
Orthodox	Married	Yes	Yes	10th	1	1	1	No
Orthodox	Married	Yes	Yes	3rd	6	5	6	No
Orthodox	Married	Yes	Yes	4th	1	1	1	No
Orthodox	Married	Yes	Yes	3rd	7	7	7	No
Orthodox	Married	Yes	Yes	2nd	1	1	1	No
Orthodox	Married	No			7	5	5	Yes
Orthodox	Married	No			4	4	4	No
Orthodox	Married	No			1	1	1	No
Orthodox	Married	No			1	1	1	No
Orthodox	Married	No			2	1	1	Yes
Orthodox	Married	No			6	4	4	Yes
Orthodox	Married	No			5	4	4	Yes
Orthodox	Married	Yes	Yes	3rd	3	3	3	No
Orthodox	Married	Yes	Yes	6th	8	7	7	Yes
Orthodox	Married	Yes	Yes	6th	5	5	5	No
Orthodox	Married	No			8	8	8	No
Orthodox	Married	No			1	1	1	No
Orthodox	Married	Yes	Yes	4th	1	1	1	No
Orthodox	Married	Yes	Yes	6th	5	5	5	No
Orthodox	Married	Yes	No	1st	4	4	4	No
Orthodox	Married	No			3	3	3	No
Orthodox	Married	No			6	6	6	No
Orthodox	Married	No			4	4	4	No

For peer review only

Orthodox	Married	Yes	Yes	4th	6	6	6	No
Orthodox	Married	Yes	Yes	4th	6	5	5	Yes
Orthodox	Married	Yes	Yes	6th	1	1	1	No
Orthodox	Married	No			2	2	2	No
Orthodox	Married	No			4	4	4	No
Orthodox	Married	Yes	Yes	8th	2	2	2	No
Orthodox	Married	Yes	Yes	4th	6	5	5	Yes
Orthodox	Married	No			7	7	7	No
Orthodox	Married	Yes	Yes	3rd	4	4	4	No
Orthodox	Married	No			6	5	5	Yes
Orthodox	Married	No			4	3	3	Yes
Orthodox	Married	No			3	3	3	No
Orthodox	Married	No			6	5	5	Yes
Orthodox	Married	Yes	Yes	3rd	4	4	4	No
Orthodox	Married	No			4	4	4	No
Orthodox	Married	No			4	4	4	No
Orthodox	Married	Yes	Yes	5th	6	6	6	No
Orthodox	Married	No			1	1	1	No
Orthodox	Married	No			8	8	8	No
Orthodox	Married	Yes	No	1st	6	6	6	No
Orthodox	Married	No			5	5	5	No
Orthodox	Married	No			5	5	5	No
Orthodox	Married	No			7	6	6	Yes
Orthodox	Married	No			5	5	5	No
Orthodox	Married	Yes	Yes	9th	2	1	1	Yes
Orthodox	Married	No			5	5	5	No
Orthodox	Married	No			4	4	4	No
Orthodox	Married	Yes	Yes	5th	3	3	3	No
Orthodox	Married	No			5	5	5	No
Orthodox	Married	No			4	4	4	No
Orthodox	Married	No			5	4	4	Yes
Orthodox	Married	No			4	3	3	Yes
Orthodox	Married	Yes	Yes	5th	2	3	2	No
Orthodox	Married	Yes	Yes	4th	6	6	6	No
Orthodox	Married	No			2	2	2	No
Orthodox	Married	Yes	Yes	6th	7	4	4	Yes
Orthodox	Married	Yes	Yes	4th	8	5	5	Yes
Orthodox	Married	Yes	Yes	3rd	4	3	3	Yes
Orthodox	Married	Yes	Yes	2nd	5	5	5	No
Orthodox	Married	No			5	5	5	No
Orthodox	Married	No			1	1	1	No
Orthodox	Married	Yes	Yes	3rd	6	6	6	No
Orthodox	Married	Yes	Yes	10th	1	1	1	No
Orthodox	Married	No			3	3	3	No
Orthodox	Married	No			3	3	3	No
Orthodox	Married	No			3	3	3	No
Orthodox	Married	No			6	6	6	No
Orthodox	Married	Yes	Yes	3rd	4	4	4	No
Orthodox	Married	Yes	Yes	9th	1	1	1	No
Orthodox	Married	No			4	4	4	No
Orthodox	Married	Yes	Yes	3rd	8	8	8	No
Orthodox	Married	No			5	5	5	No

For peer review only

Orthodox	Married	No			1	1	1	No
Orthodox	Married	No			4	4	4	No
Orthodox	Married	No			2	2	2	No
Orthodox	Married	Yes	Yes	8th	2	2	2	No
Orthodox	Married	Yes	Yes	10th	3	2	2	Yes
Orthodox	Married	No			1	1	1	No
Orthodox	Married	No			2	2	2	No
Orthodox	Married	Yes	Yes	5th	6	6	6	No
Orthodox	Married	Yes	Yes	3rd	7	7	7	No
Orthodox	Married	Yes	Yes	5th	4	4	4	No
Orthodox	Married	No			5	5	5	No
Orthodox	Married	No			3	3	3	No
Orthodox	Married	No			6	6	6	No
Orthodox	Married	No			7	7	7	No
Orthodox	Married	Yes	Yes	5th	6	6	6	No
Orthodox	Married	No			2	2	2	No
Orthodox	Married	No			5	5	5	No
Orthodox	Married	No			6	5	5	Yes
Orthodox	Married	No			3	3	3	No
Orthodox	Married	Yes	No	3rd	4	4	4	No
Orthodox	Married	No			4	3	3	Yes
Orthodox	Married	No			3	3	3	No
Orthodox	Married	No			10	10	10	No
Orthodox	Married	No			4	4	4	No
Orthodox	Married	Yes	Yes	7th	1	1	1	No
Orthodox	Married	No			4	4	4	No
Orthodox	Married	No			4	4	4	No
Orthodox	Married	No			5	4	4	Yes
Orthodox	Married	Yes	Yes	6th	3	3	3	No
Orthodox	Married	No			5	5	5	No
Orthodox	Married	No			5	5	5	No
Orthodox	Married	Yes	Yes	5th	2	2	2	No
Orthodox	Married	No			2	2	2	No
Orthodox	Married	Yes	Yes	7th	1	1	1	No
Orthodox	Married	Yes	No	2nd	4	4	4	No
Orthodox	Married	Yes	Yes	5th	1	1	1	No
Orthodox	Married	No			2	1	1	Yes
Orthodox	Married	No			5	5	5	No
Orthodox	Married	Yes	Yes	3rd	1	1	1	No
Orthodox	Married	Yes	Yes	7th	1	1	1	No
Orthodox	Married	No			7	7	7	No
Orthodox	Married	No			2	2	2	No
Orthodox	Married	No			3	3	3	No
Orthodox	Married	No			6	5	5	Yes
Orthodox	Married	No			7	7	7	No
Orthodox	Married	Yes	Yes	9th	1	1	1	No
Orthodox	Married	Yes	Yes	4th	5	5	5	No
Orthodox	Married	Yes	Yes	3rd	10	10	11	No
Orthodox	Married	No			7	7	7	No
Orthodox	Married	No			5	5	5	No
Orthodox	Married	No			11	7	7	Yes
Orthodox	Married	Yes	Yes	9th	4	4	4	No

For peer review only

Orthodox	Married	No			6	6	6	No
Orthodox	Married	Yes	No	4th	1	1	1	No
Orthodox	Married	No			6	6	6	No
Orthodox	Married	No			5	5	5	No
Orthodox	Married	No			9	7	7	Yes
Orthodox	Married	No			6	6	6	No
Orthodox	Married	No			4	3	3	Yes
Orthodox	Married	No			8	8	8	No
Orthodox	Married	No			5	5	5	No
Orthodox	Married	Yes	Yes	8th	2	2	2	No
Orthodox	Married	Yes	Yes	5th	6	6	6	No
Orthodox	Married	No			5	5	5	No
Orthodox	Married	Yes	Yes	8th	1	1	1	No
Orthodox	Married	No			4	4	4	No
Orthodox	Married	No			5	5	5	No
Orthodox	Married	No			1	1	1	No
Orthodox	Married	No			3	3	3	No
Orthodox	Married	No			2	2	2	No
Orthodox	Married	No			4	4	4	No
Orthodox	Married	Yes	Yes	4th	2	2	2	No
Orthodox	Divorced				4	4	4	No
Orthodox	Married	No			1	1	1	No
Orthodox	Married	Yes	Yes	9th	2	2	2	No
Orthodox	Married	Yes	Yes	3rd	2	2	2	No
Orthodox	Married	Yes	Yes	3rd	5	5	5	No
Orthodox	Married	Yes	No	3rd	9	7	9	Yes
Orthodox	Married	No			1	1	1	No
Orthodox	Married	Yes	Yes	4th	1	1	1	No
Orthodox	Married	Yes	No	5th	4	4	4	No
Orthodox	Married	Yes	Yes	8th	1	1	1	No
Orthodox	Married	No			1	1	1	No
Orthodox	Married	Yes	Yes	5th	4	3	3	Yes
Orthodox	Married	Yes	Yes	7th	6	6	6	No
Orthodox	Married	No			1	1	1	No
Orthodox	Married	No			1	1	1	No
Orthodox	Married	Yes	Yes	4th	7	7	7	No
Orthodox	Married	Yes	Yes	4th	4	4	4	No
Orthodox	Married	No			1	1	1	No
Orthodox	Married	No			7	7	7	No
Orthodox	Married	No			1	1	1	No
Orthodox	Married	No			4	3	3	Yes
Orthodox	Married	No			2	2	2	No
Orthodox	Married	Yes	Yes	3rd	1	1	1	No
Orthodox	Married	No			5	5	5	No
Orthodox	Married	No			7	7	7	No
Orthodox	Married	No			7	7	7	No
Orthodox	Married	No			4	3	3	Yes
Orthodox	Married	No			6	6	6	No
Orthodox	Married	Yes	Yes	5th	11	10	10	Yes
Orthodox	Married	Yes	Yes	3rd	1	1	1	No
Orthodox	Married	No			3	4	4	No
Orthodox	Married	No			4	3	3	Yes

For peer review only

Orthodox	Married	No			3	3	3	No
Orthodox	Married	No			3	3	3	No
Orthodox	Married	No			5	5	5	No
Orthodox	Married	No			8	7	7	Yes
Orthodox	Married	No			4	4	4	No
Orthodox	Married	No			7	7	7	No
Orthodox	Married	No			6	5	5	Yes
Orthodox	Married	Yes	Yes	7th	6	3	34	Yes
Orthodox	Married	Yes	Yes	10th	2	2	2	No
Orthodox	Married	No			7	7	7	No
Orthodox	Married	Yes	Yes	9th	1	1	1	No
Orthodox	Married	Yes	Yes	4th	1	1	1	No
Orthodox	Married	Yes	Yes	5th	3	3	3	No
Orthodox	Married	Yes	Yes	4th	6	6	6	No
Orthodox	Married	No			5	5	5	No
Orthodox	Married	No			1	1	1	No
Orthodox	Married	Yes	Yes	5th	3	3	3	No
Orthodox	Married	No			2	2	2	No
Orthodox	Married	Yes	Yes	5th	6	5	6	Yes
Orthodox	Married	No			2	2	2	No
Orthodox	Married	No			6	6	6	No
Orthodox	Married	No			7	7	7	No
Orthodox	Married	No			1	1	1	No
Orthodox	Married	No			6	6	6	No
Orthodox	Married	Yes	Yes	6th	4	4	4	No
Orthodox	Married	Yes	Yes	6th	1	1	1	No
Orthodox	Married	No			6	6	6	No
Orthodox	Married	Yes	Yes	9th	3	3	3	No
Orthodox	Married	Yes	Yes	9th	4	3	3	Yes
Orthodox	Married	Yes	Yes	10th	3	3	3	No
Orthodox	Married	Yes	Yes	5th	2	2	2	No
Orthodox	Married	No			8	8	5	No
Orthodox	Separated				2	2	2	No
Orthodox	Married	Yes	Yes	3rd	3	3	3	No
Orthodox	Married	No			5	5	5	No
Orthodox	Married	No			2	2	2	No
Orthodox	Married	No			5	5	4	No
Orthodox	Married	Yes	Yes	4th	7	7	7	No
Orthodox	Married	Yes	No	1st	6	6	6	No
Orthodox	Married	No			4	4	3	No
Orthodox	Never married				2	2	2	No
Orthodox	Married	Yes	Yes	5th	2	2	2	No
Orthodox	Married	No			3	3	3	No
Orthodox	Married	Yes	Yes	3rd	2	2	2	No
Orthodox	Married	No			2	2	2	No
Orthodox	Married	No			7	7	7	No
Orthodox	Married	Yes	Yes	5th	4	4	4	No
Orthodox	Married	No			2	2	2	No
Orthodox	Married	Yes	No	4th	5	6	6	Yes
Orthodox	Married	No			3	3	3	No
Orthodox	Married	Yes	No	1st	7	6	1	Yes
Orthodox	Married	No			4	4	4	Yes

For peer review only

Orthodox	Married	No			3	3	3	No
Orthodox	Married	No			7	4	4	Yes
Orthodox	Married	Yes	Yes	8th	1	1	1	No
Orthodox	Married	No			6	6	6	Yes
Orthodox	Married	Yes	No	1st	6	5	5	Yes
Orthodox	Married	No			5	5	5	No
Orthodox	Married	No			2	2	2	No
Orthodox	Married	Yes	Yes	4th	1	1	1	No
Orthodox	Married	No			5	5	5	Yes
Orthodox	Married	Yes	Yes	6th	6	6	4	No
Orthodox	Married	Yes	Yes	4th	3	3	3	No
Orthodox	Married	No			2	2	2	No
Orthodox	Married	Yes	Yes	4th	7	7	5	No
Orthodox	Married	Yes	No	1st	5	5	5	No
Orthodox	Married	No			4	4	4	No
Orthodox	Married	Yes	No	3rd	5	5	5	Yes
Orthodox	Married	No			10	10	9	No
Orthodox	Married	No			6	5	4	Yes
Orthodox	Married	No			4	4	4	No
Orthodox	Married	No			6	6	6	No
Orthodox	Married	No			4	4	4	No
Orthodox	Married	No			3	3	3	No
Orthodox	Married	Yes	Yes	2nd	7	7	5	No
Orthodox	Married	No			7	7	5	No
Orthodox	Married	Yes	Yes	9th	10	10	10	No
Orthodox	Divorced				1	1	1	No
Orthodox	Married	Yes	No	1st	5	5	4	No
Orthodox	Married	Yes	Yes	9th	2	2	2	No
Orthodox	Married	Yes	Yes	8th	4	4	4	No
Orthodox	Married	Yes	Yes	9th	2	2	2	No
Orthodox	Married	No			6	6	6	No
Orthodox	Married	Yes	Yes	1st	5	5	4	No
Orthodox	Married	Yes	Yes	4th	5	5	5	No
Orthodox	Married	Yes	Yes	7th	10	10	7	No
Orthodox	Married	Yes	Yes	4th	1	1	1	No
Orthodox	Married	No			4	4	4	No
Orthodox	Married	Yes	Yes	4th	8	8	8	Yes
Orthodox	Married	Yes	Yes	6th	5	5	5	No
Orthodox	Married	No			8	7	7	Yes
Orthodox	Married	No			5	5	5	No
Orthodox	Married	Yes	Yes	10th	2	2	2	No
Orthodox	Married	Yes	Yes	5th	2	2	2	No
Orthodox	Married	No			4	4	4	No
Orthodox	Married	No			4	3	3	Yes
Orthodox	Married	No			6	6	6	No
Orthodox	Married	Yes	Yes	6th	1	1	1	No
Orthodox	Married	No			6	5	5	Yes
Orthodox	Married	No			5	3	3	Yes
Orthodox	Married	No			2	2	2	No
Orthodox	Married	Yes	Yes	6th	3	3	3	No
Orthodox	Married	No			3	3	3	No
Orthodox	Married	Yes	Yes	10th	1	1	1	No

For peer review only

Orthodox	Married	No			2	2	2	No
Orthodox	Married	Yes	No	3rd	4	3	2	Yes
Orthodox	Married	No			5	5	5	No
Orthodox	Married	Yes	Yes	2nd	1	1	1	No
Orthodox	Married	Yes	Yes	10th	3	3	3	No
Orthodox	Married	No			6	6	6	No
Orthodox	Married	No			3	3	3	No
Orthodox	Married	No			8	8	8	No
Orthodox	Married	No			5	5	5	No
Orthodox	Married	Yes	Yes	University/	2	2	2	No
Orthodox	Married	Yes	Yes	3rd	4	4	4	No
Orthodox	Married	No			2	2	2	No
Orthodox	Married	No			4	4	4	No
Orthodox	Married	No			3	3	3	No
Orthodox	Married	No			2	2	2	No
Orthodox	Married	No			9	8	8	Yes
Orthodox	Married	No			3	3	3	No
Orthodox	Married	No			3	3	3	No
Orthodox	Married	No			5	5	5	No
Orthodox	Married	No			5	5	5	No
Orthodox	Married	No			4	3	3	Yes
Orthodox	Married	No			1	1	1	No
Orthodox	Married	No			1	1	1	No
Orthodox	Married	Yes	Yes	10th	1	1	1	No
Orthodox	Married	Yes	Yes	9th	1	1	1	No
Orthodox	Married	No			4	4	4	No
Orthodox	Married	No			6	6	6	No
Orthodox	Married	Yes	Yes	University/	2	1	1	Yes
Orthodox	Divorced				1	1	1	No
Orthodox	Married	No			1	1	1	No
Orthodox	Married	No			3	3	3	No
Orthodox	Married	Yes	Yes	5th	1	1	1	No
Orthodox	Married	Yes	Yes	7th	2	2	2	No
Orthodox	Married	No			9	9	9	No
Orthodox	Married	No			2	2	2	No
Orthodox	Married	No			2	2	2	No
Orthodox	Married	No			7	7	7	No
Orthodox	Married	Yes	Yes	3rd	4	4	4	No
Orthodox	Married	No			2	2	2	No
Orthodox	Married	No			4	3	3	Yes
Orthodox	Married	No			3	3	3	No
Orthodox	Married	No			5	5	5	No
Orthodox	Married	Yes	Yes	3rd	1	1	1	No
Orthodox	Married	Yes	Yes	4th	6	6	6	No
Orthodox	Married	No			6	4	4	Yes
Orthodox	Married	No			1	1	1	No
Orthodox	Married	No			5	5	5	No
Orthodox	Married	No			5	5	5	No
Orthodox	Married	No			1	1	1	No
Orthodox	Married	No			5	3	3	Yes
Orthodox	Married	Yes	Yes	3rd	4	4	4	No
Orthodox	Married	No			5	5	5	No

For peer review only

Orthodox	Married	Yes	Yes	5th	1	1	1	No
Orthodox	Married	Yes	Yes	4th	11	11	11	No
Orthodox	Married	No			4	4	4	No
Orthodox	Married	Yes	Yes	5th	2	2	2	No
Orthodox	Married	Yes	Yes	5th	1	1	1	No
Orthodox	Married	Yes	Yes	3rd	3	3	3	No
Orthodox	Married	Yes	Yes	8th	1	1	1	No
Orthodox	Married	Yes	Yes	8th	6	4	4	Yes
Orthodox	Married	No			2	2	2	No
Orthodox	Married	Yes	Yes	University/	3	3	3	No
Orthodox	Married	Yes	Yes	10th	1	1	1	No
Orthodox	Married	No			1	1	1	No
Orthodox	Married	No			2	2	2	No
Orthodox	Married	Yes	Yes	10th	2	2	2	No
Orthodox	Married	No			5	5	5	No
Orthodox	Married	Yes	Yes	6th	1	1	1	No
Orthodox	Married	No			5	5	5	No
Orthodox	Divorced				5	5	5	No
Orthodox	Married	No			1	1	1	No
Protestant	Married	Yes	Yes	6th	5	5	5	No
Protestant	Married	Yes	Yes	4th	1	1	1	No
Traditional	Married	Yes	Yes	3rd	3	2	2	Yes
Orthodox	Never married				1	1	1	No
Orthodox	Married	Yes	Yes	8th	2	2	2	No
Protestant	Married	No			3	3	3	No
Orthodox	Married	No			3	3	3	No
Orthodox	Married	Yes	Yes	3rd	2	2	2	No
Protestant	Married	No			2	2	2	No
Protestant	Widowed				2	2	2	No
Orthodox	Married	No			4	4	4	No
Orthodox	Married	Yes	Yes	8th	3	3	3	No
Protestant	Married	Yes	Yes	2nd	2	2	2	No
Orthodox	Married	No			1	1	1	No
Orthodox	Married	Yes	Yes	6th	2	2	2	No
Protestant	Married	No			5	5	5	No
Orthodox	Married	Yes	Yes	4th	2	2	2	No
Orthodox	Married	No			4	4	4	No
Orthodox	Married	No			1	1	1	No
Orthodox	Married	Yes	Yes	7th	2	2	2	No
Orthodox	Married	Yes	Yes	4th	3	3	3	No
Orthodox	Married	No			7	7	7	No
Protestant	Married	No			2	2	2	No
Protestant	Married	Yes	Yes	10th	2	2	2	No
Protestant	Married	No			5	5	5	No
Protestant	Married	Yes	Yes	3rd	1	1	1	No
Orthodox	Married	Yes	Yes	3rd	5	5	5	No
Orthodox	Married	No			6	6	6	No
Orthodox	Married	Yes	Yes	3rd	4	4	4	No
Orthodox	Married	No			4	4	4	No
Protestant	Married	No			4	4	4	No
Protestant	Married	No			2	2	2	No
Orthodox	Married	Yes	Yes	6th	1	1	1	Yes

For peer review only

Protestant	Divorced				3	3	3	No
Orthodox	Married	No			4	4	4	No
Protestant	Married	No			1	1	1	No
Protestant	Married	No			1	1	1	No
Protestant	Married	Yes	Yes	7th	1	1	1	No
Protestant	Married	Yes	Yes	5th	1	1	1	No
Orthodox	Married	Yes	Yes	10th	2	2	2	No
Orthodox	Married	Yes	Yes	4th	1	1	1	No
Orthodox	Married	No			2	2	2	No
Orthodox	Divorced				2	2	2	No
Orthodox	Married	No			1	1	1	No
Protestant	Married	Yes	Yes	9th	3	3	3	No
Orthodox	Married	No			1	1	1	No
Orthodox	Married	Yes	Yes	University/	1	1	1	No
Protestant	Married	Yes	Yes	5th	2	2	2	No
Orthodox	Married	No			1	1	1	No
Orthodox	Married	Yes	Yes	4th	3	3	3	No
Protestant	Married	No			3	3	3	No
Orthodox	Married	Yes	Yes	2nd	3	3	2	Yes
Orthodox	Married	Yes	Yes	9th	5	5	5	No
Orthodox	Married	No			1	1	1	No
Orthodox	Married	Yes	Yes	6th	6	6	6	No
Orthodox	Married	Yes	Yes	10th	2	1	1	Yes
Orthodox	Married	Yes	No	3rd	5	5	5	No
Orthodox	Married	Yes	Yes	10th	1	1	1	No
Orthodox	Married	Yes	Yes	4th	1	1	1	No
Orthodox	Married	Yes	Yes	8th	1	2	2	No
Orthodox	Divorced				1	1	1	No
Orthodox	Married	Yes	Yes	7th	4	4	4	No
Orthodox	Married	No			1	1	1	No
Orthodox	Divorced				6	6	6	No
Orthodox	Married	Yes	Yes	1st	6	6	6	No
Orthodox	Married	No			4	4	4	No
Orthodox	Married	No			2	2	2	No
Orthodox	Married	No			6	6	6	No
Orthodox	Married	Yes	Yes	4th	1	1	1	No
Orthodox	Married	Yes	Yes	8th	5	5	5	No
Orthodox	Married	No			3	2	2	No
Orthodox	Married	No			7	7	7	No
Orthodox	Married	Yes	Yes	3rd	4	4	4	No
Orthodox	Married	Yes	Yes	7th	3	3	3	No
Orthodox	Married	No			6	6	6	No
Orthodox	Married	No			4	4	4	No
Orthodox	Married	No			9	6	9	No
Orthodox	Married	Yes	Yes	5th	1	1	1	No
Orthodox	Married	Yes	Yes	5th	5	5	5	No
Orthodox	Married	No			3	3	3	No
Orthodox	Married	Yes	Yes	10th	3	2	2	Yes
Orthodox	Married	Yes	Yes	2nd	2	2	2	No
Orthodox	Married	Yes	Yes	4th	6	6	6	No
Orthodox	Married	Yes	Yes	5th	2	2	2	No
Orthodox	Married	No			2	2	2	No

For peer review only

Orthodox	Married	Yes	Yes	5th	1	1	1	No
Orthodox	Widowed				4	4	4	No
Orthodox	Married	Yes	Yes	6th	2	2	2	No
Orthodox	Married	Yes	Yes	9th	1	1	1	No
Orthodox	Married	No			5	5	5	No
Orthodox	Married	Yes	Yes	3rd	8	5	5	Yes
Orthodox	Married	Yes	Yes	4th	6	6	6	No
Orthodox	Married	Yes	Yes	3rd	2	2	2	No
Orthodox	Married	Yes	Yes	10th	1	1	1	No
Orthodox	Married	Yes	Yes	9th	2	2	2	No
Orthodox	Married	No			4	4	4	No
Orthodox	Married	No			2	2	2	No
Orthodox	Married	Yes	Yes	10th	2	1	1	Yes
Orthodox	Married	No			5	5	5	No
Orthodox	Married	No			2	2	2	No
Orthodox	Married	Yes	Yes	4th	2	1	1	Yes
Orthodox	Divorced				5	5	5	No
Orthodox	Married	No			2	1	1	Yes
Orthodox	Married	Yes	No	3rd	6	6	6	No
Orthodox	Married	No			6	6	6	No
Orthodox	Married	Yes	Yes	8th	2	1	1	Yes
Orthodox	Married	Yes	Yes	3rd	4	3	3	Yes
Orthodox	Married	Yes	Yes	2nd	8	8	8	No
Orthodox	Married	Yes	Yes	7th	2	2	2	No
Orthodox	Married	Yes	Yes	3rd	5	5	5	No
Orthodox	Married	Yes	Yes	5th	4	3	3	Yes
Orthodox	Married	No			5	5	5	No
Orthodox	Married	Yes	No	2nd	6	6	6	No
Orthodox	Married	No			4	4	4	No
Orthodox	Married	Yes	No	5th	9	6	6	Yes
Orthodox	Married	Yes	Yes	9th	2	2	2	No
Orthodox	Married	Yes	Yes	4th	4	4	4	Yes
Orthodox	Divorced				2	2	2	No
Orthodox	Married	Yes	Yes	9th	1	1	1	No
Orthodox	Married	No			6	6	6	No
Orthodox	Married	No			2	2	2	No
Orthodox	Married	No			8	7	5	Yes
Orthodox	Married	Yes	Yes	University/	1	1	1	No
Orthodox	Married	Yes	Yes	University/	3	3	3	No
Orthodox	Divorced				2	1	1	Yes
Orthodox	Married	Yes	Yes	6th	2	2	2	No
Orthodox	Married	No			3	3	3	No
Orthodox	Married	Yes	Yes	3rd	2	2	2	No
Orthodox	Married	No			5	5	5	No
Orthodox	Never married				1	1	1	No
Orthodox	Married	Yes	Yes	10th	1	1	1	No
Orthodox	Married	Yes	Yes	7th	1	1	1	No
Orthodox	Married	No			4	4	4	No
Orthodox	Married	Yes	Yes	6th	8	7	7	Yes
Orthodox	Married	Yes	Yes	10th	2	2	2	No
Orthodox	Married	Yes	Yes	4th	9	7	7	Yes
Orthodox	Married	Yes	Yes	8th	5	5	5	No

For peer review only

Orthodox	Married	Yes	Yes	4th	2	2	2	No
Orthodox	Married	Yes	Yes	6th	1	1	1	No
Orthodox	Married	Yes	Yes	3rd	3	2	2	Yes
Orthodox	Married	No			3	3	3	No
Orthodox	Married	Yes	Yes	6th	6	6	6	No
Orthodox	Married	No			8	8	8	No
Orthodox	Married	No			6	6	6	No
Orthodox	Married	No			7	7	7	No
Orthodox	Married	Yes	Yes	3rd	3	3	3	No
Orthodox	Married	Yes	Yes	8th	4	4	4	No
Orthodox	Married	No			9	9	9	No
Orthodox	Married	Yes	Yes	6th	2	2	2	No
Orthodox	Married	Yes	Yes	3rd	2	2	2	No
Orthodox	Married	Yes	Yes	10th	2	2	2	No
Orthodox	Married	No			5	5	5	No
Orthodox	Married	Yes	Yes	5th	1	1	1	No
Orthodox	Married	No			6	6	6	Yes
Orthodox	Married	No			3	3	3	No
Orthodox	Married	Yes	Yes	4th	10	8	8	Yes
Orthodox	Married	Yes	Yes	9th	4	3	3	No
Orthodox	Married	Yes	Yes	3rd	1	1	1	No
Orthodox	Married	Yes	No	1st	6	6	6	No
Orthodox	Married	Yes	Yes	1st	2	2	2	No
Orthodox	Divorced				2	2	2	No
Orthodox	Married	Yes	Yes	3rd	6	5	5	Yes
Orthodox	Married	No			5	5	5	No
Orthodox	Married	Yes	Yes	7th	3	3	3	No
Orthodox	Divorced				5	5	5	No
Orthodox	Married	Yes	Yes	8th	3	3	3	No
Orthodox	Married	No			2	2	2	No
Orthodox	Married	No			4	3	3	Yes
Orthodox	Married	Yes	No	1st	3	3	3	No
Orthodox	Married	Yes	No	3rd	6	6	6	No
Orthodox	Married	No			6	6	6	No
Orthodox	Married	Yes	Yes	7th	2	2	2	No
Orthodox	Married	No			7	7	7	Yes
Orthodox	Married	Yes	Yes	5th	5	5	5	No
Orthodox	Married	No			3	3	3	No
Orthodox	Married	Yes	Yes	10th	1	1	1	No
Orthodox	Married	No			4	4	4	No
Orthodox	Married	Yes	Yes	6th	4	4	4	No
Orthodox	Married	No			5	5	5	No
Orthodox	Married	No			1	1	1	No
Orthodox	Married	No			4	4	4	No
Orthodox	Married	No			8	8	8	Yes
Orthodox	Married	No			5	5	5	No
Orthodox	Married	Yes	Yes	8th	1	1	1	No
Orthodox	Married	No			4	4	4	No
Orthodox	Married	Yes	Yes	5th	1	1	1	No
Orthodox	Married	Yes	Yes	10th	2	2	2	No
Orthodox	Married	Yes	Yes	5th	6	6	6	No
Orthodox	Married	No			1	1	1	No

For peer review only

Orthodox	Married	Yes	Yes	6th	1	1	1	No
Orthodox	Married	Yes	Yes	6th	5	4	4	Yes
Orthodox	Married	No			3	3	3	No
Orthodox	Married	No			1	1	1	No
Orthodox	Married	Yes	Yes	8th	8	5	5	Yes
Orthodox	Married	No			2	2	2	No
Orthodox	Married	Yes	Yes	10th	1	1	1	No
Orthodox	Married	Yes	No	1st	6	6	6	No
Orthodox	Married	No			6	6	5	Yes
Orthodox	Married	No			5	3	2	Yes
Orthodox	Married	No			2	2	2	No
Orthodox	Married	Yes	No	1st	2	2	1	No
Orthodox	Married	No			6	6	6	No
Orthodox	Married	No			4	4	4	No
Orthodox	Married	No			7	7	5	No
Orthodox	Married	No			1	1	1	No
Orthodox	Married	No			2	2	2	No
Orthodox	Married	No			3	3	3	No
Orthodox	Married	Yes	Yes	10th	1	1	1	No
Orthodox	Married	Yes	Yes	6th	10	10	10	Yes
Orthodox	Married	No			8	8	7	Yes
Orthodox	Married	Yes	Yes	8th	1	1	1	No
Orthodox	Married	No			4	4	4	No
Orthodox	Married	Yes	Yes	4th	5	5	5	No
Orthodox	Married	No			2	2	2	No
Orthodox	Married	No			3	3	3	No
Orthodox	Married	No			6	6	6	No
Orthodox	Married	Yes	Yes	University/	1	1	1	No
Orthodox	Married	Yes	Yes	4th	6	6	6	No
Orthodox	Married	Yes	Yes	12th	1	1	1	No
Orthodox	Married	Yes	Yes	2nd	3	3	3	No
Orthodox	Married	No			4	4	4	No
Orthodox	Married	Yes	Yes	6th	2	2	2	No
Orthodox	Married	No			5	5	5	No
Orthodox	Married	Yes	Yes	1st	4	4	4	No
Orthodox	Married	No			4	4	4	No
Orthodox	Married	No			2	2	2	No
Orthodox	Married	Yes	No	1st	6	6	6	No
Orthodox	Married	No			4	4	4	No
Orthodox	Married	No			6	6	6	No
Orthodox	Married	No			2	2	1	No
Orthodox	Married	No			2	2	2	No
Orthodox	Married	Yes	No	1st	4	4	4	No
Orthodox	Married	Yes	Yes	7th	3	2	2	No
Orthodox	Married	No			2	2	2	No
Orthodox	Married	Yes	Yes	1st	9	9	7	No
Orthodox	Married	No			5	5	5	No
Orthodox	Married	No			4	4	3	No
Orthodox	Married	No			5	5	5	No
Orthodox	Married	Yes	Yes	10th	2	2	2	No
Orthodox	Married	Yes	No	1st	6	6	6	No
Orthodox	Married	Yes	Yes	4th	2	2	2	No

For peer review only

Orthodox	Married	Yes	Yes	7th	7	7	7	No
Orthodox	Married	Yes	No	1st	10	10	10	Yes
Orthodox	Married	No			4	4	4	No
Orthodox	Married	Yes	Yes	10th	3	3	3	No
Orthodox	Married	Yes	No	1st	1	1	1	No
Orthodox	Married	No			6	6	6	No
Orthodox	Married	Yes	Yes	7th	4	4	4	No
Orthodox	Married	Yes	No	1st	5	5	4	Yes
Orthodox	Married	No			2	2	2	No
Orthodox	Married	Yes	No	1st	3	2	2	Yes
Orthodox	Married	Yes	Yes	3rd	1	1	1	No
Orthodox	Married	Yes	Yes	10th	3	3	3	No
Orthodox	Married	Yes	Yes	10th	3	3	3	No
Orthodox	Married	Yes	Yes	6th	8	7	7	Yes
Orthodox	Married	No			6	6	6	No
Orthodox	Married	Yes	No	1st	4	4	4	No
Orthodox	Married	Yes	No	1st	6	6	6	No
Orthodox	Married	No			3	3	3	No
Orthodox	Married	No			9	8	8	Yes
Orthodox	Married	Yes	Yes	5th	5	5	5	No
Orthodox	Married	Yes	Yes	7th	4	4	4	No
Orthodox	Married	Yes	No	1st	5	5	5	No
Orthodox	Married	No			6	6	5	No
Orthodox	Married	No			7	7	7	No
Orthodox	Married	Yes	Yes	10th	3	3	3	No
Orthodox	Married	No			4	3	3	Yes
Orthodox	Married	No			4	4	4	No
Orthodox	Married	No			6	6	6	No
Orthodox	Married	No			3	3	3	No
Orthodox	Married	Yes	Yes	5th	3	3	3	No
Orthodox	Married	No			5	5	5	No
Orthodox	Married	Yes	Yes	7th	5	5	5	No
Orthodox	Married	Yes	Yes	6th	4	4	4	No
Orthodox	Married	Yes	Yes	3rd	2	2	2	No
Orthodox	Married	No			5	5	5	No
Orthodox	Married	No			6	6	6	No
Orthodox	Married	Yes	Yes	6th	6	6	6	No
Orthodox	Married	No			3	3	3	No
Orthodox	Married	No			5	4	5	Yes
Orthodox	Divorced				3	2	2	Yes
Orthodox	Married	Yes	Yes	6th	11	11	11	Yes
Orthodox	Married	Yes	Yes	4th	2	2	2	No
Orthodox	Married	No			2	1	1	No
Orthodox	Married	Yes	Yes	12th	1	1	1	No
Orthodox	Married	Yes	Yes	5th	2	2	2	No
Orthodox	Married	Yes	Yes	3rd	1	1	1	No
Orthodox	Married	Yes	Yes	7th	1	1	1	No
Protestant	Married	Yes	Yes	6th	1	1	1	No
Traditional	Married	No			2	2	3	No
Protestant	Married	Yes	Yes	9th	3	3	3	No
Orthodox	Married	Yes	Yes	4th	1	1	1	No
Orthodox	Married	Yes	Yes	4th	5	5	5	No

For peer review only

Protestant	Married	Yes	Yes	6th	1	1	1	No
Orthodox	Married	Yes	Yes	7th	1	1	1	No
Protestant	Married	Yes	Yes	5th	1	1	1	No
Protestant	Married	Yes	Yes	8th	3	3	3	No
Protestant	Married	Yes	Yes	5th	2	2	1	No
Protestant	Married	Yes	Yes	5th	1	1	1	No
Orthodox	Married	Yes	Yes	4th	2	2	2	No
Orthodox	Married	Yes	Yes	8th	1	1	1	No
Orthodox	Married	No			3	3	3	Yes
Protestant	Married	Yes	Yes	3rd	1	1	1	No
Orthodox	Married	Yes	Yes	4th	2	2	2	No
Orthodox	Married	Yes	Yes	2nd	1	1	1	No
Protestant	Married	Yes	Yes	3rd	4	4	4	No
Orthodox	Married	Yes	Yes	9th	2	1	1	Yes
Orthodox	Married	Yes	Yes	University/	3	3	3	No
Orthodox	Married	No			2	2	2	No
Protestant	Married	Yes	Yes	2nd	6	6	6	No
Protestant	Separated				1	1	1	No
Orthodox	Married	Yes	Yes	7th	1	1	1	No
Orthodox	Married	Yes	Yes	6th	5	5	5	No
Orthodox	Married	Yes	Yes	6th	1	1	1	No
Protestant	Married	No			3	3	3	No
Orthodox	Divorced				6	6	6	No
Orthodox	Married	Yes	Yes	4th	5	5	5	No
Protestant	Married	Yes	Yes	10th	1	1	1	No
Orthodox	Married	No			2	2	2	No
Orthodox	Married	Yes	Yes	8th	1	1	1	No
Protestant	Married	No			6	6	6	No
Protestant	Married	Yes	Yes	10th	3	3	3	No
Orthodox	Married	No			5	5	5	No
Protestant	Married	Yes	Yes	3rd	1	1	1	No
Protestant	Married	Yes	Yes	3rd	4	4	4	No
Protestant	Married	Yes	Yes	7th	3	3	3	No
Orthodox	Married	Yes	Yes	University/	1	1	1	No
Protestant	Married	Yes	Yes	4th	3	3	3	No
Orthodox	Married	No			3	3	3	No
Orthodox	Married	Yes	Yes	7th	3	3	3	No
Orthodox	Married	Yes	Yes	5th	1	1	1	No
Orthodox	Married	Yes	Yes	8th	1	1	1	No
Protestant	Married	Yes	Yes	4th	4	4	4	No
Orthodox	Married	Yes	Yes	5th	1	1	1	No
Protestant	Married	Yes	Yes	7th	2	2	2	No
Orthodox	Married	Yes	Yes	6th	1	1	1	No
Protestant	Married	Yes	Yes	5th	7	7	7	No
Protestant	Married	Yes	Yes	10th	2	1	1	Yes
Protestant	Married	Yes	Yes	4th	1	1	1	No
Protestant	Married	Yes	Yes	7th	4	4	4	No
Orthodox	Divorced				3	3	3	No
Orthodox	Married	Yes	Yes	9th	1	1	1	No
Protestant	Married	Yes	Yes	3rd	4	4	4	No
Protestant	Married	Yes	Yes	5th	2	2	2	No
Protestant	Married	Yes	Yes	7th	1	1	1	No

For peer review only

Orthodox	Married	Yes	Yes	8th	2	2	2	No
Protestant	Married	Yes	Yes	4th	1	1	1	No
Protestant	Divorced				2	2	2	No
Orthodox	Married	Yes	Yes	7th	5	5	5	Yes
Protestant	Married	Yes	Yes	2nd	2	2	2	No
Muslim	Married	Yes	Yes	University/	3	3	3	No
Orthodox	Married	Yes	Yes	5th	3	3	3	No
Protestant	Married	Yes	Yes	5th	1	1	1	No
Protestant	Married	No			1	1	1	No
Protestant	Never married				1	1	1	No
Protestant	Married	Yes	Yes	3rd	3	3	3	No
Orthodox	Married	Yes	Yes	7th	5	5	4	No
Orthodox	Married	Yes	Yes	10th	2	2	2	No
Orthodox	Married	No			1	1	1	No
Protestant	Married	Yes	Yes	8th	1	1	1	No
Orthodox	Married	Yes	Yes	3rd	3	3	3	No
Orthodox	Married	Yes	Yes	6th	1	1	1	No
Protestant	Married	Yes	Yes	6th	1	1	1	No
Orthodox	Married	Yes	Yes	10th	2	2	2	No
Protestant	Married	Yes	Yes	6th	2	2	2	No
Protestant	Married	Yes	Yes	6th	5	5	5	No
Orthodox	Married	Yes	Yes	6th	1	1	1	No
Protestant	Married	Yes	Yes	8th	1	1	1	No
Protestant	Married	Yes	Yes	6th	1	1	1	No
Orthodox	Married	Yes	Yes	3rd	3	3	3	No
Orthodox	Married	Yes	Yes	5th	3	3	3	Yes
Orthodox	Married	Yes	Yes	8th	1	1	1	No
Orthodox	Married	Yes	Yes	4th	6	6	6	No
Orthodox	Married	No			7	7	7	No
Orthodox	Married	Yes	Yes	6th	3	3	3	No
Orthodox	Married	Yes	Yes	3rd	6	6	6	No
Orthodox	Married	Yes	Yes	7th	6	6	6	No
Orthodox	Married	Yes	Yes	6th	7	2	2	Yes
Orthodox	Married	No			2	2	2	No
Orthodox	Married	No			3	3	3	No
Orthodox	Married	Yes	Yes	4th	1	0	1	No
Orthodox	Married	No			2	2	2	No
Orthodox	Married	Yes	No	1st	1	1	1	No
Orthodox	Married	Yes	Yes	4th	3	3	3	No
Orthodox	Married	Yes	Yes	4th	2	2	2	No
Orthodox	Married	Yes	Yes	3rd	1	1	1	No
Orthodox	Married	No			8	8	8	No
Orthodox	Married	Yes	Yes	4th	1	1	1	No
Orthodox	Married	Yes	Yes	7th	4	4	4	No
Orthodox	Married	Yes	Yes	4th	2	2	2	No
Orthodox	Married	No			1	1	1	No
Orthodox	Married	Yes	Yes	5th	9	10	10	No
Orthodox	Divorced				1	1	1	No
Orthodox	Married	No			4	4	4	No
Orthodox	Married	Yes	Yes	8th	1	1	1	No
Orthodox	Married	No			6	6	6	No
Orthodox	Married	No			2	2	2	No

For peer review only

Orthodox	Married	No			6	6	6	Yes
Orthodox	Married	No			3	3	3	No
Orthodox	Married	Yes	No	1st	7	7	7	No
Orthodox	Married	No			2	2	2	No
Orthodox	Married	No			6	6	6	No
Orthodox	Married	Yes	No	1st	5	5	5	No
Orthodox	Married	No			5	5	5	No
Orthodox	Married	No			9	8	8	Yes
Orthodox	Married	Yes	Yes	7th	10	9	9	Yes
Orthodox	Married	No			6	6	6	No
Orthodox	Married	No			6	6	6	No
Orthodox	Married	Yes	Yes	10th	2	2	2	No
Orthodox	Married	Yes	Yes	4th	4	4	4	No
Orthodox	Married	Yes	No	1st	11	9	7	Yes
Orthodox	Married	No			4	4	4	Yes
Orthodox	Married	Yes	Yes	2nd	3	3	3	No
Orthodox	Married	Yes	Yes	12th	1	1	1	No
Orthodox	Married	No			5	5	5	No
Orthodox	Married	No			4	4	4	No
Orthodox	Married	No			6	6	6	No
Orthodox	Married	Yes	Yes	4th	5	5	5	Yes
Orthodox	Married	Yes	Yes	4th	3	1	1	Yes
Orthodox	Married	Yes	Yes	8th	6	6	6	No
Orthodox	Married	Yes	Yes	4th	2	2	2	No
Orthodox	Married	Yes	Yes	6th	1	1	1	No
Orthodox	Married	Yes	Yes	4th	2	2	2	No
Orthodox	Married	No			8	5	5	Yes
Orthodox	Married	Yes	Yes	6th	2	2	2	No
Orthodox	Married	No			8	8	8	No
Orthodox	Married	No			6	6	6	No
Orthodox	Married	Yes	Yes	1st	5	3	3	Yes
Orthodox	Married	Yes	No	2nd	1	1	1	No
Orthodox	Married	Yes	Yes	8th	1	1	1	No
Orthodox	Married	No			4	4	4	No
Orthodox	Married	Yes	Yes	1st	6	6	6	No
Orthodox	Married	No			7	5	5	Yes
Orthodox	Married	No			4	4	3	No
Orthodox	Married	Yes	Yes	7th	1	1	1	No
Orthodox	Married	Yes	Yes	7th	1	1	1	No
Orthodox	Married	No			3	3	3	No
Orthodox	Married	Yes	Yes	4th	2	2	2	No
Orthodox	Married	Yes	No	1st	9	9	9	No
Orthodox	Married	Yes	Yes	6th	1	1	1	No
Orthodox	Married	No			5	5	5	No
Orthodox	Married	No			10	10	10	No
Orthodox	Married	Yes	No	1st	9	9	9	No
Orthodox	Married	Yes	Yes	4th	2	2	2	No
Orthodox	Married	Yes	No	1st	5	5	5	No
Orthodox	Married	No			4	4	4	No
Orthodox	Married	Yes	Yes	8th	2	2	2	No
Orthodox	Married	No			6	6	6	No
Orthodox	Married	Yes	Yes	4th	2	2	2	No

For peer review only

Orthodox	Married	Yes	Yes	9th	3	3	3	No
Orthodox	Married	No			2	2	2	Yes
Orthodox	Married	No			4	4	3	No
Orthodox	Married	No			5	5	5	No
Orthodox	Married	No			3	3	3	No
Orthodox	Married	Yes	Yes	4th	5	4	4	Yes
Orthodox	Married	Yes	Yes	4th	1	1	1	No
Orthodox	Married	Yes	Yes	5th	3	3	3	No
Orthodox	Married	Yes	Yes	5th	1	1	1	No
Orthodox	Married	No			6	6	6	No
Orthodox	Married	Yes	No	1st	4	4	4	No
Orthodox	Married	No			3	3	3	No
Orthodox	Married	Yes	Yes	8th	1	1	1	No
Orthodox	Married	No			5	4	3	Yes
Orthodox	Married	Yes	Yes	6th	1	1	1	No
Orthodox	Married	No			1	1	1	No
Orthodox	Married	Yes	Yes	7th	5	5	5	Yes
Orthodox	Married	No			9	5	5	Yes
Orthodox	Married	Yes	Yes	4th	2	2	2	No
Orthodox	Married	Yes	Yes	10th	1	1	1	No
Orthodox	Married	Yes	No	1st	6	7	7	No
Orthodox	Married	No			4	4	4	No
Orthodox	Married	No			3	3	3	No
Orthodox	Married	No			6	5	5	Yes
Orthodox	Married	No			5	5	5	Yes
Orthodox	Married	Yes	Yes	4th	6	6	6	No
Orthodox	Married	Yes	Yes	7th	6	6	6	No
Orthodox	Married	No			9	6	6	Yes
Orthodox	Married	No			5	4	4	No
Orthodox	Married	Yes	Yes	4th	6	6	6	No
Orthodox	Married	Yes	Yes	8th	1	1	1	No
Orthodox	Married	Yes	Yes	6th	6	6	5	No
Orthodox	Married	Yes	Yes	4th	1	1	1	No
Orthodox	Married	No			5	4	4	Yes
Orthodox	Married	Yes	Yes	9th	5	5	5	No
Orthodox	Married	No			2	2	2	No
Orthodox	Married	Yes	Yes	4th	4	4	4	No
Orthodox	Married	No			3	3	3	No
Orthodox	Married	No			9	7	7	Yes
Orthodox	Married	No			5	5	5	No
Orthodox	Married	No			2	2	2	No
Orthodox	Married	No			8	8	8	No
Orthodox	Married	Yes	No	1st	2	2	2	No
Orthodox	Married	Yes	No	1st	7	7	7	No
Orthodox	Married	Yes	Yes	University/	1	1	1	No
Orthodox	Married	No			2	2	2	No
Orthodox	Married	No			4	4	4	No
Orthodox	Married	Yes	Yes	4th	2	2	2	No
Orthodox	Married	Yes	Yes	4th	7	7	7	No
Orthodox	Married	Yes	Yes	5th	1	1	1	No
Orthodox	Married	No			5	5	5	No
Orthodox	Married	No			8	8	8	No

For peer review only

Orthodox	Widowed				6	6	6	No
Orthodox	Married	Yes	Yes	4th	8	8	6	No
Orthodox	Married	Yes	Yes	5th	1	1	1	No
Orthodox	Married	No			4	4	4	No
Orthodox	Married	No			5	5	5	No
Orthodox	Married	No			6	6	6	Yes
Orthodox	Married	No			5	5	5	No
Orthodox	Married	Yes	Yes	10th	1	1	1	No
Orthodox	Married	Yes	No	1st	4	4	4	Yes
Orthodox	Married	No			3	2	2	Yes
Orthodox	Married	No			7	6	4	Yes
Orthodox	Married	No			5	4	4	Yes
Orthodox	Married	No			5	5	3	No
Orthodox	Married	No			2	2	2	No
Orthodox	Married	Yes	Yes	4th	3	3	3	No
Orthodox	Married	Yes	Yes	3rd	5	4	4	Yes
Orthodox	Married	No			3	3	3	No
Orthodox	Married	Yes	Yes	9th	7	7	7	No
Orthodox	Married	No			4	4	4	No
Orthodox	Married	No			2	2	2	Yes
Orthodox	Married	No			10	10	10	No
Orthodox	Married	No			4	4	4	No
Orthodox	Married	No			5	5	5	Yes
Orthodox	Married	No			6	6	6	No
Orthodox	Married	No			7	6	6	Yes
Orthodox	Married	No			2	2	2	No
Orthodox	Married	Yes	Yes	5th	2	2	2	No
Orthodox	Married	Yes	Yes	1st	6	6	6	No
Orthodox	Married	Yes	Yes	3rd	3	3	3	No
Orthodox	Married	No			2	2	2	No
Orthodox	Married	Yes	Yes	7th	1	1	1	No
Orthodox	Married	Yes	No	1st	2	1	1	Yes
Orthodox	Married	Yes	Yes	9th	2	2	2	No
Orthodox	Married	No			9	9	9	Yes
Orthodox	Married	No			4	4	4	No
Orthodox	Married	Yes	Yes	8th	1	1	1	No
Orthodox	Married	Yes	No	3rd	1	1	1	No
Orthodox	Married	Yes	Yes	6th	2	2	2	No
Orthodox	Married	Yes	Yes	7th	2	2	2	No
Orthodox	Married	Yes	No	1st	7	10	10	Yes
Orthodox	Married	No			8	8	8	No
Orthodox	Married	Yes	Yes	7th	1	1	1	No
Orthodox	Married	No			4	4	4	No
Orthodox	Married	Yes	Yes	6th	2	2	2	No
Orthodox	Married	No			4	4	4	No
Orthodox	Married	Yes	Yes	7th	9	7	6	Yes
Orthodox	Married	No			9	9	9	No
Orthodox	Married	Yes	Yes	6th	4	4	4	No
Orthodox	Married	Yes	Yes	10th	2	2	2	No
Orthodox	Married	Yes	Yes	6th	2	2	2	No
Orthodox	Married	No			8	6	6	Yes
Orthodox	Married	Yes	No	4th	7	7	7	No

For peer review only

Orthodox	Married	No			5	5	5	No
Orthodox	Married	No			4	4	4	Yes
Orthodox	Married	Yes	Yes	10th	1	1	1	No
Orthodox	Married	Yes	Yes	University/	1	1	1	No
Orthodox	Married	Yes	Yes	4th	3	3	3	Yes
Orthodox	Married	No			8	8	8	No
Orthodox	Married	No			4	3	3	Yes
Orthodox	Married	Yes	No	1st	7	7	7	Yes
Orthodox	Married	No			1	1	1	No
Orthodox	Married	No			4	4	4	No
Orthodox	Married	No			4	4	4	No
Orthodox	Married	No			1	1	1	No
Orthodox	Married	Yes	Yes	5th	2	2	2	No
Orthodox	Married	No			6	6	6	No
Orthodox	Married	Yes	Yes	6th	4	4	4	No
Orthodox	Married	No			1	1	1	No
Orthodox	Married	Yes	Yes	10th	2	2	2	No
Orthodox	Married	Yes	Yes	6th	5	5	6	No
Orthodox	Married	Yes	Yes	5th	2	2	2	No
Orthodox	Married	No			1	1	1	No
Orthodox	Married	Yes	Yes	9th	2	2	2	No
Orthodox	Married	Yes	Yes	1st	4	4	4	No
Orthodox	Married	No			4	4	4	Yes
Orthodox	Married	No			1	1	1	No
Orthodox	Married	Yes	Yes	University/	3	3	3	Yes
Orthodox	Married	No			1	1	1	No
Orthodox	Married	No			9	8	5	Yes
Orthodox	Married	No			3	2	2	Yes
Orthodox	Married	No			3	3	3	No
Orthodox	Married	No			5	5	5	No
Orthodox	Married	Yes	No	4th	5	6	6	Yes
Orthodox	Married	No			5	5	5	No
Orthodox	Married	Yes	Yes	8th	1	1	1	No
Orthodox	Married	No			5	5	5	No
Orthodox	Divorced				4	4	4	Yes
Orthodox	Married	Yes	Yes	8th	2	2	2	No
Orthodox	Married	No			8	8	8	No
Orthodox	Married	No			2	2	2	No
Orthodox	Married	No			6	6	6	No
Orthodox	Married	Yes	Yes	8th	1	1	1	No
Orthodox	Married	No			4	4	4	No
Orthodox	Married	Yes	Yes	4th	9	8	8	Yes
Orthodox	Married	No			3	3	3	No
Orthodox	Married	No			2	2	2	No
Orthodox	Married	Yes	Yes	6th	1	1	1	No
Orthodox	Married	No			6	6	6	No
Orthodox	Married	Yes	Yes	10th	1	1	1	No
Orthodox	Married	Yes	Yes	10th	5	2	2	Yes
Orthodox	Married	Yes	Yes	8th	2	2	2	No
Orthodox	Married	Yes	Yes	6th	6	6	6	No
Orthodox	Married	No			6	6	6	No
Orthodox	Married	No			5	5	5	No

For peer review only

Orthodox	Married	No			5	5	5	No
Orthodox	Married	Yes	Yes	5th	3	3	3	No
Orthodox	Married	No			2	2	2	No
Orthodox	Married	No			6	6	6	No
Orthodox	Married	Yes	Yes	University/	1	1	1	No
Orthodox	Married	No			7	7	7	Yes
Orthodox	Married	No			2	2	2	No
Orthodox	Married	No			3	3	3	Yes
Orthodox	Married	Yes	Yes	8th	4	4	4	No
Orthodox	Married	Yes	Yes	5th	5	5	5	No
Orthodox	Married	No			2	2	2	No
Orthodox	Married	No			2	2	2	No
Orthodox	Married	No			8	8	7	Yes
Orthodox	Married	No			5	6	6	Yes
Orthodox	Married	No			3	3	3	No
Orthodox	Married	Yes	Yes	6th	6	6	6	No
Orthodox	Married	Yes	Yes	6th	1	1	1	No
Orthodox	Married	Yes	Yes	8th	1	1	1	No
Orthodox	Married	Yes	Yes	9th	3	3	3	No
Orthodox	Married	Yes	No	1st	5	5	5	No
Orthodox	Married	Yes	Yes	8th	3	2	2	Yes
Orthodox	Married	No			2	2	2	No
Orthodox	Married	Yes	Yes	5th	4	4	4	No
Orthodox	Married	No			4	4	4	No
Orthodox	Married	Yes	Yes	5th	8	8	8	No
Orthodox	Married	Yes	Yes	5th	7	7	7	No
Orthodox	Married	No			6	6	6	No
Orthodox	Married	No			2	2	2	No
Orthodox	Married	Yes	Yes	10th	1	1	1	No
Orthodox	Married	Yes	Yes	8th	2	2	2	No
Orthodox	Married	Yes	Yes	7th	6	6	6	No
Orthodox	Married	Yes	Yes	8th	3	2	2	Yes
Orthodox	Married	Yes	Yes	8th	3	3	3	No
Orthodox	Married	No			2	2	2	No
Orthodox	Widowed				3	3	3	No
Orthodox	Married	Yes	Yes	4th	2	2	2	No
Orthodox	Married	No			2	2	2	No
Orthodox	Married	Yes	Yes	8th	1	1	1	No
Orthodox	Married	No			7	5	5	No
Orthodox	Married	Yes	No	1st	6	6	6	No
Orthodox	Married	No			6	6	6	No
Orthodox	Married	No			1	1	1	No
Orthodox	Married	Yes	Yes	8th	1	1	1	No
Orthodox	Married	Yes	Yes	6th	1	1	1	No
Orthodox	Married	Yes	Yes	6th	3	3	3	No
Orthodox	Married	Yes	Yes	8th	1	1	1	No
Orthodox	Married	Yes	Yes	4th	1	1	1	No
Orthodox	Married	No			1	1	1	No
Orthodox	Married	No			5	5	5	No
Orthodox	Married	No			5	5	5	No
Orthodox	Married	Yes	Yes	7th	3	3	3	Yes
Orthodox	Married	No			5	5	5	No

For peer review only

Orthodox	Married	No			2	2	2	No
Muslim	Married	No			5	5	5	No
Orthodox	Married	No			4	4	4	No
Orthodox	Married	No			5	5	5	No
Orthodox	Married	Yes	Yes	9th	2	2	2	No
Orthodox	Married	No			1	1	1	No
Orthodox	Married	Yes	Yes	6th	1	1	1	No
Orthodox	Married	No			3	3	3	No
Orthodox	Married	Yes	No	3rd	5	5	5	Yes
Orthodox	Married	No			9	8	8	Yes
Orthodox	Married	No			1	1	1	No
Orthodox	Married	Yes	Yes	3rd	3	3	3	No
Orthodox	Married	No			5	5	5	No
Orthodox	Married	Yes	Yes	3rd	7	7	7	No
Orthodox	Married	No			6	6	6	No
Orthodox	Married	No			4	4	4	No
Orthodox	Married	Yes	No	3rd	4	4	4	No
Orthodox	Married	No			4	4	4	No
Orthodox	Married	Yes	Yes	10th	1	1	1	No
Orthodox	Married	No			2	2	2	No
Orthodox	Married	No			5	5	5	No
Orthodox	Married	No			5	6	6	No
Orthodox	Married	No			2	2	2	No
Orthodox	Married	No			7	6	6	Yes
Orthodox	Married	No			5	5	5	No
Orthodox	Married	No			8	8	7	No
Orthodox	Married	No			5	5	5	No
Orthodox	Married	No			7	7	7	No
Orthodox	Married	No			7	7	7	No
Orthodox	Married	Yes	Yes	5th	5	5	5	No
Orthodox	Married	Yes	No	1st	6	6	6	No
Orthodox	Married	Yes	Yes	5th	1	1	1	No
Orthodox	Married	No			10	8	8	Yes
Orthodox	Married	Yes	No	3rd	3	3	3	No
Orthodox	Married	No			2	2	2	No
Orthodox	Married	No			7	7	7	No
Orthodox	Married	Yes	Yes	3rd	5	5	5	No
Orthodox	Married	No			6	6	6	No
Orthodox	Married	No			6	6	6	No
Orthodox	Married	Yes	Yes	5th	2	2	2	No
Orthodox	Married	No			5	5	5	No
Orthodox	Married	Yes	Yes	6th	3	3	3	No
Orthodox	Married	Yes	Yes	11th	1	1	1	No
Orthodox	Married	Yes	No	3rd	4	4	4	No
Orthodox	Married	Yes	Yes	6th	2	2	2	No
Orthodox	Married	No			5	5	5	No
Orthodox	Married	Yes	Yes	7th	6	6	6	No
Orthodox	Married	Yes	Yes	4th	2	2	2	No
Orthodox	Married	Yes	Yes	10th	3	3	3	No
Orthodox	Divorced				1	1	1	No
Orthodox	Married	No			1	1	1	No
Orthodox	Married	Yes	Yes	7th	5	5	5	No

For peer review only

Orthodox	Married	No			6	6	6	No
Orthodox	Divorced				2	2	2	No
Orthodox	Married	Yes	Yes	4th	8	5	5	Yes
Orthodox	Married	No			1	1	1	No
Orthodox	Married	Yes	No	3rd	8	8	8	No
Orthodox	Married	No			5	3	3	Yes
Orthodox	Married	Yes	Yes	5th	6	6	6	No
Orthodox	Married	No			10	10	10	No
Orthodox	Married	No			6	6	6	No
Orthodox	Married	No			8	8	8	No
Orthodox	Married	No			3	3	3	No
Orthodox	Divorced				2	2	2	No
Orthodox	Married	Yes	Yes	4th	10	8	8	Yes
Orthodox	Married	Yes	Yes	3rd	3	4	4	No
Orthodox	Married	Yes	Yes	5th	4	4	4	No
Orthodox	Married	No			7	7	7	No
Orthodox	Married	Yes	No	1st	5	5	5	No
Orthodox	Married	No			6	6	5	No
Orthodox	Married	No			2	2	2	No
Orthodox	Married	No			5	5	5	No
Orthodox	Married	No			2	1	1	Yes
Orthodox	Married	No			2	2	2	No
Orthodox	Married	Yes	Yes	8th	3	3	3	No
Orthodox	Married	Yes	Yes	4th	5	4	4	Yes
Orthodox	Married	No			5	5	5	No
Orthodox	Married	No			4	4	4	No
Orthodox	Separated				5	4	4	Yes
Orthodox	Married	No			6	4	4	No
Orthodox	Married	Yes	Yes	10th	1	1	1	No
Orthodox	Married	Yes	Yes	4th	3	3	3	No
Orthodox	Married	Yes	Yes	7th	3	3	3	No
Orthodox	Married	No			4	4	4	No
Orthodox	Married	No			9	6	6	Yes
Orthodox	Married	Yes	Yes	3rd	2	2	2	No
Orthodox	Married	No			5	5	5	No
Orthodox	Married	No			5	4	4	Yes
Orthodox	Married	No			6	6	6	No
Orthodox	Divorced				5	5	5	No
Orthodox	Married	Yes	Yes	5th	5	5	5	No
Orthodox	Married	No			4	4	4	Yes
Orthodox	Married	No			8	6	6	Yes
Orthodox	Married	No			1	1	1	No
Orthodox	Divorced				1	1	1	No
Orthodox	Married	No			6	6	6	No
Orthodox	Married	No			3	3	3	No
Orthodox	Married	Yes	Yes	4th	3	3	3	No
Orthodox	Married	Yes	Yes	4th	2	2	2	No
Orthodox	Married	No			6	6	6	No
Orthodox	Married	No			1	1	1	No
Orthodox	Married	Yes	Yes	9th	1	1	1	No
Orthodox	Married	No			7	7	7	No
Orthodox	Married	Yes	Yes	5th	1	1	1	No

For peer review only

Orthodox	Married	No			3	3	3	No
Orthodox	Married	No			2	2	2	No
Orthodox	Married	No			3	3	3	No
Orthodox	Married	No			1	1	1	No
Orthodox	Married	No			6	6	6	No
Orthodox	Married	No			3	3	3	No
Orthodox	Married	No			5	5	5	No
Orthodox	Married	No			8	8	8	No
Orthodox	Married	No			4	4	4	No
Orthodox	Married	Yes	Yes	9th	2	2	2	No
Orthodox	Married	No			1	1	1	No
Orthodox	Married	Yes	Yes	4th	1	1	1	No
Orthodox	Married	No			5	5	5	No
Orthodox	Married	Yes	Yes	4th	2	1	1	Yes
Orthodox	Married	Yes	Yes	5th	1	1	1	No
Orthodox	Married	Yes	Yes	9th	2	2	2	No
Orthodox	Married	Yes	Yes	7th	1	1	1	No
Orthodox	Married	Yes	Yes	3rd	4	4	4	No
Orthodox	Married	Yes	Yes	University/	2	2	2	No
Orthodox	Married	Yes	Yes	3rd	2	2	2	No
Orthodox	Never married				1	1	1	No
Orthodox	Married	Yes	Yes	11th	1	1	1	No
Orthodox	Married	No			3	3	3	No
Orthodox	Married	No			4	4	4	No
Orthodox	Married	No			5	5	5	No
Orthodox	Widowed				3	3	3	No
Orthodox	Married	No			3	3	3	No
Orthodox	Married	Yes	Yes	7th	3	3	3	No
Orthodox	Married	Yes	Yes	8th	3	3	3	No
Orthodox	Married	Yes	Yes	4th	4	3	4	Yes
Orthodox	Married	Yes	Yes	6th	2	2	2	No
Orthodox	Married	No			2	2	2	No
Orthodox	Married	No			3	3	3	No
Orthodox	Married	Yes	Yes	8th	1	1	1	No
Orthodox	Married	Yes	Yes	5th	1	1	1	No
Orthodox	Never married				1	1	1	No
Orthodox	Married	No			4	4	4	No
Orthodox	Married	Yes	Yes	4th	1	1	1	No
Orthodox	Married	No			5	5	6	No
Orthodox	Married	Yes	Yes	3rd	6	6	6	No
Orthodox	Never married				1	1	1	No
Orthodox	Married	Yes	Yes	11th	3	3	3	No
Orthodox	Married	No			3	3	3	No
Orthodox	Married	Yes	Yes	6th	4	4	4	No
Orthodox	Married	Yes	Yes	10th	1	1	1	No
Orthodox	Married	No			3	3	3	No
Orthodox	Never married				1	1	1	No
Orthodox	Married	Yes	Yes	5th	4	4	4	No
Orthodox	Married	Yes	Yes	6th	2	2	2	No
Orthodox	Married	No			1	1	1	No
Orthodox	Married	Yes	Yes	8th	2	2	2	No
Orthodox	Married	Yes	Yes	2nd	2	2	2	Yes

For peer review only

Orthodox	Married	Yes	Yes	10th	1	1	1	No
Orthodox	Married	Yes	Yes	8th	1	1	1	No
Orthodox	Married	Yes	Yes	5th	1	1	1	No
Orthodox	Divorced				2	2	2	No
Orthodox	Married	Yes	Yes	8th	1	0	1	No
Orthodox	Married	Yes	Yes	10th	1	1	1	No
Orthodox	Married	Yes	Yes	9th	3	2	2	Yes
Orthodox	Married	Yes	Yes	9th	2	2	2	No
Orthodox	Married	Yes	Yes	4th	2	2	2	No
Orthodox	Married	Yes	Yes	9th	2	2	2	No
Orthodox	Married	No			5	5	5	No
Orthodox	Married	No			2	2	2	No
Orthodox	Married	Yes	Yes	3rd	2	2	2	No
Orthodox	Married	Yes	Yes	8th	1	1	1	No
Orthodox	Married	Yes	Yes	6th	4	4	4	No
Orthodox	Married	Yes	Yes	4th	3	3	3	No
Orthodox	Married	No			2	2	2	No
Orthodox	Never married				1	1	1	No
Orthodox	Married	Yes	Yes	8th	1	1	1	No
Orthodox	Married	No			4	4	4	No
Orthodox	Married	Yes	Yes	9th	2	2	2	No
Orthodox	Married	Yes	Yes	3rd	5	4	4	Yes
Orthodox	Married	Yes	Yes	8th	1	1	1	No
Orthodox	Widowed				2	2	2	No
Orthodox	Married	No			3	3	3	No
Orthodox	Married	No			2	2	2	No
Orthodox	Married	Yes	Yes	5th	4	4	4	No
Orthodox	Married	No			1	1	1	No
Orthodox	Never married				1	1	1	No
Orthodox	Married	Yes	Yes	8th	2	2	2	No
Orthodox	Married	No			7	7	7	No
Orthodox	Married	Yes	Yes	2nd	1	1	1	No
Orthodox	Married	No			2	0	2	No
Orthodox	Married	Yes	Yes	9th	1	1	1	No
Orthodox	Married	Yes	Yes	10th	1	1	1	No
Orthodox	Married	Yes	Yes	2nd	3	3	3	No
Orthodox	Married	Yes	Yes	4th	4	4	4	No
Orthodox	Married	Yes	Yes	8th	2	1	1	Yes
Protestant	Married	Yes	Yes	10th	2	2	2	Yes
Orthodox	Married	No			3	3	3	No
Orthodox	Married	Yes	Yes	9th	1	1	1	No
Orthodox	Married	Yes	Yes	10th	1	0	1	No
Orthodox	Married	Yes	Yes	University/	4	3	3	Yes
Orthodox	Married	Yes	Yes	8th	2	2	2	No
Orthodox	Married	No			3	3	3	No
Orthodox	Married	No			1	1	1	No
Orthodox	Married	Yes	Yes	10th	1	1	1	No
Orthodox	Married	No			5	5	5	No
Orthodox	Married	Yes	Yes	9th	2	2	2	No
Orthodox	Married	Yes	Yes	8th	1	1	1	No
Orthodox	Married	Yes	Yes	4th	4	4	4	No
Orthodox	Never married				1	1	1	No

For peer review only

Orthodox	Married	Yes	Yes	8th	2	2	2	No
Orthodox	Married	Yes	Yes	12th	5	5	5	No
Orthodox	Married	Yes	Yes	7th	2	2	2	No
Orthodox	Married	Yes	Yes	10th	1	0	1	No
Orthodox	Married	Yes	Yes	10th	1	1	1	No
Orthodox	Divorced				1	1	1	No
Orthodox	Married	Yes	Yes	3rd	5	5	5	No
Protestant	Married	Yes	Yes	10th	3	3	3	No
Orthodox	Married	Yes	Yes	7th	1	1	1	No
Orthodox	Married	Yes	Yes	4th	1	1	1	No
Orthodox	Married	Yes	Yes	8th	2	2	2	No
Orthodox	Divorced				1	1	1	No
Orthodox	Married	Yes	Yes	4th	1	1	1	No
Orthodox	Married	Yes	Yes	10th	2	2	2	Yes
Orthodox	Married	Yes	Yes	4th	2	2	2	No
Orthodox	Married	Yes	Yes	10th	1	1	1	No
Orthodox	Married	Yes	Yes	6th	1	1	1	No
Orthodox	Separated				1	1	1	No
Orthodox	Married	Yes	Yes	2nd	2	2	2	No
Orthodox	Married	No			3	3	3	No
Orthodox	Married	No			5	5	5	No
Orthodox	Married	No			4	4	4	No
Orthodox	Married	No			4	4	4	No
Orthodox	Married	No			2	2	2	No
Orthodox	Married	No			2	2	2	No
Orthodox	Never married				1	1	1	No
Orthodox	Married	Yes	Yes	10th	2	2	2	No
Orthodox	Married	Yes	Yes	University/	2	2	2	No
Orthodox	Married	Yes	Yes	3rd	3	3	3	No
Orthodox	Married	No			2	2	2	No
Orthodox	Married	No			2	2	2	No
Orthodox	Married	Yes	Yes	7th	2	2	2	No
Orthodox	Married	Yes	Yes	6th	1	1	1	No
Orthodox	Married	No			1	1	1	No
Orthodox	Married	No			3	3	3	No
Orthodox	Married	Yes	Yes	8th	1	1	1	No
Orthodox	Married	Yes	Yes	3rd	6	6	6	No
Orthodox	Married	Yes	Yes	7th	2	2	2	No
Orthodox	Married	Yes	Yes	4th	3	3	3	No
Orthodox	Married	No			3	3	3	No
Orthodox	Married	Yes	Yes	7th	7	7	7	No
Orthodox	Married	No			1	1	1	No
Orthodox	Married	Yes	Yes	11th	1	0	1	No
Orthodox	Married	Yes	Yes	10th	2	2	2	No
Orthodox	Divorced				2	2	2	No
Orthodox	Married	Yes	Yes	10th	1	1	1	No
Orthodox	Married	No			2	2	2	No
Orthodox	Married	Yes	Yes	8th	2	2	2	No
Orthodox	Married	Yes	Yes	8th	1	1	1	No
Orthodox	Married	Yes	Yes	4th	3	3	3	No
Orthodox	Married	No			1	1	1	No
Orthodox	Married	No			1	1	1	No

For peer review only

Orthodox	Married	No			6	6	6	No
Orthodox	Divorced				1	1	1	No
Orthodox	Married	No			3	3	3	No
Orthodox	Married	No			5	5	5	No
Orthodox	Married	No			3	3	3	No
Orthodox	Married	Yes	Yes	9th	1	1	1	No
Orthodox	Married	Yes	Yes	5th	3	3	3	No
Orthodox	Married	No			2	0	2	No
Orthodox	Married	Yes	Yes	10th	1	1	1	No
Orthodox	Married	No			6	6	6	No
Orthodox	Married	Yes	Yes	10th	4	4	4	No
Orthodox	Married	No			5	5	5	No
Orthodox	Married	No			1	1	1	No
Orthodox	Married	Yes	Yes	4th	2	2	2	No
Orthodox	Married	Yes	Yes	10th	1	1	1	No
Orthodox	Married	Yes	Yes	7th	4	4	4	No
Orthodox	Married	No			2	2	2	No
Orthodox	Married	No			2	2	2	No
Orthodox	Married	Yes	Yes	6th	2	2	2	No
Catholic	Married	Yes	Yes	3rd	2	2	2	No
Protestant	Married	Yes	Yes	11th	1	1	1	No
Orthodox	Married	Yes	Yes	11th	1	1	1	No
Protestant	Married	Yes	Yes	10th	3	3	3	No
Orthodox	Married	Yes	Yes	6th	1	1	1	No
Orthodox	Married	Yes	Yes	4th	1	1	1	No
Orthodox	Married	Yes	Yes	4th	3	3	3	No
Orthodox	Married	Yes	Yes	1st	3	3	3	No
Protestant	Married	Yes	Yes	10th	2	2	2	No
Orthodox	Married	No			4	4	5	No
Orthodox	Married	No			1	1	1	No
Orthodox	Married	No			4	4	4	No
Orthodox	Married	Yes	Yes	5th	2	2	2	No
Orthodox	Married	Yes	Yes	6th	2	2	2	No
Orthodox	Married	Yes	Yes	4th	1	1	1	No
Orthodox	Married	Yes	Yes	3rd	1	1	1	No
Orthodox	Married	Yes	Yes	5th	4	4	4	No
Orthodox	Married	Yes	Yes	8th	1	1	1	No
Orthodox	Married	Yes	Yes	3rd	3	3	3	No
Orthodox	Married	No			10	8	8	Yes
Orthodox	Married	No			4	4	4	No
Orthodox	Married	No			3	3	3	No
Orthodox	Married	No			6	5	5	Yes
Orthodox	Married	No			1	1	1	No
Orthodox	Married	Yes	Yes	9th	2	2	2	No
Orthodox	Married	Yes	Yes	3rd	5	4	4	No
Orthodox	Married	Yes	Yes	5th	1	1	1	No
Orthodox	Married	No			1	1	1	No
Orthodox	Married	Yes	Yes	4th	3	2	2	Yes
Orthodox	Married	No			2	2	2	No
Orthodox	Married	Yes	Yes	5th	5	5	5	No
Orthodox	Divorced				7	5	5	Yes
Orthodox	Married	Yes	Yes	3rd	3	3	3	No

For peer review only

Orthodox	Married	Yes	Yes	8th	2	2	2	No
Orthodox	Married	No			3	3	3	No
Orthodox	Married	Yes	Yes	4th	2	2	2	No
Orthodox	Married	Yes	No	4th	1	1	1	No
Orthodox	Married	No			3	3	3	No
Orthodox	Married	No			7	7	7	No
Orthodox	Married	Yes	Yes	3rd	1	1	1	No
Orthodox	Married	No			4	4	4	No
Orthodox	Married	Yes	Yes	6th	4	4	4	No
Orthodox	Married	No			3	3	3	No
Orthodox	Married	Yes	Yes	8th	1	1	1	No
Orthodox	Married	Yes	Yes	1st	4	4	4	No
Orthodox	Married	No			3	3	3	No
Orthodox	Married	Yes	Yes	3rd	6	6	6	No
Orthodox	Married	No			5	5	5	No
Orthodox	Married	Yes	No	6th	3	3	3	No
Orthodox	Married	No			3	3	3	No
Orthodox	Married	No			8	8	8	No
Orthodox	Married	Yes	Yes	4th	2	2	2	No
Orthodox	Married	Yes	Yes	4th	3	3	3	No
Orthodox	Married	No			3	3	3	No
Orthodox	Married	Yes	Yes	6th	6	6	6	No
Orthodox	Married	Yes	Yes	4th	4	4	4	No
Orthodox	Married	Yes	Yes	5th	1	1	1	No
Orthodox	Married	No			4	4	4	No
Orthodox	Married	Yes	Yes	8th	2	2	2	No
Orthodox	Married	No			2	1	1	Yes
Orthodox	Married	Yes	Yes	6th	1	1	1	No
Orthodox	Married	Yes	Yes	4th	1	1	1	No
Orthodox	Married	Yes	Yes	4th	4	4	4	No
Orthodox	Married	No			2	2	2	No
Orthodox	Married	Yes	Yes	4th	4	4	4	No
Orthodox	Married	No			5	5	5	No
Orthodox	Married	No			9	6	6	Yes
Orthodox	Married	Yes	Yes	2nd	3	3	3	No
Orthodox	Married	Yes	Yes	4th	1	1	1	No
Orthodox	Married	No			5	5	5	No
Orthodox	Married	Yes	Yes	8th	4	4	4	No
Orthodox	Married	No			1	1	1	No
Orthodox	Married	No			4	4	4	No
Orthodox	Married	Yes	Yes	7th	3	3	3	No
Orthodox	Married	No			2	2	2	No
Orthodox	Married	No			4	4	4	No
Orthodox	Married	Yes	No	4th	2	2	2	No
Orthodox	Married	Yes	Yes	7th	2	2	2	No
Orthodox	Widowed				9	7	7	Yes
Orthodox	Married	Yes	Yes	2nd	2	2	2	No
Orthodox	Married	No			1	1	1	No
Orthodox	Married	No			6	4	4	Yes
Orthodox	Married	Yes	No	12th	7	7	7	No
Orthodox	Married	Yes	Yes	6th	1	1	1	No
Orthodox	Married	Yes	Yes	5th	1	1	1	No

For peer review only

Orthodox	Married	Yes	Yes	10th	1	1	1	No
Orthodox	Married	No			2	2	2	No
Orthodox	Married	Yes	Yes	10th	2	2	2	No
Orthodox	Married	Yes	Yes	6th	1	1	1	No
Orthodox	Married	Yes	No	1st	5	5	5	No
Orthodox	Married	No			2	2	2	No
Orthodox	Married	Yes	Yes	4th	3	3	3	No
Orthodox	Married	Yes	Yes	5th	4	4	4	No
Orthodox	Married	No			5	4	4	Yes
Orthodox	Married	Yes	Yes	10th	2	2	2	No
Orthodox	Married	No			1	1	1	No
Orthodox	Divorced				5	3	3	Yes
Orthodox	Married	Yes	Yes	4th	3	3	3	No
Orthodox	Married	Yes	Yes	6th	1	1	1	No
Orthodox	Married	No			2	2	2	No
Orthodox	Married	Yes	Yes	6th	2	2	2	No
Orthodox	Married	Yes	No	3rd	2	2	2	No
Orthodox	Married	Yes	Yes	3rd	4	4	4	No
Orthodox	Married	Yes	Yes	University/	6	6	6	Yes
Orthodox	Married	Yes	Yes	4th	1	1	1	No
Orthodox	Married	No			7	7	7	No
Orthodox	Married	No			3	3	3	No
Orthodox	Married	Yes	Yes	9th	3	3	3	No
Orthodox	Married	Yes	Yes	5th	1	1	1	No
Orthodox	Married	Yes	Yes	5th	5	5	5	No
Orthodox	Married	Yes	Yes	8th	4	4	4	No
Orthodox	Married	Yes	Yes	9th	1	1	1	No
Orthodox	Married	No			6	6	6	No
Orthodox	Married	No			3	3	3	No
Orthodox	Married	No			3	3	3	No
Orthodox	Married	Yes	Yes	9th	2	1	1	Yes
Orthodox	Married	Yes	Yes	10th	1	1	1	No
Orthodox	Married	Yes	Yes	10th	2	1	1	No
Orthodox	Married	No			5	5	5	No
Orthodox	Married	Yes	Yes	10th	4	3	3	Yes
Orthodox	Married	No			1	1	1	No
Orthodox	Married	Yes	Yes	3rd	3	1	1	Yes
Orthodox	Married	Yes	Yes	4th	2	2	2	No
Orthodox	Married	Yes	Yes	8th	2	2	2	No
Orthodox	Married	Yes	Yes	7th	1	1	1	No
Orthodox	Married	No			4	4	4	No
Orthodox	Married	Yes	No	2nd	4	4	4	No
Orthodox	Married	Yes	Yes	7th	4	4	4	Yes
Orthodox	Married	Yes	Yes	8th	4	4	4	No
Orthodox	Married	No			1	1	1	No
Orthodox	Married	Yes	Yes	4th	2	2	2	No
Orthodox	Married	Yes	Yes	10th	1	1	1	No
Orthodox	Married	Yes	Yes	University/	2	2	2	No
Orthodox	Married	Yes	Yes	9th	2	2	2	No
Orthodox	Married	Yes	Yes	10th	2	2	2	No
Orthodox	Married	Yes	Yes	12th	1	1	1	No
Orthodox	Married	Yes	Yes	4th	5	5	5	No

For peer review only

Orthodox	Married	Yes	Yes	4th	7	7	6	No
Orthodox	Married	Yes	Yes	10th	1	1	1	No
Orthodox	Married	No			2	2	2	No
Orthodox	Married	Yes	Yes	7th	3	3	3	No
Orthodox	Married	Yes	Yes	4th	2	2	2	No
Orthodox	Married	No			4	4	4	No
Orthodox	Married	Yes	Yes	1st	4	4	4	No
Orthodox	Married	Yes	Yes	10th	2	2	2	No
Orthodox	Married	Yes	No	1st	3	3	3	No
Orthodox	Married	Yes	Yes	7th	9	9	9	No
Orthodox	Married	Yes	Yes	4th	2	2	2	No
Orthodox	Married	Yes	Yes	10th	1	1	1	No
Orthodox	Married	No			3	3	3	No
Orthodox	Married	No			3	3	3	No
Orthodox	Married	Yes	Yes	2nd	7	7	7	No
Orthodox	Married	Yes	Yes	5th	6	6	6	No
Orthodox	Married	Yes	Yes	2nd	4	4	4	No
Orthodox	Married	Yes	Yes	5th	1	1	1	No
Orthodox	Married	Yes	Yes	10th	1	1	1	No
Orthodox	Married	Yes	No	3rd	3	3	3	No
Orthodox	Married	No			4	4	4	No
Orthodox	Married	Yes	Yes	4th	3	3	3	No
Orthodox	Married	Yes	Yes	10th	1	1	1	No
Orthodox	Married	Yes	Yes	8th	2	2	2	No
Orthodox	Married	Yes	Yes	8th	4	4	4	No
Orthodox	Married	No			5	5	5	No
Orthodox	Married	Yes	No	1st	3	3	3	No
Orthodox	Married	Yes	Yes	10th	2	2	2	No
Orthodox	Married	Yes	Yes	3rd	2	2	2	No
Orthodox	Divorced				1	1	1	No
Orthodox	Married	No			1	1	1	No
Orthodox	Married	No			2	2	2	No
Orthodox	Married	Yes	Yes	University/	2	2	2	No
Orthodox	Married	Yes	Yes	9th	5	4	4	Yes
Orthodox	Married	Yes	Yes	10th	3	3	3	No
Orthodox	Married	Yes	Yes	6th	1	1	1	No
Orthodox	Married	Yes	Yes	10th	1	1	1	No
Orthodox	Married	No			1	1	1	No
Orthodox	Married	Yes	Yes	4th	1	1	1	No
Orthodox	Married	Yes	Yes	6th	1	1	1	No
Orthodox	Married	No			5	4	4	Yes
Orthodox	Married	Yes	Yes	2nd	8	8	8	No
Orthodox	Married	Yes	No	4th	2	2	2	No
Orthodox	Married	Yes	Yes	9th	2	2	2	No
Orthodox	Married	Yes	Yes	10th	2	2	2	No
Orthodox	Married	Yes	Yes	5th	6	5	5	Yes
Orthodox	Married	Yes	Yes	8th	2	1	1	Yes
Orthodox	Married	Yes	Yes	10th	2	2	2	No
Orthodox	Married	No			2	1	1	Yes
Orthodox	Married	No			4	3	3	Yes
Orthodox	Married	Yes	Yes	9th	2	2	2	No
Orthodox	Married	Yes	Yes	4th	1	1	1	No

For peer review only

Orthodox	Married	Yes	Yes	University/	6	6	6 No
Orthodox	Married	Yes	Yes	10th	2	2	2 No
Orthodox	Married	Yes	Yes	7th	2	2	2 No
Orthodox	Married	No			8	8	7 No
Orthodox	Married	No			4	4	2 No
Orthodox	Married	Yes	Yes	3rd	6	6	6 No
Orthodox	Married	Yes	Yes	5th	1	1	1 No
Orthodox	Married	Yes	Yes	4th	1	1	1 No
Orthodox	Married	Yes	Yes	3rd	2	2	2 No
Orthodox	Married	No			3	3	3 No
Orthodox	Married	No			4	4	4 No
Orthodox	Married	Yes	Yes	7th	2	2	2 No
Orthodox	Married	No			5	5	5 No
Orthodox	Married	Yes	Yes	8th	1	1	1 No
Orthodox	Married	Yes	Yes	7th	1	1	1 No
Orthodox	Married	Yes	Yes	2nd	1	1	1 No
Orthodox	Married	Yes	Yes	4th	2	2	2 No
Orthodox	Married	Yes	Yes	10th	2	2	2 No
Orthodox	Married	Yes	Yes	10th	2	2	2 No
Orthodox	Married	Yes	Yes	8th	5	5	5 No
Orthodox	Married	No			1	1	1 No
Orthodox	Married	Yes	Yes	3rd	3	2	2 Yes
Orthodox	Married	No			3	3	3 No
Orthodox	Married	Yes	Yes	9th	2	1	1 Yes
Orthodox	Married	No			5	5	5 No
Orthodox	Married	Yes	Yes	12th	3	3	3 No
Orthodox	Married	No			1	1	1 No
Orthodox	Married	Yes	Yes	5th	7	7	7 No
Orthodox	Married	Yes	Yes	3rd	5	2	2 Yes
Orthodox	Married	Yes	Yes	8th	1	1	1 No
Orthodox	Married	Yes	Yes	4th	2	2	2 No
Orthodox	Married	Yes	Yes	10th	1	1	1 No
Orthodox	Married	Yes	Yes	6th	3	3	3 No
Orthodox	Married	Yes	Yes	6th	2	2	2 No
Orthodox	Married	Yes	Yes	8th	2	2	2 No
Orthodox	Married	Yes	Yes	9th	2	2	2 No
Orthodox	Married	Yes	Yes	6th	5	5	5 No
Orthodox	Married	No			5	5	5 No
Orthodox	Married	No			2	2	2 No
Orthodox	Married	Yes	No	1st	4	4	4 No
Orthodox	Married	Yes	Yes	12th	1	1	1 No
Orthodox	Married	Yes	Yes	7th	1	1	1 No
Orthodox	Never married				1	1	1 No
Orthodox	Married	Yes	Yes	10th	1	1	1 No
Orthodox	Married	Yes	Yes	6th	3	3	3 No
Orthodox	Married	Yes	Yes	10th	2	2	2 No
Orthodox	Never married				2	2	2 No
Orthodox	Widowed				3	3	3 No
Orthodox	Married	No			2	2	2 No
Orthodox	Married	Yes	No	1st	4	3	3 Yes
Orthodox	Married	Yes	Yes	1st	3	0	3 No
Orthodox	Married	Yes	Yes	4th	3	3	3 No

For peer review only

Orthodox	Married	Yes	Yes	6th	3	0	3	No
Orthodox	Married	No			3	3	3	No
Orthodox	Married	Yes	Yes	4th	3	6	3	No
Orthodox	Married	Yes	Yes	University/	2	2	2	No
Orthodox	Married	Yes	Yes	3rd	4	4	4	Yes
Orthodox	Married	No			1	1	1	No
Orthodox	Married	No			1	1	1	No
Orthodox	Married	Yes	Yes	9th	2	2	2	No
Orthodox	Married	Yes	Yes	10th	1	1	1	No
Orthodox	Married	Yes	Yes	10th	1	1	1	No
Orthodox	Married	Yes	Yes	2nd	6	6	6	No
Orthodox	Married	Yes	Yes	7th	2	2	2	No
Orthodox	Married	Yes	Yes	3rd	1	1	1	No
Orthodox	Married	No			3	3	3	No
Orthodox	Married	No			6	6	6	No
Orthodox	Married	Yes	Yes	3rd	3	3	3	No
Orthodox	Married	Yes	Yes	12th	2	2	2	No
Orthodox	Married	Yes	Yes	7th	2	2	2	No
Orthodox	Married	Yes	Yes	University/	1	1	1	No
Orthodox	Married	Yes	Yes	5th	3	3	4	No
Orthodox	Married	Yes	Yes	10th	1	1	1	No
Orthodox	Married	Yes	Yes	4th	2	2	2	No
Orthodox	Married	Yes	Yes	7th	2	2	2	No
Orthodox	Divorced				2	2	2	No
Orthodox	Married	Yes	Yes	7th	1	1	1	No
Orthodox	Married	Yes	Yes	8th	2	2	2	No
Orthodox	Married	Yes	Yes	3rd	1	1	1	No
Orthodox	Married	Yes	Yes	5th	1	1	1	No
Orthodox	Married	Yes	No	1st	1	0	1	No
Orthodox	Married	Yes	Yes	University/	7	7	7	No
Orthodox	Married	No			1	1	1	No
Orthodox	Married	No			2	2	1	No
Orthodox	Married	Yes	Yes	10th	2	2	2	No
Orthodox	Married	Yes	Yes	3rd	1	1	1	No
Orthodox	Married	Yes	Yes	4th	2	2	2	No
Orthodox	Married	No			1	1	1	No
Orthodox	Married	Yes	Yes	6th	3	3	3	No
Orthodox	Married	Yes	Yes	8th	2	2	2	No
Orthodox	Married	Yes	Yes	8th	2	2	2	No
Orthodox	Married	Yes	Yes	10th	2	2	2	No
Orthodox	Married	Yes	Yes	7th	2	2	2	No
Orthodox	Married	Yes	Yes	7th	1	1	1	No
Orthodox	Married	Yes	Yes	8th	2	2	2	No
Orthodox	Married	Yes	Yes	4th	5	5	5	No
Orthodox	Married	Yes	Yes	8th	2	2	2	No
Orthodox	Married	Yes	Yes	10th	1	1	1	No
Orthodox	Married	Yes	Yes	8th	1	1	1	No
Orthodox	Married	Yes	Yes	9th	2	2	2	No
Orthodox	Married	No			4	4	4	No
Orthodox	Married	No			4	4	4	No
Orthodox	Married	Yes	Yes	3rd	6	6	6	No
Orthodox	Married	Yes	Yes	10th	2	2	2	No

For peer review only

Orthodox	Married	No			6	6	6	No
Orthodox	Married	No			2	2	2	No
Orthodox	Married	Yes	Yes	10th	1	1	1	No
Orthodox	Married	Yes	Yes	10th	1	1	1	No
Orthodox	Married	No			2	2	2	No
Orthodox	Married	Yes	Yes	4th	2	2	2	No
Orthodox	Married	Yes	Yes	4th	2	2	2	No
Orthodox	Married	No			2	2	2	No
Orthodox	Married	Yes	Yes	7th	1	1	1	No
Orthodox	Married	Yes	Yes	10th	1	1	1	No
Orthodox	Married	Yes	Yes	10th	1	1	1	No
Orthodox	Married	Yes	Yes	6th	3	3	3	No
Orthodox	Married	Yes	Yes	5th	3	3	3	No
Orthodox	Married	Yes	Yes	5th	2	2	2	No
Orthodox	Married	Yes	Yes	10th	3	3	3	No
Orthodox	Married	Yes	Yes	8th	2	2	2	No
Orthodox	Married	Yes	Yes	9th	1	1	1	No
Orthodox	Married	Yes	Yes	University/	1	1	1	No
Orthodox	Married	No			3	3	3	No
Orthodox	Never married				5	5	5	No
Orthodox	Married	No			3	2	2	Yes
Orthodox	Married	No			5	5	5	No
Orthodox	Married	Yes	No	1st	5	5	5	No
Orthodox	Married	Yes	Yes	8th	2	4	4	No
Orthodox	Married	Yes	No	1st	5	5	4	No
Orthodox	Married	No			4	4	4	No
Orthodox	Married	No			4	4	4	No
Orthodox	Married	Yes	Yes	4th	8	7	7	Yes
Orthodox	Married	No			1	1	1	No
Orthodox	Married	No			1	1	1	No
Orthodox	Married	Yes	No	2nd	12	10	10	Yes
Orthodox	Married	Yes	Yes	3rd	2	2	2	No
Orthodox	Married	No			3	3	3	No
Orthodox	Married	Yes	Yes	4th	4	4	4	No
Orthodox	Married	Yes	No	3rd	8	5	5	Yes
Orthodox	Married	No			5	5	7	No
Orthodox	Married	No			3	3	4	No
Orthodox	Married	Yes	Yes	9th	2	1	1	Yes
Orthodox	Married	No			3	3	3	No
Orthodox	Married	No			5	5	5	No
Orthodox	Married	No			5	5	5	No
Orthodox	Married	Yes	Yes	3rd	4	3	3	Yes
Orthodox	Married	No			7	6	6	Yes
Orthodox	Married	No			6	6	6	No
Orthodox	Married	No			6	4	4	Yes
Orthodox	Married	Yes	Yes	10th	2	2	2	No
Orthodox	Married	Yes	Yes	6th	2	2	2	No
Orthodox	Married	No			6	6	6	No
Orthodox	Married	Yes	Yes	3rd	5	2	2	Yes
Orthodox	Married	Yes	Yes	6th	2	2	2	No
Orthodox	Married	No			8	6	6	Yes
Orthodox	Married	Yes	Yes	10th	1	1	1	No

For peer review only

Orthodox	Married	Yes	Yes	2nd	1	1	1	No
Orthodox	Married	No			2	2	2	No
Orthodox	Married	No			2	2	2	No
Orthodox	Married	No			7	7	7	No
Orthodox	Married	Yes	Yes	3rd	3	3	3	No
Orthodox	Married	Yes	Yes	4th	6	5	5	Yes
Orthodox	Married	No			10	10	6	No
Orthodox	Married	Yes	Yes	4th	8	8	8	No
Orthodox	Married	Yes	Yes	8th	2	2	2	No
Orthodox	Married	Yes	No	1st	4	4	4	No
Orthodox	Married	Yes	Yes	6th	7	5	4	Yes
Orthodox	Married	Yes	Yes	2nd	5	5	5	No
Orthodox	Married	Yes	Yes	2nd	4	4	4	No
Orthodox	Married	Yes	Yes	8th	1	1	1	No
Orthodox	Married	Yes	Yes	10th	4	4	4	No
Orthodox	Married	No			2	2	2	No
Orthodox	Married	No			3	3	3	No
Orthodox	Married	No			6	6	6	No
Orthodox	Married	No			3	3	3	No
Orthodox	Married	Yes	Yes	4th	9	9	7	No
Orthodox	Married	Yes	Yes	3rd	5	5	5	No
Orthodox	Married	Yes	No	1st	8	8	8	No
Orthodox	Married	Yes	Yes	3rd	5	5	5	No
Orthodox	Married	Yes	No	1st	2	2	2	No
Orthodox	Married	Yes	Yes	1st	7	6	6	Yes
Orthodox	Married	No			7	7	7	No
Orthodox	Married	No			2	2	2	No
Orthodox	Married	Yes	Yes	3rd	4	4	1	No
Orthodox	Married	Yes	Yes	8th	2	2	2	No
Orthodox	Married	Yes	Yes	7th	6	6	6	No
Orthodox	Married	No			4	3	3	Yes
Orthodox	Married	No			2	2	2	No
Orthodox	Married	Yes	Yes	3rd	4	4	4	No
Orthodox	Married	Yes	Yes	3rd	2	2	2	No
Orthodox	Married	Yes	No	2nd	7	6	6	Yes
Orthodox	Married	Yes	No	1st	8	8	8	No
Orthodox	Married	Yes	Yes	2nd	7	7	7	No
Orthodox	Married	Yes	Yes	6th	4	4	4	No
Orthodox	Divorced				1	1	1	No
Orthodox	Married	No			3	3	3	No
Orthodox	Married	Yes	Yes	5th	6	5	5	Yes
Orthodox	Married	No			5	5	5	No
Orthodox	Married	No			5	5	5	No
Orthodox	Married	Yes	Yes	3rd	3	2	2	Yes
Orthodox	Married	Yes	Yes	4th	5	5	5	No
Orthodox	Married	No			8	8	6	No
Orthodox	Married	Yes	Yes	University/	1	1	1	No
Orthodox	Married	No			7	5	5	Yes
Orthodox	Married	Yes	Yes	6th	1	1	1	No
Orthodox	Married	No			1	1	1	No
Orthodox	Married	No			2	2	2	No
Orthodox	Married	No			4	4	4	No

For peer review only

Orthodox	Married	Yes	Yes	4th	2	2	2	No
Orthodox	Married	Yes	Yes	10th	3	3	3	No
Orthodox	Married	Yes	Yes	7th	3	3	3	No
Orthodox	Married	Yes	Yes	3rd	3	3	3	No
Orthodox	Married	No			6	5	5	No
Orthodox	Married	No			1	1	1	No
Orthodox	Married	Yes	Yes	University/	1	1	1	No
Orthodox	Married	Yes	Yes	5th	2	2	2	No
Orthodox	Married	No			6	6	6	No
Orthodox	Married	Yes	Yes	3rd	1	1	1	No
Orthodox	Married	No			5	5	5	No
Orthodox	Married	No			1	1	1	No
Orthodox	Married	Yes	Yes	9th	9	8	8	Yes
Orthodox	Married	Yes	Yes	7th	4	4	4	No
Orthodox	Married	Yes	Yes	7th	1	1	1	No
Orthodox	Married	Yes	No	1st	6	6	6	No
Orthodox	Married	No			4	4	4	No
Orthodox	Divorced				5	5	5	No
Orthodox	Separated				1	1	1	No
Protestant	Married	No			3	3	2	Yes
Orthodox	Married	Yes	Yes	6th	7	5	7	Yes
Orthodox	Married	No			1	1	1	No
Orthodox	Married	No			4	4	4	No
Protestant	Married	Yes	Yes	10th	2	2	2	No
Protestant	Married	Yes	Yes	6th	2	2	2	No
Protestant	Married	Yes	Yes	10th	2	2	2	No
Orthodox	Married	No			3	3	3	No
Orthodox	Married	No			4	3	3	Yes
Orthodox	Married	Yes	Yes	5th	1	1	1	No
Protestant	Married	Yes	Yes	5th	2	2	2	No
Orthodox	Married	No			1	1	1	No
Orthodox	Married	Yes	Yes	2nd	2	2	2	No
Orthodox	Married	No			1	1	1	No
Protestant	Married	Yes	Yes	3rd	1	1	1	No
Protestant	Married	No			2	2	2	No
Orthodox	Married	Yes	Yes	3rd	1	1	1	No
Orthodox	Married	Yes	Yes	4th	1	1	1	No
Protestant	Married	Yes	Yes	1st	2	2	2	No
Orthodox	Married	No			1	1	1	No
Orthodox	Married	No			4	4	4	No
Protestant	Married	Yes	Yes	8th	1	1	1	No
Orthodox	Married	Yes	Yes	3rd	1	1	1	No
Orthodox	Married	No			2	2	2	No
Orthodox	Married	Yes	Yes	7th	1	1	1	No
Protestant	Married	No			8	8	8	No
Orthodox	Married	Yes	Yes	8th	1	1	1	No
Protestant	Married	Yes	Yes	9th	5	5	5	No
Orthodox	Married	Yes	Yes	10th	2	2	2	No
Orthodox	Married	Yes	Yes	8th	2	2	2	No
Orthodox	Married	Yes	Yes	6th	1	1	1	No
Orthodox	Married	No			4	4	4	No
Orthodox	Married	Yes	Yes	4th	1	1	1	No

For peer review only

Orthodox	Married	Yes	Yes	9th	1	1	1	No
Orthodox	Married	No			4	4	4	No
Protestant	Married	No			3	3	3	No
Orthodox	Married	Yes	Yes	4th	6	6	6	No
Orthodox	Married	Yes	Yes	10th	1	1	1	No
Orthodox	Married	Yes	Yes	3rd	1	1	1	No
Orthodox	Married	Yes	Yes	4th	3	3	3	No
Orthodox	Married	Yes	Yes	5th	5	5	4	No
Orthodox	Married	Yes	Yes	6th	4	4	4	No
Orthodox	Married	No			4	4	4	No
Orthodox	Married	Yes	Yes	7th	2	2	2	No
Orthodox	Married	No			5	5	5	No
Orthodox	Married	Yes	Yes	5th	3	3	3	No
Orthodox	Married	Yes	Yes	10th	3	3	3	Yes
Orthodox	Married	No			2	3	3	No
Orthodox	Married	No			4	4	4	No
Orthodox	Married	No			4	3	3	Yes
Orthodox	Married	No			6	5	5	Yes
Orthodox	Married	Yes	Yes	7th	3	3	3	No
Orthodox	Married	No			3	3	3	No
Orthodox	Married	Yes	Yes	8th	2	2	2	No
Orthodox	Married	Yes	Yes	5th	4	4	4	No
Orthodox	Married	Yes	No	1st	3	3	3	No
Orthodox	Married	Yes	Yes	6th	4	4	4	No
Orthodox	Married	Yes	Yes	10th	5	5	4	No
Orthodox	Married	Yes	Yes	8th	3	3	3	No
Orthodox	Married	Yes	No	1st	4	4	4	No
Orthodox	Married	Yes	Yes	5th	3	3	3	No
Orthodox	Married	Yes	Yes	5th	5	5	5	No
Orthodox	Married	Yes	Yes	7th	4	3	3	Yes
Orthodox	Married	Yes	Yes	Technical/\	2	2	2	No
Orthodox	Married	No			2	2	2	No
Orthodox	Married	Yes	Yes	4th	2	2	2	No
Orthodox	Married	No			10	10	10	No
Orthodox	Married	Yes	Yes	10th	3	2	2	Yes
Orthodox	Married	No			5	2	2	Yes
Orthodox	Married	Yes	Yes	2nd	1	1	1	No
Orthodox	Married	Yes	Yes	3rd	3	3	3	No
Orthodox	Married	Yes	Yes	6th	3	3	3	No
Orthodox	Married	Yes	Yes	10th	3	3	3	No
Orthodox	Married	Yes	Yes	1st	4	4	4	No
Orthodox	Married	Yes	Yes	5th	6	6	7	No
Orthodox	Married	Yes	Yes	5th	2	2	2	No
Orthodox	Married	Yes	No	1st	9	8	8	Yes
Orthodox	Married	Yes	Yes	University/	2	2	2	No
Orthodox	Married	Yes	Yes	10th	10	10	10	Yes
Orthodox	Married	No			4	4	4	No
Orthodox	Married	Yes	Yes	8th	2	2	2	No
Orthodox	Married	No			3	2	2	Yes
Orthodox	Married	No			5	5	5	No
Orthodox	Married	No			2	2	2	No
Orthodox	Married	Yes	Yes	4th	6	6	6	No

For peer review only

Orthodox	Married	Yes	Yes	6th	6	6	6	No
Orthodox	Married	No			1	1	1	No
Orthodox	Divorced				1	1	1	No
Orthodox	Married	Yes	Yes	5th	2	2	2	No
Orthodox	Married	No			9	9	9	No
Orthodox	Married	No			3	3	3	No
Orthodox	Married	No			1	1	1	No
Orthodox	Married	No			5	5	5	No
Orthodox	Married	No			1	1	1	No
Orthodox	Married	Yes	Yes	6th	3	3	3	No
Orthodox	Married	Yes	Yes	3rd	3	3	3	No
Orthodox	Married	Yes	Yes	4th	4	4	4	No
Orthodox	Married	Yes	Yes	10th	2	2	2	No
Orthodox	Married	No			5	5	6	No
Orthodox	Married	Yes	Yes	5th	4	4	4	No
Orthodox	Married	Yes	Yes	10th	1	1	1	No
Orthodox	Married	Yes	Yes	1st	4	5	4	No
Orthodox	Married	Yes	Yes	7th	3	3	3	No
Orthodox	Married	Yes	Yes	8th	1	1	1	No
Orthodox	Married	No			5	5	5	No
Orthodox	Married	Yes	Yes	10th	2	2	2	No
Orthodox	Married	Yes	Yes	4th	2	2	2	No
Orthodox	Married	Yes	Yes	3rd	4	4	4	No
Orthodox	Married	No			2	2	2	No
Orthodox	Married	Yes	Yes	9th	1	1	1	No
Orthodox	Married	Yes	Yes	University/	5	4	4	No
Orthodox	Married	Yes	No	2nd	8	8	8	No
Orthodox	Married	No			5	5	5	No
Orthodox	Married	No			3	3	3	No
Orthodox	Married	No			1	1	1	No
Orthodox	Married	Yes	Yes	4th	5	5	5	No
Orthodox	Married	Yes	Yes	5th	1	1	1	No
Orthodox	Married	No			8	8	8	No
Orthodox	Married	No			7	7	7	No
Orthodox	Married	No			7	7	7	No
Orthodox	Married	Yes	Yes	6th	6	6	6	No
Orthodox	Married	Yes	No	2nd	2	2	2	No
Orthodox	Married	No			5	5	5	No
Orthodox	Married	Yes	Yes	6th	3	3	3	No
Orthodox	Married	No			3	3	3	No
Orthodox	Married	Yes	Yes	6th	1	1	1	No
Orthodox	Married	No			5	5	5	No
Orthodox	Married	No			6	6	6	No
Orthodox	Married	Yes	Yes	7th	2	2	2	No
Orthodox	Married	No			5	5	5	No
Orthodox	Married	Yes	Yes	9th	3	3	3	No
Orthodox	Married	No			5	4	4	Yes
Orthodox	Married	Yes	Yes	4th	5	5	5	No
Orthodox	Married	Yes	Yes	6th	1	1	1	No
Orthodox	Married	Yes	No	2nd	5	5	5	No
Orthodox	Married	Yes	Yes	6th	6	6	6	No
Orthodox	Married	No			3	3	3	No

For peer review only

Orthodox	Married	No			5	5	5	No
Orthodox	Married	Yes	Yes	6th	5	5	5	No
Orthodox	Married	No			8	8	8	No
Orthodox	Married	No			5	5	5	No
Orthodox	Married	No			1	0	1	No
Orthodox	Married	No			7	6	6	Yes
Orthodox	Married	Yes	Yes	6th	2	2	2	No
Orthodox	Married	Yes	Yes	5th	3	3	3	No
Orthodox	Married	No			6	6	6	No
Orthodox	Married	No			7	5	5	Yes
Orthodox	Married	Yes	Yes	3rd	1	1	1	No
Orthodox	Married	Yes	Yes	10th	1	1	1	No
Orthodox	Married	Yes	Yes	8th	2	2	2	No
Orthodox	Married	No			5	5	5	No
Orthodox	Married	No			9	8	8	Yes
Orthodox	Married	No			5	5	5	No
Orthodox	Married	Yes	Yes	3rd	4	4	4	No
Orthodox	Married	No			2	2	2	No
Orthodox	Married	No			3	3	3	No
Orthodox	Married	Yes	Yes	10th	1	1	1	No
Orthodox	Married	Yes	No	8th	5	5	5	Yes
Orthodox	Married	Yes	Yes	8th	1	1	1	No
Orthodox	Married	No			6	6	6	No
Orthodox	Married	No			3	4	3	Yes
Orthodox	Married	No			1	1	1	No
Orthodox	Married	No			3	3	3	No
Orthodox	Married	Yes	Yes	4th	2	2	2	No
Orthodox	Married	No			4	4	4	No
Orthodox	Married	No			3	3	3	No
Orthodox	Married	No			1	1	1	No
Orthodox	Married	Yes	Yes	2nd	8	8	8	No
Orthodox	Married	Yes	Yes	6th	5	5	5	No
Orthodox	Married	No			7	6	6	Yes
Orthodox	Married	No			5	5	5	No
Orthodox	Married	Yes	Yes	4th	1	1	1	No
Orthodox	Married	No			4	3	3	No
Orthodox	Married	No			6	7	7	No
Orthodox	Married	Yes	No	8th	7	7	7	No
Orthodox	Married	Yes	No	2nd	4	4	4	No
Protestant	Married	Yes	Yes	5th	1	1	1	No
Orthodox	Married	No			6	6	6	No
Orthodox	Married	Yes	Yes	5th	2	2	2	No
Protestant	Married	Yes	Yes	6th	1	1	1	No
Orthodox	Married	No			2	2	2	No
Orthodox	Married	No			3	2	2	Yes
Orthodox	Married	Yes	Yes	10th	2	2	2	No
Orthodox	Married	Yes	Yes	8th	1	1	1	No
Orthodox	Married	No			3	3	3	No
Orthodox	Married	No			3	3	3	No
Orthodox	Married	Yes	Yes	6th	2	2	2	No
Orthodox	Married	Yes	Yes	6th	1	1	1	No
Orthodox	Married	Yes	Yes	9th	4	4	4	No

For peer review only

Orthodox	Married	No			7	7	7	No
Orthodox	Married	No			1	1	1	No
Orthodox	Married	Yes	Yes	10th	5	5	6	No
Orthodox	Married	No			3	3	3	No
Orthodox	Married	Yes	Yes	4th	7	7	7	No
Orthodox	Divorced				2	2	2	No
Orthodox	Married	No			2	2	2	No
Orthodox	Married	No			2	2	2	No
Orthodox	Married	Yes	Yes	6th	1	1	1	No
Orthodox	Married	No			1	1	1	No
Orthodox	Married	Yes	Yes	10th	2	2	2	No
Orthodox	Married	No			3	3	3	No
Orthodox	Married	Yes	Yes	10th	3	3	3	No
Orthodox	Married	Yes	Yes	9th	3	3	3	No
Orthodox	Married	Yes	Yes	10th	1	1	1	No
Orthodox	Married	Yes	Yes	4th	2	2	2	No
Orthodox	Married	Yes	Yes	8th	2	1	1	Yes
Orthodox	Married	Yes	Yes	7th	2	2	2	No
Protestant	Married	Yes	Yes	4th	2	2	2	No
Orthodox	Married	Yes	Yes	4th	2	2	2	No
Orthodox	Married	Yes	Yes	10th	1	1	1	No
Orthodox	Married	Yes	Yes	6th	2	2	2	No
Protestant	Married	Yes	Yes	6th	3	3	3	No
Protestant	Married	Yes	Yes	8th	1	1	1	No
Orthodox	Married	No			10	10	10	No
Orthodox	Married	No			3	3	3	No
Orthodox	Married	Yes	Yes	10th	2	2	2	No
Protestant	Married	Yes	Yes	4th	2	2	2	No
Protestant	Married	Yes	Yes	7th	1	1	1	No
Orthodox	Married	Yes	Yes	5th	3	3	3	No
Orthodox	Married	Yes	Yes	1st	2	2	2	No
Orthodox	Married	Yes	Yes	7th	2	2	2	No
Orthodox	Married	No			4	4	4	No
Orthodox	Married	No			1	1	1	No
Orthodox	Married	Yes	Yes	10th	2	2	2	No
Orthodox	Married	No			5	5	5	No
Orthodox	Married	Yes	Yes	3rd	2	2	2	No
Orthodox	Married	Yes	Yes	11th	1	1	1	No
Orthodox	Married	Yes	Yes	7th	3	3	3	No
Orthodox	Married	Yes	Yes	12th	4	2	2	Yes
Orthodox	Married	Yes	Yes	8th	2	2	2	No
Orthodox	Married	No			4	4	4	No
Orthodox	Never married				1	1	1	No
Orthodox	Married	Yes	Yes	8th	2	2	2	No
Protestant	Married	No			5	5	5	No
Orthodox	Married	No			4	4	4	No
Orthodox	Married	No			3	3	3	No
Orthodox	Married	Yes	Yes	6th	2	2	2	No
Protestant	Married	Yes	Yes	6th	1	1	1	No
Orthodox	Married	Yes	Yes	6th	2	2	2	No
Orthodox	Married	Yes	Yes	8th	1	1	1	No
Orthodox	Married	Yes	Yes	3rd	2	2	2	No

For peer review only

Protestant	Married	Yes	Yes	12th	3	3	3	No
Protestant	Married	Yes	Yes	10th	1	1	1	No
Orthodox	Married	No			1	1	1	No
Orthodox	Married	Yes	Yes	9th	2	1	1	Yes
Orthodox	Married	Yes	Yes	10th	2	2	2	No
Orthodox	Married	Yes	Yes	8th	6	6	6	No
Protestant	Married	Yes	Yes	5th	1	1	1	No
Orthodox	Married	Yes	Yes	10th	3	3	3	No
Orthodox	Married	Yes	Yes	6th	2	2	2	No
Protestant	Married	Yes	Yes	10th	10	2	2	No
Orthodox	Married	No			3	3	3	No
Orthodox	Married	Yes	Yes	7th	1	1	1	No
Orthodox	Married	No			1	1	1	No
Orthodox	Married	Yes	Yes	6th	1	1	1	No
Protestant	Married	Yes	Yes	9th	2	2	2	No
Orthodox	Married	Yes	Yes	6th	1	1	1	No
Orthodox	Married	Yes	Yes	10th	2	2	2	No
Orthodox	Married	No			5	5	5	Yes
Orthodox	Married	Yes	Yes	10th	2	2	2	No
Orthodox	Married	Yes	Yes	6th	3	2	2	Yes
Orthodox	Married	Yes	Yes	9th	2	2	2	No
Orthodox	Married	Yes	Yes	6th	3	3	3	No
Orthodox	Married	Yes	Yes	10th	1	1	1	No
Protestant	Married	No			1	1	1	No
Orthodox	Never married				1	1	1	No
Orthodox	Married	Yes	Yes	9th	2	2	2	No
Orthodox	Married	Yes	Yes	8th	1	1	1	No
Orthodox	Married	Yes	Yes	1st	2	2	2	No
Orthodox	Married	Yes	Yes	4th	2	2	2	No
Protestant	Married	Yes	Yes	2nd	9	9	9	No
Orthodox	Married	Yes	Yes	8th	1	1	1	No
Orthodox	Married	Yes	Yes	9th	3	3	3	No
Protestant	Separated				1	1	1	No
Orthodox	Married	Yes	Yes	2nd	7	7	7	No
Orthodox	Married	No			2	2	2	No
Protestant	Married	Yes	Yes	8th	2	2	2	No
Orthodox	Married	Yes	Yes	Technical/v	2	2	2	No
Orthodox	Married	No			1	1	1	No
Orthodox	Married	Yes	Yes	5th	2	2	2	No
Orthodox	Married	No			6	6	6	No
Orthodox	Married	Yes	Yes	7th	1	1	1	No
Orthodox	Married	Yes	Yes	10th	1	1	1	No
Orthodox	Married	No			2	2	2	No
Orthodox	Married	Yes	Yes	6th	4	4	4	No
Orthodox	Married	No			2	2	2	No
Orthodox	Married	No			1	1	1	No
Orthodox	Married	Yes	No	1st	1	1	1	No
Orthodox	Married	Yes	No	2nd	10	10	10	No
Protestant	Married	Yes	Yes	10th	1	1	1	No
Protestant	Married	Yes	Yes	6th	8	8	8	No
Orthodox	Married	Yes	Yes	1st	3	3	3	No
Orthodox	Divorced				7	7	7	Yes

For peer review only

Protestant	Married	Yes	Yes	8th	2	2	2	No
Orthodox	Married	Yes	Yes	8th	1	1	1	No
Traditional	Married	Yes	Yes	11th	1	1	1	No
Orthodox	Married	No			2	1	1	Yes
Orthodox	Married	Yes	Yes	5th	2	2	2	No
Orthodox	Married	Yes	Yes	8th	1	1	1	No
Muslim	Married	Yes	Yes	Technical/\	2	2	2	No
Orthodox	Married	Yes	Yes	9th	3	3	3	No
Protestant	Married	No			4	4	4	No
Orthodox	Married	Yes	Yes	10th	1	1	1	No
Protestant	Married	Yes	Yes	6th	1	1	1	No
Protestant	Married	No			3	3	3	No
Protestant	Married	No			1	1	1	No
Orthodox	Married	Yes	Yes	10th	1	1	1	No
Orthodox	Married	Yes	Yes	10th	5	5	5	Yes
Orthodox	Married	Yes	Yes	10th	1	1	1	No
Orthodox	Married	Yes	Yes	1st	2	2	2	No
Protestant	Married	Yes	Yes	10th	3	3	3	No
Protestant	Married	No			4	4	4	No
Orthodox	Married	Yes	Yes	10th	4	4	4	No
Orthodox	Married	Yes	Yes	8th	2	2	1	No
Orthodox	Married	Yes	Yes	10th	4	4	4	No
Orthodox	Married	No			2	2	2	No
Protestant	Married	Yes	Yes	10th	1	1	1	No
Protestant	Married	Yes	Yes	10th	1	1	1	No
Orthodox	Married	Yes	Yes	4th	1	1	1	No
Orthodox	Married	Yes	Yes	3rd	1	1	1	No
Protestant	Married	Yes	Yes	7th	1	1	1	No
Orthodox	Married	Yes	Yes	6th	1	1	1	No
Orthodox	Married	Yes	Yes	6th	1	1	1	No
Protestant	Married	Yes	Yes	10th	2	2	2	No
Orthodox	Married	Yes	Yes	University/	2	2	2	No
Protestant	Married	Yes	Yes	10th	1	1	1	No
Orthodox	Married	No			4	4	4	No
Orthodox	Married	Yes	Yes	4th	5	4	4	Yes
Orthodox	Married	Yes	Yes	4th	3	3	3	No
Orthodox	Married	No			2	2	2	No
Orthodox	Married	Yes	Yes	2nd	4	4	4	No
Orthodox	Married	Yes	Yes	6th	2	2	2	No
Orthodox	Married	Yes	Yes	7th	2	2	2	No
Orthodox	Married	Yes	Yes	9th	2	2	2	No
Orthodox	Married	Yes	Yes	4th	6	6	6	No
Orthodox	Married	Yes	Yes	6th	4	4	4	No
Orthodox	Married	Yes	Yes	10th	1	1	1	No
Protestant	Married	No			8	8	8	No
Orthodox	Married	Yes	Yes	5th	3	3	3	No
Orthodox	Married	No			3	3	3	No
Orthodox	Married	Yes	Yes	9th	3	2	2	Yes
Orthodox	Never married				1	1	1	No
Orthodox	Married	No			3	3	3	No
Orthodox	Married	Yes	Yes	5th	1	1	1	No
Orthodox	Married	Yes	Yes	1st	6	6	5	No

For peer review only

Orthodox	Married	Yes	Yes	8th	2	2	2	No
Orthodox	Married	Yes	Yes	10th	2	2	1	No
Orthodox	Married	Yes	Yes	University/	3	3	3	No
Orthodox	Married	Yes	Yes	10th	1	1	1	No
Orthodox	Married	Yes	Yes	5th	1	1	1	No
Orthodox	Married	Yes	Yes	4th	3	3	3	No
Orthodox	Married	Yes	Yes	Technical/\	2	2	2	No
Orthodox	Married	No			2	2	2	No
Orthodox	Married	No			2	2	2	No
Orthodox	Married	Yes	Yes	1st	4	4	4	No
Orthodox	Married	Yes	Yes	10th	1	1	2	No
Orthodox	Married	Yes	Yes	8th	2	2	2	No
Muslim	Married	No			1	1	1	No
Orthodox	Married	Yes	Yes	12th	1	1	1	No
Orthodox	Married	Yes	Yes	8th	3	3	3	No
Orthodox	Married	No			4	4	4	No
Orthodox	Married	Yes	No	8th	7	7	7	No
Orthodox	Divorced				2	2	2	No
Orthodox	Married	Yes	Yes	10th	3	3	3	No
Orthodox	Married	No			4	4	4	No
Orthodox	Married	Yes	Yes	10th	1	1	1	No
Orthodox	Married	No			3	3	3	No
Orthodox	Married	Yes	Yes	5th	3	3	3	No
Orthodox	Married	Yes	Yes	10th	2	2	2	No
Orthodox	Married	Yes	Yes	10th	3	3	4	No
Orthodox	Married	Yes	Yes	8th	1	1	1	No
Orthodox	Married	No			8	7	7	Yes
Orthodox	Married	Yes	Yes	6th	1	1	1	No
Orthodox	Married	Yes	Yes	7th	2	2	2	No
Orthodox	Married	Yes	Yes	6th	1	1	1	No
Orthodox	Married	Yes	Yes	1st	4	4	4	No
Orthodox	Married	Yes	Yes	8th	1	1	1	No
Orthodox	Married	Yes	Yes	6th	1	1	1	No
Orthodox	Married	Yes	Yes	8th	3	3	3	No
Orthodox	Married	No			3	3	3	No
Traditional	Married	Yes	Yes	9th	4	4	4	No
Orthodox	Married	No			3	3	3	No
Orthodox	Married	Yes	Yes	5th	1	1	1	No
Orthodox	Married	No			4	4	4	No
Protestant	Married	No			2	2	2	No
Orthodox	Married	Yes	Yes	8th	1	1	1	No
Orthodox	Divorced				2	2	2	No
Orthodox	Married	Yes	Yes	7th	1	1	1	No
Orthodox	Married	No			2	2	2	No
Muslim	Married	Yes	Yes	5th	1	1	1	No
Orthodox	Married	Yes	Yes	8th	1	1	1	No
Protestant	Married	Yes	Yes	7th	1	1	1	No
Orthodox	Married	Yes	Yes	6th	1	1	1	No
Orthodox	Divorced				1	1	1	No
Orthodox	Married	Yes	Yes	10th	1	1	1	No
Orthodox	Married	Yes	Yes	10th	1	1	1	No
Protestant	Married	Yes	Yes	10th	3	3	3	No

For peer review only

Orthodox	Married	Yes	Yes	9th	3	2	2	Yes
Orthodox	Married	Yes	Yes	7th	3	3	3	No
Orthodox	Married	Yes	Yes	10th	3	2	2	Yes
Orthodox	Married	No			5	5	5	No
Protestant	Married	Yes	Yes	9th	1	1	1	No
Orthodox	Married	Yes	Yes	8th	1	1	1	No
Orthodox	Married	No			1	1	1	No
Orthodox	Married	Yes	Yes	6th	1	1	1	No
Orthodox	Married	Yes	No	2nd	5	5	5	No
Orthodox	Married	Yes	Yes	9th	1	1	1	No
Orthodox	Married	Yes	Yes	12th	3	3	3	No
Traditional	Married	No			7	7	7	No
Orthodox	Married	Yes	Yes	7th	1	1	1	No
Orthodox	Married	Yes	Yes	5th	2	2	2	No
Orthodox	Married	Yes	Yes	8th	1	1	1	No
Orthodox	Married	Yes	Yes	8th	1	1	1	No
Orthodox	Married	Yes	Yes	4th	3	3	3	Yes
Orthodox	Married	No			3	3	3	No
Protestant	Married	Yes	Yes	7th	2	2	2	No
Protestant	Married	Yes	Yes	10th	1	1	1	No
Orthodox	Married	Yes	Yes	9th	3	3	3	No
Orthodox	Married	Yes	Yes	8th	2	2	2	No
Orthodox	Married	Yes	Yes	3rd	1	1	1	No
Orthodox	Married	Yes	Yes	9th	1	1	1	No
Orthodox	Married	Yes	Yes	10th	3	3	3	No
Orthodox	Married	Yes	Yes	4th	5	5	5	No
Orthodox	Married	No			2	2	2	No
Orthodox	Married	Yes	Yes	9th	2	2	2	No
Orthodox	Married	Yes	Yes	3rd	5	5	5	No
Orthodox	Married	No			5	4	4	Yes
Orthodox	Married	Yes	Yes	8th	1	1	1	No
Orthodox	Married	Yes	Yes	8th	1	1	1	No
Orthodox	Married	No			2	2	2	No
Orthodox	Married	Yes	Yes	5th	1	1	1	No
Orthodox	Married	Yes	Yes	10th	2	2	2	No
Orthodox	Married	No			5	5	5	No
Orthodox	Married	Yes	Yes	10th	3	3	3	No
Orthodox	Married	Yes	Yes	10th	1	1	1	No
Orthodox	Married	Yes	Yes	8th	1	1	1	No
Orthodox	Married	Yes	Yes	University/	2	2	2	No
Orthodox	Married	No			6	6	6	No
Orthodox	Married	Yes	Yes	7th	4	4	4	No
Orthodox	Married	Yes	Yes	Technical/\	2	2	2	No
Orthodox	Married	Yes	Yes	University/	1	1	1	No
Orthodox	Married	Yes	Yes	5th	7	7	7	No
Orthodox	Married	No			10	10	10	No
Orthodox	Married	Yes	Yes	10th	3	3	3	No
Orthodox	Married	Yes	No	8th	4	4	4	No
Orthodox	Married	No			8	8	8	No
Orthodox	Married	Yes	Yes	10th	2	2	2	No
Orthodox	Married	Yes	Yes	6th	4	4	4	No
Orthodox	Married	Yes	Yes	5th	1	1	1	No

For peer review only

Orthodox	Married	No			5	5	5	No
Orthodox	Married	Yes	Yes	10th	8	8	8	No
Orthodox	Married	No			6	6	6	No
Orthodox	Married	Yes	Yes	7th	5	5	5	Yes
Orthodox	Married	No			2	2	2	No
Orthodox	Married	No			1	1	1	No
Orthodox	Married	Yes	Yes	6th	3	3	3	No
Orthodox	Married	Yes	Yes	9th	1	1	1	No
Orthodox	Married	Yes	Yes	5th	2	2	2	No
Orthodox	Never married				1	1	1	No
Orthodox	Married	No			7	7	7	No
Muslim	Married	Yes	Yes	University/	3	3	3	No
Orthodox	Married	Yes	Yes	7th	3	3	3	No
Orthodox	Married	Yes	Yes	3rd	2	2	2	No
Orthodox	Married	No			3	3	3	No
Orthodox	Married	Yes	Yes	9th	2	2	2	No
Orthodox	Married	Yes	Yes	5th	6	6	6	No
Orthodox	Married	Yes	Yes	9th	2	2	2	No
Orthodox	Married	No			2	2	2	No
Orthodox	Married	No			3	3	3	No
Orthodox	Married	No			3	3	3	No
Orthodox	Married	Yes	Yes	4th	3	3	3	No
Orthodox	Married	No			2	2	2	No
Orthodox	Married	No			2	2	2	No
Orthodox	Married	Yes	Yes	8th	1	1	1	No
Orthodox	Married	No			4	4	4	No
Orthodox	Married	Yes	Yes	8th	4	4	4	No
Orthodox	Married	Yes	Yes	10th	2	2	2	No
Orthodox	Married	Yes	Yes	5th	3	2	2	Yes
Orthodox	Married	No			5	5	5	No
Orthodox	Married	Yes	Yes	10th	2	2	2	No
Orthodox	Married	Yes	Yes	2nd	3	3	3	No
Orthodox	Married	No			2	2	2	No
Orthodox	Married	Yes	Yes	10th	1	1	1	No
Orthodox	Married	Yes	Yes	10th	3	3	3	No
Orthodox	Married	Yes	Yes	2nd	3	3	3	No
Orthodox	Married	Yes	Yes	5th	1	1	1	No
Orthodox	Married	No			4	4	4	No
Orthodox	Married	No			5	5	5	No
Orthodox	Married	Yes	Yes	7th	5	5	5	No
Orthodox	Married	No			3	3	3	No
Orthodox	Married	Yes	Yes	6th	2	2	2	No
Orthodox	Married	No			4	4	4	No
Orthodox	Married	Yes	Yes	6th	3	2	2	Yes
Orthodox	Married	Yes	Yes	10th	1	1	1	No
Orthodox	Married	No			6	6	6	No
Orthodox	Married	Yes	Yes	11th	1	1	1	No
Orthodox	Married	Yes	Yes	10th	1	1	1	No
Orthodox	Married	Yes	Yes	7th	1	1	1	No
Orthodox	Married	No			1	1	1	No
Orthodox	Married	No			5	5	5	No
Orthodox	Married	No			3	3	3	No

For peer review only

Orthodox	Married	Yes	Yes	5th	2	2	2	No
Orthodox	Married	No			2	2	2	No
Orthodox	Married	No			2	2	2	No
Orthodox	Married	Yes	Yes	7th	2	2	2	No
Orthodox	Married	Yes	Yes	3rd	1	1	1	No
Orthodox	Married	Yes	Yes	4th	2	2	2	No
Orthodox	Married	Yes	Yes	9th	1	1	1	No
Orthodox	Married	Yes	Yes	10th	1	1	1	No
Orthodox	Married	No			10	10	10	No
Orthodox	Married	Yes	Yes	7th	2	2	2	No
Orthodox	Married	No			3	3	3	No
Orthodox	Married	Yes	Yes	5th	1	1	1	No
Protestant	Married	No			3	3	3	No
Protestant	Married	No			1	1	1	No
Orthodox	Married	Yes	No	1st	5	5	5	No
Protestant	Married	No			4	4	4	No
Orthodox	Married	No			1	1	1	No
Orthodox	Married	Yes	Yes	10th	1	1	1	No
Orthodox	Married	No			3	3	3	No
Orthodox	Married	Yes	Yes	5th	1	1	1	No
Orthodox	Married	Yes	Yes	8th	2	2	2	No
Orthodox	Married	Yes	Yes	7th	1	1	1	No
Orthodox	Married	Yes	Yes	4th	1	1	1	No
Orthodox	Married	Yes	Yes	3rd	8	7	7	Yes
Orthodox	Married	Yes	Yes	3rd	6	6	6	No
Orthodox	Married	No			3	3	3	No
Orthodox	Married	Yes	Yes	10th	1	1	1	No
Orthodox	Married	Yes	Yes	University/	1	1	1	No
Orthodox	Married	Yes	Yes	4th	5	5	5	No
Orthodox	Married	No			6	6	6	No
Orthodox	Married	No			1	1	1	No
Orthodox	Married	Yes	Yes	4th	4	4	4	No
Orthodox	Married	Yes	No	1st	6	6	6	No
Orthodox	Married	Yes	Yes	University/	2	2	2	No
Orthodox	Married	Yes	Yes	3rd	6	6	5	No
Orthodox	Married	No			4	4	4	No
Orthodox	Married	Yes	Yes	2nd	3	3	3	No
Orthodox	Married	Yes	Yes	8th	2	2	2	No
Orthodox	Separated				1	1	1	No
Orthodox	Married	Yes	Yes	10th	1	1	1	No
Orthodox	Separated				1	1	1	No
Orthodox	Married	No			4	2	2	Yes
Orthodox	Married	No			7	7	7	No
Orthodox	Married	No			4	4	4	No
Orthodox	Married	No			3	3	3	No
Protestant	Married	No			2	2	2	No
Orthodox	Married	Yes	Yes	8th	7	7	7	No
Orthodox	Married	Yes	Yes	University/	2	2	2	No
Orthodox	Married	Yes	Yes	5th	4	4	4	No
Orthodox	Married	Yes	Yes	3rd	1	1	1	No
Orthodox	Married	Yes	Yes	4th	3	3	3	No
Orthodox	Married	Yes	Yes	10th	1	1	1	No

For peer review only

Orthodox	Married	No			1	1	1	No
Orthodox	Married	Yes	Yes	Technical/\	1	1	1	No
Orthodox	Married	No			5	5	5	No
Orthodox	Married	Yes	Yes	6th	1	1	1	No
Orthodox	Married	Yes	Yes	10th	1	1	1	No
Orthodox	Married	No			5	5	5	No
Orthodox	Married	Yes	Yes	8th	2	2	2	No
Protestant	Married	No			1	1	1	No
Orthodox	Married	No			2	2	2	No
Orthodox	Married	Yes	Yes	10th	1	1	1	No
Orthodox	Married	Yes	Yes	2nd	1	1	1	No
Orthodox	Married	Yes	Yes	University/	1	1	1	No
Orthodox	Married	Yes	Yes	7th	2	2	2	No
Orthodox	Married	Yes	Yes	6th	1	1	1	No
Orthodox	Married	Yes	Yes	3rd	6	5	5	Yes
Orthodox	Married	Yes	Yes	9th	1	1	1	No
Orthodox	Married	Yes	Yes	4th	2	2	2	No
Orthodox	Married	Yes	Yes	6th	1	1	1	No
Orthodox	Married	Yes	Yes	10th	1	1	1	No
Orthodox	Married	No			1	1	1	No
Orthodox	Married	Yes	Yes	6th	2	2	2	No
Orthodox	Married	Yes	Yes	4th	4	4	4	No
Orthodox	Married	Yes	Yes	11th	4	4	4	No
Orthodox	Married	Yes	Yes	5th	2	2	2	No
Orthodox	Married	Yes	Yes	2nd	1	1	1	No
Orthodox	Married	Yes	Yes	3rd	4	4	4	No
Orthodox	Married	No			1	1	1	No
Orthodox	Married	Yes	Yes	10th	1	1	1	No
Orthodox	Married	Yes	Yes	4th	5	5	5	No
Orthodox	Married	No			7	7	7	No
Orthodox	Married	No			2	2	2	No
Orthodox	Married	Yes	Yes	8th	1	1	1	No
Orthodox	Married	Yes	Yes	2nd	4	4	4	No
Orthodox	Married	Yes	Yes	7th	2	2	2	No
Orthodox	Married	No			2	2	2	No
Orthodox	Married	No			2	2	2	No
Orthodox	Married	Yes	Yes	6th	3	3	3	No
Orthodox	Married	Yes	Yes	6th	2	2	2	No
Orthodox	Married	Yes	Yes	5th	1	1	1	No
Orthodox	Married	No			5	5	5	No
Orthodox	Married	No			2	2	2	No
Orthodox	Married	Yes	Yes	10th	4	4	4	No
Protestant	Married	Yes	Yes	8th	3	3	3	No
Orthodox	Married	Yes	Yes	10th	3	2	2	Yes
Orthodox	Married	Yes	Yes	6th	1	1	1	No
Orthodox	Married	Yes	Yes	1st	3	3	3	No
Orthodox	Married	No			8	8	8	No
Orthodox	Married	Yes	Yes	10th	1	1	1	No
Orthodox	Married	Yes	Yes	9th	1	1	1	No
Protestant	Married	Yes	Yes	University/	2	2	2	No
Orthodox	Married	Yes	Yes	6th	3	3	3	No
Orthodox	Married	No			3	3	3	No

For peer review only

Orthodox	Married	No			1	1	1	No
Orthodox	Married	Yes	Yes	6th	1	1	1	No
Orthodox	Married	No			3	3	3	No
Orthodox	Married	No			2	2	2	No
Orthodox	Married	No			1	1	1	No
Orthodox	Married	No			1	1	1	No
Orthodox	Married	Yes	Yes	10th	1	1	1	No
Orthodox	Married	Yes	Yes	4th	5	5	5	No
Orthodox	Married	No			4	4	4	No
Orthodox	Married	No			4	3	3	Yes
Orthodox	Married	No			2	2	2	No
Orthodox	Married	No			6	4	4	Yes
Orthodox	Married	Yes	Yes	7th	2	2	2	No
Orthodox	Married	No			4	4	4	No
Orthodox	Separated				1	1	1	No
Orthodox	Married	Yes	Yes	8th	5	5	5	No
Orthodox	Married	No			3	3	3	Yes
Orthodox	Married	No			7	7	7	No
Orthodox	Married	Yes	Yes	10th	5	3	3	Yes
Orthodox	Married	Yes	Yes	8th	3	3	3	No
Orthodox	Married	Yes	Yes	4th	2	2	2	No
Orthodox	Married	No			2	2	2	No
Orthodox	Married	No			3	3	3	Yes
Orthodox	Married	Yes	Yes	10th	1	1	1	No
Orthodox	Married	Yes	Yes	9th	3	3	2	No
Protestant	Married	Yes	Yes	8th	2	2	2	No
Traditional	Married	Yes	Yes	6th	2	2	2	No
Orthodox	Married	No			2	2	2	No
Orthodox	Married	Yes	Yes	10th	2	2	2	No
Orthodox	Married	Yes	Yes	6th	3	3	3	No
Orthodox	Married	Yes	Yes	8th	8	8	8	No
Orthodox	Married	No			7	7	7	No
Orthodox	Married	No			6	6	6	No
Orthodox	Divorced				2	2	2	No
Orthodox	Married	Yes	Yes	8th	2	2	2	No
Orthodox	Married	Yes	Yes	5th	2	2	2	No
Orthodox	Married	Yes	Yes	9th	2	2	2	No
Orthodox	Married	Yes	Yes	10th	4	4	4	No
Orthodox	Married	No			2	2	2	No
Orthodox	Married	No			5	5	5	No
Orthodox	Married	Yes	Yes	6th	2	2	2	No
Orthodox	Married	No			3	3	3	No
Orthodox	Married	Yes	Yes	10th	2	2	2	No
Orthodox	Married	No			3	5	5	No
Orthodox	Married	No			3	3	3	No
Orthodox	Married	Yes	Yes	2nd	5	5	5	No
Orthodox	Separated				1	1	1	No
Orthodox	Married	No			4	4	4	No
Orthodox	Married	Yes	Yes	8th	1	1	1	No
Orthodox	Married	Yes	Yes	3rd	3	3	3	No
Orthodox	Married	Yes	Yes	6th	4	4	4	No
Orthodox	Married	No			1	1	1	No

For peer review only

Orthodox	Married	No			3	3	3	No
Orthodox	Married	Yes	Yes	5th	3	3	3	No
Orthodox	Married	No			2	2	2	No
Orthodox	Married	Yes	Yes	4th	1	1	1	No
Orthodox	Married	Yes	Yes	3rd	3	3	3	No
Orthodox	Married	No			8	8	8	No
Orthodox	Married	No			7	7	7	No
Orthodox	Married	Yes	Yes	5th	5	5	5	No
Orthodox	Married	Yes	Yes	4th	1	1	1	No
Orthodox	Married	Yes	Yes	10th	2	2	2	No
Orthodox	Married	No			2	2	2	No
Orthodox	Married	Yes	Yes	4th	3	3	3	No
Orthodox	Married	No			3	3	3	No
Orthodox	Married	Yes	Yes	11th	1	1	1	No
Orthodox	Married	Yes	Yes	5th	2	2	2	No
Orthodox	Married	Yes	Yes	10th	2	2	2	No
Orthodox	Married	No			6	6	6	No
Orthodox	Married	Yes	Yes	8th	3	3	3	No
Orthodox	Married	Yes	Yes	7th	1	1	1	No
Orthodox	Married	Yes	Yes	3rd	1	1	1	No
Orthodox	Married	No			4	4	4	No
Orthodox	Married	Yes	Yes	8th	4	4	4	No
Orthodox	Married	Yes	Yes	10th	1	1	1	No
Orthodox	Married	Yes	Yes	10th	2	2	2	No
Orthodox	Married	No			2	2	2	No
Orthodox	Married	No			4	4	4	No
Orthodox	Married	Yes	Yes	7th	2	2	2	No
Traditional	Married	Yes	Yes	4th	4	4	4	No
Orthodox	Married	Yes	Yes	9th	2	2	2	No
Orthodox	Married	No			4	4	4	No
Orthodox	Married	No			4	4	4	No
Orthodox	Married	Yes	Yes	8th	2	2	2	No
Orthodox	Married	Yes	Yes	4th	2	2	2	No
Orthodox	Married	No			1	1	1	No
Orthodox	Married	Yes	Yes	5th	3	3	3	No
Traditional	Married	Yes	Yes	6th	1	1	1	No
Orthodox	Married	No			1	1	1	No
Orthodox	Married	Yes	Yes	5th	2	2	3	No
Orthodox	Married	Yes	Yes	8th	1	1	1	No
Orthodox	Married	Yes	Yes	11th	1	1	1	No
Orthodox	Married	Yes	Yes	5th	3	3	3	No
Orthodox	Married	No			6	6	6	No
Orthodox	Married	Yes	Yes	10th	3	2	2	Yes
Orthodox	Married	Yes	Yes	6th	1	1	1	No
Orthodox	Married	No			3	3	3	Yes
Orthodox	Married	No			2	2	2	No
Orthodox	Married	No			5	5	5	No
Orthodox	Married	No			5	4	4	Yes
Orthodox	Married	Yes	Yes	4th	1	1	1	No
Orthodox	Married	Yes	Yes	9th	1	1	1	No
Orthodox	Married	No			6	6	6	No
Protestant	Married	No			2	2	2	No

For peer review only

Orthodox	Married	Yes	Yes	10th	1	1	1	No
Orthodox	Married	Yes	Yes	2nd	4	4	4	No
Orthodox	Married	No			2	2	2	No
Orthodox	Married	Yes	Yes	9th	2	2	2	No
Orthodox	Married	Yes	Yes	5th	3	3	3	No
Orthodox	Never married				1	1	1	No
Orthodox	Married	No			3	3	3	No
Orthodox	Married	No			2	2	2	No
Orthodox	Married	Yes	Yes	10th	2	1	1	Yes
Orthodox	Married	No			5	4	4	Yes
Orthodox	Married	Yes	Yes	2nd	3	3	3	Yes
Orthodox	Married	Yes	Yes	4th	1	1	1	No
Orthodox	Married	Yes	Yes	10th	6	6	6	No
Orthodox	Married	Yes	Yes	4th	3	3	3	No
Orthodox	Married	No			5	5	5	No
Orthodox	Married	No			1	1	1	No
Orthodox	Married	Yes	Yes	10th	1	1	1	No
Orthodox	Married	Yes	No	2nd	5	5	5	No
Orthodox	Married	No			4	4	4	No
Orthodox	Married	Yes	Yes	2nd	2	2	2	No
Orthodox	Married	No			2	2	2	No
Traditional	Married	Yes	Yes	8th	1	1	1	No
Orthodox	Married	Yes	Yes	4th	1	1	1	No
Orthodox	Married	Yes	Yes	4th	4	4	4	No
Orthodox	Married	Yes	Yes	4th	1	1	1	No
Orthodox	Married	No			6	5	5	Yes
Orthodox	Married	Yes	Yes	6th	2	2	2	No
Orthodox	Married	Yes	Yes	5th	2	2	2	No
Orthodox	Married	Yes	Yes	6th	1	1	1	No
Orthodox	Married	Yes	Yes	4th	2	2	2	No
Orthodox	Married	No			7	7	7	No
Orthodox	Married	Yes	Yes	10th	2	2	2	No
Orthodox	Married	Yes	Yes	9th	2	2	2	No
Orthodox	Married	Yes	Yes	University/	3	3	3	No
Orthodox	Married	No			1	1	1	No
Orthodox	Married	No			2	2	2	No
Orthodox	Married	No			1	1	1	No
Orthodox	Married	Yes	Yes	4th	3	3	3	No
Orthodox	Married	Yes	Yes	3rd	2	2	2	No
Orthodox	Married	No			2	2	2	No
Orthodox	Married	Yes	No	1st	1	1	1	No
Orthodox	Never married				1	1	1	No
Orthodox	Married	Yes	Yes	3rd	5	5	5	No
Orthodox	Married	No			4	4	5	No
Orthodox	Married	No			5	5	5	No
Orthodox	Married	Yes	Yes	University/	1	1	1	No
Orthodox	Married	Yes	Yes	3rd	2	2	2	No
Orthodox	Married	Yes	Yes	8th	1	1	1	No
Protestant	Married	Yes	Yes	University/	1	1	1	No
Orthodox	Married	No			5	5	5	No
Orthodox	Married	Yes	Yes	9th	1	1	1	No
Orthodox	Married	No			4	4	4	No

For peer review only

Orthodox	Married	No			6	4	4	Yes
Orthodox	Married	No			3	3	3	No
Orthodox	Married	Yes	Yes	2nd	1	1	1	No
Orthodox	Married	Yes	Yes	10th	2	2	2	No
Orthodox	Married	Yes	Yes	5th	4	4	4	No
Orthodox	Married	Yes	Yes	5th	2	2	2	No
Orthodox	Married	Yes	Yes	10th	1	1	1	No
Orthodox	Married	Yes	Yes	8th	8	8	8	Yes
Orthodox	Married	No			3	3	3	No
Orthodox	Married	Yes	Yes	University/	2	2	2	No
Orthodox	Married	Yes	Yes	6th	4	3	3	Yes
Orthodox	Married	Yes	Yes	2nd	2	2	2	No
Orthodox	Married	Yes	Yes	5th	3	3	3	No
Orthodox	Married	Yes	Yes	7th	1	1	1	No
Orthodox	Married	Yes	Yes	8th	2	2	2	No
Orthodox	Married	Yes	Yes	5th	1	1	1	No
Orthodox	Married	Yes	Yes	4th	1	1	1	No
Orthodox	Widowed				1	1	1	No
Traditional	Married	Yes	Yes	10th	1	1	1	No
Orthodox	Married	Yes	Yes	3rd	5	4	5	Yes
Orthodox	Married	Yes	Yes	9th	1	1	1	No
Orthodox	Married	Yes	Yes	6th	2	2	2	No
Orthodox	Married	No			7	7	7	No
Orthodox	Married	No			3	3	3	No
Orthodox	Married	No			3	3	3	No
Orthodox	Married	No			4	4	4	No
Orthodox	Married	Yes	Yes	3rd	6	6	6	No
Orthodox	Separated				1	1	1	No
Orthodox	Married	Yes	Yes	8th	2	1	1	No
Orthodox	Married	Yes	Yes	10th	2	2	2	No
Orthodox	Married	Yes	Yes	2nd	2	2	2	No
Orthodox	Married	Yes	Yes	4th	4	4	4	No
Orthodox	Married	Yes	Yes	7th	1	1	1	No
Orthodox	Married	Yes	Yes	10th	1	1	1	No
Orthodox	Married	Yes	Yes	8th	2	2	2	No
Orthodox	Married	No			3	3	3	No
Orthodox	Married	No			4	4	4	No
Orthodox	Married	No			4	4	4	No
Orthodox	Married	Yes	No	1st	8	8	6	No
Orthodox	Married	Yes	Yes	8th	1	1	1	No
Orthodox	Married	Yes	Yes	10th	1	1	1	No
Orthodox	Married	No			3	3	3	No
Orthodox	Married	Yes	No	1st	6	6	5	No
Orthodox	Married	No			1	1	1	No
Orthodox	Married	Yes	Yes	10th	2	2	2	No
Orthodox	Married	Yes	Yes	5th	1	1	1	No
Orthodox	Married	Yes	Yes	2nd	3	2	2	Yes
Orthodox	Married	Yes	Yes	9th	1	1	1	No
Orthodox	Married	No			1	1	1	No
Orthodox	Married	No			1	1	1	No
Orthodox	Married	No			2	2	2	No
Orthodox	Married	Yes	Yes	3rd	4	4	4	No

For peer review only

Orthodox	Married	Yes	Yes	8th	4	4	4	No
Orthodox	Married	No			7	7	7	No
Orthodox	Married	No			10	10	10	No
Orthodox	Married	Yes	Yes	7th	1	1	1	No
Orthodox	Married	Yes	Yes	7th	3	3	3	No
Orthodox	Married	Yes	Yes	3rd	4	4	4	No
Orthodox	Married	No			3	3	3	Yes
Orthodox	Married	Yes	Yes	5th	2	2	2	No
Orthodox	Married	Yes	Yes	5th	2	2	2	No
Orthodox	Married	Yes	Yes	4th	4	4	4	No
Orthodox	Married	No			2	2	2	No
Orthodox	Married	No			2	2	2	No
Orthodox	Married	No			2	2	2	No
Orthodox	Married	Yes	Yes	5th	2	2	2	No
Orthodox	Married	Yes	Yes	5th	3	3	3	No
Orthodox	Married	Yes	Yes	4th	11	10	10	Yes
Orthodox	Married	Yes	Yes	7th	1	1	1	No
Orthodox	Married	No			2	2	2	No
Orthodox	Married	Yes	Yes	10th	2	2	2	No
Orthodox	Married	Yes	Yes	10th	1	1	1	No
Orthodox	Married	Yes	Yes	7th	1	1	1	No
Orthodox	Married	Yes	Yes	7th	5	4	4	Yes
Orthodox	Married	No			6	5	5	Yes
Orthodox	Married	No			8	8	8	No
Orthodox	Married	No			2	2	2	No
Orthodox	Married	Yes	Yes	10th	1	1	1	No
Orthodox	Divorced				2	2	2	Yes
Orthodox	Married	No			5	5	5	No
Orthodox	Married	Yes	Yes	5th	2	2	2	No
Orthodox	Married	Yes	No	5th	5	5	5	No
Orthodox	Married	No			4	4	4	No
Orthodox	Married	No			3	3	3	No
Orthodox	Married	No			5	4	4	Yes
Orthodox	Married	No			3	3	4	No
Orthodox	Married	No			4	4	4	No
Orthodox	Married	No			4	4	4	No
Orthodox	Married	Yes	Yes	4th	3	3	3	No
Orthodox	Married	Yes	Yes	3rd	7	7	7	No
Orthodox	Married	No			4	4	4	No
Orthodox	Married	No			4	3	3	No
Orthodox	Married	No			5	5	5	No
Orthodox	Married	Yes	Yes	6th	1	1	1	No
Orthodox	Married	Yes	Yes	5th	6	6	6	No
Orthodox	Married	Yes	Yes	4th	2	2	2	No
Orthodox	Married	Yes	Yes	7th	4	4	4	No
Orthodox	Married	Yes	Yes	5th	2	2	2	No
Orthodox	Married	Yes	Yes	6th	6	6	6	No
Orthodox	Married	Yes	Yes	7th	1	1	1	No
Orthodox	Married	No			3	3	3	No
Orthodox	Married	No			3	3	3	No
Orthodox	Married	Yes	Yes	3rd	2	2	2	No
Orthodox	Married	No			3	3	3	No

For peer review only

Orthodox	Married	No			7	5	4	No
Orthodox	Married	No			3	3	3	No
Orthodox	Married	Yes	No	5th	7	7	7	No
Orthodox	Married	Yes	Yes	7th	3	3	3	No
Orthodox	Married	No			6	6	6	No
Orthodox	Married	No			3	3	3	No
Orthodox	Married	No			5	5	5	No
Orthodox	Married	No			7	7	7	No
Orthodox	Married	Yes	Yes	3rd	9	9	9	No
Orthodox	Married	Yes	No	3rd	6	6	6	No
Orthodox	Married	No			3	3	3	No
Orthodox	Married	Yes	Yes	5th	3	3	3	No
Orthodox	Married	No			8	6	6	Yes
Orthodox	Married	No			4	4	4	No
Orthodox	Married	No			5	5	5	No
Orthodox	Married	Yes	Yes	4th	8	5	5	Yes
Orthodox	Married	Yes	Yes	9th	2	2	2	No
Orthodox	Married	Yes	Yes	3rd	7	7	7	No
Orthodox	Married	Yes	Yes	3rd	6	6	6	No
Orthodox	Married	Yes	Yes	3rd	7	7	7	No
Orthodox	Married	No			4	4	4	No
Orthodox	Married	Yes	Yes	9th	1	1	1	No
Orthodox	Married	Yes	Yes	7th	2	2	2	No
Orthodox	Married	No			7	7	7	No
Orthodox	Married	No			1	1	1	No
Orthodox	Married	No			5	3	3	Yes
Orthodox	Married	No			7	7	8	No
Orthodox	Married	Yes	Yes	5th	5	5	5	Yes
Orthodox	Married	Yes	Yes	5th	8	8	8	No
Orthodox	Married	No			2	2	2	No
Orthodox	Married	Yes	Yes	7th	3	3	3	No
Orthodox	Married	No			4	4	4	No
Orthodox	Married	No			6	5	5	Yes
Orthodox	Married	No			2	2	2	Yes
Orthodox	Married	Yes	Yes	4th	4	4	4	No
Orthodox	Married	No			3	3	3	No
Orthodox	Married	Yes	Yes	10th	2	2	2	No
Orthodox	Divorced				2	2	2	No
Orthodox	Married	Yes	Yes	10th	5	5	5	No
Orthodox	Married	Yes	Yes	8th	10	10	10	Yes
Orthodox	Married	Yes	No	6th	3	3	3	No
Orthodox	Married	No			5	5	5	No
Orthodox	Married	No			3	3	3	No
Orthodox	Married	Yes	Yes	4th	4	3	3	Yes
Orthodox	Married	Yes	Yes	8th	1	1	1	No
Orthodox	Divorced				3	3	3	No
Orthodox	Married	Yes	Yes	5th	3	2	2	Yes
Orthodox	Married	Yes	Yes	5th	6	6	6	No
Orthodox	Married	Yes	Yes	7th	2	2	2	No
Orthodox	Married	No			5	5	5	No
Orthodox	Married	No			6	5	5	Yes
Orthodox	Married	No			6	6	6	No

For peer review only

Orthodox	Married	Yes	Yes	9th	3	3	4	No
Orthodox	Married	No			4	4	4	No
Orthodox	Married	No			6	6	6	No
Orthodox	Married	No			3	3	3	No
Orthodox	Married	No			4	4	4	No
Orthodox	Married	No			3	3	3	No
Orthodox	Married	No			4	4	4	No
Orthodox	Married	Yes	Yes	3rd	3	3	3	No
Orthodox	Married	No			8	8	8	No
Orthodox	Married	Yes	Yes	5th	2	2	2	No
Orthodox	Married	No			1	1	1	No
Orthodox	Married	Yes	Yes	3rd	5	5	5	No
Orthodox	Married	No			4	4	4	No
Orthodox	Married	Yes	Yes	5th	3	3	3	No
Orthodox	Married	No			1	1	1	No
Orthodox	Married	Yes	Yes	8th	2	2	2	No
Orthodox	Married	No			6	6	6	No
Orthodox	Married	Yes	Yes	8th	4	4	4	No
Orthodox	Married	No			2	2	2	No
Orthodox	Married	No			6	6	6	No
Orthodox	Married	Yes	Yes	6th	4	3	3	Yes
Orthodox	Married	Yes	Yes	7th	1	1	1	No
Orthodox	Married	Yes	Yes	5th	5	5	5	No
Orthodox	Married	Yes	Yes	6th	1	1	1	No
Orthodox	Married	Yes	Yes	University/	1	1	2	No
Orthodox	Married	No			9	5	9	Yes
Orthodox	Married	No			4	4	4	No
Orthodox	Married	No			7	7	7	No
Orthodox	Married	No			8	8	8	No
Orthodox	Married	No			5	5	5	No
Orthodox	Married	No			3	3	3	No
Orthodox	Married	No			7	7	7	No
Orthodox	Married	Yes	Yes	8th	1	1	1	No
Orthodox	Married	No			3	3	3	No
Orthodox	Married	Yes	Yes	6th	4	3	3	Yes
Orthodox	Married	Yes	Yes	7th	9	9	9	No
Orthodox	Married	No			2	2	2	No
Orthodox	Married	Yes	Yes	8th	1	1	1	No
Orthodox	Married	Yes	Yes	3rd	3	3	3	No
Orthodox	Married	No			7	7	7	No
Orthodox	Married	No			4	4	4	No
Orthodox	Married	Yes	Yes	5th	4	4	4	No
Orthodox	Married	Yes	No	5th	2	2	2	No
Orthodox	Married	Yes	No	5th	4	4	4	No
Orthodox	Married	No			5	5	5	No
Orthodox	Married	No			6	5	5	Yes
Orthodox	Married	No			3	3	3	No
Orthodox	Married	Yes	Yes	5th	4	4	4	No
Orthodox	Married	Yes	Yes	2nd	4	4	4	No
Orthodox	Married	No			5	5	5	No
Orthodox	Married	No			9	9	9	No
Orthodox	Married	No			3	3	3	No

For peer review only

Orthodox	Never married				4	3	3	Yes
Orthodox	Married	No			3	3	3	No
Orthodox	Married	No			6	5	5	No
Orthodox	Married	No			5	4	4	Yes
Orthodox	Married	Yes	Yes	4th	5	5	5	No
Orthodox	Married	Yes	Yes	5th	2	2	2	No
Orthodox	Married	No			8	8	8	No
Orthodox	Married	No			6	6	6	No
Orthodox	Married	Yes	Yes	7th	3	3	3	Yes
Orthodox	Married	Yes	Yes	6th	2	2	2	No
Orthodox	Married	No			3	3	3	No
Orthodox	Married	Yes	Yes	8th	1	1	1	No
Orthodox	Married	No			10	9	9	Yes
Orthodox	Married	No			1	1	1	No
Orthodox	Married	Yes	Yes	5th	5	0	5	No
Orthodox	Married	No			7	7	7	No
Orthodox	Married	No			5	5	5	No
Orthodox	Married	No			4	4	4	No
Orthodox	Married	Yes	No	1st	1	1	1	No
Orthodox	Married	No			3	1	1	Yes
Orthodox	Married	No			3	4	4	No
Orthodox	Married	Yes	Yes	5th	3	3	3	No
Orthodox	Married	No			3	3	3	No
Orthodox	Married	No			6	6	6	No
Orthodox	Married	No			4	4	4	No
Orthodox	Married	No			4	4	4	No
Orthodox	Married	Yes	Yes	4th	7	7	7	No
Orthodox	Married	Yes	Yes	5th	4	3	3	Yes
Orthodox	Married	Yes	Yes	8th	6	6	6	Yes
Orthodox	Married	No			3	3	3	No
Orthodox	Married	No			6	6	6	No
Orthodox	Married	No			3	3	3	No
Orthodox	Married	Yes	Yes	University/	1	1	1	No
Orthodox	Married	Yes	Yes	10th	2	2	2	No
Orthodox	Married	Yes	Yes	3rd	3	3	3	No
Orthodox	Married	Yes	Yes	3rd	7	5	5	Yes
Orthodox	Married	No			7	6	6	Yes
Orthodox	Married	Yes	Yes	8th	4	4	4	No
Orthodox	Married	No			4	4	4	No
Orthodox	Married	No			4	3	3	Yes
Orthodox	Married	Yes	Yes	10th	4	4	4	No
Orthodox	Married	Yes	Yes	8th	1	1	1	No
Orthodox	Married	Yes	Yes	8th	1	1	1	No
Protestant	Married	Yes	Yes	2nd	5	5	5	No
Orthodox	Married	No			6	6	6	No
Orthodox	Married	No			2	2	2	No
Traditional	Married	Yes	Yes	10th	1	1	1	No
Orthodox	Married	Yes	Yes	6th	2	2	2	No
Orthodox	Married	Yes	Yes	9th	1	1	1	No
Orthodox	Married	No			3	3	3	No
Orthodox	Married	Yes	Yes	10th	1	1	1	No
Orthodox	Married	No			5	5	5	No

For peer review only

Orthodox	Married	Yes	No	1st	3	3	3	No
Orthodox	Married	No			4	4	4	No
Orthodox	Married	Yes	Yes	12th	4	4	4	No
Orthodox	Married	Yes	No	1st	4	4	4	No
Orthodox	Married	No			6	6	6	No
Orthodox	Married	No			6	6	6	No
Orthodox	Married	No			2	2	2	No
Orthodox	Married	No			1	1	1	No
Protestant	Married	Yes	Yes	7th	2	2	2	No
Orthodox	Married	Yes	Yes	7th	1	1	1	No
Muslim	Married	Yes	Yes	University/	2	2	2	Yes
Orthodox	Married	Yes	Yes	University/	2	2	2	No
Orthodox	Married	Yes	Yes	3rd	2	2	2	No
Orthodox	Married	Yes	Yes	3rd	2	2	2	No
Orthodox	Married	Yes	No	3rd	4	4	4	No
Orthodox	Married	Yes	Yes	10th	1	1	1	No
Orthodox	Married	No			2	2	2	No
Protestant	Married	Yes	Yes	5th	1	1	1	No
Orthodox	Married	Yes	Yes	8th	4	4	4	No
Orthodox	Married	No			2	2	2	No
Orthodox	Married	Yes	Yes	6th	6	6	6	No
Orthodox	Married	Yes	Yes	8th	2	1	1	Yes
Orthodox	Married	Yes	Yes	3rd	6	6	6	No
Orthodox	Married	No			2	2	2	No
Orthodox	Married	Yes	Yes	University/	1	1	1	No
Orthodox	Married	Yes	Yes	10th	4	4	4	Yes
Orthodox	Married	No			5	5	5	No
Protestant	Married	Yes	Yes	5th	3	3	3	No
Orthodox	Married	No			8	8	8	No
Orthodox	Married	No			4	4	4	No
Orthodox	Married	Yes	Yes	7th	2	2	2	No
Orthodox	Married	No			1	1	1	No
Orthodox	Separated				1	1	1	No
Protestant	Married	Yes	Yes	7th	1	1	1	No
Orthodox	Married	No			5	5	5	No
Protestant	Married	Yes	Yes	7th	3	3	3	No
Orthodox	Married	Yes	Yes	4th	7	7	7	Yes
Orthodox	Married	Yes	Yes	8th	1	1	1	No
Orthodox	Married	Yes	Yes	4th	2	2	2	No
Protestant	Married	No			2	2	2	No
Orthodox	Married	Yes	Yes	8th	1	1	1	No
Orthodox	Married	No			5	5	5	No
Orthodox	Married	No			6	6	6	No
Orthodox	Married	Yes	Yes	9th	5	4	4	Yes
Orthodox	Married	Yes	No	1st	5	5	5	No
Orthodox	Married	Yes	Yes	4th	1	1	1	No
Orthodox	Married	Yes	Yes	5th	2	2	2	No
Orthodox	Married	Yes	Yes	7th	1	1	1	No
Orthodox	Married	No			1	1	1	No
Protestant	Married	No			2	2	2	No
Orthodox	Married	No			1	1	1	No
Orthodox	Married	Yes	Yes	4th	5	5	5	No

For peer review only

Orthodox	Married	No			2	2	2	No
Orthodox	Married	No			1	1	1	No
Orthodox	Married	No			3	3	3	Yes
Orthodox	Divorced				3	3	3	No
Orthodox	Married	Yes	Yes	University/	3	3	3	No
Protestant	Never married				1	1	1	No
Protestant	Married	No			2	2	3	No
Orthodox	Married	Yes	Yes	9th	4	4	4	No
Orthodox	Married	No			5	5	5	No
Orthodox	Married	Yes	Yes	12th	4	4	4	No
Orthodox	Married	No			6	6	6	No
Orthodox	Married	Yes	Yes	5th	2	2	2	No
Orthodox	Married	No			7	7	7	No
Orthodox	Married	No			6	6	6	No
Orthodox	Married	No			3	3	3	No
Orthodox	Married	Yes	Yes	10th	2	1	1	Yes
Orthodox	Married	Yes	Yes	4th	1	1	1	No
Orthodox	Married	No			7	5	5	Yes
Orthodox	Married	No			1	1	1	No
Orthodox	Married	Yes	Yes	4th	2	1	1	Yes
Orthodox	Married	Yes	Yes	10th	1	1	1	No
Orthodox	Married	Yes	Yes	10th	1	1	1	No
Protestant	Married	Yes	Yes	6th	3	3	3	No
Orthodox	Married	Yes	Yes	8th	4	4	4	No
Orthodox	Married	Yes	No	6th	7	7	7	No
Orthodox	Married	No			6	6	6	No
Orthodox	Married	Yes	Yes	10th	2	2	2	No
Protestant	Married	No			1	1	1	No
Orthodox	Married	No			2	2	2	No
Orthodox	Married	Yes	Yes	5th	6	6	6	No
Orthodox	Married	No			2	2	2	No
Orthodox	Married	No			1	1	1	No
Orthodox	Married	No			4	4	4	No
Orthodox	Married	Yes	Yes	4th	1	1	1	No
Orthodox	Separated				1	1	1	No
Orthodox	Married	No			3	3	3	No
Orthodox	Married	No			1	1	1	No
Orthodox	Married	Yes	Yes	3rd	1	1	1	No
Orthodox	Married	No			4	4	4	No
Protestant	Married	No			3	3	3	No
Orthodox	Married	No			1	1	1	No
Protestant	Married	No			1	1	1	No
Protestant	Married	Yes	Yes	2nd	6	6	6	No
Orthodox	Married	Yes	Yes	10th	1	1	1	No
Orthodox	Married	No			7	2	5	Yes
Orthodox	Married	No			1	1	1	No
Orthodox	Married	No			6	0	6	No
Orthodox	Married	Yes	Yes	7th	1	1	1	No
Orthodox	Married	Yes	Yes	6th	2	2	3	No
Protestant	Married	Yes	Yes	5th	1	1	1	No
Orthodox	Married	No			5	5	5	No
Orthodox	Married	Yes	Yes	8th	1	1	1	No

For peer review only

Orthodox	Married	No			2	2	2	No
Protestant	Married	Yes	Yes	University/	1	1	1	No
Orthodox	Married	No			5	5	5	No
Protestant	Married	Yes	Yes	6th	2	2	2	No
Orthodox	Married	Yes	Yes	5th	2	2	2	No
Orthodox	Married	Yes	Yes	9th	3	2	2	Yes
Orthodox	Married	No			2	2	2	No
Orthodox	Married	Yes	Yes	3rd	3	3	3	No
Orthodox	Married	Yes	Yes	6th	3	3	3	No
Orthodox	Married	Yes	Yes	8th	3	3	3	No
Orthodox	Married	Yes	Yes	10th	2	2	2	No
Orthodox	Married	Yes	Yes	8th	1	1	1	Yes
Protestant	Married	No			2	2	2	No
Protestant	Married	No			7	7	7	Yes
Orthodox	Married	Yes	Yes	7th	1	1	1	No
Orthodox	Married	Yes	Yes	10th	1	1	1	No
Orthodox	Married	Yes	Yes	10th	1	1	1	No
Protestant	Married	Yes	Yes	7th	2	2	2	No
Orthodox	Married	Yes	Yes	9th	1	0	1	No
Orthodox	Married	Yes	Yes	8th	1	1	1	No
Orthodox	Married	Yes	Yes	3rd	1	1	1	No
Orthodox	Married	No			2	2	2	No
Orthodox	Married	Yes	Yes	University/	2	2	2	No
Orthodox	Married	Yes	Yes	6th	3	3	3	No
Orthodox	Married	No			7	7	7	Yes
Protestant	Married	Yes	Yes	4th	2	2	2	No
Orthodox	Married	No			1	1	1	No
Orthodox	Married	Yes	Yes	10th	2	2	2	No
Orthodox	Married	Yes	Yes	8th	5	5	5	No
Orthodox	Married	Yes	Yes	Technical/v	2	2	2	No
Orthodox	Married	No			4	4	4	No
Orthodox	Widowed				1	1	1	No
Orthodox	Married	No			3	3	3	No
Orthodox	Married	No			6	6	6	No
Orthodox	Married	No			6	6	6	No
Orthodox	Married	Yes	Yes	6th	1	1	1	No
Orthodox	Married	No			8	6	6	Yes
Orthodox	Married	No			2	2	2	No
Orthodox	Married	No			11	11	11	No
Orthodox	Married	Yes	Yes	5th	6	0	6	No
Orthodox	Married	Yes	Yes	6th	3	3	3	No
Protestant	Married	No			5	5	5	No
Orthodox	Married	No			5	5	5	No
Orthodox	Married	Yes	Yes	6th	4	4	3	No
Orthodox	Married	No			3	3	3	No
Orthodox	Married	Yes	Yes	7th	7	7	7	No
Orthodox	Married	Yes	Yes	3rd	2	2	2	No
Orthodox	Married	Yes	Yes	4th	2	2	2	No
Orthodox	Married	No			8	8	8	Yes
Orthodox	Married	Yes	Yes	5th	2	2	2	No
Orthodox	Separated				1	1	1	No
Orthodox	Married	Yes	Yes	5th	2	2	2	No

For peer review only

Orthodox	Married	No			2	2	2	No
Orthodox	Married	No			3	3	3	No
Orthodox	Married	Yes	Yes	7th	3	3	3	No
Protestant	Married	No			8	8	7	No
Orthodox	Married	Yes	Yes	6th	2	2	2	No
Orthodox	Married	No			3	3	3	No
Orthodox	Married	Yes	Yes	4th	2	2	2	No
Orthodox	Married	Yes	Yes	7th	6	4	4	Yes
Orthodox	Married	Yes	Yes	3rd	6	4	4	Yes
Orthodox	Married	No			1	1	1	No
Orthodox	Married	Yes	Yes	5th	2	2	2	No
Orthodox	Separated				1	1	1	No
Orthodox	Married	Yes	Yes	8th	1	1	1	No
Orthodox	Married	No			1	1	1	No
Orthodox	Married	Yes	Yes	8th	4	4	4	No
Orthodox	Married	No			2	2	2	No
Orthodox	Married	Yes	Yes	8th	5	5	5	No
Orthodox	Married	No			7	7	7	No
Orthodox	Married	No			1	1	1	No
Orthodox	Married	Yes	Yes	3rd	2	2	2	No
Orthodox	Married	Yes	No	1st	4	4	4	No
Orthodox	Married	Yes	Yes	6th	2	2	2	No
Orthodox	Married	No			7	7	7	No
Orthodox	Married	Yes	Yes	6th	4	4	4	No
Orthodox	Married	No			4	4	4	Yes
Orthodox	Married	Yes	Yes	9th	2	2	2	No
Orthodox	Married	Yes	Yes	10th	2	2	2	No
Orthodox	Married	No			7	6	6	Yes
Orthodox	Married	Yes	Yes	10th	3	3	3	No
Orthodox	Married	Yes	No	2nd	2	3	2	No
Orthodox	Married	No			3	3	3	No
Orthodox	Married	Yes	Yes	6th	2	2	2	No
Orthodox	Married	Yes	Yes	6th	2	2	2	No
Orthodox	Married	Yes	Yes	6th	5	5	5	No
Orthodox	Never married				3	3	3	Yes
Orthodox	Married	Yes	Yes	6th	2	2	2	No
Orthodox	Married	Yes	Yes	4th	3	3	3	No
Orthodox	Married	Yes	Yes	10th	2	1	1	No
Orthodox	Married	No			1	1	1	No
Orthodox	Married	No			4	4	4	No
Orthodox	Married	Yes	Yes	4th	8	8	8	No
Orthodox	Married	Yes	Yes	4th	6	6	6	Yes
Orthodox	Married	No			3	3	3	No
Orthodox	Married	No			6	6	6	No
Orthodox	Married	Yes	Yes	4th	6	4	4	Yes
Orthodox	Married	Yes	Yes	University/	1	1	1	No
Orthodox	Married	No			6	6	6	No
Orthodox	Married	Yes	Yes	3rd	3	3	3	No
Orthodox	Married	No			2	2	2	No
Orthodox	Married	Yes	Yes	8th	2	2	2	No
Orthodox	Married	Yes	Yes	5th	2	2	2	No
Orthodox	Married	Yes	Yes	7th	1	1	1	No

For peer review only

Orthodox	Married	Yes	Yes	4th	6	6	6	No
Orthodox	Married	Yes	Yes	6th	1	1	1	No
Orthodox	Married	No			5	4	4	Yes
Orthodox	Married	Yes	Yes	4th	4	4	4	No
Orthodox	Married	Yes	Yes	6th	3	3	3	No
Orthodox	Married	No			1	1	1	No
Orthodox	Married	Yes	Yes	5th	3	3	3	No
Orthodox	Married	No			2	2	2	No
Orthodox	Married	No			6	6	6	No
Orthodox	Married	Yes	Yes	10th	1	1	1	No
Orthodox	Married	Yes	Yes	10th	1	1	1	No
Orthodox	Married	Yes	No	2nd	3	3	3	No
Orthodox	Married	Yes	No	2nd	1	1	1	No
Orthodox	Married	Yes	Yes	3rd	7	7	7	No
Orthodox	Married	No			1	1	1	No
Orthodox	Married	No			3	3	3	No
Orthodox	Married	No			2	2	2	No
Orthodox	Married	No			8	8	7	No
Orthodox	Married	Yes	Yes	University/	1	1	1	No
Orthodox	Married	Yes	Yes	6th	12	7	5	Yes
Orthodox	Married	No			4	4	4	No
Orthodox	Married	Yes	Yes	5th	1	1	1	No
Orthodox	Divorced				1	1	1	No
Orthodox	Married	Yes	Yes	1st	5	5	5	No
Orthodox	Married	Yes	Yes	4th	1	1	1	No
Orthodox	Married	No			2	2	2	No
Orthodox	Married	Yes	Yes	9th	1	1	1	No
Orthodox	Married	No			1	1	1	No
Orthodox	Married	No			4	4	4	No
Orthodox	Married	No			4	4	4	No
Orthodox	Married	Yes	Yes	University/	1	1	1	No
Orthodox	Married	Yes	Yes	6th	2	1	1	Yes
Orthodox	Married	Yes	Yes	3rd	1	1	1	No
Orthodox	Married	Yes	Yes	7th	3	3	3	No
Orthodox	Married	No			8	7	7	Yes
Orthodox	Married	No			7	7	5	Yes
Orthodox	Married	Yes	Yes	3rd	4	4	4	No
Orthodox	Married	Yes	Yes	3rd	1	1	1	No
Orthodox	Married	Yes	Yes	12th	1	1	1	No
Orthodox	Married	Yes	Yes	6th	5	5	5	No
Orthodox	Married	Yes	Yes	2nd	7	7	7	No
Orthodox	Married	No			3	3	3	No
Orthodox	Married	Yes	Yes	7th	1	1	1	No
Orthodox	Married	Yes	Yes	2nd	4	4	4	No
Orthodox	Married	Yes	Yes	7th	2	2	2	No
Orthodox	Married	Yes	Yes	8th	2	1	1	Yes
Orthodox	Married	Yes	Yes	4th	2	2	2	No
Orthodox	Married	Yes	Yes	6th	2	3	3	No
Orthodox	Married	Yes	Yes	University/	1	1	1	No
Orthodox	Married	Yes	No	4th	5	5	5	Yes
Orthodox	Married	Yes	Yes	10th	1	1	1	No
Orthodox	Married	No			5	5	5	No

For peer review only

Orthodox	Married	No			4	4	4	No
Orthodox	Married	No			5	5	5	No
Orthodox	Married	Yes	Yes	6th	5	5	5	No
Orthodox	Married	Yes	Yes	6th	4	4	4	No
Orthodox	Married	Yes	Yes	10th	1	1	1	No
Orthodox	Married	Yes	Yes	3rd	2	2	2	No
Orthodox	Married	Yes	No	2nd	7	7	7	No
Orthodox	Married	Yes	Yes	5th	4	4	4	No
Orthodox	Divorced				2	2	2	No
Orthodox	Married	Yes	Yes	6th	1	1	1	No
Orthodox	Married	Yes	Yes	3rd	6	6	6	No
Orthodox	Divorced				3	3	3	No
Orthodox	Married	Yes	Yes	4th	1	1	1	No
Orthodox	Married	Yes	Yes	4th	1	1	1	No
Orthodox	Married	Yes	Yes	7th	2	2	2	No
Orthodox	Married	Yes	Yes	4th	1	1	1	No
Orthodox	Married	Yes	Yes	6th	1	1	1	No
Orthodox	Married	No			1	1	1	No
Orthodox	Married	Yes	Yes	6th	4	4	4	No
Orthodox	Married	No			2	2	2	No
Orthodox	Married	Yes	Yes	1st	5	5	5	No
Orthodox	Married	No			5	6	6	No
Orthodox	Married	No			2	2	2	No
Orthodox	Married	Yes	Yes	10th	1	1	1	No
Orthodox	Married	Yes	Yes	6th	2	2	2	No
Orthodox	Married	Yes	Yes	4th	7	7	6	Yes
Orthodox	Married	No			4	4	4	No
Orthodox	Married	Yes	Yes	12th	1	1	1	No
Orthodox	Married	No			3	3	3	No
Orthodox	Married	No			7	7	7	Yes
Orthodox	Married	No			3	2	2	Yes
Orthodox	Divorced				3	3	3	No
Orthodox	Married	No			4	4	4	No
Orthodox	Married	Yes	Yes	10th	2	2	2	No
Orthodox	Divorced				1	1	1	No
Orthodox	Married	No			7	7	7	No
Orthodox	Married	No			6	6	6	No
Orthodox	Married	No			4	4	4	No
Orthodox	Married	Yes	Yes	6th	4	4	4	No
Orthodox	Married	No			7	7	7	No
Orthodox	Married	No			4	4	4	No
Orthodox	Married	No			6	6	6	No
Orthodox	Married	Yes	Yes	11th	1	1	1	No
Orthodox	Married	Yes	Yes	4th	3	2	2	Yes
Orthodox	Married	No			6	6	6	No
Orthodox	Married	No			4	4	4	No
Orthodox	Married	Yes	Yes	5th	7	7	7	No
Orthodox	Married	Yes	Yes	6th	8	7	7	Yes
Orthodox	Married	Yes	Yes	8th	3	3	3	Yes
Orthodox	Married	No			1	1	1	No
Orthodox	Married	No			10	6	6	Yes
Orthodox	Married	Yes	Yes	6th	1	1	1	No

For peer review only

Orthodox	Married	No			6	6	6	No
Orthodox	Married	Yes	Yes	6th	2	2	2	No
Orthodox	Married	Yes	Yes	4th	11	9	9	Yes
Orthodox	Married	No			7	6	6	Yes
Orthodox	Married	No			1	1	2	No
Orthodox	Married	Yes	Yes	5th	5	5	4	Yes
Orthodox	Divorced				1	1	1	No
Orthodox	Married	Yes	Yes	3rd	10	10	10	No
Orthodox	Married	Yes	No	2nd	3	3	3	No
Orthodox	Married	Yes	No	1st	1	1	1	No
Orthodox	Married	No			5	5	5	No
Orthodox	Married	No			2	1	1	Yes
Orthodox	Married	Yes	Yes	4th	4	3	3	Yes
Orthodox	Married	Yes	Yes	8th	1	1	1	No
Orthodox	Married	Yes	Yes	4th	5	4	4	Yes
Orthodox	Married	Yes	Yes	10th	1	1	1	No
Orthodox	Married	No			8	7	5	Yes
Orthodox	Married	Yes	Yes	3rd	9	9	9	No
Orthodox	Married	No			4	4	4	No
Orthodox	Married	Yes	Yes	10th	1	1	1	No
Orthodox	Married	Yes	No	1st	10	9	9	No
Orthodox	Married	Yes	Yes	3rd	5	3	3	Yes
Orthodox	Married	Yes	Yes	10th	2	2	2	Yes
Orthodox	Married	Yes	Yes	4th	3	3	3	No
Orthodox	Married	No			5	5	5	No
Orthodox	Married	No			6	6	6	No
Orthodox	Married	Yes	Yes	5th	5	5	5	No
Orthodox	Married	Yes	Yes	6th	6	6	6	No
Orthodox	Married	No			2	2	2	No
Orthodox	Married	No			6	6	6	No
Orthodox	Married	Yes	Yes	University/	1	1	1	No
Orthodox	Married	Yes	Yes	4th	1	1	1	No
Orthodox	Married	Yes	Yes	7th	2	2	2	No
Orthodox	Married	Yes	Yes	2nd	5	5	5	No
Orthodox	Married	Yes	Yes	1st	3	3	3	No
Orthodox	Married	No			4	4	4	No
Orthodox	Married	Yes	Yes	9th	2	2	2	No
Orthodox	Married	Yes	Yes	5th	3	3	3	No
Orthodox	Married	No			1	1	1	No
Orthodox	Married	Yes	Yes	3rd	1	1	1	No
Orthodox	Married	Yes	No	3rd	7	7	7	No
Orthodox	Married	Yes	Yes	2nd	3	3	3	No
Orthodox	Married	No			10	7	8	Yes
Orthodox	Married	Yes	Yes	3rd	1	1	1	No
Orthodox	Married	Yes	Yes	5th	1	1	1	No
Orthodox	Married	No			2	2	2	No
Orthodox	Married	Yes	Yes	5th	2	2	2	No
Orthodox	Married	No			8	7	7	Yes
Orthodox	Married	No			3	2	2	Yes
Orthodox	Married	Yes	Yes	7th	3	2	2	Yes
Orthodox	Married	Yes	Yes	3rd	5	5	5	No
Orthodox	Married	Yes	Yes	6th	2	2	2	No

For peer review only

Orthodox	Married	No			7	7	7	No
Orthodox	Married	Yes	Yes	University/	1	1	1	No
Orthodox	Married	No			1	1	1	No
Orthodox	Married	Yes	Yes	10th	2	2	2	No
Orthodox	Married	No			3	3	3	No
Orthodox	Married	Yes	Yes	10th	1	1	1	No
Orthodox	Married	Yes	Yes	8th	1	1	1	No
Orthodox	Married	No			1	1	1	No
Orthodox	Divorced				2	2	2	No
Orthodox	Married	Yes	Yes	7th	3	3	3	No
Orthodox	Married	Yes	Yes	5th	3	3	3	No
Orthodox	Married	No			1	1	1	No
Orthodox	Married	Yes	Yes	10th	1	1	1	No
Orthodox	Married	Yes	Yes	6th	2	2	2	No
Orthodox	Married	Yes	Yes	6th	5	5	5	No
Orthodox	Married	Yes	Yes	6th	3	1	3	Yes
Orthodox	Married	No			4	3	3	Yes
Orthodox	Married	No			4	3	3	Yes
Orthodox	Married	Yes	Yes	7th	3	3	3	No
Orthodox	Married	Yes	Yes	8th	2	2	2	No
Orthodox	Married	No			4	3	3	Yes
Orthodox	Married	Yes	Yes	5th	1	1	1	No
Orthodox	Married	Yes	Yes	9th	2	2	2	No
Orthodox	Married	Yes	Yes	4th	7	7	4	No
Orthodox	Married	No			1	1	1	No
Orthodox	Married	No			2	2	2	No
Orthodox	Married	Yes	Yes	3rd	1	1	1	No
Orthodox	Married	Yes	Yes	8th	4	4	4	No
Orthodox	Married	Yes	Yes	5th	3	3	3	No
Orthodox	Married	Yes	Yes	3rd	5	5	5	No
Orthodox	Married	No			3	3	3	No
Orthodox	Divorced				1	1	1	No
Orthodox	Married	Yes	No	5th	2	2	2	No
Orthodox	Married	Yes	Yes	4th	1	1	1	No
Orthodox	Married	No			5	5	5	No
Orthodox	Married	No			5	5	5	No
Orthodox	Married	Yes	Yes	3rd	2	2	2	No
Orthodox	Married	No			1	1	1	No
Orthodox	Married	No			8	8	8	No
Orthodox	Married	Yes	No	2nd	6	6	6	No
Orthodox	Married	No			4	3	3	Yes
Orthodox	Married	Yes	Yes	8th	1	1	1	No
Orthodox	Married	No			3	3	3	No
Orthodox	Married	No			1	1	1	No
Orthodox	Married	No			4	4	4	No
Orthodox	Married	No			1	1	1	No
Orthodox	Married	Yes	Yes	6th	8	8	8	No
Orthodox	Married	Yes	Yes	5th	1	1	1	No
Orthodox	Married	Yes	No	3rd	3	3	3	No
Orthodox	Married	Yes	Yes	10th	1	1	1	No
Orthodox	Married	No			5	5	5	No
Orthodox	Married	Yes	Yes	2nd	2	1	1	Yes

For peer review only

For peer review only - http://bmjopen.bmj.com/site/about/guidelines.xhtml

Orthodox	Married	No			5	5	5	No
Orthodox	Married	Yes	Yes	9th	2	2	2	No
Orthodox	Married	Yes	Yes	3rd	2	2	2	No
Orthodox	Married	Yes	Yes	10th	1	1	1	No
Orthodox	Married	Yes	Yes	Technical/\	1	1	1	No
Orthodox	Married	Yes	Yes	5th	3	1	3	Yes
Orthodox	Married	Yes	Yes	8th	2	2	2	No
Orthodox	Married	No			2	2	2	No
Orthodox	Married	Yes	Yes	9th	2	2	2	No
Orthodox	Married	Yes	No	1st	3	3	3	No
Orthodox	Married	Yes	Yes	6th	2	2	2	No
Orthodox	Married	Yes	Yes	3rd	6	6	6	No
Orthodox	Married	No			2	2	2	No
Orthodox	Married	Yes	Yes	4th	3	3	2	Yes
Orthodox	Married	Yes	Yes	7th	1	1	1	No
Orthodox	Married	No			5	5	5	No
Orthodox	Married	Yes	Yes	10th	2	1	1	No
Orthodox	Married	No			2	2	2	No
Orthodox	Married	Yes	Yes	3rd	2	2	2	No
Orthodox	Married	No			1	1	1	No
Orthodox	Married	Yes	Yes	4th	4	4	3	No
Orthodox	Married	Yes	No	1st	7	7	7	No
Orthodox	Married	Yes	Yes	4th	3	2	2	Yes
Orthodox	Married	Yes	Yes	3rd	4	4	4	No
Orthodox	Married	No			2	2	2	No
Orthodox	Married	Yes	Yes	2nd	3	3	3	No
Orthodox	Married	No			4	4	4	No
Orthodox	Married	Yes	Yes	4th	3	3	3	No
Orthodox	Married	Yes	Yes	3rd	4	4	4	No
Orthodox	Married	Yes	Yes	10th	2	2	2	No
Orthodox	Married	No			6	5	5	No
Orthodox	Married	Yes	Yes	10th	2	2	2	No
Orthodox	Married	Yes	Yes	9th	1	1	1	No
Orthodox	Married	Yes	Yes	4th	7	8	8	No
Orthodox	Married	No			8	8	8	No
Orthodox	Married	No			5	5	5	No
Orthodox	Married	No			6	6	6	No
Orthodox	Married	No			5	5	0	No
Orthodox	Divorced				4	3	3	Yes
Orthodox	Married	Yes	Yes	4th	3	3	3	No
Orthodox	Married	Yes	Yes	4th	3	3	3	No
Orthodox	Married	No			2	2	2	No
Orthodox	Married	Yes	No	2nd	6	6	6	No
Orthodox	Divorced				2	2	2	No
Orthodox	Married	Yes	Yes	9th	1	1	1	No
Orthodox	Married	No			5	5	5	No
Orthodox	Married	Yes	Yes	7th	1	1	1	No
Orthodox	Married	Yes	Yes	4th	3	3	2	Yes
Orthodox	Married	No			3	3	3	No
Orthodox	Married	Yes	Yes	9th	2	2	2	No
Orthodox	Divorced				1	1	1	No
Orthodox	Married	Yes	Yes	4th	5	4	4	Yes

For peer review only

Orthodox	Married	No			5	5	5	No
Orthodox	Married	No			2	2	2	No
Orthodox	Married	Yes	Yes	5th	6	5	5	Yes
Orthodox	Married	Yes	Yes	2nd	4	4	3	No
Orthodox	Married	Yes	Yes	3rd	3	3	3	No
Orthodox	Married	Yes	Yes	3rd	1	1	1	No
Orthodox	Married	Yes	Yes	10th	2	2	2	No
Orthodox	Widowed				5	5	5	No
Orthodox	Married	Yes	Yes	3rd	10	10	5	Yes
Orthodox	Married	No			2	2	2	No
Orthodox	Married	Yes	Yes	6th	4	4	4	No
Orthodox	Married	Yes	Yes	3rd	1	1	1	No
Orthodox	Married	No			6	6	6	No
Orthodox	Married	No			4	4	4	No
Orthodox	Married	Yes	Yes	5th	2	2	2	No
Orthodox	Married	No			5	4	4	Yes
Orthodox	Married	Yes	Yes	8th	7	6	6	Yes
Orthodox	Married	No			7	7	7	No
Orthodox	Married	No			4	4	4	No
Orthodox	Married	Yes	Yes	10th	4	4	4	No
Orthodox	Married	Yes	Yes	5th	2	2	2	No
Orthodox	Married	Yes	Yes	4th	7	7	7	No
Orthodox	Married	Yes	Yes	5th	5	5	5	No
Orthodox	Married	No			6	6	4	Yes
Orthodox	Married	Yes	Yes	4th	4	3	3	Yes
Orthodox	Married	No			6	6	6	No
Orthodox	Married	No			5	5	5	No
Orthodox	Widowed				5	5	5	No
Orthodox	Divorced				1	1	1	No
Orthodox	Married	No			3	4	4	No
Orthodox	Married	No			5	5	5	No
Orthodox	Married	Yes	Yes	5th	2	2	2	No
Orthodox	Married	Yes	Yes	8th	2	2	2	No
Orthodox	Married	Yes	Yes	5th	4	4	4	No
Orthodox	Married	Yes	No	8th	5	4	4	Yes
Orthodox	Married	No			4	4	4	No
Orthodox	Married	Yes	Yes	7th	3	3	3	No
Orthodox	Married	No			6	6	6	No
Orthodox	Married	Yes	No	1st	6	6	5	No
Orthodox	Married	Yes	Yes	3rd	1	1	1	No
Orthodox	Married	No			5	5	5	No
Orthodox	Married	Yes	Yes	2nd	5	4	4	Yes
Orthodox	Married	No			9	9	9	No
Orthodox	Married	No			4	4	4	No
Orthodox	Married	No			4	4	4	No
Orthodox	Married	No			2	2	2	No
Orthodox	Married	Yes	No	8th	4	4	4	No
Orthodox	Married	No			8	8	8	No
Orthodox	Married	Yes	Yes	4th	4	4	4	No
Orthodox	Married	Yes	Yes	3rd	5	5	5	No
Orthodox	Married	No			3	3	3	No
Orthodox	Married	No			1	1	1	No

For peer review only

Orthodox	Married	No			4	2	2	Yes
Orthodox	Married	No			8	7	6	No
Orthodox	Married	Yes	Yes	University/	2	2	2	No
Orthodox	Married	Yes	Yes	3rd	7	7	6	No
Orthodox	Married	Yes	Yes	10th	1	1	1	No
Orthodox	Married	Yes	No	1st	2	2	2	No
Orthodox	Married	No			3	3	3	No
Orthodox	Divorced				2	2	2	No
Orthodox	Married	No			4	4	4	No
Orthodox	Married	Yes	Yes	4th	1	1	1	No
Orthodox	Married	No			11	10	10	Yes
Orthodox	Divorced				1	1	1	No
Orthodox	Married	No			5	5	5	No
Orthodox	Married	No			3	3	3	No
Orthodox	Married	Yes	Yes	University/	3	3	3	No
Orthodox	Married	No			4	4	4	No
Orthodox	Married	No			2	2	2	No
Orthodox	Married	Yes	Yes	6th	3	3	3	No
Orthodox	Married	Yes	No	2nd	2	2	2	No
Orthodox	Married	Yes	Yes	4th	5	5	5	No
Orthodox	Married	No			4	4	4	No
Orthodox	Married	Yes	Yes	8th	5	5	5	No
Orthodox	Married	Yes	Yes	4th	6	6	6	No
Orthodox	Married	No			1	1	1	No
Orthodox	Married	Yes	Yes	3rd	4	5	5	No
Orthodox	Married	No			4	3	3	Yes
Orthodox	Married	No			5	5	5	No
Orthodox	Married	Yes	Yes	4th	5	5	5	No
Orthodox	Married	No			6	5	5	Yes
Orthodox	Married	No			5	5	5	No
Orthodox	Married	Yes	Yes	9th	1	1	1	No
Orthodox	Married	No			9	9	9	No
Orthodox	Married	Yes	Yes	9th	6	5	5	Yes
Orthodox	Married	Yes	No	1st	9	9	9	Yes
Orthodox	Married	Yes	Yes	7th	6	6	6	No
Orthodox	Married	No			1	1	1	No
Orthodox	Married	No			3	3	3	No
Orthodox	Married	No			3	3	3	No
Orthodox	Married	Yes	Yes	9th	9	6	6	Yes
Orthodox	Married	No			5	4	4	Yes
Orthodox	Married	Yes	Yes	3rd	1	1	1	No
Orthodox	Married	Yes	Yes	7th	2	2	2	No
Orthodox	Married	Yes	Yes	5th	2	2	2	No
Orthodox	Married	Yes	Yes	3rd	6	6	6	No
Orthodox	Married	Yes	Yes	6th	7	7	7	No
Orthodox	Married	Yes	No	2nd	9	9	9	No
Orthodox	Married	Yes	Yes	10th	1	1	1	No
Orthodox	Married	No			5	5	5	No
Orthodox	Married	Yes	Yes	5th	4	4	4	No
Orthodox	Married	Yes	No	1st	3	3	3	No
Orthodox	Married	No			7	6	6	Yes
Orthodox	Married	No			6	6	6	No

For peer review only

Orthodox	Married	No			10	9	9	Yes
Orthodox	Married	No			5	5	5	No
Orthodox	Married	Yes	Yes	3rd	2	2	2	No
Orthodox	Married	No			6	6	6	No
Orthodox	Separated				1	1	1	No
Orthodox	Married	No			4	4	4	No
Orthodox	Married	Yes	Yes	6th	2	2	2	No
Orthodox	Married	Yes	Yes	10th	1	1	1	No
Orthodox	Married	No			8	9	9	No
Orthodox	Married	No			3	3	3	No
Orthodox	Married	Yes	Yes	3rd	2	2	2	No
Orthodox	Married	No			2	2	2	No
Orthodox	Married	Yes	Yes	9th	1	1	1	No
Orthodox	Married	No			3	3	2	No
Orthodox	Married	No			10	8	8	Yes
Orthodox	Married	Yes	Yes	10th	1	1	1	No
Orthodox	Married	No			3	3	3	No
Orthodox	Married	Yes	Yes	5th	3	3	3	No
Orthodox	Married	No			5	5	5	No
Orthodox	Married	No			5	5	5	No
Orthodox	Married	Yes	Yes	7th	2	2	2	No
Orthodox	Married	Yes	Yes	4th	6	6	6	No
Orthodox	Married	Yes	Yes	9th	2	2	1	No
Orthodox	Married	No			5	5	5	No
Orthodox	Married	No			5	5	5	No
Orthodox	Married	Yes	Yes	5th	7	7	7	No
Orthodox	Married	No			5	5	5	No
Orthodox	Married	No			8	8	7	No
Orthodox	Married	Yes	Yes	6th	1	1	1	No
Orthodox	Married	No			4	4	4	No
Orthodox	Married	Yes	Yes	4th	5	4	4	Yes
Orthodox	Married	Yes	Yes	10th	3	3	3	No
Orthodox	Married	Yes	Yes	10th	1	1	1	No
Orthodox	Married	No			1	1	1	No
Orthodox	Married	Yes	Yes	4th	2	2	2	No
Orthodox	Married	Yes	Yes	5th	2	2	2	No
Orthodox	Married	No			7	7	7	No
Orthodox	Married	No			6	6	6	No
Orthodox	Married	Yes	Yes	4th	5	5	6	No
Orthodox	Married	Yes	Yes	3rd	7	7	6	No
Orthodox	Married	Yes	Yes	10th	5	5	5	No
Orthodox	Married	Yes	Yes	9th	3	3	3	No
Orthodox	Married	No			1	1	1	No
Orthodox	Married	Yes	Yes	10th	2	2	2	No
Orthodox	Married	No			8	8	3	No
Orthodox	Married	No			5	5	5	No
Orthodox	Married	No			8	7	7	Yes
Orthodox	Married	Yes	Yes	10th	2	2	2	No
Orthodox	Married	Yes	Yes	9th	1	1	1	No
Orthodox	Married	No			3	3	3	No
Orthodox	Married	Yes	Yes	4th	1	1	1	No
Orthodox	Married	Yes	Yes	8th	2	1	1	Yes

For peer review only

Orthodox	Married	Yes	Yes	3rd	2	2	2	No
Orthodox	Married	Yes	Yes	8th	8	8	8	No
Orthodox	Married	Yes	Yes	5th	9	9	9	No
Orthodox	Married	Yes	Yes	6th	3	3	3	No
Orthodox	Married	No			5	5	5	No
Orthodox	Married	Yes	Yes	6th	1	1	1	No
Orthodox	Married	Yes	Yes	10th	4	4	4	No
Orthodox	Married	No			4	4	4	No
Orthodox	Married	No			6	5	5	Yes
Orthodox	Married	No			5	4	4	Yes
Orthodox	Married	No			4	4	4	No
Orthodox	Married	Yes	Yes	10th	2	2	2	No
Orthodox	Married	No			5	4	4	Yes
Orthodox	Married	No			4	4	4	No
Orthodox	Married	Yes	Yes	10th	6	6	6	No
Orthodox	Married	Yes	Yes	9th	4	4	4	No
Orthodox	Married	Yes	Yes	10th	1	1	1	No
Orthodox	Married	No			2	2	2	No
Orthodox	Married	No			5	5	5	No
Orthodox	Married	No			1	1	1	No
Orthodox	Married	Yes	Yes	4th	6	6	6	No
Orthodox	Married	Yes	No	8th	7	7	7	No
Orthodox	Married	Yes	Yes	7th	2	2	2	No
Orthodox	Married	Yes	Yes	8th	6	6	6	No
Orthodox	Married	Yes	Yes	6th	3	3	3	No
Orthodox	Married	Yes	Yes	2nd	3	3	3	No
Orthodox	Married	Yes	Yes	7th	2	2	2	No
Orthodox	Married	Yes	Yes	5th	1	1	1	No
Orthodox	Married	Yes	Yes	University/	3	2	2	Yes
Orthodox	Married	No			5	5	5	Yes
Orthodox	Married	No			7	6	6	Yes
Orthodox	Married	Yes	No	2nd	5	5	5	No
Orthodox	Divorced				6	6	6	No
Orthodox	Married	No			5	5	5	No
Orthodox	Married	No			3	3	3	No
Orthodox	Married	Yes	Yes	5th	6	6	6	No
Orthodox	Married	Yes	Yes	3rd	4	3	3	Yes
Orthodox	Married	No			5	5	5	Yes
Orthodox	Married	No			4	3	3	Yes
Orthodox	Married	Yes	Yes	5th	1	1	1	No
Orthodox	Married	No			5	5	5	No
Orthodox	Married	Yes	Yes	4th	2	2	2	No
Orthodox	Married	Yes	Yes	4th	5	4	4	Yes
Orthodox	Married	No			5	5	5	No
Orthodox	Married	No			2	2	2	No
Orthodox	Married	Yes	Yes	10th	1	1	1	No
Orthodox	Married	No			4	4	4	No
Orthodox	Married	Yes	No	1st	4	4	4	No
Orthodox	Married	No			2	2	2	No
Orthodox	Married	Yes	Yes	6th	6	6	6	No
Orthodox	Married	Yes	Yes	5th	5	5	5	No
Orthodox	Married	Yes	Yes	10th	1	1	1	No

For peer review only

Orthodox	Never married				1	1	1	No
Orthodox	Married	Yes	Yes	3rd	8	6	6	Yes
Orthodox	Married	Yes	Yes	4th	5	5	5	No
Orthodox	Married	Yes	Yes	5th	1	1	1	No
Orthodox	Married	Yes	Yes	6th	1	1	1	No
Orthodox	Married	No			6	6	6	No
Orthodox	Married	Yes	Yes	7th	1	1	1	No
Orthodox	Married	No			1	1	1	No
Orthodox	Married	Yes	Yes	8th	1	1	1	No
Orthodox	Married	Yes	Yes	8th	6	6	6	Yes
Orthodox	Married	Yes	No	8th	3	3	3	No
Orthodox	Married	No			6	6	6	No
Orthodox	Married	Yes	Yes	4th	8	8	8	No
Orthodox	Married	No			5	4	4	Yes
Orthodox	Married	Yes	Yes	3rd	6	4	4	Yes
Orthodox	Married	No			10	8	8	Yes
Orthodox	Married	Yes	Yes	4th	8	8	8	No
Orthodox	Married	No			6	6	6	No
Orthodox	Married	No			2	2	2	No
Orthodox	Married	Yes	Yes	6th	1	1	1	No
Orthodox	Married	Yes	Yes	5th	2	2	2	No
Orthodox	Married	Yes	Yes	5th	5	5	5	No
Orthodox	Married	Yes	Yes	7th	2	2	2	No
Orthodox	Married	No			4	4	4	No
Orthodox	Married	Yes	Yes	4th	7	7	7	No
Orthodox	Married	No			4	4	4	No
Orthodox	Married	No			3	3	3	No
Orthodox	Married	Yes	Yes	6th	2	2	2	No
Orthodox	Married	No			4	4	4	No
Orthodox	Married	Yes	Yes	University/	1	1	1	No
Orthodox	Married	Yes	No	2nd	7	7	7	No
Orthodox	Married	No			4	4	4	No
Orthodox	Married	Yes	Yes	5th	1	1	1	No
Orthodox	Married	No			7	7	7	No
Orthodox	Married	No			6	6	6	No
Orthodox	Married	Yes	Yes	4th	3	3	3	No
Orthodox	Married	Yes	Yes	University/	9	9	9	No
Orthodox	Married	No			3	3	3	No
Orthodox	Married	No			9	9	9	No
Orthodox	Married	No			3	3	3	Yes
Orthodox	Married	No			4	4	4	No
Orthodox	Married	No			10	6	6	Yes
Orthodox	Married	No			4	4	4	No
Orthodox	Married	Yes	Yes	10th	1	1	1	No
Orthodox	Married	Yes	Yes	5th	2	2	2	No
Orthodox	Married	Yes	Yes	8th	1	1	1	No
Orthodox	Married	No			5	4	4	Yes
Orthodox	Married	No			4	4	4	No
Orthodox	Married	No			4	4	4	No
Orthodox	Married	No			7	6	6	Yes
Orthodox	Married	No			4	4	4	No
Orthodox	Married	Yes	Yes	2nd	5	5	5	No

For peer review only

Orthodox	Married	Yes	Yes	6th	7	7	7	No
Orthodox	Married	Yes	Yes	5th	11	11	11	No
Orthodox	Married	Yes	Yes	6th	5	5	5	No
Orthodox	Married	No			4	4	4	No
Orthodox	Married	No			2	2	2	No
Orthodox	Married	No			4	4	4	No
Orthodox	Married	Yes	Yes	9th	1	1	1	No
Orthodox	Married	No			4	4	4	No
Orthodox	Married	Yes	Yes	7th	7	7	7	No
Orthodox	Married	Yes	Yes	5th	4	4	4	Yes
Orthodox	Married	Yes	Yes	8th	4	4	4	No
Orthodox	Married	Yes	Yes	3rd	5	5	5	No
Orthodox	Married	No			5	5	5	No
Orthodox	Married	Yes	Yes	5th	2	2	2	No
Orthodox	Married	No			4	4	4	No
Orthodox	Married	No			4	4	4	No
Orthodox	Married	No			7	6	6	Yes
Orthodox	Married	No			7	7	7	No
Orthodox	Married	No			5	5	5	No
Orthodox	Married	Yes	Yes	5th	1	1	1	No
Orthodox	Married	No			5	5	5	No
Orthodox	Married	No			5	5	5	Yes
Orthodox	Married	Yes	Yes	4th	2	2	2	No
Orthodox	Married	Yes	Yes	10th	5	5	5	No
Orthodox	Married	Yes	Yes	3rd	3	3	3	No
Orthodox	Married	No			8	8	8	No
Orthodox	Married	Yes	Yes	7th	2	2	2	No
Orthodox	Married	Yes	Yes	10th	3	3	3	No
Orthodox	Married	No			3	3	3	No
Orthodox	Married	No			3	3	3	No
Orthodox	Married	Yes	Yes	8th	1	1	1	No
Orthodox	Married	Yes	Yes	12th	3	3	3	No
Orthodox	Married	Yes	No	1st	2	2	2	No
Orthodox	Married	Yes	Yes	10th	3	3	3	No
Orthodox	Married	Yes	Yes	10th	1	1	1	No
Orthodox	Married	No			5	5	5	No
Orthodox	Married	No			5	5	5	No
Orthodox	Married	No			3	3	3	No
Orthodox	Married	Yes	Yes	5th	1	1	1	No
Orthodox	Married	No			6	6	6	No
Orthodox	Married	Yes	Yes	3rd	1	1	1	No
Orthodox	Married	No			5	5	5	No
Orthodox	Married	Yes	Yes	6th	1	1	1	No
Orthodox	Married	Yes	Yes	10th	1	1	1	No
Orthodox	Married	No			4	4	4	No
Orthodox	Married	Yes	Yes	4th	8	8	8	No
Orthodox	Married	Yes	Yes	10th	1	1	1	No
Orthodox	Married	No			5	4	4	Yes
Orthodox	Married	No			7	7	7	No
Orthodox	Married	Yes	Yes	University/	1	1	1	No
Orthodox	Married	Yes	Yes	7th	6	6	6	No
Orthodox	Married	Yes	Yes	9th	2	1	1	No

For peer review only

Orthodox	Married	Yes	Yes	12th	1	1	1	No
Orthodox	Married	No			6	6	6	No
Orthodox	Married	No			2	3	3	No
Orthodox	Married	No			2	2	2	No
Orthodox	Married	Yes	No	3rd	2	2	2	No
Orthodox	Married	Yes	Yes	4th	4	4	4	No
Orthodox	Married	No			4	4	4	No
Orthodox	Married	Yes	No	3rd	2	2	2	No
Orthodox	Married	Yes	Yes	4th	3	2	2	Yes
Orthodox	Married	Yes	Yes	8th	3	3	3	No
Orthodox	Married	Yes	Yes	5th	4	4	4	No
Orthodox	Married	No			9	7	7	Yes
Orthodox	Married	No			5	5	5	No
Orthodox	Married	Yes	Yes	7th	2	2	2	No
Orthodox	Married	Yes	Yes	5th	3	3	3	No
Orthodox	Married	No			2	2	2	No
Orthodox	Married	No			10	7	7	Yes
Orthodox	Married	No			4	4	4	No
Orthodox	Married	Yes	Yes	2nd	8	7	7	Yes
Orthodox	Married	Yes	Yes	7th	4	4	4	No
Orthodox	Married	No			3	3	3	No
Orthodox	Married	No			2	2	2	No
Orthodox	Married	Yes	Yes	4th	4	4	4	No
Orthodox	Married	Yes	Yes	4th	6	6	6	No
Orthodox	Married	Yes	Yes	3rd	6	5	5	Yes
Orthodox	Married	No			5	5	5	No
Orthodox	Married	Yes	Yes	3rd	2	2	2	No
Orthodox	Married	No			2	2	2	No
Orthodox	Married	Yes	Yes	10th	1	1	1	No
Orthodox	Married	No			4	4	4	No
Orthodox	Married	No			1	1	1	No
Orthodox	Married	Yes	Yes	8th	1	1	1	No
Orthodox	Married	Yes	No	2nd	2	0	2	No
Orthodox	Married	Yes	Yes	10th	1	1	1	No
Orthodox	Married	Yes	Yes	5th	1	1	1	No
Orthodox	Married	No			1	1	1	No
Orthodox	Married	Yes	Yes	5th	4	4	4	No
Orthodox	Married	Yes	Yes	6th	1	1	1	No
Orthodox	Married	Yes	Yes	4th	3	3	3	No
Orthodox	Married	Yes	Yes	10th	1	1	1	No
Orthodox	Married	Yes	Yes	3rd	5	4	4	Yes
Orthodox	Married	Yes	Yes	4th	2	2	2	No
Orthodox	Married	Yes	Yes	5th	5	5	5	No
Orthodox	Married	No			7	7	7	No
Orthodox	Married	Yes	Yes	5th	4	4	4	No
Orthodox	Married	Yes	Yes	6th	1	1	1	No
Orthodox	Married	Yes	Yes	6th	2	2	2	No
Orthodox	Married	No			1	1	1	No
Orthodox	Married	Yes	Yes	6th	2	2	2	No
Orthodox	Married	Yes	Yes	4th	6	6	6	No
Orthodox	Married	No			3	3	3	No
Orthodox	Married	No			2	2	2	No

For peer review only

Orthodox	Married	No			5	5	5	No
Orthodox	Married	No			9	9	9	No
Orthodox	Married	Yes	Yes	8th	4	4	4	No
Orthodox	Married	Yes	Yes	2nd	7	7	7	No
Orthodox	Married	Yes	Yes	3rd	2	2	2	No
Orthodox	Married	No			5	5	5	No
Orthodox	Married	Yes	Yes	7th	2	2	2	No
Protestant	Married	Yes	Yes	University/	2	2	2	No
Orthodox	Married	No			3	3	3	No
Orthodox	Married	No			6	6	6	No
Orthodox	Married	No			3	3	3	No
Orthodox	Married	Yes	Yes	10th	1	1	1	No
Orthodox	Married	No			1	1	1	No
Orthodox	Married	No			7	7	7	No
Orthodox	Married	Yes	Yes	2nd	2	2	2	No
Orthodox	Married	No			8	8	8	Yes
Orthodox	Married	Yes	Yes	7th	2	2	2	No
Orthodox	Married	Yes	Yes	5th	3	3	3	No
Orthodox	Married	Yes	Yes	3rd	3	3	3	No
Orthodox	Married	Yes	No	1st	9	7	7	Yes
Orthodox	Married	No			2	2	2	No
Orthodox	Married	No			1	1	1	No
Orthodox	Married	No			3	3	3	No
Orthodox	Married	No			3	3	3	No
Orthodox	Married	No			1	1	1	No
Orthodox	Married	Yes	Yes	3rd	2	2	2	No
Orthodox	Married	No			6	6	6	No
Orthodox	Married	No			5	4	4	Yes
Orthodox	Married	Yes	Yes	5th	5	5	5	No
Orthodox	Married	Yes	Yes	5th	1	1	1	No
Orthodox	Married	No			9	6	6	Yes
Orthodox	Married	Yes	Yes	3rd	1	1	1	No
Orthodox	Married	No			5	5	5	No
Orthodox	Married	No			6	6	5	No
Orthodox	Married	Yes	No	1st	5	5	5	No
Orthodox	Married	Yes	Yes	3rd	5	5	5	No
Orthodox	Married	No			6	6	6	No
Orthodox	Married	Yes	Yes	5th	1	1	1	No
Orthodox	Married	No			5	5	5	No
Orthodox	Married	No			7	5	5	Yes
Orthodox	Married	No			5	5	5	No
Orthodox	Married	Yes	Yes	7th	2	2	2	No
Orthodox	Married	No			5	5	5	No
Orthodox	Married	Yes	Yes	2nd	5	5	5	No
Orthodox	Married	Yes	Yes	10th	1	1	1	No
Orthodox	Married	No			2	3	3	No
Orthodox	Married	Yes	Yes	3rd	4	3	3	Yes
Orthodox	Married	Yes	Yes	University/	1	1	1	No
Orthodox	Married	Yes	Yes	3rd	3	3	3	No
Orthodox	Married	No			3	3	3	No
Orthodox	Married	Yes	Yes	8th	1	1	1	No
Orthodox	Married	No			3	3	3	No

For peer review only

Orthodox	Married	Yes	Yes	Technical/v	2	2	2	No
Orthodox	Married	No			5	5	5	No
Orthodox	Married	No			1	1	1	No
Orthodox	Married	No			3	3	3	No
Orthodox	Married	Yes	Yes	5th	1	1	1	No
Orthodox	Married	No			4	4	4	No
Orthodox	Married	No			5	5	5	No
Orthodox	Married	No			2	2	2	No
Orthodox	Married	No			1	1	1	No
Orthodox	Married	Yes	Yes	3rd	1	1	1	No
Orthodox	Married	Yes	Yes	3rd	7	6	6	Yes
Orthodox	Married	Yes	Yes	4th	6	6	6	No
Orthodox	Married	Yes	Yes	8th	1	1	1	No
Orthodox	Married	No			3	3	3	No
Orthodox	Married	No			5	5	5	No
Orthodox	Married	No			6	5	5	Yes
Orthodox	Married	No			3	3	3	No
Orthodox	Married	No			6	6	6	No
Orthodox	Married	Yes	Yes	4th	1	1	1	No
Orthodox	Married	No			7	7	7	No
Orthodox	Married	Yes	Yes	1st	8	9	9	No
Orthodox	Married	No			2	2	2	No
Orthodox	Married	No			4	4	4	No
Orthodox	Married	No			7	7	7	No
Orthodox	Married	No			1	1	1	No
Orthodox	Married	No			6	5	5	Yes
Orthodox	Married	Yes	Yes	9th	1	1	1	No
Orthodox	Married	No			4	4	4	No
Orthodox	Married	No			4	4	4	No
Orthodox	Married	No			5	5	5	No
Orthodox	Married	Yes	Yes	3rd	6	6	6	No
Orthodox	Married	Yes	Yes	3rd	6	5	5	Yes
Orthodox	Married	No			1	1	1	No
Orthodox	Married	No			3	3	3	No
Orthodox	Married	No			5	4	4	Yes
Orthodox	Married	No			9	9	9	No
Orthodox	Married	Yes	Yes	4th	2	2	2	No
Orthodox	Married	No			1	1	1	No
Orthodox	Married	Yes	Yes	3rd	10	8	8	Yes
Orthodox	Married	Yes	Yes	4th	4	4	4	No
Orthodox	Married	Yes	Yes	5th	6	5	5	Yes
Orthodox	Married	Yes	No	1st	9	9	9	No
Orthodox	Married	No			7	7	7	No
Orthodox	Married	No			7	7	7	Yes
Orthodox	Married	No			7	5	5	Yes
Orthodox	Married	Yes	Yes	7th	2	2	2	No
Orthodox	Married	Yes	Yes	7th	2	2	2	No
Orthodox	Married	No			9	7	7	Yes
Orthodox	Married	Yes	Yes	3rd	3	1	1	Yes
Orthodox	Married	Yes	Yes	3rd	9	7	7	Yes
Orthodox	Married	No			6	6	6	No
Orthodox	Married	Yes	Yes	3rd	7	5	5	Yes

For peer review only

Orthodox	Married	No			5	5	5	No
Orthodox	Married	Yes	Yes	10th	3	3	3	No
Orthodox	Married	No			4	4	4	No
Orthodox	Married	Yes	Yes	3rd	9	9	6	No
Orthodox	Married	No			3	3	3	No
Orthodox	Married	No			5	5	4	No
Orthodox	Married	Yes	Yes	2nd	2	2	2	No
Orthodox	Married	No			7	7	7	No
Orthodox	Married	Yes	Yes	8th	3	3	3	No
Orthodox	Married	Yes	Yes	10th	8	7	7	Yes
Orthodox	Married	Yes	Yes	3rd	7	7	7	No
Orthodox	Married	No			1	1	1	No
Orthodox	Married	Yes	Yes	3rd	3	3	3	No
Orthodox	Married	Yes	Yes	3rd	12	6	6	Yes
Orthodox	Married	Yes	Yes	4th	7	7	7	No
Orthodox	Married	Yes	Yes	8th	2	1	1	Yes
Orthodox	Married	No			4	4	4	No
Orthodox	Married	Yes	Yes	10th	2	2	2	No
Orthodox	Married	Yes	Yes	12th	12	12	12	No
Orthodox	Married	No			2	2	2	No
Orthodox	Married	No			4	4	4	No
Orthodox	Married	No			6	6	6	No
Orthodox	Married	No			10	8	8	Yes
Orthodox	Married	Yes	Yes	8th	5	4	4	Yes
Orthodox	Married	No			5	5	5	No
Orthodox	Married	No			2	2	2	No
Orthodox	Married	No			3	3	3	No
Orthodox	Married	Yes	Yes	4th	5	5	5	No
Orthodox	Married	Yes	Yes	6th	1	1	1	No
Orthodox	Married	No			5	5	5	No
Orthodox	Married	Yes	No	3rd	7	7	7	No
Orthodox	Married	Yes	Yes	3rd	2	2	2	No
Orthodox	Married	Yes	Yes	8th	6	6	6	No
Orthodox	Married	No			9	7	5	Yes
Orthodox	Married	No			7	7	7	No
Orthodox	Married	Yes	Yes	3rd	1	1	1	No
Muslim	Married	No			2	2	2	No
Orthodox	Married	No			5	5	5	No
Orthodox	Married	No			5	5	5	No
Orthodox	Married	Yes	Yes	8th	7	7	7	No
Orthodox	Married	Yes	Yes	University/	1	1	1	No
Orthodox	Married	No			13	6	9	Yes
Orthodox	Married	No			5	5	5	No
Orthodox	Married	No			5	5	5	No
Orthodox	Married	No			6	6	6	No
Orthodox	Married	No			4	4	4	No
Orthodox	Married	Yes	Yes	5th	3	3	3	No
Orthodox	Married	No			6	6	6	No
Orthodox	Married	No			1	1	1	No
Orthodox	Married	No			6	6	6	No
Orthodox	Married	Yes	Yes	7th	3	3	3	No
Orthodox	Married	Yes	No	3rd	3	3	3	No

For peer review only

Orthodox	Married	No			5	5	5	No
Orthodox	Married	Yes	Yes	3rd	5	5	5	No
Orthodox	Married	No			5	5	5	No
Orthodox	Married	No			7	7	7	No
Orthodox	Married	No			2	2	2	No
Orthodox	Married	Yes	Yes	7th	2	2	2	No
Orthodox	Widowed				6	6	6	No
Orthodox	Married	Yes	Yes	4th	8	6	6	Yes
Orthodox	Married	No			3	3	3	No
Orthodox	Married	No			5	5	5	No
Orthodox	Married	No			7	7	7	No
Orthodox	Married	Yes	Yes	7th	2	2	2	No
Orthodox	Married	Yes	Yes	4th	4	4	4	No
Orthodox	Married	Yes	Yes	2nd	2	2	2	No
Orthodox	Married	Yes	Yes	10th	1	1	1	No
Orthodox	Married	Yes	Yes	4th	4	4	4	No
Orthodox	Married	Yes	Yes	7th	1	1	1	No
Orthodox	Married	Yes	Yes	12th	5	4	4	Yes
Orthodox	Married	No			6	6	6	No
Orthodox	Married	No			4	4	4	No
Orthodox	Married	Yes	Yes	7th	5	5	5	No
Orthodox	Married	No			4	4	4	No
Orthodox	Married	Yes	No	3rd	5	5	5	No
Orthodox	Married	Yes	Yes	7th	2	2	2	No
Orthodox	Married	No			4	2	2	Yes
Orthodox	Married	Yes	Yes	7th	5	5	5	No
Orthodox	Married	Yes	Yes	10th	1	1	1	No
Orthodox	Married	No			6	4	4	Yes
Orthodox	Married	No			7	7	7	No
Orthodox	Married	Yes	Yes	7th	9	9	9	No
Orthodox	Married	No			4	4	4	No
Orthodox	Married	No			3	3	3	No
Orthodox	Married	No			3	3	3	No
Orthodox	Married	No			5	5	5	No
Orthodox	Married	No			5	5	5	No
Orthodox	Married	Yes	Yes	1st	2	2	2	No
Orthodox	Married	No			9	8	8	Yes
Orthodox	Married	Yes	Yes	6th	1	1	1	No
Orthodox	Married	Yes	Yes	4th	10	9	9	Yes
Orthodox	Married	No			9	9	9	No
Orthodox	Married	No			9	9	7	No
Orthodox	Married	Yes	Yes	4th	5	5	5	No
Orthodox	Married	No			5	5	5	No
Orthodox	Married	No			7	7	7	No
Orthodox	Married	No			4	3	3	Yes
Orthodox	Married	Yes	Yes	10th	1	1	1	No
Orthodox	Married	Yes	Yes	10th	1	1	1	No
Orthodox	Married	Yes	Yes	3rd	7	7	7	No
Orthodox	Married	Yes	Yes	7th	1	1	1	No
Orthodox	Married	No			5	5	5	No
Orthodox	Married	Yes	Yes	7th	2	2	2	No
Orthodox	Married	No			2	2	2	No

For peer review only

Orthodox	Married	Yes	Yes	4th	4	4	4	No
Orthodox	Divorced				5	5	5	No
Orthodox	Married	No			1	1	1	No
Orthodox	Divorced				5	4	4	Yes
Protestant	Married	No			6	6	6	No
Orthodox	Married	No			3	3	3	No
Orthodox	Married	Yes	Yes	7th	5	5	5	No
Orthodox	Married	No			3	3	3	No
Orthodox	Married	Yes	Yes	6th	2	2	2	No
Orthodox	Married	Yes	Yes	6th	1	1	1	No
Orthodox	Married	No			3	3	2	Yes
Orthodox	Married	Yes	Yes	6th	1	1	1	No
Orthodox	Married	Yes	Yes	4th	5	5	5	No
Protestant	Married	Yes	Yes	3rd	1	1	1	No
Orthodox	Married	No			4	4	4	No
Orthodox	Married	No			4	4	4	No
Orthodox	Married	No			6	6	6	No
Orthodox	Married	Yes	Yes	10th	2	2	2	No
Orthodox	Married	No			3	3	3	No
Orthodox	Married	No			2	2	2	No
Orthodox	Married	Yes	Yes	5th	2	2	2	No
Orthodox	Married	Yes	Yes	6th	1	1	1	No
Orthodox	Married	Yes	Yes	5th	5	5	5	No
Orthodox	Married	No			2	2	2	No
Orthodox	Married	No			8	8	8	No
Orthodox	Married	Yes	Yes	10th	1	1	1	No
Orthodox	Married	Yes	Yes	6th	4	4	4	No
Orthodox	Married	No			3	3	3	No
Orthodox	Married	No			4	4	4	No
Orthodox	Married	Yes	Yes	8th	2	2	2	No
Orthodox	Married	No			6	6	6	No
Orthodox	Married	Yes	Yes	8th	2	2	2	No
Orthodox	Married	No			10	7	7	Yes
Protestant	Married	No			4	4	4	No
Protestant	Married	Yes	Yes	5th	4	4	4	No
Orthodox	Married	Yes	Yes	6th	5	5	5	No
Orthodox	Married	Yes	Yes	7th	1	1	1	No
Orthodox	Married	Yes	Yes	9th	3	3	3	No
Orthodox	Married	Yes	Yes	5th	7	7	7	No
Orthodox	Married	No			4	4	4	No
Orthodox	Married	Yes	Yes	8th	3	3	3	No
Orthodox	Married	Yes	Yes	4th	2	2	2	No
Orthodox	Married	No			2	2	2	No
Protestant	Married	Yes	Yes	10th	3	3	3	No
Orthodox	Married	No			1	1	1	No
Orthodox	Married	Yes	Yes	8th	1	1	1	No
Orthodox	Married	Yes	Yes	6th	2	2	2	No
Orthodox	Married	Yes	Yes	9th	2	2	2	No
Orthodox	Married	No			1	71	1	No
Orthodox	Married	No			6	6	6	No
Orthodox	Married	Yes	Yes	6th	2	2	2	No
Orthodox	Married	Yes	Yes	7th	2	2	2	No

For peer review only

Orthodox	Married	Yes	Yes	6th	3	3	3	No
Orthodox	Married	No			1	1	1	No
Orthodox	Married	Yes	Yes	10th	2	2	2	No
Orthodox	Married	Yes	Yes	3rd	4	4	4	No
Orthodox	Divorced				2	2	2	No
Orthodox	Married	Yes	Yes	10th	2	2	2	No
Orthodox	Married	Yes	Yes	4th	1	1	1	No
Orthodox	Married	No			4	4	4	No
Orthodox	Married	No			5	5	5	No
Orthodox	Married	No			2	2	2	No
Orthodox	Married	Yes	Yes	University/	2	1	1	Yes
Orthodox	Married	No			4	4	4	No
Orthodox	Married	No			3	3	3	No
Orthodox	Married	Yes	Yes	3rd	5	5	5	No
Orthodox	Married	Yes	Yes	10th	1	1	1	No
Orthodox	Married	No			3	3	3	No
Protestant	Married	No			3	3	3	No
Orthodox	Married	Yes	Yes	10th	4	4	4	No
Orthodox	Widowed				4	4	4	No
Orthodox	Married	Yes	Yes	7th	3	3	3	No
Orthodox	Married	Yes	Yes	2nd	4	4	4	No
Orthodox	Married	No			2	2	2	No
Orthodox	Married	No			2	2	2	No
Orthodox	Married	Yes	No	3rd	2	2	2	No
Orthodox	Married	Yes	Yes	7th	3	3	3	No
Orthodox	Married	Yes	Yes	7th	3	3	3	No
Orthodox	Married	Yes	Yes	8th	4	4	4	No
Orthodox	Married	Yes	Yes	10th	1	1	1	No
Orthodox	Married	Yes	Yes	8th	2	2	2	No
Orthodox	Married	Yes	Yes	6th	3	3	3	No
Orthodox	Married	No			2	2	2	No
Orthodox	Married	Yes	Yes	1st	2	2	2	No
Orthodox	Married	Yes	Yes	10th	3	3	3	No
Orthodox	Married	Yes	Yes	5th	1	1	1	No
Orthodox	Married	Yes	Yes	6th	4	4	4	No
Orthodox	Married	Yes	Yes	8th	2	2	2	No
Orthodox	Married	No			3	3	3	No
Orthodox	Married	No			8	8	8	Yes
Orthodox	Divorced				1	1	1	No
Orthodox	Married	No			1	1	1	No
Orthodox	Married	Yes	Yes	2nd	3	3	3	No
Orthodox	Widowed				1	1	1	No
Orthodox	Married	Yes	Yes	5th	2	2	2	No
Orthodox	Married	Yes	Yes	5th	4	4	4	No
Orthodox	Married	No			6	6	6	No
Orthodox	Married	Yes	Yes	6th	2	2	2	No
Orthodox	Married	No			1	1	1	No
Orthodox	Married	Yes	Yes	5th	2	2	2	No
Muslim	Married	No			4	4	4	No
Orthodox	Married	Yes	Yes	7th	6	6	6	Yes
Orthodox	Married	No			1	1	1	No
Orthodox	Married	No			3	3	3	No

For peer review only

Orthodox	Married	No			1	1	1	No
Orthodox	Married	No			2	2	2	No
Orthodox	Married	Yes	Yes	7th	2	2	2	No
Orthodox	Married	No			6	6	6	No
Orthodox	Married	No			5	5	5	No
Orthodox	Married	Yes	Yes	University/	2	2	2	No
Orthodox	Married	Yes	Yes	4th	2	2	2	No
Orthodox	Separated				1	1	1	No
Orthodox	Married	No			4	4	4	No
Orthodox	Married	No			2	2	2	No
Orthodox	Married	No			2	2	2	No
Orthodox	Married	No			6	5	5	Yes
Orthodox	Married	No			2	2	2	No
Orthodox	Married	No			1	1	1	No
Orthodox	Married	No			5	5	5	No
Protestant	Married	No			1	1	1	No
Orthodox	Married	No			2	2	2	No
Orthodox	Married	Yes	Yes	5th	1	1	1	No
Orthodox	Married	Yes	Yes	10th	2	2	2	No
Orthodox	Divorced				1	1	1	No
Orthodox	Married	Yes	Yes	4th	5	5	5	Yes
Orthodox	Married	Yes	Yes	7th	1	1	1	No
Orthodox	Married	Yes	Yes	8th	3	3	3	No
Orthodox	Married	Yes	Yes	5th	1	1	1	No
Orthodox	Married	Yes	Yes	4th	2	2	2	No
Orthodox	Married	No			1	1	1	No
Orthodox	Never married				1	1	1	No
Orthodox	Married	No			3	3	3	No
Orthodox	Married	Yes	Yes	6th	4	4	4	No
Orthodox	Married	Yes	Yes	5th	6	6	6	No
Orthodox	Married	No			1	1	1	No
Orthodox	Married	Yes	Yes	6th	1	1	1	No
Orthodox	Widowed				3	3	3	No
Orthodox	Married	No			6	6	6	No
Orthodox	Married	Yes	Yes	4th	3	3	3	No
Orthodox	Married	Yes	Yes	4th	1	1	1	No
Orthodox	Married	Yes	Yes	7th	4	3	4	No
Orthodox	Married	Yes	Yes	8th	3	3	3	No
Orthodox	Married	No			4	4	4	No
Orthodox	Married	No			4	4	4	No
Orthodox	Married	No			5	5	5	No
Orthodox	Married	Yes	Yes	2nd	5	5	5	No
Orthodox	Married	Yes	Yes	8th	3	3	3	No
Orthodox	Married	No			1	1	1	No
Orthodox	Married	Yes	Yes	6th	10	10	9	No
Orthodox	Married	Yes	Yes	8th	2	2	2	No
Orthodox	Married	Yes	Yes	7th	2	2	2	No
Orthodox	Married	No			3	3	3	No
Orthodox	Married	Yes	Yes	2nd	3	3	3	No
Orthodox	Married	No			3	3	3	No
Orthodox	Married	Yes	Yes	10th	1	1	1	No
Orthodox	Married	Yes	Yes	10th	1	1	1	No

For peer review only

Orthodox	Married	No			5	5	5	No
Orthodox	Married	No			3	3	3	No
Orthodox	Married	Yes	Yes	7th	4	4	4	No
Orthodox	Married	Yes	Yes	4th	1	1	1	No
Orthodox	Married	Yes	Yes	7th	1	1	1	No
Orthodox	Married	No			1	1	1	No
Orthodox	Married	Yes	Yes	1st	3	3	3	No
Orthodox	Married	No			4	4	4	No
Orthodox	Married	No			2	2	2	No
Orthodox	Married	Yes	Yes	4th	3	2	2	Yes
Orthodox	Married	Yes	No	6th	1	1	1	No
Orthodox	Married	Yes	Yes	4th	1	1	1	No
Orthodox	Married	No			4	4	4	No
Orthodox	Married	No			1	1	1	No
Orthodox	Married	No			4	4	4	No
Orthodox	Married	No			9	9	9	No
Protestant	Married	Yes	Yes	7th	2	2	2	No
Orthodox	Married	No			5	5	5	No
Orthodox	Married	Yes	Yes	12th	1	1	1	No
Orthodox	Married	No			4	4	4	No
Orthodox	Divorced				2	2	2	No
Orthodox	Married	Yes	Yes	4th	2	2	2	No
Orthodox	Married	Yes	Yes	1st	5	5	5	Yes
Orthodox	Married	Yes	Yes	7th	1	1	1	No
Orthodox	Married	Yes	Yes	8th	4	4	4	No
Orthodox	Married	No			5	5	5	No
Orthodox	Married	No			4	4	4	Yes
Protestant	Married	Yes	Yes	7th	4	4	4	No
Orthodox	Married	Yes	Yes	6th	3	3	3	No
Orthodox	Married	No			2	2	2	No
Orthodox	Married	No			4	4	4	No
Orthodox	Married	No			2	2	2	No
Orthodox	Married	No			4	4	4	No
Orthodox	Married	No			1	1	1	No
Orthodox	Married	Yes	Yes	6th	4	4	4	No
Orthodox	Married	Yes	Yes	8th	2	2	2	No
Orthodox	Married	Yes	No	3rd	3	3	3	No
Orthodox	Married	Yes	Yes	4th	1	1	1	No
Orthodox	Married	No			7	7	7	No
Orthodox	Married	No			4	4	4	No
Orthodox	Married	No			3	3	3	No
Orthodox	Married	No			1	1	1	No
Orthodox	Married	Yes	Yes	7th	3	3	3	No
Orthodox	Married	No			2	2	2	No
Orthodox	Married	No			5	5	5	No
Orthodox	Married	No			2	2	2	No
Orthodox	Married	Yes	Yes	3rd	8	7	7	Yes
Orthodox	Separated				1	1	1	No
Orthodox	Married	Yes	Yes	7th	2	2	2	No
Orthodox	Married	Yes	Yes	4th	2	2	2	No
Orthodox	Married	No			5	5	5	No
Orthodox	Married	No			1	1	1	No

For peer review only

Orthodox	Married	No			8	8	8	No
Orthodox	Married	Yes	Yes	4th	6	7	7	No
Orthodox	Married	Yes	Yes	6th	2	2	2	No
Orthodox	Married	No			5	5	5	No
Orthodox	Married	Yes	Yes	5th	6	6	6	No
Orthodox	Married	No			6	6	6	No
Orthodox	Married	Yes	No	3rd	5	5	5	No
Orthodox	Married	No			3	3	3	No
Orthodox	Married	Yes	Yes	5th	1	1	1	No
Orthodox	Married	Yes	Yes	6th	4	4	4	Yes
Orthodox	Married	No			3	3	3	No
Orthodox	Married	Yes	Yes	3rd	3	3	3	No
Orthodox	Married	Yes	Yes	7th	6	6	6	No
Orthodox	Married	No			1	1	1	No
Orthodox	Married	Yes	Yes	8th	1	1	1	No
Orthodox	Married	No			2	2	2	No
Orthodox	Married	No			1	1	1	No
Orthodox	Married	No			4	4	4	No
Orthodox	Married	Yes	Yes	8th	2	2	2	No
Orthodox	Married	Yes	Yes	10th	2	2	2	No
Orthodox	Married	Yes	Yes	5th	2	2	2	No
Orthodox	Married	No			6	6	6	No
Orthodox	Married	Yes	Yes	7th	1	1	1	No
Orthodox	Married	No			6	6	6	No
Orthodox	Married	Yes	Yes	5th	1	1	1	No
Orthodox	Married	No			4	4	4	No
Orthodox	Married	Yes	Yes	6th	2	2	2	No
Orthodox	Married	No			4	4	4	No
Orthodox	Married	Yes	Yes	6th	5	5	5	No
Orthodox	Married	No			2	2	2	No
Orthodox	Married	No			7	7	7	No
Orthodox	Married	No			6	6	6	No
Orthodox	Married	No			5	5	5	No
Orthodox	Married	Yes	Yes	10th	2	2	2	No
Orthodox	Married	Yes	Yes	5th	2	2	2	No
Orthodox	Married	No			3	3	3	No
Orthodox	Married	Yes	Yes	6th	2	2	2	No
Orthodox	Married	Yes	Yes	3rd	7	7	7	No
Orthodox	Married	Yes	Yes	8th	1	1	1	No
Orthodox	Married	No			6	6	6	No
Orthodox	Divorced				3	3	3	No
Orthodox	Married	Yes	Yes	6th	2	2	2	No
Orthodox	Married	Yes	Yes	3rd	6	6	6	No
Orthodox	Married	No			4	4	4	No
Orthodox	Married	Yes	Yes	5th	2	2	2	No
Orthodox	Married	No			3	3	3	No
Orthodox	Married	Yes	Yes	10th	1	1	1	No
Orthodox	Married	Yes	Yes	5th	6	6	6	No
Orthodox	Married	Yes	Yes	3rd	2	2	2	No
Orthodox	Married	Yes	Yes	5th	2	2	2	No
Orthodox	Married	Yes	Yes	8th	1	1	1	No
Orthodox	Married	Yes	Yes	12th	5	5	5	No

For peer review only

Orthodox	Married	Yes	Yes	4th	1	1	1	No
Orthodox	Married	Yes	Yes	5th	4	4	4	No
Orthodox	Never married				1	1	1	No
Orthodox	Married	Yes	Yes	5th	2	2	2	No
Orthodox	Married	No			3	3	3	Yes
Orthodox	Married	No			1	1	1	No
Orthodox	Married	No			3	3	3	No
Orthodox	Married	Yes	Yes	5th	3	3	3	No
Orthodox	Married	No			5	5	5	No
Orthodox	Married	Yes	Yes	4th	1	1	1	No
Orthodox	Married	Yes	Yes	6th	3	3	3	No
Orthodox	Married	Yes	Yes	6th	1	1	1	No
Orthodox	Married	Yes	Yes	12th	3	3	3	No
Orthodox	Married	Yes	Yes	8th	2	2	2	No
Orthodox	Married	Yes	Yes	4th	1	1	1	No
Orthodox	Married	Yes	Yes	3rd	4	4	4	Yes
Orthodox	Married	Yes	Yes	3rd	3	3	3	Yes
Protestant	Married	Yes	Yes	10th	3	3	3	No
Orthodox	Married	Yes	Yes	5th	2	2	2	No
Orthodox	Married	Yes	Yes	6th	4	4	4	No
Orthodox	Married	No			3	3	3	No
Orthodox	Married	Yes	Yes	3rd	5	5	5	No
Orthodox	Married	Yes	Yes	9th	1	1	1	No
Orthodox	Married	Yes	Yes	8th	1	1	1	No
Orthodox	Married	Yes	Yes	10th	2	2	2	No
Orthodox	Married	No			6	6	6	No
Orthodox	Married	No			2	2	2	No
Orthodox	Married	No			2	2	2	No
Orthodox	Married	Yes	Yes	7th	1	1	1	No
Protestant	Married	No			4	4	4	No
Orthodox	Married	No			8	8	8	No
Orthodox	Married	Yes	Yes	6th	2	2	2	No
Orthodox	Married	Yes	Yes	6th	1	1	1	No
Orthodox	Married	No			6	6	6	No
Orthodox	Married	Yes	Yes	6th	5	5	5	Yes
Orthodox	Married	Yes	Yes	9th	2	2	2	No
Orthodox	Married	Yes	Yes	4th	2	2	2	No
Traditional	Married	Yes	Yes	9th	2	2	2	No
Orthodox	Married	Yes	Yes	10th	5	5	5	No
Orthodox	Married	No			1	1	1	No
Orthodox	Married	Yes	Yes	5th	5	5	5	No
Orthodox	Married	Yes	Yes	12th	1	1	1	No
Orthodox	Married	Yes	Yes	4th	1	1	2	No
Orthodox	Married	Yes	Yes	3rd	4	4	4	No
Orthodox	Married	Yes	Yes	10th	1	1	1	No
Orthodox	Married	No			2	2	2	No
Orthodox	Married	Yes	Yes	8th	1	1	1	No
Orthodox	Married	Yes	Yes	7th	3	3	3	No
Orthodox	Married	No			3	3	3	No
Orthodox	Married	No			2	2	2	No
Orthodox	Married	Yes	Yes	7th	2	2	2	No
Orthodox	Married	Yes	Yes	2nd	4	4	4	No

For peer review only

Orthodox	Married	Yes	Yes	8th	1	1	1	No
Orthodox	Married	No			1	1	1	No
Protestant	Married	Yes	Yes	2nd	6	6	6	Yes
Orthodox	Married	Yes	Yes	3rd	3	3	3	No
Orthodox	Married	No			1	1	1	No
Orthodox	Married	Yes	Yes	10th	2	2	2	No
Orthodox	Married	Yes	Yes	4th	2	2	2	Yes
Orthodox	Married	Yes	Yes	8th	1	1	1	No
Orthodox	Married	Yes	Yes	5th	3	3	3	No
Orthodox	Married	Yes	Yes	6th	2	2	2	No
Orthodox	Married	No			1	1	1	No
Orthodox	Married	No			6	4	4	Yes
Orthodox	Married	Yes	Yes	5th	2	2	2	No
Orthodox	Married	Yes	Yes	9th	5	5	5	No
Protestant	Married	Yes	Yes	6th	1	1	1	No
Orthodox	Married	Yes	Yes	5th	2	2	2	Yes
Orthodox	Married	Yes	Yes	10th	2	2	2	No
Orthodox	Married	Yes	Yes	5th	4	4	4	No
Orthodox	Married	No			1	1	1	No
Orthodox	Married	Yes	Yes	6th	4	4	4	No
Orthodox	Married	No			2	2	2	No
Orthodox	Married	Yes	Yes	7th	2	2	2	No
Orthodox	Married	No			5	5	5	No
Orthodox	Married	Yes	Yes	5th	6	6	6	No
Orthodox	Married	Yes	Yes	7th	7	7	7	No
Orthodox	Married	No			3	3	3	No
Orthodox	Married	Yes	Yes	10th	1	1	1	No
Orthodox	Married	No			3	3	3	No
Orthodox	Married	No			2	2	2	Yes
Orthodox	Widowed				4	4	4	No
Orthodox	Married	No			1	1	1	No
Orthodox	Married	Yes	Yes	5th	3	3	3	No
Orthodox	Married	Yes	Yes	7th	2	2	2	No
Orthodox	Married	Yes	Yes	5th	3	3	3	No
Orthodox	Married	Yes	Yes	5th	2	2	2	No
Orthodox	Divorced				2	3	3	No
Orthodox	Married	No			2	2	2	No
Orthodox	Married	No			3	3	3	No
Orthodox	Married	Yes	Yes	6th	3	3	3	No
Orthodox	Married	No			2	2	2	No
Orthodox	Married	Yes	Yes	10th	1	1	1	No
Orthodox	Married	No			4	4	4	No
Orthodox	Married	Yes	Yes	6th	5	5	5	No
Orthodox	Married	No			2	2	2	No
Orthodox	Married	No			4	4	4	No
Orthodox	Married	Yes	Yes	5th	1	1	1	No
Orthodox	Married	No			7	7	7	No
Orthodox	Married	Yes	Yes	3rd	3	3	3	No
Orthodox	Married	No			5	5	5	No
Orthodox	Married	Yes	Yes	10th	5	5	5	No
Orthodox	Married	No			3	3	3	No

For peer review only

q215	q217	q220	q221	q222	q223	q224	q225	q226
1	Protected S	No		Pit Latrine/	Finished ro	Rambo/wo	1	Yes
1	Protected S	No		Pit Latrine/	Finished ro	Rambo/wo	0	Yes
	Open Sprin	No		Pit Latrine/	Finished ro	Rambo/wo	1	Yes
	Protected S	No		Pit Latrine/	Finished ro	Rambo/wo	3	Yes
	Protected S	No		Pit Latrine/	Finished ro	Rambo/wo	1	Yes
	Protected H	No		No facility/	Finished ro	Rambo/wo	0	Yes
1	Protected S	No		Pit Latrine/	Finished ro	Rambo/wo	1	Yes
	Protected S	No		Pit Latrine/	Finished ro	Rambo/wo	2	No
	Protected S	No		Pit Latrine/	Finished ro	Rambo/wo	0	Yes
	Protected H	No		No facility/	Finished ro	Rambo/wo	0	Yes
	Protected H	No		Pit Latrine/	Finished ro	Rambo/wo	1	Yes
	Protected S	No		Pit Latrine/	Finished ro	Rambo/wo	1	Yes
	Protected S	No		Pit Latrine/	Finished ro	Rambo/wo	0	No
	Protected S	No		Pit Latrine/	Finished ro	Rambo/wo	1	Yes
2	Protected H	No		No facility/	Thatch/lea	Rambo/wo	0	Yes
	Protected S	No		Pit Latrine/	Finished ro	Rambo/wo	0	Yes
1	Protected H	No		Pit Latrine/	Finished ro	Rambo/wo	3	Yes
	Protected S	No		Pit Latrine/	Finished ro	Rambo/wo	1	Yes
	Protected S	No		Pit Latrine/	Finished ro	Rambo/wo	0	Yes
	Protected H	No		Pit Latrine/	Finished ro	Rambo/wo	1	No
	Open Sprin	No		No facility/	Finished ro	Rambo/wo	3	No
	Open Sprin	No		Pit Latrine/	Finished ro	Rambo/wo	0	No
	Protected S	No		Pit Latrine/	Finished ro	Rambo/wo	1	Yes
2	Protected S	No		Pit Latrine/	Finished ro	Rambo/wo	3	Yes
	Protected S	No		No facility/	Finished ro	Rambo/wo	1	Yes
	Protected S	No		No facility/	Finished ro	Rambo/wo	0	Yes
	Protected H	No		Pit Latrine/	Finished ro	Rambo/wo	0	Yes
	Protected H	No		No facility/	Finished ro	Rambo/wo	0	Yes
	Protected S	No		Pit Latrine/	Finished ro	Rambo/wo	0	Yes
	Protected S	No		Pit Latrine/	Finished ro	Rambo/wo	3	Yes
	Protected S	No		Pit Latrine/	Finished ro	Rambo/wo	1	Yes
	Protected S	No		Pit Latrine/	Finished ro	Rambo/wo	0	Yes
	Protected S	No		Pit Latrine/	Finished ro	Rambo/wo	0	Yes
	Protected S	No		No facility/	Finished ro	Rambo/wo	3	Yes
	Protected H	No		Pit Latrine/	Finished ro	Rambo/wo	0	Yes
	Protected H	No		No facility/	Finished ro	Rambo/wo	3	Yes
	Protected S	No		Pit Latrine/	Finished ro	Rambo/wo	0	Yes
	Protected S	No		Pit Latrine/	Finished ro	Rambo/wo	1	Yes
	Open Sprin	No		No facility/	Finished ro	Rambo/wo	1	Yes
	Protected H	No		Pit Latrine/	Finished ro	Rambo/wo	1	Yes
1	Open Sprin	No		Pit Latrine/	Finished ro	Rambo/wo	3	Yes
	Protected H	No		Pit Latrine/	Finished ro	Rambo/wo	1	Yes
	Open Sprin	No		Pit Latrine/	Finished ro	Rambo/wo	0	Yes
4	Protected S	No		Pit Latrine/	Finished ro	Rambo/wo	3	Yes
	Protected S	No		Pit Latrine/	Finished ro	Stone with	1	Yes
	Protected S	No		Pit Latrine/	Finished ro	Rambo/wo	3	Yes
	Protected S	No		Pit Latrine/	Finished ro	Rambo/wo	0	Yes
	Protected H	No		No facility/	Finished ro	Rambo/wo	0	Yes
	Protected S	No		Pit Latrine/	Finished ro	Rambo/wo	0	Yes
	Protected S	No		Pit Latrine/	Finished ro	Rambo/wo	1	Yes
	Protected S	No		Pit Latrine/	Finished ro	Rambo/wo	2	Yes

For peer review only

1	Protected S	No	No facility/ Finished ro	Rambo/wo	3	Yes
	Protected S	No	Pit Latrine/ Finished ro	Rambo/wo	0	Yes
	Protected H	No	Pit Latrine/ Finished ro	Rambo/wo	1	No
	Open Sprin	No	Pit Latrine/ Finished ro	Rambo/wo	1	Yes
	Protected S	No	Pit Latrine/ Finished ro	Rambo/wo	0	Yes
	Protected S	No	No facility/ Finished ro	Rambo/wo	1	Yes
1	Open Sprin	No	No facility/ Finished ro	Rambo/wo	0	Yes
	Protected H	No	Pit Latrine/ Finished ro	Rambo/wo	3	Yes
	Protected S	No	Pit Latrine/ Finished ro	Rambo/wo	0	Yes
	Protected S	No	Pit Latrine/ Finished ro	Rambo/wo	0	Yes
	Protected H	No	Pit Latrine/ Finished ro	Rambo/wo	0	Yes
1	Protected S	No	Pit Latrine/ Finished ro	Rambo/wo	0	No
	Protected S	No	Pit Latrine/ Finished ro	Rambo/wo	1	Yes
1	Protected H	No	Pit Latrine/ Finished ro	Rambo/wo	1	Yes
1	Protected H	No	Pit Latrine/ Finished ro	Rambo/wo	1	No
	Protected S	No	Pit Latrine/ Finished ro	Rambo/wo	0	Yes
	Protected S	No	Pit Latrine/ Finished ro	Rambo/wo	0	Yes
	Protected S	No	Pit Latrine/ Finished ro	Rambo/wo	3	Yes
	Protected H	No	No facility/ Finished ro	Rambo/wo	0	Yes
	Protected H	No	Pit Latrine/ Finished ro	Rambo/wo	1	Yes
	Protected S	No	No facility/ Finished ro	Rambo/wo	0	No
	Protected S	No	Pit Latrine/ Finished ro	Rambo/wo	0	Yes
	Open Sprin	No	Pit Latrine/ Finished ro	Rambo/wo	1	Yes
	Protected H	No	No facility/ Finished ro	Rambo/wo	3	No
	Protected S	No	Pit Latrine/ Finished ro	Rambo/wo	0	Yes
	Protected S	No	Pit Latrine/ Finished ro	Rambo/wo	1	Yes
	Protected H	No	Pit Latrine/ Finished ro	Rambo/wo	0	Yes
1	Protected H	No	Pit Latrine/ Finished ro	Rambo/wo	1	Yes
	Protected S	No	No facility/ Finished ro	Rambo/wo	3	Yes
1	Open Sprin	No	Pit Latrine/ Finished ro	Rambo/wo	2	Yes
	Protected S	No	Pit Latrine/ Finished ro	Rambo/wo	0	Yes
	Protected S	No	Pit Latrine/ Finished ro	Rambo/wo	3	Yes
	Protected H	No	Pit Latrine/ Finished ro	Rambo/wo	0	No
	Protected S	No	Pit Latrine/ Finished ro	Rambo/wo	3	Yes
	Protected H	No	Pit Latrine/ Finished ro	Rambo/wo	3	Yes
	Open Sprin	No	Pit Latrine/ Finished ro	Rambo/wo	0	Yes
	Protected S	No	No facility/ Finished ro	Rambo/wo	2	Yes
	Protected S	No	Pit Latrine/ Finished ro	Rambo/wo	0	Yes
	Open Sprin	No	No facility/ Finished ro	Rambo/wo	0	Yes
	Protected S	No	Pit Latrine/ Finished ro	Rambo/wo	3	Yes
	Protected S	No	No facility/ Finished ro	Rambo/wo	1	Yes
	Protected H	No	No facility/ Finished ro	Rambo/wo	1	Yes
	Protected H	No	Pit Latrine/ Finished ro	Rambo/wo	3	Yes
1	Open Sprin	No	Pit Latrine/ Finished ro	Rambo/wo	1	Yes
	Protected S	No	Pit Latrine/ Finished ro	Rambo/wo	3	Yes
	Protected S	No	Pit Latrine/ Finished ro	Rambo/wo	1	Yes
	Protected H	No	Pit Latrine/ Finished ro	Rambo/wo	1	No
	Open Sprin	No	Pit Latrine/ Finished ro	Rambo/wo	2	No
	Protected S	No	Pit Latrine/ Finished ro	Rambo/wo	3	Yes
	Protected S	No	No facility/ Finished ro	Rambo/wo	2	No
	Protected S	No	Pit Latrine/ Finished ro	Rambo/wo	3	No
	Protected H	No	Pit Latrine/ Finished ro	Rambo/wo	3	Yes

For peer review only

	Protected S	No		Pit Latrine/Finished ro	Rambo/wo	0	No
	Protected I	No		Pit Latrine/Finished ro	Rambo/wo	0	Yes
	Open Sprin	No		Pit Latrine/Finished ro	Rambo/wo	0	Yes
3	Protected I	No		Pit Latrine/Finished ro	Rambo/wo	1	No
	Protected I	No		No facility/Finished ro	Rambo/wo	0	Yes
1	Protected S	No		Pit Latrine/Finished ro	Rambo/wo	2	Yes
	Protected I	No		Pit Latrine/Finished ro	Rambo/wo	3	No
	Open Sprin	No		Pit Latrine/Finished ro	Rambo/wo	3	Yes
	Protected S	No		Pit Latrine/Finished ro	Rambo/wo	3	Yes
	Open Sprin	No		Pit Latrine/Finished ro	Rambo/wo	1	Yes
	Protected S	No		Pit Latrine/Finished ro	Rambo/wo	1	Yes
	Protected S	No		Pit Latrine/Finished ro	Rambo/wo	0	Yes
	Protected S	No		Pit Latrine/Finished ro	Rambo/wo	1	No
	Protected S	No		No facility/Finished ro	Rambo/wo	0	No
	Protected S	No		Pit Latrine/Finished ro	Rambo/wo	1	Yes
	Protected S	No		Pit Latrine/Finished ro	Rambo/wo	0	Yes
	Protected S	No		Pit Latrine/Finished ro	Rambo/wo	1	Yes
	Protected S	No		Pit Latrine/Finished ro	Rambo/wo	1	Yes
	Protected S	No		No facility/Finished ro	Rambo/wo	0	Yes
	Protected S	No		Pit Latrine/Finished ro	Rambo/wo	3	Yes
	Protected I	No		Pit Latrine/Finished ro	Rambo/wo	0	Yes
	Protected S	No		Pit Latrine/Finished ro	Rambo/wo	1	Yes
	Open Sprin	No		Pit Latrine/Finished ro	Rambo/wo	3	Yes
	Protected S	No		Pit Latrine/Finished ro	Rambo/wo	1	No
	Protected I	No		Pit Latrine/Finished ro	Rambo/wo	0	Yes
	Protected I	No		Pit Latrine/Finished ro	Rambo/wo	0	Yes
	River	No		Pit Latrine/Finished ro	Rambo/wo	0	Yes
	Open Sprin	No		No facility/Finished ro	Rambo/wo	2	Yes
	Open Sprin	No		Pit Latrine/Finished ro	Rambo/wo	1	Yes
	Protected S	No		Pit Latrine/Finished ro	Rambo/wo	1	Yes
	River	No		Pit Latrine/Finished ro	Rambo/wo	1	Yes
	Protected I	No		Pit Latrine/Finished ro	Stone with	0	No
	River	No		Pit Latrine/Finished ro	Rambo/wo	0	Yes
	Open Sprin	No		Pit Latrine/Finished ro	Rambo/wo	1	Yes
1	River	No		Pit Latrine/Finished ro	Rambo/wo	0	Yes
	River	No		Pit Latrine/Finished ro	Rambo/wo	0	Yes
	River	No		Pit Latrine/Finished ro	Rambo/wo	1	Yes
	Open Sprin	No		Pit Latrine/Finished ro	Rambo/wo	1	Yes
1	Open Sprin	No		Pit Latrine/Finished ro	Rambo/wo	0	No
2	Protected S	No		Pit Latrine/Finished ro	Rambo/wo	1	Yes
1	Open Sprin	No		No facility/Finished ro	Rambo/wo	2	Yes
	River	No		Pit Latrine/Finished ro	Rambo/wo	1	Yes
1	River	Yes	Add bleach	Pit Latrine/Finished ro	Rambo/wo	3	Yes
	River	No		Pit Latrine/Finished ro	Rambo/wo	1	Yes
	River	No		Pit Latrine/Finished ro	Rambo/wo	0	Yes
	Protected I	No		Pit Latrine/Finished ro	Rambo/wo	1	Yes
	River	No		No facility/Finished ro	Rambo/wo	0	Yes
	Open Sprin	No		Pit Latrine/Finished ro	Rambo/wo	1	No
	Protected I	No		No facility/Finished ro	Rambo/wo	1	Yes
	River	No		Pit Latrine/Finished ro	Rambo/wo	0	Yes
	Protected I	No		Pit Latrine/Finished ro	Rambo/wo	0	Yes
	Protected I	No		No facility/Finished ro	Rambo/wo	1	No

For peer review only

	Open Sprin	No		Pit Latrine/Finished ro	Rambo/wo	0	Yes
1	Protected h	Yes	Add bleach	Pit Latrine/Finished ro	Rambo/wo	1	Yes
	Protected S	No		No facility/ Wood plan	Rambo/wo	0	Yes
	Open Sprin	Yes	Add bleach	Pit Latrine/Finished ro	Rambo/wo	1	Yes
	Protected h	Yes	Add bleach	Pit Latrine/Finished ro	Rambo/wo	1	Yes
	Protected S	No		Pit Latrine/Finished ro	Rambo/wo	1	Yes
1	Protected h	No		Pit Latrine/Finished ro	Rambo/wo	1	Yes
	Open Sprin	No		Pit Latrine/Finished ro	Rambo/wo	1	Yes
	Protected h	No		Pit Latrine/Finished ro	Rambo/wo	1	Yes
1	Open Sprin	No		No facility/ Finished ro	Rambo/wo	1	Yes
1	Open Sprin	No		Pit Latrine/Finished ro	Rambo/wo	1	Yes
	Protected S	No		Pit Latrine/Finished ro	Rambo/wo	1	Yes
1	Open Sprin	No		Pit Latrine/Finished ro	Rambo/wo	1	Yes
	River	No		No facility/ Finished ro	Rambo/wo	1	No
	Protected S	No		Pit Latrine/Finished ro	Rambo/wo	0	Yes
	River	No		No facility/ Finished ro	Rambo/wo	1	Yes
	Open Sprin	No		Pit Latrine/Finished ro	Stone with	1	Yes
	Open Sprin	No		Pit Latrine/Finished ro	Rambo/wo	1	No
	Open Sprin	No		Pit Latrine/Finished ro	Rambo/wo	1	Yes
	Protected S	No		Pit Latrine/Finished ro	Rambo/wo	0	Yes
	River	No		Pit Latrine/Finished ro	Rambo/wo	1	Yes
	River	No		Pit Latrine/Finished ro	Rambo/wo	1	Yes
1	River	No		Pit Latrine/Finished ro	Rambo/wo	0	Yes
	River	No		Pit Latrine/Finished ro	Rambo/wo	1	Yes
3	River	No		Pit Latrine/Finished ro	Stone with	9	No
	River	No		Pit Latrine/Finished ro	Rambo/wo	0	Yes
	Protected S	No		Pit Latrine/Finished ro	Rambo/wo	0	No
	Protected S	No		Pit Latrine/Finished ro	Rambo/wo	0	No
	River	No		Pit Latrine/Finished ro	Rambo/wo	1	No
	Open Sprin	No		Pit Latrine/Finished ro	Rambo/wo	1	No
1	Open Sprin	No		No facility/ Finished ro	Rambo/wo	3	Yes
1	Protected S	No		Pit Latrine/Finished ro	Rambo/wo	3	No
	Open Sprin	No		Pit Latrine/Finished ro	Rambo/wo	1	No
	Open Sprin	Yes	Other	Pit Latrine/Finished ro	Rambo/wo	0	Yes
	River	No		No facility/ Finished ro	Rambo/wo	0	Yes
3	River	No		No facility/ Finished ro	Rambo/wo	1	No
3	Protected h	No		Pit Latrine/Finished ro	Rambo/wo	0	Yes
1	River	No		Pit Latrine/Finished ro	Rambo/wo	1	Yes
	River	No		No facility/ Finished ro	Rambo/wo	0	Yes
	River	No		Pit Latrine/Finished ro	Rambo/wo	1	Yes
	River	No		No facility/ Finished ro	Rambo/wo	1	Yes
	Open Sprin	No		Pit Latrine/Finished ro	Rambo/wo	1	No
	Open Sprin	No		Pit Latrine/Finished ro	Rambo/wo	1	No
	Open Sprin	No		Pit Latrine/Finished ro	Rambo/wo	0	Yes
	Protected h	No		Pit Latrine/Finished ro	Stone with	2	No
	River	No		Pit Latrine/Finished ro	Stone with	1	Yes
	River	No		Pit Latrine/Finished ro	Rambo/wo	0	Yes
	River	No		Pit Latrine/Finished ro	Rambo/wo	1	Yes
	Open Sprin	No		Pit Latrine/Finished ro	Rambo/wo	1	No
	Protected h	No		Pit Latrine/Finished ro	Rambo/wo	1	Yes
	Protected h	No		Pit Latrine/Finished ro	Rambo/wo	0	Yes
	Protected S	No		No facility/ Finished ro	Rambo/wo	0	Yes

For peer review only

	River	No		No facility/ Finished ro	Rambo/wo	1	No
	Protected S	No		No facility/ Finished ro	Rambo/wo	1	Yes
	Protected I	No		Pit Latrine/ Finished ro	Rambo/wo	1	Yes
	Protected I	No		Pit Latrine/ Finished ro	Rambo/wo	1	Yes
1	Protected I	No		No facility/ Finished ro	Rambo/wo	1	Yes
	Protected I	No		Pit Latrine/ Finished ro	Rambo/wo	1	No
	Protected I	No		Pit Latrine/ Finished ro	Rambo/wo	1	Yes
	Open Sprin	No		Pit Latrine/ Finished ro	Rambo/wo	1	No
	Protected I	No		Pit Latrine/ Finished ro	Rambo/wo	1	Yes
	Protected I	No		Pit Latrine/ Finished ro	Stone with	1	Yes
	Covered W	No		Pit Latrine/ Finished ro	Rambo/wo	1	Yes
	Protected I	No		Pit Latrine/ Finished ro	Rambo/wo	1	Yes
	Protected I	No		Pit Latrine/ Finished ro	Rambo/wo	1	No
	Protected I	Yes	Add bleach	No facility/ Finished ro	Rambo/wo	1	Yes
	Open Sprin	No		Pit Latrine/ Finished ro	No walls	1	Yes
	Protected I	No		Pit Latrine/ Finished ro	Rambo/wo	1	No
	Protected I	Yes	Add bleach	Pit Latrine/ Finished ro	Rambo/wo	1	Yes
1	Protected I	Yes	Add bleach	No facility/ Finished ro	Rambo/wo	1	Yes
	River	No		Pit Latrine/ Finished ro	Rambo/wo	0	Yes
	Protected I	No		No facility/ Finished ro	Rambo/wo	1	Yes
1	Protected I	Yes	Add bleach	Pit Latrine/ Finished ro	Rambo/wo	1	No
	Protected I	No		No facility/ Finished ro	Rambo/wo	0	Yes
	Protected I	Yes	Add bleach	Pit Latrine/ Finished ro	Rambo/wo	1	Yes
	Open Sprin	No		Pit Latrine/ Finished ro	Rambo/wo	1	Yes
	Protected I	No		Pit Latrine/ Finished ro	Rambo/wo	1	Yes
	Protected I	Yes	Add bleach	Pit Latrine/ Finished ro	Rambo/wo	1	Yes
	Protected I	Yes	Add bleach	Pit Latrine/ Finished ro	Rambo/wo	1	Yes
1	Protected I	Yes	Add bleach	Pit Latrine/ Finished ro	Rambo/wo	1	Yes
	Piped outsi	No		No facility/ Finished ro	Rambo/wo	0	Yes
	Protected I	No		Pit Latrine/ Finished ro	Rambo/wo	1	Yes
	Protected I	No		No facility/ Finished ro	Rambo/wo	0	No
	Protected I	No		Pit Latrine/ Finished ro	Rambo/wo	1	No
	Protected I	Yes	Add bleach	Pit Latrine/ Finished ro	Rambo/wo	1	No
	Protected I	Yes	Add bleach	Pit Latrine/ Finished ro	Rambo/wo	1	No
	Open Sprin	No		No facility/ Finished ro	Rambo/wo	1	No
	Protected I	No		Pit Latrine/ Finished ro	Rambo/wo	1	No
1	Protected I	Yes	Add bleach	Pit Latrine/ Finished ro	Rambo/wo	1	Yes
	Protected I	No		Pit Latrine/ Finished ro	Rambo/wo	0	Yes
	Protected I	No		No facility/ Finished ro	Rambo/wo	0	Yes
	Protected I	No		Pit Latrine/ Finished ro	Rambo/wo	1	No
	Protected I	No		No facility/ Finished ro	Rambo/wo	1	Yes
	Protected I	No		Pit Latrine/ Finished ro	Rambo/wo	1	Yes
	Protected I	No		Pit Latrine/ Finished ro	Rambo/wo	0	Yes
1	Protected I	No		No facility/ Finished ro	Rambo/wo	1	Yes
	Protected I	No		No facility/ Finished ro	Rambo/wo	1	Yes
	River	No		No facility/ Finished ro	Rambo/wo	0	Yes
	Protected I	No		Pit Latrine/ Finished ro	Rambo/wo	1	Yes
	Open Sprin	No		Pit Latrine/ Wood plan	Rambo/wo	1	Yes
	Protected I	Yes	Add bleach	Pit Latrine/ Finished ro	Rambo/wo	1	Yes
	Open Sprin	No		Pit Latrine/ Finished ro	Rambo/wo	1	Yes
4	Protected I	No		No facility/ Finished ro	Rambo/wo	1	Yes
	Covered W	No		Pit Latrine/ Finished ro	Rambo/wo	1	Yes

For peer review only

	Protected I	No		Pit Latrine/Finished ro	Rambo/wo	1	Yes
	Protected I	No		Pit Latrine/Finished ro	Rambo/wo	0	Yes
	Covered W	No		No facility/Finished ro	Rambo/wo	0	Yes
	Protected I	Yes	Add bleach	No facility/Finished ro	Rambo/wo	1	Yes
2	Protected I	No		No facility/Finished ro	Rambo/wo	0	Yes
	Protected I	No		Pit Latrine/Finished ro	Rambo/wo	1	Yes
1	Protected I	No		No facility/Finished ro	Rambo/wo	0	Yes
	Protected I	No		Pit Latrine/Finished ro	Rambo/wo	1	Yes
	Protected I	No		Pit Latrine/Finished ro	Rambo/wo	1	Yes
	Protected I	No		Pit Latrine/Finished ro	Rambo/wo	1	No
	Protected I	No		Pit Latrine/Finished ro	Rambo/wo	1	Yes
	Open Sprin	No		Pit Latrine/Finished ro	Rambo/wo	1	Yes
	Protected I	No		No facility/Finished ro	Rambo/wo	0	Yes
	Protected I	No		Pit Latrine/Finished ro	Rambo/wo	1	Yes
	Protected I	No		Pit Latrine/Finished ro	Rambo/wo	1	Yes
	River	No		Pit Latrine/Finished ro	Rambo/wo	0	Yes
	Protected I	No		No facility/Finished ro	Rambo/wo	0	Yes
	Protected I	No		No facility/Finished ro	Rambo/wo	0	Yes
	Protected I	Yes	Add bleach	Pit Latrine/Finished ro	Rambo/wo	1	Yes
	Protected I	No		Pit Latrine/Finished ro	Rambo/wo	1	No
	Protected I	No		No facility/Finished ro	Rambo/wo	0	Yes
	Protected I	No		Pit Latrine/Finished ro	Rambo/wo	1	No
	Protected I	No		Pit Latrine/Finished ro	Rambo/wo	1	No
	Protected I	No		Pit Latrine/Finished ro	Rambo/wo	1	No
	Protected I	No		Pit Latrine/Finished ro	Rambo/wo	1	Yes
1	Protected I	No		Pit Latrine/Finished ro	Rambo/wo	1	Yes
	Protected I	No		Pit Latrine/Finished ro	Rambo/wo	0	Yes
	Protected I	No		Pit Latrine/Finished ro	Rambo/wo	1	Yes
	Covered W	No		Pit Latrine/Finished ro	Rambo/wo	1	No
	Protected I	Yes	Add bleach	Pit Latrine/Finished ro	Rambo/wo	1	Yes
	Protected I	No		Pit Latrine/Finished ro	Rambo/wo	1	Yes
1	Protected I	No		Pit Latrine/Finished ro	Rambo/wo	1	Yes
	Protected I	No		Pit Latrine/Finished ro	Rambo/wo	1	Yes
	Protected I	No		No facility/Finished ro	Rambo/wo	0	Yes
	Protected I	Yes	Add bleach	Pit Latrine/Finished ro	Rambo/wo	1	Yes
	Protected I	No		Pit Latrine/Finished ro	Rambo/wo	1	Yes
	Protected I	No		No facility/Finished ro	Rambo/wo	1	Yes
	Protected I	No		No facility/Finished ro	Rambo/wo	0	Yes
	Protected I	Yes	Add bleach	Pit Latrine/Finished ro	Rambo/wo	1	Yes
	Protected I	No		Pit Latrine/Finished ro	Rambo/wo	1	No
1	Protected I	No		No facility/Finished ro	Rambo/wo	0	No
	Protected I	No		Pit Latrine/Finished ro	Rambo/wo	1	No
	Protected I	No		Pit Latrine/Finished ro	Rambo/wo	0	No
	Protected I	No		Pit Latrine/Finished ro	Rambo/wo	1	Yes
	Protected I	No		No facility/Finished ro	Rambo/wo	0	Yes
	Protected I	Yes	Do not Kno	Pit Latrine/Finished ro	Rambo/wo	1	Yes
1	Protected I	Yes	Add bleach	Pit Latrine/Finished ro	Rambo/wo	1	Yes
	Protected I	Yes	Add bleach	Pit Latrine/Finished ro	Rambo/wo	1	Yes
1	Protected I	No		Pit Latrine/Finished ro	Stone with	1	Yes
	Covered W	No		Pit Latrine/Finished ro	Rambo/wo	0	Yes
	Protected I	No		Pit Latrine/Finished ro	Rambo/wo	1	Yes
1	Protected I	Yes	Add bleach	No facility/Finished ro	Rambo/wo	0	No

For peer review only

	Protected	Yes	Add bleach	Pit Latrine/	Finished ro	Rambo/wo	1	No
	Protected	No		Pit Latrine/	Finished ro	Rambo/wo	1	Yes
	Protected	No		No facility/	Finished ro	Rambo/wo	0	Yes
8	Protected	Yes	Add bleach	Pit Latrine/	Finished ro	Rambo/wo	1	No
	Protected	No		Pit Latrine/	Finished ro	Rambo/wo	1	Yes
	Protected	No		Pit Latrine/	Finished ro	Rambo/wo	0	Yes
1	Open Sprin	Yes	Boil	Pit Latrine/	Finished ro	Rambo/wo	1	Yes
3	Protected	No		Pit Latrine/	Finished ro	Rambo/wo	1	Yes
	Protected	No		Pit Latrine/	Finished ro	Rambo/wo	1	Yes
	Protected	Yes	Add bleach	Pit Latrine/	Finished ro	Rambo/wo	1	Yes
	Open Sprin	No		No facility/	Finished ro	Rambo/wo	1	No
	Open Sprin	No		No facility/	Finished ro	Rambo/wo	0	No
	Protected	No		Pit Latrine/	Finished ro	Rambo/wo	1	Yes
	Protected	No		Pit Latrine/	Finished ro	Rambo/wo	0	No
	Protected	No		No facility/	Finished ro	Rambo/wo	0	Yes
	Protected	No		Pit Latrine/	Finished ro	Rambo/wo	1	Yes
	Protected	No		Pit Latrine/	Finished ro	Rambo/wo	1	Yes
	Protected	No		No facility/	Finished ro	Rambo/wo	1	Yes
1	Covered W	No		Pit Latrine/	Finished ro	Rambo/wo	1	Yes
	River	No		No facility/	Finished ro	Rambo/wo	0	Yes
	Protected	Yes	Add bleach	Pit Latrine/	Finished ro	Rambo/wo	1	Yes
	Protected	No		Pit Latrine/	Finished ro	Rambo/wo	1	Yes
	Protected	No		Pit Latrine/	Finished ro	Rambo/wo	0	Yes
	Protected	No		Pit Latrine/	Finished ro	Rambo/wo	1	Yes
	Protected	No		Pit Latrine/	Finished ro	Rambo/wo	1	Yes
	Protected	No		Pit Latrine/	Finished ro	Rambo/wo	1	Yes
	Protected	No		No facility/	Finished ro	Rambo/wo	0	Yes
	Protected	Yes	Add bleach	No facility/	Finished ro	Rambo/wo	1	No
1	Protected	No		Pit Latrine/	Finished ro	Rambo/wo	1	Yes
	Protected	No		Pit Latrine/	Finished ro	Rambo/wo	1	No
	Open Sprin	No		No facility/	Finished ro	Rambo/wo	0	Yes
	Open Sprin	No		No facility/	Finished ro	Rambo/wo	0	Yes
	Open Sprin	No		Pit Latrine/	Finished ro	Rambo/wo	0	No
	Open Sprin	No		No facility/	Finished ro	Stone with	0	No
	River	No		No facility/	Finished ro	Rambo/wo	0	Yes
	Piped outsi	No		Pit Latrine/	Finished ro	Rambo/wo	0	No
	River	No		Pit Latrine/	Finished ro	Rambo/wo	0	Yes
	Protected	No		Pit Latrine/	Finished ro	Rambo/wo	1	Yes
	Open Sprin	No		No facility/	Finished ro	Rambo/wo	0	Yes
	Open Sprin	No		No facility/	Finished ro	Rambo/wo	0	No
	Open Sprin	No		Pit Latrine/	Finished ro	Rambo/wo	0	No
	Open Sprin	No		No facility/	Finished ro	Rambo/wo	0	No
	Open Sprin	No		No facility/	Finished ro	Rambo/wo	0	No
	Open Sprin	No		Pit Latrine/	Finished ro	Rambo/wo	0	Yes
	Open Sprin	No		No facility/	Finished ro	Rambo/wo	0	Yes
	Open Sprin	No		No facility/	Finished ro	Rambo/wo	0	Yes
	Open Sprin	No		Pit Latrine/	Finished ro	Rambo/wo	0	Yes
	Pond/Lake,	No		No facility/	Thatch/lea	Rambo/wo	0	No
1	Protected	No		No facility/	Finished ro	Rambo/wo	0	Yes
	Open Sprin	No		Pit Latrine/	Finished ro	Rambo/wo	0	No
1	Open Sprin	No		Pit Latrine/	Finished ro	Rambo/wo	0	Yes
1	Protected	No		No facility/	Finished ro	Rambo/wo	1	Yes

For peer review only

	Open Sprin	No	No facility/	Finished ro	Rambo/wo	0	No
3	River	No	No facility/	Finished ro	Rambo/wo	0	No
	Open Sprin	No	No facility/	Finished ro	Rambo/wo	0	No
1	Piped outsi	No	Other	Finished ro	Rambo/wo	0	Yes
1	Open Sprin	No	No facility/	Finished ro	Rambo/wo	0	Yes
	Open Sprin	No	No facility/	Finished ro	Rambo/wo	0	No
	Piped outsi	No	Other	Finished ro	Rambo/wo	1	Yes
	River	No	No facility/	Finished ro	Rambo/wo	0	No
2	Protected I	No	Pit Latrine/	Finished ro	Rambo/wo	1	Yes
	Open Sprin	No	No facility/	Finished ro	Rambo/wo	0	Yes
	Open Sprin	No	No facility/	Finished ro	Rambo/wo	0	No
	Piped outsi	No	Pit Latrine/	Finished ro	Cement	0	Yes
	Open Sprin	No	No facility/	Finished ro	Rambo/wo	0	No
	Protected I	No	Pit Latrine/	Finished ro	Rambo/wo	1	Yes
	Open Sprin	No	No facility/	Finished ro	Rambo/wo	0	No
2	Protected S	No	No facility/	Finished ro	Rambo/wo	0	Yes
	Open Sprin	No	No facility/	Finished ro	Rambo/wo	0	Yes
1	Open Sprin	No	No facility/	Finished ro	Rambo/wo	0	No
	Open Sprin	No	No facility/	Finished ro	Rambo/wo	0	No
	Open Sprin	No	No facility/	Finished ro	Rambo/wo	0	Yes
	Piped outsi	No	Pit Latrine/	Finished ro	Rambo/wo	0	Yes
	Protected I	No	Pit Latrine/	Finished ro	Rambo/wo	0	Yes
	Protected S	No	Pit Latrine/	Finished ro	Rambo/wo	0	No
	Open Sprin	No	Pit Latrine/	Finished ro	Rambo/wo	0	Yes
	Protected S	No	Pit Latrine/	Finished ro	Rambo/wo	0	Yes
	Protected I	No	Pit Latrine/	Finished ro	Rambo/wo	0	No
	Protected I	No	No facility/	Finished ro	Rambo/wo	0	Yes
	Protected I	No	No facility/	Finished ro	Rambo/wo	0	No
	Protected I	No	Pit Latrine/	Finished ro	Rambo/wo	0	Yes
	Protected S	No	Pit Latrine/	Finished ro	Rambo/wo	0	Yes
	Piped outsi	No	Pit Latrine/	Finished ro	Rambo/wo	1	Yes
	Protected I	No	Pit Latrine/	Finished ro	Rambo/wo	0	Yes
	Protected S	No	No facility/	Finished ro	Rambo/wo	0	Yes
	Protected S	No	Pit Latrine/	Finished ro	Rambo/wo	0	Yes
	Piped outsi	No	No facility/	Finished ro	Rambo/wo	1	Yes
	Piped outsi	No	Pit Latrine/	Finished ro	Rambo/wo	0	Yes
3	Piped outsi	No	No facility/	Finished ro	Rambo/wo	1	Yes
	Protected S	No	Pit Latrine/	Finished ro	Rambo/wo	1	Yes
1	Open Sprin	No	Pit Latrine/	Finished ro	Rambo/wo	0	Yes
	Piped outsi	No	No facility/	Finished ro	Rambo/wo	0	Yes
	Protected I	No	No facility/	Finished ro	Rambo/wo	0	Yes
	River	No	No facility/	Finished ro	Rambo/wo	0	No
	Piped outsi	No	Pit Latrine/	Finished ro	Rambo/wo	1	Yes
1	River	No	Pit Latrine/	Finished ro	Rambo/wo	0	Yes
	Open Sprin	No	Pit Latrine/	Finished ro	Rambo/wo	1	Yes
	Protected I	No	Pit Latrine/	Finished ro	Rambo/wo	0	No
1	Covered W	No	Pit Latrine/	Finished ro	Rambo/wo	1	Yes
2	Protected S	No	Pit Latrine/	Finished ro	Rambo/wo	0	No
	Piped outsi	No	No facility/	Finished ro	Rambo/wo	1	Yes
	Protected S	No	No facility/	Finished ro	Rambo/wo	0	Yes
	Protected S	No	No facility/	Finished ro	Rambo/wo	0	No
	Protected S	No	Pit Latrine/	Finished ro	Rambo/wo	0	No

	Protected I	No		No facility/ Finished ro	Rambo/wo	0	No
1	Protected S	No		No facility/ Finished ro	Rambo/wo	0	Yes
	Piped outsi	No		Pit Latrine/ Finished ro	Rambo/wo	1	Yes
	Protected I	No		No facility/ Finished ro	Rambo/wo	0	No
	Piped outsi	No		No facility/ Finished ro	Rambo/wo	0	Yes
	Piped outsi	No		No facility/ Finished ro	Rambo/wo	1	Yes
	Piped outsi	No		Pit Latrine/ Finished ro	Rambo/wo	0	No
	Piped outsi	No		Pit Latrine/ Finished ro	Rambo/wo	1	Yes
	Protected S	No		Pit Latrine/ Finished ro	Rambo/wo	1	No
	Piped outsi	No		Pit Latrine/ Finished ro	Rambo/wo	1	No
	River	No		Pit Latrine/ Finished ro	Rambo/wo	1	Yes
	Open Sprin	No		No facility/ Finished ro	Rambo/wo	0	Yes
	River	No		Pit Latrine/ Finished ro	Rambo/wo	1	Yes
	Piped outsi	No		Pit Latrine/ Finished ro	Rambo/wo	1	Yes
	Open Sprin	No		Pit Latrine/ Finished ro	Rambo/wo	1	Yes
1	River	Yes	Boil	Pit Latrine/ Finished ro	Rambo/wo	1	Yes
	Open Sprin	No		No facility/ Finished ro	Rambo/wo	0	Yes
	Protected I	Yes	Add bleach	Pit Latrine/ Finished ro	Rambo/wo	0	Yes
	Open Sprin	No		No facility/ Finished ro	Rambo/wo	0	Yes
	Protected S	No		Pit Latrine/ Finished ro	Rambo/wo	1	Yes
1	River	Yes	Add bleach	Pit Latrine/ Finished ro	Rambo/wo	1	Yes
	Protected I	No		Pit Latrine/ Finished ro	Stone with	1	Yes
	Protected S	No		Pit Latrine/ Finished ro	Rambo/wo	1	No
	Protected I	Yes	Add bleach	Pit Latrine/ Finished ro	Rambo/wo	0	No
	Piped outsi	No		Pit Latrine/ Finished ro	Rambo/wo	1	Yes
	Protected S	No		Pit Latrine/ Finished ro	Rambo/wo	1	No
	Open Sprin	No		No facility/ Finished ro	Rambo/wo	0	Yes
1	Piped outsi	No		Pit Latrine/ Finished ro	Rambo/wo	1	No
	Open Sprin	No		Pit Latrine/ Finished ro	Rambo/wo	0	No
	Open Sprin	No		No facility/ Finished ro	Rambo/wo	0	Yes
	Protected I	No		Pit Latrine/ Finished ro	Rambo/wo	1	Yes
	River	No		Pit Latrine/ Finished ro	Rambo/wo	1	Yes
	Open Sprin	No		Pit Latrine/ Finished ro	Rambo/wo	1	Yes
	Protected I	No		Pit Latrine/ Finished ro	Rambo/wo	1	Yes
	Open Sprin	No		Pit Latrine/ Finished ro	Rambo/wo	1	Yes
	Protected S	No		No facility/ Finished ro	Rambo/wo	1	Yes
	Piped outsi	No		Pit Latrine/ Finished ro	Rambo/wo	0	Yes
	Protected I	No		Pit Latrine/ Finished ro	Rambo/wo	1	Yes
	Protected I	Yes	Add bleach	Pit Latrine/ Finished ro	Rambo/wo	0	Yes
1	Protected I	Yes	Add bleach	No facility/ Finished ro	Rambo/wo	0	Yes
	River	No		Pit Latrine/ Finished ro	Rambo/wo	0	Yes
	Open Sprin	No		No facility/ Finished ro	Rambo/wo	0	Yes
	Piped outsi	No		Pit Latrine/ Finished ro	Rambo/wo	0	No
	River	Yes	Boil	Pit Latrine/ Finished ro	Rambo/wo	1	Yes
2	Open Sprin	No		Pit Latrine/ Finished ro	Rambo/wo	1	Yes
	Protected I	Yes	Add bleach	No facility/ Finished ro	Rambo/wo	0	No
	River	Yes	Boil	Pit Latrine/ Finished ro	Rambo/wo	1	Yes
	Open Sprin	No		No facility/ Finished ro	Rambo/wo	0	Yes
	River	No		Pit Latrine/ Finished ro	Rambo/wo	0	No
2	River	No		Pit Latrine/ Finished ro	Rambo/wo	1	Yes
	River	No		Pit Latrine/ Finished ro	Rambo/wo	1	Yes
	River	No		Pit Latrine/ Finished ro	Rambo/wo	0	Yes

For peer review only

	Piped outsi	No		No facility/	Finished ro	Rambo/wo	0	Yes
	Protected I	No		Pit Latrine/	Finished ro	Rambo/wo	1	Yes
	River	No		Pit Latrine/	Finished ro	Rambo/wo	1	No
	River	No		No facility/	Finished ro	Rambo/wo	0	No
	Protected I	No		Pit Latrine/	Finished ro	Rambo/wo	1	No
	River	No		Pit Latrine/	Finished ro	Rambo/wo	1	Yes
	Piped outsi	No		Pit Latrine/	Finished ro	Rambo/wo	0	Yes
2	Open Sprin	No		Pit Latrine/	Finished ro	Rambo/wo	1	No
	Protected I	Yes	Add bleach	Pit Latrine/	Finished ro	Rambo/wo	0	Yes
	Open Sprin	No		Pit Latrine/	Finished ro	Rambo/wo	1	Yes
	Piped outsi	No		No facility/	Finished ro	Rambo/wo	1	No
	Protected I	Yes	Add bleach	No facility/	Finished ro	Rambo/wo	0	No
	Open Sprin	No		No facility/	Finished ro	Rambo/wo	0	No
	Piped outsi	No		Pit Latrine/	Finished ro	Rambo/wo	1	Yes
	Protected I	No		Pit Latrine/	Finished ro	Rambo/wo	1	Yes
	Open Sprin	No		Pit Latrine/	Finished ro	Rambo/wo	1	Yes
	Protected S	No		Pit Latrine/	Finished ro	Rambo/wo	1	Yes
	Piped outsi	No		Pit Latrine/	Finished ro	Rambo/wo	1	No
	Piped outsi	No		No facility/	Thatch/lea	Rambo/wo	0	Yes
	Piped outsi	No		Pit Latrine/	Finished ro	Cement	1	Yes
	Piped outsi	No		Pit Latrine/	Thatch/lea	Rambo/wo	0	Yes
1	Piped outsi	No		Pit Latrine/	Thatch/lea	Rambo/wo	0	No
	Piped outsi	No		No facility/	Thatch/lea	Rambo/wo	1	Yes
	Piped outsi	No		Pit Latrine/	Finished ro	Rambo/wo	2	No
	Piped outsi	No		Pit Latrine/	Thatch/lea	Rambo/wo	1	Yes
	Piped outsi	No		No facility/	Finished ro	Rambo/wo	1	No
	Piped outsi	No		Pit Latrine/	Thatch/lea	Rambo/wo	0	No
	Piped outsi	No		No facility/	Finished ro	Rambo/wo	1	Yes
	Piped outsi	No		No facility/	Thatch/lea	Cement	0	No
	Piped outsi	No		No facility/	Thatch/lea	Rambo/wo	0	No
	Piped outsi	No		No facility/	Thatch/lea	Rambo/wo	0	Yes
	Piped outsi	No		No facility/	Thatch/lea	Cement	0	Yes
	Piped outsi	No		Pit Latrine/	Finished ro	Rambo/wo	0	Yes
	Piped outsi	No		No facility/	Thatch/lea	Rambo/wo	1	Yes
	Piped outsi	No		No facility/	Finished ro	Rambo/wo	2	Yes
	Piped outsi	No		No facility/	Thatch/lea	Rambo/wo	1	Yes
	Piped outsi	No		Pit Latrine/	Finished ro	Rambo/wo	1	Yes
	Piped outsi	No		No facility/	Thatch/lea	Rambo/wo	0	Yes
	Piped outsi	No		Pit Latrine/	Thatch/lea	Cement	1	Yes
	Piped outsi	No		No facility/	Thatch/lea	Rambo/wo	0	Yes
	Piped outsi	No		No facility/	Finished ro	Rambo/wo	2	Yes
	Piped outsi	No		No facility/	Thatch/lea	Cement	0	Yes
	Piped outsi	No		No facility/	Thatch/lea	Rambo/wo	0	Yes
	Piped outsi	No		No facility/	Thatch/lea	Rambo/wo	0	Yes
	Piped outsi	No		No facility/	Thatch/lea	Rambo/wo	0	Yes
	Piped outsi	No		No facility/	Thatch/lea	Rambo/wo	0	Yes
	Piped outsi	No		No facility/	Thatch/lea	Cement	1	Yes
	Piped outsi	No		Pit Latrine/	Finished ro	Cement	1	Yes
	Piped outsi	No		Ventilated	Thatch/lea	Rambo/wo	0	No
	Piped outsi	No		No facility/	Thatch/lea	Rambo/wo	0	Yes
	Piped outsi	No		No facility/	Finished ro	Rambo/wo	1	Yes
1	Piped outsi	No		Pit Latrine/	Thatch/lea	Rambo/wo	0	Yes

	Piped outsi	No		No facility/Thatch/lea	Cement	0	Yes
	Piped outsi	No		No facility/Thatch/lea	Rambo/wo	0	Yes
	Piped outsi	No		No facility/Thatch/lea	Rambo/wo	0	Yes
	Piped outsi	No		Pit Latrine/Thatch/lea	Cement	1	Yes
	Piped outsi	No		Pit Latrine/Thatch/lea	Rambo/wo	0	Yes
	Piped outsi	No		Pit Latrine/Thatch/lea	Rambo/wo	0	Yes
	Piped outsi	No		No facility/Finished ro	Cement	0	Yes
	Piped outsi	No		No facility/Thatch/lea	Rambo/wo	0	No
	Piped outsi	No		Pit Latrine/Thatch/lea	Rambo/wo	0	Yes
	Piped outsi	No		Pit Latrine/Finished ro	Rambo/wo	1	Yes
	Piped outsi	No		Pit Latrine/Thatch/lea	Rambo/wo	1	Yes
	Piped outsi	No		No facility/Finished ro	Cement	2	Yes
	Piped outsi	No		No facility/Thatch/lea	Cement	0	Yes
	Piped outsi	No		Pit Latrine/Finished ro	Rambo/wo	2	No
	Piped outsi	No		Pit Latrine/Finished ro	Rambo/wo	1	Yes
	Piped outsi	No		No facility/Thatch/lea	Rambo/wo	0	No
	Piped outsi	No		No facility/Thatch/lea	Rambo/wo	0	Yes
	Piped outsi	No		Pit Latrine/Thatch/lea	Cement	1	Yes
1	Piped outsi	No		Pit Latrine/Thatch/lea	Rambo/wo	0	Yes
	Piped outsi	No		No facility/Thatch/lea	Cement	0	Yes
	Piped outsi	No		No facility/Thatch/lea	Rambo/wo	0	No
	Piped outsi	No		Pit Latrine/Finished ro	Rambo/wo	1	Yes
1	Piped outsi	Yes	Add bleach	Pit Latrine/Finished ro	Rambo/wo	1	No
	Open Sprin	No		Pit Latrine/Finished ro	Rambo/wo	0	Yes
	Piped outsi	Yes	Add bleach	Pit Latrine/Finished ro	Rambo/wo	1	No
	Protected I	No		Pit Latrine/Finished ro	Rambo/wo	0	No
	Protected S	No		No facility/Finished ro	Rambo/wo	0	Yes
	River	No		Pit Latrine/Finished ro	Rambo/wo	0	No
	Protected I	No		Pit Latrine/Finished ro	Rambo/wo	1	No
	Protected I	No		Pit Latrine/Finished ro	Rambo/wo	1	No
	Piped outsi	Yes	Add bleach	Pit Latrine/Finished ro	Rambo/wo	1	No
	Protected S	No		Pit Latrine/Finished ro	Rambo/wo	0	No
	Protected I	No		Pit Latrine/Finished ro	Rambo/wo	1	Yes
	Protected S	No		Pit Latrine/Finished ro	Rambo/wo	1	Yes
	Piped outsi	No		No facility/Finished ro	Rambo/wo	0	Yes
	Protected I	No		Pit Latrine/Finished ro	Rambo/wo	1	Yes
	Piped outsi	No		Pit Latrine/Finished ro	Rambo/wo	0	Yes
	River	No		Pit Latrine/Finished ro	Rambo/wo	1	No
	Piped outsi	No		No facility/Finished ro	Rambo/wo	1	Yes
	Protected S	No		No facility/Finished ro	Rambo/wo	1	No
	Piped outsi	No		Pit Latrine/Finished ro	Rambo/wo	0	No
	River	No		Pit Latrine/Finished ro	Rambo/wo	1	Yes
	Protected S	No		Pit Latrine/Finished ro	Rambo/wo	1	Yes
	Protected I	No		Pit Latrine/Finished ro	Rambo/wo	1	No
	Protected S	No		No facility/Finished ro	Rambo/wo	1	No
	Piped outsi	No		Pit Latrine/Finished ro	Rambo/wo	3	Yes
	Piped outsi	Yes	Add bleach	Pit Latrine/Finished ro	Rambo/wo	1	No
1	Piped outsi	No		Pit Latrine/Finished ro	Rambo/wo	0	No
	Protected I	No		Pit Latrine/Finished ro	Rambo/wo	1	Yes
	Piped into (	No		Pit Latrine/Finished ro	Rambo/wo	1	Yes
	Protected S	No		No facility/Finished ro	Rambo/wo	1	No
	Piped outsi	No		No facility/Finished ro	Rambo/wo	0	Yes

For peer review only

	Protected I	No		Pit Latrine/Finished ro	Rambo/wo	1	Yes
	Piped outsi	Yes	Add bleach	No facility/Finished ro	Rambo/wo	1	Yes
	Protected S	No		No facility/Finished ro	Rambo/wo	0	No
	Piped outsi	No		Pit Latrine/Finished ro	Rambo/wo	1	No
	River	No		Pit Latrine/Finished ro	Rambo/wo	0	Yes
3	Piped into	No		Pit Latrine/Finished ro	Stone with	1	Yes
	Protected I	No		Pit Latrine/Finished ro	Rambo/wo	1	Yes
	Piped into	No		Pit Latrine/Finished ro	Rambo/wo	1	No
	Protected S	No		Pit Latrine/Finished ro	Rambo/wo	1	No
	Piped into	No		No facility/Finished ro	Rambo/wo	1	No
	Protected S	No		Pit Latrine/Finished ro	Rambo/wo	0	Yes
	Piped outsi	Yes	Add bleach	No facility/Finished ro	Rambo/wo	1	No
1	Piped outsi	No		Pit Latrine/Finished ro	Rambo/wo	0	No
	Protected S	No		Pit Latrine/Finished ro	Rambo/wo	0	Yes
	Piped outsi	No		Pit Latrine/Finished ro	Rambo/wo	1	No
1	Piped outsi	Yes	Add bleach	Pit Latrine/Finished ro	Rambo/wo	1	No
	Piped outsi	No		No facility/Finished ro	Rambo/wo	0	No
1	Piped outsi	No		Pit Latrine/Finished ro	Rambo/wo	0	No
	Protected I	No		Pit Latrine/Finished ro	Rambo/wo	0	Yes
	Protected I	No		Pit Latrine/Finished ro	Rambo/wo	1	Yes
1	Piped outsi	No		Pit Latrine/Finished ro	Rambo/wo	0	No
1	Piped into	No		Pit Latrine/Finished ro	Rambo/wo	1	Yes
	Protected S	No		No facility/Finished ro	Rambo/wo	1	No
	Piped outsi	No		Pit Latrine/Finished ro	Rambo/wo	0	No
	Protected S	No		Pit Latrine/Finished ro	Rambo/wo	0	Yes
1	Protected S	No		No facility/Finished ro	Rambo/wo	1	No
	River	No		No facility/Finished ro	Rambo/wo	0	Yes
	Piped outsi	No		Pit Latrine/Finished ro	Rambo/wo	1	Yes
	Open Sprin	No		Pit Latrine/Finished ro	Rambo/wo	1	Yes
3	Open Sprin	No		Pit Latrine/Finished ro	Rambo/wo	0	Yes
	Piped outsi	No		Pit Latrine/Finished ro	Rambo/wo	1	Yes
3	Piped outsi	No		Pit Latrine/Finished ro	Rambo/wo	1	Yes
	River	No		Pit Latrine/Finished ro	Rambo/wo	0	No
	Piped outsi	No		No facility/Finished ro	Rambo/wo	0	No
	Protected I	No		No facility/Finished ro	Rambo/wo	0	Yes
	Piped outsi	No		No facility/Finished ro	Rambo/wo	0	Yes
1	Protected I	No		Pit Latrine/Finished ro	Rambo/wo	1	Yes
	Piped outsi	Yes	Add bleach	Pit Latrine/Finished ro	Rambo/wo	1	No
	Piped into	No		Pit Latrine/Finished ro	Rambo/wo	1	No
1	Piped outsi	No		Pit Latrine/Finished ro	Rambo/wo	0	No
	Piped outsi	No		Pit Latrine/Finished ro	Rambo/wo	0	Yes
	Piped outsi	No		Pit Latrine/Finished ro	Rambo/wo	0	Yes
	Protected S	No		Pit Latrine/Finished ro	Rambo/wo	1	Yes
	Piped outsi	No		No facility/Finished ro	Rambo/wo	1	Yes
	River	No		No facility/Finished ro	Rambo/wo	0	No
	Protected I	No		Pit Latrine/Finished ro	Rambo/wo	1	No
	Piped outsi	Yes	Add bleach	Pit Latrine/Finished ro	Rambo/wo	1	Yes
	Protected I	No		Pit Latrine/Finished ro	Rambo/wo	1	Yes
1	Protected I	No		Pit Latrine/Finished ro	Rambo/wo	1	Yes
	Piped outsi	Yes	Add bleach	No facility/Finished ro	Rambo/wo	1	No
2	River	No		Pit Latrine/Finished ro	Rambo/wo	1	Yes
	River	No		Pit Latrine/Finished ro	Rambo/wo	1	No

For peer review only

	Covered W	No		Pit Latrine/	Finished ro	Rambo/wo	1	Yes
	Open Well	No		Pit Latrine/	Finished ro	Rambo/wo	0	No
1	Piped outsi	No		Pit Latrine/	Finished ro	Rambo/wo	0	No
	Piped into (	No		Pit Latrine/	Finished ro	Rambo/wo	0	Yes
	River	No		Pit Latrine/	Finished ro	Rambo/wo	0	Yes
	Piped outsi	No		Pit Latrine/	Finished ro	Rambo/wo	2	Yes
	Open Sprin	No		Pit Latrine/	Finished ro	Rambo/wo	0	Yes
	Open Well	No		No facility/	Finished ro	Rambo/wo	0	Yes
	Protected S	No		No facility/	Finished ro	Rambo/wo	0	Yes
	Piped into (	No		Pit Latrine/	Finished ro	Rambo/wo	1	No
	Piped outsi	No		No facility/	Finished ro	Rambo/wo	0	Yes
	Protected I	No		Pit Latrine/	Finished ro	Rambo/wo	1	Yes
	Piped outsi	No		Pit Latrine/	Finished ro	Rambo/wo	2	Yes
	Piped outsi	No		Pit Latrine/	Finished ro	Rambo/wo	1	No
	Protected I	No		Pit Latrine/	Finished ro	Rambo/wo	1	No
	Piped outsi	No		No facility/	Finished ro	Rambo/wo	1	No
1	Piped outsi	No		No facility/	Finished ro	Rambo/wo	1	Yes
	Piped outsi	No		Pit Latrine/	Finished ro	Rambo/wo	0	Yes
2	Protected S	No		No facility/	Finished ro	Rambo/wo	1	Yes
	Piped outsi	No		Pit Latrine/	Finished ro	Rambo/wo	1	Yes
	Protected I	No		No facility/	Finished ro	Rambo/wo	1	Yes
	Protected I	No		Pit Latrine/	Finished ro	Rambo/wo	1	Yes
	Protected I	No		No facility/	Finished ro	Rambo/wo	1	No
	Piped outsi	Yes	Add bleach	No facility/	Finished ro	Rambo/wo	1	No
1	Piped outsi	No		Pit Latrine/	Finished ro	Rambo/wo	1	Yes
	Piped outsi	No		Pit Latrine/	Finished ro	Rambo/wo	0	No
	Protected I	No		Pit Latrine/	Finished ro	Rambo/wo	0	Yes
	Piped into (	No		Pit Latrine/	Finished ro	Rambo/wo	1	No
	Piped outsi	No		Pit Latrine/	Finished ro	Rambo/wo	1	No
	Open Sprin	No		Pit Latrine/	Finished ro	Rambo/wo	0	No
1	Open Sprin	No		Other	Thatch/lea	Rambo/wo	0	Yes
	Protected I	No		Pit Latrine/	Finished ro	Rambo/wo	0	No
	Open Sprin	No		Pit Latrine/	Finished ro	Rambo/wo	2	Yes
	Piped outsi	No		Pit Latrine/	Finished ro	Rambo/wo	0	Yes
	Protected I	No		Pit Latrine/	Finished ro	Rambo/wo	0	No
4	Protected S	No		No facility/	Finished ro	Rambo/wo	0	Yes
	Open Sprin	No		Pit Latrine/	Finished ro	Rambo/wo	2	Yes
	Piped outsi	No		Pit Latrine/	Finished ro	Rambo/wo	0	No
	Open Sprin	No		Pit Latrine/	Finished ro	Rambo/wo	1	No
	Open Sprin	No		No facility/	Finished ro	Rambo/wo	2	No
	Protected I	No		No facility/	Finished ro	Rambo/wo	0	Yes
	Open Sprin	No		Pit Latrine/	Finished ro	Rambo/wo	1	Yes
	Open Sprin	No		Pit Latrine/	Finished ro	Rambo/wo	1	Yes
	River	No		No facility/	Finished ro	Rambo/wo	1	No
1	Piped outsi	No		Other	Finished ro	Rambo/wo	0	Yes
	Open Sprin	No		No facility/	Finished ro	Rambo/wo	2	Yes
	River	No		Pit Latrine/	Finished ro	Rambo/wo	0	Yes
	Open Sprin	No		Pit Latrine/	Finished ro	Rambo/wo	2	Yes
	Open Sprin	No		No facility/	Finished ro	Rambo/wo	2	Yes
	Piped outsi	No		Pit Latrine/	Finished ro	Rambo/wo	0	Yes
	Open Sprin	No		Pit Latrine/	Finished ro	Rambo/wo	2	Yes
	Protected I	No		Pit Latrine/	Finished ro	Rambo/wo	1	No

For peer review only

	Protected S	No	No facility/	Finished ro	Rambo/wo	1	Yes
1	Protected S	No	No facility/	Finished ro	Rambo/wo	0	No
	Protected I	No	Pit Latrine/	Finished ro	Rambo/wo	1	Yes
	Open Sprin	No	Pit Latrine/	Finished ro	Rambo/wo	1	No
3	Protected I	No	Pit Latrine/	Finished ro	Rambo/wo	2	Yes
	Open Sprin	No	Pit Latrine/	Finished ro	Rambo/wo	2	Yes
	Protected I	No	Pit Latrine/	Finished ro	Rambo/wo	1	Yes
	Protected I	No	Pit Latrine/	Finished ro	Rambo/wo	0	Yes
1	Piped outsi	No	Other	Finished ro	Rambo/wo	1	Yes
2	River	No	No facility/	Finished ro	Rambo/wo	0	No
	Open Sprin	No	No facility/	Finished ro	Rambo/wo	2	No
	Protected I	No	Pit Latrine/	Finished ro	Rambo/wo	1	Yes
	Open Sprin	No	No facility/	Finished ro	Rambo/wo	2	Yes
	Open Sprin	No	Pit Latrine/	Finished ro	Rambo/wo	0	Yes
	Protected I	No	Pit Latrine/	Finished ro	Rambo/wo	0	No
	Piped outsi	No	Pit Latrine/	Finished ro	Rambo/wo	0	Yes
	Open Sprin	No	No facility/	Finished ro	Rambo/wo	1	No
	Protected I	No	Pit Latrine/	Finished ro	Rambo/wo	1	Yes
	Piped outsi	No	Pit Latrine/	Finished ro	Rambo/wo	0	No
4	River	No	No facility/	Finished ro	Rambo/wo	1	Yes
1	Piped outsi	No	Other	Finished ro	Rambo/wo	0	Yes
	Protected I	No	Pit Latrine/	Finished ro	Rambo/wo	1	No
	Open Sprin	No	Pit Latrine/	Finished ro	Rambo/wo	0	Yes
	Protected I	No	Pit Latrine/	Finished ro	Rambo/wo	0	Yes
	Protected I	No	Pit Latrine/	Finished ro	Rambo/wo	0	No
	Piped outsi	No	Other	Finished ro	Rambo/wo	1	Yes
	River	No	Pit Latrine/	Finished ro	Rambo/wo	0	No
	Open Sprin	No	Pit Latrine/	Finished ro	Rambo/wo	1	Yes
	Open Sprin	No	Pit Latrine/	Finished ro	Rambo/wo	2	No
	Protected I	No	Pit Latrine/	Finished ro	Rambo/wo	1	No
	Protected S	No	No facility/	Finished ro	Rambo/wo	0	No
	Protected I	No	No facility/	Finished ro	Rambo/wo	0	No
	Protected S	No	No facility/	Finished ro	Rambo/wo	0	No
	Open Sprin	No	No facility/	Finished ro	Rambo/wo	2	Yes
	Protected S	No	Pit Latrine/	Finished ro	Rambo/wo	1	Yes
	Protected I	No	Pit Latrine/	Finished ro	Rambo/wo	0	No
	Open Sprin	No	No facility/	Finished ro	Rambo/wo	0	No
	Open Sprin	No	Pit Latrine/	Finished ro	Rambo/wo	1	Yes
	Protected I	No	Pit Latrine/	Finished ro	Rambo/wo	2	Yes
	River	No	No facility/	Finished ro	Rambo/wo	0	Yes
	Protected I	No	Pit Latrine/	Finished ro	Rambo/wo	0	Yes
	Open Sprin	No	Pit Latrine/	Finished ro	Rambo/wo	0	Yes
	Open Sprin	No	Pit Latrine/	Finished ro	Rambo/wo	0	No
	Open Well	No	Pit Latrine/	Finished ro	Rambo/wo	2	No
	Open Sprin	No	No facility/	Finished ro	Rambo/wo	0	Yes
	Protected S	No	No facility/	Finished ro	Rambo/wo	0	Yes
	Open Sprin	No	Pit Latrine/	Finished ro	Rambo/wo	2	No
	Open Sprin	No	Pit Latrine/	Finished ro	Rambo/wo	0	No
	River	No	Pit Latrine/	Finished ro	Rambo/wo	1	Yes
	Piped outsi	No	Pit Latrine/	Finished ro	Rambo/wo	1	No
	Protected I	No	No facility/	Finished ro	Rambo/wo	2	Yes
	Protected I	No	No facility/	Finished ro	Rambo/wo	0	Yes

For peer review only

	Covered W	No		Other	Finished ro	Rambo/wo	1	Yes
1	River	No		Pit Latrine/	Finished ro	Rambo/wo	0	No
	Protected I	No		Pit Latrine/	Finished ro	Rambo/wo	0	Yes
	Open Sprin	No		No facility/	Finished ro	Rambo/wo	2	Yes
	Protected I	No		Pit Latrine/	Finished ro	Rambo/wo	0	Yes
	River	No		No facility/	Finished ro	Rambo/wo	0	No
	Open Sprin	No		No facility/	Finished ro	Rambo/wo	2	No
1	River	No		No facility/	Finished ro	Rambo/wo	0	No
	Protected I	No		No facility/	Finished ro	Rambo/wo	1	Yes
1	Protected I	No		Pit Latrine/	Finished ro	Rambo/wo	1	Yes
	Open Sprin	No		Pit Latrine/	Finished ro	Rambo/wo	1	No
	River	No		Pit Latrine/	Finished ro	Rambo/wo	0	No
	Protected I	Yes	Add bleach	Pit Latrine/	Finished ro	Rambo/wo	0	No
1	Protected I	No		Pit Latrine/	Finished ro	Rambo/wo	0	Yes
	River	No		Pit Latrine/	Finished ro	Rambo/wo	1	Yes
	Protected I	No		Pit Latrine/	Finished ro	Rambo/wo	0	No
	Protected I	Yes	Add bleach	Pit Latrine/	Finished ro	Rambo/wo	1	Yes
	Protected S	No		Pit Latrine/	Finished ro	Rambo/wo	1	Yes
1	Piped outsi	No		Pit Latrine/	Finished ro	Rambo/wo	0	Yes
	Piped outsi	No		Pit Latrine/	Finished ro	Rambo/wo	1	Yes
	Protected I	No		Pit Latrine/	Finished ro	Rambo/wo	0	Yes
	Protected I	No		Pit Latrine/	Finished ro	Rambo/wo	1	Yes
	Protected S	No		No facility/	Finished ro	Rambo/wo	0	Yes
	Open Sprin	No		No facility/	Finished ro	Rambo/wo	2	Yes
	Protected S	No		Pit Latrine/	Finished ro	Rambo/wo	1	Yes
1	Piped outsi	No		Pit Latrine/	Finished ro	Rambo/wo	1	Yes
	Piped outsi	No		Pit Latrine/	Finished ro	Rambo/wo	0	Yes
	Protected I	No		No facility/	Finished ro	Rambo/wo	2	Yes
	River	No		No facility/	Finished ro	Rambo/wo	1	Yes
	Covered W	No		Pit Latrine/	Finished ro	Rambo/wo	0	No
	Piped outsi	No		Pit Latrine/	Finished ro	Rambo/wo	0	No
	River	No		No facility/	Finished ro	Stone with	0	Yes
	Protected S	No		Pit Latrine/	Finished ro	Rambo/wo	2	Yes
	Open Sprin	No		No facility/	Finished ro	Rambo/wo	1	No
	River	No		No facility/	Finished ro	Rambo/wo	0	No
	Open Sprin	No		No facility/	Finished ro	Rambo/wo	2	Yes
	Open Sprin	No		No facility/	Wood plan	Rambo/wo	2	Yes
	Protected I	No		No facility/	Finished ro	Rambo/wo	0	No
1	Open Sprin	No		Pit Latrine/	Finished ro	Rambo/wo	0	Yes
1	Protected I	No		Pit Latrine/	Finished ro	Rambo/wo	1	No
2	Open Well	No		Pit Latrine/	Finished ro	Rambo/wo	2	Yes
	Piped outsi	No		Pit Latrine/	Finished ro	Rambo/wo	1	No
	Open Sprin	No		No facility/	Finished ro	Rambo/wo	1	Yes
	Piped into (	No		Pit Latrine/	Finished ro	Rambo/wo	4	No
	Piped into (	No		Ventilated	Finished ro	Rambo/wo	1	No
	Piped outsi	No		No facility/	Finished ro	Rambo/wo	0	No
	Piped outsi	No		Ventilated	Finished ro	Rambo/wo	1	No
	Piped outsi	No		Ventilated	Finished ro	Rambo/wo	0	Yes
	Piped outsi	No		Pit Latrine/	Finished ro	Rambo/wo	1	No
	Piped outsi	No		Ventilated	Finished ro	Cement	1	Yes
	Piped into (	No		Ventilated	Finished ro	Rambo/wo	0	No
	Piped into (	No		Ventilated	Finished ro	Cement	0	No

For peer review only

	Piped into (	No	Ventilated	Finished ro	Cement	0	No
	Piped outsi	No	No facility/	Finished ro	Rambo/wo	0	Yes
	Piped outsi	No	No facility/	Finished ro	Rambo/wo	0	No
	Piped outsi	No	No facility/	Finished ro	Rambo/wo	1	Yes
	Piped into (	No	No facility/	Finished ro	Rambo/wo	1	Yes
	Piped into (	No	Ventilated	Finished ro	Rambo/wo	0	Yes
	Piped outsi	No	Pit Latrine/	Finished ro	Rambo/wo	1	No
	Piped into (	No	Pit Latrine/	Finished ro	Cement	1	Yes
1	Piped outsi	No	Pit Latrine/	Finished ro	Rambo/wo	3	Yes
	Piped into (	No	Ventilated	Finished ro	Cement	0	No
	Piped into (	No	No facility/	Finished ro	Rambo/wo	0	No
	Piped outsi	No	No facility/	Finished ro	Rambo/wo	1	No
	Piped outsi	No	Pit Latrine/	Finished ro	Rambo/wo	0	No
1	Piped outsi	No	Ventilated	Finished ro	Rambo/wo	0	No
	Piped into (	No	Ventilated	Finished ro	Rambo/wo	1	Yes
	Piped outsi	No	Ventilated	Finished ro	Cement	1	No
	Piped outsi	No	Pit Latrine/	Finished ro	Rambo/wo	3	Yes
	Piped into (	No	Pit Latrine/	Finished ro	Rambo/wo	0	No
	Piped outsi	No	Pit Latrine/	Finished ro	Rambo/wo	2	Yes
	Piped into (	No	Pit Latrine/	Finished ro	Rambo/wo	2	No
	Piped outsi	No	Pit Latrine/	Thatch/lea	Rambo/wo	0	No
	Piped outsi	No	Pit Latrine/	Finished ro	Rambo/wo	2	Yes
	Piped into (	No	Pit Latrine/	Finished ro	Rambo/wo	0	No
	Piped outsi	No	Pit Latrine/	Thatch/lea	Rambo/wo	0	Yes
	Piped outsi	No	Pit Latrine/	Finished ro	Rambo/wo	1	No
	Piped outsi	No	No facility/	Finished ro	Rambo/wo	0	No
	Piped outsi	No	Pit Latrine/	Finished ro	Rambo/wo	0	No
	Piped outsi	No	No facility/	Thatch/lea	Rambo/wo	0	Yes
	Piped outsi	No	Pit Latrine/	Finished ro	Cement	1	No
	Piped into (	No	Pit Latrine/	Finished ro	Cement	1	Yes
	Piped outsi	No	No facility/	Finished ro	Rambo/wo	1	No
	Piped outsi	No	No facility/	Thatch/lea	Rambo/wo	0	No
	Piped into (	No	Pit Latrine/	Finished ro	Rambo/wo	3	Yes
	Piped into (	No	Pit Latrine/	Finished ro	Rambo/wo	3	No
	Piped outsi	No	Pit Latrine/	Finished ro	Rambo/wo	0	No
	Piped into (	No	Pit Latrine/	Finished ro	Rambo/wo	0	No
	Piped outsi	No	Ventilated	Finished ro	Cement	0	No
	Piped outsi	No	Ventilated	Thatch/lea	Rambo/wo	0	No
	Piped into (	No	No facility/	Finished ro	Rambo/wo	2	Yes
	Piped outsi	No	No facility/	Finished ro	Rambo/wo	0	No
	Piped outsi	No	Pit Latrine/	Finished ro	Rambo/wo	0	Yes
	Piped outsi	No	No facility/	Finished ro	Rambo/wo	2	Yes
	Piped outsi	No	Ventilated	Finished ro	Cement	1	Yes
	Piped outsi	No	Pit Latrine/	Finished ro	Rambo/wo	3	Yes
1	Piped outsi	No	Ventilated	Finished ro	Rambo/wo	1	Yes
	Piped outsi	No	Pit Latrine/	Finished ro	Rambo/wo	0	Yes
	Piped outsi	No	Pit Latrine/	Finished ro	Rambo/wo	0	Yes
	Piped outsi	No	Pit Latrine/	Thatch/lea	Rambo/wo	0	No
	Piped into (	No	Pit Latrine/	Finished ro	Rambo/wo	2	Yes
	Piped into (	No	Pit Latrine/	Finished ro	Cement	1	No
	Piped into (	No	Ventilated	Finished ro	Rambo/wo	0	Yes
	Piped outsi	No	Pit Latrine/	Finished ro	Rambo/wo	3	Yes

For peer review only

	Piped outsi	No		Pit Latrine/	Finished ro	Rambo/wo	1	No
	Piped outsi	No		Pit Latrine/	Finished ro	Rambo/wo	0	Yes
	Piped into	No		Ventilated	Finished ro	Rambo/wo	0	No
1	Piped outsi	No		Pit Latrine/	Finished ro	Rambo/wo	2	Yes
	Piped into	No		Pit Latrine/	Finished ro	Rambo/wo	0	Yes
	Piped into	No		Pit Latrine/	Finished ro	Rambo/wo	0	No
	Piped outsi	No		Pit Latrine/	Thatch/lea	Rambo/wo	0	Yes
	Piped outsi	No		Ventilated	Finished ro	Cement	1	No
	Piped outsi	No		No facility/	Finished ro	Rambo/wo	0	No
	Piped outsi	No		Pit Latrine/	Finished ro	Rambo/wo	1	Yes
	Piped outsi	No		Pit Latrine/	Finished ro	Rambo/wo	0	Yes
	Piped into	No		Ventilated	Finished ro	Rambo/wo	2	No
	Piped outsi	No		Ventilated	Finished ro	Cement	0	No
	Piped into	No		Pit Latrine/	Finished ro	Rambo/wo	0	Yes
	Piped outsi	No		Ventilated	Finished ro	Rambo/wo	1	Yes
	Piped outsi	No		No facility/	Finished ro	Rambo/wo	3	Yes
	Piped into	No		Ventilated	Finished ro	Cement	1	Yes
	Piped outsi	No		No facility/	Finished ro	Rambo/wo	1	Yes
	Piped outsi	No		Pit Latrine/	Finished ro	Rambo/wo	1	Yes
	Piped into	No		Pit Latrine/	Finished ro	Rambo/wo	1	Yes
	Piped outsi	No		Pit Latrine/	Finished ro	Rambo/wo	1	No
	Piped outsi	No		Ventilated	Finished ro	Rambo/wo	1	Yes
	Piped into	No		Ventilated	Finished ro	Rambo/wo	1	Yes
	Piped into	No		Ventilated	Finished ro	Rambo/wo	0	Yes
	Protected S	No		Pit Latrine/	Finished ro	Rambo/wo	2	Yes
1	Covered W	No		Pit Latrine/	Finished ro	Rambo/wo	1	Yes
	Protected I	No		Pit Latrine/	Finished ro	Rambo/wo	2	Yes
	Covered W	Yes	Add bleach	Pit Latrine/	Finished ro	Rambo/wo	1	No
	Open Sprin	No		Pit Latrine/	Finished ro	Rambo/wo	1	Yes
	River	No		Pit Latrine/	Finished ro	Rambo/wo	0	No
	Protected S	No		Pit Latrine/	Finished ro	Rambo/wo	1	Yes
	Protected S	No		Pit Latrine/	Finished ro	Rambo/wo	2	Yes
2	Covered W	No		Pit Latrine/	Finished ro	Rambo/wo	1	Yes
	Covered W	No		Pit Latrine/	Finished ro	Rambo/wo	2	Yes
	River	No		No facility/	Finished ro	Rambo/wo	0	No
	Protected S	No		Pit Latrine/	Finished ro	Rambo/wo	0	Yes
	Protected S	No		Pit Latrine/	Finished ro	Rambo/wo	2	Yes
	River	No		Pit Latrine/	Thatch/lea	Rambo/wo	0	No
	Covered W	No		Pit Latrine/	Finished ro	Rambo/wo	0	No
	Protected S	No		No facility/	Finished ro	Rambo/wo	1	Yes
	Covered W	No		Pit Latrine/	Finished ro	Rambo/wo	1	Yes
	Covered W	No		Pit Latrine/	Finished ro	Rambo/wo	0	Yes
	Covered W	No		No facility/	Finished ro	Rambo/wo	0	No
	Protected I	No		Pit Latrine/	Finished ro	Rambo/wo	2	Yes
	River	No		No facility/	Finished ro	Rambo/wo	1	No
	Covered W	No		Pit Latrine/	Finished ro	Rambo/wo	1	No
	Covered W	Yes	Add bleach	Pit Latrine/	Finished ro	Rambo/wo	2	Yes
	Piped into	No		Pit Latrine/	Thatch/lea	Rambo/wo	1	Yes
	Protected S	No		No facility/	Finished ro	Rambo/wo	2	Yes
	Covered W	No		Pit Latrine/	Finished ro	Rambo/wo	1	No
	Open Sprin	No		Pit Latrine/	Finished ro	Rambo/wo	1	Yes
	Protected S	No		No facility/	Finished ro	Rambo/wo	0	Yes

For peer review only

1	Covered W	No		Pit Latrine/Finished ro	Rambo/wo	1	No
	Covered W	No		Pit Latrine/Thatch/lea	Rambo/wo	1	Yes
	Protected S	No		Pit Latrine/Finished ro	Rambo/wo	1	Yes
	River	No		No facility/Finished ro	Rambo/wo	1	No
	Covered W	No		Pit Latrine/Finished ro	Rambo/wo	0	Yes
	Protected I	No		Pit Latrine/Finished ro	Rambo/wo	1	Yes
	Protected S	No		No facility/Finished ro	Rambo/wo	1	Yes
1	Covered W	No		Pit Latrine/Finished ro	Rambo/wo	0	Yes
1	River	No		Pit Latrine/Finished ro	Rambo/wo	1	Yes
	Piped outsi	No		Pit Latrine/Finished ro	Rambo/wo	0	Yes
	Protected S	No		Pit Latrine/Finished ro	Rambo/wo	1	Yes
	Covered W	No		No facility/Finished ro	Rambo/wo	0	No
	River	No		Pit Latrine/Finished ro	Rambo/wo	0	Yes
2	River	No		Pit Latrine/Finished ro	Rambo/wo	0	Yes
1	Protected S	No		Pit Latrine/Finished ro	Rambo/wo	1	Yes
	Protected S	No		Pit Latrine/Finished ro	Rambo/wo	1	No
	Covered W	No		Pit Latrine/Finished ro	Rambo/wo	1	No
	River	No		Pit Latrine/Finished ro	Rambo/wo	0	No
	Protected I	No		Pit Latrine/Finished ro	Rambo/wo	2	Yes
	Covered W	No		No facility/Finished ro	Rambo/wo	1	Yes
1	Protected S	No		Pit Latrine/Finished ro	Rambo/wo	1	Yes
2	River	No		Pit Latrine/Finished ro	Rambo/wo	0	No
	Protected I	No		Pit Latrine/Finished ro	Rambo/wo	1	Yes
	River	No		Pit Latrine/Finished ro	Rambo/wo	0	No
	Protected S	No		No facility/Finished ro	Rambo/wo	1	Yes
	River	No		Pit Latrine/Finished ro	Rambo/wo	0	No
2	Protected S	No		No facility/Finished ro	Rambo/wo	1	Yes
	Protected S	No		Pit Latrine/Finished ro	Rambo/wo	1	No
	Protected S	No		No facility/Finished ro	Rambo/wo	2	Yes
	River	No		Pit Latrine/Finished ro	Rambo/wo	0	Yes
3	Open Sprin	No		Pit Latrine/Finished ro	Rambo/wo	2	Yes
	Protected S	No		No facility/Finished ro	Rambo/wo	0	Yes
	Protected S	No		Pit Latrine/Finished ro	Rambo/wo	1	No
	Protected S	No		Pit Latrine/Finished ro	Rambo/wo	2	No
	Open Sprin	No		Pit Latrine/Finished ro	Rambo/wo	2	Yes
2	Protected S	No		No facility/Finished ro	Rambo/wo	2	Yes
	River	No		No facility/Finished ro	Rambo/wo	0	No
	Covered W	No		Pit Latrine/Finished ro	Rambo/wo	0	Yes
	Open Sprin	No		No facility/Finished ro	Rambo/wo	1	No
	Covered W	No		Pit Latrine/Finished ro	Rambo/wo	0	No
	Protected S	No		No facility/Finished ro	Rambo/wo	2	Yes
	Covered W	No		Pit Latrine/Finished ro	Rambo/wo	1	Yes
	Protected S	No		No facility/Finished ro	Rambo/wo	1	Yes
	River	No		No facility/Finished ro	Rambo/wo	0	No
	River	No		Pit Latrine/Finished ro	Rambo/wo	0	Yes
	River	No		No facility/Finished ro	Rambo/wo	0	Yes
	Protected S	No		Pit Latrine/Finished ro	Rambo/wo	1	Yes
	Open Sprin	No		Pit Latrine/Finished ro	Rambo/wo	1	Yes
	Protected S	No		Pit Latrine/Finished ro	Rambo/wo	1	Yes
	River	No		Pit Latrine/Finished ro	Rambo/wo	2	Yes
	Covered W	Yes	Add bleach	Pit Latrine/Finished ro	Rambo/wo	1	Yes
	Protected S	No		Pit Latrine/Finished ro	Rambo/wo	2	Yes

For peer review only

	Covered W	Yes	Add bleach	Pit Latrine/	Finished ro	Rambo/wo	1	No
1	Covered W	No		No facility/	Finished ro	Rambo/wo	0	No
	Covered W	No		Pit Latrine/	Finished ro	Rambo/wo	0	No
	Protected I	No		Pit Latrine/	Finished ro	Rambo/wo	0	Yes
	Protected S	No		No facility/	Finished ro	Rambo/wo	1	Yes
1	Protected S	No		Pit Latrine/	Finished ro	Rambo/wo	2	Yes
	Protected S	No		No facility/	Finished ro	Rambo/wo	1	No
	Open Sprin	No		Pit Latrine/	Finished ro	Rambo/wo	0	No
	Protected S	No		No facility/	Finished ro	Rambo/wo	1	Yes
	Protected I	No		No facility/	Finished ro	Rambo/wo	1	Yes
	Protected I	No		Pit Latrine/	Finished ro	Rambo/wo	1	Yes
	River	No		No facility/	Finished ro	Rambo/wo	0	Yes
	Protected I	Yes	Add bleach	No facility/	Finished ro	Rambo/wo	1	Yes
1	River	No		Pit Latrine/	Finished ro	Rambo/wo	0	No
	Protected S	No		No facility/	Finished ro	Rambo/wo	1	Yes
	Protected S	No		Pit Latrine/	Finished ro	Rambo/wo	1	No
1	Open Sprin	No		Pit Latrine/	Finished ro	Rambo/wo	0	No
4	Protected I	No		Pit Latrine/	Finished ro	Rambo/wo	2	Yes
	Protected S	No		Pit Latrine/	Finished ro	Rambo/wo	1	No
	Covered W	No		Pit Latrine/	Finished ro	Rambo/wo	0	No
	Protected S	No		Pit Latrine/	Finished ro	Rambo/wo	1	Yes
	Protected S	No		Pit Latrine/	Finished ro	Rambo/wo	1	Yes
	Protected S	No		Pit Latrine/	Finished ro	Rambo/wo	2	Yes
1	Open Sprin	No		Pit Latrine/	Finished ro	Rambo/wo	1	No
2	Protected I	No		No facility/	Finished ro	Rambo/wo	1	Yes
	Protected S	No		Pit Latrine/	Finished ro	Rambo/wo	1	Yes
	Protected I	No		Pit Latrine/	Finished ro	Rambo/wo	2	Yes
3	Piped outsi	No		Pit Latrine/	Finished ro	Rambo/wo	1	No
	Protected S	No		Pit Latrine/	Finished ro	Rambo/wo	1	Yes
	Protected S	No		No facility/	Finished ro	Rambo/wo	1	Yes
	Covered W	No		Pit Latrine/	Finished ro	Rambo/wo	0	No
	River	No		No facility/	Finished ro	Rambo/wo	1	Yes
	Protected S	No		Pit Latrine/	Finished ro	Rambo/wo	2	No
1	Protected I	No		No facility/	Finished ro	Rambo/wo	1	Yes
	River	No		Pit Latrine/	Finished ro	Rambo/wo	1	No
	Open Sprin	No		Pit Latrine/	Finished ro	Rambo/wo	0	No
	Covered W	No		Pit Latrine/	Finished ro	Rambo/wo	2	Yes
	Protected S	No		No facility/	Thatch/lea	Rambo/wo	1	No
2	Covered W	No		Pit Latrine/	Finished ro	Rambo/wo	1	Yes
	Covered W	No		Pit Latrine/	Finished ro	Rambo/wo	0	No
	Open Sprin	No		Pit Latrine/	Finished ro	Rambo/wo	1	Yes
	Protected I	No		No facility/	Finished ro	Rambo/wo	2	Yes
	Protected I	No		Pit Latrine/	Finished ro	Rambo/wo	1	Yes
	Covered W	No		Pit Latrine/	Finished ro	Rambo/wo	1	Yes
	Covered W	Yes	Add bleach	Pit Latrine/	Finished ro	Rambo/wo	0	No
	Covered W	Yes	Boil	No facility/	Finished ro	Rambo/wo	1	No
	Open Sprin	No		Pit Latrine/	Finished ro	Rambo/wo	1	Yes
	Protected S	No		Pit Latrine/	Finished ro	Rambo/wo	1	No
	Covered W	No		Pit Latrine/	Finished ro	Rambo/wo	0	Yes
	Covered W	Yes	Add bleach	Pit Latrine/	Finished ro	Rambo/wo	1	Yes
	Protected S	No		No facility/	Finished ro	Rambo/wo	1	Yes
	Open Sprin	No		Pit Latrine/	Finished ro	Rambo/wo	2	Yes

For peer review only

	Protected S	No		No facility/	Finished ro	Rambo/wo	2	Yes
	River	No		No facility/	Finished ro	Rambo/wo	0	Yes
	River	No		No facility/	Finished ro	Rambo/wo	1	No
	Covered W	No		Pit Latrine/	Finished ro	Rambo/wo	1	No
	Pond/Lake,	No		Pit Latrine/	Finished ro	Rambo/wo	1	Yes
1	Covered W	No		Pit Latrine/	Finished ro	Rambo/wo	1	Yes
	Protected S	No		Other	Finished ro	Rambo/wo	1	Yes
	Covered W	No		Pit Latrine/	Finished ro	Rambo/wo	1	No
1	Protected S	No		Other	Finished ro	Rambo/wo	1	Yes
1	Covered W	No		Pit Latrine/	Finished ro	Rambo/wo	1	Yes
1	River	No		No facility/	Finished ro	Rambo/wo	0	Yes
1	Protected I	No		No facility/	Finished ro	Rambo/wo	0	No
	River	No		No facility/	Finished ro	Rambo/wo	0	No
	River	No		No facility/	Finished ro	Rambo/wo	0	Yes
	Covered W	No		Pit Latrine/	Finished ro	Rambo/wo	1	No
1	Protected I	No		Pit Latrine/	Finished ro	Rambo/wo	0	No
	River	No		No facility/	Finished ro	Rambo/wo	0	Yes
	River	No		Pit Latrine/	Finished ro	Rambo/wo	0	Yes
	River	No		No facility/	Finished ro	Rambo/wo	0	No
0	Covered W	No		Pit Latrine/	Finished ro	Rambo/wo	1	Yes
	Protected I	No		Pit Latrine/	Finished ro	Rambo/wo	2	Yes
	Protected S	No		No facility/	Finished ro	Rambo/wo	1	Yes
1	Open Sprin	No		Pit Latrine/	Finished ro	Rambo/wo	1	No
	River	No		Pit Latrine/	Finished ro	Rambo/wo	0	Yes
1	River	No		Pit Latrine/	Finished ro	Rambo/wo	1	Yes
	Protected S	No		No facility/	Wood plan	Rambo/wo	1	No
	Protected S	No		No facility/	Finished ro	Rambo/wo	1	Yes
	River	No		No facility/	Finished ro	Rambo/wo	0	Yes
	Protected S	No		Pit Latrine/	Finished ro	Rambo/wo	1	Yes
	River	No		Pit Latrine/	Finished ro	Rambo/wo	0	No
	Covered W	No		Pit Latrine/	Finished ro	Rambo/wo	1	No
1	Covered W	Yes	Boil	Pit Latrine/	Finished ro	Rambo/wo	1	Yes
	River	No		Pit Latrine/	Finished ro	Rambo/wo	0	No
3	Covered W	No		No facility/	Finished ro	Rambo/wo	1	Yes
	Protected I	No		Pit Latrine/	Finished ro	Rambo/wo	2	Yes
	Covered W	No		Pit Latrine/	Finished ro	Rambo/wo	0	Yes
	Protected S	No		Pit Latrine/	Finished ro	Rambo/wo	1	No
	Covered W	No		Pit Latrine/	Finished ro	Rambo/wo	1	Yes
	Covered W	No		Pit Latrine/	Finished ro	Rambo/wo	2	Yes
3	River	No		Pit Latrine/	Finished ro	Rambo/wo	1	Yes
	Covered W	No		Pit Latrine/	Finished ro	Rambo/wo	0	Yes
	Protected S	No		No facility/	Finished ro	Rambo/wo	0	No
	Protected S	No		Pit Latrine/	Finished ro	Rambo/wo	1	Yes
	Protected I	No		Pit Latrine/	Finished ro	Rambo/wo	2	Yes
	Protected S	No		Pit Latrine/	Finished ro	Rambo/wo	1	No
2	Piped outsi	No		Pit Latrine/	Finished ro	Rambo/wo	1	No
	Protected S	No		Pit Latrine/	Finished ro	Rambo/wo	2	Yes
	Covered W	No		Pit Latrine/	Finished ro	Rambo/wo	1	No
	Piped outsi	No		Pit Latrine/	Finished ro	Rambo/wo	0	Yes
	Covered W	No		No facility/	Finished ro	Rambo/wo	2	Yes
2	Covered W	No		Pit Latrine/	Finished ro	Rambo/wo	0	Yes
	Protected S	No		Pit Latrine/	Finished ro	Rambo/wo	0	Yes

For peer review only

	Covered W	No	Pit Latrine/	Finished ro	Rambo/wo	1	Yes
1	Covered W	No	No facility/	Finished ro	Rambo/wo	2	Yes
	Piped outsi	No	No facility/	Finished ro	Rambo/wo	1	No
	Piped outsi	No	Pit Latrine/	Finished ro	Rambo/wo	2	No
1	Piped outsi	No	Pit Latrine/	Finished ro	Rambo/wo	1	Yes
	Covered W	No	Pit Latrine/	Finished ro	Rambo/wo	2	Yes
1	Covered W	No	No facility/	Finished ro	Stone with	1	Yes
1	Protected I	No	Pit Latrine/	Finished ro	Rambo/wo	1	Yes
	Piped outsi	No	No facility/	Finished ro	Rambo/wo	1	Yes
	Covered W	No	Pit Latrine/	Finished ro	Rambo/wo	0	Yes
	Covered W	No	Pit Latrine/	Finished ro	Rambo/wo	1	Yes
	Protected S	No	Pit Latrine/	Finished ro	Rambo/wo	1	No
	Covered W	No	Pit Latrine/	Finished ro	Rambo/wo	1	Yes
	Protected S	No	Pit Latrine/	Finished ro	Rambo/wo	1	Yes
	Piped outsi	No	Pit Latrine/	Finished ro	Rambo/wo	0	Yes
	Covered W	No	Pit Latrine/	Finished ro	Rambo/wo	1	No
	Covered W	No	Pit Latrine/	Finished ro	Rambo/wo	2	No
	Piped outsi	No	Pit Latrine/	Finished ro	Rambo/wo	1	Yes
	Protected S	No	Pit Latrine/	Finished ro	Rambo/wo	2	Yes
	Piped outsi	No	Pit Latrine/	Finished ro	Rambo/wo	0	Yes
	Piped outsi	No	Pit Latrine/	Finished ro	Rambo/wo	0	No
	Protected S	No	No facility/	Finished ro	Rambo/wo	1	Yes
1	Protected S	No	No facility/	Finished ro	Rambo/wo	1	Yes
	Protected S	No	Pit Latrine/	Finished ro	Rambo/wo	1	No
1	Piped outsi	No	Pit Latrine/	Finished ro	Rambo/wo	1	Yes
	Covered W	No	Other	Finished ro	Rambo/wo	0	No
1	Piped outsi	No	Pit Latrine/	Finished ro	Rambo/wo	2	Yes
1	Piped outsi	No	Pit Latrine/	Finished ro	Rambo/wo	0	No
	Covered W	No	No facility/	Finished ro	Rambo/wo	1	Yes
	Protected I	No	No facility/	Finished ro	Rambo/wo	0	Yes
1	Protected I	No	Pit Latrine/	Finished ro	Rambo/wo	1	Yes
	Protected S	No	Pit Latrine/	Finished ro	Rambo/wo	2	Yes
	Piped outsi	No	Pit Latrine/	Finished ro	Rambo/wo	1	No
	Covered W	No	Pit Latrine/	Finished ro	Rambo/wo	0	Yes
1	Piped outsi	No	Pit Latrine/	Finished ro	Rambo/wo	1	No
	Protected I	No	Pit Latrine/	Finished ro	Rambo/wo	1	Yes
	Protected I	No	Pit Latrine/	Finished ro	Rambo/wo	2	Yes
	Protected S	No	Pit Latrine/	Finished ro	Rambo/wo	1	No
	Covered W	No	Pit Latrine/	Finished ro	Rambo/wo	0	Yes
	Protected S	No	Pit Latrine/	Finished ro	Rambo/wo	1	No
	Piped outsi	No	Pit Latrine/	Finished ro	Rambo/wo	1	Yes
1	Covered W	No	Pit Latrine/	Finished ro	Rambo/wo	1	Yes
	Protected S	No	Pit Latrine/	Finished ro	Rambo/wo	0	Yes
	Protected I	No	Pit Latrine/	Finished ro	Rambo/wo	1	No
	Piped outsi	No	Pit Latrine/	Finished ro	Rambo/wo	0	Yes
	Covered W	No	No facility/	Finished ro	Rambo/wo	1	Yes
	Covered W	No	Pit Latrine/	Finished ro	Rambo/wo	0	No
3	Protected I	No	Pit Latrine/	Finished ro	Rambo/wo	1	No
	Protected S	No	Pit Latrine/	Finished ro	Rambo/wo	2	No
	Covered W	No	Pit Latrine/	Finished ro	Rambo/wo	2	Yes
	Protected I	No	Pit Latrine/	Finished ro	Rambo/wo	1	Yes
	Protected S	No	Pit Latrine/	Finished ro	Rambo/wo	1	Yes

For peer review only

	Protected S	No	Pit Latrine/	Finished ro	Rambo/wo	2	Yes
	Piped outsi	No	Pit Latrine/	Finished ro	Rambo/wo	1	Yes
	Covered W	No	Pit Latrine/	Finished ro	Rambo/wo	0	Yes
	Protected S	No	Pit Latrine/	Finished ro	Rambo/wo	2	Yes
	Covered W	No	Pit Latrine/	Finished ro	Rambo/wo	0	No
1	River	No	Pit Latrine/	Finished ro	Rambo/wo	1	Yes
	Covered W	No	Pit Latrine/	Finished ro	Rambo/wo	1	No
1	Covered W	No	Pit Latrine/	Finished ro	Rambo/wo	1	Yes
	Protected S	No	No facility/	Finished ro	Rambo/wo	1	Yes
	Protected S	No	No facility/	Finished ro	Rambo/wo	2	No
	Covered W	No	Pit Latrine/	Finished ro	Rambo/wo	0	Yes
	Protected I	No	Pit Latrine/	Finished ro	Rambo/wo	2	No
1	Piped outsi	No	Pit Latrine/	Finished ro	Rambo/wo	1	Yes
1	Protected S	No	Pit Latrine/	Finished ro	Rambo/wo	1	Yes
	Protected S	No	Pit Latrine/	Finished ro	Rambo/wo	0	Yes
	Protected I	No	Pit Latrine/	Finished ro	Rambo/wo	1	Yes
	Protected I	No	Pit Latrine/	Finished ro	Rambo/wo	1	No
	Covered W	No	Pit Latrine/	Finished ro	Rambo/wo	1	No
	Open Sprin	No	Pit Latrine/	Finished ro	Rambo/wo	1	Yes
	Piped outsi	No	Pit Latrine/	Finished ro	Rambo/wo	1	No
1	Protected I	No	Pit Latrine/	Finished ro	Rambo/wo	1	Yes
	Protected I	No	Pit Latrine/	Finished ro	Rambo/wo	1	Yes
	Protected S	No	Pit Latrine/	Finished ro	Rambo/wo	0	Yes
	Piped outsi	No	Pit Latrine/	Finished ro	Rambo/wo	1	Yes
	Covered W	No	Pit Latrine/	Finished ro	Rambo/wo	2	Yes
	Protected S	No	Pit Latrine/	Finished ro	Rambo/wo	2	Yes
	Protected S	No	No facility/	Finished ro	Rambo/wo	2	Yes
	Protected I	No	Pit Latrine/	Finished ro	Rambo/wo	1	Yes
	Piped outsi	No	No facility/	Finished ro	Rambo/wo	1	No
	Protected I	No	Pit Latrine/	Finished ro	Rambo/wo	2	Yes
	Covered W	No	Pit Latrine/	Finished ro	Rambo/wo	0	Yes
1	Covered W	No	Other	Finished ro	Rambo/wo	0	No
	Piped outsi	No	Pit Latrine/	Finished ro	Rambo/wo	1	No
	Piped outsi	No	Pit Latrine/	Finished ro	Rambo/wo	0	Yes
	Piped outsi	No	Pit Latrine/	Finished ro	Rambo/wo	0	No
	Piped outsi	No	Pit Latrine/	Finished ro	Rambo/wo	1	No
	Covered W	No	Pit Latrine/	Finished ro	Rambo/wo	0	Yes
	Protected I	No	Pit Latrine/	Finished ro	Rambo/wo	1	No
	Protected S	No	Pit Latrine/	Finished ro	Rambo/wo	2	Yes
	River	No	Pit Latrine/	Finished ro	Rambo/wo	1	Yes
	River	No	No facility/	Finished ro	Rambo/wo	1	Yes
	Protected S	No	Pit Latrine/	Finished ro	Rambo/wo	1	No
	Piped outsi	No	Pit Latrine/	Finished ro	Rambo/wo	1	Yes
	Covered W	No	Pit Latrine/	Finished ro	Rambo/wo	1	Yes
	Covered W	No	Pit Latrine/	Finished ro	Rambo/wo	2	No
	Protected S	No	Pit Latrine/	Finished ro	Rambo/wo	2	No
	Covered W	No	Other	Finished ro	Rambo/wo	0	No
	Protected I	No	Pit Latrine/	Finished ro	Rambo/wo	1	No
	Protected S	No	Pit Latrine/	Finished ro	Rambo/wo	2	Yes
	Protected I	No	Pit Latrine/	Finished ro	Rambo/wo	1	Yes
1	Piped outsi	No	No facility/	Finished ro	Rambo/wo	0	Yes
	Protected S	No	No facility/	Finished ro	Rambo/wo	0	Yes

	Open Sprin	No	No facility/ Finished ro	Rambo/wo	0	Yes
	Open Sprin	No	Pit Latrine/ Finished ro	Rambo/wo	1	Yes
	Protected S	No	No facility/ Finished ro	Rambo/wo	0	Yes
	Piped outsi	No	Pit Latrine/ Finished ro	Rambo/wo	1	No
	Open Sprin	No	No facility/ Finished ro	Rambo/wo	1	Yes
	Protected S	No	No facility/ Finished ro	Rambo/wo	0	No
	Protected I	No	No facility/ Finished ro	Rambo/wo	0	No
	Piped outsi	No	No facility/ Finished ro	Rambo/wo	0	Yes
1	Protected I	No	No facility/ Finished ro	Rambo/wo	1	Yes
1	Open Sprin	No	No facility/ Finished ro	Rambo/wo	1	Yes
	Protected S	No	No facility/ Wood plan	Rambo/wo	0	No
	Protected I	No	Pit Latrine/ Finished ro	Rambo/wo	0	No
	Piped outsi	No	No facility/ Finished ro	Rambo/wo	0	No
	Protected I	No	Pit Latrine/ Finished ro	Rambo/wo	0	Yes
	Protected I	No	No facility/ Finished ro	Rambo/wo	0	No
	Open Sprin	No	No facility/ Finished ro	Rambo/wo	0	Yes
	River	No	No facility/ Finished ro	Rambo/wo	0	No
	Protected S	No	No facility/ Finished ro	Rambo/wo	0	Yes
	Open Sprin	No	Pit Latrine/ Finished ro	Rambo/wo	1	No
	Piped outsi	No	Pit Latrine/ Finished ro	Rambo/wo	1	Yes
	Open Sprin	No	Pit Latrine/ Finished ro	Rambo/wo	0	Yes
	Open Sprin	No	No facility/ Finished ro	Rambo/wo	0	Yes
	Protected I	No	No facility/ Finished ro	No walls	1	Yes
1	Protected S	No	No facility/ Finished ro	Rambo/wo	0	Yes
	Protected S	No	No facility/ Finished ro	Rambo/wo	0	Yes
	Protected I	No	Pit Latrine/ Finished ro	Rambo/wo	0	Yes
	Protected I	No	Pit Latrine/ Finished ro	Rambo/wo	0	Yes
	Protected I	No	Pit Latrine/ Finished ro	Rambo/wo	1	Yes
	River	No	Pit Latrine/ Finished ro	Rambo/wo	0	No
	Piped outsi	No	Pit Latrine/ Finished ro	Rambo/wo	1	Yes
	Protected I	No	Pit Latrine/ Finished ro	Rambo/wo	0	Yes
	Protected I	No	Pit Latrine/ Finished ro	Rambo/wo	0	Yes
2	Protected S	No	No facility/ Finished ro	Rambo/wo	0	Yes
	River	No	No facility/ Finished ro	Rambo/wo	0	No
	Piped outsi	No	No facility/ Finished ro	Rambo/wo	0	No
	Open Sprin	No	Pit Latrine/ Finished ro	Rambo/wo	1	No
	Open Sprin	No	Pit Latrine/ Finished ro	Rambo/wo	1	Yes
	Open Sprin	No	Pit Latrine/ Finished ro	Rambo/wo	1	Yes
	Open Sprin	No	No facility/ Finished ro	Rambo/wo	0	Yes
	Protected S	No	No facility/ Finished ro	Rambo/wo	0	Yes
	Piped outsi	No	Pit Latrine/ Finished ro	Rambo/wo	1	Yes
	Protected I	No	Pit Latrine/ Finished ro	Rambo/wo	1	Yes
	Open Sprin	No	No facility/ Finished ro	Rambo/wo	0	No
	Open Sprin	No	No facility/ Finished ro	Rambo/wo	0	No
	Protected S	No	No facility/ Finished ro	Rambo/wo	0	Yes
	Open Sprin	No	No facility/ Finished ro	Rambo/wo	1	Yes
	Protected I	No	Pit Latrine/ Finished ro	Rambo/wo	1	Yes
	Protected S	No	No facility/ Thatch/lea	Rambo/wo	0	No
	Open Sprin	No	No facility/ Finished ro	Rambo/wo	0	Yes
	Open Sprin	No	Pit Latrine/ Finished ro	Rambo/wo	0	No
	Piped outsi	No	No facility/ Finished ro	Rambo/wo	1	Yes
	Protected I	No	Pit Latrine/ Finished ro	Rambo/wo	1	Yes

For peer review only

	River	No	No facility/	Finished ro	Rambo/wo	0	No
	Open Sprin	No	No facility/	Roofing shi	Rambo/wo	0	Yes
3	Open Sprin	No	Pit Latrine/	Finished ro	Rambo/wo	1	Yes
	Piped outsi	No	No facility/	Finished ro	Rambo/wo	1	No
	Protected I	No	Pit Latrine/	Finished ro	Rambo/wo	1	Yes
2	Piped outsi	No	Pit Latrine/	Finished ro	Rambo/wo	1	Yes
	Protected I	No	Pit Latrine/	Finished ro	Rambo/wo	1	Yes
	Open Sprin	No	No facility/	Finished ro	Rambo/wo	0	Yes
	Open Sprin	No	No facility/	Finished ro	Rambo/wo	0	Yes
	Protected I	No	Pit Latrine/	Wood plan	Rambo/wo	1	Yes
	Open Sprin	No	No facility/	Finished ro	Rambo/wo	0	Yes
	Open Sprin	No	No facility/	Finished ro	Rambo/wo	1	No
2	Protected I	No	Pit Latrine/	Finished ro	Rambo/wo	0	Yes
	Protected I	No	Pit Latrine/	Finished ro	Rambo/wo	1	Yes
	Protected I	No	Pit Latrine/	Finished ro	Rambo/wo	1	Yes
	Protected S	No	No facility/	Finished ro	Rambo/wo	0	Yes
	Protected I	No	Pit Latrine/	Finished ro	Rambo/wo	0	Yes
	Protected I	No	No facility/	Finished ro	Rambo/wo	0	Yes
	Protected S	No	No facility/	Finished ro	Rambo/wo	0	No
	River	No	No facility/	Finished ro	Rambo/wo	0	No
1	Protected S	No	No facility/	Finished ro	Rambo/wo	0	Yes
	River	No	No facility/	Finished ro	Rambo/wo	0	No
	Protected I	No	Pit Latrine/	Finished ro	Rambo/wo	0	No
1	Piped outsi	No	No facility/	Finished ro	Rambo/wo	0	No
	Open Sprin	No	Pit Latrine/	Finished ro	Rambo/wo	0	Yes
	Protected I	No	Pit Latrine/	Finished ro	Rambo/wo	1	No
4	Protected I	No	Pit Latrine/	Finished ro	Rambo/wo	1	No
	Protected I	No	Pit Latrine/	Finished ro	Rambo/wo	0	Yes
	Open Sprin	No	Pit Latrine/	Finished ro	Rambo/wo	0	No
	Protected I	No	Pit Latrine/	Finished ro	Rambo/wo	1	Yes
	Protected S	No	No facility/	Finished ro	Rambo/wo	0	Yes
	Protected I	No	Pit Latrine/	Finished ro	Rambo/wo	1	Yes
3	Protected I	No	Pit Latrine/	Finished ro	Rambo/wo	1	Yes
	Protected I	No	No facility/	Finished ro	Rambo/wo	0	No
	Protected S	No	No facility/	Finished ro	Rambo/wo	0	Yes
1	Open Sprin	No	Pit Latrine/	Finished ro	Rambo/wo	1	No
	Piped outsi	No	No facility/	Finished ro	Rambo/wo	1	Yes
	River	No	No facility/	Finished ro	Rambo/wo	0	No
	Protected I	No	Pit Latrine/	Finished ro	Rambo/wo	0	Yes
1	Protected S	No	No facility/	Finished ro	Rambo/wo	0	No
2	Open Sprin	No	No facility/	Finished ro	Rambo/wo	1	Yes
	Open Sprin	No	No facility/	Finished ro	Rambo/wo	0	No
	Protected S	No	No facility/	Finished ro	Rambo/wo	1	Yes
	Open Sprin	No	Pit Latrine/	Finished ro	Rambo/wo	1	Yes
	Open Sprin	No	No facility/	Finished ro	Rambo/wo	0	No
	Open Sprin	No	Pit Latrine/	Finished ro	Rambo/wo	1	Yes
	Protected S	No	Pit Latrine/	Finished ro	Rambo/wo	1	Yes
	Protected I	No	No facility/	Finished ro	Rambo/wo	0	Yes
	Piped outsi	No	Pit Latrine/	Finished ro	Rambo/wo	0	No
	Protected I	No	Pit Latrine/	Finished ro	Rambo/wo	1	No
	Open Sprin	No	No facility/	Finished ro	Rambo/wo	0	Yes
	Protected I	No	No facility/	Finished ro	Rambo/wo	0	Yes

For peer review only

	River	No		No facility/	Finished ro	Rambo/wo	0	No
	Protected S	No		No facility/	Finished ro	Rambo/wo	1	No
	Open Sprin	No		Pit Latrine/	Finished ro	Rambo/wo	1	No
	Open Sprin	No		No facility/	Finished ro	Rambo/wo	0	Yes
	Protected S	No		No facility/	Finished ro	Rambo/wo	0	Yes
	Protected I	No		Pit Latrine/	Finished ro	Rambo/wo	1	Yes
	Piped outsi	No		No facility/	Finished ro	Rambo/wo	1	Yes
	Open Sprin	No		No facility/	Finished ro	Rambo/wo	0	Yes
	Protected S	No		Pit Latrine/	Finished ro	Rambo/wo	0	Yes
	Open Sprin	No		No facility/	Finished ro	Rambo/wo	1	No
	Open Sprin	No		No facility/	Finished ro	Rambo/wo	0	Yes
	Protected S	No		Pit Latrine/	Finished ro	Rambo/wo	1	Yes
	Protected S	No		No facility/	Finished ro	Rambo/wo	0	Yes
1	Covered W	No		Pit Latrine/	Finished ro	Rambo/wo	1	Yes
	Piped outsi	No		Pit Latrine/	Finished ro	Rambo/wo	1	Yes
	Open Sprin	No		No facility/	Finished ro	Rambo/wo	0	Yes
	Piped outsi	No		Pit Latrine/	Finished ro	Rambo/wo	1	Yes
	Open Well	No		No facility/	Finished ro	Rambo/wo	1	Yes
	Protected I	No		Ventilated	Finished ro	Rambo/wo	2	No
	Open Sprin	No		Pit Latrine/	Finished ro	Rambo/wo	1	Yes
	Open Well	No		No facility/	Finished ro	Rambo/wo	1	No
	Piped outsi	No		Pit Latrine/	Finished ro	Rambo/wo	1	Yes
	Piped outsi	No		Pit Latrine/	Thatch/lea	Rambo/wo	0	Yes
	Piped outsi	No		No facility/	Finished ro	Rambo/wo	1	Yes
	Piped outsi	No		Pit Latrine/	Finished ro	Rambo/wo	1	Yes
	Piped outsi	No		Pit Latrine/	Finished ro	Rambo/wo	1	Yes
	Piped outsi	No		Pit Latrine/	Finished ro	Rambo/wo	1	No
	Piped outsi	No		Pit Latrine/	Finished ro	Rambo/wo	1	Yes
	Open Well	Yes	Boil	Pit Latrine/	Finished ro	Rambo/wo	0	Yes
1	Piped outsi	No		Pit Latrine/	Finished ro	Rambo/wo	1	Yes
	Piped outsi	No		Pit Latrine/	Finished ro	Rambo/wo	2	No
	Piped outsi	No		Pit Latrine/	Finished ro	Stone with	1	Yes
	Piped outsi	No		Pit Latrine/	Finished ro	Rambo/wo	1	Yes
	Piped outsi	No		Pit Latrine/	Finished ro	Rambo/wo	1	Yes
	Piped outsi	No		Pit Latrine/	Finished ro	Rambo/wo	1	No
	Piped outsi	No		Pit Latrine/	Finished ro	Rambo/wo	1	Yes
	Pond/Lake,	No		No facility/	Finished ro	Rambo/wo	0	Yes
	Piped outsi	No		Pit Latrine/	Finished ro	Rambo/wo	1	No
	Piped outsi	No		Pit Latrine/	Finished ro	Rambo/wo	2	Yes
	Piped outsi	No		Pit Latrine/	Finished ro	Rambo/wo	1	Yes
	Piped outsi	No		Pit Latrine/	Finished ro	Rambo/wo	1	Yes
	Pond/Lake,	No		No facility/	Finished ro	Rambo/wo	0	No
	Pond/Lake,	No		No facility/	Finished ro	Rambo/wo	1	Yes
	Piped outsi	No		Pit Latrine/	Finished ro	Rambo/wo	1	Yes
	Piped outsi	No		Pit Latrine/	Finished ro	Rambo/wo	1	Yes
	Protected I	No		No facility/	Thatch/lea	Rambo/wo	0	Yes
	Piped outsi	No		Pit Latrine/	Finished ro	Rambo/wo	1	No
	Covered W	No		Pit Latrine/	Finished ro	Rambo/wo	2	Yes
	Piped outsi	No		No facility/	Finished ro	Rambo/wo	1	Yes
	Piped outsi	No		Ventilated	Finished ro	Rambo/wo	1	Yes
	Piped outsi	No		Pit Latrine/	Finished ro	Rambo/wo	1	Yes
2	Piped outsi	No		Pit Latrine/	Finished ro	Rambo/wo	1	No

For peer review only

	Protected I	No		Pit Latrine/	Finished ro	Rambo/wo	1	Yes
	Protected I	No		Pit Latrine/	Finished ro	Rambo/wo	1	No
	Covered W	No		Pit Latrine/	Finished ro	Rambo/wo	1	Yes
	Piped outsi	No		Pit Latrine/	Thatch/lea	Rambo/wo	0	No
	Piped outsi	No		Pit Latrine/	Finished ro	Rambo/wo	0	Yes
	Piped outsi	No		Pit Latrine/	Finished ro	Rambo/wo	1	Yes
2	Piped into (	No		Pit Latrine/	Finished ro	Cement	3	Yes
	Protected I	No		Pit Latrine/	Finished ro	Rambo/wo	1	Yes
	Protected I	No		Pit Latrine/	Finished ro	Rambo/wo	1	Yes
	Piped outsi	No		Pit Latrine/	Thatch/lea	Rambo/wo	0	Yes
	Open Well	No		No facility/	Finished ro	Rambo/wo	1	Yes
	Piped outsi	No		Pit Latrine/	Finished ro	Rambo/wo	1	Yes
	Covered W	No		Pit Latrine/	Finished ro	Rambo/wo	1	Yes
	Piped outsi	No		No facility/	Finished ro	Rambo/wo	1	Yes
	Piped outsi	No		Pit Latrine/	Finished ro	Rambo/wo	1	No
	Piped outsi	No		Pit Latrine/	Finished ro	Rambo/wo	1	Yes
	Piped outsi	No		Pit Latrine/	Finished ro	Rambo/wo	1	Yes
	Protected S	Yes	Boil	Pit Latrine/	Thatch/lea	Rambo/wo	0	Yes
	Piped outsi	No		Pit Latrine/	Finished ro	Rambo/wo	1	Yes
	Covered W	No		Pit Latrine/	Finished ro	Rambo/wo	1	Yes
	Open Sprin	No		Pit Latrine/	Finished ro	Rambo/wo	1	Yes
1	Pond/Lake,	No		Pit Latrine/	Finished ro	Stone with	2	No
	Piped outsi	No		Pit Latrine/	Finished ro	Rambo/wo	1	Yes
	Protected I	No		Pit Latrine/	Finished ro	Cement	1	Yes
	Piped outsi	No		Pit Latrine/	Finished ro	Rambo/wo	1	Yes
	Piped outsi	No		Flush toilet	Finished ro	Rambo/wo	1	Yes
	Piped outsi	No		Pit Latrine/	Finished ro	Rambo/wo	1	Yes
	Covered W	No		Pit Latrine/	Finished ro	Rambo/wo	1	No
	Piped outsi	No		No facility/	Finished ro	Rambo/wo	1	Yes
	Open Sprin	No		Pit Latrine/	Finished ro	Rambo/wo	1	Yes
	Pond/Lake,	No		No facility/	Finished ro	Rambo/wo	1	Yes
	Piped outsi	No		Pit Latrine/	Finished ro	Rambo/wo	1	Yes
	Piped into (	No		Pit Latrine/	Finished ro	Rambo/wo	0	No
	Piped outsi	No		Pit Latrine/	Finished ro	Rambo/wo	2	Yes
	Protected S	No		Pit Latrine/	Finished ro	Rambo/wo	1	Yes
	Piped outsi	No		Pit Latrine/	Finished ro	Rambo/wo	1	Yes
	Covered W	No		Pit Latrine/	Finished ro	Rambo/wo	1	Yes
1	Piped outsi	No		Pit Latrine/	Finished ro	Rambo/wo	1	No
1	Open Well	No		Pit Latrine/	Finished ro	Rambo/wo	0	Yes
	Piped outsi	No		No facility/	Finished ro	Rambo/wo	1	Yes
	Covered W	No		Pit Latrine/	Finished ro	Rambo/wo	1	No
	Piped outsi	No		Pit Latrine/	Finished ro	Rambo/wo	1	No
1	Covered W	No		Pit Latrine/	Finished ro	Cement	1	Yes
	Piped outsi	No		Pit Latrine/	Finished ro	Rambo/wo	1	Yes
	Covered W	No		Pit Latrine/	Finished ro	Rambo/wo	1	No
	Piped outsi	No		Pit Latrine/	Finished ro	Rambo/wo	1	Yes
	Open Sprin	No		Pit Latrine/	Finished ro	Rambo/wo	1	Yes
	Open Well	No		No facility/	Finished ro	Rambo/wo	1	Yes
	Piped outsi	No		Pit Latrine/	Finished ro	Rambo/wo	1	Yes
	Covered W	No		Pit Latrine/	Finished ro	Rambo/wo	1	Yes
	Piped outsi	No		Pit Latrine/	Finished ro	Rambo/wo	1	Yes
	Piped outsi	No		Pit Latrine/	Finished ro	Rambo/wo	0	Yes

For peer review only

	Piped outsi	No		No facility/	Finished ro	Rambo/wo	1	Yes
	Piped outsi	No		Pit Latrine/	Finished ro	Rambo/wo	1	Yes
	Pond/Lake,	No		No facility/	Finished ro	Rambo/wo	1	No
	Piped outsi	No		Pit Latrine/	Finished ro	Rambo/wo	1	No
	Piped outsi	No		Pit Latrine/	Finished ro	Rambo/wo	1	No
	Open Well	No		No facility/	Finished ro	Rambo/wo	1	No
	Piped outsi	No		Pit Latrine/	Finished ro	Rambo/wo	0	Yes
	Protected I	No		Ventilated	Finished ro	Rambo/wo	1	No
	Protected I	No		Pit Latrine/	Finished ro	Rambo/wo	1	Yes
	Piped outsi	No		Pit Latrine/	Finished ro	Rambo/wo	1	Yes
	Piped outsi	No		Pit Latrine/	Finished ro	Rambo/wo	1	Yes
	Protected I	No		Ventilated	Finished ro	Rambo/wo	1	Yes
	Piped outsi	No		No facility/	Finished ro	Rambo/wo	0	No
1	Covered W	No		Pit Latrine/	Thatch/lea	Rambo/wo	1	Yes
	Open Sprin	No		Pit Latrine/	Finished ro	Rambo/wo	1	Yes
	Piped outsi	No		Pit Latrine/	Finished ro	Rambo/wo	1	No
	Piped outsi	No		Pit Latrine/	Finished ro	Rambo/wo	1	Yes
	Piped outsi	No		Pit Latrine/	Finished ro	Rambo/wo	1	Yes
	Pond/Lake,	Yes	Strain it thr	No facility/	Finished ro	Rambo/wo	1	Yes
	Open Sprin	No		No facility/	Finished ro	Rambo/wo	1	No
	Piped outsi	No		Pit Latrine/	Finished ro	Rambo/wo	1	Yes
	Piped outsi	No		No facility/	Finished ro	Rambo/wo	1	Yes
	Pond/Lake,	No		No facility/	Finished ro	Rambo/wo	0	No
	Piped outsi	No		Pit Latrine/	Finished ro	Rambo/wo	1	No
	Pond/Lake,	No		No facility/	Finished ro	Rambo/wo	1	No
	Piped outsi	No		Pit Latrine/	Finished ro	Rambo/wo	0	Yes
	Piped outsi	No		Ventilated	Finished ro	Stone with	2	No
	Open Sprin	No		Pit Latrine/	Finished ro	Rambo/wo	2	Yes
	Pond/Lake,	No		No facility/	Finished ro	Rambo/wo	1	Yes
	Covered W	No		No facility/	Finished ro	Rambo/wo	1	Yes
	Protected I	No		Pit Latrine/	Finished ro	Rambo/wo	1	No
	Pond/Lake,	No		Pit Latrine/	Finished ro	Rambo/wo	1	No
	Protected I	No		Pit Latrine/	Finished ro	Rambo/wo	1	No
	Piped outsi	No		Pit Latrine/	Finished ro	Rambo/wo	1	Yes
	Piped outsi	No		Pit Latrine/	Finished ro	Rambo/wo	1	Yes
	Piped outsi	No		Pit Latrine/	Finished ro	Rambo/wo	1	Yes
	Piped outsi	No		Pit Latrine/	Finished ro	Rambo/wo	1	Yes
	Piped outsi	No		Pit Latrine/	Finished ro	Rambo/wo	0	Yes
	Piped outsi	No		Pit Latrine/	Finished ro	Rambo/wo	1	Yes
	Piped outsi	No		Pit Latrine/	Thatch/lea	Rambo/wo	0	Yes
	Piped outsi	No		No facility/	Finished ro	Rambo/wo	1	Yes
	Protected I	No		No facility/	Finished ro	Rambo/wo	1	No
	Piped outsi	No		Pit Latrine/	Finished ro	Rambo/wo	1	Yes
	Piped outsi	No		Pit Latrine/	Finished ro	Rambo/wo	1	Yes
	Piped outsi	No		Pit Latrine/	Thatch/lea	Rambo/wo	0	Yes
	Protected S	No		Pit Latrine/	Finished ro	Rambo/wo	1	Yes
	Covered W	No		Pit Latrine/	Finished ro	Rambo/wo	0	No
	Piped outsi	No		Pit Latrine/	Thatch/lea	Rambo/wo	1	Yes
	Piped outsi	No		Pit Latrine/	Finished ro	Stone with	1	No
	Piped outsi	No		Pit Latrine/	Finished ro	Rambo/wo	1	Yes
	Covered W	Yes	Strain it thr	No facility/	Finished ro	Stone with	1	Yes
	Open Well	No		Pit Latrine/	Finished ro	Rambo/wo	1	No

For peer review only

	Piped outsi	No	No facility/ Finished ro	Rambo/wo	1	Yes
	Piped outsi	No	Pit Latrine/ Finished ro	Rambo/wo	0	Yes
	Piped outsi	No	No facility/ Finished ro	Rambo/wo	1	Yes
	Piped outsi	No	Pit Latrine/ Finished ro	Rambo/wo	1	No
	Covered W	No	No facility/ Finished ro	Rambo/wo	1	Yes
	Protected I	No	Pit Latrine/ Finished ro	Rambo/wo	1	Yes
	Covered W	No	Pit Latrine/ Finished ro	Rambo/wo	1	Yes
	Piped outsi	No	Pit Latrine/ Finished ro	Rambo/wo	1	Yes
	Piped outsi	No	Pit Latrine/ Finished ro	Rambo/wo	1	No
	Piped outsi	No	Pit Latrine/ Finished ro	Stone with	2	Yes
	Protected S	No	Pit Latrine/ Finished ro	Rambo/wo	1	Yes
	Piped outsi	No	Pit Latrine/ Finished ro	Rambo/wo	1	Yes
	Piped outsi	No	Pit Latrine/ Finished ro	Rambo/wo	0	No
	Covered W	No	No facility/ Finished ro	Rambo/wo	1	Yes
	Piped outsi	No	Pit Latrine/ Finished ro	Rambo/wo	0	Yes
	Covered W	No	Pit Latrine/ Finished ro	Rambo/wo	1	Yes
	Piped outsi	No	Pit Latrine/ Finished ro	Rambo/wo	2	Yes
	Piped outsi	No	No facility/ Finished ro	Rambo/wo	1	Yes
	Piped outsi	No	No facility/ Finished ro	Rambo/wo	0	Yes
	Piped outsi	No	Pit Latrine/ Finished ro	Rambo/wo	1	No
	Open Sprin	No	Pit Latrine/ Finished ro	Rambo/wo	1	Yes
	Piped outsi	No	Pit Latrine/ Finished ro	Rambo/wo	1	Yes
	Piped outsi	No	Pit Latrine/ Finished ro	Rambo/wo	1	No
	Piped outsi	No	Pit Latrine/ Finished ro	Rambo/wo	2	No
	Covered W	No	No facility/ Finished ro	Rambo/wo	1	Yes
	Piped outsi	No	Pit Latrine/ Thatch/lea	Rambo/wo	1	Yes
	Piped outsi	No	Pit Latrine/ Finished ro	Rambo/wo	1	Yes
	Piped outsi	No	Pit Latrine/ Finished ro	Rambo/wo	1	Yes
	Piped into	No	Pit Latrine/ Finished ro	Rambo/wo	0	No
	Protected I	No	Pit Latrine/ Thatch/lea	Rambo/wo	0	No
	Piped outsi	No	Pit Latrine/ Finished ro	Rambo/wo	0	Yes
	Piped outsi	No	No facility/ Finished ro	Rambo/wo	1	Yes
	Piped outsi	No	No facility/ Finished ro	Rambo/wo	1	Yes
	Piped outsi	No	Pit Latrine/ Finished ro	Rambo/wo	0	No
	Piped outsi	No	Pit Latrine/ Finished ro	Rambo/wo	1	Yes
	Piped outsi	No	No facility/ Finished ro	Rambo/wo	1	Yes
	Piped outsi	No	No facility/ Finished ro	Rambo/wo	1	Yes
	Open Sprin	No	No facility/ Finished ro	Rambo/wo	1	Yes
2	Open Sprin	No	No facility/ Finished ro	Rambo/wo	1	Yes
	Open Sprin	No	No facility/ Finished ro	Rambo/wo	0	Yes
	Piped outsi	No	Pit Latrine/ Wood plan	Rambo/wo	1	No
1	Piped outsi	No	Pit Latrine/ Finished ro	Rambo/wo	0	No
	Open Sprin	No	No facility/ Roofing shi	Rambo/wo	0	Yes
	Open Well	No	Pit Latrine/ Finished ro	Rambo/wo	0	No
	River	No	No facility/ Finished ro	Rambo/wo	0	No
	Covered W	No	Pit Latrine/ Finished ro	Rambo/wo	0	No
	Open Sprin	No	Pit Latrine/ Finished ro	Rambo/wo	0	Yes
1	River	No	Pit Latrine/ Finished ro	Rambo/wo	0	Yes
	Open Sprin	No	No facility/ Finished ro	Rambo/wo	0	No
	River	No	No facility/ Finished ro	Rambo/wo	0	Yes
2	Piped outsi	No	No facility/ Finished ro	Rambo/wo	0	No
	River	No	No facility/ Finished ro	Rambo/wo	0	Yes

For peer review only

	Piped outsi	No	No facility/ Finished ro	Rambo/wo	0	Yes
	River	No	Pit Latrine/ Finished ro	Rambo/wo	0	No
	Piped outsi	No	No facility/ Finished ro	Rambo/wo	0	Yes
	Open Sprin	No	Pit Latrine/ Thatch/lea	Rambo/wo	0	No
	Open Sprin	No	No facility/ Finished ro	Rambo/wo	0	Yes
	Piped outsi	No	No facility/ Finished ro	Rambo/wo	1	Yes
	Open Sprin	No	Pit Latrine/ Finished ro	Rambo/wo	1	Yes
	Protected S	No	No facility/ Finished ro	Rambo/wo	0	Yes
	River	No	Pit Latrine/ Finished ro	Rambo/wo	0	Yes
	Open Sprin	No	Pit Latrine/ Finished ro	Rambo/wo	1	No
	Piped outsi	No	Pit Latrine/ Finished ro	Rambo/wo	1	No
	River	No	No facility/ Finished ro	Rambo/wo	1	No
	River	No	No facility/ Finished ro	Rambo/wo	1	Yes
	River	No	Pit Latrine/ Finished ro	Rambo/wo	0	Yes
	Open Sprin	No	No facility/ Finished ro	Rambo/wo	1	No
	Open Well	No	No facility/ Finished ro	Rambo/wo	0	No
	Open Sprin	No	Pit Latrine/ Finished ro	Rambo/wo	1	No
	Open Sprin	No	Pit Latrine/ Finished ro	Rambo/wo	1	Yes
	Open Well	No	No facility/ Finished ro	Rambo/wo	0	Yes
	Open Sprin	No	No facility/ Finished ro	Rambo/wo	0	Yes
	River	No	No facility/ Finished ro	Rambo/wo	1	No
	Protected S	No	No facility/ Finished ro	Rambo/wo	0	Yes
	Open Sprin	No	No facility/ Finished ro	Rambo/wo	1	Yes
	Open Well	No	No facility/ Finished ro	Rambo/wo	1	No
	River	No	No facility/ Finished ro	Rambo/wo	1	No
	Piped outsi	No	Pit Latrine/ Finished ro	Rambo/wo	0	Yes
1	Open Sprin	No	No facility/ Finished ro	Rambo/wo	0	Yes
	Open Well	No	No facility/ Finished ro	Rambo/wo	0	Yes
	Open Sprin	No	No facility/ Finished ro	Rambo/wo	0	Yes
	Open Sprin	No	No facility/ Finished ro	Rambo/wo	0	Yes
	Open Sprin	No	Pit Latrine/ Finished ro	Rambo/wo	1	Yes
	Open Well	No	No facility/ Finished ro	Rambo/wo	0	No
	Open Sprin	No	Pit Latrine/ Finished ro	Rambo/wo	0	No
3	Open Sprin	No	No facility/ Finished ro	Rambo/wo	0	Yes
	Piped outsi	No	Pit Latrine/ Finished ro	Rambo/wo	0	Yes
	Protected I	No	Pit Latrine/ Finished ro	Rambo/wo	0	No
	Open Sprin	No	Pit Latrine/ Finished ro	Rambo/wo	0	No
	Open Well	No	Pit Latrine/ Finished ro	Rambo/wo	1	Yes
	Piped outsi	No	No facility/ Finished ro	Rambo/wo	0	Yes
	Piped outsi	No	Pit Latrine/ Finished ro	Rambo/wo	0	Yes
	Open Sprin	No	Pit Latrine/ Finished ro	Rambo/wo	0	Yes
	River	No	No facility/ Finished ro	Rambo/wo	0	Yes
	Open Well	No	No facility/ Finished ro	Rambo/wo	0	Yes
	Open Sprin	No	No facility/ Finished ro	Rambo/wo	0	Yes
	Open Sprin	No	No facility/ Finished ro	Rambo/wo	0	Yes
2	River	No	Pit Latrine/ Finished ro	Rambo/wo	1	Yes
	River	No	No facility/ Finished ro	Rambo/wo	1	Yes
	Protected I	No	No facility/ Finished ro	Rambo/wo	1	No
2	Protected S	No	Pit Latrine/ Finished ro	Rambo/wo	1	Yes
	Piped into	No	Pit Latrine/ Finished ro	Rambo/wo	1	No
	Piped outsi	No	Pit Latrine/ Finished ro	Rambo/wo	1	No
	Protected I	No	No facility/ Finished ro	Rambo/wo	1	Yes

For peer review only

	Protected I	No		Pit Latrine/Finished ro	Rambo/wo	1	No
	Open Sprin	No		Pit Latrine/Finished ro	Rambo/wo	0	No
	Protected S	No		Pit Latrine/Finished ro	Rambo/wo	1	No
	Protected S	No		No facility/Finished ro	Rambo/wo	1	No
	Protected I	No		No facility/Finished ro	Rambo/wo	1	Yes
	Piped outsi	No		Pit Latrine/Finished ro	Rambo/wo	1	No
	River	No		Pit Latrine/Finished ro	Rambo/wo	0	Yes
	Piped outsi	No		Pit Latrine/Finished ro	Rambo/wo	1	Yes
1	Protected S	No		No facility/Finished ro	Rambo/wo	1	No
	Protected S	No		Pit Latrine/Finished ro	Rambo/wo	1	No
	Covered W	No		Pit Latrine/Finished ro	Rambo/wo	1	No
2	Protected S	Yes	Add bleach	Pit Latrine/Finished ro	Rambo/wo	0	No
	Open Sprin	No		Pit Latrine/Finished ro	Rambo/wo	0	Yes
	Protected I	No		Pit Latrine/Finished ro	Rambo/wo	1	No
	Piped outsi	No		Pit Latrine/Finished ro	Stone with	0	No
	Open Sprin	No		Pit Latrine/Finished ro	Rambo/wo	0	Yes
	Piped outsi	No		Pit Latrine/Finished ro	Rambo/wo	1	No
	Protected I	No		No facility/Finished ro	Rambo/wo	0	Yes
1	Piped outsi	Yes	Add bleach	Pit Latrine/Finished ro	Rambo/wo	2	Yes
	Protected S	No		Pit Latrine/Finished ro	Rambo/wo	1	No
	Piped outsi	No		Pit Latrine/Finished ro	Rambo/wo	2	Yes
	Piped outsi	No		Pit Latrine/Finished ro	Rambo/wo	1	Yes
	Covered W	No		Pit Latrine/Finished ro	Rambo/wo	1	No
	Piped outsi	No		Pit Latrine/Finished ro	Rambo/wo	1	No
	Piped outsi	No		Pit Latrine/Finished ro	Rambo/wo	0	Yes
	Piped outsi	No		Pit Latrine/Finished ro	Rambo/wo	1	Yes
	Protected I	No		No facility/Finished ro	Rambo/wo	1	No
	Piped outsi	No		Pit Latrine/Finished ro	Rambo/wo	1	No
	Piped outsi	No		No facility/Finished ro	Rambo/wo	1	No
	Protected S	No		Pit Latrine/Finished ro	Rambo/wo	1	No
1	Piped outsi	Yes	Add bleach	Pit Latrine/Finished ro	Rambo/wo	1	No
	Protected S	No		Pit Latrine/Finished ro	Rambo/wo	0	No
	Protected S	No		Pit Latrine/Finished ro	Rambo/wo	1	No
	Protected S	No		No facility/Finished ro	Rambo/wo	1	No
1	Covered W	No		Pit Latrine/Finished ro	Rambo/wo	1	No
	Protected I	No		Pit Latrine/Finished ro	Rambo/wo	1	No
2	Open Sprin	No		Pit Latrine/Finished ro	Rambo/wo	0	No
	Piped outsi	No		Pit Latrine/Finished ro	Rambo/wo	1	No
	Protected S	No		No facility/Finished ro	Rambo/wo	0	Yes
	Covered W	No		Pit Latrine/Finished ro	Rambo/wo	1	No
	Piped outsi	No		Pit Latrine/Finished ro	Rambo/wo	1	No
	Protected I	No		No facility/Finished ro	Rambo/wo	0	Yes
1	Protected S	No		Pit Latrine/Finished ro	Rambo/wo	2	No
	Piped outsi	No		Pit Latrine/Finished ro	Rambo/wo	1	Yes
	Protected S	No		Pit Latrine/Finished ro	Rambo/wo	1	No
	Piped outsi	No		Pit Latrine/Finished ro	Rambo/wo	0	No
	Protected S	No		Pit Latrine/Finished ro	Rambo/wo	1	No
	Protected S	No		Pit Latrine/Finished ro	Rambo/wo	2	No
	Protected I	No		No facility/Finished ro	Rambo/wo	0	No
	Protected S	No		Pit Latrine/Finished ro	Rambo/wo	0	No
	Covered W	No		Pit Latrine/Finished ro	Rambo/wo	1	Yes
	Covered W	No		Pit Latrine/Wood plan	Rambo/wo	2	Yes

For peer review only

	Piped outsi	No		Pit Latrine/Finished ro	Rambo/wo	1	No
	Covered W	No		Pit Latrine/Finished ro	Rambo/wo	1	No
	River	No		Pit Latrine/Finished ro	Rambo/wo	0	No
	Protected I	No		Pit Latrine/Finished ro	Rambo/wo	3	No
	Piped outsi	No		No facility/Finished ro	Rambo/wo	1	No
	Piped outsi	No		Pit Latrine/Finished ro	Rambo/wo	1	No
	Protected S	No		Pit Latrine/Finished ro	Rambo/wo	0	No
	Piped outsi	No		Pit Latrine/Finished ro	Rambo/wo	1	No
	Covered W	No		Pit Latrine/Finished ro	Rambo/wo	1	No
	Piped outsi	No		Pit Latrine/Finished ro	Rambo/wo	1	Yes
	Protected S	No		Pit Latrine/Finished ro	Rambo/wo	2	No
	Protected S	No		Pit Latrine/Finished ro	Rambo/wo	1	No
	Covered W	No		No facility/Finished ro	Rambo/wo	1	No
	Covered W	No		No facility/Finished ro	Rambo/wo	0	No
	Covered W	No		Pit Latrine/Finished ro	Rambo/wo	2	Yes
	Piped outsi	No		Pit Latrine/Finished ro	Rambo/wo	1	No
	Piped outsi	No		Pit Latrine/Finished ro	Rambo/wo	1	Yes
	Open Sprin	No		Pit Latrine/Finished ro	Rambo/wo	0	No
	Covered W	No		Pit Latrine/Finished ro	Rambo/wo	1	No
	Protected S	No		Pit Latrine/Finished ro	Stone with	0	No
	Piped outsi	No		Pit Latrine/Finished ro	Rambo/wo	1	No
	Piped outsi	Yes	Add bleach	Pit Latrine/Finished ro	Rambo/wo	2	Yes
	Piped outsi	No		Pit Latrine/Finished ro	Rambo/wo	0	No
	Covered W	No		Pit Latrine/Finished ro	Rambo/wo	0	No
	Protected S	No		No facility/Finished ro	Rambo/wo	1	No
	Piped outsi	No		Pit Latrine/Finished ro	Rambo/wo	1	Yes
	Protected I	No		Pit Latrine/Finished ro	Rambo/wo	1	No
	Piped outsi	No		Pit Latrine/Finished ro	Rambo/wo	0	No
	Protected S	No		Pit Latrine/Finished ro	Rambo/wo	0	No
	Piped outsi	No		Pit Latrine/Finished ro	Rambo/wo	0	No
	Covered W	Yes	Add bleach	Pit Latrine/Finished ro	Rambo/wo	0	No
	Covered W	Yes	Boil	No facility/Finished ro	Rambo/wo	0	No
	Protected I	No		Pit Latrine/Finished ro	Rambo/wo	1	Yes
1	Piped outsi	No		Pit Latrine/Finished ro	Rambo/wo	0	Yes
	Protected I	No		Pit Latrine/Finished ro	Rambo/wo	1	Yes
	Protected S	No		Pit Latrine/Finished ro	Rambo/wo	1	No
	Covered W	Yes	Add bleach	Pit Latrine/Finished ro	Rambo/wo	1	No
	Covered W	No		Pit Latrine/Finished ro	Rambo/wo	0	No
	Piped outsi	No		Pit Latrine/Finished ro	Rambo/wo	1	No
	Protected S	No		Pit Latrine/Finished ro	Rambo/wo	1	No
1	Piped outsi	No		Pit Latrine/Finished ro	Rambo/wo	0	Yes
	Open Sprin	No		Pit Latrine/Finished ro	Rambo/wo	1	Yes
	Covered W	Yes	Use water t	Pit Latrine/Finished ro	Rambo/wo	1	No
	Open Sprin	No		Pit Latrine/Finished ro	Rambo/wo	0	Yes
	Covered W	No		Pit Latrine/Finished ro	Rambo/wo	0	No
1	Piped outsi	No		No facility/Finished ro	Rambo/wo	0	Yes
1	Piped outsi	No		Pit Latrine/Finished ro	Rambo/wo	1	No
	Protected I	No		Pit Latrine/Finished ro	Rambo/wo	1	No
1	Covered W	No		Pit Latrine/Finished ro	Rambo/wo	0	No
1	River	No		Pit Latrine/Finished ro	Rambo/wo	0	No
	Protected S	No		Pit Latrine/Finished ro	Rambo/wo	1	No
	Piped outsi	No		Pit Latrine/Finished ro	Rambo/wo	1	No

For peer review only

	Protected I	No		Pit Latrine/	Finished ro	Rambo/wo	1	Yes
	Protected I	No		No facility/	Finished ro	Rambo/wo	0	Yes
	Covered W	No		No facility/	Finished ro	Rambo/wo	0	No
	Piped outsi	No		Pit Latrine/	Finished ro	Rambo/wo	0	Yes
	Piped outsi	No		Pit Latrine/	Finished ro	Rambo/wo	1	No
	Protected I	No		No facility/	Finished ro	Rambo/wo	1	Yes
	Piped outsi	Yes	Add bleach	Pit Latrine/	Finished ro	Rambo/wo	0	No
	Piped outsi	No		Pit Latrine/	Finished ro	Rambo/wo	0	No
	Covered W	No		Pit Latrine/	Finished ro	Rambo/wo	1	No
	Piped outsi	No		Pit Latrine/	Finished ro	Rambo/wo	0	Yes
	Protected I	No		No facility/	Finished ro	Rambo/wo	1	Yes
	Covered W	Yes	Add bleach	Pit Latrine/	Finished ro	Rambo/wo	1	No
	Protected I	No		Pit Latrine/	Finished ro	Rambo/wo	0	No
	Covered W	No		No facility/	Finished ro	Rambo/wo	0	No
	Piped outsi	No		Pit Latrine/	Finished ro	Rambo/wo	0	No
	Protected I	No		Pit Latrine/	Finished ro	Rambo/wo	0	No
	Piped outsi	No		Pit Latrine/	Finished ro	Rambo/wo	0	No
	Piped outsi	Yes	Add bleach	Pit Latrine/	Finished ro	Rambo/wo	1	No
	Protected S	Yes	Add bleach	Pit Latrine/	Finished ro	Rambo/wo	2	No
	Protected I	No		Pit Latrine/	Finished ro	Rambo/wo	1	Yes
	Piped outsi	No		Pit Latrine/	Finished ro	Rambo/wo	1	No
1	Piped outsi	No		Pit Latrine/	Finished ro	Stone with	1	No
	Protected S	No		Pit Latrine/	Finished ro	Rambo/wo	1	No
1	Covered W	No		Pit Latrine/	Finished ro	Rambo/wo	1	No
	Open Sprin	No		No facility/	Finished ro	Rambo/wo	0	Yes
	Covered W	Yes	Other	Pit Latrine/	Finished ro	Rambo/wo	1	Yes
	Covered W	No		Pit Latrine/	Finished ro	Rambo/wo	1	No
	Piped outsi	No		Pit Latrine/	Finished ro	Rambo/wo	1	Yes
3	Protected S	No		Pit Latrine/	Finished ro	Rambo/wo	0	Yes
	Covered W	No		Pit Latrine/	Finished ro	Stone with	1	No
	Open Sprin	No		Pit Latrine/	Finished ro	Rambo/wo	1	No
	River	No		Pit Latrine/	Finished ro	Rambo/wo	1	Yes
	Piped outsi	No		No facility/	Finished ro	Rambo/wo	1	Yes
	River	No		No facility/	Finished ro	Rambo/wo	1	Yes
	Piped outsi	No		Pit Latrine/	Finished ro	Rambo/wo	3	No
	Piped outsi	No		Pit Latrine/	Finished ro	Rambo/wo	1	Yes
	Piped into (	Yes	Boil	No facility/	Finished ro	Rambo/wo	3	No
	Piped outsi	No		Pit Latrine/	Finished ro	Rambo/wo	2	Yes
	Piped outsi	No		Pit Latrine/	Finished ro	Rambo/wo	1	Yes
	Piped outsi	No		Pit Latrine/	Finished ro	Other	3	No
	Piped outsi	No		No facility/	Finished ro	Rambo/wo	1	Yes
	Piped outsi	No		No facility/	Finished ro	Rambo/wo	2	Yes
	Piped outsi	No		Pit Latrine/	Finished ro	Rambo/wo	3	Yes
	Piped outsi	No		Pit Latrine/	Finished ro	Rambo/wo	1	Yes
	Piped outsi	No		No facility/	Finished ro	Rambo/wo	2	Yes
	Piped outsi	No		Pit Latrine/	Finished ro	Rambo/wo	1	No
	Protected I	Yes	Boil	Pit Latrine/	Finished ro	Rambo/wo	1	Yes
	Piped outsi	No		No facility/	Finished ro	Rambo/wo	1	Yes
	Piped outsi	No		Pit Latrine/	Finished ro	Rambo/wo	1	Yes
1	River	No		No facility/	Finished ro	Rambo/wo	1	Yes
	Piped outsi	No		Pit Latrine/	Finished ro	Rambo/wo	1	Yes
	Piped outsi	No		Pit Latrine/	Finished ro	Rambo/wo	2	Yes

For peer review only

	Piped into (	No		Pit Latrine/	Finished ro	Rambo/wo	3	No
	Piped outsi	No		Pit Latrine/	Finished ro	Rambo/wo	1	Yes
	Piped outsi	No		Pit Latrine/	Finished ro	Rambo/wo	2	Yes
	Piped into (	No		Ventilated	Finished ro	Rambo/wo	1	No
1	Piped outsi	No		Pit Latrine/	Finished ro	Rambo/wo	1	Yes
	Piped outsi	No		Pit Latrine/	Finished ro	Rambo/wo	1	Yes
	Piped outsi	No		Pit Latrine/	Finished ro	Rambo/wo	1	Yes
	Piped outsi	No		No facility/	Finished ro	Rambo/wo	1	Yes
	Piped into (	No		Pit Latrine/	Finished ro	Stone with	1	Yes
	Piped outsi	No		Pit Latrine/	Finished ro	Cement	1	Yes
	Piped outsi	No		Pit Latrine/	Finished ro	Rambo/wo	1	Yes
	Piped outsi	Yes	Boil	No facility/	Finished ro	Rambo/wo	2	No
	Piped outsi	No		Pit Latrine/	Finished ro	Rambo/wo	1	Yes
	Piped outsi	No		Pit Latrine/	Finished ro	Rambo/wo	1	Yes
	Piped outsi	No		No facility/	Finished ro	Rambo/wo	1	Yes
	Piped outsi	No		Ventilated	Finished ro	Cement	1	No
	Piped into (	No		Pit Latrine/	Finished ro	Stone with	1	Yes
	Piped outsi	No		No facility/	Finished ro	Rambo/wo	1	Yes
	Piped outsi	No		No facility/	Finished ro	Rambo/wo	2	Yes
	Piped into (	No		Ventilated	Finished ro	Cement	1	No
	River	No		Pit Latrine/	Finished ro	Rambo/wo	1	Yes
	Piped outsi	No		No facility/	Finished ro	Rambo/wo	1	Yes
	Piped outsi	No		No facility/	Finished ro	Rambo/wo	1	Yes
	Piped into (	No		Ventilated	Finished ro	Cement	2	No
	Piped into (	No		Pit Latrine/	Finished ro	Stone with	3	No
	Piped outsi	No		No facility/	Finished ro	Rambo/wo	2	No
	Piped outsi	No		No facility/	Finished ro	Rambo/wo	2	Yes
	Piped outsi	No		No facility/	Finished ro	Rambo/wo	0	Yes
	Piped outsi	Yes	Boil	Pit Latrine/	Finished ro	Rambo/wo	3	No
	Piped into (	No		Ventilated	Finished ro	Cement	1	Yes
	Piped outsi	No		No facility/	Finished ro	Rambo/wo	1	Yes
	Piped into (	Yes	Boil	Pit Latrine/	Finished ro	Rambo/wo	2	No
	Piped into (	No		No facility/	Finished ro	Cement	2	No
	Piped into (	Yes	Boil	Ventilated	Finished ro	Cement	2	No
	Piped outsi	No		No facility/	Finished ro	Rambo/wo	1	Yes
	Piped outsi	No		No facility/	Finished ro	Rambo/wo	1	Yes
	River	No		Pit Latrine/	Finished ro	Rambo/wo	1	Yes
	Piped outsi	No		Pit Latrine/	Finished ro	Rambo/wo	1	Yes
	Piped into (	No		Ventilated	Finished ro	Rambo/wo	1	No
	Piped into (	Yes	Boil	Pit Latrine/	Roofing shi	Rambo/wo	2	No
	Piped outsi	No		No facility/	Finished ro	Cement	2	No
	Piped outsi	Yes	Boil	Pit Latrine/	Finished ro	Rambo/wo	3	No
	Piped into (	No		Ventilated	Finished ro	Stone with	1	Yes
	River	No		Pit Latrine/	Thatch/lea	Rambo/wo	0	Yes
	Piped outsi	Yes	Boil	No facility/	Finished ro	Rambo/wo	1	Yes
	Piped outsi	No		Pit Latrine/	Finished ro	Rambo/wo	1	Yes
	Piped into (	No		Ventilated	Finished ro	Stone with	1	No
	Piped outsi	No		No facility/	Finished ro	Rambo/wo	1	Yes
	Piped outsi	Yes	Boil	Pit Latrine/	Finished ro	Rambo/wo	2	Yes
	Piped outsi	No		No facility/	Finished ro	Rambo/wo	1	Yes
	Piped outsi	No		No facility/	Finished ro	Rambo/wo	1	Yes
	Protected I	No		Pit Latrine/	Finished ro	Rambo/wo	2	No

For peer review only

	Piped outsi	No		Pit Latrine/	Finished ro	Rambo/wo	2	Yes
	Piped outsi	No		Pit Latrine/	Finished ro	Rambo/wo	1	No
	Piped into (	No		Pit Latrine/	Finished ro	Stone with	1	Yes
	Piped outsi	No		Pit Latrine/	Finished ro	Rambo/wo	1	Yes
	Piped outsi	No		Pit Latrine/	Finished ro	Rambo/wo	1	Yes
	Piped into (	Yes	Boil	Pit Latrine/	Finished ro	Rambo/wo	2	No
	Piped outsi	No		No facility/	Finished ro	Rambo/wo	2	Yes
	Piped outsi	Yes	Boil	No facility/	Finished ro	Rambo/wo	2	No
	Piped outsi	No		No facility/	Finished ro	Rambo/wo	1	Yes
	Piped outsi	No		Pit Latrine/	Finished ro	Rambo/wo	1	Yes
	Piped into (	Yes	Boil	Pit Latrine/	Finished ro	Rambo/wo	2	No
	Piped outsi	No		No facility/	Finished ro	Rambo/wo	2	Yes
	Piped outsi	Yes	Add bleach	Pit Latrine/	Finished ro	Rambo/wo	2	Yes
	Piped outsi	Yes	Add bleach	No facility/	Finished ro	Rambo/wo	2	Yes
	Piped outsi	No		Pit Latrine/	Finished ro	Cement	1	Yes
	Piped outsi	No		No facility/	Finished ro	Rambo/wo	1	Yes
	Piped outsi	No		Pit Latrine/	Finished ro	Rambo/wo	1	Yes
	Piped into (	Yes	Boil	Pit Latrine/	Finished ro	Rambo/wo	2	No
	Piped outsi	No		No facility/	Finished ro	Rambo/wo	1	Yes
	Piped into (	No		Pit Latrine/	Finished ro	Rambo/wo	2	Yes
1	Protected I	No		Pit Latrine/	Finished ro	Rambo/wo	1	Yes
	Protected S	No		No facility/	Finished ro	Rambo/wo	0	Yes
	Piped outsi	No		Pit Latrine/	Finished ro	Rambo/wo	1	Yes
	Piped outsi	No		Pit Latrine/	Finished ro	Rambo/wo	1	Yes
	Protected S	No		Pit Latrine/	Finished ro	Rambo/wo	1	Yes
	Open Sprin	No		Pit Latrine/	Finished ro	Rambo/wo	1	Yes
	Piped outsi	No		Pit Latrine/	Finished ro	Rambo/wo	1	Yes
1	Protected I	Yes	Use water t	Pit Latrine/	Finished ro	Rambo/wo	2	Yes
	Piped outsi	No		Pit Latrine/	Finished ro	Rambo/wo	1	Yes
	Protected I	Yes	Add bleach	Pit Latrine/	Finished ro	Rambo/wo	0	No
2	Protected I	Yes	Add bleach	No facility/	Finished ro	Rambo/wo	2	Yes
	Open Sprin	No		Pit Latrine/	Finished ro	Rambo/wo	1	Yes
	Protected I	No		Pit Latrine/	Finished ro	Rambo/wo	1	Yes
	Piped outsi	No		No facility/	Finished ro	Rambo/wo	1	Yes
3	Protected I	No		Pit Latrine/	Finished ro	Rambo/wo	1	Yes
	Protected S	No		Pit Latrine/	Finished ro	Rambo/wo	2	Yes
	Piped outsi	No		Pit Latrine/	Finished ro	Rambo/wo	1	No
1	Protected I	No		Pit Latrine/	Finished ro	Rambo/wo	1	Yes
	Protected I	No		No facility/	Finished ro	Rambo/wo	1	Yes
	Piped outsi	No		Pit Latrine/	Finished ro	Rambo/wo	1	Yes
	Piped outsi	No		No facility/	Finished ro	Rambo/wo	1	Yes
1	Protected I	Yes	Add bleach	Pit Latrine/	Finished ro	Rambo/wo	1	Yes
1	Protected S	No		Pit Latrine/	Finished ro	Rambo/wo	0	No
	Piped outsi	No		No facility/	Finished ro	Rambo/wo	1	Yes
2	Protected I	Yes	Add bleach	Pit Latrine/	Finished ro	Rambo/wo	1	Yes
	Piped outsi	No		No facility/	Finished ro	Rambo/wo	1	Yes
	Protected I	Yes	Add bleach	Pit Latrine/	Finished ro	Rambo/wo	1	Yes
	Protected S	No		Pit Latrine/	Finished ro	Rambo/wo	1	Yes
3	Protected I	Yes	Add bleach	Pit Latrine/	Finished ro	Rambo/wo	1	Yes
	Protected I	Yes	Add bleach	Pit Latrine/	Finished ro	Rambo/wo	0	Yes
2	Protected I	Yes	Add bleach	Pit Latrine/	Finished ro	Rambo/wo	1	Yes
	Protected I	No		Pit Latrine/	Finished ro	Rambo/wo	0	No

For peer review only

	Piped outsi	No		No facility/	Finished ro	Rambo/wo	0	Yes
	Piped outsi	No		Pit Latrine/	Finished ro	Rambo/wo	1	Yes
	Open Sprin	No		Pit Latrine/	Finished ro	Rambo/wo	1	No
	Open Sprin	No		Pit Latrine/	Finished ro	Rambo/wo	1	Yes
	Piped outsi	No		Pit Latrine/	Finished ro	Rambo/wo	1	Yes
1	Piped outsi	No		No facility/	Finished ro	Rambo/wo	1	No
	Piped outsi	No		Pit Latrine/	Finished ro	Rambo/wo	1	Yes
	Piped outsi	No		Pit Latrine/	Finished ro	Rambo/wo	1	Yes
	Piped outsi	No		Pit Latrine/	Finished ro	Rambo/wo	1	Yes
	Piped outsi	No		Pit Latrine/	Finished ro	Rambo/wo	1	Yes
2	Protected S	No		Pit Latrine/	Finished ro	Rambo/wo	1	Yes
	Open Sprin	No		Pit Latrine/	Finished ro	Rambo/wo	1	Yes
	Covered W	Yes	Add bleach	Pit Latrine/	Finished ro	Rambo/wo	2	Yes
	Piped into	No		Pit Latrine/	Finished ro	Rambo/wo	1	Yes
	Piped outsi	No		Pit Latrine/	Finished ro	Rambo/wo	1	Yes
	Protected I	Yes	Add bleach	Pit Latrine/	Finished ro	Rambo/wo	1	No
	Piped outsi	No		Pit Latrine/	Finished ro	Rambo/wo	1	Yes
	Protected S	No		Pit Latrine/	Finished ro	Rambo/wo	1	Yes
	Piped outsi	No		Pit Latrine/	Finished ro	Rambo/wo	1	Yes
	Open Sprin	No		No facility/	Finished ro	Rambo/wo	0	Yes
	Piped outsi	No		Pit Latrine/	Finished ro	Rambo/wo	1	Yes
	Piped outsi	No		Pit Latrine/	Finished ro	Rambo/wo	1	Yes
	Protected I	No		Pit Latrine/	Finished ro	Rambo/wo	1	Yes
	Open Sprin	No		No facility/	Finished ro	Rambo/wo	1	Yes
1	Piped outsi	No		Pit Latrine/	Finished ro	Rambo/wo	0	Yes
	Protected I	No		Pit Latrine/	Finished ro	Rambo/wo	1	Yes
	Piped outsi	No		Pit Latrine/	Finished ro	Rambo/wo	0	Yes
	Protected I	Yes	Add bleach	Pit Latrine/	Finished ro	Rambo/wo	1	Yes
	Protected S	No		No facility/	Finished ro	Rambo/wo	0	No
	Protected I	No		Pit Latrine/	Finished ro	Rambo/wo	1	Yes
1	Piped outsi	No		Pit Latrine/	Finished ro	Rambo/wo	1	Yes
	Protected S	No		No facility/	Finished ro	Rambo/wo	0	Yes
	Protected I	Yes	Add bleach	Pit Latrine/	Finished ro	Rambo/wo	2	Yes
	Protected S	No		No facility/	Finished ro	Rambo/wo	0	Yes
1	River	No		No facility/	Finished ro	Rambo/wo	0	Yes
	Open Sprin	No		No facility/	Finished ro	Rambo/wo	1	Yes
	Piped outsi	No		Pit Latrine/	Finished ro	Rambo/wo	0	Yes
	Protected S	No		No facility/	Finished ro	Rambo/wo	1	Yes
	Piped outsi	No		No facility/	Finished ro	Rambo/wo	1	No
	Piped outsi	No		Pit Latrine/	Finished ro	Rambo/wo	1	Yes
1	Open Sprin	Yes	Boil	Pit Latrine/	Finished ro	Rambo/wo	1	Yes
	Protected S	No		Pit Latrine/	Finished ro	Rambo/wo	0	Yes
	Protected I	No		Pit Latrine/	Finished ro	Rambo/wo	2	Yes
1	Protected S	No		No facility/	Finished ro	Rambo/wo	1	Yes
	Piped outsi	No		Pit Latrine/	Finished ro	Rambo/wo	1	No
	Piped outsi	No		Pit Latrine/	Finished ro	Rambo/wo	1	Yes
	Piped outsi	No		Pit Latrine/	Finished ro	Rambo/wo	1	No
2	Protected I	No		Pit Latrine/	Finished ro	Rambo/wo	1	Yes
	Protected S	No		Pit Latrine/	Finished ro	Rambo/wo	0	No
	Piped outsi	No		Pit Latrine/	Finished ro	Rambo/wo	1	No
	Protected I	No		Ventilated	Finished ro	Rambo/wo	1	Yes
	Piped into	No		Pit Latrine/	Finished ro	Rambo/wo	1	Yes

For peer review only

	Protected I	No		Pit Latrine/	Finished ro	Rambo/wo	1	Yes
	Open Sprin	No		No facility/	Finished ro	Rambo/wo	1	Yes
	Protected I	Yes	Add bleach	Pit Latrine/	Finished ro	Rambo/wo	1	No
	Piped outsi	No		No facility/	Finished ro	Rambo/wo	1	No
	Piped outsi	No		Pit Latrine/	Finished ro	Rambo/wo	1	Yes
	Piped outsi	No		Pit Latrine/	Finished ro	Rambo/wo	1	No
	Protected I	Yes	Add bleach	Pit Latrine/	Finished ro	Rambo/wo	1	Yes
	Piped outsi	No		No facility/	Finished ro	Rambo/wo	1	Yes
	Protected S	No		No facility/	Finished ro	Rambo/wo	0	Yes
	Protected I	Yes	Add bleach	Pit Latrine/	Finished ro	Rambo/wo	1	Yes
	Protected I	Yes	Add bleach	Pit Latrine/	Finished ro	Rambo/wo	1	Yes
	Piped outsi	No		Pit Latrine/	Finished ro	Rambo/wo	1	Yes
1	Protected S	No		Pit Latrine/	Finished ro	Rambo/wo	1	Yes
	Protected I	No		Pit Latrine/	Finished ro	Rambo/wo	1	Yes
	Protected I	No		Pit Latrine/	Finished ro	Rambo/wo	1	Yes
	Protected S	No		No facility/	Finished ro	Rambo/wo	0	Yes
	Protected I	No		Pit Latrine/	Finished ro	Rambo/wo	2	Yes
	Piped outsi	No		Pit Latrine/	Finished ro	Rambo/wo	1	Yes
	Piped outsi	No		Pit Latrine/	Finished ro	Rambo/wo	1	Yes
1	Piped outsi	No		No facility/	Finished ro	Rambo/wo	1	Yes
2	Piped outsi	No		No facility/	Finished ro	Rambo/wo	2	Yes
	Piped outsi	No		Pit Latrine/	Finished ro	Rambo/wo	1	No
	Piped outsi	No		No facility/	Thatch/lea	Rambo/wo	1	Yes
	Piped outsi	No		No facility/	Finished ro	Rambo/wo	2	No
	Piped outsi	No		No facility/	Thatch/lea	Rambo/wo	0	Yes
	Piped outsi	No		Pit Latrine/	Finished ro	Rambo/wo	1	No
	Piped outsi	No		No facility/	Thatch/lea	Rambo/wo	0	Yes
1	Piped outsi	No		Ventilated	Finished ro	Rambo/wo	1	No
	Piped outsi	No		Pit Latrine/	Finished ro	Rambo/wo	1	No
	Piped outsi	No		Ventilated	Finished ro	Cement	1	Yes
	Piped outsi	No		Ventilated	Thatch/lea	Rambo/wo	0	No
	Piped outsi	No		Ventilated	Finished ro	Rambo/wo	0	No
	Piped outsi	No		Pit Latrine/	Finished ro	Rambo/wo	1	Yes
	Piped outsi	No		Ventilated	Finished ro	Cement	1	Yes
	Piped outsi	No		Pit Latrine/	Thatch/lea	Rambo/wo	0	Yes
	Piped outsi	No		Pit Latrine/	Finished ro	Cement	0	Yes
	Piped outsi	No		Ventilated	Finished ro	Rambo/wo	1	No
	Piped outsi	No		Pit Latrine/	Finished ro	Rambo/wo	2	No
	Piped outsi	No		No facility/	Finished ro	Rambo/wo	0	No
	Piped outsi	No		Pit Latrine/	Finished ro	Rambo/wo	1	Yes
	Piped outsi	No		Pit Latrine/	Thatch/lea	Rambo/wo	0	Yes
	Piped outsi	No		Pit Latrine/	Finished ro	Stone with	1	Yes
	Piped outsi	No		Pit Latrine/	Finished ro	Rambo/wo	1	Yes
	Piped outsi	No		Pit Latrine/	Thatch/lea	Rambo/wo	2	Yes
	Piped outsi	No		Pit Latrine/	Finished ro	Rambo/wo	3	Yes
	Piped outsi	No		Pit Latrine/	Finished ro	Rambo/wo	1	No
	Piped outsi	No		Ventilated	Thatch/lea	Rambo/wo	0	Yes
	Piped outsi	No		No facility/	Finished ro	Rambo/wo	1	Yes
	Piped outsi	No		Pit Latrine/	Thatch/lea	Rambo/wo	0	Yes
	Piped outsi	No		Ventilated	Finished ro	Rambo/wo	1	Yes
	Piped outsi	No		Pit Latrine/	Finished ro	Rambo/wo	1	No
	Piped outsi	No		Pit Latrine/	Thatch/lea	Rambo/wo	0	No

For peer review only

	Piped outsi	No		Pit Latrine/	Finished ro	Rambo/wo	3	No
	Piped outsi	No		Ventilated	Finished ro	Cement	1	No
	Piped outsi	No		Pit Latrine/	Finished ro	Rambo/wo	2	Yes
	Piped outsi	No		Ventilated	Finished ro	Rambo/wo	1	Yes
	Open Sprin	No		No facility/	Finished ro	Rambo/wo	1	Yes
	Piped outsi	No		Pit Latrine/	Finished ro	Rambo/wo	1	Yes
	Protected S	No		No facility/	Finished ro	Rambo/wo	3	Yes
	Open Sprin	No		No facility/	Finished ro	Rambo/wo	1	Yes
	Piped outsi	No		Pit Latrine/	Finished ro	Rambo/wo	1	Yes
	Piped outsi	No		Pit Latrine/	Other	Rambo/wo	1	Yes
	Piped outsi	No		No facility/	Finished ro	Rambo/wo	1	Yes
	Protected I	No		No facility/	Finished ro	Rambo/wo	1	Yes
	Piped outsi	Yes	Add bleach	Pit Latrine/	Finished ro	Rambo/wo	3	Yes
1	Piped outsi	No		No facility/	Finished ro	Rambo/wo	1	Yes
	Piped outsi	No		No facility/	Finished ro	Rambo/wo	1	No
	Open Sprin	No		Pit Latrine/	Finished ro	Rambo/wo	1	Yes
1	Open Sprin	No		Pit Latrine/	Finished ro	Rambo/wo	0	Yes
1	Protected I	No		No facility/	Finished ro	Rambo/wo	1	Yes
	Open Sprin	No		Pit Latrine/	Finished ro	Rambo/wo	1	Yes
	Piped outsi	No		No facility/	Finished ro	Other	1	Yes
	Piped outsi	No		No facility/	Finished ro	Rambo/wo	2	Yes
	Open Sprin	No		Pit Latrine/	Finished ro	Rambo/wo	1	Yes
	Piped outsi	No		Pit Latrine/	Finished ro	Rambo/wo	1	Yes
	Piped outsi	No		Pit Latrine/	Finished ro	Rambo/wo	1	Yes
	Piped outsi	Yes	Boil	Pit Latrine/	Finished ro	Rambo/wo	2	Yes
	Protected S	No		No facility/	Finished ro	Rambo/wo	1	No
	Piped outsi	No		Pit Latrine/	Finished ro	Rambo/wo	1	Yes
	Open Sprin	No		No facility/	Finished ro	Rambo/wo	0	Yes
	Piped outsi	No		No facility/	Finished ro	Rambo/wo	1	Yes
1	Protected I	No		Pit Latrine/	Finished ro	Rambo/wo	1	Yes
	Open Sprin	No		Pit Latrine/	Finished ro	Rambo/wo	2	Yes
	Piped outsi	No		No facility/	Finished ro	Rambo/wo	1	Yes
	Piped outsi	Yes	Boil	Pit Latrine/	Finished ro	Rambo/wo	2	Yes
	Piped outsi	No		Pit Latrine/	Finished ro	Rambo/wo	0	Yes
1	Protected S	No		Pit Latrine/	Finished ro	Rambo/wo	1	Yes
3	Open Sprin	Yes	Strain it thr	Pit Latrine/	Finished ro	Rambo/wo	2	Yes
	Piped outsi	No		No facility/	Finished ro	Rambo/wo	1	Yes
	Piped outsi	No		Pit Latrine/	Finished ro	Rambo/wo	1	Yes
	Protected S	No		No facility/	Finished ro	Rambo/wo	1	Yes
	River	No		No facility/	Finished ro	Rambo/wo	1	Yes
	Open Sprin	No		Pit Latrine/	Finished ro	Rambo/wo	1	Yes
	Protected I	No		Pit Latrine/	Finished ro	Rambo/wo	0	Yes
	Protected S	No		Pit Latrine/	Finished ro	Rambo/wo	1	Yes
1	River	No		Pit Latrine/	Finished ro	Rambo/wo	1	Yes
	Piped into	Yes	Boil	Ventilated	Finished ro	Stone with	1	Yes
2	Open Sprin	No		Pit Latrine/	Finished ro	Rambo/wo	1	Yes
	Protected I	Yes	Add bleach	Pit Latrine/	Finished ro	Rambo/wo	1	Yes
	Open Sprin	No		Pit Latrine/	Finished ro	Rambo/wo	2	Yes
1	Open Well	No		Pit Latrine/	Finished ro	Rambo/wo	0	Yes
	Piped outsi	No		No facility/	Finished ro	Rambo/wo	0	Yes
	Piped outsi	Yes	Boil	Pit Latrine/	Thatch/lea	Rambo/wo	2	Yes
	Piped outsi	No		Pit Latrine/	Finished ro	Rambo/wo	1	Yes

For peer review only

	Piped outsi	No		Pit Latrine/Finished ro	Rambo/wo	1	Yes
	Piped outsi	No		No facility/ Finished ro	Rambo/wo	1	No
	Protected S	No		Pit Latrine/Finished ro	Rambo/wo	0	No
	Open Sprin	No		Pit Latrine/Finished ro	Rambo/wo	1	Yes
	River	No		Pit Latrine/Finished ro	Rambo/wo	0	Yes
	Piped outsi	Yes	Boil	Pit Latrine/Thatch/lea	Rambo/wo	3	No
	Piped outsi	No		No facility/ Thatch/lea	Rambo/wo	0	No
	Protected S	No		Pit Latrine/Finished ro	Rambo/wo	1	Yes
	Protected I	No		No facility/ Finished ro	Rambo/wo	1	Yes
	Piped into	No		Pit Latrine/Finished ro	Rambo/wo	1	Yes
	Piped outsi	Yes	Boil	Pit Latrine/Finished ro	Rambo/wo	3	Yes
	Piped outsi	No		Pit Latrine/Finished ro	Rambo/wo	1	Yes
	Piped outsi	No		No facility/ Finished ro	Other	0	Yes
	Protected S	No		No facility/ Finished ro	Rambo/wo	2	No
	Open Sprin	No		Pit Latrine/Finished ro	Rambo/wo	1	Yes
	Piped outsi	No		Pit Latrine/Finished ro	Rambo/wo	1	No
	Piped outsi	No		Pit Latrine/Finished ro	Rambo/wo	1	Yes
	Piped outsi	No		No facility/ Finished ro	Rambo/wo	2	Yes
	Protected S	No		Pit Latrine/Finished ro	Rambo/wo	1	No
	River	No		No facility/ Finished ro	Rambo/wo	2	Yes
	Protected S	No		Pit Latrine/Finished ro	Rambo/wo	1	Yes
	Protected S	Yes	Boil	Pit Latrine/Finished ro	Rambo/wo	2	Yes
	Open Sprin	No		Pit Latrine/Finished ro	Rambo/wo	1	Yes
	Protected S	No		Pit Latrine/Finished ro	Rambo/wo	1	Yes
	Protected S	No		Pit Latrine/Finished ro	Rambo/wo	1	Yes
	Piped outsi	No		Pit Latrine/Finished ro	Rambo/wo	1	No
	Open Sprin	No		Pit Latrine/Finished ro	Rambo/wo	1	Yes
	Piped outsi	No		No facility/ Finished ro	Rambo/wo	1	No
	Piped outsi	No		Pit Latrine/Finished ro	Rambo/wo	1	No
	Piped outsi	No		No facility/ Thatch/lea	Rambo/wo	1	No
	Protected S	No		No facility/ Finished ro	Rambo/wo	1	Yes
	Open Sprin	No		No facility/ Finished ro	Rambo/wo	1	No
	River	No		No facility/ Finished ro	Rambo/wo	1	Yes
	Protected S	No		Pit Latrine/Finished ro	Rambo/wo	1	Yes
	Protected S	No		Pit Latrine/Finished ro	Rambo/wo	1	No
	River	No		No facility/ Finished ro	Rambo/wo	0	Yes
	Protected S	No		No facility/ Finished ro	Rambo/wo	0	Yes
	Open Sprin	No		Pit Latrine/Finished ro	Rambo/wo	2	Yes
	River	No		No facility/ Finished ro	Rambo/wo	1	Yes
	Protected S	No		Pit Latrine/Finished ro	Rambo/wo	1	Yes
	Protected S	No		No facility/ Finished ro	Rambo/wo	1	No
	River	No		No facility/ Finished ro	Rambo/wo	0	No
	Protected S	No		Pit Latrine/Finished ro	Rambo/wo	1	Yes
	Protected S	No		Pit Latrine/Finished ro	Rambo/wo	0	No
	Open Sprin	No		Pit Latrine/Finished ro	Rambo/wo	0	Yes
	Protected S	No		Pit Latrine/Finished ro	Rambo/wo	0	Yes
1	Open Sprin	No		Pit Latrine/Finished ro	Rambo/wo	2	Yes
	River	No		Pit Latrine/Finished ro	Rambo/wo	1	No
	Piped outsi	No		No facility/ Finished ro	Rambo/wo	0	No
	Protected S	No		Pit Latrine/Finished ro	Rambo/wo	0	No
	River	No		No facility/ Finished ro	Rambo/wo	1	Yes
	River	No		Pit Latrine/Finished ro	Rambo/wo	0	Yes

For peer review only

	Open Sprin	No		Pit Latrine/	Finished ro	Rambo/wo	2	Yes
	Protected S	No		No facility/	Finished ro	Rambo/wo	0	No
	Open Sprin	No		No facility/	Finished ro	Rambo/wo	0	Yes
	River	No		No facility/	Finished ro	Rambo/wo	1	Yes
	River	No		No facility/	Finished ro	Rambo/wo	1	Yes
1	River	No		Pit Latrine/	Finished ro	Rambo/wo	1	Yes
	River	No		Pit Latrine/	Finished ro	Rambo/wo	2	Yes
	Protected S	No		No facility/	Finished ro	Rambo/wo	0	Yes
	Covered W	No		Pit Latrine/	Finished ro	Rambo/wo	2	No
2	Protected S	No		Pit Latrine/	Finished ro	Rambo/wo	2	Yes
	Protected S	No		No facility/	Finished ro	Rambo/wo	0	Yes
	River	No		No facility/	Finished ro	Rambo/wo	1	Yes
	Piped outsi	No		No facility/	Finished ro	Rambo/wo	0	No
	Protected S	No		Pit Latrine/	Finished ro	Rambo/wo	1	Yes
1	Protected S	No		Pit Latrine/	Finished ro	Rambo/wo	1	Yes
	Protected S	No		No facility/	Finished ro	Rambo/wo	1	Yes
	Protected S	No		Pit Latrine/	Finished ro	Rambo/wo	2	Yes
	River	No		No facility/	Finished ro	Rambo/wo	0	Yes
	River	No		No facility/	Thatch/lea	Rambo/wo	0	Yes
	River	No		No facility/	Finished ro	Rambo/wo	0	Yes
1	Protected S	No		No facility/	Finished ro	Rambo/wo	0	Yes
	River	No		No facility/	Finished ro	Rambo/wo	1	Yes
	Protected S	No		Pit Latrine/	Finished ro	Rambo/wo	2	Yes
1	Protected S	No		Pit Latrine/	Finished ro	Rambo/wo	1	Yes
	River	No		No facility/	Finished ro	Rambo/wo	1	No
	Open Sprin	No		No facility/	Finished ro	Rambo/wo	0	Yes
	River	No		No facility/	Finished ro	Rambo/wo	1	Yes
	Protected S	No		Pit Latrine/	Finished ro	Rambo/wo	1	Yes
	River	No		No facility/	Finished ro	Rambo/wo	0	Yes
	Protected S	No		Pit Latrine/	Finished ro	Rambo/wo	1	Yes
	River	No		No facility/	Finished ro	Rambo/wo	0	Yes
	Protected S	No		Pit Latrine/	Finished ro	Rambo/wo	2	Yes
1	Protected S	No		No facility/	Finished ro	Rambo/wo	1	Yes
	Open Sprin	No		No facility/	Finished ro	Rambo/wo	2	Yes
	Protected S	No		No facility/	Finished ro	Rambo/wo	1	No
	Protected S	No		No facility/	Finished ro	Rambo/wo	0	Yes
	Protected S	No		Pit Latrine/	Finished ro	Rambo/wo	1	Yes
	Piped outsi	No		Pit Latrine/	Finished ro	Rambo/wo	0	Yes
	Protected S	No		No facility/	Finished ro	Rambo/wo	1	Yes
	Piped outsi	No		No facility/	Finished ro	Cement	1	No
	River	No		Pit Latrine/	Finished ro	Rambo/wo	2	Yes
	Open Sprin	No		Pit Latrine/	Finished ro	Rambo/wo	0	Yes
	Piped into (	No		Pit Latrine/	Finished ro	Cement	1	Yes
	Piped outsi	No		Pit Latrine/	Finished ro	Cement	2	Yes
1	Piped into (	No		Ventilated	Finished ro	Rambo/wo	1	No
	Piped outsi	No		Pit Latrine/	Finished ro	Rambo/wo	1	No
	Piped outsi	No		Pit Latrine/	Finished ro	Cement	1	No
	Piped outsi	Yes	Boil	No facility/	Finished ro	Cement	1	Yes
	Piped outsi	No		Pit Latrine/	Finished ro	Rambo/wo	2	Yes
	Piped outsi	No		Pit Latrine/	Finished ro	Stone with	1	No
	Piped into (	No		Pit Latrine/	Finished ro	Cement	1	No
	Protected S	Yes	Boil	Pit Latrine/	Finished ro	Rambo/wo	2	Yes

For peer review only

	River	No		Pit Latrine/	Finished ro	Rambo/wo	0	Yes
	Piped into (	No		Pit Latrine/	Finished ro	Stone with	1	No
	Piped into (	No		Pit Latrine/	Finished ro	Rambo/wo	1	Yes
	Piped outsi	No		Pit Latrine/	Finished ro	Rambo/wo	1	No
	River	No		Pit Latrine/	Finished ro	Rambo/wo	0	No
	Piped outsi	No		Pit Latrine/	Finished ro	Rambo/wo	1	No
	Open Sprin	No		Pit Latrine/	Finished ro	Rambo/wo	1	No
	Piped outsi	No		Pit Latrine/	Finished ro	Rambo/wo	1	No
	Piped outsi	No		No facility/	Finished ro	Cement	1	Yes
	Piped into (	No		Pit Latrine/	Finished ro	Rambo/wo	0	No
	Piped outsi	No		Pit Latrine/	Finished ro	Rambo/wo	1	Yes
	Open Sprin	No		Pit Latrine/	Finished ro	Rambo/wo	2	Yes
	Protected S	No		Pit Latrine/	Finished ro	Rambo/wo	1	No
	Protected S	No		Pit Latrine/	Finished ro	Rambo/wo	1	Yes
	Piped outsi	No		Pit Latrine/	Thatch/lea	Rambo/wo	0	Yes
	Piped outsi	No		Pit Latrine/	Thatch/lea	Rambo/wo	0	Yes
1	Piped outsi	No		Pit Latrine/	Thatch/lea	Rambo/wo	0	No
	River	No		Pit Latrine/	Finished ro	Rambo/wo	1	Yes
	Piped into (	No		Pit Latrine/	Finished ro	Rambo/wo	0	Yes
	Piped into (	No		Pit Latrine/	Finished ro	Rambo/wo	1	No
	Piped into (	No		Pit Latrine/	Finished ro	Cement	2	No
	River	No		Pit Latrine/	Finished ro	Rambo/wo	1	Yes
	Piped into (	No		Ventilated	Finished ro	Cement	1	Yes
	Piped outsi	No		Pit Latrine/	Finished ro	Rambo/wo	1	Yes
	Open Sprin	No		No facility/	Finished ro	Rambo/wo	1	Yes
	Piped into (	No		Pit Latrine/	Finished ro	Stone with	1	No
	Piped outsi	No		Pit Latrine/	Finished ro	Rambo/wo	1	Yes
	Piped outsi	No		Pit Latrine/	Finished ro	Cement	1	No
	Piped into (	No		Pit Latrine/	Finished ro	Cement	1	Yes
	Piped outsi	No		Pit Latrine/	Finished ro	Stone with	1	Yes
	Piped outsi	No		Pit Latrine/	Finished ro	Rambo/wo	1	No
	Piped outsi	No		Pit Latrine/	Finished ro	Rambo/wo	1	Yes
	Piped outsi	No		Pit Latrine/	Finished ro	Rambo/wo	1	Yes
	Piped into (	No		Pit Latrine/	Finished ro	Rambo/wo	0	No
	Piped into (	No		Pit Latrine/	Finished ro	Rambo/wo	1	Yes
	Open Sprin	No		Pit Latrine/	Finished ro	Rambo/wo	1	Yes
	Piped outsi	Yes	Boil	No facility/	Finished ro	Cement	2	No
	River	No		No facility/	Finished ro	Other	1	No
	River	No		Pit Latrine/	Finished ro	Rambo/wo	1	Yes
2	Piped into (	No		Pit Latrine/	Finished ro	Stone with	1	Yes
	Open Well	No		Pit Latrine/	Finished ro	Rambo/wo	1	No
	Piped outsi	No		Pit Latrine/	Finished ro	Stone with	1	Yes
	Piped outsi	No		Pit Latrine/	Finished ro	Rambo/wo	1	No
	Piped into (	No		Pit Latrine/	Finished ro	Rambo/wo	2	Yes
	Piped outsi	No		Pit Latrine/	Finished ro	Rambo/wo	1	No
	Piped into (	No		No facility/	Finished ro	Rambo/wo	1	No
	Open Sprin	No		Pit Latrine/	Finished ro	Rambo/wo	1	Yes
	Piped outsi	No		No facility/	Finished ro	Rambo/wo	1	Yes
	Piped outsi	No		Pit Latrine/	Thatch/lea	Rambo/wo	0	Yes
	Piped outsi	No		Pit Latrine/	Thatch/lea	Rambo/wo	1	Yes
	Piped outsi	No		Pit Latrine/	Thatch/lea	Rambo/wo	0	Yes
	Piped outsi	No		No facility/	Finished ro	Rambo/wo	1	Yes

For peer review only

	Piped into (	No		Pit Latrine/	Finished ro	Rambo/wo	1	Yes
	Piped into (	No		Ventilated	Finished ro	Rambo/wo	1	No
	Protected S	No		Pit Latrine/	Finished ro	Rambo/wo	1	Yes
1	Open Sprin	No		Pit Latrine/	Finished ro	Rambo/wo	1	No
	Piped outsi	No		Pit Latrine/	Finished ro	Rambo/wo	2	No
	Piped into (	No		Pit Latrine/	Finished ro	Rambo/wo	5	Yes
	Piped outsi	No		Pit Latrine/	Thatch/lea	Rambo/wo	0	Yes
	Covered W	No		Pit Latrine/	Finished ro	Rambo/wo	1	Yes
	Protected S	No		Pit Latrine/	Finished ro	Rambo/wo	1	No
	Piped into (	No		Ventilated	Finished ro	Stone with	1	Yes
	River	No		Pit Latrine/	Finished ro	Rambo/wo	1	Yes
	Piped into (	Yes	Boil	Pit Latrine/	Finished ro	Rambo/wo	2	No
	Open Well	No		No facility/	Finished ro	Rambo/wo	1	No
	Piped outsi	No		Pit Latrine/	Thatch/lea	Rambo/wo	0	Yes
	Piped outsi	No		No facility/	Wood plan	Rambo/wo	2	No
	Open Well	No		Pit Latrine/	Finished ro	Rambo/wo	0	No
	Piped outsi	No		No facility/	Finished ro	Cement	1	Yes
1	Open Sprin	No		Pit Latrine/	Finished ro	Rambo/wo	2	Yes
	River	No		No facility/	Finished ro	Rambo/wo	0	No
1	Piped outsi	No		Pit Latrine/	Finished ro	Rambo/wo	1	No
	Piped outsi	No		Pit Latrine/	Finished ro	Other	1	No
	Piped outsi	No		Pit Latrine/	Thatch/lea	Rambo/wo	0	Yes
	Piped into (	No		Pit Latrine/	Finished ro	Rambo/wo	1	No
	Piped into (	No		Pit Latrine/	Finished ro	Rambo/wo	0	No
	Piped outsi	No		No facility/	Finished ro	Other	1	No
	Piped outsi	No		Pit Latrine/	Finished ro	Rambo/wo	1	Yes
	Piped outsi	No		No facility/	Finished ro	Other	1	Yes
	Piped outsi	No		Pit Latrine/	Finished ro	Rambo/wo	1	No
	Piped into (	No		Pit Latrine/	Finished ro	Stone with	1	No
	Piped into (	No		Ventilated	Finished ro	Rambo/wo	1	Yes
	Open Sprin	No		No facility/	Finished ro	Rambo/wo	1	Yes
	Covered W	No		Pit Latrine/	Finished ro	Stone with	1	Yes
	Piped into (	No		Pit Latrine/	Finished ro	Other	1	Yes
	Piped outsi	No		Pit Latrine/	Finished ro	Rambo/wo	1	Yes
	Piped outsi	No		Pit Latrine/	Finished ro	Rambo/wo	0	No
	Piped outsi	No		Pit Latrine/	Finished ro	Rambo/wo	1	No
	Piped into (	No		Pit Latrine/	Finished ro	Rambo/wo	1	Yes
	Piped into (	No		Pit Latrine/	Finished ro	Rambo/wo	1	No
	Piped outsi	No		Pit Latrine/	Finished ro	Other	1	Yes
	River	Yes	Other	Pit Latrine/	Finished ro	Rambo/wo	1	Yes
	Piped outsi	No		Pit Latrine/	Finished ro	Cement	1	No
	River	No		Pit Latrine/	Finished ro	Rambo/wo	1	Yes
	Piped into (	No		Pit Latrine/	Finished ro	Cement	1	No
	River	No		Pit Latrine/	Finished ro	Rambo/wo	1	Yes
	Piped into (	No		Ventilated	Finished ro	Rambo/wo	1	Yes
	River	No		Other	Finished ro	Stone with	1	Yes
	Open Sprin	No		Pit Latrine/	Finished ro	Rambo/wo	1	No
	River	No		No facility/	Finished ro	Rambo/wo	1	Yes
	Piped into (	No		Ventilated	Finished ro	Rambo/wo	0	No
	Piped into (	No		Pit Latrine/	Finished ro	Cement	3	Yes
	Piped outsi	No		Pit Latrine/	Finished ro	Rambo/wo	1	Yes
1	Protected S	No		Pit Latrine/	Finished ro	Rambo/wo	1	Yes

For peer review only

	Open Sprin	No		Pit Latrine/	Finished ro	Rambo/wo	1	Yes
	River	No		Pit Latrine/	Finished ro	Rambo/wo	1	Yes
	Piped into (	No		Pit Latrine/	Finished ro	Rambo/wo	1	Yes
1	Piped into (	No		Ventilated	Finished ro	Rambo/wo	1	No
	Piped outsi	No		Pit Latrine/	Finished ro	Rambo/wo	1	No
	Piped outsi	No		Pit Latrine/	Thatch/lea	Rambo/wo	0	Yes
	Piped into (	No		Pit Latrine/	Finished ro	Rambo/wo	2	No
	Piped outsi	No		Pit Latrine/	Thatch/lea	Rambo/wo	0	Yes
	Piped outsi	No		Pit Latrine/	Finished ro	Rambo/wo	2	Yes
	Piped outsi	Yes	Boil	No facility/	Finished ro	Cement	2	Yes
	Piped into (	No		Pit Latrine/	Finished ro	Rambo/wo	0	No
	Piped into (	No		Pit Latrine/	Finished ro	Stone with	1	No
	Piped outsi	No		Pit Latrine/	Finished ro	Rambo/wo	2	Yes
	Piped outsi	No		Pit Latrine/	Finished ro	Rambo/wo	0	No
1	Protected S	No		Pit Latrine/	Finished ro	Rambo/wo	1	Yes
	Piped into (	No		Pit Latrine/	Finished ro	Rambo/wo	1	Yes
	Piped outsi	No		Pit Latrine/	Finished ro	Rambo/wo	1	No
	Piped into (	No		Ventilated	Finished ro	Cement	1	No
	Piped into (	No		No facility/	Thatch/lea	Stone with	0	No
	Piped into (	No		Pit Latrine/	Finished ro	Rambo/wo	1	No
	Piped outsi	No		Pit Latrine/	Finished ro	Rambo/wo	0	No
	Protected S	No		Pit Latrine/	Finished ro	Rambo/wo	1	Yes
	Protected S	No		No facility/	Thatch/lea	Rambo/wo	0	No
	Piped outsi	No		Ventilated	Finished ro	Rambo/wo	1	No
	Piped into (	No		Ventilated	Finished ro	Stone with	1	Yes
	Piped outsi	No		Pit Latrine/	Finished ro	Cement	1	Yes
	Piped outsi	No		No facility/	Finished ro	Cement	1	Yes
	Piped outsi	No		Pit Latrine/	Thatch/lea	Rambo/wo	0	Yes
	Open Sprin	No		No facility/	Finished ro	Rambo/wo	0	No
	Piped outsi	No		No facility/	Finished ro	Cement	1	No
	Piped outsi	No		Ventilated	Finished ro	Rambo/wo	0	Yes
	Piped into (	No		Flush toilet	Finished ro	Cement	1	No
	Piped outsi	No		Pit Latrine/	Finished ro	Rambo/wo	0	No
	Piped into (	No		Pit Latrine/	Finished ro	Cement	1	No
1	Piped into (	No		Pit Latrine/	Finished ro	Rambo/wo	1	No
	Piped into (	No		Pit Latrine/	Finished ro	Rambo/wo	1	Yes
	Piped into (	No		Pit Latrine/	Finished ro	Rambo/wo	1	No
	Piped outsi	No		Pit Latrine/	Finished ro	Cement	1	Yes
	Protected S	No		Pit Latrine/	Finished ro	Rambo/wo	1	Yes
	Piped outsi	No		Pit Latrine/	Finished ro	Rambo/wo	1	Yes
	Piped into (	Yes	Boil	Pit Latrine/	Finished ro	Rambo/wo	0	No
	Piped outsi	No		Pit Latrine/	Thatch/lea	Rambo/wo	1	Yes
	Rainwater	No		No facility/	Finished ro	Rambo/wo	0	Yes
	Piped outsi	No		Pit Latrine/	Finished ro	Rambo/wo	2	No
	Piped into (	No		Pit Latrine/	Thatch/lea	Rambo/wo	1	Yes
	River	No		Pit Latrine/	Finished ro	Rambo/wo	0	Yes
	Protected S	No		Pit Latrine/	Finished ro	Rambo/wo	1	Yes
1	Piped outsi	No		Pit Latrine/	Finished ro	Rambo/wo	1	Yes
	Piped into (	Yes	Boil	No facility/	Finished ro	Cement	2	Yes
	Piped outsi	No		Pit Latrine/	Thatch/lea	Rambo/wo	0	Yes
	Piped outsi	No		Pit Latrine/	Thatch/lea	Rambo/wo	0	Yes
	Piped outsi	No		No facility/	Finished ro	Cement	1	No

	Piped into (	No		No facility/	Finished ro	Rambo/wo	2	No
	Piped outsi	No		No facility/	Finished ro	Rambo/wo	1	Yes
	Piped into (	No		Ventilated	Finished ro	Cement	1	No
	Piped into (	No		Ventilated	Finished ro	Stone with	1	No
	Piped outsi	No		Pit Latrine/	Finished ro	Rambo/wo	0	No
	Piped outsi	No		Pit Latrine/	Finished ro	Rambo/wo	1	Yes
	Piped outsi	No		Pit Latrine/	Finished ro	Rambo/wo	1	Yes
	Piped outsi	No		Pit Latrine/	Finished ro	Stone with	1	No
	Piped into (	No		Pit Latrine/	Finished ro	Rambo/wo	1	No
	Open Well	No		Pit Latrine/	Finished ro	Rambo/wo	1	Yes
	Piped outsi	No		Pit Latrine/	Finished ro	Rambo/wo	1	No
	River	No		No facility/	Finished ro	Cement	1	Yes
	Piped outsi	No		Pit Latrine/	Finished ro	Rambo/wo	3	Yes
	Piped outsi	No		Pit Latrine/	Finished ro	Cement	1	No
	Piped into (	No		Pit Latrine/	Finished ro	Stone with	2	No
	Piped outsi	No		Pit Latrine/	Finished ro	Rambo/wo	0	No
	River	No		No facility/	Finished ro	Rambo/wo	0	Yes
	Piped outsi	Yes	Boil	No facility/	Finished ro	Rambo/wo	2	No
	Piped into (	No		Ventilated	Finished ro	Rambo/wo	1	Yes
	Open Sprin	No		No facility/	Finished ro	Rambo/wo	1	Yes
	Piped into (	No		Pit Latrine/	Finished ro	Cement	1	Yes
	Piped into (	No		Pit Latrine/	Finished ro	Rambo/wo	0	No
	River	No		No facility/	Finished ro	Rambo/wo	1	Yes
	Open Sprin	No		No facility/	Finished ro	Rambo/wo	1	Yes
	Piped outsi	No		Ventilated	Finished ro	Rambo/wo	1	No
	Protected I	No		Pit Latrine/	Finished ro	Rambo/wo	1	Yes
1	River	No		Pit Latrine/	Finished ro	Rambo/wo	2	Yes
	Open Well	No		Pit Latrine/	Finished ro	Stone with	1	No
	Piped into (	No		Pit Latrine/	Finished ro	Cement	2	No
	Piped into (	No		Pit Latrine/	Finished ro	Rambo/wo	1	No
	Open Well	No		Pit Latrine/	Finished ro	Rambo/wo	1	Yes
	River	No		No facility/	Finished ro	Other	1	Yes
	River	No		No facility/	Thatch/lea	Rambo/wo	0	Yes
	Piped into (	Yes	Boil	Pit Latrine/	Finished ro	Rambo/wo	2	No
	Piped into (	No		Pit Latrine/	Finished ro	Rambo/wo	0	No
	Piped outsi	No		Pit Latrine/	Finished ro	Rambo/wo	0	Yes
	Open Well	No		Pit Latrine/	Finished ro	Rambo/wo	0	No
	Piped outsi	Yes	Boil	Pit Latrine/	Finished ro	Cement	2	No
	River	No		No facility/	Finished ro	Rambo/wo	0	Yes
	Piped outsi	No		Pit Latrine/	Finished ro	Rambo/wo	1	No
	Piped outsi	No		Pit Latrine/	Finished ro	Rambo/wo	0	Yes
	Protected S	No		Pit Latrine/	Finished ro	Rambo/wo	0	No
	Piped outsi	No		No facility/	Finished ro	Cement	2	Yes
	Open Sprin	No		Pit Latrine/	Finished ro	Rambo/wo	1	Yes
	Piped into (	No		Pit Latrine/	Finished ro	Cement	1	No
	Piped into (	No		Pit Latrine/	Finished ro	Stone with	1	No
	Piped outsi	Yes	Boil	No facility/	Finished ro	Stone with	2	Yes
	Piped outsi	No		Pit Latrine/	Thatch/lea	Rambo/wo	1	Yes
	River	No		Pit Latrine/	Finished ro	Rambo/wo	2	Yes
	Piped outsi	No		Pit Latrine/	Finished ro	Other	1	Yes
	Protected S	No		Pit Latrine/	Finished ro	Rambo/wo	1	Yes
	Piped outsi	No		No facility/	Finished ro	Other	1	Yes

For peer review only

1	Piped into (	No		Ventilated	Finished ro	Cement	1	Yes
	Piped into (	No		Pit Latrine/	Finished ro	Rambo/wo	2	No
1	Piped outsi	Yes	Strain it thr	Pit Latrine/	Finished ro	Other	1	No
	River	No		No facility/	Thatch/lea	Rambo/wo	0	Yes
	Piped into (	No		Pit Latrine/	Finished ro	Other	1	Yes
	Piped into (	No		Pit Latrine/	Finished ro	Rambo/wo	0	No
	Piped outsi	No		Pit Latrine/	Finished ro	Stone with	1	No
	Piped outsi	No		No facility/	Finished ro	Other	1	No
	River	No		Pit Latrine/	Finished ro	Rambo/wo	0	Yes
	Piped into (	No		Pit Latrine/	Finished ro	Stone with	1	No
	Piped into (	Yes	Boil	Pit Latrine/	Finished ro	Rambo/wo	1	No
	Piped into (	No		Pit Latrine/	Finished ro	Cement	1	No
	Piped outsi	No		No facility/	Finished ro	Cement	1	Yes
	River	No		Pit Latrine/	Finished ro	Rambo/wo	1	Yes
	Piped outsi	No		Pit Latrine/	Finished ro	Rambo/wo	1	No
	Piped outsi	No		Pit Latrine/	Finished ro	Cement	1	No
3	Piped into (	No		Pit Latrine/	Finished ro	Cement	1	Yes
	River	No		Pit Latrine/	Finished ro	Rambo/wo	1	Yes
	Piped outsi	No		Pit Latrine/	Finished ro	Cement	1	Yes
	Piped into (	No		Pit Latrine/	Finished ro	Rambo/wo	0	No
	Protected S	No		Pit Latrine/	Finished ro	Rambo/wo	1	Yes
	Piped into (	Yes	Boil	Pit Latrine/	Finished ro	Rambo/wo	2	No
	River	No		Pit Latrine/	Finished ro	Other	1	Yes
	Piped outsi	No		Pit Latrine/	Finished ro	Rambo/wo	1	No
	Piped outsi	No		Pit Latrine/	Finished ro	Rambo/wo	0	No
	River	No		Pit Latrine/	Finished ro	Rambo/wo	1	Yes
	Open Sprin	No		Pit Latrine/	Thatch/lea	Rambo/wo	0	No
	Piped outsi	No		Pit Latrine/	Finished ro	Rambo/wo	1	No
	River	No		Pit Latrine/	Finished ro	Rambo/wo	1	Yes
1	River	No		Pit Latrine/	Finished ro	Rambo/wo	1	Yes
	Open Sprin	No		No facility/	Finished ro	Rambo/wo	1	Yes
	Piped outsi	Yes	Boil	Pit Latrine/	Finished ro	Cement	2	Yes
	Piped into (	No		Pit Latrine/	Finished ro	Stone with	1	No
	Piped into (	No		Pit Latrine/	Finished ro	Cement	1	No
	Piped outsi	No		Pit Latrine/	Finished ro	Rambo/wo	1	Yes
	Open Sprin	No		No facility/	Finished ro	Rambo/wo	2	Yes
	Piped into (	No		Pit Latrine/	Finished ro	Cement	2	No
	Piped outsi	No		Pit Latrine/	Thatch/lea	Rambo/wo	0	Yes
	Protected S	No		No facility/	Finished ro	Rambo/wo	1	Yes
	Piped outsi	No		Pit Latrine/	Finished ro	Rambo/wo	1	Yes
	Piped outsi	No		Pit Latrine/	Finished ro	Rambo/wo	1	Yes
	Protected S	Yes	Boil	Pit Latrine/	Finished ro	Rambo/wo	2	Yes
	Piped outsi	No		Pit Latrine/	Finished ro	Cement	1	Yes
	Piped into (	No		Pit Latrine/	Finished ro	Stone with	1	No
	River	No		No facility/	Finished ro	Rambo/wo	0	Yes
	Protected S	No		Pit Latrine/	Finished ro	Rambo/wo	1	Yes
	Piped into (	No		Pit Latrine/	Finished ro	Rambo/wo	1	Yes
	River	No		Pit Latrine/	Finished ro	Rambo/wo	1	Yes
	Protected I	Yes	Add bleach	No facility/	Finished ro	Rambo/wo	0	Yes
	Piped outsi	No		Ventilated	Finished ro	Rambo/wo	1	Yes
	Open Sprin	No		Pit Latrine/	Finished ro	Rambo/wo	1	Yes
	Piped outsi	No		Pit Latrine/	Thatch/lea	Rambo/wo	0	Yes

	Piped outsi	No		No facility/	Finished ro	Rambo/wo	1	No
	Open Sprin	No		Pit Latrine/	Finished ro	Rambo/wo	1	Yes
	Protected S	No		No facility/	Finished ro	Rambo/wo	2	Yes
1	Piped into (	No		Pit Latrine/	Finished ro	Rambo/wo	1	No
	Protected S	No		No facility/	Finished ro	Rambo/wo	2	No
	Pond/Lake,	No		No facility/	Finished ro	Rambo/wo	1	Yes
	Piped outsi	No		Pit Latrine/	Finished ro	Rambo/wo	1	No
	Piped into (	No		Pit Latrine/	Finished ro	Rambo/wo	1	Yes
	Pond/Lake,	No		Pit Latrine/	Finished ro	Rambo/wo	1	Yes
	Piped outsi	No		No facility/	Finished ro	Rambo/wo	1	No
	Piped outsi	No		No facility/	Finished ro	Rambo/wo	1	Yes
	Piped outsi	No		Pit Latrine/	Finished ro	Rambo/wo	1	Yes
	Piped outsi	No		No facility/	Finished ro	Rambo/wo	1	No
	Piped outsi	No		Pit Latrine/	Finished ro	Rambo/wo	1	Yes
	Piped outsi	No		Pit Latrine/	Finished ro	Rambo/wo	1	No
	Piped outsi	No		No facility/	Finished ro	Rambo/wo	1	Yes
	Piped outsi	No		Pit Latrine/	Finished ro	Rambo/wo	1	Yes
	Piped outsi	No		Pit Latrine/	Finished ro	Rambo/wo	1	No
	Piped outsi	No		Pit Latrine/	Finished ro	Rambo/wo	1	Yes
	Piped outsi	No		Ventilated	Finished ro	Rambo/wo	1	No
	Protected I	Yes	Add bleach	No facility/	Finished ro	Rambo/wo	0	Yes
	Piped outsi	No		Pit Latrine/	Finished ro	Rambo/wo	1	No
	Piped outsi	No		No facility/	Finished ro	Rambo/wo	0	Yes
	Pond/Lake,	No		No facility/	Finished ro	Rambo/wo	1	Yes
	Piped outsi	No		Pit Latrine/	Finished ro	Rambo/wo	1	Yes
	Protected S	Yes	Add bleach	Pit Latrine/	Finished ro	Rambo/wo	2	Yes
	Piped outsi	No		Pit Latrine/	Finished ro	Rambo/wo	1	Yes
	Piped outsi	No		Pit Latrine/	Finished ro	Rambo/wo	1	Yes
1	Pond/Lake,	No		No facility/	Finished ro	Rambo/wo	1	Yes
	Open Sprin	No		Pit Latrine/	Finished ro	Rambo/wo	1	Yes
	Piped outsi	No		No facility/	Finished ro	Cement	1	Yes
	Piped outsi	No		No facility/	Finished ro	Rambo/wo	1	Yes
	Piped outsi	No		No facility/	Finished ro	Rambo/wo	1	Yes
	Piped outsi	No		Ventilated	Finished ro	Stone with	0	Yes
	Piped outsi	No		No facility/	Finished ro	Rambo/wo	1	Yes
	Pond/Lake,	Yes	Strain it thr	No facility/	Finished ro	Rambo/wo	1	Yes
	Piped outsi	No		No facility/	Finished ro	Stone with	2	Yes
	Piped outsi	No		Pit Latrine/	Finished ro	Rambo/wo	1	Yes
	Open Sprin	No		No facility/	Finished ro	Rambo/wo	1	Yes
	Pond/Lake,	No		Pit Latrine/	Finished ro	Rambo/wo	1	Yes
	Piped outsi	No		Pit Latrine/	Finished ro	Rambo/wo	1	Yes
	Piped outsi	No		Pit Latrine/	Finished ro	Rambo/wo	0	Yes
	Pond/Lake,	Yes	Strain it thr	No facility/	Finished ro	Rambo/wo	1	Yes
1	Piped outsi	No		Pit Latrine/	Finished ro	Rambo/wo	1	No
	Piped outsi	No		Pit Latrine/	Finished ro	Rambo/wo	1	Yes
	Pond/Lake,	Yes	Strain it thr	No facility/	Finished ro	Rambo/wo	1	Yes
	Covered W	No		Pit Latrine/	Finished ro	Rambo/wo	0	Yes
	Piped outsi	No		Pit Latrine/	Finished ro	Rambo/wo	1	Yes
	Piped outsi	No		No facility/	Finished ro	Rambo/wo	2	Yes
	Pond/Lake,	No		No facility/	Finished ro	Rambo/wo	1	Yes
	River	No		Pit Latrine/	Finished ro	Rambo/wo	1	Yes
	Open Well	No		No facility/	Finished ro	Rambo/wo	1	Yes

For peer review only

	Open Sprin	No		No facility/	Finished ro	Rambo/wo	1	Yes
	Piped outsi	No		No facility/	Finished ro	Rambo/wo	1	Yes
	Piped outsi	No		No facility/	Finished ro	Rambo/wo	1	Yes
	Piped outsi	No		No facility/	Finished ro	Rambo/wo	1	Yes
	Pond/Lake,	No		No facility/	Finished ro	Rambo/wo	1	Yes
	Piped outsi	No		Ventilated	Finished ro	Rambo/wo	1	No
	Piped outsi	No		Pit Latrine/	Thatch/lea	Rambo/wo	0	No
	Open Well	No		No facility/	Finished ro	Rambo/wo	1	Yes
	Piped outsi	No		No facility/	Finished ro	Rambo/wo	1	Yes
	Piped outsi	No		No facility/	Finished ro	Rambo/wo	1	Yes
	Piped outsi	No		Pit Latrine/	Finished ro	Rambo/wo	1	Yes
	Open Sprin	No		Pit Latrine/	Finished ro	Stone with	2	Yes
	Piped outsi	No		Pit Latrine/	Finished ro	Rambo/wo	1	Yes
	Piped outsi	No		Pit Latrine/	Finished ro	Rambo/wo	1	No
	Piped outsi	No		Pit Latrine/	Finished ro	Rambo/wo	1	Yes
	Piped outsi	No		Pit Latrine/	Finished ro	Rambo/wo	1	No
	Piped outsi	No		Pit Latrine/	Finished ro	Rambo/wo	2	Yes
	Piped outsi	No		Pit Latrine/	Thatch/lea	Rambo/wo	0	No
	Open Well	No		Pit Latrine/	Finished ro	Rambo/wo	1	Yes
	Piped outsi	No		Pit Latrine/	Finished ro	Rambo/wo	1	No
	Piped outsi	No		No facility/	Finished ro	Rambo/wo	1	Yes
	Piped outsi	No		Ventilated	Finished ro	Rambo/wo	0	No
	Open Well	No		No facility/	Finished ro	Rambo/wo	1	Yes
1	Open Sprin	No		No facility/	Finished ro	Rambo/wo	1	Yes
	Open Sprin	No		Pit Latrine/	Finished ro	Rambo/wo	0	Yes
	Piped outsi	No		Pit Latrine/	Finished ro	Rambo/wo	0	Yes
	Piped outsi	No		Pit Latrine/	Finished ro	Rambo/wo	1	No
	Piped into	No		Pit Latrine/	Finished ro	Rambo/wo	0	No
	Piped outsi	No		No facility/	Finished ro	Rambo/wo	1	Yes
	Open Sprin	No		No facility/	Finished ro	Rambo/wo	0	Yes
	Pond/Lake,	No		Pit Latrine/	Finished ro	Rambo/wo	1	Yes
	Open Well	No		No facility/	Finished ro	Rambo/wo	1	Yes
	Protected I	No		No facility/	Finished ro	Rambo/wo	0	No
	Piped outsi	No		Pit Latrine/	Finished ro	Rambo/wo	1	Yes
	Pond/Lake,	No		Pit Latrine/	Finished ro	Rambo/wo	0	Yes
	Piped outsi	No		No facility/	Finished ro	Rambo/wo	1	Yes
	Piped outsi	No		No facility/	Finished ro	Rambo/wo	1	Yes
	Piped outsi	No		Pit Latrine/	Finished ro	Rambo/wo	1	Yes
	Piped outsi	No		No facility/	Finished ro	Rambo/wo	1	Yes
	Pond/Lake,	No		Pit Latrine/	Finished ro	Rambo/wo	1	Yes
	Pond/Lake,	Yes	Strain it thr	No facility/	Thatch/lea	Rambo/wo	1	Yes
2	Pond/Lake,	Yes	Boil	Pit Latrine/	Finished ro	Rambo/wo	0	Yes
	Piped outsi	No		No facility/	Finished ro	Rambo/wo	1	Yes
	Protected I	No		No facility/	Finished ro	Rambo/wo	0	Yes
	Pond/Lake,	Yes	Strain it thr	No facility/	Finished ro	Rambo/wo	1	Yes
	Piped outsi	No		No facility/	Finished ro	Cement	1	Yes
	Piped outsi	No		No facility/	Finished ro	Rambo/wo	1	Yes
	Piped into	No		Pit Latrine/	Finished ro	Rambo/wo	1	Yes
	Pond/Lake,	No		Pit Latrine/	Finished ro	Stone with	1	No
	Piped outsi	No		No facility/	Finished ro	Rambo/wo	0	No
	Piped outsi	No		Pit Latrine/	Finished ro	Rambo/wo	1	Yes
	Piped into	No		No facility/	Finished ro	Rambo/wo	1	No

	Piped outsi	No		Pit Latrine/	Finished ro	Rambo/wo	0	Yes
	Piped outsi	No		Pit Latrine/	Finished ro	Rambo/wo	1	Yes
	Pond/Lake,	Yes	Strain it thr	No facility/	Finished ro	Rambo/wo	1	Yes
	Open Well	No		No facility/	Finished ro	Rambo/wo	1	Yes
	Piped outsi	No		Pit Latrine/	Finished ro	Rambo/wo	1	Yes
	Open Sprin	No		Pit Latrine/	Finished ro	Rambo/wo	1	Yes
	Piped outsi	No		Pit Latrine/	Finished ro	Rambo/wo	2	Yes
	Piped outsi	No		Pit Latrine/	Finished ro	Cement	0	Yes
	Piped outsi	No		Pit Latrine/	Finished ro	Rambo/wo	1	No
	Piped outsi	No		No facility/	Finished ro	Rambo/wo	1	Yes
	Open Well	No		No facility/	Finished ro	Rambo/wo	1	Yes
	Piped outsi	No		Pit Latrine/	Finished ro	Rambo/wo	1	No
	Piped outsi	No		Pit Latrine/	Thatch/lea	Rambo/wo	0	Yes
	Piped into	No		Pit Latrine/	Finished ro	Rambo/wo	1	Yes
1	Piped outsi	No		Pit Latrine/	Finished ro	Rambo/wo	1	Yes
	Piped outsi	No		Pit Latrine/	Finished ro	Rambo/wo	1	Yes
	Piped outsi	No		Pit Latrine/	Finished ro	Rambo/wo	1	Yes
	Piped outsi	No		Pit Latrine/	Finished ro	Rambo/wo	1	Yes
	Piped outsi	No		Pit Latrine/	Finished ro	Rambo/wo	1	Yes
	Piped outsi	No		Pit Latrine/	Finished ro	Rambo/wo	1	Yes
	Covered W	No		Pit Latrine/	Finished ro	Rambo/wo	0	Yes
	River	No		Pit Latrine/	Finished ro	Rambo/wo	1	No
	Piped outsi	Yes	Boil	No facility/	Finished ro	Cement	2	Yes
	Piped outsi	Yes	Boil	Pit Latrine/	Finished ro	Rambo/wo	1	Yes
	Piped outsi	No		Pit Latrine/	Finished ro	Rambo/wo	1	No
	Piped outsi	No		Pit Latrine/	Finished ro	Rambo/wo	1	No
	Covered W	No		No facility/	Finished ro	Rambo/wo	0	Yes
	Open Sprin	No		No facility/	Finished ro	Rambo/wo	1	No
	Piped outsi	No		Pit Latrine/	Finished ro	Rambo/wo	2	Yes
	Protected I	No		Pit Latrine/	Finished ro	Rambo/wo	2	Yes
	Piped outsi	No		Pit Latrine/	Wood plan	Rambo/wo	0	Yes
	Piped outsi	No		Pit Latrine/	Finished ro	Rambo/wo	1	Yes
	Pond/Lake,	Yes	Strain it thr	No facility/	Finished ro	Rambo/wo	1	Yes
	Protected S	No		Pit Latrine/	Finished ro	Rambo/wo	2	No
	Pond/Lake,	Yes	Strain it thr	No facility/	Finished ro	Rambo/wo	1	Yes
	Open Sprin	No		Pit Latrine/	Finished ro	Rambo/wo	1	No
	Piped outsi	No		Pit Latrine/	Thatch/lea	Rambo/wo	0	Yes
	Pond/Lake,	No		No facility/	Finished ro	Rambo/wo	1	Yes
	Piped outsi	No		No facility/	Finished ro	Rambo/wo	1	No
	Pond/Lake,	No		Pit Latrine/	Finished ro	Rambo/wo	2	Yes
	Pond/Lake,	Yes	Strain it thr	No facility/	Finished ro	Rambo/wo	1	Yes
	Covered W	No		No facility/	Finished ro	Rambo/wo	1	Yes
	Piped outsi	No		Pit Latrine/	Finished ro	Rambo/wo	1	No
1	Piped into	No		Ventilated	Finished ro	Rambo/wo	1	Yes
	Protected S	No		No facility/	Finished ro	Rambo/wo	1	Yes
	Pond/Lake,	No		Pit Latrine/	Finished ro	Rambo/wo	2	Yes
	Open Sprin	No		No facility/	Finished ro	Rambo/wo	0	Yes
	Open Well	No		Pit Latrine/	Finished ro	Rambo/wo	1	Yes
	Piped outsi	No		Pit Latrine/	Finished ro	Rambo/wo	1	No
	Piped outsi	Yes	Add bleach	Pit Latrine/	Finished ro	Rambo/wo	0	No
	Piped outsi	No		Pit Latrine/	Finished ro	Rambo/wo	1	Yes
	Pond/Lake,	No		Pit Latrine/	Finished ro	Rambo/wo	1	Yes

For peer review only

	Pond/Lake,	Yes	Strain it thr	No facility/	Finished ro	Rambo/wo	1	Yes
	Covered W	No		No facility/	Finished ro	Rambo/wo	0	No
	Open Sprin	No		No facility/	Finished ro	Rambo/wo	1	Yes
	Pond/Lake,	No		No facility/	Finished ro	Rambo/wo	0	Yes
	Protected I	No		No facility/	Finished ro	Rambo/wo	0	Yes
	Pond/Lake,	No		No facility/	Finished ro	Rambo/wo	1	Yes
	Piped outsi	No		Ventilated	Finished ro	Rambo/wo	0	No
	Piped outsi	No		Pit Latrine/	Finished ro	Rambo/wo	1	Yes
	Protected S	No		Pit Latrine/	Finished ro	Rambo/wo	1	Yes
1	Pond/Lake,	Yes	Strain it thr	No facility/	Finished ro	Rambo/wo	1	Yes
	Piped outsi	No		Ventilated	Finished ro	Rambo/wo	1	Yes
2	Open Sprin	No		Pit Latrine/	Finished ro	Rambo/wo	1	Yes
	Piped outsi	No		Pit Latrine/	Finished ro	Rambo/wo	0	Yes
	Open Well	No		No facility/	Finished ro	Rambo/wo	1	Yes
	Piped outsi	No		No facility/	Finished ro	Rambo/wo	1	Yes
	Open Sprin	No		Pit Latrine/	Finished ro	Rambo/wo	1	Yes
1	Piped outsi	No		No facility/	Finished ro	Rambo/wo	0	Yes
	Open Sprin	No		Pit Latrine/	Finished ro	Rambo/wo	1	Yes
2	Open Sprin	No		Pit Latrine/	Finished ro	Rambo/wo	1	Yes
	Piped outsi	No		Pit Latrine/	Finished ro	Rambo/wo	1	No
	Piped into (	No		Pit Latrine/	Finished ro	Rambo/wo	1	No
	Open Well	No		No facility/	Finished ro	Rambo/wo	1	Yes
1	Pond/Lake,	No		No facility/	Finished ro	Rambo/wo	1	Yes
	Piped outsi	Yes	Boil	Pit Latrine/	Thatch/lea	Rambo/wo	2	No
	Open Sprin	No		Pit Latrine/	Finished ro	Rambo/wo	0	Yes
	Piped outsi	No		No facility/	Finished ro	Rambo/wo	1	Yes
	Piped outsi	No		Ventilated	Finished ro	Rambo/wo	1	Yes
	Pond/Lake,	Yes	Strain it thr	Pit Latrine/	Finished ro	Rambo/wo	1	Yes
	Piped outsi	No		Pit Latrine/	Finished ro	Rambo/wo	1	Yes
	Piped outsi	No		No facility/	Finished ro	Rambo/wo	1	Yes
	Open Well	No		Pit Latrine/	Finished ro	Rambo/wo	1	Yes
	Protected I	Yes	Add bleach	No facility/	Finished ro	Rambo/wo	0	Yes
	Piped outsi	No		Pit Latrine/	Finished ro	Rambo/wo	1	Yes
	River	No		Pit Latrine/	Finished ro	Rambo/wo	0	No
	Covered W	No		Pit Latrine/	Finished ro	Rambo/wo	0	Yes
	Open Well	No		Pit Latrine/	Finished ro	Rambo/wo	1	Yes
	Piped outsi	No		No facility/	Finished ro	Cement	2	No
	Pond/Lake,	No		No facility/	Finished ro	Rambo/wo	1	Yes
	Piped outsi	Yes	Boil	No facility/	Finished ro	Rambo/wo	2	Yes
	Piped outsi	No		Pit Latrine/	Finished ro	Rambo/wo	1	Yes
	Piped outsi	No		Ventilated	Finished ro	Rambo/wo	1	No
	Open Well	No		Pit Latrine/	Finished ro	Rambo/wo	1	Yes
	Piped into (	No		Ventilated	Finished ro	Rambo/wo	1	No
	Piped outsi	No		No facility/	Finished ro	Rambo/wo	1	Yes
	Open Well	No		No facility/	Finished ro	Rambo/wo	0	Yes
	Open Well	No		No facility/	Finished ro	Rambo/wo	0	Yes
	Pond/Lake,	Yes	Strain it thr	No facility/	Finished ro	Rambo/wo	0	Yes
	Piped outsi	No		Pit Latrine/	Finished ro	Rambo/wo	2	Yes
	Piped outsi	No		Pit Latrine/	Finished ro	Rambo/wo	1	Yes
	Covered W	No		No facility/	Finished ro	Rambo/wo	0	Yes
	Open Well	No		No facility/	Finished ro	Rambo/wo	1	Yes
	Open Well	No		Pit Latrine/	Finished ro	Rambo/wo	1	Yes

For peer review only

	Pond/Lake,	No		Pit Latrine/	Finished ro	Rambo/wo	1	No
	Piped outsi	No		Pit Latrine/	Finished ro	Rambo/wo	1	Yes
	Pond/Lake,	Yes	Add bleach	Pit Latrine/	Finished ro	Rambo/wo	1	Yes
	Open Well	No		No facility/	Finished ro	Rambo/wo	0	Yes
	Piped outsi	No		Pit Latrine/	Finished ro	Rambo/wo	2	Yes
	Open Sprin	No		No facility/	Finished ro	Rambo/wo	1	Yes
	Open Sprin	No		No facility/	Finished ro	Rambo/wo	0	Yes
	Covered W	No		Pit Latrine/	Finished ro	Rambo/wo	1	Yes
	Pond/Lake,	Yes	Strain it thr	No facility/	Finished ro	Rambo/wo	1	Yes
	Open Sprin	No		Pit Latrine/	Finished ro	Rambo/wo	1	Yes
	Pond/Lake,	No		Pit Latrine/	Finished ro	Rambo/wo	1	No
	Piped outsi	No		Pit Latrine/	Finished ro	Rambo/wo	1	Yes
	Open Sprin	No		Pit Latrine/	Finished ro	Rambo/wo	1	Yes
	Protected S	Yes	Add bleach	Pit Latrine/	Finished ro	Cement	4	Yes
	Piped outsi	No		Ventilated	Finished ro	Rambo/wo	1	Yes
	Piped outsi	No		Pit Latrine/	Finished ro	Rambo/wo	1	No
	Open Well	No		Pit Latrine/	Finished ro	Rambo/wo	1	Yes
	Piped outsi	No		No facility/	Finished ro	Rambo/wo	1	Yes
	Piped outsi	Yes	Boil	Pit Latrine/	Finished ro	Rambo/wo	1	Yes
	Piped outsi	No		No facility/	Thatch/lea	Rambo/wo	0	Yes
	Piped outsi	No		Ventilated	Finished ro	Rambo/wo	1	No
	Covered W	No		Pit Latrine/	Finished ro	Rambo/wo	1	Yes
	Piped outsi	No		No facility/	Finished ro	Rambo/wo	0	No
	Protected I	Yes	Add bleach	No facility/	Finished ro	Rambo/wo	0	Yes
	Protected I	No		No facility/	Finished ro	Rambo/wo	0	Yes
	Protected I	No		No facility/	Finished ro	Rambo/wo	0	Yes
	Open Sprin	No		Pit Latrine/	Thatch/lea	Rambo/wo	0	No
	Protected S	No		Pit Latrine/	Finished ro	Rambo/wo	2	Yes
	Open Sprin	No		No facility/	Finished ro	Rambo/wo	0	Yes
	River	No		Pit Latrine/	Finished ro	Rambo/wo	0	No
	Piped outsi	No		Pit Latrine/	Finished ro	Rambo/wo	1	Yes
	Piped outsi	No		Pit Latrine/	Finished ro	Rambo/wo	1	Yes
	Pond/Lake,	No		No facility/	Finished ro	Rambo/wo	1	Yes
	Piped outsi	No		Pit Latrine/	Finished ro	Rambo/wo	1	Yes
	Open Well	No		No facility/	Finished ro	Rambo/wo	1	Yes
	Piped outsi	No		Pit Latrine/	Thatch/lea	Rambo/wo	0	No
	Open Sprin	No		Pit Latrine/	Finished ro	Rambo/wo	1	Yes
	Piped outsi	No		No facility/	Finished ro	Rambo/wo	1	Yes
	Covered W	No		No facility/	Finished ro	Rambo/wo	0	Yes
	Covered W	No		Pit Latrine/	Finished ro	Rambo/wo	0	Yes
	Open Well	No		No facility/	Finished ro	Rambo/wo	1	Yes
	Open Well	No		No facility/	Finished ro	Rambo/wo	1	Yes
1	Piped outsi	No		Ventilated	Finished ro	Rambo/wo	0	No
	Piped into	No		Ventilated	Finished ro	Rambo/wo	0	No
1	Protected S	No		No facility/	Finished ro	Stone with	1	No
	Protected S	No		Pit Latrine/	Finished ro	Rambo/wo	1	No
	Piped outsi	No		No facility/	Finished ro	Rambo/wo	1	No
1	River	No		No facility/	Finished ro	Rambo/wo	1	Yes
	Pond/Lake,	No		No facility/	Finished ro	Rambo/wo	0	Yes
	Open Well	No		No facility/	Finished ro	Rambo/wo	0	Yes
	Open Sprin	No		Pit Latrine/	Finished ro	Rambo/wo	0	Yes
	Piped into	No		No facility/	Finished ro	Rambo/wo	1	No

For peer review only

	Piped outsi	No		Pit Latrine/	Thatch/lea	Rambo/wo	1	Yes
	Piped outsi	No		Pit Latrine/	Finished ro	Rambo/wo	1	Yes
	Open Well	No		No facility/	Finished ro	Rambo/wo	0	Yes
	Piped outsi	No		Pit Latrine/	Finished ro	Rambo/wo	1	Yes
	Piped outsi	No		Pit Latrine/	Finished ro	Rambo/wo	0	Yes
	Piped outsi	No		No facility/	Finished ro	Rambo/wo	1	Yes
	Open Well	No		No facility/	Finished ro	Rambo/wo	1	Yes
	Pond/Lake,	Yes	Boil	Pit Latrine/	Finished ro	Rambo/wo	1	Yes
1	Piped outsi	No		Pit Latrine/	Finished ro	Rambo/wo	1	Yes
1	Open Sprin	No		Pit Latrine/	Finished ro	Rambo/wo	1	Yes
1	Piped outsi	No		Pit Latrine/	Finished ro	Rambo/wo	1	Yes
	Pond/Lake,	No		No facility/	Finished ro	Rambo/wo	1	Yes
	Protected S	No		Pit Latrine/	Wood plan	Rambo/wo	1	Yes
	Open Sprin	No		No facility/	Finished ro	Rambo/wo	1	Yes
	Piped outsi	No		No facility/	Finished ro	Rambo/wo	1	Yes
	Open Sprin	No		No facility/	Finished ro	Rambo/wo	1	Yes
	Protected S	No		Pit Latrine/	Finished ro	Rambo/wo	1	No
	Piped outsi	No		Pit Latrine/	Finished ro	Rambo/wo	1	Yes
	Piped outsi	No		No facility/	Finished ro	Rambo/wo	2	Yes
	Piped outsi	No		Pit Latrine/	Finished ro	Rambo/wo	1	Yes
	Pond/Lake,	Yes	Strain it thr	Pit Latrine/	Finished ro	Rambo/wo	1	Yes
	Piped outsi	No		No facility/	Thatch/lea	Rambo/wo	0	No
	Piped outsi	No		Ventilated	Finished ro	Rambo/wo	1	No
	Piped outsi	No		Pit Latrine/	Finished ro	Rambo/wo	1	Yes
	Piped outsi	No		No facility/	Finished ro	Rambo/wo	1	Yes
1	Pond/Lake,	No		No facility/	Finished ro	Rambo/wo	1	Yes
	Protected I	No		Pit Latrine/	Finished ro	Rambo/wo	1	Yes
	Piped outsi	No		No facility/	Finished ro	Rambo/wo	1	Yes
	Piped outsi	No		No facility/	Finished ro	Rambo/wo	2	Yes
	Piped outsi	No		Pit Latrine/	Thatch/lea	Rambo/wo	0	No
	River	No		No facility/	Finished ro	Rambo/wo	1	Yes
	Piped outsi	No		Pit Latrine/	Finished ro	Rambo/wo	2	Yes
	Piped outsi	No		No facility/	Finished ro	Rambo/wo	0	Yes
	Piped into	No		Pit Latrine/	Finished ro	Rambo/wo	1	Yes
	Piped outsi	No		No facility/	Finished ro	Rambo/wo	1	No
	Open Well	No		No facility/	Finished ro	Rambo/wo	0	Yes
	Pond/Lake,	Yes	Boil	Pit Latrine/	Finished ro	Rambo/wo	1	Yes
	Pond/Lake,	Yes	Boil	Pit Latrine/	Finished ro	Rambo/wo	1	Yes
	Piped outsi	No		No facility/	Finished ro	Rambo/wo	1	Yes
	Piped outsi	No		No facility/	Finished ro	Rambo/wo	2	Yes
	Piped outsi	No		Pit Latrine/	Finished ro	Rambo/wo	1	No
	Open Well	No		No facility/	Finished ro	Rambo/wo	0	Yes
	Piped outsi	No		Pit Latrine/	Finished ro	Rambo/wo	1	Yes
	Piped outsi	No		No facility/	Finished ro	Rambo/wo	1	Yes
	Piped outsi	No		Pit Latrine/	Finished ro	Rambo/wo	1	Yes
	Piped outsi	No		Pit Latrine/	Finished ro	Rambo/wo	1	Yes
	Piped outsi	No		No facility/	Finished ro	Rambo/wo	1	Yes
	Pond/Lake,	No		Pit Latrine/	Finished ro	Rambo/wo	1	No
	Piped outsi	No		Pit Latrine/	Finished ro	Rambo/wo	1	No
	Open Well	No		No facility/	Finished ro	Rambo/wo	1	Yes
	Piped outsi	No		Pit Latrine/	Finished ro	Rambo/wo	0	Yes
	Open Well	No		No facility/	Finished ro	Rambo/wo	1	Yes

For peer review only

2	Pond/Lake,	No		Pit Latrine/	Finished ro	Rambo/wo	1	Yes
	Pond/Lake,	No		No facility/	Finished ro	Rambo/wo	1	Yes
	Pond/Lake,	No		No facility/	Finished ro	Rambo/wo	1	Yes
	Piped outsi	No		Pit Latrine/	Finished ro	Rambo/wo	1	Yes
	Covered W	No		No facility/	Finished ro	Rambo/wo	0	Yes
	Open Well	No		Pit Latrine/	Finished ro	Rambo/wo	1	Yes
	Protected S	No		Pit Latrine/	Finished ro	Rambo/wo	1	Yes
1	Open Sprin	No		Pit Latrine/	Finished ro	Rambo/wo	1	Yes
	Piped outsi	No		Pit Latrine/	Finished ro	Rambo/wo	1	Yes
	Piped outsi	No		No facility/	Finished ro	Cement	1	Yes
1	Piped outsi	No		Pit Latrine/	Finished ro	Rambo/wo	1	Yes
	Piped outsi	No		Pit Latrine/	Finished ro	Rambo/wo	0	Yes
	Piped outsi	No		Pit Latrine/	Finished ro	Rambo/wo	2	Yes
	Piped outsi	No		No facility/	Finished ro	Rambo/wo	1	Yes
	Piped outsi	No		No facility/	Finished ro	Rambo/wo	1	Yes
	Pond/Lake,	Yes	Strain it thr	Pit Latrine/	Finished ro	Rambo/wo	1	Yes
	Pond/Lake,	No		No facility/	Finished ro	Rambo/wo	1	No
	Piped into	No		Ventilated	Finished ro	Rambo/wo	0	No
	Piped outsi	No		Ventilated	Finished ro	Rambo/wo	1	No
1	Piped outsi	No		Pit Latrine/	Finished ro	Rambo/wo	1	Yes
	Pond/Lake,	Yes	Boil	Pit Latrine/	Finished ro	Rambo/wo	1	No
	Piped outsi	No		Pit Latrine/	Finished ro	Rambo/wo	1	No
	Open Sprin	No		Pit Latrine/	Finished ro	Rambo/wo	1	Yes
	Piped outsi	No		No facility/	Finished ro	Rambo/wo	1	No
	Pond/Lake,	Yes	Boil	Pit Latrine/	Finished ro	Rambo/wo	1	Yes
	Pond/Lake,	No		No facility/	Finished ro	Rambo/wo	1	Yes
	Piped outsi	No		Pit Latrine/	Finished ro	Rambo/wo	1	Yes
	Pond/Lake,	Yes	Strain it thr	No facility/	Finished ro	Rambo/wo	0	Yes
	Piped into	No		Pit Latrine/	Finished ro	Rambo/wo	1	No
	Piped outsi	No		Pit Latrine/	Finished ro	Rambo/wo	1	Yes
	Open Well	No		No facility/	Finished ro	Rambo/wo	0	Yes
	Piped outsi	No		Pit Latrine/	Finished ro	Rambo/wo	1	Yes
	Piped outsi	No		No facility/	Finished ro	Rambo/wo	1	Yes
	Piped outsi	No		Pit Latrine/	Finished ro	Rambo/wo	0	No
	Piped outsi	No		No facility/	Finished ro	Rambo/wo	2	Yes
	Piped outsi	No		No facility/	Finished ro	Rambo/wo	1	Yes
	Piped outsi	No		Ventilated	Finished ro	Rambo/wo	1	No
	Piped outsi	No		No facility/	Finished ro	Rambo/wo	1	No
	River	No		No facility/	Finished ro	Rambo/wo	0	Yes
	Piped outsi	No		Pit Latrine/	Finished ro	Rambo/wo	1	Yes
	Piped outsi	No		Pit Latrine/	Finished ro	Rambo/wo	1	Yes
	Piped outsi	No		No facility/	Finished ro	Rambo/wo	1	No
	Open Sprin	No		No facility/	Finished ro	Rambo/wo	0	Yes
	Piped outsi	No		Pit Latrine/	Finished ro	Rambo/wo	1	Yes
	Piped outsi	No		Pit Latrine/	Finished ro	Rambo/wo	2	Yes
	Piped outsi	No		Pit Latrine/	Finished ro	Rambo/wo	1	Yes
1	Piped outsi	No		Pit Latrine/	Finished ro	Cement	1	Yes
	Piped outsi	No		Pit Latrine/	Finished ro	Rambo/wo	1	Yes
	Covered W	No		Pit Latrine/	Finished ro	Rambo/wo	0	Yes
	Open Sprin	No		No facility/	Finished ro	Rambo/wo	0	Yes
	Pond/Lake,	No		No facility/	Finished ro	Rambo/wo	0	No
	Piped into	No		No facility/	Finished ro	Rambo/wo	1	Yes

For peer review only

	Covered W	No		Pit Latrine/Finished ro	Rambo/wo	0	No
	River	No		No facility/Finished ro	Stone with	1	Yes
	River	No		No facility/Finished ro	Rambo/wo	1	Yes
	Piped outsi	No		Pit Latrine/Finished ro	Rambo/wo	1	Yes
	Piped outsi	No		No facility/Finished ro	Rambo/wo	1	Yes
	Piped outsi	No		Pit Latrine/Finished ro	Rambo/wo	1	No
1	Protected S	No		Pit Latrine/Finished ro	Rambo/wo	1	No
	Pond/Lake,	Yes	Add bleach	No facility/Finished ro	Rambo/wo	1	Yes
	Covered W	No		Pit Latrine/Finished ro	Rambo/wo	1	Yes
	Piped outsi	No		No facility/Finished ro	Rambo/wo	2	Yes
	Open Sprin	No		No facility/Finished ro	Rambo/wo	0	Yes
	Pond/Lake,	No		No facility/Finished ro	Rambo/wo	1	No
	Pond/Lake,	Yes	Boil	No facility/Finished ro	Rambo/wo	1	Yes
	Pond/Lake,	No		Pit Latrine/Finished ro	Rambo/wo	1	Yes
	Covered W	Yes	Boil	No facility/Finished ro	Rambo/wo	2	Yes
1	River	No		Pit Latrine/Finished ro	Rambo/wo	1	Yes
	Piped outsi	No		No facility/Finished ro	Rambo/wo	1	No
	Protected I	Yes	Add bleach	No facility/Finished ro	Rambo/wo	0	Yes
	Piped outsi	No		Pit Latrine/Thatch/lea	Rambo/wo	0	Yes
	Piped outsi	Yes	Boil	Pit Latrine/Finished ro	Rambo/wo	1	No
	Piped outsi	No		Pit Latrine/Finished ro	Rambo/wo	1	No
1	Protected I	No		Pit Latrine/Finished ro	Rambo/wo	2	No
1	Protected I	No		Pit Latrine/Finished ro	Rambo/wo	1	Yes
	Protected I	No		Pit Latrine/Finished ro	Rambo/wo	0	Yes
	Open Sprin	No		Pit Latrine/Finished ro	Rambo/wo	0	Yes
	Open Sprin	No		No facility/Finished ro	Rambo/wo	2	No
1	Piped outsi	No		Pit Latrine/Finished ro	Rambo/wo	0	No
	Piped outsi	No		Pit Latrine/Finished ro	Rambo/wo	0	Yes
	Protected I	No		Pit Latrine/Finished ro	Rambo/wo	1	No
	Protected I	No		Pit Latrine/Finished ro	Rambo/wo	0	Yes
	River	No		No facility/Finished ro	Rambo/wo	1	Yes
	Piped outsi	No		Pit Latrine/Finished ro	Rambo/wo	0	Yes
1	River	No		Pit Latrine/Finished ro	Rambo/wo	1	Yes
	Open Sprin	No		Pit Latrine/Finished ro	Rambo/wo	2	Yes
	Protected I	No		Pit Latrine/Wood plan	Rambo/wo	1	Yes
	River	No		No facility/Finished ro	Rambo/wo	0	Yes
	Protected I	Yes	Add bleach	Pit Latrine/Finished ro	Rambo/wo	1	Yes
	Protected I	No		Pit Latrine/Finished ro	Rambo/wo	1	Yes
	Protected I	No		Pit Latrine/Finished ro	Rambo/wo	0	Yes
	Open Sprin	No		No facility/Finished ro	Rambo/wo	0	Yes
	Protected I	No		Pit Latrine/Finished ro	Rambo/wo	0	No
	Open Sprin	No		Pit Latrine/Finished ro	Rambo/wo	0	Yes
	Open Sprin	No		Pit Latrine/Finished ro	Rambo/wo	1	Yes
	Protected I	No		No facility/Finished ro	Rambo/wo	0	Yes
	River	No		No facility/Finished ro	Rambo/wo	0	Yes
	River	No		Pit Latrine/Finished ro	Rambo/wo	1	Yes
	Protected I	No		Pit Latrine/Finished ro	Rambo/wo	1	Yes
	River	No		Pit Latrine/Finished ro	Rambo/wo	0	Yes
	River	No		No facility/Finished ro	Rambo/wo	1	Yes
	Protected I	No		No facility/Finished ro	Rambo/wo	1	Yes
	Open Sprin	No		Pit Latrine/Finished ro	Rambo/wo	1	No
	River	No		No facility/Finished ro	Rambo/wo	2	Yes

For peer review only

	Open Sprin	No		No facility/Finished ro	Rambo/wo	1	Yes
	River	No		Pit Latrine/Finished ro	Rambo/wo	1	Yes
	Protected I	No		No facility/Finished ro	Rambo/wo	1	Yes
	River	No		No facility/Finished ro	Rambo/wo	0	Yes
	River	No		Pit Latrine/Finished ro	Rambo/wo	0	Yes
	River	No		No facility/Finished ro	Rambo/wo	2	Yes
	River	No		No facility/Finished ro	Rambo/wo	0	Yes
	Protected I	Yes	Add bleach	Pit Latrine/Finished ro	Rambo/wo	0	Yes
	Protected S	No		Pit Latrine/Finished ro	Rambo/wo	1	Yes
	Protected S	No		Pit Latrine/Finished ro	Rambo/wo	0	Yes
	Protected I	No		Pit Latrine/Finished ro	Rambo/wo	1	Yes
	Protected I	No		Pit Latrine/Wood plan	Rambo/wo	1	Yes
2	Protected I	No		Pit Latrine/Finished ro	Rambo/wo	1	Yes
	Piped outsi	No		Pit Latrine/Finished ro	Rambo/wo	0	Yes
	Open Sprin	No		Pit Latrine/Finished ro	Rambo/wo	1	Yes
3	River	No		Pit Latrine/Finished ro	Rambo/wo	1	Yes
	Piped outsi	No		No facility/Finished ro	Rambo/wo	1	No
	Open Sprin	No		No facility/Finished ro	Rambo/wo	0	Yes
	Covered W	Yes	Add bleach	Pit Latrine/Finished ro	Rambo/wo	1	Yes
	Protected I	No		No facility/Finished ro	Rambo/wo	0	Yes
	Protected I	No		Pit Latrine/Finished ro	Rambo/wo	1	Yes
	Open Sprin	No		Pit Latrine/Finished ro	Rambo/wo	1	No
	Piped outsi	No		Pit Latrine/Finished ro	Rambo/wo	0	No
	Protected I	No		Pit Latrine/Finished ro	Rambo/wo	1	Yes
	River	No		No facility/Finished ro	Rambo/wo	1	No
2	Protected S	No		Pit Latrine/Finished ro	Rambo/wo	1	Yes
	Open Sprin	No		Pit Latrine/Finished ro	Rambo/wo	0	Yes
1	Covered W	No		No facility/Finished ro	Rambo/wo	1	No
	Protected I	No		Pit Latrine/Finished ro	Rambo/wo	1	Yes
	Open Sprin	No		Pit Latrine/Finished ro	Rambo/wo	0	Yes
	Covered W	No		Pit Latrine/Finished ro	Rambo/wo	1	No
	Open Sprin	No		Pit Latrine/Finished ro	Rambo/wo	1	Yes
1	Protected I	No		Pit Latrine/Finished ro	Rambo/wo	1	Yes
1	Protected I	No		Pit Latrine/Finished ro	Rambo/wo	1	Yes
	Open Sprin	No		No facility/Finished ro	Rambo/wo	1	Yes
	Covered W	No		Pit Latrine/Finished ro	Rambo/wo	1	Yes
	Piped outsi	Yes	Add bleach	Pit Latrine/Finished ro	Rambo/wo	1	No
	River	No		No facility/Finished ro	Rambo/wo	0	No
	River	No		No facility/Finished ro	Rambo/wo	0	Yes
1	River	No		Pit Latrine/Finished ro	Rambo/wo	2	Yes
	River	No		No facility/Finished ro	Rambo/wo	2	Yes
	River	No		Pit Latrine/Finished ro	Rambo/wo	1	Yes
	River	No		Pit Latrine/Finished ro	Rambo/wo	0	Yes
1	River	No		No facility/Finished ro	Rambo/wo	0	No
	Protected I	No		Pit Latrine/Finished ro	Rambo/wo	1	Yes
	Protected I	No		Pit Latrine/Finished ro	Rambo/wo	0	No
1	River	No		No facility/Finished ro	Rambo/wo	1	Yes
	Protected I	No		Pit Latrine/Finished ro	Rambo/wo	0	Yes
	Protected I	Yes	Add bleach	Pit Latrine/Finished ro	Rambo/wo	2	No
	River	No		No facility/Finished ro	Rambo/wo	0	Yes
1	Protected I	No		Pit Latrine/Finished ro	Rambo/wo	0	Yes
	River	No		No facility/Finished ro	Rambo/wo	0	Yes

	Open Sprin	Yes	Let it stand	No facility/	Finished ro	Rambo/wo	0	Yes
	River	No		No facility/	Finished ro	Rambo/wo	0	No
	Protected I	No		Pit Latrine/	Finished ro	Rambo/wo	1	Yes
	River	No		No facility/	Finished ro	Rambo/wo	0	Yes
	Protected I	No		Pit Latrine/	Finished ro	Rambo/wo	1	Yes
	River	No		Other	Finished ro	Rambo/wo	0	Yes
	Piped outsi	No		Pit Latrine/	Finished ro	Rambo/wo	1	No
	Protected I	No		Pit Latrine/	Finished ro	Rambo/wo	0	Yes
	Open Sprin	No		No facility/	Finished ro	Rambo/wo	1	Yes
	Protected S	No		Pit Latrine/	Finished ro	Rambo/wo	1	Yes
	River	No		No facility/	Finished ro	Rambo/wo	1	Yes
	Protected I	No		No facility/	Finished ro	Rambo/wo	1	No
	Piped outsi	No		Pit Latrine/	Finished ro	Rambo/wo	1	No
	Protected I	Yes	Add bleach	No facility/	Finished ro	Rambo/wo	1	Yes
	River	No		Pit Latrine/	Finished ro	Rambo/wo	1	Yes
	Piped outsi	No		No facility/	Finished ro	Rambo/wo	1	No
	River	No		Pit Latrine/	Finished ro	Rambo/wo	1	Yes
	Open Sprin	No		Pit Latrine/	Finished ro	Rambo/wo	0	Yes
	Open Sprin	No		No facility/	Finished ro	Rambo/wo	1	Yes
	River	No		Pit Latrine/	Finished ro	Rambo/wo	1	Yes
1	River	No		Pit Latrine/	Finished ro	Rambo/wo	1	No
	Protected I	No		Pit Latrine/	Finished ro	Rambo/wo	0	Yes
	Open Sprin	No		Pit Latrine/	Finished ro	Rambo/wo	1	No
	Covered W	No		Pit Latrine/	Finished ro	Rambo/wo	1	No
	Open Sprin	No		Pit Latrine/	Finished ro	Rambo/wo	1	Yes
3	River	No		Pit Latrine/	Finished ro	Rambo/wo	1	Yes
	Protected I	Yes	Add bleach	Pit Latrine/	Finished ro	Rambo/wo	1	Yes
	River	No		No facility/	Finished ro	Rambo/wo	2	Yes
	River	No		No facility/	Finished ro	Rambo/wo	2	Yes
	Protected I	No		Pit Latrine/	Finished ro	Rambo/wo	1	Yes
	River	No		No facility/	Thatch/lea	Rambo/wo	0	Yes
	Protected S	No		Pit Latrine/	Finished ro	Rambo/wo	0	Yes
	Open Sprin	No		No facility/	Finished ro	Rambo/wo	1	Yes
	Covered W	No		Pit Latrine/	Finished ro	Rambo/wo	0	Yes
1	Covered W	No		No facility/	Finished ro	Rambo/wo	1	Yes
	Protected I	No		Pit Latrine/	Finished ro	Rambo/wo	4	Yes
	River	No		No facility/	Finished ro	Rambo/wo	0	Yes
	River	Yes	Let it stand	No facility/	Finished ro	Rambo/wo	0	Yes
	River	No		Pit Latrine/	Finished ro	Rambo/wo	1	Yes
	River	No		Pit Latrine/	Finished ro	Rambo/wo	0	Yes
	Protected I	No		Pit Latrine/	Finished ro	Rambo/wo	1	Yes
	River	No		No facility/	Finished ro	Rambo/wo	0	Yes
	Protected I	No		No facility/	Finished ro	Rambo/wo	0	Yes
	River	No		Pit Latrine/	Finished ro	Rambo/wo	0	Yes
	Piped outsi	No		No facility/	Finished ro	Rambo/wo	1	Yes
1	River	No		No facility/	Finished ro	Rambo/wo	0	Yes
	River	No		No facility/	Finished ro	Rambo/wo	1	Yes
	River	No		No facility/	Finished ro	Rambo/wo	0	Yes
	Piped outsi	No		No facility/	Finished ro	Rambo/wo	0	No
	Protected I	No		Pit Latrine/	Finished ro	Rambo/wo	1	Yes
	Open Sprin	No		Pit Latrine/	Finished ro	Rambo/wo	1	Yes
	Open Sprin	No		Pit Latrine/	Finished ro	Rambo/wo	1	Yes

For peer review only

1	River	No		No facility/ Finished ro	Rambo/wo	0	No
	Protected I	No		Pit Latrine/ Finished ro	Rambo/wo	1	Yes
	River	No		No facility/ Finished ro	Rambo/wo	0	Yes
1	River	Yes	Let it stand	Pit Latrine/ Finished ro	Rambo/wo	2	Yes
	Open Sprin	No		No facility/ Finished ro	Rambo/wo	1	Yes
	Open Sprin	No		Pit Latrine/ Finished ro	Rambo/wo	2	Yes
	River	No		Pit Latrine/ Finished ro	Rambo/wo	1	Yes
	Protected I	Yes	Add bleach	Pit Latrine/ Finished ro	Rambo/wo	1	Yes
1	Open Sprin	No		No facility/ Finished ro	Rambo/wo	2	Yes
	Protected I	No		Pit Latrine/ Finished ro	Rambo/wo	1	No
	Protected I	No		Pit Latrine/ Finished ro	Rambo/wo	1	Yes
	Protected S	No		No facility/ Finished ro	Rambo/wo	1	No
1	Protected I	No		Pit Latrine/ Finished ro	Rambo/wo	1	Yes
	River	No		No facility/ Finished ro	Rambo/wo	0	No
	Piped outsi	No		No facility/ Finished ro	Rambo/wo	0	Yes
	Protected I	No		Pit Latrine/ Finished ro	Rambo/wo	0	Yes
	Protected I	No		Pit Latrine/ Finished ro	Rambo/wo	1	Yes
	Protected I	No		No facility/ Finished ro	Rambo/wo	0	No
	Covered W	No		Pit Latrine/ Finished ro	Rambo/wo	1	No
2	Open Sprin	No		No facility/ Finished ro	Rambo/wo	1	No
	Protected I	No		Pit Latrine/ Finished ro	Rambo/wo	1	Yes
	Protected S	No		Pit Latrine/ Finished ro	Rambo/wo	1	Yes
	Open Sprin	No		Pit Latrine/ Finished ro	Rambo/wo	0	Yes
	River	No		No facility/ Finished ro	Rambo/wo	0	Yes
	Open Sprin	Yes	Let it stand	No facility/ Finished ro	Rambo/wo	1	Yes
	Protected I	No		Pit Latrine/ Finished ro	Rambo/wo	1	Yes
	River	No		Pit Latrine/ Finished ro	Rambo/wo	1	Yes
1	River	No		No facility/ Finished ro	Rambo/wo	1	Yes
1	River	No		No facility/ Finished ro	Rambo/wo	2	Yes
	River	No		No facility/ Finished ro	Rambo/wo	1	Yes
	Covered W	No		Pit Latrine/ Finished ro	Rambo/wo	1	No
	Protected I	Yes	Add bleach	No facility/ Finished ro	Rambo/wo	1	No
	River	Yes	Add bleach	Pit Latrine/ Finished ro	Rambo/wo	1	No
	River	No		Pit Latrine/ Finished ro	Rambo/wo	0	No
	River	No		No facility/ Finished ro	Rambo/wo	1	Yes
2	Open Sprin	No		Pit Latrine/ Finished ro	Rambo/wo	0	No
1	River	No		Pit Latrine/ Finished ro	Rambo/wo	1	Yes
	River	No		No facility/ Finished ro	Rambo/wo	2	Yes
	River	No		No facility/ Finished ro	Rambo/wo	0	Yes
1	River	No		Pit Latrine/ Finished ro	Rambo/wo	1	Yes
	River	No		No facility/ Finished ro	Rambo/wo	1	Yes
	Piped outsi	No		No facility/ Finished ro	Rambo/wo	1	Yes
	Piped outsi	No		No facility/ Finished ro	Rambo/wo	1	No
	Open Sprin	No		No facility/ Finished ro	Other	1	Yes
	Pond/Lake,	No		Pit Latrine/ Finished ro	Rambo/wo	1	Yes
	Open Sprin	No		No facility/ Finished ro	Cement	1	No
	Piped outsi	No		Pit Latrine/ Finished ro	Rambo/wo	0	No
	Piped outsi	No		Pit Latrine/ Finished ro	Rambo/wo	1	Yes
	Open Sprin	Yes	Boil	No facility/ Finished ro	Rambo/wo	2	Yes
	Open Well	No		No facility/ Finished ro	Rambo/wo	1	Yes
	Pond/Lake,	Yes	Boil	Pit Latrine/ Finished ro	Rambo/wo	1	Yes
	Piped outsi	Yes	Boil	Pit Latrine/Thatch/lea	Rambo/wo	2	Yes

For peer review only

	Piped outsi	No		Pit Latrine/	Finished ro	Rambo/wo	1	No
	Piped into (	No		Pit Latrine/	Finished ro	Rambo/wo	1	No
	Piped outsi	No		Pit Latrine/	Finished ro	Rambo/wo	1	Yes
	Open Sprin	No		No facility/	Finished ro	Rambo/wo	1	No
	River	No		Pit Latrine/	Finished ro	Rambo/wo	2	No
	River	No		Pit Latrine/	Finished ro	Rambo/wo	1	Yes
	Open Well	No		No facility/	Finished ro	Rambo/wo	1	Yes
	Open Well	No		No facility/	Finished ro	Rambo/wo	1	Yes
	Pond/Lake,	No		No facility/	Finished ro	Other	1	Yes
	Covered W	No		Pit Latrine/	Finished ro	Rambo/wo	0	Yes
1	Piped outsi	No		Pit Latrine/	Finished ro	Rambo/wo	0	No
	Piped outsi	No		Pit Latrine/	Finished ro	Rambo/wo	1	No
	River	No		Pit Latrine/	Thatch/lea	Rambo/wo	0	Yes
	Open Well	No		No facility/	Finished ro	Rambo/wo	0	Yes
	Piped outsi	No		Pit Latrine/	Finished ro	Rambo/wo	0	No
	Piped outsi	No		Pit Latrine/	Finished ro	Rambo/wo	1	Yes
	River	No		Pit Latrine/	Finished ro	Rambo/wo	1	Yes
	Open Well	No		No facility/	Finished ro	Rambo/wo	2	Yes
	Open Sprin	No		Pit Latrine/	Finished ro	No walls	2	No
	Pond/Lake,	No		Pit Latrine/	Finished ro	Other	1	Yes
	Pond/Lake,	No		Pit Latrine/	Finished ro	Rambo/wo	1	Yes
1	Covered W	No		Pit Latrine/	Finished ro	Rambo/wo	1	Yes
	Pond/Lake,	No		No facility/	Thatch/lea	Other	1	Yes
	Pond/Lake,	Yes	Strain it thr	No facility/	Finished ro	Rambo/wo	1	Yes
	Piped outsi	No		Pit Latrine/	Finished ro	Rambo/wo	1	Yes
2	Protected I	Yes	Add bleach	Pit Latrine/	Finished ro	Rambo/wo	2	Yes
	Pond/Lake,	Yes	Strain it thr	No facility/	Finished ro	Rambo/wo	1	Yes
	Pond/Lake,	No		Pit Latrine/	Thatch/lea	Other	1	Yes
	Piped outsi	No		Pit Latrine/	Finished ro	Rambo/wo	1	Yes
	Open Sprin	No		No facility/	Finished ro	Rambo/wo	1	Yes
	River	No		No facility/	Thatch/lea	Rambo/wo	1	Yes
	Open Well	No		No facility/	Finished ro	Rambo/wo	1	Yes
	Open Sprin	No		Pit Latrine/	Finished ro	Rambo/wo	1	No
	Pond/Lake,	No		Pit Latrine/	Finished ro	Other	1	No
	Open Sprin	No		Pit Latrine/	Finished ro	Rambo/wo	1	No
	Pond/Lake,	No		No facility/	Finished ro	Rambo/wo	1	Yes
1	Protected I	No		Pit Latrine/	Finished ro	Rambo/wo	2	Yes
	River	No		Pit Latrine/	Finished ro	Rambo/wo	1	No
	Pond/Lake,	No		Pit Latrine/	Finished ro	Rambo/wo	1	Yes
	River	No		No facility/	Finished ro	Rambo/wo	1	No
	River	No		Pit Latrine/	Thatch/lea	Other	1	Yes
	Piped outsi	No		Pit Latrine/	Finished ro	Rambo/wo	1	Yes
	Pond/Lake,	No		No facility/	Thatch/lea	Rambo/wo	0	Yes
1	River	No		Pit Latrine/	Finished ro	Rambo/wo	1	Yes
	Piped outsi	No		Pit Latrine/	Finished ro	Rambo/wo	1	No
	Open Well	No		No facility/	Finished ro	Rambo/wo	1	Yes
	Piped outsi	No		Pit Latrine/	Finished ro	Rambo/wo	1	Yes
	Covered W	No		No facility/	Finished ro	Rambo/wo	0	Yes
	Open Sprin	No		No facility/	Thatch/lea	Cement	0	No
	Open Sprin	Yes	Boil	Pit Latrine/	Thatch/lea	Rambo/wo	1	Yes
	Pond/Lake,	No		No facility/	Finished ro	Rambo/wo	0	Yes
	Piped outsi	No		Pit Latrine/	Finished ro	Rambo/wo	1	Yes

For peer review only

	Pond/Lake,	Yes	Strain it thr	No facility/	Finished ro	Rambo/wo	1	Yes
	Pond/Lake,	No		No facility/	Finished ro	Rambo/wo	0	No
2	River	No		Pit Latrine/	Finished ro	Rambo/wo	2	Yes
	Open Well	No		Pit Latrine/	Thatch/lea	Rambo/wo	1	Yes
	Piped outsi	No		Pit Latrine/	Finished ro	Rambo/wo	1	No
	Open Sprin	No		No facility/	Thatch/lea	Other	0	Yes
	Open Sprin	No		No facility/	Finished ro	Rambo/wo	1	No
	River	No		No facility/	Finished ro	Rambo/wo	1	Yes
	Piped into (	No		Pit Latrine/	Finished ro	Rambo/wo	1	Yes
	Protected I	No		Pit Latrine/	Finished ro	Rambo/wo	1	Yes
	Pond/Lake,	Yes	Boil	No facility/	Finished ro	Rambo/wo	1	Yes
	Open Well	No		No facility/	Finished ro	Rambo/wo	0	Yes
	River	No		Pit Latrine/	Finished ro	Rambo/wo	1	Yes
	Protected I	No		Pit Latrine/	Finished ro	Rambo/wo	1	Yes
	Piped outsi	No		Pit Latrine/	Finished ro	Rambo/wo	1	Yes
1	Pond/Lake,	No		No facility/	Finished ro	Rambo/wo	1	No
	Open Sprin	No		Pit Latrine/	Finished ro	Cement	0	Yes
2	River	No		Pit Latrine/	Finished ro	Rambo/wo	1	Yes
	Open Sprin	No		No facility/	Finished ro	Rambo/wo	1	No
1	Open Sprin	No		No facility/	Finished ro	Rambo/wo	1	Yes
	Open Sprin	No		Pit Latrine/	Finished ro	Rambo/wo	0	Yes
	Piped outsi	No		Pit Latrine/	Finished ro	Rambo/wo	1	Yes
	Pond/Lake,	No		Pit Latrine/	Thatch/lea	Other	1	No
	Open Well	No		No facility/	Finished ro	Rambo/wo	1	Yes
	Open Sprin	No		Pit Latrine/	Finished ro	Rambo/wo	1	Yes
	Open Sprin	No		No facility/	Finished ro	Rambo/wo	1	Yes
	Pond/Lake,	Yes	Boil	Pit Latrine/	Finished ro	Rambo/wo	1	No
	Open Sprin	No		No facility/	Finished ro	Cement	1	Yes
	Open Well	No		No facility/	Finished ro	Rambo/wo	1	Yes
	Protected S	No		Pit Latrine/	Finished ro	Rambo/wo	0	Yes
	Piped outsi	No		Pit Latrine/	Finished ro	Rambo/wo	2	Yes
	Open Well	No		No facility/	Finished ro	Rambo/wo	1	Yes
	Open Sprin	No		No facility/	Finished ro	Rambo/wo	2	Yes
	Pond/Lake,	No		No facility/	Finished ro	Rambo/wo	1	Yes
	Covered W	No		No facility/	Finished ro	Rambo/wo	1	Yes
	Piped outsi	No		No facility/	Finished ro	Rambo/wo	1	Yes
	Open Well	No		Pit Latrine/	Finished ro	Rambo/wo	1	Yes
	Open Sprin	No		No facility/	Finished ro	Cement	0	No
	River	No		No facility/	Thatch/lea	Other	1	Yes
	Pond/Lake,	Yes	Add bleach	Pit Latrine/	Finished ro	Other	1	No
	Pond/Lake,	No		No facility/	Thatch/lea	Rambo/wo	0	Yes
	River	No		No facility/	Finished ro	Rambo/wo	1	Yes
	Open Sprin	No		No facility/	Thatch/lea	Cement	0	Yes
	River	No		Pit Latrine/	Finished ro	Rambo/wo	1	Yes
2	Open Sprin	No		No facility/	Finished ro	Rambo/wo	1	Yes
	Piped outsi	No		Pit Latrine/	Finished ro	Rambo/wo	1	Yes
	River	Yes	Add bleach	No facility/	Thatch/lea	Other	1	No
	Open Well	No		No facility/	Finished ro	Rambo/wo	1	Yes
	River	No		No facility/	Thatch/lea	Rambo/wo	0	Yes
	Open Sprin	No		No facility/	Finished ro	Cement	1	Yes
	Open Well	No		Pit Latrine/	Finished ro	Rambo/wo	1	Yes
	River	No		No facility/	Thatch/lea	Rambo/wo	0	Yes

For peer review only

	Piped outsi	No		No facility/	Finished ro	Rambo/wo	1	Yes
	Piped outsi	No		Pit Latrine/	Finished ro	Rambo/wo	1	No
	River	No		No facility/	Thatch/lea	Rambo/wo	1	No
	Pond/Lake,	No		No facility/	Thatch/lea	Other	1	Yes
	Open Sprin	No		No facility/	Thatch/lea	Cement	1	Yes
1	Open Sprin	No		No facility/	Finished ro	Rambo/wo	0	Yes
	Open Well	No		No facility/	Roofing shi	Rambo/wo	0	Yes
	Pond/Lake,	No		Pit Latrine/	Finished ro	Rambo/wo	0	Yes
	Pond/Lake,	Yes	Strain it thr	No facility/	Finished ro	Rambo/wo	1	Yes
	Open Sprin	No		Pit Latrine/	Finished ro	Rambo/wo	0	Yes
	Pond/Lake,	No		Pit Latrine/	Finished ro	Rambo/wo	1	No
1	Pond/Lake,	No		No facility/	Finished ro	Rambo/wo	0	Yes
	River	No		No facility/	Thatch/lea	Rambo/wo	0	Yes
2	Open Sprin	No		Pit Latrine/	Thatch/lea	Cement	0	Yes
	Open Well	No		Pit Latrine/	Finished ro	Rambo/wo	1	Yes
	Pond/Lake,	No		Pit Latrine/	Finished ro	Rambo/wo	1	Yes
	Pond/Lake,	No		Pit Latrine/	Finished ro	Rambo/wo	0	Yes
	Open Sprin	No		Pit Latrine/	Finished ro	Other	1	Yes
	Open Well	No		No facility/	Finished ro	Rambo/wo	1	Yes
	Covered W	No		Pit Latrine/	Finished ro	Rambo/wo	0	Yes
	Covered W	No		Pit Latrine/	Finished ro	Rambo/wo	0	Yes
	Open Well	No		Pit Latrine/	Finished ro	Rambo/wo	1	Yes
	Open Sprin	No		Pit Latrine/	Finished ro	Rambo/wo	1	No
	River	No		No facility/	Thatch/lea	Other	1	Yes
1	Open Sprin	No		Pit Latrine/	Thatch/lea	Cement	1	Yes
	Pond/Lake,	No		No facility/	Thatch/lea	Other	1	Yes
	Open Sprin	No		Pit Latrine/	Finished ro	Rambo/wo	1	Yes
	Piped outsi	No		Pit Latrine/	Finished ro	Rambo/wo	0	No
	Piped outsi	No		Pit Latrine/	Finished ro	Rambo/wo	1	Yes
	Piped into	No		Pit Latrine/	Finished ro	Rambo/wo	1	No
	Pond/Lake,	No		No facility/	Finished ro	Rambo/wo	1	No
	Piped outsi	No		Pit Latrine/	Finished ro	Rambo/wo	1	No
	Pond/Lake,	No		No facility/	Finished ro	Rambo/wo	0	Yes
	Open Well	No		No facility/	Thatch/lea	Rambo/wo	0	Yes
	Open Well	No		No facility/	Thatch/lea	Rambo/wo	1	Yes
	Open Sprin	No		No facility/	Thatch/lea	Cement	0	No
2	Protected S	No		No facility/	Finished ro	Rambo/wo	0	Yes
	Open Well	No		No facility/	Finished ro	Rambo/wo	1	Yes
	Open Well	No		Pit Latrine/	Finished ro	Rambo/wo	1	Yes
	Protected S	No		Pit Latrine/	Finished ro	Rambo/wo	1	Yes
	Open Sprin	No		No facility/	Finished ro	Rambo/wo	1	Yes
	River	No		No facility/	Thatch/lea	Rambo/wo	0	Yes
	Open Well	No		Pit Latrine/	Finished ro	Rambo/wo	1	Yes
	River	No		No facility/	Finished ro	Rambo/wo	2	Yes
	Piped outsi	No		No facility/	Finished ro	Rambo/wo	1	Yes
	River	No		No facility/	Finished ro	Rambo/wo	1	Yes
	Open Sprin	No		No facility/	Finished ro	Cement	1	Yes
	Open Well	No		No facility/	Thatch/lea	Rambo/wo	0	Yes
2	Open Sprin	No		Pit Latrine/	Finished ro	Rambo/wo	1	Yes
	Pond/Lake,	Yes	Strain it thr	No facility/	Finished ro	Rambo/wo	1	Yes
	Pond/Lake,	No		Pit Latrine/	Finished ro	Other	1	No
	Piped outsi	No		No facility/	Finished ro	Rambo/wo	1	Yes

For peer review only

	Open Well	No		No facility/ Finished ro	Rambo/wo	0	Yes
	Open Well	No		No facility/ Finished ro	Rambo/wo	1	Yes
	Covered W	Yes	Boil	No facility/ Thatch/lea	Rambo/wo	2	Yes
	Piped outsi	No		Pit Latrine/ Finished ro	Rambo/wo	2	Yes
	Piped outsi	No		Pit Latrine/ Finished ro	Rambo/wo	1	Yes
	Open Sprin	No		Pit Latrine/ Finished ro	Rambo/wo	2	Yes
	Open Sprin	Yes	Add bleach	No facility/ Thatch/lea	Cement	1	Yes
2	Open Sprin	No		No facility/ Finished ro	Rambo/wo	2	Yes
2	Protected I	Yes	Add bleach	Pit Latrine/ Finished ro	Rambo/wo	1	Yes
	Piped outsi	No		Pit Latrine/ Finished ro	Rambo/wo	1	Yes
	Open Sprin	No		No facility/ Thatch/lea	Cement	0	Yes
	Piped outsi	No		No facility/ Finished ro	Rambo/wo	0	Yes
	Open Sprin	No		No facility/ Thatch/lea	Cement	1	No
	Open Well	No		No facility/ Finished ro	Rambo/wo	1	Yes
	Pond/Lake,	Yes	Boil	Pit Latrine/ Finished ro	Rambo/wo	2	Yes
	Open Sprin	No		No facility/ Finished ro	Rambo/wo	1	No
	River	No		No facility/ Finished ro	Rambo/wo	1	Yes
	River	No		No facility/ Thatch/lea	Other	1	No
	River	No		No facility/ Thatch/lea	Rambo/wo	0	No
	Open Well	No		Pit Latrine/ Finished ro	Rambo/wo	1	Yes
	Protected S	No		Pit Latrine/ Finished ro	Rambo/wo	1	Yes
	Piped outsi	No		Pit Latrine/ Finished ro	Stone with	0	No
	Piped outsi	No		Pit Latrine/ Finished ro	Rambo/wo	2	Yes
	Protected S	No		Pit Latrine/ Finished ro	Rambo/wo	1	Yes
1	Covered W	No		No facility/ Finished ro	Rambo/wo	0	Yes
	Piped outsi	No		No facility/ Finished ro	Rambo/wo	1	No
	Protected I	No		Pit Latrine/ Finished ro	Rambo/wo	0	No
1	Covered W	No		Pit Latrine/ Finished ro	Rambo/wo	2	No
	Covered W	No		Pit Latrine/ Finished ro	Rambo/wo	0	No
	Piped outsi	No		Pit Latrine/ Finished ro	Rambo/wo	0	No
	Covered W	No		Pit Latrine/ Finished ro	Rambo/wo	2	No
	River	No		Pit Latrine/ Finished ro	Rambo/wo	0	Yes
	Open Well	No		No facility/ Finished ro	Rambo/wo	0	No
	Piped into (	No		Pit Latrine/ Finished ro	Rambo/wo	2	Yes
1	Piped outsi	No		Pit Latrine/ Finished ro	Rambo/wo	0	Yes
	Covered W	No		Pit Latrine/ Finished ro	Rambo/wo	0	Yes
	Covered W	No		Pit Latrine/ Finished ro	Rambo/wo	0	Yes
	Piped outsi	No		Pit Latrine/ Finished ro	Rambo/wo	0	Yes
	Covered W	Yes	Add bleach	Pit Latrine/ Finished ro	Rambo/wo	0	No
	Protected S	No		No facility/ Finished ro	Rambo/wo	0	Yes
	Protected S	No		Pit Latrine/ Finished ro	Rambo/wo	1	Yes
1	Covered W	No		Pit Latrine/ Finished ro	Rambo/wo	1	Yes
	Piped outsi	No		Pit Latrine/ Finished ro	Rambo/wo	1	No
	Covered W	No		Pit Latrine/ Finished ro	Rambo/wo	1	Yes
1	Covered W	No		Pit Latrine/ Finished ro	Rambo/wo	2	No
	Piped outsi	No		Pit Latrine/ Finished ro	Rambo/wo	0	No
	Piped outsi	No		No facility/ Finished ro	Rambo/wo	0	No
	Piped outsi	No		Pit Latrine/ Finished ro	Rambo/wo	0	Yes
	Covered W	No		Pit Latrine/ Finished ro	Rambo/wo	2	No
	Piped into (	No		Pit Latrine/ Finished ro	Rambo/wo	2	No
	Piped into (	No		Pit Latrine/ Wood plan	Rambo/wo	1	No
	Covered W	No		Pit Latrine/ Finished ro	Rambo/wo	1	No

For peer review only

	Covered W	No		Pit Latrine/	Finished ro	Rambo/wo	1	No
	Open Well	No		Pit Latrine/	Finished ro	Rambo/wo	0	Yes
1	Piped into (	No		Pit Latrine/	Finished ro	Rambo/wo	2	Yes
	Piped outsi	No		Pit Latrine/	Finished ro	Rambo/wo	1	Yes
	Covered W	No		Pit Latrine/	Finished ro	Rambo/wo	2	Yes
	Covered W	No		Pit Latrine/	Finished ro	Rambo/wo	0	No
	Piped outsi	No		Pit Latrine/	Finished ro	Rambo/wo	0	Yes
	Protected S	No		Pit Latrine/	Finished ro	Rambo/wo	0	No
	Protected S	No		Pit Latrine/	Finished ro	Rambo/wo	0	Yes
	Piped into (	No		Pit Latrine/	Finished ro	Rambo/wo	2	Yes
	Covered W	No		Pit Latrine/	Finished ro	Stone with	0	No
	Piped outsi	No		Pit Latrine/	Finished ro	Rambo/wo	0	Yes
	Covered W	No		Pit Latrine/	Finished ro	Rambo/wo	1	Yes
	River	No		Pit Latrine/	Finished ro	Rambo/wo	0	Yes
	Piped outsi	No		Pit Latrine/	Finished ro	Rambo/wo	1	No
	Piped into (	No		Pit Latrine/	Finished ro	Rambo/wo	2	Yes
	Piped outsi	No		No facility/	Finished ro	Rambo/wo	0	No
	Piped outsi	No		Pit Latrine/	Finished ro	Rambo/wo	1	Yes
	Covered W	No		Pit Latrine/	Finished ro	Rambo/wo	1	No
7	Piped outsi	No		Pit Latrine/	Finished ro	Rambo/wo	2	Yes
	Piped outsi	No		Pit Latrine/	Finished ro	Rambo/wo	3	No
	Covered W	No		Pit Latrine/	Finished ro	Rambo/wo	1	No
	Covered W	No		Pit Latrine/	Finished ro	Rambo/wo	0	Yes
	Protected S	No		Pit Latrine/	Finished ro	Rambo/wo	0	No
	Piped outsi	No		Pit Latrine/	Finished ro	Rambo/wo	0	No
	Covered W	No		Pit Latrine/	Finished ro	Rambo/wo	0	No
	Piped outsi	No		Pit Latrine/	Finished ro	Rambo/wo	0	No
	Protected S	No		Pit Latrine/	Finished ro	Rambo/wo	0	No
	Covered W	No		Pit Latrine/	Finished ro	Rambo/wo	0	Yes
	Open Sprin	No		No facility/	Finished ro	No walls	1	No
	Covered W	Yes	Add bleach	Pit Latrine/	Finished ro	Rambo/wo	1	No
1	Piped outsi	No		Pit Latrine/	Finished ro	Rambo/wo	1	No
	Protected S	No		Pit Latrine/	Finished ro	Rambo/wo	0	Yes
	Piped into (	No		Pit Latrine/	Finished ro	Rambo/wo	2	No
1	Piped outsi	Yes	Add bleach	Pit Latrine/	Finished ro	Rambo/wo	1	Yes
2	Piped into (	No		Pit Latrine/	Finished ro	Rambo/wo	0	Yes
	Piped outsi	No		Pit Latrine/	Finished ro	Rambo/wo	1	No
	Piped into (	No		Pit Latrine/	Finished ro	Rambo/wo	1	No
	Piped outsi	No		Pit Latrine/	Finished ro	Rambo/wo	1	No
	Protected S	No		Pit Latrine/	Finished ro	Rambo/wo	1	Yes
	Open Well	No		Pit Latrine/	Finished ro	Rambo/wo	0	Yes
	Protected S	No		No facility/	Finished ro	Rambo/wo	0	No
	Protected S	No		No facility/	Finished ro	Rambo/wo	0	Yes
	Piped outsi	No		Pit Latrine/	Finished ro	Rambo/wo	1	No
	Covered W	No		Pit Latrine/	Finished ro	Rambo/wo	2	No
1	Covered W	No		Pit Latrine/	Finished ro	Rambo/wo	1	No
	Piped outsi	No		Pit Latrine/	Finished ro	Rambo/wo	1	No
	Covered W	No		Pit Latrine/	Finished ro	Rambo/wo	0	No
	Piped outsi	No		Pit Latrine/	Finished ro	Rambo/wo	1	No
2	Open Well	No		Pit Latrine/	Finished ro	Rambo/wo	0	Yes
	Open Sprin	No		Pit Latrine/	Finished ro	Rambo/wo	0	Yes
	Piped into (	No		Pit Latrine/	Finished ro	Rambo/wo	2	Yes

For peer review only

	Covered W	No		Pit Latrine/ Finished ro	Rambo/wo	1	Yes
	Protected S	No		No facility/ Finished ro	Rambo/wo	1	No
	River	No		Pit Latrine/ Finished ro	Rambo/wo	0	Yes
	Open Sprin	No		Pit Latrine/ Finished ro	Rambo/wo	1	Yes
	Protected S	No		Pit Latrine/ Finished ro	Rambo/wo	1	No
	Piped outsi	No		No facility/ Finished ro	Rambo/wo	1	Yes
	Piped outsi	No		Pit Latrine/ Finished ro	Rambo/wo	1	Yes
	River	No		No facility/ Finished ro	Rambo/wo	0	No
	Covered W	No		Pit Latrine/ Finished ro	Rambo/wo	1	Yes
	Piped outsi	No		Pit Latrine/ Finished ro	Rambo/wo	1	Yes
	Piped into (	No		Pit Latrine/ Finished ro	Rambo/wo	1	Yes
	Covered W	No		Pit Latrine/ Finished ro	Rambo/wo	1	Yes
	Protected S	No		Pit Latrine/ Finished ro	Rambo/wo	0	Yes
	Covered W	No		Pit Latrine/ Finished ro	Rambo/wo	0	No
	Covered W	No		Pit Latrine/ Finished ro	Rambo/wo	0	Yes
	Covered W	No		No facility/ Finished ro	Rambo/wo	1	Yes
	Covered W	No		Pit Latrine/ Finished ro	Rambo/wo	1	Yes
	Covered W	No		Pit Latrine/ Finished ro	Rambo/wo	1	No
	Open Well	No		Pit Latrine/ Finished ro	Rambo/wo	0	No
	Covered W	No		Pit Latrine/ Finished ro	Rambo/wo	1	Yes
	Protected S	No		Pit Latrine/ Finished ro	Rambo/wo	0	Yes
	Protected S	No		No facility/ Finished ro	Rambo/wo	0	Yes
	Open Sprin	No		Pit Latrine/ Finished ro	Rambo/wo	1	No
	Piped outsi	No		Pit Latrine/ Finished ro	Rambo/wo	1	Yes
	Piped outsi	No		Pit Latrine/ Finished ro	Rambo/wo	1	No
1	Covered W	No		Pit Latrine/ Finished ro	Rambo/wo	0	Yes
	Covered W	No		No facility/ Finished ro	Rambo/wo	1	Yes
	Covered W	No		Pit Latrine/ Finished ro	Rambo/wo	0	No
	Covered W	No		No facility/ Finished ro	Rambo/wo	0	No
1	River	No		Pit Latrine/ Finished ro	Rambo/wo	1	Yes
1	Covered W	No		No facility/ Finished ro	Rambo/wo	0	Yes
	Piped outsi	No		Pit Latrine/ Finished ro	Rambo/wo	0	Yes
	Covered W	No		Pit Latrine/ Finished ro	Rambo/wo	0	Yes
	Covered W	No		Pit Latrine/ Finished ro	Rambo/wo	0	Yes
	Covered W	No		Pit Latrine/Wood plan	Rambo/wo	0	Yes
	Piped outsi	No		Pit Latrine/ Finished ro	Rambo/wo	2	Yes
	Covered W	No		Pit Latrine/ Finished ro	Rambo/wo	1	Yes
	Covered W	No		Pit Latrine/ Finished ro	Rambo/wo	0	No
	Covered W	No		No facility/ Finished ro	Rambo/wo	1	Yes
	Covered W	No		Pit Latrine/ Finished ro	Rambo/wo	1	Yes
	Covered W	No		Pit Latrine/ Finished ro	Rambo/wo	0	No
	Protected S	No		Pit Latrine/ Finished ro	Rambo/wo	1	No
	Covered W	Yes	Add bleach	Pit Latrine/ Finished ro	Rambo/wo	0	Yes
1	Covered W	No		Pit Latrine/ Finished ro	Rambo/wo	0	No
	Protected S	No		Pit Latrine/ Finished ro	Rambo/wo	1	No
	Piped outsi	No		Pit Latrine/ Finished ro	Rambo/wo	0	No
	Covered W	No		Pit Latrine/ Finished ro	Stone with	1	Yes
1	Piped outsi	Yes	Add bleach	Pit Latrine/ Finished ro	Rambo/wo	0	Yes
3	Covered W	No		Pit Latrine/ Finished ro	Rambo/wo	0	Yes
	Piped into (	No		Pit Latrine/ Finished ro	Rambo/wo	1	No
4	Covered W	No		Pit Latrine/ Finished ro	Rambo/wo	1	Yes
	Covered W	No		Pit Latrine/ Finished ro	Rambo/wo	1	Yes

For peer review only

	Covered W	No		Pit Latrine/	Finished ro	Rambo/wo	2	No
	Protected I	No		Pit Latrine/	Finished ro	Rambo/wo	1	No
2	Protected S	No		Pit Latrine/	Finished ro	Rambo/wo	1	Yes
1	Covered W	No		Pit Latrine/	Finished ro	Rambo/wo	0	Yes
	Piped outsi	No		Pit Latrine/	Finished ro	Stone with	1	No
1	Covered W	No		Pit Latrine/	Finished ro	Rambo/wo	2	Yes
	Covered W	No		Pit Latrine/	Finished ro	Rambo/wo	0	No
	Covered W	No		Pit Latrine/	Finished ro	Rambo/wo	1	Yes
	Covered W	No		Pit Latrine/	Finished ro	Rambo/wo	0	Yes
	Protected S	No		No facility/	Finished ro	Rambo/wo	1	Yes
	Covered W	No		Pit Latrine/	Finished ro	Rambo/wo	0	Yes
1	Piped outsi	No		Pit Latrine/	Finished ro	Rambo/wo	0	No
1	Covered W	No		Pit Latrine/	Finished ro	Rambo/wo	0	Yes
	Covered W	No		No facility/	Finished ro	Rambo/wo	0	Yes
1	Covered W	No		Pit Latrine/	Finished ro	Rambo/wo	1	Yes
	Piped outsi	No		Pit Latrine/	Finished ro	Rambo/wo	1	No
1	Covered W	No		Pit Latrine/	Finished ro	Rambo/wo	0	Yes
	Covered W	No		Pit Latrine/	Finished ro	Rambo/wo	0	Yes
	Piped into	No		Pit Latrine/	Finished ro	Rambo/wo	1	No
	Covered W	No		Pit Latrine/	Finished ro	Rambo/wo	1	Yes
	Protected S	No		Pit Latrine/	Finished ro	Rambo/wo	1	Yes
2	Protected S	No		No facility/	Finished ro	Rambo/wo	0	Yes
1	Protected S	No		Pit Latrine/	Finished ro	Rambo/wo	1	No
	Piped outsi	No		Pit Latrine/	Finished ro	Rambo/wo	1	Yes
	Covered W	No		Pit Latrine/	Finished ro	Rambo/wo	2	Yes
	Protected S	No		Pit Latrine/	Finished ro	Rambo/wo	0	Yes
	Covered W	No		Pit Latrine/	Finished ro	Stone with	0	No
	Protected S	No		Pit Latrine/	Finished ro	Rambo/wo	1	No
	Piped into	No		Pit Latrine/	Finished ro	Rambo/wo	3	No
	Protected S	No		Pit Latrine/	Finished ro	Rambo/wo	1	Yes
	Piped outsi	No		Pit Latrine/	Finished ro	Rambo/wo	0	No
	Piped outsi	No		No facility/	Finished ro	Rambo/wo	0	No
	Piped outsi	No		Pit Latrine/	Finished ro	Rambo/wo	1	Yes
	Covered W	No		Pit Latrine/	Finished ro	Rambo/wo	1	Yes
	Protected S	No		Pit Latrine/	Finished ro	Rambo/wo	0	No
	Piped outsi	No		Pit Latrine/	Finished ro	Rambo/wo	0	No
	Piped into	No		Pit Latrine/	Finished ro	Rambo/wo	1	No
	Open Sprin	No		Pit Latrine/	Finished ro	Rambo/wo	0	Yes
	Covered W	Yes	Add bleach	Pit Latrine/	Finished ro	Stone with	0	No
	Piped into	No		Pit Latrine/	Finished ro	Rambo/wo	2	No
	Piped outsi	No		Pit Latrine/	Finished ro	Rambo/wo	1	Yes
	Protected S	No		Pit Latrine/	Finished ro	Rambo/wo	0	Yes
2	Piped outsi	No		Pit Latrine/	Finished ro	Rambo/wo	1	Yes
	Covered W	No		Pit Latrine/	Finished ro	Rambo/wo	0	No
	Covered W	No		No facility/	Finished ro	Rambo/wo	0	No
	Piped outsi	No		Pit Latrine/	Finished ro	Rambo/wo	0	Yes
	Covered W	No		Pit Latrine/	Finished ro	Rambo/wo	1	No
1	River	No		Pit Latrine/	Finished ro	Rambo/wo	0	Yes
1	Piped outsi	No		Pit Latrine/	Finished ro	Rambo/wo	1	No
1	Covered W	No		Pit Latrine/	Finished ro	Rambo/wo	0	Yes
	Piped outsi	No		Pit Latrine/	Finished ro	Rambo/wo	1	Yes
	Open Well	No		Pit Latrine/	Finished ro	Rambo/wo	0	No

	Covered W	No	Pit Latrine/	Finished ro	Rambo/wo	1	Yes
	Covered W	No	Pit Latrine/	Finished ro	Rambo/wo	1	No
	Covered W	No	Pit Latrine/	Finished ro	Rambo/wo	0	Yes
	Piped outsi	No	Pit Latrine/	Finished ro	Rambo/wo	1	Yes
	Piped outsi	No	Pit Latrine/	Finished ro	Rambo/wo	1	No
	River	No	Pit Latrine/	Finished ro	Rambo/wo	0	Yes
	Covered W	No	Pit Latrine/	Finished ro	Rambo/wo	1	No
	Piped outsi	No	Pit Latrine/	Finished ro	Rambo/wo	0	Yes
	Protected S	No	Pit Latrine/	Finished ro	Rambo/wo	0	No
	Covered W	No	Pit Latrine/	Finished ro	Rambo/wo	1	Yes
	Covered W	No	No facility/	Finished ro	Rambo/wo	0	Yes
	Piped outsi	No	Pit Latrine/	Finished ro	Rambo/wo	0	No
	Piped outsi	No	Pit Latrine/	Finished ro	Rambo/wo	0	Yes
	Open Sprin	No	Pit Latrine/	Finished ro	Rambo/wo	0	No
	Covered W	No	Pit Latrine/	Finished ro	Rambo/wo	0	Yes
2	Covered W	No	Pit Latrine/	Finished ro	Rambo/wo	1	No
1	Covered W	No	Pit Latrine/	Finished ro	Rambo/wo	0	No
1	Protected S	No	Pit Latrine/	Finished ro	Rambo/wo	1	Yes
	Covered W	No	No facility/	Finished ro	Rambo/wo	1	Yes
	Covered W	No	Pit Latrine/	Finished ro	Rambo/wo	0	No
1	Piped outsi	No	Ventilated	Roofing shi	Cement	1	Yes
	Covered W	No	Pit Latrine/	Finished ro	Rambo/wo	1	No
	Covered W	No	Pit Latrine/	Finished ro	Stone with	0	Yes
	Piped outsi	No	Pit Latrine/	Finished ro	Rambo/wo	0	Yes
	Piped outsi	No	Pit Latrine/	Finished ro	Rambo/wo	1	No
	Covered W	No	Pit Latrine/	Finished ro	Rambo/wo	0	Yes
	Piped outsi	No	Pit Latrine/	Finished ro	Rambo/wo	1	No
	Piped into (	No	Pit Latrine/	Finished ro	Rambo/wo	0	Yes
	River	No	Pit Latrine/	Finished ro	Stone with	0	Yes
	Piped outsi	No	Pit Latrine/	Finished ro	Rambo/wo	1	Yes
	Piped outsi	No	Pit Latrine/	Finished ro	Rambo/wo	0	Yes
	Protected S	No	Pit Latrine/	Finished ro	Rambo/wo	0	Yes
	Covered W	No	Pit Latrine/	Finished ro	Rambo/wo	1	No
	Covered W	No	Pit Latrine/	Finished ro	Rambo/wo	1	Yes
	Covered W	No	Pit Latrine/	Finished ro	Rambo/wo	1	Yes
	Piped outsi	No	Pit Latrine/	Finished ro	Rambo/wo	1	No
	Covered W	No	Pit Latrine/	Finished ro	Rambo/wo	0	No
	Covered W	No	Pit Latrine/	Finished ro	Rambo/wo	2	No
	Covered W	No	Pit Latrine/	Finished ro	Rambo/wo	1	Yes
	Covered W	No	Pit Latrine/	Finished ro	Rambo/wo	0	Yes
1	Piped outsi	No	Pit Latrine/	Finished ro	Rambo/wo	2	No
	Covered W	No	Pit Latrine/	Finished ro	Rambo/wo	1	No
	Piped outsi	No	Pit Latrine/	Finished ro	Rambo/wo	1	Yes
	Covered W	No	No facility/	Finished ro	Rambo/wo	0	No
	Piped outsi	No	Pit Latrine/	Finished ro	Rambo/wo	1	Yes
	Covered W	No	Pit Latrine/	Finished ro	Rambo/wo	0	No
	Protected S	No	Pit Latrine/	Finished ro	Rambo/wo	1	Yes
	Piped outsi	No	Pit Latrine/	Finished ro	Rambo/wo	1	No
	Covered W	No	Pit Latrine/	Finished ro	Rambo/wo	0	Yes
	Piped outsi	No	Pit Latrine/	Finished ro	Rambo/wo	0	Yes
	Piped outsi	No	No facility/	Finished ro	Rambo/wo	0	Yes
1	Covered W	No	No facility/	Finished ro	Rambo/wo	0	Yes

For peer review only

	Open Well	No		Pit Latrine/	Finished ro	Rambo/wo	1	Yes
	Covered W	No		Pit Latrine/	Finished ro	Rambo/wo	0	No
	Covered W	No		Pit Latrine/	Finished ro	Rambo/wo	0	No
	Covered W	No		Pit Latrine/	Finished ro	Rambo/wo	1	No
	Piped outsi	No		Pit Latrine/	Finished ro	Rambo/wo	1	No
2	Covered W	Yes	Add bleach	Pit Latrine/	Finished ro	Rambo/wo	0	No
	Open Well	No		Pit Latrine/	Finished ro	Rambo/wo	0	No
	Covered W	Yes	Add bleach	Pit Latrine/	Finished ro	Stone with	0	No
	Piped outsi	No		Pit Latrine/	Finished ro	Rambo/wo	1	Yes
	Covered W	No		Pit Latrine/	Finished ro	Rambo/wo	1	Yes
	Protected S	No		No facility/	Finished ro	Rambo/wo	1	No
	Covered W	No		Pit Latrine/	Finished ro	Rambo/wo	1	Yes
	Protected S	No		No facility/	Finished ro	Rambo/wo	0	No
1	Covered W	No		Pit Latrine/	Finished ro	Rambo/wo	0	No
	Covered W	No		Pit Latrine/	Finished ro	Rambo/wo	1	Yes
	Piped outsi	No		Pit Latrine/	Finished ro	Rambo/wo	0	Yes
	Covered W	No		Pit Latrine/	Finished ro	Rambo/wo	1	No
	Protected S	No		No facility/	Finished ro	Rambo/wo	0	Yes
	Piped outsi	Yes	Add bleach	Pit Latrine/	Finished ro	Stone with	0	Yes
	Covered W	No		Pit Latrine/	Finished ro	Rambo/wo	0	No
	Piped into	No		Pit Latrine/	Finished ro	Rambo/wo	2	Yes
	Protected S	No		Pit Latrine/	Finished ro	Rambo/wo	1	No
1	Piped outsi	Yes	Add bleach	Pit Latrine/	Finished ro	Rambo/wo	2	No
	Covered W	No		Pit Latrine/	Finished ro	Rambo/wo	0	No
	Covered W	No		Pit Latrine/	Finished ro	Rambo/wo	1	No
	Protected S	No		Pit Latrine/	Finished ro	Rambo/wo	1	Yes
	Piped outsi	No		Pit Latrine/	Finished ro	Rambo/wo	1	No
	Covered W	No		No facility/	Finished ro	Rambo/wo	0	Yes
	Covered W	Yes	Boil	Pit Latrine/	Finished ro	Rambo/wo	0	Yes
	Covered W	No		Pit Latrine/	Finished ro	Rambo/wo	0	Yes
	Piped outsi	No		Pit Latrine/	Finished ro	Rambo/wo	0	Yes
	Piped into	No		Pit Latrine/	Finished ro	Rambo/wo	2	Yes
	Protected S	No		No facility/	Finished ro	Rambo/wo	0	No
	Piped into	No		Pit Latrine/	Finished ro	Rambo/wo	2	No
	Protected S	No		Pit Latrine/	Finished ro	Rambo/wo	0	Yes
	Covered W	No		Pit Latrine/	Finished ro	Rambo/wo	0	Yes
	Covered W	No		Pit Latrine/	Finished ro	Rambo/wo	1	No
	Piped outsi	No		No facility/	Finished ro	Rambo/wo	1	No
1	Covered W	No		Pit Latrine/	Finished ro	Rambo/wo	0	No
	Covered W	No		Pit Latrine/	Finished ro	Rambo/wo	0	No
	Piped outsi	No		Pit Latrine/	Finished ro	Rambo/wo	0	No
	Piped outsi	No		No facility/	Finished ro	Rambo/wo	0	No
	Piped outsi	No		Pit Latrine/	Finished ro	Rambo/wo	2	No
	Piped outsi	No		Pit Latrine/	Finished ro	Rambo/wo	1	Yes
	Covered W	No		Pit Latrine/	Finished ro	Rambo/wo	0	No
	Covered W	No		Pit Latrine/	Finished ro	Rambo/wo	0	No
	Covered W	No		No facility/	Finished ro	Rambo/wo	0	No
1	Covered W	Yes	Add bleach	Pit Latrine/	Finished ro	Rambo/wo	0	Yes
	Piped outsi	No		Pit Latrine/	Finished ro	Rambo/wo	0	No
	Covered W	No		No facility/	Finished ro	Rambo/wo	1	Yes
	Piped outsi	No		Pit Latrine/	Finished ro	Rambo/wo	0	No
1	Covered W	No		Pit Latrine/	Finished ro	Rambo/wo	1	No

For peer review only

	Piped outsi	No		Pit Latrine/	Finished ro	Rambo/wo	2	Yes
	Covered W	No		Pit Latrine/	Finished ro	Rambo/wo	1	Yes
1	Piped outsi	No		Pit Latrine/	Finished ro	Rambo/wo	1	Yes
	Open Well	No		Pit Latrine/	Finished ro	Rambo/wo	0	No
	Covered W	No		Pit Latrine/	Finished ro	Rambo/wo	0	No
	Piped outsi	No		Pit Latrine/	Finished ro	Rambo/wo	0	Yes
	Covered W	No		Pit Latrine/	Finished ro	Stone with	0	Yes
	Open Sprin	No		No facility/	Finished ro	Rambo/wo	0	No
5	Covered W	No		Pit Latrine/	Finished ro	Rambo/wo	1	No
	Covered W	No		No facility/	Finished ro	Rambo/wo	1	No
	Piped outsi	No		No facility/	Finished ro	Rambo/wo	1	Yes
	Piped outsi	No		Pit Latrine/	Finished ro	Rambo/wo	0	No
	Protected S	No		Pit Latrine/	Finished ro	Rambo/wo	0	Yes
	Piped outsi	No		Pit Latrine/	Finished ro	Rambo/wo	1	Yes
	Covered W	No		Pit Latrine/	Finished ro	Rambo/wo	1	No
1	Covered W	No		Pit Latrine/	Finished ro	Rambo/wo	1	No
1	Piped outsi	No		Pit Latrine/	Finished ro	Rambo/wo	0	Yes
	Piped outsi	No		Pit Latrine/	Finished ro	Rambo/wo	0	Yes
	Piped outsi	Yes	Add bleach	Pit Latrine/	Finished ro	Rambo/wo	0	Yes
	Protected H	No		Pit Latrine/	Finished ro	Rambo/wo	2	Yes
	Open Sprin	No		No facility/	Finished ro	Rambo/wo	0	Yes
	Protected H	No		Pit Latrine/	Finished ro	Rambo/wo	1	Yes
	Open Sprin	No		No facility/	Finished ro	Rambo/wo	1	Yes
2	Open Sprin	No		No facility/	Thatch/lea	Rambo/wo	0	Yes
1	Protected H	Yes	Add bleach	Pit Latrine/	Finished ro	Rambo/wo	1	Yes
	Piped outsi	No		Pit Latrine/	Finished ro	Rambo/wo	1	Yes
	River	No		Ventilated	Finished ro	Rambo/wo	1	Yes
	Open Sprin	No		Flush toilet	Finished ro	Rambo/wo	1	Yes
	Protected S	No		Pit Latrine/	Finished ro	Rambo/wo	0	Yes
	Open Sprin	No		No facility/	Finished ro	Rambo/wo	1	Yes
	Protected H	Yes	Add bleach	Pit Latrine/	Finished ro	Rambo/wo	1	Yes
	Protected S	No		Pit Latrine/	Finished ro	Rambo/wo	1	No
	Piped into	No		Pit Latrine/	Finished ro	Rambo/wo	1	No
	Protected S	No		No facility/	Finished ro	Rambo/wo	1	Yes
1	Protected S	No		No facility/	Finished ro	Rambo/wo	0	No
	River	No		Pit Latrine/	Finished ro	Rambo/wo	0	No
	Protected H	Yes	Add bleach	Pit Latrine/	Finished ro	Rambo/wo	1	Yes
	Open Sprin	No		No facility/	Finished ro	Rambo/wo	1	Yes
	Piped into	No		Flush toilet	Finished ro	Rambo/wo	1	No
	Open Sprin	No		Pit Latrine/	Finished ro	Rambo/wo	1	Yes
	Piped into	No		Pit Latrine/	Finished ro	Rambo/wo	1	No
1	Protected S	No		Pit Latrine/	Finished ro	Rambo/wo	1	Yes
	River	No		Pit Latrine/	Finished ro	Rambo/wo	0	Yes
	Protected H	Yes	Add bleach	Pit Latrine/	Finished ro	Rambo/wo	1	Yes
	Piped outsi	Yes	Add bleach	Pit Latrine/	Finished ro	Rambo/wo	1	Yes
	Protected H	Yes	Add bleach	No facility/	Finished ro	Rambo/wo	0	Yes
	Open Sprin	No		Pit Latrine/	Finished ro	Rambo/wo	0	Yes
	Piped outsi	No		No facility/	Finished ro	Rambo/wo	0	Yes
	Open Sprin	No		Pit Latrine/	Finished ro	Rambo/wo	0	Yes
	Protected H	Yes	Add bleach	Pit Latrine/	Finished ro	Rambo/wo	1	Yes
	Open Sprin	No		Pit Latrine/	Finished ro	Rambo/wo	1	Yes
	Protected S	No		Pit Latrine/	Finished ro	Rambo/wo	0	No

For peer review only

2	Open Sprin	No		No facility/	Finished ro	Rambo/wo	1	Yes
	Open Sprin	No		Pit Latrine/	Finished ro	Rambo/wo	1	Yes
	Protected I	Yes	Add bleach	Pit Latrine/	Finished ro	Rambo/wo	1	Yes
	Protected I	No		No facility/	Finished ro	Rambo/wo	0	Yes
	Protected S	No		No facility/	Finished ro	Rambo/wo	1	No
	Protected S	No		Pit Latrine/	Finished ro	Rambo/wo	0	Yes
	Protected I	No		No facility/	Finished ro	Rambo/wo	0	Yes
	Piped outsi	No		No facility/	Finished ro	Rambo/wo	0	No
	Piped into	No		Pit Latrine/	Finished ro	Rambo/wo	1	Yes
	Protected S	No		Pit Latrine/	Finished ro	Rambo/wo	0	No
1	Open Sprin	No		No facility/	Finished ro	Rambo/wo	1	Yes
	River	No		No facility/	Finished ro	Rambo/wo	0	Yes
	Open Sprin	No		Pit Latrine/	Finished ro	Rambo/wo	1	Yes
	Open Well	No		Pit Latrine/	Finished ro	Rambo/wo	1	Yes
	Open Sprin	No		Pit Latrine/	Finished ro	Rambo/wo	1	No
	Piped outsi	No		Pit Latrine/	Finished ro	Rambo/wo	1	Yes
	Piped outsi	No		Pit Latrine/	Finished ro	Rambo/wo	1	Yes
	Protected I	Yes	Add bleach	Pit Latrine/	Finished ro	Rambo/wo	0	Yes
	River	Yes	Boil	No facility/	Finished ro	Rambo/wo	0	No
	Open Sprin	No		No facility/	Finished ro	Rambo/wo	1	Yes
	Protected I	Yes	Add bleach	Pit Latrine/	Finished ro	Rambo/wo	1	Yes
	Piped outsi	No		Pit Latrine/	Finished ro	Rambo/wo	1	Yes
	River	No		Pit Latrine/	Finished ro	Rambo/wo	1	Yes
	Open Sprin	No		No facility/	Finished ro	Rambo/wo	0	No
	Protected S	No		Pit Latrine/	Finished ro	Rambo/wo	1	Yes
1	Protected I	Yes	Add bleach	Pit Latrine/	Finished ro	Rambo/wo	1	Yes
	Piped outsi	No		Pit Latrine/	Finished ro	Rambo/wo	1	No
	Protected I	Yes	Add bleach	Pit Latrine/	Finished ro	Rambo/wo	1	Yes
1	Open Sprin	No		Pit Latrine/	Finished ro	Rambo/wo	1	Yes
	Piped outsi	No		Pit Latrine/	Finished ro	Rambo/wo	0	No
	Piped outsi	No		Pit Latrine/	Finished ro	Rambo/wo	1	No
	Piped outsi	No		Pit Latrine/	Finished ro	Rambo/wo	1	Yes
1	Protected I	Yes	Add bleach	Pit Latrine/	Finished ro	Rambo/wo	1	Yes
1	Open Sprin	No		Pit Latrine/	Finished ro	Stone with	0	Yes
	Protected I	No		No facility/	Finished ro	Rambo/wo	1	Yes
	Protected I	No		Pit Latrine/	Finished ro	Rambo/wo	0	No
	River	No		No facility/	Finished ro	Rambo/wo	0	No
	Piped outsi	No		Pit Latrine/	Finished ro	Rambo/wo	1	Yes
3	Piped outsi	No		Pit Latrine/	Finished ro	Rambo/wo	1	Yes
1	Protected I	Yes	Add bleach	Pit Latrine/	Finished ro	Rambo/wo	1	Yes
	Protected I	Yes	Add bleach	Pit Latrine/	Finished ro	Rambo/wo	2	No
	Piped outsi	No		Pit Latrine/	Finished ro	Rambo/wo	1	Yes
	Open Sprin	No		Flush toilet	Finished ro	Rambo/wo	0	Yes
	Piped outsi	No		No facility/	Finished ro	Rambo/wo	0	Yes
	Protected I	Yes	Add bleach	Pit Latrine/	Finished ro	Rambo/wo	1	Yes
	River	No		Pit Latrine/	Finished ro	Rambo/wo	0	Yes
	River	No		Pit Latrine/	Finished ro	Rambo/wo	1	Yes
	Piped outsi	No		No facility/	Finished ro	Rambo/wo	0	Yes
	Open Sprin	Yes	Let it stand	Pit Latrine/	Finished ro	Rambo/wo	0	Yes
	Open Sprin	No		Pit Latrine/	Finished ro	Rambo/wo	1	Yes
1	Piped outsi	No		No facility/	Finished ro	Rambo/wo	0	Yes
	Open Sprin	No		No facility/	Finished ro	Rambo/wo	1	Yes

For peer review only

1	Open Sprin	No		Pit Latrine/Finished ro	Rambo/wo	1	Yes
	Open Sprin	No		No facility/Finished ro	Rambo/wo	1	Yes
	River	No		No facility/Finished ro	Rambo/wo	1	Yes
	Piped outsi	No		Pit Latrine/Finished ro	Rambo/wo	0	Yes
	Protected S	No		Pit Latrine/Finished ro	Rambo/wo	0	Yes
	Piped outsi	No		Pit Latrine/Finished ro	Rambo/wo	0	No
	Open Sprin	No		Pit Latrine/Finished ro	Rambo/wo	1	Yes
	Protected S	No		Pit Latrine/Finished ro	Rambo/wo	1	Yes
	Piped outsi	No		Pit Latrine/Finished ro	Rambo/wo	1	Yes
	Open Sprin	No		Pit Latrine/Finished ro	Rambo/wo	0	Yes
	River	No		No facility/Finished ro	Rambo/wo	1	No
	Piped outsi	No		No facility/Finished ro	Rambo/wo	1	No
	Piped outsi	No		Pit Latrine/Finished ro	Rambo/wo	1	No
	Piped outsi	No		Pit Latrine/Finished ro	Rambo/wo	1	Yes
2	Protected S	No		Pit Latrine/Finished ro	Rambo/wo	1	Yes
	Piped outsi	Yes	Add bleach	Pit Latrine/Finished ro	Rambo/wo	1	No
	Open Sprin	No		No facility/Finished ro	Rambo/wo	0	Yes
	Piped into	No		No facility/Finished ro	Rambo/wo	1	No
	Protected I	No		Pit Latrine/Finished ro	Rambo/wo	1	Yes
	Protected I	No		No facility/Finished ro	Rambo/wo	1	Yes
	Protected S	No		No facility/Finished ro	Rambo/wo	1	Yes
	Piped outsi	No		Pit Latrine/Finished ro	Rambo/wo	1	Yes
	Open Sprin	No		No facility/Finished ro	Rambo/wo	0	Yes
	River	No		No facility/Finished ro	Rambo/wo	0	Yes
	Open Sprin	No		No facility/Finished ro	Rambo/wo	1	Yes
	Open Sprin	No		Pit Latrine/Finished ro	Rambo/wo	1	Yes
	Open Sprin	No		No facility/Finished ro	Rambo/wo	1	Yes
	Protected I	Yes	Add bleach	Pit Latrine/Finished ro	Rambo/wo	1	Yes
	Piped outsi	Yes	Add bleach	Pit Latrine/Finished ro	Rambo/wo	1	Yes
	Protected S	No		Pit Latrine/Finished ro	Rambo/wo	1	No
1	Piped outsi	Yes	Add bleach	Pit Latrine/Finished ro	Rambo/wo	2	No
	Piped into	No		Pit Latrine/Finished ro	Rambo/wo	1	Yes
	Piped outsi	No		Pit Latrine/Finished ro	Rambo/wo	1	Yes
	Piped outsi	No		No facility/Finished ro	Rambo/wo	0	Yes
	Protected I	Yes	Add bleach	No facility/Finished ro	Rambo/wo	2	No
	Piped outsi	No		Pit Latrine/Finished ro	Rambo/wo	1	No
	Protected S	No		No facility/Finished ro	Rambo/wo	1	Yes
	Open Sprin	No		No facility/Finished ro	Rambo/wo	1	Yes
	Open Sprin	No		Pit Latrine/Finished ro	Rambo/wo	1	Yes
	Protected I	Yes	Add bleach	Pit Latrine/Finished ro	Rambo/wo	1	Yes
	Protected I	Yes	Add bleach	Pit Latrine/Finished ro	Rambo/wo	1	No
	Protected I	Yes	Add bleach	Pit Latrine/Finished ro	Rambo/wo	1	No
	Piped outsi	No		No facility/Finished ro	Rambo/wo	1	No
	Protected S	No		Pit Latrine/Finished ro	Rambo/wo	1	Yes
	Open Sprin	No		No facility/Finished ro	Rambo/wo	0	Yes
	Open Sprin	No		Pit Latrine/Finished ro	Rambo/wo	1	Yes
1	Open Sprin	No		Pit Latrine/Wood plan	Rambo/wo	1	Yes
	Piped outsi	No		Pit Latrine/Finished ro	No walls	2	No
	Piped outsi	No		No facility/Finished ro	Rambo/wo	0	Yes
	Piped outsi	Yes	Add bleach	Pit Latrine/Finished ro	Rambo/wo	2	No
	Covered W	No		Pit Latrine/Finished ro	Rambo/wo	1	No
1	Open Sprin	No		No facility/Wood plan	Rambo/wo	0	No

For peer review only

	Protected S	No		Pit Latrine/Finished ro	Rambo/wo	0	No
	Protected I	No		Pit Latrine/Finished ro	Rambo/wo	1	Yes
	Piped outsi	No		Pit Latrine/Finished ro	Rambo/wo	1	Yes
	Protected I	Yes	Add bleach	Pit Latrine/Finished ro	Rambo/wo	1	Yes
	River	No		Pit Latrine/Finished ro	Rambo/wo	0	Yes
	Protected I	Yes	Add bleach	Pit Latrine/Finished ro	Rambo/wo	1	No
	Open Sprin	No		No facility/ Finished ro	Rambo/wo	1	Yes
	Open Sprin	No		Pit Latrine/Finished ro	Rambo/wo	1	Yes
1	Piped outsi	No		No facility/ Finished ro	Rambo/wo	0	Yes
1	Protected I	Yes	Add bleach	Pit Latrine/Finished ro	Rambo/wo	2	Yes
	Protected I	No		Pit Latrine/Finished ro	Rambo/wo	1	Yes
	Covered W	No		Pit Latrine/Finished ro	Other	0	Yes
1	Open Sprin	No		Pit Latrine/Finished ro	Rambo/wo	1	No
	Protected S	No		No facility/ Finished ro	Rambo/wo	0	Yes
	Open Sprin	No		Pit Latrine/Finished ro	Rambo/wo	1	Yes
	Piped outsi	No		No facility/ Finished ro	Rambo/wo	0	Yes
	Open Sprin	No		Pit Latrine/Finished ro	Rambo/wo	0	No
	Protected S	No		No facility/ Finished ro	Rambo/wo	1	Yes
	Piped outsi	No		Pit Latrine/Finished ro	Rambo/wo	1	Yes
	Protected I	No		No facility/ Finished ro	Rambo/wo	1	No
	Open Sprin	No		Pit Latrine/Finished ro	Rambo/wo	1	Yes
	Piped outsi	No		Pit Latrine/Finished ro	Rambo/wo	1	Yes
	Protected S	No		No facility/ Finished ro	Rambo/wo	1	Yes
	Open Sprin	No		Pit Latrine/Finished ro	Rambo/wo	1	Yes
	River	No		No facility/ Finished ro	Rambo/wo	0	Yes
	Protected I	Yes	Add bleach	Pit Latrine/Finished ro	Rambo/wo	2	Yes
	Piped into (	No		Pit Latrine/Finished ro	Rambo/wo	1	No
	Open Sprin	No		No facility/ Thatch/lea	Rambo/wo	0	Yes
1	Piped into (	No		Pit Latrine/Finished ro	Rambo/wo	1	No
1	Piped outsi	No		Pit Latrine/Finished ro	Rambo/wo	0	Yes
1	Protected I	Yes	Add bleach	Pit Latrine/Finished ro	Rambo/wo	1	Yes
	Piped outsi	Yes	Add bleach	Pit Latrine/Finished ro	Rambo/wo	0	Yes
	River	No		Pit Latrine/Finished ro	Rambo/wo	0	Yes
	River	No		No facility/ Finished ro	Rambo/wo	0	Yes
	Piped outsi	No		Pit Latrine/Finished ro	Rambo/wo	1	No
	River	No		No facility/ Finished ro	Rambo/wo	0	Yes
1	Protected I	No		No facility/ Finished ro	Rambo/wo	0	Yes
1	Piped outsi	No		Pit Latrine/Finished ro	Rambo/wo	2	No
1	Protected S	No		Pit Latrine/Finished ro	Rambo/wo	1	No
	Protected S	No		No facility/ Finished ro	Rambo/wo	1	Yes
	Piped outsi	No		Pit Latrine/Finished ro	Rambo/wo	2	Yes
	Piped outsi	No		Pit Latrine/Finished ro	Rambo/wo	1	No
1	Piped outsi	No		Pit Latrine/Finished ro	Rambo/wo	0	Yes
	Protected S	No		Pit Latrine/Finished ro	Rambo/wo	1	Yes
	Protected S	No		No facility/ Finished ro	Rambo/wo	1	No
	River	No		No facility/ Finished ro	Rambo/wo	1	No
	Protected S	No		No facility/ Finished ro	Rambo/wo	1	Yes
	Protected S	No		No facility/ Finished ro	Rambo/wo	1	Yes
	Pond/Lake,	Yes	Boil	No facility/ Finished ro	Rambo/wo	1	Yes
	Piped outsi	No		No facility/ Finished ro	Rambo/wo	1	Yes
	Protected S	No		Pit Latrine/Finished ro	Rambo/wo	0	Yes
	Piped outsi	No		Pit Latrine/Finished ro	Rambo/wo	1	Yes

For peer review only

	Piped into (	Yes	Boil	Pit Latrine/	Finished ro	Cement	3	No
2	Piped outsi	No		No facility/	Finished ro	Rambo/wo	0	Yes
	Protected I	No		Pit Latrine/	Finished ro	Rambo/wo	1	No
	Protected S	No		Pit Latrine/	Finished ro	Rambo/wo	1	No
	Piped outsi	No		No facility/	Finished ro	Rambo/wo	0	Yes
	Open Sprin	No		Pit Latrine/	Finished ro	Rambo/wo	0	Yes
	Protected I	No		Pit Latrine/	Finished ro	Rambo/wo	0	Yes
	Piped outsi	No		Pit Latrine/	Finished ro	Rambo/wo	0	No
	Protected S	No		Pit Latrine/	Finished ro	Rambo/wo	1	No
2	Protected S	No		No facility/	Finished ro	Rambo/wo	1	Yes
	Open Sprin	No		Pit Latrine/	Finished ro	Rambo/wo	2	Yes
	Piped outsi	No		Pit Latrine/	Finished ro	Rambo/wo	1	Yes
	River	No		Pit Latrine/	Finished ro	Rambo/wo	1	Yes
1	Open Well	No		Pit Latrine/	Finished ro	Rambo/wo	0	Yes
2	Piped outsi	No		Pit Latrine/	Finished ro	Rambo/wo	0	Yes
2	Piped outsi	No		Pit Latrine/	Finished ro	Rambo/wo	1	Yes
	Open Well	No		No facility/	Finished ro	Rambo/wo	1	No
	Piped outsi	No		Pit Latrine/	Finished ro	Rambo/wo	1	Yes
	Open Sprin	No		Pit Latrine/	Finished ro	Rambo/wo	1	No
	Piped outsi	No		No facility/	Finished ro	Other	1	Yes
	Protected S	No		Pit Latrine/	Finished ro	Rambo/wo	1	No
	Open Sprin	No		Pit Latrine/	Finished ro	Rambo/wo	1	Yes
	Piped outsi	No		No facility/	Finished ro	Rambo/wo	0	Yes
	Piped outsi	No		Pit Latrine/	Finished ro	Rambo/wo	0	No
	Piped outsi	No		Pit Latrine/	Finished ro	Rambo/wo	0	Yes
	Open Sprin	No		No facility/	Finished ro	Rambo/wo	1	No
	Piped outsi	No		Pit Latrine/	Finished ro	Rambo/wo	1	No
	Open Sprin	No		Pit Latrine/	Finished ro	Rambo/wo	1	Yes
	Piped outsi	No		Pit Latrine/	Finished ro	Rambo/wo	1	Yes
	Piped outsi	No		Pit Latrine/	Finished ro	Rambo/wo	1	No
	Protected I	No		Pit Latrine/	Finished ro	Rambo/wo	1	No
	Open Sprin	No		No facility/	Finished ro	Rambo/wo	0	Yes
	Piped outsi	No		Pit Latrine/	Finished ro	Rambo/wo	1	Yes
	Open Sprin	No		No facility/	Finished ro	Rambo/wo	2	Yes
	Open Sprin	No		No facility/	Finished ro	Rambo/wo	2	Yes
	Piped outsi	No		Pit Latrine/	Finished ro	Rambo/wo	1	Yes
	Piped into (	No		Pit Latrine/	Finished ro	Rambo/wo	1	Yes
	Open Sprin	No		Pit Latrine/	Finished ro	Rambo/wo	0	Yes
	Open Sprin	No		No facility/	Finished ro	Rambo/wo	1	Yes
1	Piped outsi	No		No facility/	Finished ro	Rambo/wo	1	Yes
	Open Sprin	No		Pit Latrine/	Finished ro	Rambo/wo	0	Yes
4	River	No		No facility/	Finished ro	Rambo/wo	0	Yes
	Piped outsi	No		No facility/	Finished ro	Rambo/wo	0	No
	Piped outsi	No		Pit Latrine/	Finished ro	Rambo/wo	1	No
	Piped outsi	No		Pit Latrine/	Finished ro	Rambo/wo	0	Yes
	Piped outsi	No		No facility/	Finished ro	Rambo/wo	1	Yes
1	Piped outsi	No		Pit Latrine/	Finished ro	Rambo/wo	0	Yes
	Piped outsi	No		No facility/	Finished ro	Rambo/wo	1	Yes
	Protected I	No		Pit Latrine/	Finished ro	Rambo/wo	1	Yes
1	Piped outsi	No		Pit Latrine/	Finished ro	Rambo/wo	0	Yes
	Protected S	No		No facility/	Finished ro	Rambo/wo	1	No
	Piped outsi	No		Pit Latrine/	Finished ro	Rambo/wo	2	No

For peer review only

	Protected S	No		Pit Latrine/Finished ro	Rambo/wo	1	Yes
	Piped outsi	No		No facility/Finished ro	Rambo/wo	1	Yes
	Piped outsi	No		No facility/Finished ro	Rambo/wo	2	Yes
	Piped outsi	Yes	Boil	Pit Latrine/Finished ro	Rambo/wo	3	Yes
	Protected S	No		Pit Latrine/Finished ro	Rambo/wo	1	Yes
	River	No		No facility/Finished ro	Rambo/wo	1	No
	Piped outsi	No		Pit Latrine/Finished ro	Rambo/wo	1	Yes
	River	No		Pit Latrine/Finished ro	Rambo/wo	0	Yes
	Open Sprin	No		Pit Latrine/Finished ro	Rambo/wo	1	No
1	Open Sprin	No		Pit Latrine/Finished ro	Rambo/wo	0	Yes
	Piped into (	No		Pit Latrine/Finished ro	Rambo/wo	2	Yes
	River	No		Pit Latrine/Finished ro	Rambo/wo	0	Yes
	Piped outsi	Yes	Add bleach	No facility/Finished ro	Rambo/wo	2	Yes
	Piped outsi	No		Pit Latrine/Finished ro	Rambo/wo	2	Yes
	River	No		Pit Latrine/Finished ro	Rambo/wo	1	Yes
	River	No		Pit Latrine/Finished ro	Rambo/wo	0	Yes
1	River	No		Pit Latrine/Finished ro	Rambo/wo	0	Yes
	Open Sprin	No		Pit Latrine/Finished ro	Rambo/wo	2	Yes
	Protected S	No		No facility/Finished ro	Rambo/wo	0	Yes
	Piped outsi	Yes	Boil	No facility/Finished ro	Rambo/wo	3	No
	Open Well	No		Pit Latrine/Finished ro	Rambo/wo	1	No
1	Piped outsi	No		Pit Latrine/Finished ro	Rambo/wo	2	No
	Protected S	No		No facility/Finished ro	Rambo/wo	1	Yes
	Open Sprin	No		Pit Latrine/Finished ro	Rambo/wo	1	Yes
	Piped outsi	No		Pit Latrine/Finished ro	Rambo/wo	1	No
	Piped outsi	No		Pit Latrine/Finished ro	Rambo/wo	2	Yes
	Piped outsi	No		No facility/Finished ro	Rambo/wo	0	Yes
	Piped outsi	No		Pit Latrine/Finished ro	Rambo/wo	2	Yes
	Open Sprin	No		No facility/Finished ro	Rambo/wo	0	Yes
	Protected S	No		Pit Latrine/Finished ro	Rambo/wo	1	Yes
	Piped into (	Yes	Boil	No facility/Finished ro	Rambo/wo	3	Yes
	Piped outsi	No		Pit Latrine/Finished ro	Rambo/wo	3	Yes
	Piped outsi	No		Pit Latrine/Finished ro	Other	1	No
	Piped outsi	No		Pit Latrine/Finished ro	Rambo/wo	1	Yes
	Piped outsi	No		Pit Latrine/Finished ro	Rambo/wo	1	No
	Piped outsi	No		Pit Latrine/Finished ro	Rambo/wo	1	Yes
	Protected S	No		Pit Latrine/Finished ro	Rambo/wo	1	No
	Protected H	No		No facility/Finished ro	Rambo/wo	1	Yes
	Piped outsi	No		Pit Latrine/Finished ro	Rambo/wo	0	Yes
	Open Sprin	No		Pit Latrine/Finished ro	Rambo/wo	1	Yes
	Covered W	Yes	Boil	Pit Latrine/Finished ro	Rambo/wo	2	Yes
	Protected S	No		Pit Latrine/Finished ro	Rambo/wo	1	Yes
	Open Sprin	No		No facility/Finished ro	Rambo/wo	1	Yes
	Piped outsi	No		Pit Latrine/Finished ro	Rambo/wo	0	Yes
	Piped outsi	No		No facility/Finished ro	Rambo/wo	1	Yes
	Protected H	No		Pit Latrine/Finished ro	Rambo/wo	1	Yes
	Pond/Lake,	No		Pit Latrine/Finished ro	Rambo/wo	0	Yes
1	Pond/Lake,	No		Pit Latrine/Finished ro	Rambo/wo	1	Yes
	Open Sprin	No		No facility/Finished ro	Rambo/wo	0	Yes
	Piped outsi	No		Pit Latrine/Finished ro	Rambo/wo	1	No
	Piped into (	No		Pit Latrine/Finished ro	Rambo/wo	1	Yes
	Piped outsi	No		Pit Latrine/Finished ro	Rambo/wo	1	Yes

For peer review only

	Protected S	No		No facility/Finished ro	Rambo/wo	1	Yes
	Protected I	No		Pit Latrine/Finished ro	Rambo/wo	1	No
	Piped outsi	No		Pit Latrine/Thatch/lea	Rambo/wo	1	Yes
	Protected I	No		No facility/Finished ro	Rambo/wo	1	Yes
	Piped outsi	No		No facility/Finished ro	Rambo/wo	1	Yes
	Open Sprin	No		Pit Latrine/Finished ro	Rambo/wo	0	No
	Open Sprin	No		Pit Latrine/Finished ro	Rambo/wo	0	Yes
	Open Well	No		No facility/Finished ro	Rambo/wo	0	Yes
1	Piped outsi	No		Pit Latrine/Finished ro	Rambo/wo	1	Yes
	Piped into	No		No facility/Finished ro	Rambo/wo	1	Yes
	Open Sprin	No		No facility/Finished ro	Rambo/wo	0	No
2	Open Sprin	No		Pit Latrine/Finished ro	Rambo/wo	2	Yes
	Piped outsi	No		Pit Latrine/Finished ro	Rambo/wo	0	Yes
	Protected S	No		Pit Latrine/Finished ro	Rambo/wo	0	Yes
	Piped outsi	No		Pit Latrine/Finished ro	Rambo/wo	0	Yes
	Piped outsi	No		No facility/Finished ro	Rambo/wo	1	Yes
3	Piped outsi	No		Pit Latrine/Finished ro	Rambo/wo	0	Yes
	Open Sprin	No		No facility/Finished ro	Rambo/wo	1	Yes
1	Protected I	No		Pit Latrine/Finished ro	Rambo/wo	2	Yes
	Open Sprin	No		Pit Latrine/Finished ro	Rambo/wo	1	Yes
	Piped outsi	No		No facility/Finished ro	Other	1	Yes
	Piped outsi	No		No facility/Finished ro	Rambo/wo	1	Yes
	Open Sprin	No		Pit Latrine/Finished ro	Rambo/wo	0	No
	Piped outsi	No		Pit Latrine/Finished ro	Rambo/wo	1	Yes
1	Protected S	No		Pit Latrine/Finished ro	Rambo/wo	0	Yes
	Protected S	No		Pit Latrine/Finished ro	Rambo/wo	1	Yes
	Piped outsi	No		Pit Latrine/Finished ro	Rambo/wo	0	Yes
	Protected S	No		Pit Latrine/Finished ro	Rambo/wo	1	Yes
	Piped outsi	No		No facility/Finished ro	Rambo/wo	1	No
	Piped outsi	No		No facility/Finished ro	Rambo/wo	1	Yes
	Piped outsi	No		No facility/Finished ro	Rambo/wo	1	Yes
	Piped into	No		Pit Latrine/Finished ro	Rambo/wo	0	No
	Pond/Lake,	No		Pit Latrine/Finished ro	Rambo/wo	1	Yes
	Open Sprin	No		Pit Latrine/Finished ro	Rambo/wo	1	No
	Covered W	No		Pit Latrine/Finished ro	Rambo/wo	1	Yes
	Pond/Lake,	Yes	Boil	Pit Latrine/Finished ro	Rambo/wo	1	No
	Open Sprin	No		No facility/Finished ro	Rambo/wo	2	No
	Protected S	No		No facility/Finished ro	Rambo/wo	1	Yes
	Open Sprin	No		No facility/Finished ro	Rambo/wo	2	No
	Piped outsi	No		Pit Latrine/Finished ro	Rambo/wo	2	Yes
1	River	No		Pit Latrine/Finished ro	Rambo/wo	0	Yes
	Piped outsi	No		Pit Latrine/Finished ro	Rambo/wo	1	No
	Pond/Lake,	No		No facility/Finished ro	Rambo/wo	1	No
	River	No		No facility/Finished ro	Rambo/wo	0	Yes
	Piped outsi	Yes	Boil	No facility/Finished ro	Rambo/wo	2	No
	Open Sprin	No		Pit Latrine/Thatch/lea	Rambo/wo	0	No
	Piped outsi	No		Pit Latrine/Finished ro	Rambo/wo	1	No
	Open Sprin	No		Pit Latrine/Finished ro	Rambo/wo	1	Yes
	River	No		Pit Latrine/Finished ro	Rambo/wo	1	No
	Open Well	No		Pit Latrine/Finished ro	Rambo/wo	0	Yes
	Open Well	No		Pit Latrine/Finished ro	Rambo/wo	1	No
	Pond/Lake,	No		Pit Latrine/Finished ro	Rambo/wo	1	Yes

For peer review only

	Piped outsi	Yes	Boil	No facility/	Finished ro	Rambo/wo	2	Yes
	Protected S	No		Pit Latrine/	Finished ro	Rambo/wo	2	Yes
	Open Sprin	No		Pit Latrine/	Finished ro	Rambo/wo	2	No
	Open Sprin	No		Pit Latrine/	Finished ro	Rambo/wo	0	Yes
	Open Sprin	No		No facility/	Finished ro	Rambo/wo	1	Yes
	River	No		No facility/	Finished ro	Rambo/wo	0	Yes
	River	No		Pit Latrine/	Finished ro	Rambo/wo	1	Yes
	Piped outsi	No		Pit Latrine/	Finished ro	Rambo/wo	1	No
	River	No		Pit Latrine/	Finished ro	Rambo/wo	1	No
	Open Sprin	No		Pit Latrine/	Finished ro	Rambo/wo	2	No
	Protected S	No		No facility/	Finished ro	Rambo/wo	0	Yes
	Open Sprin	No		Pit Latrine/	Finished ro	Rambo/wo	0	Yes
	Protected S	No		No facility/	Finished ro	Rambo/wo	0	No
	Protected S	No		Pit Latrine/	Finished ro	Rambo/wo	1	Yes
	Piped outsi	No		Pit Latrine/	Finished ro	Rambo/wo	1	Yes
1	Protected S	No		No facility/	Finished ro	Rambo/wo	1	Yes
	Piped outsi	No		No facility/	Finished ro	Rambo/wo	1	Yes
	Piped outsi	No		Pit Latrine/	Finished ro	Rambo/wo	1	Yes
	Protected I	No		Pit Latrine/	Finished ro	Rambo/wo	2	Yes
2	Protected S	No		No facility/	Finished ro	Rambo/wo	1	Yes
	Covered W	No		Pit Latrine/	Finished ro	Rambo/wo	1	No
	Piped outsi	No		No facility/	Finished ro	Rambo/wo	0	No
	Piped outsi	No		Pit Latrine/	Finished ro	Rambo/wo	1	No
	Protected I	No		No facility/	Finished ro	Rambo/wo	0	No
	Piped outsi	No		No facility/	Finished ro	Rambo/wo	0	Yes
	Piped into (	No		Pit Latrine/	Finished ro	Rambo/wo	1	No
	Piped outsi	No		Pit Latrine/	Finished ro	Rambo/wo	1	Yes
1	Protected S	No		Pit Latrine/	Finished ro	Rambo/wo	0	No
	Piped outsi	No		Pit Latrine/	Finished ro	Rambo/wo	0	Yes
	Protected S	No		Pit Latrine/	Finished ro	Rambo/wo	1	No
3	Piped outsi	No		Pit Latrine/	Finished ro	Rambo/wo	0	Yes
	Piped outsi	No		Pit Latrine/	Finished ro	Rambo/wo	1	No
	Covered W	Yes	Add bleach	No facility/	Finished ro	Rambo/wo	0	Yes
	Piped outsi	No		Pit Latrine/	Finished ro	Rambo/wo	0	Yes
	Piped into (	No		Pit Latrine/	Finished ro	Rambo/wo	1	Yes
	Protected I	No		Pit Latrine/	Finished ro	Rambo/wo	1	No
	Piped outsi	No		No facility/	Finished ro	Rambo/wo	0	Yes
	Piped outsi	No		Pit Latrine/	Finished ro	Rambo/wo	1	No
	Piped outsi	No		Pit Latrine/	Finished ro	Rambo/wo	1	No
2	Piped outsi	No		Pit Latrine/	Finished ro	Rambo/wo	1	Yes
	Piped outsi	No		No facility/	Finished ro	Rambo/wo	1	Yes
	Protected S	No		Pit Latrine/	Finished ro	Rambo/wo	1	No
	Covered W	No		Pit Latrine/	Finished ro	Rambo/wo	1	Yes
	Protected S	No		Pit Latrine/	Finished ro	Rambo/wo	1	Yes
	Protected I	No		Pit Latrine/	Finished ro	Rambo/wo	1	No
	Protected S	No		No facility/	Finished ro	Rambo/wo	1	Yes
1	Open Sprin	No		Pit Latrine/	Finished ro	Rambo/wo	0	No
	Piped into (	No		Pit Latrine/	Finished ro	Rambo/wo	1	No
	Piped outsi	No		Pit Latrine/	Finished ro	Rambo/wo	0	Yes
	Protected S	No		Pit Latrine/	Finished ro	Rambo/wo	0	Yes
	Open Sprin	No		Pit Latrine/	Finished ro	Rambo/wo	0	Yes
	Open Sprin	No		Pit Latrine/	Finished ro	Rambo/wo	0	Yes

For peer review only

	Piped outsi	No	Pit Latrine/	Finished ro	Rambo/wo	1	No
	Protected S	No	No facility/	Finished ro	Rambo/wo	1	Yes
	Protected S	No	Pit Latrine/	Finished ro	Rambo/wo	1	Yes
	Piped outsi	No	No facility/	Finished ro	Rambo/wo	2	No
	Open Well	No	No facility/	Finished ro	Rambo/wo	1	No
	Piped outsi	No	No facility/	Finished ro	Rambo/wo	1	Yes
	Open Sprin	No	No facility/	Finished ro	Rambo/wo	1	Yes
	Piped outsi	No	No facility/	Finished ro	Rambo/wo	0	No
	Covered W	No	No facility/	Finished ro	Rambo/wo	0	No
	Protected S	No	Pit Latrine/	Finished ro	Rambo/wo	0	Yes
1	Piped outsi	No	No facility/	Finished ro	Rambo/wo	0	Yes
	Open Sprin	No	Pit Latrine/	Finished ro	Rambo/wo	1	Yes
	Piped outsi	No	No facility/	Finished ro	Rambo/wo	0	No
	Protected S	No	Pit Latrine/	Finished ro	Rambo/wo	1	Yes
	Covered W	No	Pit Latrine/	Finished ro	Rambo/wo	1	Yes
1	Piped outsi	No	No facility/	Finished ro	Rambo/wo	2	Yes
	Protected S	No	Pit Latrine/	Finished ro	Rambo/wo	1	Yes
	Piped outsi	No	No facility/	Finished ro	Rambo/wo	1	Yes
	Piped outsi	No	No facility/	Finished ro	Rambo/wo	0	Yes
	Piped outsi	No	Pit Latrine/	Finished ro	Rambo/wo	2	Yes
	Protected S	No	No facility/	Finished ro	Rambo/wo	1	Yes
	Piped outsi	No	No facility/	Finished ro	Rambo/wo	1	No
	Open Well	No	Pit Latrine/	Finished ro	Rambo/wo	0	No
	Open Well	No	No facility/	Finished ro	Rambo/wo	0	Yes
	Open Well	No	No facility/	Other	Other	0	No
1	Piped outsi	No	Pit Latrine/	Finished ro	Rambo/wo	0	Yes
	Piped into	No	Pit Latrine/	Finished ro	Rambo/wo	1	Yes
	Protected S	No	Pit Latrine/	Finished ro	Rambo/wo	0	Yes
	Piped outsi	No	Pit Latrine/	Finished ro	Rambo/wo	0	No
	Piped outsi	No	Pit Latrine/	Finished ro	Rambo/wo	2	No
	Piped into	No	Pit Latrine/	Finished ro	Rambo/wo	0	No
1	Piped outsi	No	Pit Latrine/	Finished ro	Rambo/wo	0	Yes
	Protected h	No	Pit Latrine/	Finished ro	Rambo/wo	0	Yes
	Open Sprin	No	No facility/	Finished ro	Rambo/wo	1	Yes
1	Piped outsi	No	No facility/	Finished ro	Rambo/wo	0	Yes
	Piped outsi	No	Pit Latrine/	Finished ro	Stone with	1	Yes
	Piped outsi	No	Pit Latrine/	Finished ro	Rambo/wo	1	Yes
	Protected S	No	Pit Latrine/	Finished ro	Rambo/wo	0	No
2	Protected S	No	Pit Latrine/	Finished ro	Rambo/wo	0	Yes
	Piped outsi	No	Pit Latrine/	Finished ro	Rambo/wo	0	Yes
1	Piped outsi	No	Pit Latrine/	Finished ro	Rambo/wo	0	Yes
	Protected h	No	Pit Latrine/	Finished ro	Rambo/wo	1	Yes
	Open Well	No	Pit Latrine/	Finished ro	Rambo/wo	1	Yes
1	Piped outsi	No	Pit Latrine/	Finished ro	Rambo/wo	2	Yes
2	Piped outsi	No	Pit Latrine/	Finished ro	Rambo/wo	1	Yes
	Piped outsi	No	No facility/	Finished ro	Rambo/wo	1	Yes
	Protected h	No	Pit Latrine/	Finished ro	Rambo/wo	1	No
2	Piped outsi	No	No facility/	Finished ro	Rambo/wo	0	Yes
2	Piped outsi	No	No facility/	Finished ro	Rambo/wo	0	Yes
2	Open Sprin	No	No facility/	Finished ro	Rambo/wo	1	Yes
	Piped outsi	No	No facility/	Finished ro	Rambo/wo	1	Yes
2	Piped outsi	No	Pit Latrine/	Finished ro	Rambo/wo	0	Yes

For peer review only

	Open Well	No	No facility/ Finished ro	Rambo/wo	0	Yes
	Protected I	No	Pit Latrine/ Finished ro	Rambo/wo	1	No
	Open Well	No	Pit Latrine/ Finished ro	Rambo/wo	0	Yes
	Open Well	No	No facility/ Finished ro	Rambo/wo	0	Yes
	Protected S	No	Pit Latrine/ Finished ro	Rambo/wo	0	Yes
	Open Well	No	No facility/ Finished ro	Rambo/wo	0	Yes
	Piped into	No	Pit Latrine/ Finished ro	Rambo/wo	1	No
	Piped outsi	No	No facility/ Finished ro	Rambo/wo	1	Yes
	Protected S	No	Pit Latrine/ Finished ro	Rambo/wo	1	Yes
1	Piped outsi	No	Pit Latrine/ Finished ro	Rambo/wo	0	Yes
	Piped into	No	Pit Latrine/ Finished ro	Rambo/wo	1	Yes
	Protected I	No	Pit Latrine/ Finished ro	Rambo/wo	1	Yes
	Protected S	No	No facility/ Finished ro	Rambo/wo	0	No
6	Piped outsi	No	No facility/ Finished ro	Rambo/wo	0	Yes
	Open Sprin	No	Pit Latrine/ Finished ro	Rambo/wo	2	Yes
1	Piped outsi	No	Pit Latrine/ Finished ro	Rambo/wo	0	Yes
	Piped into	No	Pit Latrine/ Finished ro	Rambo/wo	0	No
	Piped outsi	No	Pit Latrine/ Finished ro	Rambo/wo	0	Yes
	Protected S	No	Pit Latrine/ Finished ro	Rambo/wo	1	Yes
	Open Well	No	No facility/ Finished ro	Rambo/wo	0	No
	Piped outsi	No	No facility/ Finished ro	Rambo/wo	0	No
	Protected I	No	No facility/ Finished ro	Rambo/wo	0	Yes
2	Open Well	No	No facility/ Finished ro	Rambo/wo	0	Yes
1	Protected I	No	Pit Latrine/ Finished ro	Rambo/wo	1	Yes
	Open Well	No	Pit Latrine/ Finished ro	Rambo/wo	0	Yes
	Piped outsi	No	Pit Latrine/ Finished ro	Rambo/wo	0	Yes
	Protected I	No	Pit Latrine/ Finished ro	Rambo/wo	1	No
	Open Sprin	No	No facility/ Finished ro	Rambo/wo	0	Yes
	Piped outsi	No	Pit Latrine/ Finished ro	Rambo/wo	0	No
	Piped outsi	No	Pit Latrine/ Finished ro	Rambo/wo	1	Yes
	Protected I	No	Pit Latrine/ Finished ro	Rambo/wo	1	Yes
	Piped outsi	No	Pit Latrine/ Finished ro	Rambo/wo	0	Yes
	Piped outsi	No	No facility/ Finished ro	Rambo/wo	2	Yes
2	Piped outsi	No	No facility/ Finished ro	Rambo/wo	1	Yes
	Protected I	No	Pit Latrine/ Finished ro	Rambo/wo	0	No
	Protected S	No	Pit Latrine/ Finished ro	Rambo/wo	0	Yes
	Piped outsi	No	Pit Latrine/ Finished ro	Rambo/wo	1	No
	Protected S	No	Pit Latrine/ Finished ro	Rambo/wo	1	Yes
	Protected S	No	No facility/ Finished ro	Rambo/wo	2	Yes
	Piped outsi	No	Pit Latrine/ Finished ro	Rambo/wo	1	Yes
	Protected I	No	Pit Latrine/ Finished ro	Rambo/wo	0	No
4	Protected S	No	Pit Latrine/ Finished ro	Rambo/wo	1	Yes
	Piped into	No	Pit Latrine/ Finished ro	Rambo/wo	1	Yes
	Protected I	No	Pit Latrine/ Finished ro	Rambo/wo	1	No
	Piped outsi	No	Pit Latrine/ Finished ro	Rambo/wo	1	No
	Piped outsi	No	Pit Latrine/ Finished ro	Rambo/wo	0	Yes
	Piped outsi	No	No facility/ Finished ro	Rambo/wo	1	No
	River	No	Pit Latrine/ Finished ro	Rambo/wo	1	Yes
	Protected I	No	No facility/ Finished ro	Rambo/wo	1	No
	Protected S	No	No facility/ Finished ro	Rambo/wo	1	Yes
	Protected I	No	No facility/ Finished ro	Rambo/wo	0	Yes
	Protected S	No	No facility/ Finished ro	Rambo/wo	0	Yes

For peer review only

	Piped outsi	No		No facility/ Finished ro	Rambo/wo	0	Yes
	Protected I	No		No facility/ Finished ro	Rambo/wo	1	Yes
	Piped outsi	No		Pit Latrine/ Finished ro	Rambo/wo	0	No
	Protected I	No		Pit Latrine/ Finished ro	Rambo/wo	1	Yes
	Protected S	No		Pit Latrine/ Finished ro	Rambo/wo	1	No
	Protected S	No		No facility/ Finished ro	Rambo/wo	1	No
	Protected S	No		No facility/ Finished ro	Rambo/wo	1	No
2	Protected S	No		No facility/ Finished ro	Rambo/wo	0	Yes
	Piped outsi	No		No facility/ Finished ro	Rambo/wo	1	Yes
	Protected S	No		Pit Latrine/ Finished ro	Rambo/wo	1	Yes
	Protected S	No		No facility/ Finished ro	Rambo/wo	1	Yes
	Protected I	No		Pit Latrine/ Finished ro	Rambo/wo	1	Yes
	Piped outsi	No		Pit Latrine/ Finished ro	Rambo/wo	0	Yes
	Piped into (	No		Pit Latrine/ Finished ro	Rambo/wo	1	Yes
	Protected I	No		No facility/ Finished ro	Rambo/wo	1	No
	Piped into (	No		Pit Latrine/ Finished ro	Rambo/wo	0	No
	Open Well	No		Pit Latrine/ Finished ro	Rambo/wo	1	No
1	Open Sprin	No		Pit Latrine/ Finished ro	Rambo/wo	1	Yes
	Piped outsi	No		Pit Latrine/ Finished ro	Rambo/wo	1	Yes
	Protected I	No		No facility/ Finished ro	Rambo/wo	1	No
	Piped outsi	No		Pit Latrine/ Finished ro	Rambo/wo	1	No
	Protected S	No		Pit Latrine/ Finished ro	Rambo/wo	2	Yes
	Covered W	Yes	Add bleach	Pit Latrine/ Finished ro	Rambo/wo	2	Yes
	Piped outsi	No		No facility/ Finished ro	Rambo/wo	0	Yes
2	Piped outsi	No		No facility/ Finished ro	Rambo/wo	0	Yes
	Piped outsi	No		Pit Latrine/ Finished ro	Rambo/wo	0	Yes
	Protected S	No		No facility/ Finished ro	Rambo/wo	0	Yes
2	Piped outsi	No		Pit Latrine/ Finished ro	Rambo/wo	0	Yes
	Protected S	No		Pit Latrine/ Finished ro	Rambo/wo	0	Yes
	Protected S	No		No facility/ Finished ro	Rambo/wo	0	Yes
	Piped outsi	No		No facility/ Finished ro	Rambo/wo	1	Yes
	Piped outsi	No		No facility/ Finished ro	Rambo/wo	0	No
	Protected S	No		Pit Latrine/ Finished ro	Rambo/wo	1	Yes
	Piped outsi	No		No facility/ Finished ro	Rambo/wo	1	No
	Protected I	No		Pit Latrine/ Finished ro	Rambo/wo	1	Yes
	Protected S	No		Pit Latrine/ Finished ro	Rambo/wo	1	Yes
1	Piped outsi	No		Pit Latrine/ Finished ro	Rambo/wo	1	Yes
	Piped outsi	No		Pit Latrine/ Finished ro	Rambo/wo	0	No
1	Protected S	No		No facility/ Finished ro	Rambo/wo	1	Yes
	Piped outsi	No		Pit Latrine/ Finished ro	Rambo/wo	2	Yes
	Piped outsi	No		Pit Latrine/ Finished ro	Rambo/wo	1	No
	Piped outsi	No		Pit Latrine/ Finished ro	Rambo/wo	0	No
	Piped outsi	No		Pit Latrine/ Finished ro	Rambo/wo	1	No
	Piped outsi	No		Pit Latrine/ Finished ro	Rambo/wo	0	Yes
1	Protected S	No		Pit Latrine/ Finished ro	Rambo/wo	1	No
	Protected S	No		No facility/ Finished ro	Rambo/wo	0	Yes
	Piped outsi	No		No facility/ Finished ro	Rambo/wo	1	Yes
	Piped outsi	No		Pit Latrine/ Finished ro	Rambo/wo	0	Yes
	Pond/Lake,	No		Pit Latrine/ Finished ro	Other	1	No
	Covered W	No		No facility/ Thatch/lea	Other	0	Yes
	Piped outsi	No		No facility/ Finished ro	Rambo/wo	1	Yes
	Piped outsi	No		No facility/ Finished ro	Rambo/wo	0	No

For peer review only

	Piped outsi	No		Pit Latrine/Thatch/lea	Rambo/wo	0	Yes
	Piped outsi	No		Pit Latrine/Finished ro	Rambo/wo	1	No
	Pond/Lake,	No		Pit Latrine/Finished ro	Rambo/wo	1	No
1	Piped outsi	No		No facility/Finished ro	Rambo/wo	1	Yes
	Piped outsi	No		Pit Latrine/Thatch/lea	Rambo/wo	1	Yes
	Pond/Lake,	No		No facility/Finished ro	Rambo/wo	1	Yes
	River	No		No facility/Thatch/lea	Other	0	Yes
	Piped outsi	No		No facility/Finished ro	Rambo/wo	2	Yes
	Piped outsi	No		No facility/Finished ro	Rambo/wo	1	Yes
	Piped outsi	No		Pit Latrine/Thatch/lea	Rambo/wo	0	Yes
1	Piped outsi	No		Pit Latrine/Finished ro	Rambo/wo	1	Yes
	Protected S	No		Pit Latrine/Finished ro	Rambo/wo	1	Yes
	Protected S	No		No facility/Thatch/lea	Cement	1	Yes
	Piped into (	Yes	Boil	Pit Latrine/Finished ro	Rambo/wo	2	Yes
	Covered W	No		Pit Latrine/Finished ro	Rambo/wo	1	Yes
	Pond/Lake,	No		No facility/Finished ro	Rambo/wo	1	No
	Pond/Lake,	No		No facility/Finished ro	Rambo/wo	1	No
	Protected S	No		No facility/Finished ro	Rambo/wo	2	Yes
	Pond/Lake,	No		No facility/Finished ro	Rambo/wo	1	Yes
	Piped outsi	No		No facility/Finished ro	Rambo/wo	1	Yes
	Protected I	No		No facility/Finished ro	Rambo/wo	1	Yes
	River	No		No facility/Finished ro	Rambo/wo	1	Yes
	Protected S	Yes	Boil	Pit Latrine/Finished ro	Rambo/wo	2	Yes
	Protected I	No		Pit Latrine/Finished ro	Rambo/wo	1	Yes
	Piped outsi	No		Pit Latrine/Thatch/lea	Rambo/wo	1	Yes
	Piped outsi	No		Pit Latrine/Finished ro	Rambo/wo	1	No
	Piped outsi	No		No facility/Finished ro	Rambo/wo	2	Yes
	Piped outsi	No		Pit Latrine/Finished ro	Rambo/wo	0	Yes
	Protected S	No		No facility/Thatch/lea	Rambo/wo	0	Yes
	Piped outsi	No		No facility/Finished ro	Rambo/wo	1	Yes
	Piped outsi	No		Pit Latrine/Finished ro	Rambo/wo	0	Yes
	Pond/Lake,	No		Pit Latrine/Finished ro	Rambo/wo	1	No
3	Piped outsi	No		Pit Latrine/Finished ro	Rambo/wo	1	Yes
	Covered W	Yes	Boil	Pit Latrine/Thatch/lea	Rambo/wo	1	Yes
	Open Sprin	No		Pit Latrine/Finished ro	Rambo/wo	1	Yes
	Protected S	No		No facility/Finished ro	Rambo/wo	1	Yes
	Piped outsi	No		Pit Latrine/Finished ro	Rambo/wo	2	Yes
	Open Well	No		No facility/Finished ro	Rambo/wo	1	Yes
	Piped outsi	No		Pit Latrine/Finished ro	Rambo/wo	0	Yes
	Protected I	No		Pit Latrine/Finished ro	Rambo/wo	1	Yes
	Covered W	No		Pit Latrine/Finished ro	Rambo/wo	1	No
	Covered W	Yes	Boil	Pit Latrine/Thatch/lea	Rambo/wo	2	Yes
	Pond/Lake,	No		No facility/Finished ro	Rambo/wo	1	Yes
	Covered W	No		Pit Latrine/Finished ro	Other	1	Yes
	Protected S	No		No facility/Finished ro	Rambo/wo	1	Yes
	Piped outsi	No		Pit Latrine/Finished ro	Other	1	Yes
	Open Sprin	No		Pit Latrine/Finished ro	Rambo/wo	1	No
	Protected S	No		Pit Latrine/Finished ro	Rambo/wo	2	No
	Pond/Lake,	No		Pit Latrine/Finished ro	Rambo/wo	1	Yes
	Piped outsi	No		Pit Latrine/Finished ro	Rambo/wo	1	No
	River	No		Pit Latrine/Finished ro	Other	1	Yes
	Piped outsi	No		Pit Latrine/Finished ro	Cement	1	Yes

For peer review only

	Covered W	Yes	Boil	Pit Latrine/Finished ro	Rambo/wo	2	Yes
	Covered W	No		No facility/Finished ro	Rambo/wo	0	No
	Piped outsi	No		No facility/Finished ro	Rambo/wo	1	Yes
	Covered W	No		Pit Latrine/Finished ro	Rambo/wo	0	Yes
	Protected S	No		Pit Latrine/Finished ro	Rambo/wo	1	No
	Piped outsi	No		Pit Latrine/Finished ro	Rambo/wo	0	Yes
	Protected S	No		No facility/Thatch/lea	Stone with	0	Yes
	Piped outsi	No		Pit Latrine/Finished ro	Cement	2	Yes
	Piped outsi	No		No facility/Finished ro	Stone with	0	Yes
	Piped outsi	No		No facility/Finished ro	Rambo/wo	1	Yes
1	Piped outsi	No		Pit Latrine/Finished ro	Rambo/wo	1	Yes
	River	No		Pit Latrine/Finished ro	Rambo/wo	1	Yes
	Piped outsi	No		No facility/Finished ro	Rambo/wo	1	Yes
	Piped outsi	No		Pit Latrine/Finished ro	Rambo/wo	0	Yes
	Protected H	No		Pit Latrine/Finished ro	Rambo/wo	1	Yes
	Open Well	No		No facility/Finished ro	Rambo/wo	0	Yes
	Piped outsi	No		Pit Latrine/Finished ro	Rambo/wo	1	Yes
	Open Sprin	No		Pit Latrine/Finished ro	Rambo/wo	0	Yes
	Piped outsi	No		Pit Latrine/Finished ro	Rambo/wo	1	No
	Piped outsi	No		Pit Latrine/Finished ro	Rambo/wo	1	Yes
	Covered W	Yes	Boil	No facility/Finished ro	Rambo/wo	2	Yes
	Pond/Lake,	No		Pit Latrine/Finished ro	Rambo/wo	2	Yes
	Open Well	No		No facility/Finished ro	Rambo/wo	1	Yes
	Open Sprin	No		No facility/Finished ro	Rambo/wo	1	Yes
	Protected S	No		No facility/Finished ro	Rambo/wo	1	Yes
	Piped outsi	No		No facility/Thatch/lea	Rambo/wo	0	Yes
	Covered W	Yes	Boil	Pit Latrine/Finished ro	Rambo/wo	2	Yes
	Protected H	No		Pit Latrine/Finished ro	Rambo/wo	1	Yes
	River	No		Pit Latrine/Finished ro	Other	1	Yes
	Piped outsi	No		Pit Latrine/Finished ro	Rambo/wo	0	Yes
	Protected S	No		No facility/Finished ro	Rambo/wo	1	Yes
	Open Sprin	No		Pit Latrine/Finished ro	Rambo/wo	1	Yes
	Piped outsi	No		Pit Latrine/Finished ro	Cement	1	No
	Piped outsi	No		No facility/Finished ro	Rambo/wo	1	Yes
	Protected S	Yes	Boil	No facility/Finished ro	Rambo/wo	2	Yes
	Piped outsi	No		Pit Latrine/Finished ro	Rambo/wo	1	Yes
	Piped outsi	Yes	Boil	Pit Latrine/Finished ro	Rambo/wo	1	Yes
1	Pond/Lake,	No		No facility/Finished ro	Rambo/wo	1	Yes
	Open Well	No		Pit Latrine/Finished ro	Rambo/wo	0	Yes
	Protected S	No		No facility/Finished ro	Rambo/wo	1	Yes
	Covered W	No		Pit Latrine/Thatch/lea	Cement	0	No
	Protected S	No		No facility/Finished ro	Rambo/wo	1	Yes
	Protected S	No		No facility/Finished ro	Rambo/wo	1	Yes
	Open Well	No		No facility/Finished ro	Rambo/wo	0	Yes
	Open Sprin	No		Pit Latrine/Finished ro	Rambo/wo	1	Yes
	Protected H	No		No facility/Finished ro	Rambo/wo	1	Yes
	Piped outsi	No		Pit Latrine/Finished ro	Rambo/wo	1	No
	Piped outsi	No		No facility/Finished ro	Rambo/wo	1	Yes
	Piped outsi	No		Pit Latrine/Finished ro	Rambo/wo	1	Yes
2	Protected S	No		Pit Latrine/Finished ro	Rambo/wo	1	Yes
	Pond/Lake,	No		Pit Latrine/Finished ro	Rambo/wo	1	Yes
	Piped outsi	No		Pit Latrine/Finished ro	Rambo/wo	1	Yes

For peer review only

	Piped outsi	No		Pit Latrine/	Finished ro	Rambo/wo	1	Yes
	Open Sprin	No		No facility/	Finished ro	Rambo/wo	0	Yes
	Piped outsi	No		Pit Latrine/	Finished ro	Cement	1	Yes
	Piped outsi	No		Pit Latrine/	Finished ro	Rambo/wo	0	Yes
	River	No		Pit Latrine/	Finished ro	Rambo/wo	1	Yes
	Piped outsi	No		Pit Latrine/	Finished ro	Rambo/wo	1	No
	Piped outsi	Yes	Boil	Pit Latrine/	Finished ro	Rambo/wo	2	Yes
	Protected	No		Pit Latrine/	Finished ro	Rambo/wo	1	Yes
	Protected	No		Pit Latrine/	Finished ro	Rambo/wo	1	Yes
	Piped outsi	No		Pit Latrine/	Finished ro	Rambo/wo	0	No
	Protected	No		Pit Latrine/	Finished ro	Rambo/wo	2	Yes
1	Piped outsi	No		No facility/	Finished ro	Rambo/wo	1	Yes
	Pond/Lake,	No		No facility/	Finished ro	Rambo/wo	1	No
	Open Well	No		No facility/	Finished ro	Rambo/wo	0	No
	Pond/Lake,	No		Pit Latrine/	Finished ro	Rambo/wo	1	Yes
	Piped outsi	No		No facility/	Finished ro	Rambo/wo	1	Yes
	Pond/Lake,	No		No facility/	Finished ro	Rambo/wo	1	No
	Covered W	Yes	Boil	Pit Latrine/	Thatch/lea	Rambo/wo	1	Yes
	Piped outsi	No		Pit Latrine/	Finished ro	Rambo/wo	0	Yes
	Covered W	Yes	Boil	No facility/	Thatch/lea	Rambo/wo	1	Yes
1	Protected	No		No facility/	Finished ro	Rambo/wo	1	No
	Open Well	No		Pit Latrine/	Finished ro	Rambo/wo	1	No
	Protected	No		No facility/	Finished ro	Rambo/wo	1	Yes
	Covered W	Yes	Boil	No facility/	Finished ro	Rambo/wo	1	Yes
	Piped into	No		Pit Latrine/	Finished ro	Rambo/wo	1	No
	Piped outsi	No		Pit Latrine/	Finished ro	Rambo/wo	0	No
	Piped outsi	No		No facility/	Finished ro	Rambo/wo	1	No
	Piped outsi	No		Ventilated	Finished ro	Rambo/wo	1	Yes
	Piped outsi	No		No facility/	Roofing shi	Rambo/wo	1	Yes
	Open Sprin	No		Pit Latrine/	Finished ro	Rambo/wo	0	Yes
	Piped outsi	No		Pit Latrine/	Finished ro	Rambo/wo	1	No
	Piped outsi	No		Pit Latrine/	Thatch/lea	Rambo/wo	0	Yes
	Piped outsi	No		Pit Latrine/	Thatch/lea	Rambo/wo	1	Yes
	Piped outsi	No		No facility/	Finished ro	Rambo/wo	1	Yes
	Piped outsi	No		Pit Latrine/	Finished ro	Other	1	Yes
	Piped outsi	No		Pit Latrine/	Finished ro	Rambo/wo	1	Yes
	Piped outsi	No		No facility/	Finished ro	Rambo/wo	1	Yes
	Covered W	Yes	Boil	Pit Latrine/	Finished ro	Rambo/wo	2	Yes
	Piped outsi	No		No facility/	Finished ro	Rambo/wo	1	Yes
	Pond/Lake,	No		No facility/	Finished ro	Rambo/wo	1	Yes
	Piped outsi	No		No facility/	Thatch/lea	Rambo/wo	1	Yes
	Rainwater	No		Pit Latrine/	Finished ro	Rambo/wo	1	Yes
	Piped outsi	No		No facility/	Finished ro	Rambo/wo	1	Yes
	Piped outsi	No		No facility/	Finished ro	Rambo/wo	1	Yes
	Open Well	No		Pit Latrine/	Finished ro	Rambo/wo	1	Yes
	Piped outsi	Yes	Boil	Pit Latrine/	Finished ro	Rambo/wo	2	Yes
	River	No		Pit Latrine/	Finished ro	Rambo/wo	0	Yes
	Pond/Lake,	No		No facility/	Finished ro	Rambo/wo	1	Yes
	Covered W	No		Pit Latrine/	Finished ro	Rambo/wo	2	No
	Covered W	No		Pit Latrine/	Finished ro	Rambo/wo	1	Yes
	Piped outsi	No		Pit Latrine/	Finished ro	Rambo/wo	0	No
	Piped outsi	No		No facility/	Finished ro	Rambo/wo	1	No

For peer review only

	Open Sprin	No		Pit Latrine/	Finished ro	Rambo/wo	1	No
	Protected S	No		No facility/	Finished ro	Rambo/wo	1	Yes
	Covered W	No		No facility/	Finished ro	Rambo/wo	2	Yes
	Covered W	Yes	Boil	No facility/	Finished ro	Rambo/wo	2	Yes
	Piped outsi	No		Pit Latrine/	Finished ro	Rambo/wo	1	Yes
	Protected S	No		No facility/	Finished ro	Rambo/wo	0	Yes
	Open Sprin	Yes	Boil	No facility/	Finished ro	Rambo/wo	1	Yes
	Piped outsi	No		Pit Latrine/	Finished ro	Rambo/wo	1	Yes
	Pond/Lake,	No		No facility/	Finished ro	Rambo/wo	1	No
1	Open Sprin	No		Pit Latrine/	Finished ro	Rambo/wo	0	Yes
	Open Well	No		No facility/	Finished ro	Rambo/wo	0	No
	Piped outsi	No		Pit Latrine/	Finished ro	Rambo/wo	1	Yes
	Piped outsi	Yes	Boil	Pit Latrine/	Finished ro	Rambo/wo	2	Yes
	Protected S	No		No facility/	Finished ro	Rambo/wo	1	No
	Piped outsi	No		No facility/	Finished ro	Rambo/wo	1	Yes
	Piped outsi	No		Pit Latrine/	Finished ro	Rambo/wo	0	Yes
	Piped outsi	No		No facility/	Thatch/lea	Rambo/wo	0	Yes
	Piped outsi	No		No facility/	Finished ro	Rambo/wo	1	Yes
	Open Sprin	No		No facility/	Finished ro	Rambo/wo	1	Yes
	River	No		Pit Latrine/	Finished ro	Rambo/wo	1	Yes
	Piped outsi	No		Pit Latrine/	Finished ro	Rambo/wo	0	No
	Piped outsi	No		No facility/	Thatch/lea	Rambo/wo	0	No
2	Protected S	No		Pit Latrine/	Finished ro	Cement	0	Yes
	Protected S	No		No facility/	Thatch/lea	Rambo/wo	1	No
	Piped outsi	No		No facility/	Finished ro	Rambo/wo	2	Yes
	Open Sprin	No		Pit Latrine/	Finished ro	Rambo/wo	0	Yes
1	Covered W	No		No facility/	Finished ro	Rambo/wo	1	Yes
	Open Well	No		Pit Latrine/	Finished ro	Rambo/wo	1	Yes
	River	No		Pit Latrine/	Finished ro	Rambo/wo	0	Yes
	Open Sprin	No		No facility/	Finished ro	Rambo/wo	1	Yes
	Protected S	No		Pit Latrine/	Finished ro	Rambo/wo	0	Yes
	Open Well	No		Pit Latrine/	Finished ro	Rambo/wo	0	Yes
	Piped outsi	No		No facility/	Thatch/lea	Other	0	Yes
	Piped outsi	No		Pit Latrine/	Finished ro	Rambo/wo	1	No
	Piped outsi	No		No facility/	Finished ro	Rambo/wo	1	Yes
	Covered W	Yes	Boil	No facility/	Finished ro	Rambo/wo	2	Yes
	Open Sprin	No		Pit Latrine/	Finished ro	Rambo/wo	0	Yes
	Covered W	Yes	Boil	No facility/	Thatch/lea	Rambo/wo	1	Yes
	Piped outsi	No		No facility/	Finished ro	Rambo/wo	1	Yes
	Piped outsi	No		Pit Latrine/	Finished ro	Rambo/wo	1	Yes
	Piped outsi	No		No facility/	Finished ro	Rambo/wo	1	Yes
	Piped outsi	No		No facility/	Finished ro	Rambo/wo	1	Yes
	Piped outsi	No		No facility/	Finished ro	Rambo/wo	1	Yes
	Piped outsi	No		No facility/	Finished ro	Rambo/wo	0	Yes
	Piped outsi	No		Pit Latrine/	Finished ro	Rambo/wo	1	Yes
	Pond/Lake,	No		No facility/	Finished ro	Rambo/wo	1	No
1	Open Sprin	No		Pit Latrine/	Finished ro	Rambo/wo	0	Yes
	Piped outsi	No		No facility/	Finished ro	Rambo/wo	1	No
	Protected I	No		Pit Latrine/	Finished ro	Rambo/wo	1	Yes
	Open Sprin	No		Pit Latrine/	Finished ro	Rambo/wo	1	Yes
	Protected I	No		No facility/	Finished ro	Rambo/wo	1	Yes
	Open Sprin	No		Pit Latrine/	Finished ro	Rambo/wo	1	Yes

	Protected S	No		Pit Latrine/Finished ro	Rambo/wo	1	Yes
	Piped outsi	No		Pit Latrine/Finished ro	Rambo/wo	1	Yes
	Protected S	Yes	Boil	Pit Latrine/Finished ro	Rambo/wo	1	Yes
	Piped outsi	No		Pit Latrine/Finished ro	Rambo/wo	2	Yes
	Open Well	No		Pit Latrine/Finished ro	Rambo/wo	0	Yes
	Protected S	No		Pit Latrine/Finished ro	Rambo/wo	1	Yes
	Protected S	No		Pit Latrine/Finished ro	Rambo/wo	1	Yes
	Pond/Lake,	No		Pit Latrine/Finished ro	Rambo/wo	1	Yes
	Open Sprin	No		No facility/Finished ro	Rambo/wo	0	Yes
1	Protected S	No		No facility/Finished ro	Rambo/wo	1	Yes
	Piped outsi	No		No facility/Finished ro	Rambo/wo	1	Yes
	Covered W	No		Pit Latrine/Thatch/lea	Cement	0	No
	Open Well	No		No facility/Finished ro	Rambo/wo	1	Yes
	Pond/Lake,	No		No facility/Finished ro	Rambo/wo	1	No
	Piped outsi	No		Pit Latrine/Finished ro	Rambo/wo	1	No
	Piped outsi	No		No facility/Finished ro	Rambo/wo	1	Yes
	Piped outsi	No		Pit Latrine/Finished ro	Rambo/wo	1	No
	Open Well	No		Pit Latrine/Finished ro	Rambo/wo	0	Yes
	Covered W	Yes	Boil	No facility/Finished ro	Rambo/wo	2	Yes
	Piped outsi	No		Pit Latrine/Finished ro	Rambo/wo	0	No
	River	No		No facility/Finished ro	Rambo/wo	1	No
	Piped into (	No		No facility/Finished ro	Rambo/wo	1	Yes
	Open Sprin	No		No facility/Finished ro	Rambo/wo	1	No
	Open Sprin	No		Pit Latrine/Finished ro	Rambo/wo	1	Yes
	Piped outsi	No		Pit Latrine/Finished ro	Rambo/wo	1	Yes
	Piped outsi	Yes	Boil	No facility/Finished ro	Rambo/wo	1	Yes
	River	No		Pit Latrine/Finished ro	Rambo/wo	0	No
	Open Sprin	No		Pit Latrine/Finished ro	Rambo/wo	1	Yes
	Piped outsi	No		Pit Latrine/Finished ro	Rambo/wo	1	Yes
	Open Well	No		Pit Latrine/Finished ro	Rambo/wo	1	Yes
	Piped outsi	No		No facility/Finished ro	Rambo/wo	1	Yes
	Open Well	No		No facility/Finished ro	Rambo/wo	1	Yes
	Covered W	No		No facility/Finished ro	Rambo/wo	1	Yes
	Covered W	Yes	Boil	No facility/Finished ro	Rambo/wo	2	Yes
	Protected S	No		Pit Latrine/Thatch/lea	Rambo/wo	0	No
	Covered W	No		Pit Latrine/Thatch/lea	Rambo/wo	1	Yes
	Piped outsi	No		No facility/Finished ro	Rambo/wo	1	Yes
	Protected S	No		Pit Latrine/Finished ro	Rambo/wo	1	Yes
	Protected S	No		No facility/Finished ro	Rambo/wo	1	No
	Open Well	No		No facility/Finished ro	Rambo/wo	0	Yes
	Protected S	No		No facility/Finished ro	Rambo/wo	1	Yes
	Covered W	Yes	Boil	No facility/Finished ro	Rambo/wo	1	Yes
	Open Well	No		Pit Latrine/Finished ro	Rambo/wo	1	Yes
	Pond/Lake,	No		Pit Latrine/Finished ro	Rambo/wo	0	Yes
	Protected S	Yes	Boil	No facility/Thatch/lea	Rambo/wo	1	Yes
	Piped outsi	No		Pit Latrine/Finished ro	Rambo/wo	1	Yes
	Protected S	No		No facility/Finished ro	Rambo/wo	1	No
	Open Well	No		Pit Latrine/Finished ro	Rambo/wo	0	Yes
	Piped outsi	No		Pit Latrine/Finished ro	Rambo/wo	1	Yes
	Piped outsi	No		No facility/Finished ro	Rambo/wo	1	Yes
	Piped outsi	No		No facility/Finished ro	Rambo/wo	1	Yes
	Covered W	Yes	Boil	Pit Latrine/Finished ro	Rambo/wo	2	Yes

For peer review only

	Protected I	No		No facility/ Finished ro	Rambo/wo	1	Yes
	Piped outsi	No		Pit Latrine/ Finished ro	Rambo/wo	1	Yes
	Piped outsi	No		No facility/ Finished ro	Rambo/wo	1	No
	Covered W	Yes	Boil	No facility/ Finished ro	Rambo/wo	1	Yes
1	Piped outsi	No		No facility/ Finished ro	Rambo/wo	1	Yes
	Piped outsi	No		Pit Latrine/ Finished ro	Other	1	No
	Covered W	No		Pit Latrine/ Finished ro	Rambo/wo	1	Yes
	Covered W	Yes	Boil	Pit Latrine/ Thatch/lea	Rambo/wo	2	Yes
	Open Well	No		Pit Latrine/ Finished ro	Rambo/wo	1	Yes
	Protected S	Yes	Boil	No facility/ Thatch/lea	Rambo/wo	1	Yes
	Piped outsi	No		Pit Latrine/ Finished ro	Rambo/wo	1	Yes
	Piped outsi	No		Pit Latrine/ Finished ro	Rambo/wo	2	Yes
	Open Sprin	No		Pit Latrine/ Finished ro	Rambo/wo	1	Yes
	Covered W	Yes	Boil	No facility/ Finished ro	Rambo/wo	2	Yes
	Open Well	No		No facility/ Finished ro	Rambo/wo	0	Yes
1	Protected S	No		No facility/ Thatch/lea	Cement	0	Yes
1	Protected S	No		Pit Latrine/ Finished ro	Rambo/wo	1	No
	Covered W	No		Pit Latrine/ Thatch/lea	Rambo/wo	2	Yes
	Open Sprin	No		No facility/ Finished ro	Rambo/wo	1	Yes
	Protected I	No		Pit Latrine/ Finished ro	Rambo/wo	1	Yes
	Protected S	No		No facility/ Finished ro	Rambo/wo	0	No
	Open Well	No		Pit Latrine/ Finished ro	Rambo/wo	1	Yes
	Piped outsi	No		No facility/ Finished ro	Rambo/wo	1	Yes
	Piped outsi	Yes	Boil	Pit Latrine/ Finished ro	Rambo/wo	1	Yes
	Piped into	No		Pit Latrine/ Finished ro	Stone with	1	No
	Covered W	No		Pit Latrine/ Finished ro	Rambo/wo	1	Yes
	Covered W	No		No facility/ Finished ro	Rambo/wo	0	Yes
	Pond/Lake,	No		No facility/ Finished ro	Rambo/wo	1	No
	Piped outsi	No		Pit Latrine/ Finished ro	Other	1	Yes
	Piped outsi	No		Ventilated Finished ro	Rambo/wo	1	Yes
	Protected S	No		No facility/ Finished ro	Rambo/wo	1	Yes
	Piped outsi	No		Pit Latrine/ Finished ro	Rambo/wo	1	No
	Covered W	Yes	Boil	No facility/ Finished ro	Rambo/wo	2	Yes
	Piped outsi	No		No facility/ Finished ro	Rambo/wo	1	Yes
3	Open Sprin	No		Pit Latrine/ Finished ro	Rambo/wo	1	Yes
	Piped outsi	No		Pit Latrine/ Finished ro	Other	1	No
	Piped outsi	Yes	Boil	Pit Latrine/ Finished ro	Rambo/wo	2	Yes
	Protected S	Yes	Boil	No facility/ Finished ro	Rambo/wo	2	Yes
	Piped outsi	No		No facility/ Finished ro	Rambo/wo	1	Yes
	Pond/Lake,	No		Pit Latrine/ Finished ro	Rambo/wo	1	Yes
	Open Sprin	No		Pit Latrine/ Finished ro	Rambo/wo	1	No
	Protected S	No		No facility/ Finished ro	Rambo/wo	0	No
	Open Well	No		No facility/ Finished ro	Rambo/wo	0	Yes
	Piped outsi	No		Pit Latrine/ Finished ro	Rambo/wo	2	Yes
	Protected S	No		Pit Latrine/ Finished ro	Rambo/wo	1	No
	Protected S	No		No facility/ Finished ro	Rambo/wo	1	No
	Protected S	No		No facility/ Finished ro	Rambo/wo	1	Yes
	Piped outsi	No		No facility/ Finished ro	Cement	1	Yes
	Piped outsi	No		No facility/ Thatch/lea	Rambo/wo	1	Yes
	Protected I	No		Pit Latrine/ Finished ro	Rambo/wo	1	Yes
	Piped outsi	No		Pit Latrine/ Finished ro	Rambo/wo	1	Yes
	Piped outsi	No		Pit Latrine/ Finished ro	Rambo/wo	0	Yes

For peer review only

	Piped outsi	No		Pit Latrine/Finished ro	Rambo/wo	1	Yes
	River	No		No facility/Finished ro	Rambo/wo	1	No
1	Covered W	No		Pit Latrine/Finished ro	Rambo/wo	1	No
	Covered W	Yes	Boil	Pit Latrine/Finished ro	Rambo/wo	2	Yes
	Protected	No		No facility/Finished ro	Rambo/wo	1	Yes
	Pond/Lake,	No		Pit Latrine/Finished ro	Rambo/wo	1	Yes
1	Piped into	No		No facility/Finished ro	Rambo/wo	1	Yes
	Protected	No		Pit Latrine/Finished ro	Rambo/wo	1	Yes
	Piped outsi	No		No facility/Finished ro	Rambo/wo	0	Yes
	Piped outsi	No		No facility/Finished ro	Rambo/wo	1	Yes
	Piped outsi	No		No facility/Finished ro	Rambo/wo	1	Yes
2	Piped outsi	No		No facility/Finished ro	Rambo/wo	2	Yes
	Pond/Lake,	No		Pit Latrine/Finished ro	Rambo/wo	1	Yes
	Covered W	Yes	Add bleach	No facility/Thatch/lea	Rambo/wo	1	Yes
	Open Well	No		No facility/Thatch/lea	Other	0	Yes
2	Piped outsi	No		No facility/Thatch/lea	Rambo/wo	0	Yes
	Pond/Lake,	No		Pit Latrine/Finished ro	Rambo/wo	1	No
	Covered W	Yes	Boil	Pit Latrine/Thatch/lea	Rambo/wo	1	Yes
	River	No		Pit Latrine/Finished ro	Rambo/wo	0	Yes
	Protected	Yes	Boil	Pit Latrine/Thatch/lea	Rambo/wo	2	Yes
	Piped outsi	No		Pit Latrine/Finished ro	Rambo/wo	1	Yes
	Protected	No		No facility/Finished ro	Rambo/wo	1	Yes
	Protected	No		No facility/Finished ro	Rambo/wo	0	Yes
	Protected	No		No facility/Finished ro	Rambo/wo	1	Yes
	Covered W	Yes	Boil	No facility/Finished ro	Rambo/wo	2	Yes
	Pond/Lake,	No		Pit Latrine/Finished ro	Rambo/wo	1	Yes
	Covered W	Yes	Boil	No facility/Finished ro	Rambo/wo	1	No
	Pond/Lake,	No		Pit Latrine/Finished ro	Rambo/wo	1	Yes
1	Open Sprin	No		Pit Latrine/Finished ro	Rambo/wo	1	No
	Covered W	No		No facility/Finished ro	Rambo/wo	1	Yes
	Piped outsi	No		No facility/Finished ro	Rambo/wo	0	Yes
	Protected	No		Pit Latrine/Finished ro	Rambo/wo	0	No
	Piped outsi	No		Ventilated Finished ro	Cement	1	Yes
	Pond/Lake,	No		No facility/Finished ro	Rambo/wo	0	Yes
	Covered W	Yes	Boil	Pit Latrine/Finished ro	Rambo/wo	2	Yes
	Piped outsi	No		Pit Latrine/Finished ro	Rambo/wo	0	Yes
	Piped outsi	No		Pit Latrine/Finished ro	Rambo/wo	1	Yes
	Protected	No		Pit Latrine/Finished ro	Rambo/wo	1	Yes
	Piped into	Yes	Boil	Pit Latrine/Finished ro	Rambo/wo	2	No
	Piped outsi	No		No facility/Finished ro	Rambo/wo	1	Yes
	Protected	Yes	Boil	No facility/Finished ro	Rambo/wo	2	Yes
	Piped outsi	No		Pit Latrine/Finished ro	Cement	1	Yes
	Covered W	No		Pit Latrine/Thatch/lea	Rambo/wo	1	Yes
	Piped outsi	No		Pit Latrine/Finished ro	Rambo/wo	0	No
	Piped outsi	No		No facility/Finished ro	Rambo/wo	0	Yes
	Protected	No		No facility/Finished ro	Rambo/wo	1	Yes
	Piped into	No		Pit Latrine/Finished ro	Rambo/wo	1	Yes
	Covered W	No		No facility/Finished ro	Rambo/wo	0	Yes
	Piped outsi	No		Ventilated Thatch/lea	Rambo/wo	0	Yes
	Piped outsi	Yes	Boil	Pit Latrine/Finished ro	Rambo/wo	2	Yes
	Piped outsi	No		Pit Latrine/Finished ro	Rambo/wo	0	Yes

For peer review only

q227	q228	q230a	q230b	q230c	q230d	q230e	q230f	q230g
0.5	Crop residu	Yes	No	No	No	Yes	No	No
0.5	Crop residu	No	No	No	No	No	No	No
0.75	Crop residu	Yes	No	No	No	Yes	No	No
1	Mainly fire	No	No	No	No	Yes	No	No
0.25	Mainly fire	Yes	No	No	No	No	No	No
0.5	Crop residu	Yes	No	No	No	No	No	No
0.75	Crop residu	Yes	No	No	No	Yes	No	No
	Mainly fire	No	No	No	No	Yes	No	No
0.75	Crop residu	Yes	No	No	No	Yes	No	No
0.5	Crop residu	Yes	No	No	No	No	No	No
0.75	Crop residu	Yes	No	Yes	No	Yes	No	No
0.5	Mainly fire	Yes	No	No	No	Yes	No	No
	Crop residu	Yes	No	No	No	Yes	No	No
0.5	Mainly fire	Yes	No	Yes	No	Yes	No	No
0.75	Crop residu	Yes	No	No	No	Yes	No	No
0.5	Crop residu	Yes	No	No	No	Yes	No	No
1	Mainly fire	No	No	No	No	Yes	No	No
1	Crop residu	Yes	No	Yes	No	Yes	No	No
2	Mainly fire	No	No	No	No	Yes	No	No
	Crop residu	No	No	No	No	Yes	No	No
	Mainly fire	No	No	No	No	No	No	No
	Crop residu	No	No	No	No	No	No	No
1.75	Mainly fire	Yes	No	No	No	Yes	No	No
0.5	Mainly fire	No	No	No	No	Yes	No	No
2	Mainly fire	Yes	No	No	No	No	No	No
0	Crop residu	Yes	No	No	No	Yes	No	No
0.5	Crop residu	Yes	No	Yes	No	Yes	No	No
1.5	Crop residu	No	No	No	No	No	No	No
1.25	Crop residu	Yes	No	No	No	Yes	No	No
0.5	Mainly fire	No	No	Yes	No	Yes	No	No
1	Crop residu	Yes	No	No	No	Yes	No	No
1	Crop residu	No	No	No	No	Yes	No	No
2	Mainly fire	Yes	Yes	No	No	Yes	No	No
0	Mainly fire	No	No	No	No	No	No	No
1	Crop residu	Yes	No	No	No	Yes	No	No
0	Mainly fire	No	No	No	No	Yes	No	No
0.75	Crop residu	Yes	No	No	No	Yes	No	No
1	Crop residu	No	No	Yes	No	Yes	No	No
0.25	Mainly fire	Yes	No	No	No	Yes	No	No
0.5	Crop residu	Yes	No	No	No	Yes	No	No
1	Mainly fire	No	No	Yes	No	No	No	No
0.75	Crop residu	Yes	No	Yes	No	Yes	No	No
1.25	Crop residu	Yes	No	No	No	Yes	No	No
2	Mainly fire	No	No	No	No	Yes	No	No
2	Crop residu	Yes	No	No	No	Yes	No	No
0.5	Mainly fire	No	No	No	No	Yes	No	No
0.5	Mainly fire	No	No	No	No	No	No	No
0.5	Mainly fire	Yes	No	No	No	No	No	No
1	Crop residu	No	No	No	No	Yes	No	No
0.25	Crop residu	Yes	No	No	No	Yes	No	No
0.5	Mainly fire	No	No	No	No	Yes	No	No

For peer review only

1.5	Mainly fire	No	No	No	No	Yes	No	No
1	Crop residu	Yes	No	No	No	Yes	No	No
	Mainly fire	Yes	No	No	No	Yes	No	No
1.25	Crop residu	No	No	No	No	No	No	No
0.75	Crop residu	Yes	No	No	No	Yes	No	No
0.75	Animal dur	No	No	No	No	Yes	No	No
0.25	Crop residu	Yes	No	No	No	Yes	No	No
1	Mainly fire	No	No	No	No	Yes	No	No
0.75	Crop residu	Yes	No	No	No	Yes	No	No
0.5	Crop residu	Yes	No	No	No	Yes	No	No
0.75	Crop residu	No	No	No	No	Yes	No	No
	Crop residu	Yes	No	No	No	Yes	No	No
1.75	Crop residu	Yes	No	No	No	Yes	No	No
2	Mainly fire	Yes	No	No	No	Yes	No	No
	Crop residu	Yes	No	Yes	No	Yes	No	No
0.5	Crop residu	Yes	No	No	No	Yes	No	No
0.25	Crop residu	No	No	No	No	Yes	No	No
1	Mainly fire	No	No	No	No	Yes	No	No
1	Mainly fire	No	No	No	No	Yes	No	No
1.5	Crop residu	Yes	No	No	No	Yes	No	No
	Crop residu	Yes	No	Yes	No	Yes	No	No
1	Crop residu	No	No	No	No	Yes	No	No
0.25	Crop residu	Yes	No	No	No	Yes	No	No
	Mainly fire	No	No	No	No	No	No	No
1.5	Crop residu	Yes	No	No	No	No	No	No
1	Crop residu	No	No	No	No	Yes	No	No
1.5	Crop residu	Yes	No	Yes	No	Yes	No	No
1	Crop residu	Yes	No	Yes	No	Yes	No	No
0.5	Mainly fire	No	No	Yes	No	Yes	No	No
0.5	Mainly fire	No	No	No	No	Yes	No	No
0.5	Crop residu	Yes	No	No	No	No	No	No
1	Mainly fire	No	No	Yes	No	Yes	No	No
	Crop residu	Yes	No	No	No	No	No	No
1	Mainly fire	No	No	Yes	No	No	No	No
1	Mainly fire	No	No	No	No	Yes	No	No
2	Mainly fire	Yes	No	No	No	No	No	No
0.5	Mainly fire	No	No	No	No	No	No	No
0.5	Crop residu	No	No	No	No	Yes	No	No
1	Crop residu	No	No	No	No	Yes	No	No
1	Mainly fire	No	Yes	No	No	Yes	No	No
2	Crop residu	No	No	No	No	Yes	No	No
1.25	Crop residu	Yes	No	No	No	Yes	No	No
1.5	Mainly fire	No	No	No	No	Yes	No	No
0.75	Crop residu	Yes	No	No	No	Yes	No	No
1	Mainly fire	No	No	No	No	No	No	No
2.25	Crop residu	Yes	No	No	No	Yes	No	No
	Crop residu	No	No	No	No	No	No	No
	Mainly fire	No	No	No	No	Yes	No	No
0.5	Mainly fire	No	No	No	No	Yes	No	No
	Mainly fire	No	No	No	No	No	No	No
	Mainly fire	No	Yes	Yes	No	Yes	No	Yes
0	Mainly fire	No	No	Yes	No	Yes	No	No

For peer review only

	Crop residu	No	No	No	No	Yes	No	No
1.25	Crop residu	Yes	No	No	No	Yes	No	No
0.5	Crop residu	Yes	No	No	No	Yes	No	No
	Crop residu	Yes	No	No	No	Yes	No	No
1.5	Crop residu	No	No	No	No	Yes	No	No
0	Mainly fire	No	No	No	No	No	No	No
	Mainly fire	No	No	No	No	Yes	No	No
0.5	Mainly fire	No	No	No	No	No	No	No
0.5	Mainly fire	No	No	No	No	Yes	No	No
0.75	Crop residu	Yes	No	No	No	Yes	No	No
1	Mainly fire	Yes	No	No	No	Yes	No	No
1	Crop residu	Yes	No	No	No	Yes	No	No
	Crop residu	Yes	No	Yes	No	Yes	No	No
	Crop residu	Yes	No	No	No	Yes	No	No
0.5	Mainly fire	No	No	No	No	Yes	No	No
1.5	Mainly fire	Yes	No	No	No	No	No	No
3	Crop residu	Yes	No	No	No	Yes	No	No
1	Crop residu	Yes	No	No	No	Yes	No	No
0.75	Mainly fire	No	No	No	No	No	No	No
1	Mainly fire	No	No	No	No	Yes	No	No
1.25	Crop residu	Yes	No	Yes	No	Yes	No	No
0.5	Crop residu	Yes	No	No	No	Yes	No	No
0.5	Mainly fire	No	No	No	No	Yes	No	No
	Crop residu	Yes	No	No	No	Yes	No	No
1.5	Mainly fire	No	No	No	No	Yes	No	No
1.5	Crop residu	Yes	No	Yes	No	Yes	No	No
0.25	Crop residu	Yes	No	Yes	No	Yes	No	No
1	Mainly fire	No	No	No	No	No	No	No
2	Mainly fire	Yes	No	No	No	No	No	No
1	Mainly fire	Yes	No	No	No	Yes	No	No
2	Mainly fire	Yes	No	No	No	No	No	No
	Mainly fire	Yes	No	No	No	No	No	No
1.5	Mainly fire	No	No	No	No	Yes	No	No
3	Mainly fire	Yes	No	No	No	Yes	No	No
1	Mainly fire	Yes	No	No	No	No	No	No
0.5	Crop residu	Yes	No	No	No	No	No	No
1.5	Mainly fire	Yes	No	No	No	Yes	No	No
2	Mainly fire	Yes	No	No	No	Yes	No	No
	Mainly fire	No	No	No	No	No	No	No
2	Mainly fire	Yes	No	No	No	No	No	No
0.5	Mainly fire	No	No	No	No	No	No	No
0.5	Mainly fire	No	No	No	No	Yes	No	No
2	Mainly fire	No	No	No	No	Yes	No	No
0.5	Mainly fire	Yes	No	No	No	Yes	No	No
1.25	Mainly fire	No	No	No	No	Yes	No	No
0.5	Mainly fire	Yes	No	No	No	Yes	No	No
0.25	Mainly fire	No	No	No	No	No	No	No
	Mainly fire	Yes	No	No	No	Yes	No	No
0.75	Mainly fire	No	No	No	No	No	No	No
1	Mainly fire	Yes	No	No	No	Yes	No	No
1	Mainly fire	Yes	No	No	No	Yes	No	No
	Mainly fire	No	No	No	No	No	No	No

For peer review only

0.25	Crop residu	Yes	No	No	No	Yes	No	No
2	Mainly fire	No	No	No	No	Yes	No	No
1.5	Crop residu	No	No	No	No	Yes	No	No
2	Mainly fire	Yes	No	No	No	No	No	No
0.5	Mainly fire	No	No	No	No	No	No	No
0.25	Mainly fire	Yes	No	No	No	Yes	No	No
1.5	Mainly fire	Yes	No	No	No	Yes	No	No
0.5	Mainly fire	Yes	No	No	No	Yes	No	No
0.5	Mainly fire	Yes	No	No	No	No	No	No
1	Crop residu	Yes	No	No	No	No	No	No
1	Mainly fire	Yes	No	No	No	No	No	No
2	Mainly fire	Yes	No	No	No	No	No	No
0.5	Mainly fire	Yes	No	No	No	No	No	No
	Mainly fire	Yes	No	No	No	No	No	No
0.25	Mainly fire	Yes	No	No	No	No	No	No
1	Mainly fire	Yes	No	No	No	Yes	No	No
3	Mainly fire	Yes	No	No	No	Yes	No	No
	Mainly fire	Yes	No	No	No	Yes	No	No
0.5	Mainly fire	Yes	No	No	No	Yes	No	No
0.25	Mainly fire	Yes	No	No	No	Yes	No	No
1	Mainly fire	No	No	No	No	No	No	No
1.25	Crop residu	Yes	No	No	No	No	No	No
2	Mainly fire	No	No	No	No	No	No	No
1	Mainly fire	Yes	No	No	No	Yes	No	No
	Mainly fire	Yes	No	Yes	No	Yes	No	No
1	Mainly fire	Yes	No	No	No	Yes	No	No
	Crop residu	Yes	No	Yes	No	Yes	No	No
	Mainly fire	Yes	No	No	No	Yes	No	No
	Mainly fire	No	No	No	No	No	No	No
	Mainly fire	Yes	No	No	No	Yes	No	No
1	Mainly fire	No	No	No	No	No	No	No
	Mainly fire	No	No	No	No	No	No	No
	Mainly fire	Yes	No	No	No	Yes	No	No
2	Mainly fire	Yes	No	No	No	Yes	No	No
1	Mainly fire	Yes	No	No	No	No	No	No
	Mainly fire	Yes	No	No	No	Yes	No	No
0.5	Mainly fire	Yes	No	No	No	Yes	No	No
0.5	Mainly fire	Yes	No	Yes	No	Yes	No	No
0.5	Mainly fire	Yes	No	No	No	Yes	No	No
1	Mainly fire	Yes	No	No	No	No	No	No
1	Mainly fire	Yes	No	No	No	Yes	No	No
	Mainly fire	Yes	No	No	No	Yes	No	No
	Mainly fire	No	No	No	No	Yes	No	No
0.5	Mainly fire	Yes	No	No	No	No	No	No
	Mainly fire	Yes	No	No	No	No	No	No
1	Crop residu	Yes	No	No	No	No	No	No
2	Crop residu	Yes	No	No	No	Yes	No	No
4	Mainly fire	No	No	No	No	Yes	No	No
	Mainly fire	Yes	No	No	No	Yes	No	No
1	Mainly fire	Yes	No	No	No	No	No	No
0.5	Mainly fire	No	No	No	No	No	No	No
0.5	Mainly fire	Yes	No	No	No	No	No	No

For peer review only

	Mainly fire	No	No	No	No	Yes	No	No
0.5	Mainly fire	Yes	No	No	No	No	No	No
5	Mainly fire	Yes	No	No	No	Yes	No	No
0.5	Mainly fire	Yes	No	No	No	No	No	No
2	Mainly fire	Yes	No	No	No	Yes	No	No
	Mainly fire	Yes	No	No	No	Yes	No	No
4	Mainly fire	Yes	No	No	No	Yes	No	No
	Mainly fire	Yes	No	No	No	Yes	No	No
3	Mainly fire	Yes	No	No	No	Yes	No	No
8	Mainly fire	Yes	No	Yes	No	Yes	No	Yes
2	Mainly fire	Yes	No	Yes	No	Yes	No	No
4	Mainly fire	Yes	No	No	No	Yes	No	No
	Mainly fire	Yes	No	No	No	Yes	No	No
2	Mainly fire	Yes	No	No	No	Yes	No	No
1	Mainly fire	Yes	No	Yes	No	Yes	No	No
	Mainly fire	Yes	No	No	No	Yes	No	No
1	Mainly fire	Yes	No	No	No	Yes	No	No
1	Mainly fire	Yes	No	No	No	Yes	No	No
1	Mainly fire	Yes	No	No	No	No	No	No
1	Mainly fire	Yes	No	No	No	Yes	No	No
	Mainly fire	Yes	No	No	No	Yes	No	No
0.75	Mainly fire	No	No	No	No	Yes	No	No
2	Mainly fire	Yes	No	No	No	Yes	No	No
1	Mainly fire	Yes	No	No	No	Yes	No	No
1	Mainly fire	Yes	No	No	No	Yes	No	No
0.5	Mainly fire	Yes	No	No	No	Yes	No	No
1	Mainly fire	Yes	No	No	No	Yes	No	No
0.5	Mainly fire	Yes	No	No	No	No	No	No
2	Mainly fire	Yes	No	No	No	Yes	No	No
6	Mainly fire	Yes	Yes	Yes	No	Yes	No	No
	Mainly fire	Yes	No	No	No	No	No	No
	Mainly fire	No	No	No	No	Yes	No	No
	Mainly fire	Yes	No	No	No	Yes	No	No
	Mainly fire	Yes	No	No	No	Yes	No	No
	Mainly fire	Yes	No	No	No	Yes	No	No
	Mainly fire	Yes	No	No	No	Yes	No	No
1	Mainly fire	Yes	No	No	No	Yes	No	No
6	Mainly fire	Yes	No	No	No	No	No	No
1	Crop residu	Yes	No	No	No	No	No	No
	Mainly fire	Yes	No	Yes	Yes	Yes	No	No
1	Mainly fire	Yes	No	No	No	Yes	No	No
1	Mainly fire	Yes	No	No	No	Yes	No	No
1	Crop residu	Yes	No	No	No	Yes	No	No
1	Mainly fire	Yes	No	No	No	Yes	No	No
1	Mainly fire	Yes	No	No	No	Yes	No	No
1.5	Mainly fire	Yes	No	No	No	Yes	No	No
4	Mainly fire	Yes	No	Yes	No	Yes	No	No
2.5	Mainly fire	Yes	No	Yes	No	Yes	No	No
1	Mainly fire	Yes	No	No	No	Yes	No	No
4	Mainly fire	Yes	No	No	No	Yes	No	No
1	Mainly fire	Yes	No	No	No	No	No	No
1.5	Mainly fire	Yes	No	No	No	Yes	No	No

For peer review only

4	Mainly fire	Yes	No	No	No	Yes	No	No
1	Crop residu	Yes	No	No	No	Yes	No	No
2	Mainly fire	Yes	No	No	No	Yes	No	No
0.5	Mainly fire	Yes	No	No	No	No	No	No
1.5	Mainly fire	Yes	No	No	No	Yes	No	No
4	Mainly fire	Yes	No	No	Yes	Yes	No	Yes
1	Crop residu	No	No	No	No	No	No	No
2	Crop residu	Yes	No	No	Yes	Yes	No	No
4	Mainly fire	Yes	No	No	No	Yes	No	No
	Mainly fire	No	No	No	No	No	No	No
1	Mainly fire	Yes	No	No	No	Yes	No	No
0.5	Mainly fire	Yes	No	No	No	No	No	No
1.5	Crop residu	No	No	No	No	No	No	No
5	Mainly fire	Yes	No	Yes	No	Yes	No	No
1	Mainly fire	Yes	No	No	No	Yes	No	No
0.5	Crop residu	No	No	No	No	No	No	No
1.5	Mainly fire	Yes	No	No	No	Yes	No	No
1	Mainly fire	Yes	No	No	No	No	No	No
1	Mainly fire	Yes	No	No	No	Yes	No	No
	Mainly fire	Yes	No	No	No	Yes	No	No
0.5	Mainly fire	No	No	No	No	No	No	No
	Mainly fire	Yes	No	No	No	Yes	No	No
	Mainly fire	Yes	No	No	No	Yes	No	No
	Mainly fire	Yes	No	No	No	Yes	No	No
2	Mainly fire	No	No	No	No	Yes	No	No
6	Mainly fire	Yes	No	Yes	No	Yes	No	No
0.5	Crop residu	No	No	No	No	No	No	No
2	Mainly fire	Yes	No	No	No	Yes	No	No
	Crop residu	Yes	No	No	No	Yes	No	No
1	Mainly fire	Yes	No	Yes	No	Yes	No	No
1	Mainly fire	Yes	No	No	No	Yes	No	No
2	Mainly fire	Yes	No	No	No	No	No	No
1	Mainly fire	Yes	No	No	No	Yes	No	No
1	Mainly fire	Yes	No	No	No	Yes	No	No
1	Mainly fire	Yes	No	No	No	No	No	No
1	Mainly fire	Yes	No	No	No	Yes	No	No
1	Mainly fire	Yes	No	No	No	Yes	No	No
0.75	Crop residu	No	No	No	No	No	No	No
3	Mainly fire	Yes	No	No	No	No	No	No
	Mainly fire	No	No	No	No	No	No	No
	Mainly fire	Yes	No	No	No	Yes	No	No
	Mainly fire	No	No	No	No	Yes	No	No
	Mainly fire	Yes	No	No	No	Yes	No	No
1	Mainly fire	No	No	No	No	Yes	No	No
2	Mainly fire	Yes	No	No	No	Yes	No	No
1	Mainly fire	Yes	No	No	No	Yes	No	No
0	Mainly fire	Yes	No	No	No	Yes	No	No
0.5	Mainly fire	Yes	No	No	No	No	No	No
5	Mainly fire	Yes	No	Yes	Yes	Yes	No	No
2	Mainly fire	Yes	No	No	No	No	No	No
1	Mainly fire	Yes	No	No	No	Yes	No	No
	Mainly fire	Yes	No	No	No	Yes	No	No

For peer review only

	Mainly fire	Yes	No	No	No	Yes	No	No
1	Mainly fire	Yes	No	No	No	Yes	No	No
0.5	Mainly fire	Yes	No	No	No	No	No	No
	Mainly fire	Yes	No	No	No	Yes	No	No
4	Mainly fire	Yes	No	No	No	Yes	No	No
3	Mainly fire	Yes	No	No	No	Yes	No	No
4	Mainly fire	Yes	No	No	No	Yes	No	No
0.5	Mainly fire	Yes	No	No	No	Yes	No	No
1	Mainly fire	Yes	No	No	No	Yes	No	No
1	Mainly fire	Yes	No	No	No	Yes	No	No
	Mainly fire	Yes	No	No	No	Yes	No	No
	Mainly fire	Yes	No	No	No	Yes	No	No
5	Mainly fire	Yes	No	No	No	Yes	No	No
	Mainly fire	Yes	No	No	No	Yes	No	No
0.75	Mainly fire	Yes	No	No	No	No	No	No
0.5	Mainly fire	Yes	No	No	No	Yes	No	No
3	Mainly fire	No	No	No	No	Yes	No	No
1	Mainly fire	Yes	No	No	No	Yes	No	No
2	Mainly fire	Yes	No	No	No	Yes	No	No
0.25	Mainly fire	Yes	No	No	No	Yes	No	No
2	Mainly fire	Yes	No	No	No	Yes	No	No
4	Mainly fire	Yes	No	No	No	Yes	No	No
1	Mainly fire	Yes	No	No	No	Yes	No	No
4	Mainly fire	Yes	No	No	No	Yes	No	No
8	Mainly fire	Yes	No	No	No	Yes	No	No
6	Mainly fire	Yes	No	Yes	No	Yes	No	No
1.5	Crop residu	Yes	No	No	No	No	No	No
	Mainly fire	Yes	No	No	No	Yes	No	No
4	Mainly fire	Yes	No	No	No	Yes	No	No
	Mainly fire	Yes	No	No	No	Yes	No	No
1.5	Mainly fire	No	No	No	No	Yes	No	No
1	Mainly fire	Yes	No	No	No	No	No	No
	Mainly fire	Yes	No	No	No	No	No	No
	Mainly fire	No	No	No	No	No	No	No
1.5	Mainly fire	No	No	No	No	Yes	No	No
	Mainly fire	Yes	No	No	No	Yes	No	No
0.5	Mainly fire	No	No	No	No	No	No	No
0.5	Mainly fire	Yes	No	No	No	Yes	No	No
3	Mainly fire	Yes	No	Yes	No	Yes	No	No
	Mainly fire	Yes	No	No	No	No	No	No
	Mainly fire	Yes	No	No	No	Yes	No	No
	Mainly fire	Yes	No	No	No	Yes	No	No
	Mainly fire	Yes	No	Yes	No	Yes	No	No
0.5	Mainly fire	Yes	No	No	No	No	No	No
0.5	Mainly fire	Yes	No	No	No	Yes	No	No
1	Mainly fire	No	No	No	No	Yes	No	No
0.5	Mainly fire	Yes	No	No	No	Yes	No	No
	Mainly fire	No	No	No	No	No	No	No
0.5	Mainly fire	Yes	No	No	No	Yes	No	No
	Mainly fire	Yes	No	No	No	No	No	No
1	Mainly fire	Yes	No	No	No	Yes	No	No
1.5	Mainly fire	Yes	No	No	Yes	No	No	No

	Mainly fire	No	No	No	No	No	No	No
	Mainly fire	Yes	No	No	No	No	No	Yes
	Mainly fire	Yes	No	No	No	No	No	No
1	Mainly fire	No	No	No	No	Yes	No	No
0.5	Mainly fire	Yes	No	No	No	Yes	No	No
	Mainly fire	Yes	No	No	No	No	No	No
1	Mainly fire	Yes	No	No	No	No	No	No
	Mainly fire	Yes	No	No	No	No	No	No
0.5	Crop residu	No	No	No	No	Yes	No	No
0.5	Mainly fire	Yes	No	No	No	Yes	No	No
	Mainly fire	Yes	No	No	No	Yes	No	No
1	Mainly fire	No	No	No	No	No	No	No
	Mainly fire	Yes	No	No	No	Yes	No	No
0.75	Mainly fire	No	No	No	No	Yes	No	No
	Mainly fire	No	No	No	No	No	No	No
1	Mainly fire	Yes	No	No	No	Yes	No	No
0.5	Mainly fire	Yes	No	No	No	No	No	No
	Mainly fire	Yes	No	No	No	Yes	No	No
	Mainly fire	Yes	No	Yes	No	No	No	No
1	Mainly fire	Yes	No	No	No	Yes	No	No
1	Mainly fire	Yes	No	No	No	No	No	No
1	Mainly fire	No	No	No	No	No	No	No
	Mainly fire	Yes	No	Yes	No	Yes	No	No
1	Mainly fire	Yes	No	No	No	Yes	No	No
1.5	Mainly fire	Yes	No	No	No	Yes	No	No
	Mainly fire	Yes	No	No	No	Yes	No	No
0.5	Mainly fire	Yes	No	No	No	No	No	No
	Mainly fire	No	No	No	No	Yes	No	No
1	Mainly fire	No	No	No	No	Yes	No	No
1	Mainly fire	No	No	No	No	Yes	No	No
0.2	Mainly fire	Yes	No	No	No	No	No	No
0.5	Mainly fire	No	No	No	No	No	No	No
1	Mainly fire	No	No	No	No	No	No	No
0.5	Mainly fire	Yes	No	Yes	No	Yes	No	No
0.5	Mainly fire	Yes	No	No	No	No	No	No
1	Mainly fire	No	No	No	No	No	No	No
1.2	Mainly fire	Yes	No	No	No	Yes	No	No
1	Mainly fire	Yes	No	No	No	Yes	No	No
1	Mainly fire	Yes	No	No	No	No	No	No
1	Mainly fire	Yes	No	No	No	No	No	No
0.5	Mainly fire	No	No	No	No	No	No	No
	Mainly fire	No	No	No	No	Yes	No	No
1	Mainly fire	Yes	No	No	No	No	No	No
0.5	Mainly fire	Yes	No	No	No	No	No	No
1	Mainly fire	Yes	No	No	No	No	No	No
	Mainly fire	Yes	No	No	No	Yes	No	No
1	Mainly fire	No	No	No	No	No	No	No
	Mainly fire	No	No	No	No	No	No	No
1	Mainly fire	Yes	No	No	No	No	No	No
2	Mainly fire	Yes	No	No	No	Yes	No	No
	Mainly fire	No	No	No	No	No	No	No
	Mainly fire	Yes	No	No	No	Yes	No	No

For peer review only

	Mainly fire	No	No	No	No	No	No	No
0.5	Mainly fire	Yes	No	No	No	No	No	No
1	Mainly fire	Yes	No	No	No	Yes	No	No
	Mainly fire	Yes	No	No	No	No	No	No
0.7	Mainly fire	Yes	No	No	No	No	No	No
1	Mainly fire	Yes	No	No	No	No	No	No
	Mainly fire	Yes	No	No	No	No	No	No
1	Mainly fire	Yes	No	No	No	Yes	No	No
	Mainly fire	Yes	No	Yes	No	No	No	No
	Mainly fire	Yes	No	Yes	Yes	Yes	No	No
4	Mainly fire	Yes	No	Yes	No	Yes	No	No
1	Mainly fire	No	No	No	No	No	No	No
4	Mainly fire	Yes	No	No	No	No	No	No
0.5	Mainly fire	No	No	No	No	Yes	No	No
4	Mainly fire	Yes	No	No	No	No	No	No
2	Mainly fire	Yes	No	No	No	Yes	No	No
0.5	Mainly fire	No	No	No	No	No	No	No
1	Mainly fire	Yes	No	No	No	Yes	No	No
1	Mainly fire	No	No	No	No	No	No	No
4	Mainly fire	Yes	No	No	No	No	No	No
4	Mainly fire	Yes	No	No	No	Yes	No	No
3	Mainly fire	No	No	Yes	No	Yes	No	No
	Mainly fire	Yes	No	No	No	Yes	No	No
	Mainly fire	Yes	No	No	No	Yes	No	No
1	Mainly fire	Yes	No	No	No	Yes	No	No
	Mainly fire	Yes	No	No	No	No	No	No
1	Mainly fire	No	No	No	No	No	No	No
	Mainly fire	Yes	No	Yes	Yes	Yes	No	No
	Mainly fire	Yes	No	No	No	No	No	No
1	Mainly fire	No	No	No	No	No	No	No
3	Mainly fire	Yes	No	No	No	Yes	No	No
5	Mainly fire	Yes	No	No	No	Yes	No	No
0.75	Mainly fire	No	No	No	No	No	No	No
4	Mainly fire	Yes	No	No	No	Yes	No	No
1.2	Mainly fire	No	No	No	No	No	No	No
1	Mainly fire	No	No	No	No	No	No	No
1	Mainly fire	Yes	No	No	No	No	No	No
3	Mainly fire	Yes	No	No	No	Yes	No	No
0.5	Mainly fire	Yes	No	No	No	Yes	No	No
1	Mainly fire	Yes	No	No	No	No	No	No
7	Mainly fire	Yes	No	No	No	Yes	No	No
1	Mainly fire	No	No	No	No	No	No	No
	Mainly fire	Yes	No	No	Yes	Yes	No	No
3	Mainly fire	Yes	No	No	No	Yes	No	No
1	Mainly fire	Yes	No	No	No	No	No	No
	Mainly fire	Yes	No	No	No	Yes	No	No
4	Mainly fire	Yes	No	No	No	Yes	No	No
0.5	Mainly fire	No	No	No	No	No	No	No
	Mainly fire	Yes	No	No	No	Yes	No	No
2	Mainly fire	Yes	No	No	No	Yes	No	No
4	Mainly fire	Yes	No	No	No	Yes	No	No
1	Mainly fire	Yes	No	No	No	No	No	No

For peer review only

1	Mainly fire	No	No	No	No	No	No	No
4	Mainly fire	Yes	No	No	No	Yes	No	No
	Mainly fire	Yes	No	No	No	Yes	No	No
	Mainly fire	No	No	No	No	Yes	No	No
	Mainly fire	Yes	Yes	No	No	Yes	No	No
3	Mainly fire	Yes	No	No	No	Yes	No	No
1	Mainly fire	Yes	No	No	No	No	No	No
	Mainly fire	Yes	No	No	No	Yes	No	No
1	Mainly fire	Yes	No	No	No	Yes	No	No
1	Mainly fire	Yes	No	No	No	Yes	No	No
	Mainly fire	Yes	No	No	No	Yes	No	No
	Mainly fire	Yes	No	No	No	Yes	No	No
	Mainly fire	Yes	No	No	No	No	No	No
1	Mainly fire	Yes	No	No	Yes	Yes	No	No
2	Mainly fire	Yes	No	No	No	Yes	No	No
1	Mainly fire	Yes	No	No	No	Yes	No	No
0.5	Mainly fire	Yes	No	No	No	No	No	No
	Mainly fire	No	No	Yes	No	Yes	No	No
1	Mainly fire	Yes	No	Yes	No	Yes	No	No
1	Mainly fire	No	No	Yes	No	Yes	No	No
1	Mainly fire	Yes	No	Yes	No	Yes	No	No
	Mainly fire	No	No	Yes	No	Yes	No	No
0.5	Animal dur	No	No	No	No	Yes	No	No
	Mainly fire	Yes	No	Yes	No	Yes	No	No
1	Animal dur	No	No	Yes	No	Yes	No	No
	Mainly fire	No	No	Yes	No	Yes	No	No
	Mainly fire	Yes	No	Yes	No	Yes	No	No
0.5	Mainly fire	Yes	No	Yes	No	Yes	No	No
	Animal dur	No	No	No	No	No	No	No
	Animal dur	No	No	Yes	No	Yes	No	No
1	Mainly fire	No	No	Yes	No	Yes	No	No
1	Animal dur	No	No	Yes	No	Yes	Yes	No
0.5	Mainly fire	Yes	No	Yes	No	Yes	No	No
0.5	Animal dur	Yes	No	Yes	No	Yes	No	No
1	Mainly fire	No	No	No	No	Yes	No	No
2	Animal dur	No	No	No	No	Yes	No	Yes
1	Mainly fire	No	Yes	Yes	No	Yes	No	No
0.5	Mainly fire	No	No	No	No	Yes	No	No
1.5	Animal dur	No	No	Yes	No	Yes	No	No
1	Mainly fire	No	No	Yes	No	Yes	No	No
1.5	Animal dur	Yes	No	Yes	No	Yes	No	No
1	Animal dur	No	No	No	No	Yes	No	No
1.5	Mainly fire	No	No	No	No	Yes	No	No
1	Mainly fire	No	No	No	No	Yes	No	No
0.5	Mainly fire	No	No	Yes	No	Yes	No	No
1.25	Mainly fire	No	No	Yes	No	Yes	No	No
1.5	Animal dur	No	No	Yes	No	Yes	No	No
0.75	Mainly fire	No	No	Yes	No	Yes	No	No
	Mainly fire	No	No	Yes	No	Yes	No	No
0.5	Mainly fire	No	Yes	Yes	No	Yes	No	No
0	Mainly fire	No	No	No	No	Yes	No	No
0.5	Mainly fire	No	Yes	Yes	No	Yes	No	No

For peer review only

1.5	Animal dur	No	No	Yes	No	Yes	No	No
0.5	Mainly fire	No	No	No	No	Yes	No	No
1.5	Animal dur	No	No	Yes	No	Yes	No	No
1.5	Animal dur	No	No	Yes	No	Yes	No	No
1.5	Mainly fire	No	No	Yes	No	Yes	No	No
0.75	Mainly fire	No	No	Yes	No	Yes	No	No
2	Animal dur	No	No	Yes	No	Yes	No	No
	Mainly fire	No	No	No	No	Yes	No	No
1	Mainly fire	No	No	Yes	No	Yes	No	No
2	Mainly fire	Yes	No	Yes	No	Yes	No	No
0.5	Mainly fire	No	No	Yes	No	No	No	No
4	Animal dur	No	No	Yes	No	Yes	Yes	No
2	Animal dur	No	No	Yes	No	Yes	Yes	No
	Mainly fire	No	No	Yes	No	Yes	No	No
0.5	Mainly fire	Yes	No	Yes	No	Yes	No	No
	Mainly fire	No	No	No	No	Yes	No	No
1.5	Mainly fire	Yes	No	Yes	No	Yes	No	No
1	Animal dur	No	No	Yes	No	Yes	No	No
0.5	Mainly fire	No	No	Yes	No	Yes	No	No
2	Animal dur	No	No	Yes	No	Yes	No	No
	Mainly fire	No	No	No	No	Yes	No	No
2	Mainly fire	Yes	No	No	No	Yes	No	No
	Mainly fire	Yes	No	No	No	Yes	No	No
3	Mainly fire	Yes	No	No	No	Yes	No	No
	Mainly fire	Yes	No	Yes	No	Yes	No	No
	Mainly fire	No	No	No	No	Yes	No	No
1.5	Mainly fire	Yes	No	No	No	No	No	No
	Mainly fire	Yes	No	No	No	No	No	No
	Mainly fire	Yes	No	No	No	Yes	No	No
	Mainly fire	No	No	No	No	No	No	No
	Mainly fire	Yes	No	No	No	No	No	No
	Mainly fire	Yes	Yes	No	No	Yes	No	No
2	Mainly fire	No	No	No	No	No	No	No
0.5	Mainly fire	Yes	No	No	No	Yes	No	No
0.5	Mainly fire	Yes	No	No	No	Yes	No	No
3	Mainly fire	Yes	No	No	No	Yes	No	No
2	Mainly fire	Yes	No	No	No	Yes	No	No
	Mainly fire	No	No	No	No	No	No	No
2	Mainly fire	Yes	No	No	No	Yes	No	No
	Mainly fire	No	No	No	No	No	No	No
	Mainly fire	Yes	No	No	No	Yes	No	No
4	Mainly fire	Yes	No	No	No	No	No	No
2	Mainly fire	Yes	No	No	No	Yes	No	No
	Mainly fire	No	No	No	No	No	No	No
	Mainly fire	Yes	No	No	No	Yes	No	No
2	Mainly fire	Yes	No	No	No	No	No	No
	Mainly fire	Yes	No	No	No	Yes	No	No
	Mainly fire	Yes	No	Yes	No	Yes	No	No
1.75	Mainly fire	Yes	No	No	No	No	No	No
4	Mainly fire	Yes	No	No	No	No	No	No
	Mainly fire	Yes	No	No	No	Yes	No	No
2	Mainly fire	Yes	No	No	No	No	No	No

For peer review only

For peer review only

1	Mainly fire	Yes	No	No	No	Yes	No	No
1	Mainly fire	Yes	No	No	No	Yes	No	No
	Mainly fire	Yes	No	No	No	Yes	No	No
	Mainly fire	Yes	No	No	No	Yes	No	No
3	Mainly fire	Yes	No	No	No	Yes	No	No
3	Mainly fire	Yes	No	No	No	No	No	No
4	Mainly fire	Yes	No	No	No	No	No	No
	Mainly fire	Yes	No	Yes	No	Yes	No	No
	Mainly fire	Yes	No	No	No	Yes	No	No
	Mainly fire	Yes	No	No	No	Yes	No	No
0.5	Mainly fire	No	No	No	No	No	No	No
	Mainly fire	No	No	No	No	No	No	No
	Mainly fire	Yes	No	No	No	Yes	No	No
2	Mainly fire	Yes	No	No	No	Yes	No	No
	Mainly fire	Yes	No	No	No	Yes	No	No
	Mainly fire	Yes	No	No	No	Yes	No	No
	Charcoal	No	No	No	No	No	No	No
	Mainly fire	No	No	No	No	Yes	No	No
2	Mainly fire	Yes	No	No	No	Yes	No	No
5	Mainly fire	Yes	No	No	No	Yes	No	No
	Mainly fire	Yes	No	No	No	Yes	No	No
2	Mainly fire	Yes	No	No	No	Yes	No	No
	Mainly fire	Yes	No	No	No	Yes	No	No
	Mainly fire	Yes	No	No	No	Yes	No	No
0.75	Mainly fire	Yes	No	No	No	Yes	No	No
	Mainly fire	No	No	No	No	Yes	No	No
3	Mainly fire	Yes	No	No	No	No	No	No
0.5	Mainly fire	Yes	No	No	No	No	No	No
0.5	Mainly fire	No	No	No	No	No	No	No
0.5	Animal dur	No	No	No	No	No	No	No
0.5	Mainly fire	Yes	No	Yes	No	Yes	No	No
1	Mainly fire	Yes	No	No	No	No	No	No
	Mainly fire	No	No	No	No	No	No	No
	Mainly fire	Yes	No	No	No	Yes	No	No
3	Mainly fire	Yes	No	No	No	Yes	No	No
0.5	Mainly fire	Yes	No	No	No	Yes	No	No
4	Mainly fire	Yes	No	No	No	Yes	No	No
	Mainly fire	Yes	No	Yes	Yes	Yes	No	No
	Mainly fire	Yes	No	Yes	Yes	Yes	No	No
	Mainly fire	No	No	No	No	No	No	No
0.5	Mainly fire	Yes	No	No	No	Yes	No	No
2	Mainly fire	Yes	No	No	No	No	No	No
1	Mainly fire	Yes	No	No	No	Yes	No	No
0.5	Mainly fire	Yes	No	No	No	Yes	No	No
	Mainly fire	No	No	No	No	Yes	No	No
	Mainly fire	No	No	No	Yes	Yes	No	No
1	Mainly fire	Yes	No	Yes	No	Yes	No	No
5	Mainly fire	Yes	No	No	No	Yes	No	No
4	Mainly fire	Yes	No	No	No	No	No	No
	Mainly fire	Yes	No	No	No	Yes	No	No
4	Mainly fire	Yes	No	No	No	Yes	No	No
	Mainly fire	Yes	No	No	No	Yes	No	No

0.5	Mainly fire'	Yes	No	No	No	Yes	No	No
	Mainly fire'	No	No	No	No	Yes	No	No
	Mainly fire'	Yes	No	No	No	Yes	No	No
1	Mainly fire'	Yes	No	No	No	Yes	No	No
1	Mainly fire'	Yes	No	No	No	Yes	No	No
3	Mainly fire'	Yes	No	Yes	No	Yes	No	No
2	Mainly fire'	Yes	No	No	No	Yes	No	No
2	Mainly fire'	Yes	No	No	No	Yes	No	No
1	Mainly fire'	Yes	No	No	No	Yes	No	No
	Mainly fire'	Yes	No	Yes	Yes	Yes	No	No
1.5	Mainly fire'	Yes	No	No	No	No	No	No
4	Mainly fire'	Yes	No	No	No	Yes	No	No
3	Mainly fire'	Yes	No	Yes	No	Yes	No	No
	Mainly fire'	Yes	No	No	No	Yes	No	No
	Mainly fire'	No	No	No	No	No	No	No
	Mainly fire'	Yes	No	No	No	Yes	No	No
2	Mainly fire'	Yes	No	No	No	Yes	No	No
2	Mainly fire'	Yes	No	Yes	No	Yes	No	No
2	Mainly fire'	Yes	No	No	No	Yes	No	No
4	Mainly fire'	Yes	No	No	No	No	No	No
1	Mainly fire'	Yes	No	No	No	Yes	No	No
2	Mainly fire'	Yes	No	No	No	Yes	No	No
	Mainly fire'	Yes	No	No	No	Yes	No	No
	Mainly fire'	No	No	No	No	No	No	No
3.5	Mainly fire'	Yes	No	No	No	Yes	No	No
	Mainly fire'	Yes	No	No	No	Yes	No	No
1.75	Mainly fire'	Yes	No	No	No	No	No	No
	Mainly fire'	Yes	No	No	Yes	No	No	No
	Mainly fire'	Yes	No	No	No	Yes	No	No
	Mainly fire'	Yes	No	No	No	Yes	No	No
1	Mainly fire'	No	No	No	No	No	No	No
	Mainly fire'	Yes	No	No	No	Yes	No	No
0.5	Mainly fire'	Yes	No	No	No	Yes	No	No
1	Mainly fire'	Yes	No	No	No	No	No	No
	Mainly fire'	Yes	No	No	No	No	No	No
1	Mainly fire'	Yes	No	No	No	Yes	No	No
1	Mainly fire'	Yes	No	No	No	Yes	No	No
	Mainly fire'	Yes	No	No	No	Yes	No	No
	Mainly fire'	Yes	No	No	No	Yes	No	No
	Mainly fire'	Yes	No	Yes	No	Yes	No	No
1	Mainly fire'	No	No	No	No	No	No	No
1	Mainly fire'	Yes	No	No	No	Yes	No	No
0.5	Mainly fire'	Yes	No	No	No	Yes	No	No
	Mainly fire'	Yes	No	No	No	No	No	No
1	Mainly fire'	No	No	No	No	No	No	No
2	Mainly fire'	Yes	No	No	No	Yes	No	No
1	Mainly fire'	No	No	No	No	No	No	No
0.5	Mainly fire'	Yes	No	No	No	Yes	No	No
0.25	Mainly fire'	Yes	No	No	No	No	No	No
0.7	Mainly fire'	No	No	No	No	Yes	No	No
2	Mainly fire'	Yes	No	No	No	Yes	No	No
	Mainly fire'	Yes	No	No	No	No	No	No

For peer review only

1.5	Mainly fire	No	No	No	No	Yes	No	No
	Mainly fire	Yes	No	No	Yes	Yes	No	No
2	Crop residu	Yes	No	No	No	Yes	No	No
	Mainly fire	Yes	No	No	No	Yes	No	No
2	Mainly fire	Yes	No	Yes	No	Yes	No	No
1	Mainly fire	Yes	No	No	No	No	No	No
8	Mainly fire	Yes	No	No	No	Yes	No	No
1	Mainly fire	Yes	No	Yes	No	Yes	No	No
1	Mainly fire	Yes	No	Yes	No	Yes	No	No
	Mainly fire	Yes	No	No	No	No	No	No
	Mainly fire	No	No	No	No	Yes	No	No
1.5	Crop residu	Yes	No	No	Yes	Yes	No	No
4	Mainly fire	Yes	No	No	No	No	No	No
1	Mainly fire	Yes	No	No	No	Yes	No	No
	Mainly fire	Yes	No	Yes	No	Yes	No	No
1	Mainly fire	Yes	No	No	No	Yes	No	No
	Mainly fire	Yes	No	No	No	Yes	No	No
2	Crop residu	Yes	No	No	No	Yes	No	No
	Mainly fire	Yes	No	No	No	Yes	No	No
1	Mainly fire	Yes	No	Yes	No	Yes	No	No
1	Mainly fire	Yes	No	No	No	Yes	No	No
	Mainly fire	Yes	No	No	No	Yes	No	No
1	Mainly fire	Yes	No	No	No	Yes	No	No
1	Mainly fire	Yes	No	No	No	Yes	No	No
	Mainly fire	Yes	No	No	No	Yes	No	No
1	Mainly fire	Yes	No	No	No	No	No	No
	Mainly fire	Yes	No	No	No	No	No	No
0.5	Mainly fire	No	No	No	No	Yes	No	No
	Mainly fire	No	No	No	No	Yes	No	No
	Mainly fire	Yes	No	Yes	No	Yes	No	No
	Mainly fire	Yes	No	No	No	No	No	No
	Mainly fire	Yes	No	No	No	No	No	No
	Mainly fire	Yes	No	No	No	No	No	No
0.5	Mainly fire	Yes	No	No	No	No	No	No
0.75	Mainly fire	Yes	No	No	No	Yes	No	No
	Mainly fire	No	No	No	No	Yes	No	No
	Mainly fire	Yes	No	No	No	Yes	No	No
1	Mainly fire	Yes	No	No	No	Yes	No	No
2	Mainly fire	Yes	No	No	No	Yes	No	No
1	Mainly fire	Yes	No	No	No	No	No	No
0.5	Mainly fire	Yes	No	No	No	No	No	No
1	Mainly fire	Yes	No	No	No	Yes	No	No
	Mainly fire	Yes	No	No	No	Yes	No	No
	Mainly fire	Yes	No	No	No	Yes	No	No
1	Mainly fire	No	No	No	No	No	No	No
0.5	Mainly fire	Yes	No	No	No	No	No	No
	Mainly fire	Yes	No	No	No	Yes	No	No
	Mainly fire	Yes	No	No	No	No	No	No
1	Crop residu	No	No	No	No	No	No	No
	Mainly fire	Yes	No	No	No	Yes	No	No
0.5	Mainly fire	Yes	No	No	No	No	No	No
1	Mainly fire	Yes	No	No	No	No	No	No

For peer review only

1	Mainly fire	Yes	No	No	Yes	Yes	No	No
	Mainly fire	Yes	No	No	No	Yes	No	No
0.5	Mainly fire	No	No	No	No	No	No	No
1	Mainly fire	Yes	No	No	No	Yes	No	No
1	Mainly fire	No	No	No	No	Yes	No	No
	Mainly fire	Yes	No	No	No	No	No	No
	Mainly fire	Yes	No	No	No	Yes	No	No
	Mainly fire	Yes	No	No	No	No	No	No
0.5	Mainly fire	Yes	No	No	No	Yes	No	No
2	Crop residu	Yes	No	No	No	Yes	No	No
	Mainly fire	Yes	No	No	No	Yes	No	No
	Mainly fire	No	No	No	No	Yes	No	No
	Mainly fire	Yes	No	Yes	No	Yes	No	No
1	Mainly fire	Yes	No	No	No	Yes	No	No
1	Crop residu	Yes	No	No	No	No	No	No
	Mainly fire	No	No	No	No	No	No	No
1	Crop residu	Yes	No	No	No	Yes	No	No
1.5	Mainly fire	Yes	No	No	No	Yes	No	No
1	Mainly fire	Yes	No	No	No	Yes	No	No
0.5	Mainly fire	Yes	No	No	No	No	No	No
1	Mainly fire	Yes	No	No	No	Yes	No	No
0.25	Mainly fire	Yes	No	No	No	Yes	No	No
1.5	Mainly fire	No	No	No	No	No	No	No
0.5	Mainly fire	Yes	No	Yes	No	No	No	No
1.5	Mainly fire	No	No	No	No	Yes	No	No
1.7	Mainly fire	Yes	No	Yes	No	Yes	No	No
1	Mainly fire	Yes	No	No	No	No	No	No
0.5	Mainly fire	Yes	No	No	No	No	No	No
0	Crop residu	Yes	No	No	No	Yes	No	No
	Mainly fire	Yes	No	No	Yes	Yes	No	No
	Mainly fire	Yes	No	No	No	No	No	No
0.5	Mainly fire	Yes	No	Yes	No	No	No	No
2	Mainly fire	Yes	No	No	No	Yes	No	No
	Mainly fire	No	No	No	No	Yes	No	No
	Mainly fire	Yes	No	Yes	No	No	No	No
1	Mainly fire	Yes	No	No	No	No	No	No
1	Mainly fire	Yes	No	No	No	Yes	No	No
	Mainly fire	No	No	No	No	No	No	No
1	Mainly fire	Yes	No	No	No	Yes	No	No
	Charcoal	Yes	No	No	No	Yes	No	No
1	Mainly fire	Yes	No	No	No	Yes	No	No
	Mainly fire	Yes	No	No	No	Yes	No	No
0.5	Mainly fire	Yes	No	No	No	Yes	No	No
	Mainly fire	Yes	No	Yes	Yes	Yes	No	Yes
	Mainly fire	Yes	No	No	Yes	Yes	No	No
	Mainly fire	Yes	No	Yes	Yes	Yes	No	No
	Mainly fire	Yes	Yes	Yes	Yes	Yes	No	No
0.5	Mainly fire	Yes	No	No	Yes	Yes	No	No
	Mainly fire	Yes	No	No	No	Yes	No	No
0.5	Mainly fire	Yes	No	No	Yes	Yes	No	No
	Charcoal	Yes	No	No	No	Yes	No	No
	Mainly fire	Yes	No	No	No	Yes	No	No

For peer review only

	Mainly fire	Yes	No	No	Yes	Yes	No	No
99	Mainly fire	Yes	No	Yes	No	Yes	No	No
	Mainly fire	Yes	No	No	No	Yes	No	No
3	Mainly fire	Yes	Yes	No	Yes	Yes	No	No
2	Animal dur	Yes	Yes	Yes	Yes	Yes	No	No
0.375	Charcoal	Yes	No	Yes	No	Yes	No	No
	Charcoal	Yes	Yes	Yes	Yes	Yes	No	No
0.5	Mainly fire	Yes	No	No	Yes	Yes	No	No
1	Mainly fire	Yes	No	Yes	No	Yes	No	No
	Mainly fire	Yes	No	No	Yes	Yes	No	No
	Animal dur	Yes	Yes	Yes	Yes	Yes	No	No
	Mainly fire	Yes	No	Yes	No	Yes	No	No
	Mainly fire	Yes	No	Yes	Yes	Yes	No	No
	Mainly fire	Yes	No	Yes	Yes	No	No	No
0.25	Mainly fire	Yes	No	Yes	No	No	No	No
	Mainly fire	Yes	No	Yes	Yes	Yes	No	No
1	Mainly fire	Yes	No	Yes	No	No	No	No
	Mainly fire	Yes	No	No	No	Yes	No	No
1.5	Mainly fire	Yes	No	Yes	Yes	Yes	No	No
	Mainly fire	Yes	No	Yes	No	Yes	No	No
	Mainly fire	Yes	No	No	No	No	No	No
1	Mainly fire	Yes	No	Yes	Yes	Yes	No	No
	Mainly fire	Yes	No	Yes	No	Yes	No	No
1	Mainly fire	Yes	Yes	Yes	Yes	Yes	No	No
	Mainly fire	Yes	No	Yes	No	Yes	No	No
	Mainly fire	Yes	No	No	No	Yes	No	No
	Mainly fire	Yes	No	No	Yes	Yes	No	No
0.25	Mainly fire	No	No	No	No	No	No	No
	Mainly fire	Yes	No	Yes	No	Yes	No	No
2	Mainly fire	Yes	No	No	Yes	Yes	No	No
	Mainly fire	No	No	Yes	No	Yes	No	No
	Mainly fire	Yes	No	Yes	No	Yes	No	No
1	Mainly fire	Yes	No	Yes	Yes	Yes	No	No
	Mainly fire	Yes	No	Yes	Yes	Yes	Yes	Yes
	Mainly fire	Yes	No	Yes	No	Yes	No	No
	Mainly fire	Yes	No	Yes	No	Yes	No	No
	Mainly fire	Yes	No	Yes	No	Yes	No	No
	Mainly fire	Yes	Yes	Yes	Yes	Yes	No	No
1	Mainly fire	Yes	No	Yes	Yes	Yes	No	No
	Mainly fire	Yes	No	Yes	No	Yes	No	No
1	Mainly fire	Yes	No	Yes	Yes	Yes	No	No
1	Mainly fire	Yes	No	Yes	Yes	Yes	No	No
1	Mainly fire	Yes	No	Yes	No	Yes	No	No
1.5	Mainly fire	Yes	No	Yes	Yes	Yes	No	No
0.5	Charcoal	Yes	Yes	No	No	Yes	No	No
1	Mainly fire	Yes	No	Yes	No	Yes	No	No
1	Animal dur	Yes	No	Yes	Yes	Yes	No	No
	Mainly fire	No	No	No	No	Yes	No	No
1	Mainly fire	Yes	No	Yes	Yes	Yes	No	No
	Mainly fire	Yes	No	Yes	Yes	Yes	No	No
0.5	Charcoal	Yes	Yes	No	No	Yes	No	No
1	Mainly fire	Yes	No	Yes	Yes	Yes	No	No

For peer review only

	Animal dur	Yes	No	Yes	Yes	Yes	No	No
1.5	Mainly fire	Yes	No	Yes	Yes	Yes	No	No
	Charcoal	Yes	No	No	No	No	No	No
1	Mainly fire	Yes	No	Yes	Yes	Yes	No	No
0.5	Charcoal	Yes	No	No	Yes	Yes	No	No
	Mainly fire	Yes	No	Yes	Yes	Yes	No	No
1	Mainly fire	Yes	No	No	No	No	No	No
	Mainly fire	Yes	Yes	Yes	Yes	Yes	No	No
	Electricity	Yes	No	No	Yes	Yes	No	No
1.25	Mainly fire	Yes	No	No	Yes	Yes	No	No
1	Mainly fire	Yes	No	Yes	Yes	Yes	No	No
	Charcoal	Yes	No	Yes	Yes	Yes	No	No
	Mainly fire	Yes	No	No	Yes	Yes	No	No
3	Mainly fire	Yes	No	Yes	No	Yes	No	No
0.5	Mainly fire	Yes	Yes	Yes	Yes	Yes	No	No
1	Mainly fire	Yes	No	Yes	Yes	Yes	No	No
0.5	Mainly fire	Yes	Yes	Yes	Yes	Yes	No	No
1	Animal dur	Yes	Yes	Yes	No	Yes	No	No
0.25	Mainly fire	Yes	No	No	Yes	Yes	No	No
0.25	Mainly fire	Yes	Yes	Yes	Yes	Yes	No	Yes
	Mainly fire	Yes	No	No	Yes	Yes	No	No
0.75	Mainly fire	Yes	Yes	Yes	No	Yes	No	No
0.75	Charcoal	Yes	No	Yes	Yes	Yes	No	No
1	Electricity	Yes	No	Yes	Yes	Yes	No	No
1	Mainly fire	Yes	No	No	Yes	Yes	No	No
1	Mainly fire	Yes	No	No	Yes	Yes	No	No
2	Crop residu	Yes	Yes	No	Yes	Yes	No	Yes
	Mainly fire	Yes	No	Yes	No	Yes	No	No
1	Mainly fire	Yes	No	No	No	No	No	No
	Mainly fire	Yes	No	No	No	Yes	No	No
0.75	Crop residu	Yes	No	No	No	Yes	No	No
1	Mainly fire	No	No	No	No	Yes	Yes	No
0.5	Mainly fire	No	No	No	No	Yes	No	No
1.5	Mainly fire	Yes	No	No	No	No	No	No
	Mainly fire	Yes	No	No	No	Yes	No	No
0.75	Mainly fire	No	Yes	No	No	Yes	No	No
0.5	Mainly fire	Yes	No	No	No	Yes	No	No
	Mainly fire	Yes	No	No	No	Yes	No	No
	Mainly fire	Yes	No	No	No	Yes	No	No
1	Mainly fire	No	No	No	No	Yes	No	No
3	Mainly fire	Yes	No	Yes	No	Yes	No	No
2	Mainly fire	Yes	No	No	No	Yes	No	No
	Mainly fire	No	No	No	No	Yes	No	No
0.5	Mainly fire	Yes	No	No	No	Yes	No	No
	Mainly fire	Yes	No	Yes	No	Yes	No	No
	Mainly fire	Yes	No	No	Yes	Yes	No	No
4	Mainly fire	Yes	No	No	Yes	Yes	No	No
1	Mainly fire	No	No	Yes	No	No	No	No
1.5	Mainly fire	Yes	No	No	No	Yes	No	No
	Mainly fire	Yes	No	No	Yes	Yes	No	No
1	Mainly fire	Yes	No	No	No	Yes	No	No
0.75	Mainly fire	Yes	No	No	No	No	No	No

For peer review only

	Mainly fire	Yes	No	No	No	Yes	No	No
1	Mainly fire	No	No	No	No	Yes	No	No
3	Crop residu	Yes	No	No	No	No	No	No
	Mainly fire	Yes	No	No	No	Yes	No	No
1	Mainly fire	Yes	No	No	No	Yes	No	No
1.5	Mainly fire	Yes	No	Yes	No	Yes	No	No
1	Mainly fire	Yes	No	No	No	No	No	No
1	Mainly fire	Yes	No	No	No	Yes	No	No
3	Mainly fire	Yes	No	No	No	Yes	No	No
1	Mainly fire	Yes	No	No	No	Yes	No	No
1	Mainly fire	Yes	No	No	No	Yes	No	No
	Mainly fire	Yes	No	No	No	Yes	No	No
3	Mainly fire	Yes	No	No	No	Yes	No	No
3	Mainly fire	Yes	No	No	No	Yes	No	No
2	Crop residu	Yes	No	No	No	Yes	No	No
	Mainly fire	Yes	No	No	No	Yes	No	No
	Mainly fire	Yes	No	No	No	Yes	No	No
	Mainly fire	Yes	No	No	No	Yes	No	No
1	Mainly fire	Yes	No	No	No	Yes	No	No
1	Mainly fire	Yes	No	No	No	Yes	No	No
1.25	Mainly fire	Yes	Yes	No	No	Yes	No	No
	Mainly fire	Yes	No	Yes	No	Yes	No	No
4	Mainly fire	Yes	Yes	Yes	No	Yes	No	No
	Mainly fire	No	No	Yes	No	Yes	No	No
1	Mainly fire	Yes	No	No	No	Yes	No	No
	Mainly fire	Yes	No	Yes	No	Yes	No	No
1	Mainly fire	Yes	No	No	No	No	No	No
	Mainly fire	Yes	No	Yes	No	Yes	No	No
1	Mainly fire	Yes	No	Yes	No	Yes	No	No
1.5	Mainly fire	No	No	No	No	Yes	No	No
0.75	Mainly fire	No	No	No	No	No	No	No
1	Mainly fire	Yes	No	No	No	Yes	No	No
	Mainly fire	Yes	No	No	No	Yes	No	No
	Mainly fire	Yes	No	No	No	Yes	No	No
1	Mainly fire	Yes	No	No	No	Yes	No	No
1.5	Mainly fire	Yes	No	No	No	Yes	No	No
	Mainly fire	No	No	No	No	Yes	No	No
2	Mainly fire	Yes	No	Yes	No	Yes	No	No
	Mainly fire	Yes	No	Yes	No	Yes	No	No
	Mainly fire	Yes	No	No	No	No	No	No
0.5	Mainly fire	Yes	No	Yes	No	Yes	No	No
2	Crop residu	Yes	No	No	No	Yes	No	No
0.5	Mainly fire	Yes	No	No	No	No	No	No
	Mainly fire	Yes	No	No	No	Yes	No	No
1	Mainly fire	Yes	No	Yes	No	Yes	No	No
0.5	Mainly fire	Yes	No	No	No	Yes	No	No
1	Crop residu	Yes	No	No	No	Yes	No	No
2	Crop residu	Yes	No	No	No	Yes	No	No
1	Mainly fire	Yes	No	No	No	Yes	No	No
0.5	Mainly fire	Yes	No	No	No	Yes	No	No
1	Mainly fire	Yes	No	No	No	Yes	No	No
1	Crop residu	Yes	No	Yes	No	Yes	No	No

For peer review only

	Mainly fire	Yes	No	No	Yes	Yes	No	Yes
	Mainly fire	No	No	No	No	No	No	No
	Mainly fire	Yes	No	No	No	Yes	No	No
1	Mainly fire	Yes	No	No	No	Yes	No	No
1	Mainly fire	Yes	No	No	No	Yes	No	No
1	Mainly fire	Yes	No	No	Yes	Yes	No	No
	Mainly fire	Yes	No	No	No	Yes	No	No
	Mainly fire	Yes	No	No	No	Yes	No	No
1	Mainly fire	Yes	No	Yes	No	Yes	No	No
2	Mainly fire	No	Yes	No	No	No	No	No
1	Crop residu	Yes	No	No	No	Yes	No	No
2.5	Mainly fire	Yes	No	No	No	Yes	No	No
2.5	Mainly fire	Yes	No	No	No	Yes	No	No
	Mainly fire	Yes	No	No	No	Yes	No	No
0.5	Mainly fire	Yes	No	No	No	Yes	No	No
	Mainly fire	Yes	No	No	No	No	No	No
	Mainly fire	Yes	No	No	No	No	No	No
0.5	Mainly fire	Yes	No	No	No	Yes	No	No
	Mainly fire	No	No	No	No	Yes	No	No
	Mainly fire	Yes	No	Yes	No	Yes	No	No
1	Mainly fire	Yes	No	No	No	Yes	No	No
1	Mainly fire	Yes	No	No	No	Yes	Yes	No
0.5	Mainly fire	Yes	No	No	No	Yes	No	No
	Mainly fire	Yes	No	No	No	Yes	No	No
1	Mainly fire	No	No	No	No	Yes	No	No
0.75	Crop residu	Yes	No	No	No	Yes	No	No
0.5	Mainly fire	Yes	No	No	No	Yes	No	No
	Animal dur	Yes	No	No	No	No	No	No
1	Mainly fire	Yes	No	No	No	Yes	No	No
1	Mainly fire	No	No	No	No	No	No	No
	Mainly fire	Yes	No	No	No	Yes	No	No
2	Mainly fire	Yes	No	Yes	No	Yes	No	No
	Mainly fire	Yes	No	Yes	No	Yes	No	No
0.5	Mainly fire	Yes	No	No	No	Yes	No	No
	Mainly fire	Yes	No	No	No	Yes	No	No
	Mainly fire	Yes	No	No	No	Yes	No	No
1	Mainly fire	Yes	No	No	No	Yes	No	No
	Mainly fire	Yes	No	No	No	No	No	No
2	Mainly fire	Yes	No	Yes	No	Yes	No	No
	Mainly fire	Yes	No	No	No	Yes	No	No
1.5	Mainly fire	No	No	No	No	No	No	No
1	Mainly fire	Yes	No	Yes	No	Yes	Yes	No
0.75	Mainly fire	Yes	No	No	No	Yes	No	No
1.5	Crop residu	No	No	No	No	Yes	No	No
	Mainly fire	Yes	No	No	No	No	No	No
	Mainly fire	Yes	No	No	No	Yes	No	No
0.7	Mainly fire	Yes	No	No	No	No	No	No
	Mainly fire	Yes	No	Yes	No	Yes	No	No
0.5	Mainly fire	Yes	No	No	No	Yes	No	No
1	Mainly fire	Yes	No	No	No	Yes	No	No
0.75	Mainly fire	Yes	No	No	No	Yes	No	No
1.5	Crop residu	Yes	No	No	No	Yes	No	No

For peer review only

For peer review only

1.5	Mainly fire	Yes	No	No	No	No	No	No
0.5	Mainly fire	Yes	No	No	No	Yes	No	No
	Mainly fire	Yes	No	No	No	Yes	No	No
	Mainly fire	Yes	No	No	No	Yes	No	No
2	Mainly fire	Yes	No	No	No	Yes	No	No
999	Mainly fire	Yes	No	No	Yes	Yes	No	No
3	Crop residu	No	No	No	No	Yes	No	No
	Mainly fire	Yes	No	Yes	No	Yes	No	No
0	Crop residu	Yes	No	Yes	No	Yes	No	No
2.5	Mainly fire	Yes	No	No	Yes	Yes	No	No
1.1	Mainly fire	No	No	No	No	No	No	No
	Mainly fire	Yes	No	No	No	Yes	No	No
	Mainly fire	Yes	No	No	No	No	No	No
0.25	Mainly fire	Yes	No	Yes	No	Yes	No	No
	Mainly fire	Yes	No	Yes	Yes	Yes	No	Yes
	Mainly fire	Yes	No	No	No	Yes	No	No
0.5	Mainly fire	Yes	No	No	No	Yes	No	No
2	Mainly fire	Yes	No	Yes	No	Yes	No	No
	Mainly fire	Yes	No	No	No	Yes	No	No
2	Crop residu	Yes	No	No	No	Yes	No	No
1	Mainly fire	Yes	No	No	No	Yes	No	No
3	Mainly fire	No	No	No	No	Yes	No	No
	Mainly fire	Yes	No	No	No	Yes	No	No
2.5	Mainly fire	Yes	No	No	No	Yes	No	No
1.5	Mainly fire	Yes	No	No	No	Yes	No	No
	Mainly fire	Yes	No	No	No	No	No	No
1	Mainly fire	Yes	No	No	No	Yes	No	No
1	Mainly fire	Yes	No	No	No	Yes	No	No
1	Mainly fire	No	No	No	No	Yes	No	No
	Mainly fire	Yes	No	No	No	No	No	No
	Mainly fire	Yes	No	No	Yes	Yes	No	No
0.25	Mainly fire	Yes	No	No	No	Yes	No	No
	Mainly fire	Yes	No	Yes	No	Yes	No	No
1	Mainly fire	Yes	No	No	No	Yes	No	No
1	Mainly fire	Yes	No	Yes	No	Yes	No	No
0.5	Mainly fire	Yes	No	No	No	Yes	No	No
	Mainly fire	Yes	No	No	No	Yes	No	No
2	Mainly fire	Yes	No	No	No	Yes	No	No
0.5	Mainly fire	Yes	No	No	No	Yes	No	No
1	Mainly fire	Yes	No	Yes	No	Yes	No	No
2.5	Mainly fire	Yes	No	No	No	Yes	No	No
	Mainly fire	Yes	No	No	No	Yes	No	No
0.75	Mainly fire	Yes	No	No	No	Yes	No	No
1	Mainly fire	Yes	No	No	Yes	Yes	No	No
	Mainly fire	No	No	No	No	Yes	No	No
	Mainly fire	Yes	No	No	Yes	Yes	No	No
2.25	Mainly fire	Yes	No	No	No	Yes	No	No
	Mainly fire	Yes	No	Yes	No	Yes	No	No
1	Mainly fire	Yes	No	No	Yes	Yes	No	No
0.5	Mainly fire	Yes	No	No	No	Yes	No	No
0.5	Mainly fire	Yes	No	Yes	No	Yes	No	No
0.75	Mainly fire	Yes	No	No	No	Yes	No	No

0.5	Mainly fire	Yes	No	No	No	Yes	No	No
1	Mainly fire	Yes	No	Yes	No	Yes	No	No
	Mainly fire	Yes	No	No	No	Yes	No	No
	Charcoal	Yes	No	No	No	Yes	No	Yes
1	Mainly fire	Yes	No	Yes	Yes	Yes	No	No
0.5	Mainly fire	Yes	No	Yes	No	Yes	No	No
1	Mainly fire	Yes	No	No	Yes	Yes	No	No
1	Crop residu	Yes	No	No	No	Yes	No	No
1	Mainly fire	Yes	No	No	No	Yes	No	No
1	Mainly fire	Yes	No	No	No	Yes	No	No
6	Mainly fire	Yes	No	No	No	Yes	No	No
	Mainly fire	Yes	No	No	No	Yes	No	No
1	Mainly fire	Yes	No	No	No	Yes	No	No
0.75	Mainly fire	No	No	No	No	Yes	No	No
1	Mainly fire	Yes	No	No	No	Yes	No	No
	Mainly fire	Yes	No	No	No	Yes	No	No
	Mainly fire	Yes	No	Yes	No	Yes	No	No
1.5	Mainly fire	Yes	No	Yes	Yes	Yes	No	No
1	Mainly fire	Yes	No	No	No	Yes	No	No
0.5	Mainly fire	Yes	No	Yes	No	Yes	No	No
	Mainly fire	Yes	No	No	Yes	Yes	No	No
0.5	Mainly fire	Yes	No	No	No	Yes	No	No
0.75	Mainly fire	Yes	No	No	No	Yes	No	No
	Mainly fire	Yes	No	No	No	Yes	No	No
1	Mainly fire	Yes	No	No	Yes	Yes	No	No
	Mainly fire	Yes	No	No	No	Yes	No	No
0.5	Mainly fire	Yes	No	No	Yes	Yes	Yes	No
	Mainly fire	Yes	No	No	Yes	Yes	No	No
1	Mainly fire	Yes	No	No	No	No	No	No
0.75	Mainly fire	Yes	No	No	No	Yes	No	No
1	Mainly fire	Yes	No	No	No	No	No	No
1	Mainly fire	Yes	No	No	No	Yes	No	No
	Mainly fire	Yes	No	Yes	No	Yes	No	No
1	Mainly fire	Yes	No	No	No	Yes	No	No
	Mainly fire	Yes	No	No	No	No	No	No
1.5	Mainly fire	Yes	Yes	Yes	Yes	Yes	No	No
3	Mainly fire	Yes	No	Yes	No	Yes	No	No
	Mainly fire	No	No	No	No	Yes	No	No
1	Mainly fire	Yes	No	No	No	Yes	No	No
	Mainly fire	Yes	No	No	No	Yes	No	No
0.5	Mainly fire	Yes	No	No	No	Yes	No	No
1	Mainly fire	Yes	No	No	No	Yes	No	No
1	Mainly fire	No	No	No	No	Yes	No	No
	Mainly fire	Yes	No	No	Yes	Yes	No	No
1	Mainly fire	Yes	No	Yes	Yes	Yes	No	No
1	Mainly fire	Yes	No	No	No	No	No	No
	Mainly fire	Yes	No	No	No	Yes	No	No
	Mainly fire	Yes	No	Yes	No	Yes	No	No
	Mainly fire	Yes	No	No	No	Yes	No	No
1.25	Mainly fire	Yes	No	No	Yes	Yes	No	No
1	Mainly fire	Yes	No	No	No	Yes	No	No
1	Mainly fire	Yes	No	No	No	Yes	No	No

For peer review only

1	Mainly fire	Yes	No	No	No	Yes	No	No
2	Mainly fire	Yes	No	Yes	No	Yes	No	No
1	Mainly fire	Yes	No	No	No	No	No	No
2	Mainly fire	No	No	No	No	Yes	No	No
	Mainly fire	Yes	No	No	No	Yes	No	No
0.75	Mainly fire	Yes	No	No	No	Yes	No	No
	Mainly fire	Yes	No	No	No	No	No	No
1.5	Mainly fire	Yes	No	No	No	Yes	No	No
0.5	Mainly fire	No	No	Yes	No	No	No	No
	Mainly fire	No	No	No	No	Yes	No	No
1	Mainly fire	Yes	No	No	No	Yes	No	No
	Mainly fire	Yes	No	Yes	Yes	No	No	Yes
1	Mainly fire	Yes	No	No	No	Yes	No	No
0.5	Mainly fire	Yes	No	No	No	Yes	No	No
1	Mainly fire	Yes	No	No	No	Yes	No	No
2	Mainly fire	Yes	No	No	Yes	Yes	No	No
	Mainly fire	Yes	No	No	No	Yes	No	No
	Mainly fire	Yes	No	No	No	Yes	No	No
1.5	Mainly fire	No	No	No	No	Yes	No	No
	Mainly fire	Yes	No	Yes	Yes	Yes	No	No
0.5	Mainly fire	Yes	No	No	No	Yes	No	No
1	Crop residu	Yes	No	No	No	Yes	No	No
0.5	Mainly fire	Yes	No	No	No	Yes	No	No
1	Mainly fire	Yes	No	No	No	Yes	No	No
2	Mainly fire	Yes	No	No	No	Yes	No	No
0.5	Mainly fire	Yes	No	No	No	Yes	No	No
1	Mainly fire	Yes	No	Yes	No	No	No	No
0.5	Mainly fire	Yes	No	No	Yes	Yes	No	No
	Mainly fire	Yes	No	No	No	Yes	No	No
1	Mainly fire	Yes	No	No	Yes	Yes	No	No
1	Mainly fire	Yes	No	Yes	No	Yes	No	No
	Mainly fire	Yes	No	No	Yes	Yes	No	No
	Mainly fire	Yes	No	No	Yes	Yes	No	No
0.5	Mainly fire	Yes	No	Yes	No	Yes	No	No
	Mainly fire	Yes	No	No	No	Yes	No	No
	Mainly fire	Yes	No	No	No	Yes	No	No
1	Mainly fire	Yes	No	No	Yes	Yes	No	No
	Mainly fire	Yes	No	No	No	Yes	No	No
1	Mainly fire	Yes	No	No	No	Yes	No	No
1.5	Mainly fire	Yes	Yes	No	No	Yes	No	No
0.75	Mainly fire	Yes	No	No	No	Yes	No	No
	Mainly fire	Yes	No	No	No	Yes	No	No
1	Mainly fire	Yes	No	No	No	No	No	No
0.5	Mainly fire	Yes	No	Yes	Yes	No	No	No
	Mainly fire	Yes	No	No	Yes	Yes	No	No
	Mainly fire	Yes	No	No	No	Yes	No	No
	Mainly fire	Yes	No	No	No	Yes	No	No
	Mainly fire	Yes	No	No	No	Yes	No	No
2	Mainly fire	Yes	No	No	Yes	Yes	No	No
4	Mainly fire	Yes	No	No	No	Yes	No	No
1	Mainly fire	Yes	No	No	No	Yes	No	No
1.2	Mainly fire	Yes	No	No	No	Yes	No	No

For peer review only

0.5	Mainly fire	Yes	No	No	No	No	No	No
1	Mainly fire	Yes	No	No	No	Yes	No	No
1	Mainly fire	No	No	No	No	No	No	No
	Mainly fire	Yes	No	No	No	Yes	No	No
1	Mainly fire	Yes	No	No	No	Yes	No	No
	Mainly fire	Yes	No	No	No	Yes	No	No
	Mainly fire	Yes	No	No	No	Yes	No	No
1	Mainly fire	No	No	No	No	Yes	No	No
1.5	Crop residu	Yes	No	No	No	Yes	No	No
1	Mainly fire	Yes	No	No	No	Yes	No	No
	Mainly fire	Yes	No	No	No	No	No	No
	Mainly fire	Yes	No	No	No	Yes	No	No
	Mainly fire	Yes	No	No	No	Yes	No	No
0.5	Crop residu	Yes	No	No	No	Yes	No	No
	Mainly fire	Yes	No	No	No	Yes	No	No
0.5	Mainly fire	Yes	No	No	No	No	No	No
	Mainly fire	No	No	No	No	No	No	No
1.2	Mainly fire	Yes	No	No	No	Yes	No	No
	Mainly fire	Yes	No	No	No	Yes	No	No
2	Mainly fire	Yes	No	No	No	No	No	No
0.5	Mainly fire	Yes	No	No	No	Yes	No	No
1	Mainly fire	Yes	No	No	No	Yes	No	No
4	Mainly fire	No	No	No	No	No	No	No
4	Mainly fire	Yes	No	Yes	No	Yes	No	No
0.5	Mainly fire	Yes	No	No	No	No	No	No
0.5	Mainly fire	No	No	Yes	No	Yes	No	No
1	Mainly fire	Yes	No	No	Yes	Yes	No	No
2	Mainly fire	Yes	No	No	No	Yes	No	No
	Mainly fire	Yes	No	No	No	No	No	No
1.5	Mainly fire	Yes	No	Yes	No	Yes	No	No
0.5	Mainly fire	Yes	No	No	No	Yes	No	No
5	Mainly fire	Yes	No	No	No	Yes	No	No
1.2	Mainly fire	Yes	No	No	No	No	No	No
	Mainly fire	Yes	No	No	No	Yes	No	No
	Mainly fire	Yes	No	No	No	No	No	No
	Mainly fire	Yes	No	No	No	Yes	No	No
5	Mainly fire	Yes	No	No	No	No	No	No
1	Mainly fire	Yes	No	No	No	Yes	No	No
1	Mainly fire	Yes	No	No	No	Yes	No	No
0.5	Mainly fire	No	No	No	No	Yes	No	No
0.7	Mainly fire	Yes	No	Yes	No	Yes	No	No
4	Mainly fire	Yes	No	Yes	No	Yes	No	No
	Mainly fire	Yes	No	No	No	Yes	No	No
	Mainly fire	Yes	No	No	No	No	No	No
1	Mainly fire	Yes	No	No	No	No	No	No
1	Mainly fire	Yes	No	No	No	Yes	No	No
1.5	Crop residu	Yes	No	No	No	Yes	No	No
	Mainly fire	Yes	No	No	No	Yes	No	Yes
4	Mainly fire	Yes	No	No	No	Yes	No	No
	Mainly fire	No	No	No	No	No	No	No
0.75	Mainly fire	Yes	No	No	No	Yes	No	No
6	Mainly fire	Yes	Yes	No	No	Yes	No	No

For peer review only

	Mainly fire	No	No	No	No	No	No	No
1	Mainly fire	No	No	No	No	No	No	No
5	Mainly fire	Yes	No	No	No	Yes	No	No
	Mainly fire	Yes	No	No	No	Yes	No	No
1	Crop residu	Yes	No	No	No	Yes	No	No
1	Mainly fire	Yes	No	Yes	No	Yes	No	No
5	Mainly fire	Yes	No	Yes	No	Yes	No	No
1	Mainly fire	Yes	No	No	No	Yes	No	No
0.7	Mainly fire	No	No	No	No	No	No	No
1.25	Crop residu	Yes	No	No	No	Yes	No	No
1	Mainly fire	Yes	No	No	No	Yes	No	No
	Mainly fire	Yes	No	No	No	No	No	No
5	Mainly fire	No	No	No	No	Yes	No	No
2	Mainly fire	Yes	No	No	No	Yes	No	No
4	Mainly fire	Yes	No	No	No	Yes	No	No
1	Mainly fire	Yes	No	No	No	No	No	No
1	Mainly fire	Yes	No	No	No	Yes	No	No
0.5	Mainly fire	Yes	No	Yes	No	Yes	No	No
	Mainly fire	Yes	No	No	No	Yes	No	No
	Mainly fire	No	No	No	No	Yes	No	No
1	Mainly fire	No	No	No	No	Yes	No	No
	Mainly fire	Yes	No	No	No	Yes	No	No
	Mainly fire	Yes	No	No	No	Yes	No	No
	Mainly fire	Yes	No	No	No	Yes	No	No
1	Mainly fire	No	No	No	No	Yes	No	No
	Mainly fire	Yes	No	No	No	Yes	No	No
	Mainly fire	Yes	No	No	No	Yes	No	No
0.5	Mainly fire	Yes	No	Yes	No	Yes	No	No
	Mainly fire	No	No	No	No	No	No	No
4	Mainly fire	No	No	No	No	Yes	No	No
4	Mainly fire	Yes	Yes	No	No	Yes	No	No
2	Mainly fire	Yes	No	No	No	Yes	No	No
5	Mainly fire	No	No	No	No	Yes	No	No
	Mainly fire	Yes	No	No	No	No	No	No
1	Mainly fire	Yes	No	No	No	No	No	No
	Mainly fire	Yes	No	No	No	Yes	No	No
2	Mainly fire	Yes	No	No	No	Yes	No	No
	Mainly fire	No	No	No	No	Yes	No	No
4	Mainly fire	Yes	No	No	No	No	No	No
	Mainly fire	Yes	No	No	No	Yes	No	No
1	Mainly fire	Yes	No	No	No	Yes	No	No
	Mainly fire	Yes	No	No	No	Yes	No	No
1	Mainly fire	No	No	No	Yes	No	No	No
1	Mainly fire	Yes	No	No	No	Yes	No	No
	Mainly fire	No	No	No	No	Yes	No	No
4	Mainly fire	Yes	No	No	No	No	No	No
1	Mainly fire	Yes	No	No	No	Yes	No	No
1	Mainly fire	Yes	No	No	No	No	No	No
	Mainly fire	Yes	No	No	No	Yes	No	No
	Crop residu	No	No	No	No	Yes	No	No
1.2	Mainly fire	Yes	No	No	No	Yes	No	No
0.75	Mainly fire	Yes	No	No	No	Yes	No	No

For peer review only

	Mainly fire	Yes	No	No	No	No	No	No
	Mainly fire	Yes	No	No	No	No	No	No
	Mainly fire	Yes	No	No	No	Yes	No	No
1	Mainly fire	Yes	No	No	No	Yes	No	No
1	Mainly fire	Yes	No	No	No	No	No	No
3	Crop residu	Yes	No	No	No	No	No	No
1	Mainly fire	Yes	No	Yes	No	No	No	No
0.5	Mainly fire	Yes	No	No	No	No	No	No
1.2	Mainly fire	Yes	No	No	No	Yes	No	No
	Mainly fire	Yes	No	No	No	Yes	No	No
1	Mainly fire	Yes	No	No	No	No	No	No
4	Mainly fire	Yes	No	No	No	Yes	No	No
1	Mainly fire	Yes	No	No	No	No	No	No
0.25	Animal dur	Yes	Yes	Yes	No	Yes	No	No
0.5	Mainly fire	No	No	Yes	Yes	Yes	No	No
0.25	Mainly fire	No	No	Yes	No	Yes	No	No
0.25	Animal dur	Yes	No	Yes	No	Yes	No	No
0.25	Mainly fire	No	No	No	No	Yes	No	No
	Animal dur	Yes	No	Yes	Yes	Yes	No	Yes
75	Mainly fire	Yes	No	No	No	Yes	No	No
	Mainly fire	No	No	No	No	Yes	No	No
0	Mainly fire	Yes	No	No	Yes	Yes	No	No
0.2	Mainly fire	Yes	No	Yes	No	No	No	No
2	Mainly fire	Yes	Yes	No	No	Yes	No	No
0.25	Mainly fire	Yes	Yes	No	No	Yes	No	No
0.5	Mainly fire	Yes	No	No	No	Yes	No	No
	Mainly fire	Yes	No	No	No	Yes	No	No
0.25	Animal dur	Yes	No	No	No	Yes	No	No
2.5	Mainly fire	Yes	No	Yes	No	Yes	No	No
0.25	Animal dur	Yes	No	No	Yes	Yes	No	No
	Mainly fire	Yes	Yes	Yes	No	Yes	No	No
0.5	Animal dur	Yes	No	Yes	No	Yes	No	No
0.5	Mainly fire	Yes	No	Yes	Yes	Yes	No	No
0.25	Mainly fire	No	Yes	Yes	No	Yes	No	No
	Animal dur	Yes	No	Yes	No	Yes	No	No
3	Mainly fire	Yes	Yes	Yes	No	Yes	No	No
0.5	Animal dur	Yes	No	Yes	No	Yes	No	No
	Mainly fire	Yes	No	Yes	No	Yes	No	No
0.25	Mainly fire	No	No	No	No	No	No	No
0.75	Animal dur	Yes	No	No	No	Yes	No	No
0.75	Mainly fire	Yes	No	Yes	No	Yes	No	No
	Mainly fire	No	No	Yes	No	Yes	No	No
0.25	Animal dur	No	No	Yes	No	No	No	No
0.5	Mainly fire	No	No	Yes	No	Yes	No	No
1	Animal dur	Yes	No	Yes	Yes	Yes	No	No
0.75	Mainly fire	No	No	No	No	Yes	No	No
	Mainly fire	Yes	No	No	Yes	Yes	No	Yes
2.5	Mainly fire	Yes	Yes	Yes	No	Yes	No	No
0.25	Animal dur	Yes	No	Yes	No	Yes	No	No
1	Mainly fire	Yes	No	No	Yes	Yes	No	No
0	Mainly fire	Yes	Yes	Yes	Yes	Yes	No	No
	Animal dur	No	No	No	No	Yes	No	No

For peer review only

1	Mainly fire	No	No	No	No	Yes	No	No
	Animal dur	Yes	No	Yes	No	Yes	No	No
1.1	Animal dur	Yes	No	Yes	No	Yes	No	No
	Animal dur	No	No	Yes	No	Yes	No	No
0.25	Mainly fire	Yes	Yes	Yes	Yes	Yes	No	No
0.1	Animal dur	No	No	Yes	No	Yes	No	No
0.25	Mainly fire	Yes	Yes	Yes	Yes	Yes	No	No
1	Mainly fire	No	No	Yes	No	No	No	No
0.5	Mainly fire	No	No	Yes	No	No	No	No
0.2	Mainly fire	Yes	No	Yes	No	Yes	No	No
2	Mainly fire	No	No	Yes	No	Yes	No	No
0.25	Animal dur	Yes	No	Yes	Yes	Yes	No	No
2	Mainly fire	Yes	No	Yes	No	Yes	No	No
0.375	Animal dur	Yes	No	No	No	Yes	No	No
	Mainly fire	Yes	No	No	Yes	Yes	No	No
0.1	Animal dur	No	No	No	No	No	No	No
0.5	Animal dur	Yes	No	No	No	Yes	No	No
2	Mainly fire	No	No	Yes	No	Yes	No	No
0.25	Mainly fire	Yes	No	Yes	No	Yes	No	No
0.25	Animal dur	Yes	No	Yes	No	Yes	No	No
25	Mainly fire	Yes	Yes	Yes	No	Yes	No	No
	Animal dur	Yes	No	No	Yes	Yes	No	No
3	Mainly fire	Yes	No	Yes	No	Yes	No	No
0	Mainly fire	Yes	No	Yes	No	Yes	No	No
3	Animal dur	No	No	Yes	No	Yes	No	No
1	Mainly fire	Yes	No	No	No	Yes	No	No
0.25	Mainly fire	Yes	No	No	Yes	Yes	No	No
	Mainly fire	No	No	Yes	No	Yes	No	No
1	Mainly fire	Yes	No	No	No	Yes	No	No
2.5	Mainly fire	Yes	No	Yes	No	Yes	No	No
1	Mainly fire	Yes	No	No	No	Yes	No	No
0.25	Mainly fire	Yes	No	Yes	No	Yes	No	No
	Mainly fire	Yes	No	Yes	No	Yes	No	No
2	Mainly fire	Yes	No	Yes	No	Yes	No	No
0	Charcoal	Yes	No	No	No	Yes	No	No
0.2	Animal dur	No	No	No	No	Yes	No	No
0.5	Animal dur	No	Yes	Yes	No	No	No	No
	Mainly fire	Yes	No	No	Yes	Yes	No	Yes
2.5	Mainly fire	No	Yes	No	No	Yes	No	No
0.75	Animal dur	No	No	Yes	No	Yes	No	No
	Mainly fire	Yes	Yes	No	No	Yes	No	No
	Mainly fire	Yes	No	Yes	No	Yes	No	No
0	Charcoal	No	No	No	No	Yes	No	No
2	Mainly fire	Yes	No	Yes	No	Yes	No	No
	Mainly fire	No	Yes	Yes	No	Yes	No	No
0.5	Mainly fire	Yes	No	Yes	No	Yes	No	No
0.75	Mainly fire	Yes	Yes	Yes	No	Yes	No	No
0.25	Animal dur	No	No	No	No	Yes	No	No
0.5	Animal dur	No	No	Yes	No	Yes	No	No
0.25	Mainly fire	Yes	No	Yes	No	Yes	No	No
1	Animal dur	Yes	No	No	Yes	Yes	No	No
3	Mainly fire	No	No	Yes	No	Yes	No	No

For peer review only

0.5	Animal dur	Yes	No	Yes	No	Yes	No	No
4	Mainly fire	Yes	Yes	No	Yes	Yes	No	No
	Mainly fire	Yes	No	Yes	No	Yes	No	No
	Mainly fire	Yes	Yes	Yes	Yes	Yes	No	No
	Mainly fire	Yes	No	Yes	Yes	Yes	No	No
	Animal dur	No	No	No	No	Yes	No	No
3	Mainly fire	Yes	No	Yes	Yes	Yes	No	No
	Mainly fire	Yes	Yes	Yes	No	Yes	No	No
1	Mainly fire	Yes	No	Yes	No	Yes	No	No
1	Animal dur	Yes	No	Yes	No	Yes	No	No
2	Mainly fire	Yes	Yes	No	No	Yes	No	No
2	Animal dur	Yes	No	No	No	Yes	No	No
	Animal dur	No	No	No	No	Yes	No	No
0.5	Electricity	Yes	Yes	Yes	Yes	Yes	No	No
0	Charcoal	No	No	Yes	No	Yes	No	No
	Animal dur	Yes	No	Yes	Yes	Yes	No	No
0.5	Mainly fire	No	No	Yes	No	Yes	No	No
0.25	Animal dur	Yes	No	Yes	No	Yes	No	Yes
0.5	Mainly fire	Yes	No	No	No	Yes	No	No
	Animal dur	Yes	No	No	Yes	Yes	No	No
0.5	Animal dur	Yes	No	Yes	No	Yes	No	No
0.5	Mainly fire	Yes	No	Yes	Yes	Yes	No	No
	Animal dur	Yes	No	No	No	Yes	No	No
	Animal dur	No	No	Yes	No	Yes	No	No
	Animal dur	No	No	Yes	No	Yes	No	No
0.5	Mainly fire	No	No	No	No	Yes	No	No
	Mainly fire	Yes	Yes	No	Yes	Yes	No	Yes
10	Mainly fire	Yes	No	Yes	No	Yes	No	No
0	Mainly fire	No	No	No	No	Yes	No	No
0.25	Animal dur	Yes	No	No	No	Yes	No	No
	Animal dur	No	No	Yes	No	No	No	No
	Animal dur	Yes	No	Yes	No	Yes	No	No
	Mainly fire	Yes	No	Yes	No	No	No	No
0.75	Animal dur	No	No	Yes	No	No	No	No
0.5	Mainly fire	Yes	No	Yes	Yes	Yes	No	No
0.5	Animal dur	Yes	Yes	Yes	Yes	Yes	No	No
0.25	Mainly fire	Yes	Yes	Yes	No	Yes	No	No
0.5	Mainly fire	No	No	No	No	Yes	No	No
0.25	Mainly fire	Yes	No	Yes	No	Yes	No	No
0.2	Animal dur	No	No	No	No	No	No	No
1	Animal dur	No	No	Yes	No	Yes	No	No
	Animal dur	Yes	No	No	No	Yes	No	No
1	Mainly fire	Yes	Yes	No	Yes	Yes	No	No
0.25	Animal dur	No	No	Yes	No	Yes	No	No
0	Animal dur	Yes	No	Yes	No	Yes	No	No
0.1	Mainly fire	No	No	Yes	No	Yes	No	No
	Animal dur	Yes	No	Yes	No	Yes	No	No
0.5	Mainly fire	Yes	No	Yes	No	Yes	No	No
	Mainly fire	Yes	No	No	Yes	Yes	No	No
0.1	Mainly fire	No	No	Yes	No	Yes	No	No
0.25	Mainly fire	No	No	Yes	No	Yes	No	No
	Animal dur	No	No	No	No	Yes	No	No

For peer review only

1.25	Animal dur	Yes	No	Yes	No	Yes	No	No
0.5	Mainly fire	No	No	No	No	Yes	No	No
0.5	Animal dur	Yes	No	Yes	No	Yes	No	No
	Animal dur	Yes	No	No	No	No	No	No
0.5	Mainly fire	No	Yes	Yes	No	Yes	No	No
0.75	Mainly fire	No	No	Yes	No	No	No	No
0.5	Mainly fire	Yes	No	Yes	No	Yes	No	No
0.25	Mainly fire	Yes	Yes	Yes	No	Yes	No	No
	Mainly fire	Yes	Yes	Yes	Yes	Yes	No	No
0.5	Mainly fire	Yes	No	Yes	No	Yes	No	No
0	Animal dur	Yes	Yes	Yes	Yes	Yes	No	No
0.2	Animal dur	No	No	Yes	No	Yes	No	No
	Mainly fire	No	No	No	No	Yes	No	No
0.5	Animal dur	No	No	No	No	Yes	No	No
0.5	Animal dur	Yes	Yes	Yes	No	Yes	No	No
0.75	Mainly fire	Yes	No	Yes	No	Yes	No	No
0	Mainly fire	Yes	Yes	Yes	No	Yes	No	No
0.25	Animal dur	Yes	No	No	No	Yes	No	No
0.25	Mainly fire	No	No	No	No	Yes	No	No
	Mainly fire	Yes	No	No	Yes	Yes	No	No
1	Mainly fire	Yes	Yes	Yes	No	Yes	No	No
0.5	Mainly fire	No	No	Yes	Yes	Yes	No	No
	Mainly fire	Yes	Yes	Yes	No	Yes	No	No
	Mainly fire	Yes	No	No	Yes	Yes	No	Yes
0.5	Mainly fire	No	Yes	Yes	No	Yes	No	No
0.5	Animal dur	No	No	No	No	Yes	No	No
0.25	Mainly fire	Yes	No	Yes	No	Yes	No	No
1	Animal dur	Yes	No	Yes	No	Yes	No	No
	Mainly fire	Yes	Yes	Yes	Yes	Yes	No	Yes
	Animal dur	No	No	Yes	No	No	No	No
1	Mainly fire	No	Yes	Yes	No	Yes	No	No
0.5	Mainly fire	No	No	No	No	Yes	No	No
0.25	Mainly fire	Yes	No	No	No	Yes	No	No
	Mainly fire	No	No	No	No	Yes	No	Yes
0.25	Mainly fire	Yes	No	Yes	No	Yes	No	No
1.5	Animal dur	Yes	No	Yes	No	Yes	No	No
0.5	Mainly fire	Yes	No	Yes	No	Yes	No	No
999	Mainly fire	Yes	No	No	No	Yes	No	No
2	Mainly fire	Yes	No	No	No	No	No	No
1	Mainly fire	No	No	No	No	No	No	No
	Mainly fire	Yes	No	No	No	No	No	No
	Mainly fire	Yes	No	No	No	No	No	No
0.5	Mainly fire	No	No	No	No	No	No	No
	Mainly fire	Yes	No	No	No	No	No	No
	Mainly fire	No	No	No	No	No	No	No
	Mainly fire	Yes	No	No	No	No	No	No
1.5	Mainly fire	Yes	No	No	No	Yes	No	No
1	Mainly fire	Yes	No	No	No	Yes	No	No
	Mainly fire	Yes	No	No	No	No	No	No
1	Mainly fire	Yes	No	No	No	Yes	No	No
	Mainly fire	Yes	No	No	No	No	No	No
0.5	Mainly fire	Yes	No	Yes	No	No	No	No

For peer review only

0.75	Mainly fire	Yes	No	Yes	No	No	No	No
	Mainly fire	No	No	No	No	No	No	No
999	Mainly fire	Yes	No	No	No	No	No	No
	Mainly fire	No	No	No	No	No	No	No
0.25	Mainly fire	No	No	No	No	No	No	No
1	Mainly fire	Yes	No	No	No	No	No	No
0.75	Mainly fire	Yes	No	No	No	Yes	No	No
1.75	Mainly fire	Yes	No	No	No	Yes	No	No
1.75	Mainly fire	Yes	No	No	No	Yes	No	No
	Mainly fire	No	No	No	No	No	No	No
	Mainly fire	Yes	No	No	No	No	No	No
	Mainly fire	Yes	No	No	No	Yes	No	No
1.25	Mainly fire	Yes	No	No	No	No	No	No
1	Mainly fire	Yes	No	No	No	Yes	No	No
	Mainly fire	No	No	No	No	No	No	No
	Mainly fire	Yes	No	No	No	No	No	No
	Mainly fire	Yes	No	No	No	No	No	No
999	Mainly fire	Yes	No	Yes	No	No	No	No
1	Mainly fire	Yes	No	No	No	No	No	No
2	Mainly fire	No	No	No	No	Yes	No	No
	Mainly fire	No	No	No	No	No	No	No
999	Mainly fire	Yes	No	No	No	Yes	No	No
999	Mainly fire	Yes	No	No	No	No	No	No
	Mainly fire	Yes	No	No	No	No	No	No
	Mainly fire	Yes	No	No	No	No	No	No
999	Mainly fire	Yes	No	No	No	Yes	No	No
0.75	Mainly fire	Yes	No	No	No	No	No	No
1.5	Mainly fire	Yes	No	No	No	No	No	No
999	Mainly fire	No	No	No	No	No	No	No
1	Mainly fire	Yes	No	No	No	Yes	No	No
1	Mainly fire	Yes	No	No	No	Yes	No	No
	Mainly fire	Yes	No	No	No	No	No	No
	Mainly fire	Yes	No	No	No	No	No	No
0.75	Mainly fire	No	No	No	No	No	No	No
999	Mainly fire	No	No	Yes	No	No	No	No
	Mainly fire	Yes	No	No	No	No	No	No
	Mainly fire	No	No	No	No	No	No	No
1	Mainly fire	Yes	No	No	No	No	No	No
2	Mainly fire	Yes	No	No	No	Yes	No	No
0.25	Mainly fire	Yes	No	No	No	No	No	No
2	Mainly fire	Yes	No	No	No	Yes	No	No
0.25	Mainly fire	Yes	No	No	No	No	No	No
0.5	Mainly fire	Yes	No	No	No	No	No	No
0.75	Mainly fire	Yes	No	No	No	No	No	No
0.25	Mainly fire	Yes	No	No	No	No	No	No
1	Mainly fire	Yes	No	No	No	No	No	No
0.75	Mainly fire	Yes	No	No	No	No	No	No
	Mainly fire	Yes	No	No	No	Yes	No	No
0.5	Mainly fire	Yes	No	No	No	Yes	No	No
	Mainly fire	Yes	No	No	No	Yes	No	No
	Mainly fire	Yes	No	No	No	Yes	No	No
0.5	Mainly fire	Yes	No	No	No	Yes	No	No

For peer review only

	Mainly fire	Yes	No	Yes	No	Yes	No	No
	Mainly fire	No	No	No	No	No	No	No
	Mainly fire	Yes	No	Yes	No	Yes	No	No
	Mainly fire	Yes	No	No	No	Yes	No	No
2	Mainly fire	Yes	No	Yes	No	Yes	No	No
	Mainly fire	Yes	No	Yes	No	Yes	No	No
0.75	Mainly fire	No	No	No	No	Yes	No	No
0.25	Mainly fire	No	No	No	No	Yes	No	No
	Mainly fire	No	No	No	No	No	No	No
	Mainly fire	Yes	No	No	Yes	Yes	No	No
	Mainly fire	No	No	No	No	Yes	No	No
	Charcoal	Yes	No	No	No	Yes	No	No
0.25	Mainly fire	Yes	No	No	No	Yes	No	No
	Mainly fire	Yes	No	No	No	Yes	No	No
	Mainly fire	Yes	No	No	Yes	Yes	No	No
999	Mainly fire	Yes	No	Yes	No	Yes	No	No
	Mainly fire	Yes	No	No	No	No	No	No
0.5	Mainly fire	Yes	No	No	No	Yes	No	No
1	Mainly fire	Yes	No	Yes	Yes	Yes	No	No
	Mainly fire	Yes	No	Yes	No	Yes	No	No
0.25	Mainly fire	Yes	No	No	No	No	Yes	No
0.5	Mainly fire	Yes	No	No	No	No	No	No
	Mainly fire	Yes	No	No	No	Yes	No	No
	Mainly fire	Yes	No	No	Yes	Yes	No	No
1.5	Mainly fire	Yes	No	Yes	No	Yes	No	No
0.5	Mainly fire	Yes	No	No	No	Yes	No	No
	Mainly fire	Yes	No	No	No	Yes	No	No
	Mainly fire	Yes	No	No	No	Yes	No	No
	Mainly fire	Yes	No	No	No	Yes	No	No
	Mainly fire	Yes	No	No	No	Yes	No	No
	Mainly fire	Yes	No	No	No	Yes	No	No
	Charcoal	Yes	No	No	No	Yes	No	No
	Mainly fire	Yes	No	No	No	Yes	No	No
	Mainly fire	Yes	No	No	No	No	No	No
	Mainly fire	No	No	Yes	No	Yes	No	No
	Mainly fire	Yes	No	Yes	No	Yes	No	No
	Mainly fire	No	No	No	No	No	No	No
	Mainly fire	Yes	No	No	No	Yes	No	No
0.5	Mainly fire	Yes	No	Yes	No	Yes	No	No
	Mainly fire	Yes	No	Yes	No	Yes	No	No
	Mainly fire	Yes	No	No	No	No	No	No
2	Mainly fire	Yes	No	No	No	Yes	No	No
	Mainly fire	Yes	No	No	No	Yes	No	No
1.5	Mainly fire	Yes	No	No	No	Yes	No	No
	Mainly fire	Yes	No	Yes	No	Yes	No	No
	Mainly fire	No	No	No	No	Yes	No	No
	Mainly fire	Yes	No	No	No	Yes	No	No
	Mainly fire	Yes	No	No	Yes	Yes	No	No
	Mainly fire	No	No	No	No	Yes	No	No
	Mainly fire	Yes	No	No	No	Yes	No	No
1	Mainly fire	Yes	No	Yes	No	Yes	No	No
2	Mainly fire	No	No	Yes	No	Yes	No	No

For peer review only

	Mainly fire	Yes	No	No	No	No	No	No
	Mainly fire	No	No	No	No	Yes	No	No
	Mainly fire	Yes	No	No	No	No	No	No
	Mainly fire	Yes	No	No	No	Yes	No	No
	Mainly fire	Yes	No	No	No	No	No	No
	Mainly fire	Yes	No	Yes	No	Yes	No	No
	Mainly fire	No	No	No	No	Yes	No	No
	Mainly fire	Yes	Yes	No	Yes	Yes	No	No
	Mainly fire	Yes	No	No	No	Yes	No	No
1.5	Mainly fire	Yes	No	No	No	Yes	No	No
	Mainly fire	Yes	No	No	Yes	Yes	No	No
	Mainly fire	No	No	No	No	No	No	No
	Mainly fire	Yes	No	No	No	Yes	No	No
	Mainly fire	No	No	No	No	Yes	No	No
4	Mainly fire	No	No	No	No	Yes	No	No
	Mainly fire	Yes	No	No	No	No	No	No
0	Mainly fire	No	No	No	No	No	No	No
	Mainly fire	Yes	No	No	No	Yes	No	No
	Mainly fire	Yes	No	No	No	No	No	No
	Mainly fire	Yes	No	No	No	Yes	No	No
	Mainly fire	Yes	No	No	No	No	No	No
1	Mainly fire	Yes	No	Yes	No	Yes	No	No
	Charcoal	Yes	No	No	No	Yes	No	No
	Mainly fire	Yes	No	No	No	Yes	No	No
	Mainly fire	No	No	No	No	Yes	No	No
0.5	Mainly fire	Yes	No	No	No	Yes	No	No
	Mainly fire	Yes	No	Yes	Yes	Yes	No	No
	Mainly fire	Yes	No	Yes	No	No	No	No
	Mainly fire	Yes	No	Yes	No	Yes	No	No
	Mainly fire	Yes	No	No	No	No	No	No
	Mainly fire	Yes	No	No	No	Yes	No	No
	Mainly fire	No	No	No	No	Yes	No	No
1	Mainly fire	Yes	No	No	No	Yes	No	No
1	Mainly fire	Yes	No	No	No	Yes	No	No
0.5	Mainly fire	Yes	No	No	No	Yes	No	No
	Mainly fire	Yes	No	No	No	Yes	No	No
	Mainly fire	No	No	No	No	Yes	No	No
	Mainly fire	Yes	No	No	No	Yes	No	No
	Mainly fire	Yes	No	No	No	Yes	No	No
	Mainly fire	Yes	No	No	No	Yes	No	No
999	Mainly fire	Yes	No	Yes	No	Yes	No	No
0.75	Mainly fire	No	No	No	No	Yes	No	No
	Mainly fire	Yes	No	Yes	No	Yes	No	No
0	Mainly fire	Yes	No	No	No	Yes	No	No
	Mainly fire	No	No	No	No	Yes	No	No
0.5	Mainly fire	Yes	No	Yes	No	No	No	No
	Mainly fire	Yes	No	No	No	Yes	No	No
	Charcoal	Yes	No	Yes	No	Yes	No	No
	Mainly fire	Yes	No	No	Yes	Yes	No	No
	Mainly fire	No	No	No	No	Yes	No	No
	Charcoal	Yes	No	No	No	Yes	No	No
	Mainly fire	Yes	No	No	No	Yes	No	No

For peer review only

2	Mainly fire	Yes	Yes	Yes	No	Yes	No	No
0.5	Mainly fire	No	No	Yes	No	Yes	No	No
	Charcoal	No	Yes	No	No	Yes	No	No
1	Mainly fire	Yes	No	No	No	No	No	No
	Mainly fire	Yes	No	No	No	Yes	No	No
0.75	Mainly fire	Yes	No	No	No	Yes	No	No
	Mainly fire	Yes	No	No	No	Yes	No	No
	Mainly fire	Yes	No	No	No	Yes	No	No
	Mainly fire	Yes	No	No	No	Yes	No	No
0.5	Mainly fire	No	No	No	No	No	No	No
0.75	Mainly fire	Yes	No	No	No	No	No	No
	Mainly fire	Yes	No	No	No	Yes	No	No
	Mainly fire	Yes	No	No	No	Yes	No	No
	Mainly fire	Yes	No	No	No	Yes	No	No
	Mainly fire	No	No	No	No	No	No	No
	Mainly fire	Yes	No	No	No	Yes	No	No
	Mainly fire	Yes	No	No	No	No	No	No
	Mainly fire	Yes	No	Yes	Yes	Yes	No	No
	Mainly fire	Yes	No	No	No	Yes	No	No
0.5	Mainly fire	Yes	No	No	No	Yes	No	No
	Mainly fire	Yes	No	No	No	Yes	No	No
	Mainly fire	Yes	No	No	Yes	Yes	No	No
	Mainly fire	Yes	No	No	No	Yes	No	No
	Mainly fire	No	No	No	No	Yes	No	No
1	Mainly fire	Yes	No	No	No	No	No	No
1	Mainly fire	No	No	No	No	Yes	No	No
	Mainly fire	Yes	No	No	No	Yes	No	No
0	Mainly fire	No	No	No	No	No	No	No
2.5	Mainly fire	No	No	No	No	Yes	No	No
	Mainly fire	Yes	No	No	Yes	Yes	No	No
	Mainly fire	Yes	No	No	No	Yes	No	No
0.25	Mainly fire	Yes	No	Yes	No	Yes	No	No
1	Animal dur	Yes	No	Yes	No	Yes	No	No
0.25	Mainly fire	No	No	Yes	No	Yes	No	No
	Charcoal	No	No	Yes	No	Yes	No	No
0.75	Animal dur	Yes	No	Yes	No	Yes	No	No
	Charcoal	Yes	No	Yes	Yes	Yes	No	No
1.5	Animal dur	No	No	Yes	No	Yes	No	No
0.5	Mainly fire	Yes	No	Yes	No	Yes	No	No
	Animal dur	Yes	No	Yes	Yes	Yes	No	No
0.5	Animal dur	No	No	Yes	Yes	Yes	No	No
1	Mainly fire	Yes	No	Yes	No	Yes	No	No
2	Animal dur	No	No	No	No	No	No	No
0.5	Mainly fire	Yes	No	No	No	Yes	No	No
0.2	Animal dur	Yes	No	Yes	No	Yes	No	No
	Mainly fire	Yes	No	Yes	Yes	Yes	No	Yes
4	Mainly fire	Yes	No	No	Yes	Yes	No	Yes
0.5	Mainly fire	No	No	No	No	No	No	No
0.5	Animal dur	No	No	No	No	Yes	No	No
0.5	Mainly fire	No	No	No	No	Yes	No	No
0.5	Mainly fire	Yes	No	No	No	Yes	No	No
2	Animal dur	Yes	Yes	Yes	No	Yes	No	No

For peer review only

	Mainly fire	Yes	Yes	Yes	Yes	Yes	No	No
0.5	Animal dur	Yes	No	Yes	No	Yes	No	No
0.2	Mainly fire	Yes	No	Yes	No	Yes	No	No
	Butane gas	Yes	Yes	Yes	Yes	Yes	Yes	Yes
0.1	Animal dur	No	No	Yes	No	Yes	No	No
0.2	Animal dur	No	No	No	No	Yes	No	No
0.1	Animal dur	No	No	No	No	Yes	No	No
0.25	Mainly fire	Yes	No	Yes	No	Yes	No	No
0.25	Mainly fire	Yes	Yes	Yes	Yes	Yes	No	No
1	Mainly fire	Yes	No	No	No	Yes	No	No
0.5	Mainly fire	No	No	No	No	Yes	No	No
	Mainly fire	Yes	No	No	Yes	Yes	No	No
0.1	Animal dur	Yes	No	No	No	Yes	No	No
0.75	Animal dur	No	No	No	No	Yes	No	No
1	Mainly fire	No	No	No	No	Yes	No	No
	Charcoal	Yes	No	Yes	No	Yes	No	No
1	Charcoal	Yes	No	Yes	Yes	Yes	No	No
0.1	Mainly fire	Yes	No	No	No	Yes	No	No
0.2	Mainly fire	No	No	Yes	No	Yes	No	No
	Mainly fire	Yes	No	No	Yes	Yes	No	No
1.5	Charcoal	No	No	No	No	Yes	No	No
0.3	Animal dur	No	Yes	Yes	No	No	No	No
0.5	Animal dur	No	No	Yes	No	Yes	No	No
	Charcoal	Yes	Yes	Yes	Yes	Yes	No	Yes
	Charcoal	Yes	No	Yes	Yes	Yes	No	No
	Animal dur	No	No	No	No	No	No	No
2	Mainly fire	Yes	No	Yes	No	Yes	No	No
0.1	Animal dur	No	No	No	No	Yes	No	No
	Charcoal	Yes	No	Yes	Yes	Yes	No	No
2	Charcoal	Yes	No	No	Yes	Yes	No	Yes
0.5	Mainly fire	No	No	No	No	Yes	No	No
	Mainly fire	Yes	No	No	No	Yes	No	No
	Mainly fire	Yes	Yes	Yes	No	Yes	No	No
	Mainly fire	Yes	No	No	No	Yes	No	No
0.75	Animal dur	Yes	No	Yes	No	Yes	No	No
0.75	Animal dur	Yes	No	Yes	No	Yes	No	No
0.25	Mainly fire	No	No	Yes	No	Yes	No	No
0.5	Animal dur	Yes	No	Yes	No	Yes	No	No
	Electricity	Yes	No	No	Yes	Yes	No	No
	Animal dur	Yes	No	Yes	Yes	No	No	No
	Mainly fire	Yes	No	No	Yes	Yes	No	No
	Charcoal	No	No	No	No	No	No	No
1	Charcoal	Yes	No	No	Yes	Yes	No	Yes
0.5	Mainly fire	Yes	No	Yes	No	Yes	No	No
0.25	Mainly fire	Yes	No	No	Yes	Yes	No	No
0.5	Mainly fire	Yes	No	Yes	No	Yes	No	No
	Charcoal	Yes	No	No	Yes	Yes	No	No
1	Mainly fire	Yes	No	Yes	No	Yes	No	No
0.5	Animal dur	Yes	No	Yes	No	Yes	No	No
0.75	Mainly fire	No	No	Yes	No	Yes	No	No
0.2	Animal dur	No	No	Yes	No	Yes	No	No
	Mainly fire	Yes	No	No	Yes	Yes	No	No

For peer review only

4	Animal dur	No	No	No	No	No	No	No
	Mainly fire	Yes	No	No	Yes	Yes	No	No
0.5	Electricity	No	No	Yes	Yes	Yes	No	Yes
0.1	Animal dur	No	No	Yes	No	Yes	No	No
0.5	Animal dur	No	No	No	No	Yes	Yes	No
	Mainly fire	Yes	No	No	Yes	Yes	No	No
0.1	Animal dur	Yes	No	Yes	No	Yes	No	No
	Animal dur	Yes	No	No	Yes	No	No	No
0.5	Mainly fire	Yes	No	Yes	No	No	No	No
0.5	Mainly fire	No	No	Yes	No	Yes	No	No
	Charcoal	Yes	No	No	Yes	Yes	No	No
0.25	Animal dur	Yes	No	No	No	Yes	No	No
2	Mainly fire	No	No	Yes	No	Yes	No	No
3	Mainly fire	Yes	No	Yes	Yes	Yes	No	No
0.5	Mainly fire	Yes	No	Yes	Yes	Yes	No	No
1.25	Mainly fire	Yes	No	No	No	Yes	No	No
0.3	Animal dur	Yes	No	Yes	No	Yes	No	No
	Charcoal	Yes	No	No	Yes	Yes	No	No
0.75	Mainly fire	Yes	No	No	No	Yes	No	No
2.5	Charcoal	Yes	No	Yes	Yes	Yes	No	No
1	Mainly fire	Yes	No	Yes	No	No	No	No
0.5	Mainly fire	Yes	No	Yes	No	Yes	No	Yes
1	Mainly fire	Yes	No	Yes	No	No	No	No
1	Mainly fire	Yes	No	No	No	No	No	No
3	Mainly fire	Yes	No	No	No	Yes	No	No
1.5	Mainly fire	No	Yes	No	No	Yes	No	No
1	Mainly fire	Yes	No	Yes	No	No	No	No
1	Mainly fire	Yes	Yes	Yes	No	Yes	No	No
0.5	Crop residu	No	No	No	No	Yes	No	No
	Mainly fire	Yes	No	Yes	No	Yes	No	No
2	Mainly fire	Yes	Yes	Yes	No	Yes	No	No
1	Mainly fire	Yes	No	No	No	Yes	No	No
1	Mainly fire	No	Yes	Yes	No	Yes	Yes	No
0.5	Mainly fire	Yes	No	Yes	No	No	No	No
2	Mainly fire	Yes	No	Yes	No	Yes	No	No
2	Mainly fire	Yes	No	Yes	No	Yes	No	No
	Mainly fire	Yes	No	No	No	No	No	No
1	Mainly fire	Yes	No	Yes	No	Yes	No	No
1	Mainly fire	Yes	No	No	No	No	No	No
0.5	Mainly fire	Yes	No	No	No	No	No	No
0	Mainly fire	Yes	No	No	No	No	No	No
2	Mainly fire	Yes	No	Yes	No	Yes	No	No
	Mainly fire	No	No	No	No	Yes	No	No
3	Mainly fire	Yes	No	No	No	Yes	No	No
1	Mainly fire	Yes	No	No	No	Yes	No	No
0.5	Mainly fire	No	No	No	No	Yes	No	No
1	Mainly fire	Yes	No	Yes	No	Yes	No	No
2	Mainly fire	Yes	No	No	No	Yes	No	No
1	Mainly fire	No	Yes	No	No	Yes	No	No
1.5	Mainly fire	Yes	No	Yes	No	Yes	No	No
2	Mainly fire	Yes	Yes	No	No	Yes	No	No
	Mainly fire	No	No	No	No	Yes	No	No

For peer review only

0.75	Mainly fire	Yes	No	Yes	No	No	No	No
2	Mainly fire	Yes	No	No	No	No	No	No
	Mainly fire	Yes	No	No	No	No	No	No
3	Mainly fire	Yes	No	No	No	Yes	No	No
1	Mainly fire	Yes	No	Yes	No	No	No	No
	Crop residu	Yes	No	Yes	No	Yes	No	No
6	Mainly fire	No	No	No	No	No	No	No
1	Mainly fire	Yes	No	No	No	No	No	No
3	Mainly fire	Yes	No	Yes	No	Yes	No	No
1	Crop residu	Yes	No	Yes	No	Yes	No	No
1	Mainly fire	Yes	No	Yes	No	Yes	No	No
1	Mainly fire	Yes	No	No	No	Yes	No	No
6	Mainly fire	Yes	No	Yes	No	Yes	No	No
1	Mainly fire	Yes	Yes	No	No	No	No	No
1	Mainly fire	Yes	No	Yes	No	Yes	No	No
	Mainly fire	Yes	No	Yes	No	Yes	No	No
1	Mainly fire	No	No	No	No	No	No	No
1.5	Crop residu	Yes	No	Yes	No	Yes	No	No
1	Mainly fire	No	No	No	No	No	No	No
1	Mainly fire	Yes	No	Yes	No	Yes	No	No
999	Mainly fire	Yes	No	No	No	No	No	No
0.5	Mainly fire	Yes	No	No	No	No	No	No
2	Mainly fire	Yes	Yes	No	No	Yes	No	No
3	Mainly fire	Yes	No	Yes	No	Yes	No	No
0.5	Crop residu	Yes	No	No	No	Yes	No	No
1	Mainly fire	Yes	No	Yes	No	Yes	No	No
0.25	Crop residu	Yes	No	Yes	No	Yes	No	No
2	Mainly fire	Yes	Yes	No	No	Yes	No	No
	Mainly fire	Yes	No	No	No	Yes	No	No
2	Mainly fire	Yes	Yes	No	No	Yes	No	No
1.5	Crop residu	Yes	No	No	No	Yes	No	No
0.5	Crop residu	No	No	No	No	Yes	No	No
2	Mainly fire	Yes	No	Yes	No	Yes	No	No
2	Mainly fire	No	No	No	No	Yes	No	No
0.5	Crop residu	Yes	No	Yes	No	Yes	No	No
1	Mainly fire	No	No	No	No	No	No	No
1.25	Crop residu	Yes	No	Yes	No	Yes	No	No
0	Crop residu	Yes	No	No	No	No	No	No
	Mainly fire	Yes	No	No	No	No	No	No
1	Mainly fire	Yes	No	No	No	No	No	No
1	Mainly fire	Yes	No	Yes	No	Yes	No	No
0.5	Mainly fire	Yes	No	Yes	No	Yes	No	No
1	Mainly fire	Yes	Yes	No	No	Yes	No	No
0.5	Crop residu	No	No	No	No	Yes	No	No
	Mainly fire	Yes	No	Yes	No	No	No	No
0.5	Mainly fire	Yes	No	No	No	No	No	No
	Mainly fire	Yes	No	No	No	Yes	No	No
1	Mainly fire	Yes	No	Yes	No	Yes	No	No
	Mainly fire	Yes	No	Yes	No	Yes	No	No
	Mainly fire	Yes	No	No	No	No	No	No
4	Mainly fire	Yes	No	Yes	No	Yes	No	No
0.5	Mainly fire	No	No	No	No	No	No	No

For peer review only

2	Mainly fire	Yes	Yes	Yes	No	Yes	No	No
3	Mainly fire	Yes	No	No	No	Yes	No	No
	Mainly fire	Yes	No	Yes	No	No	No	No
	Mainly fire	Yes	No	Yes	No	No	No	No
99.99	Mainly fire	Yes	No	No	No	No	No	No
	Crop residu	Yes	No	No	No	No	No	No
2	Mainly fire	Yes	No	Yes	No	Yes	No	No
1	Mainly fire	Yes	No	Yes	No	Yes	No	No
1	Mainly fire	No	No	No	No	Yes	No	No
1	Mainly fire	Yes	Yes	Yes	No	Yes	No	No
1	Mainly fire	Yes	Yes	Yes	No	Yes	No	No
3	Mainly fire	No	No	No	No	Yes	No	No
1.5	Crop residu	Yes	No	Yes	No	Yes	No	No
1	Mainly fire	Yes	No	Yes	No	No	No	No
2	Mainly fire	Yes	Yes	Yes	No	No	No	No
1.25	Mainly fire	Yes	No	Yes	No	Yes	No	No
2	Mainly fire	Yes	No	No	No	Yes	No	No
1	Mainly fire	Yes	No	No	No	No	No	No
4	Mainly fire	Yes	No	Yes	Yes	Yes	No	No
1.25	Mainly fire	No	Yes	Yes	No	Yes	No	No
5	Animal dur	Yes	No	No	Yes	Yes	No	No
	Mainly fire	Yes	No	No	Yes	Yes	No	No
0.25	Mainly fire	Yes	No	No	Yes	Yes	No	No
	Mainly fire	Yes	No	No	Yes	Yes	No	No
1	Mainly fire	No	No	No	No	Yes	No	No
	Animal dur	Yes	No	Yes	Yes	Yes	No	No
0.25	Mainly fire	Yes	Yes	Yes	Yes	Yes	No	No
	Mainly fire	Yes	No	No	Yes	Yes	No	No
	Mainly fire	Yes	No	No	No	Yes	No	No
1	Mainly fire	Yes	No	Yes	Yes	Yes	No	No
	Mainly fire	Yes	No	No	Yes	Yes	No	No
	Mainly fire	Yes	No	Yes	Yes	Yes	No	No
0.5	Mainly fire	No	No	Yes	No	Yes	No	No
0.5	Mainly fire	Yes	Yes	No	No	Yes	No	No
0.5	Mainly fire	No	No	Yes	No	Yes	No	No
1	Mainly fire	Yes	Yes	Yes	Yes	Yes	No	No
	Mainly fire	Yes	No	Yes	Yes	Yes	No	No
	Mainly fire	Yes	No	Yes	Yes	Yes	No	No
	Mainly fire	No	No	No	No	No	No	No
0.5	Mainly fire	Yes	No	Yes	No	Yes	No	No
1	Mainly fire	Yes	No	No	No	Yes	No	No
0.5	Mainly fire	No	No	No	No	Yes	No	No
0.25	Animal dur	Yes	No	No	No	Yes	No	No
1	Mainly fire	Yes	No	Yes	No	Yes	No	No
1	Mainly fire	Yes	No	Yes	Yes	Yes	No	No
	Animal dur	Yes	No	Yes	No	Yes	No	No
1	Mainly fire	Yes	No	Yes	No	Yes	No	No
0.5	Mainly fire	Yes	Yes	Yes	No	Yes	No	No
1	Animal dur	Yes	No	Yes	No	Yes	No	No
0.5	Mainly fire	Yes	No	No	No	Yes	No	No
	Animal dur	Yes	No	Yes	No	Yes	No	No
	Mainly fire	No	No	Yes	No	Yes	No	No

	Mainly fire'	Yes	No	Yes	No	Yes	No	No
	Mainly fire'	Yes	No	Yes	No	Yes	No	No
1	Mainly fire'	No	No	Yes	No	Yes	No	No
0.5	Mainly fire'	Yes	No	No	Yes	Yes	No	No
0.5	Mainly fire'	Yes	No	No	No	Yes	No	No
0.5	Mainly fire'	Yes	Yes	Yes	No	Yes	No	No
0.5	Mainly fire'	No	No	Yes	No	Yes	No	No
1	Mainly fire'	Yes	No	No	No	Yes	No	No
2	Mainly fire'	No	No	No	No	Yes	No	No
0.5	Mainly fire'	Yes	No	No	No	Yes	No	No
0.25	Mainly fire'	Yes	No	Yes	No	Yes	No	No
1.5	Mainly fire'	Yes	Yes	Yes	No	Yes	No	No
3	Mainly fire'	No	No	Yes	No	Yes	No	No
0.5	Mainly fire'	No	No	Yes	No	Yes	No	No
	Mainly fire'	No	No	No	No	No	No	No
0.5	Mainly fire'	Yes	No	Yes	No	Yes	No	No
2	Mainly fire'	Yes	No	No	No	Yes	No	No
1	Mainly fire'	Yes	No	Yes	No	Yes	No	No
1	Mainly fire'	No	No	No	No	No	No	No
0.25	Mainly fire'	No	No	Yes	No	Yes	No	No
2	Mainly fire'	Yes	No	Yes	Yes	Yes	Yes	No
1	Mainly fire'	Yes	No	No	No	Yes	No	No
99.99	Mainly fire'	Yes	No	No	No	No	No	No
0.75	Mainly fire'	No	No	No	No	Yes	No	No
2	Mainly fire'	No	No	Yes	Yes	Yes	No	No
	Mainly fire'	No	No	Yes	No	Yes	No	No
1	Crop residu	Yes	No	No	No	Yes	No	No
0.5	Mainly fire'	Yes	No	No	No	Yes	No	No
0.75	Mainly fire'	Yes	No	Yes	No	Yes	No	No
2	Mainly fire'	No	No	No	No	Yes	No	No
1	Mainly fire'	Yes	No	No	No	Yes	No	No
0.25	Mainly fire'	No	No	Yes	No	Yes	No	No
3	Mainly fire'	No	No	Yes	No	No	No	No
1.25	Crop residu	Yes	Yes	No	No	Yes	No	No
1	Charcoal	Yes	Yes	No	Yes	Yes	No	No
1.5	Mainly fire'	Yes	No	No	No	Yes	No	No
0.25	Mainly fire'	Yes	No	No	No	Yes	No	No
0.5	Mainly fire'	Yes	No	Yes	No	Yes	No	No
1	Mainly fire'	No	No	Yes	No	No	No	No
2	Mainly fire'	Yes	No	Yes	No	Yes	No	No
99.99	Mainly fire'	No	No	No	No	No	No	No
1	Mainly fire'	Yes	No	No	No	Yes	No	No
1.5	Mainly fire'	Yes	No	No	No	Yes	No	No
0.5	Crop residu	Yes	No	No	No	No	No	No
0	Charcoal	No	No	Yes	No	No	No	No
0.5	Mainly fire'	No	No	No	No	Yes	No	No
0.5	Mainly fire'	No	No	No	No	No	No	No
0.5	Mainly fire'	No	No	No	No	Yes	No	No
1	Electricity	No	No	No	No	Yes	No	No
0.5	Mainly fire'	Yes	No	No	No	Yes	No	No
2	Mainly fire'	Yes	No	No	No	No	No	No
1	Mainly fire'	Yes	No	Yes	No	Yes	No	No

For peer review only

2	Mainly fire	Yes	No	No	No	Yes	No	No
	Mainly fire	No	No	Yes	No	Yes	No	No
	Mainly fire	Yes	Yes	Yes	No	Yes	No	No
2	Mainly fire	No	No	No	No	No	No	No
0.75	Crop residu	Yes	No	No	No	No	No	No
	Mainly fire	No	No	Yes	No	No	No	No
	Mainly fire	No	No	No	No	Yes	No	No
1.5	Mainly fire	Yes	No	No	No	No	No	No
0.5	Mainly fire	No	No	No	No	Yes	No	No
0.5	Mainly fire	No	No	No	No	Yes	No	No
2	Mainly fire	Yes	No	No	No	No	No	No
0.5	Mainly fire	No	No	No	No	No	No	No
0.25	Mainly fire	No	No	Yes	No	Yes	No	No
	Mainly fire	No	No	Yes	No	Yes	No	No
1	Mainly fire	Yes	No	No	No	Yes	No	No
	Mainly fire	Yes	No	Yes	Yes	Yes	No	No
0.5	Mainly fire	Yes	No	Yes	No	Yes	No	No
3	Mainly fire	No	No	Yes	No	Yes	No	No
	Mainly fire	No	No	Yes	No	No	No	No
2	Mainly fire	Yes	No	No	No	No	No	No
1	Mainly fire	No	No	Yes	No	Yes	No	No
0.5	Mainly fire	No	No	Yes	No	Yes	No	No
2.5	Mainly fire	Yes	No	No	No	Yes	No	No
1	Mainly fire	Yes	No	No	No	Yes	No	No
1.5	Mainly fire	Yes	Yes	Yes	No	Yes	No	No
	Mainly fire	Yes	No	Yes	No	Yes	No	No
1.25	Mainly fire	Yes	No	No	No	No	Yes	No
	Mainly fire	No	No	Yes	No	Yes	No	No
	Mainly fire	No	No	Yes	No	Yes	No	No
	Mainly fire	Yes	No	Yes	No	Yes	No	No
0.5	Mainly fire	No	No	Yes	No	No	No	No
	Mainly fire	Yes	No	No	No	No	No	No
2	Mainly fire	Yes	No	No	No	Yes	No	No
999	Mainly fire	Yes	No	No	No	Yes	No	No
	Mainly fire	Yes	No	No	No	No	No	No
2	Mainly fire	Yes	No	No	No	No	No	No
1.5	Mainly fire	Yes	No	No	No	Yes	No	No
8	Mainly fire	Yes	No	No	No	Yes	No	No
6	Mainly fire	Yes	No	No	No	Yes	No	No
0.5	Mainly fire	Yes	No	Yes	No	Yes	No	No
	Mainly fire	Yes	No	Yes	No	Yes	No	No
	Mainly fire	Yes	No	No	No	No	No	No
1	Mainly fire	Yes	No	Yes	No	Yes	No	No
	Crop residu	Yes	Yes	No	No	Yes	No	No
2	Mainly fire	Yes	No	No	No	No	No	No
5	Mainly fire	Yes	No	Yes	No	No	No	No
4	Crop residu	Yes	No	No	No	Yes	No	No
	Mainly fire	Yes	No	No	No	Yes	No	No
	Mainly fire	Yes	No	No	No	Yes	No	No
	Mainly fire	Yes	No	No	No	Yes	No	No
1	Mainly fire	Yes	No	Yes	No	Yes	No	No
3	Mainly fire	Yes	No	No	No	Yes	No	No

For peer review only

3	Crop residu	Yes	No	No	No	Yes	No	No
	Crop residu	Yes	No	No	No	Yes	No	No
999	Mainly fire	Yes	No	No	No	Yes	No	No
1	Mainly fire	Yes	No	Yes	No	No	No	No
3	Mainly fire	No	No	No	No	No	No	No
2	Mainly fire	Yes	No	Yes	No	Yes	No	No
3	Mainly fire	Yes	No	No	No	No	No	No
0.75	Mainly fire	Yes	No	No	No	Yes	No	No
	Mainly fire	Yes	No	Yes	No	Yes	No	No
1	Mainly fire	Yes	No	No	No	Yes	No	No
1	Mainly fire	No	No	No	No	Yes	No	No
0	Mainly fire	Yes	No	No	No	No	No	No
	Mainly fire	Yes	No	Yes	No	Yes	No	No
0.5	Mainly fire	Yes	No	Yes	No	Yes	No	No
1	Mainly fire	Yes	No	Yes	No	Yes	No	No
0.5	Mainly fire	Yes	No	No	No	Yes	No	No
1	Mainly fire	Yes	No	No	No	Yes	No	No
6	Mainly fire	No	No	No	No	Yes	No	No
4	Mainly fire	Yes	No	No	No	Yes	No	No
0.5	Mainly fire	Yes	No	No	No	Yes	No	No
0.5	Mainly fire	Yes	No	No	No	Yes	No	No
6	Crop residu	Yes	No	No	No	Yes	No	No
3	Mainly fire	Yes	No	No	No	Yes	No	No
3	Mainly fire	Yes	No	No	No	No	No	No
	Mainly fire	Yes	Yes	Yes	No	Yes	No	No
3	Mainly fire	Yes	No	No	No	Yes	No	No
1	Mainly fire	Yes	Yes	Yes	No	Yes	No	No
1	Mainly fire	Yes	No	Yes	No	Yes	No	No
3	Mainly fire	Yes	No	No	No	Yes	No	No
0.5	Mainly fire	Yes	No	Yes	No	Yes	No	No
2.25	Mainly fire	Yes	No	No	No	Yes	No	No
1.5	Mainly fire	Yes	No	Yes	No	Yes	No	No
1	Mainly fire	Yes	No	No	No	Yes	No	No
4	Mainly fire	No	No	No	No	Yes	No	No
	Crop residu	Yes	No	No	No	Yes	No	No
2	Mainly fire	Yes	No	No	No	Yes	No	No
0.5	Mainly fire	Yes	No	No	No	No	No	No
1.25	Mainly fire	Yes	No	No	No	Yes	No	No
0.5	Crop residu	Yes	No	No	No	Yes	No	No
	Mainly fire	Yes	No	No	Yes	Yes	No	No
999	Mainly fire	Yes	No	Yes	No	Yes	No	No
0.5	Mainly fire	Yes	No	Yes	No	Yes	No	No
1.5	Animal dur	Yes	No	Yes	Yes	Yes	No	No
2	Animal dur	Yes	No	Yes	Yes	Yes	No	No
	Charcoal	Yes	No	Yes	Yes	Yes	No	No
	Mainly fire	Yes	Yes	Yes	Yes	Yes	No	No
	Mainly fire	Yes	Yes	No	Yes	Yes	No	No
4	Mainly fire	Yes	No	No	No	Yes	No	No
1	Mainly fire	Yes	No	No	Yes	Yes	No	No
	Mainly fire	Yes	No	No	Yes	Yes	No	No
	Charcoal	Yes	No	No	Yes	Yes	No	No
5	Mainly fire	Yes	No	Yes	Yes	Yes	No	No

For peer review only

1.5	Mainly fire	Yes	No	No	No	Yes	No	No
	Animal dur	Yes	No	No	Yes	Yes	No	No
2	Mainly fire	Yes	No	Yes	Yes	Yes	No	No
	Mainly fire	Yes	No	Yes	Yes	Yes	No	No
	Animal dur	Yes	No	No	No	Yes	No	No
	Mainly fire	No	No	No	No	Yes	No	No
	Mainly fire	No	No	Yes	No	No	No	No
	Mainly fire	Yes	No	No	Yes	Yes	No	No
0.25	Mainly fire	Yes	No	Yes	No	Yes	No	No
	Mainly fire	Yes	No	No	No	Yes	No	No
0.75	Mainly fire	Yes	No	Yes	Yes	Yes	No	No
2	Mainly fire	Yes	No	No	No	No	No	No
	Mainly fire	No	No	No	No	Yes	No	No
0.5	Mainly fire	Yes	No	No	No	Yes	No	No
1	Mainly fire	No	Yes	Yes	No	Yes	No	No
0.5	Mainly fire	No	Yes	Yes	No	Yes	No	No
	Mainly fire	No	No	Yes	No	Yes	No	No
0.5	Mainly fire	No	No	No	No	No	No	No
0.25	Mainly fire	Yes	No	No	Yes	Yes	No	No
	Mainly fire	Yes	Yes	No	Yes	Yes	No	No
	Charcoal	Yes	No	No	Yes	Yes	No	No
1.5	Mainly fire	Yes	No	No	No	No	No	No
0.5	Mainly fire	Yes	Yes	Yes	Yes	Yes	No	Yes
1	Mainly fire	Yes	No	Yes	No	Yes	No	No
1	Mainly fire	Yes	No	Yes	No	Yes	No	No
	Mainly fire	Yes	No	No	Yes	Yes	No	No
1	Mainly fire	Yes	Yes	Yes	No	Yes	No	No
	Mainly fire	Yes	Yes	Yes	Yes	Yes	No	No
1.5	Animal dur	Yes	No	Yes	Yes	Yes	No	No
0.75	Mainly fire	Yes	No	Yes	Yes	Yes	No	No
	Mainly fire	Yes	Yes	Yes	Yes	Yes	No	No
1	Mainly fire	Yes	No	Yes	No	Yes	No	No
0.5	Mainly fire	Yes	No	No	Yes	Yes	No	Yes
	Charcoal	Yes	Yes	No	No	Yes	No	No
0.25	Mainly fire	Yes	Yes	No	Yes	Yes	No	No
1	Mainly fire	Yes	No	Yes	No	Yes	No	No
	Mainly fire	Yes	No	No	No	No	No	No
	Mainly fire	Yes	No	Yes	No	Yes	No	No
1	Mainly fire	Yes	No	No	No	Yes	No	No
0.5	Electricity	Yes	No	No	Yes	Yes	No	No
	Animal dur	Yes	Yes	Yes	No	Yes	No	No
0.5	Mainly fire	Yes	No	No	No	Yes	No	No
	Mainly fire	Yes	No	No	Yes	Yes	No	No
1.5	Mainly fire	Yes	No	Yes	Yes	Yes	No	No
	Mainly fire	Yes	Yes	Yes	Yes	Yes	No	No
	Mainly fire	Yes	Yes	Yes	No	Yes	No	No
999	Mainly fire	No	No	No	No	Yes	No	No
0.25	Mainly fire	No	No	No	No	Yes	No	No
0.5	Mainly fire	No	Yes	Yes	No	Yes	No	No
1	Mainly fire	No	No	Yes	No	Yes	No	No
0.75	Mainly fire	No	Yes	Yes	No	Yes	No	No
1	Mainly fire	Yes	No	Yes	No	Yes	No	No

For peer review only

0.25	Electricity	Yes	Yes	Yes	Yes	Yes	No	No
	Charcoal	Yes	No	No	Yes	Yes	No	No
2	Mainly fire	Yes	No	No	No	Yes	No	No
	Mainly fire	Yes	No	Yes	No	Yes	No	No
	Mainly fire	Yes	No	No	Yes	Yes	No	No
4	Mainly fire	Yes	No	Yes	No	Yes	No	No
0.5	Mainly fire	No	Yes	Yes	No	Yes	No	No
1	Animal dur	Yes	Yes	Yes	Yes	Yes	No	No
	Mainly fire	Yes	No	No	No	Yes	No	No
0.5	Mainly fire	Yes	No	No	Yes	Yes	No	Yes
99.99	Mainly fire	Yes	No	No	No	No	No	No
	Mainly fire	Yes	Yes	No	Yes	Yes	No	No
	Mainly fire	No	No	No	No	Yes	No	No
0.5	Mainly fire	No	No	Yes	No	Yes	No	No
	Crop residu	No	No	Yes	No	Yes	No	No
	Mainly fire	No	No	No	No	Yes	No	No
0.25	Mainly fire	Yes	No	No	Yes	Yes	No	No
2	Mainly fire	Yes	No	No	No	Yes	No	Yes
	Mainly fire	No	No	No	No	Yes	No	No
	Mainly fire	No	Yes	Yes	No	Yes	No	No
	Mainly fire	Yes	No	No	No	Yes	No	No
0.75	Mainly fire	No	Yes	Yes	No	Yes	No	No
	Mainly fire	Yes	Yes	Yes	Yes	Yes	No	No
	Animal dur	Yes	No	Yes	No	Yes	No	Yes
	Mainly fire	Yes	No	No	No	No	No	No
0.75	Mainly fire	Yes	No	Yes	No	Yes	No	No
0.5	Kerosene	Yes	No	Yes	Yes	Yes	No	No
	Charcoal	No	No	Yes	No	Yes	No	No
	Animal dur	Yes	No	No	Yes	Yes	No	No
1.5	Mainly fire	Yes	Yes	Yes	Yes	Yes	No	No
1	Mainly fire	No	No	Yes	No	Yes	No	No
0.25	Animal dur	Yes	No	Yes	No	Yes	No	No
0.25	Mainly fire	Yes	No	No	No	Yes	No	No
1	Mainly fire	Yes	No	Yes	Yes	Yes	No	No
	Animal dur	Yes	No	No	Yes	Yes	No	No
	Mainly fire	Yes	No	No	Yes	Yes	No	No
0.75	Mainly fire	Yes	Yes	Yes	Yes	Yes	No	No
	Mainly fire	Yes	No	Yes	No	Yes	No	No
3	Charcoal	Yes	No	Yes	Yes	No	No	No
1	Mainly fire	No	No	No	No	Yes	No	No
	Charcoal	Yes	No	Yes	Yes	Yes	No	No
1	Mainly fire	Yes	No	No	No	Yes	No	No
	Mainly fire	No	Yes	Yes	Yes	Yes	No	No
1	Mainly fire	Yes	No	No	No	Yes	No	No
1	Charcoal	Yes	No	Yes	Yes	Yes	No	No
1	Mainly fire	Yes	No	No	No	Yes	No	No
	Mainly fire	Yes	No	Yes	No	Yes	No	No
0.75	Mainly fire	No	No	No	No	Yes	No	No
	Mainly fire	Yes	Yes	Yes	No	No	No	No
4	Mainly fire	Yes	Yes	Yes	Yes	Yes	No	Yes
0.5	Mainly fire	Yes	No	Yes	Yes	Yes	No	No
1	Mainly fire	No	No	No	No	No	No	No

For peer review only

1	Mainly fire	No	No	No	No	Yes	No	No
3	Mainly fire	Yes	No	No	No	Yes	No	No
0.5	Mainly fire	Yes	Yes	Yes	Yes	Yes	No	No
	Animal dur	Yes	No	Yes	Yes	Yes	No	No
	Charcoal	No	No	Yes	No	Yes	No	No
0.75	Mainly fire	No	No	Yes	No	Yes	No	No
	Mainly fire	Yes	No	No	Yes	Yes	No	No
1	Mainly fire	No	Yes	Yes	No	Yes	No	No
2	Animal dur	Yes	No	Yes	Yes	Yes	No	No
2	Charcoal	Yes	No	Yes	No	Yes	No	No
	Mainly fire	No	Yes	Yes	No	Yes	No	No
	Mainly fire	Yes	No	No	Yes	Yes	No	Yes
1	Mainly fire	Yes	No	Yes	Yes	Yes	No	Yes
	Mainly fire	Yes	No	Yes	No	Yes	No	No
1	Mainly fire	No	No	Yes	No	Yes	No	No
2	Charcoal	Yes	No	No	Yes	Yes	No	No
	Mainly fire	Yes	No	Yes	Yes	Yes	No	No
	Mainly fire	Yes	No	Yes	Yes	Yes	No	Yes
	Mainly fire	No	No	No	No	Yes	No	No
	Electricity	Yes	Yes	Yes	Yes	Yes	No	No
	Charcoal	No	No	Yes	No	Yes	No	No
1	Mainly fire	No	No	Yes	No	Yes	No	No
	Mainly fire	No	No	No	No	No	No	No
	Electricity	Yes	No	No	Yes	Yes	No	No
0.25	Mainly fire	Yes	Yes	No	Yes	Yes	No	No
0.25	Mainly fire	Yes	Yes	Yes	No	Yes	No	No
0.25	Mainly fire	No	No	No	No	Yes	No	No
1	Mainly fire	No	No	Yes	Yes	Yes	No	No
	Mainly fire	No	No	No	No	Yes	No	No
	Charcoal	Yes	No	Yes	No	No	No	No
0.5	Animal dur	Yes	No	Yes	Yes	Yes	No	No
	Electricity	Yes	Yes	Yes	Yes	Yes	No	Yes
	Mainly fire	Yes	No	No	Yes	Yes	No	No
	Charcoal	Yes	No	Yes	No	Yes	No	No
	Mainly fire	Yes	Yes	No	Yes	Yes	No	No
1	Mainly fire	Yes	No	No	No	Yes	No	No
	Mainly fire	Yes	No	No	Yes	Yes	No	No
1	Mainly fire	Yes	No	No	Yes	Yes	No	No
1	Mainly fire	No	No	Yes	No	Yes	No	No
0.5	Charcoal	Yes	No	No	Yes	No	No	No
	Electricity	Yes	Yes	No	Yes	Yes	No	No
1.5	Animal dur	Yes	No	No	No	No	No	No
2	Mainly fire	Yes	No	No	No	Yes	No	No
	Mainly fire	Yes	No	No	Yes	Yes	No	No
2.5	Animal dur	Yes	No	Yes	No	Yes	No	No
999	Mainly fire	Yes	No	No	No	Yes	No	No
1	Mainly fire	Yes	No	No	No	No	No	No
0.25	Charcoal	Yes	No	Yes	Yes	No	No	No
2	Mainly fire	Yes	No	Yes	Yes	No	No	No
0.75	Mainly fire	No	No	Yes	No	Yes	No	No
0.75	Mainly fire	No	Yes	Yes	No	Yes	No	No
	Mainly fire	Yes	No	No	No	Yes	No	No

	Mainly fire	Yes	No	No	Yes	Yes	No	No
1	Mainly fire	Yes	No	Yes	Yes	Yes	No	No
	Animal dur	Yes	Yes	Yes	Yes	Yes	No	No
	Mainly fire	Yes	No	No	Yes	Yes	No	No
	Mainly fire	Yes	No	No	Yes	Yes	No	No
1	Mainly fire	Yes	No	No	Yes	Yes	No	No
0.5	Mainly fire	Yes	Yes	No	Yes	Yes	No	No
	Mainly fire	Yes	No	No	No	No	No	No
	Mainly fire	Yes	Yes	No	Yes	Yes	No	No
999	Mainly fire	Yes	No	No	No	No	No	No
	Charcoal	Yes	No	No	No	Yes	No	No
0.5	Mainly fire	No	No	No	No	Yes	No	No
1	Animal dur	Yes	Yes	Yes	Yes	Yes	No	No
	Charcoal	Yes	No	Yes	Yes	Yes	No	No
	Mainly fire	Yes	No	No	Yes	Yes	No	No
	Mainly fire	Yes	No	No	No	Yes	No	No
999	Mainly fire	Yes	No	No	No	Yes	No	No
	Mainly fire	No	No	No	No	No	No	No
1.5	Mainly fire	Yes	Yes	Yes	Yes	Yes	No	No
0.5	Mainly fire	Yes	No	Yes	No	Yes	No	No
0	Mainly fire	Yes	No	Yes	Yes	Yes	No	No
	Animal dur	Yes	No	Yes	Yes	Yes	No	No
1.25	Mainly fire	Yes	No	No	No	Yes	No	No
1	Mainly fire	No	No	Yes	No	Yes	No	No
	Charcoal	Yes	No	No	No	Yes	No	No
0.25	Mainly fire	Yes	Yes	No	No	Yes	No	No
2	Mainly fire	Yes	No	No	No	No	No	No
	Animal dur	Yes	No	Yes	Yes	Yes	Yes	No
	Charcoal	Yes	No	Yes	No	Yes	No	No
	Mainly fire	Yes	No	Yes	No	Yes	No	No
999	Mainly fire	Yes	No	No	No	No	No	No
0.25	Mainly fire	Yes	No	No	Yes	Yes	No	No
0.25	Mainly fire	No	No	No	No	Yes	No	No
	Charcoal	Yes	No	No	No	Yes	No	No
	Mainly fire	Yes	No	No	No	Yes	Yes	No
0.5	Mainly fire	No	Yes	Yes	No	Yes	No	No
	Mainly fire	Yes	Yes	Yes	No	Yes	No	No
	Mainly fire	Yes	Yes	No	No	Yes	No	No
2	Mainly fire	Yes	No	No	No	Yes	No	No
	Animal dur	No	No	Yes	No	Yes	No	No
0.5	Mainly fire	Yes	Yes	Yes	Yes	Yes	No	No
	Animal dur	Yes	Yes	No	No	Yes	Yes	No
2	Mainly fire	Yes	No	No	No	No	No	No
0.75	Mainly fire	Yes	No	No	No	Yes	No	No
	Mainly fire	Yes	Yes	No	Yes	Yes	No	No
	Animal dur	Yes	No	No	Yes	Yes	No	No
1	Mainly fire	No	No	No	No	Yes	No	No
0.25	Mainly fire	Yes	No	No	No	Yes	No	No
2	Mainly fire	Yes	No	No	No	Yes	No	No
1	Charcoal	Yes	No	Yes	Yes	Yes	No	No
1	Mainly fire	No	No	Yes	No	Yes	No	No
1.5	Mainly fire	Yes	No	No	Yes	Yes	No	No

For peer review only

0.5	Mainly fire	Yes	Yes	Yes	Yes	Yes	No	No
	Mainly fire	Yes	No	Yes	Yes	Yes	No	Yes
	Charcoal	Yes	No	Yes	Yes	Yes	No	No
1	Mainly fire	No	Yes	No	No	Yes	No	No
0.5	Mainly fire	Yes	No	Yes	No	Yes	No	No
	Mainly fire	Yes	No	No	No	Yes	No	No
	Animal dur	Yes	No	No	Yes	Yes	No	No
	Mainly fire	Yes	No	No	No	Yes	No	No
0.75	Mainly fire	Yes	No	No	No	No	No	No
	Charcoal	Yes	No	Yes	Yes	Yes	No	No
	Mainly fire	Yes	No	No	Yes	Yes	No	No
	Mainly fire	No	Yes	Yes	No	Yes	No	No
0.25	Mainly fire	Yes	No	No	No	Yes	No	No
1	Mainly fire	Yes	No	No	No	Yes	No	No
	Mainly fire	Yes	No	Yes	Yes	Yes	No	No
	Mainly fire	Yes	Yes	Yes	Yes	Yes	No	Yes
1.5	Mainly fire	Yes	No	Yes	Yes	Yes	No	Yes
1	Mainly fire	Yes	No	No	No	Yes	No	No
0.75	Mainly fire	Yes	Yes	Yes	Yes	Yes	No	No
	Mainly fire	Yes	No	No	No	Yes	No	No
1	Mainly fire	No	No	Yes	No	Yes	No	No
	Mainly fire	Yes	Yes	No	Yes	Yes	No	No
0.75	Mainly fire	No	No	No	No	Yes	No	No
	Mainly fire	Yes	No	Yes	No	Yes	No	No
	Mainly fire	Yes	No	No	Yes	Yes	No	No
1	Mainly fire	Yes	No	Yes	No	Yes	No	No
	Mainly fire	No	No	No	No	Yes	No	No
	Mainly fire	Yes	No	No	Yes	Yes	Yes	No
4	Mainly fire	Yes	No	No	No	Yes	No	No
1	Mainly fire	Yes	No	No	No	Yes	No	No
1	Mainly fire	No	No	No	No	Yes	No	No
1	Mainly fire	Yes	No	No	No	Yes	No	No
	Charcoal	Yes	No	No	Yes	Yes	No	No
	Charcoal	Yes	No	No	Yes	Yes	No	No
0.5	Charcoal	Yes	No	Yes	Yes	Yes	No	No
2	Mainly fire	Yes	No	No	No	No	No	No
	Mainly fire	Yes	No	No	Yes	Yes	No	Yes
0.5	Mainly fire	No	Yes	Yes	No	Yes	No	No
1	Mainly fire	No	No	Yes	No	Yes	No	No
0.5	Mainly fire	Yes	No	Yes	Yes	Yes	No	No
2	Mainly fire	Yes	No	Yes	No	Yes	No	No
3	Mainly fire	Yes	No	Yes	No	Yes	No	No
0.5	Mainly fire	Yes	No	Yes	Yes	Yes	No	Yes
	Mainly fire	Yes	No	No	Yes	Yes	No	Yes
6	Mainly fire	Yes	No	No	No	Yes	No	No
999	Mainly fire	Yes	No	No	No	No	No	No
1	Mainly fire	Yes	No	Yes	Yes	Yes	No	No
0.75	Mainly fire	Yes	No	No	No	Yes	No	No
1	Mainly fire	No	No	No	No	No	No	No
0.5	Charcoal	Yes	No	No	Yes	Yes	No	No
1	Mainly fire	Yes	No	No	No	Yes	No	No
1	Mainly fire	No	No	Yes	No	Yes	No	No

For peer review only

	Mainly fire	Yes	Yes	No	Yes	Yes	No	No
1	Mainly fire	Yes	No	No	No	Yes	No	No
1	Animal dur	No	No	No	No	Yes	No	No
	Mainly fire	Yes	No	No	Yes	Yes	No	Yes
	Mainly fire	Yes	No	Yes	Yes	Yes	Yes	No
0.5	Animal dur	Yes	No	No	No	Yes	No	No
	Mainly fire	No	No	Yes	No	Yes	No	No
1	Animal dur	Yes	No	Yes	Yes	Yes	No	No
0.5	Animal dur	Yes	Yes	No	No	Yes	No	No
	Mainly fire	Yes	No	No	No	Yes	No	No
3	Animal dur	Yes	No	Yes	No	Yes	No	No
0.5	Animal dur	Yes	Yes	No	Yes	Yes	No	No
	Charcoal	No	No	No	No	Yes	No	No
0.25	Mainly fire	Yes	No	No	No	Yes	No	No
	Mainly fire	No	No	Yes	No	Yes	No	No
0.5	Animal dur	Yes	Yes	Yes	No	Yes	No	No
1.5	Mainly fire	No	No	No	No	No	No	No
	Mainly fire	Yes	No	No	Yes	Yes	No	No
1	Animal dur	No	No	Yes	No	Yes	No	No
	Charcoal	Yes	No	No	Yes	Yes	No	No
1	Mainly fire	No	No	No	No	No	No	No
	Mainly fire	Yes	No	Yes	Yes	Yes	No	No
1	Animal dur	No	No	Yes	No	Yes	No	No
0.75	Animal dur	No	No	Yes	No	Yes	No	No
0.25	Animal dur	Yes	No	No	No	Yes	No	No
0.5	Mainly fire	Yes	No	No	Yes	Yes	No	No
0.375	Mainly fire	Yes	Yes	Yes	No	Yes	No	No
2	Animal dur	No	No	Yes	No	Yes	No	No
0.5	Animal dur	Yes	No	No	No	Yes	No	No
4	Mainly fire	Yes	No	No	No	Yes	No	No
0.5	Mainly fire	Yes	Yes	Yes	Yes	Yes	No	No
1.25	Animal dur	No	No	No	No	Yes	No	No
1.5	Animal dur	No	No	Yes	No	Yes	No	No
0.5	Charcoal	Yes	No	Yes	No	Yes	No	No
0.5	Mainly fire	No	No	No	No	Yes	No	No
0.5	Animal dur	Yes	Yes	No	No	No	No	No
1	Mainly fire	Yes	No	Yes	No	Yes	No	No
1	Mainly fire	Yes	No	Yes	No	No	No	No
6	Mainly fire	Yes	No	No	No	Yes	No	No
0.5	Animal dur	No	No	No	No	Yes	No	No
0.75	Mainly fire	No	No	No	No	Yes	No	No
0.5	Animal dur	No	No	Yes	No	Yes	No	No
0.75	Mainly fire	Yes	No	No	No	Yes	No	No
	Electricity	Yes	No	Yes	Yes	Yes	No	No
0.25	Mainly fire	Yes	No	No	No	Yes	No	No
0.75	Animal dur	Yes	No	Yes	Yes	Yes	No	No
0.5	Mainly fire	No	No	Yes	No	Yes	No	No
0.5	Mainly fire	Yes	Yes	Yes	Yes	Yes	No	No
0.3	Animal dur	No	No	No	No	Yes	No	No
0.5	Mainly fire	Yes	No	Yes	No	Yes	No	No
4	Mainly fire	Yes	No	No	No	Yes	No	No
0.5	Animal dur	No	No	Yes	No	Yes	No	No

For peer review only

1	Mainly fire	Yes	No	No	No	Yes	No	No
0	Animal dur	Yes	No	Yes	No	Yes	No	No
1	Animal dur	No	No	No	No	Yes	No	No
0.5	Mainly fire	No	No	Yes	No	Yes	No	No
0.75	Animal dur	Yes	No	Yes	No	Yes	No	No
	Animal dur	Yes	No	No	Yes	Yes	No	No
	Animal dur	Yes	Yes	No	No	Yes	No	No
0.2	Animal dur	Yes	No	Yes	Yes	Yes	No	No
2	Mainly fire	Yes	No	No	No	Yes	No	No
0.5	Mainly fire	Yes	No	Yes	No	Yes	No	No
1	Mainly fire	Yes	No	Yes	Yes	Yes	No	No
2	Mainly fire	Yes	No	Yes	No	Yes	No	No
0.5	Mainly fire	Yes	No	Yes	Yes	Yes	No	No
	Mainly fire	No	No	Yes	No	Yes	No	No
1	Mainly fire	No	No	Yes	No	Yes	No	No
	Mainly fire	Yes	No	Yes	Yes	Yes	No	No
1	Animal dur	Yes	No	Yes	No	Yes	No	No
	Mainly fire	No	No	Yes	No	Yes	No	No
0.2	Animal dur	Yes	No	Yes	No	Yes	No	No
	Charcoal	Yes	No	No	Yes	Yes	No	No
0.5	Animal dur	Yes	Yes	Yes	No	Yes	No	No
	Animal dur	Yes	No	No	Yes	Yes	No	No
0.5	Mainly fire	Yes	No	No	No	Yes	No	No
4	Mainly fire	Yes	No	No	No	Yes	No	No
0.5	Mainly fire	No	No	No	No	No	No	No
0.75	Mainly fire	No	No	Yes	No	Yes	No	No
	Mainly fire	Yes	No	Yes	Yes	Yes	No	No
	Animal dur	Yes	Yes	Yes	Yes	Yes	No	No
0.5	Mainly fire	Yes	No	No	No	Yes	No	No
0.5	Mainly fire	No	No	No	No	No	No	No
1	Mainly fire	Yes	No	Yes	No	Yes	No	No
0.3	Animal dur	Yes	No	Yes	No	Yes	No	No
	Mainly fire	Yes	No	No	No	No	No	No
0.75	Mainly fire	Yes	No	No	Yes	Yes	No	No
0.5	Mainly fire	Yes	No	Yes	No	Yes	No	No
0.5	Animal dur	Yes	No	Yes	No	Yes	No	No
0.75	Mainly fire	No	No	No	No	Yes	No	No
2	Mainly fire	Yes	No	Yes	Yes	Yes	No	No
1.5	Animal dur	Yes	No	No	No	Yes	No	No
0.75	Mainly fire	No	No	Yes	No	Yes	No	No
0.5	Animal dur	Yes	No	No	No	Yes	No	No
0.5	Animal dur	Yes	No	No	No	No	No	No
3.5	Animal dur	No	No	Yes	No	Yes	No	No
0.5	Mainly fire	No	No	No	No	No	No	No
0.75	Mainly fire	Yes	No	No	No	Yes	No	No
1	Animal dur	Yes	No	Yes	Yes	Yes	No	No
0.5	Animal dur	Yes	No	Yes	No	Yes	No	No
8	Mainly fire	Yes	No	No	Yes	Yes	No	No
	Mainly fire	Yes	No	Yes	No	Yes	No	No
	Charcoal	Yes	Yes	No	No	Yes	No	No
0	Animal dur	Yes	No	No	No	Yes	No	No
	Charcoal	No	No	Yes	No	No	No	No

For peer review only

0.5	Mainly fire	Yes	No	Yes	No	Yes	No	No
0.75	Animal dur	Yes	No	Yes	No	Yes	No	No
0.25	Mainly fire	No	No	No	No	Yes	No	No
0.5	Mainly fire	Yes	No	Yes	No	Yes	No	No
0.5	Animal dur	Yes	No	Yes	Yes	Yes	No	No
0.5	Mainly fire	Yes	No	No	No	Yes	No	No
0.5	Animal dur	Yes	Yes	Yes	Yes	Yes	No	No
2	Animal dur	No	No	Yes	No	Yes	No	No
	Mainly fire	Yes	No	No	Yes	Yes	No	No
0.5	Mainly fire	Yes	No	Yes	No	Yes	No	No
0.2	Mainly fire	No	No	Yes	No	No	No	No
	Mainly fire	No	No	Yes	No	Yes	No	No
0.65	Animal dur	No	No	No	No	Yes	No	No
0.5	Mainly fire	Yes	Yes	Yes	No	Yes	No	No
1	Mainly fire	No	No	Yes	No	Yes	No	No
0.25	Charcoal	Yes	Yes	Yes	No	Yes	No	No
0.5	Animal dur	Yes	Yes	Yes	No	Yes	No	No
0.25	Mainly fire	No	No	No	No	No	No	No
0.1	Animal dur	Yes	No	Yes	No	Yes	No	No
0.5	Mainly fire	Yes	No	Yes	No	Yes	No	No
0.75	Mainly fire	No	No	Yes	No	Yes	No	No
	Mainly fire	Yes	No	No	No	No	No	No
2	Mainly fire	Yes	No	No	No	Yes	No	No
2	Mainly fire	No	No	No	No	No	No	No
	Mainly fire	No	No	No	No	Yes	No	No
	Mainly fire	Yes	No	Yes	No	Yes	No	No
1.25	Mainly fire	No	No	Yes	No	Yes	No	No
	Animal dur	No	No	Yes	No	Yes	No	No
1	Animal dur	Yes	No	Yes	No	Yes	No	No
1.5	Mainly fire	Yes	No	No	No	Yes	No	No
1	Animal dur	Yes	No	Yes	No	Yes	No	No
0.5	Animal dur	Yes	Yes	No	No	Yes	No	No
0.75	Mainly fire	Yes	No	No	No	Yes	No	No
	Mainly fire	No	Yes	Yes	No	Yes	No	No
1.5	Mainly fire	Yes	No	No	No	Yes	No	No
	Mainly fire	No	No	Yes	No	No	No	No
0.5	Mainly fire	No	No	Yes	No	Yes	No	No
0.5	Animal dur	Yes	No	Yes	No	Yes	No	No
	Mainly fire	Yes	No	No	Yes	Yes	No	No
1	Mainly fire	Yes	No	Yes	No	Yes	No	No
0.25	Mainly fire	No	No	No	No	Yes	No	No
3	Mainly fire	No	No	Yes	No	Yes	No	No
	Mainly fire	Yes	No	No	Yes	Yes	No	No
0.5	Charcoal	Yes	No	Yes	Yes	Yes	No	Yes
0.5	Mainly fire	Yes	No	No	Yes	Yes	No	No
1	Mainly fire	Yes	No	Yes	No	Yes	No	No
0.5	Mainly fire	Yes	No	Yes	No	No	No	No
0.5	Mainly fire	No	No	Yes	No	Yes	No	No
	Mainly fire	Yes	Yes	Yes	Yes	Yes	No	No
	Mainly fire	Yes	Yes	Yes	No	Yes	No	No
0.75	Mainly fire	Yes	No	Yes	No	Yes	No	No
0.5	Animal dur	No	No	No	No	Yes	No	No

For peer review only

0.5	Animal dur	No	No	No	No	Yes	No	No
	Mainly fire	No	No	Yes	No	Yes	No	No
2	Mainly fire	No	No	No	No	No	No	No
0.5	Mainly fire	No	No	Yes	No	Yes	No	No
1	Mainly fire	No	No	No	No	No	No	No
1	Animal dur	No	No	Yes	No	No	No	No
	Charcoal	Yes	No	No	Yes	Yes	No	No
1	Animal dur	No	No	Yes	No	Yes	No	No
1.5	Mainly fire	No	No	Yes	No	Yes	No	No
0.25	Mainly fire	Yes	No	No	No	No	No	No
0.5	Charcoal	Yes	No	Yes	Yes	Yes	No	No
0.5	Mainly fire	Yes	No	No	No	Yes	No	No
0.5	Mainly fire	No	No	Yes	No	Yes	No	No
1.1	Animal dur	Yes	No	No	Yes	Yes	No	No
0.5	Mainly fire	Yes	No	No	No	Yes	No	No
0.5	Mainly fire	Yes	No	No	No	Yes	No	No
1	Animal dur	No	No	Yes	No	Yes	No	No
0.5	Mainly fire	Yes	No	No	No	Yes	No	No
0.7	Mainly fire	Yes	No	No	No	Yes	No	No
	Animal dur	Yes	Yes	No	No	No	No	No
	Mainly fire	Yes	No	No	Yes	Yes	No	No
1.1	Mainly fire	Yes	Yes	No	No	Yes	No	No
0.75	Mainly fire	No	No	No	No	Yes	No	No
	Mainly fire	No	No	No	No	No	No	No
0.5	Mainly fire	No	No	No	No	No	No	No
5	Charcoal	Yes	Yes	Yes	Yes	Yes	No	No
0.25	Charcoal	Yes	No	No	Yes	Yes	No	No
1	Animal dur	No	No	No	No	No	No	No
0.25	Mainly fire	No	No	Yes	No	Yes	No	No
1	Animal dur	No	No	Yes	No	Yes	No	No
1	Animal dur	Yes	No	Yes	No	Yes	No	No
0.5	Mainly fire	Yes	No	No	No	No	No	No
0.5	Animal dur	No	No	No	No	Yes	No	No
	Mainly fire	Yes	No	No	No	No	No	No
2	Mainly fire	No	Yes	Yes	No	Yes	No	No
0.1	Animal dur	Yes	No	Yes	No	No	Yes	No
	Mainly fire	No	No	Yes	No	No	No	No
1.5	Mainly fire	No	No	No	No	Yes	No	No
3	Animal dur	No	No	No	No	Yes	No	No
1	Mainly fire	Yes	No	Yes	No	No	No	No
	Animal dur	No	No	Yes	No	Yes	No	No
1	Mainly fire	Yes	No	Yes	No	Yes	No	No
	Mainly fire	Yes	No	Yes	Yes	Yes	No	No
0.25	Mainly fire	Yes	No	No	No	Yes	No	No
0.3	Animal dur	Yes	No	Yes	No	No	No	No
0.2	Animal dur	Yes	No	Yes	No	No	No	No
1.5	Mainly fire	Yes	No	No	No	Yes	No	No
2	Mainly fire	Yes	Yes	Yes	No	Yes	No	No
0.2	Animal dur	Yes	Yes	Yes	No	Yes	No	No
1	Mainly fire	No	No	Yes	No	Yes	No	No
0.3	Animal dur	Yes	No	Yes	No	No	No	No
0.5	Mainly fire	Yes	No	No	No	Yes	No	No

For peer review only

	Animal dur	No	No	Yes	No	Yes	No	No
1	Mainly fire	Yes	No	Yes	No	Yes	No	No
0.25	Animal dur	Yes	No	Yes	No	Yes	No	No
0.2	Animal dur	No	No	Yes	No	Yes	No	No
2	Mainly fire	No	No	No	No	Yes	No	No
1	Mainly fire	Yes	No	No	No	No	No	No
1	Mainly fire	Yes	No	No	No	No	No	No
1.5	Mainly fire	No	Yes	Yes	No	Yes	No	No
0.25	Animal dur	Yes	No	Yes	No	Yes	No	No
3	Mainly fire	Yes	No	No	No	Yes	No	No
	Animal dur	No	No	Yes	No	Yes	No	No
0.25	Mainly fire	No	No	No	No	No	No	No
1	Mainly fire	No	No	Yes	No	Yes	No	No
0.5	Mainly fire	No	Yes	Yes	No	Yes	No	No
0.75	Mainly fire	Yes	No	No	No	Yes	No	No
	Mainly fire	Yes	Yes	Yes	Yes	Yes	No	No
0.75	Mainly fire	Yes	No	Yes	No	Yes	No	No
0.5	Animal dur	Yes	No	Yes	No	Yes	No	No
1	Mainly fire	No	No	No	No	No	No	No
0.5	Animal dur	Yes	No	No	No	Yes	No	No
	Charcoal	Yes	Yes	No	Yes	Yes	No	No
0.75	Mainly fire	No	No	Yes	No	Yes	No	No
	Electricity	Yes	No	No	Yes	Yes	No	No
1	Mainly fire	Yes	No	No	No	No	No	No
0.5	Mainly fire	No	No	No	No	No	No	No
1	Mainly fire	Yes	No	No	No	No	No	No
	Mainly fire	Yes	No	No	No	Yes	No	No
0.5	Mainly fire	No	No	Yes	Yes	Yes	No	No
1	Mainly fire	Yes	No	Yes	No	Yes	No	No
	Mainly fire	Yes	No	No	No	Yes	No	No
1	Animal dur	Yes	Yes	No	No	Yes	No	No
2	Animal dur	Yes	No	Yes	No	Yes	No	No
0.5	Animal dur	No	No	No	No	Yes	No	No
0.5	Mainly fire	No	No	Yes	No	No	No	No
0.5	Mainly fire	Yes	Yes	Yes	No	Yes	No	No
	Mainly fire	No	Yes	Yes	No	Yes	No	No
0.5	Mainly fire	No	No	Yes	No	Yes	No	No
0.375	Charcoal	Yes	Yes	Yes	No	Yes	No	No
1	Mainly fire	No	No	Yes	No	Yes	No	No
0.5	Mainly fire	No	No	Yes	No	Yes	No	No
0.1	Kerosene	No	No	Yes	No	Yes	No	No
0.2	Animal dur	Yes	No	Yes	No	Yes	No	No
	Charcoal	Yes	No	No	Yes	Yes	No	No
	Charcoal	Yes	No	No	Yes	Yes	No	No
	Animal dur	No	No	No	No	Yes	No	No
	Mainly fire	No	No	Yes	No	Yes	No	No
	Mainly fire	Yes	No	Yes	No	Yes	No	No
4	Mainly fire	Yes	No	No	No	No	No	No
0.2	Animal dur	Yes	No	Yes	No	Yes	No	No
0.3	Animal dur	Yes	No	No	No	Yes	No	No
0.5	Mainly fire	Yes	No	No	No	No	No	No
	Mainly fire	Yes	No	Yes	Yes	Yes	No	No

For peer review only

0.5	Mainly fire	Yes	Yes	Yes	No	Yes	No	No
2	Animal dur	Yes	No	Yes	No	Yes	No	No
0.1	Animal dur	No	No	Yes	No	Yes	No	No
1.25	Mainly fire	No	No	Yes	No	Yes	No	No
0.75	Mainly fire	No	No	Yes	No	Yes	No	No
1	Animal dur	Yes	No	Yes	No	Yes	No	No
0.1	Animal dur	Yes	Yes	Yes	No	Yes	No	No
0.5	Animal dur	Yes	No	Yes	Yes	Yes	No	No
0.5	Mainly fire	No	No	Yes	No	Yes	No	No
1	Mainly fire	Yes	No	No	No	No	No	No
0.5	Animal dur	Yes	No	Yes	No	Yes	No	No
0.5	Mainly fire	Yes	No	Yes	No	Yes	No	No
0.5	Mainly fire	Yes	No	Yes	No	No	No	No
0.625	Animal dur	Yes	No	Yes	No	Yes	No	No
1	Animal dur	Yes	No	Yes	No	Yes	No	No
1	Animal dur	No	No	Yes	No	Yes	No	No
	Mainly fire	No	No	Yes	No	Yes	No	No
0.25	Animal dur	Yes	No	No	No	Yes	No	No
0.75	Mainly fire	Yes	No	No	No	Yes	No	No
0.75	Animal dur	Yes	No	Yes	No	Yes	No	No
0.25	Mainly fire	Yes	No	No	No	Yes	No	No
	Mainly fire	Yes	Yes	Yes	No	Yes	No	No
	Mainly fire	Yes	No	No	Yes	Yes	No	No
1	Animal dur	No	No	No	No	Yes	No	No
0.25	Animal dur	Yes	No	Yes	No	Yes	No	No
1	Animal dur	No	No	No	No	Yes	No	No
0.5	Animal dur	No	No	Yes	No	Yes	No	No
1	Animal dur	No	No	Yes	No	Yes	No	No
1	Mainly fire	Yes	No	Yes	No	Yes	Yes	No
	Mainly fire	Yes	No	Yes	No	Yes	No	No
2	Mainly fire	Yes	No	No	No	Yes	No	No
0.25	Animal dur	Yes	Yes	No	No	Yes	No	No
0.75	Mainly fire	No	No	Yes	No	Yes	No	No
0.75	Charcoal	Yes	No	No	Yes	Yes	No	Yes
	Mainly fire	No	No	Yes	No	Yes	No	No
0.1	Animal dur	Yes	No	No	No	Yes	No	No
0.5	Animal dur	Yes	No	Yes	No	Yes	No	No
0.5	Animal dur	Yes	No	No	No	Yes	No	No
0.75	Mainly fire	No	No	No	No	Yes	No	No
0.5	Animal dur	No	No	Yes	No	Yes	No	No
	Mainly fire	No	No	Yes	No	Yes	No	No
0.2	Mainly fire	Yes	No	Yes	No	Yes	No	No
1	Mainly fire	Yes	No	No	No	Yes	No	No
3	Mainly fire	Yes	No	No	No	Yes	No	No
0.2	Animal dur	No	No	Yes	No	Yes	No	No
0.75	Mainly fire	Yes	No	Yes	No	Yes	No	No
0.5	Mainly fire	Yes	Yes	Yes	Yes	Yes	No	No
	Mainly fire	No	No	No	No	Yes	No	No
	Mainly fire	Yes	Yes	No	Yes	Yes	No	No
0.2	Mainly fire	Yes	No	No	No	Yes	No	No
1	Mainly fire	No	No	No	No	Yes	No	No
1	Animal dur	No	No	No	No	Yes	No	No

For peer review only

0.25	Animal dur	No	No	No	No	Yes	No	No
0.75	Animal dur	No	No	No	No	Yes	No	No
1.5	Mainly fire'	No	No	No	No	Yes	No	No
1	Mainly fire'	Yes	No	No	No	Yes	No	No
2	Mainly fire'	No	No	Yes	No	Yes	No	No
0.2	Mainly fire'	Yes	No	Yes	No	Yes	No	No
1	Mainly fire'	No	No	Yes	No	Yes	No	No
1	Mainly fire'	Yes	No	Yes	No	Yes	No	No
0.1	Mainly fire'	Yes	No	Yes	No	Yes	No	No
0	Animal dur	Yes	No	No	No	Yes	No	No
0.75	Animal dur	Yes	No	Yes	No	Yes	No	No
0.5	Animal dur	No	No	Yes	No	Yes	No	No
1	Mainly fire'	Yes	No	Yes	No	Yes	No	No
0.25	Mainly fire'	Yes	No	Yes	No	Yes	No	No
0.25	Mainly fire'	Yes	Yes	Yes	No	Yes	No	No
0.5	Mainly fire'	Yes	No	Yes	No	Yes	No	No
	Mainly fire'	No	No	No	No	Yes	No	No
	Mainly fire'	Yes	No	No	No	No	No	No
	Animal dur	Yes	No	Yes	Yes	Yes	No	No
1	Mainly fire'	Yes	Yes	Yes	No	Yes	No	No
	Animal dur	Yes	No	Yes	No	Yes	No	No
	Mainly fire'	Yes	Yes	Yes	Yes	Yes	No	No
0.5	Mainly fire'	Yes	No	No	No	Yes	No	No
	Mainly fire'	Yes	Yes	Yes	Yes	Yes	No	Yes
0.75	Animal dur	Yes	No	No	No	Yes	No	No
0.5	Animal dur	No	No	No	No	Yes	No	No
0.5	Mainly fire'	Yes	No	No	No	Yes	No	No
0.5	Mainly fire'	Yes	No	Yes	No	Yes	No	No
	Charcoal	Yes	No	No	Yes	Yes	No	No
0.5	Mainly fire'	No	No	No	No	Yes	No	No
0.1	Animal dur	Yes	No	No	No	Yes	No	No
1	Animal dur	No	No	Yes	Yes	Yes	No	No
0.5	Mainly fire'	Yes	Yes	Yes	Yes	Yes	No	No
	Animal dur	Yes	No	Yes	No	Yes	No	No
0.5	Animal dur	No	No	Yes	No	Yes	No	No
1	Animal dur	No	No	Yes	No	Yes	No	No
	Animal dur	Yes	No	Yes	Yes	Yes	No	No
	Mainly fire'	No	Yes	Yes	No	Yes	No	No
0.5	Mainly fire'	Yes	No	Yes	No	Yes	No	No
0.25	Mainly fire'	Yes	No	No	No	Yes	No	No
0.5	Animal dur	Yes	Yes	No	No	Yes	No	No
	Animal dur	No	No	Yes	No	Yes	No	No
1	Mainly fire'	Yes	No	No	No	Yes	No	No
0.5	Mainly fire'	No	No	Yes	No	Yes	No	No
0.5	Animal dur	Yes	No	No	No	Yes	No	No
0.1	Mainly fire'	Yes	No	Yes	No	Yes	No	No
0.5	Mainly fire'	Yes	Yes	Yes	Yes	No	No	No
0.5	Mainly fire'	No	No	Yes	No	Yes	No	No
0.5	Mainly fire'	No	No	Yes	No	Yes	No	No
0.5	Mainly fire'	No	No	No	No	No	No	No
	Animal dur	No	No	No	No	No	No	No
1	Animal dur	Yes	No	Yes	No	Yes	No	No

For peer review only

	Mainly fire	No	No	Yes	No	Yes	No	No
4	Mainly fire	Yes	No	No	No	No	No	No
4	Mainly fire	Yes	No	No	No	Yes	No	No
0.25	Charcoal	Yes	No	No	No	Yes	No	No
0.25	Animal dur	Yes	No	Yes	No	Yes	No	No
	Mainly fire	Yes	Yes	Yes	No	Yes	No	No
	Mainly fire	No	No	Yes	Yes	Yes	No	No
0.5	Animal dur	Yes	Yes	No	No	Yes	No	No
1.5	Mainly fire	No	Yes	No	No	No	No	No
0	Animal dur	Yes	No	No	No	Yes	No	No
1	Mainly fire	Yes	No	No	No	No	No	No
	Animal dur	No	No	No	No	No	No	No
0.5	Animal dur	Yes	No	Yes	No	Yes	No	No
1	Mainly fire	Yes	No	No	No	Yes	No	No
2	Mainly fire	No	No	No	No	No	No	No
4	Mainly fire	Yes	No	No	No	Yes	No	No
	Mainly fire	Yes	No	No	No	Yes	No	No
0.5	Mainly fire	No	No	No	No	No	No	No
1	Animal dur	No	No	Yes	No	Yes	No	No
	Mainly fire	Yes	No	No	No	Yes	No	No
	Mainly fire	No	No	Yes	No	Yes	No	No
	Mainly fire	Yes	No	No	No	Yes	No	No
1	Mainly fire	Yes	No	No	No	No	No	No
1.25	Crop residu	Yes	No	No	No	Yes	No	No
1.5	Mainly fire	Yes	No	No	No	Yes	No	No
	Mainly fire	Yes	No	No	No	No	No	No
	Charcoal	Yes	No	No	Yes	No	No	No
0.5	Mainly fire	Yes	No	No	No	No	No	No
	Mainly fire	Yes	No	No	No	Yes	No	No
1.25	Crop residu	Yes	No	No	No	Yes	No	No
1	Mainly fire	No	No	Yes	No	Yes	No	No
0.5	Mainly fire	Yes	No	No	No	Yes	No	No
0.5	Mainly fire	Yes	No	Yes	No	Yes	No	No
2	Mainly fire	Yes	No	No	No	Yes	No	No
1	Crop residu	Yes	No	Yes	No	Yes	No	No
0.5	Mainly fire	Yes	No	No	No	No	No	No
1	Mainly fire	Yes	No	No	No	Yes	No	No
1	Crop residu	Yes	No	No	No	Yes	No	No
0.5	Mainly fire	Yes	No	No	No	Yes	No	No
1	Mainly fire	No	No	No	No	Yes	No	No
	Mainly fire	Yes	No	No	No	No	No	No
2	Mainly fire	Yes	No	No	No	Yes	No	No
1	Crop residu	Yes	No	No	No	Yes	No	No
1.5	Mainly fire	No	No	No	No	Yes	No	No
999	Mainly fire	Yes	No	No	No	No	No	No
1	Mainly fire	Yes	No	Yes	No	Yes	No	No
1	Mainly fire	Yes	No	Yes	No	Yes	No	No
0.75	Crop residu	No	No	No	No	Yes	No	No
2	Mainly fire	Yes	No	No	No	No	No	No
0.25	Mainly fire	Yes	No	No	No	No	No	No
	Mainly fire	Yes	No	No	No	Yes	No	No
2	Mainly fire	Yes	No	No	No	Yes	No	No

For peer review only

2	Mainly fire	Yes	No	No	No	Yes	No	No
1	Mainly fire	Yes	No	Yes	No	Yes	No	No
0.5	Crop residu	Yes	No	No	No	Yes	No	No
2	Mainly fire	No	No	No	No	No	No	No
1.5	Mainly fire	Yes	No	No	No	Yes	No	No
1.5	Mainly fire	Yes	No	No	No	No	No	No
0.25	Mainly fire	Yes	No	No	No	Yes	No	No
1.5	Mainly fire	Yes	No	Yes	No	Yes	No	No
3	Crop residu	Yes	No	No	No	Yes	No	No
1	Crop residu	Yes	No	No	No	Yes	No	No
0.5	Mainly fire	Yes	No	No	No	No	No	No
1	Crop residu	Yes	No	No	No	Yes	No	No
1	Mainly fire	Yes	No	No	No	Yes	No	No
2	Mainly fire	Yes	No	No	No	Yes	No	No
2.5	Mainly fire	Yes	No	No	No	No	No	No
0.5	Mainly fire	No	No	No	No	Yes	No	No
	Mainly fire	No	No	No	No	Yes	No	No
1	Crop residu	Yes	No	No	No	No	No	No
2	Crop residu	Yes	No	No	No	Yes	No	No
2	Crop residu	Yes	No	No	No	Yes	No	No
1.5	Crop residu	No	No	No	No	No	No	No
	Mainly fire	Yes	No	No	No	No	No	No
	Mainly fire	Yes	No	No	No	Yes	No	No
3	Crop residu	Yes	No	No	No	Yes	No	No
	Mainly fire	Yes	No	No	No	No	No	No
2	Mainly fire	Yes	No	No	No	Yes	No	Yes
1	Crop residu	Yes	No	No	No	Yes	No	No
	Mainly fire	No	No	No	No	No	No	No
1	Crop residu	Yes	No	No	No	Yes	No	No
1.5	Mainly fire	Yes	No	No	No	No	No	No
	Mainly fire	Yes	No	No	No	Yes	No	No
0.75	Crop residu	Yes	No	No	No	No	No	No
1.5	Crop residu	Yes	No	No	No	No	No	No
0.5	Crop residu	Yes	No	No	No	Yes	No	No
999	Mainly fire	Yes	No	No	No	Yes	No	No
1	Mainly fire	Yes	No	No	No	No	No	No
	Mainly fire	Yes	No	No	Yes	Yes	No	No
	Mainly fire	No	No	No	No	No	No	No
999	Mainly fire	Yes	No	No	No	No	No	No
3	Mainly fire	Yes	No	No	No	Yes	No	No
1.5	Mainly fire	Yes	No	No	No	No	No	No
0.5	Mainly fire	Yes	No	Yes	No	Yes	No	No
2	Mainly fire	Yes	No	No	No	Yes	No	No
	Mainly fire	Yes	No	No	No	Yes	No	No
1	Crop residu	Yes	No	No	No	Yes	No	No
	Mainly fire	Yes	No	No	No	Yes	No	No
0.5	Mainly fire	Yes	No	Yes	No	Yes	No	No
1	Crop residu	Yes	No	No	No	Yes	No	No
	Mainly fire	Yes	No	No	Yes	Yes	No	No
0.75	Mainly fire	Yes	No	No	No	No	No	No
1.5	Mainly fire	Yes	No	No	No	No	No	No
999	Mainly fire	Yes	No	No	No	No	No	No

For peer review only

1.5	Mainly fire	Yes	No	No	No	Yes	No	No
	Mainly fire	Yes	No	No	No	Yes	No	No
1	Mainly fire	Yes	No	Yes	No	Yes	No	No
2	Mainly fire	No	No	No	No	No	No	No
1	Mainly fire	Yes	No	No	No	No	No	No
999	Mainly fire	Yes	No	No	No	Yes	Yes	No
	Mainly fire	Yes	Yes	No	Yes	Yes	No	No
1	Crop residu	No	No	No	No	Yes	No	No
999	Mainly fire	Yes	No	Yes	No	No	No	No
0.5	Mainly fire	No	No	No	No	No	No	No
0.5	Mainly fire	Yes	No	Yes	No	Yes	No	No
	Mainly fire	Yes	No	No	No	Yes	No	No
	Mainly fire	Yes	No	No	No	Yes	No	No
1.5	Mainly fire	Yes	No	No	No	Yes	No	No
1.5	Mainly fire	Yes	No	No	No	Yes	No	No
	Mainly fire	Yes	No	No	No	Yes	No	No
0.5	Mainly fire	Yes	No	Yes	No	Yes	No	No
1	Mainly fire	No	No	No	No	No	No	No
999	Mainly fire	No	No	No	No	No	No	No
1	Mainly fire	Yes	No	Yes	No	Yes	No	No
	Mainly fire	Yes	No	No	No	Yes	No	No
1	Crop residu	Yes	No	No	No	Yes	No	No
	Mainly fire	Yes	No	No	Yes	Yes	No	No
	Mainly fire	Yes	No	No	No	Yes	No	No
1	Crop residu	Yes	No	No	No	Yes	No	No
999	Mainly fire	Yes	No	No	No	Yes	No	No
1	Mainly fire	Yes	No	No	No	Yes	No	No
1	Mainly fire	Yes	No	Yes	No	Yes	No	No
3	Mainly fire	Yes	No	No	No	No	No	No
1	Mainly fire	Yes	No	No	No	Yes	No	No
2	Mainly fire	Yes	No	No	No	Yes	No	No
2	Crop residu	Yes	No	No	No	Yes	No	No
1.5	Mainly fire	No	No	No	No	No	No	No
1	Mainly fire	Yes	No	No	No	Yes	No	No
1.5	Crop residu	Yes	No	No	No	Yes	No	No
1.5	Crop residu	Yes	No	No	No	Yes	No	No
1	Mainly fire	No	No	No	No	No	No	No
2	Mainly fire	No	No	No	No	No	No	No
0.5	Mainly fire	Yes	No	Yes	No	Yes	No	No
999	Crop residu	Yes	No	No	No	No	No	No
999	Crop residu	Yes	No	No	No	Yes	No	No
1	Mainly fire	Yes	No	No	No	Yes	No	No
0.25	Crop residu	Yes	No	No	No	Yes	No	No
0.5	Mainly fire	Yes	No	No	No	Yes	No	No
1	Mainly fire	Yes	No	No	No	No	No	No
999	Mainly fire	No	No	No	No	No	No	No
1	Mainly fire	Yes	No	Yes	No	Yes	No	No
1.25	Mainly fire	Yes	No	Yes	No	Yes	No	No
	Mainly fire	Yes	No	No	No	No	No	No
1	Crop residu	Yes	No	No	No	Yes	No	No
1	Mainly fire	Yes	No	Yes	No	Yes	No	No
3	Crop residu	Yes	No	No	No	Yes	No	No

For peer review only

	Mainly fire	No	No	No	No	No	No	No
1	Crop residu	Yes	No	No	No	Yes	No	No
2	Mainly fire	Yes	No	No	No	Yes	No	No
2	Mainly fire	Yes	No	No	No	Yes	No	No
1	Crop residu	Yes	No	No	No	Yes	No	No
1	Mainly fire	Yes	No	No	No	No	No	No
1.5	Crop residu	Yes	No	No	No	Yes	No	No
2	Mainly fire	Yes	No	Yes	No	Yes	No	No
2	Mainly fire	Yes	No	No	No	No	No	No
	Mainly fire	Yes	No	No	No	Yes	No	No
2	Mainly fire	Yes	No	No	No	No	No	No
	Mainly fire	Yes	No	Yes	No	Yes	No	No
2	Mainly fire	Yes	No	Yes	No	Yes	No	No
	Mainly fire	Yes	No	No	No	No	No	No
3	Mainly fire	Yes	No	No	No	Yes	No	No
1	Crop residu	Yes	No	No	No	No	No	No
0.75	Mainly fire	Yes	No	No	No	Yes	No	No
	Mainly fire	No	No	No	No	Yes	No	No
	Mainly fire	Yes	No	No	No	Yes	No	No
	Mainly fire	Yes	No	No	No	Yes	No	No
1	Mainly fire	Yes	No	No	No	Yes	No	No
1	Mainly fire	Yes	No	No	No	No	No	No
0.75	Crop residu	Yes	No	No	No	Yes	No	No
999	Mainly fire	Yes	No	No	No	No	No	No
2	Mainly fire	Yes	No	No	No	Yes	No	No
0.25	Mainly fire	Yes	No	No	No	Yes	No	No
1.5	Mainly fire	Yes	No	No	No	Yes	No	No
1.5	Mainly fire	Yes	No	No	No	No	No	No
2.5	Mainly fire	Yes	No	No	No	Yes	No	No
1	Mainly fire	Yes	No	Yes	No	Yes	No	No
	Mainly fire	Yes	Yes	No	No	Yes	No	No
	Mainly fire	Yes	No	No	No	Yes	No	No
	Mainly fire	Yes	Yes	Yes	No	Yes	No	No
	Mainly fire	Yes	No	No	No	No	No	No
0.5	Mainly fire	Yes	No	Yes	No	Yes	No	No
	Mainly fire	Yes	No	No	No	Yes	No	No
0.5	Mainly fire	Yes	Yes	Yes	No	Yes	No	No
2.75	Mainly fire	Yes	No	No	No	No	No	No
2	Mainly fire	Yes	No	No	No	No	No	No
1	Crop residu	Yes	No	No	No	Yes	No	No
1	Mainly fire	Yes	No	No	No	Yes	No	No
3	Mainly fire	Yes	No	No	No	Yes	No	No
	Mainly fire	Yes	No	Yes	No	Yes	No	No
1	Mainly fire	Yes	No	Yes	No	Yes	No	No
2	Mainly fire	Yes	No	No	No	Yes	No	No
	Mainly fire	No	No	Yes	No	Yes	No	No
	Mainly fire	Yes	No	No	Yes	Yes	No	No
999	Mainly fire	Yes	No	No	No	No	No	No
2	Mainly fire	No	No	Yes	No	Yes	No	No
0.5	Mainly fire	No	No	No	No	Yes	No	No
0.5	Mainly fire	Yes	No	Yes	No	Yes	No	No
2	Mainly fire	Yes	No	Yes	No	Yes	No	No

For peer review only

	Mainly fire	Yes	No	No	No	No	No	No
	Animal dur	Yes	No	Yes	No	Yes	No	No
2	Animal dur	Yes	Yes	Yes	Yes	Yes	No	No
	Mainly fire	Yes	No	No	No	No	No	No
	Mainly fire	Yes	No	No	No	Yes	No	No
999	Mainly fire	Yes	No	No	No	Yes	No	No
0.7	Mainly fire	No	No	Yes	No	Yes	No	No
0.75	Mainly fire	Yes	No	No	No	Yes	No	No
0.5	Mainly fire	No	No	No	No	Yes	No	No
0	Animal dur	No	No	No	No	Yes	No	No
	Animal dur	Yes	No	No	No	Yes	No	No
	Charcoal	Yes	Yes	Yes	Yes	Yes	No	No
0.5	Mainly fire	Yes	No	Yes	No	Yes	No	No
0.1	Animal dur	No	No	No	No	Yes	No	No
	Mainly fire	Yes	No	No	No	Yes	No	No
3	Mainly fire	Yes	No	Yes	Yes	Yes	No	No
0.25	Mainly fire	No	No	Yes	No	Yes	No	No
2	Mainly fire	No	No	No	No	No	No	No
	Mainly fire	Yes	No	No	No	No	No	No
0.5	Mainly fire	No	No	No	No	Yes	No	No
0.2	Animal dur	Yes	No	No	No	Yes	No	No
0.75	Mainly fire	Yes	Yes	Yes	No	Yes	No	No
0.5	Mainly fire	Yes	No	No	No	Yes	No	No
0.5	Mainly fire	Yes	No	Yes	No	No	No	No
0.25	Charcoal	Yes	No	No	Yes	Yes	No	No
3	Mainly fire	Yes	No	No	No	Yes	Yes	No
0.75	Mainly fire	No	No	No	No	Yes	No	No
0.5	Mainly fire	No	No	No	No	Yes	No	No
2	Mainly fire	Yes	No	No	No	No	No	No
1	Mainly fire	Yes	No	Yes	No	Yes	No	No
0.5	Mainly fire	No	No	No	No	Yes	No	No
0.75	Mainly fire	No	No	No	No	Yes	No	No
	Animal dur	No	No	No	No	No	No	No
	Mainly fire	No	No	No	No	Yes	No	No
	Mainly fire	Yes	No	Yes	No	Yes	No	No
0.25	Mainly fire	Yes	No	No	No	Yes	No	No
2.5	Mainly fire	Yes	No	No	No	Yes	No	No
	Mainly fire	Yes	No	No	No	No	No	No
0.2	Animal dur	Yes	No	No	No	Yes	No	No
	Mainly fire	Yes	No	Yes	No	Yes	No	No
0.25	Mainly fire	No	No	No	No	Yes	No	No
1	Mainly fire	Yes	No	Yes	No	Yes	No	No
0.25	Mainly fire	No	No	No	No	Yes	No	No
0.75	Mainly fire	Yes	No	No	No	No	No	No
	Mainly fire	No	No	No	No	No	No	No
1	Animal dur	Yes	No	Yes	No	Yes	No	No
0.1	Animal dur	Yes	No	No	Yes	Yes	No	No
1	Animal dur	No	No	Yes	No	Yes	No	No
	Mainly fire	No	No	No	No	Yes	No	No
3	Mainly fire	Yes	No	Yes	No	Yes	No	No
0.1	Animal dur	Yes	No	Yes	No	Yes	No	No
999	Mainly fire	Yes	No	No	No	No	No	No

For peer review only

0.25	Mainly fire	Yes	No	No	No	Yes	No	No
	Animal dur	No	No	Yes	No	Yes	No	No
1	Mainly fire	Yes	No	No	No	Yes	No	No
3	Mainly fire	Yes	No	Yes	No	Yes	No	No
	Electricity	Yes	Yes	Yes	Yes	Yes	No	Yes
4	Mainly fire	No	No	No	No	Yes	No	No
	Mainly fire	No	No	Yes	No	Yes	No	No
999	Mainly fire	Yes	No	No	No	No	No	No
0.1	Animal dur	Yes	No	No	No	Yes	No	No
1	Mainly fire	Yes	No	No	No	Yes	Yes	No
0.5	Animal dur	Yes	No	Yes	No	Yes	No	No
0.3	Mainly fire	No	No	Yes	No	Yes	No	No
1	Mainly fire	Yes	No	No	No	No	No	No
1	Mainly fire	Yes	No	No	No	Yes	No	No
0.7	Animal dur	No	No	Yes	No	Yes	No	No
	Mainly fire	Yes	No	Yes	No	Yes	No	No
0.5	Mainly fire	No	No	No	No	Yes	No	No
0.5	Mainly fire	Yes	No	No	No	Yes	No	No
	Mainly fire	No	No	No	No	No	No	No
0.25	Mainly fire	Yes	No	No	No	Yes	No	No
0.5	Mainly fire	Yes	No	No	No	Yes	No	No
2	Mainly fire	Yes	Yes	Yes	Yes	Yes	No	No
	Mainly fire	No	No	No	No	Yes	No	No
3	Mainly fire	No	No	Yes	No	No	No	No
1.5	Mainly fire	Yes	No	No	No	Yes	No	No
1	Animal dur	No	No	Yes	No	Yes	No	No
	Mainly fire	Yes	No	Yes	No	Yes	No	No
1	Mainly fire	No	No	Yes	No	Yes	No	No
0.75	Mainly fire	No	No	No	No	No	No	No
1	Mainly fire	No	No	Yes	No	Yes	No	No
0.5	Mainly fire	No	No	No	No	Yes	No	No
0.5	Mainly fire	Yes	No	Yes	No	Yes	No	No
2	Mainly fire	Yes	No	No	No	Yes	No	No
1.25	Mainly fire	No	No	Yes	No	No	No	No
1	Mainly fire	No	Yes	Yes	No	Yes	No	No
0.5	Mainly fire	Yes	No	Yes	No	Yes	No	No
0.2	Animal dur	Yes	No	No	Yes	Yes	No	No
	Mainly fire	No	No	Yes	No	No	No	No
999	Mainly fire	No	No	No	No	No	No	No
	Mainly fire	No	No	No	No	Yes	No	No
0.5	Mainly fire	No	No	No	No	No	No	No
2	Mainly fire	Yes	No	No	No	Yes	No	No
0.5	Mainly fire	No	No	No	No	Yes	No	No
0.5	Mainly fire	Yes	No	Yes	No	Yes	No	No
0.5	Mainly fire	Yes	No	No	No	Yes	No	No
1	Mainly fire	No	No	Yes	Yes	Yes	No	No
	Mainly fire	Yes	No	No	No	Yes	No	No
0.5	Mainly fire	No	No	Yes	No	Yes	No	No
2	Mainly fire	No	No	Yes	No	Yes	No	No
0.5	Mainly fire	No	No	No	No	Yes	No	No
1	Mainly fire	Yes	No	Yes	No	Yes	No	No
1	Mainly fire	No	No	Yes	No	No	No	No

For peer review only

2	Mainly fire	No	No	No	No	No	No	No
	Charcoal	Yes	Yes	No	Yes	Yes	No	No
	Mainly fire	No	No	No	No	No	No	No
0.5	Mainly fire	No	No	No	No	Yes	No	No
0.5	Mainly fire	No	No	Yes	No	Yes	No	No
0.25	Mainly fire	No	No	Yes	No	No	No	No
0.2	Mainly fire	No	No	Yes	No	Yes	No	No
0.1	Animal dur	No	No	Yes	No	Yes	No	No
0.25	Animal dur	Yes	No	Yes	No	Yes	No	No
2.5	Mainly fire	Yes	Yes	No	No	Yes	No	No
	Animal dur	Yes	Yes	No	Yes	Yes	No	No
0.1	Mainly fire	No	No	No	No	No	No	No
0.5	Mainly fire	Yes	No	No	No	Yes	No	No
0.2	Mainly fire	No	No	No	No	Yes	No	No
0.5	Animal dur	No	No	No	No	Yes	No	No
0.1	Animal dur	Yes	No	Yes	No	No	No	No
0.1	Animal dur	Yes	No	No	No	Yes	No	No
1.25	Mainly fire	Yes	No	Yes	No	Yes	No	No
1.5	Mainly fire	Yes	No	No	No	No	No	No
0.1	Animal dur	No	No	Yes	No	Yes	No	No
0.1	Animal dur	Yes	No	Yes	No	Yes	No	No
0.75	Mainly fire	Yes	No	Yes	No	Yes	No	No
	Mainly fire	Yes	No	No	No	Yes	No	No
0.5	Charcoal	No	No	No	No	Yes	No	No
0.5	Mainly fire	No	No	Yes	No	Yes	No	No
0.72	Mainly fire	Yes	No	Yes	No	Yes	No	No
1	Mainly fire	Yes	No	Yes	No	Yes	No	No
	Animal dur	Yes	No	No	No	Yes	No	No
0.2	Animal dur	Yes	No	No	Yes	Yes	No	Yes
	Animal dur	Yes	No	No	No	Yes	No	No
	Mainly fire	Yes	No	Yes	No	Yes	No	No
	Mainly fire	Yes	Yes	Yes	Yes	Yes	No	No
0.2	Mainly fire	Yes	No	Yes	No	Yes	No	No
1	Mainly fire	No	No	Yes	No	Yes	No	No
2	Mainly fire	No	No	Yes	No	Yes	No	No
	Mainly fire	No	No	No	No	No	No	No
1.25	Mainly fire	Yes	No	No	No	Yes	No	No
0.5	Mainly fire	No	No	Yes	No	Yes	No	No
3	Mainly fire	No	No	Yes	No	No	Yes	No
1.5	Mainly fire	No	No	No	No	Yes	No	No
0	Mainly fire	Yes	No	No	No	Yes	No	No
1.25	Mainly fire	Yes	No	Yes	No	Yes	No	No
0.75	Animal dur	No	No	No	No	No	No	No
2	Mainly fire	Yes	No	No	No	Yes	No	No
0.5	Animal dur	No	No	Yes	No	Yes	No	No
1	Mainly fire	Yes	No	No	No	Yes	No	No
0.5	Mainly fire	No	No	No	No	No	No	No
0.5	Animal dur	No	No	Yes	No	Yes	No	No
0.5	Mainly fire	No	No	Yes	No	Yes	No	No
0.5	Mainly fire	No	No	Yes	No	Yes	No	No
	Mainly fire	No	No	No	No	No	No	No
1.1	Animal dur	Yes	No	Yes	Yes	Yes	No	No

For peer review only

0.1	Animal dur	No	No	No	No	Yes	No	No
0.75	Mainly fire	Yes	No	Yes	Yes	Yes	No	No
3	Mainly fire	Yes	No	No	No	No	No	No
3	Mainly fire	Yes	No	No	No	Yes	No	No
0.375	Animal dur	No	No	Yes	No	Yes	No	No
2	Mainly fire	Yes	No	No	No	Yes	No	No
0.5	Mainly fire	No	No	No	No	Yes	No	No
2	Mainly fire	Yes	No	No	No	Yes	No	No
0.5	Mainly fire	Yes	No	Yes	No	Yes	No	No
0.5	Mainly fire	Yes	No	Yes	No	Yes	No	No
0.2	Mainly fire	No	No	No	No	Yes	No	No
0.25	Mainly fire	No	No	No	No	Yes	No	No
	Mainly fire	No	No	Yes	No	Yes	No	No
0.5	Mainly fire	No	No	No	No	No	No	No
0.5	Mainly fire	Yes	No	Yes	No	Yes	No	No
	Mainly fire	Yes	No	No	No	Yes	No	No
1	Mainly fire	Yes	No	No	No	No	No	No
	Mainly fire	No	No	No	No	No	No	No
	Mainly fire	Yes	No	No	No	Yes	No	No
0	Mainly fire	Yes	No	Yes	No	No	No	No
1.25	Mainly fire	Yes	No	No	No	Yes	No	No
	Mainly fire	No	No	No	No	Yes	No	No
1	Mainly fire	Yes	No	No	Yes	Yes	No	No
0.5	Mainly fire	Yes	No	No	No	Yes	No	No
999	Mainly fire	No	No	No	No	No	No	No
	Mainly fire	Yes	No	Yes	No	Yes	No	No
	Mainly fire	Yes	No	No	Yes	Yes	No	No
	Mainly fire	Yes	No	No	No	Yes	No	No
	Mainly fire	Yes	No	Yes	Yes	Yes	No	No
	Mainly fire	Yes	No	No	Yes	Yes	No	No
	Mainly fire	Yes	No	No	Yes	Yes	No	No
2.25	Mainly fire	Yes	No	No	No	Yes	No	No
	Mainly fire	No	No	No	No	No	Yes	No
1	Mainly fire	Yes	No	Yes	Yes	Yes	No	No
0	Mainly fire	Yes	No	No	No	No	No	No
0.5	Mainly fire	Yes	No	No	No	Yes	No	No
1	Mainly fire	Yes	No	No	No	Yes	No	No
1	Mainly fire	No	No	No	Yes	Yes	No	No
	Mainly fire	No	No	No	No	Yes	No	No
0.5	Mainly fire	Yes	No	No	No	Yes	No	No
1	Mainly fire	Yes	No	No	No	No	No	No
0.5	Mainly fire	Yes	No	No	No	No	Yes	No
	Mainly fire	No	No	No	No	Yes	No	No
0.5	Mainly fire	No	No	No	No	No	No	No
	Mainly fire	Yes	No	Yes	Yes	Yes	No	No
	Mainly fire	Yes	No	No	Yes	Yes	No	Yes
	Mainly fire	Yes	No	No	No	Yes	No	No
0.75	Mainly fire	Yes	No	No	No	Yes	No	No
	Mainly fire	Yes	No	No	Yes	Yes	No	No
	Mainly fire	Yes	No	No	Yes	Yes	No	No
	Mainly fire	Yes	No	No	Yes	Yes	No	No
	Mainly fire	No	No	No	No	Yes	No	No

For peer review only

	Mainly fire	Yes	No	Yes	Yes	Yes	No	Yes
1	Mainly fire	Yes	No	No	No	No	No	No
0.5	Mainly fire	Yes	No	Yes	Yes	Yes	No	No
0.75	Mainly fire	Yes	No	No	No	Yes	No	No
0.75	Mainly fire	Yes	No	Yes	Yes	Yes	No	No
	Mainly fire	Yes	No	No	No	No	No	No
0.5	Mainly fire	Yes	No	No	No	Yes	No	No
	Mainly fire	Yes	No	No	No	No	No	No
0.75	Mainly fire	Yes	No	No	No	Yes	No	No
0.5	Mainly fire	Yes	No	Yes	Yes	Yes	No	Yes
	Mainly fire	Yes	No	No	Yes	Yes	No	No
0	Mainly fire	Yes	No	No	No	Yes	No	No
0.75	Mainly fire	Yes	No	No	No	Yes	No	No
1	Mainly fire	Yes	No	No	No	Yes	No	No
	Mainly fire	Yes	No	No	No	No	No	No
0.5	Mainly fire	Yes	No	No	Yes	Yes	No	Yes
	Mainly fire	No	No	No	No	No	No	No
2	Mainly fire	Yes	No	No	No	No	No	No
	Mainly fire	Yes	No	No	Yes	Yes	No	No
0	Mainly fire	Yes	No	No	Yes	Yes	No	No
	Mainly fire	Yes	No	No	No	Yes	No	No
	Mainly fire	No	No	No	No	Yes	No	No
1	Mainly fire	Yes	No	No	No	Yes	No	No
	Mainly fire	Yes	No	No	No	Yes	No	No
	Mainly fire	Yes	No	No	Yes	Yes	No	Yes
	Mainly fire	Yes	No	No	No	Yes	No	No
	Mainly fire	No	No	No	No	No	No	No
	Mainly fire	No	No	No	No	Yes	No	No
1.5	Mainly fire	Yes	No	No	No	No	No	No
	Mainly fire	Yes	No	No	No	No	No	No
	Mainly fire	Yes	No	No	Yes	Yes	No	No
	Mainly fire	Yes	No	No	No	Yes	No	No
0.25	Mainly fire	Yes	No	No	No	Yes	No	No
	Mainly fire	Yes	No	Yes	Yes	Yes	No	No
1	Mainly fire	Yes	No	No	No	Yes	No	No
0.75	Mainly fire	Yes	No	No	Yes	Yes	No	No
	Mainly fire	No	No	No	No	No	No	No
	Mainly fire	Yes	No	No	No	Yes	No	No
	Mainly fire	Yes	No	No	Yes	Yes	No	No
0.25	Mainly fire	Yes	No	No	No	Yes	No	No
2	Mainly fire	Yes	No	No	No	No	No	No
	Mainly fire	No	No	No	No	Yes	No	No
0.25	Mainly fire	Yes	No	No	No	Yes	No	No
	Mainly fire	No	No	No	No	Yes	No	No
	Mainly fire	No	No	No	No	Yes	No	No
	Mainly fire	Yes	No	No	Yes	Yes	No	No
	Mainly fire	Yes	No	No	Yes	Yes	No	No
	Mainly fire	Yes	No	Yes	No	Yes	No	No
	Mainly fire	Yes	No	No	Yes	Yes	No	No
0.25	Mainly fire	Yes	No	No	No	Yes	No	No
1.25	Mainly fire	Yes	No	No	No	Yes	No	No
0.5	Mainly fire	Yes	No	No	Yes	Yes	No	No

For peer review only

0	Mainly fire	Yes	No	No	No	No	No	No
	Mainly fire	No	No	No	No	Yes	No	No
1	Mainly fire	Yes	No	No	No	Yes	No	No
0.5	Mainly fire	Yes	No	Yes	No	Yes	No	No
	Mainly fire	Yes	No	No	Yes	Yes	No	No
0.5	Mainly fire	Yes	No	No	No	Yes	No	No
2	Mainly fire	Yes	No	Yes	No	No	No	No
	Mainly fire	No	No	No	No	Yes	No	No
2	Mainly fire	Yes	No	No	No	Yes	No	No
0	Mainly fire	Yes	No	No	No	Yes	No	No
0.75	Mainly fire	Yes	No	No	Yes	Yes	No	No
0.5	Mainly fire	Yes	No	No	No	Yes	No	No
1	Mainly fire	Yes	No	No	No	Yes	No	No
	Charcoal	Yes	No	No	Yes	Yes	No	No
0.25	Mainly fire	Yes	No	No	No	Yes	No	No
1	Mainly fire	Yes	No	No	Yes	Yes	No	No
0.25	Mainly fire	Yes	No	No	No	Yes	No	No
	Mainly fire	No	No	No	No	Yes	No	No
	Mainly fire	Yes	No	No	No	No	No	No
1	Mainly fire	No	No	No	No	No	No	No
1.5	Mainly fire	Yes	Yes	No	No	Yes	No	No
0.5	Mainly fire	Yes	No	No	No	Yes	No	No
	Mainly fire	Yes	No	No	No	Yes	No	No
0.5	Mainly fire	No	No	No	No	Yes	No	No
	Mainly fire	Yes	No	No	No	No	No	No
0.5	Mainly fire	Yes	No	No	Yes	Yes	No	No
0.75	Mainly fire	No	No	No	No	Yes	No	No
	Mainly fire	Yes	No	No	Yes	Yes	No	No
	Mainly fire	Yes	No	No	No	Yes	No	No
0.75	Mainly fire	Yes	No	Yes	No	Yes	No	No
0.25	Mainly fire	No	No	Yes	No	Yes	No	No
0.5	Mainly fire	No	No	No	No	No	No	No
1	Mainly fire	Yes	No	Yes	No	Yes	No	No
0.75	Mainly fire	Yes	No	No	No	Yes	No	No
0	Mainly fire	No	No	No	No	Yes	No	No
2	Mainly fire	Yes	No	No	No	Yes	No	No
0	Mainly fire	Yes	No	No	No	No	Yes	No
	Mainly fire	No	No	No	No	Yes	No	No
1	Mainly fire	Yes	No	Yes	No	Yes	No	No
1	Mainly fire	No	No	No	No	No	No	No
	Mainly fire	Yes	No	No	No	Yes	No	No
	Mainly fire	Yes	No	No	No	Yes	No	No
0.25	Charcoal	Yes	Yes	No	Yes	Yes	No	No
	Mainly fire	Yes	No	No	No	Yes	No	No
	Mainly fire	Yes	No	No	No	Yes	No	No
	Mainly fire	Yes	No	No	No	No	No	No
0.5	Mainly fire	Yes	No	Yes	No	Yes	No	No
1	Mainly fire	Yes	No	No	No	Yes	No	No
0.75	Mainly fire	Yes	No	No	No	Yes	No	No
	Mainly fire	Yes	No	No	No	Yes	No	No
2	Mainly fire	Yes	No	No	No	Yes	No	No
0.5	Mainly fire	No	No	No	No	Yes	No	No

For peer review only

	Mainly fire	Yes	No	No	No	Yes	No	No
	Mainly fire	Yes	No	No	No	No	No	No
1	Mainly fire	Yes	No	No	Yes	Yes	No	No
0.25	Mainly fire	Yes	No	No	No	Yes	No	No
	Mainly fire	Yes	No	No	No	Yes	No	No
0.5	Mainly fire	No	No	No	No	Yes	No	No
	Mainly fire	Yes	No	No	No	Yes	No	No
2.5	Mainly fire	Yes	No	No	No	Yes	No	No
0.25	Mainly fire	Yes	No	No	No	No	No	No
0.5	Mainly fire	Yes	No	No	No	Yes	No	No
0.5	Mainly fire	Yes	No	No	No	Yes	No	No
	Mainly fire	No	No	No	No	Yes	No	Yes
0.5	Mainly fire	No	No	No	No	Yes	No	No
0.75	Does not u	No	No	No	No	Yes	No	No
2	Mainly fire	Yes	No	No	Yes	Yes	No	No
	Charcoal	Yes	No	Yes	Yes	Yes	No	No
0.25	Mainly fire	Yes	No	Yes	No	No	No	No
0	Mainly fire	Yes	No	No	Yes	Yes	No	No
	Mainly fire	Yes	No	No	Yes	No	No	No
1.25	Mainly fire	Yes	No	No	Yes	Yes	No	No
1.5	Mainly fire	Yes	No	Yes	No	Yes	No	No
1.5	Mainly fire	Yes	No	No	No	Yes	No	No
	Mainly fire	No	No	No	No	Yes	No	No
0.5	Mainly fire	Yes	No	No	No	No	No	No
0.5	Mainly fire	Yes	No	No	No	Yes	No	No
1.25	Mainly fire	Yes	No	Yes	No	Yes	No	No
	Mainly fire	Yes	No	No	Yes	Yes	No	No
	Mainly fire	Yes	Yes	Yes	No	Yes	No	No
	Electricity	Yes	No	No	Yes	Yes	No	No
0.75	Mainly fire	Yes	Yes	No	No	Yes	No	No
	Mainly fire	Yes	No	No	Yes	Yes	No	No
	Mainly fire	Yes	No	No	Yes	Yes	No	No
1	Mainly fire	Yes	No	No	No	Yes	No	No
0	Mainly fire	Yes	No	No	No	Yes	No	No
	Mainly fire	Yes	No	No	No	Yes	No	No
	Mainly fire	Yes	No	No	No	No	No	No
	Mainly fire	Yes	No	Yes	Yes	Yes	No	No
1.25	Mainly fire	Yes	No	No	No	No	No	No
	Mainly fire	Yes	No	No	No	Yes	No	No
	Mainly fire	Yes	No	No	Yes	Yes	No	Yes
1	Mainly fire	Yes	Yes	Yes	Yes	Yes	No	No
0.75	Mainly fire	Yes	No	No	No	Yes	No	No
2	Mainly fire	Yes	No	Yes	No	No	No	No
	Charcoal	Yes	No	No	Yes	Yes	No	No
	Mainly fire	No	No	No	No	Yes	No	No
2	Mainly fire	Yes	No	Yes	Yes	Yes	No	No
	Mainly fire	Yes	No	No	Yes	Yes	No	No
1	Mainly fire	Yes	No	No	No	Yes	No	No
	Mainly fire	Yes	No	No	No	Yes	No	No
0.75	Mainly fire	Yes	No	No	No	Yes	No	No
0.5	Mainly fire	Yes	No	No	Yes	Yes	No	Yes
	Mainly fire	Yes	No	No	No	No	No	No

For peer review only

0.5	Mainly fire	No	No	No	No	Yes	Yes	No
	Mainly fire	Yes	No	No	Yes	Yes	No	No
0.5	Mainly fire	Yes	Yes	No	No	Yes	No	No
1	Mainly fire	Yes	No	No	Yes	Yes	No	No
	Mainly fire	Yes	No	No	No	Yes	No	No
0.5	Mainly fire	Yes	No	No	No	Yes	No	No
	Mainly fire	Yes	No	No	No	Yes	No	No
1	Mainly fire	Yes	No	No	No	Yes	No	No
	Mainly fire	Yes	No	No	No	No	No	No
1.5	Mainly fire	Yes	No	No	No	Yes	No	No
2.25	Mainly fire	Yes	No	No	No	Yes	No	No
	Mainly fire	Yes	No	No	Yes	Yes	No	No
0.5	Mainly fire	Yes	No	Yes	No	Yes	No	No
	Mainly fire	Yes	No	No	No	Yes	No	No
1	Mainly fire	No	No	No	No	Yes	No	No
	Mainly fire	Yes	No	No	No	Yes	No	No
	Mainly fire	Yes	No	No	No	Yes	No	No
2	Mainly fire	No	No	No	No	Yes	No	No
0.5	Mainly fire	Yes	No	No	Yes	Yes	No	No
	Mainly fire	Yes	No	No	Yes	Yes	No	No
0.25	Charcoal	Yes	No	Yes	Yes	Yes	No	Yes
	Mainly fire	Yes	No	Yes	Yes	Yes	No	No
0.25	Charcoal	Yes	No	No	No	Yes	No	No
1	Mainly fire	Yes	No	No	No	No	No	No
	Mainly fire	Yes	No	No	No	Yes	No	No
0.5	Mainly fire	Yes	No	No	No	No	No	No
	Mainly fire	Yes	No	No	Yes	Yes	No	No
0.25	Mainly fire	Yes	No	No	Yes	Yes	No	No
1	Mainly fire	Yes	No	No	No	Yes	No	No
0.5	Mainly fire	Yes	No	No	Yes	Yes	No	No
1.5	Mainly fire	Yes	No	No	No	No	No	No
1.75	Mainly fire	Yes	No	No	No	Yes	No	No
	Mainly fire	Yes	No	No	No	Yes	No	No
0.5	Mainly fire	Yes	No	No	Yes	Yes	No	No
5	Mainly fire	Yes	No	No	Yes	Yes	No	No
	Mainly fire	Yes	No	No	No	Yes	No	No
	Mainly fire	Yes	No	No	No	No	No	No
	Mainly fire	Yes	No	No	Yes	Yes	No	No
0.5	Mainly fire	Yes	No	Yes	No	Yes	No	No
0.5	Mainly fire	Yes	No	No	No	No	No	No
	Mainly fire	Yes	No	Yes	No	Yes	No	No
	Mainly fire	Yes	No	Yes	No	No	No	No
0.5	Mainly fire	Yes	No	No	No	Yes	No	No
	Mainly fire	Yes	No	No	No	No	No	No
1.5	Mainly fire	Yes	No	No	Yes	Yes	No	No
	Mainly fire	Yes	No	No	No	Yes	No	No
2	Mainly fire	Yes	No	No	No	Yes	No	No
	Mainly fire	Yes	No	No	No	No	No	No
0.5	Mainly fire	No	No	No	No	Yes	No	No
0.5	Mainly fire	Yes	No	No	Yes	Yes	No	No
0.5	Mainly fire	Yes	No	No	No	No	No	No
1.75	Mainly fire	Yes	No	Yes	No	Yes	No	No

For peer review only

2	Mainly fire	Yes	No	Yes	No	Yes	No	No
	Mainly fire	Yes	No	No	No	Yes	No	No
	Mainly fire	Yes	No	No	No	Yes	No	No
	Mainly fire	Yes	No	No	No	Yes	No	No
	Mainly fire	Yes	No	No	No	Yes	No	No
	Mainly fire	Yes	No	No	Yes	Yes	No	Yes
	Mainly fire	No	No	No	No	No	No	No
	Mainly fire	Yes	No	No	No	Yes	No	No
0.5	Charcoal	Yes	No	No	Yes	Yes	No	No
1.5	Mainly fire	Yes	No	No	No	No	No	No
	Mainly fire	Yes	No	No	Yes	Yes	No	No
0.5	Mainly fire	Yes	No	No	Yes	Yes	Yes	No
	Mainly fire	Yes	No	No	No	Yes	No	No
	Mainly fire	Yes	No	No	Yes	Yes	No	No
1.5	Mainly fire	Yes	No	No	Yes	Yes	No	No
0.5	Mainly fire	Yes	No	No	No	No	No	No
	Mainly fire	Yes	No	No	Yes	Yes	No	No
1	Mainly fire	No	No	No	No	Yes	No	No
0.5	Mainly fire	Yes	No	No	Yes	Yes	No	No
	Mainly fire	Yes	No	No	No	Yes	No	No
0	Mainly fire	Yes	No	No	Yes	Yes	No	Yes
	Mainly fire	Yes	No	No	No	Yes	No	No
	Mainly fire	No	No	No	No	Yes	No	No
	Mainly fire	Yes	No	No	No	Yes	No	No
	Mainly fire	No	No	No	No	No	No	No
1	Mainly fire	Yes	No	No	No	Yes	No	No
	Mainly fire	Yes	No	No	No	Yes	No	No
0.25	Mainly fire	Yes	No	No	No	Yes	No	No
1	Mainly fire	No	No	No	No	Yes	No	No
999	Mainly fire	Yes	No	No	Yes	Yes	No	No
2	Mainly fire	Yes	No	No	No	No	No	No
1	Mainly fire	Yes	No	No	Yes	Yes	No	No
	Mainly fire	Yes	No	No	No	Yes	No	No
	Charcoal	Yes	No	No	Yes	Yes	No	No
3	Mainly fire	Yes	No	No	No	Yes	No	No
1.25	Mainly fire	Yes	No	No	No	Yes	No	No
	Mainly fire	Yes	No	No	No	Yes	No	No
	Mainly fire	Yes	No	No	Yes	Yes	No	No
	Mainly fire	Yes	No	No	Yes	Yes	No	No
	Mainly fire	Yes	No	No	Yes	Yes	No	Yes
	Mainly fire	Yes	No	No	No	No	Yes	No
	Mainly fire	Yes	No	No	No	Yes	No	No
	Mainly fire	Yes	No	No	Yes	Yes	No	No
1	Mainly fire	Yes	No	No	No	No	No	No
	Mainly fire	No	Yes	Yes	No	Yes	No	No
	Mainly fire	No	No	No	No	No	No	No
	Mainly fire	No	No	No	No	Yes	No	No
0.5	Mainly fire	Yes	No	No	No	Yes	No	No
	Mainly fire	Yes	No	Yes	No	Yes	No	No
0.75	Mainly fire	No	No	No	Yes	Yes	No	No
	Mainly fire	Yes	No	No	No	No	No	No
	Mainly fire	No	Yes	Yes	No	Yes	No	No

For peer review only

2	Mainly fire	Yes	No	No	No	Yes	No	No
0.5	Mainly fire	Yes	No	Yes	Yes	Yes	No	No
1.25	Mainly fire	Yes	No	No	No	Yes	No	No
	Mainly fire	Yes	No	No	No	Yes	No	No
	Mainly fire	Yes	No	No	No	Yes	No	No
1.25	Mainly fire	Yes	No	No	Yes	Yes	No	No
1	Mainly fire	Yes	No	No	No	Yes	No	No
	Mainly fire	Yes	No	No	No	Yes	No	No
	Mainly fire	Yes	No	No	Yes	Yes	No	Yes
	Mainly fire	Yes	No	No	No	Yes	No	No
0.5	Mainly fire	Yes	No	No	No	Yes	No	No
	Mainly fire	Yes	No	No	No	No	No	No
0.25	Mainly fire	Yes	No	No	No	Yes	No	No
0.75	Mainly fire	Yes	No	No	No	No	No	No
	Mainly fire	Yes	No	No	Yes	Yes	No	No
	Mainly fire	Yes	No	No	No	Yes	No	No
2	Mainly fire	Yes	No	Yes	No	Yes	No	No
1	Mainly fire	Yes	No	No	No	Yes	No	No
1	Mainly fire	No	No	Yes	No	Yes	No	No
2	Mainly fire	Yes	No	Yes	No	Yes	No	No
1.5	Mainly fire	Yes	No	No	No	Yes	No	No
0.5	Mainly fire	Yes	No	No	No	Yes	No	No
2	Mainly fire	Yes	No	No	No	Yes	No	No
1	Mainly fire	No	No	No	No	No	No	No
0.5	Mainly fire	Yes	No	No	No	Yes	No	No
1	Mainly fire	No	No	No	No	No	No	No
2.5	Mainly fire	Yes	No	No	No	Yes	No	No
1.5	Mainly fire	No	No	No	No	No	No	No
1.75	Mainly fire	Yes	No	Yes	No	Yes	No	No
2	Mainly fire	No	No	No	No	No	No	No
2	Mainly fire	Yes	No	Yes	No	Yes	No	No
	Mainly fire	No	No	No	No	No	No	No
	Mainly fire	Yes	Yes	Yes	Yes	Yes	No	No
2	Mainly fire	Yes	No	No	No	Yes	No	No
	Mainly fire	Yes	No	No	No	Yes	No	No
	Crop residu	Yes	No	No	No	No	No	No
2	Mainly fire	Yes	No	No	Yes	Yes	No	No
0.5	Mainly fire	Yes	No	No	No	Yes	No	No
	Mainly fire	Yes	No	Yes	Yes	Yes	No	Yes
0.5	Mainly fire	Yes	Yes	Yes	No	Yes	No	No
	Mainly fire	Yes	No	No	No	Yes	No	No
2	Mainly fire	Yes	No	No	No	Yes	No	No
1.5	Mainly fire	Yes	No	Yes	No	No	No	No
1	Mainly fire	Yes	No	Yes	No	Yes	No	No
1	Mainly fire	Yes	No	No	No	Yes	No	No
0.5	Mainly fire	No	No	No	No	No	No	No
0.25	Crop residu	No	No	No	No	No	No	No
999	Mainly fire	Yes	No	No	No	Yes	No	No
2	Mainly fire	Yes	No	No	No	Yes	No	No
0.5	Mainly fire	Yes	No	No	No	Yes	No	No
0.5	Mainly fire	Yes	No	Yes	No	No	No	No
	Crop residu	Yes	No	No	No	Yes	No	No

For peer review only

1	Mainly fire	Yes	No	No	No	Yes	No	No
2	Mainly fire	Yes	No	No	No	No	No	No
0.5	Mainly fire	Yes	No	No	No	Yes	No	No
2	Mainly fire	Yes	No	No	No	Yes	No	No
	Mainly fire	No	No	No	No	Yes	No	No
1	Mainly fire	Yes	No	No	No	Yes	No	No
1.5	Mainly fire	Yes	No	No	No	No	No	No
	Mainly fire	No	No	No	No	No	No	No
999	Mainly fire	Yes	No	No	No	Yes	No	No
	Mainly fire	No	No	No	No	Yes	No	No
1	Mainly fire	No	No	No	No	Yes	No	No
999	Mainly fire	Yes	No	No	No	Yes	No	No
1	Mainly fire	No	No	No	No	Yes	No	No
999	Mainly fire	Yes	No	No	No	No	No	No
	Mainly fire	Yes	No	No	No	No	No	No
0.5	Mainly fire	Yes	No	No	No	Yes	No	No
0.5	Mainly fire	Yes	No	No	No	No	No	No
1.5	Mainly fire	Yes	No	No	No	Yes	No	No
	Mainly fire	Yes	No	No	No	Yes	No	No
0.5	Mainly fire	Yes	No	No	No	Yes	No	No
1	Mainly fire	No	No	No	No	Yes	No	No
0.5	Mainly fire	Yes	No	No	No	Yes	No	No
1	Mainly fire	Yes	Yes	Yes	No	Yes	No	No
	Mainly fire	Yes	No	Yes	No	Yes	No	No
1	Mainly fire	Yes	No	Yes	Yes	No	No	No
2.5	Mainly fire	Yes	No	Yes	No	Yes	No	No
	Crop residu	No	No	No	No	No	No	No
0.5	Mainly fire	Yes	No	Yes	No	Yes	No	No
0.5	Mainly fire	Yes	No	No	No	Yes	No	No
	Mainly fire	Yes	No	No	No	No	No	No
	Mainly fire	Yes	No	No	No	No	No	No
0.5	Mainly fire	Yes	No	No	No	Yes	No	No
1	Mainly fire	Yes	No	Yes	No	Yes	No	No
1.5	Mainly fire	No	No	No	No	Yes	No	No
2	Mainly fire	No	No	No	No	Yes	No	No
	Mainly fire	Yes	Yes	No	No	Yes	No	No
	Mainly fire	Yes	No	No	No	Yes	No	No
0.5	Mainly fire	No	No	No	No	No	No	No
1.5	Mainly fire	Yes	No	Yes	No	Yes	No	No
1	Mainly fire	Yes	No	No	No	Yes	No	No
	Mainly fire	Yes	No	Yes	No	Yes	No	No
1	Mainly fire	Yes	No	No	No	Yes	No	No
2	Mainly fire	Yes	No	No	No	Yes	No	No
0.75	Mainly fire	Yes	No	No	No	No	No	No
1	Mainly fire	Yes	No	No	No	Yes	No	No
1.5	Mainly fire	Yes	No	Yes	No	No	No	No
1.5	Mainly fire	Yes	No	No	No	Yes	No	No
0.75	Mainly fire	Yes	No	No	No	No	No	No
2	Mainly fire	No	No	Yes	No	Yes	No	No
1	Mainly fire	Yes	No	No	No	Yes	No	No
0.5	Mainly fire	Yes	No	Yes	No	Yes	No	No
1.5	Mainly fire	Yes	No	Yes	No	Yes	No	No

For peer review only

1.5	Mainly fire	No	No	No	No	Yes	No	No
0.5	Mainly fire	No	No	No	No	No	No	No
1.25	Mainly fire	Yes	No	No	No	Yes	No	No
1	Crop residu	Yes	No	Yes	No	No	No	No
2.05	Mainly fire	Yes	No	No	No	Yes	No	No
	Crop residu	Yes	No	Yes	No	Yes	No	No
0.5	Mainly fire	Yes	No	No	No	Yes	No	No
1	Mainly fire	No	No	Yes	No	Yes	No	No
1	Mainly fire	Yes	No	No	No	Yes	No	No
1.5	Mainly fire	No	No	No	No	Yes	No	No
	Mainly fire	Yes	No	No	No	Yes	No	No
	Mainly fire	No	No	No	No	No	No	No
	Mainly fire	Yes	No	No	No	Yes	No	No
1	Mainly fire	No	No	No	No	No	No	No
2	Mainly fire	Yes	No	No	No	Yes	No	No
	Mainly fire	Yes	No	No	Yes	Yes	No	No
2	Mainly fire	Yes	No	No	No	No	No	No
	Mainly fire	No	No	No	No	No	No	Yes
2	Mainly fire	Yes	No	No	No	Yes	No	No
0.5	Mainly fire	Yes	No	Yes	No	Yes	No	No
999	Mainly fire	No	No	No	No	Yes	No	No
0.5	Mainly fire	Yes	No	No	No	Yes	No	No
2	Mainly fire	No	No	No	No	No	No	No
0.5	Mainly fire	Yes	No	No	No	No	No	No
0.5	Mainly fire	Yes	No	No	No	Yes	No	No
3	Mainly fire	Yes	No	No	No	Yes	No	No
1	Mainly fire	Yes	No	No	Yes	No	No	No
3	Mainly fire	Yes	No	No	No	Yes	No	No
1.5	Mainly fire	Yes	No	No	No	Yes	No	No
	Mainly fire	No	No	No	No	Yes	No	No
	Mainly fire	Yes	No	No	No	Yes	No	No
0.5	Mainly fire	Yes	No	No	Yes	Yes	No	No
0.5	Mainly fire	Yes	Yes	Yes	No	Yes	No	No
1	Mainly fire	Yes	No	No	No	Yes	No	No
	Mainly fire	Yes	No	No	No	Yes	Yes	Yes
	Mainly fire	Yes	Yes	Yes	No	Yes	No	No
1	Mainly fire	Yes	No	No	No	No	No	No
2	Mainly fire	Yes	No	Yes	No	No	No	No
4	Mainly fire	Yes	No	No	No	Yes	No	No
1.5	Mainly fire	Yes	No	Yes	No	Yes	No	No
	Mainly fire	Yes	No	Yes	No	Yes	No	No
	Mainly fire	Yes	No	Yes	No	Yes	No	No
	Mainly fire	Yes	No	No	No	Yes	No	No
0.5	Mainly fire	Yes	No	No	No	Yes	No	No
5	Mainly fire	Yes	No	No	No	Yes	No	No
2	Mainly fire	Yes	No	No	No	Yes	No	No
1	Mainly fire	Yes	No	No	No	Yes	No	No
	Mainly fire	Yes	No	No	No	Yes	No	No
0.5	Mainly fire	No	No	No	No	Yes	No	No
	Mainly fire	Yes	No	Yes	No	Yes	No	No
	Mainly fire	Yes	No	No	Yes	Yes	No	No
	Crop residu	Yes	No	No	No	Yes	No	No

For peer review only

	Mainly fire	Yes	No	No	No	Yes	No	No
3	Mainly fire	Yes	No	No	No	Yes	No	No
999	Mainly fire	Yes	No	No	No	Yes	No	No
1	Mainly fire	Yes	No	Yes	No	Yes	No	No
1.75	Crop residu	Yes	No	Yes	No	Yes	No	No
	Mainly fire	Yes	No	No	No	Yes	No	No
1.5	Mainly fire	Yes	No	No	No	No	Yes	No
99.99	Mainly fire	Yes	No	No	No	Yes	No	No
1.5	Crop residu	Yes	No	No	No	No	No	No
1	Mainly fire	Yes	No	No	No	Yes	No	No
1	Mainly fire	Yes	No	No	No	Yes	No	No
0.25	Mainly fire	Yes	No	No	No	Yes	No	No
	Mainly fire	Yes	No	Yes	No	Yes	No	No
1.25	Crop residu	No	No	No	No	Yes	No	No
2	Mainly fire	No	No	No	No	Yes	No	No
0.75	Mainly fire	Yes	No	No	No	Yes	No	No
	Mainly fire	No	No	No	No	Yes	No	No
1	Mainly fire	No	No	No	No	No	No	No
999	Mainly fire	Yes	No	No	No	Yes	No	No
	Mainly fire	Yes	No	No	No	Yes	No	No
3	Mainly fire	Yes	No	No	No	Yes	No	No
0.5	Mainly fire	Yes	No	No	No	Yes	No	No
1	Mainly fire	Yes	No	No	No	Yes	No	No
0.5	Mainly fire	Yes	No	Yes	No	Yes	No	No
999	Mainly fire	No	No	No	No	No	No	No
2	Mainly fire	Yes	No	No	No	No	No	No
	Mainly fire	Yes	No	No	Yes	Yes	No	No
1	Mainly fire	No	No	No	No	Yes	No	No
	Mainly fire	Yes	Yes	Yes	No	No	No	No
99	Crop residu	Yes	No	Yes	No	Yes	No	No
1	Mainly fire	Yes	No	Yes	No	Yes	No	No
1	Crop residu	Yes	No	Yes	No	Yes	No	No
1.75	Mainly fire	Yes	No	No	No	Yes	No	No
1.25	Crop residu	No	No	No	No	No	No	No
	Mainly fire	No	No	No	No	Yes	No	No
0.25	Mainly fire	Yes	No	No	No	Yes	No	No
2	Mainly fire	Yes	No	No	No	No	No	No
	Mainly fire	Yes	No	No	No	Yes	No	No
	Mainly fire	Yes	No	No	No	Yes	No	No
1	Mainly fire	No	No	Yes	No	Yes	No	No
2	Does not u	Yes	No	Yes	No	Yes	No	No
	Animal dur	Yes	No	Yes	No	Yes	No	No
1	Mainly fire	Yes	No	No	No	Yes	No	No
1	Mainly fire	No	No	No	No	Yes	No	No
	Mainly fire	No	No	No	No	Yes	No	No
	Mainly fire	No	No	No	No	Yes	No	No
1	Mainly fire	Yes	No	Yes	No	Yes	No	No
1	Mainly fire	Yes	No	No	No	Yes	No	No
0	Mainly fire	Yes	No	No	No	Yes	No	No
1.5	Mainly fire	Yes	No	No	No	Yes	No	No
6	Mainly fire	Yes	No	No	No	Yes	No	No
0.5	Mainly fire	Yes	No	Yes	No	Yes	No	No

For peer review only

	Animal dur	Yes	No	No	No	No	No	No
3	Mainly fire	Yes	No	No	No	Yes	No	No
	Mainly fire	Yes	No	Yes	No	Yes	No	No
	Mainly fire	Yes	No	No	No	Yes	No	No
0.5	Mainly fire	No	No	No	No	Yes	No	No
1	Mainly fire	Yes	No	No	No	No	No	No
0.25	Mainly fire	Yes	No	No	No	Yes	No	No
	Mainly fire	Yes	No	No	Yes	Yes	No	No
	Mainly fire	No	No	Yes	No	Yes	No	No
1	Mainly fire	Yes	No	No	No	Yes	No	No
2	Mainly fire	Yes	No	No	No	No	No	No
0.5	Mainly fire	Yes	No	No	No	Yes	No	No
1.5	Mainly fire	Yes	No	No	No	Yes	No	No
1	Mainly fire	Yes	No	No	No	No	No	No
3	Mainly fire	Yes	No	Yes	No	Yes	No	No
2	Mainly fire	Yes	No	Yes	No	Yes	No	No
	Mainly fire	Yes	No	No	No	No	No	No
3	Mainly fire	Yes	No	No	No	Yes	No	No
	Mainly fire	Yes	No	Yes	No	Yes	No	No
0.75	Animal dur	No	No	No	No	Yes	No	No
	Mainly fire	Yes	No	No	No	Yes	No	No
1	Mainly fire	No	No	Yes	No	Yes	Yes	No
0.75	Mainly fire	Yes	No	No	No	Yes	No	No
	Mainly fire	Yes	No	No	No	Yes	No	No
1	Mainly fire	Yes	No	Yes	No	Yes	No	No
	Mainly fire	Yes	No	No	No	Yes	No	No
	Crop residu	Yes	No	No	No	No	Yes	No
1.25	Mainly fire	No	No	No	No	No	No	Yes
0.5	Mainly fire	Yes	No	No	No	No	No	No
	Charcoal	Yes	Yes	No	Yes	Yes	No	No
	Mainly fire	Yes	No	No	No	Yes	No	No
1	Mainly fire	Yes	No	No	No	No	No	No
0.25	Charcoal	Yes	No	Yes	No	Yes	No	No
0.5	Mainly fire	Yes	No	No	No	No	No	No
0.5	Mainly fire	Yes	No	No	No	No	No	No
0	Mainly fire	Yes	No	No	No	Yes	No	No
1	Mainly fire	Yes	No	Yes	No	Yes	No	No
1	Mainly fire	No	No	No	No	No	No	No
1	Mainly fire	Yes	No	No	No	No	No	No
4	Mainly fire	Yes	No	No	No	Yes	No	No
0.1	Animal dur	No	No	Yes	No	Yes	No	No
4.5	Mainly fire	Yes	No	No	No	Yes	No	No
	Mainly fire	Yes	No	No	No	Yes	No	No
	Mainly fire	Yes	No	Yes	No	Yes	No	No
0.1	Animal dur	Yes	No	Yes	Yes	Yes	No	No
1	Animal dur	No	No	No	No	Yes	No	No
1	Mainly fire	Yes	No	No	No	Yes	No	No
0.5	Mainly fire	No	No	Yes	No	Yes	No	No
3	Mainly fire	Yes	No	No	No	Yes	No	No
1.25	Mainly fire	Yes	No	Yes	No	Yes	No	No
	Mainly fire	No	No	No	No	No	No	No
	Mainly fire	Yes	No	No	No	Yes	No	No

For peer review only

0.75	Mainly fire	Yes	Yes	No	No	Yes	No	No
19	Mainly fire	Yes	No	Yes	No	Yes	No	No
3	Charcoal	Yes	No	Yes	No	Yes	No	No
2	Animal dur	No	No	No	No	Yes	No	No
1	Mainly fire	No	No	No	No	Yes	No	No
	Mainly fire	Yes	No	No	No	Yes	No	No
1.2	Mainly fire	Yes	No	Yes	No	Yes	No	No
0.5	Mainly fire	Yes	No	No	No	Yes	No	No
	Mainly fire	Yes	No	No	No	No	No	No
1.25	Mainly fire	Yes	No	No	No	No	No	No
1	Mainly fire	Yes	No	Yes	No	Yes	No	No
1.75	Mainly fire	No	No	No	No	No	No	No
3	Mainly fire	No	No	Yes	No	No	No	No
1	Animal dur	Yes	No	No	No	Yes	No	No
0.75	Mainly fire	Yes	No	No	No	Yes	No	No
1	Does not u	Yes	No	No	No	No	No	No
999	Mainly fire	Yes	No	No	No	Yes	No	No
0.5	Mainly fire	Yes	No	No	No	Yes	No	No
1	Mainly fire	Yes	No	No	No	No	No	No
	Mainly fire	Yes	Yes	No	Yes	Yes	Yes	No
	Mainly fire	Yes	No	No	No	No	No	No
	Mainly fire	Yes	No	No	No	Yes	No	No
0.5	Mainly fire	Yes	No	No	No	Yes	No	No
1.5	Mainly fire	Yes	No	No	No	Yes	No	No
	Mainly fire	Yes	No	No	No	No	No	No
2	Animal dur	Yes	No	No	No	Yes	No	No
0.5	Mainly fire	Yes	No	Yes	No	Yes	No	No
0	Animal dur	Yes	No	Yes	Yes	Yes	No	No
0.5	Mainly fire	Yes	No	No	No	No	No	Yes
2	Mainly fire	Yes	No	No	No	Yes	No	No
2	Charcoal	Yes	No	No	No	Yes	No	No
3	Mainly fire	No	No	No	No	No	No	No
	Mainly fire	No	No	No	No	Yes	No	No
0.375	Mainly fire	Yes	Yes	No	Yes	Yes	No	No
	Mainly fire	Yes	Yes	Yes	Yes	No	Yes	Yes
0.25	Crop residu	Yes	No	No	No	No	No	No
	Mainly fire	Yes	No	Yes	No	Yes	No	No
0.75	Mainly fire	Yes	No	Yes	No	Yes	No	No
1	Mainly fire	Yes	No	No	No	Yes	No	No
0.5	Mainly fire	Yes	No	No	No	Yes	No	No
2	Animal dur	Yes	No	Yes	No	Yes	Yes	Yes
3	Mainly fire	Yes	No	Yes	No	Yes	No	No
0.5	Mainly fire	No	No	Yes	Yes	Yes	No	No
0.5	Animal dur	Yes	No	Yes	No	Yes	No	No
0.75	Mainly fire	No	No	No	No	Yes	No	No
0.75	Mainly fire	Yes	No	No	No	Yes	No	No
0.1	Animal dur	Yes	No	Yes	No	Yes	No	No
1.5	Mainly fire	Yes	No	No	No	Yes	No	No
0.5	Mainly fire	Yes	No	No	No	Yes	No	No
	Charcoal	Yes	No	Yes	Yes	Yes	No	No
2.5	Mainly fire	Yes	Yes	Yes	Yes	Yes	No	No
1	Mainly fire	Yes	No	Yes	No	Yes	No	No

For peer review only

0.5	Mainly fire	No	Yes	No	No	Yes	No	No
	Mainly fire	Yes	No	Yes	No	Yes	No	No
0.75	Animal dur	No	No	No	No	Yes	No	No
1.5	Mainly fire	No	No	No	No	No	No	No
0.75	Animal dur	Yes	No	Yes	No	Yes	No	No
	Mainly fire	Yes	No	No	No	Yes	No	No
0.5	Mainly fire	No	No	No	No	No	No	No
0.5	Mainly fire	Yes	No	No	No	Yes	No	No
0.25	Mainly fire	No	No	No	No	Yes	No	No
0.2	Animal dur	Yes	No	Yes	No	Yes	No	No
	Mainly fire	Yes	No	No	No	Yes	No	No
4	Mainly fire	Yes	No	No	No	Yes	No	No
1	Mainly fire	Yes	No	No	No	No	No	No
1.75	Mainly fire	Yes	No	No	No	Yes	No	No
0.75	Crop residu	Yes	No	No	No	No	No	No
1	Mainly fire	No	No	Yes	No	Yes	No	No
1	Mainly fire	Yes	No	Yes	No	Yes	No	No
1	Mainly fire	Yes	No	No	No	No	No	No
1.5	Mainly fire	Yes	No	No	No	Yes	No	No
1	Mainly fire	Yes	No	No	No	Yes	No	No
0.5	Mainly fire	Yes	No	No	Yes	Yes	No	No
1	Animal dur	Yes	No	Yes	No	Yes	No	No
	Mainly fire	Yes	No	No	No	No	No	No
9	Mainly fire	Yes	No	No	No	Yes	No	No
0.5	Mainly fire	Yes	No	No	No	No	No	No
0.5	Mainly fire	Yes	No	No	No	No	No	No
0.5	Mainly fire	No	No	No	No	No	No	No
2	Mainly fire	Yes	No	No	No	Yes	No	No
	Mainly fire	Yes	No	Yes	No	Yes	No	No
1	Mainly fire	No	No	No	No	Yes	No	No
0.5	Mainly fire	No	No	No	No	Yes	No	No
	Animal dur	Yes	No	No	No	Yes	No	No
0.5	Mainly fire	Yes	No	Yes	No	Yes	No	No
	Mainly fire	Yes	No	No	No	No	No	No
1.25	Mainly fire	Yes	No	No	No	Yes	No	No
	Mainly fire	No	No	No	No	Yes	No	No
	Mainly fire	Yes	No	No	No	No	No	No
0.5	Mainly fire	No	No	Yes	No	Yes	No	No
	Mainly fire	Yes	No	No	No	Yes	No	No
0.2	Animal dur	Yes	No	No	Yes	Yes	No	Yes
0.75	Mainly fire	Yes	No	Yes	No	Yes	No	No
	Animal dur	Yes	No	Yes	No	Yes	No	No
	Animal dur	No	No	No	No	Yes	No	No
1.75	Mainly fire	Yes	No	No	No	No	No	No
	Mainly fire	Yes	No	No	No	No	No	No
	Mainly fire	No	No	No	No	No	No	No
	Mainly fire	No	No	No	No	Yes	No	No
1	Mainly fire	Yes	No	No	No	No	Yes	No
	Mainly fire	Yes	No	Yes	No	Yes	No	No
2	Mainly fire	Yes	No	No	No	No	No	No
	Mainly fire	Yes	No	No	No	No	No	No
0.1	Animal dur	Yes	No	Yes	No	Yes	No	No

For peer review only

2	Mainly fire	No	No	Yes	No	No	No	No
0.5	Mainly fire	Yes	No	No	No	No	No	No
	Mainly fire	Yes	No	No	No	No	No	No
0.5	Mainly fire	Yes	No	No	No	No	No	No
1	Mainly fire	No	No	Yes	No	Yes	Yes	No
0.5	Mainly fire	Yes	No	No	No	No	No	No
3.5	Mainly fire	Yes	No	No	No	Yes	No	No
	Animal dur	Yes	Yes	Yes	Yes	Yes	No	No
	Mainly fire	Yes	No	No	No	Yes	No	No
	Mainly fire	Yes	No	No	No	No	No	No
1	Mainly fire	Yes	No	No	No	Yes	No	No
1	Mainly fire	No	No	No	No	Yes	No	No
	Mainly fire	Yes	No	Yes	No	Yes	No	No
1	Mainly fire	Yes	No	No	No	Yes	No	No
0.5	Mainly fire	No	No	No	No	Yes	No	No
1	Mainly fire	Yes	No	No	No	Yes	No	No
0.5	Mainly fire	No	No	Yes	No	Yes	No	No
0.5	Mainly fire	Yes	No	No	No	Yes	No	No
0.5	Mainly fire	Yes	No	No	Yes	Yes	No	No
3	Mainly fire	Yes	No	Yes	No	Yes	No	No
	Mainly fire	Yes	No	No	No	No	No	No
	Mainly fire	Yes	No	No	No	Yes	No	No
	Mainly fire	Yes	No	Yes	No	Yes	No	No
	Mainly fire	Yes	No	No	No	Yes	No	No
0.5	Mainly fire	No	No	No	No	No	No	No
	Mainly fire	Yes	Yes	No	Yes	Yes	No	No
1.5	Mainly fire	Yes	No	No	No	No	No	No
	Mainly fire	No	No	No	No	No	No	No
1.5	Mainly fire	Yes	No	No	No	Yes	No	No
	Mainly fire	Yes	No	No	No	Yes	No	No
2.5	Mainly fire	Yes	No	No	No	Yes	No	No
	Crop residu	Yes	No	No	No	No	No	No
0.5	Mainly fire	No	No	No	No	Yes	No	No
0.75	Mainly fire	Yes	No	No	No	No	No	No
0.5	Mainly fire	Yes	No	No	No	Yes	No	No
	Mainly fire	Yes	No	No	No	Yes	No	No
1.25	Mainly fire	No	No	No	No	No	No	No
	Mainly fire	Yes	No	No	No	No	No	No
	Mainly fire	Yes	No	No	No	Yes	No	No
1.75	Mainly fire	Yes	No	No	No	Yes	No	No
1.5	Mainly fire	Yes	No	No	No	Yes	No	No
	Mainly fire	Yes	No	No	No	Yes	No	No
1.5	Mainly fire	Yes	No	No	No	Yes	No	No
1.5	Mainly fire	Yes	No	No	No	Yes	No	No
	Mainly fire	Yes	No	No	Yes	Yes	No	No
0.75	Mainly fire	Yes	No	No	No	Yes	No	No
	Mainly fire	Yes	No	No	No	Yes	No	No
	Mainly fire	Yes	No	No	Yes	Yes	No	No
1.5	Mainly fire	Yes	No	No	No	No	No	No
1	Mainly fire	Yes	No	No	No	Yes	No	No
0.75	Mainly fire	Yes	No	No	No	Yes	No	No
1	Mainly fire	Yes	No	No	No	Yes	No	No

For peer review only

	Mainly fire	Yes	No	No	Yes	Yes	No	No
1	Mainly fire	Yes	No	No	No	Yes	No	No
0.5	Mainly fire	Yes	No	No	No	No	No	No
	Mainly fire	Yes	No	No	No	Yes	No	No
	Mainly fire	Yes	No	No	No	Yes	No	No
0.25	Mainly fire	Yes	No	No	No	No	No	No
1.5	Mainly fire	No	No	No	No	No	No	No
	Mainly fire	No	No	No	No	No	No	No
	Mainly fire	No	No	No	No	No	No	No
1.5	Mainly fire	Yes	No	No	No	Yes	No	No
1.25	Mainly fire	Yes	No	No	No	Yes	No	No
1.5	Mainly fire	Yes	No	No	No	No	No	No
	Mainly fire	Yes	No	No	No	Yes	No	No
1	Mainly fire	Yes	No	No	Yes	Yes	No	No
1.25	Mainly fire	Yes	No	No	Yes	Yes	No	No
1.5	Mainly fire	Yes	No	Yes	No	Yes	No	No
1	Mainly fire	Yes	No	Yes	No	Yes	No	No
1	Mainly fire	Yes	No	No	No	No	No	No
1.25	Mainly fire	Yes	No	No	No	Yes	No	No
0.5	Mainly fire	Yes	No	No	No	Yes	No	No
0.5	Mainly fire	Yes	No	No	No	Yes	No	No
	Mainly fire	Yes	No	No	No	No	No	No
	Mainly fire	Yes	No	No	No	No	No	No
1.25	Mainly fire	Yes	No	No	No	No	No	No
	Mainly fire	No	No	No	No	No	No	No
2.5	Mainly fire	Yes	No	No	No	Yes	No	No
0.5	Mainly fire	Yes	No	No	No	No	No	No
0.5	Mainly fire	No	No	No	No	No	No	No
	Mainly fire	Yes	No	No	No	No	No	No
	Mainly fire	Yes	No	No	No	Yes	No	No
	Mainly fire	Yes	No	No	No	Yes	No	No
2.5	Mainly fire	Yes	No	No	No	Yes	No	No
0.25	Mainly fire	Yes	No	No	No	Yes	No	No
1.5	Mainly fire	Yes	No	No	No	No	No	No
2	Mainly fire	Yes	No	No	No	No	No	No
1.5	Mainly fire	Yes	No	Yes	No	Yes	No	No
0.5	Mainly fire	Yes	No	No	No	Yes	No	No
	Mainly fire	Yes	No	No	No	Yes	No	No
1.5	Mainly fire	Yes	No	No	No	Yes	No	No
1.5	Mainly fire	Yes	No	Yes	No	Yes	No	No
1.25	Mainly fire	Yes	No	Yes	No	Yes	No	No
2	Mainly fire	Yes	No	No	No	Yes	No	No
1.5	Mainly fire	No	No	No	No	Yes	No	No
1.5	Mainly fire	Yes	No	No	No	Yes	No	No
2	Mainly fire	Yes	No	No	No	Yes	No	No
1	Mainly fire	Yes	No	No	No	Yes	No	No
	Mainly fire	Yes	No	No	No	Yes	No	No
2.5	Mainly fire	Yes	No	No	No	Yes	No	No
0.5	Mainly fire	Yes	No	No	No	Yes	No	No
0.5	Mainly fire	Yes	No	Yes	No	Yes	No	No
0.5	Mainly fire	Yes	No	Yes	No	Yes	No	No
1.5	Mainly fire	Yes	No	No	No	Yes	No	No

For peer review only

1.5	Mainly fire	Yes	No	No	No	Yes	No	No
	Mainly fire	Yes	No	No	Yes	Yes	No	No
2.5	Mainly fire	Yes	No	No	No	No	No	No
2	Mainly fire	Yes	No	No	No	No	No	No
1	Mainly fire	Yes	No	No	No	Yes	No	No
1.5	Mainly fire	Yes	No	No	No	No	No	No
	Mainly fire	Yes	No	No	Yes	Yes	No	No
1.5	Mainly fire	Yes	No	No	No	No	No	No
0.5	Mainly fire	Yes	No	No	No	Yes	No	No
3	Mainly fire	Yes	No	No	No	Yes	No	No
2	Mainly fire	Yes	No	No	Yes	Yes	No	No
4	Mainly fire	Yes	No	No	No	Yes	No	No
	Mainly fire	Yes	No	No	No	Yes	No	No
1	Mainly fire	Yes	No	No	No	Yes	No	No
999	Crop residu	Yes	No	No	No	Yes	No	No
1.25	Mainly fire	Yes	No	No	No	Yes	No	No
	Mainly fire	Yes	No	No	No	Yes	No	No
0.5	Mainly fire	Yes	No	No	No	Yes	No	No
3	Mainly fire	Yes	No	No	No	Yes	No	No
	Mainly fire	Yes	No	No	No	Yes	No	No
	Mainly fire	Yes	No	No	No	No	No	No
2	Mainly fire	Yes	No	No	No	Yes	No	No
1.25	Mainly fire	Yes	No	No	No	No	No	No
0.75	Mainly fire	Yes	Yes	No	Yes	Yes	No	No
0.5	Mainly fire	Yes	No	No	No	No	No	No
1	Mainly fire	Yes	No	No	No	No	No	No
	Mainly fire	Yes	No	No	No	Yes	No	No
999	Mainly fire	Yes	No	Yes	No	Yes	No	No
	Crop residu	Yes	No	No	No	Yes	No	No
1.5	Mainly fire	Yes	No	No	No	Yes	No	No
0.75	Mainly fire	Yes	No	No	No	Yes	No	No
1	Mainly fire	Yes	No	No	No	Yes	No	No
0.5	Mainly fire	Yes	No	No	No	No	No	No
1.5	Mainly fire	Yes	No	Yes	No	Yes	No	No
	Mainly fire	Yes	No	No	No	Yes	No	No
1	Mainly fire	Yes	No	No	No	Yes	No	No
	Mainly fire	Yes	No	Yes	No	Yes	No	No
0.5	Mainly fire	Yes	No	No	No	No	No	No
1.5	Mainly fire	Yes	No	Yes	No	Yes	No	No
3	Mainly fire	Yes	No	No	No	No	No	No
	Mainly fire	Yes	No	No	No	Yes	No	No
1.5	Mainly fire	Yes	No	No	No	Yes	No	No
1.25	Mainly fire	Yes	No	No	Yes	Yes	No	No
	Mainly fire	No	No	No	No	No	No	No
	Mainly fire	Yes	No	No	No	Yes	No	No
0.5	Mainly fire	Yes	No	No	No	Yes	No	No
	Mainly fire	Yes	No	No	No	Yes	No	No
2	Mainly fire	Yes	No	No	No	Yes	No	No
	Mainly fire	Yes	No	Yes	No	Yes	No	No
1	Mainly fire	Yes	No	No	No	No	No	No
0.5	Mainly fire	Yes	No	No	Yes	Yes	No	No
0.75	Mainly fire	Yes	No	No	No	Yes	No	No

For peer review only

1.25	Animal dur	Yes	No	No	No	No	No	No
2	Mainly fire	Yes	No	No	No	Yes	No	No
	Mainly fire	Yes	No	No	No	No	No	No
2	Mainly fire	Yes	No	No	No	No	No	No
	Mainly fire	Yes	No	Yes	No	Yes	No	No
	Mainly fire	Yes	No	No	No	Yes	No	No
	Mainly fire	No	No	No	No	No	No	No
3	Mainly fire	Yes	No	Yes	No	Yes	No	No
3	Mainly fire	No	No	No	No	Yes	No	No
0.5	Mainly fire	Yes	No	Yes	No	Yes	No	No
0.5	Mainly fire	Yes	No	No	No	Yes	No	No
0.25	Mainly fire	Yes	No	Yes	No	Yes	No	No
2	Mainly fire	Yes	No	No	No	No	No	No
0.5	Mainly fire	Yes	No	No	No	Yes	No	No
	Mainly fire	Yes	No	No	No	No	No	No
	Mainly fire	Yes	No	No	No	Yes	No	No
	Mainly fire	No	No	No	No	No	No	No
3.5	Mainly fire	Yes	No	No	No	Yes	No	No
1	Mainly fire	Yes	No	No	No	Yes	No	No
	Mainly fire	Yes	No	No	No	Yes	No	No
	Mainly fire	Yes	No	Yes	No	Yes	No	No
2	Mainly fire	Yes	No	No	No	Yes	No	No
2.25	Mainly fire	Yes	No	No	No	Yes	No	No
1.25	Mainly fire	No	No	No	No	Yes	No	No
1	Mainly fire	Yes	No	No	No	Yes	No	No
2	Mainly fire	Yes	No	No	No	Yes	No	No
999	Mainly fire	Yes	No	No	No	Yes	No	No
2.5	Mainly fire	Yes	No	Yes	No	No	No	No
1	Mainly fire	Yes	No	No	No	Yes	No	No
999	Mainly fire	Yes	No	No	No	Yes	No	No
0.5	Mainly fire	Yes	No	No	No	Yes	No	No
	Mainly fire	Yes	No	No	No	No	No	No
1.5	Mainly fire	Yes	No	No	No	No	No	No
	Mainly fire	Yes	No	No	No	No	No	No
2	Mainly fire	Yes	No	No	No	Yes	No	No
0.5	Mainly fire	Yes	No	No	No	No	No	No
1.2	Mainly fire	Yes	No	No	No	Yes	No	No
	Mainly fire	Yes	No	Yes	No	Yes	No	No
3	Mainly fire	Yes	No	No	No	Yes	No	No
1.5	Mainly fire	Yes	No	No	No	Yes	No	No
	Mainly fire	Yes	No	No	No	No	No	No
	Mainly fire	Yes	No	No	No	No	No	No
	Mainly fire	Yes	No	No	No	No	No	No
0.5	Mainly fire	Yes	No	No	No	Yes	No	No
	Mainly fire	Yes	No	No	No	Yes	No	No
3	Mainly fire	No	No	No	No	Yes	No	No
1.5	Mainly fire	No	No	No	No	Yes	No	No
1.75	Mainly fire	Yes	No	No	No	Yes	No	No
	Mainly fire	No	No	Yes	No	Yes	No	No
0.5	Mainly fire	No	No	Yes	No	No	No	No
0.25	Mainly fire	No	No	No	No	Yes	No	No
	Mainly fire	Yes	No	Yes	No	Yes	No	No

2	Mainly fire	No	No	No	No	Yes	No	No
	Mainly fire	Yes	No	Yes	No	Yes	No	No
	Mainly fire	Yes	No	Yes	No	Yes	No	No
0.25	Mainly fire	Yes	No	Yes	No	Yes	No	No
1.5	Animal dur	Yes	No	Yes	No	Yes	No	No
0.5	Animal dur	Yes	No	No	No	Yes	No	No
0.5	Mainly fire	No	No	No	No	Yes	No	No
2	Animal dur	Yes	No	Yes	No	Yes	No	No
0.5	Mainly fire	No	No	Yes	No	Yes	No	No
0.5	Mainly fire	No	No	Yes	No	Yes	No	No
1	Animal dur	No	No	Yes	No	Yes	No	No
0.4	Mainly fire	No	No	No	No	Yes	No	No
0.5	Mainly fire	No	No	No	No	Yes	No	No
2	Mainly fire	Yes	No	Yes	Yes	Yes	No	No
1	Mainly fire	Yes	No	No	No	No	No	No
	Animal dur	Yes	No	Yes	No	Yes	No	No
	Animal dur	No	No	Yes	No	Yes	No	No
0.5	Mainly fire	No	No	No	No	Yes	No	No
1	Animal dur	Yes	No	Yes	No	Yes	No	No
1.5	Animal dur	No	No	Yes	No	Yes	No	No
0.5	Animal dur	Yes	No	No	No	Yes	No	No
0.5	Mainly fire	No	Yes	No	No	Yes	No	No
2	Animal dur	Yes	No	Yes	No	No	No	No
0.5	Animal dur	Yes	No	Yes	No	Yes	No	No
1	Animal dur	Yes	No	Yes	No	Yes	No	No
	Charcoal	Yes	No	Yes	No	Yes	No	No
0.5	Animal dur	Yes	No	Yes	No	Yes	No	No
0.25	Mainly fire	Yes	Yes	Yes	No	Yes	No	No
1	Mainly fire	No	No	Yes	No	Yes	No	No
0.5	Animal dur	Yes	No	Yes	No	Yes	No	No
0.5	Animal dur	No	No	Yes	No	Yes	No	No
	Mainly fire	Yes	No	No	No	Yes	No	No
2	Mainly fire	Yes	No	No	No	Yes	No	No
3	Mainly fire	Yes	No	Yes	No	No	No	No
0.5	Mainly fire	Yes	No	Yes	No	Yes	No	No
1	Mainly fire	No	No	No	No	Yes	No	No
0.5	Mainly fire	Yes	Yes	Yes	No	Yes	No	No
0.25	Animal dur	Yes	No	No	No	Yes	No	No
0.5	Mainly fire	Yes	No	No	No	Yes	No	No
1	Animal dur	Yes	No	No	No	Yes	No	No
	Mainly fire	No	No	Yes	No	Yes	No	No
2	Mainly fire	No	No	No	No	No	No	No
0.5	Animal dur	Yes	No	Yes	No	Yes	No	No
0.25	Mainly fire	Yes	No	Yes	No	Yes	No	No
1.5	Mainly fire	Yes	No	Yes	No	Yes	No	No
0.5	Mainly fire	Yes	No	Yes	No	Yes	No	No
	Mainly fire	No	No	No	No	No	No	No
	Mainly fire	No	Yes	Yes	No	Yes	No	No
0.75	Mainly fire	Yes	No	Yes	No	Yes	No	No
	Mainly fire	Yes	No	No	No	Yes	No	No
0.5	Mainly fire	No	No	Yes	No	Yes	No	No
1.5	Animal dur	Yes	No	Yes	No	Yes	No	No

For peer review only

2	Mainly fire	Yes	No	Yes	Yes	No	Yes	No
	Mainly fire	No	No	No	No	No	No	No
1	Mainly fire	No	No	Yes	No	Yes	No	No
1	Mainly fire	Yes	No	No	No	Yes	No	No
	Mainly fire	No	No	No	No	No	No	No
1	Animal dur	Yes	No	No	No	Yes	No	No
0.5	Mainly fire	No	No	No	Yes	No	No	No
1.5	Animal dur	No	No	Yes	No	Yes	No	No
1.5	Animal dur	No	No	Yes	No	Yes	No	No
0.5	Animal dur	Yes	No	Yes	No	Yes	No	No
1.5	Mainly fire	Yes	No	No	No	Yes	No	No
1.25	Mainly fire	Yes	No	No	No	Yes	No	No
0.5	Mainly fire	Yes	No	Yes	No	Yes	No	No
0.5	Mainly fire	Yes	No	No	No	Yes	No	No
0.25	Animal dur	Yes	No	Yes	Yes	Yes	No	No
0.75	Mainly fire	Yes	No	No	No	No	No	No
0.75	Charcoal	Yes	No	Yes	No	Yes	No	No
2	Mainly fire	Yes	No	No	No	Yes	No	No
	Mainly fire	Yes	No	No	No	Yes	No	No
0.75	Mainly fire	No	No	Yes	No	Yes	No	No
3	Mainly fire	No	No	No	No	No	No	No
0.25	Mainly fire	Yes	No	No	No	Yes	No	No
0.75	Mainly fire	Yes	No	No	No	No	No	No
2	Mainly fire	Yes	No	No	No	Yes	No	No
0.5	Mainly fire	No	Yes	Yes	No	Yes	No	No
0.5	Mainly fire	Yes	Yes	Yes	Yes	Yes	No	No
5	Mainly fire	No	No	Yes	No	No	No	No
1	Animal dur	Yes	No	Yes	No	Yes	No	No
0.5	Mainly fire	Yes	No	No	No	Yes	No	Yes
0.5	Mainly fire	Yes	No	No	No	Yes	No	No
0.5	Mainly fire	No	No	No	No	No	No	No
0.75	Mainly fire	No	No	No	No	Yes	No	No
	Electricity	Yes	Yes	Yes	Yes	Yes	No	No
1	Animal dur	No	No	Yes	No	Yes	No	No
2	Mainly fire	No	No	No	No	Yes	No	No
0.75	Animal dur	Yes	No	No	No	Yes	No	No
2	Mainly fire	No	No	Yes	No	Yes	No	No
0.5	Animal dur	No	No	Yes	No	Yes	No	No
1	Mainly fire	No	Yes	Yes	No	Yes	No	No
0.2	Mainly fire	No	No	No	No	Yes	No	No
	Mainly fire	No	No	Yes	No	Yes	No	No
2	Mainly fire	No	No	No	No	No	No	No
0.2	Mainly fire	No	No	No	No	Yes	No	No
0.75	Mainly fire	Yes	No	No	No	No	No	No
3	Animal dur	No	Yes	Yes	No	Yes	No	No
0.25	Mainly fire	No	No	No	No	No	No	No
	Mainly fire	Yes	No	Yes	No	Yes	No	No
0.25	Mainly fire	Yes	Yes	Yes	No	Yes	No	No
1.25	Mainly fire	No	Yes	Yes	No	Yes	No	No
0.5	Mainly fire	No	No	Yes	No	Yes	No	No
0.5	Animal dur	Yes	No	Yes	No	Yes	No	No
0.5	Mainly fire	Yes	No	No	Yes	Yes	No	No

For peer review only

0.75	Mainly fire	No	No	Yes	No	Yes	No	No
1.5	Mainly fire	No	No	Yes	No	Yes	No	No
1.5	Animal dur	Yes	No	Yes	No	Yes	No	No
3.5	Mainly fire	Yes	No	No	No	Yes	No	No
2	Mainly fire	No	No	No	No	Yes	No	No
	Animal dur	Yes	No	Yes	Yes	Yes	No	No
2	Mainly fire	No	No	Yes	No	Yes	No	No
0.5	Mainly fire	No	No	Yes	No	Yes	No	No
1	Mainly fire	Yes	No	No	No	Yes	No	No
	Mainly fire	Yes	No	Yes	No	Yes	No	No
0.25	Mainly fire	No	No	Yes	No	Yes	No	No
0.5	Mainly fire	Yes	No	Yes	No	Yes	No	No
	Mainly fire	Yes	No	Yes	No	No	No	No
	Animal dur	No	No	Yes	No	No	No	No
1	Animal dur	Yes	No	No	No	Yes	No	No
1	Animal dur	No	No	No	No	Yes	No	No
	Animal dur	Yes	No	Yes	No	Yes	No	No
3	Mainly fire	No	No	Yes	No	Yes	No	No
1.25	Mainly fire	No	No	No	No	Yes	No	No
2	Mainly fire	No	No	No	No	No	No	No
	Mainly fire	No	No	Yes	No	Yes	No	No
	Mainly fire	Yes	No	No	No	No	No	No
0.75	Mainly fire	Yes	No	No	Yes	Yes	No	No
1	Mainly fire	No	No	No	No	No	No	No
	Mainly fire	Yes	Yes	Yes	Yes	Yes	No	No
	Charcoal	Yes	No	No	No	Yes	No	No
	Mainly fire	Yes	Yes	Yes	No	Yes	No	No
0.5	Mainly fire	Yes	Yes	Yes	No	Yes	No	No
0.25	Animal dur	Yes	No	No	No	Yes	No	No
1	Mainly fire	Yes	No	No	No	No	No	No
	Mainly fire	Yes	Yes	Yes	Yes	Yes	No	No
0.5	Mainly fire	No	Yes	Yes	No	Yes	No	No
1	Animal dur	Yes	No	Yes	No	Yes	No	No
0.25	Animal dur	Yes	No	No	No	Yes	No	No
0.5	Mainly fire	No	No	Yes	No	No	No	No
0.25	Mainly fire	Yes	Yes	Yes	Yes	Yes	No	No
0.5	Mainly fire	No	No	Yes	No	Yes	No	No
6	Mainly fire	Yes	No	No	Yes	Yes	No	No
0.5	Mainly fire	Yes	No	Yes	No	Yes	No	No
3	Animal dur	Yes	No	Yes	No	Yes	No	No
0.25	Mainly fire	No	No	No	No	Yes	No	No
0.25	Mainly fire	No	No	No	No	Yes	No	No
0.5	Mainly fire	No	No	No	No	Yes	No	No
0.25	Mainly fire	Yes	Yes	Yes	No	Yes	No	No
1	Mainly fire	Yes	No	No	No	No	No	No
3	Mainly fire	No	No	Yes	No	Yes	No	No
0.5	Mainly fire	Yes	No	No	No	No	No	No
2	Animal dur	Yes	No	Yes	No	Yes	No	No
	Mainly fire	No	No	Yes	No	Yes	No	No
0.5	Animal dur	No	No	No	No	No	No	No
	Mainly fire	Yes	No	No	No	Yes	No	No
	Animal dur	Yes	No	Yes	No	Yes	No	No

For peer review only

	Mainly fire'	No	No	No	No	Yes	No	No
0.5	Mainly fire'	No	No	Yes	No	Yes	No	No
0.5	Animal dur	Yes	No	Yes	No	Yes	No	No
3	Mainly fire'	No	No	No	No	No	No	No
0.25	Mainly fire'	Yes	No	Yes	No	Yes	No	No
0.5	Mainly fire'	No	Yes	Yes	No	Yes	No	No
0.5	Mainly fire'	No	No	No	No	Yes	No	No
0.25	Mainly fire'	Yes	No	No	No	No	No	No
	Animal dur	Yes	No	Yes	No	Yes	No	No
0.5	Mainly fire'	No	No	No	No	Yes	No	No
	Mainly fire'	No	No	No	No	No	No	No
0.5	Animal dur	Yes	No	Yes	No	Yes	No	No
3	Mainly fire'	Yes	No	Yes	No	No	No	No
	Mainly fire'	No	No	Yes	No	Yes	No	No
1	Mainly fire'	No	No	No	No	No	No	No
0.75	Animal dur	No	No	Yes	No	Yes	No	No
0.25	Mainly fire'	No	No	Yes	No	Yes	No	No
0.5	Mainly fire'	Yes	No	No	No	Yes	No	No
0.5	Mainly fire'	No	No	No	No	Yes	No	Yes
1	Mainly fire'	No	No	No	No	No	No	No
	Mainly fire'	Yes	No	Yes	No	No	No	No
	Mainly fire'	Yes	No	Yes	No	Yes	No	No
1	Mainly fire'	No	No	No	No	No	No	No
	Mainly fire'	No	No	No	No	Yes	No	No
3	Mainly fire'	Yes	No	No	No	Yes	No	No
0.5	Mainly fire'	Yes	No	Yes	No	Yes	No	No
1.5	Animal dur	No	No	Yes	No	Yes	No	No
0.75	Mainly fire'	Yes	No	Yes	No	Yes	No	No
1	Mainly fire'	Yes	No	No	No	No	No	No
1.5	Mainly fire'	Yes	No	No	No	No	No	No
1.25	Mainly fire'	No	No	Yes	No	Yes	No	No
1.25	Mainly fire'	No	No	Yes	No	Yes	No	No
0.25	Mainly fire'	No	No	No	No	No	No	No
	Mainly fire'	Yes	No	No	No	No	No	No
0.5	Animal dur	Yes	No	No	No	Yes	No	No
3	Mainly fire'	Yes	No	Yes	No	Yes	No	No
2	Mainly fire'	Yes	No	No	No	Yes	No	No
2	Animal dur	Yes	No	Yes	No	No	Yes	No
1	Animal dur	No	No	No	No	Yes	No	No
0.25	Mainly fire'	No	No	No	No	No	No	No
0.5	Animal dur	Yes	No	No	No	No	No	No
1	Mainly fire'	No	No	No	No	No	No	No
0.5	Mainly fire'	Yes	Yes	Yes	No	Yes	No	No
1.5	Animal dur	Yes	No	Yes	No	Yes	No	No
0.25	Mainly fire'	No	No	No	No	Yes	No	No
	Animal dur	Yes	No	No	No	Yes	No	No
0.5	Mainly fire'	Yes	No	No	No	Yes	No	No
	Mainly fire'	No	No	No	No	Yes	No	No
0.5	Animal dur	Yes	No	Yes	No	Yes	No	No
2	Mainly fire'	No	No	No	No	Yes	No	No
0.5	Mainly fire'	No	No	Yes	No	Yes	No	No
1.5	Mainly fire'	Yes	No	No	No	Yes	No	No

0.5	Mainly fire	Yes	No	No	No	Yes	No	No
0.5	Animal dur	Yes	No	Yes	No	Yes	No	No
3	Mainly fire	No	No	Yes	No	Yes	No	No
0.25	Animal dur	Yes	No	No	No	Yes	No	No
1	Mainly fire	Yes	No	No	No	No	No	No
0.5	Mainly fire	Yes	No	No	No	Yes	No	No
3	Mainly fire	Yes	No	No	No	Yes	No	No
0.25	Animal dur	Yes	No	No	No	No	No	No
1	Mainly fire	No	No	No	No	Yes	No	No
0.2	Mainly fire	No	No	Yes	No	Yes	No	No
0.5	Mainly fire	No	No	No	No	No	No	No
	Mainly fire	No	No	Yes	No	Yes	No	No
1.25	Mainly fire	Yes	No	No	No	No	No	No
	Animal dur	No	No	Yes	No	Yes	No	No
	Mainly fire	Yes	No	No	Yes	No	No	No
0.25	Mainly fire	No	No	Yes	No	No	No	No
	Charcoal	Yes	No	Yes	No	Yes	No	No
1.25	Mainly fire	No	No	No	No	No	No	No
5	Mainly fire	No	No	Yes	No	No	No	No
	Mainly fire	Yes	No	Yes	No	Yes	No	No
	Mainly fire	No	No	No	No	Yes	No	No
0.75	Animal dur	Yes	No	Yes	No	Yes	No	No
	Mainly fire	Yes	No	No	No	Yes	No	No
1.5	Mainly fire	Yes	No	No	No	Yes	No	No
0.25	Animal dur	Yes	No	No	No	Yes	No	No
2	Mainly fire	No	No	No	No	No	No	No
	Mainly fire	Yes	No	No	No	No	No	No
1	Mainly fire	Yes	No	No	No	Yes	No	No
0.75	Mainly fire	Yes	No	Yes	No	Yes	No	No
0.5	Animal dur	Yes	No	Yes	No	Yes	No	No
0.5	Mainly fire	Yes	No	No	No	Yes	No	No
1	Mainly fire	Yes	No	No	No	No	No	No
2	Mainly fire	No	No	No	No	No	No	No
2	Mainly fire	No	No	No	No	No	No	No
	Mainly fire	No	No	Yes	No	Yes	No	No
0.5	Mainly fire	No	No	No	No	Yes	No	No
0.5	Mainly fire	Yes	No	Yes	No	Yes	No	No
0.5	Mainly fire	No	No	No	No	No	No	No
	Mainly fire	No	No	No	No	Yes	No	No
0.5	Mainly fire	No	No	No	No	No	No	No
1	Mainly fire	No	No	No	No	Yes	No	No
3	Mainly fire	No	No	Yes	No	No	No	No
1	Mainly fire	Yes	No	No	No	No	No	No
0.5	Animal dur	No	No	No	No	Yes	No	No
2	Mainly fire	Yes	No	Yes	No	Yes	No	No
1.5	Mainly fire	No	No	Yes	No	Yes	No	No
	Mainly fire	No	No	Yes	No	Yes	No	No
0.5	Mainly fire	Yes	No	No	No	No	No	No
0.5	Mainly fire	No	No	Yes	No	Yes	No	No
0.25	Animal dur	Yes	No	No	No	Yes	No	No
0.25	Mainly fire	Yes	No	No	No	Yes	No	No
5	Mainly fire	No	No	Yes	No	Yes	No	No

For peer review only

0.25	Mainly fire	No	No	No	No	Yes	No	No
2	Mainly fire	No	No	No	No	No	No	No
	Animal dur	No	No	Yes	No	Yes	No	No
2	Animal dur	No	No	No	No	No	No	No
0.25	Animal dur	Yes	No	Yes	No	Yes	No	No
	Mainly fire	No	No	Yes	No	Yes	No	No
0.5	Mainly fire	Yes	No	No	No	No	No	No
3	Mainly fire	Yes	No	No	No	Yes	No	Yes
0.75	Mainly fire	Yes	No	No	No	No	No	No
3	Mainly fire	No	No	No	No	Yes	No	No
0.75	Mainly fire	No	No	No	No	Yes	No	No
2	Mainly fire	No	No	No	No	No	No	No
0.25	Mainly fire	Yes	No	Yes	No	Yes	No	No
3	Mainly fire	No	No	No	No	No	No	No
1	Mainly fire	No	No	No	No	No	No	No
0.5	Mainly fire	No	Yes	No	No	No	No	No
	Mainly fire	No	No	Yes	No	Yes	No	No
2	Mainly fire	Yes	No	No	No	No	No	No
0.25	Mainly fire	Yes	Yes	Yes	No	Yes	No	No
0.5	Mainly fire	Yes	No	Yes	No	Yes	No	No
	Mainly fire	Yes	No	No	No	Yes	No	No
1	Mainly fire	Yes	No	No	No	No	No	No
0.5	Animal dur	No	No	No	No	Yes	No	No
3	Mainly fire	No	No	Yes	No	No	No	No
	Charcoal	Yes	No	No	Yes	Yes	No	No
2	Mainly fire	No	Yes	Yes	No	Yes	No	No
0.75	Mainly fire	No	No	No	No	Yes	No	No
	Animal dur	Yes	No	Yes	No	Yes	No	No
0.5	Charcoal	No	No	Yes	No	Yes	No	No
1.25	Mainly fire	Yes	No	No	Yes	Yes	No	No
3	Mainly fire	Yes	No	No	No	No	No	No
	Mainly fire	Yes	Yes	No	Yes	Yes	No	No
2	Mainly fire	No	No	No	No	No	No	No
0.5	Animal dur	Yes	No	No	No	Yes	No	No
1	Mainly fire	Yes	No	No	No	Yes	No	No
	Mainly fire	No	No	Yes	No	Yes	No	No
2	Mainly fire	No	No	Yes	No	No	No	No
2	Mainly fire	No	No	Yes	No	Yes	No	No
1.5	Mainly fire	No	No	Yes	No	Yes	No	No
0.5	Animal dur	No	No	No	No	Yes	No	No
	Mainly fire	Yes	No	No	No	Yes	No	No
	Mainly fire	No	No	Yes	No	Yes	No	No
0.75	Mainly fire	No	No	Yes	No	Yes	No	No
3	Mainly fire	Yes	No	Yes	No	No	No	No
	Mainly fire	No	No	Yes	No	Yes	No	No
	Mainly fire	No	No	No	No	Yes	No	No
1.25	Mainly fire	Yes	No	Yes	No	Yes	No	No
0.5	Mainly fire	Yes	Yes	Yes	Yes	Yes	No	No
0.75	Mainly fire	No	No	Yes	No	Yes	No	No
0.5	Animal dur	Yes	No	Yes	Yes	Yes	No	No
1	Mainly fire	No	No	Yes	No	Yes	No	No
1.5	Mainly fire	No	No	Yes	No	Yes	No	No

For peer review only

0.5	Mainly fire	Yes	No	Yes	No	Yes	No	No
	Mainly fire	Yes	No	No	No	No	No	No
	Mainly fire	No	No	Yes	No	Yes	No	No
3	Animal dur	No	No	No	No	No	No	No
0.1	Mainly fire	No	No	Yes	No	Yes	No	No
0.5	Animal dur	Yes	No	Yes	No	Yes	No	No
0.5	Mainly fire	Yes	No	No	No	Yes	No	No
2	Mainly fire	Yes	No	Yes	No	Yes	No	No
0.5	Animal dur	No	No	Yes	No	Yes	No	No
0.5	Mainly fire	No	No	Yes	No	Yes	No	No
0.5	Charcoal	Yes	No	Yes	No	Yes	No	No
2	Mainly fire	Yes	No	No	No	Yes	No	No
0.5	Animal dur	No	No	Yes	No	Yes	No	No
3	Mainly fire	No	No	No	No	No	No	No
0.75	Mainly fire	No	No	No	No	Yes	No	No
1	Animal dur	No	No	Yes	No	Yes	No	No
	Mainly fire	Yes	Yes	Yes	Yes	Yes	No	No
2	Mainly fire	No	No	No	No	No	No	No
0.25	Mainly fire	Yes	No	No	No	No	No	No
2	Mainly fire	Yes	No	Yes	No	Yes	No	No
1.5	Animal dur	Yes	No	Yes	No	Yes	No	No
0.5	Mainly fire	No	No	Yes	No	Yes	No	No
2	Mainly fire	Yes	No	No	No	Yes	No	No
3	Mainly fire	No	No	No	No	Yes	No	No
5	Mainly fire	No	No	Yes	No	No	No	No
0.5	Animal dur	Yes	No	No	No	Yes	No	No
	Animal dur	No	No	Yes	No	Yes	No	No
0.5	Animal dur	Yes	No	Yes	No	Yes	No	No
	Mainly fire	No	No	No	No	Yes	No	No
1	Mainly fire	No	No	Yes	No	No	No	No
1.5	Animal dur	Yes	No	Yes	No	Yes	No	No
	Mainly fire	No	Yes	Yes	No	Yes	No	No
0.5	Mainly fire	Yes	Yes	Yes	Yes	Yes	No	No
0.5	Animal dur	No	No	Yes	No	Yes	No	No
3	Animal dur	No	No	Yes	No	Yes	No	No
999	Mainly fire	Yes	No	No	No	Yes	No	No
0.5	Mainly fire	No	No	Yes	No	Yes	No	No
1.75	Animal dur	Yes	No	Yes	No	Yes	No	No
	Charcoal	Yes	No	No	Yes	Yes	No	Yes
0.5	Animal dur	Yes	No	Yes	No	Yes	No	No
5	Mainly fire	Yes	No	Yes	No	No	No	No
1.5	Animal dur	Yes	No	Yes	No	Yes	No	No
2	Mainly fire	Yes	No	No	No	No	No	No
	Mainly fire	Yes	No	Yes	No	Yes	No	No
1	Animal dur	No	No	Yes	No	Yes	No	No
1	Mainly fire	No	Yes	No	No	Yes	No	No
1	Animal dur	No	No	Yes	No	Yes	No	No
0.5	Mainly fire	No	No	Yes	No	Yes	No	No
10	Charcoal	Yes	No	Yes	No	Yes	No	No
3	Mainly fire	Yes	No	Yes	No	Yes	No	No
0.75	Mainly fire	No	No	Yes	No	Yes	No	No

For peer review only

q230h	q230i	q230j	q230k	q230l	q230m	q230n	q232a	q232b
No	Yes	Yes	No	No	Yes	No	10	6
No	No	Yes	No	No	Yes	No	0	0
No	No	Yes	No	No	Yes	No	6	0
No	No	No	No	Yes	Yes	Yes	0	0
Yes	Yes	Yes	No	No	Yes	No	7	10
No	No	Yes	No	No	Yes	No	0	0
No	No	Yes	No	No	Yes	No	0	0
No	No	No	No	Yes	Yes	Yes	0	0
No	No	Yes	No	No	Yes	No	0	15
No	No	No	No	No	Yes	No	0	0
No	No	Yes	No	No	Yes	No	30	0
Yes	No	Yes	No	No	Yes	No	10	0
No	No	Yes	No	No	Yes	No	12	0
No	No	Yes	No	No	Yes	Yes	0	0
No	No	Yes	No	No	Yes	No	15	0
No	No	Yes	No	No	Yes	No	1	0
No	No	No	No	Yes	Yes	Yes	4	0
No	No	Yes	No	No	Yes	No	4	0
No	No	Yes	No	Yes	Yes	No	0	0
No	No	Yes	No	No	Yes	Yes	14	0
No	No	No	No	Yes	No	No	0	0
No	No	No	No	No	Yes	No	2	0
Yes	Yes	Yes	No	No	Yes	No	1	0
No	No	No	No	Yes	Yes	Yes	0	0
Yes	Yes	Yes	No	No	Yes	No	7	0
No	No	Yes	No	No	Yes	No	0	0
No	No	Yes	No	No	Yes	No	4	0
Yes	Yes	Yes	No	Yes	Yes	No	4	0
Yes	No	Yes	No	No	Yes	No	0	0
Yes	No	Yes	No	Yes	Yes	Yes	0	0
No	No	No	No	No	Yes	No	2	0
No	No	Yes	No	No	Yes	No	0	0
Yes	No	Yes	No	No	Yes	No	0	0
No	No	No	No	Yes	Yes	Yes	0	0
No	No	Yes	No	Yes	Yes	No	5	0
Yes	Yes	Yes	No	Yes	Yes	Yes	0	0
No	No	Yes	No	No	Yes	No	0	3
No	No	Yes	No	Yes	Yes	No	12	0
Yes	No	Yes	No	No	Yes	No	10	0
No	No	Yes	No	No	Yes	No	15	15
No	No	Yes	No	Yes	Yes	Yes	0	0
No	No	Yes	No	No	Yes	No	4	0
Yes	Yes	Yes	No	No	Yes	No	10	0
No	No	No	No	Yes	Yes	Yes	0	0
No	No	Yes	No	No	Yes	No	0	0
No	Yes	No	No	Yes	Yes	No	0	0
No	No	Yes	No	Yes	Yes	No	8	0
No	No	Yes	No	No	Yes	No	3	0
Yes	Yes	Yes	No	Yes	No	No	0	0
No	No	Yes	No	No	Yes	No	2	0
No	Yes	No	No	Yes	Yes	Yes	0	0

For peer review only

No	No	Yes	No	Yes	Yes	Yes	4	0
No	No	Yes	No	No	Yes	No	4	10
Yes	Yes	Yes	No	No	Yes	No	6	0
Yes	Yes	Yes	No	Yes	Yes	No	2	0
No	No	Yes	No	No	Yes	No	0	0
No	No	Yes	No	Yes	Yes	No	0	0
Yes	No	Yes	No	No	Yes	No	2	0
No	No	No	No	Yes	Yes	Yes	0	0
Yes	No	Yes	No	No	Yes	No	4	0
No	Yes	Yes	No	No	Yes	No	0	0
No	No	No	No	No	No	No	0	0
No	No	Yes	No	No	Yes	No	0	0
No	No	Yes	No	No	Yes	No	0	0
Yes	No	Yes	No	No	Yes	No	2	0
No	Yes	Yes	No	No	Yes	Yes	0	0
Yes	Yes	Yes	No	No	Yes	No	10	0
No	No	Yes	No	No	Yes	No	8	0
Yes	Yes	No	No	Yes	Yes	Yes	0	0
No	No	No	No	Yes	Yes	No	0	0
Yes	No	Yes	No	No	Yes	No	6	0
No	No	Yes	No	No	Yes	No	1	0
No	No	Yes	No	Yes	Yes	No	6	0
No	No	No	No	No	Yes	No	4	0
No	No	No	No	Yes	No	No	0	0
No	No	Yes	No	No	Yes	No	15	0
No	No	Yes	No	No	Yes	No	10	0
No	No	Yes	No	No	Yes	No	12	0
No	No	Yes	No	No	Yes	No	0	0
Yes	Yes	No	No	Yes	Yes	Yes	0	0
No	No	No	No	Yes	Yes	Yes	0	0
No	No	Yes	No	No	Yes	No	10	0
Yes	Yes	Yes	No	Yes	Yes	Yes	0	0
No	No	Yes	No	No	Yes	No	0	0
Yes	Yes	Yes	No	Yes	Yes	Yes	0	0
No	No	No	No	Yes	Yes	Yes	0	0
No	No	Yes	No	No	Yes	No	15	0
No	No	No	No	Yes	Yes	No	0	0
No	No	Yes	No	Yes	Yes	No	0	0
No	No	Yes	No	Yes	Yes	No	0	0
No	No	No	No	Yes	Yes	No	0	0
No	No	Yes	No	Yes	Yes	No	0	0
Yes	Yes	Yes	No	No	Yes	No	10	0
Yes	Yes	Yes	No	Yes	Yes	Yes	0	5
No	No	No	No	No	Yes	No	0	0
No	No	No	No	Yes	Yes	Yes	0	5
Yes	Yes	Yes	No	No	No	No	0	0
No	No	Yes	No	No	Yes	No	0	0
No	Yes	Yes	No	Yes	Yes	Yes	0	0
Yes	Yes	Yes	No	Yes	Yes	Yes	5	0
No	No	No	No	Yes	Yes	Yes	0	0
Yes	Yes	Yes	No	Yes	Yes	Yes	0	0
Yes	Yes	Yes	No	Yes	Yes	Yes	2	0

For peer review only

No	No	Yes	No	Yes	Yes	No	0	0
No	No	Yes	No	No	Yes	No	0	0
No	No	No	No	No	Yes	No	50	0
No	No	Yes	No	No	Yes	No	0	0
No	No	No	No	No	Yes	No	0	0
No	No	No	No	Yes	No	No	0	0
No	No	Yes	No	Yes	Yes	Yes	0	0
No	No	No	No	Yes	Yes	Yes	0	2
No	No	No	No	Yes	Yes	Yes	2	0
Yes	Yes	Yes	No	No	Yes	No	10	9
Yes	No	Yes	No	No	Yes	No	5	0
No	No	Yes	No	No	Yes	No	10	0
No	No	Yes	No	No	Yes	No	2	0
No	No	Yes	No	No	Yes	No	0	0
Yes	Yes	Yes	No	No	Yes	No	6	0
No	No	Yes	No	No	Yes	No	8	0
Yes	No	Yes	No	No	Yes	No	4	0
No	No	No	No	No	Yes	No	0	0
No	No	No	No	Yes	Yes	No	6	0
Yes	Yes	Yes	No	Yes	Yes	Yes	2	0
No	No	Yes	No	No	Yes	Yes	5	0
No	No	No	No	No	Yes	No	0	0
No	No	No	No	Yes	Yes	Yes	0	0
No	No	Yes	No	No	Yes	Yes	2	0
Yes	No	Yes	No	Yes	Yes	No	0	0
No	No	Yes	No	No	Yes	No	3	0
No	No	Yes	No	No	Yes	No	5	0
No	No	No	No	Yes	Yes	Yes	5	0
No	No	No	No	No	Yes	No	0	0
No	No	Yes	No	No	Yes	Yes	0	0
Yes	Yes	Yes	No	No	Yes	No	0	0
No	Yes	Yes	No	No	Yes	No	5	0
No	No	No	No	Yes	Yes	No	0	0
Yes	Yes	Yes	No	No	Yes	No	3	0
Yes	No	Yes	No	No	Yes	Yes	0	0
No	No	Yes	No	No	Yes	No	2	0
No	No	Yes	No	No	Yes	No	2	0
No	No	Yes	No	No	Yes	No	3	0
No	Yes	Yes	No	No	Yes	No	5	0
No	No	Yes	No	No	Yes	No	3	0
No	No	No	No	Yes	Yes	Yes	0	0
No	No	Yes	No	Yes	Yes	No	2	0
Yes	Yes	Yes	No	Yes	Yes	Yes	10	0
Yes	Yes	Yes	No	No	Yes	No	2	0
No	No	No	No	Yes	Yes	No	2	0
No	No	No	No	No	Yes	No	0	0
No	No	Yes	No	No	Yes	No	2	0
Yes	Yes	Yes	No	No	Yes	No	5	0
No	No	Yes	No	Yes	Yes	No	5	0
Yes	Yes	Yes	No	No	Yes	Yes	0	0
Yes	Yes	Yes	No	No	Yes	No	2	0
No	Yes	Yes	No	Yes	Yes	Yes	0	0

For peer review only

Yes	Yes	Yes	No	No	Yes	No	4	0
Yes	No	Yes	No	Yes	Yes	Yes	4	0
No	No	No	No	Yes	Yes	No	10	0
Yes	Yes	Yes	No	No	Yes	No	0	0
No	No	No	No	Yes	Yes	Yes	0	0
No	No	Yes	No	No	Yes	No	3	0
Yes	Yes	Yes	No	No	Yes	No	10	0
No	No	Yes	No	No	Yes	Yes	0	0
Yes	Yes	Yes	No	No	Yes	No	3	0
No	No	Yes	No	No	Yes	No	10	0
No	No	No	No	No	Yes	No	0	0
Yes	Yes	Yes	No	No	Yes	No	4	0
No	No	No	No	No	Yes	No	0	0
No	No	Yes	No	No	Yes	No	2	0
No	No	Yes	No	No	Yes	No	2	0
No	No	Yes	No	No	Yes	No	0	0
No	Yes	Yes	No	No	Yes	No	5	0
Yes	No	Yes	No	No	Yes	No	0	0
Yes	No	Yes	No	No	Yes	No	8	0
No	No	Yes	No	No	Yes	No	0	0
No	No	Yes	No	Yes	Yes	No	4	0
No	No	Yes	No	No	Yes	Yes	3	0
No	No	Yes	No	No	Yes	No	5	0
Yes	Yes	Yes	No	No	Yes	Yes	0	0
Yes	Yes	Yes	No	No	No	No	0	0
Yes	Yes	Yes	No	No	Yes	No	3	0
No	No	Yes	No	No	Yes	No	7	0
No	Yes	Yes	No	No	Yes	No	0	0
No	No	Yes	No	No	Yes	No	2	0
Yes	Yes	Yes	No	No	Yes	No	1	0
No	No	No	No	Yes	Yes	Yes	0	0
No	No	No	No	Yes	Yes	Yes	0	0
No	Yes	Yes	No	No	Yes	No	5	0
Yes	Yes	Yes	No	No	Yes	No	5	0
No	No	No	No	No	Yes	No	0	0
No	No	No	No	No	Yes	No	0	0
Yes	Yes	Yes	No	No	Yes	No	3	0
Yes	Yes	Yes	No	No	Yes	Yes	0	0
Yes	Yes	Yes	No	No	Yes	No	0	0
No	No	Yes	No	No	Yes	Yes	0	0
No	No	Yes	No	No	Yes	No	2	0
Yes	Yes	Yes	No	No	Yes	No	4	0
Yes	Yes	Yes	No	Yes	Yes	No	2	0
Yes	Yes	No	No	No	Yes	Yes	0	0
No	Yes	Yes	No	No	Yes	No	0	0
Yes	No	Yes	No	No	Yes	No	1	0
No	No	Yes	No	No	Yes	No	7	2
Yes	Yes	Yes	No	No	Yes	No	4	0
Yes	Yes	Yes	No	No	Yes	No	2	2
Yes	Yes	Yes	No	No	Yes	No	0	0
No	No	Yes	No	Yes	Yes	No	0	0
No	No	No	No	No	Yes	No	1	0

For peer review only

No	No	No	No	Yes	Yes	Yes	0	0
No	No	No	No	No	Yes	Yes	0	0
No	No	Yes	No	No	Yes	Yes	4	0
No	No	No	No	No	No	Yes	0	0
Yes	No	Yes	No	No	Yes	No	0	0
Yes	No	Yes	No	No	No	Yes	0	0
No	No	Yes	No	No	Yes	No	9	0
No	Yes	Yes	No	No	Yes	No	0	2
No	No	Yes	No	No	Yes	No	0	0
Yes	Yes	Yes	No	No	Yes	No	4	0
Yes	Yes	Yes	No	No	Yes	Yes	0	0
No	No	Yes	No	No	Yes	No	2	0
Yes	Yes	Yes	No	No	Yes	Yes	5	0
Yes	Yes	Yes	No	No	Yes	No	0	0
Yes	Yes	Yes	No	No	Yes	No	7	0
Yes	Yes	Yes	No	No	Yes	No	15	0
Yes	No	Yes	No	No	Yes	Yes	0	0
No	No	Yes	No	No	Yes	Yes	0	0
No	No	No	No	No	Yes	No	0	0
Yes	Yes	Yes	No	No	Yes	Yes	0	0
Yes	Yes	Yes	No	No	Yes	Yes	0	0
No	No	Yes	No	Yes	Yes	No	0	0
Yes	Yes	Yes	No	No	Yes	Yes	0	0
No	No	Yes	No	No	Yes	Yes	0	0
No	No	Yes	No	No	No	No	0	0
Yes	Yes	Yes	Yes	No	No	No	0	0
Yes	Yes	No	No	No	Yes	Yes	0	0
No	No	No	No	No	No	No	0	0
Yes	Yes	No	No	No	No	Yes	0	0
Yes	Yes	Yes	No	No	Yes	No	0	0
No	No	No	No	No	Yes	No	0	0
Yes	Yes	Yes	No	No	Yes	Yes	0	0
Yes	Yes	Yes	No	No	Yes	Yes	0	0
Yes	Yes	Yes	No	No	Yes	Yes	0	0
Yes	Yes	Yes	No	No	Yes	Yes	0	0
No	Yes	No	No	Yes	No	No	0	0
No	No	No	No	No	Yes	Yes	0	0
No	No	No	No	No	Yes	No	5	0
No	No	No	No	No	Yes	No	0	0
Yes	Yes	Yes	Yes	Yes	Yes	Yes	0	0
Yes	Yes	Yes	No	No	Yes	Yes	0	0
Yes	Yes	Yes	Yes	No	No	Yes	0	0
No	No	No	No	No	Yes	No	2	0
No	No	Yes	No	No	Yes	No	0	0
No	No	Yes	No	No	Yes	No	2	7
No	No	No	No	No	No	No	0	0
Yes	Yes	Yes	No	No	Yes	No	20	0
Yes	Yes	Yes	No	No	Yes	No	4	0
Yes	Yes	Yes	No	No	Yes	Yes	0	0
Yes	Yes	Yes	No	No	Yes	No	20	0
No	No	No	No	No	Yes	No	2	0
Yes	Yes	Yes	No	No	No	No	0	0

For peer review only

Yes	Yes	Yes	No	No	Yes	No	0	0
Yes	Yes	Yes	No	No	Yes	No	10	2
No	No	No	No	No	Yes	No	7	0
No	No	No	No	No	Yes	Yes	0	0
No	No	No	No	No	Yes	No	2	9
Yes	Yes	Yes	No	No	Yes	No	4	0
No	No	No	No	Yes	No	No	0	0
Yes	Yes	Yes	No	No	Yes	No	0	0
Yes	Yes	Yes	No	No	Yes	No	0	0
No	No	Yes	No	No	Yes	No	0	0
No	No	Yes	No	No	No	No	0	0
No	No	No	No	No	Yes	No	2	0
No	No	Yes	No	Yes	Yes	No	0	2
Yes	Yes	Yes	No	No	Yes	No	0	0
No	No	Yes	No	No	Yes	Yes	0	0
No	No	No	No	Yes	Yes	No	0	0
No	Yes	Yes	No	No	Yes	No	8	0
No	No	No	No	No	Yes	No	0	0
Yes	Yes	Yes	No	No	Yes	Yes	0	0
No	Yes	Yes	No	No	Yes	No	0	0
No	No	No	No	Yes	No	No	0	0
No	No	Yes	No	No	Yes	No	0	0
Yes	Yes	Yes	Yes	No	No	No	0	0
No	No	Yes	No	No	No	No	0	0
No	Yes	Yes	No	No	Yes	No	0	0
Yes	Yes	Yes	No	No	Yes	Yes	4	0
No	No	No	No	Yes	Yes	No	4	0
Yes	No	Yes	No	No	Yes	Yes	4	0
No	No	Yes	No	No	Yes	No	8	0
Yes	Yes	Yes	No	No	Yes	Yes	0	0
No	No	No	No	No	Yes	Yes	0	0
No	Yes	Yes	No	No	Yes	Yes	5	0
Yes	Yes	Yes	No	No	Yes	Yes	0	0
No	No	No	No	No	Yes	No	0	0
No	No	Yes	No	No	No	Yes	0	0
No	No	Yes	No	No	Yes	No	2	0
No	No	Yes	No	No	Yes	No	0	0
No	No	No	No	Yes	Yes	No	0	2
No	No	Yes	No	No	Yes	Yes	4	0
No	No	No	No	Yes	No	No	0	0
No	No	Yes	No	No	Yes	No	3	0
No	Yes	Yes	No	No	Yes	No	0	0
Yes	Yes	Yes	No	No	No	No	0	0
Yes	Yes	Yes	No	Yes	Yes	Yes	0	0
No	No	No	No	No	Yes	No	2	3
No	No	Yes	No	No	Yes	Yes	0	0
Yes	Yes	Yes	No	No	Yes	Yes	0	0
Yes	Yes	Yes	No	No	Yes	Yes	0	0
Yes	Yes	Yes	No	No	Yes	No	0	0
Yes	Yes	Yes	No	No	Yes	No	1	0
No	No	No	No	No	No	Yes	0	0
No	No	No	No	No	Yes	Yes	0	0

For peer review only

Yes	Yes	Yes	No	No	Yes	Yes	0	0
Yes	No	Yes	No	No	Yes	Yes	0	0
No	No	No	No	No	Yes	No	10	0
No	No	No	No	No	Yes	Yes	0	0
No	No	Yes	No	No	Yes	No	3	0
No	No	Yes	No	No	Yes	No	4	0
Yes	Yes	Yes	No	No	Yes	No	20	0
No	No	Yes	No	No	Yes	No	1	0
Yes	Yes	Yes	No	No	Yes	No	4	0
Yes	Yes	Yes	No	No	Yes	Yes	0	0
Yes	Yes	Yes	No	No	No	Yes	0	0
No	No	No	No	No	Yes	No	0	0
Yes	Yes	Yes	No	No	Yes	No	5	0
No	No	No	No	No	Yes	No	7	0
No	No	No	No	No	Yes	No	0	0
No	No	No	No	No	No	No	0	0
Yes	Yes	Yes	No	No	Yes	No	0	0
No	No	No	No	No	No	No	0	0
Yes	Yes	Yes	Yes	Yes	Yes	No	0	0
No	No	No	No	No	Yes	No	0	0
Yes	Yes	Yes	No	No	Yes	Yes	0	0
No	Yes	Yes	No	No	Yes	No	5	0
Yes	No	Yes	No	No	Yes	No	0	0
Yes	Yes	Yes	No	No	Yes	Yes	0	0
Yes	Yes	Yes	No	No	Yes	No	0	4
No	Yes	Yes	No	No	Yes	Yes	4	0
No	No	No	No	No	Yes	No	1	11
Yes	Yes	Yes	No	No	Yes	Yes	0	0
No	No	Yes	No	No	No	No	0	0
Yes	Yes	Yes	No	No	Yes	No	20	0
No	No	No	No	No	Yes	No	1	0
No	No	Yes	No	No	Yes	No	6	0
No	No	Yes	No	No	No	No	0	0
No	No	No	No	No	Yes	No	4	0
No	No	Yes	No	No	Yes	No	2	0
No	No	No	No	No	Yes	No	0	0
No	No	Yes	No	No	Yes	No	5	0
No	No	Yes	No	No	Yes	No	4	0
No	No	No	No	No	Yes	No	10	0
No	No	No	No	No	Yes	No	2	0
No	No	Yes	No	No	No	No	0	0
No	No	No	No	No	Yes	No	1	0
No	No	No	No	No	Yes	No	0	0
No	No	Yes	No	No	Yes	No	3	0
No	No	Yes	No	No	Yes	No	4	0
No	No	No	No	No	Yes	No	2	0
No	No	Yes	No	No	Yes	No	2	0
No	No	No	No	No	Yes	No	0	0
No	No	Yes	No	No	Yes	Yes	5	0
No	No	Yes	No	No	Yes	Yes	6	0
No	No	Yes	No	No	Yes	No	0	0
No	No	Yes	No	No	Yes	No	5	0

For peer review only

No	No	No	No	No	Yes	No	3	0
No	No	Yes	No	No	Yes	No	2	0
No	No	Yes	No	No	Yes	No	10	0
No	No	No	No	Yes	Yes	No	3	0
No	No	Yes	No	No	Yes	No	0	0
No	No	Yes	No	No	Yes	No	5	0
No	No	Yes	No	No	Yes	No	0	0
No	No	Yes	No	No	Yes	No	3	0
No	No	Yes	No	No	Yes	No	3	0
No	No	No	No	No	Yes	No	0	0
No	No	Yes	No	No	Yes	No	5	0
No	No	Yes	No	No	Yes	No	0	0
No	No	Yes	No	No	Yes	No	0	0
No	No	Yes	Yes	No	Yes	No	5	0
No	No	Yes	No	No	Yes	No	5	0
No	No	No	No	No	Yes	No	0	0
No	No	Yes	No	No	Yes	No	7	0
No	No	Yes	No	No	Yes	No	0	0
No	No	Yes	No	No	Yes	No	1	0
No	No	No	No	No	Yes	No	4	0
No	No	Yes	No	No	No	No	0	0
No	No	Yes	No	No	Yes	No	5	0
No	No	No	No	No	Yes	No	3	0
No	No	Yes	No	No	Yes	No	5	0
No	No	Yes	No	No	Yes	No	0	0
No	No	Yes	No	No	Yes	No	0	0
No	No	No	No	No	Yes	No	6	0
No	No	Yes	No	No	Yes	No	5	0
No	No	No	No	No	Yes	No	0	0
No	No	Yes	No	No	Yes	No	0	0
No	No	Yes	No	No	Yes	No	2	4
No	No	Yes	No	No	Yes	No	0	0
No	No	No	No	No	Yes	No	5	0
No	No	Yes	No	No	Yes	No	10	0
No	No	No	No	No	Yes	No	3	0
No	No	No	No	Yes	Yes	No	0	0
No	No	No	No	No	Yes	No	6	0
No	No	Yes	No	No	Yes	No	10	0
No	No	Yes	No	No	Yes	No	2	0
No	No	No	No	No	Yes	No	0	0
No	No	No	No	No	Yes	No	9	0
No	No	No	No	No	Yes	No	3	0
No	No	Yes	No	No	Yes	No	2	0
No	No	No	No	No	Yes	No	0	0
No	No	No	No	No	Yes	No	0	0
No	No	Yes	Yes	No	Yes	No	2	0
Yes	No	Yes	No	Yes	Yes	No	10	0
No	No	No	No	No	Yes	No	0	0
No	No	No	No	No	Yes	No	3	0
No	No	Yes	No	No	Yes	No	6	0
No	No	Yes	No	No	Yes	No	1	0
Yes	No	Yes	No	No	Yes	No	2	0

For peer review only

No	No	No	No	No	Yes	No	0	0
No	No	Yes	No	No	Yes	No	0	0
Yes	Yes	Yes	No	No	Yes	No	8	12
No	No	No	No	No	Yes	No	3	0
No	No	Yes	No	No	Yes	No	2	0
No	No	Yes	No	No	Yes	No	6	0
No	No	No	No	No	No	No	0	0
No	No	No	No	No	Yes	No	0	0
Yes	Yes	Yes	Yes	Yes	Yes	No	0	0
Yes	Yes	Yes	No	No	No	No	0	0
Yes	Yes	Yes	No	No	Yes	No	0	0
No	No	No	No	Yes	Yes	Yes	0	0
No	No	Yes	No	No	Yes	No	5	0
No	No	No	No	No	No	No	0	0
No	No	No	No	No	Yes	No	0	0
Yes	Yes	Yes	No	No	Yes	No	6	0
No	No	No	No	Yes	Yes	Yes	0	0
No	No	Yes	No	No	No	Yes	0	0
No	No	No	No	Yes	Yes	Yes	0	0
No	No	No	No	No	Yes	No	0	0
Yes	Yes	Yes	No	No	Yes	No	3	0
Yes	Yes	Yes	No	No	No	No	0	0
No	Yes	Yes	No	No	Yes	No	0	0
No	No	Yes	No	No	No	Yes	0	0
Yes	Yes	Yes	No	No	Yes	No	3	0
No	No	Yes	No	No	Yes	No	0	0
No	No	No	No	Yes	Yes	Yes	0	0
Yes	Yes	Yes	No	No	No	Yes	0	0
No	No	No	No	No	No	No	0	0
No	No	No	No	Yes	Yes	No	0	0
Yes	Yes	Yes	No	No	Yes	No	0	0
No	No	Yes	No	No	Yes	No	3	0
No	No	No	No	No	No	No	0	0
No	No	Yes	No	No	Yes	No	2	0
No	No	Yes	No	No	Yes	No	0	0
No	No	No	No	No	Yes	No	1	0
No	No	No	No	No	Yes	No	3	0
Yes	Yes	Yes	No	No	Yes	No	0	0
No	Yes	Yes	No	No	No	Yes	0	0
No	No	Yes	No	No	Yes	Yes	0	0
No	No	Yes	No	No	Yes	No	8	0
No	No	No	No	Yes	Yes	Yes	0	0
Yes	No	Yes	No	No	No	No	0	0
Yes	Yes	Yes	No	No	Yes	No	3	0
No	No	Yes	No	No	Yes	No	0	0
No	No	Yes	No	No	Yes	Yes	0	0
Yes	Yes	Yes	No	No	Yes	No	3	0
No	No	No	No	Yes	Yes	Yes	0	0
No	No	Yes	No	No	Yes	Yes	0	0
No	Yes	Yes	No	No	Yes	No	0	0
No	Yes	Yes	No	No	Yes	No	0	0
No	No	No	No	No	Yes	Yes	0	0

No	No	No	No	No	Yes	No	0	0
Yes	Yes	Yes	No	No	Yes	No	8	0
No	Yes	Yes	No	No	Yes	No	8	0
No	No	No	No	No	No	No	0	0
Yes	Yes	Yes	No	No	Yes	No	0	0
No	Yes	Yes	No	No	Yes	No	6	0
No	No	Yes	No	No	Yes	No	0	0
No	No	Yes	No	No	Yes	No	1	0
Yes	Yes	Yes	No	No	Yes	Yes	0	0
Yes	Yes	Yes	No	No	Yes	No	0	0
Yes	No	Yes	No	No	Yes	No	0	0
Yes	Yes	Yes	No	No	Yes	Yes	0	0
No	No	No	No	No	No	No	0	0
No	No	Yes	No	No	No	No	0	0
No	No	Yes	No	No	No	No	0	0
No	No	Yes	No	No	No	Yes	0	0
No	No	Yes	No	No	Yes	No	2	0
Yes	Yes	Yes	No	No	Yes	No	6	0
Yes	Yes	Yes	No	Yes	No	No	0	0
Yes	Yes	Yes	No	No	Yes	Yes	31	0
Yes	Yes	Yes	No	Yes	No	No	0	0
Yes	Yes	Yes	No	No	Yes	Yes	4	4
Yes	Yes	Yes	No	No	Yes	No	0	4
Yes	Yes	Yes	No	Yes	No	No	0	0
Yes	Yes	Yes	No	Yes	Yes	Yes	4	0
Yes	Yes	Yes	No	No	Yes	No	2	2
Yes	Yes	Yes	No	No	Yes	Yes	0	0
Yes	Yes	Yes	No	No	Yes	Yes	0	0
Yes	Yes	Yes	Yes	No	No	No	0	0
Yes	Yes	Yes	No	No	Yes	Yes	0	0
Yes	Yes	Yes	No	Yes	Yes	No	0	4
Yes	Yes	Yes	No	Yes	Yes	Yes	0	0
Yes	Yes	Yes	No	No	Yes	No	0	0
Yes	Yes	Yes	No	Yes	Yes	No	0	0
Yes	Yes	Yes	No	Yes	Yes	No	2	0
Yes	Yes	Yes	No	Yes	Yes	No	3	9
Yes	Yes	Yes	No	No	Yes	Yes	6	4
Yes	Yes	Yes	No	Yes	Yes	No	0	0
Yes	Yes	Yes	No	Yes	Yes	No	0	0
Yes	Yes	Yes	No	Yes	Yes	No	0	0
Yes	Yes	Yes	No	No	Yes	No	2	4
Yes	Yes	Yes	No	Yes	Yes	No	0	0
Yes	Yes	Yes	No	Yes	Yes	No	10	4
Yes	Yes	Yes	No	No	Yes	No	0	5
Yes	Yes	Yes	No	No	Yes	Yes	0	0
Yes	Yes	Yes	No	No	Yes	Yes	4	0
Yes	Yes	Yes	No	No	Yes	Yes	10	10
Yes	Yes	Yes	No	No	Yes	Yes	5	2
Yes	Yes	Yes	No	No	Yes	Yes	0	0
Yes	Yes	Yes	No	No	Yes	No	7	0
Yes	Yes	Yes	No	No	No	Yes	0	0
Yes	Yes	Yes	No	No	Yes	No	5	3

For peer review only

Yes	Yes	Yes	No	Yes	No	Yes	0	0
No	No	Yes	No	No	No	No	0	0
Yes	Yes	Yes	No	No	Yes	Yes	2	3
Yes	Yes	Yes	No	Yes	Yes	No	0	0
Yes	Yes	Yes	No	Yes	No	Yes	0	0
Yes	Yes	Yes	No	No	Yes	Yes	2	0
Yes	Yes	Yes	No	Yes	Yes	No	4	0
Yes	Yes	Yes	No	No	Yes	No	0	0
Yes	Yes	Yes	No	No	Yes	Yes	5	3
Yes	Yes	Yes	No	No	Yes	Yes	10	0
Yes	Yes	Yes	No	No	Yes	No	3	0
Yes	Yes	Yes	No	Yes	Yes	Yes	9	0
Yes	Yes	Yes	No	Yes	Yes	Yes	0	0
Yes	Yes	Yes	No	No	No	No	0	0
Yes	Yes	Yes	No	No	Yes	Yes	2	0
Yes	Yes	No	No	Yes	No	No	0	0
Yes	Yes	Yes	No	No	No	No	0	0
Yes	Yes	Yes	No	No	Yes	Yes	0	3
Yes	Yes	Yes	No	No	Yes	Yes	3	0
Yes	Yes	Yes	No	Yes	Yes	No	1	0
Yes	Yes	Yes	No	Yes	No	No	0	0
Yes	Yes	Yes	No	No	Yes	Yes	10	0
Yes	Yes	Yes	No	No	No	Yes	0	0
Yes	Yes	Yes	No	No	Yes	Yes	6	0
Yes	Yes	Yes	No	No	No	Yes	0	0
No	No	Yes	No	Yes	No	No	0	0
Yes	Yes	Yes	No	No	Yes	Yes	2	0
No	No	No	No	No	No	No	0	0
Yes	Yes	Yes	No	No	No	Yes	0	0
No	No	Yes	No	No	No	No	0	0
No	No	No	No	Yes	No	No	0	0
Yes	Yes	Yes	No	No	Yes	Yes	1	0
Yes	No	Yes	No	No	Yes	No	13	0
Yes	Yes	Yes	No	No	Yes	Yes	4	0
Yes	Yes	No	No	No	Yes	No	15	0
Yes	Yes	Yes	No	No	Yes	No	5	0
Yes	Yes	Yes	No	No	Yes	Yes	0	0
No	No	Yes	No	No	Yes	No	10	0
Yes	Yes	Yes	No	No	Yes	No	12	0
Yes	Yes	Yes	No	Yes	No	Yes	0	0
No	No	Yes	No	No	No	Yes	0	0
Yes	Yes	Yes	No	No	Yes	No	5	0
Yes	Yes	Yes	No	No	Yes	Yes	5	0
No	Yes	Yes	No	No	Yes	No	0	0
Yes	Yes	Yes	No	No	Yes	Yes	3	0
Yes	Yes	Yes	No	No	Yes	Yes	20	3
Yes	Yes	Yes	No	No	No	Yes	0	0
No	No	Yes	No	No	No	Yes	0	0
Yes	Yes	Yes	No	No	Yes	Yes	3	0
No	No	Yes	No	No	Yes	No	0	0
Yes	Yes	Yes	No	No	Yes	Yes	2	0
Yes	Yes	Yes	No	No	Yes	Yes	3	0

For peer review only

No	Yes	Yes	No	No	No	No	0	0
Yes	Yes	Yes	No	No	No	Yes	0	0
Yes	Yes	Yes	No	No	No	Yes	0	0
Yes	Yes	Yes	No	No	No	Yes	0	0
Yes	Yes	Yes	No	No	Yes	Yes	5	0
Yes	Yes	Yes	No	No	Yes	No	6	0
No	Yes	No	No	No	Yes	No	12	0
Yes	Yes	Yes	No	No	No	No	0	0
Yes	Yes	Yes	No	No	No	Yes	0	0
Yes	Yes	Yes	No	No	No	No	0	0
No	No	No	No	Yes	Yes	No	1	0
No	No	No	No	Yes	No	No	0	0
No	No	Yes	No	No	Yes	Yes	0	0
Yes	Yes	Yes	No	No	Yes	Yes	5	0
Yes	Yes	Yes	No	No	No	No	0	0
Yes	Yes	Yes	No	No	No	Yes	0	0
No	No	Yes	No	Yes	No	No	0	0
No	No	No	No	No	No	Yes	0	0
No	No	Yes	No	No	Yes	No	6	0
No	Yes	Yes	No	No	Yes	No	5	0
Yes	Yes	Yes	No	No	Yes	No	0	0
Yes	Yes	Yes	No	No	Yes	No	0	0
Yes	Yes	Yes	No	No	No	Yes	0	0
Yes	Yes	Yes	No	No	Yes	Yes	0	0
No	Yes	Yes	No	No	Yes	Yes	3	8
Yes	Yes	Yes	No	Yes	Yes	Yes	0	0
Yes	Yes	Yes	No	No	Yes	Yes	10	3
Yes	Yes	Yes	No	No	Yes	Yes	4	0
No	No	No	No	No	Yes	No	5	0
No	No	No	No	Yes	Yes	No	2	0
Yes	Yes	Yes	No	Yes	Yes	Yes	7	3
Yes	Yes	Yes	No	No	Yes	Yes	1	0
No	No	No	No	No	No	No	0	0
Yes	Yes	Yes	No	No	Yes	Yes	0	0
Yes	Yes	Yes	No	No	Yes	Yes	3	9
Yes	Yes	Yes	No	No	Yes	No	7	0
No	Yes	Yes	No	No	Yes	No	0	0
No	Yes	Yes	No	No	No	Yes	0	0
Yes	Yes	Yes	No	No	No	Yes	0	0
No	No	No	No	No	No	No	0	0
No	No	Yes	No	No	No	No	0	0
No	No	No	No	No	Yes	No	5	0
No	No	No	No	No	No	Yes	0	0
Yes	Yes	Yes	No	No	Yes	Yes	10	1
No	No	No	No	Yes	No	No	0	0
Yes	Yes	Yes	No	No	Yes	No	2	0
Yes	Yes	Yes	No	No	No	Yes	0	0
No	Yes	Yes	No	No	Yes	No	5	0
No	No	Yes	No	No	Yes	No	6	0
No	Yes	Yes	No	No	No	Yes	0	0
Yes	No	Yes	No	No	Yes	No	3	0
No	Yes	Yes	No	No	Yes	No	12	0

For peer review only

No	Yes	Yes	No	No	Yes	Yes	12	0
No	No	No	No	No	No	No	0	0
No	No	No	No	No	Yes	Yes	0	0
No	No	Yes	No	No	Yes	Yes	10	0
No	No	Yes	No	No	Yes	No	0	0
Yes	Yes	Yes	No	No	Yes	No	7	9
Yes	Yes	Yes	No	No	Yes	Yes	3	0
No	No	No	No	No	Yes	Yes	0	0
No	No	Yes	No	No	Yes	Yes	2	0
Yes	Yes	Yes	No	Yes	Yes	No	7	0
No	No	No	No	No	Yes	Yes	0	0
Yes	Yes	Yes	No	No	Yes	No	8	0
Yes	Yes	Yes	No	No	Yes	Yes	4	5
No	Yes	Yes	Yes	No	No	Yes	0	0
No	Yes	Yes	No	No	Yes	No	5	0
No	No	Yes	No	No	No	No	0	0
Yes	Yes	Yes	No	No	Yes	Yes	2	0
Yes	Yes	Yes	No	No	Yes	Yes	0	0
Yes	Yes	Yes	No	No	Yes	Yes	6	5
Yes	Yes	Yes	No	No	Yes	No	0	0
Yes	Yes	Yes	No	No	Yes	Yes	6	0
Yes	No	Yes	No	No	Yes	No	10	0
Yes	Yes	Yes	No	No	Yes	Yes	4	0
No	No	No	No	Yes	No	No	0	0
Yes	Yes	Yes	No	No	Yes	Yes	0	0
Yes	Yes	No	No	No	No	Yes	0	0
No	No	Yes	No	No	Yes	No	2	1
Yes	Yes	Yes	No	No	Yes	No	0	0
Yes	Yes	Yes	No	No	Yes	Yes	0	0
Yes	Yes	Yes	No	No	No	Yes	0	0
No	No	No	No	No	Yes	No	0	0
No	No	Yes	No	No	Yes	No	0	0
No	No	Yes	No	No	Yes	Yes	4	0
No	No	No	No	No	Yes	Yes	0	0
No	No	Yes	No	No	Yes	No	0	0
No	No	No	No	No	Yes	No	0	0
Yes	No	Yes	No	No	Yes	Yes	0	0
No	No	Yes	No	No	Yes	No	0	0
No	No	Yes	No	No	Yes	Yes	0	7
No	No	Yes	No	No	Yes	Yes	0	0
No	No	Yes	No	No	Yes	No	10	0
No	No	Yes	No	No	Yes	No	6	0
No	No	Yes	No	No	Yes	No	0	0
No	No	Yes	No	No	Yes	No	4	0
No	No	Yes	No	No	Yes	No	7	0
No	No	Yes	No	No	Yes	Yes	0	0
No	Yes	Yes	No	No	No	No	0	0
No	No	No	No	No	Yes	Yes	4	0
No	No	Yes	No	No	Yes	Yes	0	0
No	No	Yes	No	Yes	Yes	No	1	0
No	No	Yes	No	No	Yes	Yes	5	0
No	No	Yes	No	No	Yes	Yes	1	0

No	Yes	Yes	No	Yes	Yes	Yes	3	0
No	No	Yes	No	No	Yes	No	10	0
Yes	Yes	Yes	No	No	Yes	No	0	0
No	No	Yes	No	No	Yes	Yes	0	0
Yes	Yes	Yes	No	No	Yes	Yes	0	0
No	No	Yes	No	No	Yes	Yes	6	0
No	No	Yes	No	No	Yes	Yes	5	0
No	No	Yes	No	No	Yes	No	5	0
No	No	No	No	No	Yes	No	0	0
No	No	Yes	No	No	Yes	No	7	0
No	No	No	No	Yes	Yes	Yes	0	0
No	Yes	Yes	No	No	Yes	No	5	0
No	No	Yes	No	No	Yes	Yes	6	0
No	No	No	No	No	Yes	No	0	0
No	No	Yes	No	No	Yes	No	10	0
No	No	Yes	No	No	Yes	No	3	0
No	No	Yes	No	No	Yes	Yes	4	0
Yes	Yes	Yes	No	No	Yes	No	0	0
Yes	Yes	Yes	No	No	Yes	No	0	0
Yes	No	Yes	No	No	Yes	Yes	5	0
Yes	No	Yes	No	No	Yes	No	0	0
No	No	No	No	No	Yes	Yes	6	0
No	No	No	No	No	Yes	No	0	0
No	No	Yes	No	No	Yes	No	10	0
Yes	No	Yes	No	No	Yes	No	0	0
No	No	Yes	No	No	Yes	No	0	0
No	No	Yes	No	No	Yes	No	3	0
Yes	Yes	Yes	No	Yes	Yes	Yes	0	0
No	No	Yes	No	No	Yes	Yes	0	0
No	No	Yes	No	No	No	No	0	0
No	No	No	No	No	Yes	No	3	0
No	No	No	No	No	Yes	No	3	0
No	No	No	No	No	Yes	No	1	0
No	No	Yes	Yes	Yes	Yes	Yes	3	0
No	No	No	No	No	Yes	Yes	0	0
No	No	Yes	No	No	Yes	No	2	0
No	No	Yes	No	No	Yes	No	3	0
No	No	Yes	No	Yes	Yes	Yes	3	0
No	No	Yes	No	No	Yes	Yes	0	0
No	No	Yes	No	No	Yes	No	7	0
No	No	Yes	No	No	Yes	No	0	0
No	No	No	No	No	Yes	No	0	0
No	No	Yes	No	No	Yes	No	0	0
No	No	Yes	No	No	Yes	Yes	3	0
No	No	No	No	Yes	Yes	Yes	0	0
No	No	Yes	No	No	Yes	No	2	0
No	No	Yes	No	No	Yes	No	2	3
No	No	Yes	No	No	Yes	No	5	0
No	No	Yes	No	No	Yes	Yes	0	0
Yes	Yes	Yes	No	No	No	No	0	0
No	No	Yes	No	No	Yes	Yes	0	0
No	No	Yes	No	No	Yes	Yes	2	0

For peer review only

Yes	Yes	No	No	No	Yes	No	3	0
No	No	Yes	No	No	Yes	No	0	0
Yes	No	No	No	Yes	Yes	No	0	0
Yes	No	Yes	No	Yes	Yes	Yes	0	0
No	No	Yes	No	No	Yes	No	10	0
No	No	No	No	Yes	No	No	0	0
No	No	Yes	No	No	Yes	Yes	2	0
No	No	Yes	No	No	Yes	No	4	0
No	No	Yes	No	No	Yes	Yes	0	0
Yes	No	Yes	No	No	Yes	No	10	0
No	No	Yes	No	No	Yes	Yes	0	0
No	No	Yes	No	No	Yes	No	0	0
Yes	Yes	Yes	No	No	Yes	Yes	0	0
No	No	Yes	No	No	Yes	No	2	0
No	No	Yes	No	No	Yes	No	5	0
No	No	Yes	No	No	Yes	No	0	0
Yes	Yes	Yes	No	Yes	Yes	No	0	0
No	No	Yes	No	No	Yes	No	5	0
No	No	Yes	No	No	Yes	No	3	0
No	No	No	No	No	No	No	0	0
No	No	Yes	No	No	Yes	No	3	0
No	No	Yes	No	No	Yes	Yes	0	0
Yes	No	Yes	No	No	Yes	No	7	0
No	No	Yes	No	No	Yes	Yes	0	0
No	No	Yes	No	No	Yes	No	0	0
Yes	Yes	Yes	No	No	Yes	Yes	3	0
No	No	Yes	No	No	Yes	No	0	0
No	No	Yes	No	No	Yes	Yes	0	0
No	No	Yes	No	No	Yes	No	6	0
Yes	Yes	Yes	No	No	Yes	No	0	0
No	No	No	No	No	No	No	0	0
No	No	Yes	No	No	Yes	No	4	0
Yes	No	Yes	No	No	Yes	Yes	0	0
No	No	Yes	Yes	No	No	No	0	0
No	No	Yes	No	No	Yes	No	0	0
No	No	Yes	No	No	Yes	No	9	0
No	No	Yes	No	No	Yes	Yes	10	0
No	No	Yes	No	No	Yes	No	0	0
No	Yes	Yes	No	No	Yes	No	4	0
Yes	Yes	Yes	No	No	No	Yes	0	0
No	No	Yes	No	No	Yes	Yes	3	0
No	No	Yes	No	No	No	No	0	0
No	No	Yes	No	No	Yes	Yes	0	0
Yes	Yes	Yes	No	Yes	No	No	0	0
Yes	Yes	Yes	No	No	Yes	No	0	0
Yes	Yes	Yes	No	Yes	No	Yes	0	0
Yes	Yes	Yes	Yes	No	Yes	No	0	0
Yes	Yes	Yes	No	No	No	No	0	0
Yes	Yes	Yes	No	No	Yes	No	0	4
Yes	Yes	Yes	No	No	Yes	No	3	0
No	No	No	No	No	No	No	0	0
No	No	Yes	No	No	No	No	0	0

For peer review only

Yes	Yes	Yes	No	No	No	No	0	0
No	No	Yes	No	No	No	No	0	0
Yes	Yes	Yes	No	No	No	No	0	0
Yes	Yes	Yes	No	No	Yes	Yes	5	0
No	Yes	Yes	No	No	Yes	Yes	5	4
Yes	Yes	Yes	No	No	No	No	0	0
Yes	Yes	Yes	No	No	No	Yes	0	0
Yes	Yes	Yes	No	No	No	No	0	0
Yes	Yes	Yes	No	Yes	Yes	No	0	0
Yes	Yes	Yes	No	No	No	No	0	0
Yes	Yes	Yes	No	No	No	No	0	0
Yes	No	Yes	No	No	No	No	0	0
Yes	Yes	Yes	No	Yes	No	Yes	0	0
No	No	No	No	No	No	No	0	0
Yes	Yes	Yes	No	No	Yes	No	0	0
Yes	Yes	Yes	No	No	No	No	0	0
Yes	Yes	Yes	No	Yes	No	No	0	0
No	No	No	No	No	No	No	0	0
Yes	Yes	Yes	No	Yes	No	No	0	0
Yes	Yes	Yes	No	Yes	No	No	0	0
Yes	Yes	Yes	No	No	No	No	0	0
Yes	Yes	Yes	No	Yes	No	No	0	0
Yes	Yes	Yes	No	Yes	No	No	0	0
Yes	Yes	Yes	No	Yes	Yes	No	0	0
Yes	Yes	Yes	No	No	No	No	0	0
Yes	No	Yes	No	No	No	No	0	0
Yes	Yes	Yes	No	No	No	No	0	0
No	No	Yes	No	No	Yes	Yes	6	0
Yes	Yes	Yes	No	No	Yes	No	0	0
Yes	Yes	Yes	No	No	Yes	Yes	0	0
Yes	Yes	No	No	No	No	No	0	0
Yes	Yes	Yes	No	No	No	No	0	0
Yes	Yes	Yes	No	Yes	No	No	0	0
Yes	Yes	Yes	No	Yes	No	No	0	0
Yes	Yes	Yes	No	Yes	No	No	0	0
Yes	Yes	Yes	No	Yes	No	No	0	0
No	No	Yes	No	No	No	No	0	0
Yes	Yes	Yes	No	No	Yes	Yes	0	0
Yes	Yes	Yes	No	Yes	No	Yes	0	0
Yes	Yes	Yes	No	Yes	No	No	0	0
Yes	Yes	Yes	No	Yes	No	No	0	0
Yes	Yes	Yes	No	Yes	No	No	0	0
Yes	Yes	Yes	No	No	No	No	0	0
Yes	Yes	Yes	No	Yes	Yes	No	0	0
Yes	Yes	Yes	No	No	Yes	No	0	0
Yes	No	Yes	No	Yes	No	No	0	0
Yes	Yes	Yes	No	Yes	No	No	0	0
Yes	Yes	Yes	No	Yes	No	No	0	0
Yes	Yes	Yes	No	Yes	No	No	0	0
Yes	Yes	Yes	No	No	Yes	No	2	3
No	No	Yes	No	No	Yes	No	0	5
Yes	Yes	Yes	No	Yes	No	Yes	0	0

For peer review only

Yes	Yes	Yes	No	No	No	No	0	0
Yes	Yes	Yes	No	Yes	No	No	0	0
No	No	Yes	No	No	No	Yes	0	0
Yes	Yes	Yes	No	Yes	No	No	0	0
Yes	Yes	Yes	No	No	No	Yes	0	0
Yes	Yes	Yes	No	Yes	No	Yes	0	0
Yes	Yes	Yes	No	Yes	No	No	0	0
Yes	Yes	Yes	No	No	No	Yes	0	0
Yes	No	Yes	No	No	No	Yes	0	0
Yes	Yes	Yes	No	No	No	Yes	0	0
Yes	Yes	Yes	No	No	No	Yes	0	0
Yes	Yes	Yes	No	No	No	Yes	0	0
Yes	Yes	Yes	No	No	No	No	0	0
Yes	Yes	Yes	No	Yes	No	No	0	0
Yes	Yes	Yes	No	No	Yes	No	3	0
Yes	Yes	Yes	No	Yes	Yes	Yes	0	5
Yes	Yes	Yes	No	No	No	No	0	0
Yes	No	Yes	No	No	No	Yes	0	0
Yes	Yes	Yes	No	No	Yes	No	0	0
Yes	No	Yes	No	No	Yes	Yes	0	0
Yes	Yes	Yes	No	No	Yes	No	0	0
Yes	Yes	Yes	No	No	Yes	Yes	5	2
No	Yes	Yes	No	No	No	Yes	0	0
Yes	Yes	Yes	No	No	No	No	0	0
No	Yes	Yes	No	No	Yes	Yes	0	0
No	No	Yes	No	No	Yes	No	5	0
Yes	Yes	Yes	No	Yes	No	No	0	0
No	No	Yes	No	No	Yes	No	0	0
No	Yes	Yes	No	No	Yes	No	1	0
No	No	No	No	No	Yes	No	1	0
No	No	Yes	No	No	Yes	No	0	0
Yes	Yes	Yes	No	No	Yes	Yes	0	0
Yes	Yes	Yes	No	No	Yes	No	1	0
No	No	No	No	No	Yes	Yes	5	0
No	No	Yes	No	No	Yes	No	0	0
No	No	Yes	Yes	No	Yes	No	5	0
Yes	No	Yes	No	No	Yes	Yes	5	0
No	No	Yes	No	No	Yes	No	0	9
No	No	Yes	No	No	Yes	No	8	0
No	No	Yes	No	No	Yes	No	0	0
Yes	Yes	Yes	Yes	No	Yes	Yes	4	0
Yes	Yes	Yes	No	No	Yes	No	4	0
No	No	Yes	No	No	Yes	No	0	0
No	No	No	No	No	Yes	Yes	0	0
No	No	Yes	No	No	Yes	No	0	0
Yes	Yes	Yes	No	No	No	No	0	0
Yes	Yes	Yes	No	Yes	Yes	No	25	0
No	No	Yes	No	Yes	Yes	No	0	0
No	No	No	No	No	Yes	Yes	18	0
Yes	Yes	Yes	No	No	No	No	0	0
No	No	No	No	No	Yes	No	10	0
No	No	No	No	No	Yes	No	0	0

For peer review only

No	No	No	No	No	Yes	No	3	0
No	No	Yes	No	No	Yes	No	0	0
No	No	Yes	No	No	Yes	No	0	0
No	No	Yes	No	No	Yes	Yes	5	4
No	No	No	No	No	Yes	No	0	7
Yes	No	No	No	No	Yes	Yes	0	0
No	No	Yes	No	No	Yes	Yes	10	3
No	No	Yes	No	No	Yes	No	5	0
No	Yes	Yes	No	No	Yes	No	10	0
No	No	No	No	No	Yes	No	0	0
No	No	Yes	No	No	Yes	Yes	4	0
No	No	Yes	No	No	Yes	No	4	0
No	No	Yes	No	No	Yes	No	0	0
No	No	Yes	No	No	Yes	No	0	0
No	No	Yes	No	No	Yes	No	0	0
No	No	No	No	No	Yes	Yes	0	0
Yes	Yes	Yes	No	No	No	No	0	0
No	No	Yes	No	No	Yes	No	0	0
No	No	Yes	No	No	Yes	Yes	15	0
No	No	No	No	No	Yes	Yes	4	0
No	No	Yes	No	No	Yes	Yes	0	0
No	No	Yes	No	No	Yes	No	0	0
No	No	Yes	No	No	Yes	Yes	0	0
No	No	Yes	No	No	Yes	No	0	0
Yes	Yes	Yes	No	No	Yes	Yes	2	9
No	No	Yes	No	No	Yes	No	0	0
No	No	Yes	No	No	Yes	Yes	0	0
No	No	Yes	No	No	Yes	No	0	0
No	No	Yes	No	No	Yes	Yes	0	0
No	No	Yes	No	No	Yes	No	0	0
No	No	Yes	No	No	Yes	Yes	0	0
Yes	Yes	Yes	No	No	Yes	Yes	6	0
No	No	Yes	No	No	Yes	Yes	0	0
No	Yes	Yes	No	No	Yes	Yes	3	0
No	No	Yes	No	No	Yes	Yes	0	0
Yes	Yes	Yes	No	No	Yes	Yes	2	2
No	No	Yes	No	No	Yes	No	3	10
No	Yes	Yes	No	No	Yes	No	0	0
No	No	Yes	No	No	Yes	No	0	0
No	No	No	No	No	No	No	0	0
No	No	Yes	No	No	Yes	Yes	0	0
Yes	Yes	No	No	No	Yes	No	0	0
No	No	Yes	No	No	Yes	Yes	0	0
No	No	No	No	No	Yes	No	4	0
No	No	Yes	No	No	Yes	No	0	0
No	No	Yes	No	No	Yes	No	0	3
No	Yes	Yes	No	No	No	No	0	0
No	Yes	Yes	No	Yes	No	No	0	0
No	No	Yes	No	No	Yes	No	0	0
No	No	Yes	No	No	Yes	No	2	0
No	No	Yes	No	No	Yes	No	0	0
No	Yes	Yes	No	No	Yes	No	3	0

For peer review only

Yes	Yes	Yes	No	No	No	No	0	0
No	No	No	No	Yes	No	No	0	0
No	No	Yes	No	No	Yes	No	0	0
No	No	Yes	No	No	Yes	No	0	0
No	No	Yes	No	No	Yes	No	5	0
No	No	Yes	No	No	Yes	Yes	6	0
No	No	Yes	No	No	Yes	Yes	0	0
No	No	No	No	No	Yes	No	0	0
No	No	No	No	No	Yes	Yes	0	20
No	No	No	No	No	Yes	Yes	5	0
Yes	Yes	Yes	No	No	Yes	No	0	0
No	No	Yes	No	No	Yes	No	5	0
Yes	Yes	Yes	No	No	Yes	Yes	20	6
No	No	Yes	No	No	Yes	No	0	0
No	No	No	No	No	Yes	Yes	0	0
No	No	Yes	No	No	Yes	Yes	0	0
No	No	Yes	No	No	Yes	No	1	0
No	No	No	No	No	Yes	Yes	0	0
No	No	Yes	No	No	Yes	No	0	0
No	No	Yes	No	No	Yes	No	0	0
No	No	Yes	No	No	Yes	No	3	0
Yes	Yes	No	No	No	Yes	No	5	0
No	No	Yes	No	No	Yes	Yes	0	0
No	No	No	No	No	Yes	No	2	0
No	No	Yes	No	No	Yes	Yes	6	0
No	No	Yes	No	No	No	No	0	0
No	No	Yes	No	No	Yes	Yes	10	0
No	No	Yes	No	No	Yes	No	15	0
No	No	Yes	No	No	Yes	No	0	2
Yes	Yes	No	No	No	Yes	No	5	0
Yes	Yes	Yes	No	No	Yes	No	2	0
No	No	No	No	No	Yes	No	7	0
No	No	Yes	No	No	Yes	Yes	2	0
No	No	No	No	No	Yes	Yes	10	0
Yes	Yes	Yes	No	No	Yes	No	10	0
No	No	Yes	No	No	Yes	No	6	0
Yes	Yes	Yes	No	No	Yes	Yes	0	0
No	No	No	No	No	Yes	Yes	1	0
Yes	Yes	Yes	No	No	Yes	No	0	0
No	No	Yes	No	No	Yes	No	0	0
Yes	Yes	Yes	No	No	Yes	Yes	0	0
No	No	Yes	No	No	Yes	Yes	4	0
No	No	Yes	No	No	Yes	No	0	0
Yes	Yes	Yes	No	Yes	Yes	No	0	0
No	No	Yes	No	No	No	Yes	0	0
No	No	Yes	No	No	Yes	No	0	3
No	Yes	Yes	No	No	Yes	No	30	2
No	No	Yes	No	No	Yes	Yes	4	0
No	Yes	Yes	No	No	Yes	No	1	0
Yes	Yes	No	No	Yes	Yes	No	0	0
No	No	Yes	Yes	No	Yes	No	5	0
Yes	Yes	Yes	No	No	Yes	No	0	0

For peer review only

No	No	No	No	No	Yes	Yes	0	0
No	No	Yes	No	No	Yes	No	0	0
No	No	Yes	No	No	Yes	Yes	3	0
No	No	Yes	No	No	Yes	No	15	1
Yes	No	Yes	No	No	Yes	No	5	6
Yes	Yes	Yes	No	No	Yes	No	0	0
No	Yes	Yes	No	No	Yes	No	13	0
Yes	Yes	Yes	No	No	No	No	0	0
Yes	Yes	Yes	No	No	Yes	No	10	0
Yes	Yes	Yes	No	No	Yes	No	0	0
No	No	Yes	No	No	Yes	No	10	0
No	No	No	No	No	Yes	No	0	3
No	No	Yes	No	No	Yes	No	15	4
No	No	No	No	No	Yes	No	3	0
Yes	Yes	Yes	No	No	No	No	0	0
No	No	Yes	No	No	Yes	No	3	0
No	No	Yes	No	No	Yes	No	0	0
No	No	Yes	No	No	Yes	No	15	0
No	No	Yes	No	No	Yes	No	2	0
Yes	Yes	Yes	No	No	Yes	No	5	0
No	No	No	No	No	Yes	Yes	0	0
No	No	Yes	No	No	Yes	Yes	5	0
No	No	Yes	No	No	Yes	No	0	0
No	No	Yes	No	No	Yes	No	4	0
No	No	Yes	No	No	Yes	No	3	0
No	No	No	No	No	Yes	No	4	0
Yes	Yes	Yes	No	No	Yes	Yes	15	0
No	No	No	No	No	Yes	No	0	0
No	No	Yes	No	No	Yes	No	4	2
No	No	No	No	No	Yes	No	3	0
No	No	Yes	No	No	Yes	No	0	0
Yes	Yes	Yes	No	No	Yes	Yes	0	0
No	No	Yes	No	No	Yes	Yes	2	0
No	No	Yes	No	No	Yes	No	20	0
No	No	Yes	No	No	Yes	Yes	4	0
No	Yes	Yes	No	No	Yes	No	4	0
No	No	Yes	No	No	No	Yes	0	0
Yes	Yes	Yes	No	No	Yes	Yes	0	0
Yes	Yes	Yes	No	No	No	No	0	0
Yes	Yes	Yes	No	No	Yes	No	0	0
No	No	Yes	No	No	Yes	No	0	0
No	No	Yes	No	No	No	No	0	0
Yes	Yes	Yes	No	No	Yes	Yes	0	0
Yes	Yes	Yes	No	No	No	Yes	0	0
No	Yes	Yes	No	No	No	No	0	0
Yes	Yes	Yes	No	No	Yes	No	0	0
Yes	Yes	Yes	No	No	Yes	Yes	0	0
Yes	Yes	Yes	No	No	Yes	Yes	0	0
No	Yes	Yes	No	No	Yes	No	2	0
No	No	No	No	No	Yes	Yes	0	0
No	No	Yes	Yes	No	Yes	No	4	0
No	No	No	No	No	No	Yes	0	0

No	Yes	Yes	No	No	Yes	Yes	2	0
Yes	No	Yes	No	No	Yes	Yes	0	0
Yes	Yes	Yes	No	No	No	Yes	0	0
Yes	Yes	Yes	No	No	No	Yes	0	0
Yes	Yes	Yes	No	No	Yes	No	0	0
Yes	Yes	Yes	No	No	Yes	No	2	0
Yes	Yes	Yes	Yes	No	Yes	Yes	0	0
Yes	Yes	Yes	No	No	Yes	No	0	0
No	No	Yes	No	No	No	No	0	0
No	No	No	No	No	Yes	No	4	0
No	No	Yes	No	No	No	No	0	0
No	No	Yes	No	No	Yes	No	0	0
No	No	Yes	No	No	Yes	No	0	0
No	Yes	Yes	No	No	Yes	No	0	0
No	No	Yes	No	No	Yes	No	0	0
No	Yes	Yes	No	No	No	Yes	0	0
No	No	Yes	No	No	Yes	Yes	0	0
Yes	Yes	Yes	No	No	Yes	No	0	0
No	No	Yes	No	No	Yes	Yes	1	0
No	No	Yes	No	No	No	No	0	0
Yes	Yes	Yes	No	No	Yes	No	4	0
Yes	Yes	Yes	No	Yes	Yes	Yes	0	0
Yes	Yes	Yes	No	No	Yes	Yes	0	0
No	No	No	No	No	No	No	0	0
Yes	Yes	Yes	No	No	Yes	No	0	0
No	No	No	No	No	No	No	0	0
Yes	Yes	Yes	No	No	Yes	No	0	0
Yes	Yes	Yes	No	No	No	No	0	0
No	No	Yes	No	No	Yes	No	0	0
No	No	Yes	No	No	Yes	No	3	0
Yes	Yes	Yes	No	No	Yes	No	5	0
No	No	Yes	Yes	No	Yes	Yes	0	0
Yes	Yes	Yes	No	No	Yes	No	0	0
No	No	Yes	No	No	Yes	No	0	0
No	Yes	Yes	No	No	No	No	0	0
Yes	Yes	Yes	No	No	Yes	No	3	0
No	No	Yes	No	No	Yes	Yes	20	0
No	No	Yes	No	No	Yes	No	0	0
No	No	No	No	No	Yes	No	0	4
Yes	No	Yes	No	No	Yes	Yes	6	0
Yes	Yes	Yes	No	No	Yes	No	4	0
No	Yes	Yes	No	No	Yes	No	3	0
No	No	Yes	No	No	Yes	No	0	0
No	No	Yes	No	No	Yes	Yes	0	0
Yes	Yes	Yes	No	No	No	No	0	0
No	No	Yes	No	No	No	Yes	0	0
No	No	No	No	No	No	No	0	0
Yes	Yes	Yes	No	No	No	Yes	0	0
No	No	Yes	No	No	Yes	Yes	0	0
Yes	Yes	Yes	No	No	Yes	Yes	0	0
Yes	Yes	No	No	No	No	No	0	0
Yes	Yes	Yes	No	No	Yes	No	3	0

For peer review only

For peer review only - http://bmjopen.bmj.com/site/about/guidelines.xhtml

No	Yes	Yes	No	No	Yes	Yes	3	0
Yes	Yes	Yes	No	No	Yes	No	3	0
No	No	No	No	No	Yes	No	0	0
No	No	Yes	No	No	Yes	Yes	10	0
Yes	Yes	No	No	No	No	No	0	0
No	No	No	No	No	Yes	No	4	0
No	Yes	No	No	No	No	Yes	0	0
No	Yes	Yes	No	No	Yes	No	0	0
No	No	Yes	Yes	No	Yes	No	2	0
No	No	Yes	No	No	Yes	Yes	0	0
No	Yes	Yes	No	No	Yes	No	1	0
Yes	Yes	Yes	No	No	No	No	0	0
No	No	Yes	No	No	Yes	No	0	0
No	Yes	Yes	No	No	Yes	Yes	3	0
No	Yes	No	No	No	Yes	No	0	0
Yes	Yes	Yes	No	Yes	No	Yes	0	0
No	No	Yes	No	No	Yes	No	0	0
Yes	Yes	Yes	No	No	Yes	Yes	0	0
No	No	Yes	No	No	Yes	No	0	0
Yes	Yes	Yes	No	No	Yes	No	0	0
Yes	Yes	Yes	No	No	Yes	Yes	0	0
Yes	Yes	Yes	No	No	Yes	No	3	0
No	No	Yes	No	No	Yes	No	2	0
No	No	No	No	No	Yes	No	0	0
Yes	Yes	Yes	No	No	Yes	Yes	0	0
No	No	Yes	No	Yes	Yes	Yes	0	0
Yes	No	Yes	No	No	Yes	Yes	7	0
Yes	Yes	Yes	No	No	No	Yes	0	0
Yes	Yes	Yes	No	No	No	Yes	0	0
Yes	Yes	Yes	No	No	Yes	Yes	0	0
No	No	Yes	No	No	Yes	No	9	0
No	Yes	Yes	No	No	Yes	No	0	0
Yes	Yes	Yes	No	No	No	No	0	0
Yes	Yes	Yes	No	No	Yes	No	0	0
No	Yes	No	No	No	No	No	0	0
Yes	Yes	Yes	No	No	Yes	No	1	0
Yes	Yes	Yes	No	No	No	No	0	0
No	No	Yes	No	No	No	Yes	0	0
No	No	Yes	No	No	Yes	Yes	2	0
Yes	Yes	Yes	No	No	Yes	No	1	0
No	No	Yes	No	No	No	No	0	0
Yes	Yes	Yes	No	No	Yes	Yes	0	0
No	Yes	Yes	No	No	Yes	No	4	0
Yes	Yes	Yes	No	No	No	Yes	0	0
Yes	Yes	Yes	No	No	No	No	0	0
No	No	Yes	No	No	Yes	Yes	0	0
No	Yes	No	No	No	No	No	0	0
No	No	No	Yes	No	Yes	Yes	0	0
Yes	Yes	Yes	No	No	Yes	Yes	2	0
Yes	Yes	Yes	No	No	Yes	No	5	0
No	No	No	No	No	Yes	No	0	0
No	No	Yes	No	No	Yes	No	0	0

For peer review only

No	No	Yes	No	No	Yes	No	0	0
No	No	Yes	No	No	Yes	No	0	0
No	No	No	No	No	Yes	No	0	1
No	No	Yes	No	No	Yes	No	0	0
Yes	Yes	Yes	No	No	Yes	Yes	0	0
No	No	No	No	No	Yes	No	0	0
Yes	No	No	No	No	Yes	No	12	0
No	No	No	No	No	Yes	No	0	0
No	No	No	No	No	Yes	No	4	0
Yes	Yes	Yes	No	No	Yes	Yes	0	0
No	No	No	No	No	Yes	No	3	0
No	No	Yes	No	No	Yes	No	10	0
No	No	No	No	No	Yes	No	6	0
No	No	Yes	No	No	Yes	No	4	0
No	No	Yes	No	No	Yes	No	0	0
No	No	No	No	Yes	Yes	Yes	0	0
No	No	No	No	Yes	No	Yes	0	0
No	No	Yes	No	No	Yes	No	2	0
Yes	No	Yes	No	No	No	No	0	0
Yes	Yes	Yes	No	No	Yes	No	10	0
Yes	Yes	Yes	No	No	Yes	No	0	0
Yes	Yes	Yes	No	No	No	Yes	0	0
No	No	Yes	No	No	Yes	No	9	0
No	No	Yes	No	No	Yes	No	0	0
No	No	No	No	No	Yes	No	0	3
No	No	Yes	No	No	Yes	No	0	0
No	No	Yes	No	No	Yes	No	5	0
Yes	Yes	Yes	No	No	Yes	No	3	0
No	No	No	No	Yes	Yes	Yes	0	0
Yes	Yes	Yes	No	No	Yes	No	0	0
No	No	Yes	No	No	Yes	No	0	10
Yes	Yes	Yes	No	No	Yes	No	0	0
No	No	Yes	No	No	Yes	No	3	0
No	No	No	No	Yes	Yes	Yes	0	0
No	No	No	No	No	Yes	No	0	0
No	No	Yes	No	No	Yes	No	0	0
Yes	No	Yes	No	No	Yes	No	5	0
No	No	No	No	No	Yes	No	0	0
Yes	Yes	No	No	No	Yes	Yes	0	0
No	No	No	No	No	Yes	No	0	0
No	No	No	No	No	Yes	No	7	0
Yes	Yes	Yes	No	No	Yes	No	3	0
No	Yes	Yes	No	No	No	Yes	0	0
No	No	No	No	Yes	Yes	Yes	0	0
No	No	Yes	No	No	Yes	No	7	0
Yes	Yes	Yes	No	No	Yes	Yes	0	0
No	No	No	No	No	Yes	No	10	0
No	No	Yes	No	No	Yes	No	6	0
No	No	No	No	No	Yes	No	0	0
No	No	Yes	No	No	No	No	0	0
No	No	No	No	No	Yes	No	0	0
Yes	Yes	Yes	No	No	Yes	No	0	0

For peer review only

No	No	No	No	Yes	Yes	Yes	0	0
No	Yes	No	No	No	No	No	0	0
No	No	Yes	No	No	Yes	No	5	0
No	No	Yes	No	No	Yes	No	0	0
No	Yes	Yes	No	No	Yes	No	0	0
No	No	Yes	No	No	Yes	No	1	0
Yes	No	Yes	No	No	Yes	No	1	0
Yes	Yes	Yes	No	No	Yes	Yes	0	0
No	No	No	No	No	Yes	No	0	0
No	No	Yes	No	No	Yes	No	0	0
No	No	Yes	No	No	Yes	No	0	0
No	No	No	No	No	No	No	0	0
No	No	Yes	No	No	Yes	No	4	0
Yes	Yes	Yes	No	No	Yes	No	0	0
Yes	Yes	Yes	No	No	Yes	Yes	0	1
No	No	No	No	No	Yes	No	0	0
No	No	Yes	No	No	Yes	No	0	0
No	No	Yes	No	No	Yes	No	7	0
No	No	Yes	No	No	Yes	No	0	0
No	No	No	No	No	Yes	No	5	0
No	No	No	No	No	Yes	No	10	0
No	No	No	No	Yes	Yes	Yes	4	0
No	Yes	Yes	No	No	Yes	No	3	0
No	No	No	No	No	Yes	No	2	0
No	No	No	No	Yes	Yes	Yes	0	0
Yes	Yes	Yes	No	No	Yes	No	0	0
Yes	Yes	Yes	No	No	Yes	No	5	0
No	No	Yes	No	No	Yes	No	1	0
No	No	No	No	Yes	Yes	Yes	0	0
No	No	Yes	No	No	Yes	No	2	0
No	No	Yes	No	No	Yes	No	0	0
No	No	Yes	No	No	Yes	No	4	0
No	No	Yes	No	No	Yes	No	0	0
No	No	No	No	No	Yes	No	9	0
No	No	Yes	No	No	Yes	No	0	4
Yes	Yes	Yes	No	No	Yes	No	0	0
No	Yes	Yes	No	No	Yes	No	0	0
Yes	Yes	No	No	No	No	No	0	0
No	No	Yes	No	No	Yes	No	0	0
No	No	No	No	No	Yes	No	10	0
No	No	Yes	No	No	Yes	Yes	0	0
Yes	Yes	No	No	Yes	No	Yes	0	0
No	Yes	Yes	No	No	No	Yes	0	0
No	No	Yes	No	No	Yes	No	0	0
No	No	No	No	Yes	Yes	Yes	0	0
No	Yes	Yes	No	No	Yes	No	2	0
No	No	Yes	No	No	Yes	No	0	0
No	No	No	No	Yes	Yes	Yes	0	2
No	No	No	No	No	Yes	No	4	0
No	No	No	No	Yes	No	No	0	0
No	No	Yes	No	No	Yes	No	2	0
No	No	No	No	No	Yes	No	2	0

For peer review only

No	No	No	No	Yes	Yes	Yes	0	0
No	No	No	No	No	Yes	No	10	0
No	No	Yes	No	No	Yes	No	0	0
No	No	Yes	No	No	No	Yes	0	0
No	No	No	No	No	Yes	No	2	0
Yes	Yes	Yes	No	No	Yes	No	0	0
No	No	No	No	No	Yes	No	3	0
No	No	No	No	No	No	No	0	0
No	No	No	No	No	Yes	No	0	0
No	No	Yes	No	No	Yes	No	0	0
No	No	No	No	Yes	Yes	Yes	0	0
No	No	Yes	No	No	Yes	No	0	0
No	No	No	No	No	Yes	No	10	0
No	Yes	Yes	No	No	Yes	Yes	10	0
No	Yes	Yes	No	No	Yes	No	11	4
Yes	Yes	Yes	No	No	Yes	No	0	3
No	Yes	Yes	No	No	Yes	No	0	0
No	No	No	No	Yes	Yes	No	0	0
Yes	Yes	Yes	No	No	No	Yes	0	0
Yes	Yes	Yes	No	No	No	Yes	0	0
No	No	Yes	No	No	Yes	No	2	0
Yes	Yes	Yes	No	No	Yes	Yes	0	0
No	No	Yes	No	No	No	No	0	0
Yes	No	Yes	No	No	Yes	No	0	0
Yes	Yes	Yes	No	No	Yes	No	0	2
No	No	Yes	No	No	Yes	No	0	2
Yes	Yes	Yes	No	No	Yes	Yes	4	0
Yes	Yes	Yes	No	No	Yes	Yes	0	0
Yes	Yes	Yes	No	No	Yes	No	6	0
Yes	Yes	Yes	No	No	Yes	No	0	0
Yes	Yes	Yes	No	No	Yes	No	11	0
No	Yes	No	No	No	Yes	No	0	0
Yes	Yes	Yes	No	No	Yes	No	0	0
Yes	Yes	Yes	No	No	Yes	Yes	100	0
No	No	Yes	No	No	Yes	No	1	0
Yes	No	Yes	No	No	Yes	No	6	0
Yes	Yes	No	No	No	Yes	No	2	4
Yes	Yes	Yes	No	No	No	Yes	0	0
Yes	No	Yes	No	No	Yes	Yes	0	0
No	Yes	Yes	No	No	Yes	No	40	17
Yes	Yes	Yes	No	No	Yes	Yes	0	0
No	No	No	No	Yes	No	No	0	0
No	No	No	No	Yes	Yes	No	0	0
No	No	Yes	No	Yes	Yes	No	7	0
Yes	Yes	Yes	No	No	Yes	Yes	3	3
No	No	No	No	No	Yes	No	0	0
Yes	Yes	Yes	No	No	No	Yes	0	0
Yes	Yes	Yes	No	No	Yes	Yes	0	0
Yes	No	Yes	No	No	Yes	No	4	0
Yes	Yes	No	No	No	Yes	Yes	0	0
Yes	Yes	Yes	No	No	Yes	No	1	0
No	Yes	Yes	No	Yes	Yes	No	0	0

For peer review only

Yes	Yes	Yes	No	No	Yes	No	0	0
Yes	Yes	Yes	No	No	Yes	Yes	4	0
Yes	Yes	No	No	No	No	No	0	0
No	Yes	Yes	No	No	No	No	0	0
Yes	Yes	Yes	No	No	Yes	No	0	10
Yes	Yes	Yes	No	No	No	Yes	0	0
No	Yes	Yes	No	No	Yes	No	0	0
Yes	Yes	Yes	No	No	Yes	No	6	1
Yes	Yes	Yes	No	No	Yes	No	1	0
Yes	Yes	Yes	No	No	Yes	Yes	6	0
Yes	Yes	Yes	No	No	Yes	No	2	0
No	No	Yes	No	No	Yes	No	3	0
Yes	Yes	Yes	No	No	Yes	No	6	0
Yes	Yes	Yes	No	No	No	No	0	0
Yes	Yes	Yes	No	No	Yes	Yes	10	0
No	No	No	No	No	No	Yes	0	0
Yes	Yes	No	No	No	Yes	No	6	0
No	Yes	Yes	No	No	No	No	0	0
No	Yes	Yes	No	No	No	No	0	0
No	Yes	Yes	No	No	Yes	No	0	0
Yes	Yes	Yes	No	No	Yes	Yes	0	0
No	Yes	Yes	No	No	Yes	No	5	4
Yes	Yes	Yes	No	No	Yes	Yes	6	0
Yes	Yes	Yes	No	No	No	No	0	0
No	Yes	Yes	No	No	Yes	Yes	20	4
Yes	Yes	Yes	No	Yes	Yes	Yes	8	0
Yes	Yes	Yes	No	No	Yes	No	0	0
No	Yes	Yes	No	No	Yes	No	6	0
Yes	No	No	No	No	Yes	No	2	0
Yes	Yes	Yes	Yes	No	Yes	Yes	6	0
No	No	Yes	No	No	Yes	No	2	3
No	No	Yes	No	No	Yes	No	0	0
Yes	Yes	Yes	No	No	No	No	0	0
Yes	Yes	Yes	No	No	Yes	Yes	1	0
Yes	Yes	Yes	No	No	No	No	0	0
No	Yes	Yes	No	No	Yes	No	0	0
No	No	Yes	No	No	Yes	Yes	3	0
Yes	Yes	Yes	No	No	No	Yes	0	0
Yes	Yes	Yes	No	No	Yes	No	6	0
No	Yes	Yes	No	No	Yes	Yes	6	3
Yes	Yes	Yes	No	No	No	Yes	0	0
Yes	Yes	Yes	No	No	Yes	Yes	13	0
No	Yes	Yes	No	No	No	No	0	0
Yes	Yes	Yes	No	No	Yes	No	3	0
No	Yes	Yes	No	No	No	No	0	0
Yes	Yes	Yes	No	No	No	No	0	0
Yes	Yes	Yes	No	No	Yes	No	0	0
Yes	Yes	Yes	No	Yes	Yes	No	2	1
No	Yes	Yes	No	No	Yes	Yes	9	0
No	Yes	Yes	No	No	Yes	Yes	0	3
No	Yes	Yes	No	No	Yes	No	0	0
No	Yes	Yes	No	No	Yes	No	4	0

For peer review only

Yes	No	No	No	No	Yes	No	2	2
Yes	Yes	Yes	No	No	Yes	Yes	7	0
No	No	Yes	No	No	No	No	0	0
Yes	Yes	Yes	No	No	Yes	Yes	3	0
Yes	Yes	Yes	No	No	Yes	Yes	3	0
No	No	No	No	Yes	Yes	No	1	0
No	Yes	Yes	No	No	Yes	Yes	2	0
Yes	Yes	Yes	No	No	No	No	0	0
No	Yes	Yes	No	No	Yes	No	4	0
No	Yes	Yes	No	No	No	No	0	0
Yes	Yes	Yes	No	No	Yes	Yes	8	0
Yes	Yes	Yes	No	No	Yes	Yes	50	0
No	No	No	No	No	Yes	No	6	0
Yes	Yes	Yes	No	No	Yes	Yes	8	0
Yes	Yes	Yes	No	No	Yes	No	8	0
Yes	Yes	Yes	No	No	Yes	No	0	0
No	Yes	Yes	No	No	Yes	No	2	0
Yes	Yes	Yes	No	No	Yes	No	0	0
No	No	Yes	No	No	Yes	No	3	2
Yes	Yes	Yes	No	No	Yes	No	4	0
Yes	Yes	Yes	No	No	Yes	No	0	2
No	Yes	Yes	No	No	Yes	No	11	0
No	No	No	No	No	Yes	No	5	0
Yes	Yes	Yes	No	No	Yes	No	8	0
No	No	No	No	Yes	No	No	0	0
No	Yes	Yes	No	No	No	No	0	0
Yes	Yes	Yes	No	No	Yes	Yes	4	0
Yes	Yes	Yes	No	No	Yes	Yes	0	0
No	No	Yes	No	Yes	Yes	No	6	3
No	No	Yes	No	No	Yes	No	5	0
No	Yes	Yes	No	No	Yes	No	7	0
Yes	Yes	Yes	No	No	Yes	No	3	0
Yes	Yes	Yes	No	No	No	No	0	0
No	Yes	Yes	No	No	Yes	No	0	3
Yes	Yes	Yes	No	No	Yes	Yes	3	0
Yes	Yes	Yes	No	No	Yes	No	0	0
Yes	Yes	Yes	No	No	Yes	Yes	10	5
Yes	Yes	Yes	No	No	No	No	0	0
Yes	Yes	No	No	No	Yes	Yes	2	0
No	No	No	No	No	Yes	Yes	0	0
Yes	Yes	Yes	No	No	Yes	Yes	1	0
Yes	Yes	Yes	No	No	No	No	0	0
Yes	Yes	Yes	No	No	Yes	Yes	1	0
Yes	Yes	Yes	No	Yes	Yes	No	4	0
No	Yes	Yes	No	No	Yes	Yes	10	0
Yes	Yes	Yes	No	No	No	Yes	0	0
Yes	Yes	No	No	No	No	No	0	0
No	Yes	Yes	No	No	Yes	Yes	8	4
Yes	Yes	Yes	No	No	No	Yes	0	0
No	No	Yes	No	No	No	Yes	0	0
No	No	No	No	No	Yes	No	9	0
No	No	Yes	No	Yes	Yes	No	2	0

For peer review only

Yes	Yes	Yes	No	No	Yes	Yes	3	4
Yes	Yes	Yes	No	No	Yes	Yes	3	0
Yes	Yes	Yes	No	No	Yes	Yes	7	0
Yes	Yes	No	No	Yes	No	No	0	0
No	No	Yes	No	No	Yes	No	0	3
Yes	Yes	Yes	No	No	Yes	No	6	0
No	No	No	No	No	Yes	No	0	10
Yes	Yes	Yes	No	No	Yes	No	0	0
Yes	Yes	Yes	No	No	Yes	Yes	3	0
Yes	No	Yes	No	No	Yes	No	4	2
Yes	Yes	Yes	No	No	Yes	No	4	0
No	No	No	No	No	Yes	Yes	0	0
Yes	Yes	Yes	No	No	Yes	No	0	0
No	Yes	Yes	No	No	Yes	Yes	3	0
No	Yes	Yes	No	No	Yes	Yes	9	0
No	No	Yes	No	No	Yes	No	5	3
Yes	Yes	Yes	No	No	Yes	No	4	2
Yes	No	No	No	No	Yes	No	2	2
No	Yes	No	No	Yes	Yes	No	2	0
Yes	Yes	Yes	No	No	Yes	Yes	12	3
Yes	Yes	Yes	No	No	Yes	Yes	0	0
No	Yes	Yes	No	No	Yes	No	0	0
Yes	Yes	Yes	No	No	No	Yes	0	0
Yes	Yes	Yes	No	No	Yes	Yes	0	0
No	Yes	Yes	No	No	Yes	No	0	0
No	Yes	Yes	No	No	Yes	No	11	7
Yes	Yes	Yes	No	No	Yes	Yes	1	0
Yes	Yes	Yes	No	No	No	No	0	0
Yes	Yes	Yes	No	No	No	No	0	0
No	No	No	No	No	No	Yes	0	0
Yes	Yes	Yes	No	No	Yes	Yes	5	4
No	No	Yes	No	No	Yes	No	6	2
Yes	No	Yes	No	No	Yes	Yes	6	0
Yes	Yes	Yes	No	No	No	No	0	0
Yes	Yes	Yes	No	No	Yes	Yes	0	0
Yes	Yes	Yes	No	No	Yes	Yes	0	0
No	Yes	Yes	No	No	Yes	No	1	0
No	No	No	No	No	Yes	Yes	2	0
No	No	No	No	No	Yes	Yes	7	0
No	No	Yes	No	No	Yes	Yes	4	0
No	No	No	No	No	Yes	No	0	0
No	No	No	No	No	Yes	Yes	4	0
No	No	No	No	No	Yes	No	0	0
No	No	Yes	No	No	Yes	Yes	3	0
No	No	Yes	No	No	Yes	No	0	0
No	No	Yes	No	No	Yes	No	3	0
No	No	Yes	No	No	Yes	No	5	0
Yes	No	Yes	No	No	Yes	Yes	4	0
No	No	Yes	No	No	No	No	0	0
No	No	Yes	No	No	Yes	No	6	0
No	No	Yes	No	No	Yes	Yes	2	0
No	No	Yes	No	No	Yes	Yes	2	0

For peer review only

Yes	No	No	No	No	Yes	Yes	3	0
No	No	Yes	No	No	Yes	Yes	5	0
No	No	No	No	No	Yes	Yes	3	0
No	No	No	No	No	Yes	Yes	0	0
No	No	Yes	No	No	Yes	No	5	0
No	No	Yes	No	No	Yes	Yes	2	0
No	No	No	No	No	Yes	Yes	2	0
No	No	No	No	No	Yes	No	3	0
Yes	Yes	Yes	No	No	Yes	Yes	3	0
No	No	No	No	No	Yes	Yes	4	0
Yes	Yes	Yes	No	No	Yes	Yes	1	0
No	No	No	No	No	Yes	Yes	4	0
No	No	Yes	No	No	Yes	No	0	0
No	No	Yes	No	No	Yes	Yes	3	0
No	No	No	No	No	Yes	Yes	0	0
No	No	Yes	No	No	Yes	No	0	0
No	No	No	No	No	Yes	Yes	0	0
No	No	No	No	No	Yes	Yes	6	0
No	No	Yes	No	No	Yes	Yes	0	0
No	No	Yes	No	No	Yes	Yes	2	0
No	No	Yes	No	No	Yes	Yes	0	0
No	No	Yes	No	No	Yes	Yes	0	0
No	No	No	No	No	No	No	0	0
No	No	Yes	No	No	Yes	Yes	2	0
No	No	Yes	No	No	Yes	Yes	0	0
No	No	No	No	No	Yes	Yes	0	0
No	No	No	No	No	Yes	Yes	2	0
No	No	No	No	No	Yes	Yes	4	0
No	No	Yes	No	No	Yes	Yes	0	0
No	No	Yes	No	No	Yes	Yes	0	0
No	No	No	No	No	Yes	Yes	0	0
No	No	No	No	No	Yes	Yes	6	0
No	No	No	No	No	Yes	Yes	6	0
No	No	Yes	No	No	Yes	Yes	0	0
No	No	Yes	No	No	Yes	Yes	3	0
No	No	No	No	No	Yes	Yes	9	0
No	No	No	No	No	Yes	Yes	0	0
No	No	Yes	No	No	Yes	Yes	0	0
No	No	No	No	No	Yes	Yes	1	0
No	No	Yes	No	No	Yes	Yes	2	0
No	No	No	No	No	Yes	Yes	4	0
No	No	No	No	No	Yes	Yes	0	0
No	No	Yes	No	No	Yes	No	5	0
No	No	No	No	No	Yes	No	5	0
No	No	Yes	No	No	Yes	Yes	1	0
No	No	Yes	No	No	Yes	No	4	0
No	No	No	No	No	Yes	Yes	0	0
No	No	No	No	No	No	Yes	0	0
Yes	Yes	Yes	No	No	Yes	No	0	0
Yes	Yes	Yes	No	No	Yes	Yes	0	0
Yes	Yes	Yes	No	No	Yes	Yes	0	0
Yes	Yes	Yes	No	No	Yes	Yes	3	0

Yes	Yes	Yes	No	No	Yes	No	0	0
No	No	No	No	No	No	No	0	0
Yes	Yes	Yes	No	No	No	Yes	0	0
Yes	Yes	Yes	No	No	Yes	Yes	1	0
Yes	Yes	Yes	No	No	Yes	Yes	2	0
No	Yes	Yes	No	No	No	Yes	0	0
No	No	No	No	No	Yes	Yes	0	0
Yes	Yes	Yes	No	No	Yes	No	0	0
No	No	No	No	Yes	Yes	No	0	0
Yes	Yes	Yes	No	No	Yes	Yes	1	0
Yes	Yes	Yes	No	Yes	Yes	No	2	0
Yes	Yes	Yes	No	No	No	No	0	0
Yes	Yes	Yes	No	No	Yes	Yes	0	0
Yes	Yes	Yes	No	No	No	Yes	0	0
Yes	Yes	Yes	No	No	No	Yes	0	0
Yes	No	Yes	No	No	Yes	Yes	0	0
No	No	Yes	No	No	No	Yes	0	0
Yes	Yes	Yes	No	No	Yes	Yes	0	0
Yes	Yes	Yes	No	No	Yes	Yes	4	0
Yes	Yes	Yes	No	No	Yes	Yes	0	0
Yes	Yes	Yes	No	No	No	No	0	0
Yes	Yes	Yes	No	No	Yes	Yes	2	0
Yes	Yes	Yes	No	No	Yes	No	0	0
Yes	Yes	Yes	No	No	Yes	Yes	0	0
No	No	Yes	No	No	Yes	Yes	5	0
Yes	Yes	Yes	No	No	Yes	Yes	0	0
Yes	Yes	Yes	No	No	No	Yes	0	0
Yes	Yes	Yes	No	No	Yes	Yes	1	0
Yes	Yes	Yes	No	No	Yes	Yes	1	0
No	No	Yes	No	No	No	Yes	0	0
Yes	Yes	Yes	No	No	Yes	Yes	0	0
Yes	Yes	Yes	No	No	Yes	Yes	0	0
Yes	Yes	Yes	No	No	No	Yes	0	0
No	No	No	No	No	Yes	Yes	0	0
Yes	Yes	No	No	No	No	Yes	0	0
Yes	Yes	Yes	No	No	No	Yes	0	0
No	No	Yes	No	No	No	No	0	0
Yes	No	Yes	No	No	Yes	Yes	0	0
Yes	Yes	Yes	No	No	Yes	Yes	7	0
Yes	Yes	Yes	No	No	No	Yes	0	0
Yes	Yes	Yes	No	No	Yes	No	0	0
Yes	Yes	Yes	No	No	Yes	Yes	0	0
Yes	Yes	Yes	No	No	Yes	Yes	0	0
Yes	Yes	Yes	No	No	No	No	0	0
Yes	Yes	Yes	No	No	No	Yes	0	0
Yes	Yes	No	No	No	No	Yes	0	0
Yes	Yes	Yes	No	No	No	No	0	0
Yes	Yes	Yes	No	No	No	Yes	0	0
No	No	Yes	No	No	Yes	Yes	3	0
Yes	Yes	Yes	No	No	No	Yes	0	0
Yes	Yes	Yes	No	No	Yes	Yes	12	0
Yes	Yes	Yes	No	No	Yes	Yes	0	0

For peer review only

Yes	Yes	Yes	No	No	Yes	Yes	0	0
Yes	Yes	Yes	No	No	Yes	No	0	0
Yes	Yes	Yes	No	No	No	Yes	0	0
Yes	No	Yes	No	No	Yes	Yes	4	0
No	No	Yes	No	No	No	No	0	0
Yes	Yes	No	No	No	Yes	No	0	0
Yes	Yes	Yes	No	No	Yes	Yes	2	0
Yes	Yes	Yes	No	No	Yes	Yes	0	0
Yes	Yes	Yes	No	No	Yes	Yes	0	0
No	No	Yes	No	No	Yes	No	0	0
Yes	Yes	Yes	No	No	Yes	Yes	20	0
Yes	Yes	No	No	Yes	Yes	No	0	0
Yes	Yes	Yes	No	No	No	No	0	0
No	Yes	No	No	No	Yes	No	0	0
Yes	Yes	Yes	No	Yes	Yes	Yes	0	0
Yes	Yes	Yes	No	No	Yes	No	0	0
No	No	Yes	No	No	Yes	Yes	3	0
Yes	Yes	Yes	No	No	No	Yes	0	0
Yes	Yes	Yes	No	No	Yes	Yes	0	0
Yes	Yes	Yes	No	No	Yes	Yes	0	0
Yes	Yes	Yes	No	No	Yes	Yes	8	0
Yes	Yes	Yes	No	No	Yes	Yes	6	0
Yes	Yes	Yes	No	No	No	Yes	0	0
Yes	Yes	Yes	No	No	Yes	Yes	0	0
No	No	Yes	No	No	Yes	Yes	5	0
Yes	Yes	Yes	No	No	No	No	0	0
Yes	Yes	Yes	No	No	Yes	Yes	0	0
Yes	Yes	Yes	No	No	No	Yes	0	0
Yes	Yes	Yes	No	No	No	Yes	0	0
Yes	No	Yes	No	No	No	Yes	0	0
No	Yes	Yes	No	No	No	Yes	0	0
No	No	No	No	No	No	No	0	0
Yes	Yes	Yes	No	No	Yes	Yes	0	0
No	No	No	No	No	Yes	Yes	0	0
Yes	Yes	Yes	No	No	Yes	Yes	5	0
Yes	Yes	Yes	No	No	Yes	No	0	0
Yes	Yes	Yes	No	No	Yes	Yes	1	0
No	No	Yes	No	No	Yes	No	0	0
Yes	Yes	Yes	No	No	Yes	Yes	7	0
Yes	Yes	Yes	No	No	No	Yes	0	0
No	No	No	No	No	No	No	0	0
Yes	Yes	No	No	No	Yes	No	2	0
Yes	Yes	Yes	No	No	Yes	Yes	11	0
Yes	Yes	Yes	No	No	No	No	0	0
No	No	Yes	No	No	Yes	Yes	1	0
No	No	No	No	Yes	Yes	Yes	0	0
Yes	Yes	Yes	No	No	No	Yes	0	0
Yes	Yes	Yes	No	No	Yes	Yes	0	0
Yes	Yes	Yes	No	No	Yes	Yes	2	0
Yes	Yes	Yes	No	No	No	Yes	0	0
Yes	Yes	Yes	No	No	Yes	No	0	0
Yes	Yes	Yes	No	No	No	Yes	0	0

For peer review only

Yes	Yes	Yes	No	No	Yes	Yes	17	0
Yes	Yes	Yes	No	No	No	Yes	0	0
Yes	Yes	Yes	No	No	No	Yes	0	0
Yes	Yes	Yes	No	No	Yes	Yes	0	0
Yes	Yes	Yes	No	No	Yes	Yes	0	0
Yes	Yes	Yes	No	No	Yes	Yes	0	0
Yes	Yes	Yes	No	Yes	Yes	No	0	0
No	No	No	No	No	Yes	Yes	0	0
Yes	Yes	Yes	No	No	Yes	Yes	0	0
No	No	No	No	No	Yes	No	0	0
Yes	Yes	Yes	Yes	Yes	Yes	Yes	0	0
Yes	Yes	Yes	No	No	Yes	No	0	0
Yes	Yes	Yes	No	No	No	Yes	0	0
No	No	Yes	No	No	No	Yes	0	0
No	Yes	Yes	No	No	No	No	0	0
Yes	Yes	Yes	No	No	Yes	Yes	2	0
Yes	Yes	Yes	No	No	No	Yes	0	0
Yes	Yes	Yes	No	No	Yes	Yes	2	0
Yes	Yes	Yes	No	No	Yes	Yes	1	0
Yes	Yes	Yes	No	No	Yes	Yes	0	0
Yes	Yes	Yes	No	No	No	Yes	0	0
Yes	Yes	Yes	No	No	Yes	Yes	6	0
Yes	Yes	Yes	No	No	No	No	0	0
Yes	Yes	Yes	No	No	Yes	No	0	0
Yes	No	Yes	No	No	Yes	Yes	0	0
Yes	Yes	Yes	No	No	Yes	No	20	0
Yes	Yes	Yes	No	No	No	No	0	0
No	Yes	Yes	No	No	Yes	No	5	0
No	No	Yes	No	No	Yes	Yes	0	0
Yes	Yes	Yes	No	No	No	Yes	0	0
Yes	Yes	Yes	No	No	Yes	Yes	0	0
Yes	Yes	Yes	No	Yes	No	Yes	0	0
No	No	Yes	No	No	No	Yes	0	0
Yes	Yes	Yes	No	No	Yes	Yes	4	0
Yes	Yes	Yes	Yes	No	No	No	0	0
No	Yes	Yes	No	No	Yes	No	4	0
Yes	Yes	Yes	Yes	No	No	No	0	0
Yes	No	Yes	No	No	Yes	No	2	0
No	Yes	Yes	No	Yes	Yes	No	11	0
Yes	Yes	Yes	No	No	Yes	Yes	0	0
No	No	Yes	No	No	Yes	No	2	0
Yes	Yes	No	No	No	Yes	No	10	0
No	No	No	No	No	Yes	No	0	0
No	Yes	Yes	No	No	Yes	No	2	0
Yes	Yes	Yes	No	No	Yes	Yes	6	0
Yes	Yes	Yes	No	Yes	Yes	Yes	0	0
Yes	Yes	Yes	No	No	Yes	No	10	5
No	No	Yes	No	Yes	Yes	No	2	0
No	No	Yes	No	No	No	No	0	0
Yes	Yes	Yes	No	No	Yes	Yes	20	0
Yes	Yes	Yes	No	No	Yes	Yes	20	6
No	No	Yes	No	No	Yes	Yes	10	6

For peer review only

No	No	No	No	No	No	No	0	0
No	Yes	Yes	No	No	Yes	Yes	6	0
Yes	No	Yes	No	No	No	Yes	0	0
Yes	Yes	Yes	Yes	Yes	Yes	No	12	0
No	No	Yes	No	No	Yes	Yes	2	9
No	No	No	No	No	No	Yes	0	0
No	No	No	No	No	No	Yes	0	0
Yes	Yes	Yes	No	No	Yes	No	2	0
Yes	Yes	Yes	No	No	Yes	Yes	0	8
Yes	Yes	Yes	No	No	Yes	No	1	0
No	No	Yes	No	Yes	Yes	No	0	0
Yes	Yes	Yes	No	No	No	No	0	0
Yes	No	Yes	No	No	No	Yes	0	0
Yes	Yes	Yes	No	No	Yes	Yes	0	0
No	No	Yes	No	No	Yes	No	2	0
Yes	Yes	Yes	No	No	No	No	0	0
Yes	Yes	Yes	No	No	Yes	No	20	0
No	No	No	No	No	No	Yes	0	0
No	No	Yes	No	No	No	Yes	0	0
Yes	Yes	Yes	No	No	Yes	No	10	0
Yes	Yes	Yes	No	No	Yes	Yes	6	0
No	No	Yes	No	No	Yes	Yes	0	3
No	No	Yes	No	Yes	Yes	No	0	3
Yes	Yes	Yes	Yes	No	Yes	No	10	0
No	Yes	Yes	No	No	Yes	Yes	5	0
Yes	Yes	Yes	No	No	Yes	No	16	0
Yes	Yes	Yes	No	No	No	No	0	0
No	No	No	No	Yes	No	Yes	0	0
No	No	No	No	No	No	No	0	0
Yes	Yes	Yes	No	No	Yes	No	10	8
No	No	Yes	No	Yes	Yes	No	0	0
No	Yes	Yes	No	No	No	No	0	0
Yes	Yes	Yes	No	No	No	No	0	0
Yes	Yes	Yes	No	No	No	No	0	0
No	No	Yes	No	No	Yes	No	3	0
No	No	Yes	No	No	Yes	No	4	6
Yes	Yes	Yes	No	No	No	No	0	0
No	No	Yes	No	No	Yes	No	4	0
Yes	Yes	Yes	No	No	No	No	0	0
No	Yes	Yes	No	No	Yes	No	5	0
Yes	Yes	Yes	No	No	No	No	0	0
Yes	Yes	Yes	No	No	Yes	No	3	0
Yes	Yes	Yes	No	No	No	No	0	0
Yes	Yes	Yes	No	No	Yes	Yes	2	4
Yes	Yes	Yes	Yes	No	Yes	Yes	0	0
Yes	Yes	Yes	No	No	Yes	Yes	10	2
Yes	Yes	Yes	No	No	No	No	0	0
No	No	Yes	No	No	No	No	0	0
No	No	Yes	No	No	Yes	Yes	8	0
No	No	Yes	No	Yes	Yes	No	0	0
No	No	Yes	No	No	Yes	Yes	0	0
Yes	Yes	Yes	No	No	Yes	Yes	0	2

For peer review only

Yes	Yes	Yes	No	No	Yes	No	0	1
Yes	Yes	Yes	No	No	Yes	No	1	0
Yes	Yes	Yes	No	No	Yes	No	4	0
No	No	Yes	No	No	Yes	Yes	2	0
No	No	Yes	No	Yes	Yes	Yes	10	3
Yes	Yes	Yes	Yes	No	Yes	No	25	0
No	No	Yes	No	No	Yes	Yes	3	0
Yes	Yes	Yes	No	No	No	No	0	0
Yes	Yes	Yes	No	No	Yes	Yes	2	0
No	No	Yes	No	Yes	Yes	No	6	0
Yes	Yes	Yes	No	No	No	No	0	0
No	No	No	No	No	No	No	0	0
Yes	Yes	Yes	No	No	Yes	No	6	5
Yes	Yes	Yes	No	No	Yes	No	9	2
Yes	Yes	Yes	No	No	Yes	Yes	6	2
No	No	Yes	No	No	Yes	No	0	0
No	No	No	No	No	Yes	Yes	3	0
Yes	Yes	Yes	Yes	No	No	No	0	0
Yes	No	Yes	No	Yes	Yes	No	20	0
Yes	Yes	Yes	Yes	No	Yes	Yes	0	0
No	No	Yes	No	No	Yes	Yes	0	0
No	No	Yes	No	No	Yes	Yes	3	0
No	No	No	No	No	Yes	No	5	0
No	No	No	No	No	Yes	No	0	0
Yes	Yes	Yes	Yes	No	Yes	Yes	0	0
Yes	Yes	Yes	No	Yes	Yes	Yes	5	0
No	No	No	No	No	Yes	No	8	0
Yes	Yes	Yes	No	No	Yes	Yes	10	0
No	No	No	No	No	Yes	Yes	0	0
Yes	Yes	Yes	No	No	Yes	Yes	14	0
Yes	Yes	Yes	No	No	Yes	Yes	0	0
Yes	Yes	Yes	No	No	Yes	Yes	3	0
Yes	Yes	Yes	No	Yes	Yes	Yes	0	0
No	No	No	No	No	Yes	No	1	0
No	No	Yes	No	No	Yes	Yes	1	0
Yes	Yes	Yes	No	No	Yes	Yes	5	0
No	No	No	No	No	No	No	0	0
No	No	Yes	No	No	No	Yes	0	0
Yes	Yes	Yes	No	No	Yes	Yes	20	0
No	No	No	No	No	Yes	Yes	3	0
Yes	No	Yes	No	No	Yes	Yes	15	0
Yes	Yes	Yes	No	Yes	Yes	Yes	6	0
No	No	No	No	No	No	No	0	0
No	No	Yes	No	No	Yes	No	0	0
Yes	Yes	Yes	No	No	Yes	Yes	1	0
No	No	Yes	No	No	Yes	No	4	0
Yes	Yes	Yes	No	No	Yes	Yes	2	0
Yes	Yes	Yes	Yes	No	Yes	Yes	5	0
Yes	Yes	Yes	No	No	Yes	Yes	0	0
Yes	Yes	Yes	No	No	Yes	Yes	7	0
Yes	Yes	Yes	No	Yes	Yes	Yes	6	0
No	No	Yes	No	Yes	No	Yes	0	0

For peer review only

Yes	No	No	No	No	Yes	Yes	0	0
No	No	No	No	No	Yes	No	4	0
No	No	No	No	No	Yes	No	7	0
Yes	Yes	Yes	No	Yes	Yes	Yes	8	0
No	No	No	No	No	Yes	No	3	0
No	No	Yes	No	No	Yes	Yes	6	0
No	No	No	No	No	Yes	No	20	0
No	No	No	No	No	Yes	No	30	0
No	No	Yes	No	No	Yes	No	0	0
Yes	No	Yes	No	No	Yes	No	3	0
Yes	Yes	Yes	No	No	Yes	Yes	25	0
No	Yes	Yes	No	No	No	No	0	0
Yes	Yes	Yes	No	Yes	Yes	Yes	5	0
No	No	Yes	No	No	Yes	No	5	0
No	No	No	No	No	Yes	No	2	0
No	No	Yes	No	No	Yes	Yes	3	0
No	No	No	No	No	Yes	No	15	0
Yes	Yes	Yes	No	No	Yes	Yes	0	0
No	No	Yes	No	No	Yes	No	0	0
No	No	No	No	No	Yes	Yes	12	0
No	No	Yes	No	No	Yes	No	15	0
No	Yes	Yes	No	No	Yes	No	3	0
Yes	Yes	Yes	No	Yes	Yes	Yes	2	0
No	No	Yes	No	No	Yes	No	5	0
Yes	No	Yes	No	No	Yes	No	0	0
Yes	Yes	Yes	No	Yes	Yes	Yes	4	0
No	No	No	No	No	Yes	Yes	0	0
Yes	Yes	Yes	No	No	Yes	Yes	10	0
Yes	Yes	Yes	No	No	Yes	Yes	0	0
Yes	Yes	Yes	No	Yes	Yes	Yes	2	0
Yes	Yes	Yes	No	No	Yes	Yes	4	0
Yes	No	No	No	No	No	No	0	0
Yes	Yes	Yes	No	Yes	Yes	Yes	3	0
No	Yes	Yes	No	No	Yes	No	5	0
No	No	Yes	No	No	Yes	Yes	7	0
No	No	Yes	No	No	Yes	No	4	0
Yes	No	No	No	No	Yes	Yes	12	0
Yes	No	Yes	No	No	Yes	Yes	12	0
No	No	Yes	No	No	Yes	No	0	0
No	No	No	No	No	Yes	No	2	0
Yes	Yes	Yes	No	No	Yes	Yes	5	0
Yes	No	Yes	No	No	Yes	Yes	3	0
Yes	Yes	Yes	No	No	Yes	No	40	0
No	No	Yes	No	No	Yes	Yes	3	0
No	No	Yes	No	No	Yes	No	3	0
No	No	Yes	No	No	Yes	No	4	0
No	No	No	No	No	No	No	0	0
Yes	Yes	Yes	No	Yes	Yes	Yes	11	0
Yes	No	No	No	No	Yes	Yes	0	0
No	No	No	No	No	No	No	0	0
Yes	Yes	Yes	No	No	Yes	Yes	15	0
No	No	No	No	No	Yes	No	9	0

For peer review only

Yes	Yes	Yes	No	No	Yes	Yes	0	0
No	No	Yes	No	No	Yes	No	5	0
Yes	Yes	Yes	No	Yes	Yes	Yes	0	0
No	No	No	No	No	No	No	0	0
No	No	No	No	No	Yes	No	8	0
No	No	No	No	No	Yes	No	0	0
Yes	Yes	Yes	No	Yes	Yes	Yes	15	0
No	No	No	No	No	Yes	No	1	0
Yes	Yes	Yes	No	No	Yes	Yes	2	0
Yes	Yes	Yes	No	Yes	Yes	Yes	12	0
Yes	Yes	Yes	No	No	Yes	Yes	5	0
No	No	No	No	Yes	Yes	No	4	0
Yes	Yes	Yes	No	No	Yes	Yes	8	0
Yes	Yes	Yes	No	No	Yes	Yes	13	0
Yes	Yes	Yes	No	No	Yes	Yes	2	0
Yes	No	Yes	No	No	Yes	Yes	4	0
Yes	Yes	Yes	No	Yes	Yes	Yes	5	0
No	No	No	No	No	No	No	0	0
Yes	Yes	Yes	No	No	Yes	Yes	0	2
Yes	Yes	Yes	No	No	Yes	Yes	10	0
Yes	Yes	Yes	No	Yes	Yes	Yes	6	0
No	Yes	Yes	No	No	No	Yes	0	0
Yes	Yes	Yes	No	Yes	Yes	No	0	0
Yes	Yes	Yes	No	Yes	Yes	No	4	0
Yes	No	Yes	No	Yes	No	No	0	0
Yes	Yes	Yes	No	No	Yes	Yes	10	0
Yes	Yes	Yes	No	No	Yes	Yes	0	0
Yes	Yes	Yes	No	No	Yes	No	10	0
Yes	No	Yes	No	No	Yes	Yes	3	3
Yes	Yes	Yes	No	No	No	Yes	0	0
Yes	Yes	Yes	No	No	No	No	0	0
Yes	Yes	Yes	No	No	No	No	0	0
Yes	Yes	Yes	No	No	Yes	Yes	6	0
Yes	Yes	Yes	No	No	Yes	No	0	3
Yes	Yes	Yes	No	No	Yes	Yes	0	0
Yes	Yes	Yes	No	No	No	Yes	0	0
Yes	Yes	Yes	No	No	No	Yes	0	0
Yes	Yes	Yes	No	No	Yes	No	0	0
Yes	No	Yes	No	No	No	No	0	0
Yes	Yes	Yes	No	No	Yes	No	15	2
Yes	No	Yes	No	Yes	No	No	0	0
Yes	Yes	No	No	No	No	No	0	0
Yes	Yes	Yes	No	Yes	Yes	Yes	7	0
Yes	Yes	Yes	No	Yes	No	No	0	0
Yes	Yes	Yes	No	Yes	No	No	0	0
Yes	Yes	Yes	No	No	No	Yes	0	0
Yes	Yes	Yes	No	No	Yes	No	4	0
Yes	Yes	Yes	No	No	No	Yes	0	0
Yes	No	Yes	No	Yes	No	No	0	0
Yes	Yes	Yes	No	No	Yes	No	3	0
Yes	Yes	Yes	No	No	Yes	No	5	0
Yes	Yes	Yes	No	Yes	No	No	0	0

For peer review only

Yes	No	Yes	No	Yes	No	No	0	0
Yes	Yes	Yes	No	No	No	No	0	0
Yes	Yes	Yes	No	Yes	No	No	0	0
Yes	Yes	Yes	No	Yes	Yes	Yes	0	0
Yes	Yes	Yes	No	No	Yes	No	2	0
Yes	Yes	Yes	No	No	Yes	Yes	5	0
Yes	Yes	Yes	No	No	Yes	No	0	2
Yes	No	Yes	No	No	Yes	Yes	0	0
No	No	Yes	No	No	Yes	No	5	0
Yes	Yes	Yes	No	No	Yes	No	5	5
Yes	No	Yes	No	No	Yes	Yes	5	0
Yes	Yes	Yes	No	No	Yes	Yes	0	0
Yes	Yes	Yes	No	No	No	No	0	0
Yes	Yes	No	No	No	Yes	No	4	0
Yes	Yes	No	No	Yes	Yes	Yes	0	4
No	No	Yes	No	No	Yes	No	5	0
No	Yes	Yes	No	No	Yes	Yes	10	0
Yes	Yes	Yes	No	No	Yes	Yes	0	0
No	No	No	No	No	No	No	0	0
Yes	Yes	Yes	No	No	Yes	No	10	0
Yes	Yes	Yes	No	No	Yes	No	12	2
Yes	Yes	Yes	No	No	Yes	No	2	0
No	No	Yes	No	No	Yes	No	2	0
Yes	Yes	Yes	No	Yes	Yes	Yes	10	0
Yes	Yes	Yes	No	No	Yes	No	2	4
Yes	Yes	Yes	No	No	Yes	No	4	2
No	No	Yes	No	No	Yes	No	0	0
Yes	Yes	Yes	No	No	Yes	No	3	0
Yes	Yes	Yes	No	No	Yes	Yes	10	7
No	Yes	Yes	No	No	Yes	Yes	4	0
Yes	Yes	Yes	No	No	Yes	Yes	5	2
Yes	Yes	Yes	No	No	Yes	Yes	3	2
Yes	Yes	Yes	No	No	No	No	0	0
No	No	Yes	No	No	Yes	Yes	8	0
Yes	Yes	Yes	No	No	Yes	Yes	3	0
No	Yes	Yes	No	No	Yes	Yes	2	0
Yes	Yes	Yes	No	No	Yes	Yes	5	0
Yes	Yes	Yes	No	No	Yes	Yes	10	4
Yes	Yes	No	No	No	Yes	No	5	0
Yes	Yes	Yes	No	No	Yes	No	5	1
No	No	Yes	No	No	Yes	No	0	0
Yes	Yes	Yes	No	No	Yes	Yes	2	0
Yes	Yes	Yes	No	No	Yes	Yes	4	0
Yes	No	No	No	No	Yes	No	3	0
Yes	Yes	Yes	No	No	Yes	No	4	0
No	No	Yes	No	No	Yes	No	0	0
No	No	No	No	Yes	Yes	Yes	2	0
Yes	Yes	Yes	No	Yes	No	Yes	0	0
Yes	Yes	Yes	No	No	Yes	No	8	0
Yes	No	Yes	No	No	Yes	Yes	7	11
Yes	Yes	Yes	No	No	Yes	No	10	0
No	No	Yes	No	No	Yes	No	0	0

For peer review only

No	No	Yes	No	No	Yes	No	7	8
Yes	Yes	No	No	No	Yes	No	0	0
Yes	Yes	Yes	No	No	No	Yes	0	0
No	Yes	No	No	Yes	Yes	No	0	0
No	No	Yes	No	No	Yes	No	1	0
Yes	Yes	Yes	No	No	Yes	No	8	0
Yes	Yes	No	No	No	Yes	Yes	5	0
Yes	Yes	Yes	No	No	Yes	Yes	10	0
Yes	Yes	Yes	No	No	Yes	No	3	0
Yes	Yes	Yes	No	Yes	Yes	Yes	12	6
Yes	Yes	Yes	Yes	No	No	No	0	0
Yes	Yes	Yes	No	Yes	Yes	Yes	10	4
Yes	Yes	No	No	No	Yes	No	3	0
Yes	Yes	Yes	No	No	Yes	No	1	0
Yes	Yes	Yes	No	No	Yes	No	2	0
Yes	Yes	Yes	No	No	No	Yes	0	0
No	No	Yes	No	No	Yes	No	0	0
Yes	Yes	Yes	No	No	Yes	No	12	0
Yes	Yes	No	No	No	Yes	No	3	0
Yes	Yes	Yes	No	No	Yes	Yes	8	0
Yes	Yes	Yes	No	No	Yes	No	10	0
Yes	Yes	Yes	No	No	Yes	No	5	2
Yes	Yes	Yes	No	No	Yes	Yes	0	0
No	No	Yes	No	No	Yes	No	1	0
Yes	Yes	Yes	No	No	Yes	Yes	4	0
No	No	Yes	No	No	No	Yes	0	0
No	No	Yes	No	No	Yes	No	8	0
Yes	Yes	Yes	No	Yes	Yes	Yes	5	5
Yes	Yes	Yes	No	Yes	Yes	Yes	4	0
Yes	Yes	Yes	No	No	Yes	Yes	0	0
Yes	Yes	Yes	No	No	Yes	No	10	0
No	No	Yes	No	No	Yes	No	2	0
Yes	Yes	Yes	No	No	Yes	Yes	30	0
No	Yes	Yes	No	No	Yes	No	0	0
No	No	No	No	No	Yes	No	0	0
No	Yes	Yes	No	No	Yes	Yes	8	0
Yes	Yes	Yes	No	No	Yes	Yes	2	0
Yes	Yes	Yes	No	No	Yes	Yes	8	0
Yes	Yes	Yes	No	No	Yes	No	8	0
Yes	Yes	Yes	No	No	Yes	Yes	5	0
No	No	Yes	No	No	Yes	No	5	0
Yes	Yes	Yes	No	No	Yes	Yes	8	10
Yes	Yes	Yes	No	No	Yes	Yes	0	0
No	No	Yes	No	No	Yes	Yes	3	0
Yes	Yes	Yes	No	No	Yes	No	5	0
No	No	No	No	No	Yes	Yes	8	0
Yes	Yes	Yes	No	No	Yes	Yes	12	0
Yes	Yes	Yes	No	No	Yes	Yes	3	0
Yes	No	Yes	No	No	Yes	Yes	0	0
No	No	Yes	No	No	Yes	No	10	0
Yes	Yes	Yes	No	No	Yes	No	10	7
Yes	Yes	Yes	No	No	Yes	Yes	12	0

For peer review only

No	No	Yes	No	No	Yes	No	0	0
No	No	No	No	No	Yes	No	6	0
Yes	Yes	Yes	No	No	Yes	Yes	6	18
Yes	Yes	Yes	No	No	Yes	Yes	15	0
No	No	No	No	Yes	Yes	Yes	0	0
Yes	Yes	Yes	No	No	No	Yes	0	0
Yes	Yes	Yes	No	No	Yes	Yes	3	0
Yes	No	No	No	No	Yes	Yes	10	0
Yes	Yes	Yes	No	No	Yes	Yes	5	0
Yes	Yes	Yes	No	No	Yes	Yes	0	0
No	Yes	Yes	No	No	Yes	Yes	15	0
No	No	Yes	No	No	Yes	Yes	5	0
Yes	Yes	Yes	No	No	Yes	Yes	0	0
No	No	Yes	No	No	Yes	Yes	3	0
No	No	Yes	No	No	Yes	Yes	0	0
No	No	Yes	No	No	Yes	No	5	0
Yes	Yes	Yes	No	No	Yes	Yes	2	0
No	Yes	Yes	No	No	Yes	No	0	0
No	Yes	Yes	No	No	Yes	No	0	0
Yes	Yes	No	No	No	Yes	No	2	0
No	No	No	No	No	Yes	No	10	0
Yes	Yes	No	No	No	Yes	Yes	12	5
Yes	Yes	Yes	No	No	Yes	Yes	6	0
Yes	Yes	Yes	No	No	Yes	Yes	5	0
Yes	Yes	Yes	No	No	Yes	Yes	0	0
Yes	Yes	Yes	No	No	Yes	Yes	8	0
Yes	Yes	Yes	No	No	Yes	Yes	0	0
Yes	Yes	Yes	No	No	Yes	Yes	10	0
Yes	Yes	Yes	No	No	Yes	Yes	6	0
No	Yes	Yes	No	No	Yes	No	10	0
Yes	Yes	Yes	No	No	Yes	Yes	5	6
Yes	Yes	Yes	No	No	Yes	Yes	0	0
No	No	Yes	No	No	Yes	Yes	7	0
Yes	Yes	Yes	No	Yes	Yes	Yes	8	0
Yes	No	Yes	No	No	Yes	No	10	0
Yes	Yes	Yes	No	No	Yes	No	8	0
No	Yes	Yes	No	No	Yes	No	0	0
Yes	No	Yes	No	No	Yes	Yes	0	0
Yes	Yes	Yes	No	No	Yes	No	1	0
Yes	Yes	Yes	No	No	Yes	Yes	0	0
Yes	No	Yes	No	No	Yes	Yes	0	0
Yes	Yes	Yes	No	No	No	Yes	0	0
Yes	Yes	Yes	No	No	Yes	Yes	2	3
Yes	Yes	Yes	No	No	Yes	Yes	0	0
Yes	No	Yes	No	No	Yes	No	2	0
Yes	Yes	Yes	No	No	Yes	Yes	1	0
Yes	Yes	Yes	No	No	Yes	Yes	8	0
No	Yes	Yes	No	No	No	No	0	0
Yes	Yes	Yes	No	No	No	No	0	0
Yes	Yes	Yes	No	No	No	No	0	0
Yes	Yes	Yes	No	No	No	No	0	0
Yes	Yes	Yes	No	No	Yes	No	12	6

For peer review only

No	No	Yes	No	No	Yes	No	0	0
Yes	Yes	Yes	No	No	No	No	0	0
Yes	Yes	Yes	No	Yes	Yes	Yes	0	0
Yes	Yes	Yes	No	No	No	No	0	0
Yes	No	Yes	No	No	Yes	Yes	0	0
Yes	Yes	Yes	No	No	No	Yes	0	0
No	Yes	Yes	Yes	No	Yes	No	0	0
Yes	Yes	Yes	No	No	Yes	Yes	5	0
Yes	Yes	Yes	No	No	No	No	0	0
Yes	Yes	No	No	No	No	No	0	0
Yes	Yes	Yes	No	No	Yes	Yes	10	0
Yes	Yes	Yes	No	No	Yes	Yes	2	0
Yes	Yes	Yes	No	No	Yes	No	8	0
No	Yes	Yes	No	No	Yes	No	0	0
Yes	Yes	Yes	No	No	Yes	Yes	6	3
Yes	Yes	Yes	No	No	Yes	No	5	0
Yes	Yes	Yes	No	No	No	Yes	0	0
Yes	Yes	No	No	No	No	No	0	0
Yes	Yes	Yes	No	No	No	Yes	0	0
Yes	Yes	Yes	No	No	No	Yes	0	0
Yes	Yes	Yes	Yes	No	Yes	No	0	0
No	No	No	No	No	No	No	0	0
Yes	Yes	Yes	No	No	No	Yes	0	0
Yes	Yes	Yes	No	No	Yes	No	0	0
Yes	Yes	Yes	No	No	Yes	Yes	3	0
Yes	Yes	Yes	No	No	No	No	0	0
Yes	Yes	Yes	No	No	Yes	No	2	0
Yes	Yes	Yes	No	No	No	Yes	0	0
Yes	Yes	Yes	No	No	Yes	Yes	0	0
Yes	Yes	Yes	No	No	Yes	No	4	0
Yes	Yes	Yes	No	No	No	Yes	0	0
Yes	Yes	Yes	No	No	Yes	Yes	0	0
Yes	Yes	Yes	No	No	Yes	Yes	7	0
No	Yes	Yes	No	No	No	No	0	0
Yes	Yes	Yes	No	No	Yes	Yes	0	0
Yes	Yes	Yes	No	No	Yes	Yes	5	0
Yes	Yes	Yes	No	No	No	No	0	0
Yes	Yes	No	No	No	Yes	No	4	0
Yes	Yes	Yes	No	No	Yes	Yes	5	0
Yes	Yes	Yes	No	No	Yes	Yes	0	0
Yes	Yes	Yes	No	No	Yes	Yes	0	0
No	Yes	No	No	No	No	No	0	0
Yes	Yes	Yes	No	No	Yes	No	50	0
Yes	Yes	Yes	No	No	No	Yes	0	0
Yes	Yes	Yes	No	No	No	Yes	0	0
Yes	Yes	Yes	No	No	No	No	0	0
No	Yes	Yes	No	No	Yes	No	0	0
Yes	Yes	Yes	No	No	Yes	Yes	0	0
Yes	Yes	Yes	No	No	Yes	Yes	5	0
Yes	Yes	Yes	No	No	Yes	Yes	3	5
Yes	No	Yes	No	No	Yes	No	3	2
Yes	Yes	Yes	No	No	Yes	Yes	0	0

For peer review only

Yes	Yes	Yes	Yes	No	No	Yes	0	0
Yes	Yes	Yes	No	No	Yes	Yes	12	0
Yes	Yes	Yes	No	No	Yes	No	1	0
Yes	Yes	Yes	No	No	Yes	Yes	0	4
Yes	Yes	Yes	No	No	Yes	Yes	3	0
Yes	Yes	Yes	No	No	Yes	Yes	2	36
Yes	Yes	Yes	No	No	Yes	Yes	3	2
Yes	Yes	Yes	No	No	Yes	Yes	0	0
Yes	Yes	No	No	No	Yes	No	0	0
Yes	Yes	Yes	No	No	Yes	Yes	6	0
Yes	Yes	Yes	No	No	Yes	No	0	0
Yes	Yes	Yes	No	No	No	No	0	0
No	Yes	Yes	No	No	No	No	0	0
Yes	Yes	Yes	No	No	Yes	Yes	3	0
Yes	Yes	Yes	No	Yes	Yes	Yes	0	0
Yes	Yes	Yes	No	No	No	No	0	0
Yes	Yes	Yes	No	No	No	Yes	0	0
Yes	Yes	Yes	No	No	Yes	No	2	0
No	No	Yes	No	No	Yes	Yes	0	0
Yes	Yes	Yes	No	No	Yes	No	2	0
Yes	Yes	Yes	No	No	No	Yes	0	0
Yes	Yes	Yes	Yes	No	Yes	Yes	3	2
Yes	Yes	Yes	No	No	Yes	Yes	2	0
Yes	Yes	Yes	No	No	No	Yes	0	0
No	No	No	No	No	No	No	0	0
Yes	Yes	Yes	No	No	Yes	Yes	1	0
Yes	Yes	Yes	No	No	Yes	Yes	6	0
Yes	Yes	Yes	No	No	No	No	0	0
Yes	Yes	Yes	No	No	Yes	No	2	0
Yes	Yes	Yes	No	No	Yes	Yes	0	0
Yes	Yes	Yes	No	No	Yes	No	8	2
Yes	Yes	Yes	No	No	Yes	Yes	150	0
Yes	Yes	Yes	No	No	Yes	Yes	5	0
Yes	Yes	Yes	No	No	Yes	Yes	4	10
No	Yes	Yes	No	No	Yes	Yes	0	2
Yes	Yes	Yes	No	No	No	No	0	0
Yes	Yes	Yes	No	No	Yes	Yes	4	0
Yes	Yes	No	No	No	No	No	0	0
Yes	Yes	Yes	No	No	No	No	0	0
Yes	No	Yes	No	No	Yes	Yes	0	0
Yes	Yes	Yes	No	No	No	Yes	0	0
No	Yes	Yes	No	No	Yes	No	1	0
Yes	Yes	Yes	No	No	Yes	No	3	0
Yes	Yes	Yes	No	No	Yes	Yes	0	0
Yes	Yes	Yes	No	No	Yes	No	0	0
Yes	Yes	Yes	No	No	Yes	Yes	7	0
No	No	Yes	No	No	Yes	No	0	0
No	No	Yes	No	Yes	Yes	Yes	3	0
Yes	Yes	Yes	No	No	No	No	0	0
Yes	Yes	Yes	No	No	Yes	Yes	0	28
Yes	Yes	Yes	No	No	Yes	Yes	5	2
Yes	Yes	Yes	No	No	No	No	0	0

For peer review only

Yes	Yes	Yes	No	No	Yes	No	0	0
Yes	Yes	Yes	No	No	Yes	Yes	8	0
Yes	Yes	Yes	No	No	Yes	Yes	0	0
Yes	Yes	Yes	No	No	Yes	No	1	0
Yes	Yes	Yes	No	No	No	No	0	0
Yes	No	Yes	No	No	Yes	No	5	0
Yes	Yes	Yes	No	No	No	No	0	0
Yes	No	Yes	No	No	Yes	No	5	0
Yes	Yes	Yes	No	No	No	No	0	0
Yes	Yes	Yes	No	No	No	No	0	0
Yes	Yes	Yes	No	No	No	Yes	0	0
Yes	Yes	Yes	No	No	Yes	Yes	16	0
Yes	Yes	Yes	No	No	Yes	Yes	0	0
Yes	Yes	Yes	No	No	No	No	0	0
Yes	Yes	No	No	No	Yes	No	8	0
Yes	Yes	Yes	No	No	No	Yes	0	0
Yes	Yes	Yes	No	No	Yes	Yes	3	0
Yes	Yes	Yes	No	No	No	No	0	0
Yes	Yes	Yes	No	No	No	No	0	0
Yes	Yes	Yes	Yes	Yes	Yes	Yes	25	0
No	No	No	No	No	No	No	0	0
Yes	Yes	Yes	No	No	Yes	No	0	0
Yes	Yes	No	No	No	No	No	0	0
Yes	Yes	Yes	No	No	Yes	Yes	200	0
Yes	Yes	Yes	No	No	Yes	Yes	0	0
Yes	Yes	Yes	No	No	Yes	Yes	2	0
Yes	Yes	No	No	No	No	Yes	0	0
Yes	Yes	Yes	No	No	Yes	Yes	2	2
No	No	No	No	No	Yes	No	9	2
Yes	Yes	Yes	No	No	Yes	No	1	0
Yes	Yes	Yes	No	No	No	Yes	0	0
Yes	Yes	Yes	No	No	No	Yes	0	0
Yes	Yes	Yes	No	No	No	Yes	0	0
Yes	Yes	Yes	No	No	Yes	No	2	0
Yes	Yes	Yes	No	No	No	No	0	0
Yes	Yes	Yes	No	No	Yes	Yes	3	0
Yes	Yes	Yes	No	No	Yes	Yes	0	0
Yes	Yes	Yes	No	No	Yes	Yes	2	0
Yes	Yes	No	No	No	Yes	No	0	0
Yes	Yes	Yes	No	No	Yes	Yes	4	0
Yes	Yes	Yes	No	No	No	No	0	0
Yes	Yes	Yes	No	No	No	Yes	0	0
No	Yes	Yes	No	No	Yes	Yes	0	0
Yes	Yes	Yes	No	No	No	No	0	0
Yes	Yes	Yes	No	Yes	Yes	Yes	0	0
No	No	Yes	No	No	Yes	No	0	0
No	Yes	Yes	No	No	Yes	No	0	0
Yes	Yes	Yes	No	No	Yes	Yes	0	1
Yes	Yes	Yes	No	No	No	No	0	0
Yes	Yes	Yes	No	No	Yes	Yes	6	3
Yes	Yes	Yes	No	No	Yes	No	2	0
Yes	Yes	Yes	No	No	Yes	No	0	0

For peer review only

Yes	Yes	Yes	No	No	No	No	0	0
Yes	Yes	Yes	No	No	Yes	Yes	4	0
Yes	Yes	Yes	No	No	Yes	Yes	0	0
Yes	Yes	Yes	No	No	No	Yes	0	0
Yes	Yes	Yes	No	No	Yes	No	0	0
Yes	Yes	Yes	No	No	Yes	Yes	0	0
Yes	Yes	Yes	No	No	Yes	Yes	0	0
No	Yes	No	No	No	No	No	0	0
Yes	Yes	Yes	No	No	Yes	No	0	0
No	Yes	Yes	No	No	Yes	No	0	0
Yes	Yes	Yes	No	No	No	Yes	0	0
Yes	Yes	Yes	No	No	Yes	Yes	2	0
Yes	Yes	Yes	No	No	Yes	No	0	0
Yes	Yes	Yes	No	No	No	Yes	0	0
No	Yes	Yes	No	No	Yes	No	0	0
Yes	Yes	Yes	No	No	No	No	0	0
No	No	Yes	No	No	Yes	Yes	0	0
No	Yes	Yes	No	No	No	No	0	0
No	No	Yes	No	No	Yes	Yes	7	0
Yes	Yes	Yes	No	No	Yes	Yes	3	0
Yes	Yes	Yes	No	No	No	Yes	0	0
No	No	Yes	No	No	Yes	No	5	0
Yes	No	Yes	No	No	Yes	No	4	0
Yes	Yes	Yes	No	No	Yes	No	6	1
Yes	Yes	Yes	No	No	Yes	Yes	0	0
Yes	Yes	Yes	No	No	No	No	0	0
Yes	Yes	Yes	No	No	Yes	Yes	8	0
Yes	Yes	Yes	No	No	No	No	0	0
Yes	Yes	No	No	No	Yes	No	6	0
Yes	Yes	Yes	No	No	No	No	0	0
No	Yes	Yes	No	No	Yes	No	2	0
Yes	Yes	Yes	No	No	Yes	Yes	0	0
Yes	No	No	No	No	Yes	Yes	1	0
Yes	Yes	Yes	No	No	Yes	No	10	0
No	Yes	Yes	No	No	No	No	0	0
Yes	Yes	Yes	No	No	No	No	0	0
Yes	Yes	Yes	No	No	Yes	Yes	10	0
Yes	Yes	Yes	No	No	No	No	0	0
Yes	Yes	Yes	No	No	Yes	No	0	0
Yes	Yes	Yes	No	Yes	No	No	0	0
Yes	Yes	Yes	No	No	Yes	Yes	2	1
Yes	Yes	Yes	No	No	Yes	Yes	1	0
No	Yes	Yes	No	No	Yes	No	0	0
Yes	Yes	Yes	No	No	Yes	Yes	0	0
Yes	Yes	Yes	No	No	No	Yes	0	0
Yes	Yes	Yes	No	No	No	No	0	0
Yes	Yes	Yes	No	No	No	No	0	0
Yes	Yes	Yes	No	No	Yes	No	3	0
Yes	Yes	Yes	No	No	Yes	Yes	12	0
Yes	Yes	Yes	No	No	No	Yes	0	0
Yes	Yes	Yes	No	No	No	No	0	0
Yes	Yes	Yes	No	No	Yes	Yes	0	0

For peer review only

Yes	Yes	Yes	No	No	Yes	Yes	5	0
Yes	Yes	Yes	No	No	No	No	0	0
Yes	Yes	Yes	Yes	No	Yes	Yes	15	0
Yes	Yes	No	No	No	Yes	No	1	2
Yes	Yes	Yes	No	No	Yes	Yes	0	0
Yes	Yes	Yes	No	No	No	No	0	0
Yes	Yes	Yes	No	No	No	No	0	0
Yes	Yes	Yes	No	No	No	Yes	0	0
Yes	No	Yes	No	No	Yes	No	0	0
Yes	Yes	Yes	No	No	No	No	0	0
Yes	Yes	Yes	No	No	No	No	0	0
Yes	Yes	Yes	No	No	Yes	Yes	0	0
Yes	Yes	Yes	No	No	Yes	Yes	2	0
No	Yes	Yes	No	No	Yes	No	9	0
Yes	Yes	Yes	No	No	Yes	No	0	0
Yes	Yes	Yes	No	No	No	No	0	0
Yes	Yes	Yes	No	No	No	Yes	0	0
Yes	Yes	Yes	No	No	Yes	No	2	0
Yes	Yes	Yes	No	No	Yes	Yes	0	0
Yes	Yes	Yes	No	No	No	Yes	0	0
Yes	Yes	Yes	No	No	Yes	No	2	0
Yes	Yes	Yes	No	No	No	No	0	0
Yes	Yes	No	No	No	Yes	Yes	10	0
Yes	Yes	Yes	No	No	Yes	No	5	0
Yes	Yes	Yes	No	No	No	Yes	0	0
Yes	Yes	Yes	No	No	Yes	Yes	4	0
No	No	No	No	No	Yes	No	10	0
Yes	Yes	Yes	No	No	No	No	0	0
Yes	Yes	Yes	No	No	Yes	No	0	0
Yes	Yes	Yes	No	No	Yes	Yes	2	0
Yes	Yes	No	No	No	Yes	No	0	0
No	No	Yes	No	No	Yes	No	0	0
Yes	Yes	Yes	No	No	Yes	No	0	0
No	No	No	No	No	No	No	0	0
Yes	Yes	Yes	No	No	Yes	Yes	10	0
Yes	Yes	Yes	No	No	Yes	No	3	2
Yes	Yes	Yes	No	No	Yes	Yes	100	4
Yes	Yes	Yes	No	No	Yes	No	3	1
Yes	Yes	Yes	No	Yes	Yes	No	5	0
Yes	Yes	Yes	Yes	No	Yes	Yes	8	8
Yes	Yes	Yes	No	No	Yes	Yes	0	0
Yes	Yes	Yes	No	No	Yes	No	0	0
Yes	Yes	Yes	Yes	Yes	Yes	Yes	15	0
Yes	Yes	Yes	No	No	No	Yes	0	0
No	No	No	No	No	Yes	No	0	0
No	Yes	Yes	No	No	Yes	No	0	0
Yes	Yes	Yes	No	No	No	No	0	0
Yes	No	Yes	No	No	Yes	No	2	0
No	No	No	No	Yes	Yes	Yes	0	0
Yes	Yes	Yes	No	No	Yes	No	0	0
Yes	Yes	Yes	No	No	Yes	No	3	0
Yes	No	Yes	No	No	Yes	Yes	4	0

For peer review only

Yes	Yes	Yes	No	No	No	Yes	0	0
Yes	Yes	Yes	No	No	Yes	No	0	0
No	Yes	No	No	Yes	Yes	No	20	0
Yes	Yes	Yes	No	No	Yes	No	0	0
Yes	Yes	Yes	No	No	Yes	No	5	0
No	Yes	No	No	No	Yes	No	0	0
Yes	Yes	Yes	No	Yes	Yes	Yes	4	0
Yes	Yes	Yes	No	No	Yes	Yes	2	0
No	No	Yes	No	No	Yes	No	3	0
No	No	No	No	No	No	No	0	0
No	No	Yes	No	No	Yes	No	1	0
Yes	Yes	Yes	No	No	Yes	Yes	0	0
Yes	Yes	Yes	No	No	Yes	Yes	8	0
Yes	Yes	Yes	No	No	Yes	Yes	2	1
Yes	Yes	Yes	No	Yes	Yes	Yes	3	2
Yes	No	Yes	No	No	Yes	No	8	0
Yes	Yes	Yes	No	Yes	Yes	No	0	0
Yes	Yes	Yes	No	No	Yes	Yes	4	0
No	Yes	Yes	No	No	Yes	No	3	0
Yes	Yes	Yes	No	No	No	Yes	0	0
No	No	No	No	Yes	Yes	Yes	0	0
No	No	Yes	No	No	Yes	Yes	0	0
Yes	No	Yes	No	No	Yes	No	10	0
No	No	Yes	No	No	Yes	No	3	3
Yes	Yes	Yes	No	No	Yes	No	8	0
Yes	Yes	Yes	No	No	Yes	No	6	0
Yes	Yes	Yes	No	No	Yes	No	5	0
No	Yes	Yes	No	No	Yes	No	7	0
No	No	Yes	No	No	Yes	No	9	0
No	No	Yes	No	No	Yes	No	5	0
Yes	Yes	Yes	No	No	No	Yes	0	0
No	No	Yes	No	No	Yes	No	5	7
Yes	Yes	Yes	No	Yes	Yes	No	8	0
Yes	Yes	Yes	No	No	No	No	0	0
Yes	Yes	Yes	No	Yes	Yes	Yes	0	0
No	No	Yes	No	No	Yes	No	3	0
No	Yes	No	No	No	Yes	No	0	0
Yes	Yes	No	No	No	Yes	Yes	0	0
Yes	Yes	Yes	No	No	Yes	No	15	0
No	No	Yes	No	Yes	Yes	No	5	0
No	No	Yes	No	Yes	Yes	No	5	0
No	No	No	No	Yes	Yes	Yes	2	0
No	No	Yes	No	No	Yes	No	3	5
Yes	Yes	Yes	No	No	No	Yes	0	0
Yes	Yes	Yes	No	No	Yes	No	10	0
Yes	Yes	Yes	No	No	Yes	Yes	5	2
Yes	Yes	Yes	No	No	Yes	No	6	0
Yes	Yes	Yes	No	No	Yes	Yes	19	1
Yes	Yes	Yes	No	No	No	Yes	0	0
No	No	Yes	No	No	Yes	No	4	0
No	No	Yes	No	No	Yes	No	12	0
No	Yes	Yes	No	Yes	Yes	No	0	3

For peer review only

No	No	No	No	No	Yes	No	0	0
No	No	No	No	No	Yes	No	0	1
No	Yes	No	No	Yes	Yes	No	0	0
Yes	Yes	Yes	No	No	Yes	Yes	6	0
No	No	Yes	No	No	Yes	No	0	0
Yes	Yes	Yes	No	No	Yes	Yes	5	0
Yes	Yes	Yes	No	No	Yes	No	4	0
No	No	Yes	No	No	Yes	Yes	5	0
Yes	Yes	Yes	No	No	Yes	Yes	5	0
Yes	Yes	Yes	No	No	Yes	Yes	0	0
Yes	Yes	Yes	No	No	Yes	No	2	5
Yes	Yes	Yes	No	No	Yes	Yes	5	3
Yes	Yes	Yes	No	No	Yes	Yes	0	0
Yes	Yes	Yes	No	No	No	Yes	0	0
Yes	Yes	Yes	No	Yes	Yes	Yes	0	5
Yes	Yes	Yes	No	No	Yes	Yes	2	0
Yes	Yes	Yes	No	No	Yes	No	0	0
Yes	Yes	Yes	No	No	Yes	No	3	1
No	Yes	Yes	No	No	Yes	Yes	4	3
Yes	Yes	Yes	No	No	Yes	No	13	0
Yes	Yes	Yes	No	No	Yes	No	0	0
No	No	Yes	No	No	No	Yes	0	0
No	No	Yes	No	No	Yes	Yes	6	1
No	No	Yes	No	No	Yes	No	0	0
No	No	Yes	No	No	Yes	No	5	0
Yes	No	Yes	No	No	Yes	No	5	0
Yes	Yes	Yes	No	No	No	Yes	0	0
Yes	Yes	Yes	No	No	No	Yes	0	0
No	No	Yes	No	No	Yes	No	0	5
No	No	No	No	Yes	Yes	Yes	0	0
No	Yes	No	No	Yes	Yes	No	4	0
No	No	No	No	No	Yes	Yes	4	0
No	No	Yes	No	No	Yes	No	2	0
Yes	Yes	Yes	No	No	Yes	Yes	9	0
No	No	Yes	No	No	Yes	No	3	6
No	No	Yes	No	No	Yes	No	0	2
Yes	Yes	Yes	No	No	Yes	No	10	0
Yes	Yes	Yes	No	No	Yes	Yes	15	0
No	No	Yes	No	No	Yes	No	0	0
Yes	Yes	Yes	No	No	Yes	Yes	0	0
No	No	No	No	No	Yes	Yes	0	0
No	No	No	No	No	Yes	No	0	0
Yes	Yes	Yes	No	No	Yes	No	0	0
No	No	No	No	Yes	Yes	Yes	0	0
No	Yes	Yes	No	No	Yes	No	0	0
Yes	Yes	Yes	No	No	Yes	Yes	0	0
Yes	Yes	Yes	No	No	Yes	No	4	0
Yes	Yes	Yes	No	No	Yes	Yes	9	0
Yes	Yes	Yes	No	No	Yes	No	4	0
No	Yes	No	No	No	Yes	Yes	4	0
No	No	Yes	No	No	Yes	Yes	10	0
Yes	Yes	Yes	No	No	No	Yes	0	0

For peer review only

Yes	Yes	Yes	No	No	Yes	No	3	0
Yes	Yes	Yes	No	No	Yes	Yes	2	0
Yes	No	Yes	No	No	Yes	No	6	0
No	Yes	Yes	No	No	Yes	No	0	0
Yes	Yes	Yes	No	No	Yes	Yes	0	1
No	No	Yes	No	No	Yes	No	6	0
Yes	Yes	Yes	No	No	Yes	No	0	0
Yes	Yes	Yes	No	No	Yes	No	0	0
Yes	Yes	Yes	No	No	Yes	Yes	4	0
Yes	Yes	Yes	No	No	Yes	Yes	0	0
No	No	No	No	No	Yes	Yes	5	0
Yes	Yes	Yes	No	Yes	No	Yes	0	0
No	Yes	Yes	No	Yes	Yes	No	3	0
Yes	Yes	Yes	No	No	Yes	Yes	0	0
Yes	Yes	Yes	No	No	Yes	No	5	4
Yes	Yes	Yes	No	No	Yes	Yes	0	3
Yes	Yes	Yes	No	No	Yes	No	6	0
Yes	Yes	No	No	No	No	No	0	0
No	No	Yes	No	No	No	Yes	0	0
Yes	Yes	Yes	No	No	Yes	Yes	1	0
Yes	Yes	Yes	No	No	Yes	No	5	0
No	No	Yes	No	No	Yes	No	0	0
Yes	Yes	Yes	No	No	Yes	No	0	0
Yes	Yes	Yes	No	No	No	No	0	0
Yes	Yes	Yes	No	No	Yes	No	5	0
No	No	Yes	No	No	Yes	No	6	0
Yes	Yes	Yes	No	No	Yes	No	3	0
No	No	No	No	No	No	Yes	0	0
Yes	Yes	Yes	No	No	Yes	Yes	14	12
Yes	No	Yes	No	No	Yes	Yes	0	0
Yes	Yes	Yes	No	No	Yes	No	6	0
No	No	Yes	No	No	Yes	No	7	0
No	No	Yes	No	No	Yes	No	5	0
Yes	Yes	No	No	No	Yes	No	5	0
No	No	Yes	No	No	Yes	Yes	2	5
No	Yes	No	No	Yes	No	No	0	0
No	No	Yes	No	No	No	No	0	0
Yes	No	Yes	No	No	Yes	No	2	0
Yes	Yes	Yes	No	No	Yes	Yes	0	0
No	No	No	No	No	Yes	Yes	0	19
No	No	Yes	No	No	Yes	No	2	3
Yes	Yes	Yes	No	No	Yes	No	10	0
Yes	Yes	Yes	No	No	No	No	0	0
Yes	Yes	Yes	No	No	Yes	Yes	0	0
Yes	Yes	Yes	No	No	Yes	No	0	0
Yes	Yes	Yes	No	No	Yes	Yes	9	0
No	No	Yes	No	No	Yes	No	6	0
No	Yes	Yes	No	No	Yes	Yes	11	0
Yes	Yes	Yes	No	No	Yes	No	0	2
Yes	Yes	Yes	No	No	No	Yes	0	0
Yes	Yes	Yes	No	No	Yes	No	0	10
No	No	Yes	No	Yes	Yes	No	3	0

For peer review only

Yes	No	Yes	No	No	Yes	Yes	7	2
Yes	Yes	Yes	No	No	Yes	No	5	3
No	No	No	No	Yes	Yes	No	0	0
No	Yes	No	No	Yes	Yes	No	0	2
No	No	No	No	Yes	Yes	Yes	0	0
No	No	No	No	Yes	Yes	No	3	0
Yes	Yes	Yes	No	No	No	Yes	0	0
No	Yes	Yes	No	Yes	Yes	No	35	0
No	No	No	No	Yes	No	No	0	0
No	No	Yes	No	No	Yes	No	5	3
Yes	Yes	Yes	No	No	Yes	Yes	1	0
No	No	Yes	No	No	Yes	No	0	0
Yes	Yes	Yes	No	No	Yes	Yes	8	0
Yes	Yes	Yes	No	No	Yes	Yes	4	0
No	No	Yes	No	No	Yes	Yes	0	2
No	No	No	No	No	Yes	No	7	0
Yes	Yes	Yes	No	No	No	Yes	0	0
No	No	Yes	No	No	Yes	Yes	10	0
No	No	Yes	No	No	Yes	No	3	0
Yes	Yes	Yes	No	No	Yes	Yes	2	0
Yes	Yes	No	No	No	Yes	No	0	0
No	No	Yes	No	No	Yes	Yes	5	0
Yes	Yes	Yes	No	Yes	Yes	Yes	0	0
Yes	Yes	Yes	No	No	No	No	0	0
No	No	Yes	No	No	Yes	No	0	0
Yes	Yes	Yes	No	No	Yes	Yes	0	4
Yes	Yes	Yes	No	No	Yes	No	0	0
No	No	Yes	No	No	Yes	No	4	3
No	No	No	No	No	Yes	No	0	0
No	No	Yes	No	No	No	Yes	0	0
No	Yes	Yes	No	No	Yes	Yes	4	2
No	No	No	No	No	Yes	Yes	0	0
Yes	Yes	Yes	No	No	Yes	No	0	0
No	No	No	No	No	No	No	0	0
No	Yes	No	No	No	Yes	No	6	0
No	No	No	No	No	Yes	Yes	0	0
Yes	Yes	Yes	No	No	No	No	0	0
No	No	Yes	No	Yes	Yes	No	5	0
Yes	Yes	Yes	No	No	Yes	No	0	2
Yes	No	No	No	No	Yes	No	0	0
Yes	Yes	Yes	No	No	Yes	No	2	0
Yes	Yes	Yes	No	No	Yes	No	15	0
Yes	Yes	Yes	No	No	No	Yes	0	0
No	No	No	No	No	Yes	No	10	0
No	No	Yes	No	No	Yes	Yes	5	0
No	No	No	No	No	Yes	Yes	6	3
No	No	No	No	No	Yes	No	0	0
Yes	Yes	Yes	No	No	Yes	Yes	20	15
Yes	Yes	Yes	No	No	Yes	Yes	5	4
Yes	Yes	Yes	No	No	Yes	No	5	0
No	Yes	Yes	No	No	Yes	Yes	6	0
No	No	Yes	No	Yes	Yes	Yes	4	0

For peer review only

Yes	Yes	Yes	No	No	No	No	0	0
No	No	No	No	No	Yes	No	5	6
Yes	No	Yes	No	No	Yes	No	4	0
No	No	No	No	No	No	Yes	0	0
Yes	Yes	Yes	No	No	No	No	0	0
No	No	Yes	No	No	Yes	No	0	0
No	No	No	No	Yes	Yes	Yes	0	0
No	Yes	Yes	No	No	Yes	Yes	0	0
Yes	No	Yes	No	No	Yes	No	0	0
No	Yes	Yes	No	No	Yes	No	10	0
No	No	No	No	Yes	Yes	No	2	0
No	No	Yes	No	Yes	Yes	No	0	0
Yes	No	No	No	No	Yes	No	3	0
Yes	Yes	Yes	No	No	Yes	No	10	0
Yes	Yes	Yes	No	No	Yes	Yes	6	0
Yes	Yes	Yes	No	No	Yes	Yes	10	0
No	Yes	Yes	Yes	No	Yes	No	0	6
Yes	Yes	Yes	No	No	Yes	No	6	0
Yes	Yes	Yes	No	No	Yes	No	0	4
No	No	No	No	No	Yes	No	0	0
Yes	Yes	Yes	No	No	No	Yes	0	0
Yes	Yes	Yes	No	No	Yes	Yes	5	0
Yes	Yes	Yes	No	No	Yes	Yes	10	0
No	No	No	No	No	Yes	Yes	0	0
No	No	No	No	Yes	Yes	Yes	0	0
No	No	No	No	Yes	Yes	Yes	0	0
No	No	Yes	No	No	Yes	No	8	10
Yes	Yes	No	No	No	Yes	No	11	0
No	No	Yes	No	No	Yes	No	5	0
Yes	Yes	Yes	No	No	Yes	No	0	0
Yes	Yes	Yes	No	No	Yes	No	2	4
Yes	Yes	Yes	No	No	Yes	No	3	2
No	No	Yes	No	Yes	Yes	No	5	0
No	No	No	No	Yes	Yes	No	2	0
No	Yes	Yes	No	No	Yes	No	7	3
Yes	Yes	Yes	No	No	Yes	Yes	6	4
Yes	Yes	No	No	No	No	No	0	0
No	No	Yes	No	No	Yes	Yes	0	0
Yes	Yes	No	No	No	Yes	No	4	0
Yes	Yes	Yes	No	No	Yes	No	4	0
No	No	Yes	No	No	No	Yes	0	0
No	No	No	No	No	No	Yes	0	0
No	Yes	Yes	No	No	No	No	0	0
Yes	No	Yes	No	No	No	Yes	0	0
No	Yes	No	No	Yes	No	No	0	0
Yes	Yes	Yes	No	No	Yes	No	3	6
No	No	Yes	No	No	Yes	No	0	0
No	No	Yes	No	Yes	No	No	0	0
No	No	Yes	No	No	No	Yes	0	0
No	No	Yes	No	No	Yes	Yes	4	0
No	No	No	No	No	Yes	No	0	0
Yes	Yes	Yes	No	No	No	Yes	0	0

For peer review only

Yes	Yes	Yes	No	No	Yes	Yes	5	2
Yes	Yes	Yes	No	No	Yes	No	0	4
No	No	Yes	No	No	Yes	Yes	2	0
Yes	Yes	Yes	No	No	Yes	No	4	0
Yes	Yes	Yes	No	No	Yes	No	3	0
No	No	No	No	No	Yes	No	10	3
No	No	Yes	No	No	Yes	Yes	0	0
Yes	Yes	Yes	No	No	Yes	No	3	0
Yes	Yes	Yes	No	No	Yes	Yes	0	0
No	No	Yes	No	No	Yes	No	0	0
Yes	Yes	Yes	No	No	Yes	Yes	0	3
Yes	Yes	No	No	No	Yes	No	3	0
No	No	Yes	No	No	Yes	No	3	0
Yes	Yes	Yes	No	No	Yes	No	12	0
No	Yes	No	No	No	Yes	No	2	1
Yes	Yes	No	No	Yes	No	No	0	0
Yes	Yes	Yes	No	No	Yes	No	0	4
No	No	Yes	No	No	Yes	No	5	3
Yes	Yes	Yes	No	No	Yes	No	0	1
Yes	Yes	Yes	No	No	Yes	No	12	3
Yes	Yes	Yes	No	No	Yes	No	5	0
Yes	Yes	Yes	No	No	No	Yes	0	0
No	No	Yes	No	No	No	No	0	0
No	Yes	Yes	No	No	Yes	No	1	0
No	No	Yes	No	No	Yes	Yes	9	0
No	No	Yes	No	Yes	Yes	No	10	0
Yes	Yes	Yes	No	No	Yes	Yes	0	0
Yes	Yes	Yes	No	Yes	Yes	No	1	2
Yes	Yes	Yes	No	No	Yes	No	3	0
Yes	Yes	Yes	No	No	No	No	0	0
Yes	Yes	Yes	No	No	Yes	No	4	0
No	No	Yes	No	No	Yes	No	4	0
Yes	Yes	Yes	No	No	Yes	No	6	0
Yes	Yes	Yes	No	No	Yes	Yes	4	0
Yes	Yes	Yes	No	No	No	No	0	0
No	No	Yes	No	No	Yes	Yes	5	0
No	No	Yes	No	No	Yes	No	10	0
No	No	Yes	No	No	Yes	No	5	0
No	Yes	Yes	No	No	Yes	No	0	0
No	No	Yes	No	No	Yes	Yes	3	0
Yes	Yes	Yes	No	Yes	No	Yes	0	0
No	No	Yes	No	No	Yes	Yes	0	0
Yes	No	Yes	No	No	Yes	No	4	0
Yes	Yes	Yes	No	No	Yes	No	0	0
Yes	Yes	Yes	No	No	Yes	No	2	5
Yes	Yes	Yes	No	No	Yes	Yes	2	0
Yes	Yes	Yes	No	No	Yes	Yes	6	0
No	Yes	No	No	Yes	No	No	0	0
Yes	Yes	Yes	No	No	No	No	0	0
No	No	Yes	No	No	Yes	Yes	5	3
No	Yes	Yes	No	No	Yes	No	3	0
No	Yes	Yes	No	No	Yes	Yes	3	0

For peer review only

No	No	Yes	No	Yes	Yes	No	3	0
No	No	Yes	No	Yes	Yes	No	0	2
No	No	Yes	No	Yes	Yes	No	2	0
Yes	No	Yes	No	No	Yes	Yes	5	0
Yes	No	No	No	No	Yes	No	4	0
No	No	Yes	No	No	Yes	Yes	5	0
Yes	Yes	Yes	No	No	Yes	Yes	2	3
Yes	Yes	Yes	No	No	Yes	No	2	0
No	No	Yes	No	No	Yes	Yes	5	0
No	No	No	No	No	No	No	0	0
Yes	Yes	Yes	No	No	Yes	No	6	0
No	No	Yes	Yes	No	Yes	No	0	0
Yes	Yes	Yes	No	No	Yes	Yes	3	0
No	No	Yes	No	No	Yes	No	0	1
Yes	Yes	Yes	No	No	Yes	Yes	4	0
No	No	Yes	No	No	Yes	No	6	0
No	No	Yes	No	No	Yes	No	2	0
Yes	Yes	Yes	No	No	No	No	0	0
Yes	Yes	Yes	No	No	Yes	Yes	1	0
Yes	Yes	Yes	No	No	Yes	Yes	8	4
No	Yes	Yes	No	No	Yes	No	3	3
Yes	Yes	Yes	No	No	No	No	0	0
Yes	Yes	Yes	No	No	Yes	No	0	0
Yes	Yes	Yes	No	No	Yes	Yes	0	0
No	No	Yes	No	No	Yes	No	10	0
No	No	Yes	No	Yes	Yes	No	3	0
No	No	Yes	No	No	Yes	No	0	0
No	No	No	No	No	Yes	No	2	0
Yes	Yes	Yes	No	No	No	No	0	0
Yes	Yes	Yes	No	Yes	Yes	No	0	0
No	No	No	No	No	Yes	Yes	8	3
Yes	Yes	Yes	No	No	Yes	No	12	11
Yes	Yes	Yes	No	No	Yes	No	1	0
Yes	Yes	Yes	No	No	No	Yes	0	0
No	No	No	No	No	No	No	0	0
Yes	Yes	Yes	No	No	Yes	No	0	2
Yes	Yes	Yes	No	No	Yes	Yes	3	9
Yes	Yes	Yes	No	No	Yes	No	0	0
No	No	Yes	No	No	Yes	No	6	0
Yes	Yes	Yes	No	No	Yes	Yes	2	2
Yes	Yes	Yes	No	No	Yes	Yes	2	0
Yes	Yes	Yes	No	No	No	No	0	0
No	No	Yes	No	No	Yes	No	6	0
Yes	Yes	Yes	No	No	Yes	No	3	0
No	Yes	Yes	No	No	Yes	No	0	0
No	No	Yes	No	No	Yes	Yes	0	8
Yes	Yes	Yes	No	No	Yes	No	0	0
Yes	Yes	Yes	No	No	Yes	Yes	0	0
Yes	No	Yes	No	No	Yes	No	8	0
No	No	No	No	Yes	Yes	Yes	0	0
No	No	No	No	Yes	Yes	No	0	0
Yes	Yes	Yes	No	No	Yes	Yes	4	0

For peer review only

Yes	Yes	No	No	No	Yes	No	4	0
No	No	Yes	No	No	Yes	No	4	0
No	No	Yes	No	No	Yes	No	6	0
Yes	Yes	Yes	No	No	No	Yes	0	0
No	Yes	Yes	No	Yes	Yes	No	10	0
Yes	No	Yes	No	No	No	No	0	0
Yes	Yes	Yes	No	No	No	No	0	0
No	No	Yes	No	No	Yes	Yes	4	0
Yes	No	Yes	No	No	Yes	No	6	0
No	No	No	No	No	Yes	No	5	0
No	No	No	No	Yes	Yes	Yes	0	0
No	No	Yes	No	Yes	Yes	No	4	1
No	No	Yes	No	No	Yes	No	4	0
Yes	No	Yes	No	No	Yes	Yes	3	2
No	No	No	No	No	Yes	No	2	2
No	No	Yes	No	No	Yes	No	13	0
Yes	Yes	Yes	No	No	No	No	0	0
No	No	No	No	Yes	Yes	No	0	0
Yes	Yes	Yes	No	No	Yes	No	10	0
Yes	Yes	Yes	No	No	No	Yes	0	0
Yes	Yes	Yes	No	Yes	Yes	Yes	4	0
Yes	Yes	Yes	No	No	No	Yes	0	0
Yes	Yes	Yes	No	No	Yes	Yes	0	0
No	No	No	No	No	Yes	No	0	0
Yes	Yes	Yes	No	No	Yes	No	3	0
No	No	Yes	No	No	Yes	No	0	0
Yes	Yes	Yes	No	No	No	Yes	0	0
No	No	No	No	No	Yes	No	0	0
Yes	Yes	Yes	No	No	Yes	Yes	0	0
No	No	No	No	No	Yes	No	25	0
Yes	Yes	Yes	No	Yes	Yes	Yes	2	0
No	No	Yes	No	No	Yes	No	3	0
Yes	Yes	Yes	No	No	Yes	Yes	8	0
Yes	Yes	Yes	No	No	Yes	No	0	0
No	No	Yes	No	No	Yes	No	4	0
No	No	No	No	No	Yes	No	0	0
Yes	Yes	Yes	No	No	No	Yes	0	0
No	No	Yes	No	No	Yes	No	5	0
No	No	Yes	No	No	Yes	No	0	0
No	Yes	Yes	No	No	No	Yes	0	0
No	No	No	No	No	Yes	No	0	0
No	No	No	No	No	No	No	0	0
No	No	No	No	No	Yes	No	0	0
Yes	Yes	Yes	No	No	Yes	No	0	0
No	No	Yes	No	No	Yes	Yes	0	0
Yes	Yes	Yes	No	No	Yes	Yes	3	0
Yes	Yes	Yes	No	No	Yes	Yes	0	0
No	No	No	No	Yes	Yes	No	3	0
Yes	Yes	Yes	No	No	Yes	Yes	5	0
No	No	Yes	No	No	Yes	No	0	0
No	No	No	No	No	Yes	No	3	0
Yes	Yes	Yes	No	No	Yes	No	8	0

For peer review only

Yes	Yes	Yes	No	No	Yes	No	2	0
Yes	Yes	Yes	No	No	Yes	Yes	5	0
No	No	No	No	No	Yes	No	0	0
No	Yes	Yes	No	No	Yes	No	3	0
No	No	No	No	No	Yes	No	5	0
Yes	No	Yes	No	No	Yes	No	8	0
No	No	No	No	No	Yes	No	10	5
Yes	Yes	Yes	No	No	Yes	Yes	0	0
Yes	Yes	Yes	No	No	Yes	No	3	8
No	No	No	No	No	Yes	No	2	0
Yes	Yes	Yes	No	No	Yes	Yes	0	0
Yes	No	Yes	No	No	Yes	No	7	0
Yes	No	Yes	No	No	Yes	Yes	0	0
No	No	No	No	No	Yes	No	0	0
No	Yes	Yes	No	No	Yes	No	0	4
Yes	Yes	Yes	No	Yes	Yes	Yes	0	0
No	No	Yes	No	No	Yes	Yes	2	0
No	No	Yes	No	No	Yes	No	1	0
Yes	Yes	Yes	No	No	Yes	No	4	0
No	No	No	No	No	Yes	No	0	0
No	No	No	No	Yes	Yes	No	0	0
Yes	No	No	No	No	Yes	Yes	0	0
Yes	Yes	Yes	No	No	No	No	0	0
No	No	No	No	No	Yes	No	4	0
No	No	Yes	No	No	Yes	No	0	0
Yes	Yes	Yes	No	No	Yes	Yes	6	0
No	No	Yes	No	No	Yes	No	5	2
No	No	No	No	No	Yes	No	4	0
No	No	Yes	No	No	Yes	No	3	0
No	Yes	Yes	No	No	Yes	No	0	0
Yes	Yes	No	No	No	Yes	Yes	0	0
No	No	No	No	No	No	No	0	0
Yes	Yes	Yes	No	No	Yes	No	4	0
No	No	Yes	No	No	Yes	No	7	0
No	No	Yes	No	No	Yes	No	0	0
No	No	No	No	No	Yes	No	5	0
Yes	Yes	Yes	No	No	No	Yes	0	0
No	No	No	No	No	No	No	0	0
No	No	Yes	No	Yes	Yes	No	3	0
Yes	Yes	Yes	No	No	Yes	Yes	12	0
Yes	Yes	Yes	No	No	Yes	Yes	12	0
Yes	Yes	Yes	No	No	Yes	Yes	4	0
Yes	Yes	Yes	No	No	Yes	Yes	5	2
No	No	Yes	No	No	Yes	No	0	0
Yes	No	Yes	No	No	Yes	No	17	0
No	No	No	No	No	No	No	0	0
Yes	Yes	Yes	No	No	Yes	Yes	3	0
No	No	No	No	No	Yes	No	4	0
Yes	Yes	Yes	No	No	No	Yes	0	0
Yes	No	Yes	No	No	Yes	Yes	2	0
Yes	Yes	Yes	No	No	Yes	Yes	6	8
No	No	No	No	No	Yes	No	0	0

For peer review only

Yes	Yes	Yes	No	No	Yes	No	0	0
No	No	No	No	No	Yes	Yes	0	0
Yes	Yes	Yes	No	No	No	No	0	0
No	No	Yes	No	No	Yes	No	0	0
Yes	Yes	Yes	No	No	Yes	Yes	0	0
No	No	Yes	No	No	Yes	No	2	0
Yes	Yes	Yes	No	No	No	No	0	0
No	No	No	No	No	Yes	No	0	0
No	No	No	No	No	Yes	No	0	0
No	No	Yes	No	No	Yes	No	0	0
Yes	No	Yes	No	No	Yes	Yes	5	0
No	No	No	No	No	Yes	No	10	0
Yes	Yes	Yes	Yes	No	No	No	0	0
Yes	Yes	Yes	No	No	Yes	Yes	0	0
Yes	Yes	Yes	No	No	Yes	Yes	2	0
No	No	Yes	Yes	No	No	No	0	0
Yes	Yes	Yes	No	No	Yes	Yes	5	0
No	Yes	Yes	No	No	Yes	No	8	0
No	No	Yes	No	No	Yes	No	0	0
Yes	Yes	Yes	No	No	Yes	Yes	3	0
Yes	Yes	Yes	No	No	No	No	0	0
No	No	Yes	No	No	Yes	No	2	0
Yes	Yes	Yes	No	No	No	Yes	0	0
Yes	Yes	Yes	Yes	No	No	No	0	0
Yes	Yes	Yes	No	No	Yes	No	0	0
No	Yes	Yes	No	No	Yes	No	0	0
Yes	Yes	Yes	No	No	Yes	Yes	0	0
Yes	No	Yes	No	No	Yes	Yes	3	0
No	Yes	Yes	No	No	Yes	Yes	8	0
Yes	Yes	Yes	No	No	Yes	Yes	0	0
Yes	Yes	No	No	No	Yes	No	2	3
No	No	No	No	No	Yes	No	8	4
No	Yes	Yes	No	No	Yes	No	0	0
Yes	Yes	Yes	No	No	Yes	No	4	0
No	Yes	Yes	No	No	Yes	No	10	0
Yes	Yes	Yes	No	No	Yes	No	0	0
No	Yes	Yes	No	No	Yes	No	10	0
No	Yes	Yes	No	No	Yes	No	0	0
Yes	Yes	Yes	No	No	Yes	Yes	5	0
No	No	No	No	No	Yes	No	3	0
No	No	Yes	No	No	Yes	No	0	0
No	No	Yes	No	No	Yes	Yes	5	0
No	No	Yes	No	No	Yes	No	10	0
No	No	No	No	No	Yes	No	10	0
No	No	Yes	No	No	Yes	No	0	0
No	No	No	No	No	Yes	Yes	1	0
Yes	Yes	Yes	No	No	Yes	Yes	7	0
No	No	Yes	No	No	Yes	Yes	0	0
No	No	Yes	No	No	Yes	No	0	0
No	No	Yes	No	No	Yes	No	2	0
No	No	Yes	No	No	Yes	No	0	0
No	Yes	Yes	No	No	Yes	No	1	0

For peer review only

Yes	No	Yes	No	Yes	No	No	0	0
No	No	No	No	No	Yes	No	15	0
Yes	No	No	No	No	Yes	No	2	0
Yes	Yes	Yes	No	No	Yes	Yes	12	6
No	No	Yes	No	No	Yes	No	0	0
No	Yes	Yes	No	No	Yes	Yes	0	0
No	No	Yes	No	No	Yes	No	0	0
Yes	Yes	Yes	No	No	Yes	Yes	0	0
No	Yes	Yes	No	No	Yes	No	12	0
Yes	Yes	Yes	No	No	Yes	No	0	0
Yes	Yes	Yes	No	No	Yes	Yes	0	0
Yes	Yes	Yes	No	No	No	No	0	0
Yes	Yes	Yes	No	No	Yes	Yes	0	0
No	No	Yes	No	No	Yes	Yes	0	0
No	No	Yes	No	No	Yes	Yes	7	15
No	No	Yes	No	No	Yes	No	0	0
No	No	Yes	No	No	Yes	No	8	0
Yes	Yes	Yes	No	No	Yes	Yes	0	0
Yes	Yes	Yes	Yes	No	No	No	0	0
Yes	Yes	Yes	No	No	No	Yes	0	0
Yes	Yes	Yes	No	No	Yes	Yes	0	0
No	No	Yes	No	No	Yes	No	0	0
No	No	No	No	No	Yes	No	0	0
No	No	Yes	No	No	Yes	Yes	4	0
Yes	Yes	Yes	No	No	Yes	No	0	0
No	No	Yes	No	No	Yes	No	0	0
Yes	Yes	Yes	No	No	Yes	No	12	0
Yes	Yes	Yes	No	No	Yes	Yes	8	0
Yes	Yes	Yes	No	No	Yes	Yes	12	0
Yes	Yes	Yes	No	No	Yes	Yes	5	0
No	Yes	Yes	Yes	No	No	No	0	0
Yes	Yes	Yes	No	No	No	Yes	0	0
Yes	Yes	Yes	No	No	No	Yes	0	0
No	No	No	No	No	Yes	No	2	0
Yes	Yes	Yes	No	No	Yes	Yes	3	0
No	No	No	No	No	No	No	0	0
Yes	Yes	Yes	No	No	Yes	Yes	5	0
Yes	No	Yes	No	No	Yes	Yes	0	0
Yes	Yes	Yes	No	No	Yes	Yes	8	0
Yes	Yes	Yes	No	No	Yes	No	5	0
No	Yes	Yes	No	No	Yes	Yes	0	0
Yes	Yes	Yes	No	No	No	No	0	0
No	Yes	Yes	No	No	Yes	No	1	0
Yes	Yes	Yes	No	No	Yes	Yes	5	0
No	No	Yes	No	No	Yes	No	0	0
Yes	Yes	Yes	No	No	Yes	No	5	0
Yes	Yes	Yes	No	No	No	No	0	0
No	No	Yes	No	No	Yes	Yes	0	0
Yes	Yes	Yes	No	No	No	No	0	0
No	Yes	Yes	No	Yes	Yes	No	6	0
Yes	Yes	Yes	No	Yes	Yes	No	5	2
Yes	Yes	Yes	No	No	Yes	No	0	0

For peer review only

No	Yes	Yes	No	No	Yes	No	7	0
Yes	Yes	Yes	No	No	Yes	No	0	0
Yes	Yes	Yes	No	No	Yes	Yes	12	0
No	No	Yes	No	No	Yes	No	10	0
No	Yes	Yes	No	No	Yes	Yes	2	0
Yes	No	Yes	No	No	Yes	Yes	4	0
No	Yes	Yes	No	No	Yes	No	8	0
No	Yes	Yes	No	No	Yes	Yes	5	0
No	Yes	No	No	Yes	Yes	Yes	0	0
No	No	No	No	No	No	Yes	0	0
No	No	No	No	No	No	No	0	0
Yes	Yes	Yes	No	No	No	Yes	0	0
Yes	Yes	Yes	No	No	Yes	Yes	20	0
No	No	No	No	No	Yes	Yes	10	1
Yes	Yes	Yes	No	No	Yes	No	0	0
No	No	Yes	No	No	Yes	No	4	0
Yes	Yes	Yes	No	No	Yes	Yes	0	0
Yes	Yes	Yes	No	No	No	No	0	0
Yes	No	Yes	No	No	Yes	Yes	0	0
No	Yes	Yes	No	No	No	No	0	0
No	No	Yes	No	No	No	Yes	0	0
Yes	Yes	Yes	No	No	Yes	Yes	0	0
No	Yes	No	No	No	Yes	Yes	10	0
No	No	No	No	No	Yes	No	0	0
Yes	No	Yes	No	No	Yes	Yes	0	0
No	Yes	Yes	No	No	Yes	Yes	5	0
No	No	No	No	No	Yes	No	5	0
Yes	Yes	Yes	No	Yes	Yes	Yes	1	0
No	Yes	Yes	No	No	Yes	No	0	0
Yes	Yes	Yes	No	No	Yes	Yes	0	0
Yes	Yes	Yes	No	No	No	Yes	0	0
Yes	Yes	Yes	No	Yes	Yes	No	13	0
No	No	No	No	No	No	Yes	0	0
Yes	Yes	No	No	Yes	Yes	Yes	5	0
No	No	Yes	No	No	Yes	No	4	0
Yes	Yes	Yes	No	No	Yes	Yes	0	0
Yes	Yes	Yes	No	No	Yes	Yes	0	0
No	Yes	Yes	No	No	Yes	No	8	0
Yes	Yes	Yes	No	No	Yes	Yes	2	0
Yes	Yes	Yes	No	No	Yes	Yes	0	0
Yes	Yes	Yes	No	Yes	No	No	0	0
No	No	Yes	No	No	Yes	No	4	0
Yes	Yes	No	No	No	Yes	No	0	0
Yes	Yes	Yes	No	No	Yes	Yes	1	0
No	No	Yes	No	No	No	No	0	0
No	Yes	Yes	No	No	Yes	Yes	0	0
Yes	Yes	Yes	No	No	Yes	Yes	5	0
No	No	Yes	No	Yes	Yes	Yes	0	0
Yes	Yes	Yes	No	No	No	No	0	0
Yes	Yes	Yes	No	No	Yes	No	2	0
No	No	Yes	No	No	Yes	Yes	4	0
No	No	Yes	No	No	Yes	No	2	0

For peer review only

No	No	Yes	No	No	Yes	No	5	0
Yes	Yes	Yes	No	No	No	Yes	0	0
No	Yes	Yes	No	No	Yes	Yes	0	0
No	Yes	Yes	No	No	No	No	0	0
Yes	Yes	Yes	Yes	No	No	Yes	0	0
Yes	Yes	Yes	No	Yes	Yes	Yes	8	4
Yes	No	Yes	No	No	Yes	No	5	0
No	No	Yes	No	No	Yes	No	6	0
No	Yes	No	No	No	Yes	Yes	4	0
Yes	Yes	Yes	No	No	Yes	Yes	0	0
No	Yes	Yes	No	No	Yes	No	2	4
No	No	Yes	No	No	Yes	No	2	0
No	No	Yes	No	No	Yes	No	3	0
Yes	Yes	No	No	Yes	Yes	Yes	4	0
No	Yes	Yes	No	No	Yes	Yes	2	2
Yes	Yes	Yes	No	No	Yes	No	0	2
No	No	No	No	No	No	No	0	0
Yes	Yes	Yes	No	No	Yes	Yes	1	0
No	No	Yes	No	No	Yes	No	2	0
Yes	Yes	Yes	No	No	Yes	Yes	3	0
Yes	Yes	Yes	No	No	Yes	Yes	10	0
Yes	Yes	Yes	No	No	Yes	Yes	11	5
Yes	Yes	Yes	No	Yes	Yes	Yes	0	0
Yes	Yes	Yes	No	No	Yes	No	3	0
Yes	Yes	Yes	No	No	Yes	Yes	0	0
No	Yes	Yes	No	No	Yes	Yes	0	5
Yes	Yes	Yes	No	No	Yes	No	10	0
Yes	Yes	Yes	No	No	Yes	No	8	0
No	Yes	Yes	No	No	Yes	No	0	0
Yes	Yes	Yes	No	No	Yes	Yes	7	0
No	Yes	Yes	Yes	Yes	No	Yes	0	0
No	Yes	Yes	No	Yes	Yes	No	8	0
No	No	Yes	No	No	Yes	Yes	0	0
Yes	Yes	Yes	No	No	Yes	No	0	0
No	No	Yes	No	No	Yes	Yes	2	8
No	No	Yes	No	No	Yes	No	3	2
Yes	Yes	Yes	No	No	Yes	Yes	5	0
Yes	Yes	No	No	No	No	No	0	0
No	Yes	Yes	No	Yes	Yes	No	0	0
Yes	Yes	Yes	No	No	Yes	Yes	4	0
Yes	Yes	No	No	No	Yes	Yes	6	0
Yes	Yes	Yes	No	No	Yes	Yes	10	0
Yes	Yes	No	No	No	Yes	No	2	0
Yes	Yes	Yes	No	No	Yes	Yes	4	0
Yes	Yes	Yes	No	No	Yes	Yes	3	0
No	Yes	Yes	No	Yes	Yes	Yes	6	0
Yes	Yes	Yes	No	Yes	Yes	Yes	0	0
No	Yes	Yes	No	No	Yes	Yes	3	0
Yes	Yes	Yes	No	No	Yes	No	0	0
Yes	Yes	Yes	No	No	No	No	0	0
No	Yes	Yes	No	No	Yes	Yes	2	2
Yes	Yes	No	No	No	Yes	Yes	4	0

For peer review only

Yes	Yes	Yes	No	No	No	No	0	0
Yes	No	Yes	No	No	No	Yes	0	0
No	No	No	No	No	No	No	0	0
Yes	Yes	No	No	No	Yes	Yes	2	0
Yes	Yes	Yes	No	No	Yes	No	0	0
Yes	Yes	Yes	No	No	Yes	No	0	0
No	No	Yes	No	No	No	Yes	0	0
No	No	Yes	No	No	Yes	Yes	2	0
Yes	No	Yes	No	No	Yes	No	3	2
Yes	Yes	Yes	No	No	Yes	Yes	0	0
Yes	Yes	Yes	No	No	No	No	0	0
No	No	Yes	No	No	No	Yes	0	0
Yes	Yes	Yes	No	No	Yes	Yes	3	0
Yes	Yes	Yes	No	No	Yes	No	6	0
No	No	Yes	No	Yes	Yes	No	2	0
No	No	No	No	No	Yes	Yes	0	0
Yes	Yes	Yes	No	No	No	No	0	0
Yes	Yes	Yes	No	No	Yes	Yes	2	0
No	No	Yes	No	No	Yes	No	10	0
Yes	Yes	Yes	No	Yes	Yes	Yes	0	1
No	No	Yes	No	No	No	Yes	0	0
No	Yes	Yes	No	No	Yes	Yes	3	3
No	Yes	Yes	No	No	Yes	Yes	0	0
No	Yes	Yes	No	No	Yes	Yes	0	0
Yes	Yes	Yes	No	No	Yes	No	12	14
Yes	Yes	Yes	No	No	Yes	Yes	6	0
Yes	Yes	Yes	No	No	Yes	No	1	0
Yes	Yes	Yes	No	No	No	Yes	0	0
Yes	Yes	Yes	Yes	No	Yes	Yes	10	0
No	No	No	No	No	No	No	0	0
No	No	Yes	No	No	Yes	No	8	4
Yes	Yes	Yes	Yes	No	No	Yes	0	0
No	No	Yes	No	No	No	Yes	0	0
No	Yes	Yes	No	Yes	Yes	No	3	2
Yes	Yes	Yes	No	No	No	No	0	0
Yes	Yes	Yes	No	No	No	No	0	0
Yes	Yes	Yes	No	No	Yes	Yes	5	0
No	Yes	Yes	No	Yes	Yes	No	5	0
No	Yes	Yes	No	Yes	Yes	Yes	2	0
No	No	Yes	No	No	Yes	No	3	0
Yes	No	Yes	No	No	Yes	No	0	0
Yes	Yes	Yes	No	No	Yes	Yes	10	0
No	Yes	Yes	No	Yes	Yes	Yes	0	0
Yes	Yes	Yes	No	No	Yes	Yes	12	0
No	No	No	No	No	Yes	No	2	0
Yes	Yes	Yes	No	No	Yes	Yes	0	0
Yes	Yes	Yes	No	No	Yes	No	2	0
No	Yes	Yes	No	Yes	Yes	Yes	6	2
Yes	Yes	Yes	No	No	Yes	No	2	0
No	No	Yes	No	No	Yes	No	0	2
No	No	No	Yes	No	No	No	0	0
Yes	Yes	Yes	No	No	Yes	No	1	4

For peer review only

No	No	Yes	No	No	No	Yes	0	0
No	Yes	Yes	No	Yes	Yes	No	23	6
No	No	No	No	No	No	No	0	0
Yes	Yes	Yes	No	No	Yes	No	3	5
No	No	Yes	No	No	Yes	No	0	0
No	Yes	No	No	No	Yes	Yes	8	0
Yes	Yes	Yes	No	No	Yes	No	1	2
Yes	Yes	Yes	No	No	Yes	Yes	0	0
Yes	Yes	Yes	No	No	Yes	Yes	3	0
Yes	Yes	Yes	No	No	Yes	Yes	8	2
Yes	Yes	Yes	No	No	Yes	No	7	0
No	No	Yes	No	No	Yes	Yes	2	0
Yes	Yes	No	No	No	Yes	No	2	0
No	No	No	No	Yes	No	No	0	0
Yes	Yes	Yes	No	No	Yes	No	4	0
No	Yes	Yes	No	No	Yes	No	5	0
No	No	Yes	No	No	Yes	Yes	0	0
No	Yes	No	No	Yes	Yes	No	3	4
Yes	Yes	No	No	No	Yes	No	1	2
No	No	No	No	No	No	No	0	0
Yes	Yes	Yes	No	No	Yes	Yes	4	0
No	Yes	Yes	No	No	No	Yes	0	0
Yes	Yes	Yes	No	No	Yes	No	0	0
No	No	Yes	No	No	Yes	Yes	0	0
No	No	No	No	No	No	No	0	0
No	No	No	No	No	Yes	No	6	0
Yes	Yes	Yes	No	No	No	Yes	0	0
No	No	No	No	No	Yes	No	0	0
Yes	Yes	Yes	No	No	No	No	0	0
Yes	Yes	Yes	No	No	Yes	Yes	0	0
Yes	Yes	Yes	No	No	No	No	0	0
No	No	Yes	No	No	Yes	Yes	0	0
Yes	Yes	Yes	No	No	Yes	Yes	2	0
Yes	Yes	Yes	No	No	Yes	No	0	0
No	Yes	Yes	No	No	No	Yes	0	0
Yes	Yes	Yes	No	No	Yes	No	0	0
Yes	Yes	Yes	No	No	Yes	Yes	0	0
Yes	Yes	Yes	No	No	No	Yes	0	0
No	No	No	No	No	No	Yes	0	0
Yes	No	Yes	No	No	No	No	0	0
No	No	Yes	No	No	Yes	No	4	0
Yes	Yes	Yes	No	No	Yes	Yes	0	0
Yes	Yes	Yes	No	No	Yes	No	1	0
No	Yes	Yes	No	No	Yes	No	2	6
Yes	Yes	Yes	No	No	Yes	Yes	0	0
Yes	Yes	Yes	No	No	No	Yes	0	0
No	No	No	No	No	Yes	No	2	0
Yes	Yes	Yes	No	No	Yes	Yes	0	0
Yes	Yes	Yes	No	No	No	No	0	0
Yes	Yes	Yes	No	No	No	Yes	0	0
Yes	Yes	Yes	No	No	No	No	0	0
Yes	Yes	Yes	No	No	Yes	Yes	9	0

For peer review only

Yes	Yes	Yes	No	Yes	No	Yes	0	0
No	No	No	No	No	Yes	Yes	0	0
Yes	Yes	Yes	No	No	Yes	Yes	0	0
No	No	No	No	No	No	Yes	0	0
Yes	Yes	Yes	No	No	No	Yes	0	0
No	No	No	No	No	Yes	Yes	0	0
Yes	Yes	Yes	No	No	Yes	No	0	0
Yes	Yes	Yes	No	No	No	No	0	0
No	No	No	No	No	Yes	Yes	0	0
Yes	Yes	Yes	No	No	No	Yes	0	0
Yes	Yes	Yes	No	No	Yes	Yes	0	0
No	No	No	No	No	Yes	Yes	15	0
No	No	Yes	No	No	Yes	No	0	0
No	No	No	No	No	Yes	Yes	0	0
Yes	Yes	Yes	No	No	No	No	0	0
Yes	Yes	Yes	No	No	Yes	No	0	0
Yes	Yes	No	No	No	Yes	Yes	0	0
No	No	Yes	No	No	Yes	Yes	10	0
Yes	Yes	Yes	No	No	No	Yes	0	0
Yes	Yes	Yes	No	No	Yes	Yes	0	0
Yes	Yes	Yes	No	No	Yes	No	0	0
No	No	Yes	No	No	No	No	0	0
No	Yes	Yes	No	No	Yes	Yes	0	0
Yes	Yes	Yes	No	No	Yes	Yes	5	0
Yes	Yes	Yes	No	No	No	Yes	0	0
Yes	Yes	Yes	No	No	Yes	Yes	0	0
No	No	No	No	No	No	No	0	0
Yes	Yes	Yes	No	No	No	Yes	0	0
No	No	Yes	No	No	Yes	Yes	3	0
No	No	No	No	No	Yes	No	5	0
Yes	Yes	Yes	No	No	Yes	Yes	0	2
Yes	Yes	Yes	No	No	Yes	No	7	0
No	No	Yes	No	No	Yes	Yes	0	0
Yes	Yes	Yes	No	No	No	Yes	0	0
Yes	Yes	Yes	No	No	Yes	No	20	0
Yes	Yes	Yes	No	No	Yes	Yes	0	0
No	No	Yes	No	No	Yes	No	4	0
Yes	Yes	Yes	No	No	No	No	0	0
Yes	Yes	Yes	No	No	No	Yes	0	0
No	Yes	Yes	No	No	Yes	Yes	2	0
No	No	No	No	No	Yes	Yes	1	0
No	No	No	No	No	Yes	No	0	0
No	No	No	No	No	Yes	Yes	0	0
Yes	Yes	Yes	No	No	Yes	No	2	0
Yes	Yes	Yes	No	No	No	Yes	0	0
Yes	Yes	Yes	No	No	No	Yes	0	0
Yes	Yes	Yes	No	No	No	Yes	0	0
No	No	Yes	No	No	No	Yes	0	0
No	Yes	Yes	No	No	No	No	0	0
No	No	No	No	No	Yes	No	0	0
No	Yes	No	No	No	Yes	Yes	0	0
Yes	Yes	Yes	No	No	No	No	0	0

For peer review only

No	Yes	No	No	No	Yes	Yes	0	0
Yes	No	Yes	No	No	Yes	No	2	0
No	No	Yes	No	No	Yes	Yes	13	0
No	No	Yes	No	No	Yes	Yes	0	0
Yes	Yes	Yes	No	No	No	Yes	0	0
Yes	Yes	Yes	No	No	Yes	Yes	10	0
Yes	Yes	Yes	No	No	Yes	Yes	0	0
No	No	No	No	No	Yes	Yes	0	0
Yes	Yes	Yes	No	No	Yes	Yes	6	5
Yes	Yes	Yes	No	No	Yes	Yes	0	0
Yes	Yes	Yes	No	No	Yes	Yes	3	0
Yes	Yes	Yes	No	No	No	No	0	0
No	Yes	Yes	No	No	Yes	Yes	4	0
No	No	No	No	No	No	Yes	0	0
Yes	Yes	Yes	No	No	Yes	Yes	7	0
Yes	Yes	Yes	No	No	No	No	0	0
No	No	Yes	No	No	No	Yes	0	0
Yes	Yes	Yes	No	No	Yes	Yes	0	0
No	No	No	No	No	Yes	Yes	0	0
No	No	No	No	No	Yes	Yes	0	0
No	No	Yes	No	No	Yes	Yes	15	0
No	No	Yes	No	No	Yes	Yes	10	0
Yes	No	Yes	No	No	Yes	Yes	4	0
Yes	Yes	Yes	No	No	Yes	No	0	0
No	No	Yes	No	No	Yes	Yes	0	0
Yes	Yes	Yes	No	No	Yes	Yes	0	0
Yes	Yes	Yes	Yes	No	Yes	No	0	0
Yes	Yes	Yes	No	No	No	Yes	0	0
Yes	Yes	Yes	No	No	No	No	0	0
Yes	Yes	Yes	No	No	Yes	Yes	0	0
Yes	Yes	No	No	No	Yes	No	0	0
No	No	Yes	No	No	Yes	Yes	0	0
No	No	Yes	No	No	Yes	Yes	0	0
Yes	Yes	Yes	No	No	No	Yes	0	0
No	No	Yes	No	No	Yes	Yes	0	0
Yes	Yes	Yes	No	No	Yes	Yes	0	0
No	No	No	No	No	Yes	No	0	0
No	Yes	Yes	No	No	No	No	0	0
Yes	Yes	Yes	No	No	Yes	Yes	12	0
No	No	No	No	No	Yes	Yes	15	2
Yes	Yes	Yes	No	No	No	No	0	0
No	No	No	No	No	No	Yes	0	0
Yes	Yes	Yes	No	No	No	Yes	0	0
Yes	Yes	Yes	No	No	Yes	Yes	2	0
No	No	Yes	No	No	Yes	No	0	0
No	No	No	No	No	No	No	0	0
Yes	Yes	Yes	No	No	Yes	Yes	0	0
Yes	Yes	Yes	Yes	No	Yes	No	4	0
No	No	Yes	No	No	Yes	Yes	1	0
Yes	Yes	Yes	No	No	No	No	0	0
Yes	No	No	No	No	Yes	No	0	0
Yes	Yes	Yes	No	No	No	No	0	0

For peer review only

Yes	Yes	Yes	No	No	Yes	No	3	0
Yes	Yes	Yes	No	No	No	No	0	0
No	No	No	No	No	Yes	Yes	2	0
Yes	Yes	Yes	No	No	Yes	No	0	0
Yes	Yes	Yes	No	No	No	Yes	0	0
Yes	Yes	Yes	No	No	Yes	No	0	0
Yes	Yes	Yes	No	No	No	Yes	0	0
Yes	Yes	Yes	No	No	Yes	Yes	12	1
No	No	No	No	No	Yes	Yes	5	0
No	No	Yes	No	No	Yes	Yes	0	0
No	No	Yes	No	No	Yes	Yes	23	0
No	No	Yes	No	No	Yes	No	0	0
No	No	Yes	No	No	No	Yes	0	0
No	No	No	No	No	No	No	0	0
Yes	Yes	Yes	No	No	Yes	Yes	0	0
Yes	Yes	Yes	No	No	No	Yes	0	0
No	No	No	No	No	Yes	Yes	0	0
Yes	Yes	Yes	No	No	Yes	Yes	0	0
Yes	Yes	Yes	No	No	No	Yes	0	0
Yes	Yes	Yes	No	No	Yes	Yes	6	0
Yes	Yes	Yes	No	No	Yes	Yes	10	0
No	No	Yes	No	No	Yes	Yes	0	0
Yes	Yes	Yes	No	No	Yes	No	3	0
Yes	Yes	Yes	No	No	Yes	No	0	0
Yes	Yes	Yes	No	No	Yes	No	1	0
Yes	Yes	Yes	No	No	Yes	Yes	3	5
Yes	Yes	Yes	No	Yes	No	Yes	0	0
Yes	Yes	Yes	No	No	Yes	Yes	0	0
Yes	Yes	Yes	No	No	No	Yes	0	0
Yes	Yes	Yes	No	No	Yes	Yes	7	0
Yes	Yes	Yes	No	No	No	Yes	0	0
Yes	Yes	Yes	No	No	No	Yes	0	0
No	No	No	No	No	Yes	No	0	0
Yes	Yes	Yes	No	No	Yes	Yes	0	0
No	No	No	No	No	Yes	Yes	0	0
No	No	No	No	No	No	No	0	0
Yes	Yes	Yes	No	No	No	Yes	0	0
No	Yes	Yes	No	No	Yes	Yes	5	0
No	Yes	Yes	No	No	No	No	0	0
Yes	Yes	Yes	No	No	No	Yes	0	0
Yes	Yes	Yes	No	No	Yes	Yes	5	0
No	No	Yes	No	No	Yes	Yes	0	0
Yes	Yes	Yes	No	No	Yes	No	7	0
Yes	Yes	Yes	No	No	No	Yes	0	0
No	No	No	No	No	No	No	0	0
Yes	Yes	Yes	No	No	Yes	Yes	5	0
Yes	Yes	Yes	No	No	No	Yes	0	0
No	No	Yes	No	No	Yes	Yes	0	0
No	No	Yes	No	No	Yes	No	10	0
No	Yes	Yes	No	No	Yes	Yes	4	0
Yes	Yes	Yes	No	No	No	Yes	0	0
No	No	No	No	No	No	Yes	0	0

For peer review only

Yes	Yes	Yes	No	No	Yes	Yes	0	0
Yes	Yes	Yes	No	No	No	Yes	0	0
Yes	Yes	Yes	No	No	Yes	No	0	0
Yes	Yes	Yes	No	No	Yes	Yes	4	0
No	No	Yes	No	No	Yes	No	4	0
No	No	Yes	No	No	Yes	Yes	6	0
Yes	Yes	Yes	No	No	No	Yes	0	0
No	No	No	No	No	No	Yes	0	0
No	No	No	No	No	Yes	Yes	0	0
Yes	No	Yes	No	No	Yes	No	0	0
No	No	No	No	No	Yes	Yes	0	0
Yes	Yes	Yes	No	Yes	No	No	0	0
Yes	Yes	Yes	No	No	No	Yes	0	0
No	No	No	No	No	Yes	Yes	3	0
Yes	Yes	No	No	No	Yes	Yes	0	0
Yes	Yes	Yes	No	No	Yes	No	5	0
No	Yes	Yes	No	No	No	Yes	0	0
Yes	No	Yes	No	No	No	No	0	0
Yes	Yes	Yes	No	No	No	Yes	0	0
Yes	Yes	Yes	No	No	No	Yes	0	0
Yes	Yes	Yes	No	No	No	Yes	0	0
Yes	Yes	No	No	No	No	No	0	0
No	No	Yes	No	No	No	Yes	0	0
No	No	No	No	No	Yes	No	0	0
Yes	Yes	Yes	No	No	Yes	Yes	0	0
Yes	Yes	Yes	No	No	Yes	No	0	0
Yes	Yes	Yes	No	No	No	Yes	0	0
Yes	Yes	Yes	No	No	No	Yes	0	0
No	No	No	No	No	Yes	Yes	0	0
Yes	Yes	Yes	No	No	No	Yes	0	0
Yes	Yes	Yes	No	No	Yes	No	0	0
Yes	Yes	Yes	No	No	Yes	Yes	4	0
Yes	Yes	Yes	No	No	Yes	Yes	1	0
Yes	Yes	Yes	No	No	No	Yes	0	0
Yes	Yes	Yes	No	No	Yes	Yes	1	0
No	No	No	No	No	Yes	No	0	0
Yes	Yes	Yes	No	No	No	No	0	0
Yes	Yes	Yes	No	No	No	No	0	0
Yes	Yes	No	No	No	Yes	No	5	0
Yes	No	No	No	No	No	No	0	0
Yes	Yes	Yes	No	No	No	No	0	0
Yes	Yes	Yes	No	No	Yes	No	3	0
Yes	Yes	Yes	No	No	Yes	No	10	0
No	No	No	No	No	Yes	No	0	0
Yes	Yes	Yes	No	No	No	Yes	0	0
Yes	Yes	Yes	No	No	No	Yes	0	0
Yes	Yes	Yes	No	No	Yes	Yes	4	0
Yes	Yes	Yes	No	No	No	No	0	0
Yes	Yes	Yes	No	No	No	Yes	0	0
Yes	Yes	Yes	No	No	No	Yes	0	0
No	No	Yes	No	No	Yes	Yes	0	0
No	No	Yes	No	No	Yes	Yes	0	0

Yes	Yes	Yes	No	No	Yes	Yes	4	0
Yes	Yes	Yes	No	No	No	No	0	0
No	No	Yes	No	No	No	Yes	0	0
Yes	Yes	Yes	No	No	No	Yes	0	0
No	No	No	No	No	No	No	0	0
Yes	Yes	Yes	No	No	No	Yes	0	0
No	No	Yes	No	No	Yes	Yes	6	0
Yes	Yes	Yes	No	No	No	No	0	0
Yes	Yes	Yes	No	No	No	Yes	0	0
No	No	Yes	No	No	Yes	Yes	0	0
No	Yes	Yes	No	No	Yes	Yes	4	0
Yes	Yes	Yes	No	No	No	Yes	0	0
No	No	No	No	Yes	Yes	No	3	0
Yes	Yes	Yes	No	No	No	Yes	0	0
No	No	Yes	No	No	Yes	Yes	0	0
No	No	No	No	No	Yes	No	4	0
Yes	Yes	Yes	No	No	No	Yes	0	0
No	Yes	No	No	No	Yes	Yes	0	0
Yes	Yes	Yes	No	No	Yes	Yes	0	0
Yes	Yes	Yes	No	No	No	Yes	0	0
Yes	Yes	Yes	No	No	Yes	Yes	0	8
No	No	No	No	No	Yes	Yes	4	0
Yes	Yes	Yes	No	No	No	No	0	0
No	No	No	Yes	No	Yes	No	2	0
Yes	Yes	Yes	No	No	Yes	No	0	0
Yes	Yes	Yes	No	No	No	No	0	0
No	Yes	Yes	No	No	Yes	No	15	0
No	No	No	No	No	Yes	Yes	0	0
Yes	Yes	Yes	No	No	Yes	Yes	0	0
Yes	Yes	Yes	No	No	No	Yes	0	0
No	No	Yes	No	No	Yes	Yes	4	0
Yes	Yes	Yes	No	No	No	Yes	0	0
No	No	No	No	No	Yes	Yes	0	0
Yes	Yes	Yes	No	No	Yes	Yes	10	0
Yes	Yes	Yes	No	No	Yes	Yes	0	0
Yes	Yes	Yes	No	No	Yes	Yes	0	0
Yes	Yes	Yes	No	No	Yes	Yes	0	0
No	No	No	No	No	Yes	No	3	0
Yes	Yes	Yes	No	No	No	No	0	0
Yes	Yes	Yes	No	No	No	Yes	0	0
No	No	Yes	No	No	Yes	No	2	0
Yes	Yes	Yes	No	No	Yes	Yes	0	0
Yes	Yes	Yes	No	No	Yes	Yes	3	0
No	No	Yes	No	No	No	Yes	0	0
Yes	Yes	Yes	No	No	No	No	0	0
Yes	Yes	Yes	No	No	Yes	Yes	0	0
No	No	No	No	No	No	Yes	0	0
No	No	Yes	No	No	Yes	Yes	0	0
Yes	Yes	Yes	No	No	Yes	Yes	2	0
Yes	Yes	Yes	No	No	No	Yes	0	0
No	No	No	No	No	No	No	0	0
No	Yes	Yes	No	No	Yes	Yes	0	0

For peer review only

Yes	Yes	Yes	No	No	Yes	No	0	0
Yes	Yes	Yes	No	No	Yes	Yes	0	0
No	No	No	No	No	Yes	No	5	5
No	No	Yes	No	No	No	Yes	0	0
No	No	Yes	No	No	No	No	0	0
Yes	Yes	No	No	No	No	Yes	0	0
No	No	Yes	No	No	No	Yes	0	0
Yes	Yes	Yes	No	No	No	Yes	0	0
Yes	Yes	Yes	No	No	No	Yes	0	0
Yes	Yes	No	No	No	Yes	Yes	0	0
No	No	No	No	No	Yes	No	0	0
No	Yes	Yes	No	No	No	No	0	0
Yes	Yes	Yes	No	No	Yes	Yes	5	20
No	No	Yes	No	No	Yes	Yes	15	0
Yes	Yes	Yes	No	No	No	Yes	0	0
Yes	Yes	Yes	No	No	Yes	No	3	0
Yes	Yes	Yes	No	No	Yes	Yes	5	0
Yes	No	Yes	No	No	Yes	No	3	0
Yes	Yes	Yes	No	Yes	Yes	Yes	5	0
Yes	Yes	Yes	No	No	No	Yes	0	0
No	Yes	Yes	No	No	Yes	Yes	2	0
No	No	Yes	No	No	Yes	Yes	2	0
No	Yes	Yes	No	No	Yes	Yes	2	0
Yes	Yes	Yes	No	Yes	No	No	0	0
Yes	No	Yes	No	No	Yes	Yes	5	0
Yes	Yes	Yes	No	No	Yes	No	0	0
Yes	Yes	Yes	Yes	Yes	Yes	Yes	12	0
Yes	Yes	No	Yes	Yes	No	No	0	0
Yes	No	No	No	No	Yes	Yes	5	0
Yes	Yes	Yes	No	Yes	Yes	No	8	0
Yes	Yes	Yes	No	No	Yes	Yes	20	0
No	No	Yes	No	No	Yes	No	0	8
Yes	Yes	Yes	No	No	No	Yes	0	0
No	Yes	No	No	No	No	No	0	0
Yes	No	No	No	No	Yes	No	2	0
No	No	Yes	No	No	No	No	0	0
Yes	Yes	Yes	No	No	Yes	Yes	3	0
Yes	Yes	Yes	No	No	Yes	Yes	2	0
Yes	Yes	Yes	No	No	No	Yes	0	0
No	No	Yes	No	No	No	No	0	0
No	No	Yes	No	No	Yes	No	0	0
Yes	Yes	Yes	Yes	No	Yes	Yes	8	0
Yes	No	Yes	No	No	Yes	Yes	15	0
Yes	Yes	Yes	No	No	Yes	Yes	0	0
No	No	Yes	No	No	Yes	Yes	0	0
No	No	No	No	Yes	No	No	0	0
No	No	Yes	No	Yes	Yes	No	5	0
Yes	No	Yes	No	No	Yes	Yes	4	0
Yes	Yes	Yes	No	No	Yes	Yes	8	0
Yes	Yes	Yes	No	No	Yes	Yes	0	0
No	No	Yes	No	No	Yes	No	3	0
Yes	No	Yes	No	No	Yes	Yes	3	0

For peer review only

Yes	Yes	Yes	No	No	Yes	Yes	3	0
Yes	Yes	Yes	No	No	Yes	Yes	8	2
Yes	Yes	Yes	No	No	Yes	Yes	4	0
No	No	Yes	No	No	Yes	No	8	0
No	No	Yes	No	No	No	No	0	0
Yes	No	Yes	No	No	Yes	Yes	5	0
No	No	No	No	No	Yes	No	0	0
No	No	No	No	No	No	No	0	0
Yes	Yes	Yes	No	No	Yes	No	5	0
Yes	No	Yes	No	No	Yes	Yes	1	0
No	No	Yes	No	Yes	Yes	Yes	8	0
No	No	Yes	No	No	Yes	No	2	0
No	No	Yes	No	No	Yes	No	2	0
No	No	Yes	No	No	Yes	No	7	0
No	No	Yes	No	No	Yes	No	7	0
Yes	Yes	Yes	No	No	Yes	No	0	0
Yes	Yes	Yes	No	No	Yes	No	0	0
No	Yes	Yes	No	No	Yes	Yes	8	0
Yes	No	No	No	No	Yes	Yes	1	0
Yes	Yes	Yes	No	No	Yes	Yes	6	0
No	Yes	Yes	No	No	Yes	Yes	6	0
Yes	Yes	Yes	No	No	Yes	Yes	40	0
Yes	Yes	Yes	No	No	Yes	Yes	4	0
No	No	Yes	No	No	No	Yes	0	0
Yes	Yes	Yes	No	No	Yes	No	0	0
Yes	Yes	Yes	No	No	Yes	Yes	0	0
Yes	No	Yes	No	No	No	No	0	0
Yes	Yes	Yes	No	No	Yes	Yes	0	0
Yes	Yes	Yes	No	No	Yes	Yes	5	0
No	No	No	Yes	No	Yes	No	0	0
No	No	Yes	No	No	Yes	No	0	0
Yes	Yes	Yes	No	No	Yes	No	0	0
No	No	Yes	No	No	Yes	Yes	8	0
No	No	Yes	No	No	Yes	No	3	0
Yes	Yes	Yes	No	No	Yes	Yes	8	4
Yes	Yes	Yes	No	No	Yes	Yes	0	0
Yes	No	Yes	No	No	Yes	Yes	0	0
No	No	Yes	No	No	Yes	No	0	0
Yes	Yes	Yes	No	No	Yes	No	10	0
Yes	No	Yes	No	No	Yes	Yes	2	0
Yes	Yes	Yes	No	No	Yes	No	2	0
No	No	Yes	No	No	Yes	No	0	20
Yes	Yes	Yes	No	No	Yes	Yes	8	0
No	No	No	No	No	Yes	No	0	0
Yes	No	Yes	No	No	Yes	Yes	7	0
Yes	Yes	Yes	No	No	Yes	No	6	0
No	No	Yes	No	No	Yes	No	0	0
Yes	No	Yes	No	No	Yes	Yes	6	0
Yes	Yes	Yes	No	No	Yes	Yes	5	0
No	No	Yes	No	No	Yes	No	0	0
Yes	No	Yes	No	No	Yes	Yes	5	0
Yes	Yes	Yes	No	No	Yes	Yes	7	0

For peer review only

Yes	Yes	Yes	No	No	Yes	Yes	5	0
No	No	No	No	No	Yes	No	0	0
Yes	No	Yes	No	No	Yes	No	6	0
Yes	No	Yes	No	No	Yes	No	5	0
Yes	No	Yes	No	Yes	Yes	No	0	0
Yes	No	No	No	No	Yes	No	5	0
No	Yes	Yes	No	No	Yes	No	2	0
Yes	Yes	Yes	No	Yes	Yes	Yes	10	0
Yes	Yes	Yes	No	No	Yes	No	0	0
Yes	Yes	Yes	No	No	Yes	Yes	8	0
No	No	Yes	No	No	Yes	Yes	0	0
No	Yes	Yes	No	No	No	No	0	0
No	Yes	Yes	No	No	No	No	0	0
No	No	Yes	No	No	No	No	0	0
Yes	Yes	Yes	No	No	Yes	Yes	0	0
Yes	Yes	Yes	No	No	Yes	Yes	0	0
No	No	Yes	No	Yes	Yes	No	8	5
Yes	Yes	Yes	No	No	Yes	No	0	0
Yes	Yes	Yes	No	No	Yes	Yes	8	0
Yes	Yes	Yes	No	No	Yes	Yes	3	0
No	Yes	Yes	No	No	Yes	No	13	0
Yes	Yes	Yes	No	No	Yes	No	0	0
Yes	No	Yes	No	No	Yes	No	2	0
No	No	No	No	No	Yes	No	15	0
Yes	Yes	Yes	No	No	Yes	No	25	1
Yes	Yes	Yes	No	No	Yes	Yes	7	0
No	No	Yes	No	No	Yes	No	10	0
Yes	Yes	Yes	No	No	Yes	Yes	5	0
Yes	Yes	Yes	No	No	Yes	Yes	5	0
Yes	No	Yes	No	No	No	No	0	0
Yes	Yes	Yes	No	No	Yes	Yes	2	0
No	Yes	Yes	No	No	Yes	No	0	0
Yes	Yes	Yes	No	No	Yes	Yes	0	0
No	No	Yes	No	No	Yes	Yes	0	0
Yes	Yes	Yes	No	No	No	Yes	0	0
No	No	Yes	No	No	Yes	No	0	0
No	No	Yes	No	No	Yes	No	0	0
No	No	Yes	No	No	Yes	No	6	0
Yes	Yes	Yes	No	No	Yes	Yes	9	0
No	No	Yes	No	No	Yes	Yes	0	8
Yes	Yes	Yes	No	No	Yes	No	0	0
Yes	Yes	Yes	No	No	Yes	Yes	1	0
No	No	Yes	No	No	No	No	0	0
No	No	Yes	No	No	Yes	No	15	0
Yes	Yes	Yes	No	No	Yes	Yes	0	0
Yes	No	Yes	No	No	Yes	Yes	5	0
Yes	Yes	Yes	No	No	Yes	Yes	0	4
Yes	Yes	Yes	No	No	No	Yes	0	0
No	No	Yes	No	No	No	Yes	0	0
Yes	Yes	Yes	No	No	Yes	Yes	6	12
Yes	Yes	No	No	No	No	No	0	0
Yes	No	Yes	No	Yes	Yes	Yes	0	0

For peer review only

Yes	Yes	Yes	No	No	Yes	Yes	0	0
Yes	Yes	Yes	No	No	No	Yes	0	0
Yes	Yes	Yes	No	No	Yes	Yes	8	0
Yes	Yes	Yes	No	No	Yes	Yes	4	0
Yes	No	Yes	No	No	Yes	Yes	1	0
Yes	Yes	Yes	No	No	No	Yes	0	0
No	No	Yes	No	No	Yes	No	5	0
No	No	Yes	No	No	Yes	No	0	0
Yes	No	Yes	No	No	Yes	No	0	6
Yes	Yes	Yes	No	No	Yes	Yes	0	0
Yes	Yes	Yes	No	No	Yes	Yes	8	0
Yes	Yes	Yes	No	No	Yes	Yes	1	0
Yes	Yes	Yes	No	No	Yes	Yes	0	0
Yes	No	Yes	No	No	Yes	Yes	0	0
Yes	Yes	Yes	No	No	Yes	No	7	0
Yes	No	No	No	No	Yes	Yes	30	0
No	Yes	Yes	No	No	Yes	Yes	8	0
No	No	No	No	No	Yes	No	2	0
No	No	Yes	No	No	Yes	No	0	0
Yes	Yes	Yes	No	No	No	Yes	0	0
Yes	Yes	Yes	No	No	Yes	Yes	8	0
Yes	No	Yes	No	No	Yes	Yes	5	0
Yes	Yes	Yes	No	No	Yes	Yes	0	0
Yes	Yes	No	No	No	Yes	Yes	1	0
No	No	No	No	Yes	Yes	No	1	0
Yes	Yes	Yes	No	No	Yes	Yes	3	0
Yes	Yes	Yes	No	No	Yes	Yes	0	2
No	No	No	No	Yes	Yes	Yes	0	5
Yes	Yes	Yes	No	No	No	Yes	0	0
No	No	Yes	No	No	No	No	0	0
Yes	Yes	Yes	No	No	Yes	Yes	0	0
Yes	No	Yes	No	No	Yes	No	0	0
Yes	No	Yes	No	No	Yes	No	10	0
No	No	Yes	No	Yes	Yes	No	3	0
No	No	Yes	No	No	No	No	0	0
Yes	No	No	No	Yes	Yes	No	4	0
No	No	No	No	No	Yes	Yes	2	0
Yes	Yes	Yes	No	No	Yes	Yes	5	0
No	No	Yes	No	No	No	Yes	0	0
Yes	Yes	Yes	No	No	Yes	No	16	3
Yes	Yes	Yes	No	No	Yes	Yes	6	0
Yes	Yes	No	No	No	Yes	Yes	6	0
No	No	Yes	No	No	Yes	Yes	0	0
Yes	No	Yes	No	No	Yes	No	4	0
Yes	Yes	No	No	No	Yes	Yes	0	4
No	No	No	No	No	Yes	Yes	5	0
Yes	Yes	No	No	No	Yes	No	0	0
No	Yes	Yes	No	No	Yes	No	2	0
No	No	No	No	Yes	Yes	No	7	6
Yes	Yes	Yes	No	No	Yes	Yes	5	0
No	No	Yes	No	No	Yes	Yes	10	0
No	Yes	Yes	No	No	Yes	No	6	0

For peer review only

Yes	Yes	Yes	No	No	No	No	0	0
Yes	Yes	Yes	No	No	Yes	Yes	0	0
No	No	Yes	No	No	Yes	Yes	6	0
No	No	Yes	No	No	Yes	Yes	0	0
No	No	Yes	No	No	Yes	No	2	6
No	No	No	No	No	Yes	No	5	2
Yes	Yes	Yes	No	No	No	Yes	0	0
No	No	Yes	No	No	No	Yes	0	0
No	No	Yes	No	No	No	No	0	0
Yes	No	No	No	No	Yes	No	2	0
No	No	Yes	No	No	Yes	Yes	4	0
Yes	No	Yes	No	Yes	Yes	Yes	8	0
No	No	Yes	No	No	Yes	Yes	0	0
No	No	No	No	No	Yes	Yes	0	0
No	No	Yes	No	No	Yes	Yes	0	0
Yes	Yes	Yes	No	No	Yes	Yes	12	12
No	No	Yes	No	No	Yes	Yes	2	3
Yes	Yes	Yes	No	No	Yes	Yes	0	2
Yes	Yes	Yes	No	No	Yes	Yes	2	0
Yes	Yes	Yes	No	Yes	Yes	Yes	0	0
Yes	Yes	Yes	No	No	No	No	0	0
No	Yes	Yes	No	No	Yes	No	0	0
No	No	Yes	No	No	Yes	Yes	0	0
No	No	Yes	No	No	Yes	Yes	7	0
No	No	Yes	No	No	Yes	Yes	0	0
No	Yes	No	No	No	Yes	No	0	3
No	No	Yes	No	No	Yes	Yes	2	0
Yes	Yes	Yes	No	No	Yes	Yes	0	0
No	No	Yes	No	No	Yes	Yes	4	0
No	No	Yes	No	No	Yes	Yes	3	0
Yes	Yes	Yes	No	No	Yes	Yes	0	0
No	No	No	No	No	Yes	No	4	0
Yes	Yes	Yes	No	No	Yes	Yes	12	0
No	No	No	No	No	Yes	Yes	5	0
No	No	Yes	No	No	Yes	Yes	6	3
No	No	Yes	No	No	Yes	No	4	0
Yes	Yes	Yes	No	No	Yes	No	3	0
No	No	No	No	No	Yes	No	0	0
No	No	Yes	No	No	Yes	No	4	3
No	No	Yes	No	No	Yes	Yes	2	0
No	No	No	No	No	No	No	0	0
No	No	Yes	No	No	Yes	Yes	0	0
No	No	Yes	No	No	Yes	No	30	0
No	No	No	No	No	No	No	0	0
No	No	No	No	No	No	No	0	0
Yes	Yes	Yes	No	No	Yes	Yes	7	1
No	No	Yes	No	No	Yes	Yes	0	0
Yes	Yes	Yes	No	No	Yes	No	2	0
Yes	Yes	Yes	No	No	Yes	Yes	3	0
Yes	No	Yes	No	No	Yes	Yes	0	0
No	No	No	No	No	No	No	0	0
Yes	Yes	Yes	No	No	Yes	Yes	6	0

For peer review only

No	No	Yes	No	No	Yes	No	4	1
No	No	Yes	No	No	Yes	No	15	0
Yes	Yes	Yes	No	No	No	No	0	0
Yes	Yes	Yes	No	No	Yes	No	5	9
No	No	Yes	No	No	Yes	Yes	0	0
Yes	Yes	Yes	No	No	Yes	Yes	5	0
Yes	Yes	Yes	No	No	Yes	Yes	0	0
No	No	No	No	No	Yes	Yes	0	0
Yes	No	Yes	No	No	Yes	No	3	0
No	No	No	No	No	Yes	Yes	0	0
Yes	Yes	Yes	No	No	Yes	No	7	0
Yes	No	Yes	No	No	Yes	Yes	0	0
Yes	Yes	Yes	No	No	No	No	0	0
No	No	Yes	No	Yes	Yes	Yes	1	0
Yes	Yes	Yes	No	No	Yes	Yes	0	0
No	No	Yes	No	No	Yes	No	6	0
No	No	Yes	No	No	Yes	Yes	0	0
Yes	No	Yes	No	No	Yes	Yes	20	4
No	No	No	No	No	Yes	No	0	4
Yes	Yes	Yes	No	No	No	No	0	0
No	No	Yes	No	No	Yes	Yes	0	0
Yes	Yes	Yes	No	No	Yes	Yes	5	0
Yes	Yes	Yes	No	No	Yes	No	0	0
No	No	Yes	No	No	Yes	No	5	6
Yes	Yes	Yes	No	No	Yes	Yes	9	0
Yes	Yes	Yes	No	Yes	Yes	Yes	12	0
No	No	No	No	No	Yes	Yes	6	0
Yes	Yes	Yes	No	No	Yes	No	0	0
No	No	Yes	No	No	Yes	No	2	0
No	No	Yes	No	No	Yes	Yes	2	0
Yes	Yes	Yes	No	No	No	No	0	0
Yes	Yes	Yes	No	No	No	No	0	0
Yes	Yes	Yes	No	No	Yes	Yes	9	0
Yes	Yes	Yes	No	No	Yes	Yes	3	6
Yes	Yes	Yes	No	No	No	No	0	0
Yes	Yes	Yes	No	No	Yes	Yes	20	16
Yes	Yes	Yes	No	No	Yes	Yes	1	0
Yes	Yes	Yes	No	No	Yes	Yes	9	0
Yes	Yes	Yes	No	No	Yes	Yes	5	0
Yes	Yes	Yes	No	No	Yes	No	0	0
Yes	Yes	Yes	Yes	No	No	No	0	0
Yes	Yes	Yes	No	No	Yes	Yes	10	0
No	No	Yes	No	No	No	No	0	0
No	Yes	Yes	No	No	No	No	0	0
Yes	Yes	Yes	No	No	Yes	No	2	3
Yes	Yes	Yes	No	No	Yes	Yes	10	0
No	No	No	No	No	No	No	0	0
No	No	Yes	No	No	Yes	No	0	0
No	No	Yes	No	No	Yes	No	2	5
Yes	No	Yes	No	No	No	Yes	0	0
Yes	Yes	Yes	No	No	Yes	Yes	2	0
Yes	Yes	Yes	No	No	Yes	No	3	0

For peer review only

Yes	Yes	Yes	No	No	Yes	No	0	0
Yes	Yes	Yes	No	No	Yes	Yes	9	0
Yes	Yes	Yes	No	Yes	Yes	Yes	7	0
No	No	Yes	No	No	Yes	No	0	0
Yes	Yes	Yes	No	No	Yes	No	1	0
Yes	Yes	Yes	No	No	Yes	Yes	5	0
No	No	No	No	No	Yes	No	0	0
No	Yes	Yes	No	No	Yes	Yes	3	0
Yes	Yes	Yes	No	No	Yes	Yes	2	0
Yes	Yes	Yes	No	No	Yes	No	4	0
No	No	No	No	No	Yes	No	5	3
No	No	No	No	No	Yes	Yes	0	0
No	No	No	No	No	Yes	Yes	0	0
No	No	No	No	No	Yes	Yes	0	0
No	No	Yes	No	No	Yes	Yes	0	0
Yes	Yes	Yes	No	No	Yes	No	5	0
Yes	Yes	Yes	No	No	Yes	Yes	0	0
No	Yes	Yes	No	No	Yes	No	5	0
No	No	Yes	No	No	Yes	Yes	10	0
Yes	Yes	Yes	No	No	Yes	Yes	5	0
Yes	Yes	Yes	No	No	Yes	Yes	5	20
Yes	Yes	Yes	No	No	Yes	No	3	6
No	No	No	No	No	Yes	No	0	0
Yes	Yes	No	No	No	Yes	No	20	0
No	No	No	No	No	Yes	No	4	0
No	No	No	No	No	Yes	Yes	0	0
No	No	Yes	No	No	Yes	Yes	0	0
Yes	Yes	Yes	No	No	Yes	Yes	0	1
Yes	Yes	Yes	No	No	No	No	0	0
Yes	Yes	Yes	No	No	Yes	No	1	0
Yes	Yes	Yes	No	No	Yes	No	11	0
Yes	Yes	Yes	No	No	Yes	Yes	0	0
No	No	Yes	No	No	Yes	No	11	0
No	No	Yes	No	No	Yes	Yes	0	0
No	No	No	No	No	Yes	Yes	2	0
No	No	Yes	No	Yes	Yes	No	7	0
No	Yes	Yes	No	No	Yes	Yes	3	0
No	Yes	Yes	No	No	Yes	No	5	3
No	No	Yes	No	No	Yes	Yes	10	0
Yes	Yes	Yes	No	No	No	Yes	0	0
No	No	Yes	No	No	Yes	Yes	0	0
Yes	Yes	No	No	No	Yes	Yes	6	0
No	No	Yes	No	Yes	Yes	No	4	0
No	No	No	No	No	Yes	Yes	0	0
Yes	Yes	Yes	No	No	No	No	0	0
No	No	Yes	No	Yes	Yes	No	5	0
No	No	Yes	No	Yes	Yes	No	6	0
No	No	Yes	No	No	Yes	Yes	1	0
No	No	Yes	No	No	Yes	Yes	6	0
No	No	Yes	No	No	Yes	Yes	0	0
No	No	Yes	No	No	Yes	Yes	4	0
No	No	No	No	No	Yes	Yes	2	0

For peer review only

For peer review only - http://bmjopen.bmj.com/site/about/guidelines.xhtml

Yes	Yes	Yes	No	No	No	No	0	0
Yes	No	No	No	No	Yes	Yes	3	0
No	No	Yes	No	No	Yes	Yes	5	1
No	No	No	No	No	Yes	Yes	2	0
Yes	Yes	Yes	No	No	Yes	Yes	6	0
No	No	Yes	No	No	Yes	No	1	0
Yes	Yes	Yes	No	No	Yes	Yes	6	0
No	Yes	Yes	No	No	No	No	0	0
Yes	Yes	Yes	No	No	Yes	Yes	1	0
No	No	No	No	No	Yes	No	0	0
No	No	No	No	No	Yes	Yes	0	0
No	No	No	No	No	Yes	No	0	0
Yes	Yes	Yes	No	No	Yes	Yes	6	0
Yes	Yes	Yes	No	No	Yes	Yes	0	0
No	No	Yes	No	Yes	Yes	No	4	0
Yes	Yes	Yes	No	Yes	Yes	No	11	0
Yes	Yes	Yes	No	No	Yes	Yes	2	0
No	No	Yes	No	No	Yes	Yes	3	0
Yes	Yes	Yes	No	No	Yes	Yes	0	0
Yes	Yes	Yes	No	No	Yes	Yes	4	0
No	No	Yes	No	Yes	Yes	Yes	0	0
Yes	Yes	Yes	No	No	No	No	0	0
No	No	Yes	No	No	Yes	Yes	5	0
Yes	Yes	Yes	No	No	Yes	Yes	3	0
No	No	Yes	No	No	Yes	No	7	0
Yes	Yes	Yes	No	No	No	Yes	0	0
Yes	Yes	Yes	No	No	Yes	Yes	5	0
No	No	No	No	No	Yes	Yes	0	0
No	No	Yes	No	No	Yes	Yes	0	0
No	No	No	No	No	Yes	Yes	15	0
No	No	No	No	No	Yes	Yes	10	0
No	No	Yes	No	No	Yes	Yes	0	0
Yes	No	Yes	No	No	Yes	Yes	3	0
No	No	Yes	No	No	Yes	Yes	0	0
Yes	Yes	Yes	No	No	Yes	Yes	3	0
Yes	Yes	Yes	No	No	Yes	Yes	2	0
No	No	Yes	No	No	Yes	Yes	4	0
No	No	Yes	No	No	Yes	Yes	0	0
No	No	Yes	No	No	Yes	Yes	4	0
Yes	Yes	Yes	No	No	Yes	No	0	0
Yes	Yes	Yes	No	No	Yes	Yes	4	0
Yes	Yes	Yes	No	No	No	Yes	0	0
Yes	Yes	Yes	No	No	Yes	Yes	0	0
Yes	Yes	Yes	No	No	Yes	Yes	10	0
Yes	Yes	Yes	No	No	Yes	Yes	0	0
Yes	Yes	Yes	No	No	Yes	Yes	5	0
Yes	Yes	Yes	No	No	Yes	Yes	8	0
Yes	Yes	Yes	No	No	No	Yes	0	0
No	No	No	No	No	Yes	Yes	0	0
Yes	Yes	No	No	No	Yes	Yes	0	0
No	No	Yes	No	No	Yes	Yes	3	0
No	No	Yes	No	No	Yes	Yes	3	0

For peer review only

Yes	Yes	Yes	No	No	No	Yes	0	0
Yes	Yes	Yes	No	No	Yes	No	0	0
Yes	Yes	Yes	No	No	Yes	Yes	0	0
Yes	Yes	Yes	No	No	Yes	No	20	0
No	No	Yes	No	No	Yes	No	0	0
No	No	Yes	No	No	Yes	Yes	3	0
Yes	Yes	Yes	No	No	Yes	Yes	0	0
No	No	No	No	No	Yes	Yes	0	0
No	No	No	No	No	Yes	Yes	0	1
No	No	Yes	No	No	Yes	Yes	3	0
No	No	Yes	No	No	Yes	Yes	0	0
Yes	Yes	Yes	No	No	Yes	Yes	4	0
Yes	Yes	Yes	No	Yes	Yes	Yes	10	0
Yes	Yes	Yes	No	No	Yes	Yes	4	0
Yes	Yes	Yes	No	No	Yes	Yes	0	0
Yes	Yes	Yes	No	No	Yes	Yes	10	0
No	No	Yes	No	No	Yes	Yes	3	0
No	No	Yes	No	No	Yes	No	0	0
No	No	Yes	No	No	Yes	Yes	0	0
Yes	Yes	Yes	No	No	Yes	Yes	6	3
Yes	Yes	Yes	No	No	Yes	Yes	6	0
Yes	No	Yes	No	No	Yes	Yes	0	0
No	No	No	No	No	Yes	Yes	0	0
No	No	Yes	No	No	Yes	Yes	0	0
No	No	No	No	Yes	Yes	Yes	0	0
No	No	No	No	No	Yes	Yes	0	0
Yes	Yes	Yes	No	No	Yes	Yes	0	10
No	No	Yes	No	No	Yes	Yes	0	0
No	No	No	No	No	Yes	Yes	4	0
No	No	Yes	No	No	Yes	Yes	4	0
Yes	Yes	Yes	No	No	Yes	Yes	0	0
Yes	Yes	Yes	No	No	Yes	Yes	0	0
Yes	Yes	Yes	No	No	Yes	Yes	0	0
Yes	Yes	Yes	No	No	Yes	Yes	2	0
No	No	No	No	No	Yes	Yes	0	0
Yes	Yes	No	No	No	Yes	Yes	2	0
Yes	Yes	Yes	No	Yes	Yes	Yes	0	0
No	No	No	No	No	Yes	Yes	1	0
No	No	Yes	No	No	Yes	Yes	10	0
Yes	Yes	No	No	No	Yes	Yes	3	0
Yes	No	Yes	No	No	Yes	Yes	0	0
Yes	Yes	Yes	No	No	Yes	Yes	20	0
No	No	Yes	No	No	Yes	Yes	0	0
Yes	Yes	Yes	No	No	Yes	Yes	6	0
Yes	Yes	Yes	No	No	Yes	Yes	0	0
Yes	No	Yes	No	No	Yes	Yes	0	0
Yes	Yes	Yes	No	No	Yes	Yes	0	0
No	Yes	Yes	No	No	Yes	Yes	0	0
No	No	Yes	No	No	Yes	Yes	0	0
Yes	Yes	Yes	No	No	Yes	Yes	5	3
Yes	Yes	Yes	No	No	Yes	Yes	0	0
No	No	Yes	No	No	Yes	Yes	0	0

For peer review only

No	No	No	No	No	Yes	Yes	0	0
Yes	Yes	Yes	No	No	Yes	Yes	1	0
No	No	Yes	No	No	Yes	Yes	3	0
No	No	Yes	No	No	Yes	Yes	0	7
Yes	Yes	Yes	No	No	Yes	Yes	10	0
No	No	No	No	No	Yes	Yes	3	10
Yes	Yes	Yes	No	No	No	Yes	0	0
No	No	No	No	No	Yes	Yes	0	0
Yes	Yes	Yes	Yes	No	Yes	Yes	4	0
Yes	Yes	Yes	No	No	Yes	Yes	0	0
Yes	Yes	Yes	No	No	Yes	Yes	0	0
Yes	Yes	Yes	No	No	Yes	Yes	5	6
Yes	Yes	No	No	No	No	Yes	0	0
Yes	No	Yes	No	No	Yes	Yes	0	0
No	No	Yes	No	No	Yes	Yes	4	0
No	No	No	No	No	Yes	Yes	6	0
Yes	Yes	Yes	No	No	Yes	Yes	3	0
No	No	Yes	No	No	Yes	Yes	0	0
Yes	Yes	Yes	No	No	Yes	Yes	10	1
No	No	No	No	No	Yes	Yes	0	0
No	No	No	No	No	Yes	Yes	0	0
Yes	Yes	Yes	No	No	Yes	Yes	5	0
No	No	No	No	No	Yes	Yes	20	0
Yes	Yes	Yes	No	No	Yes	Yes	3	0
No	No	Yes	No	No	Yes	Yes	5	0
No	No	Yes	No	No	Yes	Yes	0	0
Yes	Yes	Yes	No	No	Yes	Yes	2	0
Yes	Yes	Yes	No	No	Yes	Yes	0	0
No	No	No	No	No	Yes	Yes	2	0
Yes	Yes	Yes	No	No	Yes	Yes	5	0
Yes	Yes	Yes	No	No	Yes	Yes	0	0
Yes	Yes	Yes	No	No	Yes	Yes	0	0
No	Yes	Yes	No	No	Yes	Yes	0	0
Yes	No	Yes	No	No	Yes	Yes	1	0
Yes	Yes	Yes	No	No	Yes	Yes	0	0
Yes	Yes	Yes	No	No	Yes	Yes	4	0
No	No	Yes	No	No	Yes	Yes	0	0
Yes	Yes	Yes	No	No	Yes	Yes	3	0
Yes	No	Yes	No	No	Yes	Yes	3	0
Yes	No	Yes	No	No	Yes	Yes	1	0
Yes	Yes	Yes	Yes	No	Yes	Yes	2	0
Yes	Yes	Yes	No	No	Yes	Yes	1	0
Yes	Yes	Yes	No	No	No	Yes	0	0
No	No	Yes	No	No	No	No	0	0
No	No	No	No	No	Yes	Yes	0	0
Yes	Yes	Yes	No	No	Yes	Yes	5	0
No	No	Yes	No	No	Yes	Yes	0	0
Yes	Yes	Yes	No	No	Yes	No	3	0
Yes	Yes	Yes	No	No	No	Yes	0	0
Yes	Yes	Yes	No	No	Yes	Yes	8	0
Yes	Yes	Yes	No	No	No	Yes	0	0
Yes	Yes	Yes	No	No	No	No	0	0

For peer review only

No	No	Yes	No	No	No	No	0	0
Yes	Yes	Yes	No	No	Yes	Yes	6	0
Yes	Yes	Yes	No	No	Yes	Yes	5	0
No	No	No	No	No	Yes	No	0	0
Yes	Yes	Yes	No	No	Yes	Yes	0	0
Yes	No	Yes	No	No	Yes	Yes	0	0
Yes	Yes	Yes	No	No	Yes	Yes	2	0
Yes	Yes	Yes	No	No	Yes	Yes	0	0
Yes	Yes	Yes	No	No	No	No	0	0
Yes	Yes	Yes	No	No	Yes	Yes	20	0
Yes	Yes	Yes	No	No	Yes	Yes	10	0
Yes	Yes	Yes	No	No	Yes	Yes	3	0
No	No	No	No	No	Yes	Yes	4	0
Yes	Yes	Yes	No	No	No	Yes	0	0
Yes	Yes	Yes	No	No	No	No	0	0
Yes	Yes	Yes	No	No	No	Yes	0	0
No	No	No	No	No	Yes	Yes	0	0
Yes	Yes	Yes	No	No	Yes	Yes	0	0
Yes	Yes	Yes	No	No	Yes	Yes	0	0
Yes	Yes	Yes	No	No	Yes	Yes	0	6
Yes	Yes	Yes	No	No	Yes	Yes	0	0
Yes	Yes	Yes	No	No	Yes	Yes	5	0
Yes	Yes	Yes	No	No	Yes	Yes	1	0
No	No	No	No	No	Yes	Yes	0	0
Yes	No	Yes	No	No	Yes	Yes	6	0
Yes	No	Yes	No	No	Yes	Yes	4	0
No	No	No	No	No	Yes	Yes	0	0
No	Yes	Yes	No	No	Yes	Yes	8	0
Yes	Yes	Yes	No	No	No	Yes	0	0
No	No	Yes	No	No	Yes	Yes	3	0
No	No	No	No	No	Yes	Yes	0	0
Yes	Yes	Yes	No	No	Yes	Yes	0	0
Yes	Yes	Yes	No	Yes	Yes	Yes	3	0
No	No	Yes	No	No	Yes	Yes	4	0
Yes	Yes	Yes	Yes	No	Yes	Yes	0	0
Yes	Yes	Yes	No	No	Yes	Yes	0	0
Yes	Yes	Yes	No	No	Yes	Yes	6	0
Yes	Yes	Yes	No	No	No	Yes	0	0
Yes	No	Yes	No	No	Yes	Yes	3	0
No	No	Yes	No	No	Yes	Yes	12	0
No	No	Yes	No	No	Yes	Yes	0	0
No	No	No	No	No	Yes	Yes	2	0
No	No	Yes	No	No	Yes	Yes	4	0
No	No	Yes	No	No	Yes	Yes	5	0
Yes	No	Yes	No	No	Yes	Yes	0	0
Yes	Yes	Yes	No	No	Yes	Yes	0	0
Yes	Yes	Yes	No	No	Yes	Yes	3	0
No	No	Yes	No	No	Yes	Yes	0	0
Yes	Yes	Yes	No	Yes	Yes	Yes	0	0
Yes	Yes	Yes	No	No	Yes	No	4	0
Yes	No	No	No	No	Yes	No	0	0
Yes	Yes	Yes	No	No	No	No	0	0

For peer review only

Yes	Yes	No	No	No	Yes	No	1	0
Yes	No	No	No	No	No	No	0	0
Yes	Yes	Yes	No	No	Yes	No	5	0
Yes	Yes	Yes	No	No	Yes	No	2	0
Yes	Yes	Yes	No	No	Yes	No	3	0
No	No	No	No	No	Yes	No	0	0
Yes	Yes	Yes	No	No	Yes	No	0	0
Yes	Yes	Yes	No	No	Yes	Yes	3	0
Yes	Yes	No	No	No	Yes	Yes	2	0
Yes	No	Yes	No	No	Yes	Yes	5	0
Yes	Yes	Yes	No	Yes	No	No	0	0
Yes	Yes	Yes	No	No	Yes	No	4	0
No	No	No	No	No	Yes	No	6	7
Yes	Yes	Yes	No	No	No	No	0	0
No	No	Yes	No	No	Yes	No	2	0
No	No	No	No	No	Yes	No	2	1
No	No	No	No	No	No	No	0	0
Yes	Yes	Yes	No	No	Yes	No	0	0
No	Yes	No	No	No	No	No	0	0
Yes	Yes	Yes	No	No	Yes	No	0	7
Yes	Yes	Yes	No	No	Yes	No	0	0
Yes	Yes	No	No	No	Yes	No	3	0
No	Yes	Yes	No	No	No	No	0	0
Yes	Yes	Yes	No	No	Yes	Yes	5	2
Yes	Yes	Yes	No	No	Yes	No	0	0
Yes	No	No	No	No	No	No	0	0
Yes	Yes	Yes	No	No	Yes	No	20	0
Yes	Yes	Yes	No	No	No	No	0	0
Yes	No	Yes	No	No	Yes	No	3	2
Yes	Yes	Yes	No	No	Yes	Yes	0	0
Yes	No	Yes	No	No	Yes	No	7	5
Yes	Yes	Yes	No	No	No	Yes	0	0
Yes	Yes	Yes	No	No	Yes	Yes	5	0
Yes	No	Yes	No	No	No	No	0	0
Yes	Yes	Yes	No	No	Yes	Yes	3	0
No	Yes	No	No	No	Yes	No	10	8
Yes	Yes	Yes	No	No	Yes	Yes	4	3
Yes	Yes	Yes	No	No	Yes	Yes	4	0
No	No	Yes	No	No	Yes	Yes	0	0
No	Yes	Yes	No	No	Yes	No	4	0
Yes	Yes	No	No	No	Yes	No	5	0
Yes	Yes	Yes	No	No	No	No	0	0
Yes	Yes	Yes	No	No	Yes	No	4	0
Yes	Yes	Yes	No	No	Yes	Yes	10	0
Yes	Yes	Yes	No	No	Yes	Yes	5	0
Yes	Yes	Yes	No	No	Yes	Yes	4	6
No	No	No	No	No	Yes	Yes	4	2
Yes	Yes	Yes	No	No	Yes	No	7	0
Yes	Yes	Yes	No	No	Yes	Yes	0	0
Yes	Yes	Yes	No	No	Yes	Yes	0	0
Yes	Yes	Yes	No	Yes	Yes	Yes	2	10
Yes	Yes	Yes	No	No	Yes	Yes	0	10

For peer review only

Yes	Yes	Yes	No	No	No	No	0	0
No	No	Yes	No	No	Yes	No	0	0
Yes	Yes	Yes	No	No	Yes	Yes	3	0
No	No	No	No	No	Yes	Yes	0	0
No	Yes	No	No	No	No	Yes	0	0
Yes	Yes	Yes	No	No	Yes	Yes	10	0
No	No	No	No	No	Yes	No	4	0
Yes	Yes	Yes	No	Yes	Yes	No	4	0
Yes	Yes	Yes	No	Yes	Yes	No	5	0
Yes	Yes	Yes	No	No	Yes	Yes	3	0
No	No	Yes	No	No	Yes	Yes	3	0
No	No	Yes	No	No	Yes	No	6	0
Yes	Yes	Yes	No	No	Yes	No	5	0
No	No	Yes	No	No	Yes	Yes	0	0
Yes	Yes	Yes	No	No	Yes	Yes	30	2
No	No	Yes	No	No	Yes	No	0	0
Yes	No	Yes	No	No	Yes	No	6	0
No	No	No	No	No	Yes	Yes	3	3
Yes	Yes	Yes	No	No	No	No	0	0
Yes	Yes	Yes	No	No	Yes	Yes	0	0
No	No	No	No	No	No	No	0	0
Yes	Yes	Yes	No	No	Yes	No	0	0
No	No	Yes	No	No	Yes	No	0	0
No	Yes	Yes	No	No	Yes	Yes	3	1
Yes	No	Yes	No	No	Yes	No	2	0
Yes	Yes	Yes	No	No	Yes	Yes	3	0
Yes	Yes	Yes	No	No	No	No	0	0
Yes	Yes	Yes	No	No	Yes	Yes	6	0
Yes	Yes	Yes	No	Yes	Yes	Yes	0	0
No	No	Yes	No	No	Yes	Yes	0	0
No	No	No	No	No	No	No	0	0
No	No	No	No	No	Yes	Yes	10	0
Yes	Yes	Yes	Yes	No	No	Yes	0	0
No	Yes	Yes	No	No	Yes	No	5	0
Yes	Yes	Yes	No	No	No	No	0	0
Yes	Yes	Yes	No	No	Yes	Yes	5	5
Yes	Yes	Yes	No	No	No	No	0	0
Yes	No	Yes	No	No	Yes	Yes	4	0
No	Yes	Yes	No	Yes	Yes	Yes	2	0
No	No	No	No	No	Yes	No	1	2
Yes	Yes	Yes	No	No	Yes	No	6	0
No	Yes	Yes	No	No	No	No	0	0
No	No	Yes	No	No	Yes	No	5	0
No	No	Yes	No	No	Yes	No	3	0
Yes	Yes	Yes	No	No	Yes	No	6	0
No	No	Yes	No	No	Yes	No	5	0
Yes	Yes	No	No	No	No	No	0	0
Yes	No	Yes	No	No	Yes	Yes	5	0
Yes	Yes	Yes	No	No	Yes	No	6	12
Yes	Yes	Yes	No	No	Yes	No	6	0
No	Yes	Yes	No	No	No	No	0	0
Yes	Yes	Yes	No	No	Yes	No	10	4

For peer review only

Yes	Yes	No	No	No	Yes	No	1	1
Yes	Yes	Yes	No	No	Yes	No	10	0
Yes	Yes	Yes	No	No	Yes	Yes	0	10
No	No	Yes	No	No	Yes	Yes	0	0
Yes	Yes	Yes	No	No	Yes	No	8	0
Yes	Yes	Yes	No	No	No	No	0	0
Yes	Yes	Yes	No	No	Yes	No	3	0
Yes	Yes	Yes	No	No	Yes	No	5	0
No	No	No	No	No	Yes	Yes	5	0
Yes	Yes	Yes	No	No	Yes	No	4	0
No	No	Yes	No	No	Yes	No	0	0
Yes	Yes	Yes	No	Yes	Yes	Yes	12	0
Yes	Yes	No	No	No	Yes	No	0	3
Yes	No	No	No	No	Yes	No	0	0
No	No	Yes	No	No	Yes	No	2	0
Yes	Yes	Yes	No	No	Yes	No	0	0
Yes	Yes	No	No	No	Yes	No	2	3
Yes	Yes	Yes	No	No	No	No	0	0
Yes	Yes	Yes	No	No	Yes	Yes	5	0
No	Yes	Yes	No	No	No	No	0	0
No	No	Yes	No	No	Yes	No	4	6
No	No	No	No	No	No	No	0	0
No	No	No	No	No	Yes	Yes	3	0
No	No	No	No	No	No	No	0	0
Yes	Yes	Yes	No	No	No	No	0	0
Yes	Yes	No	No	No	No	No	0	0
Yes	Yes	Yes	No	No	No	No	0	0
Yes	Yes	Yes	No	No	Yes	Yes	2	0
No	No	Yes	No	No	Yes	No	0	0
No	No	Yes	No	No	Yes	Yes	0	0
Yes	Yes	Yes	No	No	Yes	Yes	8	0
Yes	Yes	Yes	No	No	Yes	Yes	5	0
Yes	Yes	Yes	No	No	No	No	0	0
Yes	Yes	Yes	No	No	Yes	Yes	0	1
Yes	Yes	Yes	No	No	Yes	Yes	0	0
Yes	Yes	Yes	No	No	Yes	Yes	2	0
No	Yes	Yes	No	No	Yes	No	9	0
Yes	Yes	Yes	No	No	No	No	0	0
Yes	Yes	Yes	No	No	Yes	Yes	4	3
Yes	Yes	Yes	No	No	Yes	Yes	7	15
Yes	Yes	Yes	No	Yes	Yes	Yes	6	5
No	No	Yes	No	Yes	Yes	No	0	2
Yes	Yes	Yes	No	Yes	Yes	Yes	0	0
Yes	Yes	Yes	No	No	Yes	Yes	4	0
Yes	Yes	Yes	No	No	Yes	Yes	5	3
No	No	No	No	No	No	No	0	0
No	No	Yes	No	No	Yes	No	0	0
Yes	Yes	Yes	No	No	Yes	No	5	0
Yes	Yes	Yes	No	No	Yes	No	3	0
Yes	Yes	Yes	No	No	Yes	No	1	2
Yes	Yes	Yes	No	No	No	No	0	0
Yes	Yes	Yes	No	No	Yes	No	1	0

For peer review only

No	No	No	No	No	Yes	Yes	3	0
Yes	Yes	Yes	No	No	Yes	Yes	3	0
Yes	Yes	Yes	No	No	Yes	Yes	5	3
Yes	Yes	Yes	No	No	No	No	0	0
Yes	Yes	Yes	No	No	Yes	No	1	0
Yes	No	Yes	No	No	No	No	0	0
Yes	Yes	Yes	No	No	Yes	Yes	4	3
Yes	Yes	Yes	No	No	Yes	No	1	0
No	No	Yes	No	No	No	No	0	0
No	No	No	No	No	Yes	No	0	0
No	No	No	No	No	Yes	Yes	0	0
Yes	Yes	Yes	No	No	Yes	Yes	20	0
Yes	Yes	Yes	No	No	Yes	No	3	5
Yes	Yes	Yes	No	No	Yes	No	4	0
Yes	Yes	Yes	No	No	Yes	No	2	0
Yes	Yes	Yes	No	No	Yes	No	4	0
Yes	Yes	Yes	No	No	Yes	No	5	0
Yes	Yes	Yes	No	No	Yes	No	0	0
No	No	Yes	No	No	Yes	Yes	5	0
No	No	No	No	No	Yes	Yes	6	8
Yes	Yes	No	No	No	No	No	0	0
Yes	Yes	Yes	No	No	Yes	Yes	3	0
Yes	Yes	No	No	No	Yes	No	2	0
Yes	Yes	No	No	No	Yes	No	0	3
Yes	Yes	Yes	No	No	No	No	0	0
No	Yes	Yes	No	No	Yes	No	6	6
Yes	Yes	Yes	No	No	Yes	Yes	8	0
Yes	Yes	Yes	No	Yes	Yes	Yes	15	0
Yes	Yes	Yes	No	No	Yes	Yes	6	0
No	No	No	No	No	Yes	Yes	0	0
Yes	No	Yes	No	No	Yes	No	0	0
Yes	Yes	No	No	No	Yes	No	4	2
Yes	Yes	No	No	No	Yes	No	2	0
No	Yes	No	No	No	No	No	0	0
No	No	Yes	No	No	Yes	No	1	0
Yes	Yes	Yes	No	No	No	No	0	0
No	No	No	No	No	Yes	Yes	3	0
Yes	Yes	Yes	No	No	No	No	0	0
No	No	No	No	No	Yes	No	4	0
No	No	No	No	No	Yes	No	0	0
Yes	Yes	Yes	No	No	Yes	No	3	0
Yes	Yes	Yes	No	No	No	No	0	0
Yes	Yes	Yes	No	No	Yes	Yes	3	0
Yes	Yes	Yes	No	No	Yes	No	10	0
Yes	Yes	Yes	Yes	No	Yes	No	0	0
No	No	No	No	No	No	No	0	0
No	No	Yes	No	No	Yes	Yes	0	0
No	Yes	Yes	No	No	No	No	0	0
No	No	Yes	No	No	Yes	No	3	0
No	No	No	No	No	Yes	Yes	10	0
Yes	No	Yes	No	No	Yes	Yes	0	0
No	No	No	No	No	Yes	Yes	7	0

For peer review only

No	No	Yes	No	No	Yes	Yes	6	0
Yes	Yes	Yes	No	No	Yes	Yes	4	3
Yes	Yes	Yes	No	No	No	No	0	0
Yes	Yes	Yes	No	No	Yes	Yes	0	2
No	No	No	No	No	Yes	No	0	0
No	Yes	Yes	No	No	Yes	No	3	5
No	No	Yes	No	No	Yes	Yes	5	4
No	No	No	No	No	Yes	No	6	0
Yes	Yes	Yes	No	No	Yes	No	0	0
No	No	Yes	No	No	Yes	No	2	0
No	No	No	No	No	Yes	No	6	0
Yes	Yes	Yes	No	No	Yes	No	0	0
Yes	Yes	Yes	No	No	Yes	Yes	2	0
Yes	Yes	No	No	No	No	No	0	0
Yes	Yes	Yes	No	No	Yes	No	4	0
No	No	No	No	Yes	Yes	No	6	0
Yes	Yes	No	No	No	No	No	0	0
No	No	No	No	No	Yes	No	12	0
Yes	Yes	Yes	No	No	No	No	0	0
No	Yes	No	No	No	No	No	0	0
Yes	No	No	No	No	Yes	No	12	1
Yes	Yes	Yes	No	No	Yes	Yes	4	0
No	No	No	No	No	Yes	Yes	6	3
No	No	Yes	No	No	Yes	Yes	3	3
Yes	Yes	Yes	No	No	Yes	No	6	0
No	No	No	No	No	No	No	0	0
Yes	No	Yes	No	No	Yes	Yes	0	0
No	No	No	No	No	Yes	Yes	0	0
Yes	Yes	Yes	No	No	Yes	Yes	10	5
Yes	Yes	Yes	No	No	Yes	Yes	5	0
No	No	No	No	No	Yes	Yes	3	0
No	No	Yes	No	No	Yes	Yes	10	3
Yes	Yes	Yes	No	No	No	No	0	0
Yes	Yes	Yes	No	No	No	No	0	0
Yes	No	Yes	No	No	No	No	0	0
Yes	Yes	Yes	No	No	Yes	No	2	0
Yes	Yes	Yes	No	No	Yes	Yes	4	2
No	No	No	No	No	Yes	Yes	2	2
No	No	No	No	No	Yes	No	2	0
No	No	Yes	No	No	Yes	No	0	0
Yes	Yes	Yes	No	No	Yes	No	13	8
No	No	Yes	No	No	No	No	0	0
Yes	No	Yes	No	No	Yes	Yes	6	0
Yes	No	Yes	No	Yes	Yes	No	4	0
Yes	Yes	Yes	No	No	No	No	0	0
Yes	Yes	Yes	No	No	Yes	No	6	0
No	No	Yes	No	No	Yes	No	0	4
No	No	Yes	No	No	Yes	No	0	2
Yes	Yes	Yes	No	No	No	No	0	0
Yes	Yes	No	No	No	Yes	No	3	0
Yes	Yes	Yes	No	No	Yes	Yes	9	0
Yes	Yes	Yes	No	No	No	No	0	0

For peer review only

No	No	Yes	No	No	Yes	No	5	0
Yes	Yes	Yes	No	No	Yes	No	9	3
No	No	No	No	Yes	No	No	0	0
Yes	Yes	Yes	No	No	No	No	0	0
Yes	Yes	Yes	No	No	Yes	Yes	1	0
Yes	Yes	Yes	No	Yes	No	No	0	0
Yes	Yes	Yes	No	No	Yes	No	7	0
Yes	Yes	Yes	No	No	No	No	0	0
No	No	Yes	No	No	Yes	No	0	0
Yes	Yes	Yes	No	No	No	No	0	0
Yes	Yes	Yes	No	No	Yes	Yes	3	4
Yes	No	Yes	No	No	Yes	No	3	0
No	Yes	Yes	No	No	Yes	Yes	5	8
Yes	Yes	Yes	No	No	No	No	0	0
No	No	Yes	No	No	Yes	No	4	0
Yes	Yes	Yes	No	No	Yes	No	5	0
Yes	Yes	Yes	No	No	Yes	No	0	0
Yes	Yes	Yes	No	No	No	No	0	0
Yes	Yes	Yes	No	No	Yes	Yes	0	0
Yes	Yes	Yes	No	No	Yes	No	6	0
Yes	Yes	Yes	No	No	Yes	Yes	0	6
No	No	Yes	No	No	Yes	No	3	0
No	No	Yes	No	Yes	Yes	No	8	0
Yes	Yes	Yes	No	No	Yes	No	6	5
Yes	Yes	No	No	No	No	Yes	0	0
Yes	Yes	Yes	No	No	Yes	Yes	6	0
No	No	Yes	No	No	Yes	No	0	0
Yes	Yes	Yes	No	No	No	No	0	0
Yes	Yes	Yes	No	Yes	Yes	Yes	8	0
Yes	Yes	Yes	No	No	Yes	Yes	4	0
No	Yes	Yes	No	No	Yes	Yes	12	0
Yes	Yes	Yes	No	No	Yes	No	3	0
No	No	No	No	No	No	No	0	0
Yes	Yes	Yes	No	No	Yes	No	4	3
No	No	No	No	No	Yes	Yes	3	0
Yes	Yes	Yes	No	Yes	Yes	Yes	8	0
Yes	Yes	Yes	No	No	No	No	0	0
Yes	Yes	Yes	No	No	No	No	0	0
Yes	Yes	Yes	No	No	Yes	Yes	10	0
No	Yes	No	No	No	Yes	No	2	0
No	No	No	No	No	Yes	Yes	6	0
Yes	No	Yes	No	No	No	No	0	0
Yes	No	Yes	No	No	Yes	No	7	0
Yes	Yes	Yes	No	No	Yes	No	6	3
Yes	Yes	Yes	No	Yes	Yes	No	10	0
No	No	Yes	No	No	Yes	No	12	0
No	No	Yes	No	No	Yes	Yes	0	0
Yes	Yes	Yes	No	No	Yes	Yes	2	0
Yes	Yes	Yes	No	Yes	Yes	No	8	0
Yes	Yes	Yes	No	No	Yes	No	0	0
Yes	Yes	Yes	No	No	Yes	No	4	0
Yes	Yes	Yes	No	No	Yes	No	6	6

For peer review only

Yes	Yes	No	No	No	Yes	Yes	10	0
Yes	Yes	Yes	No	No	Yes	Yes	0	0
Yes	Yes	No	No	No	Yes	No	0	0
No	No	Yes	No	No	No	No	0	0
No	No	No	No	No	Yes	No	6	1
Yes	Yes	Yes	No	No	Yes	Yes	0	0
No	No	Yes	No	No	Yes	No	0	2
Yes	Yes	Yes	No	No	Yes	Yes	6	0
Yes	Yes	No	No	Yes	Yes	No	4	0
Yes	Yes	Yes	No	Yes	Yes	No	4	0
Yes	Yes	Yes	No	No	Yes	Yes	0	2
No	No	No	No	No	Yes	Yes	4	3
No	No	No	No	Yes	Yes	No	4	0
No	No	Yes	No	No	No	No	0	0
Yes	Yes	No	No	No	Yes	Yes	1	0
Yes	Yes	Yes	No	No	Yes	No	0	0
Yes	Yes	Yes	No	No	Yes	No	2	0
Yes	Yes	Yes	No	No	No	No	0	0
No	No	Yes	No	No	Yes	No	0	0
Yes	Yes	Yes	No	No	No	No	0	0
Yes	Yes	Yes	No	No	Yes	No	4	0
No	No	Yes	No	No	Yes	No	2	7
No	No	No	No	No	Yes	Yes	6	0
No	No	Yes	No	No	Yes	No	8	0
Yes	Yes	Yes	No	No	No	No	0	0
No	No	No	No	No	Yes	No	3	0
Yes	Yes	Yes	No	No	No	No	0	0
No	No	Yes	No	No	Yes	No	2	0
Yes	Yes	No	No	No	Yes	No	1	3
Yes	No	Yes	No	No	Yes	No	15	0
Yes	Yes	Yes	No	No	Yes	Yes	0	0
Yes	Yes	Yes	No	No	No	No	0	0
Yes	Yes	Yes	No	No	Yes	Yes	12	13
Yes	No	Yes	No	No	Yes	No	4	0
Yes	Yes	Yes	No	No	No	No	0	0
No	No	Yes	No	No	Yes	Yes	0	0
Yes	Yes	Yes	No	No	Yes	No	3	0
Yes	Yes	Yes	No	No	Yes	No	1	0
Yes	Yes	Yes	Yes	No	No	No	0	0
No	Yes	No	No	No	No	No	0	0
Yes	Yes	Yes	No	No	No	No	0	0
Yes	Yes	Yes	No	No	Yes	No	0	0
Yes	Yes	Yes	No	No	No	No	0	0
Yes	Yes	Yes	No	No	Yes	No	0	0
Yes	Yes	Yes	No	Yes	No	No	0	0
No	No	Yes	No	No	Yes	No	3	0
Yes	Yes	Yes	No	Yes	Yes	No	10	0
Yes	No	Yes	No	No	Yes	No	6	0
Yes	Yes	Yes	No	No	Yes	Yes	5	0
Yes	Yes	Yes	No	No	Yes	No	3	0
Yes	Yes	Yes	No	No	Yes	No	6	11

For peer review only

q232c	q232d	q232e	q232f	q232g	q232h	q232i	q300hph	q300hch
15	6	0	1	0	3	4	40	80
0	0	0	0	0	2	1	25	60
0	2	0	1	0	2	2	60	120
0	1	0	0	0	2	2	60	90
0	0	0	1	0	3	3	15	60
0	0	0	0	0	4	2	30	120
0	0	0	1	0	2	3	60	120
0	0	0	0	0	0	0	90	60
1	0	1	1	0	1	3	60	90
3	0	0	1	0	1	2	50	60
0	0	0	1	0	1	2	60	90
2	0	0	0	0	2	2	15	60
3	0	0	1	0	0	0	30	60
0	1	0	1	0	2	2	10	60
0	0	0	0	0	0	0	40	60
0	0	0	1	0	1	2	15	60
0	0	0	0	0	2	2	40	60
1	0	0	1	0	2	3	60	120
0	0	0	0	0	0	1	10	60
2	0	0	1	0	1	2	50	50
0	0	0	0	0	0	0	60	90
0	0	0	0	0	0	0	60	120
1	2	0	0	0	1	3	30	60
0	0	8	0	0	1	2	40	120
13	0	0	0	0	6	3	10	60
1	0	1	1	0	1	2	40	60
0	0	0	1	0	3	4	60	120
4	0	0	0	0	0	2	30	60
0	0	0	1	0	2	3	15	60
3	0	0	0	0	2	2	90	60
1	0	0	1	0	1	2	50	90
0	0	0	0	0	2	2	15	60
0	0	0	0	0	1	3	5	60
0	0	0	0	0	0	1	40	50
1	1	0	0	0	2	2	50	90
2	0	0	1	0	1	4	40	60
0	0	0	0	0	2	2	90	120
0	0	0	0	0	3	4	90	120
0	0	0	1	0	3	2	60	120
0	0	0	1	0	3	2	50	70
0	0	0	1	0	2	2	60	120
3	0	0	1	0	5	4	50	60
0	0	0	1	0	3	3	60	120
2	0	0	0	0	2	2	90	60
0	0	0	0	0	2	2	40	60
0	0	0	0	0	2	2	40	60
0	0	0	0	0	0	2	15	60
0	1	0	0	0	2	4	40	90
0	0	0	0	0	0	0	10	60
0	0	0	0	0	2	2	25	60
0	0	0	0	0	2	2	60	40

For peer review only

0	0	0	8	0	4	3	40	60
0	0	0	1	0	3	2	90	120
0	4	0	1	0	3	3	15	60
1	2	0	0	0	6	2	40	80
1	0	0	1	0	3	3	20	60
0	1	0	0	0	0	2	15	60
0	0	0	0	0	0	2	60	120
0	0	0	0	0	2	2	60	60
0	0	0	0	0	1	1	30	60
0	0	0	2	0	2	2	50	90
0	0	0	0	0	0	0	50	60
4	0	0	1	0	2	3	50	90
5	0	0	1	0	3	2	30	60
1	2	0	0	0	2	2	15	60
0	0	0	0	0	3	1	60	80
0	0	0	0	0	0	2	30	60
0	0	0	0	0	2	2	30	60
0	0	0	0	0	1	2	90	60
0	0	0	0	0	1	2	5	40
0	0	0	0	0	1	2	40	90
0	0	0	1	0	1	2	25	60
0	1	0	0	0	2	2	15	60
0	0	0	2	0	2	2	60	120
0	0	0	0	0	0	0	60	60
0	0	0	0	0	0	2	30	60
0	0	0	1	0	2	4	15	60
4	0	0	1	0	2	3	50	90
0	0	0	1	0	0	3	40	60
0	8	0	0	0	1	2	40	90
0	0	0	0	0	2	2	60	60
0	0	0	1	0	1	5	60	120
2	0	0	0	0	2	1	90	60
0	0	0	0	0	0	3	40	60
0	0	0	0	0	4	2	40	60
0	0	0	0	0	2	2	40	60
5	2	0	0	0	1	2	20	60
0	0	0	0	0	1	1	60	40
0	0	0	0	0	1	3	25	60
0	0	0	1	0	4	2	20	60
2	0	0	0	0	0	3	60	90
0	0	0	0	0	3	2	10	60
0	0	0	1	0	4	3	60	120
0	0	0	0	0	3	3	60	60
0	0	0	2	0	2	2	60	120
0	0	0	1	0	2	2	60	60
0	0	0	0	0	0	0	30	60
0	0	0	0	0	4	2	40	90
0	0	0	0	0	1	2	30	60
0	0	0	0	0	0	2	120	60
0	0	0	0	0	2	2	60	60
0	0	0	0	0	0	0	60	40
14	0	0	0	0	1	2	40	90

For peer review only

0	0	0	0	0	1	2	50	60
0	0	0	0	0	3	2	20	60
0	0	0	1	0	2	2	60	120
0	0	0	1	0	2	2	60	90
0	0	0	1	0	1	2	50	90
0	0	0	0	0	0	0	90	60
0	0	0	0	0	3	3	40	60
0	0	0	0	0	1	2	40	60
0	0	0	1	0	0	2	90	60
0	0	0	1	0	2	3	60	120
0	1	0	0	0	0	4	5	60
0	0	0	1	0	2	2	40	60
0	0	0	1	0	2	2	60	120
5	0	0	1	0	2	3	15	60
4	0	0	1	0	1	4	5	60
10	2	0	1	0	3	2	40	60
0	0	0	0	0	1	2	15	60
0	0	0	1	0	10	2	60	120
1	0	0	1	0	0	3	30	80
0	0	0	1	0	2	2	60	120
0	0	0	1	0	0	2	40	90
0	0	0	1	0	2	2	40	90
0	0	8	0	0	4	2	60	60
0	0	0	0	0	0	1	40	60
0	2	0	0	0	2	1	15	60
4	1	0	0	0	1	2	30	60
6	0	0	1	0	2	3	15	60
6	0	0	0	0	0	2	30	60
0	0	0	0	0	2	2	60	90
4	0	0	0	0	0	2	50	60
2	0	0	0	0	1	2	40	15
0	0	0	0	0	1	1	40	120
0	0	0	0	0	1	1	40	80
5	0	0	0	0	2	4	30	40
7	0	0	0	0	2	2	90	90
3	1	0	0	0	1	2	30	60
10	1	0	0	0	1	1	30	60
0	0	0	0	0	0	2	40	120
0	1	0	0	0	1	1	30	40
0	0	0	0	0	2	2	30	60
4	0	0	0	0	1	2	60	90
3	1	0	0	0	1	2	40	90
7	1	0	0	0	0	2	20	60
0	1	0	0	0	3	2	10	40
0	1	0	0	0	1	3	30	30
4	0	0	0	0	0	0	30	60
2	0	0	0	0	0	1	10	60
2	0	0	0	0	1	2	30	50
3	1	0	0	0	2	2	40	90
2	0	0	0	0	2	2	60	60
5	2	0	0	0	2	2	5	60
4	1	0	0	0	0	2	40	70

For peer review only

4	1	0	0	0	1	2	30	60
4	1	0	0	0	2	2	40	120
0	0	0	0	0	1	2	40	90
0	0	0	0	0	0	2	40	60
0	0	0	0	0	1	2	40	120
4	1	0	0	0	1	2	30	60
0	1	0	1	0	2	3	20	60
5	1	0	0	0	1	2	60	90
3	1	0	0	0	2	2	30	60
13	1	0	0	0	2	3	10	60
2	0	0	0	0	2	2	60	90
3	1	0	0	0	0	2	40	70
0	0	0	0	0	1	2	60	90
3	1	0	0	0	1	2	30	60
4	2	0	0	0	2	2	30	60
5	1	0	0	0	2	2	40	60
3	1	0	0	0	0	2	30	70
2	0	0	0	0	1	2	25	30
5	0	1	0	0	2	2	30	60
0	1	0	0	0	1	2	40	90
4	1	0	0	0	2	2	40	90
0	0	0	0	0	2	3	40	60
0	1	0	0	0	0	2	30	40
0	0	0	0	0	2	2	60	90
0	0	0	0	0	0	0	40	15
4	1	0	0	0	0	2	40	90
0	1	0	0	0	0	3	20	60
0	2	0	0	0	2	1	50	70
4	1	0	0	0	1	2	30	40
0	2	0	0	0	2	3	30	40
2	0	0	0	0	0	2	30	60
8	0	0	1	0	1	2	30	60
3	0	0	0	0	0	2	30	25
0	0	0	0	0	1	2	30	25
0	0	0	0	0	0	1	40	60
3	3	0	0	0	2	3	30	30
2	1	0	0	0	2	2	10	60
0	0	0	0	0	1	2	40	60
8	0	0	0	0	1	2	10	40
4	0	0	0	0	0	3	30	40
2	1	0	0	0	1	2	40	60
0	0	0	0	0	1	2	30	45
0	0	0	0	0	0	0	50	40
4	0	0	0	0	1	2	60	90
0	0	0	0	0	1	2	30	40
0	1	0	0	0	1	2	30	60
2	1	0	0	0	0	3	60	90
0	1	0	0	0	1	2	40	90
0	0	0	0	0	2	2	30	40
0	1	0	0	0	0	4	30	60
0	1	1	0	0	1	2	30	80
3	1	0	0	0	2	2	40	90

For peer review only

For peer review only - http://bmjopen.bmj.com/site/about/guidelines.xhtml

4	0	0	0	0	0	0	30	60
2	1	0	0	0	1	1	60	90
0	0	0	0	0	2	2	15	60
0	0	0	0	0	0	0	40	60
0	1	0	0	0	0	2	15	60
0	0	0	0	0	0	0	20	60
0	0	0	0	0	0	2	30	60
0	1	0	0	0	3	2	25	60
0	0	0	0	0	1	3	5	80
0	2	0	0	0	1	2	25	60
0	0	0	0	0	1	3	5	60
0	1	0	0	0	0	2	15	60
0	1	0	0	0	0	2	10	60
0	0	0	0	0	2	4	60	20
0	1	0	0	0	2	3	25	60
0	1	0	0	0	0	2	25	60
0	0	0	0	0	1	2	60	90
0	0	0	0	0	2	1	60	30
0	1	0	0	0	2	2	50	90
0	0	0	0	0	2	2	90	40
0	0	0	0	0	2	2	40	90
0	0	0	0	0	2	2	10	80
0	0	0	0	0	1	2	40	90
0	0	0	0	0	2	1	60	30
0	0	0	0	0	0	0	60	60
0	0	0	0	0	0	0	30	120
0	0	0	0	0	2	2	60	90
0	0	0	0	0	0	0	40	90
0	0	0	0	0	0	0	5	60
0	1	0	0	0	2	2	5	30
0	2	0	0	0	2	2	30	60
0	0	0	0	0	0	2	10	60
0	0	0	0	0	0	2	20	90
0	0	0	0	0	1	2	90	60
0	0	0	0	0	2	2	60	30
0	0	0	0	0	0	0	5	30
0	0	0	0	0	0	2	40	90
0	1	0	0	0	1	5	15	60
0	1	0	0	0	2	2	50	110
0	1	0	0	0	0	0	5	30
0	0	0	0	0	1	2	90	60
0	0	0	0	0	0	0	30	120
0	1	0	0	0	2	2	50	110
0	1	0	0	0	1	2	5	80
0	1	0	0	0	3	2	30	80
0	0	0	0	0	0	0	30	60
0	1	0	0	0	2	3	15	60
1	1	0	0	0	3	3	30	60
0	0	0	0	0	1	2	60	20
0	1	0	1	0	3	2	25	60
0	0	0	0	0	0	2	50	110
0	0	0	0	0	0	0	5	80

For peer review only

0	2	0	0	0	2	2	5	60
0	0	0	0	0	2	2	15	60
0	1	0	0	0	0	2	30	60
0	0	0	0	0	0	2	40	90
0	0	0	0	0	0	1	30	60
4	0	0	0	0	1	3	5	60
0	0	0	0	0	0	0	5	80
2	1	0	0	0	0	3	5	80
0	2	0	0	0	0	3	25	60
2	0	0	0	0	1	2	5	60
0	0	0	0	0	0	0	5	80
0	0	0	0	0	1	1	30	80
0	1	0	0	0	1	2	15	60
5	1	0	0	0	1	3	5	60
0	0	0	0	0	1	2	40	60
0	0	0	0	0	1	2	50	90
0	1	0	0	0	0	2	15	60
0	1	0	0	0	1	2	60	120
0	0	0	0	0	1	2	60	30
0	8	0	0	0	1	1	60	60
0	0	0	0	0	0	0	30	80
0	0	0	0	0	0	1	30	60
0	0	0	0	0	0	0	5	68
0	0	0	0	0	0	0	5	80
0	0	0	0	0	0	2	5	60
2	1	0	0	0	1	2	5	60
0	0	0	0	0	0	2	50	110
1	3	0	0	0	0	2	25	60
0	1	0	0	0	4	3	30	60
0	1	0	0	0	0	3	30	90
0	0	0	0	0	1	1	60	40
0	2	0	0	0	0	2	15	60
0	0	0	0	0	2	3	90	50
0	1	0	0	0	2	3	30	80
0	0	0	0	0	0	0	30	120
0	1	0	0	0	10	4	5	80
0	0	0	0	0	1	1	5	60
0	0	0	0	0	1	1	50	110
8	1	0	0	0	3	4	30	90
0	0	0	0	0	0	0	90	60
0	1	0	0	0	1	3	5	80
5	0	0	0	0	1	2	5	60
0	0	0	0	0	0	0	5	80
0	0	0	0	0	2	3	60	60
0	1	0	0	0	1	2	50	110
0	0	0	0	0	1	2	20	90
0	2	0	0	0	1	3	40	90
0	1	0	0	0	2	3	40	90
0	0	0	0	0	1	1	5	30
0	0	0	0	0	1	2	5	30
0	0	0	0	0	0	0	60	60
0	0	0	0	0	1	1	30	90

For peer review only

0	0	0	1	0	1	2	40	120
0	0	0	0	0	1	2	60	90
0	1	0	0	0	1	2	30	80
0	0	0	0	0	1	2	40	90
0	1	0	0	0	0	2	15	60
0	1	0	0	0	0	2	5	80
0	1	0	0	0	1	3	30	60
0	1	0	0	0	3	2	5	80
0	0	0	0	0	1	1	5	80
0	0	0	0	0	2	3	60	20
0	0	0	0	0	0	0	60	30
0	0	0	2	0	1	0	20	80
6	1	0	0	0	1	3	5	60
5	1	0	0	0	3	2	30	80
0	0	0	0	0	0	1	50	110
0	0	0	0	0	0	0	5	80
0	1	0	0	0	0	2	15	60
0	0	0	0	0	0	0	90	40
0	0	0	0	0	1	2	5	60
0	0	0	0	0	0	1	30	80
0	1	0	0	0	2	3	30	90
0	1	0	0	0	0	3	30	60
0	1	0	0	0	0	2	5	80
0	0	0	1	0	1	2	15	60
0	0	0	0	0	0	0	15	60
0	0	0	0	0	0	2	30	60
0	1	0	0	0	0	1	50	110
0	0	0	0	0	2	2	60	40
0	0	0	0	0	0	0	5	80
0	1	0	0	0	1	1	5	30
2	1	0	0	0	2	2	40	180
3	1	0	0	0	0	3	40	120
0	0	0	0	0	0	0	40	180
2	1	0	0	0	3	2	40	180
0	2	0	0	0	1	1	40	180
1	0	0	0	0	2	2	15	120
0	0	0	0	0	2	2	40	180
0	1	0	0	0	1	2	25	90
6	1	0	0	0	3	2	40	180
1	1	0	0	0	0	2	40	30
0	0	0	0	0	0	0	40	180
1	1	0	0	0	2	2	40	180
0	1	0	0	0	1	2	40	180
0	1	0	0	0	1	2	40	180
0	0	0	0	0	1	0	40	180
2	1	0	0	0	0	2	40	180
3	2	0	0	0	2	3	40	180
5	0	0	0	0	0	2	60	180
3	1	0	0	0	1	2	30	90
5	1	0	0	0	1	2	30	180
0	1	0	0	0	2	2	40	180
0	1	0	0	0	2	3	35	80

For peer review only

0	0	0	0	0	1	1	40	180
0	1	0	0	0	1	1	40	180
0	1	0	0	0	1	1	30	180
0	1	0	0	0	1	2	10	120
7	1	0	0	0	2	4	30	180
0	0	0	0	0	1	2	40	180
0	0	0	0	0	1	1	5	120
4	0	0	0	0	1	1	30	60
0	1	0	0	0	1	3	25	60
0	1	0	0	0	0	2	40	180
0	1	0	0	0	2	1	40	180
0	0	0	0	0	1	2	5	120
0	1	0	0	0	1	2	30	180
0	1	0	0	0	1	2	20	90
2	1	0	0	0	1	2	40	180
0	1	0	0	0	0	2	10	60
0	1	0	0	0	2	3	40	180
0	0	0	0	0	1	2	40	180
0	1	0	0	0	1	2	60	180
0	0	0	1	0	1	4	90	120
0	0	0	0	0	0	0	15	60
0	0	0	0	0	2	2	10	60
2	2	0	0	0	2	2	20	60
3	2	0	0	0	1	2	30	90
0	0	0	2	0	2	3	15	60
0	0	8	0	0	1	1	50	60
0	0	0	0	0	2	0	40	60
0	0	0	1	0	2	2	15	60
4	1	0	0	0	2	2	30	90
0	1	0	0	0	0	0	10	60
0	1	0	0	0	2	2	30	60
0	1	0	0	0	1	2	20	60
2	1	0	0	0	1	3	30	90
15	2	0	0	0	3	6	15	80
4	0	0	0	0	1	1	5	40
5	2	0	0	0	2	3	60	90
5	2	0	0	0	2	2	15	90
4	2	0	0	0	2	2	30	60
0	1	0	0	0	1	4	10	60
2	1	0	0	0	2	3	30	60
1	0	0	0	0	2	3	40	60
0	0	0	0	0	2	1	40	40
0	2	0	0	0	2	5	30	60
0	1	0	0	0	0	0	30	90
0	0	0	0	0	1	2	60	90
0	0	0	0	0	1	2	40	60
10	0	0	0	0	1	3	60	90
0	0	0	0	0	2	2	10	60
0	1	0	0	0	2	2	60	120
2	0	0	0	0	0	2	20	120
0	2	0	0	0	1	2	20	90
6	1	0	0	0	2	2	30	45

For peer review only

0	0	0	0	0	1	2	10	60
0	1	0	0	0	2	2	20	60
0	3	0	2	0	3	3	25	60
0	0	0	0	0	2	2	15	60
5	1	0	1	0	1	4	30	60
0	1	0	0	0	2	3	60	190
0	0	0	0	0	0	0	5	180
0	1	0	0	0	0	2	10	180
0	0	0	0	8	2	0	10	5
0	0	0	0	0	0	0	3	180
6	0	1	0	0	0	2	25	120
0	0	0	0	0	1	2	90	180
10	1	0	0	0	1	2	60	180
0	0	0	0	0	0	0	5	180
0	1	0	0	0	1	2	20	120
5	0	0	0	0	1	2	25	120
0	1	0	0	0	2	3	90	180
0	0	0	0	0	0	0	20	180
0	0	0	0	0	1	2	90	180
3	1	0	0	0	1	2	15	120
3	1	0	0	0	2	2	15	120
0	0	0	0	0	0	0	60	180
4	0	0	0	0	0	1	40	260
0	0	0	0	0	0	0	30	180
0	0	0	0	0	0	1	1	180
4	0	1	0	0	0	3	40	260
0	1	0	0	0	2	2	120	180
0	0	0	0	0	0	0	2	180
0	0	0	0	0	0	0	5	60
0	0	0	0	0	1	2	90	180
0	0	0	0	0	0	1	15	120
8	1	0	0	0	1	1	120	180
0	0	0	0	0	0	0	3	180
5	1	0	0	0	1	2	25	180
4	0	0	0	0	0	1	50	260
4	1	0	0	0	0	1	15	120
3	1	0	0	0	1	3	50	240
2	1	0	0	0	1	2	25	120
0	0	0	0	0	0	0	30	180
0	0	0	0	0	2	1	20	180
0	0	0	0	0	0	2	60	150
0	0	0	0	0	2	2	90	180
0	0	0	0	0	0	0	5	180
2	1	0	0	0	1	2	20	120
2	0	0	0	0	0	3	30	240
0	0	0	0	0	1	1	30	180
0	1	0	0	0	2	2	25	120
0	1	0	0	0	2	2	120	180
0	0	0	0	0	2	2	40	240
0	0	0	0	0	1	2	15	120
0	1	0	0	0	1	1	25	120
0	0	0	0	0	2	2	30	180

For peer review only

0	0	0	0	0	1	1	50	260
18	1	0	0	0	1	2	60	180
3	1	0	0	0	1	2	15	120
0	0	0	0	0	0	0	60	180
0	0	0	0	0	0	1	25	120
2	1	0	0	0	1	2	20	120
2	0	0	0	0	1	2	50	240
4	0	1	0	0	2	2	15	120
0	1	0	0	0	2	3	30	180
0	0	0	0	1	3	1	10	180
0	0	0	0	0	0	1	5	180
4	0	0	0	0	1	1	20	180
0	0	0	0	0	0	0	5	60
0	0	0	0	0	0	0	2	180
0	0	0	0	0	0	0	20	120
0	0	0	0	0	0	0	15	240
2	0	0	0	0	0	2	40	240
0	1	0	0	0	2	2	15	120
0	0	0	0	0	0	0	3	60
40	4	0	0	0	3	9	30	120
0	0	0	0	0	0	0	3	65
0	2	0	0	0	1	5	30	150
0	2	0	0	0	1	4	20	125
0	0	0	0	0	0	0	5	125
0	1	0	0	0	0	4	45	240
0	1	0	0	0	1	0	5	60
0	1	0	0	0	0	2	15	120
0	1	0	0	0	0	2	15	120
0	0	0	0	0	0	0	35	120
0	1	0	0	0	0	1	10	120
8	2	0	0	0	2	2	20	130
0	0	0	0	0	0	2	45	245
0	1	0	0	0	0	0	15	60
0	0	0	0	0	2	2	15	120
15	3	0	0	0	2	4	5	65
0	4	0	0	0	0	4	20	120
3	2	0	0	0	3	5	25	150
0	3	0	0	0	4	2	20	125
0	1	0	0	0	1	2	60	240
0	1	0	0	0	0	2	5	120
0	2	0	0	0	1	3	60	115
0	0	0	0	0	0	3	20	90
0	4	0	0	0	3	2	15	130
6	1	0	0	0	2	2	20	90
6	2	0	0	0	2	5	35	150
0	1	0	0	0	0	2	30	120
0	1	0	0	0	0	2	45	145
2	1	0	0	0	2	4	20	90
0	1	0	0	0	1	2	20	120
0	2	0	0	0	1	4	30	120
0	0	0	0	0	0	0	5	90
4	1	0	0	0	2	3	30	120

For peer review only

0	0	0	0	0	0	0	60	180
0	0	0	0	0	0	0	5	120
0	1	0	0	0	0	3	20	120
0	1	0	0	0	0	2	45	260
0	0	0	0	0	0	0	10	120
0	1	0	0	0	2	4	25	120
0	1	0	0	0	1	4	50	240
0	1	0	0	0	0	0	30	120
4	1	0	0	0	3	6	30	120
0	2	0	0	0	4	4	10	120
0	1	0	0	0	2	3	30	120
0	3	0	0	0	0	2	40	240
0	0	0	0	0	0	4	45	120
0	0	0	0	0	0	0	20	120
0	1	0	0	0	0	2	5	90
0	0	0	0	0	0	0	5	120
0	0	0	0	0	0	0	5	110
0	1	0	0	0	0	4	20	160
5	1	0	0	0	1	3	30	120
0	1	0	0	0	0	2	50	245
0	0	0	0	0	0	0	15	125
3	3	0	0	0	0	0	90	300
0	0	0	0	0	0	0	30	20
4	1	0	0	0	1	3	90	300
0	0	0	0	0	0	0	20	30
0	0	0	0	0	0	0	15	15
4	0	0	0	0	1	1	5	300
0	0	0	0	0	0	0	50	50
0	0	0	0	0	0	0	5	280
0	0	0	0	0	0	0	25	25
0	0	0	0	0	0	0	10	20
0	0	0	0	0	0	0	5	300
2	1	0	0	0	0	2	15	15
5	2	0	0	0	2	2	5	260
2	1	0	0	0	0	0	50	180
12	0	0	0	0	1	2	15	15
0	0	0	0	0	0	9	30	180
5	1	0	0	0	0	2	30	30
8	2	0	0	0	1	4	60	180
0	0	0	0	0	0	0	30	20
0	0	0	0	0	0	0	45	180
15	2	0	1	0	0	2	60	60
2	1	0	0	0	2	0	5	260
7	0	0	0	0	0	2	30	30
6	0	0	0	0	0	0	5	59
0	2	0	0	0	0	3	40	180
0	0	0	0	0	0	0	20	20
0	0	0	0	0	0	0	45	180
0	1	0	0	0	0	3	6	270
9	0	0	0	0	0	2	15	15
0	2	0	0	0	0	0	6	290
0	2	0	0	0	0	0	80	250

For peer review only

0	0	0	0	0	0	0	7	280
0	0	0	0	0	0	0	20	10
0	0	0	0	0	0	0	5	59
0	0	0	0	0	0	0	10	20
0	2	0	0	0	2	0	5	280
5	0	0	0	0	1	2	15	15
9	0	0	0	0	1	2	20	20
0	0	0	0	0	0	0	10	10
0	0	0	0	0	0	0	30	20
0	0	0	0	0	0	0	10	10
3	0	0	0	0	0	2	30	30
0	0	0	0	0	0	0	30	20
0	1	0	0	0	0	2	30	180
3	1	0	0	0	1	2	5	280
0	0	0	0	0	0	0	5	10
0	0	0	0	0	0	0	20	30
0	0	0	0	0	0	0	5	10
0	0	0	0	0	0	0	30	180
6	2	0	0	0	2	2	40	40
9	1	0	0	0	1	2	30	30
2	0	0	0	0	1	3	5	10
0	1	0	0	0	0	2	15	15
0	0	0	0	0	0	0	5	59
6	0	0	0	0	0	0	10	15
0	0	0	0	0	0	2	15	280
0	0	0	0	0	1	1	30	30
0	2	0	0	0	0	0	95	305
3	1	0	0	0	1	2	85	295
4	0	0	0	0	1	2	30	30
0	1	0	0	0	2	2	40	40
0	2	0	0	0	2	3	60	180
0	2	0	0	0	0	0	80	280
0	0	0	0	0	0	0	50	50
0	0	0	0	0	2	5	20	180
4	2	0	0	0	2	3	6	290
2	1	0	0	0	1	3	60	180
6	0	0	0	0	1	2	30	30
0	0	0	0	0	0	0	10	20
0	0	0	0	0	0	0	10	20
0	0	0	0	0	0	0	5	10
0	0	0	0	0	0	0	30	180
1	2	0	0	0	0	0	100	310
0	0	0	0	0	0	0	5	260
5	2	0	0	0	2	0	60	180
0	0	0	0	0	0	0	50	50
3	0	0	0	0	0	0	15	15
0	0	0	0	0	0	0	10	30
6	1	0	0	0	1	2	20	20
5	0	0	0	0	1	2	20	20
0	0	0	0	0	0	0	20	30
8	1	0	0	0	1	2	30	30
8	0	0	0	0	2	2	30	30

For peer review only

4	1	0	0	0	1	4	60	180
0	0	0	0	0	0	0	90	300
2	0	0	0	0	0	0	45	180
2	1	0	0	0	0	3	30	180
0	1	0	0	0	0	2	30	30
6	3	0	0	0	1	3	60	180
8	2	0	0	0	1	2	90	300
0	2	0	0	0	2	2	90	300
0	0	0	0	0	0	3	5	280
3	1	0	0	0	1	0	10	10
3	2	0	0	0	0	2	95	305
4	1	2	1	0	1	2	15	15
0	1	0	0	0	1	2	60	180
0	0	0	0	0	0	0	60	180
0	0	0	0	0	0	2	30	30
0	0	0	0	0	0	0	40	180
3	1	0	0	0	3	4	60	180
3	1	0	0	0	4	8	20	180
0	1	0	0	0	1	3	5	280
0	0	0	0	0	2	0	10	15
0	3	0	0	0	0	2	5	260
2	0	0	0	0	1	2	15	15
6	2	0	0	0	0	0	5	280
0	0	0	0	0	0	0	20	30
3	2	0	0	0	0	5	30	180
0	0	0	0	0	0	0	20	180
0	0	0	0	0	0	2	40	40
5	0	0	0	0	0	2	10	10
0	0	0	0	0	2	2	20	180
0	0	0	0	0	0	0	30	180
0	0	0	0	0	1	0	30	30
4	0	0	0	0	2	2	60	60
4	1	0	0	0	2	1	40	40
4	0	0	0	0	2	2	30	30
2	0	0	0	0	2	1	60	60
0	1	0	0	0	1	2	120	120
6	1	0	0	0	2	2	45	45
4	0	0	0	0	3	2	30	30
5	0	0	0	0	2	0	40	40
2	1	0	0	0	3	2	50	50
0	1	0	0	0	1	1	5	5
0	1	0	0	0	1	3	40	40
0	0	0	0	0	0	2	5	5
0	1	0	0	0	0	2	45	45
0	0	0	1	0	1	1	10	10
4	1	0	0	0	1	1	50	50
0	0	0	0	0	0	0	30	30
0	1	0	0	0	2	3	30	30
4	1	0	0	0	0	2	45	45
3	0	0	1	0	1	2	15	15
5	2	0	0	0	2	3	60	60
0	0	0	0	0	2	2	25	25

0	1	0	0	0	1	2	180	180
10	2	0	0	0	2	2	60	60
2	0	0	0	0	1	3	10	10
2	2	0	0	0	1	2	30	30
0	1	0	0	0	4	4	60	60
3	1	0	0	0	0	2	60	60
4	1	0	0	0	2	4	20	20
7	1	0	0	0	1	3	40	40
0	0	0	0	0	0	2	10	10
2	2	0	0	0	1	2	30	30
0	1	0	0	0	2	2	30	30
0	1	0	0	0	1	3	10	10
2	2	0	0	0	2	2	60	60
0	1	0	0	0	1	1	30	30
7	0	0	1	0	4	2	60	60
0	0	0	0	0	0	2	10	10
3	0	0	0	0	1	2	30	30
0	0	0	0	0	1	4	15	15
0	0	0	1	0	0	1	10	10
0	0	0	0	0	2	2	40	40
6	1	0	0	0	2	1	5	5
0	1	0	0	0	1	2	40	120
2	1	0	0	0	1	2	30	30
10	1	0	0	0	2	4	30	30
4	0	0	0	0	0	1	10	10
8	1	0	0	0	2	4	30	30
3	2	0	1	0	2	2	30	30
0	0	0	0	0	0	1	240	240
8	1	0	0	0	1	3	45	45
0	0	0	0	0	0	0	60	60
2	0	0	0	0	1	1	60	60
4	1	0	0	0	2	2	60	60
0	1	0	0	0	2	0	60	60
0	0	0	0	0	0	2	60	60
0	1	0	0	0	1	3	50	50
2	1	0	0	0	1	1	30	30
0	0	0	0	0	0	2	60	60
4	2	0	0	0	1	2	40	40
0	1	0	0	0	3	1	30	30
0	0	0	1	0	2	2	30	30
0	1	0	0	0	2	2	10	10
0	1	0	0	0	2	3	20	20
8	0	0	0	0	1	1	60	60
0	0	0	0	0	1	1	30	30
0	0	0	0	0	1	2	90	180
3	1	0	0	0	1	4	60	60
0	1	0	0	0	1	2	30	30
3	1	0	0	0	1	4	60	60
0	0	0	0	0	0	2	45	45
0	0	0	0	0	0	0	7	7
5	1	0	0	0	1	2	20	20
0	0	0	0	0	1	1	25	25

For peer review only

0	0	0	0	0	1	2	10	10
6	3	0	0	0	0	2	30	30
0	0	0	0	0	1	2	10	10
7	1	0	0	0	0	3	60	60
15	1	0	0	0	0	2	30	30
0	0	0	0	0	0	0	30	180
0	1	0	0	0	0	2	35	35
0	0	0	0	0	3	3	30	30
0	1	0	0	0	1	1	60	60
0	1	0	0	0	1	3	10	10
0	0	0	0	0	0	1	30	30
5	0	0	0	0	0	2	40	40
0	0	0	0	0	2	2	20	180
4	2	0	0	0	2	3	60	60
0	1	0	0	0	1	2	30	30
3	0	0	0	0	0	1	30	30
6	0	0	0	0	1	3	5	5
3	1	0	0	0	2	3	40	40
2	1	0	0	0	2	2	30	30
0	0	0	0	0	0	0	10	10
8	1	0	0	0	1	3	60	60
2	0	0	0	0	1	2	40	40
0	0	0	0	0	2	2	60	60
2	0	0	0	0	0	2	20	20
0	1	0	0	0	1	2	120	120
0	1	0	0	0	2	4	30	30
3	1	0	0	0	1	1	5	5
2	1	0	0	0	0	1	25	25
2	1	0	0	0	1	2	45	45
0	0	0	0	0	2	2	5	60
0	0	0	0	0	0	0	120	120
4	1	0	0	0	2	3	60	60
0	1	0	0	0	1	2	90	90
0	0	0	0	0	0	0	30	30
1	0	0	0	0	1	1	30	30
6	1	0	0	0	3	2	50	50
0	1	0	0	0	3	2	30	30
0	1	0	0	0	0	2	30	30
3	1	0	0	0	2	3	30	30
0	0	0	0	0	0	0	2	2
4	1	0	0	0	1	1	25	25
0	0	0	0	0	0	0	3	3
0	0	0	0	0	1	1	60	60
0	0	0	0	0	0	0	25	100
0	2	0	0	0	1	3	15	60
0	0	0	0	0	0	0	25	60
6	0	0	0	0	2	4	15	60
0	0	0	0	0	0	0	10	60
3	0	0	0	0	0	3	15	120
0	0	0	0	0	0	0	20	120
0	0	0	0	0	0	0	10	60
0	0	0	0	0	0	0	15	60

For peer review only

0	0	0	0	0	0	0	15	120
0	0	0	0	0	0	0	20	60
0	0	0	0	0	0	0	30	60
0	0	0	0	0	0	0	20	60
0	1	0	0	0	2	4	10	30
0	0	0	0	0	0	0	20	60
0	0	0	0	0	0	0	20	60
0	0	0	0	0	0	0	10	40
0	0	0	0	0	0	1	10	60
0	0	0	0	0	0	0	15	45
0	0	0	0	0	0	0	20	60
0	0	0	0	0	0	0	10	30
0	0	0	0	0	0	0	20	120
0	0	0	0	0	0	0	10	60
0	0	0	0	0	0	1	40	120
0	0	0	0	0	0	0	20	90
0	0	0	0	0	0	0	25	120
0	0	0	0	0	0	0	20	60
0	0	0	0	0	0	0	10	165
0	0	0	0	0	0	0	25	80
0	0	0	0	0	0	0	15	30
0	0	0	0	0	0	0	20	60
0	0	0	0	0	0	0	25	120
0	1	0	0	0	0	2	10	65
0	0	0	0	0	0	0	25	90
0	0	0	0	0	0	0	20	60
0	0	0	0	0	0	0	15	90
0	0	0	0	0	0	0	30	90
0	0	0	0	0	3	3	30	90
8	0	0	0	0	1	1	5	60
0	0	0	0	0	0	0	30	90
0	0	0	0	0	0	0	15	60
0	0	0	0	0	0	0	15	60
0	0	0	0	0	0	0	20	120
0	0	0	0	0	0	0	30	80
0	0	0	0	0	0	0	20	125
0	0	0	0	0	0	0	15	45
2	0	0	0	0	2	2	20	60
0	0	0	0	0	0	0	5	125
0	0	0	0	0	0	0	10	120
0	0	0	0	0	0	0	30	90
0	0	0	0	0	0	0	30	120
0	0	0	0	0	0	0	30	60
6	2	0	0	0	3	2	15	150
0	0	0	0	0	1	0	30	90
0	0	0	0	0	0	0	20	130
0	0	0	0	0	0	0	15	120
0	0	0	0	0	0	0	25	80
0	0	0	0	0	0	0	15	120
3	4	0	0	0	3	2	15	60
0	0	0	0	0	0	0	30	60
0	0	0	0	0	0	0	15	60

For peer review only

0	0	0	0	0	0	0	20	60
0	0	0	0	0	0	0	5	60
0	0	0	0	0	0	0	20	40
0	0	0	0	0	0	0	25	60
0	0	0	0	0	0	0	15	60
0	0	0	0	0	0	0	25	100
0	0	0	0	0	0	0	25	120
0	0	0	0	0	0	0	15	45
0	0	0	0	0	0	0	20	90
0	0	0	0	0	0	0	15	90
0	0	0	0	0	0	0	20	60
0	0	0	0	0	0	0	20	60
0	0	0	0	0	0	0	10	60
0	0	0	0	0	0	0	30	90
0	1	0	0	0	1	2	15	90
4	4	0	0	0	0	4	20	60
0	0	0	0	0	0	0	10	90
0	0	0	0	0	0	0	15	60
0	0	0	0	0	1	1	15	120
0	0	0	0	0	2	0	6	40
12	1	0	0	0	0	1	30	60
1	1	0	0	0	1	2	30	120
0	0	0	0	0	0	0	20	60
0	0	0	0	0	0	0	10	60
0	1	0	0	0	1	2	5	300
0	0	0	0	0	0	2	30	60
0	0	0	0	0	0	0	5	120
0	0	0	1	0	3	2	10	60
0	1	1	1	0	1	4	40	120
1	1	0	1	0	1	2	20	300
0	1	0	0	0	1	3	5	180
1	0	0	0	0	2	4	15	90
0	0	0	0	0	3	3	5	60
0	0	0	0	0	0	2	5	120
0	1	0	0	0	2	3	180	180
0	0	0	0	0	0	2	40	220
0	5	0	0	0	2	2	5	300
0	1	0	0	0	1	2	20	300
2	0	0	0	0	0	0	5	60
0	0	0	0	0	1	2	45	225
0	1	0	0	0	0	2	25	60
1	1	0	0	0	3	4	10	60
0	0	0	0	0	0	1	5	60
0	0	0	1	0	1	2	20	90
2	1	0	0	0	3	4	10	300
0	0	0	0	0	0	0	10	120
0	0	0	0	0	2	6	2	100
0	0	0	0	0	0	0	30	60
0	6	0	0	0	0	0	60	360
0	0	0	0	0	0	0	5	60
1	1	0	0	0	2	5	180	180
0	1	0	0	0	1	2	45	225

For peer review only

2	0	0	0	0	2	2	30	120
0	0	0	0	0	0	2	30	60
0	2	0	0	0	4	5	15	180
0	1	0	0	0	2	2	60	180
0	0	0	1	0	1	2	5	30
0	1	0	0	0	1	2	5	80
0	3	0	0	0	0	0	60	360
0	0	0	0	0	1	4	40	120
3	1	0	0	0	4	7	15	300
0	0	0	0	0	4	5	120	180
0	0	0	0	0	1	2	40	225
0	0	0	0	0	3	1	15	70
0	1	0	0	0	4	5	20	300
0	1	0	0	0	0	0	20	300
0	0	0	0	0	3	2	5	180
0	1	0	0	0	0	1	15	300
0	0	0	0	0	0	0	5	30
0	2	0	0	0	4	3	20	300
0	0	0	0	0	2	2	20	90
0	1	0	0	0	2	2	25	60
3	1	0	0	0	2	3	35	200
0	1	0	0	0	0	2	20	300
4	2	0	0	0	0	8	120	240
0	1	0	0	0	0	2	20	300
0	4	0	0	0	0	0	60	360
0	1	0	0	0	0	2	10	300
0	2	0	0	0	0	2	10	300
0	1	0	0	0	1	2	10	120
0	1	0	0	0	3	2	5	300
0	1	0	0	0	3	4	5	300
0	1	0	0	0	1	1	10	90
0	1	0	0	0	1	3	60	240
0	1	0	0	0	2	2	15	300
0	2	0	0	0	1	0	20	300
0	1	0	0	0	2	3	5	300
0	2	0	0	0	3	5	10	90
2	1	0	0	1	1	3	120	120
1	0	0	0	0	1	1	5	60
0	0	0	0	0	0	1	40	120
0	0	0	0	0	0	0	20	90
2	1	0	0	0	2	3	10	300
0	0	0	0	0	1	3	3	120
0	3	0	0	0	0	0	60	360
0	2	0	0	0	0	3	10	300
0	4	0	0	0	2	3	20	300
0	1	0	0	0	3	4	15	300
0	0	0	0	0	0	0	5	180
0	0	0	0	0	0	0	20	120
0	1	0	0	0	1	3	45	125
0	1	0	0	0	1	3	10	300
0	1	0	0	0	3	3	10	60
0	2	0	0	0	2	4	3	128

For peer review only

0	0	0	0	0	0	0	5	60
0	0	0	0	0	0	0	30	120
0	0	0	0	0	0	3	5	90
10	1	0	0	0	1	4	120	180
3	1	0	0	0	1	2	10	120
1	1	0	0	0	1	3	10	300
1	0	0	0	0	0	0	5	300
0	0	0	0	0	0	2	40	120
0	4	0	0	0	0	0	60	360
4	2	0	0	0	2	3	120	240
0	0	0	0	0	2	4	15	110
0	2	0	0	0	3	4	120	180
0	1	0	0	0	2	6	120	240
0	1	0	0	0	2	3	20	300
0	1	0	0	0	0	0	60	360
0	1	0	0	0	1	2	15	300
0	0	0	0	0	0	2	4	120
0	1	0	0	0	2	5	25	80
0	0	0	0	0	0	1	10	120
0	0	0	0	0	0	2	5	60
0	0	0	0	0	1	3	45	225
2	1	0	0	0	1	3	45	225
0	1	0	0	0	1	3	20	300
0	1	0	0	0	4	3	40	120
1	1	0	0	0	1	3	120	240
0	0	0	0	0	0	0	5	180
0	1	0	0	0	2	2	20	90
0	0	0	0	0	0	3	30	60
0	1	0	0	0	1	2	10	120
2	1	0	0	0	2	2	40	240
0	2	0	0	0	1	6	10	60
0	1	0	0	0	2	2	10	300
0	0	0	1	0	0	0	5	300
0	0	0	0	0	2	2	20	90
0	1	0	0	0	1	3	10	60
0	0	0	0	0	2	3	180	180
0	1	0	0	0	2	2	20	90
0	0	0	0	0	2	2	60	360
0	2	0	0	0	0	3	10	60
0	1	0	0	0	2	3	30	120
0	1	0	0	0	1	2	10	90
0	1	0	0	0	3	3	10	300
0	1	0	0	0	3	4	5	160
0	0	0	0	0	2	3	10	100
0	0	0	0	0	0	0	5	60
0	0	0	0	0	0	1	5	60
0	1	0	0	0	1	4	180	180
1	0	0	0	0	3	1	15	300
1	2	0	0	0	1	3	15	80
0	0	0	0	0	0	0	2	100
2	1	0	0	0	1	3	30	180
0	0	0	0	0	2	2	30	120

For peer review only

0	1	0	0	0	0	2	20	300
0	0	0	0	0	2	2	10	300
2	1	0	0	0	0	2	60	180
0	0	0	0	0	2	2	15	40
3	2	0	0	0	3	4	180	180
0	0	0	0	0	2	2	5	120
0	2	0	0	0	0	0	5	180
0	0	0	0	0	0	0	10	60
0	2	0	0	0	2	5	5	180
0	1	0	0	0	2	2	5	60
0	1	0	0	0	0	2	10	300
0	2	0	0	0	1	3	180	180
4	2	0	0	0	0	0	180	180
0	1	0	0	0	0	1	20	300
0	0	0	0	0	0	0	6	60
0	1	0	0	0	2	1	15	120
0	0	0	0	0	1	2	15	300
2	1	0	0	0	3	4	10	300
0	3	0	0	0	0	2	10	300
0	0	0	0	0	1	3	5	120
0	1	0	0	0	0	5	25	80
0	2	0	0	0	0	0	60	360
0	1	0	0	0	1	4	40	120
1	1	0	0	0	3	4	15	300
0	1	0	0	0	2	4	10	300
0	0	0	0	0	0	0	60	360
0	2	0	0	0	3	5	120	240
1	1	0	0	0	1	3	10	300
0	1	0	0	0	2	2	45	225
0	0	0	0	0	2	2	5	300
0	1	0	0	0	0	2	30	60
0	0	0	0	0	0	1	5	60
2	0	0	0	0	2	4	5	300
0	1	0	0	0	2	2	30	120
0	1	0	0	0	1	2	25	90
0	0	0	0	0	0	1	30	120
0	0	0	0	0	0	0	60	180
0	0	0	0	0	3	2	10	180
0	0	0	0	0	0	0	15	180
2	1	0	0	0	1	6	60	180
0	0	0	0	0	3	2	10	120
0	0	0	0	0	0	0	95	225
0	1	0	0	0	0	2	95	215
0	0	0	0	0	0	0	15	180
0	0	0	0	0	0	0	95	235
0	0	0	0	0	1	0	20	180
0	1	0	0	0	3	7	60	180
0	0	0	0	0	2	4	15	180
0	0	0	0	0	2	4	10	90
3	0	0	1	0	1	1	15	180
0	0	0	0	0	2	2	10	120
0	0	0	0	0	0	0	95	215

For peer review only

1	0	0	0	0	0	0	10	180
2	1	0	0	0	2	4	15	180
0	0	0	0	0	0	0	10	180
0	0	0	0	0	0	0	15	180
0	0	0	0	0	1	2	5	60
0	0	0	1	0	2	3	20	180
0	0	0	0	0	0	2	10	180
0	0	0	0	0	2	3	20	120
0	0	0	0	0	0	0	3	60
0	2	0	0	0	0	0	15	120
0	0	0	0	0	0	0	20	180
0	1	0	0	0	1	3	95	215
0	0	0	0	0	0	1	10	120
0	1	0	0	0	1	3	95	215
0	0	0	0	0	4	3	10	120
0	0	0	0	0	0	0	12	180
0	0	0	0	0	1	1	5	180
1	0	0	0	0	2	0	5	120
0	1	0	0	0	3	3	60	180
0	0	0	0	0	0	0	5	60
0	0	0	0	0	0	3	10	120
0	0	0	0	0	1	2	95	225
0	1	0	0	0	3	2	60	180
0	0	0	0	0	0	0	95	215
0	0	0	0	0	3	0	5	60
0	0	0	0	0	0	0	15	120
0	0	0	0	0	1	2	4	120
0	0	0	0	0	0	0	5	60
0	0	0	0	0	2	2	15	120
2	1	0	0	0	1	3	95	215
0	1	0	0	0	1	2	95	235
0	1	0	0	0	3	5	60	180
0	0	0	0	0	0	2	20	120
0	1	0	0	0	3	3	10	120
0	0	0	0	0	0	0	2	60
0	1	0	0	0	1	3	15	90
1	1	0	0	0	2	3	60	180
0	0	0	0	0	1	3	95	215
1	0	0	0	0	2	4	10	120
0	0	0	0	0	1	1	60	180
0	0	0	0	0	2	2	30	180
0	0	0	1	0	2	3	10	120
0	1	0	0	0	1	3	95	215
0	0	0	0	0	0	1	10	180
0	0	0	0	0	0	0	15	60
0	0	0	0	0	0	0	5	180
0	0	0	0	0	0	0	15	120
0	0	0	0	0	0	0	15	180
0	1	0	0	0	0	2	60	180
0	0	0	0	0	2	3	15	180
0	0	0	0	0	0	0	5	60
0	0	0	0	0	1	2	15	120

For peer review only

0	0	0	0	0	3	3	60	180
0	0	0	0	0	0	2	20	180
0	0	0	0	0	2	2	15	120
1	1	0	0	0	2	4	60	180
0	0	0	0	0	0	0	10	60
0	1	0	0	0	3	4	60	180
0	0	0	0	0	0	0	10	180
2	0	0	0	0	1	3	25	90
0	0	0	0	0	1	3	95	225
0	0	0	0	0	1	2	60	180
0	0	0	0	0	1	2	15	120
0	0	0	0	0	0	0	5	60
0	1	0	0	0	1	2	5	120
0	1	0	0	0	1	2	95	215
2	0	0	0	0	1	2	95	225
0	0	0	0	0	0	0	10	90
0	1	0	0	0	1	2	10	120
0	0	0	0	0	4	2	5	180
0	1	0	0	0	1	0	60	180
0	0	0	0	0	1	3	20	180
0	0	0	0	0	1	2	30	180
0	0	0	0	0	1	2	15	120
0	1	0	0	0	1	3	15	120
0	2	0	0	0	0	0	5	120
0	1	0	0	0	0	3	10	180
0	1	0	0	0	2	4	60	180
2	1	0	0	0	3	3	60	180
0	0	0	0	0	0	0	15	180
0	0	0	0	0	0	0	10	180
0	0	0	0	0	1	2	15	180
0	0	0	0	0	0	3	15	20
0	0	0	0	0	0	1	5	90
0	0	0	0	0	0	0	10	120
2	1	0	0	0	2	2	10	60
0	0	0	0	0	0	0	10	60
0	0	0	0	0	0	0	5	120
0	0	0	0	0	0	0	5	90
0	0	0	0	0	0	0	20	180
0	1	0	0	0	3	4	60	180
0	1	0	0	0	3	4	60	60
0	0	0	0	0	0	0	95	225
0	1	0	0	0	3	3	60	180
3	0	0	0	0	2	3	20	60
0	0	0	0	0	0	0	15	180
0	0	0	0	0	0	0	10	180
0	1	0	0	0	2	2	60	180
0	0	0	0	0	0	0	5	90
0	0	0	0	0	1	2	15	180
0	1	0	0	0	0	0	10	180
0	1	0	0	0	1	2	15	60
0	2	0	0	0	2	2	20	60
1	4	0	0	0	1	2	60	160

For peer review only

0	0	0	0	0	0	1	20	60
0	0	0	0	0	0	4	50	90
2	1	0	0	0	2	2	60	160
0	0	0	0	0	0	1	15	60
0	0	0	1	0	2	3	60	90
0	0	0	0	0	0	2	30	60
0	1	0	0	0	0	2	15	45
0	0	0	0	0	2	2	20	60
0	0	0	0	0	2	2	10	40
0	0	0	0	0	2	3	40	90
0	0	0	0	0	1	1	60	160
0	1	0	0	0	1	2	15	60
0	1	0	0	0	0	2	5	60
3	0	0	0	0	0	2	5	40
0	1	0	0	0	0	2	15	60
0	0	0	1	0	2	2	60	120
0	0	0	0	0	0	0	60	90
0	1	0	0	0	1	2	60	160
0	0	0	0	0	0	0	25	60
0	2	0	0	0	1	2	20	60
2	1	0	0	0	0	2	5	40
0	0	0	0	0	0	0	60	90
0	1	0	0	0	1	2	10	60
0	1	0	0	0	1	3	30	60
0	2	0	0	0	2	2	60	160
0	1	0	0	0	2	2	15	60
0	3	0	0	0	3	4	15	60
0	0	0	0	0	0	0	15	60
0	0	0	0	0	2	1	60	120
0	2	0	0	0	2	4	10	60
0	2	0	0	0	3	2	15	60
0	1	0	0	0	0	2	10	60
0	1	0	0	0	2	4	60	160
2	0	0	0	0	1	1	40	90
0	0	0	0	0	0	2	30	60
0	1	0	1	0	0	2	30	180
2	1	0	0	0	0	3	15	60
0	0	0	0	0	0	2	20	60
0	0	0	1	0	3	2	60	90
0	1	0	0	0	1	2	60	160
0	1	0	0	0	2	4	10	60
0	0	0	1	0	0	2	15	60
0	0	0	0	0	0	0	40	90
0	0	0	0	0	2	2	40	90
0	1	0	0	0	1	2	60	160
0	0	0	1	0	2	2	60	90
0	1	0	0	0	3	4	10	40
0	1	0	0	0	0	1	15	60
2	1	0	0	0	1	2	15	60
0	0	0	0	0	0	0	25	60
0	0	0	0	0	1	2	20	60
0	3	0	0	0	1	4	30	50

For peer review only

2	0	0	0	0	2	1	90	120
0	0	0	0	0	0	0	15	60
0	1	0	0	0	1	2	15	60
0	0	0	0	0	0	2	10	60
0	1	0	1	0	3	2	10	40
0	0	0	0	0	0	2	20	60
0	0	0	0	0	1	2	30	60
0	0	0	1	0	3	2	60	90
0	0	0	0	0	0	2	50	90
0	2	0	0	0	0	3	10	40
0	1	0	0	0	3	2	30	60
0	0	0	0	0	0	0	50	90
0	1	0	0	0	1	3	15	60
0	1	0	0	0	1	2	15	60
0	1	0	0	0	2	2	15	60
1	1	0	0	0	2	2	60	160
0	1	0	0	0	1	2	10	60
0	1	0	1	0	4	4	15	60
3	1	0	0	0	1	2	30	60
0	0	0	0	0	1	2	15	60
0	0	0	0	0	1	2	60	160
0	1	0	0	0	0	2	60	90
0	1	0	0	0	1	3	30	60
0	2	0	0	0	0	2	20	60
0	1	0	0	0	1	4	60	120
0	1	0	0	0	1	3	15	60
0	0	0	0	0	1	1	10	60
0	1	0	0	0	3	3	10	60
0	0	0	0	0	1	2	60	120
0	1	0	0	0	1	2	15	60
0	3	0	0	0	2	4	30	60
8	2	0	1	0	0	4	15	60
0	0	0	0	0	1	2	15	60
0	1	0	0	0	1	2	10	60
1	2	0	0	0	1	4	60	160
0	0	0	0	0	1	2	25	60
0	0	0	0	0	2	4	20	60
0	0	0	0	0	0	0	5	40
0	0	0	0	0	1	2	15	60
0	1	0	0	0	1	3	60	160
0	0	0	0	0	2	3	40	90
0	0	0	0	0	0	0	40	120
0	0	0	0	0	0	0	15	60
0	1	0	1	0	3	4	50	90
0	0	0	0	0	2	2	40	120
0	1	0	0	0	1	2	15	60
2	1	0	0	0	2	2	20	60
0	0	0	0	0	2	2	60	120
0	1	0	0	0	2	1	5	40
0	0	0	0	0	0	0	10	40
0	0	0	1	0	1	2	20	60
0	1	0	0	0	1	2	5	40

0	2	0	0	0	1	2	60	90
0	1	0	0	0	2	3	60	160
0	0	0	0	0	1	2	60	120
0	0	0	0	0	0	0	60	90
2	1	0	0	0	2	4	60	160
0	1	0	0	0	5	3	10	40
0	1	0	0	0	1	4	10	60
0	0	0	0	0	0	0	60	90
0	1	0	0	0	2	3	60	160
0	1	0	0	0	0	2	20	60
2	0	0	0	0	2	3	40	120
0	0	0	0	0	0	2	15	60
0	2	0	0	0	1	2	70	180
0	1	0	0	0	1	2	25	40
0	2	0	0	0	0	2	20	60
0	1	0	0	0	0	0	30	50
0	1	0	0	0	0	2	10	30
2	1	0	0	0	1	1	15	150
0	0	0	0	0	0	0	40	30
0	0	0	0	0	0	0	15	60
0	1	0	0	0	0	1	45	120
0	1	0	0	0	0	1	5	60
0	0	0	0	0	0	0	25	60
4	2	0	0	0	1	3	30	120
5	3	0	0	0	2	6	60	120
0	1	0	0	0	1	1	45	120
12	0	1	0	0	0	0	60	20
0	2	1	0	0	0	2	5	60
4	2	0	0	0	1	1	30	45
0	1	0	0	0	0	2	3	45
0	0	0	0	0	0	0	25	60
0	1	0	0	0	0	2	10	150
2	2	0	0	0	1	2	30	150
4	1	0	0	0	0	0	10	10
5	1	0	0	0	0	0	10	30
8	2	0	0	0	2	4	60	240
0	1	0	0	0	0	2	30	180
0	0	0	0	0	0	0	25	40
0	1	0	0	0	0	2	2	120
2	2	0	0	0	3	3	10	60
0	1	0	0	0	0	1	25	40
0	0	0	0	0	0	0	15	120
3	2	0	0	0	1	2	40	180
0	2	0	0	0	1	1	15	15
0	0	0	0	0	0	0	5	60
11	1	0	0	0	0	0	15	40
0	0	0	0	0	0	0	20	15
0	1	0	0	0	1	4	30	50
2	1	0	0	0	2	2	60	180
4	0	1	0	0	1	1	20	50
0	2	0	0	0	0	4	15	60
0	1	0	0	0	1	2	60	180

For peer review only

For peer review only

0	2	0	0	0	2	1	30	50
5	0	0	0	0	1	1	30	50
0	0	0	0	0	0	0	30	60
0	0	0	0	0	0	0	20	90
0	1	1	0	0	4	4	60	30
0	0	0	0	0	0	0	30	90
0	0	3	0	0	0	0	60	30
2	0	0	0	0	1	2	40	110
0	0	0	0	0	0	2	30	60
4	3	0	0	0	0	1	30	60
4	0	0	0	0	0	1	30	60
0	2	0	0	1	1	2	10	60
3	1	0	0	0	0	0	25	55
0	0	0	0	0	0	0	30	15
0	3	1	0	0	5	2	38	10
0	0	0	0	0	0	0	15	60
0	1	0	0	0	0	2	60	180
0	0	0	0	0	0	0	60	90
0	0	0	0	0	0	0	30	40
3	1	0	0	0	1	1	15	40
0	1	1	0	0	2	3	45	60
4	2	0	0	1	1	4	5	60
3	5	1	0	0	2	3	0.2	30
0	0	0	0	0	0	0	25	60
0	2	0	0	0	1	4	30	120
0	1	0	0	0	0	2	15	30
0	1	0	0	0	0	1	25	40
0	0	0	0	0	0	0	60	120
6	1	0	0	0	0	2	60	240
4	3	0	0	0	1	2	5	25
2	1	0	0	0	1	2	40	180
0	2	0	0	0	1	2	50	90
0	0	0	0	0	0	0	30	40
0	3	0	0	0	1	4	20	30
0	0	0	0	0	0	0	25	40
0	1	0	0	0	0	2	30	60
0	0	0	0	0	2	2	50	120
0	0	0	0	0	0	0	30	10
5	3	0	0	0	2	2	30	20
2	2	0	0	0	0	4	30	60
0	0	0	0	0	0	0	40	60
0	0	0	0	0	0	0	45	60
0	0	0	0	0	0	0	15	30
0	2	0	0	0	1	2	30	60
0	0	0	0	0	0	0	30	45
0	0	0	0	0	0	0	15	25
5	1	0	0	0	0	3	30	60
0	0	0	0	0	0	2	30	15
0	1	0	0	0	1	1	30	120
0	1	0	0	0	1	2	45	80
0	1	0	0	0	0	2	5	60
3	1	1	0	0	1	2	15	45

1	2	0	0	0	1	2	30	180
2	4	0	0	0	0	2	30	15
0	0	0	0	0	0	0	40	180
0	0	0	0	0	2	0	30	60
0	0	0	0	0	1	2	15	15
0	0	0	0	0	0	0	60	180
5	3	0	0	0	1	5	20	60
0	0	0	0	0	0	0	30	25
2	1	0	0	0	1	1	15	40
0	0	0	0	0	0	0	30	60
5	4	0	0	0	3	2	15	120
14	1	0	0	0	6	4	30	60
4	1	0	0	0	0	2	40	90
6	3	0	0	0	1	2	20	45
0	2	0	0	0	1	2	25	45
0	1	0	0	0	0	2	5	60
0	0	0	0	1	0	2	15	60
0	3	0	0	0	1	2	30	60
0	2	0	0	0	1	2	40	90
0	1	0	0	0	0	2	60	180
0	2	0	0	0	1	2	20	120
0	2	0	0	0	1	2	10	60
2	2	0	0	0	0	2	60	240
0	1	0	0	0	1	2	60	180
0	0	0	0	0	0	0	60	240
0	0	0	0	0	0	0	30	10
0	0	0	0	0	0	0	20	10
20	8	0	0	0	9	8	45	60
0	1	0	0	0	0	2	20	120
2	2	0	0	0	2	3	30	75
5	2	0	0	0	2	0	40	110
0	1	0	0	0	0	3	10	120
0	0	0	0	0	0	0	30	60
0	1	0	0	0	0	1	20	60
3	2	0	0	0	2	3	20	90
0	0	0	0	0	1	0	10	60
7	2	0	0	0	0	3	30	120
0	0	0	0	0	0	0	10	30
0	1	0	0	0	1	2	60	120
0	1	0	0	0	0	1	15	60
1	1	0	0	0	1	1	15	60
0	0	0	0	0	0	0	40	60
0	0	1	0	0	0	3	15	30
0	1	0	0	0	1	1	15	60
0	5	0	0	0	2	2	10	60
0	0	0	0	0	0	0	60	120
0	0	0	0	0	0	0	20	10
0	1	0	0	0	0	2	40	120
0	0	0	0	0	0	0	30	15
0	0	0	0	0	0	0	40	90
0	0	0	0	0	0	0	20	60
0	1	0	0	0	0	0	60	180

For peer review only

10	2	0	0	0	2	2	35	150
0	4	0	0	0	1	1	30	10
6	2	0	0	0	0	2	10	90
0	0	0	0	0	0	0	20	180
0	1	0	0	0	1	2	45	90
6	0	1	0	0	0	0	35	60
0	1	0	0	0	2	2	60	120
0	1	0	0	0	0	0	25	60
0	0	0	0	0	2	0	30	30
5	1	0	0	0	2	2	5	90
0	2	0	0	0	1	4	30	45
0	2	0	0	0	2	2	10	60
0	0	0	0	0	1	0	30	20
0	1	0	0	0	1	2	30	90
0	1	0	0	0	0	2	15	120
2	1	0	0	0	2	3	30	90
0	2	0	0	0	2	2	10	60
2	1	0	0	0	2	4	40	180
0	1	0	0	0	1	1	15	150
0	0	0	0	0	0	2	10	30
0	0	1	0	0	0	0	30	15
0	1	0	0	0	0	0	10	60
0	0	0	0	0	0	0	10	10
0	0	0	0	0	0	2	15	45
0	2	0	0	0	2	2	30	60
0	3	0	0	0	0	2	20	60
0	1	0	0	0	0	1	30	240
0	0	0	0	0	0	0	30	20
0	0	0	0	0	0	0	30	30
0	0	0	0	0	0	0	20	60
0	2	0	0	0	0	2	25	10
0	2	0	0	0	2	4	15	45
4	2	0	0	0	0	2	30	240
0	0	0	0	0	0	0	15	30
2	1	0	0	0	0	2	60	24
3	1	0	0	0	0	4	30	90
0	1	0	0	0	1	2	10	75
0	1	0	0	0	1	2	120	180
0	1	0	0	0	1	2	120	180
4	1	0	0	0	2	2	60	60
3	1	0	0	0	2	1	15	120
0	0	1	0	0	2	1	15	120
0	0	0	1	0	1	2	120	180
0	2	0	0	0	1	2	50	160
0	0	0	0	0	0	2	50	160
8	0	0	0	0	0	1	70	180
0	1	0	0	0	0	3	20	80
5	1	0	0	0	0	4	60	60
0	0	0	0	0	0	0	120	180
10	1	0	0	0	1	3	50	160
0	0	0	0	0	2	0	90	90
0	0	0	0	0	1	3	60	60

For peer review only

0	0	1	0	0	0	2	90	90
4	0	0	0	0	2	2	15	120
6	2	0	0	0	1	1	90	90
0	0	0	0	0	1	1	120	180
3	1	0	0	0	2	1	55	175
6	1	0	0	0	2	2	25	120
4	1	0	0	0	1	2	120	180
4	1	0	0	0	0	2	60	120
5	1	0	0	0	1	5	60	60
0	1	0	0	0	0	2	120	180
2	0	0	0	0	0	0	10	120
4	1	0	0	0	0	2	60	120
4	1	0	0	0	1	1	60	170
4	1	0	0	0	0	5	60	60
10	1	0	0	0	0	2	120	180
0	0	0	0	0	2	0	45	170
2	1	0	0	0	1	1	120	180
0	1	0	0	0	1	1	120	180
3	0	0	0	0	1	2	60	180
0	1	0	0	0	1	3	60	60
6	0	0	0	0	2	1	25	120
0	2	0	0	0	0	4	60	60
0	0	0	0	0	0	0	120	180
4	1	0	0	0	1	2	60	180
5	1	0	0	0	1	1	20	120
4	0	0	0	0	0	2	60	60
1	0	0	0	0	0	1	60	60
0	0	0	0	0	2	2	50	160
10	0	0	0	0	0	1	90	90
0	0	0	0	0	0	3	90	90
0	1	0	0	0	1	1	120	180
0	1	0	0	0	1	1	45	160
5	0	0	0	0	2	1	120	180
3	0	0	0	0	1	2	90	90
0	1	0	0	0	0	4	90	90
9	0	3	0	0	1	2	60	120
0	1	0	0	0	1	1	120	180
0	1	0	0	0	1	2	50	170
3	0	0	0	0	0	2	90	90
3	0	0	0	0	1	2	90	90
0	1	0	0	0	1	2	60	120
4	1	0	0	0	1	2	60	120
3	1	0	0	0	2	2	20	120
3	0	0	0	0	1	1	60	180
5	0	0	0	0	1	2	90	90
0	0	0	0	0	1	2	50	160
4	1	0	0	0	1	2	60	120
0	0	0	0	0	0	0	30	40
0	0	0	0	0	2	4	40	50
0	0	0	0	0	1	2	30	20
0	0	0	0	0	0	2	25	25
0	0	0	0	0	0	3	20	40

For peer review only

0	0	0	0	0	0	1	30	20
0	0	0	0	0	0	0	10	10
0	0	0	0	0	0	0	30	60
0	0	0	0	0	0	2	20	60
0	0	0	0	0	0	1	10	60
0	0	0	0	0	0	0	60	60
0	0	0	0	0	0	6	60	60
3	0	0	0	0	1	3	15	15
4	0	0	0	0	0	0	60	40
0	0	0	0	0	0	2	60	40
5	0	0	0	0	0	0	30	60
0	0	0	0	0	0	0	20	60
0	0	0	0	0	0	3	20	20
0	0	0	0	0	0	0	10	30
0	0	0	0	0	0	0	15	30
0	1	0	1	0	2	6	60	90
0	0	0	0	0	0	0	10	30
0	0	0	1	0	2	2	30	20
15	0	0	0	0	0	0	30	60
0	0	0	0	0	1	3	30	60
0	0	0	0	0	0	0	8	8
0	0	0	0	0	2	1	10	30
2	0	0	0	0	0	0	30	60
0	0	0	0	0	0	1	5	25
0	0	0	1	0	2	4	30	60
0	0	0	0	0	2	3	30	30
0	0	0	0	0	0	0	60	40
0	0	0	0	0	1	0	60	60
6	1	0	1	0	2	3	30	60
0	0	0	0	0	0	0	30	60
0	0	0	0	0	0	2	30	60
0	0	0	0	0	0	1	20	30
0	0	0	0	0	0	0	30	60
2	0	0	0	0	2	2	60	30
0	0	0	0	0	0	0	30	60
0	0	0	0	0	0	0	60	60
0	0	0	0	0	0	0	30	30
2	0	0	0	0	3	4	10	15
0	0	0	0	0	2	5	30	60
0	0	0	0	0	0	0	40	40
0	0	0	0	0	0	1	7	7
0	0	0	0	0	2	2	30	60
0	0	0	0	0	0	1	30	30
0	0	0	0	0	0	0	10	60
0	0	0	0	0	0	0	20	30
0	0	0	0	0	0	0	45	60
0	0	0	0	0	0	0	15	30
0	0	0	0	0	0	0	30	60
0	0	0	1	0	0	2	40	20
0	0	0	0	0	0	0	40	40
0	0	0	0	0	1	1	15	15
0	0	0	0	0	0	2	10	60

For peer review only

0	0	0	0	0	2	0	10	30
0	0	0	3	0	0	0	40	20
0	0	0	0	0	0	0	30	30
0	0	0	1	0	2	4	60	30
0	0	0	0	0	0	0	10	30
0	0	0	0	0	0	2	10	10
4	0	0	0	0	0	0	30	20
0	0	0	0	0	0	2	7	90
0	0	0	0	0	0	1	15	60
0	0	0	0	0	3	6	15	15
0	0	0	0	0	1	1	30	60
0	0	0	0	0	0	1	30	30
0	0	0	0	0	0	0	15	30
0	0	0	0	0	0	2	30	45
0	0	0	1	0	0	3	30	30
0	0	0	0	0	1	0	10	20
0	0	0	0	0	0	1	10	10
0	0	0	0	0	0	0	30	30
1	0	0	0	0	0	0	30	12
5	0	0	0	0	0	3	20	1
0	0	0	1	0	3	2	10	30
0	0	0	0	0	0	1	30	60
0	0	0	0	0	0	0	30	60
0	0	0	0	0	0	2	30	60
0	0	0	0	0	1	2	50	30
0	0	0	0	0	0	0	40	40
0	0	0	0	0	0	2	10	30
0	0	0	0	0	0	0	10	30
0	0	0	0	0	0	0	10	60
0	0	0	0	0	0	0	8	15
0	0	0	0	0	0	0	40	60
0	0	0	0	0	0	0	30	60
0	0	0	0	0	2	2	30	60
0	2	0	0	0	0	3	60	60
3	1	0	0	0	1	2	30	60
0	0	0	0	0	0	2	30	30
0	0	0	1	0	0	2	30	30
0	0	0	0	0	0	2	10	10
1	0	0	0	0	0	2	10	30
0	0	0	0	0	0	0	30	60
0	0	0	0	0	0	0	60	60
0	0	0	1	0	0	2	30	20
5	0	0	0	0	1	1	40	20
0	0	0	0	0	0	0	15	15
0	0	0	0	0	2	0	40	20
0	2	0	0	0	0	5	60	60
0	0	0	0	0	0	0	45	60
4	0	0	1	0	1	0	50	20
0	0	0	0	0	0	1	20	60
0	0	0	0	0	0	0	30	60
10	0	0	0	0	0	0	50	30
0	0	0	0	0	0	0	15	60

For peer review only

0	0	0	1	0	3	2	30	60
0	0	0	0	0	0	0	20	60
0	0	0	0	0	0	0	15	60
0	0	0	1	0	3	2	10	30
0	0	0	1	0	3	2	10	30
4	0	0	0	0	2	2	30	60
5	0	0	0	0	0	0	15	30
0	0	0	0	0	0	3	60	60
0	0	0	0	0	0	1	30	60
0	0	0	0	0	2	5	15	25
0	2	0	0	0	2	1	30	60
3	0	0	0	0	0	0	10	40
0	0	0	0	0	0	0	40	60
0	0	0	0	0	0	0	30	60
0	0	0	0	0	0	0	50	50
2	0	0	1	0	0	2	30	20
0	0	0	0	0	0	0	30	60
2	0	0	0	0	1	0	30	60
0	0	0	0	0	0	3	50	60
0	0	0	0	0	4	4	40	30
0	0	0	0	0	0	0	50	40
4	0	0	0	0	3	0	30	60
0	0	0	0	0	0	0	50	50
5	0	0	0	0	0	0	20	20
0	0	0	0	0	2	2	20	15
0	0	0	1	0	2	2	20	20
0	0	0	0	0	0	0	40	40
2	0	0	0	0	1	3	10	20
0	0	0	0	0	1	5	20	20
0	0	0	0	0	0	0	30	60
2	0	0	1	0	1	2	40	25
0	0	0	0	0	0	0	60	60
0	0	0	0	0	0	0	30	120
0	2	0	0	0	0	2	60	60
0	0	0	0	0	0	0	40	40
0	1	0	0	1	1	2	20	60
0	0	0	0	0	0	0	30	30
0	1	0	0	0	1	2	10	120
0	1	0	0	0	1	2	20	60
0	2	0	0	0	1	2	10	10
2	0	0	0	0	0	2	30	60
0	2	0	0	0	0	0	35	150
0	0	0	0	0	2	4	5	120
1	1	0	0	0	0	1	10	120
2	1	0	0	0	0	2	45	120
0	0	0	0	0	1	2	15	15
5	3	0	0	0	1	2	10	10
0	0	0	0	0	1	0	30	120
0	0	0	0	0	0	0	15	60
0	1	0	0	0	0	0	60	60
0	2	0	0	0	2	4	60	120
0	2	0	0	1	1	6	60	90

For peer review only

0	0	0	0	0	0	0	15	15
0	2	0	0	0	2	2	15	80
0	0	0	0	0	0	0	10	120
3	0	0	0	0	0	0	15	15
6	1	0	0	0	1	2	15	60
0	0	0	0	0	0	0	25	120
0	0	0	0	0	0	0	30	60
3	3	0	0	0	1	3	5	5
0	0	0	0	0	0	0	5	5
0	1	0	0	0	0	2	35	35
0	1	0	0	0	1	2	60	120
0	0	0	0	0	0	0	10	10
0	0	0	0	0	0	0	30	120
0	2	0	0	0	2	3	10	60
5	2	0	0	0	1	2	30	120
0	0	0	0	0	0	0	20	20
0	2	0	0	0	0	3	30	30
0	0	0	0	0	0	0	30	60
0	0	0	0	0	0	0	15	120
0	0	0	0	0	1	0	30	30
4	1	0	0	0	0	0	60	60
0	1	0	0	0	0	2	40	90
0	1	0	0	0	1	2	30	90
0	0	0	0	0	2	6	5	5
0	3	0	0	0	1	1	30	30
0	3	0	0	0	0	2	25	25
0	0	0	0	0	0	0	30	30
0	0	0	0	0	0	0	10	60
0	0	0	0	0	0	0	30	30
7	4	0	0	0	1	4	10	10
0	1	0	0	0	1	2	30	120
0	0	0	0	0	0	0	20	20
0	0	0	0	0	0	0	30	150
0	0	0	0	0	0	0	20	20
2	1	0	0	0	0	2	30	90
0	2	0	0	0	0	2	60	120
0	0	0	0	0	0	0	60	60
0	1	0	0	1	2	2	10	90
0	0	0	0	0	0	0	10	10
0	0	0	0	0	0	0	30	30
0	0	0	0	0	0	0	30	30
0	5	0	0	0	2	0	30	30
0	0	0	0	0	0	0	10	10
0	2	0	0	0	1	0	60	60
0	0	0	0	0	2	3	5	10
10	1	0	0	0	1	2	45	90
0	0	0	0	0	0	0	20	20
0	0	0	0	0	0	0	30	120
0	1	0	0	0	1	2	40	90
0	1	0	0	0	1	2	20	120
0	1	0	0	0	0	2	10	120
0	0	0	0	0	0	0	10	10

For peer review only

0	2	0	0	0	2	5	30	180
0	1	0	0	0	0	0	20	20
4	2	0	0	0	0	0	5	5
2	0	0	0	0	0	2	20	60
15	1	0	0	0	0	2	10	120
3	3	0	0	0	0	2	25	25
2	1	0	0	0	1	1	30	60
0	0	0	0	0	0	0	25	25
0	1	0	0	0	1	2	10	60
2	1	0	0	0	0	2	15	120
0	0	0	0	0	0	0	20	20
0	0	0	0	0	0	0	90	120
0	3	0	0	0	1	2	45	150
0	3	0	0	0	0	0	5	5
0	0	0	0	0	0	0	5	5
0	0	0	0	0	2	2	40	180
0	2	0	0	0	0	2	30	90
0	0	0	0	0	0	0	30	30
5	2	0	0	0	1	2	30	120
0	0	0	0	0	0	4	5	5
0	0	0	0	0	1	2	20	60
0	1	0	0	0	2	2	90	60
1	1	0	0	0	3	6	30	90
1	1	0	0	0	1	2	60	40
8	1	0	0	0	3	4	120	180
1	0	0	0	0	0	0	10	90
3	1	0	0	0	0	4	5	120
0	1	0	0	0	4	3	30	60
0	0	0	1	0	0	2	60	120
0	0	0	0	0	1	0	10	60
0	1	0	0	0	5	3	30	60
2	0	0	0	0	4	2	10	55
0	0	0	0	0	4	1	15	50
2	1	0	0	0	3	3	5	120
0	0	0	0	0	1	2	10	60
0	1	0	0	0	2	2	30	60
0	0	0	0	0	0	0	40	90
0	0	0	0	0	0	0	20	60
0	0	0	1	0	2	3	30	90
1	0	0	0	0	0	3	15	100
1	1	0	0	0	2	2	30	90
2	0	0	1	0	1	2	15	50
0	0	0	0	0	0	0	40	120
1	2	0	0	0	0	4	30	120
5	0	0	0	0	2	3	20	60
0	1	0	0	0	0	3	30	120
0	1	0	0	0	1	2	40	90
8	1	0	0	0	3	4	60	180
0	1	0	0	0	1	1	60	120
0	0	0	0	0	1	3	10	60
0	0	0	0	0	2	2	10	45
0	0	0	0	0	0	0	20	60

0	0	0	0	0	2	2	60	120
0	0	0	0	0	0	2	20	120
0	0	0	0	0	1	2	60	60
2	1	0	0	0	1	3	10	40
0	0	0	1	0	1	2	15	120
2	0	0	1	0	2	2	50	90
0	0	0	0	0	0	3	30	180
0	0	0	1	0	6	4	60	180
0	3	0	0	0	3	2	40	180
0	0	0	0	0	1	0	30	120
1	1	0	0	0	3	3	20	1
0	0	0	0	0	0	0	30	120
4	0	0	1	0	4	3	60	2
0	0	0	0	0	1	4	10	120
0	1	0	0	0	0	2	40	90
0	0	0	0	0	0	2	10	60
0	1	0	0	0	0	2	60	90
0	1	0	0	0	2	3	40	120
0	0	0	0	0	0	1	20	120
0	1	0	0	0	2	3	50	100
0	0	0	0	0	0	4	50	120
10	0	0	0	0	5	2	60	180
0	0	0	1	0	1	4	15	50
0	1	0	1	0	3	3	20	120
0	0	0	1	0	2	5	60	130
0	0	0	0	0	2	2	15	60
0	0	0	1	0	3	3	5	120
0	1	0	0	0	2	3	30	60
0	1	0	0	0	3	3	60	240
0	1	0	1	0	2	2	10	60
0	0	0	1	0	2	4	35	90
0	0	0	0	0	0	0	30	120
3	1	0	1	0	3	2	20	50
0	0	0	0	0	2	3	60	180
0	0	0	1	0	2	4	20	120
0	2	0	0	0	2	2	30	150
2	1	0	0	0	3	4	60	120
0	0	0	0	0	3	7	15	120
0	1	0	0	0	3	3	60	120
0	1	0	0	0	1	2	60	60
0	0	0	0	0	2	2	30	90
1	0	0	1	0	1	2	20	120
0	0	0	0	0	0	0	15	45
0	1	0	0	0	0	2	60	90
0	0	0	0	0	0	3	60	120
0	0	0	1	0	0	2	20	120
0	0	0	0	0	0	0	20	120
0	0	0	0	0	3	2	10	45
0	0	0	0	0	2	5	120	60
0	0	0	0	0	0	0	30	120
0	0	0	1	0	1	3	25	60
5	0	0	0	0	0	3	30	120

For peer review only

0	0	0	1	0	4	2	30	60
0	2	0	0	0	0	4	60	180
0	0	0	0	0	1	1	20	60
0	0	0	0	0	0	0	20	120
3	3	0	0	0	0	5	60	150
0	0	0	0	0	0	0	40	120
0	3	0	0	0	2	2	15	55
0	0	0	0	0	0	2	15	180
0	1	0	0	0	2	3	60	180
0	1	0	0	0	1	2	10	60
0	1	0	0	0	3	3	30	60
0	0	0	0	0	0	6	20	180
3	3	0	0	0	3	5	60	120
0	0	0	0	0	2	2	15	60
0	1	0	0	0	2	2	20	60
1	2	0	1	0	3	4	40	60
2	0	0	0	0	2	3	25	60
0	0	0	0	0	0	0	40	120
0	1	0	0	0	1	4	30	90
0	1	0	0	0	0	3	60	90
4	4	0	0	0	0	0	5	120
0	0	0	0	0	0	0	10	60
0	0	0	0	0	0	1	20	90
0	1	0	0	0	0	0	30	230
0	0	0	0	0	0	0	30	120
0	1	0	0	0	1	2	30	90
2	2	0	0	0	0	0	25	90
0	2	0	0	0	0	2	10	120
0	0	0	0	0	0	0	30	90
0	0	0	0	0	0	0	15	120
0	0	0	0	0	0	0	30	150
0	0	0	0	0	0	0	30	120
0	0	1	0	0	0	0	30	120
0	0	0	0	0	0	1	30	120
0	1	0	0	0	0	0	40	120
0	0	0	0	0	0	0	30	360
0	0	0	0	0	0	0	5	90
0	2	0	0	0	0	0	25	125
0	0	0	0	0	0	0	10	180
5	3	0	0	0	2	4	10	90
0	0	0	0	0	0	0	30	120
0	0	0	0	0	0	0	30	120
0	2	0	0	0	0	2	20	90
0	0	0	0	0	0	0	25	120
0	0	0	0	0	0	0	60	180
0	0	0	0	0	0	0	30	60
4	1	0	0	0	1	2	30	120
0	0	0	0	0	0	0	30	90
0	0	0	0	0	0	0	45	360
0	0	0	0	0	1	1	10	90
0	0	0	0	0	1	2	30	60
0	0	0	0	0	0	0	45	180

For peer review only

0	0	0	0	0	0	0	30	120
0	0	0	0	0	0	0	5	150
0	0	0	0	0	0	0	30	180
0	1	0	0	0	1	1	15	150
5	1	0	0	0	2	3	30	60
0	1	0	0	0	0	2	60	180
2	1	0	0	0	0	2	90	150
1	1	0	1	0	1	2	60	60
0	0	0	0	0	0	2	5	30
0	2	0	0	0	1	2	60	180
6	1	0	0	0	0	2	5	80
0	0	0	0	0	0	2	10	90
0	0	0	0	0	0	0	50	120
0	3	0	0	0	2	2	30	90
0	2	0	0	0	1	2	20	45
4	0	0	0	0	2	2	5	30
4	1	0	1	0	3	4	45	120
0	0	0	0	0	2	2	5	90
0	0	0	0	0	0	0	5	40
7	2	0	0	0	1	2	30	90
0	2	0	0	0	1	2	35	90
0	0	0	1	0	3	3	20	40
0	0	0	0	0	0	2	20	30
0	2	0	0	0	2	2	5	30
0	3	0	0	0	1	3	30	120
8	1	0	0	0	1	1	60	120
0	0	0	0	0	0	1	50	120
0	0	0	1	0	2	3	40	60
5	3	0	0	0	2	4	80	180
2	1	0	0	0	3	4	30	80
0	1	0	0	0	4	3	60	80
0	1	0	0	0	1	2	60	120
0	0	0	0	0	0	0	50	240
4	0	0	1	0	3	5	25	60
0	0	0	0	0	2	1	25	120
3	1	0	1	0	2	2	40	70
6	1	0	0	1	1	2	60	180
0	1	0	0	1	0	2	60	180
4	1	0	0	0	1	3	5	60
0	0	0	0	0	0	4	40	60
0	0	0	0	0	0	2	10	30
6	2	0	0	0	2	3	30	80
0	0	0	0	0	3	3	5	90
0	1	0	0	0	0	2	30	60
0	0	0	0	0	0	0	5	60
8	0	0	0	0	0	2	15	40
3	0	0	0	0	2	2	60	40
0	0	0	0	0	0	0	60	45
0	1	0	0	0	2	4	30	45
0	0	0	1	0	2	3	30	60
0	0	0	0	0	1	1	45	120
3	0	0	0	0	0	2	2	30

For peer review only

0	0	0	0	0	1	2	5	30
0	1	0	0	0	0	2	30	90
0	0	0	0	0	0	0	10	90
0	0	0	0	0	3	2	45	60
3	0	0	0	0	0	2	50	120
0	2	0	0	0	2	2	20	150
0	1	0	0	0	0	2	60	180
0	0	0	1	0	2	3	10	90
1	1	0	0	0	1	1	15	70
0	2	0	0	0	1	2	5	45
0	0	0	0	0	0	0	10	90
0	2	0	0	0	2	2	25	50
0	1	0	0	1	0	2	30	90
0	2	0	0	0	1	2	30	90
0	0	0	1	0	3	3	20	40
0	0	0	0	0	0	0	20	40
0	0	0	0	0	2	2	1	30
3	2	0	0	0	1	2	35	150
0	1	0	0	0	0	0	60	120
0	1	0	0	0	2	3	30	80
2	3	0	0	0	1	3	60	120
0	1	0	0	0	2	3	15	60
2	0	0	1	0	1	3	60	50
2	0	0	1	0	1	2	5	90
0	0	0	0	0	2	4	25	100
0	0	0	0	0	0	0	20	30
0	1	0	0	0	1	2	40	60
4	1	0	0	0	1	2	25	50
3	1	0	0	0	0	2	5	45
0	1	0	0	0	0	3	60	120
1	2	0	0	0	1	2	90	150
0	0	0	0	0	0	4	15	40
7	1	0	0	0	2	4	60	120
1	1	0	0	0	3	8	5	30
0	0	0	0	0	0	3	10	60
0	2	0	0	0	4	2	90	120
0	1	0	1	0	3	2	20	45
5	1	0	0	0	8	4	45	60
0	1	0	0	0	5	6	70	160
0	1	0	0	0	1	2	15	40
0	0	0	0	0	0	3	60	90
0	2	0	0	0	2	4	60	72
0	0	0	1	0	0	3	30	60
0	0	1	0	0	0	3	20	60
7	1	0	0	0	4	3	20	40
0	2	0	0	0	3	8	10	60
8	1	0	1	0	6	4	30	75
0	1	0	0	0	0	2	90	120
3	0	0	0	0	0	1	25	50
0	1	0	0	0	1	3	25	50
0	2	0	0	0	2	2	60	120
0	0	0	0	0	5	6	120	140

For peer review only

0	1	0	0	0	3	5	90	120
0	0	0	0	0	0	3	20	30
0	1	0	0	0	4	5	30	60
7	1	0	0	0	1	2	60	120
2	1	0	0	0	1	2	30	60
0	0	0	0	0	0	0	60	120
0	1	0	0	0	3	4	30	30
7	2	0	0	0	1	3	20	60
0	0	0	1	0	1	3	20	40
0	1	0	0	0	2	3	20	46
0	1	0	0	0	1	2	30	60
5	1	0	0	0	0	2	40	60
2	0	0	0	0	0	2	40	80
0	1	0	0	0	1	2	20	45
0	1	0	0	0	0	2	20	45
0	0	0	0	0	0	0	120	180
0	1	0	1	0	2	2	20	40
0	0	0	0	0	3	6	60	150
0	2	0	0	0	3	2	120	180
0	2	0	0	0	0	0	60	120
3	0	0	0	0	2	4	20	60
0	1	0	0	0	3	4	60	120
0	1	0	1	0	5	4	30	60
2	1	0	0	0	3	4	30	60
0	1	0	0	0	4	2	60	120
10	1	0	0	0	3	4	20	40
0	1	0	0	0	0	2	60	120
0	1	0	0	0	0	2	30	60
0	0	0	0	0	4	4	75	130
0	1	0	0	0	0	2	5	30
1	1	0	0	0	2	6	30	60
2	1	0	1	0	1	3	20	45
0	1	0	0	0	2	1	25	40
2	1	0	0	0	6	8	20	60
0	1	0	0	0	0	2	20	60
0	1	0	0	0	4	3	30	60
0	0	0	0	0	0	3	10	60
0	1	0	1	0	0	2	15	50
0	1	0	0	0	2	3	15	40
0	1	0	0	0	0	0	15	60
5	1	0	0	0	1	2	80	160
0	0	0	0	0	0	0	30	60
0	2	0	0	0	4	5	30	25
0	3	0	0	0	3	6	15	25
0	1	0	0	0	0	0	2	10
0	0	0	0	0	0	0	20	60
0	0	8	0	0	1	1	10	40
0	0	0	0	0	0	0	40	120
0	0	0	0	0	0	0	30	60
0	0	0	0	0	0	0	25	60
0	0	0	0	0	0	0	20	60
0	3	0	0	0	0	0	20	120

For peer review only

5	1	0	0	0	1	2	60	120
0	0	0	0	0	0	0	30	70
0	2	0	0	0	2	4	10	15
0	0	0	0	0	0	0	30	60
3	1	0	0	0	2	1	60	120
0	0	0	0	0	0	0	10	25
4	0	0	0	0	0	1	15	90
0	1	0	0	0	0	0	30	60
0	0	0	0	0	0	0	30	60
0	0	0	0	0	0	0	30	60
0	1	0	0	0	0	2	5	60
0	0	0	0	0	3	2	90	60
0	1	0	0	0	0	2	30	60
0	1	0	0	0	0	3	15	90
4	2	0	0	0	3	4	45	90
0	1	0	0	0	2	4	30	90
0	0	0	0	0	0	0	25	40
0	0	0	0	0	0	0	60	120
0	0	0	0	0	0	0	10	15
0	0	0	0	0	0	0	30	60
0	0	0	0	0	0	2	30	40
0	0	0	0	0	0	0	40	90
0	0	0	0	0	0	0	30	60
0	0	0	0	0	2	0	25	60
0	1	0	0	0	2	2	50	60
0	0	0	0	0	0	0	30	60
5	0	0	0	0	1	2	10	120
0	0	0	0	0	0	0	25	75
0	1	0	0	0	3	4	10	20
0	0	0	0	0	2	0	30	65
0	0	0	0	0	0	0	15	45
0	1	0	0	0	0	1	10	30
0	2	0	0	0	1	2	10	60
0	0	0	0	0	0	0	30	60
0	0	0	0	0	0	2	30	45
0	1	0	0	0	1	2	50	60
0	0	0	0	0	0	0	30	120
0	5	0	0	0	0	3	30	90
5	0	0	0	0	1	1	30	60
0	0	0	0	0	0	0	10	10
0	1	0	0	0	0	1	30	40
0	0	0	0	0	0	0	25	60
0	0	1	0	0	2	2	15	60
0	0	0	0	0	0	0	15	20
0	0	0	0	0	0	0	10	20
0	0	0	0	0	0	0	55	180
0	1	0	0	0	1	2	10	60
0	1	0	0	0	0	0	10	60
0	0	0	0	0	2	3	15	30
3	2	0	0	0	3	4	25	60
0	1	0	0	0	2	4	20	60
0	3	0	0	0	0	2	10	60

For peer review only

0	0	0	0	0	0	0	30	90
1	1	0	0	0	1	0	15	20
0	2	0	0	0	1	2	10	90
2	0	0	0	0	0	0	30	60
0	0	0	0	0	0	0	30	60
0	5	0	0	0	12	12	10	20
2	1	0	0	0	2	3	45	90
0	3	0	0	0	1	2	30	40
0	2	0	0	0	0	2	60	120
5	2	0	0	0	1	2	5	20
2	2	0	0	0	1	3	30	60
0	0	0	0	0	0	0	35	120
0	0	0	0	0	0	0	10	60
4	2	0	0	0	2	3	60	90
0	2	0	0	0	2	5	10	25
0	0	0	0	0	0	0	30	45
0	0	0	0	0	0	0	30	60
0	1	0	0	0	3	2	45	60
0	1	0	0	0	0	1	50	60
2	2	0	0	0	2	3	25	90
0	0	0	0	0	0	0	20	60
1	1	0	0	0	2	3	20	30
0	0	0	0	0	1	1	30	45
0	0	0	0	0	0	0	20	10
0	0	0	0	0	0	0	30	60
0	1	0	0	0	1	2	10	120
0	23	0	0	0	1	2	15	60
0	0	0	0	0	0	0	20	10
0	0	0	0	0	0	0	25	50
0	0	0	0	0	3	5	15	40
4	1	0	0	0	2	2	120	120
0	0	0	0	0	0	0	25	35
0	3	0	0	0	1	4	30	60
0	1	0	0	0	1	4	30	60
8	0	0	0	0	0	0	15	20
0	0	0	0	0	0	0	30	60
2	0	0	0	0	0	0	25	60
0	0	0	0	0	0	0	30	70
0	0	0	0	0	0	0	2	60
2	0	0	0	0	0	2	30	40
0	0	0	0	0	0	0	15	60
0	0	0	0	0	1	2	30	120
0	0	0	0	0	0	3	25	6
5	3	0	0	0	1	2	25	40
5	0	0	0	0	2	1	5	30
4	1	0	0	0	1	1	40	40
6	0	0	0	0	0	0	20	60
10	1	0	0	0	1	2	40	60
0	0	0	0	0	0	0	30	60
40	0	0	0	0	5	25	20	10
9	3	0	0	0	2	2	30	60
0	0	0	0	0	0	0	30	60

For peer review only

0	1	0	0	0	0	0	120	120
4	1	0	0	0	4	3	30	75
0	0	0	0	0	1	0	15	45
0	0	0	0	0	0	0	5	15
0	0	0	0	0	0	0	5	40
0	1	0	0	0	1	3	30	60
0	0	0	0	0	0	0	30	60
0	1	0	0	0	2	4	30	90
0	0	0	0	0	0	0	5	20
0	0	0	0	0	0	0	80	120
0	0	0	0	0	0	0	25	60
0	0	0	0	0	1	0	35	60
0	0	0	0	0	2	2	20	10
0	0	0	0	0	0	0	25	45
0	2	0	0	0	0	2	30	120
0	0	0	0	0	0	0	5	30
0	1	0	0	0	0	0	10	60
0	0	0	0	0	0	0	25	45
0	0	0	0	0	0	0	15	20
6	0	0	0	0	0	0	30	60
0	0	0	0	0	0	0	5	10
0	1	0	0	0	0	1	30	90
0	0	0	0	0	0	0	60	90
9	0	0	0	0	0	0	10	10
0	0	0	0	0	0	2	10	10
0	0	0	0	0	0	0	30	60
0	0	0	0	0	0	0	30	60
3	1	0	0	0	2	2	25	75
0	1	0	0	0	0	1	90	90
2	0	0	0	0	0	0	5	60
0	0	0	0	0	0	0	15	20
0	0	0	0	0	0	0	10	20
0	0	0	0	0	0	0	30	60
2	0	0	0	0	0	1	30	60
0	0	0	0	0	0	0	60	90
0	3	0	0	0	1	2	30	60
4	0	0	0	0	0	1	10	30
3	2	0	0	0	0	2	5	30
0	1	0	0	0	0	1	10	60
0	1	0	0	0	1	2	10	60
0	0	0	0	0	0	0	30	60
0	0	0	0	0	0	0	10	20
0	1	0	0	0	2	4	30	90
0	0	0	0	0	0	0	30	75
0	1	0	0	0	3	5	25	20
2	1	0	0	0	0	2	70	140
3	0	0	0	0	0	2	20	90
0	1	0	0	0	0	1	10	60
0	0	0	0	0	0	0	15	120
2	2	0	0	0	3	4	45	90
0	0	0	0	0	1	3	30	90
0	2	0	0	0	0	2	30	45

For peer review only

0	0	0	0	0	0	0	40	300
0	0	0	0	0	0	2	2	60
0	1	0	0	0	2	4	15	30
0	0	0	0	0	0	0	30	40
0	0	1	0	0	1	1	30	60
0	2	0	0	0	1	2	20	60
0	1	0	0	0	0	2	15	60
0	0	0	0	0	0	0	30	60
4	0	0	0	0	0	0	10	10
0	0	0	0	0	0	2	15	90
0	0	0	0	0	0	0	10	60
0	2	0	0	0	0	2	60	120
0	0	0	0	0	2	4	15	10
0	0	0	0	0	0	0	15	60
3	0	0	0	0	0	0	10	15
0	0	0	0	0	0	0	30	60
2	1	0	0	0	2	2	60	120
0	0	0	0	0	0	0	30	50
0	3	0	0	0	0	4	15	30
6	1	0	0	0	0	2	50	60
0	0	0	0	0	0	0	30	60
0	0	0	0	0	0	0	10	30
4	3	0	0	0	1	2	60	40
4	1	0	0	0	2	2	120	120
12	0	0	0	0	3	0	5	30
0	0	0	0	0	0	0	15	30
0	0	0	0	0	2	2	60	60
0	0	0	0	0	0	0	30	40
2	0	0	0	0	0	0	30	40
0	0	0	0	0	0	0	5	30
0	0	0	0	0	2	2	15	90
0	2	0	0	0	0	1	80	120
0	1	0	0	0	0	0	40	90
0	0	0	0	0	0	0	10	120
0	0	0	0	0	0	0	30	60
0	0	0	0	0	0	0	25	90
2	0	0	0	0	3	0	30	40
0	0	0	0	0	0	0	40	120
0	1	0	0	0	2	2	30	60
0	0	0	0	0	0	0	5	10
2	1	0	0	0	2	2	20	60
0	2	0	0	0	2	2	30	40
0	1	0	0	0	0	0	30	120
4	2	0	0	0	1	2	40	60
0	0	0	0	0	0	0	5	45
0	0	0	0	0	0	0	30	60
0	0	0	0	0	0	0	60	120
0	0	0	0	0	0	0	30	60
8	1	0	0	0	4	3	50	90
0	0	0	0	0	0	0	15	60
0	0	0	0	0	0	0	30	60
0	0	0	0	0	0	3	15	60

0	0	0	0	0	1	4	30	45
0	0	0	0	0	0	0	30	60
0	0	0	0	0	0	0	5	60
0	1	0	0	0	0	2	60	90
0	1	0	0	0	0	1	30	60
0	0	0	0	0	0	0	30	60
0	0	0	0	0	0	0	30	60
0	0	0	0	0	0	0	15	60
5	0	0	0	0	1	1	70	140
0	0	0	0	0	0	0	30	60
0	0	0	0	0	0	0	50	120
0	1	0	0	0	3	3	10	30
0	2	0	0	0	0	1	40	60
5	2	0	0	0	1	2	20	120
2	0	0	0	0	0	0	15	20
0	0	0	0	0	0	0	20	10
0	0	0	0	0	0	0	10	20
2	1	0	0	0	2	2	10	60
0	0	0	0	0	3	3	10	30
0	0	0	0	0	0	0	30	60
0	1	0	0	0	0	2	30	60
0	0	0	0	0	0	0	35	120
0	2	0	0	0	0	1	45	80
0	1	0	0	0	0	1	10	25
0	0	0	0	0	0	0	20	60
0	1	0	0	0	1	2	30	60
0	2	0	0	0	0	1	120	150
0	0	0	0	0	0	0	30	60
5	2	0	0	0	0	2	35	128
2	0	0	0	0	0	2	40	60
0	1	0	0	0	0	1	120	120
0	2	0	0	0	0	0	30	50
0	0	0	0	0	0	2	30	45
0	0	0	0	0	0	0	30	60
3	1	0	0	0	1	2	5	60
0	0	0	0	0	2	3	60	120
0	0	0	0	0	2	2	5	20
2	1	0	0	0	2	4	45	90
4	1	0	0	0	2	2	25	60
0	1	0	0	0	0	1	5	60
0	0	0	0	0	2	4	15	20
0	3	0	0	0	0	2	30	120
2	2	0	0	0	1	4	15	60
0	0	0	0	0	0	0	10	25
3	2	0	0	0	2	4	15	120
0	0	0	0	0	0	2	10	90
0	0	0	0	0	0	0	5	30
3	1	0	0	0	1	3	60	130
2	0	0	0	0	1	2	60	150
2	2	0	0	0	0	1	60	50
5	2	0	0	0	2	2	30	120
2	1	0	0	0	2	3	45	90

For peer review only

0	0	0	0	0	0	0	20	120
0	0	0	0	0	3	3	30	90
10	4	0	0	0	1	3	30	60
0	1	0	0	0	0	2	20	120
0	0	0	0	0	0	0	20	120
0	0	0	0	0	1	1	20	120
4	4	0	0	0	1	2	30	45
0	1	0	0	0	1	2	3	60
0	1	0	0	1	1	2	10	45
0	0	0	0	0	0	0	5	60
1	3	0	0	0	2	4	40	60
0	0	0	0	0	1	4	5	15
0	1	0	0	0	1	2	15	30
0	1	0	0	1	1	2	60	120
0	1	0	0	0	0	1	15	30
0	2	0	0	0	1	2	15	60
3	1	0	0	0	1	2	20	120
3	2	0	8	0	8	1	20	60
0	2	0	0	0	0	4	10	90
0	0	0	0	0	0	0	30	60
0	0	0	0	0	1	2	60	150
0	0	0	0	0	2	0	20	120
4	2	0	0	0	1	6	10	120
2	2	0	0	0	1	2	60	120
3	0	0	0	0	0	2	15	150
3	1	0	0	0	0	2	20	120
0	1	0	0	0	1	2	40	60
8	2	0	0	0	2	4	20	90
2	1	0	0	0	0	2	15	90
8	2	0	0	0	1	2	60	180
0	0	0	0	0	0	0	60	120
0	2	0	0	1	0	2	60	45
0	1	0	0	0	1	0	30	120
0	0	0	0	0	0	0	30	60
0	2	0	0	0	1	2	35	60
5	2	0	0	0	0	2	45	120
5	1	0	0	0	1	1	15	90
0	2	0	0	0	2	4	30	50
6	1	0	0	0	1	2	60	180
0	1	0	0	0	0	2	5	45
0	1	0	0	0	1	2	30	90
0	2	0	0	0	0	2	30	60
0	1	0	0	0	1	2	45	110
0	0	0	0	0	0	0	5	180
0	1	0	0	0	0	2	20	40
1	1	0	0	0	1	2	15	60
3	2	0	0	0	3	4	45	90
3	1	0	0	0	0	0	20	50
0	0	0	0	0	0	0	60	40
0	4	0	0	0	1	2	10	120
9	1	0	0	0	1	2	60	180
0	1	0	0	0	0	2	30	60

For peer review only

2	1	0	0	0	2	3	60	120
0	2	0	0	1	0	2	20	120
2	1	0	0	0	0	1	30	90
0	3	0	0	0	2	2	20	80
2	1	0	0	0	0	2	60	120
0	1	0	0	0	0	0	30	60
0	0	0	0	0	0	0	30	60
2	1	0	0	0	0	2	20	60
0	3	0	0	0	1	2	5	90
0	2	0	0	0	2	2	40	120
0	2	0	0	0	2	2	10	60
0	0	0	0	0	2	2	20	60
0	1	0	0	0	1	2	20	120
0	0	0	0	0	0	0	30	120
1	5	0	0	0	0	4	20	40
0	2	0	0	0	0	2	30	60
0	1	0	0	0	1	2	30	120
2	1	0	0	0	2	3	20	45
3	1	0	0	0	0	2	15	90
3	2	0	0	0	0	0	30	60
0	1	0	0	0	0	2	15	150
0	0	0	0	0	0	0	60	60
0	0	0	0	0	0	0	30	60
5	1	0	0	0	1	2	60	180
0	1	0	0	0	1	2	60	120
4	3	0	0	0	2	6	20	120
0	0	0	0	0	0	0	10	180
0	0	0	0	0	0	0	5	120
0	2	0	0	0	2	4	50	45
4	1	0	0	0	2	2	90	180
5	1	0	0	0	1	4	50	90
2	1	0	0	0	0	2	15	60
1	1	0	0	0	2	2	20	80
0	0	0	0	0	0	2	2	60
0	1	0	0	0	2	3	60	120
3	1	0	0	0	1	2	50	40
8	2	0	0	0	1	1	2	90
0	0	0	0	0	1	0	40	90
2	1	0	0	0	0	2	25	45
0	2	0	0	0	0	3	30	60
0	0	0	0	0	0	1	40	90
0	1	0	0	0	0	2	15	75
0	3	0	0	0	2	0	15	160
2	0	1	0	0	1	2	60	150
1	1	0	0	0	0	2	10	60
0	1	0	0	0	0	2	30	60
4	1	0	0	0	1	2	15	150
0	0	0	0	0	0	2	10	60
0	1	0	0	0	2	5	30	60
0	0	0	0	0	0	0	30	60
0	1	0	0	0	1	2	10	90
0	0	0	0	0	0	0	15	180

For peer review only

0	0	0	0	0	0	0	2	60
0	1	0	0	0	0	2	60	90
5	0	0	0	0	0	1	45	120
0	2	0	0	0	1	2	30	60
1	1	0	0	0	2	2	20	120
1	0	0	1	0	0	2	30	120
0	0	0	0	0	0	2	5	15
0	1	0	0	0	0	4	5	90
0	1	0	0	0	1	4	10	60
3	0	0	0	1	0	2	30	90
0	1	0	0	0	0	2	8	60
0	0	0	0	0	0	0	30	45
0	2	0	0	0	0	2	15	50
0	0	0	0	0	1	2	5	90
4	3	1	0	0	2	7	15	75
2	1	0	0	0	0	2	50	30
2	2	0	0	0	0	3	10	60
0	0	0	0	0	0	0	20	90
0	0	0	0	0	0	0	20	60
4	1	0	0	0	1	2	30	120
2	0	0	0	0	1	3	60	120
7	1	0	0	0	1	2	60	120
0	2	0	0	0	2	2	60	120
0	0	0	0	0	0	0	30	120
0	1	0	0	0	0	1	20	90
0	2	0	0	0	0	0	30	60
3	2	0	0	0	2	4	45	180
0	0	0	0	0	0	0	20	30
10	1	0	0	1	4	6	30	120
6	3	0	0	0	2	4	10	90
0	1	0	0	0	0	0	30	60
2	1	0	0	0	0	0	30	60
3	1	0	0	0	0	0	30	50
0	1	0	0	0	1	3	5	60
0	1	0	0	0	1	2	30	75
0	0	0	0	0	0	0	35	90
0	0	0	0	0	0	0	25	180
3	1	0	0	1	1	2	90	240
0	0	0	0	0	0	1	20	60
3	2	0	0	0	0	4	30	70
0	1	0	0	0	0	2	30	100
4	3	0	0	0	4	6	60	150
0	0	0	0	0	0	0	10	120
0	0	0	0	0	0	4	5	120
0	3	0	0	0	2	4	20	120
6	3	0	0	0	0	3	30	60
0	2	0	0	0	2	3	60	120
0	2	0	0	0	0	2	45	90
0	0	0	0	0	0	2	25	120
0	0	0	0	0	0	0	5	60
10	4	0	0	1	1	4	60	45
2	1	0	0	0	1	2	120	180

For peer review only

1	1	0	0	0	0	2	45	120
0	2	0	0	0	2	4	30	180
0	0	0	0	0	0	2	2	120
4	1	0	0	0	1	2	40	90
2	1	0	0	0	2	3	90	150
3	2	0	0	0	1	0	30	60
0	0	0	0	0	0	0	60	50
4	1	0	0	0	1	3	10	90
0	0	0	0	0	0	0	25	60
4	1	0	0	1	1	2	50	90
0	0	1	0	0	0	0	60	180
3	1	0	1	0	2	2	60	120
4	2	0	0	0	2	4	20	120
2	1	0	0	0	0	2	5	90
0	1	0	0	0	0	2	60	40
3	0	0	0	0	1	2	50	120
0	0	0	0	0	0	0	3	60
4	1	0	1	0	3	3	60	120
6	1	0	0	0	2	3	30	90
0	3	0	0	0	1	2	60	30
0	1	0	0	0	0	2	10	60
0	3	0	0	0	0	2	35	120
0	1	0	0	0	0	2	15	60
0	0	0	0	0	0	0	45	120
0	1	0	0	0	2	3	60	120
0	1	0	0	0	2	2	10	80
0	0	1	0	0	0	1	30	60
0	2	0	0	0	1	2	25	70
0	1	0	0	0	0	1	50	70
0	0	0	0	0	0	0	10	120
0	1	0	0	0	1	2	30	90
2	1	1	0	0	2	2	60	150
4	4	1	0	0	5	4	25	150
0	0	0	0	0	0	0	60	120
3	3	0	0	0	2	3	60	120
3	1	0	0	0	1	2	30	90
0	0	0	0	0	0	0	30	120
2	2	0	0	0	1	2	45	30
2	2	0	0	0	2	2	50	80
0	0	0	0	0	2	1	2	120
0	0	0	0	0	0	0	30	60
4	2	0	0	0	2	2	30	90
0	0	0	0	0	0	0	5	180
0	1	0	0	0	1	4	30	120
4	3	0	0	0	2	2	30	90
4	1	0	0	0	0	3	30	90
0	1	0	0	0	4	1	5	30
10	2	0	0	0	1	4	60	90
0	1	0	0	0	1	2	60	120
3	2	0	0	0	2	4	25	150
5	0	0	0	1	0	2	40	120
3	1	0	0	0	1	2	40	90

For peer review only - http://bmjopen.bmj.com/site/about/guidelines.xhtml

0	0	0	0	0	0	0	30	120
0	2	0	0	0	0	2	45	60
1	2	0	0	0	0	2	5	60
0	0	0	0	0	0	0	35	90
0	0	0	0	0	0	0	150	240
0	1	0	0	0	0	2	5	120
2	0	0	0	0	2	2	120	180
3	2	0	0	0	2	4	45	120
0	1	0	0	1	0	2	45	120
5	1	0	0	0	1	2	60	180
2	1	0	0	0	1	2	30	60
0	1	0	0	0	0	2	60	30
3	2	0	0	0	1	2	15	60
0	0	0	0	0	0	0	20	60
0	0	1	0	0	1	0	30	60
2	0	0	0	0	0	2	25	120
11	2	0	0	0	2	2	10	90
0	1	0	0	0	0	1	30	120
8	1	0	0	0	0	2	60	120
0	1	0	0	0	0	0	5	30
0	0	0	0	0	0	0	30	60
0	4	1	0	0	3	5	60	120
0	1	0	0	0	0	0	30	60
0	1	0	0	0	2	1	60	150
3	0	0	0	0	2	2	120	180
2	0	0	0	0	1	2	60	150
1	0	0	0	0	1	3	40	120
0	2	0	0	0	0	5	20	60
0	0	0	0	0	1	2	60	120
4	1	0	0	0	0	2	60	180
0	2	0	0	0	1	4	20	70
0	1	0	0	0	0	4	30	60
0	1	0	0	0	0	2	60	120
0	1	0	0	0	1	0	25	60
0	0	0	0	1	1	1	10	60
0	2	0	0	0	3	2	30	150
0	0	0	0	0	0	0	30	90
0	1	0	0	0	0	1	15	40
0	2	0	0	0	1	2	60	45
0	2	0	0	0	1	3	90	60
0	0	0	0	0	0	0	25	30
0	0	0	0	0	0	0	15	35
0	0	0	0	0	0	0	30	60
0	0	0	0	0	0	0	10	60
0	0	0	0	0	0	0	30	90
0	2	0	0	0	1	2	10	120
0	2	0	0	0	0	2	30	60
0	0	0	0	0	0	0	60	180
0	0	0	0	0	0	0	5	45
0	2	0	0	0	0	2	60	120
3	1	0	0	0	1	2	60	120
0	0	0	0	0	0	0	15	90

For peer review only

3	1	0	0	0	2	4	45	150
4	4	0	0	0	3	4	30	120
3	1	0	0	0	1	2	35	120
0	2	0	0	0	2	3	35	60
0	2	0	0	0	1	3	15	60
0	1	0	0	0	0	2	25	120
0	1	0	0	1	0	2	5	90
4	1	0	0	0	0	2	15	45
0	0	0	0	0	1	2	20	60
0	1	0	0	0	1	2	30	120
0	2	0	0	0	2	3	30	160
2	1	0	0	0	1	4	40	90
0	0	0	1	0	1	3	30	120
0	3	0	0	0	2	4	25	120
7	2	0	0	0	1	2	20	60
0	0	0	0	0	0	0	30	60
0	2	0	0	0	0	2	30	120
0	0	0	0	0	0	2	10	120
0	1	0	0	0	0	2	5	180
0	2	0	0	0	0	2	15	180
5	1	0	0	1	1	2	30	90
0	0	0	0	0	0	0	60	150
0	0	0	0	0	0	0	40	120
4	1	0	0	0	1	4	40	120
3	1	0	0	0	1	2	30	50
2	1	0	0	0	1	3	90	120
0	1	0	0	0	1	1	15	60
0	1	0	0	0	0	0	30	120
5	2	0	0	0	0	2	20	60
0	0	0	0	0	0	0	10	150
0	1	0	0	0	0	2	60	180
0	1	0	0	0	0	2	10	30
4	2	1	0	0	2	4	2	120
0	0	0	0	0	0	0	15	60
0	0	0	0	0	0	0	25	90
3	0	0	0	1	0	2	8	90
1	0	0	0	0	0	0	2	60
3	1	0	0	0	0	1	5	60
0	1	0	0	0	2	4	20	120
0	1	0	0	0	0	1	30	120
0	0	0	0	0	0	0	30	60
4	3	0	0	0	0	4	5	90
0	2	0	0	0	1	2	30	120
0	1	0	0	0	1	2	5	90
0	1	0	0	0	1	2	10	60
1	1	0	0	0	1	1	15	60
8	1	0	0	0	1	2	9	60
0	0	0	0	0	0	0	50	120
0	0	0	0	0	0	0	5	60
0	0	0	0	1	2	2	30	90
2	1	0	0	0	1	3	3	60
4	2	0	0	0	2	2	25	90

2	3	0	0	0	2	2	10	60
0	2	0	0	0	0	3	120	180
0	2	0	0	0	1	2	120	180
8	1	0	0	0	0	2	10	30
0	2	0	0	0	1	3	90	120
0	2	0	0	0	1	2	30	120
2	1	0	0	0	1	3	45	210
0	2	0	0	0	2	2	30	120
8	2	0	0	0	1	4	15	120
0	0	0	0	0	0	0	25	90
4	2	0	0	0	2	3	15	120
0	1	0	0	0	0	2	60	120
0	3	0	0	0	4	8	30	90
0	1	0	0	0	0	2	60	45
0	4	0	0	0	0	5	30	90
0	1	0	0	0	1	2	40	120
0	1	0	0	0	0	2	120	180
0	0	0	0	0	0	0	15	180
0	2	1	0	0	2	0	30	60
0	3	0	0	0	1	4	60	40
0	0	0	0	0	1	2	15	45
0	0	0	0	0	0	0	20	150
4	1	0	0	0	1	3	20	90
0	1	0	0	0	0	1	25	90
0	2	0	0	1	0	2	60	120
2	1	0	0	0	1	2	120	180
0	1	0	0	0	0	2	45	60
0	1	0	0	0	0	2	35	100
0	0	0	0	0	0	0	10	90
2	1	0	0	0	0	2	60	30
4	0	0	0	0	0	2	5	60
7	1	0	0	0	3	2	60	90
0	0	0	0	0	0	1	20	120
0	0	0	0	0	0	0	30	50
0	0	0	0	0	0	0	45	60
0	2	0	0	0	0	4	15	60
0	2	0	0	0	1	2	30	60
0	1	0	0	0	0	1	45	180
0	1	0	0	0	2	3	60	60
0	0	0	0	0	0	2	10	60
0	0	1	0	0	0	0	30	60
0	0	0	0	0	0	0	15	60
0	1	0	0	0	2	5	60	120
2	1	0	0	0	0	2	15	60
0	0	0	0	1	0	2	5	15
7	1	0	0	0	0	2	60	60
0	3	0	0	0	1	4	5	45
0	1	0	0	0	0	2	30	120
3	4	0	0	0	2	4	90	60
4	1	0	0	0	1	2	60	150
0	0	0	0	0	0	0	90	120
0	1	0	0	0	0	2	30	120

For peer review only

2	3	0	0	0	1	3	60	180
0	0	0	0	0	0	2	60	180
5	0	0	0	0	1	2	60	180
0	0	0	0	0	0	0	30	40
0	0	0	0	1	1	2	30	40
0	0	0	0	0	0	0	30	150
0	0	0	0	0	0	0	15	60
0	1	0	0	1	0	2	10	70
0	2	0	0	0	1	3	60	90
0	2	0	0	0	1	2	20	45
4	1	0	0	0	2	3	90	180
3	1	0	0	0	1	1	90	120
0	1	0	0	0	1	2	120	180
2	2	0	0	1	1	2	70	135
0	4	0	0	0	0	0	25	60
11	1	0	0	0	1	2	60	180
0	0	0	0	0	0	0	20	90
2	1	0	0	0	1	2	60	150
6	1	0	0	0	2	1	30	120
0	0	0	0	0	0	0	5	60
0	1	0	0	0	1	2	20	40
0	0	0	0	0	0	0	15	60
0	0	0	0	0	2	2	30	90
0	1	0	0	0	3	3	15	45
0	0	0	0	0	2	2	45	120
0	0	0	0	0	2	1	90	240
0	0	0	0	0	0	0	10	80
5	0	0	0	0	0	0	15	120
0	0	0	0	0	1	1	10	60
4	2	0	0	0	2	2	120	120
2	1	0	0	0	0	1	60	120
4	0	0	0	0	0	2	20	120
5	0	0	0	0	0	3	60	120
0	2	0	0	0	2	2	100	220
0	1	0	0	0	2	2	10	35
2	0	0	0	0	1	2	25	60
0	0	0	0	0	0	0	15	45
0	1	0	0	0	3	3	15	45
0	1	0	0	0	1	2	120	120
0	0	0	0	0	0	0	15	120
0	1	0	0	0	1	3	15	45
0	0	0	0	0	0	0	5	40
7	1	0	0	0	2	4	10	35
0	0	0	0	0	1	2	5	120
4	1	0	0	0	1	1	90	150
8	2	0	0	0	2	3	60	120
0	0	0	0	0	3	2	40	90
0	1	0	0	0	2	2	120	120
2	0	0	0	0	2	5	120	180
0	0	0	0	0	1	2	10	30
0	0	0	0	0	0	2	10	35
0	1	0	0	0	3	2	135	180

For peer review only

For peer review only - http://bmjopen.bmj.com/site/about/guidelines.xhtml

2	0	0	0	0	3	2	15	120
6	1	0	0	0	1	2	60	120
0	0	0	0	0	1	3	20	20
6	1	0	0	0	2	3	120	240
0	1	0	0	0	2	2	120	120
0	0	0	0	0	4	5	120	240
0	2	0	0	0	1	3	120	120
2	1	0	0	0	2	2	10	40
2	1	0	0	0	2	3	120	120
0	0	0	0	0	1	2	120	120
0	0	0	0	0	2	2	40	90
2	2	0	0	0	1	0	120	120
0	0	0	0	0	2	3	30	90
0	0	0	0	0	5	2	15	90
0	1	0	0	0	2	2	120	240
4	1	0	0	0	1	1	90	180
0	0	0	0	0	1	1	20	120
0	0	0	0	0	2	2	15	45
0	1	0	1	0	5	4	20	20
1	1	0	0	0	4	4	120	120
0	1	0	0	0	2	2	20	20
0	0	0	2	0	2	2	30	120
0	0	0	0	0	0	0	15	90
1	1	0	0	0	3	4	10	35
0	1	0	0	0	0	2	50	60
3	1	0	0	0	5	3	15	120
0	1	0	0	0	1	2	10	40
0	1	0	0	0	1	2	100	180
0	1	0	0	0	2	2	15	45
0	0	0	0	0	2	2	90	180
0	1	0	0	0	2	3	10	120
0	0	0	0	0	0	0	15	45
0	1	0	0	0	1	3	20	20
0	1	0	0	0	0	2	10	35
0	0	0	0	0	1	2	90	120
0	0	0	0	0	0	2	20	20
0	0	0	0	0	0	0	10	120
0	0	0	0	0	0	0	120	120
2	1	0	0	0	2	3	10	120
8	2	0	0	0	2	4	90	180
2	1	0	0	0	2	2	90	180
4	2	0	0	0	2	2	60	120
2	1	0	0	0	5	2	120	240
1	1	0	0	0	1	2	20	60
0	0	0	0	0	0	2	10	40
0	0	0	0	0	0	0	120	120
0	0	0	0	0	0	2	60	120
4	1	0	0	0	2	2	10	35
0	0	0	0	0	0	0	15	60
3	1	0	0	0	2	3	40	90
6	1	0	0	0	2	3	15	90
0	1	0	0	0	1	2	90	180

For peer review only

3	1	0	0	0	2	2	130	240
6	1	0	0	0	1	1	60	120
0	0	0	0	0	0	0	10	60
2	0	0	0	0	5	2	120	240
0	0	0	1	0	2	3	40	90
0	0	0	0	0	1	2	15	120
0	0	0	0	0	0	0	10	120
0	0	0	0	0	0	2	20	20
0	0	0	0	0	1	2	120	180
2	0	0	0	0	0	2	30	60
2	1	0	0	0	0	2	90	180
0	2	0	0	0	5	2	120	120
0	0	0	0	0	0	0	10	60
0	0	0	0	0	3	2	5	120
0	1	0	0	0	1	2	60	120
0	0	0	0	0	0	0	10	120
3	1	0	0	0	2	2	60	120
0	0	0	0	0	2	2	120	240
0	0	0	0	0	1	2	20	50
2	1	0	0	0	1	2	60	120
0	0	0	0	0	0	0	10	90
0	0	0	0	0	0	1	5	40
0	0	0	0	0	0	0	10	40
0	0	0	0	0	0	0	10	120
0	0	0	0	0	0	2	15	45
2	1	0	0	0	2	2	20	120
0	0	0	0	0	2	3	20	60
4	1	0	0	0	1	2	60	120
6	2	0	0	0	5	3	90	180
0	0	0	0	0	2	2	20	90
0	1	0	0	0	3	2	120	240
3	2	0	0	0	3	4	120	120
0	0	0	0	0	2	2	90	180
0	0	0	0	0	0	2	20	20
0	1	0	0	0	1	2	20	20
0	1	0	0	0	4	4	15	45
0	0	0	0	0	2	2	90	180
0	0	0	0	0	2	3	135	260
0	1	0	0	0	2	2	60	120
0	1	0	0	0	2	2	120	120
0	0	0	0	0	0	2	10	35
7	1	0	0	0	2	2	40	60
4	1	0	0	0	1	3	120	120
3	1	0	0	0	2	3	120	120
0	1	0	0	0	1	2	40	40
0	1	0	0	0	1	1	90	120
5	1	0	0	0	2	1	60	120
0	1	0	0	0	3	3	60	120
0	0	0	0	0	1	3	20	120
0	1	0	0	0	1	2	20	20
0	1	0	0	0	0	2	60	120
0	0	0	0	0	0	5	15	45

For peer review only

0	0	0	0	0	0	0	10	90
0	1	0	0	0	3	2	5	40
5	1	0	0	0	2	2	20	120
8	1	0	0	0	4	3	120	180
0	0	0	0	0	0	2	15	45
0	1	0	0	0	2	3	90	180
0	1	0	0	0	2	3	120	120
0	0	0	0	0	2	3	30	90
0	1	0	0	0	2	2	90	180
0	0	0	0	0	0	1	5	40
0	0	0	0	0	2	2	30	60
0	0	0	0	0	0	0	10	120
0	0	0	0	0	2	4	40	60
5	0	0	0	0	1	2	60	180
0	1	0	0	0	0	2	15	50
0	1	0	0	0	1	1	15	45
0	1	0	0	0	1	2	10	30
0	0	0	0	0	2	2	10	90
0	0	0	0	0	0	0	30	120
0	0	0	0	0	0	0	15	60
0	0	0	0	0	2	2	30	90
0	1	0	0	0	1	2	30	60
0	1	0	0	0	0	2	15	45
0	0	0	0	0	1	2	90	120
0	0	0	0	0	4	2	120	240
0	0	0	0	0	1	3	15	45
0	1	0	0	0	2	3	120	120
0	1	0	0	0	3	2	120	240
0	1	0	0	0	2	4	90	180
4	0	0	0	0	0	2	60	120
0	0	0	0	0	0	0	30	120
0	0	0	0	0	0	0	5	60
0	0	0	0	0	0	0	60	120
0	1	0	0	0	1	2	120	120
4	0	0	0	0	1	1	60	120
0	0	0	0	0	0	0	10	35
2	1	0	0	0	0	2	60	120
0	1	0	0	0	2	2	90	240
0	0	0	0	0	2	2	120	240
0	1	0	0	0	2	2	120	120
0	1	0	0	0	0	0	30	120
0	0	0	0	0	0	0	18	60
0	0	0	0	0	1	1	120	90
0	2	0	0	0	1	3	20	60
0	3	0	0	1	0	4	180	45
0	1	0	0	0	0	1	20	60
0	0	0	0	0	0	0	120	5
2	1	0	1	0	1	2	30	90
0	0	0	0	0	0	0	60	120
7	2	0	0	0	1	2	15	60
0	1	0	0	0	0	2	120	60
0	1	0	0	0	0	2	90	150

For peer review only

0	0	0	1	0	1	2	15	60
7	1	0	0	0	2	4	120	60
10	3	0	0	0	3	3	30	20
0	1	0	0	0	1	2	20	60
0	1	0	0	0	3	3	30	240
0	0	0	0	0	2	4	20	90
0	0	0	0	1	2	3	45	150
0	0	0	0	1	1	2	30	90
0	1	0	0	0	0	2	20	90
0	0	0	0	0	0	0	15	60
0	0	0	0	0	0	0	10	5
0	0	0	0	0	0	0	4	20
0	1	0	0	0	1	2	60	120
5	1	0	0	0	0	2	5	60
0	0	0	0	0	1	2	20	120
10	0	0	1	0	1	26	20	120
0	0	0	0	0	0	1	5	60
0	0	0	0	0	0	0	60	120
2	1	0	1	0	2	2	20	180
0	0	0	0	0	0	0	25	90
0	0	0	0	0	0	0	12	10
0	0	0	0	0	0	2	40	30
11	1	0	0	0	3	2	15	90
2	1	0	0	0	0	0	13	120
0	1	0	0	0	1	0	30	10
0	1	0	0	0	2	5	20	120
6	2	0	0	0	0	4	45	70
8	2	0	0	0	0	0	25	60
0	1	0	0	0	0	6	10	60
5	1	0	0	0	2	3	15	90
0	0	0	0	0	0	0	45	90
6	0	0	0	0	0	1	5	45
0	0	0	0	0	0	0	10	20
0	0	0	0	0	0	1	20	90
2	1	0	1	0	1	2	15	60
3	2	0	0	0	2	2	15	120
5	1	0	0	0	4	4	20	60
0	1	0	0	0	2	4	30	180
0	2	0	0	0	1	2	20	15
0	1	0	0	0	0	0	5	60
0	0	0	0	0	0	0	45	90
5	0	0	0	1	1	4	120	60
0	1	0	0	0	0	1	15	30
0	0	0	0	0	4	3	5	120
0	0	0	0	0	0	0	30	120
3	3	0	0	1	2	3	30	60
3	2	0	0	0	1	2	10	20
1	1	0	0	0	1	2	50	90
0	0	0	0	0	0	0	10	60
0	0	0	0	0	0	1	45	110
0	2	0	0	0	1	0	10	5
3	0	0	1	0	1	2	40	90

For peer review only

0	2	0	0	0	0	2	50	120
0	0	0	0	0	0	0	15	20
0	1	0	0	0	2	3	38	180
0	0	0	0	0	0	0	45	120
0	0	0	0	0	0	0	30	5
0	2	0	0	0	0	2	60	75
0	2	0	0	0	1	2	20	60
0	0	0	0	0	0	2	10	90
4	2	0	0	0	1	2	20	5
4	1	0	1	0	1	2	20	60
0	1	0	0	0	1	2	120	90
3	1	0	0	0	0	2	35	40
3	1	0	0	0	0	2	20	120
4	0	0	1	0	1	2	20	60
2	2	0	0	0	1	4	5	60
0	1	0	0	0	0	2	120	90
0	0	0	0	0	0	0	5	60
5	1	0	0	0	0	2	15	90
0	1	0	0	0	2	2	30	90
10	0	0	0	0	0	2	60	60
0	1	0	0	0	2	2	20	90
6	3	0	0	0	2	5	30	25
0	2	0	0	0	2	2	25	90
0	0	0	0	0	0	2	30	120
0	1	0	0	0	1	4	20	120
0	2	0	0	0	1	2	40	30
4	1	0	0	0	0	2	120	60
4	2	0	0	0	0	3	30	60
0	1	0	0	0	2	1	20	60
4	1	0	0	0	2	3	60	120
0	0	0	0	0	0	0	60	30
3	1	0	0	0	0	1	40	150
0	1	0	0	0	2	2	30	180
0	1	0	0	0	0	0	2	30
14	2	0	0	1	2	6	50	65
2	1	0	0	0	1	2	45	120
1	1	0	0	1	4	4	3	90
0	0	0	0	0	0	0	10	90
0	1	0	0	0	1	2	45	90
0	1	0	0	0	0	1	5	90
0	1	0	0	0	0	0	45	45
0	3	0	0	0	0	3	30	60
0	0	0	0	0	0	1	20	120
4	1	0	0	0	2	1	15	90
0	1	0	0	0	2	2	25	60
5	2	0	0	0	2	2	20	15
0	0	0	0	0	0	2	45	90
0	1	0	0	0	1	2	20	60
0	0	0	0	0	1	2	60	120
0	0	0	0	0	0	0	15	120
0	2	0	0	0	3	2	60	120
0	0	0	0	0	0	0	5	60

For peer review only

0	0	0	0	0	0	0	60	120
0	0	0	0	0	0	0	15	5
0	0	0	0	0	0	0	45	90
0	1	0	0	0	0	2	20	90
0	0	0	0	0	0	2	30	120
0	0	0	0	0	0	2	30	150
0	0	0	0	0	0	0	35	40
1	1	0	0	0	1	2	40	30
1	2	0	0	0	1	2	10	90
3	0	0	0	0	0	0	20	130
0	0	0	0	0	0	0	60	5
0	0	0	0	0	0	0	30	30
0	1	0	0	0	1	2	20	60
0	2	0	0	0	1	2	10	60
0	1	0	0	0	0	1	35	50
0	0	0	0	0	0	2	20	5
0	0	0	0	0	0	0	30	20
7	2	0	0	0	2	2	20	45
10	1	0	0	0	0	2	30	180
0	0	0	0	0	0	0	2	90
0	0	0	0	0	0	0	20	30
1	1	0	0	0	1	2	30	90
0	0	0	0	0	0	2	20	90
0	2	0	0	0	1	2	45	90
35	10	0	0	0	5	8	30	150
0	1	0	0	0	0	2	20	90
0	1	0	0	0	2	6	60	60
0	0	0	0	0	0	0	3	2
0	0	0	0	0	1	0	30	5
0	0	0	0	0	0	0	25	10
6	2	0	0	1	1	4	180	30
0	0	0	0	0	0	0	15	5
0	0	0	0	0	0	0	35	40
0	1	0	0	0	0	2	35	120
0	0	0	0	0	0	0	50	150
0	0	0	0	0	0	0	30	120
0	1	0	0	0	2	3	30	120
2	1	0	0	0	1	2	20	75
0	2	0	0	0	1	2	15	90
7	0	0	1	0	2	2	30	90
0	2	0	0	0	1	2	180	60
0	2	0	0	0	1	4	30	60
0	1	0	0	0	1	2	60	90
3	1	0	0	0	3	2	20	120
0	1	0	0	0	1	1	15	90
5	1	0	0	0	1	3	30	60
0	1	0	0	0	0	2	20	120
0	2	0	0	0	2	2	30	60
0	3	0	0	0	1	3	30	120
0	1	0	0	0	0	2	35	90
0	0	0	0	0	0	0	20	90
5	2	0	0	0	0	2	15	5

For peer review only

0	0	0	0	0	0	0	40	45
0	2	0	0	1	0	2	30	120
0	0	0	0	0	0	0	60	150
0	4	0	0	0	0	2	60	90
0	2	0	0	0	0	2	25	75
0	1	0	0	0	2	3	38	120
0	1	0	0	0	0	0	30	120
3	1	0	0	0	4	2	15	60
4	0	0	0	0	0	3	10	60
2	3	0	0	0	2	2	30	25
0	1	0	0	0	0	0	10	60
0	1	0	0	0	0	1	40	90
0	1	0	0	0	0	1	25	60
0	0	0	0	0	0	0	30	45
6	2	0	0	0	1	2	180	30
0	0	0	0	0	0	2	15	90
0	0	0	0	0	1	2	30	150
4	3	0	0	0	8	4	35	90
0	0	0	0	0	0	1	5	60
0	0	0	0	0	0	0	60	60
3	1	0	0	0	5	3	60	60
0	0	0	0	0	0	0	20	20
2	0	0	0	0	0	0	30	30
3	1	0	0	0	2	3	40	40
0	0	0	0	0	0	0	15	15
0	0	0	1	0	2	4	40	40
0	0	0	0	0	0	0	10	10
0	1	0	0	0	0	3	60	60
0	0	0	0	0	0	0	30	30
0	0	0	0	0	0	3	5	5
0	0	0	0	0	0	0	10	20
0	1	0	1	0	1	3	150	150
0	0	0	0	0	0	2	25	25
0	0	0	0	0	2	0	30	30
0	0	0	0	0	0	0	5	5
0	0	0	0	0	0	3	60	60
0	0	0	0	0	0	2	10	10
0	0	0	0	0	0	0	30	30
0	0	0	0	0	0	0	5	5
0	0	0	0	0	0	0	60	60
1	1	0	1	0	1	4	60	60
0	0	0	0	0	2	2	10	10
0	0	0	1	0	1	1	60	60
0	0	0	0	0	1	2	30	30
0	0	0	0	0	2	1	10	10
0	0	0	0	0	0	0	5	5
0	1	0	0	0	1	4	60	60
0	0	0	0	0	2	2	10	105
0	0	0	0	0	0	0	30	30
0	0	0	0	0	0	0	20	20
0	0	0	0	0	0	0	8	8
2	0	0	0	0	2	2	120	120

For peer review only

0	0	0	0	0	0	0	40	40
0	1	0	0	0	0	2	75	75
1	0	0	0	0	0	0	30	30
0	0	0	0	0	0	0	12	12
0	0	0	0	0	0	0	8	8
0	0	0	0	0	0	2	75	75
0	1	0	0	0	0	2	30	30
0	0	0	0	0	0	0	20	20
2	0	0	0	0	0	3	30	30
0	0	0	0	0	0	0	30	30
1	0	0	0	0	0	0	10	10
2	0	0	1	0	2	2	10	10
0	1	0	0	0	0	3	8	8
0	0	0	0	0	1	6	30	30
0	0	0	0	0	0	0	60	60
0	0	0	0	0	1	2	30	30
0	1	0	0	0	0	1	30	30
1	1	0	0	0	1	5	30	30
0	0	0	0	0	0	0	15	15
0	0	0	0	0	2	2	10	10
1	1	0	0	0	0	4	60	60
0	0	0	0	0	0	0	5	5
0	1	0	1	0	4	5	120	120
0	0	0	1	0	2	3	60	60
0	0	0	0	0	0	0	20	20
0	0	0	0	0	0	1	20	20
0	0	0	0	0	0	0	12	12
0	0	0	0	0	0	0	30	30
0	0	0	0	0	1	2	60	60
0	1	0	0	0	2	2	50	50
0	1	0	0	0	0	5	5	5
0	1	0	0	0	2	5	60	60
0	1	0	0	0	1	2	30	30
0	0	0	0	0	0	0	10	10
0	1	0	0	0	2	2	30	30
0	1	0	0	0	0	2	15	15
0	0	0	0	0	0	2	25	25
0	0	0	0	0	0	0	10	10
0	0	0	0	0	0	0	20	20
2	1	0	0	0	3	3	40	40
0	1	0	0	0	1	4	25	25
0	0	0	0	0	0	1	60	60
0	0	0	1	0	0	2	30	30
0	1	0	0	0	1	3	40	40
0	0	0	0	0	0	0	20	20
0	0	0	0	0	0	0	20	20
0	0	0	0	0	0	0	10	10
0	0	0	0	0	0	0	15	15
0	0	0	0	0	0	0	5	5
0	1	0	0	0	0	2	20	20
0	0	0	0	0	0	3	120	120
0	0	0	0	0	0	0	30	30

0	0	0	0	0	0	2	75	75
1	1	0	0	0	0	3	50	50
0	2	0	0	0	1	3	60	60
0	1	0	0	0	0	4	20	20
0	0	0	0	0	0	0	10	10
2	2	0	0	0	2	0	60	180
0	0	0	0	0	3	6	10	10
0	2	0	0	0	1	1	120	120
0	2	0	0	0	2	3	30	30
0	0	0	0	0	0	0	8	8
0	0	0	1	0	0	2	5	5
0	0	0	0	0	0	0	30	30
0	0	0	1	0	2	4	30	30
0	0	0	0	0	0	0	20	20
0	0	0	0	0	0	0	30	30
0	0	0	0	0	0	0	30	30
0	0	0	0	0	0	0	5	5
0	0	0	0	0	0	1	90	90
0	0	0	0	0	2	3	35	35
0	0	0	0	0	0	2	60	60
0	0	0	1	0	0	4	60	60
0	0	0	1	0	1	2	120	120
0	1	0	0	0	1	3	120	120
0	0	0	0	0	0	2	30	30
0	0	0	1	0	1	2	15	15
0	0	0	0	0	1	0	20	20
3	0	0	0	0	0	0	20	20
0	0	0	0	0	0	0	10	10
0	0	0	0	0	0	0	20	20
0	0	0	0	0	2	3	30	30
2	0	0	0	0	0	2	5	5
0	0	0	0	0	0	2	10	10
1	0	0	1	0	2	3	30	30
0	0	0	0	0	0	0	30	30
2	0	0	0	0	0	0	12	12
0	0	0	2	0	2	4	12	12
8	0	0	0	0	3	3	60	60
0	0	0	0	0	0	0	30	30
6	1	0	0	0	2	3	80	80
0	1	0	0	0	0	3	5	5
0	0	0	0	0	0	0	40	10
0	0	0	0	0	0	0	60	60
0	0	0	0	0	0	0	3	3
2	0	0	0	0	0	0	20	20
0	0	0	0	0	0	3	60	60
0	0	0	0	0	0	0	30	30
0	0	0	0	0	2	3	30	30
0	0	0	0	0	1	3	30	30
0	0	0	0	0	1	1	60	60
0	0	0	0	0	0	0	30	30
0	1	0	0	0	2	4	60	60
0	0	0	0	0	0	0	30	30

For peer review only

1	0	0	0	0	1	3	60	60
0	0	0	0	0	0	0	15	15
0	1	0	0	0	2	2	60	60
0	0	0	0	0	1	3	60	60
0	0	0	0	0	0	0	40	40
0	0	0	0	0	1	2	12	12
0	0	0	0	0	0	0	30	30
1	1	0	1	0	1	3	90	90
0	0	0	0	0	0	1	7	7
0	0	0	0	0	1	2	60	60
7	0	0	0	0	2	2	60	60
0	1	0	0	0	2	3	40	40
0	0	0	0	0	0	0	25	25
0	0	0	0	0	0	0	5	5
0	0	0	0	0	0	2	10	10
0	0	0	0	0	0	0	10	10
1	0	0	0	0	2	2	10	10
0	0	0	0	0	1	0	12	12
0	0	0	0	0	0	0	10	10
0	0	0	1	0	0	0	10	10
12	1	0	1	0	1	4	90	90
0	0	0	0	0	1	2	40	40
0	1	0	0	0	0	2	60	60
0	0	0	0	0	0	0	10	10
1	0	0	0	0	2	3	30	30
0	1	0	0	0	2	3	30	30
0	0	0	0	0	0	0	10	10
0	0	0	0	0	1	2	120	120
0	0	0	0	0	0	0	20	20
5	1	0	0	0	2	3	40	40
0	0	0	0	0	0	0	5	5
0	0	0	0	0	0	0	20	20
0	0	0	0	0	0	2	15	15
0	0	0	0	0	2	0	15	15
0	0	0	0	0	0	2	60	60
0	0	0	0	0	0	0	60	60
0	0	0	0	0	0	0	15	15
0	1	0	0	0	3	1	120	120
0	0	0	0	0	0	0	20	20
0	0	0	0	0	0	0	5	5
5	0	0	1	0	1	4	7	7
9	0	0	0	0	2	3	60	60
0	1	0	1	0	1	4	60	60
0	0	0	0	0	0	0	15	15
0	0	0	0	0	0	0	50	50
0	1	0	0	0	1	2	30	30
0	0	0	0	0	0	0	20	20
1	1	0	0	0	2	5	60	60
0	0	0	0	0	0	2	60	60
2	0	0	0	0	1	3	90	90
0	0	0	0	0	0	0	7	7
0	0	0	0	0	0	0	35	35

For peer review only

0	0	0	0	0	2	2	10	10
0	0	0	0	0	0	0	8	8
0	0	0	1	0	1	1	60	60
0	0	0	0	0	0	1	30	30
0	1	0	0	0	2	2	40	40
0	0	0	1	0	0	2	120	120
0	0	0	0	0	0	0	10	10
0	0	0	0	0	0	0	6	6
0	0	0	0	0	1	2	60	60
0	1	0	0	0	0	2	10	10
2	0	0	0	0	2	3	120	120
0	0	0	0	0	0	0	20	20
0	0	0	0	0	0	0	150	150
0	1	0	1	0	2	2	120	120
0	0	0	0	0	1	4	40	40
0	1	0	0	0	1	5	50	50
0	0	0	0	0	0	0	20	20
0	0	0	0	0	0	0	5	5
0	0	0	0	0	0	0	20	20
0	0	0	0	0	0	0	30	30
0	0	0	0	0	0	0	10	10
0	0	0	0	0	0	0	15	15
0	0	0	0	0	0	0	20	20
1	0	0	0	0	0	2	7	7
0	0	0	0	0	2	3	60	60
0	1	0	0	0	0	2	60	60
0	0	0	0	0	0	0	6	6
0	0	0	0	0	0	0	20	20
3	0	0	0	0	0	2	40	40
0	0	0	0	0	0	0	15	15
0	1	0	0	0	1	2	60	60
0	1	0	0	0	2	4	30	30
0	0	0	1	0	1	2	120	120
0	0	0	0	0	0	0	8	8
2	0	0	0	0	2	2	10	10
0	1	0	1	0	0	3	40	40
0	0	0	0	0	0	0	6	6
0	0	0	0	0	0	0	30	30
0	1	0	0	0	1	3	30	30
0	0	0	0	0	0	0	70	70
0	0	0	0	0	0	0	60	60
0	0	0	1	0	1	2	60	60
0	0	0	1	0	1	2	60	60
0	0	0	0	0	0	2	60	60
0	0	0	0	0	0	0	25	25
0	0	0	0	0	0	0	10	10
0	1	0	0	0	5	4	40	40
0	0	0	0	0	0	0	10	10
0	0	0	0	0	0	0	15	15
0	0	0	0	0	0	0	30	30
0	1	0	0	0	1	3	8	8
0	0	0	1	0	0	3	40	40

For peer review only

0	1	0	0	0	2	4	45	45
0	0	0	0	0	0	0	12	12
0	0	0	0	0	0	0	6	6
0	0	0	0	0	0	0	10	10
0	0	0	0	0	0	0	15	15
0	0	0	0	0	0	0	15	15
0	0	0	0	0	0	2	15	15
0	0	0	0	0	0	0	30	30
0	0	0	0	0	0	0	20	20
1	0	0	1	0	0	4	60	60
2	0	0	0	0	2	4	60	60
0	0	0	0	0	0	0	13	13
0	0	0	0	0	0	2	60	60
0	0	0	0	0	0	0	20	20
0	0	0	0	0	0	3	70	70
0	0	0	0	0	2	3	8	8
0	0	0	0	0	0	0	10	10
0	1	0	0	0	0	2	90	90
1	0	0	0	0	0	0	15	15
0	0	0	0	0	0	0	30	30
0	0	0	0	0	0	0	12	12
0	0	0	0	0	0	2	60	60
0	0	0	0	0	0	0	30	30
0	0	0	0	0	2	2	8	8
0	0	0	0	0	0	2	30	30
0	0	0	0	0	0	0	40	40
0	0	0	0	0	1	3	60	60
0	0	0	0	0	0	2	120	120
3	0	0	0	0	0	0	10	10
0	0	0	0	0	0	0	20	20
3	1	0	0	0	5	4	20	20
0	0	0	0	0	0	0	10	10
0	1	0	0	0	0	2	60	60
0	0	0	1	0	0	2	10	10
0	0	0	0	0	1	2	10	10
1	1	0	0	0	1	4	120	120
0	1	0	0	0	3	5	80	80
0	1	0	0	0	4	3	60	60
0	0	0	0	0	0	0	10	10
0	0	0	0	0	0	0	20	20
0	0	0	0	0	2	2	60	60
0	1	0	0	0	0	2	20	20
0	0	0	0	0	3	2	15	15
0	0	0	0	0	0	0	8	8
0	0	0	0	0	0	0	30	30
0	0	0	0	0	1	3	30	30
0	0	0	0	0	0	0	30	30
0	0	0	0	0	0	2	20	20
1	0	0	0	0	0	2	40	40
0	0	0	0	0	0	0	20	20
0	0	0	0	0	0	0	12	12
0	0	0	0	0	1	1	7	7

For peer review only

0	2	0	0	0	2	3	60	60
2	0	0	0	0	0	0	30	30
0	0	0	0	0	1	2	16	16
0	0	0	0	0	0	0	20	20
0	0	0	0	0	0	0	25	25
0	0	0	0	0	0	0	15	15
0	0	0	0	0	0	0	20	20
0	0	0	0	0	0	0	120	120
0	0	0	0	0	0	0	10	10
0	0	0	0	0	0	2	5	5
0	1	0	0	0	0	2	60	60
0	0	0	0	0	0	0	60	60
1	1	0	0	0	0	2	90	90
0	1	0	0	0	1	5	20	20
0	0	0	0	0	0	0	15	15
0	0	0	0	0	1	4	60	60
0	0	0	1	0	2	3	60	30
0	0	0	0	0	0	2	25	60
0	0	0	0	0	1	2	45	30
0	0	0	0	0	0	0	30	15
2	1	0	0	0	3	2	90	60
0	0	0	1	0	3	3	20	40
5	1	0	0	0	5	3	60	60
0	0	0	0	0	0	0	60	40
0	1	0	1	0	2	3	30	50
0	0	0	0	0	3	2	15	30
8	1	0	1	0	5	8	45	90
0	0	0	0	0	0	0	45	30
0	1	0	1	0	1	2	40	50
0	0	0	0	0	4	2	85	75
4	0	0	0	0	1	4	45	45
0	0	0	0	0	1	2	30	60
0	0	0	0	0	0	0	10	10
0	0	0	0	0	0	0	30	90
0	1	0	0	0	1	2	20	40
0	0	0	0	0	0	0	60	30
2	0	0	0	0	0	5	20	30
0	1	0	0	0	1	2	90	40
0	0	0	0	0	0	0	60	5
0	0	0	0	0	0	0	60	150
0	0	0	0	0	0	2	5	60
5	1	0	1	0	6	4	50	75
0	0	0	0	0	2	3	15	60
2	3	0	1	0	3	3	38	45
0	0	0	0	0	3	7	30	45
0	0	0	0	0	0	0	45	15
6	0	0	0	0	1	2	30	60
13	3	0	0	0	4	3	20	60
5	1	0	1	0	5	4	30	60
0	0	0	0	0	0	2	30	45
0	0	0	1	0	0	2	20	60
0	0	0	0	0	0	2	30	80

For peer review only

0	2	0	0	0	3	3	60	50
5	1	0	1	0	3	5	60	45
0	0	0	1	0	0	2	30	40
0	1	0	0	0	2	3	40	40
0	0	0	0	0	0	0	20	30
0	1	0	0	0	0	3	10	30
0	1	0	0	0	2	3	60	45
0	0	0	0	0	0	0	30	60
0	0	0	0	0	0	3	20	30
0	0	0	1	0	0	1	10	30
2	0	0	0	0	2	3	30	40
3	1	0	0	0	3	2	25	30
0	1	0	0	0	1	2	20	60
0	0	0	0	0	0	1	50	60
0	0	0	0	0	0	0	20	30
0	0	0	0	0	1	2	60	10
0	0	0	0	0	0	3	15	20
0	1	0	0	0	4	2	30	45
1	0	0	0	0	0	2	20	60
8	2	0	0	0	3	2	90	40
0	0	0	0	0	0	0	25	45
0	0	0	1	0	3	3	25	40
4	0	0	1	0	2	4	30	60
0	0	0	0	0	0	0	45	25
0	0	0	0	0	0	3	50	30
0	0	0	0	0	1	1	30	20
0	0	0	0	0	0	0	25	60
0	0	0	0	0	0	2	30	45
8	3	0	0	0	1	2	40	60
0	0	0	0	0	2	2	10	30
0	0	0	0	0	0	1	5	60
0	0	0	0	0	0	0	30	20
0	0	0	0	0	2	2	35	50
2	1	0	0	0	0	1	120	90
2	1	0	0	0	3	4	120	90
0	0	0	0	0	1	2	45	45
0	1	0	0	0	1	2	120	140
0	0	0	0	0	0	4	40	60
0	0	0	0	0	2	4	20	30
0	0	0	0	0	1	2	30	45
0	0	0	0	0	0	1	30	15
0	0	0	0	0	1	3	60	90
0	1	0	0	0	5	4	45	30
0	0	0	0	0	0	1	5	30
0	0	0	1	0	2	2	30	45
1	2	0	0	0	2	3	25	60
0	0	0	0	0	0	2	30	60
0	0	0	0	0	2	3	40	60
3	1	0	0	0	3	4	20	60
0	0	0	0	0	0	2	50	90
0	0	0	0	0	3	2	20	60
2	4	0	0	0	4	2	60	50

For peer review only

0	0	0	0	0	1	1	45	35
0	0	0	0	0	0	3	30	40
0	1	0	0	0	1	2	120	130
1	0	0	0	0	1	3	15	60
0	0	0	0	0	0	0	25	60
0	0	0	0	0	2	2	40	40
0	0	0	0	0	0	1	90	60
0	2	0	0	0	1	2	30	40
0	0	0	0	0	0	2	20	30
0	1	0	0	0	2	4	45	60
0	0	0	0	0	1	3	40	70
0	0	0	0	0	0	0	50	40
0	0	0	0	0	0	0	50	40
0	0	0	0	0	0	0	50	30
1	1	0	1	0	5	4	60	40
0	0	0	0	0	0	0	10	10
0	0	0	0	0	5	4	45	60
0	0	0	0	0	0	0	10	25
5	1	0	1	0	5	5	55	60
14	0	0	1	0	2	3	20	40
0	0	0	0	0	0	2	120	60
0	0	0	0	0	0	1	20	30
2	1	0	0	0	2	2	90	60
0	1	0	1	0	1	2	40	70
0	3	0	0	0	2	2	60	40
0	1	0	0	0	1	2	30	60
2	2	0	0	0	2	2	30	60
2	1	0	0	0	1	2	50	50
1	0	0	0	0	1	2	40	45
0	0	0	0	0	0	0	50	30
0	1	0	1	0	3	2	50	30
0	0	0	0	0	2	6	50	10
0	0	0	0	0	2	2	50	10
0	1	0	0	0	1	2	120	90
0	0	0	0	0	0	0	45	30
0	0	0	0	0	2	3	20	30
0	0	0	0	0	0	2	60	90
0	0	0	0	0	0	7	60	60
8	1	0	1	0	8	8	30	80
0	1	0	1	0	4	3	30	45
0	0	0	0	0	2	1	30	15
2	0	0	0	0	0	0	30	20
0	0	0	0	0	0	0	40	60
0	0	0	0	0	2	4	60	5
0	1	0	0	0	2	2	10	30
0	0	0	1	0	3	2	60	45
0	2	0	0	0	3	3	40	60
0	0	0	0	0	0	0	10	10
0	0	0	0	0	0	0	20	30
0	0	0	0	0	1	1	30	45
0	0	0	0	0	0	0	50	20
0	1	0	0	0	1	2	30	50

0	0	0	0	0	0	1	50	30
0	0	0	0	0	0	0	40	40
9	0	0	1	0	4	7	20	40
0	0	0	0	0	2	0	25	20
1	3	0	0	0	2	3	20	60
0	0	0	0	0	0	0	30	30
0	0	0	0	0	0	4	60	90
0	0	0	0	0	0	2	10	60
0	1	0	0	0	1	2	5	30
0	0	0	0	0	1	3	30	45
2	1	0	0	0	5	2	120	90
0	0	0	0	0	0	0	65	25
0	0	0	1	0	3	3	40	60
0	0	0	0	0	2	3	30	30
0	0	0	0	0	0	2	30	60
0	1	0	1	0	1	3	10	30
0	1	0	1	0	2	2	60	80
0	0	0	0	0	0	3	40	60
0	0	0	0	0	0	4	90	5
0	0	0	0	0	0	0	20	60
0	1	0	0	0	2	4	20	60
8	1	0	1	0	5	5	20	60
6	0	0	1	0	1	3	60	45
0	0	0	0	0	0	3	40	60
0	0	0	0	0	2	2	25	60
0	0	0	0	0	2	2	25	40
0	1	0	0	0	2	0	10	10
0	0	0	0	0	2	2	40	60
0	0	0	0	0	0	0	10	10
0	0	0	0	0	0	0	15	60
0	0	0	1	0	2	4	30	45
0	1	0	0	0	2	3	60	60
0	1	0	0	0	1	2	40	70
0	0	0	0	0	1	3	5	60
0	0	0	0	0	0	0	50	30
0	0	0	0	0	2	2	25	60
0	1	0	0	0	4	2	45	68
0	1	0	0	0	1	2	30	60
0	0	0	0	0	0	0	60	60
0	2	0	0	0	1	2	40	30
0	1	0	0	0	8	3	30	60
4	1	0	0	0	0	0	5	8
0	1	0	0	0	0	2	30	30
0	1	0	0	0	2	2	5	10
2	2	0	0	0	0	2	10	60
0	1	0	0	0	0	2	30	120
4	1	0	0	0	0	1	10	60
3	2	0	0	0	2	4	40	120
6	5	0	0	0	1	2	30	45
0	1	0	0	0	0	3	25	25
0	1	0	0	0	1	3	15	60
3	1	0	0	0	1	2	10	5

For peer review only

0	0	0	0	0	0	0	5	30
0	1	0	0	0	1	5	45	45
4	0	0	0	0	1	4	40	120
0	0	0	0	0	1	2	5	80
0	1	0	0	0	0	2	45	40
0	1	0	0	0	1	2	60	120
0	0	0	0	0	0	0	15	120
0	0	0	0	0	0	0	10	10
0	0	0	0	0	0	0	40	120
0	1	0	0	0	1	1	5	80
0	1	0	0	0	2	1	60	180
0	1	0	0	0	1	2	30	60
0	1	0	0	0	2	2	60	60
0	1	0	0	0	2	4	30	90
0	1	0	0	0	1	3	30	30
0	1	0	0	0	0	3	30	60
2	1	0	0	0	4	2	30	40
1	1	0	0	0	5	4	30	60
0	1	0	0	0	1	2	30	120
0	1	0	0	8	0	1	15	30
0	0	0	0	0	0	0	20	80
5	2	0	0	0	0	2	60	50
0	1	0	0	0	0	2	45	45
0	1	0	0	0	4	2	30	120
0	1	0	0	0	0	3	30	30
0	2	0	0	0	1	2	60	180
0	1	0	0	0	0	2	10	60
0	0	0	0	0	0	2	30	90
0	1	0	0	0	4	3	10	80
0	0	0	0	0	0	0	10	5
0	0	0	0	0	2	3	30	120
2	1	0	0	0	1	3	60	160
0	0	0	0	0	0	0	5	10
0	0	0	0	0	1	2	20	120
0	1	0	0	0	0	2	30	180
2	1	0	0	0	0	2	40	45
3	2	0	0	0	0	3	10	60
2	0	0	0	0	0	2	30	120
0	1	0	0	0	1	2	60	160
0	1	0	0	0	3	2	15	120
0	0	0	0	0	0	0	20	20
1	1	0	0	0	2	2	30	30
0	0	0	0	0	2	2	30	60
0	0	0	0	0	0	0	5	10
0	0	0	0	0	0	0	10	12
6	3	0	0	1	2	4	40	35
0	0	0	0	0	0	2	30	30
6	3	0	0	0	2	2	90	120
0	3	0	0	0	4	4	30	120
0	0	0	0	0	3	3	20	20
0	0	0	0	0	0	0	20	80
0	1	0	0	0	1	2	30	60

For peer review only

0	1	0	0	0	1	4	40	120
0	2	0	0	0	0	6	10	60
0	0	0	0	0	0	0	5	30
4	3	0	0	0	0	4	60	90
0	1	0	0	0	1	2	30	60
0	0	0	0	0	3	2	60	60
0	0	0	0	0	1	2	30	60
0	1	0	1	0	2	4	30	120
0	1	0	0	0	1	2	40	120
0	1	0	0	0	2	3	15	25
2	3	0	0	0	1	4	5	60
0	1	0	0	0	0	3	30	30
0	0	0	0	0	0	0	60	120
0	1	0	0	0	0	1	5	10
0	1	0	0	0	2	3	30	120
3	1	0	0	0	1	3	180	220
0	2	0	0	0	0	3	30	30
3	1	0	0	0	2	2	20	120
1	1	0	0	0	2	2	20	120
0	0	0	0	0	0	0	15	120
0	1	0	0	0	3	4	30	90
0	1	0	0	0	1	2	30	60
0	0	0	1	0	2	2	30	30
4	1	0	0	0	2	2	40	60
0	1	0	0	0	1	3	10	60
4	2	0	0	0	1	2	20	25
0	1	0	0	0	0	2	30	60
0	1	0	0	0	1	1	15	10
3	1	0	0	0	1	2	30	120
0	1	0	0	0	2	2	30	120
0	0	0	0	0	0	0	20	30
0	0	0	0	0	0	0	90	120
3	3	8	0	0	0	3	20	45
6	2	0	0	1	1	3	15	10
0	0	0	0	0	0	0	10	10
0	1	0	0	0	5	3	30	50
0	1	0	0	0	2	3	30	60
3	2	0	0	0	1	4	30	60
0	1	0	0	0	1	3	60	60
0	2	0	0	0	2	3	40	120
0	0	0	0	0	0	0	20	30
2	1	0	0	0	2	3	30	60
0	0	0	0	0	0	0	50	40
0	0	0	0	0	0	0	15	5
0	1	0	0	0	0	2	90	120
0	1	0	0	0	3	2	30	120
0	0	0	0	0	0	0	25	30
0	1	0	0	0	1	2	45	60
5	1	0	0	0	2	2	120	180
0	0	0	0	0	0	0	7	8
5	2	0	0	0	1	0	10	15
0	2	0	0	0	2	2	10	5

For peer review only

0	0	0	0	0	0	1	15	95
0	0	0	1	0	2	3	30	120
0	2	0	0	0	0	1	15	25
0	1	0	0	0	1	2	40	120
0	1	0	0	0	0	2	5	15
0	1	0	0	0	2	5	30	90
0	0	0	0	0	1	1	60	160
2	1	0	0	0	2	4	30	60
0	1	0	0	0	2	0	20	90
0	2	0	0	0	0	2	3	4
3	1	0	0	0	1	2	10	90
0	2	0	0	0	2	4	60	180
0	0	0	0	0	2	2	30	45
1	1	0	0	0	0	2	45	45
0	1	0	0	0	0	2	10	60
0	1	0	0	1	1	2	90	120
0	0	0	0	0	0	2	30	30
2	1	0	0	0	0	3	60	120
5	3	0	0	0	2	4	60	180
0	1	0	0	0	1	3	30	60
0	4	0	0	0	1	2	60	30
0	3	0	0	0	2	2	5	15
2	1	0	0	0	2	2	60	120
2	1	1	1	0	3	7	15	60
0	0	0	0	0	0	1	15	120
0	1	0	0	0	1	2	10	95
0	0	0	0	0	0	2	30	30
1	1	0	0	0	0	2	30	60
0	0	0	0	0	0	0	10	60
0	1	0	0	0	0	1	90	120
0	1	0	0	0	0	1	90	120
0	1	0	0	0	0	1	10	20
5	1	0	0	0	1	2	50	60
0	0	0	0	0	0	2	30	90
1	1	0	0	0	1	2	30	60
3	1	0	0	0	1	0	25	30
1	1	0	0	0	0	1	60	180
0	0	0	0	0	0	1	40	30
0	1	0	0	0	1	2	60	180
0	0	0	0	0	0	0	5	3
0	1	0	0	0	1	3	45	45
4	1	0	0	0	0	0	5	8
2	1	0	0	0	0	2	90	120
0	1	0	0	0	1	3	30	90
0	0	0	0	0	0	0	10	30
0	0	0	0	0	0	2	60	120
2	2	0	0	0	1	2	40	45
0	1	0	0	0	1	0	60	180
0	0	0	0	0	0	2	60	60
0	0	0	0	0	3	2	25	35
0	0	0	0	0	1	2	15	120
5	1	0	0	0	0	2	50	30

For peer review only

0	0	0	0	0	0	0	90	120
0	0	0	0	0	1	1	15	120
1	0	0	0	0	0	2	20	120
2	1	0	0	0	2	2	60	120
2	0	0	0	1	1	2	40	30
0	1	0	0	0	1	4	15	120
0	0	0	1	0	3	4	60	60
0	0	0	0	0	0	0	20	10
0	1	0	0	0	1	2	30	120
0	0	0	0	0	2	0	10	120
0	1	0	0	0	0	2	30	30
0	1	0	0	0	1	3	30	120
4	0	0	0	0	0	0	10	60
0	1	0	0	0	2	3	30	60
5	2	0	0	0	1	2	40	44
10	1	0	0	0	1	2	10	60
0	3	0	0	0	1	2	90	120
0	0	0	1	0	1	2	60	60
0	0	0	0	0	1	0	15	15
0	1	0	0	0	3	3	60	30
0	0	0	0	0	3	2	10	10
0	0	0	0	0	0	0	130	130
1	1	0	0	0	1	3	60	120
0	0	0	0	0	1	2	5	15
0	0	0	0	0	0	2	60	120
0	0	0	0	0	0	0	20	20
1	1	0	0	0	5	2	30	60
0	0	0	0	0	0	2	45	60
0	0	0	0	0	0	2	60	60
0	2	0	0	0	2	2	60	40
0	0	0	1	0	0	2	30	45
0	0	0	0	0	0	0	10	10
6	0	0	0	0	0	2	5	5
0	0	0	1	0	2	2	15	15
0	0	0	0	0	0	2	10	10
0	0	0	0	0	2	2	15	15
0	0	0	0	0	2	2	30	30
0	0	0	0	0	0	1	10	10
0	1	0	0	0	2	3	60	120
0	0	0	1	0	2	2	30	60
0	1	0	0	0	1	5	60	60
0	0	0	0	0	0	0	5	15
0	0	3	0	0	3	4	60	120
0	1	0	0	0	2	2	20	40
0	0	0	0	0	0	3	5	15
0	0	0	0	0	0	1	30	40
0	0	0	2	0	3	4	30	45
0	0	0	0	0	0	0	15	20
0	0	0	0	0	3	2	25	25
0	0	0	0	0	0	2	60	120
0	0	0	0	0	1	3	20	20
0	0	0	0	0	0	3	30	60

For peer review only

0	0	0	0	0	0	0	20	20
1	1	0	1	0	1	3	60	120
0	0	0	0	0	0	2	60	120
0	1	0	0	0	1	2	60	120
0	0	0	0	0	3	2	90	90
0	0	0	0	0	2	3	15	15
0	0	0	0	0	3	3	60	30
0	0	0	0	0	3	2	90	90
0	0	0	0	0	1	2	35	35
0	0	0	1	0	1	2	90	90
0	2	0	0	0	4	3	90	90
0	3	0	0	0	2	2	60	30
0	0	0	1	0	0	2	30	60
0	1	0	0	0	1	2	60	120
1	1	0	0	0	3	3	20	10
0	0	0	1	0	4	4	60	120
0	1	0	0	0	4	2	120	120
0	0	0	0	0	0	2	25	25
0	0	0	0	0	1	5	30	60
0	1	0	0	0	4	4	120	120
0	0	0	1	0	1	0	5	30
0	0	0	0	0	1	3	30	30
0	1	0	0	0	1	2	15	15
0	1	0	0	0	1	3	15	15
0	0	0	0	0	1	1	90	90
0	1	0	0	0	0	6	90	90
0	0	0	0	0	0	0	20	20
0	0	0	0	0	3	2	90	90
0	1	0	0	0	2	3	60	120
1	0	0	1	0	0	3	120	120
0	0	0	0	0	2	2	20	20
0	0	0	1	0	2	4	45	60
0	1	0	0	0	0	2	60	50
0	1	0	0	0	1	2	60	50
0	2	0	0	0	0	3	90	90
0	2	0	0	0	2	3	45	45
0	0	0	1	0	2	3	30	60
0	0	0	0	0	0	3	60	120
0	0	0	1	0	2	4	90	90
0	3	0	0	0	2	15	90	90
0	0	0	0	0	1	2	30	60
0	2	0	1	0	3	4	60	50
2	1	0	0	0	4	4	30	30
0	0	0	1	0	2	4	60	120
0	1	0	0	0	1	4	60	120
0	0	0	0	0	1	3	120	120
0	0	0	0	0	2	2	60	50
0	0	0	0	0	2	7	30	30
0	0	0	0	0	0	1	20	20
0	1	0	0	0	0	4	40	30
0	0	0	1	0	1	4	120	120
0	0	0	1	0	3	3	30	30

For peer review only

0	1	0	0	0	1	2	30	30
0	0	0	0	0	1	3	5	15
0	0	0	0	0	0	2	35	35
0	3	0	0	0	4	4	25	25
1	2	0	0	0	1	2	30	30
0	1	0	0	0	2	4	35	35
0	0	0	0	0	0	0	20	20
0	3	0	0	0	2	3	90	90
3	1	0	0	0	1	2	30	40
0	0	0	1	0	4	5	30	60
0	0	0	0	0	0	2	20	20
3	3	0	0	0	5	4	60	50
0	0	0	0	0	0	0	20	20
0	0	0	0	0	2	4	20	20
1	0	0	0	0	1	5	2	30
0	0	0	0	0	0	3	90	90
0	0	0	0	0	3	2	15	5
0	0	0	1	0	1	2	20	60
3	1	0	0	0	2	2	5	30
0	0	0	0	0	0	2	35	35
0	1	0	0	0	3	4	90	90
0	0	0	0	0	4	2	5	15
0	1	0	0	0	1	2	15	15
5	0	0	0	0	0	3	5	15
0	1	0	1	0	2	3	15	15
0	0	0	0	0	1	5	15	15
0	0	0	0	0	0	0	10	10
0	0	0	1	0	0	2	45	90
0	0	0	0	0	2	2	90	90
0	2	0	0	0	4	4	60	120
0	1	0	0	0	3	3	60	50
0	1	0	0	0	1	3	20	60
0	0	0	1	0	3	3	60	120
0	0	0	1	0	2	4	60	120
1	0	0	0	0	0	3	5	15
0	0	0	0	0	1	4	40	60
0	0	0	0	0	0	2	120	120
0	1	0	0	0	2	3	60	40
0	0	0	1	0	3	3	30	30
0	0	0	0	0	3	4	60	120
1	0	0	0	0	0	2	10	10
0	1	0	1	0	3	3	60	120
0	0	0	0	0	0	0	10	10
0	0	0	0	0	0	0	15	20
0	0	0	0	0	0	3	120	120
0	2	0	0	0	2	2	20	40
0	2	0	0	0	1	3	90	90
0	1	0	0	0	3	2	60	50
0	0	0	0	0	0	0	5	15
0	0	0	0	0	3	2	40	60
0	0	0	0	0	0	0	15	15
0	0	0	0	0	0	0	20	10

For peer review only

0	0	0	0	0	0	0	15	15
0	2	0	0	0	5	4	60	50
0	1	0	0	0	1	2	10	10
0	0	0	0	0	2	2	5	15
0	0	0	1	0	0	2	20	15
0	0	0	1	0	0	2	130	130
0	0	0	0	0	0	2	5	20
0	0	0	0	0	1	2	90	90
0	0	0	0	0	0	0	20	10
0	1	0	0	0	0	4	30	40
2	1	0	0	0	2	2	30	50
0	0	0	0	0	2	0	5	15
0	0	0	0	0	0	3	15	30
0	0	0	0	0	0	0	20	10
0	0	0	0	0	0	0	5	15
0	0	0	0	0	0	0	20	10
0	0	0	0	0	0	2	30	30
0	4	0	0	0	4	4	60	50
0	0	0	1	0	3	6	60	120
0	1	0	0	0	0	3	30	30
0	1	0	1	0	2	2	30	60
1	1	0	0	0	2	4	60	120
2	0	0	1	0	2	2	2	30
0	0	0	1	0	3	3	90	90
0	1	0	0	0	1	2	30	45
0	1	0	0	0	2	4	90	90
0	1	0	0	0	0	1	90	90
0	0	0	0	0	1	5	45	60
0	0	0	0	0	0	0	30	30
0	0	0	1	0	1	3	30	45
0	0	0	0	0	0	3	40	60
0	0	0	0	0	2	1	120	120
0	0	0	1	0	1	2	120	120
0	2	1	0	0	4	5	90	90
0	2	0	0	0	2	3	60	50
0	0	0	0	0	0	1	5	15
0	1	0	1	0	4	4	60	60
0	0	0	0	0	0	0	20	20
0	0	0	1	0	4	3	60	120
0	0	0	0	0	3	3	120	120
0	1	0	0	0	3	4	35	35
0	1	0	0	0	1	3	90	90
0	0	0	1	0	1	3	10	10
0	1	0	0	0	1	1	90	90
0	0	0	1	0	2	2	30	30
0	0	0	1	0	6	2	90	90
0	1	0	0	0	1	2	10	120
0	1	0	0	0	0	6	30	60
0	1	0	0	0	0	2	30	60
0	1	0	0	0	2	3	30	75
2	0	0	0	0	1	0	10	60
0	0	0	0	0	0	0	60	25

For peer review only

0	1	0	0	0	1	0	25	120
0	0	0	0	0	0	0	40	5
0	2	0	0	0	1	2	25	40
0	0	0	0	0	0	0	40	30
0	1	0	0	0	0	0	20	120
0	1	0	0	0	0	2	30	180
6	3	0	0	0	0	1	15	60
7	2	0	0	0	3	3	20	60
0	2	0	0	0	1	4	30	120
3	1	0	0	0	1	2	35	150
0	0	0	0	0	0	0	15	120
0	4	0	0	0	1	2	20	120
0	1	0	0	0	2	0	15	150
0	0	0	0	0	0	0	30	120
3	0	0	0	0	0	2	15	95
3	1	0	0	1	1	2	40	60
0	0	0	0	0	0	0	30	90
5	4	0	0	0	0	4	40	150
0	0	0	0	0	0	0	40	120
0	1	0	0	0	0	2	15	120
0	1	0	0	0	0	1	5	20
2	0	0	0	0	1	1	25	120
0	0	0	0	0	0	0	30	120
0	1	0	0	0	2	1	15	40
0	2	0	0	0	0	0	20	130
0	0	0	0	0	0	0	40	5
7	2	0	0	0	6	2	30	30
0	0	0	0	0	0	0	45	10
2	1	0	0	0	2	4	45	120
0	1	0	0	0	1	2	40	120
0	2	0	0	0	1	4	60	60
0	0	0	0	0	0	0	30	60
9	2	0	0	0	2	2	30	160
0	0	0	0	0	0	0	30	120
5	1	0	0	0	1	2	30	120
0	2	0	0	0	0	4	25	150
0	1	1	0	0	2	4	10	120
1	1	0	0	0	0	2	30	60
0	1	0	0	0	0	4	45	90
4	1	0	0	0	1	2	10	25
0	1	0	0	0	1	2	30	120
0	0	0	0	0	0	0	60	120
4	2	0	0	0	0	2	40	60
0	1	1	0	0	0	2	30	180
4	1	0	0	0	1	3	30	90
0	2	0	0	0	0	1	10	25
3	1	0	0	0	1	2	20	80
0	1	0	0	0	1	4	15	120
0	1	0	0	0	1	2	25	60
2	1	0	0	0	0	2	20	130
0	1	0	0	0	0	2	25	45
0	1	1	1	1	0	2	15	90

For peer review only

0	0	0	0	0	0	0	35	120
0	0	0	0	0	1	2	15	115
0	2	0	0	0	2	4	10	120
2	0	0	0	0	0	5	60	90
0	0	0	0	0	0	0	20	60
0	2	0	0	0	1	2	30	120
5	2	0	0	0	0	4	25	120
5	2	0	0	0	1	2	5	120
0	1	0	0	0	1	2	15	120
3	2	0	0	0	0	2	15	60
7	1	0	0	0	0	1	160	30
5	1	0	0	0	1	3	25	110
2	2	0	0	0	4	3	30	120
0	1	0	0	0	0	5	30	60
3	1	0	0	0	2	4	5	15
2	0	0	0	0	1	2	30	110
0	2	0	0	0	0	0	30	120
2	1	0	0	0	1	3	30	90
0	0	0	0	0	0	0	60	15
0	2	0	0	0	2	2	20	60
0	0	0	0	0	0	0	35	120
0	1	0	0	0	2	0	30	40
0	0	0	0	0	1	3	25	110
3	1	0	0	0	1	2	30	90
0	2	0	0	0	1	3	30	120
4	3	0	0	0	1	3	10	180
0	0	0	0	0	0	0	20	150
5	2	0	0	0	1	2	5	30
0	1	0	0	0	0	2	30	60
0	1	0	0	0	0	3	45	60
0	0	0	0	0	0	0	30	120
0	1	0	0	0	2	3	20	80
0	0	0	0	0	0	0	60	5
0	1	0	0	0	1	2	30	120
0	0	0	0	0	0	0	35	120
0	1	0	0	0	1	2	30	120
0	0	0	0	0	0	0	15	80
0	0	0	0	0	2	1	30	60
3	2	0	0	0	2	3	60	45
0	2	0	0	0	0	2	40	120
0	2	0	0	0	1	2	40	150
0	0	0	0	0	0	0	40	90
0	2	0	0	0	1	0	30	150
0	1	0	0	0	1	2	15	95
2	2	0	1	0	0	2	30	60
0	1	0	0	0	0	0	30	15
0	0	0	0	0	0	0	40	5
0	1	0	0	0	0	1	30	60
0	2	0	0	0	2	4	45	60
0	4	0	0	1	0	2	90	180
0	0	0	0	0	0	0	30	60
0	2	0	0	0	2	2	30	120

For peer review only

0	1	0	0	0	0	1	10	60
0	2	0	0	0	1	3	10	120
0	1	0	0	0	0	2	15	90
4	3	0	0	0	3	8	60	90
0	1	0	0	0	0	0	30	120
0	0	0	0	0	0	0	60	5
0	0	0	0	0	0	2	10	90
0	0	0	0	0	0	4	20	120
1	1	0	0	0	2	2	20	80
5	0	0	0	0	0	0	60	5
0	2	0	0	0	1	2	30	45
0	4	0	0	0	1	4	30	120
0	2	0	0	0	1	2	40	90
4	1	0	0	0	1	2	25	120
5	1	0	0	0	2	2	40	180
0	1	0	0	0	0	0	15	120
5	1	0	0	0	1	2	60	180
0	0	0	0	0	0	0	35	90
6	4	0	0	0	4	6	60	30
0	0	0	0	0	0	0	25	50
0	2	0	0	0	2	2	40	150
0	0	0	0	0	0	0	20	120
3	1	0	0	0	1	2	40	100
0	0	0	0	0	0	0	30	120
0	0	0	0	0	0	0	45	10
0	0	0	0	0	0	0	30	10
0	0	0	0	0	0	0	60	10
0	1	0	0	0	1	2	30	180
2	1	0	0	0	0	0	60	120
0	1	0	0	0	0	5	60	90
10	3	0	0	0	1	0	60	5
0	1	0	0	0	0	0	15	120
0	0	0	0	0	0	0	15	120
0	1	0	0	0	1	2	30	120
1	2	0	0	0	1	3	10	90
0	1	0	0	0	2	2	15	120
0	2	0	0	0	0	2	40	120
0	0	0	0	0	0	0	35	150
0	3	0	0	0	1	3	30	120
4	2	0	0	0	2	4	30	80
0	1	0	0	0	1	0	30	120
0	1	0	0	0	1	2	30	45
0	1	0	0	0	0	2	30	120
0	1	0	0	0	2	2	25	120
3	1	0	0	0	0	2	15	90
0	0	0	0	0	0	0	5	120
0	0	0	0	0	2	2	20	110
1	1	0	0	1	0	2	60	120
3	2	0	0	0	0	3	30	150
0	0	0	0	0	2	2	20	60
0	0	0	0	0	0	0	60	10
0	0	0	0	0	0	0	60	10

For peer review only

0	1	0	0	0	0	2	20	80
0	2	0	0	0	1	2	30	120
0	1	0	0	0	2	2	20	60
0	0	0	0	0	0	0	30	60
0	1	0	0	0	0	0	30	15
0	0	0	0	0	0	0	30	180
0	1	0	0	0	0	0	30	120
0	0	0	0	0	0	0	20	120
0	0	0	0	0	0	0	60	300
0	2	0	0	0	2	1	30	90
0	0	0	0	0	1	2	20	120
5	1	0	0	0	1	2	15	20
0	3	0	0	0	2	2	5	60
0	2	1	0	0	0	0	15	150
0	1	0	0	0	0	2	90	120
3	2	0	0	0	1	4	45	90
0	1	0	0	0	0	0	30	120
0	2	0	0	0	0	0	20	120
0	2	0	0	0	0	2	40	120
9	1	0	0	0	5	2	30	90
0	0	0	0	0	0	0	40	10
0	1	0	0	0	0	0	30	120
0	1	0	0	0	0	1	30	150
0	1	0	0	0	0	0	30	90
0	0	0	0	0	0	0	30	90
0	2	0	0	0	2	3	1	30
2	2	0	0	0	0	2	20	60
0	3	0	0	0	2	2	45	120
3	1	0	0	0	0	2	30	95
2	1	0	0	0	1	2	60	120
0	2	0	0	0	0	2	30	120
0	2	0	0	0	2	4	30	90
0	1	0	0	0	1	0	30	120
0	0	0	0	0	0	0	40	5
3	2	0	0	0	1	4	30	120
0	0	0	0	0	0	0	40	80
1	1	0	0	0	1	2	30	90
0	0	0	0	0	0	0	35	120
0	2	0	0	0	0	2	30	120
0	1	0	0	0	0	1	30	60
2	0	0	0	0	1	2	20	60
0	0	0	0	0	0	0	40	90
0	1	0	0	0	0	2	15	60
0	1	0	0	0	0	3	5	120
0	1	0	0	0	2	2	20	120
0	0	0	0	0	0	0	40	240
0	2	0	0	0	0	4	45	90
0	0	0	0	0	0	0	60	120
0	1	0	0	0	0	1	20	40
2	1	0	0	0	1	3	30	90
2	3	1	0	0	2	4	15	90
2	2	0	0	0	1	2	30	90

2	1	0	0	0	1	2	20	130
0	1	0	0	0	1	2	60	15
0	0	0	0	0	0	0	35	60
0	1	0	0	0	1	4	30	120
0	1	0	0	0	1	4	15	90
1	1	0	0	0	2	4	40	100
0	1	0	0	0	2	4	40	100
7	1	0	0	0	0	0	45	180
3	2	0	0	0	1	2	30	90
5	2	0	0	0	1	2	30	150
0	1	0	0	0	0	0	30	60
0	2	0	0	0	0	4	20	120
4	1	0	0	0	1	2	35	120
0	0	0	0	0	0	0	40	200
0	0	0	0	0	0	0	60	5
3	0	0	0	0	0	0	15	60
0	0	0	0	0	0	0	40	5
6	2	0	0	0	1	3	30	120
0	0	0	0	0	0	0	25	25
0	0	0	0	0	0	0	30	15
14	3	0	0	0	1	5	30	120
3	2	0	0	0	1	2	30	120
7	1	0	0	0	0	2	30	90
3	2	0	0	0	2	3	20	80
6	1	1	0	0	0	2	30	120
0	0	0	0	0	0	0	35	120
0	1	0	0	0	0	5	60	90
0	2	0	0	0	1	4	30	90
0	4	0	0	0	2	4	20	120
5	2	0	0	0	1	2	10	30
0	1	0	0	0	2	2	160	30
0	2	0	0	0	2	2	25	120
0	0	0	0	0	0	0	30	60
0	0	0	0	0	0	0	40	180
0	0	0	0	0	0	0	35	90
0	0	0	0	0	0	2	30	120
0	1	0	0	0	0	2	5	120
2	1	0	0	0	1	2	20	80
3	1	0	0	0	0	2	30	150
3	0	0	0	0	1	2	25	100
7	3	1	0	0	1	6	15	150
0	0	0	0	0	0	0	30	120
6	2	0	0	0	2	2	25	95
4	2	0	0	0	1	3	30	180
0	0	0	0	0	0	0	60	120
5	3	0	0	0	2	4	45	30
0	2	0	0	0	1	2	40	150
2	1	0	0	0	0	2	15	90
0	0	0	0	0	0	0	30	150
1	0	0	0	0	0	1	60	180
0	1	0	0	0	0	2	25	120
0	0	0	0	0	0	0	20	120

0	0	0	0	0	0	0	10	20
0	3	0	0	0	1	0	25	120
0	0	0	0	0	0	0	15	60
0	0	0	0	0	0	0	10	180
0	1	0	0	0	2	2	5	60
0	0	0	0	0	0	0	10	25
0	1	0	0	0	0	1	30	120
0	0	0	0	0	0	0	30	180
0	0	0	0	0	2	2	20	110
0	0	0	0	0	0	0	20	120
3	1	0	0	0	4	2	20	120
0	0	0	0	0	0	0	35	120
0	0	0	0	0	0	1	40	120
0	0	0	0	0	0	0	15	120
0	1	0	0	0	1	3	25	110
0	1	0	0	0	0	0	30	120
0	1	0	0	0	1	2	25	120
0	0	0	0	0	0	0	30	120
0	1	0	0	0	1	1	30	90
10	2	0	0	0	1	1	50	25
2	1	0	0	0	2	4	40	100
0	1	0	0	0	0	2	25	100
0	1	0	0	0	0	2	30	120
0	3	0	0	0	1	2	35	120
0	0	0	0	0	0	0	40	5
0	3	0	0	0	4	6	60	120
0	1	0	0	0	1	1	20	110
0	0	0	0	0	0	0	40	10
0	3	0	0	0	1	2	15	25
6	3	0	0	0	1	4	30	120
9	2	0	0	0	2	4	30	90
3	0	0	0	0	0	0	60	10
0	0	0	0	0	0	0	25	150
2	2	0	0	0	1	3	60	20
4	1	0	0	0	1	2	20	80
0	3	0	0	0	1	2	20	40
0	0	0	0	0	0	0	5	60
0	0	0	0	0	0	0	30	90
3	2	0	0	0	0	2	30	120
0	1	0	0	0	0	2	60	120
1	1	0	1	0	2	2	20	80
0	0	0	0	0	0	0	30	90
2	3	0	0	0	1	5	30	90
0	3	0	0	0	1	2	25	120
0	3	1	0	0	0	4	30	150
0	0	0	0	0	1	0	30	150
3	1	0	0	0	1	2	40	100
10	4	0	0	0	0	6	10	180
5	2	0	0	0	0	2	20	45
3	1	0	0	0	0	1	15	60
4	3	0	0	2	3	5	30	90
4	3	0	0	2	4	8	90	45

For peer review only

4	1	0	0	0	0	1	25	120
0	1	0	0	0	1	2	20	120
23	2	1	0	0	1	4	20	120
0	0	0	0	0	0	0	30	120
0	3	1	0	0	0	0	30	150
0	1	0	0	0	1	1	60	240
0	1	0	0	0	0	1	30	100
4	1	0	0	0	2	3	40	100
2	2	0	0	0	1	4	30	180
0	3	0	0	0	1	3	60	120
0	1	0	0	0	0	1	30	100
4	2	0	0	0	3	2	160	30
3	2	0	0	0	2	2	30	90
0	0	0	0	0	0	0	25	25
0	0	0	0	0	1	1	30	120
0	1	0	0	0	0	2	15	120
1	0	0	0	0	0	2	10	30
0	0	0	0	0	0	0	25	120
3	1	0	0	0	0	2	20	100
0	0	0	0	0	0	0	30	120
0	1	0	0	0	1	2	15	90
8	3	0	0	0	1	2	40	120
0	1	0	0	0	1	2	30	160
0	2	0	0	0	1	2	30	120
0	0	0	0	0	0	0	25	120
0	1	1	0	0	1	2	25	90
0	0	0	0	0	0	0	30	120
1	2	0	0	0	1	1	30	60
0	1	0	0	0	1	0	15	120
0	2	0	0	0	2	4	30	120
0	1	0	0	0	0	2	15	90
0	0	0	0	0	0	0	2	80
0	2	0	0	0	0	2	15	120
2	4	0	0	0	2	4	60	120
0	0	0	0	0	0	0	30	150
0	0	0	0	0	0	6	45	60
4	2	0	0	0	1	3	45	90
4	2	0	0	0	0	2	30	60
0	0	0	0	0	0	0	10	120
0	0	0	0	0	0	0	30	60
0	0	0	0	0	0	0	10	150
0	1	0	0	0	0	0	15	180
0	0	0	0	0	0	0	60	120
0	2	0	0	0	0	0	45	20
0	0	0	0	0	0	0	20	145
0	1	0	0	0	5	2	20	150
8	2	0	0	0	1	2	10	25
4	2	0	0	0	2	3	60	120
0	3	0	0	0	4	4	30	120
3	4	0	0	0	2	2	30	120
0	1	0	0	0	1	2	30	90

For peer review only

q301	q302	q303	q304	q305	q306	q307	q308	q309
Yes	Satisfied	No	Yes	No				No
Yes	Satisfied	No	Yes	No				No
No		No	Yes	No				No
No		No	No					
Yes	Satisfied	No	Yes	No				No
Yes	Satisfied	No	No					
No		No	No					
No		No	No					
No		No	No					
Yes	Satisfied	No	No					
Yes	Satisfied	No	Yes	Yes	1 to 5			
Yes	Satisfied	No	Yes	No				No
Yes	Satisfied	No	No					
No		No	No					
No		No	No					
Yes	Satisfied	No	Yes	No				No
No		No	No					
No		No	Yes	No				No
Yes	Satisfied	No	No					
Yes	Satisfied	No	Yes	No				Yes
No		No	No					
No		No	Yes	No				No
Yes	Satisfied	No	Yes	No				No
Yes	Satisfied	No	No					
Yes	Not satisfie	No	Yes	No				No
No		No	No					
No		No	Yes	No				No
Yes	Very satisfi	No	Yes	No				No
Yes	Not satisfie	No	Yes	No				No
No		No	No					
No		No	Yes	No				No
Yes	Satisfied	No	Yes	No				No
Yes	Satisfied	No	No					
No		No	No					
Yes	Satisfied	No	Yes	No				No
No		No	Yes	No				No
No		No	No					
No		No	No					
No		No	No					
No		No	Yes	No				No
No		No	No					
No		No	Yes	No				No
No		No	Yes	No				No
No		No	No					
No		No	No					
No		No	Yes	No				Yes
Yes	Satisfied	No	No					
Yes	Satisfied	No	Yes	No				No
Yes	Not satisfie	No	Yes	No				No
Yes	Satisfied	No	Yes	No				No
No		No	No					

For peer review only

No		No	No			
No		No	No			
Yes	Satisfied	Yes	Yes	No		Yes
No		No	Yes	No		No
Yes	Satisfied	No	Yes	No		No
Yes	Satisfied	No	No			
No		No	Yes	No		No
No		No	No			
Yes	Satisfied	No	Yes	No		No
No		No	Yes	No		No
No		No	No			
No		No	No			
Yes	Satisfied	No	Yes	No		No
Yes	Satisfied	No	Yes	No		No
No		No	Yes	No		No
Yes	Satisfied	No	Yes	No		No
Yes	Satisfied	No	Yes	No		No
No		No	No			
Yes	Satisfied	No	Yes	No		No
Yes	Satisfied	No	No			
Yes	Satisfied	No	Yes	No		No
No		No	Yes	No		No
No		No	No			
No		No	No			
Yes	Satisfied	No	Yes	No		No
Yes	Satisfied	No	Yes	No		No
No		No	Yes	No		No
No		No	Yes	No		Yes
No		No	Yes	No		No
No		No	No			
No		No	No			
No		No	No			
No		No	No			
No		No	No			
No		No	No			
Yes	Not satisfie	No	No			
No		No	No			
Yes	Satisfied	No	Yes	No		No
No		No	No			
No		No	No			
Yes	Satisfied	No	No			
No		No	Yes	No		No
No		No	Yes	No		No
No		No	No			
No		No	No			
Yes	Satisfied	No	Yes	No		No
No		No	No			
No		No	No			
No		No	No			
No		No	No			
No		No	No			
Yes	Satisfied	No	Yes	Yes	1 to 30	

For peer review only

No		No	No			
Yes	Very satisfi	No	Yes	No		No
No		No	No			
No		No	No			
No		No	Yes	No		No
No		No	No			
No		No	Yes	Yes	1 to 30	
No		No	No			
No		No	No			
No		No	Yes	No		No
Yes	Satisfied	Yes	Yes	No		No
Yes	Satisfied	No	No			
No		No	Yes	No		Yes
Yes	Satisfied	No	No			
Yes	Satisfied	No	No			
Yes	Satisfied	No	No			
Yes	Very satisfi	No	Yes	Yes	1 to 30	
No		No	Yes	No		No
Yes	Satisfied	No	No			
No		No	No			
No		No	Yes	No		No
No		No	Yes	No		No
No		No	No			
Yes	Satisfied	No	Yes	No		No
Yes	Not satisfie	Yes	Yes	Yes	1 to 30	
Yes	Satisfied	No	Yes	No		No
Yes	Satisfied	Yes	Yes	No		No
No		No	No			
No		No	No			
No		No	No			
Yes	Satisfied	Yes	Yes	No		Yes
Yes	Satisfied	No	Yes	No		Yes
Yes	Satisfied	No	Yes	No		No
Yes	Satisfied	Yes	Yes	No		Yes
Yes	Satisfied	No	No			
Yes	Satisfied	No	Yes	No		No
Yes	Satisfied	No	Yes	No		No
Yes	Satisfied	No	No			
Yes	Satisfied	No	Yes	No		Yes
No		No	No			
Yes	Satisfied	No	No			
Yes	Satisfied	No	No			
Yes	Satisfied	Yes	Yes	No		No
Yes	Satisfied	No	Yes	No		No
No		No	Yes	No		No
No		No	No			
Yes	Satisfied	No	No			
Yes	Satisfied	Yes	Yes	No		Yes
No		No	Yes	No		No
No		No	No			
Yes	Satisfied	No	Yes	No		No
Yes	Satisfied	No	No			

For peer review only

Yes	Satisfied	No	Yes	No		No
Yes	Satisfied	No	No			
Yes	Satisfied	No	Yes	No		No
Yes	Satisfied	No	Yes	No		No
No		No	No			
Yes	Not satisfie	No	No			
No		Yes	Yes	No		No
No		No	No			
Yes	Satisfied	No	Yes	No		No
No		No	Yes	No		No
No		No	No			
Yes	Satisfied	Yes	Yes	No		Yes
Yes	Satisfied	No	No			
Yes	Satisfied	No	No			
No		No	No			
No		No	No			
No		Yes	Yes	No		Yes
Yes	Satisfied	No	No			
Yes	Very satisfi	No	Yes	No		No
No		No	Yes	No		No
Yes	Satisfied	No	No			
Yes	Satisfied	No	Yes	No		Yes
Yes	Satisfied	No	Yes	No		Yes
No		No	No			
Yes	Satisfied	Yes	Yes	No		Yes
Yes	Very satisfi	No	Yes	No		No
Yes	Satisfied	Yes	Yes	No		Yes
Yes	Satisfied	Yes	Yes	No		Yes
Yes	Satisfied	No	No			
Yes	Satisfied	Yes	Yes	No		Yes
Yes	Satisfied	Yes	No			
Yes	Satisfied	No	No			
Yes	Satisfied	No	No			
Yes	Satisfied	Yes	Yes	No		Yes
No		No	No			
No		No	Yes	No		No
Yes	Satisfied	No	Yes	No		No
No		No	Yes	Yes	1 to 30	
No		No	Yes	No		No
No		No	No			
No		No	No			
Yes	Satisfied	Yes	Yes	No		Yes
Yes	Satisfied	Yes	Yes	No		Yes
Yes	Satisfied	No	No			
Yes	Satisfied	No	Yes	No		Yes
Yes	Satisfied	No	No			
No		No	No			
Yes	Satisfied	Yes	Yes	No		No
Yes	Satisfied	Yes	Yes	No		Yes
Yes	Satisfied	No	Yes	No		No
No		No	Yes	No		No
No		No	No			

For peer review only

No		No	No			
No		No	No			
Yes	Satisfied	No	Yes	No		No
No		No	No			
Yes	Satisfied	No	No			
No		No	No			
No		No	Yes	No		No
Yes	Satisfied	No	Yes	No		No
Yes	Satisfied	No	No			
Yes	Satisfied	No	Yes	No		No
Yes	Satisfied	Yes	Yes	No		No
Yes	Satisfied	No	Yes	No		No
Yes	Satisfied	Yes	Yes	No		Yes
No		No	No			
Yes	Satisfied	No	Yes	No		No
Yes	Satisfied	No	Yes	No		No
No		No	No			
No		No	No			
No		No	No			
No		No	No			
No		No	No			
Yes	Very satisfi	No	Yes	Yes	1 to 5	
No		No	No			
No		No	No			
No		No	No			
No		No	No			
No		No	No			
No		No	No			
Yes	Satisfied	No	No			
Yes	Satisfied	No	Yes	No		No
No		No	No			
Yes	Satisfied	No	No			
Yes	Satisfied	No	No			
No		No	No			
No		No	No			
Yes	Satisfied	No	No			
No		No	No			
Yes	Satisfied	No	No			
No		No	No			
Yes	Satisfied	No	Yes	No		No
No		No	No			
No		No	No			
No		No	No			
No		No	No			
Yes	Satisfied	No	No			
No		No	No			
Yes	Satisfied	No	Yes	No		No
No		No	No			
No		No	No			
No		No	No			
No		No	No			
Yes	Not satisfie	No	No			

For peer review only

Yes	Satisfied	No	Yes	No	No
No		No	No		
No		No	No		
No		No	No		
No		No	No		
Yes	Satisfied	Yes	Yes	No	No
Yes	Satisfied	No	No		
Yes	Not satisfie	No	No		
No		No	Yes	No	No
Yes	Satisfied	No	No		
Yes	Satisfied	No	No		
No		No	No		
No		No	No		
Yes	Satisfied	No	Yes	No	No
No		No	No		
No		No	No		
No		No	No		
No		No	No		
No		No	No		
No		No	No		
No		No	No		
Yes	Satisfied	No	Yes	No	No
No		No	Yes	No	No
Yes	Satisfied	No	No		
Yes	Satisfied	Yes	Yes	No	No
No		Yes	Yes	No	No
No		No	No		
No		No	No		
Yes	Satisfied	No	No		
No		No	No		
No		No	No		
Yes	Satisfied	No	Yes	No	No
No		No	No		
No		No	No		
No		No	No		
Yes	Satisfied	No	No		
No		No	No		
No		No	No		
No		No	No		
No		No	No		
Yes	Very satisfi	No	No		
Yes	Satisfied	No	Yes	No	No
Yes	Satisfied	No	No		
No		No	No		
Yes	Not satisfie	No	No		
No		No	No		
No		No	No		
No		No	No		
Yes	Satisfied	Yes	Yes	No	Yes
Yes	Satisfied	Donâ€™t k	No		
No		No	No		
No		No	No		

For peer review only

No		No	No			
No		No	No			
No		No	Yes	No		No
No		No	No			
Yes	Satisfied	No	Yes	No		No
Yes	Satisfied	No	No			
Yes	Satisfied	No	No			
Yes	Very satisfi	Yes	Yes	No		No
Yes	Satisfied	No	No			
No		No	No			
No		No	No			
Yes	Satisfied	No	No			
Yes	Satisfied	No	Yes	No		No
No		No	No			
No		No	No			
Yes	Very satisfi	No	No			
Yes	Satisfied	No	No			
No		No	No			
Yes	Satisfied	No	Yes	No		No
No		No	No			
No		No	No			
Yes	Satisfied	No	No			
Yes	Satisfied	No	Yes	No		No
Yes	Satisfied	No	Yes	No		No
Yes	Satisfied	No	Yes	No		No
No		No	No			
No		No	No			
No		No	No			
Yes	Satisfied	No	No			
Yes	Satisfied	Yes	Yes	No		Yes
Yes	Satisfied	No	No			
Yes	Satisfied	Yes	No			
Yes	Satisfied	No	No			
Yes	Satisfied	No	No			
Yes	Satisfied	No	No			
No		No	No			
Yes	Satisfied	No	No			
No		No	No			
Yes	Satisfied	Yes	Yes	Yes	1 to 5	
Yes	Satisfied	No	No			
Yes	Satisfied	Yes	No			
Yes	Satisfied	Yes	No			
Yes	Satisfied	Yes	No			
Yes	Satisfied	Yes	No			
Yes	Satisfied	No	No			
Yes	Satisfied	Yes	No			
Yes	Satisfied	No	No			
Yes	Satisfied	No	No			
Yes	Satisfied	No	No			
Yes	Satisfied	Yes	No			
Yes	Satisfied	Yes	No			
No		No	Yes	No		No

For peer review only

Yes	Satisfied	Yes	No			
Yes	Satisfied	No	No			
No		Yes	No			
Yes	Satisfied	Yes	No			
Yes	Satisfied	Yes	No			
Yes	Satisfied	Yes	No			
Yes	Satisfied	No	No			
Yes	Satisfied	Yes	No			
No		No	No			
Yes	Satisfied	Yes	No			
Yes	Satisfied	Yes	No			
Yes	Satisfied	Yes	No			
Yes	Satisfied	No	No			
No		No	Yes	No		No
No		No	No			
No		No	Yes	No		No
Yes	Satisfied	Yes	No			
Yes	Satisfied	Yes	No			
Yes	Satisfied	Yes	No			
No		No	No			
No		No	No			
Yes	Satisfied	No	No			
Yes	Satisfied	Yes	Yes	No		No
Yes	Satisfied	No	No			
Yes	Satisfied	Yes	Yes	Yes	1 to 5	
Yes	Satisfied	No	No			
No		No	No			
Yes	Satisfied	Yes	No			
Yes	Satisfied	Yes	No			
Yes	Satisfied	Yes	No			
No		No	No			
Yes	Satisfied	Yes	No			
Yes	Satisfied	No	No			
Yes	Satisfied	No	No			
No		No	No			
No		No	No			
Yes	Satisfied	No	No			
Yes	Satisfied	No	No			
Yes	Satisfied	No	No			
No		No	No			
Yes	Satisfied	Yes	No			
Yes	Satisfied	No	No			
No		Yes	No			
No		Yes	No			
Yes	Satisfied	No	No			
Yes	Satisfied	No	No			
No		No	No			
Yes	Satisfied	Yes	Yes	No		No
No		No	No			
Yes	Satisfied	Yes	No			
Yes	Satisfied	No	No			
Yes	Satisfied	No	No			

Yes	Satisfied	No	No		
Yes	Satisfied	Yes	No		
Yes	Satisfied	Yes	No		
Yes	Satisfied	Yes	No		
No		Yes	No		
Yes	Satisfied	No	No		
No		No	No		
Yes	Satisfied	No	No		
Yes	Satisfied	Yes	Yes	No	No
Yes	Satisfied	No	No		
Yes	Satisfied	Yes	Yes	No	Yes
No		No	No		
No		No	Yes	No	No
Yes	Satisfied	No	No		
Yes	Satisfied	No	Yes	No	No
Yes	Satisfied	No	No		
No		No	No		
No		No	No		
No		No	No		
Yes	Satisfied	Yes	No		
Yes	Satisfied	Yes	Yes	No	No
Yes	Satisfied	No	Yes	No	No
No		No	No		
Yes	Satisfied	No	No		
No		No	No		
No		No	No		
No		No	No		
Yes	Satisfied	Yes	No		
No		No	No		
No		No	No		
Yes	Satisfied	Yes	Yes	No	No
Yes	Satisfied	No	No		
Yes	Satisfied	Yes	No		
Yes	Satisfied	No	No		
No		No	No		
Yes	Satisfied	No	No		
No		No	No		
Yes	Satisfied	Yes	Yes	No	No
Yes	Satisfied	No	No		
No		No	No		
Yes	Satisfied	Yes	Yes	No	No
No		No	No		
Yes	Satisfied	No	No		
Yes	Satisfied	No	Yes	No	No
No		No	No		
Yes	Satisfied	No	No		
Yes	Satisfied	Yes	Yes	No	Yes
No		No	No		
Yes	Satisfied	No	No		
Yes	Satisfied	No	Yes	No	No
Yes	Satisfied	Yes	Yes	No	Yes
Yes	Satisfied	No	No		

For peer review only

No		No	No					
Yes	Satisfied	No	Yes	No				No
Yes	Satisfied	Yes	Yes	No				No
Yes	Satisfied	No	No					
Yes	Satisfied	Yes	Yes	No				No
Yes	Satisfied	Yes	Yes	No				No
No		No	No					
Yes	Satisfied	No	No					
No		No	No					
No		Yes	No					
No		No	No					
Yes	Satisfied	No	No					
Yes	Satisfied	No	No					
No		No	No					
Yes	Satisfied	Yes	No					
No		No	No					
No		No	No					
Yes	Very satisfi	Yes	Yes	Yes	1 to 30			
Yes	Satisfied	Yes	No					
Yes	Satisfied	Yes	No					
Yes	Satisfied	Yes	No					
Yes	Satisfied	No	No					
No		No	No					
Yes	Satisfied	No	No					
No		No	No					
Yes	Very satisfi	No	No					
Yes	Very satisfi	No	Yes	No				No
Yes	Very satisfi	No	No					
No		No	No					
Yes	Very satisfi	No	Yes	No				No
Yes	Satisfied	No	Yes	No				No
No		No	No					
Yes	Very satisfi	No	No					
Yes	Not satisfie	No	No					
Yes	Satisfied	Yes	No					
No		No	No					
Yes	Satisfied	Yes	No					
Yes	Satisfied	No	No					
No		No	No					
Yes	Satisfied	No	No					
Yes	Satisfied	No	No					
No		No	No					
No		No	No					
Yes	Satisfied	No	No					
Yes	Satisfied	No	No					
Yes	Not satisfie	No	Yes	No				No
No		No	No					
Yes	Satisfied	No	No					
Yes	Satisfied	No	No					
Yes	Satisfied	No	No					
Yes	Satisfied	No	No					
No		No	No					

For peer review only

No		No	No		
Yes	Very satisfi	No	No		
No		No	No		
No		No	No		
No		No	No		
Yes	Not satisfie	No	Yes	No	No
No		No	No		
No		No	Yes	No	No
Yes	Satisfied	No	No		
No		No	No		
No		No	No		
No		No	No		
No		No	No		
Yes	Satisfied	No	No		
Yes	Satisfied	No	No		
No		No	No		
Yes	Satisfied	No	No		
No		No	No		
Yes	Not satisfie	No	No		
No		No	No		
No		No	No		
No		No	No		
No		No	No		
No		No	No		
No		No	No		
Yes	Satisfied	No	No		
Yes	Satisfied	No	No		
No		No	No		
No		No	No		
Yes	Satisfied	No	Yes	No	No
No		No	No		
Yes	Satisfied	No	No		
Yes	Satisfied	No	No		
Yes	Satisfied	No	No		
No		No	No		
Yes	Satisfied	No	Yes	No	No
Yes	Satisfied	Yes	No		
Yes	Satisfied	No	Yes	No	No
No		No	No		
No		No	No		
No		No	No		
Yes	Satisfied	Donâ€™t k	No		
Yes	Satisfied	No	No		
Yes	Satisfied	Yes	Yes	No	No
Yes	Satisfied	Yes	No		
No		No	No		
No		No	No		
Yes	Satisfied	Yes	No		
Yes	Satisfied	Yes	No		
Yes	Satisfied	No	No		
Yes	Very satisfi	No	No		
No		Donâ€™t k	No		

For peer review only

No		No	No		
Yes	Satisfied	No	No		
No		No	No		
No		No	No		
No		No	No		
Yes	Satisfied	No	No		
Yes	Satisfied	Yes	Yes	No	No
Yes	Satisfied	Yes	Yes	No	Yes
No		No	No		
Yes	Satisfied	No	No		
Yes	Satisfied	No	Yes	No	No
No		No	No		
No		No	No		
Yes	Satisfied	No	No		
No		No	No		
No		No	No		
Yes	Satisfied	No	No		
No		Yes	No		
No		No	Yes	No	No
Yes	Satisfied	Yes	Yes	No	Yes
Yes	Satisfied	No	No		
Yes	Satisfied	Yes	Yes	No	Yes
No		No	No		
Yes	Satisfied	No	No		
No		No	No		
No		No	No		
No		No	No		
No		No	No		
Yes	Satisfied	No	No		
No		No	No		
No		No	No		
No		No	No		
No		No	No		
Yes	Satisfied	No	No		
Yes	Not satisfie	Yes	No		
No		No	No		
Yes	Satisfied	No	Yes	No	No
Yes	Satisfied	No	No		
No		Yes	No		
No		No	No		
No		No	No		
No		No	No		
No		No	No		
No		No	No		
No		No	No		
Yes	Satisfied	No	No		
No		No	No		
Yes	Satisfied	Yes	Yes	No	Yes
Yes	Satisfied	Yes	Yes	No	Yes
No		No	No		
Yes	Satisfied	Yes	Yes	No	Yes
Yes	Satisfied	No	Yes	No	No

For peer review only

No		No	No			
No		No	No			
No		No	No			
No		No	No			
Yes	Satisfied	No	No			
No		No	Yes	Yes	1 to 30	
No		No	Yes	No		No
No		No	No			
Yes	Satisfied	Yes	No			
No		No	No			
No		No	No			
Yes	Satisfied	Yes	Yes	No		Yes
No		No	No			
No		No	No			
Yes	Satisfied	No	Yes	No		No
No		No	No			
No		No	No			
Yes	Satisfied	Yes	No			
Yes	Satisfied	Yes	No			
Yes	Satisfied	Yes	Yes	No		Yes
Yes	Satisfied	No	No			
Yes	Satisfied	Yes	Yes	No		Yes
No		No	No			
No		No	No			
No		Yes	No			
Yes	Satisfied	No	No			
No		No	Yes	No		No
Yes	Satisfied	Yes	Yes	No		Yes
Yes	Satisfied	Yes	No			
Yes	Satisfied	No	No			
No		No	No			
Yes	Satisfied	Yes	No			
Yes	Satisfied	No	No			
Yes	Satisfied	No	No			
Yes	Satisfied	Yes	No			
No		No	Yes	No		No
No		No	No			
Yes	Satisfied	Yes	No			
Yes	Satisfied	No	No			
Yes	Satisfied	No	No			
No		No	No			
No		No	Yes	No		No
No		No	No			
No		No	No			
No		No	No			
Yes	Satisfied	No	No			
No		No	No			
Yes	Satisfied	No	No			
Yes	Satisfied	No	No			
Yes	Satisfied	Yes	No			
Yes	Satisfied	No	No			
Yes	Satisfied	No	No			

For peer review only

No		No	No					
Yes	Satisfied	No	No					
Yes	Satisfied	No	Yes	No				No
No		No	No					
Yes	Satisfied	Yes	Yes	No				No
Yes	Satisfied	No	No					
Yes	Satisfied	No	No					
Yes	Satisfied	Yes	No					
Yes	Satisfied	Yes	No					
Yes	Satisfied	Yes	No					
No		No	No					
Yes	Satisfied	No	Yes	No				No
Yes	Satisfied	No	No					
No		No	No					
Yes	Satisfied	Yes	Yes	Yes	1 to 5			
Yes	Satisfied	No	No					
No		No	No					
Yes	Satisfied	No	Yes	No				No
Yes	Satisfied	Yes	No					
No		Yes	No					
No		No	Yes	Yes	1 to 5			
Yes	Satisfied	No	No					
No		No	No					
Yes	Satisfied	Yes	Yes	No				No
Yes	Satisfied	No	No					
No		No	No					
Yes	Satisfied	No	No					
No		No	No					
Yes	Satisfied	No	No					
Yes	Satisfied	Yes	No					
No		No	No					
Yes	Satisfied	No	No					
Yes	Satisfied	Yes	No					
No		No	No					
No		No	No					
Yes	Satisfied	No	Yes	No				No
Yes	Satisfied	Yes	No					
No		No	No					
Yes	Satisfied	No	No					
No		Yes	No					
Yes	Satisfied	No	No					
No		No	No					
Yes	Satisfied	No	No					
No		No	No					
No		No	No					
Yes	Satisfied	No	No					
No		No	No					
Yes	Satisfied	No	No					
No		No	No					
No		No	No					
Yes	Satisfied	No	No					
Yes	Satisfied	No	No					

For peer review only

Yes	Satisfied	No	No					
No		Yes	No					
Yes	Satisfied	No	No					
Yes	Satisfied	No	No					
Yes	Satisfied	Yes	No					
No		No	No					
No		No	No					
Yes	Satisfied	No	Yes	No				No
Yes	Satisfied	No	No					
Yes	Satisfied	No	No					
Yes	Satisfied	No	No					
No		No	No					
Yes	Satisfied	No	No					
Yes	Satisfied	Yes	No					
No		No	No					
Yes	Satisfied	No	No					
Yes	Satisfied	No	No					
No		No	No					
No		No	No					
Yes	Satisfied	No	No					
Yes	Satisfied	Yes	No					
Yes	Satisfied	No	No					
No		No	No					
Yes	Satisfied	No	No					
No		No	No					
No		Yes	No					
No		No	No					
Yes	Satisfied	No	Yes	Yes	1 to 30			
No		No	No					
Yes	Satisfied	Yes	Yes	No				No
No		No	No					
Yes	Satisfied	Yes	No					
No		No	No					
No		No	No					
Yes	Satisfied	Yes	No					
Yes	Satisfied	No	Yes	No				No
No		No	No					
Yes	Satisfied	No	No					
No		No	No					
Yes	Satisfied	Yes	Yes	Yes	1 to 30			
Yes	Satisfied	No	Yes	No				No
No		No	No					
Yes	Satisfied	No	No					
No		No	No					
Yes	Not satisfie	Yes	Yes	No				No
Yes	Satisfied	No	No					
Yes	Not satisfie	No	No					
Yes	Satisfied	No	No					
No		No	No					
Yes	Satisfied	Yes	No					
No		No	No					
No		No	No					

For peer review only

No		No	No		
Yes	Very satisfi	No	No		
Yes	Very satisfi	No	No		
Yes	Satisfied	No	No		
Yes	Very satisfi	Yes	Yes	No	No
No		No	No		
No		No	No		
No		No	No		
Yes	Satisfied	No	No		
Yes	Satisfied	No	No		
Yes	Satisfied	No	No		
No		Yes	No		
Yes	Satisfied	No	No		
No		No	No		
Yes	Not satisfie	No	No		
Yes	Not satisfie	No	Yes	No	No
Yes	Satisfied	No	No		
No		No	No		
Yes	Satisfied	Yes	No		
No		No	No		
No		No	No		
No		No	No		
No		No	No		
Yes	Satisfied	Yes	No		
Yes	Satisfied	No	No		
No		No	No		
No		Yes	No		
Yes	Satisfied	No	No		
Yes	Satisfied	No	No		
Yes	Satisfied	No	No		
Yes	Satisfied	No	No		
Yes	Satisfied	Yes	No		
No		No	No		
No		No	No		
No		No	No		
No		No	No		
No		No	Yes	No	No
Yes	Not satisfie	Yes	No		
Yes	Satisfied	No	No		
No		No	No		
No		No	No		
Yes	Satisfied	No	Yes	No	No
Yes	Not satisfie	Yes	Yes	No	No
No		No	No		
No		No	No		
No		No	No		
No		No	No		
Yes	Satisfied	No	No		
Yes	Satisfied	No	No		
No		No	No		
No		No	No		
No		No	Yes	No	No

For peer review only

Yes	Very satisfi	No	No			
Yes	Satisfied	No	No			
Yes	Very satisfi	No	No			
Yes	Satisfied	No	Yes	No		No
Yes	Very satisfi	No	Yes	No		Yes
Yes	Satisfied	No	No			
Yes	Satisfied	No	No			
Yes	Not satisfie	No	No			
No		No	No			
No		No	No			
Yes	Satisfied	Yes	Yes	No		No
No		No	No			
Yes	Satisfied	No	No			
No		No	No			
No		No	No			
No		No	No			
No		No	No			
No		No	No			
Yes	Satisfied	Yes	No			
Yes	Very satisfi	Yes	Yes	Yes	1 to 30	
No		No	Yes	No		No
Yes	Satisfied	No	No			
No		No	No			
No		No	No			
Yes	Satisfied	No	Yes	No		No
No		No	No			
No		No	No			
Yes	Satisfied	No	Yes	No		Yes
No		No	No			
Yes	Satisfied	No	No			
Yes	Satisfied	No	No			
Yes	Satisfied	No	Yes	No		No
Yes	Satisfied	Yes	Yes	No		No
No		Yes	Yes	No		No
No		Yes	No			
No		No	No			
Yes	Satisfied	Yes	Yes	No		No
Yes	Satisfied	No	No			
Yes	Satisfied	Yes	No			
No		No	No			
Yes	Satisfied	No	No			
Yes	Satisfied	No	No			
Yes	Satisfied	No	No			
Yes	Satisfied	No	Yes	No		No
Yes	Satisfied	No	No			
Yes	Satisfied	No	No			
Yes	Satisfied	Yes	Yes	No		No
No		No	No			
Yes	Satisfied	No	Yes	No		No
No		No	No			
No		No	No			
No		No	Yes	No		No

For peer review only

No		No	No					
No		No	No					
Yes	Satisfied	No	No					
No		No	No					
No		No	No					
No		No	No					
Yes	Satisfied	No	No					
No		No	No					
Yes	Satisfied	No	No					
No		No	No					
No		No	Yes	No				No
Yes	Satisfied	No	No					
Yes	Satisfied	No	No					
Yes	Satisfied	No	No					
Yes	Satisfied	No	No					
Yes	Satisfied	No	No					
Yes	Satisfied	No	No					
Yes	Satisfied	No	No					
Yes	Satisfied	No	No					
Yes	Satisfied	No	No					
No		No	Yes	No				No
Yes	Satisfied	No	No					
No		No	No					
Yes	Satisfied	Yes	No					
Yes	Satisfied	No	Yes	No				No
Yes	Satisfied	No	No					
Yes	Not satisfie	No	No					
No		No	Yes	No				No
Yes	Satisfied	No	No					
Yes	Satisfied	Yes	Yes	No				No
Yes	Satisfied	No	No					
No		No	Yes	No				No
Yes	Satisfied	No	No					
Yes	Satisfied	No	No					
Yes	Satisfied	Yes	No					
Yes	Satisfied	No	No					
No		Yes	No					
Yes	Satisfied	Yes	No					
No		No	No					
Yes	Satisfied	No	No					
Yes	Satisfied	Yes	No					
Yes	Satisfied	No	Yes	No				No
Yes	Satisfied	No	No					
Yes	Satisfied	No	No					
Yes	Satisfied	No	No					
Yes	Satisfied	No	No					
No		No	No					
No		No	Yes	No				No
No		No	No					
Yes	Satisfied	No	No					
Yes	Satisfied	Yes	Yes	Yes	1 to 30			
Yes	Satisfied	No	No					

For peer review only

No		No	No			
Yes	Satisfied	No	Yes	Yes	1 to 30	
No		No	No			
No		No	No			
No		No	Yes	No		No
Yes	Satisfied	Yes	Yes	No		No
No		No	No			
No		No	No			
Yes	Satisfied	No	No			
No		No	No			
No		No	No			
No		No	No			
No		No	No			
Yes	Satisfied	No	No			
Yes	Satisfied	No	Yes	No		No
Yes	Satisfied	No	Yes	No		No
No		No	Yes	No		No
No		No	No			
No		No	No			
Yes	Satisfied	No	No			
No		No	No			
No		No	Yes	No		No
Yes	Satisfied	No	Yes	No		No
Yes	Not satisfie	Yes	No			
No		No	No			
Yes	Satisfied	No	No			
Yes	Satisfied	No	No			
No		Yes	No			
No		No	Yes	No		No
No		No	Yes	No		No
Yes	Satisfied	No	No			
Yes	Satisfied	Yes	No			
Yes	Satisfied	No	Yes	No		No
Yes	Satisfied	No	No			
Yes	Satisfied	Yes	Yes	No		No
No		No	No			
Yes	Satisfied	No	Yes	No		No
No		No	No			
Yes	Satisfied	Yes	Yes	No		No
No		No	No			
Yes	Satisfied	No	No			
Yes	Satisfied	No	Yes	No		No
Yes	Satisfied	No	No			
Yes	Satisfied	No	Yes	No		No
Yes	Satisfied	No	No			
Yes	Satisfied	No	No			
No		No	No			
Yes	Satisfied	No	No			
Yes	Satisfied	No	No			
Yes	Satisfied	Yes	No			
No		No	Yes	No		No
No		No	No			

For peer review only

For peer review only - http://bmjopen.bmj.com/site/about/guidelines.xhtml

Yes	Satisfied	No	No			
Yes	Satisfied	No	No			
No		No	No			
Yes	Satisfied	No	Yes	No		No
No		No	No			
Yes	Satisfied	No	Yes	No		No
No		No	No			
Yes	Satisfied	Donâ€™t k	No			
Yes	Satisfied	No	No			
Yes	Satisfied	No	No			
Yes	Satisfied	No	No			
No		No	No			
No		Yes	No			
Yes	Satisfied	No	Yes	Yes	1 to 5	
Yes	Satisfied	No	Yes	No		No
Yes	Satisfied	No	No			
Yes	Satisfied	Yes	Yes	No		No
Yes	Satisfied	No	Yes	No		No
Yes	Satisfied	No	No			
Yes	Satisfied	No	Yes	No		No
Yes	Satisfied	Yes	No			
No		No	No			
No		No	Yes	No		No
No		No	No			
Yes	Satisfied	No	No			
Yes	Satisfied	No	No			
No		No	No			
Yes	Satisfied	No	Yes	No		No
No		No	Yes	No		No
Yes	Satisfied	No	No			
No		No	No			
Yes	Very satisfi	Yes	Yes	No		No
No		No	No			
Yes	Satisfied	No	No			
Yes	Satisfied	No	No			
Yes	Satisfied	Yes	No			
Yes	Satisfied	No	No			
Yes	Satisfied	No	No			
Yes	Satisfied	No	No			
No		No	Yes	No		No
No		No	No			
No		No	No			
No		No	Yes	No		No
Yes	Satisfied	No	No			
No		No	Yes	No		No
Yes	Satisfied	No	No			
Yes	Satisfied	No	Yes	No		No
Yes	Satisfied	No	No			
No		No	No			
Yes	Satisfied	No	No			
No		No	No			
No		No	Yes	No		No

For peer review only

Yes	Satisfied	No	No		
Yes	Satisfied	No	No		
No		No	No		
Yes	Satisfied	No	No		
Yes	Satisfied	No	No		
Yes	Satisfied	No	No		
Yes	Satisfied	No	No		
No		No	Yes	No	No
Yes	Satisfied	Yes	No		
No		No	No		
Yes	Not satisfie	No	No		
No		No	Yes	No	No
Yes	Satisfied	No	No		
No		No	Yes	No	No
No		No	No		
Yes	Satisfied	No	No		
Yes	Satisfied	No	No		
No		No	No		
No		No	No		
No		No	No		
No		No	No		
No		No	Yes	No	No
No		No	No		
No		No	Yes	No	No
No		No	No		
No		No	No		
Yes	Satisfied	No	No		
Yes	Satisfied	No	No		
No		No	No		
No		No	Yes	No	No
No		No	Yes	No	No
Yes	Satisfied	No	No		
Yes	Satisfied	No	No		
No		No	No		
Yes	Satisfied	No	No		
No		No	No		
Yes	Satisfied	Yes	Yes	No	Yes
No		No	Yes	No	No
Yes	Satisfied	No	No		
Yes	Satisfied	No	No		
Yes	Satisfied	No	No		
No		No	No		
No		No	Yes	No	No
Yes	Satisfied	No	No		
No		No	No		
Yes	Satisfied	No	Yes	No	No
Yes	Satisfied	No	No		
Yes	Satisfied	No	No		
No		No	No		
Yes	Satisfied	No	No		
No		No	No		
No		No	No		

For peer review only

For peer review only - http://bmjopen.bmj.com/site/about/guidelines.xhtml

No		No	No		
Yes	Satisfied	No	No		
Yes	Satisfied	No	No		
Yes	Satisfied	No	No		
No		No	No		
No		No	No		
Yes	Satisfied	No	No		
No		No	No		
No		No	Yes	No	No
No		No	No		
No		No	No		
No		Yes	Yes	No	No
No		No	No		
No		No	Yes	No	No
Yes	Satisfied	No	Yes	No	No
No		No	No		
No		No	Yes	No	No
Yes	Satisfied	No	No		
No		No	Yes	No	No
Yes	Satisfied	No	No		
Yes	Satisfied	Yes	No		
No		No	Yes	No	No
No		No	Yes	No	No
No		No	No		
Yes	Satisfied	No	No		
No		No	No		
No		No	No		
Yes	Satisfied	No	No		
Yes	Satisfied	No	No		
Yes	Satisfied	No	No		
No		No	No		
Yes	Satisfied	Yes	No		
No		No	No		
No		No	No		
Yes	Satisfied	No	No		
Yes	Satisfied	No	No		
Yes	Satisfied	No	No		
Yes	Satisfied	No	No		
Yes	Satisfied	No	No		
No		No	No		
No		No	Yes	No	No
Yes	Satisfied	No	No		
No		No	No		
Yes	Satisfied	No	No		
Yes	Satisfied	No	No		
Yes	Satisfied	No	No		
Yes	Satisfied	No	No		
No		No	No		
Yes	Satisfied	No	No		
Yes	Satisfied	No	Yes	No	No
No		No	No		
No		No	No		

For peer review only

No		No	No			
No		No	No			
No		No	No			
No		No	No			
No		No	No			
No		No	No			
Yes	Satisfied	Yes	Yes	No		Yes
No		No	No			
No		No	No			
No		No	No			
No		No	No			
Yes	Satisfied	Donâ€™t k	No			
No		No	No			
Yes	Satisfied	No	No			
Yes	Satisfied	No	No			
No		No	No			
No		No	No			
No		No	No			
Yes	Satisfied	No	No			
No		No	No			
Yes	Satisfied	No	Yes	No		Yes
No		No	No			
Yes	Satisfied	No	No			
Yes	Satisfied	No	Yes	No		No
No		No	No			
Yes	Satisfied	Yes	No			
Yes	Satisfied	No	No			
Yes	Satisfied	Yes	Yes	No		No
No		No	No			
No		No	No			
Yes	Satisfied	Yes	No			
Yes	Satisfied	No	Yes	No		No
No		No	No			
No		No	No			
No		No	No			
Yes	Satisfied	No	No			
Yes	Satisfied	No	No			
No		No	No			
No		No	No			
No		No	No			
Yes	Satisfied	No	No			
Yes	Satisfied	No	Yes	No		No
No		No	No			
No		No	No			
No		No	No			
No		No	No			
No		No	Yes	Yes	1 to 5	
Yes	Satisfied	Yes	No			
Yes	Satisfied	No	No			
Yes	Satisfied	No	No			
No		No	No			
Yes	Satisfied	No	No			

For peer review only

No		No	No		
Yes	Satisfied	No	No		
Yes	Satisfied	No	No		
No		No	No		
Yes	Satisfied	No	No		
No		No	No		
Yes	Satisfied	Yes	Yes	No	No
No		No	No		
No		No	No		
Yes	Satisfied	No	Yes	No	No
No		No	No		
No		No	No		
Yes	Satisfied	No	Yes	No	No
Yes	Satisfied	Yes	No		
Yes	Satisfied	No	No		
No		No	No		
Yes	Satisfied	No	No		
Yes	Satisfied	No	No		
No		No	No		
Yes	Satisfied	No	Yes	No	No
No		No	No		
No		No	No		
Yes	Satisfied	No	No		
Yes	Satisfied	Yes	No		
No		No	No		
Yes	Satisfied	No	Yes	No	No
Yes	Satisfied	Yes	Yes	No	No
Yes	Satisfied	Yes	No		
No		No	No		
Yes	Satisfied	No	No		
Yes	Satisfied	No	Yes	No	No
Yes	Satisfied	No	Yes	No	No
Yes	Satisfied	No	Yes	No	No
Yes	Satisfied	No	Yes	No	No
No		No	No		
Yes	Satisfied	No	Yes	No	No
No		No	No		
Yes	Satisfied	No	No		
Yes	Satisfied	No	Yes	No	No
No		No	No		
No		No	No		
No		No	No		
Yes	Satisfied	No	No		
No		No	No		
No		No	No		
Yes	Satisfied	No	No		
No		No	No		
No		No	No		
No		No	No		
Yes	Satisfied	No	No		
No		Donâ€™t k	No		
Yes	Satisfied	No	No		

For peer review only

No		No	No			
No		No	No			
No		No	No			
No		No	No			
No		No	No			
Yes	Very satisfi	No	No			
No		No	No			
No		No	No			
No		No	No			
No		No	No			
No		No	No			
Yes	Satisfied	Yes	No			
No		No	No			
No		No	No			
Yes	Not satisfie	No	No			
No		No	Yes	No		No
Yes	Not satisfie	No	No			
Yes	Satisfied	Yes	No			
No		No	No			
No		No	No			
Yes	Not satisfie	No	No			
Yes	Satisfied	Yes	Yes	No		No
Yes	Satisfied	Yes	No			
Yes	Very satisfi	No	No			
Yes	Very satisfi	No	No			
Yes	Satisfied	Yes	Yes	No		No
No		No	No			
Yes	Satisfied	Yes	No			
No		No	Yes	Yes	1 to 5	
Yes	Very satisfi	Yes	Yes	Yes	1 to 30	
No		No	No			
Yes	Satisfied	Yes	No			
Yes	Satisfied	Yes	No			
No		Yes	Yes	No		No
No		No	No			
No		No	No			
Yes	Satisfied	Yes	No			
No		Yes	Yes	No		No
Yes	Very satisfi	No	No			
Yes	Not satisfie	Yes	No			
No		No	Yes	No		No
Yes	Satisfied	Yes	No			
No		No	No			
No		No	Yes	No		No
No		No	No			
Yes	Satisfied	No	Yes	No		No
No		No	No			
No		Yes	Yes	No		No
Yes	Satisfied	No	No			
No		No	Yes	No		No
Yes	Satisfied	No	No			
Yes	Satisfied	No	No			

For peer review only

Yes	Satisfied	Yes	No			
No		No	No			
Yes	Satisfied	Yes	No			
Yes	Satisfied	Yes	No			
No		No	No			
Yes	Satisfied	No	No			
No		No	No			
Yes	Satisfied	Yes	Yes	No		No
No		Yes	No			
Yes	Satisfied	Yes	No			
No		Yes	No			
Yes	Not satisfie	Yes	Yes	No		No
Yes	Satisfied	No	Yes	No		No
No		Yes	Yes	No		No
No		No	Yes	No		No
Yes	Satisfied	Yes	Yes	No		No
Yes	Very satisfi	No	No			
No		Yes	Yes	No		Yes
No		No	Yes	No		No
No		No	Yes	No		No
No		No	No			
Yes	Satisfied	No	No			
No		No	No			
No		No	No			
Yes	Not satisfie	Yes	Yes	No		No
No		No	No			
No		No	Yes	No		No
No		Yes	No			
No		No	No			
Yes	Satisfied	Yes	Yes	Yes	1 to 30	
Yes	Satisfied	No	No			
Yes	Satisfied	No	No			
No		No	No			
No		No	No			
Yes	Very satisfi	No	No			
Yes	Satisfied	Yes	No			
Yes	Satisfied	No	No			
No		No	Yes	No		Yes
No		No	Yes	No		No
Yes	Not satisfie	Yes	No			
No		Yes	No			
No		No	No			
Donâ€™t know		Donâ€™t k	No			
Yes	Satisfied	No	No			
No		No	No			
No		Yes	Yes	No		No
No		No	No			
No		No	No			
Yes	Not satisfie	Yes	No			
Yes	Very satisfi	No	No			
Yes	Not satisfie	Yes	Yes	No		No
No		Yes	No			

For peer review only

Yes	Satisfied	No	No			
No		No	No			
Yes	Satisfied	No	No			
No		No	No			
No		No	No			
Yes	Satisfied	No	No			
Yes	Satisfied	Yes	No			
No		No	Yes	No		No
Yes	Satisfied	Yes	Yes	No		No
Yes	Satisfied	No	No			
No		No	No			
No		No	No			
Yes	Satisfied	No	Yes	No		No
No		No	No			
No		No	No			
No		Yes	No			
Yes	Not satisfie	No	No			
Yes	Satisfied	Yes	Yes	No		No
Yes	Satisfied	No	No			
No		No	No			
Yes	Satisfied	No	Yes	No		No
Yes	Not satisfie	Yes	Yes	Yes	1 to 30	
Yes	Satisfied	No	No			
Yes	Satisfied	No	No			
Yes	Satisfied	No	No			
No		No	No			
No		Yes	Yes	No		No
No		No	No			
Yes	Satisfied	No	No			
Yes	Not satisfie	Donâ€™t k	Yes	No		No
Yes	Satisfied	Yes	Yes	No		No
Yes	Satisfied	No	No			
No		No	No			
Yes	Very satisfi	Yes	Yes	No		Yes
Yes	Not satisfie	No	No			
Yes	Not satisfie	Yes	No			
Yes	Very satisfi	No	No			
No		No	No			
Yes	Very satisfi	No	No			
Yes	Satisfied	No	No			
No		Yes	Yes	No		No
No		No	Yes	No		No
No		No	No			
Yes	Satisfied	No	No			
No		No	No			
Yes	Not satisfie	No	No			
No		Yes	No			
Yes	Not satisfie	No	No			
No		No	Yes	No		No
Yes	Very satisfi	Yes	Yes	No		Yes
Yes	Satisfied	No	No			
No		No	No			

For peer review only

For peer review only - http://bmjopen.bmj.com/site/about/guidelines.xhtml

Yes	Satisfied	No	No			
No		No	No			
Yes	Not satisfie	No	No			
No		No	No			
Yes	Very satisfi	Yes	No			
Yes	Satisfied	Yes	Yes	No		No
Yes	Not satisfie	Yes	No			
No		No	No			
No		No	No			
Yes	Satisfied	Yes	No			
No		No	No			
Yes	Satisfied	No	No			
Yes	Not satisfie	No	Yes	No		No
Yes	Not satisfie	No	No			
Yes	Not satisfie	No	No			
Yes	Not satisfie	No	No			
Yes	Satisfied	No	No			
Yes	Satisfied	No	No			
Yes	Satisfied	No	No			
No		No	Yes	No		No
No		No	No			
Yes	Not satisfie	Yes	Yes	No		No
No		No	Yes	No		Yes
No		No	No			
Yes	Not satisfie	Yes	No			
Yes	Satisfied	Yes	Yes	Yes	1 to 30	
Yes	Very satisfi	Yes	No			
No		No	No			
No		No	No			
No		Yes	No			
No		No	Yes	Yes	1 to 5	
Yes	Very satisfi	No	No			
Yes	Very satisfi	No	No			
No		No	Yes	No		No
Yes	Very satisfi	No	No			
Yes	Very satisfi	No	No			
Yes	Satisfied	No	Yes	Yes	1 to 5	
No		No	No			
No		No	No			
No		No	No			
No		No	No			
No		No	Yes	No		No
No		No	No			
No		No	Yes	No		No
No		No	No			
No		No	Yes	Yes	1 to 5	
No		No	No			
Yes	Satisfied	No	No			
No		No	No			
No		No	No			
Yes	Satisfied	Donâ€™t k	No			
Yes	Satisfied	Yes	No			

For peer review only

No		No	No		
No		No	Yes	No	No
Yes	Satisfied	No	No		
No		No	No		
No		No	No		
No		No	No		
No		No	No		
No		No	No		
Yes	Satisfied	No	No		
No		No	No		
Yes	Satisfied	No	No		
No		No	No		
No		No	No		
Yes	Satisfied	No	No		
No		No	No		
No		No	Yes	No	No
No		No	No		
No		No	No		
No		No	Yes	No	No
Yes	Satisfied	No	No		
No		No	No		
No		Yes	No		
No		No	No		
Donâ€™t know		No	No		
No		No	No		
Yes	Satisfied	Yes	No		
Yes	Satisfied	No	No		
No		No	Yes	No	No
Yes	Satisfied	Yes	No		
Yes	Satisfied	No	No		
No		No	No		
No		No	No		
No		No	No		
No		No	No		
Yes	Satisfied	No	No		
No		No	No		
No		No	No		
No		No	Yes	No	No
Yes	Satisfied	No	No		
Yes	Satisfied	No	No		
No		Yes	No		
No		No	Yes	No	No
No		No	No		
No		No	No		
Yes	Satisfied	Yes	No		
No		No	No		
Yes	Satisfied	No	Yes	No	Yes
No		No	No		
No		No	No		
No		Yes	Yes	No	No
No		No	No		
No		No	No		

For peer review only

No		No	No			
No		No	Yes	Yes	1 to 5	
No		No	No			
No		Yes	No			
No		No	No			
No		No	No			
Yes	Satisfied	No	No			
No		No	No			
No		No	No			
No		No	No			
No		No	No			
Yes	Satisfied	No	No			
No		No	No			
Yes	Satisfied	Yes	No			
Yes	Satisfied	Donâ€™t k	No			
No		No	No			
No		No	Yes	No		No
No		Yes	No			
No		No	No			
No		No	No			
No		No	Yes	No		No
No		No	Yes	No		No
No		Yes	Yes	No		Yes
No		No	No			
No		No	No			
No		No	No			
No		No	No			
No		No	No			
No		No	No			
No		No	No			
No		No	No			
No		No	No			
No		No	No			
No		No	No			
No		No	No			
No		No	No			
Yes	Satisfied	No	No			
Yes	Very satisfi	No	No			
No		No	No			
No		No	No			
No		No	No			
No		No	No			
No		No	No			
Yes	Satisfied	Yes	No			
Yes	Satisfied	Yes	No			
No		No	No			
Yes	Satisfied	No	No			
No		No	No			
No		No	Yes	No		No
No		No	No			
No		No	No			
No		No	No			

For peer review only

No		No	No			
No		No	No			
Yes	Satisfied	No	No			
No		No	No			
No		No	Yes	No		No
No		No	No			
No		No	No			
No		No	No			
No		No	No			
No		No	Yes	No		No
No		No	No			
No		No	No			
Yes	Satisfied	No	No			
No		No	No			
Yes	Satisfied	No	Yes	No		Yes
No		No	Yes	No		No
No		No	No			
No		No	No			
No		No	No			
No		No	No			
No		No	No			
No		No	No			
No		No	No			
No		No	No			
No		No	No			
No		No	No			
Yes	Very satisfi	No	Yes	Yes	1 to 5	
No		No	Yes	No		No
No		No	No			
No		Yes	No			
No		No	No			
No		No	No			
No		No	No			
Yes	Satisfied	No	No			
No		No	No			
No		No	No			
Yes	Satisfied	No	Yes	No		No
No		No	No			
No		No	Yes	No		No
No		No	No			
Yes	Satisfied	No	No			
No		No	Yes	No		No
No		No	No			
No		Donâ€™t k	No			
No		No	Yes	No		No
Yes	Satisfied	No	No			
No		No	No			
No		No	Yes	No		No
Yes	Not satisfie	No	Yes	No		No
No		No	No			
No		No	No			
No		No	No			

For peer review only

No		No	No			
No		No	No			
No		No	No			
No		No	No			
No		No	Yes	No		No
No		No	Yes	No		Yes
Yes	Satisfied	Yes	Yes	No		Yes
No		No	No			
Yes	Satisfied	No	No			
No		No	No			
No		No	Yes	Yes	1 to 30	
No		No	No			
No		No	No			
No		No	No			
No		No	No			
No		No	Yes	No		No
No		No	No			
Yes	Satisfied	Yes	No			
No		No	No			
No		No	No			
No		No	No			
No		No	No			
No		No	No			
Yes	Satisfied	No	No			
No		No	No			
Yes	Satisfied	No	Yes	No		No
No		No	No			
No		No	No			
No		No	No			
No		No	No			
No		No	No			
No		No	No			
Yes	Satisfied	Yes	No			
Yes	Satisfied	No	No			
Yes	Very satisfi	No	No			
Yes	Not satisfie	Yes	No			
Yes	Satisfied	Yes	No			
Yes	Not satisfie	Yes	No			
Yes	Satisfied	No	No			
Yes	Satisfied	No	No			
Yes	Satisfied	Yes	No			
Yes	Satisfied	Yes	No			
No		No	No			
No		Yes	No			
Yes	Not satisfie	No	No			
Yes	Not satisfie	No	No			
Yes	Satisfied	Yes	Yes	No		Yes
Yes	Satisfied	Yes	Yes	No		No
Yes	Not satisfie	No	No			
Yes	Satisfied	No	No			
Yes	Not satisfie	No	No			
Yes	Not satisfie	Yes	Yes	No		Yes
Yes	Very satisfi	Yes	No			

For peer review only

Yes	Very satisfi	No	Yes	Yes	1 to 30	
Yes	Not satisfie	No	No			
Yes	Satisfied	Yes	No			
No		Yes	No			
Yes	Satisfied	Yes	No			
Yes	Satisfied	No	No			
Yes	Satisfied	No	No			
Yes	Satisfied	No	Yes	No		No
Yes	Satisfied	No	No			
Yes	Not satisfie	Yes	No			
Yes	Not satisfie	Yes	No			
Yes	Not satisfie	Yes	Yes	No		No
Yes	Satisfied	No	No			
Yes	Not satisfie	No	Yes	No		No
Yes	Not satisfie	No	No			
No		Yes	No			
No		No	No			
Yes	Satisfied	Yes	No			
Yes	Not satisfie	No	No			
Yes	Satisfied	Yes	No			
Yes	Very satisfi	No	Yes	No		Yes
No		No	No			
Yes	Satisfied	No	No			
No		Yes	No			
Yes	Not satisfie	No	No			
Yes	Not satisfie	Yes	Yes	No		Yes
Yes	Not satisfie	Yes	No			
Yes	Not satisfie	No	No			
Yes	Not satisfie	No	No			
Yes	Satisfied	No	No			
Yes	Satisfied	No	Yes	No		Yes
Yes	Not satisfie	Yes	No			
No		No	No			
Yes	Not satisfie	No	No			
Yes	Satisfied	No	No			
No		No	No			
Yes	Very satisfi	Yes	Yes	No		No
Yes	Not satisfie	No	No			
Yes	Satisfied	Yes	No			
Yes	Not satisfie	No	No			
Yes	Not satisfie	Yes	No			
Yes	Not satisfie	Yes	No			
Yes	Satisfied	Yes	No			
Yes	Satisfied	Yes	No			
No		No	No			
Yes	Satisfied	No	No			
Yes	Satisfied	Yes	No			
Yes	Very satisfi	No	No			
Yes	Not satisfie	No	No			
Yes	Satisfied	Yes	No			
No		No	No			
No		No	No			

For peer review only

Yes	Not satisfie	No	Yes	No		No
No		No	No			
Yes	Satisfied	Yes	Yes	Yes	1 to 30	
Yes	Satisfied	No	No			
Yes	Satisfied	Yes	No			
Yes	Not satisfie	No	No			
Yes	Very satisfi	No	No			
No		Yes	No			
Yes	Satisfied	No	Yes	No		No
Yes	Not satisfie	Yes	No			
Yes	Not satisfie	No	Yes	No		No
Donâ€™t know		No	Yes	No		No
Yes	Not satisfie	Yes	No			
Yes	Not satisfie	Yes	No			
No		No	No			
Yes	Not satisfie	Yes	No			
Yes	Satisfied	Yes	No			
Yes	Not satisfie	No	Yes	No		Yes
Yes	Satisfied	No	No			
Yes	Satisfied	Yes	No			
No		No	Yes	No		Yes
No		No	No			
No		No	No			
No		Yes	Yes	No		Yes
No		No	Yes	No		No
No		No	Yes	No		No
Yes	Satisfied	No	Yes	No		No
No		No	Yes	No		Yes
Yes	Satisfied	No	No			
Yes	Satisfied	Yes	Yes	No		Yes
No		No	Yes	No		No
No		No	Yes	No		No
No		No	Yes	No		No
Yes	Very satisfi	Yes	Yes	No		No
Yes	Satisfied	Yes	Yes	No		Yes
No		No	No			
No		No	No			
No		No	Yes	No		No
No		Yes	Yes	No		No
No		No	No			
No		No	No			
No		Yes	No			
Yes	Not satisfie	No	No			
Yes	Satisfied	No	No			
Yes	Satisfied	Yes	Yes	No		Yes
No		No	No			
No		Yes	Yes	No		Yes
No		No	No			
Yes	Satisfied	No	Yes	No		No
Yes	Satisfied	Yes	Yes	No		Yes
No		No	No			
Yes	Satisfied	Yes	Yes	No		Yes

For peer review only

Yes	Satisfied	Yes	Yes	No	No
No		No	No		
No		No	No		
No		No	Yes	No	No
No		No	No		
Yes	Very satisfi	No	Yes	No	No
Yes	Satisfied	No	No		
No		No	No		
Yes	Satisfied	No	No		
Yes	Satisfied	Yes	No		
Yes	Very satisfi	Yes	Yes	No	Yes
No		No	No		
Yes	Satisfied	Yes	Yes	No	No
No		Donâ€™t k	No		
No		Yes	Yes	No	No
Yes	Satisfied	Yes	Yes	No	Yes
No		No	No		
No		No	Yes	No	No
Yes	Satisfied	No	No		
Yes	Satisfied	No	No		
No		No	No		
No		No	No		
No		No	Yes	No	No
Yes	Satisfied	No	No		
No		No	Yes	No	No
No		No	Yes	No	No
Yes	Satisfied	No	No		
Yes	Satisfied	No	Yes	No	No
No		No	Yes	No	No
No		No	Yes	No	Yes
Yes	Not satisfie	No	No		
No		No	No		
No		No	Yes	No	No
No		No	No		
No		No	Yes	No	No
Yes	Satisfied	No	No		
No		No	Yes	No	No
Yes	Not satisfie	No	Yes	No	Yes
Yes	Satisfied	No	No		
No		Yes	Yes	No	No
Yes	Satisfied	Yes	Yes	No	Yes
Yes	Satisfied	No	Yes	No	No
No		No	No		
No		No	Yes	No	No
Yes	Satisfied	No	No		
No		No	No		
No		No	No		
No		No	Yes	No	No
No		No	Yes	No	No
No		No	No		
Yes	Not satisfie	Yes	Yes	No	No
Yes	Satisfied	No	No		

For peer review only

Yes	Satisfied	No	Yes	Yes	1 to 30			
No		No	No					
No		No	Yes	No				Yes
Yes	Not satisfie	No	No					
No		No	No					
No		No	No					
No		No	Yes	No				No
Yes	Satisfied	No	No					
No		No	Yes	No				No
No		No	Yes	No				No
No		No	Yes	No				No
No		No	No					
Yes	Satisfied	No	Yes	No				No
Yes	Satisfied	Yes	Yes	No				Yes
No		No	Yes	No				Yes
No		No	Yes	No				No
No		No	Yes	No				No
No		No	No					
Yes	Very satisfi	Yes	No					
Yes	Very satisfi	No	Yes	No				No
Yes	Satisfied	Yes	Yes	No				Yes
Yes	Very satisfi	Yes	Yes	No				Yes
Yes	Very satisfi	Yes	Yes	No				No
Yes	Satisfied	No	No					
Yes	Satisfied	Yes	Yes	No				No
Yes	Satisfied	Yes	Yes	No				No
Yes	Very satisfi	Yes	Yes	No				No
Yes	Satisfied	Yes	Yes	Yes	1 to 30			
Yes	Very satisfi	Yes	No					
Yes	Satisfied	Yes	Yes	Yes	1 to 30			
Yes	Satisfied	No	Yes	Yes	1 to 30			
Yes	Satisfied	No	No					
Yes	Very satisfi	Yes	Yes	No				No
Yes	Satisfied	No	Yes	No				No
Yes	Very satisfi	Yes	No					
Yes	Satisfied	Yes	Yes	No				No
No		Yes	No					
Yes	Satisfied	Yes	No					
Yes	Satisfied	Yes	No					
Yes	Satisfied	Yes	No					
Yes	Satisfied	No	No					
Yes	Very satisfi	No	No					
Yes	Very satisfi	Yes	Yes	No				No
Yes	Satisfied	Yes	No					
Yes	Satisfied	No	No					
Yes	Very satisfi	Yes	No					
Yes	Satisfied	No	No					
Yes	Very satisfi	Yes	No					
Yes	Satisfied	No	No					
Yes	Satisfied	Yes	Yes	Yes	1 to 5			
Yes	Very satisfi	Yes	Yes	No				No
Yes	Satisfied	Yes	Yes	No				No

For peer review only

Yes	Satisfied	No	No			
Yes	Satisfied	Yes	Yes	Yes	1 to 30	
Yes	Satisfied	Yes	No			
Yes	Satisfied	Yes	Yes	No		Yes
Yes	Satisfied	No	No			
Yes	Satisfied	No	No			
Yes	Satisfied	Yes	No			
No		No	No			
Yes	Satisfied	Yes	No			
Yes	Very satisfi	No	No			
Yes	Very satisfi	Yes	No			
No		Yes	Yes	No		No
Yes	Satisfied	No	No			
Yes	Satisfied	No	No			
No		No	No			
No		Yes	Yes	No		Yes
Yes	Satisfied	No	No			
Yes	Satisfied	No	Yes	No		No
No		No	No			
Yes	Very satisfi	No	No			
Yes	Satisfied	No	No			
Yes	Satisfied	Yes	No			
No		Donâ€™t k	No			
No		No	No			
Yes	Not satisfie	Yes	No			
No		No	No			
No		Donâ€™t k	No			
Yes	Satisfied	Yes	Yes	No		No
Yes	Satisfied	No	No			
Yes	Satisfied	No	No			
Yes	Satisfied	Yes	Yes	No		Yes
Yes	Very satisfi	No	No			
Yes	Not satisfie	No	No			
No		No	No			
Yes	Satisfied	Yes	No			
Yes	Satisfied	No	Yes	No		Yes
Yes	Very satisfi	No	No			
Yes	Satisfied	No	No			
Yes	Satisfied	Yes	No			
No		Yes	No			
No		No	No			
Yes	Satisfied	Yes	Yes	No		No
Yes	Satisfied	Yes	Yes	No		Yes
No		No	Yes	No		No
Yes	Satisfied	Yes	No			
No		No	No			
No		No	No			
Yes	Satisfied	No	No			
Yes	Satisfied	Yes	No			
No		No	No			
Yes	Satisfied	No	No			
No		No	No			

For peer review only

No		No	No			
Yes	Satisfied	No	No			
No		No	No			
Yes	Satisfied	No	No			
No		No	No			
Yes	Not satisfie	Yes	No			
Yes	Very satisfi	No	No			
Yes	Satisfied	No	Yes	No		No
Yes	Satisfied	Yes	No			
Yes	Satisfied	Yes	No			
No		Yes	No			
No		No	No			
Yes	Very satisfi	No	No			
Yes	Satisfied	No	No			
Yes	Satisfied	Yes	No			
No		No	No			
Yes	Satisfied	Yes	No			
Yes	Not satisfie	Yes	No			
Yes	Satisfied	Yes	No			
Yes	Satisfied	No	Yes	No		No
No		No	No			
Yes	Satisfied	Yes	Yes	Yes	1 to 30	
Yes	Satisfied	No	Yes	No		No
Yes	Satisfied	Yes	Yes	No		No
Yes	Satisfied	No	Yes	No		No
Yes	Satisfied	Yes	No			
No		Donâ€™t k	No			
No		No	No			
No		No	No			
Yes	Very satisfi	No	No			
Yes	Satisfied	Yes	No			
No		Yes	No			
No		No	No			
No		No	No			
No		No	No			
No		No	No			
No		No	No			
No		No	No			
No		No	No			
No		No	Yes	No		No
No		No	No			
No		No	Yes	No		No
Yes	Satisfied	Yes	Yes	No		No
Yes	Satisfied	No	Yes	No		No
No		No	No			
Yes	Satisfied	No	Yes	No		No
No		No	No			
Yes	Very satisfi	No	Yes	No		No
No		No	No			
No		No	No			
Yes	Very satisfi	Yes	Yes	No		No
No		No	No			

For peer review only

No		No	No			
No		No	No			
No		No	No			
Yes	Very satisfi	Yes	Yes	No		No
No		No	No			
Yes	Satisfied	Yes	Yes	No		No
Yes	Satisfied	No	Yes	Yes	1 to 5	
No		No	No			
No		No	No			
Yes	Very satisfi	Yes	Yes	No		No
No		No	No			
No		No	No			
No		No	No			
Yes	Very satisfi	Yes	Yes	No		No
Yes	Not satisfie	Yes	Yes	No		Yes
No		No	No			
Yes	Very satisfi	Yes	Yes	No		Yes
No		No	Yes	Yes	1 to 30	
No		Donâ€™t k	No			
Yes	Satisfied	Yes	Yes	No		Yes
No		No	No			
No		No	Yes	No		No
No		No	No			
No		No	No			
Yes	Satisfied	Yes	No			
No		No	No			
Yes	Satisfied	Yes	Yes	No		Yes
Yes	Satisfied	Yes	Yes	No		No
Yes	Satisfied	No	Yes	No		No
No		No	No			
Yes	Satisfied	No	Yes	No		No
Yes	Very satisfi	Yes	Yes	No		No
Yes	Satisfied	No	Yes	No		No
No		No	No			
No		No	No			
Yes	Satisfied	No	Yes	No		No
No		No	No			
No		No	No			
Yes	Satisfied	No	Yes	No		No
No		No	No			
No		Yes	No			
Yes	Satisfied	Yes	No			
No		No	No			
No		No	No			
Yes	Very satisfi	Yes	No			
No		No	No			
Yes	Satisfied	No	Yes	No		Yes
Yes	Not satisfie	No	No			
No		No	No			
No		No	No			
No		No	No			
Yes	Not satisfie	No	No			

For peer review only

No		No	No		
No		No	No		
Yes	Satisfied	No	No		
No		No	No		
No		No	No		
No		No	No		
Yes	Satisfied	Yes	No		
Yes	Very satisfi	Yes	Yes	No	No
Yes	Satisfied	Yes	No		
No		Yes	No		
Yes	Satisfied	Yes	No		
No		No	No		
Yes	Satisfied	No	No		
Yes	Satisfied	Yes	No		
Yes	Not satisfie	No	No		
Yes	Satisfied	No	No		
Yes	Satisfied	Yes	No		
Yes	Very satisfi	No	No		
No		No	Yes	No	No
No		No	No		
Yes	Satisfied	Yes	No		
Yes	Satisfied	Yes	No		
No		No	No		
No		No	No		
Yes	Satisfied	Yes	Yes	No	No
No		No	No		
Yes	Satisfied	No	Yes	No	No
No		No	No		
No		No	No		
No		Yes	No		
Yes	Satisfied	No	No		
Yes	Satisfied	Yes	No		
Yes	Satisfied	Yes	No		
No		Yes	No		
Yes	Satisfied	Yes	Yes	No	No
Yes	Satisfied	Yes	No		
Yes	Not satisfie	No	No		
Yes	Very satisfi	Yes	No		
Yes	Not satisfie	Yes	Yes	No	No
Yes	Satisfied	Yes	Yes	No	No
No		No	Yes	No	No
Yes	Satisfied	No	Yes	No	No
Yes	Very satisfi	Yes	Yes	No	No
No		No	No		
No		No	No		
Yes	Not satisfie	No	No		
Yes	Satisfied	Donâ€™t k	No		
Yes	Very satisfi	No	Yes	No	Yes
Yes	Satisfied	Yes	No		
Yes	Not satisfie	No	No		
Yes	Not satisfie	No	No		
No		No	Yes	No	No

For peer review only

No		No	Yes	No		No
Yes	Very satisfi	No	No			
Yes	Satisfied	Yes	No			
Yes	Satisfied	Yes	Yes	No		No
No		No	No			
Yes	Satisfied	No	No			
Yes	Not satisfie	No	No			
No		Yes	No			
Yes	Satisfied	Yes	Yes	No		No
Yes	Very satisfi	Yes	Yes	No		No
No		No	No			
Yes	Satisfied	No	No			
No		No	No			
Yes	Not satisfie	No	No			
No		No	No			
No		No	Yes	No		No
Yes	Satisfied	No	No			
No		Yes	Yes	No		No
No		No	No			
Yes	Not satisfie	Yes	No			
No		No	No			
No		No	No			
No		Yes	No			
No		No	No			
No		No	No			
Yes	Very satisfi	Yes	No			
Yes	Satisfied	Yes	No			
Yes	Very satisfi	Yes	No			
Yes	Satisfied	No	No			
Yes	Satisfied	Yes	No			
Yes	Satisfied	No	No			
Yes	Satisfied	Yes	No			
No		No	No			
Yes	Satisfied	Yes	No			
Yes	Very satisfi	No	Yes	No		No
No		No	No			
No		No	No			
Yes	Very satisfi	No	No			
Yes	Satisfied	Yes	No			
No		No	Yes	No		No
No		No	No			
Yes	Satisfied	No	No			
Yes	Very satisfi	Yes	No			
Yes	Satisfied	Yes	Yes	No		No
Yes	Very satisfi	Yes	No			
Yes	Not satisfie	Yes	Yes	No		No
Yes	Satisfied	No	No			
No		No	No			
No		No	No			
No		No	Yes	No		No
Yes	Satisfied	No	No			
Yes	Satisfied	No	Yes	Yes	1 to 5	

Yes	Satisfied	Yes	No		
No		No	No		
Yes	Satisfied	No	No		
No		Yes	No		
No		No	No		
Yes	Satisfied	No	No		
No		No	No		
Yes	Satisfied	No	No		
Yes	Satisfied	No	Yes	No	No
Yes	Not satisfie	Yes	No		
No		No	No		
Yes	Very satisfi	No	No		
No		No	No		
No		No	No		
Yes	Satisfied	No	No		
Yes	Satisfied	Yes	Yes	No	Yes
Yes	Satisfied	Yes	No		
No		No	No		
No		No	No		
Yes	Satisfied	Yes	No		
Yes	Very satisfi	Yes	No		
No		No	No		
Yes	Satisfied	Yes	No		
Yes	Very satisfi	No	Yes	No	No
Yes	Satisfied	No	Yes	No	No
Yes	Very satisfi	No	No		
Yes	Satisfied	No	No		
Yes	Satisfied	No	No		
Yes	Satisfied	No	No		
No		No	No		
Yes	Very satisfi	No	Yes	No	No
Yes	Very satisfi	Yes	Yes	No	No
No		Yes	No		
Yes	Satisfied	No	No		
No		No	No		
Yes	Very satisfi	No	No		
Yes	Very satisfi	Yes	No		
Yes	Very satisfi	Yes	No		
Yes	Satisfied	Yes	No		
No		Yes	No		
No		No	No		
No		No	No		
No		No	No		
No		No	No		
No		No	No		
No		No	No		
Yes	Satisfied	Yes	No		
No		No	No		
Yes	Not satisfie	Yes	No		
Yes	Not satisfie	No	No		
Yes	Not satisfie	No	No		
No		No	No		

For peer review only

Yes	Satisfied	No	No		
Yes	Very satisfi	Yes	No		
Yes	Satisfied	Yes	Yes	No	No
Yes	Very satisfi	No	Yes	No	Yes
No		No	No		
Yes	Very satisfi	No	No		
Yes	Very satisfi	Yes	No		
Yes	Satisfied	Yes	No		
No		Yes	No		
No		Yes	No		
No		No	No		
Yes	Very satisfi	Yes	No		
No		No	No		
No		No	No		
Yes	Satisfied	No	No		
No		No	No		
No		No	No		
Yes	Not satisfie	Yes	No		
Yes	Satisfied	Yes	Yes	No	No
Yes	Satisfied	Yes	Yes	No	No
No		No	No		
No		No	No		
No		No	No		
Yes	Satisfied	No	No		
Yes	Very satisfi	Yes	No		
Yes	Satisfied	Yes	Yes	No	No
No		No	No		
Yes	Satisfied	Yes	No		
Yes	Satisfied	Yes	No		
Yes	Satisfied	No	Yes	No	No
Yes	Satisfied	Yes	Yes	No	No
Yes	Satisfied	No	No		
Yes	Very satisfi	Yes	No		
Yes	Not satisfie	No	No		
Yes	Satisfied	No	No		
Yes	Satisfied	No	No		
No		No	No		
No		No	No		
Yes	Satisfied	Yes	No		
Yes	Satisfied	No	No		
No		No	No		
Yes	Satisfied	No	No		
Yes	Not satisfie	No	No		
Yes	Satisfied	No	Yes	No	No
No		No	No		
No		No	No		
Yes	Not satisfie	No	No		
Yes	Satisfied	No	No		
Yes	Satisfied	Yes	Yes	No	No
No		No	No		
No		No	No		
Yes	Satisfied	Yes	No		

For peer review only

No		No	No			
No		No	No			
Yes	Satisfied	Yes	Yes	No		No
Yes	Very satisfi	No	No			
No		No	No			
No		No	No			
No		No	No			
No		No	No			
No		No	No			
No		No	No			
Yes	Not satisfie	No	No			
No		No	No			
No		No	No			
Yes	Satisfied	Yes	Yes	No		No
No		No	Yes	No		No
No		No	No			
No		No	No			
Yes	Satisfied	No	Yes	No		No
No		No	No			
No		No	No			
Yes	Satisfied	No	No			
Yes	Not satisfie	No	No			
Yes	Satisfied	Yes	No			
Yes	Satisfied	Yes	Yes	No		Yes
No		No	No			
Yes	Satisfied	No	Yes	No		No
Yes	Satisfied	No	Yes	No		No
No		Yes	No			
Yes	Satisfied	No	Yes	No		No
Yes	Satisfied	No	Yes	No		No
Yes	Satisfied	Yes	Yes	No		No
Yes	Not satisfie	Yes	Yes	No		No
Yes	Satisfied	Yes	No			
No		Yes	No			
Yes	Not satisfie	Yes	No			
No		No	No			
Yes	Not satisfie	Yes	No			
Yes	Not satisfie	No	No			
Yes	Satisfied	No	No			
Yes	Satisfied	Yes	Yes	No		No
No		No	No			
Yes	Not satisfie	No	No			
Yes	Satisfied	Yes	Yes	No		No
No		No	No			
Yes	Satisfied	Yes	Yes	No		Yes
Yes	Satisfied	Yes	No			
Yes	Satisfied	Yes	Yes	Yes	1 to 5	
No		No	No			
No		No	No			
No		No	No			
Yes	Satisfied	No	No			
Yes	Satisfied	No	No			

For peer review only

No		No	No			
No		No	No			
Yes	Satisfied	Yes	Yes	No		No
Yes	Satisfied	Yes	No			
Yes	Satisfied	Yes	Yes	No		No
Yes	Satisfied	Yes	No			
Yes	Not satisfie	No	No			
Yes	Satisfied	No	No			
Yes	Satisfied	No	No			
Yes	Satisfied	Yes	No			
No		Yes	No			
Yes	Very satisfi	Yes	Yes	No		No
Yes	Satisfied	No	No			
Yes	Very satisfi	Yes	Yes	No		No
Yes	Satisfied	No	No			
Yes	Satisfied	No	No			
Yes	Satisfied	No	No			
Yes	Satisfied	No	No			
Yes	Satisfied	No	No			
No		No	No			
No		No	No			
Yes	Very satisfi	No	No			
Yes	Not satisfie	Yes	Yes	Yes	1 to 5	
Yes	Satisfied	No	No			
Yes	Very satisfi	Yes	Yes	No		No
Yes	Satisfied	Yes	No			
Yes	Not satisfie	No	Yes	No		Yes
Yes	Not satisfie	Yes	No			
Yes	Satisfied	No	No			
Yes	Satisfied	No	No			
No		No	Yes	Yes	1 to 5	
No		No	No			
Yes	Satisfied	No	No			
No		No	No			
Yes	Satisfied	Yes	No			
Yes	Satisfied	Yes	No			
Yes	Satisfied	Yes	No			
Yes	Satisfied	No	Yes	No		No
Yes	Satisfied	No	Yes	No		No
Yes	Satisfied	No	No			
Yes	Satisfied	No	No			
Yes	Very satisfi	Yes	No			
Yes	Very satisfi	No	No			
Yes	Very satisfi	Yes	Yes	No		No
Yes	Not satisfie	No	No			
No		Yes	No			
No		No	No			
Yes	Very satisfi	Yes	Yes	No		No
No		No	No			
Yes	Satisfied	No	No			
Yes	Satisfied	No	No			
Yes	Not satisfie	No	No			

For peer review only

No		No	No			
Yes	Satisfied	No	No			
Yes	Satisfied	No	No			
Yes	Very satisfi	No	Yes	No		No
Yes	Not satisfie	No	No			
No		No	No			
No		No	No			
Yes	Satisfied	No	No			
Yes	Very satisfi	Yes	Yes	No		No
Yes	Satisfied	No	Yes	No		No
Yes	Satisfied	Yes	No			
Yes	Satisfied	Yes	Yes	No		No
No		No	No			
No		No	No			
Yes	Satisfied	No	No			
No		No	No			
Yes	Satisfied	No	No			
Yes	Not satisfie	No	No			
Yes	Satisfied	No	No			
No		No	No			
Yes	Very satisfi	No	Yes	No		No
No		No	No			
Yes	Satisfied	No	No			
No		No	Yes	No		Yes
Yes	Satisfied	No	No			
Yes	Not satisfie	Yes	Yes	Yes	1 to 5	
No		No	No			
Yes	Very satisfi	No	No			
No		No	No			
No		No	No			
Yes	Satisfied	Yes	No			
No		No	No			
Yes	Satisfied	Yes	No			
Yes	Very satisfi	Yes	Yes	No		No
Yes	Satisfied	No	No			
No		No	No			
Yes	Very satisfi	Yes	Yes	No		No
Yes	Very satisfi	Yes	No			
Yes	Satisfied	No	No			
Yes	Very satisfi	No	No			
Yes	Satisfied	No	No			
No		No	No			
No		No	No			
No		No	No			
Yes	Satisfied	Yes	Yes	No		No
Yes	Satisfied	No	No			
Yes	Satisfied	Yes	Yes	No		No
Yes	Satisfied	Yes	No			
Yes	Satisfied	No	No			
No		No	No			
Yes	Satisfied	No	No			
No		No	No			

For peer review only

Yes	Satisfied	No	No			
No		Yes	No			
Yes	Satisfied	No	Yes	No		Yes
Yes	Not satisfie	No	No			
Yes	Satisfied	No	Yes	No		No
No		No	No			
Yes	Satisfied	No	No			
Yes	Satisfied	No	No			
Yes	Very satisfi	No	No			
Yes	Very satisfi	Yes	No			
Yes	Satisfied	Yes	No			
Yes	Not satisfie	Yes	No			
Yes	Satisfied	Yes	No			
Yes	Satisfied	No	No			
Yes	Not satisfie	No	No			
No		Yes	No			
Yes	Satisfied	No	No			
Yes	Satisfied	No	No			
Yes	Satisfied	No	No			
Yes	Very satisfi	Yes	No			
Yes	Satisfied	No	Yes	Yes	1 to 5	
Yes	Satisfied	No	Yes	No		No
No		No	No			
Yes	Not satisfie	No	No			
Yes	Not satisfie	No	No			
Yes	Very satisfi	No	No			
Yes	Satisfied	Yes	Yes	Yes	1 to 5	
No		No	No			
Yes	Satisfied	No	No			
Yes	Satisfied	Yes	No			
Yes	Satisfied	No	Yes	No		No
Yes	Satisfied	Yes	No			
Yes	Satisfied	No	No			
Yes	Satisfied	Yes	Yes	Yes	1 to 30	
Yes	Not satisfie	Yes	No			
Yes	Satisfied	No	Yes	No		No
Yes	Very satisfi	Yes	No			
Yes	Satisfied	No	No			
Yes	Satisfied	No	No			
Yes	Satisfied	No	No			
Yes	Satisfied	No	No			
Yes	Satisfied	No	Yes	Yes	1 to 5	
No		No	No			
Yes	Not satisfie	Yes	No			
Yes	Satisfied	No	No			
Yes	Very satisfi	No	No			
No		No	No			
Yes	Not satisfie	No	No			
Yes	Satisfied	Yes	No			
Yes	Very satisfi	No	No			
No		Yes	Yes	No		No
No		No	No			

For peer review only

Yes	Satisfied	No	No					
Yes	Satisfied	Yes	No					
No		No	No					
Yes	Satisfied	No	No					
No		No	No					
Yes	Satisfied	Yes	No					
Donâ€™t know		No	No					
Yes	Satisfied	No	No					
Yes	Satisfied	Yes	No					
Yes	Satisfied	No	No					
No		No	No					
No		No	No					
Yes	Not satisfie	Yes	Yes	Yes	1 to 5			
Yes	Satisfied	Yes	No					
No		No	No					
No		No	No					
Yes	Satisfied	No	No					
No		No	No					
Yes	Satisfied	No	No					
No		No	No					
Yes	Satisfied	No	No					
No		No	No					
No		No	No					
Yes	Satisfied	Yes	No					
Yes	Satisfied	No	No					
No		No	No					
No		No	No					
Yes	Satisfied	Yes	No					
Yes	Not satisfie	Yes	Yes	No				No
Yes	Satisfied	No	No					
Yes	Not satisfie	Yes	No					
No		No	No					
Yes	Very satisfi	Yes	Yes	No				No
No		No	No					
No		No	No					
No		No	No					
Yes	Not satisfie	No	No					
No		No	No					
Yes	Satisfied	Yes	Yes	No				No
No		No	No					
No		No	No					
No		No	No					
Yes	Very satisfi	Yes	Yes	No				Yes
Yes	Satisfied	No	No					
Yes	Satisfied	Yes	No					
Yes	Satisfied	Yes	No					
Yes	Satisfied	Yes	No					
Yes	Very satisfi	No	No					
No		Yes	No					
No		No	No					
Yes	Satisfied	No	No					
Yes	Not satisfie	No	No					

For peer review only

Yes	Satisfied	No	No			
No		No	No			
Yes	Satisfied	Yes	Yes	No		No
Yes	Satisfied	No	No			
Yes	Not satisfie	Yes	No			
No		No	No			
No		No	No			
Yes	Satisfied	Yes	Yes	No		No
Yes	Satisfied	No	No			
Yes	Satisfied	No	Yes	No		No
Yes	Satisfied	Yes	No			
No		No	No			
Yes	Satisfied	No	No			
Yes	Satisfied	Yes	No			
Yes	Very satisfi	No	No			
No		Yes	No			
Yes	Not satisfie	Yes	No			
Yes	Satisfied	No	Yes	No		No
Yes	Not satisfie	Yes	No			
Yes	Satisfied	Yes	No			
No		No	No			
No		No	Yes	Yes	1 to 5	
No		No	No			
No		No	No			
No		No	No			
No		No	No			
No		No	No			
No		No	No			
No		No	No			
Yes	Satisfied	Yes	Yes	No		No
No		No	No			
Yes	Satisfied	No	No			
No		No	No			
Yes	Satisfied	Yes	No			
Yes	Not satisfie	No	No			
Yes	Satisfied	Yes	No			
Yes	Satisfied	No	No			
Yes	Very satisfi	Yes	Yes	No		No
Yes	Satisfied	No	No			
No		No	No			
No		No	No			
No		No	No			
No		No	No			
No		No	No			
Yes	Not satisfie	No	No			
Yes	Satisfied	No	No			
No		No	No			
Yes	Satisfied	No	Yes	No		No
Yes	Satisfied	No	No			
Yes	Satisfied	No	No			
Yes	Satisfied	No	No			
Yes	Very satisfi	Yes	No			

For peer review only

Yes	Not satisfie	Yes	No			
No		Yes	Yes	No		Yes
Yes	Not satisfie	No	No			
Yes	Satisfied	No	No			
Yes	Satisfied	No	Yes	Yes	1 to 5	
No		No	No			
Yes	Satisfied	No	No			
Yes	Satisfied	Yes	No			
Yes	Very satisfi	No	Yes	No		No
No		No	No			
Yes	Satisfied	Yes	Yes	No		No
Yes	Satisfied	Yes	No			
No		No	No			
Yes	Satisfied	No	Yes	No		No
Yes	Satisfied	Yes	No			
Yes	Satisfied	Yes	No			
Yes	Satisfied	No	No			
Yes	Satisfied	No	No			
Yes	Very satisfi	Yes	Yes	No		No
Yes	Very satisfi	Yes	Yes	No		No
Yes	Satisfied	Yes	No			
Yes	Satisfied	No	No			
No		No	No			
Yes	Satisfied	Yes	No			
No		No	No			
Yes	Satisfied	No	No			
Yes	Satisfied	Yes	Yes	No		No
Yes	Satisfied	No	No			
No		No	No			
No		No	No			
Yes	Satisfied	Yes	Yes	No		Yes
Yes	Satisfied	Yes	No			
Yes	Not satisfie	Yes	Yes	No		No
Yes	Very satisfi	Yes	No			
No		No	No			
Yes	Satisfied	No	No			
Yes	Satisfied	No	No			
Yes	Satisfied	Yes	No			
Yes	Satisfied	No	No			
Yes	Not satisfie	No	No			
No		No	No			
Yes	Satisfied	No	No			
Yes	Satisfied	No	No			
Yes	Very satisfi	Yes	No			
Yes	Not satisfie	Yes	No			
Yes	Very satisfi	No	No			
Yes	Very satisfi	Yes	Yes	No		No
Yes	Satisfied	No	No			
Yes	Very satisfi	No	Yes	No		No
Yes	Satisfied	Yes	No			
Yes	Satisfied	Yes	No			
Yes	Not satisfie	No	No			

For peer review only

Yes	Satisfied	No	No			
Yes	Not satisfie	No	No			
Yes	Not satisfie	No	No			
Yes	Very satisfi	Yes	Yes	No		No
Yes	Satisfied	No	No			
No		Yes	No			
No		No	No			
Yes	Not satisfie	No	No			
No		No	No			
Yes	Satisfied	No	No			
Yes	Satisfied	No	Yes	No		No
No		No	No			
Yes	Very satisfi	Yes	Yes	No		No
No		No	No			
Yes	Very satisfi	Yes	No			
Yes	Satisfied	No	No			
No		No	No			
Yes	Very satisfi	Yes	No			
No		No	No			
No		No	Yes	No		Yes
Yes	Satisfied	Yes	No			
No		No	No			
Yes	Satisfied	No	Yes	No		No
No		No	No			
No		No	No			
No		No	No			
Yes	Not satisfie	Yes	Yes	No		No
No		No	No			
Yes	Satisfied	Yes	No			
No		No	No			
Yes	Satisfied	Yes	No			
No		No	No			
Yes	Satisfied	Yes	No			
No		No	No			
Yes	Very satisfi	Yes	Yes	No		Yes
Yes	Satisfied	No	No			
No		No	No			
Yes	Satisfied	Yes	No			
Yes	Satisfied	Yes	No			
Yes	Very satisfi	Yes	No			
No		No	No			
Yes	Satisfied	No	No			
Yes	Satisfied	Yes	No			
Yes	Satisfied	Yes	No			
Yes	Satisfied	Yes	No			
No		Yes	No			
Yes	Satisfied	No	Yes	Yes	1 to 30	
Yes	Satisfied	No	Yes	Yes	1 to 30	
Yes	Satisfied	No	No			
No		No	No			
Yes	Not satisfie	No	No			
Yes	Very satisfi	Yes	Yes	No		Yes

For peer review only

Yes	Satisfied	No	No					
No		No	No					
Yes	Satisfied	No	Yes	No				No
No		Yes	No					
Yes	Very satisfi	Yes	No					
Yes	Satisfied	Yes	No					
Yes	Satisfied	No	No					
Yes	Not satisfie	No	Yes	No				No
Yes	Satisfied	No	Yes	Yes	1 to 5			
Yes	Satisfied	No	No					
No		No	No					
Yes	Not satisfie	No	No					
Yes	Satisfied	No	No					
Yes	Satisfied	Yes	No					
Yes	Satisfied	No	No					
Yes	Satisfied	No	Yes	No				No
Yes	Very satisfi	Yes	No					
No		No	No					
No		No	No					
No		No	No					
No		No	No					
Yes	Satisfied	No	Yes	No				No
No		No	No					
Yes	Satisfied	No	No					
No		No	Yes	No				No
No		No	No					
Yes	Not satisfie	No	No					
Yes	Satisfied	Yes	Yes	No				No
Yes	Satisfied	No	Yes	No				No
No		No	No					
Yes	Satisfied	No	No					
Yes	Satisfied	No	No					
Yes	Satisfied	No	Yes	No				No
Yes	Satisfied	No	No					
Yes	Satisfied	No	Yes	No				No
Yes	Satisfied	No	Yes	No				No
Yes	Satisfied	No	Yes	No				No
Yes	Satisfied	No	No					
No		No	No					
Yes	Satisfied	No	No					
Yes	Satisfied	No	No					
No		No	No					
Yes	Satisfied	No	Yes	No				No
Yes	Not satisfie	No	Yes	No				No
No		No	Yes	No				No
Yes	Satisfied	No	Yes	No				No
No		No	No					
No		No	No					
No		No	No					
Yes	Satisfied	No	Yes	No				No
Yes	Satisfied	No	No					
No		No	No					

For peer review only

No		No	No		
Yes	Satisfied	Yes	Yes	No	No
Yes	Satisfied	No	No		
Yes	Satisfied	No	No		
Yes	Satisfied	No	No		
No		No	Yes	No	No
No		No	No		
Yes	Satisfied	Yes	Yes	No	No
No		No	No		
No		No	No		
No		No	No		
No		No	No		
No		No	No		
Yes	Satisfied	Yes	Yes	No	No
No		No	No		
Yes	Satisfied	Yes	Yes	No	No
Yes	Satisfied	No	No		
Yes	Satisfied	No	No		
No		No	No		
No		No	No		
Yes	Satisfied	No	No		
No		No	No		
Yes	Satisfied	Yes	No		
Yes	Satisfied	No	No		
No		No	No		
Yes	Satisfied	No	No		
No		No	Yes	No	No
Yes	Satisfied	No	No		
Yes	Satisfied	No	Yes	No	No
Yes	Satisfied	No	Yes	No	No
No		No	No		
Yes	Satisfied	No	Yes	No	No
Yes	Satisfied	No	No		
Yes	Satisfied	No	Yes	No	No
No		No	No		
Yes	Satisfied	No	No		
Yes	Satisfied	No	Yes	No	No
No		No	No		
No		No	No		
Yes	Satisfied	No	Yes	No	No
No		No	No		
Yes	Satisfied	Yes	Yes	No	No
No		No	No		
Yes	Satisfied	No	No		
Yes	Satisfied	No	No		
No		No	No		
Yes	Satisfied	No	Yes	No	No
Yes	Satisfied	No	No		
Yes	Satisfied	Yes	Yes	No	No
No		No	No		
No		No	No		
Yes	Satisfied	No	No		

For peer review only

For peer review only - http://bmjopen.bmj.com/site/about/guidelines.xhtml

No		No	No		
No		No	No		
Yes	Satisfied	Yes	Yes	No	No
No		No	No		
No		No	No		
Yes	Satisfied	No	Yes	No	Yes
Yes	Satisfied	No	No		
Yes	Satisfied	No	No		
Yes	Satisfied	No	No		
No		No	No		
Yes	Satisfied	No	Yes	No	No
No		No	No		
Yes	Satisfied	Yes	No		
Yes	Satisfied	Yes	Yes	No	No
Yes	Very satisfi	No	Yes	No	No
Yes	Satisfied	No	No		
Yes	Satisfied	Yes	Yes	No	No
No		No	No		
Yes	Satisfied	No	No		
Yes	Satisfied	No	Yes	No	No
No		No	No		
Yes	Satisfied	No	No		
Yes	Satisfied	No	Yes	No	No
Yes	Satisfied	No	No		
Yes	Satisfied	No	Yes	No	No
No		No	No		
Yes	Satisfied	No	No		
Yes	Very satisfi	Yes	No		
No		No	Yes	No	No
No		No	No		
Yes	Satisfied	No	Yes	No	No
No		No	No		
No		No	No		
Yes	Satisfied	No	No		
Yes	Satisfied	No	No		
Yes	Satisfied	No	Yes	No	No
No		No	No		
Yes	Satisfied	No	Yes	No	No
Yes	Satisfied	Yes	Yes	No	No
No		No	No		
Yes	Satisfied	No	No		
Yes	Satisfied	No	Yes	No	No
No		No	No		
No		No	No		
No		No	No		
Yes	Satisfied	No	Yes	No	No
Yes	Satisfied	No	Yes	No	No
Yes	Satisfied	No	Yes	No	No
No		Yes	No		
Yes	Satisfied	No	No		
No		No	No		
Yes	Satisfied	No	No		

For peer review only

Yes	Not satisfie	No	No			
Yes	Satisfied	No	Yes	No		No
No		No	No			
Yes	Satisfied	No	Yes	Yes	1 to 30	
Yes	Satisfied	No	Yes	No		No
No		No	No			
No		No	No			
No		No	No			
No		No	No			
Yes	Satisfied	Yes	No			
No		No	No			
Yes	Satisfied	No	No			
No		No	No			
Yes	Satisfied	No	No			
Yes	Satisfied	No	No			
Yes	Satisfied	No	No			
Yes	Satisfied	No	No			
No		No	No			
No		No	No			
Yes	Satisfied	No	No			
Yes	Satisfied	No	No			
Yes	Satisfied	No	No			
Yes	Satisfied	No	No			
Yes	Satisfied	No	Yes	No		No
Yes	Satisfied	No	No			
Yes	Satisfied	No	No			
No		No	No			
No		No	Yes	No		No
No		No	No			
Yes	Satisfied	No	Yes	No		No
No		No	No			
Yes	Satisfied	No	No			
Yes	Satisfied	Yes	Yes	No		No
No		No	No			
Yes	Satisfied	Yes	Yes	No		No
Yes	Satisfied	No	Yes	Yes	1 to 30	
Yes	Satisfied	Yes	Yes	No		No
Yes	Satisfied	No	No			
Yes	Not satisfie	No	No			
No		No	No			
Yes	Satisfied	Yes	No			
Yes	Not satisfie	No	Yes	No		No
No		No	No			
Yes	Very satisfi	Yes	No			
No		No	No			
Yes	Satisfied	No	Yes	No		No
No		No	No			
No		No	No			
Yes	Satisfied	No	No			
Yes	Satisfied	No	No			
No		No	No			
Yes	Not satisfie	No	No			

For peer review only

No		No	No			
No		No	No			
No		No	No			
No		No	No			
Yes	Satisfied	No	Yes	No		No
Yes	Not satisfie	No	Yes	No		No
No		No	No			
No		No	No			
No		No	No			
Yes	Not satisfie	No	No			
No		No	No			
No		Yes	No			
Yes	Very satisfi	No	No			
Yes	Satisfied	Yes	No			
Yes	Satisfied	No	No			
Yes	Satisfied	Yes	No			
Yes	Very satisfi	Yes	No			
Yes	Satisfied	No	No			
No		No	No			
No		No	No			
No		No	No			
No		No	No			
Yes	Satisfied	No	No			
Yes	Not satisfie	No	No			
No		No	Yes	No		No
No		No	Yes	Yes	1 to 30	
No		No	No			
Yes	Satisfied	No	No			
No		No	No			
Yes	Satisfied	Yes	Yes	No		No
Yes	Satisfied	No	No			
Yes	Not satisfie	No	No			
Yes	Satisfied	No	No			
Yes	Satisfied	No	No			
No		No	No			
Yes	Very satisfi	Yes	No			
No		No	No			
No		No	No			
No		No	No			
Yes	Very satisfi	Yes	No			
No		No	No			
No		No	No			
Yes	Very satisfi	Yes	No			
Yes	Satisfied	No	Yes	No		No
No		No	No			
No		No	No			
No		No	No			
No		No	No			
Yes	Satisfied	No	No			
No		No	No			
No		No	No			
No		No	No			

For peer review only

Yes	Satisfied	No	No			
No		No	No			
No		No	No			
Yes	Satisfied	No	No			
No		No	Yes	No		No
Yes	Very satisfi	No	No			
Yes	Satisfied	Yes	No			
Yes	Satisfied	No	No			
No		No	No			
Yes	Satisfied	Yes	No			
No		No	No			
Yes	Satisfied	Yes	Yes	No		No
Yes	Satisfied	Yes	No			
Yes	Satisfied	Yes	Yes	No		No
Yes	Not satisfie	No	Yes	Yes	1 to 5	
No		No	No			
Yes	Satisfied	No	No			
Yes	Satisfied	No	Yes	No		No
Yes	Satisfied	No	No			
Yes	Satisfied	Yes	Yes	No		No
Yes	Satisfied	No	No			
No		No	No			
No		No	No			
Yes	Satisfied	No	No			
No		No	No			
Yes	Not satisfie	No	No			
No		No	No			
Yes	Satisfied	No	Yes	No		No
Yes	Not satisfie	Yes	No			
No		No	No			
No		No	No			
Yes	Satisfied	No	No			
Yes	Satisfied	No	Yes	No		No
Yes	Very satisfi	Yes	No			
No		No	No			
Yes	Satisfied	No	No			
Yes	Satisfied	No	No			
Yes	Satisfied	Yes	No			
No		No	No			
No		No	No			
No		No	No			
Yes	Very satisfi	No	No			
Yes	Satisfied	No	Yes	Yes	1 to 30	
Yes	Satisfied	No	Yes	No		No
Yes	Very satisfi	Yes	Yes	No		No
No		No	No			
Yes	Satisfied	No	No			
No		No	No			
Yes	Very satisfi	Yes	No			
Yes	Satisfied	No	Yes	No		No
No		No	No			
Yes	Very satisfi	Yes	No			

For peer review only

Yes	Not satisfie	No	No			
No		Yes	No			
No		No	No			
No		No	No			
Yes	Satisfied	No	No			
Yes	Satisfied	No	No			
Yes	Satisfied	Yes	No			
No		No	No			
No		No	No			
Yes	Satisfied	No	Yes	No		No
No		No	No			
No		No	No			
Yes	Very satisfi	No	No			
Yes	Satisfied	Yes	No			
No		Yes	No			
No		No	No			
No		No	No			
Yes	Very satisfi	Yes	No			
Yes	Satisfied	No	No			
Yes	Satisfied	Yes	No			
No		No	No			
Yes	Satisfied	No	No			
No		No	No			
No		No	No			
Yes	Satisfied	Yes	Yes	No		No
No		No	No			
Yes	Satisfied	Yes	Yes	No		No
No		No	No			
No		No	No			
No		No	No			
No		No	No			
Yes	Not satisfie	No	No			
Yes	Satisfied	Yes	No			
No		No	No			
Yes	Satisfied	No	No			
Yes	Satisfied	No	No			
Yes	Satisfied	No	No			
No		No	No			
No		No	No			
No		No	No			
No		No	No			
Yes	Very satisfi	Yes	No			
No		No	No			
Yes	Very satisfi	No	No			
Yes	Satisfied	Yes	No			
Yes	Satisfied	Yes	No			
Yes	Satisfied	No	No			
No		No	No			
Yes	Not satisfie	No	Yes	Yes	1 to 30	
No		No	No			
No		No	No			
No		No	No			

For peer review only

Yes	Not satisfie	No	No		
No		No	No		
Yes	Satisfied	No	No		
No		No	No		
No		No	No		
No		No	No		
No		No	Yes	No	No
Yes	Satisfied	No	No		
Yes	Satisfied	Yes	Yes	No	No
Yes	Not satisfie	No	No		
Yes	Satisfied	Yes	Yes	No	No
No		No	No		
Yes	Satisfied	No	No		
No		No	No		
No		No	No		
No		No	No		
Yes	Very satisfi	No	No		
No		No	No		
Yes	Very satisfi	Yes	No		
No		No	No		
No		No	No		
Yes	Satisfied	No	No		
Yes	Satisfied	No	No		
No		No	No		
Yes	Satisfied	Yes	No		
No		No	No		
Yes	Very satisfi	No	Yes	No	No
No		No	No		
Yes	Satisfied	No	No		
No		No	Yes	No	No
Yes	Satisfied	No	No		
Yes	Satisfied	No	No		
No		No	No		
Yes	Satisfied	No	No		
Yes	Satisfied	No	No		
No		No	No		
Yes	Satisfied	No	No		
Yes	Satisfied	Yes	No		
No		No	No		
No		No	No		
Yes	Satisfied	No	No		
No		No	No		
Yes	Satisfied	No	No		
Yes	Satisfied	No	No		
Yes	Not satisfie	Yes	No		
Yes	Very satisfi	No	No		
No		No	No		
Yes	Satisfied	Yes	Yes	No	No
Yes	Satisfied	Yes	No		
No		No	No		
Yes	Satisfied	No	No		
No		No	No		

For peer review only

Yes	Satisfied	No	No		
No		No	No		
Yes	Satisfied	Yes	No		
No		No	No		
Yes	Satisfied	No	Yes	No	Yes
No		Donâ€™t k	No		
Yes	Satisfied	No	No		
No		No	No		
Yes	Satisfied	No	Yes	No	No
Yes	Satisfied	Yes	No		
Yes	Not satisfie	No	No		
Yes	Satisfied	No	No		
No		No	No		
Yes	Satisfied	No	No		
Yes	Satisfied	No	No		
Yes	Satisfied	No	No		
No		No	No		
No		No	No		
No		No	Yes	No	No
Yes	Satisfied	No	No		
Yes	Satisfied	No	No		
No		No	No		
Yes	Satisfied	No	No		
No		No	No		
Yes	Not satisfie	No	No		
Yes	Satisfied	No	No		
No		No	No		
No		No	No		
No		No	No		
No		No	No		
No		No	No		
No		No	No		
No		No	No		
No		No	No		
Yes	Satisfied	Yes	No		
Yes	Satisfied	No	No		
Yes	Satisfied	No	No		
Yes	Satisfied	No	No		
Yes	Not satisfie	No	No		
No		No	No		
No		No	No		
No		No	No		
Yes	Satisfied	No	No		
No		No	No		
No		No	No		
Yes	Satisfied	No	No		
No		No	No		
Yes	Satisfied	No	No		
No		No	No		
No		No	No		
Yes	Satisfied	No	No		
Yes	Satisfied	Yes	No		

For peer review only

No		Donâ€™t k	No			
No		No	No			
Yes	Satisfied	No	No			
Yes	Not satisfie	No	Yes	No		No
No		No	No			
No		No	No			
No		No	Yes	No		No
Yes	Satisfied	No	No			
Yes	Satisfied	No	No			
No		No	No			
No		No	No			
Yes	Satisfied	No	No			
Yes	Satisfied	No	No			
No		No	No			
No		No	No			
Yes	Satisfied	Yes	No			
No		No	No			
No		No	No			
No		No	No			
No		No	No			
No		No	No			
Yes	Satisfied	No	No			
No		No	No			
Yes	Satisfied	Yes	No			
No		No	No			
Yes	Satisfied	No	No			
Yes	Satisfied	No	No			
Yes	Satisfied	No	No			
No		No	No			
Yes	Satisfied	No	No			
Yes	Satisfied	Yes	No			
Yes	Not satisfie	No	No			
Yes	Satisfied	No	Yes	No		No
Yes	Satisfied	No	No			
Yes	Not satisfie	No	Yes	No		No
No		No	No			
No		No	No			
No		No	No			
No		No	No			
No		No	No			
No		No	No			
No		No	No			
Yes	Not satisfie	No	No			
Yes	Satisfied	No	No			
Yes	Satisfied	No	No			
No		Donâ€™t k	No			
Yes	Satisfied	Yes	No			
Yes	Satisfied	Yes	Yes	Yes	1 to 30	
No		No	No			
Yes	Satisfied	No	Yes	No		No
No		No	No			
Yes	Satisfied	No	No			

For peer review only

Yes	Satisfied	No	No			
Yes	Satisfied	No	No			
No		No	No			
No		No	No			
No		No	No			
Yes	Satisfied	No	Yes	Yes	1 to 5	
No		No	Yes	No		No
No		No	No			
No		No	No			
No		No	No			
No		No	No			
Yes	Satisfied	Yes	No			
No		No	No			
Yes	Satisfied	Yes	Yes	No		No
Yes	Satisfied	No	No			
No		No	No			
No		No	No			
Yes	Satisfied	No	No			
Yes	Satisfied	No	No			
Yes	Very satisfi	No	No			
No		No	Yes	Yes	1 to 30	
Yes	Satisfied	No	Yes	No		No
Yes	Satisfied	No	No			
No		No	No			
Yes	Satisfied	No	No			
No		No	No			
No		No	No			
No		No	No			
Yes	Satisfied	No	Yes	No		No
No		Yes	Yes	No		Yes
No		No	Yes	No		No
Yes	Satisfied	No	No			
Yes	Satisfied	No	Yes	No		No
No		No	No			
No		No	No			
No		No	No			
No		Yes	No			
Yes	Satisfied	No	No			
Yes	Satisfied	No	No			
No		Yes	Yes	No		No
Yes	Satisfied	No	No			
Yes	Satisfied	No	Yes	No		No
No		No	No			
Yes	Satisfied	No	No			
Yes	Satisfied	No	No			
Yes	Satisfied	No	Yes	No		Yes
Yes	Satisfied	No	No			
Yes	Satisfied	No	No			
Yes	Satisfied	No	No			
No		No	No			
No		No	No			
No		No	No			

For peer review only

Yes	Satisfied	No	No			
Yes	Satisfied	No	Yes	No		No
No		Yes	Yes	No		Yes
Yes	Satisfied	No	No			
Yes	Satisfied	No	No			
No		No	No			
No		No	Yes	Yes	1 to 5	
No		No	No			
No		No	No			
Yes	Satisfied	No	Yes	No		No
Yes	Satisfied	No	No			
No		No	No			
No		No	No			
No		No	No			
Yes	Satisfied	No	No			
Yes	Satisfied	No	No			
No		No	No			
No		No	Yes	No		No
Yes	Satisfied	Yes	Yes	No		No
Yes	Satisfied	No	No			
Yes	Satisfied	Yes	No			
Yes	Satisfied	Yes	No			
Yes	Very satisfi	Yes	No			
No		No	No			
Yes	Satisfied	No	No			
No		No	No			
No		No	No			
Yes	Satisfied	No	No			
Yes	Satisfied	No	No			
Yes	Satisfied	No	No			
Yes	Satisfied	No	No			
Yes	Satisfied	No	No			
No		No	No			
Yes	Satisfied	No	No			
No		Yes	Yes	Yes	1 to 30	
No		No	No			
No		No	No			
Yes	Satisfied	No	No			
Yes	Satisfied	No	No			
No		No	No			
No		No	No			
Yes	Satisfied	No	No			
Yes	Satisfied	No	No			
No		No	No			
Yes	Satisfied	No	No			
Yes	Satisfied	No	No			
No		No	No			
No		No	Yes	No		No
No		No	No			
Yes	Satisfied	No	No			
No		No	No			
Yes	Satisfied	No	No			

No		No	Yes	No		No
No		No	No			
Yes	Satisfied	No	No			
No		No	No			
No		No	No			
Yes	Not satisfie	No	No			
No		No	No			
Yes	Satisfied	No	No			
Yes	Satisfied	No	No			
No		No	No			
No		No	No			
No		No	No			
No		No	No			
No		No	No			
No		No	No			
No		No	Yes	No		No
No		No	No			
Yes	Satisfied	No	No			
No		No	No			
Yes	Satisfied	No	No			
No		No	No			
No		No	No			
Yes	Satisfied	No	No			
No		No	No			
No		No	No			
No		No	No			
Yes	Satisfied	No	No			
Yes	Satisfied	No	No			
Yes	Not satisfie	No	Yes	No		No
No		No	No			
No		No	No			
Yes	Satisfied	Yes	Yes	No		No
No		No	No			
No		No	Yes	Yes	1 to 5	
No		No	No			
No		No	No			
No		No	No			
No		No	No			
Yes	Very satisfi	No	No			
No		No	No			
Yes	Satisfied	No	No			
No		No	No			
No		No	No			
No		No	Yes	No		No
No		No	No			
No		No	No			
Yes	Satisfied	No	No			
Yes	Satisfied	No	No			
No		No	No			
Yes	Satisfied	No	No			
No		No	No			
No		No	No			

For peer review only

Yes	Satisfied	No	No			
Yes	Satisfied	No	No			
Yes	Satisfied	No	No			
No		No	No			
No		No	Yes	No		No
Yes	Satisfied	No	No			
Yes	Satisfied	No	No			
No		No	No			
Yes	Satisfied	No	No			
No		No	No			
No		No	No			
No		No	No			
No		No	No			
Yes	Satisfied	No	Yes	No		No
No		No	Yes	No		No
No		No	No			
No		No	No			
Yes	Satisfied	No	No			
No		No	Yes	No		Yes
No		No	Yes	No		No
No		Yes	No			
Yes	Satisfied	No	Yes	No		No
Yes	Satisfied	No	No			
Yes	Satisfied	Yes	Yes	No		No
Yes	Not satisfie	Yes	Yes	No		No
No		No	No			
No		No	No			
No		No	No			
No		No	No			
No		No	No			
Yes	Satisfied	No	Yes	No		No
No		No	No			
No		Yes	No			
No		No	No			
Yes	Satisfied	No	No			
Yes	Not satisfie	No	No			
No		No	No			
Yes	Satisfied	No	Yes	No		No
Donâ€™t know		Donâ€™t k	No			
No		No	No			
No		No	No			
No		No	Yes	Yes	1 to 5	
Yes	Satisfied	Donâ€™t k	No			
Yes	Not satisfie	Yes	Yes	No		Yes
No		No	Yes	No		No
No		No	Yes	No		No
No		No	Yes	No		No
Yes	Satisfied	No	No			
Yes	Satisfied	No	No			
Yes	Satisfied	Yes	Yes	No		Yes
Yes	Satisfied	No	No			
No		No	No			

For peer review only

Yes	Satisfied	No	Yes	No		No
No		No	No			
Yes	Satisfied	Yes	Yes	No		Yes
No		No	No			
No		No	No			
Yes	Satisfied	Yes	No			
No		No	No			
Yes	Satisfied	No	No			
No		No	No			
No		No	No			
Yes	Satisfied	No	Yes	No		Yes
No		No	No			
No		No	No			
No		No	No			
No		No	No			
No		No	No			
No		No	No			
No		No	No			
Yes	Satisfied	Donâ€™t k	No			
Yes	Satisfied	Yes	Yes	No		No
Yes	Not satisfie	Yes	Yes	Yes	1 to 30	
Yes	Satisfied	No	No			
Yes	Satisfied	No	Yes	No		No
No		No	Yes	No		No
No		No	No			
No		Yes	Yes	No		Yes
Yes	Satisfied	No	No			
Yes	Very satisfi	No	Yes	No		Yes
Yes	Satisfied	Yes	Yes	No		No
Yes	Satisfied	No	No			
No		No	No			
No		No	No			
Yes	Satisfied	Yes	Yes	No		No
No		Yes	Yes	No		No
Yes	Satisfied	No	Yes	Yes	1 to 30	
No		No	No			
No		No	No			
No		No	No			
No		No	No			
Yes	Satisfied	Yes	Yes	No		No
No		No	Yes	No		No
No		Donâ€™t k	No			
No		No	No			
Yes	Satisfied	No	No			
Yes	Satisfied	Yes	Yes	No		No
Yes	Not satisfie	No	Yes	No		No
No		No	No			
No		No	No			
Yes	Satisfied	Yes	Yes	Yes	1 to 30	
No		No	No			
No		No	Yes	No		No
Yes	Satisfied	No	Yes	No		No

For peer review only

No		No	Yes	No		Yes
No		No	Yes	No		No
Yes	Satisfied	No	No			
No		Donâ€™t k	No			
Yes	Satisfied	Yes	No			
No		No	Yes	Yes	1 to 5	
No		No	No			
No		Yes	Yes	No		No
No		No	No			
No		No	No			
Yes	Satisfied	Donâ€™t k	Yes	No		No
No		No	No			
No		No	No			
No		No	No			
No		No	Yes	No		No
No		No	No			
No		No	No			
No		No	No			
No		Yes	Yes	No		No
Yes	Satisfied	Yes	Yes	No		Yes
Yes	Satisfied	No	No			
No		No	No			
No		No	No			
Yes	Satisfied	Yes	No			
Yes	Satisfied	No	Yes	No		No
No		No	Yes	No		No
No		No	No			
No		No	Yes	No		No
Yes	Satisfied	Yes	Yes	No		Yes
No		No	No			
Yes	Satisfied	Yes	Yes	No		No
No		No	No			
No		No	No			
Yes	Satisfied	No	No			
No		No	No			
No		No	No			
No		No	No			
No		No	No			
No		No	No			
Yes	Satisfied	Yes	No			
No		No	Yes	No		No
No		No	No			
No		No	No			
No		No	No			
Yes	Satisfied	No	Yes	No		No
No		No	Yes	No		No
Yes	Satisfied	Yes	Yes	No		No
No		Yes	No			
No		Yes	Yes	No		No
No		No	Yes	No		No
No		No	No			
Yes	Satisfied	No	No			

For peer review only

No		No	No			
Yes	Satisfied	Yes	No			
Yes	Satisfied	No	Yes	No		No
No		Yes	Yes	No		Yes
Yes	Satisfied	Donâ€™t k	No			
No		No	Yes	No		Yes
Donâ€™t know		Donâ€™t k	No			
No		No	No			
Yes	Satisfied	No	No			
Yes	Satisfied	Yes	Yes	No		Yes
Yes	Satisfied	No	Yes	Yes	1 to 5	
No		No	No			
No		Yes	Yes	No		No
No		No	No			
No		Yes	No			
Yes	Satisfied	Yes	Yes	No		No
No		No	No			
No		No	No			
No		No	No			
No		No	Yes	No		No
No		No	Yes	No		No
Yes	Satisfied	Donâ€™t k	Yes	Yes	1 to 5	
No		No	Yes	No		No
Yes	Satisfied	Yes	Yes	No		No
No		No	No			
Yes	Satisfied	Yes	Yes	No		Yes
No		Yes	Yes	No		No
Yes	Satisfied	Yes	Yes	No		Yes
No		Yes	No			
No		No	No			
Yes	Satisfied	Yes	Yes	No		Yes
No		Donâ€™t k	Yes	No		No
No		No	Yes	No		No
Yes	Satisfied	No	No			
No		No	No			
Yes	Satisfied	Yes	No			
No		No	Yes	No		No
No		No	No			
No		No	No			
Yes	Not satisfie	Yes	No			
No		No	No			
Yes	Satisfied	Yes	Yes	Yes	1 to 5	
Yes	Satisfied	No	No			
No		Yes	No			
Yes	Satisfied	Yes	No			
No		No	No			
Yes	Satisfied	Yes	No			
No		No	No			
Yes	Satisfied	No	No			
No		Donâ€™t k	No			
Yes	Satisfied	No	Yes	No		No
Yes	Very satisfi	Yes	No			

For peer review only

Yes	Not satisfie	No	No			
Yes	Satisfied	No	No			
No		No	No			
Yes	Satisfied	No	Yes	No		No
Yes	Not satisfie	No	No			
Yes	Satisfied	No	No			
Yes	Satisfied	No	No			
No		Yes	No			
No		No	No			
Yes	Satisfied	Yes	Yes	No		Yes
Yes	Satisfied	No	No			
No		No	No			
No		No	No			
Yes	Very satisfi	No	Yes	No		No
Yes	Satisfied	No	No			
No		No	No			
Yes	Satisfied	No	No			
No		No	No			
No		No	No			
No		No	No			
Yes	Satisfied	Yes	No			
No		No	No			
Yes	Satisfied	No	No			
No		No	No			
Yes	Satisfied	No	No			
No		No	Yes	No		No
Yes	Satisfied	Donâ€™t k	No			
Yes	Very satisfi	No	Yes	Yes	1 to 5	
Yes	Very satisfi	No	No			
No		No	No			
No		No	No			
Yes	Satisfied	No	No			
Yes	Satisfied	No	Yes	No		No
Yes	Satisfied	No	No			
Yes	Satisfied	Yes	No			
Yes	Not satisfie	No	No			
Yes	Satisfied	Yes	No			
No		No	No			
No		No	No			
Yes	Satisfied	No	Yes	No		No
Yes	Not satisfie	Yes	No			
Yes	Satisfied	No	No			
No		No	No			
Yes	Satisfied	No	No			
Yes	Satisfied	No	No			
No		No	No			
Yes	Satisfied	No	No			
Yes	Very satisfi	Yes	No			
No		No	No			
Yes	Satisfied	No	No			
No		Yes	No			
No		No	No			

For peer review only

No		No	No		
No		No	Yes	No	No
Yes	Not satisfie	No	No		
No		No	No		
No		No	No		
No		No	No		
No		No	No		
No		No	No		
Yes	Satisfied	No	No		
Yes	Very satisfi	No	No		
Yes	Satisfied	Yes	Yes	No	No
Yes	Satisfied	No	No		
No		No	No		
Yes	Satisfied	Yes	No		
No		No	No		
Yes	Satisfied	No	No		
No		No	No		
Yes	Satisfied	No	No		
No		No	No		
Yes	Not satisfie	Yes	Yes	No	Yes
Yes	Satisfied	No	No		
No		No	No		
No		No	No		
Yes	Satisfied	Yes	No		
Yes	Very satisfi	No	No		
No		No	No		
No		No	No		
No		Yes	No		
Yes	Satisfied	No	No		
No		No	No		
Yes	Not satisfie	No	No		
No		No	No		
No		No	No		
No		No	No		
No		No	No		
Yes	Very satisfi	No	No		
No		No	No		
Yes	Satisfied	Yes	No		
No		No	No		
Yes	Satisfied	No	No		
Yes	Not satisfie	No	No		
No		No	No		
Yes	Not satisfie	Yes	No		
Yes	Satisfied	No	No		
Yes	Very satisfi	No	No		
No		No	No		
No		No	No		
Yes	Satisfied	No	No		
No		No	No		
Yes	Satisfied	Yes	No		
Yes	Satisfied	Yes	No		
Yes	Satisfied	Yes	No		

For peer review only

No		No	No			
No		No	No			
Yes	Satisfied	No	No			
No		No	Yes	Yes	1 to 30	
Yes	Not satisfie	No	No			
Yes	Satisfied	No	No			
No		No	No			
No		No	No			
Yes	Very satisfi	No	No			
Yes	Satisfied	Yes	No			
Yes	Satisfied	Yes	No			
Yes	Satisfied	No	Yes	Yes	1 to 30	
Yes	Satisfied	No	No			
Yes	Satisfied	No	No			
Yes	Satisfied	No	No			
Yes	Very satisfi	No	No			
No		No	No			
No		No	No			
Yes	Satisfied	No	Yes	No		No
No		No	No			
Yes	Satisfied	Yes	No			
Yes	Satisfied	No	No			
Yes	Satisfied	No	No			
Yes	Satisfied	No	Yes	No		No
No		Yes	No			
Yes	Very satisfi	No	No			
Yes	Satisfied	Yes	No			
No		No	No			
Yes	Satisfied	No	No			
Yes	Satisfied	No	No			
Yes	Very satisfi	Yes	No			
Yes	Satisfied	Yes	No			
Yes	Satisfied	Yes	No			
Yes	Very satisfi	No	No			
No		No	No			
Yes	Satisfied	Yes	No			
Yes	Satisfied	No	Yes	No		No
Yes	Not satisfie	No	No			
Yes	Satisfied	No	No			
No		No	No			
No		No	No			
Yes	Satisfied	Yes	Yes	Yes	1 to 5	
No		No	No			
Yes	Very satisfi	Yes	Yes	Yes	1 to 30	
Yes	Not satisfie	No	No			
No		No	No			
Yes	Satisfied	No	No			
No		No	No			
No		No	No			
Yes	Very satisfi	No	No			
Yes	Very satisfi	No	No			
No		No	No			

For peer review only

For peer review only - http://bmjopen.bmj.com/site/about/guidelines.xhtml

Yes	Satisfied	Yes	No					
Yes	Satisfied	Yes	No					
Yes	Satisfied	No	No					
Yes	Satisfied	No	No					
Yes	Not satisfie	Yes	No					
Yes	Satisfied	No	No					
No		No	No					
No		No	No					
No		No	No					
Yes	Satisfied	No	Yes	Yes	1 to 30			
Yes	Satisfied	No	No					
No		No	No					
Yes	Satisfied	No	No					
No		No	No					
Yes	Satisfied	No	No					
No		Yes	No					
Yes	Very satisfi	No	No					
No		No	No					
No		No	No					
No		No	No					
No		No	No					
No		No	No					
No		No	No					
No		No	No					
No		No	No					
Yes	Satisfied	No	No					
No		No	No					
Yes	Satisfied	No	No					
No		No	No					
No		No	No					
Yes	Satisfied	No	No					
No		No	No					
Yes	Satisfied	No	Yes	No				No
Yes	Satisfied	No	No					
No		No	No					
No		No	No					
No		No	No					
No		No	No					
No		No	No					
No		No	No					
No		No	No					
No		No	No					
No		No	No					
Yes	Satisfied	Yes	Yes	Yes	1 to 30			
No		No	No					
No		No	No					
Yes	Satisfied	No	No					
No		No	No					
Yes	Satisfied	Yes	Yes	No				No
No		No	No					
Yes	Satisfied	No	No					
No		No	No					

For peer review only

No		No	No		
No		No	No		
No		No	No		
No		No	No		
No		No	No		
No		No	No		
No		No	No		
No		No	No		
No		No	No		
Yes	Satisfied	No	No		
No		No	No		
No		No	No		
No		No	No		
No		No	No		
No		No	No		
No		No	No		
No		No	No		
No		No	No		
No		No	No		
No		No	No		
No		No	No		
No		No	No		
No		No	Yes	No	No
No		No	Yes	No	No
No		No	No		
No		No	No		
No		No	No		
Yes	Satisfied	Donâ€™t k	No		
No		No	No		
No		No	No		
Yes	Satisfied	No	No		
Yes	Satisfied	No	No		
No		No	No		
No		No	No		
No		No	No		
No		No	No		
No		No	No		
No		No	No		
Yes	Satisfied	No	No		
No		No	No		
No		No	No		
No		No	No		
No		No	No		
No		No	No		
No		No	No		
No		No	No		
No		No	No		
Yes	Satisfied	No	No		
No		No	No		
No		No	No		
No		No	No		
Yes	Satisfied	No	No		

For peer review only

No		No	Yes	Yes	1 to 5			
No		No	No					
No		No	No					
No		No	No					
Yes	Satisfied	No	No					
No		No	No					
Yes	Satisfied	No	No					
No		No	No					
No		Yes	Yes	No				Yes
Yes	Satisfied	No	No					
Yes	Satisfied	No	No					
No		No	No					
No		No	No					
Yes	Satisfied	No	No					
Yes	Very satisfi	Yes	Yes	No				No
Yes	Satisfied	No	No					
No		No	No					
Yes	Satisfied	No	No					
No		No	No					
No		No	No					
No		No	No					
No		No	No					
No		No	Yes	No				No
No		No	No					
No		No	Yes	No				No
No		No	No					
No		No	No					
No		No	No					
No		No	No					
No		No	No					
No		No	No					
Yes	Satisfied	No	No					
No		No	No					
No		No	No					
No		No	No					
No		No	No					
No		No	No					
No		No	No					
No		No	No					
No		No	No					
No		No	No					
No		No	No					
No		No	No					
No		No	No					
No		No	No					
No		No	No					
No		No	No					
No		No	No					
No		No	No					
No		No	No					
No		No	No					
No		No	No					

For peer review only

Yes	Very satisfi	No	No		
No		No	No		
No		No	No		
No		No	No		
No		No	No		
No		No	No		
No		No	No		
Yes	Satisfied	No	No		
No		No	No		
No		Yes	Yes	No	Yes
Yes	Satisfied	No	No		
No		No	No		
No		No	No		
No		No	No		
No		No	No		
No		No	No		
No		No	No		
No		No	No		
No		No	No		
No		No	No		
No		No	No		
No		No	No		
Yes	Very satisfi	Yes	No		
Yes	Satisfied	No	No		
Yes	Satisfied	No	No		
Yes	Satisfied	Yes	No		
Yes	Satisfied	No	No		
Yes	Satisfied	No	No		
No		Yes	No		
Yes	Satisfied	No	No		
No		No	No		
No		No	No		
No		No	No		
No		No	No		
No		No	No		
No		No	No		
No		No	No		
No		No	No		
No		No	No		
No		No	No		
No		No	Yes	No	No
No		No	No		
Yes	Satisfied	Yes	Yes	No	No
No		No	No		
No		No	No		
No		No	No		
Yes	Very satisfi	No	No		
Yes	Satisfied	Yes	No		
No		No	No		
Yes	Very satisfi	No	No		
Yes	Very satisfi	Yes	No		
No		No	No		

For peer review only

No		No	No			
No		No	No			
Yes	Satisfied	No	No			
Yes	Satisfied	No	Yes	No		No
Yes	Satisfied	No	No			
Yes	Satisfied	No	No			
Yes	Very satisfi	No	No			
No		No	No			
Yes	Very satisfi	No	No			
Yes	Not satisfie	No	No			
Yes	Satisfied	No	No			
Yes	Satisfied	No	Yes	No		No
Yes	Satisfied	No	Yes	Yes	1 to 5	
Yes	Satisfied	No	No			
No		No	No			
Yes	Satisfied	Yes	No			
Yes	Satisfied	No	No			
No		Yes	No			
Yes	Satisfied	Yes	No			
Yes	Satisfied	No	No			
Yes	Satisfied	No	Yes	No		No
No		No	No			
Yes	Satisfied	No	No			
Yes	Very satisfi	No	Yes	No		No
Yes	Satisfied	No	No			
No		No	No			
Yes	Satisfied	No	Yes	No		No
No		No	No			
No		No	No			
Yes	Satisfied	No	No			
Yes	Satisfied	No	No			
Yes	Very satisfi	No	No			
No		No	No			
Yes	Satisfied	No	No			
Yes	Very satisfi	No	No			
Yes	Satisfied	No	No			
Yes	Very satisfi	No	No			
Yes	Very satisfi	Yes	No			
No		Yes	No			
Yes	Satisfied	No	Yes	No		No
Yes	Satisfied	Yes	Yes	No		No
Yes	Satisfied	No	No			
Yes	Satisfied	Yes	No			
Yes	Very satisfi	No	No			
No		No	No			
Yes	Satisfied	No	No			
No		No	Yes	No		No
Yes	Satisfied	No	Yes	No		No
Yes	Very satisfi	No	No			
No		No	No			
No		No	No			
Yes	Satisfied	No	No			

For peer review only

Yes	Satisfied	No	No			
No		No	No			
Yes	Very satisfi	No	No			
Yes	Satisfied	Yes	No			
No		No	No			
No		No	No			
No		No	No			
Yes	Satisfied	No	No			
Yes	Satisfied	No	No			
Yes	Very satisfi	No	No			
No		No	No			
No		No	Yes	No		No
Yes	Very satisfi	No	No			
No		No	No			
Yes	Satisfied	Yes	Yes	No		No
Yes	Satisfied	No	Yes	No		No
Yes	Satisfied	Yes	No			
Yes	Very satisfi	No	Yes	Yes	1 to 5	
No		No	No			
Yes	Very satisfi	No	No			
Yes	Satisfied	No	No			
Yes	Not satisfie	Yes	No			
No		No	Yes	No		No
No		No	No			
Yes	Satisfied	Yes	No			
Yes	Satisfied	Yes	Yes	Yes	1 to 30	
Yes	Satisfied	No	No			
Yes	Satisfied	No	Yes	No		No
Yes	Satisfied	Yes	No			
No		No	No			
Yes	Satisfied	Yes	No			
Yes	Not satisfie	Yes	No			
No		No	No			
Yes	Very satisfi	No	No			
Yes	Satisfied	No	No			
Yes	Very satisfi	Yes	No			
Yes	Satisfied	No	No			
Yes	Very satisfi	Yes	No			
No		No	No			
Yes	Not satisfie	No	No			
Yes	Satisfied	Yes	No			
No		No	No			
No		No	No			
Yes	Satisfied	No	No			
Yes	Very satisfi	Yes	No			
Yes	Satisfied	No	Yes	No		No
No		No	No			
Yes	Very satisfi	Yes	Yes	No		Yes
Yes	Satisfied	No	No			
Yes	Not satisfie	No	Yes	No		No
Yes	Satisfied	No	No			
No		Yes	No			

For peer review only

Yes	Very satisfi	No	No			
Yes	Not satisfie	No	Yes	No		No
Yes	Satisfied	No	No			
Yes	Satisfied	Yes	No			
Yes	Very satisfi	Yes	No			
No		No	No			
Yes	Not satisfie	No	No			
Yes	Satisfied	No	No			
No		No	Yes	No		No
No		No	No			
Yes	Satisfied	No	No			
Yes	Satisfied	No	No			
Yes	Satisfied	No	No			
No		No	No			
Yes	Satisfied	No	Yes	No		No
Yes	Satisfied	No	No			
Yes	Satisfied	No	No			
No		No	No			
No		No	Yes	No		No
Yes	Satisfied	No	No			
No		Yes	No			
Yes	Satisfied	No	Yes	No		No
No		No	No			
No		No	No			
No		No	No			
No		No	No			
No		No	No			
Yes	Satisfied	Yes	Yes	Yes	1 to 30	
Yes	Very satisfi	Yes	Yes	No		Yes
No		Donâ€™t k	No			
No		No	No			
No		No	Yes	No		No
No		No	No			
Yes	Very satisfi	No	No			
Yes	Very satisfi	No	No			
Yes	Satisfied	No	No			
No		No	No			
Yes	Satisfied	No	No			
No		No	No			
Yes	Satisfied	Yes	No			
Yes	Satisfied	Donâ€™t k	No			
No		No	No			
Yes	Satisfied	No	No			
No		No	No			
Yes	Satisfied	No	No			
Yes	Satisfied	No	No			
No		No	Yes	No		No
Yes	Satisfied	Yes	No			
Yes	Satisfied	Yes	Yes	Yes	1 to 30	
Yes	Very satisfi	Yes	No			
No		No	No			
No		No	Yes	Yes	1 to 30	

For peer review only

Yes	Not satisfie	No	No			
Yes	Satisfied	No	No			
Yes	Very satisfi	No	No			
No		No	No			
Yes	Satisfied	No	Yes	No		No
Yes	Not satisfie	No	No			
No		No	No			
No		No	No			
Yes	Satisfied	No	No			
Yes	Satisfied	Yes	Yes	Yes	1 to 5	
Yes	Satisfied	No	No			
Yes	Satisfied	No	Yes	No		No
Yes	Not satisfie	No	No			
Yes	Satisfied	No	Yes	No		No
Yes	Very satisfi	No	No			
Yes	Satisfied	No	No			
No		No	No			
Yes	Very satisfi	No	No			
Yes	Satisfied	No	Yes	No		No
No		No	No			
No		No	No			
Yes	Satisfied	No	No			
Yes	Satisfied	No	Yes	No		No
Yes	Satisfied	No	No			
Yes	Satisfied	No	No			
Yes	Satisfied	No	Yes	No		No
Yes	Very satisfi	Yes	No			
Yes	Very satisfi	Yes	No			
No		No	No			
Yes	Not satisfie	Yes	Yes	Yes	1 to 5	
No		No	No			
Yes	Satisfied	No	No			
Yes	Very satisfi	No	No			
No		No	No			
Yes	Very satisfi	Yes	No			
Yes	Satisfied	No	No			
Yes	Very satisfi	Yes	Yes	No		Yes
Yes	Satisfied	No	No			
Yes	Very satisfi	Yes	No			
Yes	Very satisfi	Yes	No			
Yes	Very satisfi	Yes	No			
Yes	Satisfied	Yes	No			
Yes	Very satisfi	Yes	Yes	No		Yes
Yes	Satisfied	No	No			
No		No	No			
Yes	Satisfied	No	No			
No		No	No			
No		No	No			
Yes	Satisfied	No	Yes	No		No
Yes	Satisfied	Yes	Yes	No		No
Yes	Not satisfie	No	No			
Yes	Not satisfie	Yes	Yes	Yes	1 to 30	

For peer review only

No		No	No			
No		No	No			
No		No	No			
Yes	Very satisfi	No	No			
Yes	Very satisfi	No	Yes	No		No
No		No	No			
No		No	No			
Yes	Satisfied	No	No			
Yes	Satisfied	No	No			
No		No	No			
Yes	Very satisfi	Yes	No			
Yes	Satisfied	Yes	Yes	Yes	1 to 30	
Yes	Satisfied	No	No			
Yes	Satisfied	No	No			
No		No	No			
Yes	Satisfied	Yes	No			
No		No	No			
Yes	Satisfied	No	No			
Yes	Satisfied	Yes	No			
No		No	No			
Yes	Satisfied	No	No			
Yes	Very satisfi	Yes	Yes	No		Yes
Yes	Very satisfi	Yes	Yes	Yes	1 to 5	
Yes	Not satisfie	Yes	Yes	No		No
Yes	Very satisfi	No	No			
Yes	Satisfied	No	No			
Yes	Satisfied	No	No			
Yes	Satisfied	No	No			
Yes	Very satisfi	No	No			
Yes	Satisfied	Yes	No			
No		No	No			
Yes	Satisfied	No	Yes	No		No
Yes	Satisfied	No	No			
Yes	Satisfied	No	No			
Yes	Not satisfie	No	No			
Yes	Satisfied	No	No			
Yes	Very satisfi	No	No			
Yes	Satisfied	No	Yes	Yes	1 to 30	
Yes	Satisfied	No	No			
No		No	No			
Yes	Satisfied	No	No			
No		No	No			
Yes	Satisfied	No	No			
Yes	Satisfied	No	No			
Yes	Satisfied	No	No			
No		No	No			
Yes	Satisfied	Yes	No			
Yes	Satisfied	No	Yes	No		No
Yes	Satisfied	Yes	No			
Yes	Very satisfi	Yes	No			
No		No	No			
No		No	No			

For peer review only

Yes	Satisfied	No	Yes	No		No
Yes	Not satisfie	No	No			
No		No	No			
Yes	Satisfied	No	No			
Yes	Very satisfi	No	No			
Donâ€™t know		No	No			
Yes	Very satisfi	Yes	No			
Yes	Satisfied	No	No			
No		Donâ€™t k	No			
Yes	Satisfied	No	No			
Yes	Very satisfi	No	No			
Yes	Satisfied	No	No			
No		No	No			
Yes	Satisfied	No	No			
Donâ€™t know		No	No			
Yes	Satisfied	No	Yes	No		No
Yes	Satisfied	Yes	Yes	Yes	1 to 5	
Yes	Not satisfie	No	No			
No		No	No			
Yes	Satisfied	No	Yes	No		No
No		No	No			
Yes	Satisfied	No	Yes	No		No
Yes	Very satisfi	Yes	No			
Yes	Not satisfie	Yes	Yes	Yes	1 to 5	
No		No	No			
No		No	No			
No		No	No			
No		No	No			
No		No	No			
Yes	Satisfied	No	No			
No		No	No			
No		No	No			
Yes	Satisfied	No	No			
Donâ€™t know		Donâ€™t k	No			
Yes	Not satisfie	Yes	No			
No		No	No			
Yes	Not satisfie	Yes	No			
No		No	No			
Yes	Very satisfi	No	No			
Yes	Satisfied	Yes	No			
Yes	Satisfied	Yes	No			
Yes	Not satisfie	No	No			
Yes	Satisfied	No	No			
Yes	Not satisfie	Yes	Yes	No		Yes
Yes	Satisfied	No	No			
No		No	No			
No		No	No			
Yes	Satisfied	Yes	Yes	No		No
Yes	Satisfied	No	No			
No		No	Yes	No		No
Yes	Satisfied	Yes	No			
No		No	Yes	No		No

For peer review only

No		No	No			
Yes	Very satisfi	No	No			
Yes	Satisfied	Yes	No			
No		No	No			
No		No	No			
Yes	Satisfied	No	No			
Yes	Very satisfi	Yes	Yes	No		Yes
Yes	Very satisfi	No	Yes	No		No
Yes	Satisfied	No	No			
Yes	Satisfied	No	No			
Yes	Very satisfi	No	No			
No		No	No			
Yes	Satisfied	No	No			
Yes	Satisfied	No	No			
Yes	Very satisfi	No	No			
No		No	No			
Yes	Very satisfi	No	No			
Yes	Satisfied	No	No			
No		No	Yes	No		No
Yes	Not satisfie	No	No			
Yes	Satisfied	No	No			
Yes	Not satisfie	No	No			
No		No	No			
No		No	No			
Yes	Satisfied	No	No			
Yes	Satisfied	No	No			
No		No	No			
Yes	Satisfied	No	Yes	No		No
Yes	Satisfied	Yes	No			
Yes	Not satisfie	Yes	No			
Yes	Satisfied	No	No			
Yes	Satisfied	No	No			
Yes	Satisfied	No	Yes	No		No
Yes	Satisfied	No	No			
Yes	Not satisfie	No	No			
Yes	Satisfied	Yes	No			
Yes	Not satisfie	No	No			
Yes	Satisfied	Yes	Yes	No		No
Yes	Satisfied	No	Yes	No		No
Yes	Satisfied	No	No			
Yes	Satisfied	No	No			
Yes	Satisfied	No	No			
Yes	Satisfied	No	No			
No		No	No			
Yes	Not satisfie	No	No			
Yes	Satisfied	No	No			
Yes	Satisfied	Yes	Yes	No		No
Yes	Very satisfi	No	Yes	Yes	1 to 5	
Yes	Very satisfi	No	No			
Yes	Not satisfie	No	No			
Yes	Satisfied	No	No			

For peer review only

q310	q311a	q311b	q311c	q311d	q311e	q311f	q311g	q311h
No	No problen	No problen	No problen	No problen	Small probl	No problen	No problen	No problen
No	No problen	No problen	No problen	No problen	No problen	No problen	No problen	No problen
No	No problen	No problen	Small probl	No problen	Big probler	No problen	No problen	No problen
	Small probl	Small probl	Small probl	Small probl	Small probl	Small probl	Small probl	Small probl
No	No problen	No problen	No problen	No problen	No problen	No problen	No problen	Big probler
	No problen	No problen	No problen	No problen	No problen	No problen	No problen	No problen
	No problen	No problen	Small probl	No problen	Big probler	No problen	No problen	No problen
	Small probl	Small probl	Small probl	Small probl	Small probl	Small probl	Small probl	Small probl
	No problen	No problen	No problen	No problen	Big probler	Small probl	No problen	No problen
	No problen	No problen	No problen	No problen	Small probl	Small probl	No problen	No problen
	No problen	No problen	No problen	No problen	Small probl	No problen	No problen	No problen
No	No problen	No problen	No problen	No problen	No problen	No problen	No problen	No problen
	Small probl	No problen	No problen	No problen	No problen	No problen	No problen	No problen
	Small probl	Big probler	No problen	No problen	Small probl	Big probler	Small probl	Small probl
	No problen	No problen	Small probl	Small probl	Small probl	No problen	No problen	No problen
No	No problen	No problen	Small probl	No problen	Small probl	No problen	No problen	Big probler
	Big probler	Big probler	Big probler	Big probler	Big probler	Big probler	Small probl	Small probl
No	No problen	No problen	No problen	No problen	Big probler	Big probler	No problen	No problen
	Small probl	No problen	Small probl	No problen	Small probl	Small probl	No problen	No problen
Yes	No problen	No problen	No problen	No problen	No problen	No problen	No problen	No problen
	Small probl	Small probl	Big probler	Small probl	Big probler	Small probl	Small probl	Small probl
No	No problen	No problen	Small probl	Small probl	Big probler	No problen	No problen	No problen
No	No problen	No problen	No problen	No problen	Small probl	No problen	No problen	Small probl
	No problen	Small probl	Small probl	Small probl	Small probl	Small probl	Small probl	Small probl
No	Small probl	No problen	No problen	No problen	No problen	No problen	No problen	No problen
	No problen	No problen	No problen	No problen	No problen	No problen	No problen	No problen
No	No problen	No problen	Big probler	Big probler	Big probler	No problen	No problen	No problen
No	Small probl	No problen	No problen	No problen	Small probl	No problen	No problen	Big probler
No	No problen	No problen	No problen	No problen	No problen	No problen	No problen	No problen
	No problen	No problen	No problen	No problen	No problen	No problen	No problen	No problen
No	No problen	No problen	No problen	No problen	Small probl	No problen	No problen	No problen
No	No problen	No problen	Small probl	No problen	Small probl	No problen	No problen	Big probler
	No problen	Small probl	No problen	No problen	Small probl	Small probl	No problen	No problen
	Small probl	Small probl	Small probl	Small probl	Small probl	Small probl	Small probl	Small probl
No	No problen	No problen	Small probl	Small probl	Big probler	No problen	No problen	Big probler
No	No problen	Small probl	Small probl	Small probl	Small probl	Small probl	Small probl	Small probl
	No problen	No problen	Big probler	Big probler	Big probler	No problen	No problen	No problen
	No problen	No problen	Small probl	Big probler	Big probler	No problen	No problen	No problen
	No problen	No problen	Small probl	No problen	Small probl	No problen	No problen	No problen
No	No problen	No problen	No problen	No problen	Small probl	Small probl	No problen	Small probl
	Small probl	Small probl	Small probl	Small probl	Small probl	Small probl	Small probl	Small probl
No	No problen	No problen	No problen	No problen	No problen	No problen	No problen	No problen
No	No problen	No problen	No problen	No problen	Big probler	No problen	No problen	Big probler
	Big probler	Big probler	Big probler	Big probler	Big probler	Small probl	Small probl	Small probl
	No problen	No problen	No problen	No problen	Small probl	No problen	No problen	No problen
Yes	Small probl	Small probl	Small probl	Small probl	Small probl	Small probl	Small probl	Small probl
	Small probl	Small probl	Big probler	No problen	Big probler	Big probler	No problen	Big probler
No	No problen	No problen	Small probl	Small probl	Small probl	No problen	No problen	Big probler
No	No problen	No problen	No problen	No problen	No problen	No problen	No problen	Big probler
No	No problen	No problen	No problen	No problen	No problen	No problen	No problen	No problen
	Small probl	Small probl	Small probl	Small probl	Small probl	Small probl	Small probl	Small probl

	Small probl	Small probl	Small probl	Small probl	Small probl	Small probl	Small probl	Small probl
	No problen	No problen	Big probler	Big probler	Big probler	No problen	No problen	No problen
No	No problen	No problen	No problen	No problen	No problen	No problen	No problen	No problen
No	No problen	No problen	Small probl	Small probl	Small probl	Small probl	No problen	Big probler
No	No problen	No problen	No problen	No problen	Small probl	No problen	No problen	No problen
	No problen	No problen	Big probler	No problen	Big probler	Small probl	No problen	No problen
No	No problen	No problen	No problen	Big probler	Big probler	No problen	No problen	Big probler
	Small probl	Small probl	Small probl	Small probl	Small probl	Small probl	Small probl	Small probl
No	Small probl	No problen	No problen	No problen	Small probl	No problen	No problen	Small probl
No	No problen	No problen	No problen	No problen	No problen	No problen	No problen	No problen
	No problen	No problen	No problen	No problen	No problen	No problen	No problen	No problen
	No problen	No problen	No problen	No problen	No problen	No problen	No problen	No problen
No	No problen	No problen	Small probl	No problen	Small probl	No problen	No problen	No problen
No	No problen	No problen	No problen	No problen	Small probl	Small probl	No problen	No problen
No	No problen	No problen	No problen	No problen	Small probl	No problen	No problen	No problen
No	Small probl	No problen	Small probl	No problen	Small probl	Small probl	No problen	Big probler
No	No problen	No problen	No problen	No problen	Small probl	No problen	No problen	No problen
	Small probl	Small probl	Small probl	Small probl	Small probl	No problen	No problen	No problen
No	No problen	No problen	No problen	No problen	No problen	No problen	No problen	No problen
	No problen	No problen	No problen	Small probl	Small probl	Small probl	No problen	Big probler
No	No problen	No problen	No problen	No problen	No problen	No problen	No problen	No problen
No	No problen	No problen	No problen	No problen	No problen	No problen	No problen	No problen
	No problen	No problen	No problen	No problen	Big probler	Big probler	No problen	No problen
	Big probler	Big probler	Big probler	Small probl	Small probl	Small probl	Small probl	Small probl
No	Small probl	No problen	No problen	No problen	No problen	No problen	No problen	Small probl
No	No problen	No problen	No problen	No problen	Small probl	No problen	No problen	No problen
No	No problen	No problen	No problen	No problen	Big probler	Small probl	No problen	No problen
No	No problen	No problen	No problen	No problen	No problen	No problen	No problen	No problen
No	Small probl	Small probl	Small probl	Small probl	Big probler	Big probler	Small probl	Small probl
	Small probl	Small probl	Small probl	Small probl	Small probl	Small probl	Small probl	Small probl
	No problen	No problen	Small probl	Small probl	Small probl	No problen	No problen	No problen
	Small probl	Small probl	Small probl	Small probl	Big probler	Big probler	Small probl	Small probl
	No problen	No problen	No problen	No problen	No problen	No problen	No problen	No problen
	Small probl	Small probl	Big probler	Big probler	Big probler	Small probl	Small probl	Small probl
	Small probl	Small probl	Small probl	Small probl	Small probl	Small probl	Small probl	Small probl
	No problen	No problen	No problen	No problen	Small probl	No problen	No problen	No problen
	Small probl	Small probl	Small probl	Small probl	Small probl	Small probl	Small probl	Small probl
No	No problen	No problen	Small probl	No problen	Small probl	Small probl	No problen	Big probler
	Small probl	No problen	Small probl	No problen	Small probl	No problen	No problen	No problen
	Small probl	Big probler	Big probler	Big probler	Big probler	Big probler	Small probl	Small probl
	No problen	No problen	Small probl	No problen	Small probl	No problen	No problen	No problen
No	No problen	No problen	No problen	Small probl	Big probler	No problen	No problen	Big probler
No	Big probler	Small probl	Big probler	Small probl	Small probl	Small probl	Small probl	Small probl
	No problen	No problen	Small probl	No problen	Big probler	Big probler	No problen	No problen
	Small probl	Small probl	Small probl	Small probl	Big probler	Big probler	Small probl	Small probl
No	Small probl	Big probler	No problen	No problen	Small probl	Big probler	No problen	Big probler
	No problen	No problen	Big probler	No problen	Small probl	No problen	No problen	No problen
	Small probl	Small probl	Small probl	Small probl	Big probler	Big probler	Small probl	Small probl
	No problen	No problen	Small probl	Small probl	Small probl	Small probl	Small probl	No problen
	Small probl	Small probl	Big probler	Small probl	Big probler	Small probl	Small probl	Small probl
	No problen	No problen	No problen	No problen	No problen	No problen	No problen	No problen
	No problen	No problen	No problen	No problen	Small probl	Small probl	No problen	No problen

For peer review only

	No problen	No problen	No problen	No problen	No problen	No problen	No problen	No problen
No	No problen	No problen	No problen	No problen	Small probl	No problen	No problen	Big probler
	No problen	No problen	No problen	No problen	Big probler	No problen	No problen	No problen
	No problen	No problen	No problen	No problen	No problen	No problen	No problen	No problen
No	No problen	No problen	No problen	No problen	Small probl	No problen	No problen	No problen
	Small probl	Small probl	Small probl	Small probl	Small probl	Small probl	Small probl	Small probl
	Small probl	Big probler	Big probler	Big probler	Big probler	Big probler	Big probler	Small probl
	Small probl	Small probl	Small probl	Small probl	Small probl	Small probl	Small probl	Small probl
	Small probl	Big probler	Big probler	Small probl	Small probl	Small probl	Small probl	Small probl
No	No problen	No problen	No problen	No problen	Big probler	No problen	No problen	No problen
No	No problen	No problen	Small probl	No problen	Small probl	No problen	No problen	No problen
	No problen	No problen	No problen	No problen	No problen	No problen	No problen	No problen
No	No problen	No problen	Small probl	Big probler	Big probler	No problen	No problen	No problen
	No problen	No problen	No problen	No problen	No problen	No problen	No problen	No problen
	No problen	No problen	No problen	No problen	Big probler	No problen	No problen	No problen
	No problen	No problen	No problen	No problen	Small probl	No problen	No problen	Big probler
	No problen	No problen	No problen	No problen	No problen	No problen	No problen	No problen
No	No problen	No problen	Small probl	Small probl	Big probler	No problen	No problen	No problen
	No problen	Small probl	No problen	No problen	Small probl	Small probl	Big probler	Big probler
	Small probl	Small probl	Small probl	Small probl	Big probler	Big probler	Small probl	Small probl
No	No problen	No problen	No problen	No problen	Small probl	No problen	No problen	No problen
No	No problen	No problen	No problen	No problen	No problen	No problen	No problen	No problen
	Small probl	Small probl	Small probl	Small probl	Small probl	Small probl	Small probl	Small probl
No	No problen	No problen	No problen	No problen	No problen	No problen	No problen	No problen
	No problen	No problen	Small probl	No problen	No problen	No problen	No problen	No problen
No	No problen	No problen	Big probler	Big probler	Big probler	No problen	No problen	No problen
No	No problen	No problen	Small probl	Small probl	Small probl	Small probl	No problen	No problen
	Small probl	Small probl	Small probl	Small probl	Small probl	Small probl	Small probl	Small probl
	Small probl	Small probl	Small probl	Small probl	Small probl	Small probl	Small probl	Small probl
	Small probl	Small probl	Small probl	Small probl	Small probl	Small probl	Small probl	Small probl
Yes	No problen	No problen	Small probl	No problen	No problen	No problen	Small probl	Small probl
No	No problen	Small probl	Small probl	Small probl	Small probl	Small probl	Small probl	Small probl
No	No problen	No problen	Big probler	No problen	No problen	No problen	No problen	No problen
Yes	No problen	Small probl	Small probl	Small probl	No problen	No problen	No problen	No problen
	Small probl	Small probl	Small probl	Small probl	Small probl	Small probl	Small probl	Small probl
Yes	No problen	No problen	Big probler	Big probler	Big probler	No problen	No problen	No problen
No	No problen	No problen	No problen	No problen	No problen	No problen	No problen	No problen
	No problen	No problen	Small probl	Small probl	No problen	No problen	No problen	No problen
Yes	No problen	No problen	Small probl	Small probl	Small probl	No problen	No problen	No problen
	No problen	No problen	No problen	No problen	Small probl	No problen	No problen	No problen
	Small probl	Small probl	Small probl	Small probl	Big probler	Small probl	Small probl	Small probl
	No problen	No problen	Big probler	Small probl	Small probl	No problen	No problen	Big probler
No	No problen	Small probl	Small probl	Small probl	Big probler	Small probl	Small probl	Small probl
No	No problen	No problen	No problen	No problen	No problen	No problen	No problen	No problen
No	No problen	No problen	Small probl	No problen	No problen	No problen	No problen	No problen
	Small probl	Small probl	Small probl	Small probl	Small probl	Small probl	Small probl	Small probl
	No problen	No problen	Small probl	No problen	No problen	No problen	No problen	Small probl
Yes	No problen	No problen	Small probl	No problen	No problen	No problen	No problen	No problen
No	No problen	No problen	Big probler	Small probl	Big probler	No problen	No problen	No problen
	Small probl	Small probl	Small probl	Small probl	Small probl	Small probl	Small probl	Small probl
No	No problen	No problen	Big probler	No problen	No problen	No problen	No problen	No problen
	No problen	No problen	No problen	No problen	No problen	No problen	Small probl	Small probl

For peer review only

No	No problen	No problen	Small probl	No problen	Small probl	No problen	No problen	Small probl
	Small probl	Small probl	Small probl	Small probl	Big probler	Small probl	Small probl	Small probl
No	No problen	No problen	Big probler	No problen	Big probler	No problen	No problen	Small probl
No	No problen	No problen	Small probl	No problen	Small probl	No problen	Small probl	Small probl
	Small probl	Small probl	Small probl	Small probl	Small probl	Small probl	Small probl	Small probl
	No problen	No problen	Small probl	No problen	Small probl	No problen	No problen	No problen
No	No problen	No problen	No problen	No problen	No problen	No problen	No problen	No problen
	Small probl	Small probl	Small probl	Small probl	Small probl	Small probl	Small probl	Small probl
No	No problen	No problen	Small probl	No problen	Small probl	No problen	No problen	No problen
No	No problen	No problen	Small probl	No problen	No problen	No problen	No problen	Small probl
	Small probl	Small probl	Small probl	Small probl	Small probl	Small probl	Small probl	Small probl
Yes	No problen	No problen	Small probl	Small probl	Small probl	No problen	No problen	Small probl
	Small probl	Small probl	Small probl	Small probl	Small probl	Small probl	Small probl	Small probl
	No problen	No problen	Small probl	No problen	Small probl	No problen	No problen	No problen
	No problen	Big probler	Small probl	No problen	Small probl	Small probl	No problen	No problen
	No problen	No problen	No problen	No problen	No problen	No problen	No problen	No problen
No	No problen	No problen	Small probl	No problen	No problen	No problen	Small probl	No problen
	No problen	No problen	No problen	No problen	No problen	No problen	No problen	No problen
No	No problen	No problen	Big probler	No problen	No problen	No problen	No problen	Big probler
No	No problen	No problen	Big probler	Big probler	Big probler	No problen	No problen	No problen
	No problen	No problen	Big probler	Small probl	Big probler	No problen	No problen	No problen
No	No problen	No problen	Small probl	Small probl	Small probl	No problen	No problen	No problen
No	No problen	No problen	Small probl	No problen	Small probl	No problen	No problen	Small probl
	Small probl	Small probl	Small probl	Small probl	Big probler	Small probl	Small probl	Small probl
Yes	No problen	No problen	No problen	No problen	No problen	No problen	Small probl	Small probl
No	No problen	No problen	No problen	No problen	Big probler	No problen	No problen	No problen
Yes	No problen	No problen	Small probl	Small probl	Small probl	Small probl	No problen	No problen
No	No problen	No problen	No problen	No problen	No problen	No problen	No problen	No problen
	No problen	No problen	Small probl	Small probl	Small probl	No problen	Small probl	Small probl
Yes	No problen	No problen	Small probl	Small probl	Small probl	Small probl	Small probl	Small probl
	Small probl	Small probl	Small probl	Small probl	Big probler	Big probler	Small probl	Small probl
	Small probl	Small probl	Small probl	Small probl	Big probler	Small probl	Small probl	Small probl
	No problen	No problen	Small probl	No problen	Small probl	No problen	No problen	Small probl
No	No problen	No problen	Small probl	Small probl	Small probl	No problen	No problen	Small probl
	No problen	Big probler	Big probler	No problen	Small probl	Big probler	No problen	Small probl
No	No problen	No problen	No problen	No problen	No problen	No problen	No problen	Small probl
No	No problen	No problen	No problen	No problen	No problen	No problen	No problen	No problen
	Small probl	Small probl	Small probl	Small probl	Small probl	Small probl	Small probl	Small probl
No	No problen	No problen	No problen	No problen	No problen	No problen	No problen	Big probler
	Small probl	Small probl	Small probl	Small probl	Small probl	Small probl	Small probl	Small probl
	No problen	No problen	No problen	No problen	No problen	No problen	No problen	No problen
Yes	Small probl	No problen	Small probl	Small probl	Small probl	Small probl	Small probl	Small probl
Yes	No problen	No problen	No problen	No problen	No problen	No problen	No problen	No problen
	Small probl	Small probl	Small probl	Small probl	Small probl	Small probl	Small probl	Small probl
Yes	No problen	No problen	No problen	No problen	Small probl	Small probl	No problen	Small probl
	No problen	No problen	No problen	No problen	No problen	No problen	No problen	No problen
	No problen	No problen	Big probler	Big probler	Big probler	No problen	No problen	No problen
No	No problen	No problen	Small probl	Small probl	Small probl	No problen	No problen	No problen
Yes	No problen	No problen	No problen	No problen	No problen	No problen	No problen	No problen
No	Small probl	No problen	No problen	No problen	Small probl	No problen	No problen	No problen
No	No problen	No problen	Big probler	No problen	Small probl	No problen	No problen	No problen
	No problen	No problen	No problen	No problen	Small probl	No problen	No problen	No problen

For peer review only

	Small probl	Small probl	Small probl	Small probl	Big probler	Small probl	Small probl	Small probl
	Small probl	Small probl	Small probl	Small probl	Big probler	Small probl	Small probl	Small probl
No	No problen	No problen	Small probl	Small probl	Big probler	No problen	No problen	No problen
	Small probl	Small probl	Small probl	Small probl	Small probl	Small probl	Small probl	Small probl
	No problen	No problen	No problen	No problen	No problen	No problen	No problen	No problen
	Small probl	Small probl	Small probl	Small probl	Small probl	Small probl	Small probl	Small probl
No	No problen	No problen	Small probl	No problen	No problen	No problen	No problen	No problen
No	No problen	No problen	Small probl	Small probl	No problen	No problen	No problen	No problen
	No problen	Small probl	Big probler	No problen	No problen	No problen	No problen	No problen
No	No problen	No problen	No problen	No problen	Small probl	No problen	No problen	No problen
No	No problen	No problen	No problen	No problen	No problen	No problen	No problen	No problen
No	No problen	No problen	Small probl	Small probl	Small probl	No problen	No problen	No problen
Yes	No problen	No problen	Small probl	No problen	No problen	No problen	No problen	No problen
	Small probl	Small probl	Small probl	Small probl	Small probl	Small probl	Small probl	Small probl
No	No problen	No problen	Small probl	No problen	No problen	No problen	No problen	No problen
No	No problen	No problen	Small probl	Small probl	Small probl	Small probl	Small probl	No problen
	Small probl	Small probl	Small probl	Small probl	Small probl	Small probl	Small probl	Small probl
	Small probl	Small probl	Small probl	Small probl	Big probler	Small probl	Small probl	Small probl
	No problen	No problen	Small probl	No problen	Small probl	No problen	No problen	Big probler
	Small probl	Small probl	Small probl	Small probl	Small probl	Small probl	Small probl	Small probl
	Small probl	Small probl	Small probl	Small probl	Small probl	Small probl	Small probl	Small probl
	No problen	No problen	No problen	No problen	No problen	No problen	No problen	No problen
	Small probl	Small probl	Small probl	Small probl	Small probl	Small probl	Small probl	Small probl
	Small probl	Small probl	Small probl	Small probl	Small probl	Small probl	Small probl	Small probl
	Small probl	Small probl	Small probl	Small probl	Small probl	Small probl	Small probl	Small probl
	Small probl	Small probl	Small probl	Small probl	Small probl	Small probl	Small probl	Small probl
	Small probl	Small probl	Small probl	Small probl	Big probler	Small probl	Small probl	Small probl
	Small probl	Small probl	Small probl	Small probl	Big probler	Small probl	Small probl	Small probl
	Small probl	Small probl	Big probler	Small probl	Small probl	Small probl	Small probl	Small probl
No	No problen	No problen	No problen	No problen	No problen	No problen	No problen	No problen
	No problen	Big probler	No problen	No problen	No problen	Big probler	No problen	No problen
	No problen	No problen	No problen	No problen	No problen	No problen	No problen	No problen
	Small probl	Small probl	Small probl	Small probl	Big probler	Small probl	Small probl	Small probl
	Small probl	Small probl	Small probl	Small probl	Small probl	Small probl	Small probl	Small probl
	Small probl	Small probl	Small probl	Small probl	Small probl	Small probl	Small probl	Small probl
	No problen	No problen	No problen	No problen	No problen	No problen	No problen	No problen
	Small probl	Small probl	Small probl	Small probl	Small probl	Small probl	Small probl	Small probl
	No problen	No problen	Small probl	Big probler	Big probler	No problen	Small probl	Small probl
	No problen	No problen	Small probl	No problen	Small probl	Small probl	No problen	Big probler
No	No problen	No problen	No problen	No problen	No problen	No problen	No problen	No problen
	Small probl	Small probl	Small probl	Small probl	Small probl	Small probl	Small probl	Small probl
	Small probl	Small probl	Small probl	Small probl	Small probl	Small probl	Small probl	Small probl
	No problen	No problen	No problen	No problen	No problen	No problen	No problen	No problen
	No problen	No problen	No problen	No problen	No problen	No problen	No problen	No problen
	No problen	No problen	Small probl	No problen	Small probl	No problen	No problen	No problen
	No problen	Big probler	Small probl	No problen	No problen	Big probler	No problen	Big probler
No	No problen	No problen	Small probl	Small probl	Small probl	No problen	No problen	No problen
	No problen	No problen	Small probl	No problen	Small probl	Small probl	No problen	Small probl
	Small probl	Small probl	Small probl	Small probl	Small probl	Small probl	Small probl	Small probl
	No problen	No problen	No problen	Big probler	Big probler	No problen	No problen	No problen
	No problen	No problen	Small probl	Small probl	Small probl	Small probl	No problen	No problen
	No problen	No problen	No problen	No problen	No problen	No problen	No problen	Big probler

For peer review only

No	No problen	No problen	No problen	No problen	No problen	No problen	No problen	No problen
	No problen	No problen	No problen	No problen	No problen	No problen	No problen	No problen
	No problen	No problen	No problen	No problen	No problen	No problen	No problen	No problen
	Small probl	Small probl	Small probl	Small probl	Small probl	Small probl	Small probl	Small probl
	No problen	Small probl	No problen	No problen	No problen	Small probl	No problen	Big probler
No	No problen	No problen	No problen	No problen	No problen	No problen	No problen	No problen
	No problen	No problen	Big probler	No problen	No problen	No problen	No problen	No problen
	No problen	Small probl	No problen	No problen	No problen	Big probler	No problen	Big probler
No	No problen	No problen	Small probl	No problen	Small probl	No problen	No problen	No problen
	No problen	No problen	Small probl	No problen	Small probl	No problen	No problen	No problen
	No problen	No problen	No problen	No problen	No problen	No problen	No problen	No problen
	No problen	No problen	No problen	No problen	No problen	No problen	No problen	No problen
	No problen	No problen	No problen	No problen	No problen	No problen	No problen	No problen
No	No problen	No problen	No problen	No problen	No problen	No problen	No problen	No problen
	Small probl	Small probl	Small probl	Small probl	Small probl	Small probl	Small probl	Small probl
	No problen	No problen	Small probl	No problen	Small probl	Small probl	No problen	Small probl
	No problen	No problen	No problen	No problen	No problen	No problen	No problen	No problen
	No problen	No problen	Big probler	Small probl	Small probl	Small probl	No problen	Big probler
	Small probl	Small probl	Small probl	Small probl	Small probl	Small probl	Small probl	Small probl
	Small probl	Small probl	Small probl	Small probl	Small probl	Small probl	Small probl	Small probl
	No problen	No problen	Big probler	No problen	No problen	No problen	No problen	No problen
No	No problen	No problen	Small probl	Small probl	Small probl	No problen	No problen	No problen
No	No problen	No problen	No problen	No problen	No problen	No problen	No problen	No problen
	No problen	No problen	Big probler	No problen	No problen	No problen	No problen	No problen
No	No problen	No problen	No problen	No problen	No problen	No problen	No problen	No problen
No	No problen	No problen	No problen	No problen	No problen	No problen	No problen	No problen
	No problen	Big probler	Big probler	No problen	Small probl	Big probler	No problen	No problen
	No problen	No problen	No problen	No problen	No problen	No problen	No problen	No problen
	No problen	Small probl	Small probl	No problen	No problen	Small probl	No problen	No problen
	Small probl	Small probl	Small probl	Small probl	Small probl	Small probl	Small probl	Small probl
	Small probl	Small probl	Small probl	Small probl	Big probler	Small probl	Small probl	Small probl
No	No problen	No problen	Small probl	Big probler	Big probler	No problen	No problen	Small probl
	Small probl	Small probl	Small probl	Small probl	Small probl	Small probl	Small probl	Small probl
	No problen	No problen	No problen	No problen	No problen	No problen	No problen	No problen
	Small probl	Small probl	Small probl	Small probl	Small probl	Small probl	Small probl	Small probl
	No problen	Big probler	Small probl	No problen	No problen	Big probler	No problen	No problen
	Small probl	No problen	Big probler	No problen	No problen	No problen	No problen	No problen
	No problen	Small probl	Small probl	No problen	Small probl	Small probl	No problen	Big probler
	Small probl	Small probl	Small probl	Small probl	Big probler	Small probl	Small probl	Small probl
	Small probl	Small probl	Small probl	Small probl	Small probl	Small probl	Small probl	Small probl
	No problen	No problen	Big probler	No problen	No problen	No problen	No problen	Big probler
No	No problen	No problen	No problen	No problen	No problen	No problen	No problen	No problen
	No problen	No problen	No problen	No problen	No problen	No problen	No problen	No problen
	Small probl	Small probl	Small probl	Small probl	Small probl	Small probl	Small probl	Small probl
	No problen	No problen	Big probler	No problen	Small probl	Small probl	No problen	No problen
	Small probl	Small probl	Small probl	Small probl	Small probl	Small probl	Small probl	Small probl
	Small probl	Small probl	Small probl	Small probl	Small probl	Small probl	Small probl	Small probl
	Small probl	Small probl	Small probl	Small probl	Big probler	Big probler	Small probl	Small probl
No	No problen	No problen	No problen	No problen	No problen	No problen	No problen	No problen
	No problen	No problen	No problen	No problen	No problen	No problen	No problen	No problen
	Small probl	Small probl	Small probl	Small probl	Small probl	Small probl	Small probl	Small probl
	Small probl	Small probl	Big probler	Small probl	Big probler	Small probl	Small probl	Small probl

	Small probl	Small probl	Small probl	Small probl	Small probl	Small probl	Small probl	Small probl
	Small probl	Small probl	Small probl	Small probl	Big probler	Small probl	Small probl	Small probl
No	No problen	No problen	No problen	No problen	No problen	No problen	No problen	No problen
	Small probl	Small probl	Small probl	Small probl	Small probl	Small probl	Small probl	Small probl
No	No problen	No problen	Small probl	Small probl	Big probler	No problen	No problen	No problen
	No problen	No problen	No problen	No problen	No problen	No problen	No problen	No problen
	No problen	No problen	Small probl	Big probler	Big probler	No problen	No problen	No problen
No	No problen	Small probl	Big probler	No problen	No problen	Big probler	No problen	Big probler
	No problen	No problen	No problen	No problen	No problen	No problen	No problen	No problen
	Small probl	Small probl	Small probl	Small probl	Big probler	Small probl	Small probl	Small probl
	Small probl	Small probl	Small probl	Small probl	Small probl	Small probl	Small probl	Small probl
	No problen	No problen	Big probler	No problen	No problen	No problen	No problen	Big probler
No	No problen	No problen	No problen	No problen	No problen	No problen	No problen	No problen
	No problen	No problen	Small probl	No problen	Small probl	Small probl	No problen	Big probler
	No problen	Small probl	No problen	Small probl	Small probl	Small probl	No problen	Small probl
	No problen	No problen	No problen	No problen	No problen	No problen	No problen	No problen
	No problen	No problen	Small probl	Small probl	Small probl	No problen	No problen	Small probl
	Small probl	Small probl	Small probl	Small probl	Small probl	Small probl	Small probl	Small probl
No	No problen	No problen	No problen	No problen	No problen	No problen	No problen	No problen
	No problen	Big probler	Big probler	No problen	Small probl	Big probler	No problen	Big probler
	Small probl	Small probl	Small probl	Small probl	Small probl	Small probl	Small probl	Small probl
	No problen	No problen	Small probl	Small probl	Small probl	No problen	No problen	No problen
No	No problen	No problen	No problen	No problen	No problen	No problen	No problen	No problen
No	No problen	No problen	Small probl	Small probl	Small probl	No problen	No problen	No problen
No	No problen	No problen	No problen	Small probl	Small probl	Small probl	Small probl	Small probl
	No problen	No problen	Small probl	No problen	No problen	No problen	No problen	No problen
	No problen	No problen	No problen	No problen	No problen	No problen	No problen	Big probler
	Small probl	Small probl	Small probl	Small probl	Small probl	Small probl	Small probl	Small probl
	No problen	No problen	No problen	No problen	No problen	No problen	No problen	No problen
Yes	No problen	No problen	No problen	No problen	No problen	No problen	No problen	No problen
	No problen	No problen	No problen	No problen	Big probler	No problen	No problen	No problen
	No problen	No problen	No problen	No problen	Big probler	No problen	No problen	No problen
	No problen	No problen	No problen	No problen	Big probler	No problen	No problen	No problen
	No problen	No problen	No problen	No problen	Big probler	No problen	No problen	No problen
	No problen	No problen	No problen	No problen	Big probler	No problen	No problen	No problen
	No problen	No problen	No problen	No problen	No problen	No problen	No problen	No problen
	No problen	No problen	No problen	No problen	Big probler	No problen	No problen	No problen
	No problen	No problen	No problen	No problen	No problen	No problen	No problen	No problen
	No problen	No problen	No problen	No problen	Big probler	No problen	No problen	No problen
	No problen	No problen	No problen	No problen	Big probler	No problen	No problen	No problen
	No problen	No problen	No problen	No problen	Big probler	No problen	No problen	No problen
	No problen	No problen	No problen	No problen	Big probler	No problen	No problen	No problen
	No problen	No problen	No problen	No problen	Big probler	No problen	No problen	No problen
	No problen	No problen	No problen	No problen	Big probler	No problen	No problen	No problen
	Small probl	Small probl	Small probl	No problen	Big probler	No problen	No problen	No problen
	No problen	No problen	No problen	No problen	Big probler	No problen	No problen	No problen
	No problen	No problen	No problen	No problen	Big probler	No problen	No problen	No problen
	No problen	No problen	No problen	No problen	Big probler	No problen	No problen	No problen
	No problen	No problen	No problen	No problen	No problen	No problen	No problen	No problen
	No problen	No problen	No problen	No problen	Big probler	No problen	No problen	No problen
	No problen	No problen	No problen	No problen	Big probler	No problen	No problen	No problen
No	No problen	No problen	No problen	No problen	No problen	No problen	No problen	No problen

For peer review only

	No problen	No problen	No problen	No problen	Big probler	No problen	No problen	No problen
	No problen	No problen	No problen	No problen	Big probler	No problen	No problen	No problen
	No problen	No problen	No problen	No problen	Big probler	No problen	No problen	No problen
	No problen	No problen	Small probl	No problen	No problen	No problen	No problen	No problen
	No problen	No problen	No problen	No problen	Big probler	No problen	No problen	No problen
	No problen	No problen	No problen	No problen	Big probler	No problen	No problen	No problen
	No problen	No problen	No problen	No problen	No problen	No problen	No problen	No problen
	No problen	No problen	No problen	No problen	Big probler	No problen	No problen	No problen
	No problen	No problen	No problen	No problen	No problen	No problen	No problen	No problen
	No problen	No problen	No problen	No problen	Big probler	No problen	No problen	No problen
	No problen	No problen	No problen	No problen	Big probler	No problen	No problen	No problen
	No problen	No problen	No problen	No problen	No problen	No problen	No problen	No problen
	No problen	No problen	No problen	No problen	Big probler	No problen	No problen	No problen
No	No problen	No problen	No problen	No problen	No problen	No problen	No problen	No problen
	No problen	No problen	No problen	No problen	Big probler	No problen	No problen	No problen
No	No problen	No problen	No problen	No problen	No problen	No problen	No problen	No problen
	No problen	No problen	No problen	No problen	Big probler	No problen	No problen	No problen
	No problen	No problen	No problen	No problen	Big probler	No problen	No problen	No problen
	No problen	No problen	No problen	No problen	Big probler	No problen	No problen	No problen
	No problen	No problen	No problen	No problen	No problen	No problen	No problen	No problen
	No problen	No problen	No problen	No problen	No problen	No problen	No problen	No problen
	No problen	No problen	No problen	No problen	Big probler	No problen	No problen	No problen
No	No problen	No problen	No problen	No problen	Big probler	No problen	No problen	No problen
	No problen	No problen	No problen	No problen	Small probl	No problen	No problen	No problen
	No problen	No problen	No problen	No problen	Big probler	No problen	No problen	No problen
	No problen	No problen	No problen	No problen	Big probler	No problen	No problen	No problen
	No problen	No problen	No problen	No problen	Big probler	No problen	No problen	No problen
	No problen	No problen	No problen	No problen	Small probl	No problen	No problen	No problen
	No problen	No problen	No problen	No problen	Big probler	No problen	No problen	No problen
	No problen	No problen	No problen	No problen	No problen	No problen	No problen	No problen
	No problen	No problen	No problen	No problen	No problen	No problen	No problen	No problen
	No problen	No problen	No problen	No problen	Big probler	No problen	No problen	No problen
	No problen	No problen	No problen	No problen	Small probl	No problen	No problen	No problen
	No problen	No problen	No problen	No problen	Big probler	No problen	No problen	No problen
	No problen	No problen	No problen	No problen	No problen	No problen	No problen	No problen
	No problen	No problen	No problen	No problen	No problen	No problen	No problen	No problen
	No problen	No problen	No problen	No problen	No problen	No problen	No problen	No problen
	No problen	No problen	No problen	No problen	No problen	No problen	No problen	No problen
	No problen	No problen	No problen	No problen	No problen	No problen	Small probl	No problen
	No problen	No problen	No problen	No problen	No problen	No problen	No problen	No problen
	No problen	No problen	No problen	No problen	Big probler	No problen	No problen	No problen
	No problen	No problen	No problen	No problen	Big probler	No problen	No problen	No problen
	No problen	No problen	No problen	No problen	No problen	No problen	No problen	No problen
	No problen	No problen	No problen	No problen	Big probler	No problen	No problen	No problen
	No problen	No problen	No problen	No problen	No problen	No problen	No problen	No problen
	No problen	No problen	No problen	No problen	Big probler	No problen	No problen	No problen
	No problen	No problen	No problen	No problen	No problen	No problen	No problen	No problen
No	No problen	No problen	No problen	No problen	Big probler	No problen	No problen	No problen
	No problen	No problen	No problen	No problen	No problen	No problen	No problen	No problen
	No problen	No problen	No problen	No problen	Small probl	No problen	No problen	No problen
	No problen	No problen	No problen	No problen	Big probler	No problen	No problen	No problen
	No problen	No problen	No problen	No problen	Big probler	No problen	No problen	No problen

	No problen	No problen	No problen	No problen	Small probl	No problen	No problen	No problen
	No problen	No problen	No problen	No problen	Big probler	No problen	No problen	No problen
	No problen	No problen	No problen	No problen	No problen	No problen	No problen	No problen
	No problen	No problen	No problen	No problen	Big probler	No problen	No problen	No problen
	No problen	No problen	No problen	No problen	No problen	No problen	No problen	No problen
	No problen	No problen	No problen	No problen	No problen	No problen	No problen	No problen
	No problen	No problen	No problen	No problen	No problen	No problen	No problen	No problen
	No problen	No problen	No problen	No problen	No problen	No problen	No problen	No problen
No	No problen	No problen	No problen	Big probler	Big probler	No problen	No problen	No problen
	No problen	No problen	No problen	No problen	No problen	No problen	No problen	No problen
Yes	No problen	No problen	No problen	Small probl	Big probler	No problen	No problen	No problen
	Small probl	Small probl	Small probl	Big probler	Big probler	Big probler	Small probl	Small probl
No	No problen	No problen	Small probl	Big probler	Big probler	No problen	No problen	No problen
	No problen	No problen	No problen	No problen	No problen	No problen	No problen	No problen
No	No problen	No problen	No problen	No problen	No problen	No problen	No problen	No problen
	No problen	No problen	No problen	No problen	No problen	No problen	No problen	No problen
	Small probl	Small probl	Small probl	Big probler	Big probler	Big probler	Small probl	Small probl
	Small probl	Small probl	Small probl	Big probler	Big probler	Small probl	Small probl	Small probl
	Small probl	Small probl	Small probl	Big probler	Big probler	Big probler	Small probl	Small probl
	No problen	No problen	No problen	Big probler	Big probler	No problen	No problen	No problen
No	No problen	No problen	No problen	Big probler	Big probler	No problen	No problen	No problen
No	No problen	No problen	Small probl	Big probler	Big probler	Small probl	No problen	No problen
	No problen	No problen	No problen	No problen	No problen	No problen	No problen	No problen
	Small probl	Small probl	Small probl	Big probler	Big probler	Small probl	Small probl	Small probl
	No problen	No problen	No problen	No problen	No problen	No problen	No problen	No problen
	No problen	No problen	No problen	No problen	No problen	No problen	No problen	No problen
	Small probl	Small probl	Small probl	Big probler	Big probler	Big probler	Small probl	Small probl
	No problen	No problen	No problen	No problen	No problen	No problen	No problen	No problen
	No problen	No problen	No problen	No problen	No problen	No problen	No problen	No problen
	Small probl	Small probl	Small probl	Big probler	Big probler	Big probler	Small probl	Small probl
No	No problen	No problen	No problen	Big probler	Big probler	No problen	No problen	No problen
	No problen	No problen	No problen	Big probler	Big probler	No problen	No problen	No problen
	No problen	No problen	No problen	No problen	No problen	No problen	No problen	No problen
	No problen	No problen	No problen	No problen	Small probl	No problen	No problen	No problen
	No problen	No problen	No problen	No problen	No problen	No problen	No problen	No problen
	No problen	No problen	No problen	Big probler	Big probler	No problen	No problen	No problen
	No problen	No problen	No problen	No problen	No problen	No problen	No problen	No problen
Yes	No problen	No problen	No problen	Big probler	Big probler	No problen	No problen	No problen
	Small probl	Small probl	Small probl	Big probler	Big probler	Small probl	Small probl	Small probl
	Big probler	Small probl	Small probl	Big probler	Big probler	Small probl	Small probl	Small probl
No	No problen	No problen	No problen	Small probl	Big probler	No problen	No problen	No problen
	Small probl	Small probl	Small probl	Big probler	Big probler	Big probler	Small probl	Small probl
	No problen	No problen	No problen	No problen	No problen	No problen	No problen	No problen
No	No problen	No problen	No problen	Big probler	Big probler	No problen	No problen	No problen
	No problen	No problen	No problen	Small probl	Small probl	No problen	No problen	No problen
	Small probl	Small probl	Small probl	Big probler	Big probler	Small probl	Small probl	Small probl
No	No problen	No problen	No problen	Small probl	Big probler	No problen	No problen	No problen
	Small probl	Small probl	Small probl	Big probler	Big probler	Big probler	Small probl	Small probl
	Small probl	Small probl	Small probl	Big probler	Big probler	Small probl	Small probl	Small probl
No	No problen	No problen	No problen	Small probl	Big probler	No problen	No problen	No problen
No	No problen	No problen	No problen	Big probler	Big probler	No problen	No problen	No problen
	Small probl	Small probl	Small probl	Big probler	Big probler	Small probl	Small probl	Small probl

For peer review only

	No problen	No problen	No problen	No problen	No problen	No problen	No problen	No problen
No	No problen	No problen	No problen	Big probler	Big probler	No problen	No problen	No problen
No	No problen	No problen	No problen	Small probl	Big probler	No problen	No problen	No problen
	No problen	No problen	Small probl	Big probler	Big probler	No problen	No problen	No problen
No	No problen	No problen	No problen	No problen	No problen	No problen	No problen	No problen
No	No problen	No problen	No problen	Big probler	Small probl	No problen	No problen	No problen
	No problen	No problen	No problen	No problen	Small probl	No problen	No problen	No problen
	No problen	No problen	No problen	Big probler	Big probler	No problen	No problen	No problen
	Small probl	Small probl	Small probl	Big probler	Big probler	Big probler	Small probl	Small probl
	No problen	No problen	No problen	No problen	No problen	No problen	No problen	No problen
	No problen	No problen	No problen	No problen	No problen	No problen	No problen	No problen
	No problen	No problen	Small probl	Big probler	Big probler	Small probl	Small probl	Small probl
	No problen	No problen	No problen	No problen	No problen	No problen	No problen	No problen
	No problen	No problen	No problen	No problen	No problen	No problen	No problen	No problen
	No problen	No problen	No problen	Small probl	Big probler	No problen	No problen	No problen
	No problen	No problen	No problen	No problen	No problen	No problen	No problen	No problen
	No problen	No problen	No problen	No problen	No problen	No problen	No problen	No problen
	No problen	No problen	Big probler	Big probler	Big probler	Big probler	No problen	Big probler
	No problen	No problen	No problen	No problen	No problen	No problen	No problen	No problen
	Small probl	Small probl	Small probl	Small probl	Small probl	Small probl	Small probl	Small probl
	No problen	No problen	No problen	No problen	No problen	No problen	Small probl	Big probler
	Small probl	No problen	Big probler	Small probl	Small probl	Small probl	Small probl	Small probl
	No problen	No problen	No problen	No problen	No problen	No problen	Small probl	Big probler
	No problen	No problen	No problen	No problen	No problen	No problen	Small probl	Big probler
	No problen	No problen	No problen	Small probl	No problen	No problen	Small probl	Small probl
	No problen	No problen	Big probler	Big probler	Big probler	Big probler	No problen	Big probler
No	No problen	No problen	Small probl	Small probl	No problen	Big probler	No problen	No problen
	Small probl	No problen	Big probler	Big probler	Small probl	No problen	No problen	Big probler
	No problen	No problen	No problen	No problen	No problen	No problen	Small probl	Small probl
No	Small probl	No problen	Big probler	Big probler	Big probler	No problen	No problen	No problen
No	No problen	No problen	No problen	No problen	No problen	No problen	Small probl	Small probl
	No problen	No problen	No problen	No problen	No problen	No problen	No problen	No problen
	No problen	No problen	Small probl	Small probl	No problen	No problen	No problen	Big probler
	No problen	No problen	No problen	No problen	No problen	No problen	Small probl	Big probler
	No problen	No problen	No problen	No problen	No problen	No problen	No problen	No problen
	No problen	No problen	No problen	No problen	No problen	No problen	Small probl	Small probl
	Small probl	No problen	Big probler	Small probl	Big probler	Small probl	Big probler	Big probler
	No problen	No problen	No problen	No problen	No problen	No problen	Big probler	Big probler
	No problen	No problen	No problen	No problen	No problen	No problen	No problen	No problen
	No problen	No problen	No problen	No problen	No problen	No problen	Small probl	Small probl
	No problen	No problen	No problen	No problen	No problen	No problen	Small probl	Small probl
	No problen	No problen	No problen	No problen	No problen	No problen	No problen	Small probl
	No problen	No problen	No problen	Small probl	No problen	No problen	Small probl	Small probl
	No problen	No problen	Small probl	Big probler	Big probler	No problen	No problen	Big probler
	No problen	No problen	Big probler	Small probl	Small probl	Small probl	Small probl	Small probl
No	Small probl	No problen	Big probler	Big probler	Big probler	Small probl	Small probl	Small probl
	No problen	No problen	No problen	Small probl	No problen	No problen	Small probl	Small probl
	No problen	No problen	Small probl	No problen	Small probl	Small probl	Big probler	Big probler
	No problen	No problen	Big probler	Small probl	Small probl	Small probl	Small probl	Small probl
	Small probl	No problen	Big probler	Big probler	Big probler	Small probl	Big probler	Big probler
	No problen	No problen	Small probl	No problen	No problen	Small probl	No problen	No problen
	Small probl	Small probl	Big probler	Small probl	Small probl	Small probl	Small probl	Small probl

For peer review only

	No problen	No problen	No problen	No problen	No problen	No problen	No problen	No problen
	No problen	No problen	Big probler	Small probl	Big probler	Big probler	No problen	No problen
	No problen	No problen	No problen	No problen	No problen	No problen	No problen	No problen
	No problen	No problen	No problen	Small probl	Small probl	Small probl	Small probl	Small probl
	No problen	No problen	No problen	No problen	No problen	No problen	Small probl	Big probler
No	Small probl	No problen	Big probler	Small probl	Small probl	Small probl	Small probl	Small probl
	No problen	No problen	No problen	No problen	No problen	No problen	No problen	No problen
No	Small probl	Small probl	Big probler	Small probl	Big probler	Big probler	Big probler	Big probler
	Small probl	No problen	Big probler	Big probler	Big probler	Small probl	Small probl	Small probl
	No problen	No problen	Big probler	Small probl	Big probler	Big probler	No problen	No problen
	No problen	No problen	Big probler	Small probl	Small probl	Small probl	Small probl	Big probler
	No problen	No problen	No problen	No problen	No problen	No problen	No problen	No problen
	No problen	No problen	No problen	Small probl	No problen	No problen	Small probl	Small probl
	No problen	No problen	No problen	No problen	No problen	No problen	Small probl	Small probl
	No problen	No problen	Big probler	Big probler	Big probler	Big probler	No problen	Big probler
	No problen	No problen	No problen	No problen	No problen	No problen	Big probler	Big probler
	No problen	No problen	No problen	No problen	No problen	No problen	No problen	Big probler
	No problen	No problen	No problen	No problen	No problen	No problen	No problen	Small probl
	Small probl	Small probl	Big probler	Big probler	Big probler	Big probler	Big probler	Big probler
	No problen	No problen	No problen	Small probl	Small probl	No problen	No problen	Small probl
	No problen	No problen	No problen	No problen	No problen	No problen	Big probler	Big probler
	Small probl	Small probl	Big probler	Big probler	Big probler	Small probl	Small probl	Small probl
	Small probl	Small probl	Small probl	Small probl	Small probl	Small probl	Small probl	Small probl
	Small probl	Small probl	Big probler	Big probler	Big probler	Small probl	Small probl	No problen
	No problen	No problen	No problen	No problen	No problen	No problen	No problen	No problen
	No problen	No problen	Small probl	No problen	No problen	No problen	No problen	No problen
	No problen	No problen	No problen	No problen	No problen	No problen	No problen	No problen
	No problen	No problen	Big probler	No problen	No problen	No problen	No problen	No problen
	No problen	No problen	No problen	No problen	No problen	No problen	No problen	No problen
No	No problen	No problen	No problen	No problen	No problen	No problen	No problen	No problen
	Small probl	Small probl	Small probl	Small probl	Small probl	Small probl	Small probl	Small probl
	No problen	No problen	Big probler	Big probler	Big probler	Big probler	No problen	No problen
	No problen	No problen	No problen	No problen	No problen	No problen	No problen	No problen
	No problen	No problen	No problen	Big probler	Big probler	Big probler	No problen	No problen
	No problen	No problen	No problen	No problen	No problen	No problen	No problen	No problen
No	No problen	No problen	Small probl	No problen	No problen	No problen	No problen	No problen
	Small probl	No problen	No problen	No problen	Big probler	No problen	No problen	No problen
No	No problen	No problen	No problen	No problen	No problen	No problen	No problen	No problen
	No problen	No problen	No problen	No problen	No problen	No problen	No problen	No problen
	Small probl	Small probl	Small probl	Small probl	Small probl	Small probl	Small probl	Small probl
	No problen	No problen	No problen	Small probl	Small probl	No problen	No problen	No problen
	No problen	No problen	Small probl	No problen	Small probl	No problen	No problen	No problen
	No problen	No problen	No problen	No problen	No problen	No problen	No problen	No problen
No	No problen	No problen	Small probl	No problen	Small probl	No problen	No problen	No problen
	No problen	No problen	No problen	Big probler	Big probler	No problen	No problen	No problen
	No problen	No problen	No problen	No problen	No problen	No problen	No problen	No problen
	Small probl	Small probl	Small probl	Small probl	Small probl	Small probl	Small probl	Small probl
	No problen	No problen	No problen	Small probl	Small probl	No problen	No problen	No problen
	No problen	No problen	No problen	No problen	No problen	No problen	No problen	No problen
	No problen	No problen	Small probl	No problen	No problen	No problen	No problen	No problen
	No problen	No problen	No problen	Small probl	Small probl	Small probl	No problen	No problen
	No problen	No problen	Big probler	Big probler	Big probler	No problen	No problen	No problen

For peer review only

	No problen	No problen	No problen	No problen	No problen	No problen	No problen	No problen
	Small probl	Small probl	Small probl	Small probl	Small probl	Small probl	Small probl	Small probl
	No problen	No problen	No problen	No problen	No problen	No problen	No problen	No problen
	Small probl	Small probl	Small probl	Small probl	Small probl	Small probl	Small probl	Small probl
	No problen	No problen	No problen	Small probl	Small probl	Small probl	No problen	No problen
	No problen	No problen	No problen	No problen	No problen	No problen	No problen	No problen
No	No problen	No problen	No problen	No problen	No problen	No problen	No problen	No problen
No	No problen	No problen	No problen	No problen	No problen	No problen	No problen	No problen
	Small probl	Small probl	Small probl	Small probl	Small probl	Small probl	Small probl	Small probl
	Small probl	Small probl	Small probl	Small probl	Small probl	Small probl	Small probl	Small probl
No	No problen	No problen	Small probl	No problen	No problen	No problen	No problen	No problen
	Small probl	Small probl	Small probl	Small probl	Small probl	Small probl	Small probl	Small probl
	No problen	No problen	No problen	No problen	Small probl	No problen	No problen	No problen
	No problen	No problen	No problen	Big probler	Big probler	Big probler	No problen	No problen
	No problen	No problen	Big probler	No problen	No problen	No problen	No problen	No problen
	Small probl	Small probl	Small probl	Small probl	Small probl	Small probl	Small probl	Small probl
	No problen	No problen	Big probler	No problen	No problen	No problen	No problen	No problen
	No problen	No problen	No problen	Small probl	Small probl	No problen	No problen	No problen
No	No problen	No problen	Small probl	No problen	Small probl	No problen	No problen	No problen
Yes	No problen	No problen	No problen	No problen	Small probl	No problen	No problen	No problen
	No problen	No problen	No problen	No problen	No problen	No problen	No problen	No problen
Yes	No problen	No problen	No problen	No problen	No problen	No problen	No problen	No problen
	No problen	No problen	No problen	Big probler	Big probler	No problen	No problen	No problen
	Small probl	Small probl	Small probl	Small probl	Small probl	Small probl	Small probl	Small probl
	No problen	No problen	No problen	Big probler	Big probler	Big probler	No problen	No problen
	Small probl	Small probl	Small probl	Small probl	Small probl	Small probl	Small probl	Small probl
	Small probl	Small probl	Big probler	Big probler	Big probler	Small probl	Small probl	Small probl
	No problen	No problen	Big probler	Big probler	Big probler	Small probl	Small probl	Small probl
	No problen	No problen	No problen	No problen	No problen	No problen	No problen	No problen
	No problen	No problen	Big probler	No problen	No problen	No problen	No problen	No problen
	No problen	No problen	No problen	No problen	No problen	No problen	No problen	No problen
	No problen	Small probl	Big probler	Big probler	Big probler	Small probl	Small probl	Small probl
	No problen	No problen	Big probler	No problen	Small probl	No problen	No problen	No problen
	No problen	No problen	No problen	Big probler	No problen	No problen	No problen	No problen
	No problen	No problen	No problen	No problen	No problen	No problen	No problen	No problen
	No problen	No problen	No problen	No problen	No problen	No problen	No problen	No problen
No	No problen	No problen	Small probl	No problen	Small probl	No problen	No problen	No problen
	Small probl	Small probl	Small probl	Small probl	Small probl	Small probl	Small probl	Small probl
	No problen	No problen	Small probl	No problen	No problen	No problen	No problen	No problen
	No problen	No problen	Big probler	No problen	No problen	No problen	No problen	No problen
	No problen	No problen	No problen	Small probl	Small probl	No problen	No problen	No problen
	Small probl	No problen	Small probl	Big probler	Big probler	Small probl	Small probl	No problen
	No problen	No problen	No problen	No problen	No problen	No problen	No problen	No problen
	No problen	No problen	No problen	No problen	No problen	No problen	No problen	No problen
	No problen	No problen	Big probler	No problen	No problen	No problen	No problen	Small probl
	No problen	No problen	No problen	No problen	No problen	No problen	No problen	No problen
	Small probl	Small probl	Small probl	Small probl	Small probl	Small probl	Small probl	Small probl
Yes	No problen	No problen	No problen	No problen	No problen	No problen	No problen	No problen
Yes	No problen	No problen	Small probl	No problen	No problen	No problen	No problen	No problen
	Small probl	Small probl	Small probl	Small probl	Small probl	Small probl	Small probl	Small probl
No	No problen	No problen	No problen	No problen	No problen	No problen	No problen	No problen
No	No problen	No problen	Small probl	No problen	Small probl	No problen	No problen	No problen

For peer review only

	No problen	No problen	No problen	No problen	No problen	No problen	No problen	No problen
	Small probl	No problen	Big probler	Big probler	Big probler	Small probl	No problen	No problen
	No problen	No problen	No problen	Small probl	Small probl	No problen	No problen	No problen
	No problen	No problen	No problen	Big probler	Big probler	No problen	No problen	No problen
	No problen	No problen	Small probl	No problen	No problen	No problen	No problen	No problen
	No problen	No problen	No problen	No problen	No problen	No problen	No problen	No problen
No	Small probl	No problen	Small probl	Big probler	Big probler	Small probl	Small probl	Small probl
	Small probl	Small probl	Small probl	Big probler	Big probler	Small probl	Small probl	Small probl
	No problen	No problen	No problen	Big probler	Big probler	Big probler	No problen	No problen
	No problen	No problen	No problen	No problen	No problen	No problen	No problen	No problen
	No problen	Small probl	Big probler	Big probler	Big probler	Small probl	No problen	No problen
Yes	No problen	No problen	No problen	No problen	No problen	No problen	No problen	No problen
	No problen	No problen	No problen	No problen	No problen	No problen	No problen	No problen
	No problen	No problen	No problen	No problen	No problen	No problen	No problen	No problen
No	No problen	No problen	Small probl	Small probl	Small probl	No problen	No problen	No problen
	No problen	No problen	No problen	No problen	No problen	No problen	No problen	No problen
	No problen	No problen	No problen	No problen	No problen	No problen	No problen	No problen
	No problen	No problen	No problen	No problen	Big probler	No problen	No problen	No problen
	No problen	No problen	No problen	Big probler	Big probler	Big probler	No problen	No problen
Yes	No problen	No problen	No problen	No problen	No problen	No problen	No problen	No problen
	No problen	No problen	No problen	No problen	No problen	No problen	No problen	No problen
No	No problen	No problen	Small probl	No problen	No problen	No problen	No problen	No problen
	No problen	No problen	No problen	No problen	No problen	No problen	No problen	No problen
	Small probl	Small probl	Small probl	Small probl	Small probl	Small probl	Small probl	Small probl
	No problen	No problen	No problen	Small probl	Big probler	No problen	No problen	No problen
	No problen	No problen	No problen	No problen	Big probler	No problen	No problen	No problen
No	No problen	No problen	No problen	No problen	No problen	No problen	No problen	No problen
Yes	No problen	No problen	No problen	No problen	No problen	No problen	No problen	No problen
	No problen	No problen	No problen	No problen	Big probler	No problen	No problen	No problen
	No problen	No problen	No problen	Small probl	Small probl	No problen	No problen	No problen
	No problen	No problen	No problen	No problen	No problen	No problen	No problen	No problen
	No problen	No problen	No problen	No problen	Small probl	No problen	No problen	No problen
	Small probl	Small probl	Small probl	Small probl	Small probl	Small probl	Small probl	Small probl
	No problen	No problen	No problen	No problen	No problen	No problen	No problen	No problen
	No problen	No problen	No problen	No problen	Small probl	No problen	No problen	No problen
No	No problen	No problen	No problen	Big probler	Big probler	No problen	No problen	No problen
	Small probl	Small probl	Big probler	Big probler	Big probler	Small probl	Small probl	Small probl
	No problen	No problen	No problen	No problen	No problen	No problen	No problen	No problen
	Small probl	Small probl	Big probler	Big probler	Big probler	Big probler	Small probl	Small probl
	Small probl	Small probl	Big probler	Big probler	Big probler	Small probl	Small probl	Small probl
	No problen	No problen	Small probl	No problen	Small probl	No problen	No problen	No problen
No	No problen	No problen	No problen	No problen	Small probl	No problen	No problen	No problen
	No problen	No problen	No problen	No problen	No problen	No problen	No problen	No problen
	No problen	No problen	No problen	No problen	No problen	No problen	No problen	No problen
	No problen	No problen	Small probl	No problen	No problen	No problen	No problen	No problen
	Small probl	Small probl	Small probl	Big probler	Big probler	Small probl	Small probl	Small probl
	No problen	No problen	No problen	No problen	No problen	No problen	No problen	No problen
	Small probl	Big probler	Big probler	Big probler	Big probler	Small probl	Small probl	Small probl
	Small probl	Small probl	Big probler	Big probler	Big probler	Small probl	Small probl	Small probl
	No problen	No problen	No problen	No problen	No problen	No problen	No problen	No problen
	Small probl	Small probl	Small probl	Big probler	Big probler	Small probl	Small probl	Small probl
	Small probl	Small probl	Big probler	Small probl	Small probl	Small probl	Small probl	Small probl

For peer review only

	No problen	No problen	No problen	No problen	Small probl	No problen	No problen	No problen
	No problen	No problen	No problen	No problen	Small probl	No problen	No problen	No problen
No	No problen	No problen	No problen	No problen	No problen	No problen	No problen	No problen
	No problen	No problen	No problen	No problen	Small probl	No problen	No problen	No problen
No	Small probl	Small probl	Small probl	Small probl	Small probl	Small probl	Small probl	Small probl
	Small probl	Small probl	Small probl	Big probler	Big probler	Big probler	Small probl	Big probler
	Small probl	Small probl	Small probl	Small probl	Small probl	Small probl	No problen	Small probl
	No problen	No problen	No problen	No problen	Small probl	No problen	No problen	No problen
	No problen	No problen	No problen	No problen	No problen	No problen	No problen	No problen
	No problen	No problen	No problen	No problen	Small probl	No problen	No problen	No problen
	Small probl	Small probl	Big probler	Big probler	Big probler	Small probl	Small probl	Small probl
No	No problen	No problen	No problen	No problen	No problen	No problen	No problen	No problen
	Small probl	Small probl	Big probler	Big probler	Big probler	Small probl	Small probl	Small probl
	No problen	No problen	No problen	No problen	No problen	No problen	No problen	No problen
	No problen	No problen	No problen	No problen	Small probl	No problen	No problen	No problen
	No problen	No problen	No problen	No problen	No problen	No problen	No problen	No problen
	Small probl	Small probl	Big probler	Big probler	Big probler	Small probl	Small probl	Small probl
No	No problen	No problen	No problen	No problen	No problen	No problen	No problen	No problen
	No problen	No problen	No problen	No problen	No problen	No problen	No problen	No problen
	No problen	No problen	No problen	No problen	No problen	No problen	No problen	No problen
	No problen	No problen	No problen	No problen	No problen	No problen	No problen	No problen
	Small probl	Small probl	Small probl	Big probler	Big probler	Small probl	Small probl	Small probl
	No problen	No problen	No problen	No problen	No problen	No problen	No problen	No problen
No	No problen	No problen	No problen	No problen	Small probl	No problen	No problen	No problen
	No problen	No problen	No problen	No problen	No problen	No problen	No problen	No problen
	No problen	No problen	No problen	No problen	No problen	No problen	No problen	No problen
	No problen	No problen	No problen	No problen	Small probl	No problen	No problen	No problen
	No problen	No problen	No problen	No problen	No problen	No problen	No problen	No problen
	Small probl	Small probl	Small probl	Big probler	Big probler	Small probl	Small probl	Small probl
	No problen	No problen	No problen	No problen	Small probl	No problen	No problen	No problen
	No problen	No problen	No problen	No problen	Small probl	No problen	No problen	No problen
	No problen	No problen	No problen	No problen	Small probl	No problen	No problen	No problen
	No problen	No problen	No problen	No problen	Small probl	No problen	No problen	No problen
	Small probl	Small probl	Big probler	Big probler	Big probler	Big probler	Small probl	Big probler
	No problen	No problen	No problen	No problen	Small probl	No problen	No problen	Small probl
No	No problen	No problen	No problen	No problen	Small probl	No problen	No problen	No problen
	No problen	No problen	Small probl	No problen	Small probl	No problen	No problen	No problen
	No problen	No problen	No problen	No problen	Small probl	No problen	No problen	No problen
	Small probl	Small probl	Small probl	Small probl	Small probl	Small probl	Small probl	Small probl
	No problen	No problen	No problen	No problen	Small probl	Small probl	Small probl	Small probl
	No problen	No problen	Small probl	No problen	No problen	No problen	No problen	No problen
	No problen	No problen	No problen	No problen	No problen	No problen	No problen	No problen
	No problen	No problen	No problen	No problen	No problen	No problen	No problen	No problen
	No problen	No problen	No problen	No problen	No problen	No problen	No problen	No problen
	Small probl	Small probl	Small probl	Big probler	Big probler	Big probler	Big probler	Small probl
	No problen	No problen	No problen	No problen	Small probl	No problen	No problen	No problen
	No problen	No problen	No problen	No problen	No problen	No problen	No problen	No problen
	No problen	No problen	No problen	No problen	Small probl	No problen	No problen	No problen
	No problen	No problen	No problen	No problen	Big probler	No problen	No problen	No problen
	No problen	No problen	No problen	No problen	No problen	No problen	No problen	No problen
	No problen	Small probl	Big probler	Small probl	Small probl	Small probl	Small probl	Small probl
	No problen	Small probl	Small probl	No problen	Small probl	Small probl	Small probl	Small probl

For peer review only

	No problen	No problen	No problen	No problen	No problen	No problen	No problen	No problen
	No problen	No problen	Small probl	No problen	Small probl	No problen	No problen	No problen
	No problen	No problen	No problen	No problen	No problen	No problen	No problen	No problen
	No problen	No problen	No problen	No problen	No problen	No problen	No problen	No problen
	No problen	No problen	No problen	No problen	Small probl	No problen	No problen	No problen
	Small probl	Small probl	Small probl	Big probler	Big probler	Small probl	Small probl	Small probl
	No problen	No problen	No problen	No problen	No problen	No problen	No problen	No problen
No	No problen	No problen	No problen	No problen	Small probl	No problen	No problen	No problen
	Small probl	Small probl	Big probler	Small probl	Small probl	Small probl	Small probl	Small probl
	No problen	No problen	No problen	No problen	No problen	No problen	No problen	No problen
	No problen	No problen	No problen	No problen	Small probl	No problen	No problen	No problen
	No problen	No problen	Small probl	Small probl	Big probler	No problen	No problen	No problen
	Small probl	Small probl	Small probl	Big probler	Big probler	Big probler	Small probl	Small probl
	No problen	No problen	No problen	No problen	Small probl	No problen	No problen	No problen
	No problen	No problen	No problen	No problen	No problen	No problen	No problen	No problen
	No problen	No problen	No problen	No problen	Small probl	No problen	No problen	No problen
	No problen	No problen	No problen	No problen	No problen	No problen	No problen	No problen
	No problen	No problen	No problen	No problen	No problen	No problen	No problen	No problen
	No problen	No problen	No problen	No problen	No problen	No problen	No problen	No problen
	No problen	No problen	No problen	No problen	No problen	No problen	No problen	No problen
	No problen	No problen	No problen	No problen	Small probl	No problen	No problen	No problen
	Small probl	Small probl	Small probl	Small probl	Small probl	Small probl	No problen	Small probl
	No problen	No problen	No problen	No problen	Small probl	No problen	No problen	No problen
	No problen	No problen	Big probler	No problen	Small probl	Small probl	Small probl	Small probl
	No problen	No problen	No problen	No problen	No problen	No problen	No problen	No problen
	No problen	No problen	No problen	No problen	No problen	No problen	No problen	No problen
	No problen	No problen	No problen	No problen	No problen	No problen	No problen	No problen
	No problen	No problen	Big probler	Small probl	Small probl	No problen	Small probl	Small probl
	No problen	No problen	No problen	No problen	No problen	No problen	No problen	No problen
No	Small probl	No problen	No problen	No problen	No problen	No problen	No problen	Small probl
	No problen	No problen	No problen	No problen	No problen	No problen	No problen	No problen
	No problen	No problen	No problen	No problen	Small probl	No problen	No problen	No problen
	Small probl	Small probl	Big probler	Big probler	Big probler	Small probl	Small probl	Small probl
	No problen	No problen	No problen	No problen	No problen	No problen	No problen	No problen
	No problen	No problen	No problen	No problen	Small probl	No problen	No problen	No problen
No	Small probl	Small probl	Small probl	Big probler	Big probler	Small probl	Small probl	Big probler
	Small probl	Small probl	Big probler	Big probler	Big probler	Small probl	Small probl	Big probler
	No problen	No problen	Small probl	No problen	Small probl	No problen	No problen	No problen
	No problen	No problen	No problen	No problen	No problen	No problen	No problen	No problen
	No problen	No problen	Big probler	No problen	No problen	No problen	Small probl	Small probl
No	No problen	Small probl	Big probler	No problen	Small probl	Small probl	No problen	Small probl
	No problen	No problen	No problen	No problen	No problen	No problen	No problen	No problen
	Small probl	Small probl	Big probler	Big probler	Big probler	Small probl	Small probl	Small probl
	No problen	No problen	No problen	No problen	No problen	No problen	Small probl	Small probl
No	Big probler	Big probler	Big probler	Big probler	Big probler	Big probler	Big probler	Big probler
	Small probl	No problen	No problen	No problen	No problen	No problen	No problen	No problen
	Small probl	Small probl	Small probl	Small probl	Small probl	Small probl	Small probl	Small probl
	Small probl	Small probl	Big probler	Big probler	Big probler	Small probl	Big probler	Big probler
	Big probler	No problen	Big probler	Small probl	Big probler	Small probl	Small probl	Small probl
	Small probl	Small probl	Big probler	Small probl	Small probl	Small probl	Small probl	Small probl
	No problen	No problen	Small probl	No problen	No problen	Big probler	No problen	No problen
	Small probl	No problen	Big probler	Small probl	Small probl	Small probl	Big probler	Small probl

	Small probl	Small probl	Big probler	Big probler	Big probler	Small probl	Small probl	Small probl
	No problen	No problen	Big probler	Small probl	No problen	No problen	No problen	No problen
	No problen	No problen	Big probler	No problen	No problen	No problen	No problen	Big probler
	No problen	No problen	Small probl	No problen	No problen	No problen	No problen	No problen
No	No problen	No problen	Big probler	No problen	No problen	No problen	No problen	No problen
	No problen	No problen	Big probler	No problen	No problen	Big probler	No problen	No problen
	No problen	No problen	Big probler	No problen	Big probler	Big probler	No problen	No problen
	Small probl	Small probl	Small probl	Small probl	No problen	No problen	Big probler	Small probl
	No problen	No problen	No problen	No problen	No problen	No problen	No problen	No problen
	Small probl	Small probl	Big probler	Small probl	Big probler	Big probler	Small probl	Small probl
	No problen	No problen	Big probler	Big probler	Big probler	Big probler	No problen	No problen
	No problen	No problen	Big probler	Small probl	No problen	No problen	No problen	No problen
	No problen	No problen	No problen	No problen	No problen	No problen	No problen	Small probl
	No problen	No problen	Big probler	No problen	No problen	No problen	No problen	No problen
	Small probl	No problen	Big probler	Big probler	Big probler	Small probl	Big probler	Big probler
No	Small probl	No problen	Big probler	Small probl	Small probl	Small probl	Small probl	Small probl
	No problen	No problen	No problen	No problen	No problen	No problen	No problen	No problen
	No problen	No problen	Big probler	Small probl	Big probler	No problen	No problen	No problen
	No problen	No problen	No problen	No problen	No problen	No problen	No problen	No problen
	No problen	No problen	No problen	No problen	No problen	No problen	Big probler	Big probler
	Small probl	No problen	Big probler	Small probl	Small probl	Small probl	Small probl	Small probl
	No problen	No problen	No problen	No problen	No problen	No problen	Small probl	Big probler
	Small probl	No problen	No problen	No problen	No problen	No problen	Big probler	Big probler
	No problen	No problen	No problen	No problen	No problen	No problen	No problen	No problen
	Small probl	No problen	Big probler	Small probl	Small probl	Small probl	Small probl	Small probl
	No problen	No problen	Big probler	No problen	No problen	No problen	No problen	No problen
	Small probl	No problen	Big probler	Small probl	Small probl	Small probl	Small probl	Small probl
	No problen	No problen	No problen	No problen	No problen	No problen	No problen	Small probl
	Small probl	Small probl	Big probler	Big probler	Big probler	Big probler	Big probler	Big probler
	Small probl	Small probl	Big probler	Small probl	Small probl	Small probl	Small probl	Big probler
	No problen	No problen	No problen	No problen	Small probl	Big probler	No problen	No problen
	Small probl	Small probl	Big probler	Big probler	Big probler	Small probl	Small probl	Small probl
	No problen	No problen	No problen	No problen	No problen	No problen	No problen	No problen
	No problen	No problen	No problen	No problen	No problen	No problen	No problen	Small probl
	No problen	No problen	No problen	No problen	No problen	No problen	No problen	Small probl
	No problen	No problen	No problen	No problen	No problen	No problen	Small probl	Small probl
No	Small probl	Small probl	Big probler	Small probl	Big probler	Big probler	Small probl	Small probl
	Small probl	No problen	Big probler	Small probl	Small probl	Small probl	Small probl	Small probl
	No problen	No problen	No problen	No problen	No problen	No problen	No problen	No problen
	No problen	No problen	No problen	No problen	No problen	No problen	No problen	No problen
	No problen	No problen	No problen	No problen	No problen	No problen	Big probler	Big probler
No	No problen	No problen	No problen	No problen	No problen	No problen	No problen	Big probler
No	Small probl	Small probl	Small probl	Small probl	Small probl	Small probl	Small probl	Small probl
	No problen	No problen	No problen	No problen	No problen	No problen	No problen	Small probl
	No problen	No problen	No problen	Big probler	Big probler	Big probler	No problen	Big probler
	No problen	No problen	No problen	No problen	No problen	No problen	Small probl	Small probl
	No problen	No problen	No problen	No problen	No problen	No problen	Small probl	Small probl
	No problen	No problen	No problen	No problen	No problen	No problen	No problen	No problen
	No problen	No problen	No problen	No problen	No problen	No problen	No problen	No problen
	Small probl	Small probl	Big probler	Small probl	Small probl	Big probler	Small probl	Small probl
	No problen	No problen	No problen	No problen	No problen	No problen	No problen	Big probler
No	No problen	No problen	No problen	No problen	No problen	No problen	Small probl	Big probler

	No problen	No problen	No problen	No problen	Small probl	Small probl	No problen	No problen
	No problen	No problen	No problen	No problen	No problen	No problen	No problen	No problen
	No problen	No problen	Big probler	No problen	No problen	No problen	No problen	Small probl
No	No problen	No problen	No problen	No problen	No problen	No problen	No problen	No problen
Yes	No problen	No problen	Big probler	No problen	No problen	No problen	No problen	No problen
	No problen	No problen	No problen	No problen	No problen	No problen	No problen	No problen
	No problen	No problen	No problen	No problen	No problen	No problen	No problen	No problen
	Small probl	Small probl	Small probl	Small probl	Small probl	Small probl	Small probl	Small probl
	No problen	No problen	No problen	No problen	No problen	No problen	No problen	No problen
	No problen	No problen	Small probl	No problen	No problen	No problen	No problen	No problen
No	No problen	No problen	No problen	No problen	No problen	No problen	No problen	No problen
	No problen	No problen	No problen	Small probl	No problen	Big probler	No problen	Small probl
	Small probl	Small probl	Big probler	Small probl	Big probler	Small probl	Small probl	Small probl
	No problen	No problen	No problen	No problen	No problen	No problen	No problen	Small probl
	Small probl	No problen	Big probler	Small probl	Small probl	Small probl	Small probl	Big probler
	No problen	No problen	No problen	No problen	No problen	No problen	Small probl	No problen
	Small probl	No problen	Big probler	Small probl	Small probl	Small probl	Small probl	Big probler
	No problen	No problen	No problen	No problen	No problen	No problen	No problen	No problen
	Small probl	Small probl	Small probl	Small probl	Small probl	Small probl	Small probl	Small probl
	No problen	No problen	Small probl	No problen	No problen	Small probl	No problen	No problen
No	No problen	No problen	Big probler	No problen	No problen	No problen	No problen	No problen
	Small probl	No problen	Big probler	Small probl	Small probl	Small probl	Small probl	Small probl
	No problen	No problen	No problen	No problen	No problen	No problen	No problen	Big probler
	No problen	No problen	No problen	No problen	No problen	No problen	No problen	Big probler
No	Small probl	Small probl	Big probler	Big probler	Big probler	Small probl	Small probl	Small probl
	No problen	No problen	No problen	No problen	No problen	No problen	No problen	No problen
	No problen	No problen	No problen	No problen	No problen	No problen	No problen	No problen
No	No problen	No problen	No problen	Small probl	Small probl	No problen	No problen	No problen
	No problen	No problen	No problen	No problen	No problen	No problen	No problen	No problen
	No problen	No problen	No problen	No problen	Small probl	No problen	No problen	No problen
	No problen	No problen	No problen	No problen	Big probler	No problen	No problen	No problen
No	No problen	Small probl	Big probler	Small probl	Big probler	Small probl	Small probl	Small probl
No	No problen	No problen	No problen	Small probl	Big probler	No problen	Small probl	No problen
No	No problen	No problen	Small probl	Small probl	No problen	No problen	No problen	No problen
	No problen	No problen	No problen	No problen	Big probler	No problen	No problen	No problen
	No problen	No problen	No problen	Big probler	Big probler	No problen	No problen	No problen
No	No problen	Small probl	Small probl	Big probler	Big probler	Small probl	Small probl	Big probler
	No problen	No problen	Small probl	No problen	Small probl	Small probl	No problen	No problen
	No problen	No problen	No problen	No problen	No problen	No problen	No problen	No problen
	No problen	No problen	No problen	Big probler	Small probl	No problen	No problen	No problen
	Small probl	Small probl	Small probl	Small probl	Small probl	Small probl	Small probl	Small probl
	No problen	No problen	No problen	No problen	Small probl	No problen	No problen	No problen
	No problen	No problen	Small probl	No problen	Small probl	Small probl	Small probl	Small probl
No	Small probl	Small probl	Big probler	Small probl	Big probler	Small probl	No problen	Small probl
	No problen	No problen	Small probl	No problen	Big probler	Small probl	Small probl	No problen
	No problen	No problen	Big probler	Small probl	Big probler	No problen	No problen	No problen
No	No problen	No problen	No problen	No problen	No problen	No problen	No problen	No problen
	Small probl	No problen	Big probler	No problen	Big probler	No problen	No problen	No problen
No	Small probl	Small probl	Big probler	Big probler	Big probler	Big probler	No problen	Big probler
	No problen	No problen	No problen	No problen	No problen	No problen	No problen	No problen
	No problen	No problen	No problen	No problen	No problen	No problen	No problen	No problen
No	No problen	No problen	Small probl	Big probler	Small probl	No problen	No problen	No problen

	No problen	No problen	No problen	No problen	No problen	No problen	No problen	No problen
	No problen	No problen	No problen	No problen	No problen	No problen	No problen	No problen
	No problen	No problen	No problen	No problen	No problen	No problen	No problen	No problen
	Small probl	Small probl	Big probler	Big probler	Big probler	Small probl	Small probl	Small probl
	No problen	No problen	Small probl	No problen	Small probl	No problen	No problen	No problen
	No problen	No problen	No problen	No problen	No problen	No problen	No problen	No problen
	Small probl	Small probl	Big probler	Big probler	Big probler	Big probler	Small probl	Big probler
	No problen	No problen	No problen	No problen	No problen	No problen	No problen	No problen
	No problen	No problen	Big probler	No problen	Big probler	Small probl	Small probl	No problen
	No problen	No problen	No problen	No problen	No problen	No problen	No problen	No problen
No	No problen	No problen	Big probler	Big probler	Big probler	No problen	No problen	No problen
	No problen	No problen	No problen	No problen	Small probl	No problen	No problen	No problen
	No problen	No problen	No problen	No problen	Small probl	No problen	No problen	No problen
	No problen	No problen	No problen	No problen	Small probl	No problen	No problen	No problen
	No problen	No problen	No problen	No problen	No problen	No problen	No problen	No problen
	Small probl	Small probl	Big probler	Big probler	Big probler	Small probl	Small probl	Small probl
	No problen	No problen	No problen	No problen	Small probl	No problen	No problen	No problen
	No problen	No problen	No problen	No problen	Small probl	No problen	No problen	No problen
	No problen	Small probl	Big probler	Small probl	Big probler	Small probl	Small probl	Small probl
	Small probl	Small probl	Small probl	Small probl	Small probl	Small probl	Small probl	Small probl
No	No problen	No problen	No problen	Small probl	Small probl	No problen	No problen	No problen
	No problen	No problen	No problen	No problen	Small probl	No problen	No problen	No problen
	No problen	No problen	No problen	No problen	No problen	No problen	No problen	No problen
	No problen	No problen	No problen	Small probl	Small probl	No problen	No problen	No problen
No	Small probl	Small probl	Big probler	Big probler	Big probler	Big probler	Small probl	Big probler
	No problen	No problen	No problen	No problen	Small probl	No problen	No problen	No problen
	Small probl	No problen	Big probler	Big probler	Small probl	Small probl	Small probl	Big probler
No	No problen	No problen	No problen	No problen	No problen	No problen	No problen	No problen
	Small probl	No problen	Big probler	Big probler	Big probler	Small probl	Small probl	Small probl
No	No problen	No problen	No problen	No problen	Small probl	Small probl	No problen	No problen
	No problen	No problen	Big probler	Small probl	Small probl	Big probler	Small probl	Small probl
No	No problen	No problen	No problen	Small probl	Big probler	No problen	No problen	No problen
	Small probl	Small probl	Small probl	Big probler	Small probl	Small probl	Small probl	Small probl
	Small probl	Small probl	Small probl	Small probl	Big probler	Small probl	Small probl	Small probl
	Small probl	Small probl	Big probler	Big probler	Big probler	Big probler	Small probl	Small probl
	No problen	No problen	Small probl	Small probl	Small probl	Small probl	Small probl	Small probl
	No problen	No problen	No problen	No problen	Big probler	No problen	No problen	No problen
	No problen	No problen	No problen	No problen	No problen	No problen	No problen	No problen
	No problen	No problen	No problen	No problen	No problen	No problen	No problen	No problen
	No problen	No problen	No problen	No problen	Small probl	No problen	No problen	No problen
	Small probl	Small probl	No problen	Big probler	Big probler	Small probl	Small probl	Small probl
No	No problen	No problen	No problen	No problen	Small probl	No problen	No problen	No problen
	Small probl	Small probl	Big probler	Big probler	Big probler	Big probler	No problen	Big probler
	No problen	No problen	Small probl	No problen	Small probl	No problen	No problen	No problen
	No problen	No problen	No problen	No problen	Small probl	No problen	No problen	No problen
	No problen	No problen	No problen	No problen	Small probl	No problen	No problen	No problen
	No problen	No problen	No problen	No problen	No problen	No problen	No problen	No problen
No	No problen	No problen	No problen	No problen	No problen	No problen	No problen	No problen
	No problen	No problen	No problen	Small probl	Big probler	No problen	No problen	No problen
	No problen	No problen	Small probl	No problen	Big probler	No problen	No problen	No problen
	No problen	No problen	No problen	No problen	Small probl	No problen	No problen	No problen
	No problen	No problen	No problen	No problen	No problen	No problen	No problen	No problen

For peer review only

	No problen	No problen	No problen	No problen	No problen	No problen	No problen	No problen
	No problen	No problen	Small probl	No problen	No problen	No problen	No problen	No problen
	No problen	No problen	No problen	No problen	No problen	No problen	No problen	No problen
	No problen	No problen	No problen	No problen	Big probler	No problen	No problen	No problen
No	No problen	No problen	No problen	Small probl	Big probler	No problen	No problen	No problen
No	Small probl	Small probl	Big probler	Big probler	Big probler	Big probler	Small probl	Small probl
	Small probl	No problen	Big probler	Big probler	Big probler	Small probl	Small probl	Small probl
	Big probler	Small probl	Big probler	No problen	Small probl	Small probl	No problen	No problen
	Small probl	Small probl	Big probler	Big probler	Big probler	Big probler	No problen	Big probler
	No problen	No problen	No problen	No problen	No problen	No problen	No problen	No problen
	No problen	No problen	No problen	No problen	Small probl	No problen	No problen	Small probl
	No problen	No problen	No problen	No problen	Big probler	No problen	No problen	No problen
	No problen	No problen	No problen	No problen	No problen	No problen	No problen	No problen
	No problen	No problen	Small probl	No problen	Small probl	No problen	No problen	No problen
No	Small probl	Small probl	Big probler	Big probler	Big probler	Big probler	Small probl	Big probler
No	Small probl	Small probl	Small probl	Small probl	Small probl	Small probl	Small probl	Big probler
No	No problen	No problen	No problen	No problen	No problen	No problen	No problen	No problen
	Small probl	No problen	Big probler	Small probl	Big probler	Small probl	Small probl	Small probl
	No problen	No problen	No problen	Small probl	Small probl	No problen	No problen	No problen
	No problen	No problen	No problen	Small probl	Small probl	No problen	No problen	Small probl
	No problen	No problen	No problen	Big probler	Big probler	No problen	No problen	No problen
No	No problen	No problen	No problen	Small probl	Small probl	No problen	No problen	No problen
No	No problen	No problen	No problen	Big probler	Big probler	Small probl	Small probl	Small probl
	No problen	No problen	No problen	No problen	No problen	No problen	No problen	No problen
	No problen	No problen	No problen	No problen	Big probler	No problen	No problen	No problen
	No problen	No problen	No problen	No problen	No problen	No problen	No problen	No problen
	No problen	Small probl	Small probl	Small probl	Big probler	Small probl	Small probl	Small probl
	No problen	No problen	Small probl	No problen	No problen	No problen	No problen	No problen
No	No problen	No problen	No problen	Small probl	Small probl	No problen	No problen	No problen
No	No problen	No problen	Small probl	Big probler	Big probler	No problen	No problen	No problen
	No problen	No problen	No problen	Small probl	Small probl	Small probl	Small probl	Small probl
	No problen	No problen	Small probl	Small probl	Big probler	Small probl	Small probl	Small probl
No	Small probl	Small probl	Big probler	Big probler	Big probler	Big probler	Small probl	Small probl
	Small probl	Small probl	Big probler	Small probl	Big probler	Small probl	Small probl	Small probl
No	No problen	No problen	No problen	No problen	Small probl	Small probl	Small probl	Small probl
	No problen	No problen	No problen	No problen	No problen	No problen	No problen	No problen
No	Small probl	Small probl	Big probler	Small probl	Big probler	Small probl	Small probl	Small probl
	Small probl	Small probl	Big probler	Big probler	Big probler	Big probler	Small probl	Big probler
No	No problen	No problen	No problen	No problen	No problen	No problen	No problen	No problen
	No problen	No problen	Big probler	No problen	Small probl	No problen	No problen	No problen
	No problen	Small probl	Big probler	Small probl	Big probler	Small probl	Small probl	Small probl
No	No problen	Small probl	Big probler	Big probler	Big probler	Small probl	Small probl	Small probl
	No problen	No problen	No problen	No problen	No problen	No problen	No problen	No problen
No	No problen	No problen	No problen	No problen	Small probl	No problen	No problen	No problen
	No problen	No problen	No problen	No problen	No problen	No problen	No problen	No problen
	No problen	No problen	No problen	Small probl	Small probl	No problen	Small probl	Small probl
	No problen	No problen	No problen	No problen	No problen	No problen	No problen	No problen
	Small probl	Small probl	Big probler	Big probler	Big probler	Small probl	Small probl	Big probler
	No problen	No problen	No problen	No problen	Small probl	No problen	No problen	No problen
	No problen	No problen	No problen	No problen	Small probl	No problen	No problen	No problen
No	No problen	No problen	Small probl	No problen	Small probl	No problen	No problen	No problen
	No problen	No problen	No problen	No problen	Big probler	No problen	No problen	No problen

For peer review only

	Small probl	No problen	Big probler	Big probler	Big probler	Small probl	Small probl	Small probl
	No problen	No problen	Small probl	No problen	Small probl	No problen	No problen	No problen
	Small probl	Small probl	Big probler	Big probler	Big probler	Big probler	No problen	Big probler
No	No problen	No problen	No problen	No problen	No problen	No problen	No problen	No problen
	No problen	No problen	No problen	No problen	No problen	No problen	No problen	No problen
No	No problen	No problen	No problen	No problen	Big probler	No problen	No problen	No problen
	No problen	No problen	No problen	No problen	No problen	No problen	No problen	No problen
	No problen	No problen	No problen	No problen	No problen	No problen	No problen	No problen
	No problen	No problen	No problen	No problen	No problen	No problen	No problen	No problen
	No problen	No problen	No problen	No problen	No problen	No problen	No problen	No problen
	No problen	No problen	Big probler	Big probler	Big probler	Small probl	No problen	No problen
	No problen	No problen	No problen	No problen	Big probler	No problen	No problen	No problen
	No problen	No problen	No problen	No problen	Big probler	No problen	No problen	No problen
	No problen	No problen	Small probl	No problen	Small probl	No problen	No problen	No problen
No	No problen	No problen	No problen	Small probl	No problen	No problen	No problen	No problen
	No problen	No problen	No problen	No problen	Small probl	No problen	No problen	No problen
No	No problen	No problen	Big probler	Big probler	Big probler	Small probl	No problen	No problen
No	No problen	No problen	No problen	No problen	Small probl	No problen	No problen	No problen
	No problen	No problen	No problen	No problen	No problen	No problen	No problen	No problen
No	No problen	No problen	No problen	No problen	Small probl	No problen	No problen	No problen
	No problen	Small probl	Big probler	Big probler	Big probler	Small probl	Small probl	Small probl
	Small probl	Small probl	Big probler	Big probler	Big probler	Small probl	Small probl	Small probl
No	No problen	No problen	No problen	No problen	No problen	No problen	No problen	No problen
	No problen	No problen	Small probl	No problen	Big probler	No problen	No problen	No problen
	No problen	No problen	Small probl	Big probler	Big probler	Small probl	Small probl	No problen
	Small probl	Small probl	Big probler	Big probler	Big probler	Big probler	Small probl	Big probler
	No problen	No problen	No problen	No problen	No problen	No problen	No problen	No problen
No	No problen	No problen	No problen	No problen	Small probl	No problen	No problen	No problen
No	No problen	No problen	No problen	Big probler	Big probler	No problen	No problen	No problen
	No problen	No problen	No problen	No problen	Big probler	No problen	No problen	No problen
	No problen	No problen	Small probl	No problen	No problen	No problen	No problen	Small probl
No	No problen	No problen	Big probler	Small probl	Small probl	No problen	No problen	No problen
	No problen	No problen	No problen	No problen	Big probler	No problen	No problen	No problen
	No problen	No problen	No problen	No problen	No problen	No problen	No problen	No problen
	Small probl	Small probl	Small probl	Small probl	Small probl	Small probl	Small probl	Small probl
	No problen	No problen	No problen	No problen	Small probl	No problen	No problen	No problen
	Small probl	Small probl	Small probl	Big probler	Big probler	Small probl	Small probl	Small probl
	Small probl	Small probl	Big probler	Big probler	Big probler	Small probl	Small probl	Small probl
	Small probl	Small probl	Small probl	Small probl	Small probl	Small probl	Small probl	Small probl
No	No problen	No problen	No problen	No problen	No problen	No problen	No problen	No problen
	No problen	No problen	No problen	No problen	No problen	No problen	No problen	No problen
	No problen	No problen	No problen	Small probl	Small probl	No problen	No problen	No problen
No	No problen	No problen	No problen	No problen	No problen	No problen	No problen	No problen
	Small probl	Small probl	Small probl	Big probler	Big probler	Big probler	Small probl	Small probl
No	No problen	No problen	No problen	No problen	No problen	No problen	No problen	No problen
	No problen	No problen	No problen	No problen	No problen	No problen	No problen	No problen
No	Small probl	Small probl	Small probl	Big probler	Big probler	Small probl	Small probl	Small probl
	Small probl	Small probl	Small probl	Small probl	Small probl	Small probl	Small probl	Small probl
	No problen	No problen	No problen	No problen	No problen	No problen	No problen	No problen
	Small probl	Small probl	Small probl	Small probl	Small probl	Small probl	Small probl	Small probl
	No problen	No problen	Small probl	No problen	No problen	No problen	No problen	No problen
No	No problen	No problen	No problen	No problen	No problen	No problen	No problen	No problen

For peer review only

	Small probl	Small probl	Small probl	Small probl	Small probl	Small probl	Small probl	Small probl
	Small probl	Small probl	Big probler	Big probler	Big probler	Small probl	Small probl	Small probl
	Small probl	Small probl	Small probl	Small probl	Small probl	Small probl	Small probl	Small probl
	No problen	No problen	No problen	Big probler	Big probler	No problen	No problen	No problen
	No problen	No problen	No problen	No problen	No problen	No problen	No problen	No problen
	No problen	No problen	Small probl	No problen	Small probl	No problen	No problen	No problen
	Small probl	Small probl	Small probl	Small probl	Small probl	Small probl	Small probl	Small probl
No	No problen	No problen	No problen	No problen	No problen	No problen	No problen	No problen
	No problen	No problen	No problen	No problen	No problen	No problen	No problen	No problen
	No problen	No problen	No problen	No problen	No problen	No problen	No problen	No problen
	No problen	No problen	No problen	No problen	Small probl	No problen	No problen	No problen
No	No problen	No problen	No problen	No problen	No problen	No problen	No problen	No problen
	No problen	No problen	No problen	No problen	No problen	No problen	No problen	No problen
No	No problen	No problen	No problen	No problen	No problen	No problen	No problen	No problen
	No problen	No problen	No problen	No problen	No problen	No problen	No problen	No problen
	Small probl	Small probl	Small probl	Big probler	Big probler	Small probl	No problen	Small probl
	Small probl	Small probl	Small probl	Small probl	Small probl	Small probl	Small probl	Small probl
	No problen	No problen	No problen	No problen	No problen	No problen	No problen	No problen
	Small probl	Small probl	Small probl	Big probler	Big probler	Big probler	Small probl	Small probl
	No problen	No problen	No problen	No problen	No problen	No problen	No problen	No problen
	No problen	No problen	No problen	No problen	No problen	No problen	No problen	No problen
No	No problen	No problen	No problen	Big probler	Big probler	No problen	No problen	No problen
	No problen	No problen	No problen	No problen	No problen	No problen	No problen	No problen
No	No problen	No problen	No problen	No problen	No problen	No problen	No problen	No problen
	No problen	No problen	No problen	No problen	No problen	No problen	No problen	No problen
	No problen	No problen	No problen	No problen	No problen	No problen	No problen	No problen
	No problen	No problen	No problen	No problen	Small probl	No problen	No problen	No problen
	No problen	No problen	No problen	No problen	No problen	No problen	No problen	No problen
	No problen	No problen	No problen	No problen	No problen	No problen	No problen	No problen
No	No problen	No problen	No problen	No problen	No problen	No problen	No problen	No problen
No	No problen	No problen	No problen	Small probl	Small probl	No problen	No problen	No problen
	Small probl	Small probl	Big probler	Big probler	Big probler	Small probl	Small probl	Small probl
	No problen	No problen	No problen	No problen	Small probl	No problen	No problen	No problen
	No problen	No problen	No problen	No problen	No problen	No problen	No problen	No problen
	No problen	No problen	No problen	No problen	No problen	No problen	No problen	No problen
	No problen	No problen	No problen	No problen	No problen	No problen	No problen	No problen
Yes	Small probl	Small probl	Small probl	Big probler	Big probler	Big probler	Small probl	Small probl
No	No problen	No problen	No problen	No problen	No problen	No problen	No problen	No problen
	No problen	No problen	No problen	No problen	No problen	No problen	No problen	No problen
	Small probl	Small probl	Big probler	Big probler	Big probler	No problen	No problen	No problen
	No problen	No problen	No problen	No problen	No problen	No problen	No problen	No problen
	No problen	No problen	No problen	No problen	No problen	No problen	No problen	No problen
No	No problen	No problen	No problen	No problen	No problen	No problen	No problen	No problen
	Small probl	Small probl	Big probler	Big probler	Big probler	Small probl	Small probl	Small probl
	No problen	No problen	No problen	No problen	No problen	No problen	No problen	No problen
No	Small probl	Small probl	Small probl	Small probl	Small probl	Small probl	Small probl	Small probl
	No problen	No problen	No problen	No problen	No problen	No problen	No problen	No problen
	Small probl	Small probl	Big probler	Big probler	Big probler	Small probl	Small probl	Small probl
	Small probl	Small probl	Big probler	Big probler	Big probler	Small probl	Small probl	Small probl
	No problen	No problen	Small probl	Big probler	Big probler	Small probl	Small probl	Small probl
	No problen	No problen	No problen	No problen	No problen	No problen	No problen	No problen
	No problen	No problen	No problen	No problen	No problen	No problen	No problen	No problen

For peer review only

	Small probl	Small probl	Small probl	Small probl	Small probl	Small probl	Small probl	Small probl
	No problen	No problen	No problen	No problen	No problen	No problen	No problen	No problen
	No problen	No problen	No problen	No problen	No problen	No problen	No problen	No problen
	Small probl	Small probl	Small probl	Big probler	Big probler	Small probl	Small probl	Small probl
	No problen	No problen	No problen	No problen	No problen	No problen	No problen	No problen
	No problen	No problen	No problen	No problen	No problen	No problen	No problen	No problen
	Small probl	Small probl	Small probl	Big probler	Big probler	Small probl	Small probl	Big probler
	No problen	No problen	No problen	No problen	No problen	No problen	No problen	No problen
No	No problen	No problen	No problen	No problen	No problen	No problen	No problen	No problen
	Small probl	Small probl	Small probl	Small probl	Small probl	Small probl	Small probl	Small probl
	No problen	No problen	Big probler	No problen	No problen	No problen	No problen	No problen
No	No problen	No problen	No problen	No problen	No problen	No problen	No problen	No problen
	No problen	No problen	No problen	No problen	No problen	No problen	No problen	No problen
No	No problen	No problen	No problen	No problen	No problen	No problen	No problen	No problen
No	No problen	No problen	No problen	Small probl	Big probler	No problen	No problen	No problen
	No problen	No problen	No problen	No problen	No problen	No problen	No problen	No problen
No	No problen	No problen	No problen	No problen	Small probl	No problen	No problen	No problen
	Small probl	Small probl	Small probl	Small probl	Small probl	Small probl	Small probl	Small probl
No	No problen	No problen	No problen	No problen	Small probl	No problen	No problen	No problen
	No problen	No problen	No problen	No problen	Small probl	No problen	No problen	No problen
	Small probl	Small probl	Small probl	Small probl	Small probl	Small probl	Small probl	Small probl
No	No problen	No problen	No problen	No problen	No problen	No problen	No problen	No problen
No	No problen	No problen	No problen	No problen	No problen	No problen	No problen	No problen
	No problen	No problen	No problen	No problen	No problen	No problen	No problen	No problen
	Small probl	Small probl	Small probl	Small probl	Small probl	Small probl	Small probl	Small probl
	Small probl	Small probl	Small probl	Small probl	Small probl	Small probl	Small probl	Small probl
	Small probl	Small probl	Small probl	Big probler	Big probler	Small probl	Small probl	Small probl
	Small probl	Small probl	Big probler	Big probler	Big probler	Small probl	Small probl	Small probl
	Small probl	Small probl	Small probl	Small probl	Small probl	Small probl	Small probl	Small probl
	Small probl	Small probl	Big probler	Big probler	Big probler	No problen	No problen	Small probl
	No problen	No problen	No problen	No problen	No problen	No problen	No problen	No problen
	No problen	No problen	No problen	No problen	No problen	No problen	No problen	No problen
	No problen	No problen	No problen	No problen	No problen	No problen	No problen	No problen
	No problen	No problen	No problen	No problen	No problen	No problen	No problen	No problen
	No problen	No problen	Small probl	No problen	Small probl	No problen	No problen	No problen
	No problen	No problen	No problen	No problen	Small probl	No problen	No problen	No problen
	No problen	No problen	No problen	No problen	No problen	No problen	No problen	No problen
	Small probl	Small probl	Big probler	Big probler	Big probler	Small probl	Small probl	Small probl
	Small probl	Small probl	Big probler	Big probler	Big probler	Small probl	Small probl	Small probl
	No problen	No problen	No problen	No problen	No problen	No problen	No problen	No problen
No	No problen	No problen	No problen	Small probl	Small probl	No problen	No problen	No problen
	Small probl	Small probl	Small probl	Small probl	Small probl	Small probl	Small probl	Small probl
	No problen	No problen	No problen	No problen	No problen	No problen	No problen	No problen
	Small probl	Small probl	Small probl	Big probler	Big probler	Small probl	No problen	Small probl
	No problen	No problen	No problen	No problen	Small probl	No problen	Small probl	No problen
	Small probl	Small probl	Big probler	Big probler	Big probler	Small probl	Small probl	Big probler
	No problen	No problen	No problen	No problen	No problen	No problen	No problen	No problen
	Small probl	Small probl	Big probler	Big probler	Big probler	Small probl	Small probl	Small probl
	Small probl	Small probl	Small probl	Small probl	Small probl	Small probl	Small probl	Small probl
No	No problen	No problen	No problen	No problen	Small probl	No problen	No problen	No problen
	No problen	No problen	No problen	No problen	No problen	No problen	No problen	No problen
	No problen	No problen	No problen	No problen	No problen	No problen	No problen	No problen

For peer review only

	No problen	No problen	No problen	No problen	No problen	No problen	No problen	No problen
	No problen	No problen	No problen	No problen	No problen	No problen	No problen	No problen
	No problen	No problen	No problen	No problen	No problen	No problen	No problen	No problen
	No problen	No problen	No problen	No problen	No problen	No problen	No problen	No problen
	Small probl	Small probl	Small probl	Small probl	Small probl	Small probl	Small probl	Small probl
	No problen	No problen	No problen	No problen	No problen	No problen	No problen	No problen
No	No problen	Small probl	No problen	No problen	Small probl	No problen	No problen	No problen
	No problen	No problen	No problen	No problen	No problen	No problen	No problen	No problen
	No problen	No problen	No problen	No problen	No problen	No problen	No problen	No problen
	Small probl	Small probl	Small probl	Small probl	Small probl	Small probl	Small probl	Small probl
	No problen	No problen	No problen	No problen	No problen	No problen	No problen	No problen
	No problen	No problen	No problen	No problen	Small probl	No problen	No problen	No problen
	No problen	No problen	No problen	No problen	No problen	No problen	No problen	No problen
	No problen	Small probl	Small probl	No problen	No problen	Small probl	No problen	Big probler
	No problen	No problen	No problen	No problen	Small probl	No problen	No problen	No problen
	Small probl	Small probl	Small probl	Small probl	Small probl	Small probl	Small probl	Small probl
	Small probl	Small probl	Small probl	Small probl	Small probl	Small probl	Small probl	Small probl
	No problen	No problen	No problen	No problen	No problen	No problen	No problen	No problen
	No problen	No problen	No problen	No problen	No problen	No problen	No problen	No problen
	No problen	No problen	No problen	No problen	No problen	No problen	No problen	No problen
No	Small probl	No problen	No problen	No problen	No problen	No problen	No problen	No problen
	Small probl	Small probl	Small probl	Small probl	Big probler	Big probler	Small probl	Small probl
	No problen	No problen	No problen	No problen	No problen	No problen	No problen	No problen
No	No problen	No problen	No problen	Small probl	Big probler	No problen	No problen	No problen
	No problen	No problen	No problen	No problen	No problen	No problen	No problen	No problen
	No problen	No problen	No problen	No problen	Small probl	No problen	No problen	No problen
	No problen	Small probl	No problen	No problen	Small probl	No problen	No problen	No problen
No	No problen	No problen	No problen	No problen	No problen	No problen	No problen	No problen
	Small probl	Small probl	Small probl	Small probl	Small probl	Small probl	Small probl	Small probl
	No problen	No problen	No problen	No problen	No problen	No problen	No problen	No problen
	No problen	No problen	No problen	No problen	Small probl	No problen	No problen	No problen
No	No problen	No problen	No problen	No problen	No problen	No problen	No problen	No problen
	No problen	No problen	No problen	No problen	No problen	No problen	No problen	No problen
	Small probl	Small probl	Small probl	Small probl	Small probl	Small probl	Small probl	Small probl
	No problen	No problen	No problen	No problen	No problen	No problen	No problen	No problen
	No problen	No problen	No problen	No problen	No problen	No problen	No problen	No problen
	No problen	No problen	No problen	No problen	No problen	No problen	No problen	No problen
	No problen	No problen	No problen	No problen	No problen	No problen	No problen	No problen
	Small probl	Small probl	Small probl	Small probl	Small probl	Small probl	Small probl	Small probl
	No problen	No problen	No problen	No problen	No problen	No problen	No problen	No problen
	No problen	No problen	No problen	No problen	No problen	No problen	No problen	No problen
No	No problen	No problen	Small probl	No problen	Big probler	No problen	No problen	No problen
	Small probl	Small probl	Small probl	Small probl	Small probl	Small probl	Small probl	Small probl
	Small probl	Small probl	Small probl	Small probl	Small probl	Small probl	Small probl	Small probl
	No problen	No problen	No problen	No problen	No problen	No problen	No problen	No problen
	Small probl	Small probl	Small probl	Small probl	Small probl	Small probl	Small probl	Small probl
	No problen	No problen	No problen	No problen	No problen	No problen	No problen	No problen
	No problen	No problen	No problen	No problen	Small probl	No problen	No problen	No problen
	No problen	No problen	No problen	No problen	Small probl	No problen	No problen	No problen
	No problen	No problen	No problen	No problen	No problen	No problen	No problen	No problen
	No problen	No problen	No problen	No problen	No problen	No problen	No problen	No problen
	No problen	No problen	No problen	No problen	No problen	No problen	No problen	No problen

For peer review only

	Small probl	Small probl	Small probl	Small probl	Small probl	Small probl	Small probl	Small probl
	No problen	No problen	No problen	No problen	No problen	No problen	No problen	No problen
	No problen	No problen	No problen	No problen	No problen	No problen	No problen	No problen
	No problen	No problen	No problen	No problen	No problen	No problen	No problen	No problen
	No problen	No problen	No problen	No problen	No problen	No problen	No problen	No problen
	No problen	No problen	No problen	No problen	No problen	No problen	No problen	No problen
No	No problen	No problen	No problen	Big probler	Big probler	No problen	No problen	No problen
	Small probl	Small probl	Small probl	Small probl	Small probl	Small probl	Small probl	Small probl
	No problen	No problen	No problen	No problen	No problen	No problen	No problen	No problen
No	No problen	No problen	No problen	No problen	No problen	No problen	No problen	Big probler
	No problen	No problen	No problen	No problen	No problen	No problen	No problen	No problen
	No problen	No problen	No problen	No problen	No problen	No problen	No problen	No problen
No	No problen	No problen	No problen	No problen	No problen	No problen	No problen	No problen
	No problen	No problen	No problen	No problen	No problen	No problen	No problen	No problen
	No problen	No problen	No problen	Small probl	Big probler	No problen	No problen	No problen
	No problen	No problen	No problen	No problen	No problen	No problen	No problen	No problen
	No problen	No problen	No problen	No problen	Small probl	No problen	No problen	No problen
	No problen	No problen	No problen	No problen	Small probl	No problen	No problen	No problen
	No problen	No problen	No problen	No problen	No problen	No problen	No problen	No problen
No	No problen	No problen	No problen	No problen	No problen	No problen	No problen	No problen
	No problen	No problen	No problen	No problen	No problen	No problen	No problen	No problen
	Small probl	Small probl	Small probl	Small probl	Small probl	Small probl	Small probl	Small probl
	No problen	No problen	No problen	Big probler	Big probler	No problen	No problen	No problen
	No problen	No problen	No problen	No problen	No problen	No problen	No problen	No problen
	Small probl	Small probl	Small probl	Small probl	Small probl	Small probl	Small probl	Small probl
No	No problen	No problen	No problen	No problen	No problen	No problen	No problen	No problen
No	No problen	No problen	Small probl	Small probl	Big probler	No problen	No problen	Small probl
	No problen	No problen	No problen	No problen	Small probl	No problen	No problen	No problen
	Small probl	Small probl	Small probl	Big probler	Big probler	Big probler	Big probler	Small probl
	No problen	No problen	No problen	No problen	Big probler	No problen	No problen	No problen
No	No problen	No problen	No problen	Big probler	Big probler	No problen	No problen	No problen
No	No problen	No problen	No problen	Small probl	Big probler	No problen	No problen	Small probl
No	No problen	No problen	Small probl	Small probl	Big probler	No problen	No problen	No problen
No	No problen	No problen	No problen	No problen	Small probl	No problen	No problen	No problen
	No problen	No problen	No problen	No problen	No problen	No problen	No problen	No problen
No	No problen	No problen	No problen	No problen	No problen	No problen	No problen	No problen
	No problen	No problen	No problen	No problen	No problen	No problen	No problen	No problen
	No problen	No problen	Big probler	No problen	No problen	No problen	No problen	No problen
No	No problen	No problen	No problen	No problen	No problen	No problen	No problen	No problen
	No problen	No problen	No problen	No problen	No problen	No problen	No problen	No problen
	Small probl	Small probl	Small probl	Small probl	Small probl	Small probl	Small probl	Small probl
	Small probl	Small probl	Small probl	Big probler	Big probler	Small probl	Small probl	Small probl
	No problen	No problen	No problen	No problen	No problen	No problen	No problen	No problen
	No problen	No problen	No problen	No problen	No problen	No problen	No problen	No problen
	Small probl	Small probl	Small probl	Small probl	Big probler	Big probler	Small probl	Small probl
	No problen	No problen	No problen	Small probl	No problen	No problen	No problen	No problen
	No problen	No problen	No problen	No problen	No problen	No problen	No problen	No problen
	Small probl	Small probl	Small probl	Small probl	Big probler	Big probler	Small probl	Small probl
	No problen	No problen	No problen	No problen	No problen	No problen	No problen	No problen
	Small probl	Big probler	Small probl	No problen	No problen	Big probler	No problen	No problen
	No problen	No problen	No problen	No problen	No problen	No problen	No problen	No problen
	No problen	No problen	No problen	No problen	No problen	No problen	No problen	No problen

For peer review only

	Small probl	Small probl	Small probl	Small probl	Small probl	Small probl	Small probl	Small probl
	No problen	No problen	No problen	No problen	No problen	No problen	No problen	No problen
	No problen	No problen	No problen	No problen	No problen	No problen	No problen	No problen
	Small probl	Small probl	Small probl	Small probl	Big probler	Big probler	Small probl	Small probl
	No problen	No problen	No problen	No problen	No problen	No problen	No problen	No problen
	No problen	No problen	No problen	No problen	No problen	No problen	No problen	Big probler
	No problen	No problen	No problen	No problen	No problen	No problen	No problen	No problen
	Small probl	Small probl	Small probl	Small probl	Small probl	Small probl	Small probl	Small probl
	No problen	No problen	No problen	No problen	No problen	No problen	No problen	No problen
	No problen	No problen	No problen	No problen	No problen	No problen	No problen	No problen
	Small probl	Small probl	Small probl	Big probler	Big probler	Big probler	Small probl	Small probl
	No problen	No problen	No problen	No problen	No problen	No problen	No problen	No problen
	No problen	No problen	No problen	No problen	No problen	No problen	No problen	No problen
	No problen	No problen	Small probl	Small probl	Small probl	No problen	No problen	No problen
	Small probl	Small probl	No problen	Small probl	Big probler	Small probl	Small probl	Small probl
No	No problen	No problen	Big probler	No problen	Big probler	Small probl	No problen	No problen
	No problen	No problen	Small probl	Small probl	Small probl	No problen	No problen	No problen
	No problen	No problen	No problen	Big probler	Big probler	No problen	No problen	Small probl
	No problen	No problen	No problen	No problen	No problen	No problen	No problen	No problen
	Big probler	Small probl	Big probler	Big probler	Big probler	Small probl	Small probl	Small probl
	No problen	No problen	Small probl	Big probler	Big probler	No problen	Small probl	Small probl
No	No problen	No problen	Small probl	Big probler	Big probler	No problen	Big probler	Small probl
	Small probl	No problen	No problen	Small probl	Big probler	Small probl	No problen	No problen
	Big probler	No problen	Big probler	Big probler	Big probler	Small probl	No problen	No problen
	Big probler	Small probl	Big probler	Big probler	Big probler	Small probl	No problen	No problen
No	No problen	No problen	Big probler	Small probl	Big probler	No problen	No problen	No problen
	Big probler	Small probl	Big probler	Big probler	Big probler	Small probl	No problen	No problen
	No problen	No problen	Big probler	Big probler	Small probl	No problen	Small probl	Small probl
	No problen	No problen	No problen	Small probl	Small probl	No problen	Small probl	Small probl
	No problen	No problen	Small probl	No problen	Big probler	Small probl	Small probl	Small probl
	Big probler	Small probl	Big probler	Big probler	Big probler	Small probl	No problen	No problen
	No problen	No problen	Small probl	Big probler	Big probler	Small probl	No problen	No problen
	No problen	No problen	Small probl	Big probler	Big probler	Small probl	No problen	Small probl
Yes	No problen	No problen	Big probler	No problen	Small probl	Big probler	No problen	No problen
	No problen	No problen	Small probl	Small probl	Small probl	No problen	No problen	No problen
	Big probler	Small probl	Big probler	Big probler	Big probler	Small probl	No problen	No problen
	No problen	No problen	Big probler	Big probler	Big probler	Small probl	No problen	Small probl
No	No problen	No problen	Small probl	No problen	No problen	No problen	No problen	No problen
	Big probler	No problen	Big probler	Big probler	Big probler	No problen	No problen	No problen
	No problen	No problen	No problen	No problen	Small probl	No problen	Small probl	Small probl
Yes	No problen	No problen	Big probler	No problen	Small probl	No problen	No problen	No problen
	No problen	Small probl	Big probler	Big probler	Big probler	Small probl	No problen	No problen
	No problen	No problen	Small probl	Big probler	Big probler	Big probler	No problen	Small probl
No	No problen	No problen	Small probl	No problen	No problen	Small probl	No problen	No problen
	No problen	Small probl	Big probler	Big probler	Big probler	Big probler	Small probl	Small probl
Yes	No problen	No problen	Small probl	No problen	Small probl	No problen	Small probl	No problen
	No problen	No problen	Big probler	No problen	No problen	Big probler	No problen	No problen
No	No problen	No problen	No problen	No problen	No problen	No problen	No problen	No problen
	No problen	No problen	Big probler	Small probl	Big probler	Big probler	Small probl	Small probl
No	No problen	No problen	Small probl	No problen	Small probl	Small probl	No problen	No problen
	No problen	No problen	No problen	Small probl	Small probl	No problen	Small probl	No problen
	No problen	No problen	No problen	Big probler	Big probler	No problen	Small probl	Small probl

For peer review only

	No problen	No problen	No problen	No problen	Small probl	No problen	No problen	No problen
	No problen	No problen	Small probl	No problen	Small probl	Small probl	No problen	No problen
	No problen	No problen	Big probler	Big probler	Big probler	Big probler	Big probler	Small probl
	No problen	No problen	Big probler	No problen	Big probler	No problen	Small probl	Small probl
	Big probler	Small probl	Big probler	Big probler	Big probler	Small probl	No problen	No problen
	Small probl	Small probl	Big probler	Big probler	Big probler	Big probler	Small probl	Small probl
	Big probler	Small probl	Big probler	Big probler	Big probler	Small probl	No problen	No problen
No	No problen	No problen	Small probl	No problen	Small probl	No problen	No problen	No problen
	No problen	No problen	Big probler	No problen	Big probler	No problen	No problen	No problen
	No problen	No problen	Big probler	Big probler	Big probler	Big probler	No problen	Small probl
	No problen	No problen	Small probl	Small probl	Big probler	No problen	Small probl	Small probl
No	No problen	No problen	Small probl	Small probl	Big probler	Small probl	Small probl	Small probl
No	No problen	No problen	Small probl	No problen	Small probl	No problen	No problen	No problen
No	No problen	No problen	Big probler	No problen	Small probl	Small probl	No problen	No problen
No	Big probler	Small probl	Big probler	Big probler	Big probler	No problen	No problen	No problen
No	Small probl	Small probl	Big probler	Big probler	Big probler	Big probler	No problen	No problen
	No problen	No problen	Big probler	Big probler	Big probler	Big probler	Big probler	Small probl
No	No problen	No problen	Small probl	Small probl	Big probler	Big probler	Small probl	Small probl
No	No problen	No problen	Big probler	No problen	Small probl	No problen	No problen	No problen
No	No problen	No problen	No problen	No problen	No problen	No problen	No problen	No problen
	Big probler	Big probler	Big probler	Big probler	Big probler	Small probl	Small probl	Small probl
	No problen	Small probl	No problen	Big probler	Big probler	No problen	Small probl	Small probl
	No problen	Small probl	Small probl	No problen	Big probler	No problen	Small probl	Small probl
	No problen	No problen	No problen	No problen	No problen	No problen	No problen	No problen
No	No problen	No problen	No problen	Small probl	No problen	No problen	Small probl	Small probl
	No problen	No problen	Big probler	No problen	Big probler	No problen	No problen	No problen
No	No problen	No problen	Small probl	No problen	Small probl	No problen	No problen	No problen
	No problen	No problen	No problen	Small probl	Big probler	No problen	Small probl	Small probl
	Big probler	Small probl	Big probler	Big probler	Big probler	Small probl	No problen	No problen
	No problen	No problen	No problen	Small probl	No problen	No problen	Small probl	Small probl
	No problen	No problen	Small probl	Big probler	Big probler	Small probl	No problen	Small probl
	No problen	No problen	No problen	Small probl	Big probler	No problen	No problen	No problen
	Big probler	Small probl	Big probler	Big probler	Big probler	Small probl	No problen	No problen
	Big probler	Small probl	Big probler	Big probler	Big probler	Small probl	No problen	No problen
	No problen	No problen	No problen	No problen	Small probl	No problen	Small probl	Small probl
	Small probl	No problen	Small probl	Small probl	Big probler	Small probl	Small probl	No problen
	No problen	No problen	Small probl	Small probl	Big probler	No problen	No problen	Small probl
No	No problen	No problen	Small probl	No problen	Small probl	No problen	No problen	No problen
No	No problen	No problen	No problen	Small probl	No problen	No problen	Small probl	Small probl
	No problen	No problen	No problen	No problen	Small probl	No problen	Small probl	No problen
	No problen	No problen	No problen	No problen	No problen	No problen	No problen	No problen
	Big probler	Small probl	Big probler	Big probler	Big probler	Small probl	No problen	No problen
	No problen	No problen	No problen	No problen	Small probl	No problen	Small probl	No problen
	No problen	No problen	No problen	No problen	Small probl	No problen	Small probl	Small probl
	No problen	No problen	No problen	Small probl	Small probl	No problen	Small probl	Small probl
Yes	No problen	No problen	Small probl	No problen	Big probler	No problen	No problen	No problen
	Big probler	Small probl	Big probler	Big probler	Big probler	No problen	Small probl	Small probl
	No problen	No problen	Small probl	Small probl	Big probler	Small probl	No problen	No problen
	No problen	No problen	No problen	No problen	No problen	No problen	Small probl	No problen
	No problen	No problen	Small probl	Small probl	Big probler	No problen	No problen	No problen
No	Small probl	No problen	No problen	Small probl	Small probl	Small probl	No problen	No problen
	No problen	No problen	No problen	Small probl	Small probl	No problen	Small probl	Small probl

For peer review only

	No problen	No problen	Small probl	Big probler	Big probler	Small probl	No problen	Small probl
	Big probler	Small probl	Big probler	Big probler	Big probler	Small probl	No problen	No problen
	No problen	No problen	Small probl	Big probler	Big probler	Small probl	No problen	Small probl
	Big probler	Small probl	Big probler	Big probler	Big probler	Small probl	No problen	No problen
	Big probler	Small probl	Big probler	Big probler	Big probler	No problen	No problen	No problen
	No problen	No problen	Big probler	Big probler	Big probler	No problen	Big probler	Small probl
	No problen	Big probler	Big probler	Big probler	Big probler	Big probler	No problen	Big probler
No	No problen	No problen	No problen	No problen	No problen	No problen	No problen	No problen
Yes	No problen	No problen	Small probl	Small probl	Big probler	Small probl	No problen	Small probl
	Big probler	No problen	Big probler	Big probler	Big probler	No problen	Small probl	No problen
	Big probler	Small probl	Big probler	Big probler	Big probler	Small probl	No problen	No problen
	No problen	No problen	Big probler	No problen	Big probler	Small probl	No problen	No problen
No	No problen	No problen	Big probler	Small probl	Big probler	Big probler	Small probl	Small probl
	No problen	No problen	No problen	No problen	Small probl	No problen	No problen	Small probl
	No problen	No problen	Big probler	Small probl	Big probler	No problen	Small probl	No problen
	No problen	No problen	Small probl	Big probler	Small probl	No problen	Small probl	Small probl
	No problen	Small probl	Small probl	Small probl	Small probl	No problen	Small probl	No problen
No	No problen	No problen	No problen	No problen	Small probl	Small probl	No problen	No problen
	No problen	No problen	Small probl	Small probl	Big probler	No problen	No problen	No problen
	No problen	No problen	No problen	Big probler	Big probler	No problen	Big probler	Small probl
No	No problen	No problen	Small probl	Small probl	Big probler	Big probler	Small probl	Small probl
	No problen	Small probl	Small probl	No problen	Big probler	Small probl	No problen	No problen
	No problen	Small probl	Big probler	Big probler	Big probler	Big probler	Small probl	No problen
	No problen	No problen	No problen	Big probler	Big probler	No problen	Small probl	Small probl
	Small probl	Small probl	Big probler	Big probler	Big probler	Small probl	No problen	Small probl
	No problen	No problen	No problen	Small probl	No problen	No problen	Small probl	Small probl
No	No problen	No problen	No problen	No problen	No problen	No problen	No problen	No problen
	Big probler	Small probl	Big probler	Big probler	Big probler	Small probl	Small probl	No problen
	No problen	No problen	Small probl	Big probler	Big probler	No problen	Small probl	Small probl
No	No problen	No problen	Small probl	Small probl	Small probl	No problen	No problen	Small probl
Yes	No problen	No problen	Small probl	No problen	Small probl	No problen	No problen	No problen
	No problen	No problen	Small probl	Small probl	Small probl	No problen	Small probl	Small probl
	No problen	No problen	Small probl	No problen	Small probl	No problen	No problen	No problen
No	No problen	No problen	Small probl	Small probl	Big probler	Small probl	No problen	Small probl
	Small probl	Small probl	No problen	No problen	Small probl	No problen	No problen	No problen
	Small probl	No problen	Small probl	No problen	Big probler	No problen	Big probler	Small probl
	Big probler	Small probl	Big probler	Big probler	Big probler	Small probl	No problen	No problen
	No problen	No problen	Small probl	Small probl	Small probl	Small probl	Small probl	Small probl
	Big probler	Small probl	Big probler	Big probler	Big probler	Small probl	No problen	No problen
	No problen	Small probl	Big probler	Big probler	Big probler	Small probl	No problen	No problen
No	No problen	No problen	No problen	No problen	No problen	No problen	No problen	No problen
No	No problen	No problen	Big probler	No problen	Big probler	Small probl	No problen	No problen
	Big probler	Small probl	Big probler	Big probler	Big probler	Small probl	No problen	No problen
	No problen	No problen	Small probl	Small probl	Small probl	No problen	Small probl	No problen
	No problen	No problen	Big probler	Big probler	Big probler	No problen	Small probl	Small probl
	No problen	No problen	Big probler	Big probler	Big probler	Small probl	No problen	Small probl
	No problen	Small probl	Small probl	No problen	Small probl	No problen	No problen	No problen
	No problen	No problen	No problen	Small probl	Small probl	No problen	No problen	No problen
No	No problen	No problen	No problen	No problen	No problen	No problen	No problen	No problen
Yes	No problen	No problen	Small probl	Small probl	Big probler	Small probl	No problen	Small probl
	No problen	Small probl	Big probler	Big probler	Small probl	No problen	Big probler	Small probl
	Small probl	Small probl	Big probler	Big probler	Big probler	No problen	Big probler	Small probl

For peer review only

	No problen	No problen	Big probler	Small probl	Big probler	Big probler	Small probl	Small probl
	No problen	No problen	No problen	No problen	No problen	No problen	No problen	Small probl
	Small probl	No problen	Small probl	No problen	Small probl	Small probl	No problen	No problen
	No problen	No problen	Small probl	Big probler	Big probler	Small probl	No problen	No problen
	No problen	No problen	Small probl	Small probl	Small probl	No problen	No problen	No problen
Yes	No problen	No problen	Small probl	No problen	Small probl	Small probl	No problen	Small probl
	No problen	No problen	Small probl	Big probler	Big probler	No problen	No problen	Small probl
	Big probler	Small probl	Big probler	Big probler	Big probler	No problen	No problen	No problen
	Big probler	Small probl	Big probler	Big probler	Big probler	Small probl	Small probl	Small probl
	No problen	No problen	Small probl	Big probler	Big probler	Small probl	No problen	No problen
	No problen	No problen	No problen	Small probl	Small probl	No problen	Small probl	Small probl
	Small probl	No problen	Big probler	Big probler	Big probler	Small probl	No problen	No problen
No	No problen	No problen	No problen	No problen	No problen	No problen	No problen	No problen
	Small probl	Small probl	Small probl	No problen	Big probler	No problen	No problen	Small probl
	No problen	No problen	No problen	No problen	Small probl	No problen	Small probl	No problen
	No problen	No problen	No problen	Small probl	Big probler	No problen	No problen	No problen
	No problen	No problen	Small probl	Big probler	Big probler	No problen	Big probler	Small probl
	No problen	No problen	Small probl	Big probler	Big probler	Small probl	No problen	No problen
	No problen	No problen	Small probl	Big probler	Big probler	Small probl	No problen	No problen
No	Big probler	Big probler	Big probler	Big probler	Big probler	Small probl	No problen	No problen
	Big probler	Small probl	Big probler	Big probler	Big probler	No problen	No problen	No problen
No	No problen	No problen	Small probl	Big probler	Big probler	No problen	No problen	No problen
No	No problen	No problen	No problen	No problen	No problen	No problen	Small probl	Small probl
	Big probler	Small probl	Big probler	Big probler	Big probler	No problen	No problen	No problen
	Small probl	Small probl	No problen	Small probl	Big probler	Small probl	Small probl	No problen
	Small probl	Small probl	Small probl	Small probl	Big probler	Big probler	Small probl	Small probl
	Big probler	Small probl	Big probler	Big probler	Big probler	Small probl	No problen	No problen
	No problen	No problen	No problen	No problen	No problen	No problen	No problen	No problen
	No problen	No problen	No problen	Small probl	Small probl	No problen	Small probl	Small probl
	No problen	Small probl	Big probler	Small probl	Big probler	Big probler	Big probler	Small probl
	No problen	No problen	No problen	Small probl	No problen	No problen	Small probl	Small probl
	No problen	No problen	No problen	Small probl	Small probl	No problen	No problen	No problen
	Big probler	Small probl	Big probler	Big probler	Big probler	Small probl	No problen	No problen
No	No problen	No problen	No problen	Small probl	Small probl	Small probl	Small probl	Small probl
	Big probler	Small probl	Big probler	Big probler	Big probler	Small probl	No problen	No problen
	No problen	No problen	Small probl	Small probl	Small probl	No problen	No problen	Small probl
	Small probl	Big probler	Small probl	Small probl	Big probler	Small probl	Small probl	Big probler
	No problen	No problen	No problen	Big probler	Big probler	No problen	No problen	No problen
	No problen	No problen	No problen	Big probler	Big probler	No problen	No problen	Big probler
	No problen	No problen	No problen	No problen	Small probl	No problen	No problen	No problen
	No problen	No problen	Small probl	Small probl	Small probl	Small probl	Small probl	No problen
No	No problen	No problen	Small probl	Big probler	Big probler	No problen	No problen	No problen
	No problen	No problen	No problen	Big probler	Big probler	Small probl	Small probl	No problen
No	No problen	No problen	Big probler	Big probler	Big probler	No problen	No problen	No problen
	No problen	No problen	Small probl	Small probl	Small probl	No problen	No problen	No problen
	No problen	No problen	Small probl	Small probl	Small probl	No problen	No problen	No problen
	No problen	No problen	No problen	No problen	No problen	No problen	No problen	No problen
	No problen	No problen	No problen	No problen	No problen	No problen	No problen	No problen
	No problen	No problen	No problen	Big probler	Big probler	No problen	No problen	No problen
	No problen	No problen	Small probl	Big probler	Big probler	No problen	No problen	No problen
	No problen	No problen	No problen	No problen	No problen	No problen	No problen	No problen
	No problen	No problen	No problen	Small probl	Small probl	No problen	No problen	No problen

For peer review only

	No problen	No problen	No problen	No problen	Small probl	No problen	No problen	No problen
No	No problen	No problen	No problen	Big probler	Big probler	No problen	No problen	No problen
	No problen	No problen	No problen	No problen	No problen	No problen	No problen	No problen
	No problen	No problen	No problen	Big probler	Big probler	No problen	No problen	No problen
	No problen	No problen	Small probl	Big probler	Big probler	Small probl	No problen	No problen
	No problen	No problen	Small probl	Big probler	Big probler	No problen	No problen	No problen
	No problen	No problen	No problen	Big probler	Big probler	No problen	No problen	No problen
	No problen	No problen	Big probler	Big probler	Big probler	Big probler	Big probler	Big probler
	No problen	No problen	No problen	No problen	No problen	No problen	No problen	No problen
	No problen	No problen	No problen	Big probler	Big probler	No problen	No problen	No problen
	No problen	No problen	Big probler	Big probler	Big probler	No problen	No problen	No problen
	No problen	No problen	No problen	Small probl	Small probl	No problen	No problen	No problen
	No problen	No problen	Small probl	Small probl	Small probl	No problen	No problen	No problen
	No problen	No problen	No problen	No problen	No problen	No problen	No problen	No problen
	No problen	No problen	No problen	Big probler	Big probler	No problen	No problen	No problen
No	No problen	No problen	Small probl	Big probler	Big probler	No problen	No problen	No problen
	No problen	No problen	No problen	Big probler	Big probler	No problen	No problen	No problen
	No problen	No problen	No problen	Big probler	Big probler	No problen	No problen	No problen
No	No problen	No problen	Small probl	Big probler	Big probler	No problen	No problen	No problen
	No problen	No problen	No problen	No problen	No problen	No problen	No problen	No problen
	No problen	No problen	Small probl	Big probler	Big probler	No problen	No problen	No problen
	No problen	No problen	No problen	No problen	No problen	No problen	No problen	No problen
	No problen	No problen	No problen	Big probler	Big probler	No problen	No problen	No problen
	No problen	No problen	Small probl	Big probler	Big probler	No problen	No problen	No problen
	No problen	No problen	Big probler	Big probler	Big probler	No problen	Small probl	No problen
	No problen	No problen	No problen	No problen	No problen	No problen	No problen	No problen
	No problen	No problen	No problen	No problen	Small probl	No problen	No problen	No problen
No	No problen	No problen	Small probl	Small probl	Small probl	No problen	No problen	Small probl
	No problen	No problen	No problen	No problen	Small probl	No problen	No problen	No problen
	No problen	No problen	No problen	No problen	Small probl	No problen	No problen	No problen
	No problen	No problen	No problen	Big probler	Big probler	Small probl	No problen	No problen
	No problen	No problen	Big probler	Big probler	Big probler	No problen	No problen	No problen
	No problen	No problen	No problen	Big probler	Big probler	No problen	No problen	No problen
	No problen	No problen	No problen	No problen	Small probl	No problen	No problen	No problen
	No problen	No problen	No problen	No problen	Small probl	No problen	No problen	No problen
	No problen	No problen	No problen	Big probler	Big probler	No problen	No problen	No problen
	No problen	No problen	No problen	Big probler	Big probler	Big probler	No problen	No problen
No	No problen	No problen	Small probl	Big probler	Small probl	No problen	No problen	No problen
	No problen	No problen	No problen	No problen	Small probl	No problen	No problen	No problen
	No problen	No problen	No problen	No problen	Small probl	No problen	No problen	No problen
	No problen	No problen	No problen	Big probler	Big probler	Big probler	No problen	No problen
No	No problen	No problen	No problen	Small probl	Small probl	No problen	No problen	No problen
	No problen	No problen	Small probl	Big probler	Big probler	No problen	No problen	No problen
	No problen	No problen	Small probl	Big probler	Big probler	Small probl	No problen	No problen
	No problen	No problen	No problen	No problen	Small probl	No problen	No problen	No problen
	No problen	Small probl	Small probl	Small probl	Small probl	No problen	No problen	No problen
Yes	No problen	No problen	No problen	Big probler	Big probler	No problen	No problen	No problen
	Small probl	Small probl	Small probl	Small probl	Small probl	Small probl	Small probl	Small probl
	No problen	No problen	No problen	No problen	No problen	No problen	No problen	No problen
No	No problen	No problen	No problen	No problen	No problen	No problen	No problen	No problen
	No problen	No problen	No problen	No problen	No problen	No problen	No problen	No problen
	No problen	No problen	No problen	No problen	No problen	No problen	No problen	No problen

For peer review only

For peer review only - http://bmjopen.bmj.com/site/about/guidelines.xhtml

	No problen	Big probler	Small probl	No problen	No problen	No problen	No problen	No problen
	No problen	No problen	Small probl	No problen	No problen	No problen	No problen	No problen
	No problen	No problen	No problen	No problen	No problen	No problen	No problen	No problen
	No problen	No problen	Big probler	Small probl	No problen	No problen	No problen	No problen
	No problen	No problen	No problen	No problen	No problen	No problen	No problen	No problen
	No problen	No problen	No problen	No problen	No problen	No problen	No problen	No problen
	No problen	No problen	No problen	No problen	No problen	No problen	No problen	No problen
	No problen	No problen	No problen	No problen	No problen	No problen	No problen	No problen
	Small probl	Small probl	Small probl	Small probl	Small probl	Small probl	Small probl	Small probl
	No problen	No problen	No problen	No problen	No problen	No problen	No problen	No problen
	No problen	No problen	No problen	No problen	No problen	No problen	No problen	No problen
	No problen	No problen	Small probl	No problen	No problen	No problen	No problen	No problen
	No problen	No problen	No problen	No problen	No problen	No problen	No problen	No problen
	No problen	No problen	No problen	No problen	No problen	No problen	No problen	Small probl
	No problen	No problen	Big probler	No problen	No problen	Small probl	No problen	No problen
	No problen	No problen	No problen	No problen	No problen	No problen	No problen	No problen
No	No problen	No problen	Big probler	No problen	No problen	No problen	No problen	No problen
	No problen	No problen	No problen	No problen	No problen	No problen	No problen	No problen
	No problen	No problen	No problen	No problen	No problen	No problen	No problen	No problen
	No problen	No problen	No problen	No problen	No problen	No problen	No problen	No problen
No	No problen	No problen	No problen	No problen	No problen	No problen	No problen	No problen
No	No problen	No problen	No problen	No problen	No problen	No problen	No problen	No problen
Yes	No problen	No problen	No problen	No problen	No problen	No problen	No problen	No problen
	No problen	No problen	Small probl	No problen	No problen	No problen	No problen	No problen
	No problen	No problen	No problen	No problen	No problen	No problen	No problen	No problen
	No problen	No problen	No problen	No problen	No problen	No problen	No problen	No problen
	Small probl	Small probl	Small probl	Small probl	Small probl	Small probl	Small probl	Small probl
	No problen	No problen	No problen	No problen	No problen	No problen	No problen	No problen
	No problen	No problen	No problen	No problen	No problen	No problen	No problen	No problen
	No problen	No problen	No problen	No problen	No problen	No problen	No problen	No problen
	No problen	No problen	No problen	No problen	No problen	No problen	No problen	No problen
	No problen	No problen	Big probler	No problen	No problen	No problen	No problen	No problen
	No problen	No problen	No problen	No problen	No problen	No problen	No problen	No problen
	Small probl	Small probl	Small probl	Small probl	Small probl	Small probl	Small probl	Small probl
	No problen	No problen	No problen	No problen	No problen	No problen	No problen	No problen
	Small probl	Small probl	Small probl	Small probl	Small probl	Small probl	Small probl	Small probl
	No problen	No problen	No problen	No problen	No problen	No problen	No problen	No problen
	No problen	No problen	No problen	No problen	No problen	No problen	No problen	No problen
	No problen	No problen	No problen	No problen	No problen	No problen	No problen	No problen
	No problen	No problen	No problen	No problen	No problen	No problen	No problen	No problen
	No problen	No problen	No problen	No problen	No problen	No problen	No problen	No problen
	No problen	No problen	No problen	No problen	No problen	No problen	No problen	No problen
	No problen	No problen	No problen	No problen	No problen	No problen	No problen	No problen
	No problen	No problen	No problen	No problen	No problen	No problen	No problen	No problen
	No problen	No problen	No problen	No problen	No problen	No problen	No problen	No problen
	No problen	No problen	No problen	No problen	No problen	No problen	No problen	No problen
	No problen	No problen	No problen	No problen	No problen	No problen	No problen	No problen
	No problen	No problen	No problen	No problen	No problen	No problen	No problen	No problen
No	No problen	Big probler	Big probler	No problen	No problen	No problen	No problen	No problen
	No problen	No problen	No problen	No problen	No problen	No problen	No problen	No problen
	No problen	No problen	No problen	No problen	Small probl	No problen	Small probl	Small probl
	No problen	No problen	No problen	No problen	No problen	No problen	No problen	No problen

For peer review only

For peer review only - http://bmjopen.bmj.com/site/about/guidelines.xhtml

	No problen	No problen	Small probl	No problen	No problen	No problen	No problen	No problen
	No problen	No problen	Big probler	No problen	Small probl	No problen	No problen	No problen
	No problen	No problen	No problen	No problen	No problen	No problen	No problen	No problen
	Small probl	Small probl	Small probl	Small probl	Small probl	Small probl	Small probl	Small probl
No	No problen	No problen	Big probler	No problen	No problen	No problen	No problen	No problen
	No problen	No problen	Small probl	No problen	No problen	No problen	No problen	No problen
	No problen	No problen	Small probl	Small probl	Small probl	No problen	No problen	Small probl
	No problen	No problen	No problen	No problen	No problen	No problen	No problen	No problen
	No problen	No problen	No problen	No problen	No problen	No problen	No problen	No problen
No	Small probl	Small probl	Small probl	No problen	Small probl	No problen	No problen	No problen
	No problen	No problen	No problen	No problen	No problen	No problen	No problen	No problen
	No problen	No problen	No problen	No problen	No problen	No problen	No problen	No problen
	No problen	No problen	Big probler	No problen	No problen	No problen	No problen	No problen
	No problen	No problen	No problen	No problen	No problen	No problen	No problen	No problen
Yes	No problen	No problen	No problen	No problen	No problen	No problen	No problen	No problen
No	No problen	No problen	Small probl	No problen	No problen	No problen	No problen	No problen
	No problen	No problen	No problen	No problen	No problen	No problen	No problen	No problen
	No problen	No problen	No problen	No problen	No problen	No problen	No problen	No problen
	No problen	No problen	Small probl	No problen	No problen	No problen	No problen	No problen
	No problen	No problen	Small probl	No problen	No problen	No problen	No problen	No problen
	No problen	No problen	No problen	No problen	No problen	No problen	No problen	No problen
	No problen	No problen	No problen	No problen	No problen	No problen	No problen	No problen
	No problen	No problen	Small probl	Small probl	No problen	No problen	No problen	No problen
	No problen	No problen	No problen	No problen	No problen	No problen	No problen	No problen
	No problen	No problen	Big probler	Small probl	No problen	No problen	No problen	No problen
	No problen	No problen	No problen	No problen	No problen	No problen	No problen	No problen
	No problen	No problen	No problen	No problen	No problen	No problen	No problen	No problen
No	No problen	No problen	Big probler	No problen	No problen	No problen	No problen	No problen
	No problen	No problen	No problen	No problen	No problen	No problen	No problen	No problen
	No problen	No problen	Big probler	No problen	No problen	No problen	No problen	No problen
	No problen	No problen	No problen	No problen	No problen	No problen	No problen	No problen
	No problen	No problen	Big probler	No problen	No problen	No problen	No problen	No problen
	Small probl	Small probl	Small probl	Small probl	Small probl	Small probl	Small probl	Small probl
	No problen	No problen	No problen	No problen	No problen	No problen	No problen	No problen
	No problen	No problen	No problen	No problen	No problen	No problen	No problen	No problen
	No problen	No problen	No problen	No problen	No problen	No problen	No problen	No problen
No	No problen	No problen	No problen	No problen	No problen	No problen	No problen	No problen
	No problen	No problen	Small probl	No problen	No problen	No problen	Small probl	No problen
No	No problen	No problen	No problen	No problen	No problen	No problen	No problen	No problen
	No problen	No problen	No problen	No problen	No problen	No problen	No problen	No problen
	No problen	No problen	No problen	No problen	No problen	No problen	No problen	No problen
No	No problen	No problen	Small probl	Small probl	Small probl	Small probl	Big probler	Big probler
	No problen	Small probl	No problen	No problen	No problen	No problen	No problen	No problen
	No problen	No problen	No problen	No problen	No problen	No problen	No problen	No problen
No	No problen	No problen	No problen	No problen	Small probl	No problen	No problen	No problen
	No problen	No problen	No problen	No problen	No problen	No problen	No problen	No problen
	No problen	No problen	No problen	No problen	No problen	No problen	No problen	No problen
No	No problen	No problen	No problen	No problen	No problen	No problen	No problen	No problen
No	No problen	No problen	No problen	No problen	No problen	No problen	No problen	No problen
	No problen	No problen	No problen	No problen	No problen	No problen	No problen	No problen
	No problen	No problen	No problen	No problen	No problen	No problen	No problen	No problen
	Small probl	Small probl	Small probl	Small probl	Small probl	Small probl	Small probl	Small probl

For peer review only

	No problen	No problen	No problen	No problen	No problen	No problen	No problen	No problen
	No problen	No problen	No problen	No problen	No problen	No problen	No problen	No problen
	No problen	No problen	No problen	No problen	No problen	No problen	No problen	No problen
	No problen	No problen	No problen	No problen	No problen	No problen	No problen	No problen
No	No problen	No problen	No problen	No problen	No problen	No problen	No problen	No problen
Yes	No problen	No problen	No problen	No problen	No problen	No problen	No problen	No problen
Yes	No problen	No problen	No problen	No problen	No problen	No problen	No problen	No problen
	No problen	No problen	No problen	No problen	No problen	No problen	No problen	No problen
	No problen	No problen	No problen	No problen	No problen	No problen	No problen	No problen
	Small probl	No problen	Small probl	Small probl	Small probl	Small probl	No problen	No problen
	No problen	No problen	No problen	No problen	No problen	No problen	No problen	No problen
	No problen	No problen	No problen	No problen	No problen	No problen	No problen	No problen
	Small probl	Small probl	Small probl	Small probl	Small probl	Small probl	Small probl	Small probl
	No problen	No problen	No problen	No problen	No problen	No problen	No problen	No problen
	No problen	No problen	No problen	No problen	No problen	No problen	No problen	No problen
No	Big probler	No problen	No problen	No problen	Small probl	No problen	No problen	No problen
	No problen	No problen	No problen	No problen	No problen	No problen	No problen	No problen
	No problen	No problen	No problen	No problen	No problen	No problen	No problen	No problen
	No problen	No problen	No problen	No problen	No problen	No problen	No problen	No problen
	Small probl	Small probl	Small probl	Small probl	Small probl	Small probl	Small probl	Small probl
	No problen	No problen	No problen	No problen	No problen	No problen	No problen	No problen
	No problen	No problen	No problen	No problen	No problen	No problen	No problen	No problen
	No problen	No problen	No problen	No problen	No problen	No problen	No problen	No problen
	No problen	No problen	No problen	No problen	No problen	No problen	Small probl	Small probl
	No problen	No problen	Small probl	No problen	No problen	No problen	No problen	No problen
No	No problen	No problen	No problen	No problen	No problen	No problen	No problen	No problen
	No problen	No problen	No problen	No problen	No problen	No problen	No problen	No problen
	No problen	No problen	No problen	No problen	No problen	No problen	No problen	No problen
	No problen	No problen	No problen	No problen	No problen	No problen	No problen	No problen
	No problen	No problen	No problen	No problen	No problen	No problen	No problen	No problen
	No problen	No problen	Small probl	No problen	No problen	No problen	No problen	No problen
	No problen	No problen	No problen	No problen	No problen	No problen	No problen	No problen
	No problen	No problen	Small probl	Big probler	Big probler	Big probler	No problen	No problen
	No problen	No problen	No problen	Big probler	No problen	No problen	No problen	No problen
	No problen	No problen	No problen	No problen	No problen	No problen	No problen	No problen
	Small probl	Big probler	Big probler	Big probler	Big probler	Small probl	Big probler	Small probl
	No problen	No problen	No problen	No problen	No problen	No problen	No problen	No problen
	No problen	No problen	No problen	Small probl	Small probl	No problen	Small probl	Small probl
	Big probler	Big probler	Small probl	Big probler	Small probl	Big probler	Small probl	Big probler
	No problen	No problen	No problen	No problen	No problen	No problen	No problen	No problen
	Small probl	Big probler	Small probl	Small probl	No problen	Small probl	Small probl	Big probler
	No problen	No problen	No problen	No problen	Small probl	Small probl	Small probl	Big probler
	No problen	No problen	Big probler	No problen	Small probl	Small probl	Small probl	Small probl
	Small probl	Small probl	No problen	Small probl	Big probler	Small probl	Small probl	Small probl
	No problen	No problen	Small probl	Big probler	Big probler	Big probler	No problen	Small probl
Yes	No problen	No problen	No problen	No problen	No problen	No problen	No problen	No problen
Yes	No problen	No problen	No problen	No problen	No problen	No problen	No problen	No problen
	Big probler	Big probler	Big probler	Big probler	Big probler	Big probler	No problen	No problen
	No problen	Small probl	Small probl	No problen	Big probler	No problen	No problen	No problen
	No problen	No problen	No problen	Small probl	No problen	No problen	No problen	No problen
Yes	No problen	No problen	Small probl	Big probler	Big probler	Small probl	Small probl	Small probl
	No problen	No problen	No problen	Small probl	Big probler	Big probler	No problen	Small probl

For peer review only

	No problen	No problen	No problen	No problen	No problen	No problen	No problen	No problen
	Small probl	No problen	No problen	No problen	Small probl	No problen	No problen	No problen
	No problen	Big probler	Big probler	Big probler	Big probler	Big probler	No problen	No problen
	No problen	No problen	Small probl	No problen	No problen	No problen	No problen	No problen
	Small probl	Small probl	Big probler	Small probl	Big probler	Big probler	Small probl	Small probl
	Small probl	Small probl	Big probler	Big probler	Big probler	Big probler	No problen	No problen
	Big probler	Small probl	Big probler	Big probler	Big probler	Big probler	Small probl	Small probl
No	No problen	Small probl	Small probl	Big probler	Big probler	Small probl	Small probl	Small probl
	No problen	No problen	No problen	No problen	No problen	No problen	No problen	No problen
	No problen	No problen	Small probl	No problen	Small probl	No problen	No problen	No problen
	Small probl	Small probl	Small probl	Small probl	Big probler	Small probl	Small probl	Small probl
No	No problen	No problen	No problen	No problen	No problen	No problen	No problen	No problen
	Small probl	Big probler	Big probler	Big probler	Big probler	Big probler	No problen	Small probl
No	Small probl	No problen	No problen	No problen	Small probl	No problen	No problen	Small probl
	Small probl	No problen	No problen	No problen	Big probler	Small probl	No problen	No problen
	No problen	No problen	Small probl	No problen	No problen	No problen	No problen	No problen
	No problen	No problen	No problen	No problen	No problen	No problen	No problen	No problen
	Small probl	Small probl	Big probler	Big probler	Big probler	Small probl	Small probl	No problen
	No problen	Small probl	Small probl	Small probl	Big probler	Small probl	Small probl	No problen
	No problen	No problen	Small probl	No problen	Small probl	No problen	No problen	No problen
No	No problen	No problen	No problen	Small probl	No problen	No problen	No problen	No problen
	No problen	No problen	Big probler	Small probl	Big probler	Small probl	No problen	No problen
	Small probl	Small probl	Small probl	Small probl	Big probler	Small probl	Big probler	Small probl
	No problen	Small probl	Big probler	No problen	No problen	No problen	Small probl	No problen
	No problen	No problen	No problen	No problen	No problen	No problen	No problen	No problen
No	No problen	No problen	Big probler	Small probl	Small probl	No problen	Small probl	Small probl
	No problen	No problen	No problen	No problen	No problen	No problen	No problen	No problen
	No problen	No problen	Big probler	No problen	Big probler	Big probler	Small probl	Small probl
	No problen	No problen	No problen	No problen	No problen	No problen	No problen	No problen
	No problen	No problen	No problen	No problen	No problen	No problen	No problen	No problen
Yes	Small probl	Big probler	Small probl	Big probler	Big probler	Small probl	Small probl	Small probl
	No problen	No problen	No problen	No problen	No problen	No problen	No problen	No problen
	No problen	No problen	No problen	No problen	Small probl	No problen	No problen	No problen
	No problen	No problen	No problen	No problen	No problen	No problen	No problen	No problen
	Small probl	Big probler	Small probl	Big probler	Big probler	Big probler	Small probl	No problen
	No problen	No problen	Small probl	Big probler	Big probler	No problen	Small probl	Small probl
No	No problen	No problen	Big probler	No problen	Small probl	No problen	No problen	No problen
	Small probl	Big probler	Small probl	Big probler	Big probler	Big probler	Small probl	Big probler
	No problen	No problen	No problen	No problen	No problen	No problen	No problen	No problen
	No problen	No problen	Small probl	No problen	No problen	No problen	No problen	No problen
	No problen	No problen	No problen	No problen	No problen	No problen	No problen	No problen
	No problen	No problen	No problen	No problen	No problen	No problen	No problen	No problen
	No problen	No problen	Small probl	No problen	No problen	No problen	No problen	No problen
	No problen	No problen	No problen	Small probl	No problen	No problen	No problen	No problen
	No problen	No problen	No problen	No problen	No problen	No problen	No problen	No problen
	Small probl	No problen	No problen	Big probler	Big probler	Small probl	Small probl	Small probl
	No problen	No problen	Small probl	No problen	Small probl	No problen	No problen	No problen
	No problen	No problen	Small probl	Small probl	Big probler	Big probler	No problen	No problen
	No problen	No problen	No problen	Big probler	Big probler	No problen	No problen	No problen
	Small probl	Small probl	Small probl	Small probl	Big probler	Small probl	Small probl	Small probl
	No problen	Small probl	Big probler	Small probl	Big probler	Big probler	No problen	No problen
	No problen	No problen	No problen	No problen	No problen	No problen	No problen	No problen

For peer review only

No	No problen	No problen	Small probl	Big probler	Big probler	No problen	Big probler	Small probl
	No problen	No problen	Small probl	No problen	No problen	No problen	No problen	No problen
	No problen	No problen	Small probl	No problen	No problen	No problen	No problen	No problen
	No problen	Small probl	Big probler	Big probler	Big probler	Big probler	No problen	No problen
	No problen	Small probl	Big probler	Small probl	Small probl	Small probl	Small probl	Small probl
	No problen	No problen	No problen	No problen	No problen	No problen	No problen	No problen
	No problen	Small probl	Big probler	Big probler	Big probler	Big probler	No problen	Small probl
	No problen	No problen	No problen	No problen	No problen	No problen	No problen	No problen
No	No problen	No problen	No problen	No problen	Big probler	No problen	Small probl	No problen
	Small probl	Small probl	Small probl	No problen	Big probler	Small probl	No problen	Small probl
No	No problen	No problen	No problen	No problen	No problen	No problen	No problen	No problen
No	No problen	No problen	No problen	Big probler	Big probler	No problen	Big probler	Small probl
	No problen	No problen	Small probl	Small probl	Small probl	Small probl	Small probl	Small probl
	No problen	No problen	No problen	No problen	No problen	No problen	No problen	No problen
	No problen	No problen	No problen	No problen	No problen	No problen	No problen	No problen
	Small probl	No problen	No problen	Small probl	Big probler	Big probler	No problen	No problen
	Small probl	Small probl	Big probler	Small probl	Big probler	Small probl	No problen	No problen
No	No problen	No problen	No problen	No problen	No problen	No problen	No problen	Small probl
	Small probl	No problen	No problen	Big probler	Big probler	Big probler	No problen	No problen
	No problen	No problen	No problen	No problen	No problen	No problen	No problen	No problen
Yes	No problen	Small probl	Big probler	Small probl	Big probler	Small probl	Small probl	Small probl
	No problen	No problen	Small probl	Big probler	Big probler	Big probler	No problen	No problen
	No problen	No problen	No problen	No problen	No problen	No problen	No problen	No problen
Yes	No problen	No problen	Small probl	No problen	No problen	No problen	No problen	No problen
No	No problen	No problen	No problen	No problen	Small probl	No problen	No problen	Small probl
No	Small probl	Small probl	Big probler	Small probl	Big probler	No problen	No problen	Small probl
No	No problen	No problen	Small probl	Small probl	Small probl	Small probl	Small probl	Small probl
Yes	Big probler	Small probl	Big probler	Big probler	Big probler	Small probl	Small probl	No problen
	No problen	No problen	No problen	No problen	No problen	No problen	No problen	No problen
No	Small probl	No problen	Big probler	Big probler	Big probler	Small probl	No problen	Big probler
No	Big probler	Big probler	Big probler	Small probl	Big probler	Big probler	Small probl	Small probl
No	Small probl	Small probl	Big probler	No problen	Big probler	No problen	No problen	No problen
No	Small probl	Big probler	Big probler	Big probler	Big probler	Big probler	Big probler	Big probler
Yes	Small probl	No problen	Small probl	Small probl	Small probl	Small probl	Small probl	Small probl
No	No problen	Small probl	Big probler	Small probl	Big probler	Small probl	Small probl	Small probl
	Small probl	No problen	No problen	No problen	No problen	No problen	No problen	No problen
	Small probl	No problen	No problen	No problen	Small probl	Small probl	Small probl	Small probl
No	Small probl	Small probl	Big probler	Small probl	Big probler	No problen	No problen	Small probl
No	Small probl	Small probl	Big probler	Small probl	Big probler	No problen	No problen	Big probler
	Small probl	Small probl	Small probl	Small probl	Small probl	Small probl	No problen	No problen
	Small probl	Small probl	Small probl	Small probl	Small probl	Small probl	Small probl	Small probl
	Small probl	Small probl	Big probler	Big probler	Big probler	Small probl	No problen	Small probl
	Big probler	No problen	Big probler	Big probler	Big probler	Small probl	No problen	Small probl
	Small probl	Small probl	Small probl	Small probl	Small probl	Small probl	Small probl	Small probl
No	Small probl	Small probl	Big probler	Small probl	Big probler	No problen	No problen	Big probler
	Small probl	Small probl	Small probl	Small probl	Small probl	Small probl	Small probl	Small probl
No	Big probler	Big probler	Big probler	Big probler	Big probler	Small probl	Small probl	No problen
	No problen	No problen	No problen	No problen	Small probl	No problen	No problen	Small probl
No	Big probler	Big probler	Big probler	Big probler	Big probler	Small probl	No problen	No problen
Yes	Small probl	No problen	Big probler	Small probl	Big probler	Small probl	Small probl	Small probl
	Small probl	Small probl	Big probler	Big probler	Big probler	Big probler	Big probler	Big probler
No	No problen	Small probl	Big probler	Small probl	Big probler	Big probler	Small probl	Big probler

For peer review only

No	No problen	No problen	No problen	Small probl	Big probler	No problen	No problen	No problen
	Small probl	Small probl	Small probl	Small probl	Small probl	Small probl	Small probl	Small probl
	No problen	No problen	Small probl	Small probl	Small probl	Small probl	Small probl	Small probl
No	Small probl	Small probl	Big probler	Big probler	Big probler	Big probler	Small probl	Small probl
	Small probl	Small probl	Small probl	No problen	Small probl	Small probl	Small probl	Small probl
No	Big probler	No problen	No problen	Big probler	Big probler	Big probler	No problen	No problen
	No problen	No problen	No problen	No problen	No problen	No problen	No problen	No problen
	No problen	No problen	Small probl	No problen	Small probl	No problen	No problen	No problen
	Small probl	Small probl	Small probl	Small probl	Small probl	Small probl	Small probl	Small probl
	Small probl	No problen	Big probler	Big probler	Big probler	Small probl	No problen	No problen
Yes	No problen	Small probl	Big probler	Small probl	Big probler	Big probler	Small probl	No problen
	Small probl	Small probl	Small probl	Small probl	Small probl	Small probl	Small probl	Small probl
No	Big probler	Small probl	Big probler	Big probler	Big probler	Small probl	Small probl	Small probl
	No problen	No problen	No problen	No problen	No problen	No problen	No problen	No problen
Yes	No problen	No problen	No problen	No problen	No problen	No problen	No problen	No problen
Yes	Small probl	Small probl	Big probler	Small probl	Big probler	Big probler	No problen	No problen
	No problen	No problen	No problen	No problen	Big probler	Big probler	Small probl	Big probler
No	No problen	No problen	Small probl	Big probler	Big probler	Small probl	No problen	No problen
	Small probl	Small probl	Small probl	Small probl	Small probl	Small probl	Small probl	Small probl
	Small probl	Small probl	Big probler	Small probl	Big probler	No problen	No problen	Small probl
	Small probl	Small probl	Small probl	Small probl	Small probl	Small probl	Small probl	Small probl
	Small probl	Small probl	Small probl	Small probl	Small probl	Small probl	Small probl	Small probl
No	Big probler	Small probl	Big probler	Big probler	Big probler	Big probler	Big probler	Big probler
	Small probl	Small probl	Small probl	Small probl	Small probl	Small probl	No problen	No problen
No	No problen	No problen	No problen	Small probl	Small probl	No problen	No problen	No problen
No	No problen	Small probl	Big probler	Small probl	Big probler	Small probl	No problen	Small probl
	No problen	No problen	No problen	Big probler	Big probler	No problen	No problen	No problen
No	Big probler	Small probl	Big probler	Big probler	Big probler	Small probl	No problen	No problen
No	No problen	No problen	No problen	No problen	Small probl	No problen	No problen	Small probl
Yes	Small probl	Small probl	Big probler	No problen	Big probler	No problen	No problen	Big probler
	Big probler	No problen	Big probler	Big probler	Big probler	Small probl	No problen	No problen
	Small probl	No problen	Big probler	Big probler	Big probler	Big probler	Small probl	No problen
No	Small probl	Small probl	Big probler	Big probler	Big probler	Big probler	Big probler	Big probler
	No problen	No problen	No problen	No problen	Small probl	No problen	No problen	No problen
No	No problen	No problen	No problen	No problen	No problen	No problen	No problen	No problen
	Small probl	Small probl	Small probl	Small probl	Big probler	Small probl	Small probl	Small probl
No	No problen	No problen	No problen	No problen	No problen	No problen	No problen	No problen
No	No problen	No problen	No problen	No problen	Small probl	Small probl	No problen	No problen
	Small probl	Small probl	Small probl	Small probl	Small probl	Small probl	Small probl	No problen
No	No problen	No problen	Small probl	Small probl	Small probl	Small probl	Small probl	Small probl
No	Small probl	Small probl	Big probler	Small probl	Big probler	No problen	No problen	Small probl
No	No problen	No problen	No problen	No problen	No problen	No problen	No problen	No problen
	Small probl	Small probl	Big probler	Big probler	Big probler	Big probler	Big probler	Big probler
No	Big probler	No problen	Big probler	Big probler	Big probler	Big probler	No problen	No problen
	Small probl	Small probl	Small probl	Small probl	Small probl	Small probl	No problen	Small probl
	Small probl	Small probl	Small probl	Small probl	Small probl	Big probler	No problen	Big probler
	No problen	No problen	No problen	No problen	No problen	No problen	No problen	No problen
No	Small probl	No problen	Big probler	Small probl	Big probler	Small probl	Small probl	Small probl
No	No problen	No problen	No problen	Small probl	Small probl	Small probl	No problen	No problen
	Small probl	Small probl	Small probl	Small probl	Small probl	No problen	No problen	No problen
No	Small probl	Small probl	Small probl	No problen	Big probler	Small probl	No problen	No problen
	No problen	Small probl	Small probl	Small probl	Small probl	Small probl	Small probl	Small probl

	Small probl	Small probl	Big probler	Big probler	Big probler	Big probler	Big probler	Big probler
	Big probler	Small probl	Small probl	Small probl	Small probl	Small probl	Small probl	Small probl
Yes	Small probl	Small probl	Big probler	Small probl	Big probler	No problen	Small probl	No problen
	Big probler	Small probl	Small probl	Small probl	Small probl	Small probl	No problen	No problen
	Small probl	Small probl	Small probl	Small probl	Small probl	No problen	No problen	No problen
	Small probl	Small probl	Small probl	Small probl	Small probl	No problen	No problen	No problen
No	Small probl	No problen	Big probler	No problen	Big probler	No problen	No problen	Small probl
	Small probl	Small probl	Small probl	Small probl	Small probl	Small probl	Small probl	Small probl
No	No problen	No problen	No problen	No problen	Big probler	No problen	No problen	No problen
Yes	Small probl	Small probl	Big probler	Big probler	Big probler	Big probler	Small probl	No problen
Yes	Big probler	Small probl	Big probler	Small probl	Big probler	Small probl	No problen	Big probler
	No problen	No problen	No problen	No problen	Big probler	Big probler	Big probler	Small probl
No	Small probl	No problen	No problen	Small probl	Small probl	Small probl	Small probl	No problen
Yes	No problen	Small probl	Big probler	Big probler	Big probler	Small probl	Small probl	Big probler
No	Small probl	Small probl	Big probler	Big probler	Big probler	Small probl	Small probl	No problen
No	No problen	No problen	No problen	Small probl	Small probl	Small probl	Small probl	Small probl
No	Big probler	Big probler	Big probler	Big probler	Big probler	Small probl	Small probl	Small probl
	No problen	No problen	No problen	No problen	No problen	No problen	No problen	No problen
	No problen	No problen	No problen	No problen	No problen	No problen	No problen	No problen
No	No problen	No problen	Big probler	Big probler	Big probler	Big probler	No problen	Big probler
No	No problen	No problen	No problen	Small probl	Small probl	No problen	No problen	No problen
Yes	No problen	No problen	Small probl	No problen	Small probl	Big probler	No problen	Small probl
No	No problen	No problen	Big probler	Small probl	Big probler	No problen	No problen	Small probl
	No problen	No problen	No problen	No problen	No problen	No problen	No problen	No problen
No	No problen	No problen	No problen	No problen	No problen	No problen	No problen	No problen
No	No problen	No problen	No problen	No problen	No problen	Big probler	No problen	No problen
Yes	No problen	No problen	Big probler	No problen	Big probler	Small probl	No problen	No problen
	Small probl	Small probl	Big probler	Big probler	Big probler	Big probler	Big probler	Big probler
	No problen	No problen	No problen	No problen	No problen	No problen	No problen	No problen
	Small probl	No problen	Big probler	Big probler	Big probler	Big probler	Big probler	Big probler
	Small probl	Small probl	Big probler	Big probler	Big probler	Small probl	Small probl	Big probler
	Small probl	Small probl	Big probler	Big probler	Big probler	Small probl	Big probler	Small probl
No	No problen	No problen	No problen	Small probl	Big probler	Big probler	No problen	No problen
No	Small probl	Big probler	Big probler	Big probler	Big probler	Big probler	Big probler	Small probl
	No problen	No problen	Small probl	Big probler	Big probler	No problen	No problen	No problen
No	No problen	No problen	No problen	No problen	No problen	No problen	No problen	Small probl
	No problen	No problen	Small probl	No problen	No problen	Small probl	No problen	Big probler
	No problen	No problen	No problen	No problen	No problen	No problen	No problen	No problen
	No problen	No problen	Big probler	Small probl	Small probl	Small probl	Small probl	Small probl
	No problen	No problen	Big probler	No problen	No problen	No problen	No problen	No problen
	No problen	No problen	No problen	No problen	No problen	No problen	No problen	No problen
	No problen	No problen	Big probler	Big probler	Big probler	No problen	No problen	No problen
No	No problen	No problen	No problen	No problen	No problen	No problen	No problen	No problen
	No problen	No problen	No problen	No problen	No problen	No problen	No problen	No problen
	No problen	No problen	No problen	No problen	No problen	No problen	No problen	No problen
	No problen	No problen	Small probl	No problen	Small probl	No problen	No problen	No problen
	Big probler	Big probler	Big probler	Small probl	Big probler	Big probler	Small probl	Big probler
	No problen	No problen	No problen	No problen	No problen	No problen	No problen	No problen
	No problen	No problen	No problen	No problen	No problen	No problen	No problen	No problen
	Small probl	Small probl	Big probler	Big probler	Big probler	Big probler	Big probler	Big probler
Yes	No problen	No problen	Big probler	No problen	No problen	No problen	No problen	No problen
No	No problen	No problen	No problen	No problen	No problen	No problen	No problen	No problen

For peer review only

	No problen	No problen	No problen	No problen	No problen	No problen	No problen	No problen
	Small probl	Small probl	Big probler	Big probler	Big probler	Small probl	Big probler	Big probler
	No problen	No problen	No problen	No problen	No problen	No problen	No problen	No problen
Yes	Small probl	Small probl	Big probler	Big probler	Big probler	Big probler	Big probler	Big probler
	No problen	No problen	No problen	No problen	Small probl	Small probl	No problen	No problen
	No problen	No problen	No problen	Big probler	Big probler	No problen	No problen	No problen
	No problen	No problen	No problen	Big probler	Big probler	Big probler	No problen	No problen
	Small probl	Small probl	Big probler	Big probler	Big probler	Small probl	No problen	Small probl
	Small probl	Small probl	Small probl	Small probl	Small probl	Small probl	Small probl	Small probl
	No problen	No problen	No problen	Big probler	Big probler	No problen	No problen	No problen
	No problen	No problen	No problen	No problen	No problen	No problen	No problen	No problen
No	No problen	No problen	Big probler	Small probl	Small probl	Small probl	No problen	No problen
	No problen	No problen	Big probler	No problen	Big probler	Small probl	Small probl	Small probl
	No problen	No problen	No problen	No problen	No problen	No problen	Small probl	No problen
	No problen	No problen	No problen	No problen	No problen	No problen	No problen	No problen
Yes	No problen	No problen	No problen	No problen	No problen	No problen	No problen	No problen
	No problen	No problen	No problen	No problen	Small probl	Small probl	No problen	No problen
No	No problen	Small probl	Big probler	Small probl	Big probler	No problen	No problen	No problen
	Small probl	Small probl	Small probl	Small probl	Small probl	Small probl	Small probl	Small probl
	No problen	No problen	No problen	No problen	No problen	No problen	No problen	No problen
	No problen	No problen	No problen	No problen	No problen	No problen	No problen	No problen
	No problen	No problen	No problen	No problen	Small probl	No problen	No problen	No problen
	Small probl	Small probl	Small probl	Small probl	Small probl	Small probl	Small probl	No problen
	No problen	No problen	No problen	No problen	No problen	No problen	No problen	No problen
	No problen	No problen	No problen	No problen	No problen	No problen	No problen	No problen
	No problen	No problen	Big probler	Big probler	Big probler	No problen	No problen	No problen
	No problen	No problen	No problen	No problen	Big probler	Small probl	No problen	No problen
No	No problen	No problen	No problen	No problen	Small probl	No problen	No problen	Small probl
	No problen	No problen	No problen	No problen	Small probl	No problen	No problen	No problen
	No problen	No problen	No problen	No problen	Small probl	Small probl	No problen	No problen
Yes	No problen	No problen	No problen	No problen	Small probl	No problen	No problen	No problen
	No problen	No problen	No problen	No problen	No problen	No problen	No problen	No problen
	No problen	No problen	No problen	No problen	No problen	No problen	No problen	No problen
	No problen	No problen	Small probl	Small probl	Small probl	No problen	No problen	No problen
	No problen	No problen	Small probl	No problen	Big probler	Small probl	No problen	Small probl
No	No problen	No problen	No problen	No problen	Big probler	No problen	No problen	No problen
	No problen	No problen	No problen	Small probl	Small probl	No problen	No problen	No problen
	No problen	No problen	No problen	Small probl	Small probl	No problen	No problen	No problen
	No problen	No problen	No problen	No problen	No problen	No problen	No problen	No problen
	No problen	No problen	Small probl	No problen	Small probl	No problen	No problen	No problen
	Small probl	No problen	Small probl	No problen	Small probl	No problen	No problen	Small probl
No	No problen	No problen	No problen	No problen	Small probl	No problen	No problen	No problen
No	No problen	Small probl	Big probler	No problen	Big probler	No problen	Small probl	Small probl
No	No problen	No problen	No problen	Small probl	Small probl	Small probl	No problen	No problen
	No problen	No problen	No problen	No problen	Small probl	No problen	No problen	No problen
	Small probl	Small probl	Small probl	Small probl	Small probl	Small probl	Small probl	Small probl
	No problen	No problen	Big probler	Big probler	Big probler	Small probl	No problen	Small probl
	No problen	Small probl	Big probler	Big probler	Big probler	Small probl	No problen	No problen
	No problen	No problen	No problen	No problen	No problen	No problen	No problen	No problen
	Small probl	Small probl	Small probl	Small probl	Small probl	Small probl	No problen	No problen
	No problen	No problen	No problen	No problen	No problen	No problen	No problen	No problen
	Small probl	Small probl	Small probl	No problen	No problen	No problen	No problen	No problen

For peer review only

	Small probl	Small probl	Small probl	Small probl	Small probl	Small probl	Small probl	Small probl
	No problen	No problen	No problen	No problen	No problen	No problen	No problen	No problen
	No problen	No problen	Big probler	No problen	Big probler	No problen	No problen	Small probl
	No problen	No problen	No problen	No problen	Small probl	No problen	No problen	No problen
	No problen	No problen	Small probl	Big probler	Big probler	Small probl	No problen	No problen
	No problen	No problen	No problen	No problen	No problen	No problen	No problen	No problen
	No problen	No problen	No problen	Big probler	Big probler	No problen	No problen	No problen
No	No problen	No problen	Big probler	Small probl	Big probler	No problen	Small probl	Small probl
	No problen	No problen	No problen	No problen	No problen	No problen	No problen	No problen
	No problen	No problen	No problen	No problen	No problen	No problen	No problen	No problen
	No problen	No problen	No problen	No problen	No problen	No problen	No problen	No problen
	No problen	No problen	No problen	No problen	No problen	No problen	No problen	No problen
	No problen	No problen	No problen	Small probl	Small probl	No problen	No problen	No problen
	No problen	No problen	No problen	No problen	No problen	No problen	Small probl	No problen
	No problen	No problen	No problen	No problen	Small probl	No problen	No problen	No problen
	No problen	No problen	No problen	No problen	No problen	No problen	No problen	No problen
	Small probl	Small probl	Small probl	Small probl	Small probl	Small probl	Small probl	Small probl
	No problen	No problen	Small probl	Small probl	Big probler	Small probl	No problen	No problen
	No problen	No problen	Big probler	Big probler	Big probler	No problen	No problen	No problen
No	No problen	No problen	No problen	No problen	Small probl	Small probl	No problen	No problen
	No problen	No problen	Small probl	Big probler	Big probler	No problen	No problen	No problen
	No problen	No problen	No problen	No problen	No problen	No problen	No problen	No problen
No	Small probl	Small probl	Big probler	Big probler	Big probler	No problen	No problen	No problen
No	Small probl	Small probl	Big probler	Big probler	Big probler	Small probl	No problen	Small probl
No	No problen	No problen	Big probler	Small probl	Big probler	Small probl	Small probl	Small probl
	Small probl	No problen	No problen	Small probl	Small probl	Small probl	No problen	Small probl
	No problen	No problen	No problen	Big probler	Big probler	Big probler	No problen	No problen
	No problen	No problen	No problen	No problen	No problen	No problen	No problen	No problen
	No problen	No problen	No problen	No problen	No problen	No problen	No problen	No problen
	No problen	No problen	No problen	No problen	Small probl	No problen	No problen	No problen
	No problen	No problen	Big probler	Big probler	Big probler	No problen	No problen	No problen
	Small probl	Small probl	Small probl	Small probl	Small probl	Small probl	No problen	Small probl
	No problen	No problen	No problen	No problen	Big probler	No problen	No problen	No problen
	Small probl	Small probl	Small probl	Small probl	Small probl	Small probl	Small probl	Small probl
	No problen	No problen	No problen	No problen	No problen	No problen	No problen	No problen
	No problen	No problen	No problen	No problen	Small probl	Small probl	No problen	No problen
	No problen	No problen	No problen	No problen	No problen	No problen	No problen	No problen
	No problen	No problen	No problen	No problen	No problen	No problen	No problen	No problen
	No problen	No problen	No problen	Small probl	Big probler	Big probler	No problen	No problen
No	Small probl	Small probl	Big probler	Small probl	Big probler	No problen	Small probl	No problen
	Small probl	Small probl	No problen	No problen	No problen	No problen	No problen	No problen
No	No problen	No problen	No problen	Small probl	Big probler	Small probl	No problen	No problen
No	Small probl	No problen	Big probler	No problen	Big probler	Small probl	No problen	No problen
No	No problen	No problen	No problen	Small probl	Small probl	No problen	No problen	No problen
	No problen	No problen	No problen	No problen	No problen	No problen	No problen	No problen
No	No problen	No problen	No problen	No problen	No problen	No problen	No problen	No problen
	No problen	No problen	No problen	No problen	No problen	No problen	No problen	No problen
No	Small probl	Small probl	Big probler	No problen	Big probler	Small probl	Small probl	Small probl
	Small probl	Small probl	Small probl	Small probl	Small probl	No problen	No problen	No problen
	Small probl	Small probl	Small probl	Small probl	Small probl	Small probl	Small probl	No problen
No	Small probl	Small probl	Big probler	Small probl	No problen	No problen	Small probl	No problen
	No problen	No problen	No problen	No problen	Big probler	Big probler	No problen	No problen

For peer review only

	No problen	No problen	No problen	Small probl	Small probl	Small probl	No problen	No problen
	No problen	No problen	No problen	Small probl	Small probl	No problen	No problen	Small probl
	Small probl	No problen	No problen	Small probl	Small probl	No problen	No problen	No problen
Yes	Small probl	Small probl	Big probler	Big probler	Big probler	Small probl	No problen	No problen
	No problen	No problen	No problen	No problen	No problen	No problen	No problen	No problen
No	Small probl	Small probl	Big probler	Small probl	Big probler	No problen	No problen	Small probl
	No problen	No problen	No problen	No problen	No problen	No problen	No problen	No problen
	No problen	No problen	No problen	No problen	No problen	No problen	No problen	No problen
	No problen	No problen	Big probler	Small probl	Big probler	Small probl	Small probl	Small probl
No	Small probl	Small probl	Big probler	Small probl	Big probler	No problen	No problen	Small probl
	No problen	No problen	No problen	No problen	No problen	No problen	No problen	No problen
	No problen	No problen	Small probl	No problen	No problen	Small probl	No problen	No problen
	No problen	No problen	No problen	No problen	No problen	No problen	No problen	No problen
Yes	Small probl	Small probl	Big probler	No problen	Big probler	No problen	No problen	No problen
Yes	Small probl	Small probl	Big probler	Small probl	Big probler	Small probl	No problen	No problen
	Small probl	No problen	Small probl	Small probl	Big probler	No problen	No problen	No problen
No	Small probl	Small probl	Small probl	No problen	Big probler	No problen	No problen	Small probl
	No problen	No problen	No problen	No problen	Big probler	Small probl	No problen	No problen
	No problen	No problen	No problen	Small probl	Small probl	Small probl	No problen	No problen
No	Small probl	Small probl	Small probl	Small probl	Big probler	No problen	No problen	No problen
	No problen	No problen	No problen	Small probl	Small probl	Small probl	No problen	No problen
No	No problen	No problen	No problen	Big probler	Big probler	Big probler	No problen	No problen
	No problen	No problen	No problen	No problen	No problen	No problen	No problen	No problen
	No problen	No problen	No problen	No problen	No problen	No problen	No problen	No problen
	No problen	No problen	Big probler	No problen	Big probler	No problen	No problen	No problen
	No problen	No problen	No problen	No problen	No problen	No problen	No problen	No problen
No	No problen	No problen	Big probler	Small probl	Big probler	Small probl	Small probl	Small probl
No	Small probl	Small probl	Big probler	Small probl	Big probler	No problen	Small probl	No problen
No	No problen	No problen	No problen	No problen	Small probl	Small probl	No problen	No problen
	Small probl	No problen	No problen	No problen	No problen	No problen	No problen	No problen
No	No problen	No problen	Small probl	Big probler	Small probl	No problen	No problen	No problen
No	No problen	No problen	Big probler	No problen	Big probler	Small probl	Small probl	Big probler
No	Small probl	Small probl	Big probler	Small probl	Big probler	Small probl	Small probl	No problen
	No problen	No problen	No problen	No problen	No problen	No problen	No problen	No problen
	Small probl	No problen	No problen	Small probl	Small probl	No problen	No problen	No problen
No	No problen	No problen	No problen	No problen	No problen	No problen	No problen	No problen
	No problen	No problen	No problen	No problen	No problen	No problen	No problen	No problen
	No problen	No problen	No problen	No problen	No problen	No problen	No problen	No problen
No	No problen	No problen	No problen	No problen	No problen	No problen	No problen	No problen
	No problen	No problen	No problen	No problen	No problen	No problen	No problen	No problen
	No problen	No problen	No problen	Small probl	Small probl	No problen	No problen	No problen
	Small probl	Small probl	Small probl	Big probler	Big probler	No problen	No problen	No problen
	No problen	No problen	No problen	No problen	No problen	No problen	No problen	No problen
	No problen	No problen	No problen	No problen	No problen	No problen	No problen	No problen
	No problen	No problen	Small probl	No problen	No problen	No problen	No problen	Big probler
	Big probler	No problen	Big probler	Big probler	Big probler	No problen	No problen	No problen
No	No problen	No problen	No problen	No problen	No problen	No problen	No problen	No problen
	No problen	No problen	Small probl	Small probl	Small probl	Small probl	Small probl	Small probl
	Big probler	No problen	Big probler	Big probler	Big probler	No problen	No problen	No problen
	Big probler	No problen	Big probler	Big probler	Big probler	No problen	No problen	No problen
	No problen	No problen	Small probl	No problen	No problen	No problen	No problen	No problen
	No problen	No problen	Small probl	No problen	Small probl	No problen	Small probl	No problen

	Small probl	Small probl	Small probl	Big probler	Big probler	Small probl	No problen	Small probl
	No problen	No problen	Small probl	No problen	Big probler	No problen	No problen	No problen
	No problen	No problen	No problen	No problen	No problen	No problen	No problen	No problen
	Small probl	Small probl	Big probler	Big probler	Big probler	Big probler	Big probler	Big probler
	No problen	No problen	Small probl	Big probler	Big probler	Small probl	No problen	Small probl
	No problen	No problen	Big probler	Small probl	Small probl	Small probl	Small probl	Small probl
	No problen	Small probl	No problen	No problen	Big probler	No problen	No problen	No problen
Yes	Big probler	Small probl	Big probler	Big probler	Big probler	Small probl	No problen	No problen
	No problen	No problen	No problen	No problen	No problen	No problen	No problen	No problen
	No problen	No problen	Big probler	Small probl	Small probl	No problen	No problen	No problen
	No problen	No problen	No problen	No problen	No problen	No problen	No problen	No problen
	No problen	No problen	No problen	No problen	Small probl	Small probl	No problen	No problen
	No problen	No problen	Big probler	Big probler	Big probler	No problen	No problen	No problen
	No problen	No problen	No problen	No problen	Big probler	Big probler	Small probl	No problen
	Small probl	Small probl	Big probler	Small probl	Big probler	Small probl	Small probl	Small probl
	Small probl	Small probl	Big probler	Small probl	Big probler	Big probler	Small probl	Big probler
	Small probl	No problen	Big probler	Small probl	Big probler	Small probl	Small probl	Small probl
	No problen	No problen	No problen	No problen	No problen	No problen	No problen	No problen
No	No problen	No problen	Big probler	No problen	No problen	No problen	No problen	No problen
	Big probler	Small probl	Big probler	Big probler	Big probler	Small probl	No problen	No problen
	No problen	No problen	Small probl	No problen	No problen	Small probl	No problen	No problen
	No problen	No problen	No problen	No problen	No problen	No problen	No problen	No problen
	Small probl	Small probl	Big probler	Small probl	Big probler	Small probl	Big probler	Big probler
	Big probler	Small probl	Big probler	Big probler	Big probler	Small probl	No problen	No problen
No	No problen	Big probler	Big probler	Big probler	Small probl	No problen	No problen	No problen
	Big probler	Small probl	Big probler	Big probler	Big probler	No problen	No problen	No problen
No	No problen	No problen	No problen	No problen	Small probl	Small probl	No problen	No problen
	Small probl	Small probl	Big probler	No problen	Small probl	Small probl	Small probl	Small probl
	No problen	No problen	No problen	No problen	No problen	No problen	No problen	No problen
	No problen	No problen	Small probl	No problen	Small probl	No problen	No problen	No problen
[truncated: 4,565,778 more chars]
